# Summary of anti-malarial prophylactic efficacy of tafenoquine from three placebo-controlled studies of residents of malaria-endemic countries

**DOI:** 10.1186/s12936-015-0991-x

**Published:** 2015-11-26

**Authors:** Geoffrey S. Dow, Jun Liu, Gina Lin, Brian Hetzell, Sarah Thieling, William F. McCarthy, Douglas Tang, Bryan Smith

**Affiliations:** 60° Pharmaceuticals LLC, Washington, DC, 20036 USA; PPD, Inc., Richmond, VA 23230 USA; USAMMDA, Ft Detrick, MD 21702 USA

**Keywords:** Malaria, Prophylaxis, Prophylactic efficacy, Tafenoquine, Mefloquine, Malaria-endemic countries

## Abstract

**Background:**

Tafenoquine is a long half-life primaquine analog being developed for malaria prophylaxis. The US Army recently performed a unified analysis of efficacy in preparation for a regulatory submission, utilizing legacy data from three placebo-controlled studies conducted in the late 1990s and early 2000s. The subjects were residents of Africa who were naturally exposed to *Plasmodium falciparum* for 12–26 weeks.

**Methods:**

The prophylactic efficacy of tafenoquine and mefloquine (included in some studies as a comparator) was calculated using incidence density among subjects who had completed the three-day loading doses of study drug, had at least one maintenance dose and had at least one blood smear assessed during the prophylactic period. The three placebo-controlled studies were analysed separately and then in two pooled analyses: one for tafenoquine *versus* placebo (three studies) and one for tafenoquine and mefloquine *versus* placebo (two studies).

**Results:**

The pooled protective efficacy (PE) of a tafenoquine regimen with three daily loading doses plus weekly maintenance at 200-mg for 10 weeks or longer (referred to as 200-mg weekly hereafter) relative to placebo in three placebo-controlled studies was 93.1 % [95 % confidence interval (CI) 89.1–95.6 %; total N = 492]. The pooled PEs of regimens of tafenoquine 200-mg weekly and mefloquine 250-mg weekly relative to placebo in two placebo-controlled studies (total N = 519) were 93.5 % (95 % CI 88.6–96.2 %) and 94.5 % (95 % CI 88.7–97.3 %), respectively. Three daily loading plus weekly maintenance doses of 50- and 100-mg, but not 25-mg, exhibited similar PEs. The PEs of tafenoquine regimens of a three-day loading dose at 400-mg with and without follow-up weekly maintenance doses at 400-mg were 93.7 % (95 % CI 85.4–97.3 %) and 81.0 % (95 % CI 66.8–89.1 %), respectively.

**Conclusions:**

Tafenoquine provided the same level of prophylactic efficacy as mefloquine in residents of Africa. These data support the prophylactic efficacy of tafenoquine and mefloquine that has already been demonstrated in the intended malaria naive population.

**Electronic supplementary material:**

The online version of this article (doi:10.1186/s12936-015-0991-x) contains supplementary material, which is available to authorized users.

## Background

Tafenoquine, due to its long half-life, blood schizonticidal activity, inhibitory effect on developing exoerythrocytic schizonts and anti-relapse efficacy against *Plasmodium vivax* hypnozoites, may fill a valuable niche that is unoccupied by marketed anti-malarial agents. In the 1990s and early 2000s, a series of three placebo-controlled studies (Studies 030, 043, and 045) were conducted to assess the safety and prophylactic efficacy of weekly and monthly regimens of tafenoquine at various doses as well as the comparator prophylactic drug, mefloquine, among the residents of malaria-endemic countries (Kenya and Ghana) [1–4]. The results of two of those three studies (Studies 043, and 045) were reported elsewhere. The objective of the present work was to present the protective efficacy (PE) of tafenoquine in all three of these studies and pooled analysis results in a uniform manner, including a retrospective re-analysis of one of the studies that has not been previously described (Study 030). This analysis is consistent with the observations of a prior prophylaxis study in malaria-naive Australian soldiers deployed to Timor-Leste for peace keeping operations who received either mefloquine or tafenoquine chemoprophylaxis [5]. No malaria cases were observed in subjects taking either drug, and the point estimates of efficacy were estimated retrospectively to be 100 % (95 % CIS 93–100 % for tafenoquine; 79–100 % for mefloquine) [5, 6].

## Methods

### Original study designs

All three studies, either presented as individual studies or used in the pooled analysis, were placebo-controlled, randomized, double-blind clinical evaluations of the efficacy of tafenoquine compared with that of mefloquine or placebo in the prophylaxis of malaria. All three study protocols were very similar and the studies were conducted in a similar manner. Study participants were adult Africans who were living in an area endemic for *Plasmodium falciparum* malaria. All three studies employed a design that started with a clearance period to clear existing parasitaemia; then, usually a few days later, the loading period began, with daily study drug administration for three consecutive days. This period was followed immediately by a maintenance period with weekly study drug administration. In all studies, blood samples were collected weekly from study participants during the entire study for parasitological assessment. Informed consent was obtained from all study subjects, and all studies were approved by competent institutional review boards. Below, a brief summary is provided for each of the studies.

**Fig. 1:**
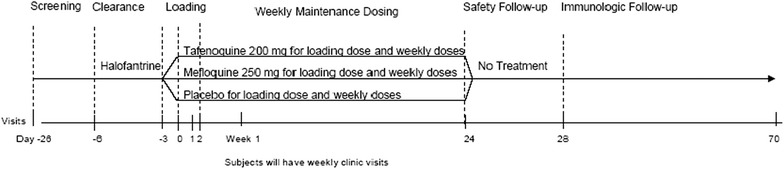
Study schematic for Study 030

*Study 030* was conducted in 2000 among healthy adults who were 18–55 years old and living in the village of Kombewa in the Nyanza Province of western Kenya [1]. Study participants who met the study entry criteria were first treated for 3 days (day −5 to day −3) with halofantrine (at 250 mg once daily). At the end of the clearance period (on day 0), study participants who were free from malaria parasitaemia, as confirmed by a sample for thick blood smear taken on day −2, were randomized into the tafenoquine 200-mg, mefloquine 250-mg or placebo group. The maintenance period lasted for 24 weeks with weekly administration of study drugs after 3 days of loading doses (day 0–day 2). After the treatment period, study participants attended weekly follow-up safety visits until week 28. During the maintenance period, subjects who tested positive for Plasmodium species were considered prophylactic failures and where withdrawn from treatment. They were entered into the safety follow-up phase of the study and given treatment such as quinine or doxycycline. A schematic for this study is displayed in Fig. 1. Peak malaria season in western Kenya runs from May to July, the first dose of prophylaxis medication was given in May of 2000 and the final dose of prophylaxis medication was given in October of 2000 [7]. The protocol and statement of informed consent were approved by KEMRI and the US Army Human Subjects Research Review Board (HSRRB) prior to study initiation.

**Fig. 2:**
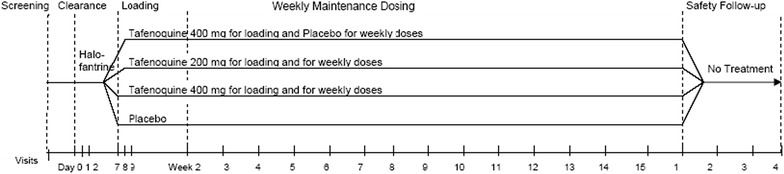
Study schematic for Study 043

*Study 043* was conducted in 1997 among healthy adults who were 18–55 years old and living in the village of Ndori, Nyanza Province, Kenya [2]. Study participants who met the study entry criteria were given a three-day (day 0–day 2) eradication course of halofantrine (at 250 mg once daily). On day 7, after confirmation of being parasitaemia-free by blood smear samples, they were then randomized into one of four groups to receive one of three dosage regimens of tafenoquine (400-, 200- or 400-mg as a loading dose only) or a placebo regimen. For the 400- and 200-mg groups, the maintenance period lasted for a period of 10–15 weeks with weekly tafenoquine administration at the same dosage level as the loading doses taken on days 7–9. With the exception of the loading doses, study participants in the tafenoquine 400-mg loading dose only group did not receive additional tafenoquine doses during the maintenance period. After the maintenance period, study participants were followed for an additional 4 weeks (Fig. 2). Participants received prophylaxis between May 1997 and September 1997. Details regarding informed consent, treatment of symptomatic failures and the institutional review board are provided in the original publication [2].

**Fig. 3:**
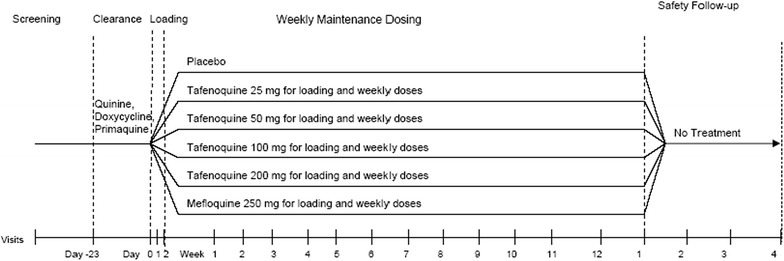
Study schematic for Study 045

*Study 045* was conducted in 1998 among healthy adults (aged 18–60 years for men and 40–60 years for women) living in the Kassena Nankana District of northern Ghana [3]. All study participants initially completed a course of eradication treatment of quinine (10 mg/kg tid) for 4 days followed by doxycycline (100 mg po bd) for 7 days and primaquine (30 mg daily) for 14 days. Five days later, after confirmation of being parasitaemia-free by blood smear samples, study participants were randomized into one of the four tafenoquine regimens (25-, 50-, 100- or 200-mg), the mefloquine 250-mg group or the placebo group. The weekly maintenance period lasted for 12 weeks after 3 days of daily loading doses corresponding to the randomized treatment. There was a follow-up period for an additional 4 weeks after drug administration ended. A schematic for this study is displayed in Fig. 3. The major malaria season in northern Ghana runs from May to October with a peak in August, the first dose of prophylaxis medication was given in May of 2000 and the final dose of prophylaxis medication was given in October of 2000 [8]. Details regarding informed consent, treatment of symptomatic failures and institutional review board are provided in the original publication [3].

The following were the major exclusion criteria used by each of the original studies:

- Presence of clinically significant abnormalities (that included but were not limited to abnormal hepatic or renal function) as determined by history, physical examinations or routine blood chemistries or haematology values or any medical condition that, in the opinion of the investigator, made the subject unsuitable to enter the study.
- Use of any other anti-malarial product (excluding those taken during the clearance period) within the previous 2 weeks.
- Hypersensitivity to any of the study drugs, especially to any other 8-aminoquinolines.
- Presence of glucose-6-phosphate dehydrogenase (G6PD) deficiency. In Study 030, glucose 6 phosphate dehydrogenase deficiency (G6PD) status was determined on two occasions during the screening period using standard laboratory tests (Sigma Diagnostics kit, Procedure #203—fluorescence of sample vs normal, intermediate and deficient controls). The same technique was used for both tests. Only subjects with a ‘normal’ reading on both occasions were eligible for the study [1]. In Study 043, G6PD status was determined using Sigma Diagnostics kit [2]. In Study 045, G6PD deficiency was determined by two separate qualitative tests using distinct methods—visual dye and filter paper [3].
- Use of investigational drugs (new chemical entities not registered for use) within 30 days or five half-lives, whichever was longer.
- Positive serum beta-human chorionic gonadotropin test results (tested during screening and within 48 h of the first drug administration and approximately monthly thereafter), pregnancy or lactation or, in the opinion of the investigator, risk of becoming pregnant.

Studies had various definitions of abnormal laboratory values for haemoglobin, platelets, white blood cell count, and creatinine and alanine aminotransferase. Typically, abnormal laboratory values were defined as more than twice the upper limit of normal for that subject’s age. Some studies (Studies 030 and 045) excluded individuals with histories of psychiatric disorders or personal or family histories of seizures. In addition, Study 030 excluded anyone with an abnormal electrocardiogram finding, particularly an extended QTc interval >0.42 s.

## Efficacy data from the original studies

### Efficacy data collection

The primary efficacy endpoint for all studies was prophylactic outcome (success/failure) at the end of the prophylactic treatment phase. Prophylactic outcome was based on the absence/presence of asexual stage parasites of any *Plasmodium* species on a blood smear. For efficacy data collection, duplicate thick and thin smears were prepared and stained with Giemsa and each slide was read by two separate microscopists; each microscopist was blinded to the other’s diagnosis. Asexual stage parasites in 50 high-power fields were counted and reported as the number of parasites per 500 white blood cells; a minimum of 200 high-powered fields were to be examined before a slide could be declared negative. Negative/positive discrepancies between the two microscopists were resolved by a third microscopist, who usually was a senior microscopist, by re-reading the slide. The result of this re-reading of the slide was considered final.

All studies, except Study 043, considered a study participant a prophylactic failure based on the first positive smear result of any malaria species. In Study 043, a second confirmatory blood sample was required to be taken within 7 days of when the first smear reading was positive. Prophylactic failure was only declared if the confirmative smear reading was also positive.

### Special note on Study 030 microscopy data

During the conduct of Study 030, it was found that unusually high proportions of the participants failed prophylaxis for all three treatment groups (75/101 for tafenoquine, 85/98 for mefloquine, and 91/99 for placebo at the time of review). This led to the setting up of an Independent Data Monitoring Committee (IDMC) and a series of investigations to eliminate possible technical problems. The technical investigations focused on (1) slide reading, (2) parasite resistance, (3) drug packaging, (4) analysis of pharmacokinetic samples, and (5) procedural errors. Drug supply packaging and stability were found to be in order, and analysis of blood samples showed tafenoquine at target levels. As a part of the investigation, the original slides were re-read by Naval Medical Research Unit 2 (NAMRU-2) in Jakarta, Indonesia, and the Australian Malaria Institute (AMI).

There were two batches of slides sent to be re-read. The first batch of 364 slide pairs was sent to NAMRU-2 whilst the study was still ongoing. These were all of the first positive slides (113 duplicate pairs) up to approximately week 12, plus a random selection of negative slides (251 duplicate pairs). A second batch, which included all remaining positive slides that could be located at the study site plus a randomly selected number of negatives, was also shipped to NAMRU-2 for re-read at the end of the study. NAMRU-2 microscopists followed the USAMRU-K slide reading SOP that was being used for the study. Each slide was independently read by two readers. Any discrepancies were adjudicated by a third, senior microscopist whose decision was considered the final result. The re-reading was blinded, i.e. the microscopist did not know whether the slide had been originally categorized by USAMRU-K as positive or negative. The results are shown in Table 1. Of the 113 slides identified as positive by the local slide readers in Kenya, only 19 were also read positive by NAMRU-2. The re-reading at NAMRU-2 resulted in an overall concordance rate (i.e. slides determined either as positive at both sites or negative at both sites) of 74.8 % (264/353 not including unevaluable slides) and a positive concordance rate of only 17.6 % (19/108). Following the re-read by NAMRU-2, a subset consisting of the 113 positive slide pairs was sent to a world-leading malaria parasite morphologist at AMI for a further re-read. Their conclusion was also that only 19 of the 113 USAMRU-K positives contained malaria parasites. Of these 19, sixteen were the same slide pairs as the NAMRU-2 positive slides; AMI also reported a further 3 slide pairs to be positive which NAMRU-2 had reported as negative and 3 slide pairs as negative which NAMRU-2 reported as positive. No statistical comparisons between the NAMRU-2 and AMI readings were made because it cannot be guaranteed that the identity of the NAMRU-2 positive slides was not disclosed to AMI.

**Table 1:** Comparison of USAMRU-K and NAMRU-2 slide-readings for Study 030

USAMRU-K Reading	NAMRU-2 reading			
	Positive	Negative	Unevaluable	Total
Positive	19	89	5	113
Negative	0	245	6	251
Total	19	334	11	364

*USAMRU-K* US Army Medical Research Unit—Kenya, *NAMRU-2* Naval Medical Research Unit-2

In parallel, and in concert with this conclusion, the IDMC conducted an unblinding of parasitaemia results from the center and identified low apparent protective efficacies for both tafenoquine and mefloquine groups. Taking this into consideration, plus a review of serious adverse event data from the study, the IDMC concluded that the study should continue in order to meet secondary objectives of evaluating the long-term safety and efficacy of tafenoquine.

A comparison of the original slide-reading as performed at USAMRU-K with the result of the re-reading performed at NAMRU-2 is shown in Table 2 for all re-read slides, including those re-read after study completion, irrespective of treatment group. The all re-read slide set included all slides which could be readily located which were diagnosed as positive by USAMRU-K plus a random selection of negative slides diagnosed by the USAMRU-K slide-readers.

**Table 2:** Study 030 slide re-reading results by NAMRU-2

Original reading	NAMRU-2 re-reading			
	Positive	Negative	Missing	Total
Positive	31	220^a^	0	251
Negative	5	507	0	512
Missing	0	3	0	3
Total	36	730	0	766

^a^One participant had two negative slide results recorded on the same date

Based on the cause identified for the higher than expected failure rate being the microscopists’ false positive readings, it is reasonable to conclude the principle analysis results from the study cannot therefore be treated as an accurate reflection of the true chemoprophylactic efficacy of tafenoquine. On the other hand, with the exception of the errors in slides read by USAMRU-K, study 030 was a well-executed study. Among the original study ITT set of 306 study participants, 289 of them have at least one valid slide re-read data by NAMRU-2 available (Table 3). A valid slide re-read is one that resulted in either positive or negative classification. In addition, the selection of the slides for re-read does not appear to bias the outcomes in anyway. The accuracy and reliability of the results of the re-read slides have been confirmed by the second re-read of a subset of slides by AMI. Therefore, it has been determined that the slide re-read data can be used as a reasonable proxy to investigate the prophylactic efficacy of tafenoquine. Details regarding the investigation of false-positive slide reading results and the validation of the slide data re-read by AMI are provided in Additional file 1.

**Table 3:** Classification of subject IDs with valid re-read data

	Treatment group (randomized and received at least one dose)			
	Placebo (N = 101)	TQ 200 mg (N = 104)	MQ 250 mg (N = 101)	Total (N = 306)
No. of subjects with valid re-read results	93	100	96	289
No. of subjects with positive smears	31	2	2	35
No. of subjects without positive smears	62	98	94	254
No. of subjects without valid re-read results	8	4	5	17

Valid re-read slides are those with either an outcome of “positive” or “negative” and were collected on or after the 1st dose

## Definitions used in unified efficacy analysis

### Prophylactic phase

For the unified efficacy analysis, the prophylactic phase is defined from the date of the first dose of study drug administration until the date of the last dose plus one additional scheduled maintenance dosing interval. For all three studies, the prophylactic phase ended at 1 week after the date of the last maintenance dose.

### Primary efficacy analysis set

This set is defined as all study participants who met the following five criteria:

1. Completed the clearance phase successfully,
2. Were randomized to a study treatment group,
3. Completed the loading dose phase,
4. Received at least one dose of study drug in the prophylactic phase, and
5. Had at least one usable blood smear result in the prophylactic phase.

This definition is slightly different from the ITT set defined in the Food and Drug Administration (FDA) draft guidance [9], which defines the ITT set as the set of subjects who had completed the clearance phase successfully, had been randomized and had received at least one dose of study drug. Due to this difference, the primary analysis set used for the unified analyses will be referred to as the modified ITT (mITT) set hereafter. This definition is used to be consistent with the primary analysis sets used in the original studies, which were all designed and conducted well before the publication of the FDA guidance.

Although not used in the analyses presented here, the FDA ITT set (referred to as ITT) is displayed alongside the mITT set in Table 4. As can be seen, there are minimal differences between the two sets. Also, it can be seen from Table 4 that the amount of missing data is below 10 % for the treatment group of interest—tafenoquine 200-mg weekly. For Study 030, the last two criteria were modified to include all study participants with at least one valid re-read smear result. A valid re-read smear result was a re-read record with a classification of either positive or negative and with a collection date that was on or after the date of the first dose. If a smear re-read result existed in both the NAMRU-2 and AMI data, the AMI result was used in the unified analysis.

**Table 4:** Study participant disposition by individual study

Study	Treatment	Randomized	ITT	mITT	Total
030	Placebo	101 (100.0 %)	93 (92.1 %)	93 (92.1 %)	101
	Tafenoquine 200-mg weekly	104 (100.0 %)	100 (96.2 %)	99 (95.2 %)	104
	Mefloquine 200-mg weekly	101 (100.0 %)	96 (95.0 %)	96 (95.0 %)	101
043	Placebo	62 (100.0 %)	61 (98.4 %)	60 (96.8 %)	62
	Tafenoquine 400-mg load only	64 (100.0 %)	60 (93.8 %)	57 (89.1 %)	64
	Tafenoquine 200-mg weekly	61 (100.0 %)	55 (90.2 %)	55 (90.2 %)	61
	Tafenoquine 400-mg weekly	62 (100.0 %)	59 (95.2 %)	57 (91.9 %)	62
045	Placebo	96 (100.0 %)	94 (97.9 %)	94 (97.9 %)	96
	Tafenoquine 25-mg weekly	95 (100.0 %)	93 (97.9 %)	93 (97.9 %)	95
	Tafenoquine 50-mg weekly	94 (100.0 %)	93 (98.9 %)	91 (96.8 %)	94
	Tafenoquine 100-mg weekly	94 (100.0 %)	94 (100.0 %)	94 (100.0 %)	94
	Tafenoquine 200-mg weekly	94 (100.0 %)	93 (98.9 %)	91 (96.8 %)	94
	Mefloquine 250-mg weekly	48 (100.0 %)	46 (95.8 %)	46 (95.8 %)	48

Percentages are based on the number of randomized study participants in each treatment group of each study

*ITT* intent-to-treat, *mITT* modified intent-to-treat

### Prophylactic failure

For the purpose of the unified analysis, a study participant was considered a prophylactic failure if during the prophylactic phase at least one positive smear from the participant was present. When raw smear data were not available, prophylactic outcome classification of the original study was used for the unified analysis.

### Estimation of protective efficacy

Protective efficacy (PE) was defined as the percent reduction in the incidence density (ID) of prophylactic failures (all species) in those study participants who received tafenoquine (or mefloquine) compared to placebo, which was calculated as follows:

1 $$\begin{aligned} {\text{PE}}\, ( {\text{\% )}} &= \left( {\frac{{{\text{ID}}_{\text{p}} - {\text{ID}}_{\text{t}} }}{{{\text{ID}}_{\text{p}} }}} \right) \times 1 0 0 \nonumber \\ &= ( 1- {\text{IDR}_{\text{t,p}})} \times 1 0 0 \end{aligned}$$

where ID_p_ is the incidence density of prophylactic failure in the placebo group, ID_t_ is the incidence density of prophylactic failure in the tafenoquine group (or mefloquine group), and IDR_t,p_ is the incidence density ratio (ID_t_/ID_p_) of the treatment group [tafenoquine (or mefloquine)] relative to placebo.

The ID was defined as the cumulative number of prophylactic failures divided by the sum of time at risk for each subject within each treatment group. For subjects who did not discontinue from the study early and did not have positive smear results, their time at risk was the same as the length of the prophylactic phase. For subjects who had positive smears, their time at risk stopped on the date of the first positive smear. For subjects who discontinued early, their time at risk stopped at the time they discontinued. When a discontinuation date was not available, either the last contact date or the last dose date was used, depending on which one was available. Total person-time at risk for the treatment groups was obtained as the sum of the time at risk for each subject in the group.

The incidence density ratio (IDR) in Eq. (1) was estimated using a Poisson regression model with no intercept and treatment as covariate. The logarithm of total time at risk for each treatment group was used as an offset. PE was calculated from PE = (1 – IDR). An approach based on the likelihood ratio test was used for constructing the corresponding 95 % 5 CI. The calculation was implemented using SAS software version 9.2.

## Data integration

### Rationale for the pooling of subject-level records across studies

The rationale for the unified analysis and pooling of subject-level records of similar regimens of the three studies are due to (1) the identically defined study objectives and primary efficacy endpoints, and almost identical study designs, (2) similar study populations (Africans resident in an areas of high malaria transmission), (3) similar sample sizes of the respective regimens, and (4) the availability of subject-level study records. This analysis strategy fully utilizes the data available and is strongly supported by the sensitivity analysis presented below. Two pooled data sets were created by merging subject-level records of the respective regimens: (1) placebo and tafenoquine (200-mg) groups from Studies 030, 043 and 045; and (2) placebo, mefloquine and tafenoquine (200-mg) groups from Studies 030 and 043. Mefloquine was the historical standard of care. The 200-mg dose of tafenoquine is the intended dose for marketing and achieves the desired steady-state trough concentrations [6].

As a sensitivity analysis of the method of pooling of the subject-level results across studies, we combined the study-specific protective efficacy estimates from the individual tafenoquine 200-mg weekly regimens (intended dose) using meta-analysis methodologies. To help determine whether a fixed effect or random effects model should be used to combine the individual PE estimates, the heterogeneity (variability) of the individual PE estimates was assessed using Cochran’s Q statistic [10], and the *I*^*2*^ and *H* statistics of Higgins and Thomson [11]. The latter is sometimes recommended when the meta-analysis is based on a small number of studies [12].

The approach in DerSimonian and Laird [13] was used to estimate the between study variance in PE estimates and, subsequently, *I*^*2*^ and *H*. If the *Q*-statistic was less than the number of studies (2 or 3), the *I*^*2*^ and *H* statistics were truncated to their minimum values, respectively [11]. Based on the results of those investigations (see “Results” section below for more details), a fixed effect model [14] was used for the meta-analysis. It is worth noting that this method is equivalent to the better known Mantel–Haenszel method [14].

### Disposition and early withdrawal

Table 4 shows the disposition of subjects by individual original study. The main reason for the reduction in the number of subjects from the randomized to the mITT population was due to screening failure. The second most common reason was the lack of blood smears for the efficacy assessment. Table 5 shows reasons and number of early withdrawals from the original studies. The large number of early withdrawals due to insufficient therapeutic effect observed in the tafenoquine and mefloquine groups in Study 030 reflected the false-positive smear readings by the original study site. In the original Study 030, all subjects who were considered to have had positive smears were removed from the study prior to the end of the treatment period.

**Table 5:** Primary reason for early discontinuation among all randomized study participants by study

Study	Treatment	Total early withdrawal	Adverse experience	Insufficient therapeutic effect^a^	Protocol deviation	Lost to follow-up	Moved^b^	Other or unknown
030	Placebo	100	0	93 (93.0 %)	2 (2.0 %)	3 (3.0 %)	NR	2 (2.0 %)
	TQ 200-mg weekly	97	2 (2.1 %)	88 (90.7 %)	1 (1.0 %)	3 (3.1 %)	NR	3 (3.1 %)
	MQ 250-mg weekly	97	1 (1.0 %)	89 (91.8 %)	3 (3.1 %)	1 (1.0 %)	NR	3 (3.1 %)
	Total	294	3 (1.0 %)	270 (91.8 %)	6 (2.0 %)	7 (2.4 %)	NR	8 (2.7 %)
043	Placebo	27	0	22 (81.5 %)	1 (3.7 %)	4 (14.8 %)	NR	0
	TQ 400-mg load only	18	1 (5.6 %)	5 (27.8 %)	2 (11.1 %)	9 (50.0 %)	NR	1 (5.6 %)
	TQ 200-mg weekly	13	1 (7.7 %)	1 (7.7 %)	8 (61.5 %)	3 (23.1 %)	NR	0
	TQ 400-mg weekly	10	0	0	6 (60.0 %)	4 (40.0 %)	NR	0
	Total	68	2 (2.9 %)	28 (41.2 %)	17 (25.0 %)	20 (29.4 %)	NR	1 (1.5 %)
045	Placebo	72	NR	62 (86.1 %)	NR	NR	NR	10 (13.9 %)
	TQ 25-mg weekly	35	NR	26 (74.3 %)	NR	NR	NR	9 (25.7 %)
	TQ 50-mg weekly	16	NR	2 (12.5 %)	NR	NR	NR	14 (87.5 %)
	TQ 100-mg weekly	8	NR	0	NR	NR	NR	8 (100.0 %)
	TQ 200-mg weekly	18	NR	1 (5.6 %)	NR	NR	NR	17 (94.4 %)
	MQ 250-mg weekly	4	NR	0	NR	NR	NR	4 (100.0 %)
	Total	153	NR	91 (59.5 %)	NR	NR	NR	62 (40.5 %)

The percentage for each reason is based on the total number of subjects withdrawing early in each treatment group

*TQ* tafenoquine, *MQ* mefloquine, *NR* information not reported in the original studies

^a^Insufficient therapeutic effect or confirmed parasitemia

^b^Moved outside of endemic area with no reported malaria infection

### Key demographics

Table 6 shows only the key demographics of subjects from the pooled data of the three studies. Among the displayed demographic characteristics, there is no obvious imbalance between the placebo and tafenoquine treatment groups.

**Table 6:** Demographics of study participants in the mITT population—individual and pooled studies 030, 043 and 045

	Placebo				Tafenoquine 200-mg weekly				Total			
	030	043	045	Pooled	030	043	045	Pooled	030	043	045	Pooled
N	93	60	94	247	99	55	91	245	192	115	185	492
Age (years)												
Median	30	32	48	38	25	34	48	35	27	34	48	36
Min, max	17, 56	18, 55	17, 60	17, 60	17, 54	18, 54	18, 69	17, 69	17, 56	18, 55	17, 69	17, 69
Age categories (years)												
<20	18 (19.4 %)	13 (21.7 %)	5 (5.3 %)	36 (14.6 %)	19 (19.2 %)	9 (16.4 %)	4 (4.4 %)	32 (13.1 %)	37 (19.3 %)	22 (19.1 %)	9 (4.9 %)	68 (13.8 %)
20–29	25 (26.9 %)	16 (26.7 %)	14 (14.9 %)	55 (22.3 %)	40 (40.4 %)	15 (27.3 %)	6 (6.6 %)	61 (24.9 %)	65 (33.9 %)	31 (27.0 %)	20 (10.8 %)	116 (23.6 %)
30–39	23 (24.7 %)	9 (15.0 %)	9 (9.6 %)	41 (16.6 %)	17 (17.2 %)	12 (21.8 %)	20 (22.0 %)	49 (20.0 %)	40 (20.8 %)	21 (18.3 %)	29 (15.7 %)	90 (18.3 %)
40–49	16 (17.2 %)	19 (31.7 %)	27 (28.7 %)	62 (25.1 %)	12 (12.1 %)	11 (20.0 %)	19 (20.9 %)	42 (17.1 %)	28 (14.6 %)	30 (26.1 %)	46 (24.9 %)	104 (21.1 %)
≥50	11 (11.8 %)	3 (5.0 %)	39 (41.5 %)	53 (21.5 %)	11 (11.1 %)	8 (14.5 %)	42 (46.2 %)	61 (24.9 %)	22 (11.5 %)	11 (9.6 %)	81 (43.8 %)	114 (23.2 %)
Sex												
Male	59 (63.4 %)	34 (56.7 %)	62 (66.0 %)	155 (62.8 %)	65 (65.7 %)	38 (69.1 %)	59 (64.8 %)	162 (66.1 %)	124 (64.6 %)	72 (62.6 %)	121 (65.4 %)	317 (64.4 %)
Female	34 (36.6 %)	26 (43.3 %)	32 (34.0 %)	92 (37.2 %)	34 (34.3 %)	17 (30.9 %)	32 (35.2 %)	83 (33.9 %)	68 (35.4 %)	43 (37.4 %)	64 (34.6 %)	175 (35.6 %)
Height (cm)												
Median	NC	169.5	166.5	167	NC	170	166	168	NC	170	166	168
Min, max		146, 192	150, 189	146, 192		148, 194	149, 188	148, 194		146, 194	149, 189	146, 194
Weight (kg)												
Median	60	60	53	57	60.5	61	50	57	60	60	52	57
Min, max	44, 81	39, 83	35, 73	35, 83	42, 90	45, 79	35, 68	35, 90	42, 90	39, 83	35, 73	35, 90

Percentages are based on the number of study participants in each treatment group from the mITT population (N)

*Max* maximum, *Min* minimum, *mITT* modified intent-to-treat, *N* number of subjects, *SD* standard deviation

## Results

Efficacy results, from individual studies or pooled datasets, were obtained using a unified set of working definitions, as presented in the Methods section. Individual study efficacy results are summarized in Table 7 and pooled analyses are summarized in Table 8. The number of prophylactic failures includes all species of malaria.

**Table 7:** Protective efficacy based on mITT by individual study

Study	Treatment	N	# of failures	Median failure time (wk)^a^	Person-years	Incidence density^b^	PE	95 % CI lower limit	95 % CI upper limit
30	Placebo	93	30	10.1	13.26	226.2			
	Tafenoquine 200-mg weekly	99	2	NA	20.96	9.5	95.8	82.3	99
	Mefloquine 250-mg weekly	96	2	NA	17.9	11.2	95.1	79.3	98.8
43	Placebo	60	54	7.0	7.68	703.2			
	Tafenoquine 400-mg load only	57	16	NA	11.96	133.7	81	66.8	89.1
	Tafenoquine 200-mg weekly	55	7	NA	12.21	57.3	91.8	82.1	96.3
	Tafenoquine 400-mg weekly	57	6	NA	13.58	44.2	93.7	85.4	97.3
45	Placebo	94	86	4.6	9.65	891.6			
	Tafenoquine 25-mg weekly	93	58	7.7	15.32	378.7	57.5	40.7	69.6
	Tafenoquine 50-mg weekly	91	13	NA	20.67	62.9	92.9	87.4	96.1
	Tafenoquine 100-mg weekly	94	11	NA	21.99	50	94.4	89.5	97
	Tafenoquine 200-mg weekly	91	12	NA	21.27	56.4	93.7	88.4	96.5
	Mefloquine 250-mg weekly	46	6	NA	10.77	55.7	93.8	85.7	97.3

*CI* confidence interval, *mITT* modified intent-to-treat, *N* number of subjects, *PE* protective efficacy

^a^Median time for treatment groups with small number of events cannot be estimated due to the sparseness of the data

^b^Incidence density rate is expressed in terms of number of prophylactic failures per 100 person-years

**Table 8:** Protective efficacy based on mITT by pooled group

Studies pooled	Treatment	N	# of failures	Person-years	Incidence density^a^	PE	95 % CI lower limit	95 % CI upper limit
030 and 045	Placebo	187	116	22.91	506.3			
	Tafenoquine 200-mg weekly	190	14	42.23	33.2	93.5	88.6	96.2
	Mefloquine 250-mg weekly	142	8	28.67	27.9	94.5	88.7	97.3
030, 043 and 045	Placebo	247	170	30.59	555.7			
	Tafenoquine 200-mg weekly	245	21	54.44	38.6	93.1	89.1	95.6

*CI* confidence interval, *mITT* modified intent-to-treat, *N* number of subjects, *PE* protective efficacy

^a^Incidence density rate is expressed in terms of number of prophylactic failures per 100 person-years

Individual study results (Table 7) of Study 043 using the unified definitions (instead of original study definitions) are different from the previously published results in some notable ways. The number of prophylactic failures in Study 043 matched that obtained from the original efficacy results. However, six more subjects (one in the placebo arm, three in the tafenoquine 400-mg arm and two in the tafenoquine 200-mg arm) were included in the mITT set of the unified analysis. Those subjects each had one valid smear data record but with missing blood sample collection dates. For the unified analysis, they were assumed to have been collected during the prophylactic phase of the study and were included in the analysis.

Protective efficacy for the pooled analyses (Table 8) was based on the respective pooled treatment groups from each of the individual studies in the pool.

The pooling increases the sample sizes for analysis, and, as a consequence, the pooled results provided more precise estimates of the corresponding PEs (i.e., narrower CI). The PEs for the pooled analyses (Table 8) of tafenoquine load plus weekly maintenance at 200-mg were consistent with that of each of the individual studies (Table 7), although with a substantially higher lower limit for the 95 % CI of the PE. The pooled PE of tafenoquine load plus weekly maintenance at 200-mg relative to placebo for Studies 030, 043, and 045 was 93.1 % (95 % CI 89.1–95.6 %, total N = 492).

From individual study results of Studies 030 and 045, PE values did not differ appreciably between load plus weekly maintenance dose at tafenoquine 200-mg and mefloquine 250-mg. The PEs of the pooled data for tafenoquine load plus weekly maintenance at 200-mg weekly and mefloquine load plus weekly maintenance at 250-mg weekly relative to placebo for Studies 030 and 045 were 93.5 % (95 % CI 88.6–96.2 %) and 94.5 % (95 % CI 88.7–97.3 %), respectively (total N = 519). The lower confidence limits for PEs of both pooled results are practically indistinguishable at approximately 89 %.

Load plus weekly maintenance of tafenoquine at doses of 50- and 100-mg from Study 045, but not 25-mg, exhibited similar point estimates of PE. The PE of tafenoquine regimens of 3 days of loading dose at 400-mg with and without follow-up 400-mg weekly maintenance doses were 93.7 % (95 % CI 85.4–97.3 %) and 81.0 % (95 % CI 66.8–89.1 %), respectively.

**Fig. 4:**
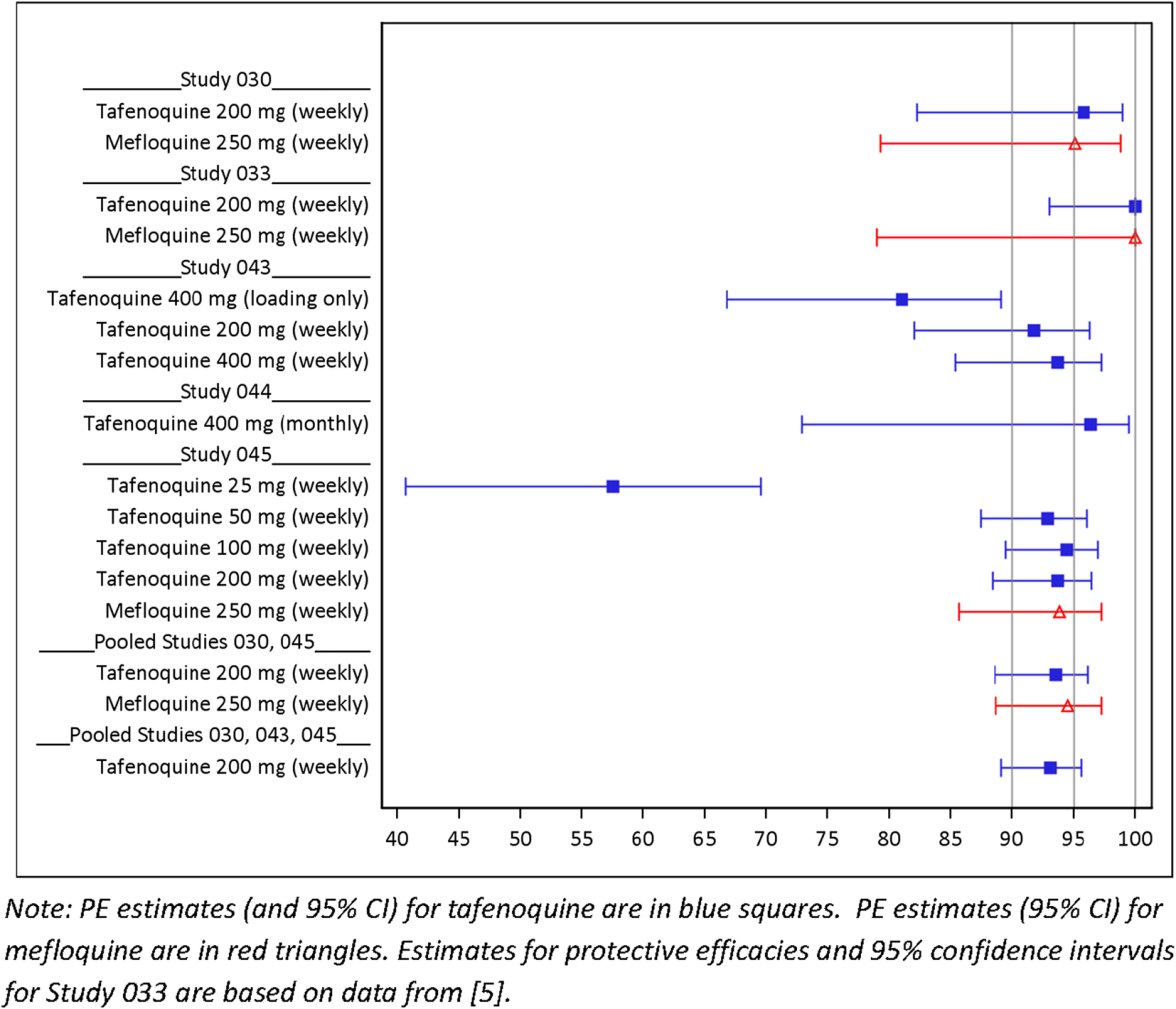
Plot of estimated protective efficacies and 95 % confidence intervals

Figure 4 shows the estimated PEs and their 95 % CIs for both the individual and pooled studies.

Table 9 provides measures of heterogeneity in PE estimates from the individual studies used in the pooled analysis (Table 8). Results in Table 9 provide strong evidence for consistency (little heterogeneity) amongst the PEs from the individual studies. As a result, a fixed effect meta-analysis model was used to provide an overall summary estimate of PE. Results of the meta-analysis for the 200-mg weekly regimen are given in Table 10. The similarities in the PE estimates (and CIs) in Table 8 (simple pooling) and Table 10 (meta-analysis using a fixed effect model) are evident. It should be noted that other meta-analysis methods (fixed or random effects) including the familiar Mantel-Haenzel method for a stratified analysis, yield similar results.

**Table 9:** Homogeneity of estimates of protective efficacy of pooled studies

Studies pooled	Treatment	Cochran’s *Q*-test		Between study variance^a^	Higgins and Thompson statistics^b^	
		*Q* Stat	P value*		*H* (95 % CI)	*I* ^*2*^ (95 % CI)
030 and 045	Placebo					
	Tafenoquine 200 mg weekly	0.26	0.61	0.00	1.00 (1.0, 1.7)	0.00 (0.0, 0.7)
030, 043 and 045	Placebo					
	Tafenoquine 200 mg weekly	0.70	0.71	0.00	1.00 (1.0, 1.4)	0.00 (0.0, 0.5)

* p value is from a Chi square test of homogeneity of PE s between studies with degree of freedom *k* − *1*, where *k* is the number of studies

^a^Between-study variance is estimated using the random effect model of DerSimonian and Laird [13]

^b^ *H* and *I* ^*2*^ calculations are based on Higgins and Thomson [8] and 95 % confidence intervals are based on Biggerstaff and Tweedie

**Table 10:** Protective efficacy based on mITT using a fixed effect model (meta analysis)

Studies pooled	Treatment	PE^a^	95 % CI lower limit	95 % CI upper limit
030 and 045	Placebo			
	Tafenoquine 200-mg weekly	94.1	89.7	96.6
030, 043 and 045	Placebo			
	Tafenoquine 200-mg weekly	93.4	89.6	95.8

*CI* confidence interval, *mITT* modified intent-to-treat

^a^Combined estimate of PE (and CI) are based on the fixed effect model of DerSimonian and Laird [13]

## Discussion

Currently available medications for malaria prophylaxis include mefloquine, doxycycline, atovaquone-proguanil and chloroquine. Because of the importance of compliance in an occupational health setting, mefloquine has historically been the preferred choice as it can be administered weekly and is effective in areas of chloroquine-resistant malaria [15, 16]. However, the association of mefloquine with adverse neuropsychiatric effects has curtailed its use (e.g., by the US military [17]). Tafenoquine is a logical alternative to mefloquine for G6PD-normal individuals as it can also be administered weekly and has activity against *P. vivax* hypnozoites.

As discussed elsewhere, traditional placebo-controlled or active-controlled (non-inferiority) trials to determine the efficacy of prophylactic drugs in the intended population, non-immune travelers, are usually not feasible [18]. A placebo-controlled trial in this population is unethical because these individuals cannot be hospitalized in a deployed setting and *P.* *falciparum* is potentially fatal [18]. Given the expected very low failure rates, a non-inferiority design will necessitate very large sample sizes for an efficacious drug [19]. The demonstration that tafenoquine at the intended dose was effective in non-immune subjects [5, 6] was due to a unique combination of circumstances that is unlikely to be repeated: fortunate timing with a large non-immune deployment to a malarious area, a study drug ready to be tested, a competent study team and a low incidence of post-exposure *P. vivax* relapses that allowed an attack rate to be determined retrospectively.

As a consequence of these challenges, the marketing applications for all other prophylactic anti-malarial agents submitted to regulators have incorporated placebo-controlled studies that enrolled residents of regions of high malaria transmission in malaria-endemic countries. In most cases, as adults, such study subjects, due to prior exposure to malaria, generally do not contract symptomatic malaria even when parasitaemic. Also, malaria prophylactic drugs are generally not used and/or are not recommended in such regions. Historically, therefore, the administration of placebo has been considered ethical, and approvals for such studies have been granted by reputable local and international institutional review boards. In such studies, an attack rate can be calculated from the placebo arm. As a consequence, the efficacy of both atovaquone-proguanil and tafenoquine, which were both developed in parallel in the 1990s, were evaluated primarily in these populations.

At the intended dose in the intended malaria naive population, tafenoquine exhibited a point estimate of prophylactic efficacy of 100 % (95 % CI 93–100 %, n = 490) compared with 100 % (95 % CI 79–100 %, n = 161) for mefloquine [5, 6]. The efficacy of tafenoquine and the similarity of its prophylactic effect to that of mefloquine were also evident from the studies conducted in high transmission areas in malaria-endemic countries that were analysed here. In the three separate placebo-controlled studies (Studies 045, 043 and 030), the intended dose was found to be more effective than placebo, and the magnitude of the prophylactic effect was similar between all three studies, whether analysed individually or pooled (Tables 7, 8). In addition, the magnitude of the prophylactic effect was similar to that of mefloquine in both studies (Studies 043 and 030) in which it was included (Tables 7, 8) and to that of mefloquine reported elsewhere (94 %).

Amongst residents of Africa, the estimates of efficacy were slightly lower for mefloquine and tafenoquine (94.5 and 93.5 %, respectively, Table 8) than in non-immune subjects. In a recent paper we speculated that the difference between the apparent efficacy is of a magnitude that could be explained by a small false positive error rate in microscopy reads in the high attack rate setting of the African studies [6]. The data also imply that doses lower than that intended might be efficacious because a loading-only regimen was partially efficacious and because weekly regimens of lower doses (50- and 100-mg) appeared to be as efficacious as the weekly 200-mg regimen and mefloquine. However, based on tafenoquine concentrations associated with symptomatic breakthroughs in Thai soldiers and non-immune North American volunteers, the proposed threshold concentration for protection in the intended population is 80 ng/ml [20]. Furthermore, trough concentrations above this level can be maintained at the intended dose, but not indefinitely with monthly administration of 400 mg following a 1200 mg loading dose. A lower dose in malaria-naïve subjects could be considered once more information about tafenoquine concentrations in association with symptomatic failures is known.

Thus far, regulatory dossiers for all prophylactic anti-malarial agents incorporate data from efficacy studies conducted in subjects resident in the high transmission zones of malaria endemic countries. Since the last time a prophylactic anti-malarial agent, atovaquone-proguanil, was approved in 2001, evolving international ethics standards have led many to argue that such studies may not be ethical [18]. This is primarily due to the perception that the residents of high malaria transmission zones do not benefit from malaria prophylaxis, as symptomatic malaria is infrequent in this population even when parasitaemia is microscopically confirmed. However, this perception may not be true to the same extent in the future as it arguably once was. As the incidence of malaria declines in endemic countries but is not eradicated, the proportion of malaria naïve and non-immune adults will increase. These individuals could in theory benefit from tafenoquine if they are traveling to an area of high transmission within their own (or other) countries. Also, since tafenoquine is the only long half-life anti-malarial with activity against all stages of the parasite, sub-chronic regimens of the drug may have a useful role for community prophylaxis drug in the context of accelerating malaria control efforts.

The most common adverse events associated with tafenoquine are known and include gastrointestinal stress, reversible asymptomatic methaemoglobinemia, reversible vortex keratopathy and, in individuals with G6PD deficiency, haemolytic anaemia [21, 22]. Safety data were collected for Study 043 and 045 and were reported individually in the original publications [2, 3]. Safety data were also collected for Study 030, and were included in the final clinical study report included here as supplemental information [1]. In a future publication, we will report on the integrated safety and tolerability of tafenoquine across multiple prophylaxis studies. We hope that that report, together with this summary of efficacy, will be sufficient to allow the regulatory, health care and scientific communities to determine whether the potential benefits of tafenoquine are worth the risk relative to alternative anti-malarials that might be used for malaria prevention in travelers or for community prophylaxis in malaria endemic countries (with the applicability of alternatives being based on jurisdiction and intended use).

## Conclusions

At the intended dose, (200 mg once per day for 3 days followed by weekly 200 mg maintenance doses), the pooled protective efficacy of tafenoquine in three placebo controlled studies was 93.1 % (95 % CI 89.1–95.6 %; total N = 492). In the two studies where mefloquine was included as a comparator arm, the efficacy of both drugs was similar. Furthermore, the efficacy of tafenoquine was dose-related and similar across three placebo-controlled studies. Collectively these studies support prior observations that tafenoquine and mefloquine have similar efficacy in malaria naïve individuals.

## Additional file

10.1186/s12936-015-0991-x Tafenoquine—SB 252263 (WR 238605). A randomized, double blind, placebo controlled evaluation of weekly tafenoquine (WR 238605/SB252263) compared to mefloquine for chemosuppression of *Plasmodium falciparum* in Western Kenya.

## Abbreviations

AMI: Australian Malaria Institute
CI: confidence interval
FDA: food and drug administration
G6PD: glucose-6-phosphate dehydrogenase
ID: identification
ITT: intent-to-treat
mITT: modified intent-to-treat
NAMRU-2: Naval Medical Research Unit 2
PE: protective efficacy

## References

[1] US Army Unpublished Clinical Study Report. A randomized, double blind, placebo controlled evaluation of weekly tafenoquine (WR 238605/SB252263) compared to mefloquine for chemosuppression of Plasmodium falciparum in Western Kenya (SB Document Number: SB-252263/RSD-101KZH/1), 2003.

[2] Hale BR, Owusu-Agyei S, Fryauff DJ, Koram KA, Adjuik M, Oduro AR (2003). A randomized, double-blind, placebo-controlled, dose-ranging trial of tafenoquine for weekly prophylaxis against Plasmodium falciparum. Clin Infect Dis.

[3] Shanks GD, Oloo AJ, Aleman GM, Ohrt C, Klotz FW, Braitman D (2001). A new primaquine analogue, tafenoquine (WR 238605), for prophylaxis against Plasmodium falciparum malaria. Clin Infect Dis.

[4] Walsh DS, Eamsila C, Sasiprapha T, Sangkharomya S, Khaewsathien P, Supakalin P (2004). Efficacy of monthly tafenoquine for prophylaxis of Plamodium vivax and multidrug-resistant P. falciparum malaria. J Infect Dis.

[5] Nasveld PE, Edstein MD, Reid M, Brennan L, Harris IE, Kitchener SJ (2010). Randomized, double-blind study of the safety, tolerability, and efficacy of tafenoquine versus mefloquine for malaria prophylaxis in nonimmune subjects. Antimicrob Agents Chemother.

[6] Dow GS, McCarthy WF, Reid M, Smith B, Tang D, Shanks GD (2014). A retrospective analysis of the protective efficacy of tafenoquine and mefloquine as prophylactic antimalarials in non-immune individuals. Malar J..

[7] Zhou G, Afrane YA, Dixit A, Atieli HE, Lee MC, Wanjala CL (2013). Modest additive effects of integrated vector control measures on malaria prevalence and transmission in western Kenya. Malar J..

[8] Aborah S, Akweongo P, Adjuik M, Atinga RA, Welanga P, Adongo P (2013). The use of non-prescribed anti-malarial drugs for the treatment of malaria in the Bolgatanga municipality, northern Ghana. Malar J..

[9] FDA. Guidance for industry malaria: developing drug and nonvaccine biological products for treatment and prophylaxis. 2007.

[10] Cochran WG (1954). The combination of estimates from different experiments. Biometrics.

[11] Higgins JPT, Thompson SG (2002). Quantifying heterogeneity in a meta-analysis. Stat Med.

[12] Hardy RJ, Thompson SG (1998). Detecting and describing heterogeneity in meta-analysis. Stat Med.

[13] DerSimonian R, Laird N (1986). Meta-analysis in clinical trials. Control Clin Trials.

[14] Mantel N, Haenszel MW (1959). Statistical aspects of the analysis of data from retrospective studies of disease. J Natl Cancer Institute.

[15] Steffen R, Fuchs E, Schildknecht J, Naef U, Funk M, Schlagenhauf P (1993). Mefloquine compared with other malaria chemoprophylactic regimens in tourists visiting east Africa. Lancet.

[16] Lobel HO, Miani M, Eng T, Bernard KW, Hightower AW, Campbell CC (1993). Long-term malaria prophylaxis with weekly mefloquine. Lancet.

[17] Department of Defense. Guidance on medications for prophylaxis of malaria. 2013.

[18] Dow GS, Magill AJ, Ohrt C (2008). Clinical development of new prophylactic antimalarial drugs after the 5th Amendment to the Declaration of Helsinki. Ther Clin Risk Manag.

[19] Hogh B, Clarke PD, Camus D, Nothdurft HD, Overbosch D, Gunther M (2000). Atovaquone-proguanil versus chloroquine-proguanil for malaria prophylaxis in non-immune travelers: a randomised, double-blind study. Malarone International Study Team. Lancet.

[20] Edstein MD, Kocisko DA, Walsh DS, Eamsila C, Charles BG, Rieckmann KH (2003). Plasma concentrations of tafenoquine, a new long-acting antimalarial agent, in Thai soldiers receiving monthly prophylaxis. Clin Infect Dis.

[21] Leary KJ, Riel MA, Roy MJ, Cantilena LR, Bi D, Brater DC (2009). A randomized, double-blind, safety and tolerability study to assess the ophthalmic and renal effects of tafenoquine 200-mg weekly versus placebo for 6 months in healthy volunteers. Am J Trop Med Hyg.

[22] Elmes NJ, Nasveld PE, Kitchener SJ, Kocisko DA, Edstein MD (2008). The efficacy and tolerability of three different regimens of tafenoquine versus primaquine for post-exposure prophylaxis of Plasmodium vivax malaria in the Southwest Pacific. Trans R Soc Trop Med Hyg.

