# Supplementary material for: Summary of anti-malarial prophylactic efficacy of tafenoquine from three placebo-controlled studies of residents of malaria-endemic countries
Source: Malar J. 2015 Nov 26;14:473. doi: 10.1186/s12936-015-0991-x (PMC4661991; doi:10.1186/s12936-015-0991-x)
Supplement: Supplementary file 1 — 10.1186/s12936-015-0991-x Tafenoquine—SB 252263 (WR 238605). A randomized, double blind, placebo controlled evaluation of weekly tafenoquine (WR 238605/SB252263) compared to mefloquine for chemosuppression of Plasmodium falciparum in Western Kenya. [file 12936_2015_991_MOESM1_ESM.pdf]

Confidential

SmithKline Beecham

Tafenoquine

SB 252263 (WR 238605)

A Randomized, Double Blind, Placebo Controlled Evaluation of Weekly Tafenoquine (WR 238605/SB252263) Compared to Mefloquine for Chemosuppression of *Plasmodium falciparum* in Western Kenya

030

Final Clinical Report

Lorraine Martin BSc*, Philip Pickford PhD*,
Rachel Moate MSc**, Josephine Bartlett MSc***

*Anti-Infective Therapeutic Unit SB, **Biostatistics and Data Sciences SB,
***Broadway Medical Limited

SB Document Number: SB-252263/RSD-101KZH/1

Issue Date: 14th March 2003

Signature Page

Report Title: A Randomized, Double Blind, Placebo Controlled Evaluation of Weekly Tafenoquine (WR 238605/SB252263) Compared to Mefloquine for Chemosuppression of *Plasmodium falciparum* in Western Kenya

Names of Report Authors: Lorraine Martin BSc, Philip Pickford PhD, Rachel Moate MSc, Josephine Bartlett MSc

I have read this report and confirm that to the best of my knowledge it accurately describes the conduct and results of the study.

Didier Lapierre, MD<br>Vice President<br>MDC Infectious Diseases<br>GlaxoSmithKline	Date

Report Synopsis

Study Title

A Randomized, Double Blind, Placebo Controlled Evaluation of Weekly Tafenoquine (WR 238605/SB252263) Compared to Mefloquine for Chemosuppression of *Plasmodium falciparum* in Western Kenya
Study number: SB 252263/030

Investigator and Centre

The study was conducted at Kombewa Clinic, Kenya, with Dr Jose Stoute as Principle Investigator, United States Army Medical Research Unit – Kenya (USAMRU-K).

Publication

None at issue.

Study Dates

The first dose of prophylaxis study medication was taken on 8 May 2000 and the last dose of prophylaxis medication was taken on 25 October 2000.

Objectives

The primary study objective was to determine the chemoprophylactic efficacy of weekly tafenoquine in preventing falciparum parasitaemia compared to placebo.

The secondary study objectives were (i) to compare the safety and tolerability of weekly tafenoquine given for 24 weeks with that of placebo; (ii) to provide comparative data on the effectiveness, safety and tolerability of tafenoquine versus mefloquine; (iii) to determine the impact of chemoprophylaxis with mefloquine and tafenoquine on the subsequent susceptibility to malaria; (iv) if an increase in the susceptibility to malaria was observed following prophylaxis, to identify the immune responses that were most affected by each regimen; (v) to characterise the population pharmacokinetics of tafenoquine and evaluate the effects of various subject characteristics on tafenoquine pharmacokinetics. Due to the early withdrawal rate the immunological follow-up, i.e. objectives (iii) and (iv), was not carried out and the study completed after 24 weeks plus follow-up rather than the original 70 weeks. The results of the pharmacokinetic analysis, i.e. objective (v), are to be presented in a separate report.

Study Design

This was a placebo controlled, randomised, double-blind, double-dummy study of the efficacy of weekly tafenoquine compared with weekly mefloquine or placebo in the chemosuppression of *P. falciparum* in Nyanza Province, western Kenya. Subjects who met the study entry criteria were treated for three days with halofantrine to clear any existing parasitaemia. At the end of the clearance period, subjects free from malaria parasitaemia were randomised and received a loading dose of study treatment (tafenoquine 200 mg, mefloquine 250 mg or placebo) for three days, followed by study treatment (tafenoquine 200 mg, mefloquine 250 mg or placebo, respectively) once a week for 24 weeks. After the treatment period subjects attended weekly follow-up safety visits until Week 28.

Study Population

Healthy volunteers of either sex aged between 18 and 55 years planning to reside in the study area for the entire study period of approximately 70 weeks were eligible for entry into the study.

Treatment and Administration

Subjects received tafenoquine 200 mg base, mefloquine 250 mg base or placebo, once daily for three days, then once weekly for 24 weeks. Batch nos: N99354 (tafenoquine); N00061 (tafenoquine placebo); N00067 (mefloquine); N99330 (mefloquine placebo).

Evaluation Criteria

Efficacy Parameters

The primary efficacy variable was prophylactic outcome (success/failure) at the end of the prophylactic treatment phase (time of last dose, Week 24, plus 7 days); prophylactic outcome was based on absence/presence of asexual stage parasites of any *Plasmodium* species on a single blood smear. The secondary efficacy variables were: number of subjects with two consecutive positive smears during prophylactic treatment; number of subjects with a first positive smear (*P. falciparum* species only) during prophylactic treatment; time to first positive smear; time to two consecutive positive smears. (Secondary efficacy analyses from results during the safety follow-up period were not performed due to the high early withdrawal rate from the study.)

Safety Parameters

Adverse experiences, vital signs and laboratory results were collected to assess safety.

Statistical Methods

The primary efficacy analysis was based on a calculation of the protective efficacy (PE) of tafenoquine, defined as (1 – relative risk of developing parasitaemia tafenoquine:placebo)x100%, and 95.2% confidence intervals were constructed for the relative risk using Koopman's method. The confidence intervals were adjusted from the 95% specified in the protocol due to an interim analysis by the Independent Data Monitoring Committee. The primary comparison of interest was the PE of tafenoquine compared with placebo. The PE of mefloquine was also calculated but no formal statistical tests of comparison were used as the study was not powered to detect differences between tafenoquine and mefloquine.

The PEs of tafenoquine and mefloquine based on the secondary endpoints were also calculated, together with 95% confidence intervals. Kaplan-Meier survival curves were produced for time to parasitaemia for both first positive smear and two consecutive positive smears.

Subject Disposition and Key Demographic Data

Subject disposition is shown below.

**Population**	**Treatment Group**			**Total**
	**Placebo**	**Tafenoquine 200 mg**	**Mefloquine 250 mg**	
Screened	-	-	-	517
Randomised	101	104	101	306
Intent-to-treat population	99	102	99	300
Completed study	1	7	4	12
Per protocol population	92	94	90	276
Safety population	101	104	101	306

Almost all premature withdrawals from the study were as a result of observed parasitaemia (see later under High Failure Rate).

Demographic data are shown below for the intent-to-treat population.

**Demographic Characteristic**		**Treatment Group**		
		**Placebo** **N=99**	**Tafenoquine 200 mg N=102**	**Mefloquine 250 mg N=99**
**Sex n(%)**	male	63 (63.6%)	66 (64.7%)	66 (66.7%)
	female	36 (36.4%)	36 (35.3%)	33 (33.3%)
**Age (years)**	mean (sd)	32.0 (11.9)	29.5 (11.2)	29.4 (10.4)
	range	17 – 56	17 – 54	17 – 55
**Race n(%)**	Black	99 (100%)	102 (100%)	99 (100%)

Efficacy Results

High Failure Rate

Early in the study it it became apparent that the failure rate was higher than would be expected for a design including two effective chemoprophylactic treatment arms. This lead to the initiation of a series of investigations into the possible cause, plus the set-up of an Independent Data Monitoring Committee (IDMC) to assess whether it was appropriate to continue the study.

Drug supply packaging and stability were found to be in order, and analysis of blood samples showed tafenoquine at target levels. A re-read of a sample of study slides by two separate, experienced, slide-reading centres, the Naval Medical Research Unit-2 (NAMRU-2), Jakarta, and the Australian Army Malaria Institute, Brisbane, suggested high rates of false positives were being identified at the study site. In parallel, and in concert with this conclusion, the IDMC conducted an unblinding of parasitaemia results from the centre and identified low apparent protective efficacies for both tafenoquine and mefloquine groups. Taking this into consideration, plus a review of serious adverse event data from the study, the IDMC concluded that the study should continue in order to meet secondary objectives of evaluating the long-term safety and efficacy of tafenoquine.

A comparison of the original slide reading as performed at USAMRU-K with the result of the re-reading performed at NAMRU-2 is shown below for all re-read slides, including those re-read after study completion, irrespective of treatment group.

	**NAMRU-2 Reading**		
**USAMRU-K Reading**	**Positive**	**Negative**	**Total**
Positive	31	219	250
Negative	5	507	512
Total	36	726	762

Assuming that the NAMRU-2 readings were correct, for all re-read slides the sensitivity (the proportion of true positives correctly identified by USAMRU-K) was 86.1% (31/36) and the specificity (the proportion of true negatives correctly identified) was 69.8% (507/726). The overall concordance rate was 70.6% (538/762), with the negative concordance rate being 99% (507/512) and the positive concordance rate being only 12.4% (31/250).

As a result of these findings the efficacy results and analyses from this study cannot be considered a true reflection of the chemoprophylactic efficacy of tafenoquine in this trial setting.

Data Sets

The principal efficacy analysis was based on the intention-to-treat population and the per protocol population was used to confirm the findings of the principal analysis.

Primary Efficacy Variable

Prophylactic outcome and protective efficacy (PE) based on first positive smear (all species) during prophylactic treatment is summarised below for the intent-to-treat population. As expected following the slide-reading errors, PEs for both tafenoquine and mefloquine relative to placebo are low.

Prophylactic Outcome	Treatment Group		
	Placebo<br>N=99	Tafenoquine 200 mg<br>N=102	Mefloquine 250 mg<br>N=99
Prophylactic failure	93 (93.9%)	90 (88.2%)	92 (92.9%)
Prophylactic success (total*)	6 (6.1%)	12 (11.8%)	7 (7.1%)
Prophylactic success (known**)	0	6 (5.9%)	0
Prophylactic success (assumed†)	6 (6.1%)	6 (5.9%)	7 (7.1%)
PE†† (%)	-	6.1%	1.1%
95.2% CI	-	-2.8%, 15.0%	-7.4%, 9.1%

* Total success = known successes + assumed successes.
** Known success = subject had negative smears up to and including Week 24.
† Assumed success = subject had negative smears throughout the study but withdrew prior to Week 24.
†† Protective efficacy relative to placebo based on failure rate.

Secondary Efficacy Variables

Protective efficacy based on two consecutive positive smears (all species) and on first positive smear (*P. falciparum* only) during prophylactic treatment is summarised below for the intent-to-treat population. Protective efficacies are higher using this endpoint but cannot be regarded as a valid result due to slide-reading errors. The results for protective efficacy of tafenoquine based on *P. falciparum* only were similar to those for any species.

Protective Efficacy	Treatment Group		
	Placebo<br>N=99	Tafenoquine 200 mg<br>N=102	Mefloquine 250 mg<br>N=99
**Two consecutive positive smears**			
PE†† (%)	-	77.9%	56.8%
95% CI	-	59.7%, 88.2%	32.5%, 72.9%
***P. falciparum* only**			
PE†† (%)	-	6.1%	0
95% CI	-	-3.2%, 15.3%	-8.9%, 8.2%

†† Protective efficacy relative to placebo based on failure rate.

The time to first positive smear and two consecutive positive smears is summarised below for the intent-to-treat population.

Protective efficacy based on two consecutive positive smears (all species) and on first positive smear (*P. falciparum* only) during prophylactic treatment is summarised below for the intent-to-treat population. Protective efficacies are higher using this endpoint but cannot be regarded as a valid result due to slide-reading errors. The results for protective efficacy of tafenoquine based on *P. falciparum* only were similar to those for any species.

**Time to Smear (days)**	**Treatment Group**		
	**Placebo**<br><br>**N=99**	**Tafenoquine 200 mg**<br>**N=102**	**Mefloquine 250 mg**<br>**N=99**
**First positive smear**			
Median	43	57	50
95% CI	42, 50	53, 70	44, 64
**Two consecutive positive smears**			
Median	63	NE	165
95% CI	57, 79	-	154, NE

NB NE = no estimate available

Although there was a statistically significant difference between the survival curves for the tafenoquine and placebo groups for the time to first positive smear and the time to two consecutive positive smears ($p<0.01$), the results should be treated with caution as almost all subjects had at least one positive smear by the end of the study. Although in most survival analyses delaying the onset of the event is a successful outcome, for malaria prophylaxis the primary objective is to prevent people from experiencing the event at all.

Safety Results

Adverse Experiences

The most commonly reported experiences (i.e. those occurring in at least 20% of subjects in any treatment group) are summarised below for the safety population.

**Adverse Experience**	**Treatment Group**		
	**Placebo**<br><br>**N=101**	**Tafenoquine 200 mg**<br>**N=104**	**Mefloquine 250 mg**<br>**N=101**
At least one AE	92 (91.1%)	98 (94.2%)	97 (96.0%)
Headache	37 (36.6%)	45 (43.3%)	49 (48.5%)
Upper respiratory tract infection	17 (16.8%)	30 (28.8%)	26 (25.7%)
Back pain	12 (11.9%)	28 (26.9%)	10 (9.9%)
Myalgia	25 (24.8%)	27 (26.0%)	29 (28.7%)
Abdominal pain	24 (23.8%)	25 (24.0%)	19 (18.8%)
Coughing	13 (12.9%)	24 (23.1%)	29 (28.7%)
Rhinitis	21 (20.8%)	12 (11.5%)	20 (19.8%)

Most adverse experiences were of mild or moderate intensity. Headache was the most commonly reported adverse experience in all treatment groups. Amongst Central Nervous System adverse experiences, dizziness appeared to be reported at slightly higher incidences in both the tafenoquine (11.5%) and mefloquine (13.9%) groups compared to placebo (6.9%).

Serious Adverse Experiences

There were no deaths reported during the study, either during the prophylaxis treatment phase or during follow-up.

Eleven subjects (3.6%) had serious adverse experiences during the prophylaxis treatment phase and 5 subjects (1.6%) had serious adverse experiences in the follow-up period. Only two subjects had serious adverse experiences reported with a suspected relationship to study treatment: a subject in the tafenoquine group with haemolytic anaemia and a subject in the mefloquine group with anxiety.

Withdrawals Due to Adverse Experiences

Thirteen subjects had adverse experiences recorded as leading to withdrawal: 10 (3.3%) during the prophylaxis treatment phase and 5 (1.6%) during follow-up (two subjects had events during both phases). Most of these withdrawals were due to apparent parasitaemia, with the reason for withdrawal from the study being given as ‘insufficient therapeutic effect’. Two subjects were withdrawn from the study due to adverse experiences with a suspected relationship to study treatment – these are noted under Serious Adverse Experiences above.

Laboratory Tests

In general, there were only limited changes from baseline in mean laboratory values during the prophylactic treatment period and few marked differences between treatment groups. However, there did appear to be trends towards small mean decreases from baseline in haemoglobin, haematocrit and platelet counts in the tafenoquine group compared to the placebo and mefloquine groups. In clinical chemistry parameters, there appeared to be small mean increases from baseline in creatinine in both the tafenoquine and mefloquine groups at Week 4 compared to placebo with a tendency to return back towards baseline values with continued treatment.

Vital Signs

There were no results of clinical significance and no marked differences between the treatment groups.

Conclusions

- A failure of the slide-reading process in this study lead to an inability to assess the primary objective ie. determining the chemoprophylactic efficacy of tafenoquine against *P.falciparum* infection. Measures will have to be taken to ensure the quality of slide-reading in future Phase III studies.

- Tafenoquine was well tolerated with the most frequently reported adverse experience being mild or moderate headache.

- There was some evidence of small decreases in laboratory measures of haemoglobin, haematocrit and platelets on tafenoquine therapy, and small and transient increases in creatinine. None of these reached clinical relevance, apart from a single case of investigator-diagnosed haemolytic anaemia reported as a serious adverse experience; this in spite of the subject testing normal for G6PD status.

Table of Contents

Report Synopsis 3
1 Introduction 22
2 Objectives 25
2.1 Primary Objective 25
2.2 Secondary Objectives 25
3 Methodology 26
3.1 Study Design 26
3.1.1 Protocol Amendments 27
3.2 Investigators 29
3.3 Ethics 29
3.4 Eligibility Criteria 30
3.4.1 Inclusion Criteria 30
3.4.2 Exclusion Criteria 30
3.5 Study Medication and Administration 31
3.5.1 Study Medication 31
3.5.2 Dosage and Administration 32
3.5.3 Other Protocol Specified Therapy 33
3.6 Compliance with Study Medication 33
3.7 Prior and Concomitant Medication 33
3.8 Study Procedures 34
3.8.1 Schedule of Assessments 34
3.8.2 Prestudy Screening and Enrollment 36
3.8.3 Baseline Phase 36
3.8.4 Treatment Phase(s) 37
3.8.5 Post-treatment Phase 38
3.8.6 Reasons for Concluding Study 39
3.9 Efficacy Assessments 40
3.9.1 Evaluation of Malaria 40
3.9.2 Primary Efficacy Parameter 41
3.9.3 Secondary Efficacy Parameters 41
3.9.4 Other Efficacy Parameters 41
3.9.5 Investigations into High Failure Rate 42
3.9.5.1 Drug packaging and supplies 42
3.9.5.2 Slide Reading 42
3.9.5.3 Pharmacokinetic samples 43
3.9.5.4 Parasite Resistance Testing 44
3.10 Safety Assessments 44

3.10.1 Adverse Experiences ... 44
3.10.2 Pregnancy ... 45
3.10.3 Physical Examination, Vital Signs and ECG ... 46
3.10.4 Laboratory Tests ... 46
3.11 Pharmacokinetic Assessments ... 47
3.11.1 Sampling Times ... 47
3.11.2 Specimen Preparation ... 47
3.11.3 Pharmacokinetic Analysis ... 48
3.12 Data Quality Assurance ... 48
3.13 Statistical Evaluation ... 49
3.13.1 Target Sample Size ... 49
3.13.2 Method of Randomization ... 49
3.13.3 Planned Efficacy Evaluations ... 49
3.13.3.1 Primary Efficacy Evaluation ... 49
3.13.3.2 Secondary Efficacy Evaluations ... 50
3.13.3.3 Other Efficacy Evaluations ... 51
3.13.4 Methods of Analysis ... 51
3.13.4.1 Primary Efficacy Analysis ... 51
3.13.4.2 Secondary Efficacy Analysis ... 53
3.13.4.3 Other Efficacy Analysis ... 54
3.13.4.3 Other Efficacy Analysis ... 56
3.13.4.4 Interim Analysis and IDMC Review ... 56
3.13.5 Populations/Data Sets to be Evaluated ... 57
3.13.6 Safety Evaluations ... 58
4 Study Population ... 63
4.1 Study Dates ... 63
4.2 Subject Disposition ... 63
4.2.1 Number and Distribution of Subjects ... 63
4.2.2 Number of Subjects Present at Each Visit ... 64
4.2.3 Withdrawal Reasons ... 65
4.3 Protocol Violations ... 67
4.4 Demographic and Baseline Characteristics ... 68
4.4.1 Demographic Characteristics ... 68
4.4.2 Baseline Characteristics ... 70
4.4.2.1 Clinical Characteristics ... 70
4.4.2.2 Parasitology ... 72
4.5 Presenting Conditions and Medical History ... 74
4.5.1 Medical/Surgical History ... 74
4.5.2 Pregnancy Screen ... 75
4.5.3 G6PD Status ... 75

4.6 Baseline Signs and Symptoms ... 76
4.7 Prior and Concomitant Medications ... 76
4.7.1 Prior Medication ... 76
4.7.2 Concomitant Medication ... 77
4.7.3 Follow-up Medication ... 78
4.8 Treatment Compliance ... 78
5 Efficacy Results ... 79
5.1 Efficacy Evaluation ... 79
5.1.1 Data Sets Analysed ... 79
5.2 Primary Efficacy Parameters ... 79
5.2.1 Principal Analysis ... 80
5.2.2 Confirmatory Analyses ... 80
5.3 Secondary Efficacy Parameters ... 82
5.3.1 Two Consecutive Positive Smears During Prophylactic Treatment ... 82
5.3.2 First Positive Smear (P. *falciparum* only) During Prophylactic Treatment ... 83
5.3.3 Time to First Positive Smear ... 84
5.3.4 Time to Two Consecutive Positive Smears ... 86
5.4 Other Efficacy Variables ... 87
5.4.1 First Positive Smear by Species ... 87
5.4.2 Malaria Status ... 89
6 Investigations into High Failure Rate During Study ... 89
6.1 IDMC Review of Safety and Efficacy ... 89
6.2 Technical Investigations ... 90
6.2.1 Re-reading of Slides-during study ... 90
6.2.2 Sensitivity and Specificity of Slide Readings ... 91
6.2.3 Parasite Resistance ... 92
6.2.4 Drug Packaging ... 92
6.2.5 Analysis of Pharmacokinetic Samples ... 92
6.2.6 Procedural Errors ... 92
7 Safety Results ... 93
7.1 Extent of Exposure ... 93
7.2 Adverse Experiences ... 95
7.2.1 Continuing Baseline Signs and Symptoms ... 95
7.2.2 Adverse Experiences by Frequency ... 95
7.2.3 Adverse Experiences by Relationship to Study Medication ... 97
7.2.4 Adverse Experiences by Severity ... 98
7.2.5 Adverse Experiences by Time of First Occurrence ... 100
7.3 Deaths ... 102
7.4 Serious Adverse Experiences ... 102

7.5 Withdrawals Due to Adverse Experiences 104
7.6 Pregnancy 106
7.7 Vital Signs 107
7.8 Laboratory Tests 109
8 Pharmacokinetic Evaluation 112
9 Discussion 112
10 Conclusions 117
11 References 117
12 Source Tables: Study Population 120
13 Source Tables: Efficacy Results 122
14 Source Tables: Safety Results 124
15 Source Figures 126
Appendices 127

List of Tables

Table 1 Appearance, formulation and dosage strength of drugs used 32
Table 2 Outline of study procedures and assessments... 35
Table 3 Sponsor defined criteria for evaluating vital signs.................................. 60
Table 4 Sponsor defined criteria for evaluating laboratory variables.................. 62
Table 5 Subject disposition (number of subjects).. 63
Table 6 Subjects excluded from the intent-to-treat population: all randomised subjects.. 64
Table 7 Number (%) of subjects assessed at each visit on-dose: intent-to-treat population.. 65
Table 8 Reason for study conclusion: intent-to-treat population (number (%) of subjects) 66
Table 9 Cumulative proportion of subjects withdrawn from the study: intent-to-treat population 67
Table 10 Protocol violations leading to exclusion from the per-protocol population (number (%) of subjects) 68
Table 11 Demographic characteristics: intent-to-treat population....................... 69
Table 12 Demographic characteristics: per-protocol population......................... 70
Table 13 History of malaria: intent-to-treat population....................................... 71
Table 14 History of malaria: per-protocol population 72
Table 15 Baseline parasitology: all randomised subjects (number (%) of subjects)73
Table 16 Parasite count at screening: intent-to-treat population.......................... 74
Table 17 Most commonly occurring (in ≥5% of subjects overall) active conditions at screening: intent-to-treat population (number (%) of subjects)....... 75
Table 18 Most commonly occurring (in ≥2% of subjects overall) baseline signs and symptoms: safety population (number (%) of subjects)................................. 76
Table 19 Most frequently received (by ≥10% of subjects in any treatment group) concomitant medication: intent-to-treat population (number (%) of subjects)..... 77
Table 20 Most Commonly Received Medication During Follow-up 78
Table 21 Study medication compliance: intent-to-treat population (number (%) of subjects) 79
Table 22 Prophylactic outcome and protective efficacy during the prophylaxis treatment period: intent-to-treat population (number (%) of subjects)................. 80
Table 23 Prophylactic outcome during the prophylaxis treatment period: per-protocol population (number (%) of subjects) 81

Table 24 Protective efficacy based on incidence density during the prophylaxis treatment period: intent-to-treat population 81
Table 25 Prophylactic outcome (worst case) during the prophylaxis treatment period: intent-to-treat population (number (%) of subjects) 82
Table 26 Prophylactic outcome (two consecutive positive smears) and protective efficacy during the prophylaxis treatment period: intent-to-treat population (number (%) of subjects) 83
Table 27 Prophylactic outcome (P. *falciparum* only) and protective efficacy during the prophylaxis treatment period: intent-to-treat population (number (%) of subjects) 84
Table 28 Time to first positive smear: intent-to-treat population 84
Table 29 Time to two consecutive positive smears: intent-to-treat population ... 86
Table 30 First positive smear by *Plasmodium* species: intent-to-treat population (number of subjects) 88
Table 31 Parasite count at first positive smear: intent-to-treat population 88
Table 32 Comparison of USAMRU-K and NAMRU-2 slide readings 90
Table 33 Comparison of USAMRU-K and NAMRU-2 slide readings: all re-read slides 91
Table 34 Extent of exposure to prophylactic study medication: safety population (number (%) of subjects) 94
Table 35 Number (%) of subjects with the most frequently reported adverse experiences (≥10% of subjects in any treatment group): safety population 96
Table 36 Number (%) of subjects with CNS/psychiatric adverse experiences: safety population 97
Table 37 Number (%) of subjects with the most frequently reported adverse experiences (≥2 subjects in any treatment group) related to study treatment: safety population 98
Table 38 Number (%) of subjects with adverse experiences by intensity: safety population 99
Table 39 Number (%) of subjects with severe adverse experiences: safety population 99
Table 40 Number (%) of subjects with most frequently reported adverse experiences by time of first occurrence: safety population 101
Table 41 Number (%) of subjects with serious adverse experiences: safety population 103
Table 42 Number (%) of subjects with adverse experiences leading to withdrawal: safety population 105
Table 43 Mean vital signs at baseline and mean change from baseline: safety population 108

Table 44 Number (%) of subjects with vital signs of potential clinical concern: safety population 109
Table 45 Mean Change from Baseline by Visit 110
Table 46 Number (%) of subjects with F2 flagged laboratory values during the prophylactic period: safety population 111

List of Figures

Figure 1 Study design 27
Figure 2 Time to first positive smear: intent -to- treat population....................... 85
Figure 3 Time to two consecutive positive smears: intent-to-treat population.... 87

List of Appendices

Appendix A: Protocol, Sample Blank CRF, Investigator CV, Randomisation Code, Certificates of Analysis 127
Appendix B: Subject Data Listings of Demographic and Baseline Characteristics128
Appendix C: Subject Data Listings of Efficacy.. 129
Appendix D: Subject Data Listings of Adverse Experiences 130
Appendix E: Subject Data Listings of Vital Signs... 131
Appendix F: Subject Data Listings of Laboratory Tests.................................... 132
Appendix G: CRF Copies for Deaths, Serious Adverse Experiences and Withdrawals due to Adverse Experiences.. 133

List of Abbreviations & Definitions

Abbreviation	Unabridged Term
AE	adverse experience
ALT (SGPT)	alanine transaminase (serum glutamic-pyruvic transaminase)
AMI	Army Malaria Institute in Australia
ART	Adverse Reaction Terminology
AST (SGOT)	aspartate transaminase (serum glutamic-oxalic transaminase)
ATC	Anatomical Therapeutic Chemical
BP	blood pressure
bpm	beats per minute
°C	degree Celsius
CFR	Code of Federal Regulations
CI	confidence interval
CRF	case report form
CTS-P	Clinical Trial Supplies Packaging
dL	decilitre
DMPK	Drug Metabolism and Pharmacokinetics
EDTA	ethylenediaminetetraacetic acid
EU CPMP	European Union Committee for Proprietary Medicinal Products
g	gram
G6PD	glucose-6-phosphate dehydrogenase
HCG	human chorionic gonadotrophin
IDMC	Independent Data Monitoring Committee
ITT	intent-to-treat
KEMRI	Kenya Medical Research Institute
kg	kilogram
L	litre
mg	milligram
min	minute
mL	millilitre
mmHg	millimetres of mercury
NAMRU-2	Naval Medical Research Unit 2, Jakarta
ng	nanogram
NRH	normal range high
NRL	normal range low
NRS	normal range span
PBMC	peripheral blood mononuclear cell
PE	protective efficacy

**Abbreviation**	**Unabridged Term**
PP	per protocol
SB	SmithKline Beecham Pharmaceuticals
sd	standard deviation
SOP	standard operating procedure
spp	species
ug	microgram
uL	microlitre
U/L	units/litre
umol	micromole
URTI	upper respiratory tract infection
US/USA	United States of America
USAMRMC	United States Army Medical Research and Materiel Command
USAMRU-K	United States Army Medical Research Unit – Kenya
WBC	white blood cell
WHO	World Health Organisation

1 Introduction

Malaria is a leading cause of morbidity and mortality in many developing countries with an estimated 100 to 300 million infections worldwide and 1.0-1.5 million deaths. *Plasmodium falciparum* is considered the most important malaria parasite because it causes high morbidity and mortality particularly in Africa, Southeast Asia and the tropical zones of the Americas [1]. Malaria has been, and continues to be a disease of military concern throughout the history of the US Armed Forces until the present day [2].

The sporozoite form of the *P. falciparum* parasite is transmitted throughout the saliva of *Anopheles* mosquitoes as they feed on human blood during bites. Within minutes of inoculation, the sporozoites travel through the blood and invade the liver where they undergo asexual division and mature [3]. The pre-patent period (the time between the mosquito bite and the first appearance of plasmodia in the peripheral blood) for the *P. falciparum* parasite in man normally ranges between 9 and 12 days although pre-patent periods in man as short as 5 days and as long as 25 days have been reported [4]. From the liver, merozoites are released into the blood and invade the erythrocytes, where they develop into schizonts. In the case of *P. falciparum* malaria there are no residual parasites in the liver after the initial cycle of entry, division, maturation and release. Drugs active in the exoerythrocytic stage (i.e. the hepatic stage) of the parasites disrupt the life-cycle of the parasites, preventing parasitaemia, systemic illness and further transmission. Drugs active on the erythrocytic schizonts are called blood schizontocides and are used to treat clinically apparent malaria infections, or as a suppressive prophylactic drug, which prevents clinical symptoms by destroying schizonts before they can cause illness.

There are currently no effective vaccines to prevent malaria infections. For non-immune individuals, drugs such as chloroquine, pyrimethamine-sulfadoxine, mefloquine and doxycycline have been used successfully in the past to prevent malaria infections, but the advent of chloroquine-resistant strains of *P. falciparum* and *Plasmodium vivax* malaria parasites has necessitated the development of new drugs which can be administered prophylactically to prevent malaria in those exposed to the parasite.

At present, the principal drugs available for malaria prophylaxis are mefloquine and doxycycline. Mefloquine can be given weekly and is usually recommended for travellers to sub-Saharan Africa because of its high efficacy and weekly dosage regimen which is thought to enhance compliance. Mefloquine resistance

is widespread in SE Asia and has been reported in Africa as well [5][6]. Mefloquine is used as suppressive prophylaxis against the erythrocytic stages of *P. falciparum* as well as *P. vivax* malaria. Doxycycline 100 mg daily has been shown to be effective in western Kenya but it cannot be given to children and pregnant women and often has distressing gastrointestinal adverse effects.

Proguanil combinations such as atovaquone/proguanil (Malarone®, Glaxo Wellcome) have been shown effective against intense falciparum malaria exposure in Gabon, Zambia and western Kenya [7][8] [9] requiring a daily dosing regimen.

Tafenoquine, SB 252263/WR 238605, is a synthetic primaquine analogue in development by the US Army and SmithKline Beecham as a chemoprophylactic agent against all forms of malaria. In preclinical models it has shown activity against both the erythrocytic stages and the hepatic stages of the parasite [10] [11]. At the time of conduct of the current study, it had undergone extensive preclinical toxicology studies and microbiology assessments and progressed through single and multiple dose rising Phase I volunteer studies [12]. In 1997, 249 subjects in Kenya participated in a randomised, double-blind Phase II study assessing a tafenoquine 200mg three day loading dose followed by 200 mg weekly, tafenoquine 400mg three day load followed by 400mg weekly and tafenoquine 400mg administered as a three day load only dosing regime; these were compared to a placebo arm and followed for 12 weeks [13]. Tafenoquine was shown to be highly efficacious ($\geq$ 86% protective) in preventing *P.falciparum* malaria parasitaemia in both weekly dosing regimens on a background of a 92% attack rate in the placebo group. In a subsequent study conducted in Ghana in 1998, tafenoquine was assessed in a double-blind, randomised manner against placebo using a 3 day load-plus weekly regimen at a range of doses from 25mg – 200 mg base [14]. This showed high protective efficacies relative to placebo, in the range of 84-87%, in this high transmission area.

In Phase II studies the drug has been shown to be generally safe and well tolerated, with some evidence of limited gastrointestinal disturbance. Of note, in the 1997 Kenyan study, two women deficient in glucose-6-phosphate dehydrogenase (G6PD) who were entered into the study in error, subsequently experienced haemolysis on tafenoquine therapy, thereby confirming this as a class-effect of the 8-aminoquinolines.

The need to develop new drugs and new regimens of already licensed antimalarial drugs is driven by the introduction of large numbers of non-immune persons into areas of high malaria endemicity. Prophylaxis or the prevention of symptomatic

malaria infections has an important role for Kenya in that epidemics of falciparum malaria are now endangering a new non-immune population as previously non-malarious areas develop seasonal transmission [15]. Medications such as tafenoquine that have the ability to block the further transmission of malaria infections may have a particular role in combating epidemic malaria.

The study site was Kombewa village in Nyanza Province, Kenya, about 25 km from the town of Kisumu on the shore of Lake Victoria in East Africa. Malaria in this region is holoendemic and representative of the general situation in sub-Saharan Africa. Its chief characteristic is intense transmission via *Anopheles gambiae*. Malaria transmission occurs at a low level all year but at a very high level during the two rainy seasons: the “long” rains from April to June producing intense transmission from May to July; the “short” rains of August to October producing another transmission season from September to November. Cumulative malaria attack rates have been in the order of 95% during the “long” and 75% during the “short” rains. Over several years of study, the adult population has been shown to have approximately 90% incidence of malaria parasitaemia over 10 weeks during a high transmission period. Adults permanently living in the study area have all had multiple malaria infections and should be considered at least semi-immune.

Studies involving a 3-day loading regimen followed by weekly prophylaxis performed to date (see studies in Kenya and Ghana described above) indicated that doses of tafenoquine of 100 mg and above were highly efficacious in prevention of malaria in a semi-immune population in intensely endemic conditions. From a safety and tolerability standpoint, the main issue for tafenoquine appeared to be gastrointestinal side effects and there was evidence that these were dose related. In choosing a dose of tafenoquine for Phase III, consideration had to be given to selecting the highest well tolerated dose of tafenoquine – since a higher dose of the drug would confer better protection in non-immune users of the drug and tend to protect against the development of resistance. It was therefore proposed to investigate a dosing regime of 200 mg of tafenoquine as a 3-day load followed by weekly treatment, as the optimal balance between efficacy and tolerability, in Phase III trials. The mefloquine regimen (loading of 750 mg over 3 days and then 250 mg/week) is known to be safe and well tolerated from studies carried out in US Marines and Indonesian soldiers [16] [17].

2 Objectives

2.1 Primary Objective

The primary study objective was to determine the chemoprophylactic efficacy of weekly tafenoquine in preventing *P. falciparum* parasitaemia compared to placebo.

2.2 Secondary Objectives

The secondary study objectives were as follows.

1. To compare the safety and tolerability of weekly tafenoquine given for 24 weeks with that of placebo.

2. To provide comparative data on the effectiveness, safety and tolerability of tafenoquine versus mefloquine.

3. To determine the impact of chemoprophylaxis with mefloquine and tafenoquine on the subsequent susceptibility to malaria.

4. If an increase in the susceptibility to malaria was observed following prophylaxis, to identify the immune responses that were most affected by each regimen.

5. To characterise the population pharmacokinetics of tafenoquine and evaluate the effects of various subject characteristics on tafenoquine pharmacokinetics.

Secondary objectives 3 and 4 were not met since the immunological follow-up was abandoned due to the high early withdrawal rate during the study. The pharmacokinetics results will form part of a separate report.

3 Methodology

3.1 Study Design[1]

This was a 24 week placebo controlled, randomised, double-blind, double-dummy study of the efficacy, tolerability and safety of weekly tafenoquine compared with weekly mefloquine or placebo in the chemosuppression of *P. falciparum* in Nyanza Province, western Kenya. Subjects were then followed-up for a further 4 weeks after the last dose of study medication.

Subjects who met the study entry criteria and for whom written or oral informed consent was obtained were treated for three days with halofantrine 250 mg to clear any existing parasitaemia. At the end of the clearance period, subjects whose thick blood smears showed absence of malaria parasitaemia were randomised to one of the three study groups. Subjects received a loading dose of study medication (tafenoquine 200 mg, mefloquine 250 mg or placebo) for three days, followed by study medication (tafenoquine 200 mg, mefloquine 250 mg or placebo, respectively) once a week for 24 weeks.

Subjects were evaluated for malaria parasitaemia by weekly blood smears, and safety was assessed by recording adverse experiences and periodic blood tests. After the 24 week treatment period subjects continued to be monitored for the following 4 weeks and attended a safety follow-up visit during Week 28. All subjects attended a safety follow-up visit four weeks after their last dose, whenever they withdrew from the study.

The planned immunological follow-up part of the study, i.e. up until Week 70, was not carried out since the number of subjects prematurely withdrawn was too large to yield meaningful information. Adults permanently living in the study area have all had multiple malaria infections and should be considered at least semi-immune. This relative immunity protects against clinical disease but does not appear to influence the rate of parasitaemia appearing after initial clearance of parasites (1995-97 chemosuppression studies done at USAMRU-Kenya). Subjects would have continued to be monitored on a weekly basis for a further 9 weeks until week 37 as a washout period of study drug. At week 37, subjects would have been given a further treatment course of halofantrine 250mg for 3 consecutive days, and followed up until week 70. It was proposed to study the efficacy of tafenoquine to prevent both patent infection measured by parasitaemia

---

[1] Appendix A contains the protocol and sample case report form.

and malaria disease measured by parasitaemia in conjunction with symptoms consistent with malaria, and to determine whether or not previous prophylactic medication had compromised immunity.

Figure 1 Study design

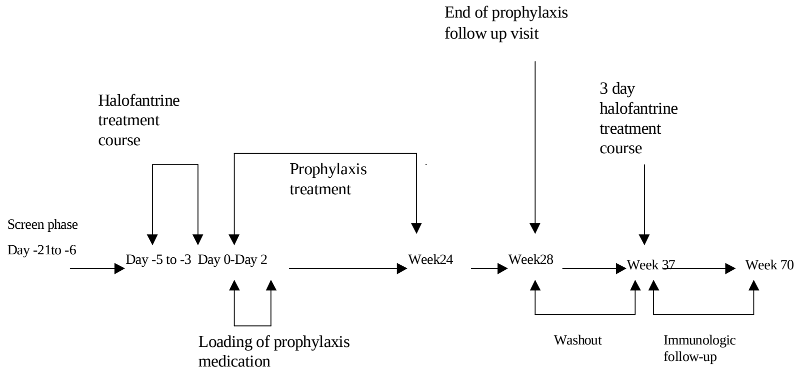

An Independent Data Monitoring Committee (IDMC) was convened to evaluate available efficacy and safety data after subjects had completed at least 8 weeks of study medication, or had been withdrawn from the study. If prophylactic outcome was unsatisfactory the IDMC were to consider stopping the study.

3.1.1 Protocol Amendments[2]

The protocol was initially approved on 20 January 2000, amended on 3 March, 15 March and 12 July 2000 and modified on 3 August 2000. Summary details of the amendments and modification are given below.

2 Appendix A contains the protocol amendments and modification.

Amendment 1, 3 March 2000

- The primary efficacy variable was changed from confirmed parasitaemia (two consecutive positive smears) to a single positive smear. This was considered to be a more conservative endpoint in the semi-immune population being studied.

- The eligibility criterion excluding subjects who were taking other antimalarial drugs was extended to include those who had taken other antimalarial drugs in the two weeks before the start of the study.

- The procedure to be followed in the event of a subject vomiting within one hour of taking study drug was amended because it was not considered practical for the health worker to stay with the subject for an hour or more. The subject was now to be visited the following day to determine whether or not vomiting had occurred.

- Other minor changes of wording were made to the protocol, appendices and consent form.

Amendment 2, 15 March 2000

- The upper age limit of subjects was adjusted to 55 as it had been specified as 65 in the original protocol in error.

- The requirement for smears on Days 1 and 2 (loading dose days) was removed.

- Examples of alternative treatments which might be prescribed should the subject develop malaria were added.

- Other minor changes of wording were made to the protocol and consent form.

Amendment 3, 12 July 2000

An interim analysis of the data was introduced and an Independent Data Monitoring Committee (IDMC) was convened to consider stopping the study if prophylactic efficacy was unsatisfactory. This was a result of finding, from about Week 8, that an unexpectedly high number of subjects had been withdrawn from the study due to positive parasitaemia. Since there was a possibility that one or both of the active treatment arms were not conferring the protective efficacy expected there was concern over the appropriateness of continuing the study.

Modification, 3 August 2000

A number of minor errors in the protocol were corrected and the charter of the IDMC was added as an appendix.

3.2 Investigators

The study was carried out at the Kombewa Clinic, Kenya by Dr Jose Stoute, United States Army Medical Research Unit, Kenya (USAMRU-K). Appendix A contains the curriculum vitae of the principal investigator which provides details of his qualifications and experience.

3.3 Ethics

The study was conducted in accordance with Good Clinical Practice[3] and the Declaration of Helsinki as amended in Somerset West, Republic of South Africa 1996. Volunteers were recruited by non-coercive methods according to existing policies of the Kenya Medical Research Institute (KEMRI) and United States Army Medical Research and Materiel Command (USAMRMC) regulation 70-25 (Use of Volunteers as Subjects of Research). Trained field workers from the region spoke to potential volunteers to inform them of the study and its aims, and further meetings were held to inform village elders and interested individuals. The protocol and statement of informed consent were approved by KEMRI and the US Army Human Subjects Research Review Board (HSRRB) prior to study initiation. Written informed consent (or witnessed oral consent for illiterate volunteers)[4] was obtained from each subject prior to entry into the study. Case report forms (CRFs) were provided for each subject's data to be recorded.

It was recognised that requesting female subjects to not become pregnant for up to two weeks after the second course of halofantrine (i.e. 39 weeks after entering the study) had implications for their sexual partners. Female subjects were asked to invite their partners to be present whilst giving Informed Consent. They were not, however, prohibited from entering the study if they refused to involve their partners in the Informed Consent process.

---

[3] as stated in EU CPMP for European multi-national studies, ICH GCP and 21 CFR for studies filed to the US IND.

[4] Appendix A contains the protocol and the sample informed consent is an appendix to the protocol.

3.4 Eligibility Criteria

3.4.1 Inclusion Criteria

The following inclusion criteria were applied.

- Healthy male or female volunteers who provided informed consent (a healthy volunteer was defined as one who was free of ailments that might cause difficulty in evaluating drug efficacy or adverse experiences).
- Subjects aged 18-55 years.
- Subjects planning to reside in the study area for the entire study duration of approximately 70 weeks.

3.4.2 Exclusion Criteria

The following exclusion criteria were applied.

- Subjects with positive parasitaemia following halofantrine treatment for radical cure.
- Subjects with any medical condition which, in the opinion of the investigator, made the subject unsuitable to enter the study.
- Subjects with personal or family history of seizures.
- Female subjects with a positive serum beta-HCG[5] (tested during screening and within 48 hours of first drug administration and approximately monthly thereafter).
- Women who were pregnant or lactating or who in the opinion of the investigator were at risk of becoming pregnant.
- Subjects with clinically significant abnormalities (to include but not limited to abnormal hepatic or renal function) as determined by history, physical and routine blood chemistries and haematology values.

---

[5] All female volunteers had β-HCG tested. Those requiring repeat testing due to a lapse of more than 48 hours were to have their original negative test confirmed by an additional urine test within 48 hours prior to initiation of study medication.

- Subjects who had demonstrated hypersensitivity to any of the study drugs especially to any other 8-aminoquinolines.
- Subjects unwilling to report for drug administration or blood drawing during the 70 week duration of the study.
- Subjects with G6PD deficiency.
- Subjects with laboratory guideline values for exclusion: haemoglobin <10 gm/dL, platelets <80,000/mm^3, WBC <3000ul^3, creatinine or ALT more than twice the upper limit of normal for age.
- Subjects with an abnormal ECG, particularly an extended QTc interval > 0.42 seconds.
- Subjects taking any other anti-malarial product, or who had taken an antimalarial drug other than halofantrine within the previous two weeks.
- Subjects who had received an investigational drug (a new chemical entity not registered for use) within 30 days or 5 half-lives whichever was the longer.
- Subjects with a history of psychiatric disorder.

3.5 Study Medication and Administration

3.5.1 Study Medication

Tafenoquine was supplied as gelatine capsules containing the succinate salt equivalent to 200 mg pure free base. Placebo tafenoquine capsules were of identical appearance. Mefloquine was supplied as over-encapsulated tablets containing mefloquine 250-mg free base, together with placebo mefloquine capsules of identical appearance.

Details of the appearance, formulation, dosage strength and batch numbers of drugs used are given in Table 1.

Table 1 Appearance, formulation and dosage strength of drugs used

Study Drug	Appearance	Formulation	Dose Unit	Batch Numbers
Tafenoquine	Swedish orange, size 1	Capsule	200 mg	N99354
Tafenoquine-placebo	Swedish orange, size 1	Capsule	-	N00061
Mefloquine	Swedish orange, size DB-AA	Over-encapsulated tablet	250 mg	N00067
Mefloquine-placebo	Swedish orange, size DB-AA	Over-encapsulated tablet	-	N99330

Data source: Appendix A contains the batch numbers and certificates of analysis.

Study medication was provided in opaque white high-density polyethylene bottles with child-resistant caps. Each bottle contained 30 dose units. The supply for each subject consisted of two bottles contained in a small carton labelled with at least the following information: subject identification, job number, protocol number, medication number, description of contents, dosage directions, storage instructions, warnings and sponsor address. Study medication was stored protected from light in secure conditions at room temperature (15 to 30°C).

3.5.2 Dosage and Administration

Subjects took a loading regimen of study medication once daily on Days 0 to 2, followed by a once weekly regimen during Weeks 1 to 24 using a double-dummy design. Two capsules were taken on each occasion; either one containing active drug and one containing placebo, or both containing placebo. Details of the capsules taken for each treatment group are shown below.

Treatment Group	Loading Regimen (Days 0-2)	Weekly Regimen (Weeks 1-24)
Tafenoquine	Tafenoquine 1 x 200 mg capsule<br>Mefloquine placebo 1 capsule	Tafenoquine 1 x 200 mg capsule<br>Mefloquine placebo 1 capsule
Mefloquine	Mefloquine 1 x 250 mg capsule<br>Tafenoquine placebo 1 capsule	Mefloquine 1 x 250 mg capsule<br>Tafenoquine placebo 1 capsule
Placebo	Tafenoquine placebo 1 capsule<br>Mefloquine placebo 1 capsule	Tafenoquine placebo 1 capsule<br>Mefloquine placebo 1 capsule

All doses of study drug were taken under the observation of health workers. A small meal was given to participants to eat before taking medication to minimise gastrointestinal effects. The health worker visited the subject on the following

day and if vomiting had occurred after an hour of dosing, the dose was to be re-administered. If the subject vomited after two sequential doses of study medication he was to be regarded as intolerant and withdrawn from the study.

Subjects who missed their once weekly dose of study medication were given the same dose on the following day. Subjects who missed two consecutive doses were to be withdrawn from the study. If a subject missed two non-consecutive doses the investigator was to contact the study monitor to discuss whether or not the subject could remain in the study.

3.5.3 Other Protocol Specified Therapy

All subjects were treated initially with halofantrine 250 mg for three days (Day –5 to –3) in order to clear any pre-existing malaria parasites/parasitaemia. This regimen, a modification of the usual treatment regimen licensed in the USA and Kenya (total 1500mg; divided into three doses of 500 mg given at intervals of 6 hours, without food), was chosen to minimise the peak plasma level of halofantrine (the best indicator of cardiac toxicity) and reduce the variability in absorption which could affect the clearance of halofantrine before the introduction of prophylaxis medication. Pharmacokinetic modelling suggested this regimen, taken with food (which increases bioavailability) would give adequate peak plasma levels for clearance.

If female subjects had not had a negative pregnancy test within the previous 48 hours, the test was to be repeated before the start of halofantrine treatment on Day –5. Absence of parasitaemia was confirmed by thick blood smear on Day –2. The halofantrine used was commercial stock supplied locally by the investigator.

3.6 Compliance with Study Medication

All study medication (including halofantrine) was taken under supervision of a health worker and both study subject and health worker were to sign that the medication had been taken. Whether or not the dose had been taken was recorded in the CRF.

3.7 Prior and Concomitant Medication

No other anti-malarial medication, other than halofantrine for parasite clearance, was permitted in the two weeks prior to starting study medication or during the 70 week study period.

The investigator was to approve any concomitant medication taken during the study and details were to be recorded in the CRF with daily dose, indication and dates of administration.

3.8 Study Procedures

3.8.1 Schedule of Assessments

The schedule of study procedures and assessments for the prophylaxis part of the study is shown in Table 2.

Table 2 Outline of study procedures and assessments

Procedure/Assessment	Screening	Parasitaemia Clearance Days –5 to -3	Day –2	Prophylaxis Loading Days 0 to 2	Prophylaxis Weekly Regimen Weeks 1 to 24	Safety Follow-up Weeks 25 to 28
Informed consent	x					
Demography	x					
Pregnancy test	x	x[1]	x[1]		x[2]	
Blood pressure/Pulse	x			x[3]	x[4]	x[5]
Temperature	x			x	x	x
G6PD[6]	x					
Medical history	x					
Physical examination	x					x[5]
Finger prick smear	x		x		x	x
Blood chemistry	x			x[3]	x[4]	x[5]
Eligibility	x			x[3]		
Randomisation				x[3]		
12-lead ECG	x					
Baseline signs/symptoms	x	x	x	x[3]		
Prior/Concomitant medication	x	x	x	x	x	x
Clearance medication		x				
Prophylaxis medication				x	x	
PBMCs (120 subjects only)				x[3]		x[5]
Plasma antibody				x[3]	x[4]	x[5]
Adverse experiences		x	x	x	x	x
Pharmacokinetic sample				x[7]	x[8]	x[9]

Data source: protocol/CRF in Appendix A. [1] During screening and within 48 hours of starting study medication; [2] Weeks 4, 8, 12, 16, 20 and 24 only; [3] Day 0 only; [4] Weeks 4, 8, 16 and 24 only; [5] Week 28 only; [6] Two tests from two different samples; [7] Day 2, 100 subjects only; [8] Weeks 4, 8, 16 only plus Week 17 for 100 subjects only; [9] Week 28 only for subjects developing parasitaemia.

3.8.2 Prestudy Screening and Enrollment

Subjects were screened for the study on Days –21 to –6, during which time the following assessments and procedures were undertaken.

- Informed consent was obtained.
- Demographic data were collected.
- Details of medical history (including history of malaria) and concomitant medication were obtained.
- A physical examination was performed.
- G6PD status was determined on two occasions during the screening period using standard laboratory tests (Sigma Diagnostics kit, Procedure # 203 – fluorescence of sample vs normal, intermediate and deficient controls). The same technique was used for both tests. Only subjects with a 'normal' reading on both occasions were eligible for the study.
- A blood sample was taken for haematology and clinical chemistry.
- Female subjects underwent a serum or urine beta-HCG pregnancy test within 48 hours of first receiving clearance medication with halofantrine (Stanbio Laboratory, USA, Qupid Plus Procedure # 1230 – qualitative immunoassay for detection of HCG in serum or urine). Only subjects with a negative test result were eligible for the study.
- A 12-lead ECG was performed.
- Vital signs (weight, blood pressure, pulse rate and temperature) were measured.

Subjects fulfilling all the entry criteria and laboratory requirements started three days of treatment with halofantrine.

3.8.3 Baseline Phase

Subjects received treatment with halofantrine 250 mg once daily on Days –5 to –3 to clear any pre-existing malaria parasites/parasitaemia. Baseline signs and symptoms and any changes to concomitant medication were recorded. On Day -2, the health worker obtained a sample for thick blood smear by finger prick to determine whether the subject had positive parasitaemia.

3.8.4 Treatment Phase(s)

Randomisation: Day 0

Eligibility for study treatment was determined on Day 0 with the following subjects being excluded from the study.

- Subjects with positive parasitaemia.
- Subjects with G6PD deficiency.
- Subjects with the following laboratory values: haemoglobin <10 gm/dL, platelets <80,000/mm^3, WBC <3000ul^3, creatinine or ALT more than twice the upper limit of normal for age.
- Subjects with a positive serum or urine beta-HCG.

Eligible subjects were randomised to one of the three treatment groups (tafenoquine, mefloquine or placebo) and the following assessments and procedures were undertaken.

- Details of any baseline signs and symptoms were recorded.
- Details of any concomitant therapy were recorded.
- A blood sample was taken for laboratory assessment (WBC, haematocrit, haemoglobin, platelets, creatinine, ALT and bilirubin) and a pregnancy test.
- A blood sample was taken for measurement of peripheral blood mononuclear cells (PBMCs) from 120 volunteers only.
- A blood sample was taken for malaria antibody measurement.
- Vital signs (blood pressure, pulse rate and temperature) were measured.

Loading of Prophylaxis Medication: Days 0 to 2

Subjects received three loading doses of double-blind study medication on Days 0 to 2 under the observation of a health worker. On Days 1 and 2, any adverse experiences or changes in concomitant medication were recorded. Additional blood samples were taken from 100 volunteers 6-12 hours after the third loading dose for analysis of plasma drug levels.

Weekly Regimen of Prophylaxis Medication: Weeks 1 to 24

Subjects then received once weekly doses of double-blind study medication, starting on Day 7, during visits from health workers. Blood smears were taken for parasitology, subjects were assessed for symptoms of malaria and their temperature was measured. Any adverse experiences or changes in concomitant medication were recorded.

A pregnancy test for female subjects was performed at Weeks 4, 8, 12, 16, 20 and 24. Blood pressure and pulse rate were measured at Weeks 4, 8, 16 and 24 and blood samples for routine laboratory assessment were taken at the same visits. Blood samples for malaria antibody measurement were taken at Weeks 4, 8 and 24.

At Weeks 4, 8 and 16, blood samples for pharmacokinetic analysis were taken at the same time as those for routine laboratory testing. At Week 17, the same 100 subjects who provided a sample for analysis of plasma drug levels on Day 2, provided an additional blood sample before their weekly dose of study medication.

If a blood sample was to be taken at any visit, the subject was required to attend the clinic.

If the field worker felt that the subject appeared ill or was thought to have malaria, additional blood smears for parasitology were taken and read immediately in the clinic. In addition, subjects were able to visit the clinic at any time if they felt unwell for an unscheduled visit.

During study treatment, subjects who tested positive for *Plasmodium* species were considered prophylactic failures and were withdrawn from treatment. They were entered into the safety follow-up phase of the study and given treatment according to local clinical practice, such as quinine or doxycycline.

3.8.5 Post-treatment Phase

Safety Follow-up: Week 28

All subjects were to attend the clinic for a safety follow-up visit, 4 weeks after their last dose of study medication, even if they withdraw from the prophylaxis

part of the study before the end of the 24 week treatment period. The following assessments and procedures were undertaken.

- A physical examination was performed.
- Details of any adverse experiences were recorded.
- Details of any concomitant therapy were recorded.
- A blood sample was taken for laboratory assessment (WBC, haematocrit, haemoglobin, platelets, creatinine, ALT and bilirubin).
- A blood sample was taken for measurement of peripheral blood mononuclear cells (PBMCs) from the same 120 volunteers who provided a sample on Day 0.
- A blood sample was taken for malaria antibody measurement.
- A blood sample was taken pharmacokinetic analysis.
- Vital signs (blood pressure, pulse rate and temperature) were measured.

Subjects continued to be monitored weekly for parasitaemia during the post-treatment safety follow-up. Subjects developing parasitaemia were given appropriate treatment according to the judgement of the investigator.

3.8.6 Reasons for Concluding Study

Subjects could withdraw or be withdrawn from the study at any time. The reason for withdrawal was to be documented on the CRF as follows.

- Adverse experience
- Insufficient therapeutic effect (development of parasitaemia)
- Protocol deviation (including non-compliance)
- Lost to follow-up
- Failed to meet eligibility criteria
- Other (reason to be specified)

3.9 Efficacy Assessments

3.9.1 Evaluation of Malaria

Thick and thin smears for evaluation of malaria were obtained by finger prick at screening, after clearance medication on Day –2 and then once a week during prophylaxis study treatment (scheduled slides). Additional smears were obtained at any time that a subject was ill or thought to have malaria (unscheduled slides). Every attempt was to be made for the smears to be taken 7 days apart, but any smear obtained during the week was to be counted as compliant with the parasitaemia monitoring requirement.

The slide reading procedure followed was that documented in the Standard Operating Procedure (SOP) of the United States Army Medical Research Unit, Kenya. Duplicate thick and thin smears were prepared and stained with Giemsa and each slide was read by a separate microscopist; each microscopist was blinded to the other's diagnosis. Asexual stage parasites in 50 high power fields were counted and reported as the number of parasites per 500 white blood cells; a minimum of 200 high powered fields were to be examined before a slide could be declared negative. Negative/positive discrepancies between the two microscopists were resolved by a third microscopist, usually the senior technician, who was to re-read the negative slide. The result of this re-read was considered final (ie. if negative slide found negative by the third microscopist the overall result was negative; if found positive then the overall result was positive).

Subjects with blood smears identified as positive for *Plasmodium* species through this procedure were termed as having a ‘first positive’ parasitaemia. Subjects who had two consecutive positive blood smears during follow-up were referred to in the protocol as having a ‘confirmed’ parasitaemia, but will be referred to elsewhere in this report as 'two consecutive positive' smears.

In addition to evaluation through blood smears, subjects were assessed for symptoms of malaria. For the purposes of data analysis only, subjects were classified as having ‘symptomatic’ and ‘asymptomatic’ parasitaemia. Symptomatic malaria was defined as either first positive or confirmed parasitaemia (see above) plus the presence of clinical symptoms consistent with malaria, such as fever, chills, headache, myalgia, nausea/vomiting or diarrhoea not otherwise explained. Asymptomatic malaria was defined as first positive or confirmed parasitaemia with no associated symptoms.

3.9.2 Primary Efficacy Parameter

The primary efficacy variable was the prophylactic outcome (success/failure) at the end of the prophylactic treatment phase (Week 24) (see Section 3.13.3). Success was defined by the subject having no positive smears. Failure was defined by a single positive smear during prophylactic study drug administration up to and including 7 days after the last dose.

3.9.3 Secondary Efficacy Parameters

The secondary efficacy variables were as follows.

- Number of subjects with two consecutive positive smears at Week 24.
- Number of subjects with a single positive smear (*P. falciparum* only) at Week 24.
- Number of subjects with a single positive smear during the prophylactic treatment phase plus follow-up (Week 28).
- Number of subjects with two consecutive positive smears at Week 28.
- Time to a single positive smear.
- Time to two consecutive positive smears.

In this study a single positive smear was used to determine the primary efficacy endpoint since this was thought to be the most sensitive means to detect failure and is a more conservative approach. Other researchers have required a repeat positive smear to confirm the initial result to reduce the possibility of the first slide being a false positive reading. This approach can be open to criticism in a semi-immune population due to their ability to quickly clear an infection due to naturally acquired immunity. It was decided to include two consecutive positive smears as a secondary efficacy parameter.

3.9.4 Other Efficacy Parameters

In addition to the primary and secondary efficacy variables, the number of subjects with symptomatic and asymptomatic malaria and the number of subjects with parasites of each species were summarised.

3.9.5 Investigations into High Failure Rate

Early in the study it became apparent that the failure rate was higher than would be expected from the previously observed protective efficacy of both tafenoquine and mefloquine. After 10 weeks over 200 subjects had been withdrawn due to positive parasitaemia. This led to the initiation of the following series of investigations into the cause.

3.9.5.1 Drug packaging and supplies

All unused supplies (twenty four patient packs) were shipped back from the site to SmithKline Beecham, Harlow, to be analysed for content, degradation and dissolution rate in the Sponsor's Drug Analysis Department. To ensure that optimum data was obtained, a sample from each un-opened (i.e. tamper-evident seal intact) bottle within each patient pack was tested.

The in house packaging documentation, packaging and labelling system and randomisation codes were audited for possible errors in packaging of the study supplies.

3.9.5.2 Slide Reading

To try to determine whether or not the apparent high failure rate could be explained by false positive slide reading, an independent second read of a selection of the study blood smears was performed. The 'first positive' slides for each subject (ie. duplicate pairs), plus a randomly generated selection of negative slides (also duplicate pairs) were re-read by experienced malaria microscopists at the Naval Medical Research Unit-2 (NAMRU-2) in Jakarta. The slide set consisted of 364 selected slide pairs (113 read as positive by USAMRU-K and 251 negative). NAMRU-2 has a rigorous microscopists training programme and each individual has to pass a slide reading test of a set of 25 test slides (with differing species, parasite densities and positive/negative status) with 100% specificity (ie. not reading false positives) and 90% sensitivity (false negatives). NAMRU-2 has 7 microscopists designated as 'expert' whose mean test scores are 100% specificity and 97% sensitivity and it was these expert readers who were assigned to the re-read task. The NAMRU-2 microscopy training Standard Operating Procedure (SOP) can be found in Appendix A.

NAMRU-2 microscopists followed the USAMRU-K slide reading SOP that was being used for the study. This required 200 high power fields to be read before a slide was declared negative. Each slide was read independently by two readers.

Any discrepancies were adjudicated by a third, senior microscopist who's decision was the final result after reading the slide initially declared negative. The re-reading was blinded, i.e. the microscopist did not know whether the slide had been originally categorised by USAMRU-K as positive or negative for *Plasmodia*. A protocol for this re-read process is supplied in Appendix A.

Following the re-read by NAMRU-2, a further subset of the slide set was sent to the Australian Malaria Institute (AMI) to be read by a world-leading malaria parasite morphologist, Professor Rieckmann, and his senior technician. This second re-read was suggested following the finding of far fewer positive slides by NAMRU-2 compared to the USAMRU-K readers and was an attempt to further validate one of the two groups' readings. This subset consisted of the 113 slide pairs which had been classified by USAMRU-K as positive for malaria parasites. AMI were not informed which of these slide pairs had been determined as positive for *Plasmodia* by NAMRU-2.

As a separate exercise at the end of the study, all of the first-positive slides remaining at site which could be readily located (a total of 250), plus a randomly selected number of negatives (a total of 512), were also shipped to NAMRU-2 to be re-read. This was in order to better estimate how many of all the subjects assigned as prophylactic failures by USAMRU-K were 'true' positive parasitaemias according to the re-read site. This would, in turn, permit a better evaluation of the protection afforded by tafenoquine and mefloquine in the study, although it would not be appropriate to derive protective efficacy values from this data.

3.9.5.3 Pharmacokinetic samples

All of the pharmacokinetic samples that had been collected at the time (approximately 12 weeks into the study) were sent to the Drug Metabolism and Pharmacokinetics Department SmithKline Beecham, Harlow, UK. Samples from those subjects randomised to receive tafenoquine (83 samples) were assayed for plasma drug levels to assess subject compliance with study medication and systemic availability of the drug. These consisted of 28 samples samples taken post third loading dose and 55 samples prior to dosing at Week 4. Analyses were performed of results per field worker to look for trends and also by prophylactic success/failure as determined by the USAMRU-K microscopy result.

3.9.5.4 Parasite Resistance Testing

Parasites cultured from two subjects withdrawn due to USAMRU-K-diagnosed parasitaemia were sent to WRAIR for *in vitro* testing of resistance to tafenoquine and mefloquine. It had not been possible to culture parasites from any other study samples so parasite isolates from other non-study individuals with malaria were also shipped to increase the numbers available for testing of local isolate drug sensitivities.

3.10 Safety Assessments

3.10.1 Adverse Experiences

An adverse experience was defined as any noxious, pathological or unintended change in anatomical, physiological or metabolic functions as indicated by physical signs, symptoms and/or laboratory changes occurring in any phase of the clinical study, whether associated with the study medication and whether or not considered drug related. This included an exacerbation of pre-existing conditions or events, intercurrent illnesses, drug interaction, or the significant worsening of the disease under investigation that was not recorded elsewhere in the CRF under specific efficacy assessments.

Anticipated day-to-day fluctuations of pre-existing conditions, including the disease under study, that did not represent a clinically significant exacerbation or worsening were not to be considered adverse experiences. Discrete episodes of chronic conditions occurring during the study period were to be reported as adverse experiences in order to assess changes in frequency or severity.

Pre-existing conditions or signs/symptoms present before the start of the study were to be recorded on the Medical/Surgical History form of the CRF. Adverse experiences occurring after informed consent was obtained but before starting randomised treatment were to be documented on the Baseline Signs and Symptoms form of the CRF, although serious baseline events were to be documented on the Serious Adverse Experience form of the CRF.

Adverse experiences (AEs) were elicited by asking the subject a non-leading question such as ***"Do you feel differently in any way since starting the new treatment?"*** Since subjects normally attended the clinic only at visits when blood samples were scheduled to be taken AEs were initially elicited by field workers on study specific forms during their weekly visits. If the subject responded "yes"

they were referred to the clinic for examination by the investigator or a Clinical Officer who was responsible for recording the AE diagnosis into the CRF, and prescribing treatment if deemed necessary. Details of the treatment emergent adverse experience and its severity including any change in study drug administration, investigator attribution to study drug, any corrective therapy given and outcome status were documented on the CRF. Attribution or relationship to study drug was judged by the investigator to be not related, unlikely, suspected (reasonable possibility) or probable. All adverse experiences were coded from the verbatim term according to the WHO Adverse Reaction Terminology (ART) dictionary by body system and preferred term.

Subjects were able to attend the clinic at any time in between scheduled visits for medical attention. Subjects with adverse experiences were to be followed-up until the event subsided or until the condition stabilised.

A serious adverse experience was any event which was fatal, life-threatening, permanently or temporarily disabling or incapacitating or resulted in hospitalisation, prolonged a hospital stay or was associated with cancer or overdose (either accidental or intentional). In addition, any experience which the investigator regarded as serious or which suggested any significant hazard, contra-indication, side effect or precaution that may have been associated with the use of the drug was reported as serious.

Any serious adverse experience which occurred during the study or within 12 weeks of the last dose of study medication was to be reported by the investigator to the local study monitor, the KEMRI Human Use Review Committee, USAMRMC Deputy Chief of Staff for Regulatory Compliance and Quality, and Commander USAMRU by telephone or facsimile transmission within 24 hours. This was to be followed by a full written report within three working days. In addition, any death, cancer or congenital abnormality brought to the attention of the investigator at any time after the end of study medication and considered by him to be possibly related to study medication was to be reported to the site monitor and medical monitor.

3.10.2 Pregnancy

All female subjects underwent a serum or urine beta-HCG pregnancy test at screening and the result was to be negative for entry into the study. If this test had not been carried out within 48 hours before the first administration of halofantrine clearance treatment, the test was to be repeated and a negative result obtained for eligibility. Female subjects then underwent repeat pregnancy tests on Day 0 and

at Weeks 4, 8, 12, 16, 20 and 24. At visits where blood samples were taken for routine laboratory assessment a serum test was performed, on other occasions a urine test was used.

Subjects who became pregnant during the study period were to discontinue medication immediately. Subjects were instructed to notify the investigator if, after the completion of the study, pregnancy during treatment or within 12 weeks of the end of treatment was confirmed. Whenever possible, a pregnancy was to be followed to term, any premature terminations reported and the status of mother and child after delivery reported to the sponsor. In addition, any premature terminations, including elective abortions, were to be reported.

3.10.3 Physical Examination, Vital Signs and ECG

A physical examination was performed at screening and Week 28, and a 12-lead ECG was performed at screening.

Vital signs (sitting blood pressure, pulse rate and oral temperature) were measured at screening, Day 0 and Weeks 4, 8, 16, 24 and 28, and temperature alone was measured at all other visits during prophylactic treatment and safety follow-up.

3.10.4 Laboratory Tests

Routine Laboratory Tests

Blood samples (2.5 mL) for routine haematology and clinical chemistry evaluation were taken at screening, Day 0 and Weeks 4, 8, 16, 24 and 28. The following variables were measured: WBC, haematocrit, haemoglobin, platelets, creatinine, ALT and bilirubin. At the screening visit, G6PD was also measured and subjects who were G6PD deficient were not eligible for treatment. Any abnormalities considered clinically significant were to be recorded in the adverse experience pages of the CRF.

The timing of the blood samples taken at Weeks 4, 8 and 16 could be co-ordinated with the time of the sample for pharmacokinetic analysis taken on these visits (see Section 3.11.1).

Plasma Malaria Antibodies

Blood samples (5 mL) were taken into EDTA tubes for measurement of malarial antibody levels at Day 0 and at Weeks 4, 8, 16, 24 and 28.

Peripheral Blood Mononuclear Cells

A blood sample (20 mL) was taken from 120 volunteers (40 in each treatment group) at screening and at Week 28 for measurement of PBMCs. These analyses do not form part of this report.

Parasite Drug Sensitivity

Subjects with confirmed patent parasitaemia provided blood samples (I mL) for storage of parasites, malaria parasite culture and in vitro drug sensitivity to be performed using standard techniques. It was possible to culture parasites from only two of these samples. These analyses do not form part of this report.

3.11 Pharmacokinetic Assessments

3.11.1 Sampling Times

Subjects provided up to 7 blood samples for pharmacokinetic assessment, three of these being routinely collected from all subjects. The three samples collected from all subjects were taken pre-dose at Week 4, between 2 and 5 hours post-dose at Week 8 and between 48 and 120 hours (2-5 days) following the Week 16 dose. In addition, samples were taken on Day 2 between 6 and 12 hours after the third loading dose and pre-dose at Week 17 from a subset of 100 volunteers. These two samples required additional clinic visits and so were not collected from all subjects. Finally subjects who developed parasitaemia were asked to provide samples at the time of development of parasitaemia and at the 4 week follow-up after failure.

For those sample collected within a specific time window, attempts were to be made to avoid all samples being collected at one end of the window, e.g. for the Day 2, 6-12 hour window not all samples were to be collected at 6 hours post-dose.

The date and exact time that the sample was taken was recorded on the CRF, as was the date and exact time of the dose of study medication prior to sample collection.

3.11.2 Specimen Preparation

Blood samples (2.5 mL) were drawn into EDTA tubes and chilled on crushed water ice. Plasma was separated by centrifugation and transferred to

appropriately labelled polypropylene tubes. Plasma samples were stored frozen at approximately –20°C or colder until being shipped to the Department of Drug Analysis, SmithKline Beecham, The Frythe, UK or its designee. Samples were shipped intermittently during the study period.

3.11.3 Pharmacokinetic Analysis

The pharmacokinetic analysis was the responsibility of the Department of Pharmacokinetics, DMPK, SmithKline Beecham. All pharmacokinetic data were stored in the Archives, SmithKline Beecham, Research and Development. The pharmacokinetic analysis will be reported separately.

3.12 Data Quality Assurance

This study was carried out at a single centre in Kenya. The protocol, CRF and safety reporting were reviewed with the investigator and his personnel responsible for the conduct of the study by the Company representative at the investigator site.

Adherence to the protocol requirements and verification of data generation accuracy was achieved through monitoring visits to each investigator site. Subsequent data handling and reporting processes were subject to in-process Quality Control and this final clinical report has, in addition, been subject to an end-stage Quality Control review. All the above procedures were performed according to methodologies detailed in SmithKline Beecham Standard Operating Procedures (SOPs).

Data on serious adverse experiences come from two different databases and have been collected at different time points. Although key data in the databases have been reconciled, minor discrepancies in details of the serious adverse experiences between individual subject narratives and the data source tables and listings may remain. It is considered, however, that these differences, if any, will be of a minor nature and will not change the overall significance or understanding of the serious adverse experiences.

No study specific audits were performed for this study at the time of writing.

3.13 Statistical Evaluation

3.13.1 Target Sample Size

The protective efficacy (PE) of tafenoquine was to be considered to be adequate if the lower limit of the 95% confidence interval was no lower than 70%. Based on the method of Farrington and Manning [18], 300 randomised subjects would be sufficient to give at least 90% power to reject protective efficacies of less than 70% using a two sided 95% confidence interval, given a placebo attack rate of 90% and a tafenoquine attack rate of 13.5% (estimated PE = 85%). This assumed a randomisation ratio of 1:1:1 for tafenoquine: placebo: mefloquine.

With 100 randomised subjects per treatment group receiving tafenoquine, placebo and mefloquine, the following incidence rates for adverse experiences could be estimated using a 95% confidence interval (CI) with accuracy as shown below.

Adverse Experience Incidence	Accuracy of 95% CI
5%	± 4.3%
10%	± 5.9%

3.13.2 Method of Randomization

All subjects meeting the study criteria were randomly assigned, in equal proportions and in a double-blind fashion, to receive one of the three treatment regimens according to a pre-determined code provided by SmithKline Beecham. The block size was 12.

3.13.3 Planned Efficacy Evaluations

3.13.3.1 Primary Efficacy Evaluation

For analysis purposes, a first positive smear was defined as follows:

First Positive Smear: presence of malaria parasites in a single blood sample, as recorded on the CRF.

The primary efficacy variable was prophylactic outcome (success/failure) at the end of the prophylactic treatment phase (Week 24). The definitions of prophylactic success and failure were as follows.

Prophylactic Success: Subject had no positive smear during prophylactic study drug administration up until the last available blood smear and including 7 days after the last dose of prophylactic study medication.

Prophylactic Failure: Subject had a first positive smear during prophylactic study drug administration up until last available blood smear and including 7 days after last dose of prophylactic study medication.

The prophylactic outcome for each subject was evaluated using all available blood smears up to and including 7 days after the last dose of prophylactic study medication. Thus, a subject who took a dose at Week 24 and subsequently tested positive at Week 25 was counted as a prophylactic failure since the dose given at Week 24 would be expected to offer protection up to Week 25.

In the tabulations of results, 'prophylactic success' is further categorised as known success or assumed success, defined as follows.

Prophylactic Success (Known): Subject met the definition of prophylactic success and also remained in the study for 24 weeks.

Prophylactic Success (Assumed): Subject met the definition of prophylactic success but was withdrawn from the study prior to Week 24.

For the primary efficacy assessment, the presence of parasites of any *Plasmodium* species (*P. falciparum*, *P. vivax*, *P. ovale* or *P. malariae*) was considered as a failure of prophylaxis.

3.13.3.2 Secondary Efficacy Evaluations

For analysis purposes, two consecutive positive smears was defined as follows:

Two Consecutive Positive Smears: Two consecutive positives smears no more than 9 days apart.

The secondary efficacy variables were as follows.

- Number of subjects with two consecutive positive smears during prophylactic treatment (Week 0 to Week 24) including 7 days after the last dose of prophylactic medication.

- Number of subjects with a first positive smear (*P. falciparum* species only) during prophylactic treatment (Week 0 to Week 24) including 7 days after the last dose of prophylactic medication.
- Number of subjects with a first positive smear at any time during prophylactic treatment and the 4 week follow-up period (Week 0 to Week 28).
- Number of subjects with two consecutive positive smears at any time during prophylactic treatment and the 4 week follow-up period (Week 0 to Week 28).
- Time to first positive smear
- Time to two consecutive positive smears

The third and fourth variables in the list above were not analysed because of the poor results during the prophylaxis treatment period and, therefore, the small numbers of subjects remaining in the study from Week 24.

3.13.3.3 Other Efficacy Evaluations

The number of subjects with parasites by species (i.e. *P. falciparum, P. vivax, P. ovale, P. malariae*) were summarised together with the parasite count at the first positive smear.

In addition, malaria status was tabulated, categorised as the number of subjects with a symptomatic first positive smear, an asymptomatic first positive smear, symptomatic two consecutive positive smears and asymptomatic two consecutive positive smears (see Section 3.13.4.2).

3.13.4 Methods of Analysis

3.13.4.1 Primary Efficacy Analysis

Principal Analysis

The primary efficacy analysis was based on a calculation of the protective efficacy (PE) of tafenoquine. Protective efficacy (PE) was defined as follows.

$$PE_I(\%) = \frac{I_{placebo} - I_{drug}}{I_{placebo}} * 100$$

where

$$I_{placebo} = \frac{\text{number of prophylactic failures in the placebo group}}{\text{number of subjects at risk in the placebo group}}$$

$$I_{drug} = \frac{\text{number of prophylactic failures in the drug group}}{\text{number of subjects at risk in the drug group}}$$

Corresponding 95.2%[6] confidence limits for the PE were calculated based on the method of Koopman [19]. A conclusion of adequate efficacy of tafenoquine was to be drawn if the lower limit of this 95.2% confidence interval was no lower than 70%. The PE was calculated based on the intent-to-treat (ITT) and per-protocol populations.

The primary comparison of interest was the PE of tafenoquine compared with placebo. In addition, the PE of mefloquine was also calculated and is presented, but no formal statistical tests of comparison were used as the study was not powered to detect differences between tafenoquine and mefloquine. It was anticipated that the PEs of tafenoquine and mefloquine would be within 5% of each other.

The primary analysis was based on all species of malaria parasite.

Additional Analyses

1. *Protective Efficacy Based on Incidence Density*: To assess the robustness of the calculation to drop-outs in the ITT analysis, a calculation of PE based on incidence density was produced. In this case, PE was calculated as follows.

$$PE_D(\%) = \frac{D_{placebo} - D_{drug}}{D_{placebo}} * 100$$

where

$$D_{placebo} = \frac{\text{number of prophylactic failures in the placebo group}}{\text{number of subject-years exposure in the placebo group}}$$

$$D_{drug} = \frac{\text{number of prophylactic failures in the drug group}}{\text{number of subject-years exposure in the drug group}}$$

---

6 Adjusted due to the interim analysis by the IDMC. Details given in section 3.13.4.4).

Incidence density and PE, together with 95.2% confidence intervals were tabulated.

2. *Worst Case Analysis*: A worst case analysis whereby all subjects who withdrew during the prophylactic study medication phase were regarded as prophylactic failures was performed. Worst case prophylactic outcome was tabulated and the worst case PE was calculated together with a 95.2% confidence interval.

3. *Covariate Analysis*: Details of the covariate analysis are provided in the Statistical Appendix in Appendix H of the report.

3.13.4.2 Secondary Efficacy Analysis

Protective Efficacy Analyses

The number of subjects with two consecutive positive smears and with a first positive smear, (*P. falciparum* only) during prophylactic treatment (Week 0 to Week 24) including 7 days after last dose of medication were tabulated. The PEs of tafenoquine and mefloquine based on these endpoints were calculated, together with 95% confidence intervals. These tabulations were produced for the ITT and per-protocol populations.

The final model from the covariate analysis was fitted to the endpoint of two consecutive positive smears and odd ratios and 95% CIs were calculated for the ITT population only. Details are given in the statistical appendix (appendix H).

Time to Event Analyses

For analysis purposes, time to parasitaemia was calculated as follows:

Time to Parasitaemia: For a prophylactic failure, the time to first positive smear was calculated as (date of first positive smear – date of first loading dose of prophylactic medication + 1). For a prophylactic success, the subject was censored at the time of the last negative smear within 7 days of the last dose of study medication, even if a subsequent negative smear existed. The censored survival time was calculated as (date of last negative smear (within 7 days of last dose) – date of first loading dose +1). For the time to two consecutive positive

smears, this definition was applied using the date of the first of the two consecutive positive smears.

A Kaplan-Meier curve was produced showing the survival curves for time to parasitaemia in each treatment group. This was carried out for both first positive smear and two consecutive positive smears during the prophylaxis phase, for both the ITT and per-protocol populations. If a subject was free of parasitaemia then the time to parasitaemia was censored at the date of the last negative smear, within 7 days of the last dose of study medication. It had been anticipated that there would be too few incidences of parasitaemia in the active treatment arms to enable any further survival analysis techniques to be used. However, due to slide reading issues (see Section 5.5) sufficient cases of apparent parasitaemia did occur to allow the survival curves to be compared using the PROC LIFETEST (log-rank test) procedure in SAS®. The null hypothesis in each case was that there was no difference between the survival curves, versus the alternative that there was a difference.

3.13.4.3 Other Efficacy Analysis

Parasite Count By Species

For tables of summary statistics of parasite count by species, parasite count was calculated as follows:

Parasite Count: The parasite count was recorded per 500 WBCs and then converted for reporting purposes to a count per microlitre of blood using the following formula.

$$\text{Parasite count per microlitre} = \frac{\left(\frac{\text{parasite count per 500 WBC}}{500}\right) \times \text{WBC}^{*} \times 10^{9}/l}{10^{6}/l}$$

$$= \left(\frac{\text{parasite count per 500 WBC}}{500}\right) \times \text{WBC}^{*} \times 1000$$

$$= \text{parasite count per 500 WBC} \times \text{WBC}^{*} \times 2$$

* WBC is expressed as count x 10^9/L and the valued needed was to be:

1 Within 7 days of the positive smear,
2 or, if no value met this criterion, the subject's baseline value,

3 or, if there was no baseline value, the mean of all the other subject's baseline values.

Malaria Status

For tables of malaria status, subjects were classified as follows:

Symptomatic/Asymptomatic Parasitaemia: Each of the parasitaemias above (first positive smear and two consecutive positive smears) was classed as symptomatic in the presence of clinical symptoms of malaria, and otherwise as asymptomatic. The following categories were defined for presentation of these data. Each subject was allocated to their worst category, in the order of ascending severity of infection in the list below:

- *No parasitaemia*: a subject was included in this category if all blood smears whilst receiving prophylactic study medication, up to and including 7 days after the last dose, were free of malaria parasites (all species).

- *Asymptomatic first positive smear*: a subject was included in this category if he had a first positive smear (that did not subsequently become two consecutive positive smears) without symptoms of malaria, whilst receiving prophylactic study medication, up to and including 7 days after the last dose.

- *Symptomatic first positive smear*: a subject was included in this category if he had a first positive smear (that did not subsequently become two consecutive positive smears) in the presence of symptoms of malaria whilst receiving prophylactic study medication, up to and including 7 days after the last dose.

- *Asymptomatic two consecutive positive smears*: a subject was included in this category if he had two consecutive positive smears without symptoms of malaria (at both visits) whilst receiving prophylactic study medication, up to and including 7 days after the last dose.

- *Symptomatic two consecutive positive smears*: a subject was included in this category if he had two consecutive positive smears in the presence of symptoms of malaria (at either visit) whilst receiving prophylactic study medication, up to and including 7 days after the last dose.

Analysis of Re-read Slide Data

After approximately 12 weeks into the study all of the 'first positive' slides (first occurrence of a single positive parasitaemia) and a random selection of negatives were sent to the Naval Medical Research Unit 2, Jakarta (NAMRU-2) for re-reading. At the end of the study the remaining 'first positive' blood smear slides and a further random selection of negative slides were also sent to NAMRU-2 to be re-read. The results of these re-reads were sent to SB in an Excel spreadsheet and entered onto the study database. Tables were produced comparing the USAMRU-K and NAMRU-2 results, and sensitivity and specificity of the original USAMRU-K read to the NAMRU-2 re-read were calculated. Listings were produced showing the results of the NAMRU-2 re-reads and also comparing the NAMRU-2 read to the original USAMRU-K read.

Visit windows

For many analyses, time intervals in days were specified for each week of the study. These were defined as follow:

**Week**	**Days**	**Week**	**Days**	**Week**	**Days**
1	0-10	11	74-80	21	144-150
2	11-17	12	81-87	22	151-157
3	18-24	13	88-94	23	158-164
4	25-31	14	95-101	24	165-171
5	32-38	15	102-108	25	172-178
6	39-45	16	109-115	26	179-185
7	46-52	17	116-122	27	186-192
8	53-59	18	123-129	28	193-199
9	60-66	19	130-136	≥29	≥200
10	67-73	20	137-143		

3.13.4.4 Interim Analysis and IDMC Review

No interim analysis was performed. An Independent Data Monitoring Committee (IDMC), however, was convened to evaluate efficacy and safety following the observation, after subjects had completed approximately 8 weeks of study medication, of an apparent high prophylactic failure rate. As a result, in order to allow for the multiple comparisons, all confidence intervals presented for the primary efficacy analysis were at the 95.2% level [20] rather than the 95% level specified in the protocol. Proceedings to set up an IDMC were initiated in early July 2000 and the IDMC met and reviewed data on 21st August 2000.

The IDMC consisted of three external members, who were supported by a Statistical and Data Analysis Centre (SDAC) based at the London School of Hygiene and Tropical Medicine. Serious Adverse Event data (to assess potential safety concerns) and parasitaemia data (to assess efficacy) were provided to the SDAC who were also provided with the randomisation code. Analyses were performed and tabulated and sent to the IDMC in order for them to make their recommendations about study continuation. Neither the Sponsor nor the Investigator were unblinded to individual study subject data during this process.

The IDMC were to consider a number of efficacy and safety considerations before making a recommendation as to whether the study should be stopped or whether it should continue. One of the requests made to the IDMC was to calculate protective efficacies of the tafenoquine and mefloquine arms relative to placebo, to assess whether there was significant failure of the test medication, tafenoquine, to protect against infection. Full details of these considerations along with membership of the IDMC can be found in the IDMC charter Appendix A.

3.13.5 Populations/Data Sets to be Evaluated

Two populations were defined as follows for the analysis of clinical efficacy. Subjects who were not randomised to treatment, including those who received clearance medication, were denoted as ‘screening only’.

Intent-to-treat Population

The intent-to-treat treat population consisted of all randomised subjects who were free from parasitaemia following clearance medication, took at least one dose of prophylactic study medication and attended at least one follow-up visit at which assessment of a blood smear took place.

Per-protocol Population

All randomised subjects who satisfied those inclusion/exclusion with the potential to affect efficacy, and subsequently adhered to the protocol were included in the per-protocol population which was a subset of the ITT population. Subjects were excluded from the per-protocol population if any of the following criteria applied.

1. Subject was taking any other anti-malarial product, or had taken an anti-malarial drug other than halofantrine within the previous two weeks.

2. Subject had received an investigational drug (a new chemical entity not registered for use) within 30 days or 5 half-lives whichever was the longer.

3. Subject had a medical condition which had the potential to bias efficacy.

4. Subject was non-compliant with clearance medication (halofantrine).

5. Subject was non-compliant with randomised prophylactic study medication.

6. Subject was non-compliant with the parasitaemia monitoring requirement as detailed below:

Parasitaemia Monitoring Requirement: A subject was considered non-compliant with the parasitaemia monitoring requirement if he had a single smear gap of greater than 17 days or four or more non-consecutive smear gaps of greater than 11 days. Violation of this criterion only led to exclusion for subjects who were a prophylactic success.

7. Subject did not complete the prophylactic medication phase.

Subjects were only be excluded from the per-protocol population from the time at which the violation occurred. Subjects who were prophylactic failures were only excluded if the date of violation was prior to the date of the first positive smear; if the violation occurred after the first positive smear a subject was not excluded from the per-protocol population since the criteria for prophylactic failure had already been met.

All decisions on eligibility for inclusion in these populations were made prior to code-break or any data evaluation.

The ITT population was the population of primary interest in this study, while the per-protocol population was used to confirm the results.

3.13.6 Safety Evaluations

The safety population consisted of all subjects who had received at least one dose of prophylactic study medication. Subjects who received the incorrect coded prophylactic study medication were to be analysed according to the treatment actually received.

Adverse Experiences

Adverse experience reporting was performed using the World Health Organisation (WHO) coding system. Each adverse experience coded using the WHO system can be associated with up to three body systems (e.g. headache is

associated with ‘Central Nervous and Peripheral Nervous System’ and with ‘Body as a Whole General’). Any adverse experience was associated with the primary body system only.

Counting was based on the number of subjects not the number of adverse experiences; for example, if a subject reported the same adverse experience on three occasions within a time interval, that adverse experience was counted only once.

Adverse experiences were tabulated for the following time intervals.

- *Baseline*: all adverse experiences that started before receiving prophylactic study medication. Baseline adverse experiences were reported with baseline signs and symptoms with the exception of serious baseline adverse experiences.
- *Prophylaxis Treatment Phase*: all adverse experiences that started after the first dose of prophylactic study medication up to and including the last dose of prophylactic study medication.
- *Safety Follow-up*: all adverse experiences that started after the last dose of prophylactic study medication.

Evaluation of adverse experiences included determination of the proportion of subjects with adverse experiences, the proportion of subjects with CNS/psychiatric adverse experiences (selected using the WHO codes listed in Appendix A), the proportion of subjects with adverse experiences by maximum severity, the proportion of subjects with adverse experiences with a suspected or probable relationship to study treatment (including those of unknown relationship), the proportion of subjects with adverse experiences leading to withdrawal, the proportion of subjects with serious adverse experiences and the time of first occurrence of adverse experiences.

Vital Signs

Vital signs data were evaluated by summarising change from baseline (Day 0 or screening, i.e. Day –21 to Day –4, if missing on Day 0) at subsequent visits. If more than one value was recorded within a time window for a particular variable, then the earliest value was used in the change from baseline calculations. Vital signs were also evaluated by a flagging system for individual results to denote

whether values were above or below pre-determined values of clinical concern. The flagging criteria are shown in Table 3.

Table 3 Sponsor defined criteria for evaluating vital signs

Vital Sign	Flagging Criterion
Systolic blood pressure (mmHg)	<80 mmHg or >140 mmHg
Diastolic blood pressure (mmHg)	>100 mmHg
Heart rate (beats/min)	<50 bpm or >100 bpm
Temperature (degrees Celsius)	>37.5°C

The proportions of subjects with vital signs results of clinical concern were tabulated for the intervals: Baseline, Week 4, Week 8, Week 16, Week 24 and Week 28. A subject with the same variable above or below the pre-determined levels at different timepoints was counted twice. Where more than one visit was made in a time period, a subject was recorded as having a high or low value if a high or low value was recorded at any one of the visits.

Clinical Laboratory Tests

Laboratory data were evaluated by summarising change from baseline (Day 0 or screening, i.e. Day –14 to Day –4, if missing on Day 0) at subsequent visits. If more than one value was recorded within a time window for a particular variable, then the earliest value was used in the change from baseline calculations. Laboratory data were also evaluated by a flagging system for individual results, as described below.

- *Change from baseline*: this flag denoted a value that had increased or decreased from baseline by more than a specified amount (F2 flag).

- *Extended normal range*: this flag denoted a value that fell outside an extended normal range (F3 flag).

An F2F3 flag denoted a value that had changed by more than a predefined limit from baseline and fell outside an extended normal range.

The F2 range for a particular laboratory test was calculated in one of three ways.

- *Absolute*: pre-specified change; for example, haemoglobin had a lower limit of a reduction of 2.0 g/dL.

- *Proportional*: the upper and lower limits were defined by multiplying the baseline value by different factors; for example, haematocrit had a lower limit equal to 95% of baseline.

- *Normal range span*: the normal range span (NRS) was the difference between the upper and lower limits of the normal range. The upper limit of the F2 range was calculated by adding a factor of the NRS to the baseline value and the lower limit of the F2 range was calculated by subtracting the same factor of the NRS from the baseline value; for example, the upper limit for creatinine was baseline plus 50% of the NRS.

The F3 range for a particular laboratory measurement was calculated in one of two ways.

- *Absolute*: pre-specified limits; for example, platelets had a lower limit of 50000 x 10^9/L.

- *Normal range limit*: the upper limit was the normal range high (NRH) multiplied by a factor and the lower limit was the normal range low (NRL) multiplied by a factor; for example, the upper limit for creatinine was 150% of the NRH.

The F2 and F3 ranges are defined in Table 4.

Table 4 Sponsor defined criteria for evaluating laboratory variables

Laboratory Variable	Standard Units	F1 Higher Limit<br>F1 Lower Limit	F2 Higher Limit<br>F2 Lower Limit	F3 Higher Limit<br>F3 Lower Limit
**Haematology**				
Haemoglobin	g/L	180	None	None
		100	Reduction of 20.0	< 70.0
Haematocrit	%	51	None	None
		31	< 0.95 x baseline	< 25
White blood cells	$x10^9$/L	8	> 1.5 x baseline	> 14000
		2	< 0.75 x baseline	< 2500
Platelets	$x10^9$/L	400	None	None
		100	< 0.75 x baseline	< 50000
**Clinical Chemistry**				
ALT	U/L	42	> Baseline + 75% NRS	> 200% NRH
		0	None	None
Creatinine	umol/L	97.24	> Baseline + 50% NRS	> 150% NRH
		44.2	None	None
Bilirubin	umol/L	34.2	> Baseline + 50% NRS	> 150% NRH
		0	None	None

Note Reference ranges are provided in the laboratory listings in Appendix F

The proportions of subjects with laboratory results of clinical concern were tabulated for the intervals: Baseline, Week 4, Week 8, Week 16, Week 20, Week 24 and Week 28. A subject with the same variable above or below the pre-determined levels at different timepoints was counted twice. Where more than one visit was made in a time period, a subject was recorded as having a high or low value if a high or low value was recorded at any one of the visits.

4 Study Population

4.1 Study Dates

The first subject received the first dose of halofantrine on 3 May 2000. The first dose of prophylaxis study medication was taken on 8 May 2000 and the last dose of prophylaxis medication was taken on 25 October 2000 (Listing B.02 in Appendix B). The date of the last thick blood smear was 24 November 2000.

4.2 Subject Disposition

4.2.1 Number and Distribution of Subjects

In total, 517 subjects were screened for entry into the study. Two hundred and eleven of these subjects were not randomised to treatment due either to abnormal laboratory results (62), G6PD deficiency (45), extended QTc interval(3), failure to clear parasitaemia (2) or ‘other’ reasons (105). The numbers of subjects who failed for each reason are given in Table 11.2 in Section 11 (Listing B.04 in Appendix B). As a result, 306 subjects were randomised[7] to prophylactic study treatment. The numbers of subjects who were randomised and completed the study, and the numbers in each study population are shown in Table 5.

Table 5 Subject disposition (number of subjects)

**Population**	**Treatment Group**			**Total**
	**Placebo**	**Tafenoquine 200 mg**	**Mefloquine 250 mg**	
Screened	-	-	-	517
Randomised	101	104	101	306
Intent-to-treat population	99	102	99	300
Completed study	1	7	4	12
Per protocol population	92	94	90	276
Safety population	101	104	101	306

Data source: Table 11.1 in Section 11; Listings B.01, B.03, B.04, B.05, B.06 in Appendix B

Six subjects were excluded from the intent-to-treat population. The reasons for exclusion and the numbers in each treatment group are shown in Table 6.

---

[7] Appendix A contains the randomisation code.

Table 6 Subjects excluded from the intent-to-treat population: all randomised subjects

Reason for Exclusion	Treatment Group		
	Placebo<br><br>N=101	Tafenoquine 200 mg<br>N=104	Mefloquine 250 mg<br>N=101
No negative smear before first dose	1 (1.0%)	0	0
No smears post-first dose	1 (1.0%)	2 (1.9%)	2 (2.0%)
Total number excluded	2 (2.0%)	2 (1.9%)	2 (2.0%)

Data source: Table 11.5.1 in Section 11; Listing B.05 in Appendix B

The reasons for exclusion from the per-protocol population are discussed in Section 4.3.

4.2.2 Number of Subjects Present at Each Visit

The numbers of subjects in each treatment group assessed at each study visit on-dose are shown in Table 7. From as early as Week 3 the number of subjects attending each visit started a marked decline, reflecting the number of subjects being classified as prophylactic failures (Section 5.5). This affected all treatment groups although the tafenoquine group had the least steep decline and the placebo the most marked.

Table 7 Number (%) of subjects assessed at each visit on-dose: intent-to-treat population

Visit	Treatment Group		
	Placebo<br>N=99	Tafenoquine 200 mg<br>N=102	Mefloquine 250 mg<br>N=99
Screening	99 (100%)	102 (100%)	99 (100%)
Clearance 1 (Day –5)	99 (100%)	102 (100%)	99 (100%)
Clearance 2 (Day –4)	99 (100%)	102 (100%)	99 (100%)
Clearance 3 (Day –3)	99 (100%)	102 (100%)	99 (100%)
Loading 1 (Day 0)	99 (100%)	102 (100%)	99 (100%)
Loading 2 (Day 1)	99 (100%)	102 (100%)	99 (100%)
Loading 3 (Day 2)	99 (100%)	101 (99.0%)	99 (100%)
Week 1	99 (100%)	102 (100%)	99 (100%)
Week 2	98 (99.0%)	99 (97.1%)	98 (99.0%)
Week 3	88 (88.9%)	92 (90.2%)	90 (90.9%)
Week 4	82 (82.8%)	86 (84.3%)	88 (88.9%)
Week 5	78 (78.8%)	82 (80.4%)	79 (79.8%)
Week 6	58 (58.6%)	77 (75.5%)	72 (72.7%)
Week 7	43 (43.4%)	71 (69.6%)	58 (58.6%)
Week 8	27 (27.3%)	59 (57.8%)	42 (42.4%)
Week 9	18 (18.2%)	47 (46.1%)	40 (40.4%)
Week 10	11 (11.1%)	40 (39.2%)	33 (33.3%)
Week 11	9 (9.1%)	34 (33.3%)	23 (23.2%)
Week 12	6 (6.1%)	32 (31.4%)	18 (18.2%)
Week 13	5 (5.1%)	26 (25.5%)	13 (13.1%)
Week 14	4 (4.0%)	22 (21.6%)	12 (12.1%)
Week 15	3 (3.0%)	21 (20.6%)	10 (10.1%)
Week 16	3 (3.0%)	21 (20.6%)	11 (11.1%)
Week 17	3 (3.0%)	16 (15.7%)	11 (11.1%)
Week 18	3 (3.0%)	14 (13.7%)	11 (11.1%)
Week 19	3 (3.0%)	13 (12.7%)	10 (10.1%)
Week 20	2 (2.0%)	11 (10.8%)	9 (9.1%)
Week 21	1 (1.0%)	9 (8.8%)	9 (9.1%)
Week 22	1 (1.0%)	7 (6.9%)	8 (8.1%)
Week 23	1 (1.0%)	7 (6.9%)	8 (8.1%)
Week 24	1 (1.0%)	7 (6.9%)	2 (2.0%)

Data source: Table 11.3.1a in Section 11; Listing B.02 in Appendix B

Results for the per-protocol population are shown in Tables 11.3.1b and 11.3.2b in Section 11.

4.2.3 Withdrawal Reasons

In the intent-to-treat population, 12 subjects (4.0%) completed the study. A summary of the reasons for withdrawal is shown by treatment group in Table 8.

Table 8 Reason for study conclusion: intent-to-treat population (number (%) of subjects)

**Study Conclusion Reason**	**Treatment Group**		
	**Placebo N=99**	**Tafenoquine 200 mg N=102**	**Mefloquine 250 mg N=99**
**Completed Study***	1 (1.0%)	7 (6.9%)	4 (4.0%)
**Reason for Withdrawal**			
Adverse experience	0	1 (1.0%)	0
Insufficient therapeutic effect**	92 (92.9%)	88 (86.3%)	89† (89.9%)
Protocol deviation††	2 (2.0%)	1 (1.0%)	2 (2.0%)
Lost to follow-up	2 (2.0%)	3 (2.9%)	1 (1.0%)
Other	2 (2.0%)	2 (2.0%)	3 (3.0%)
**Total Withdrawn**	98 (99.0%)	95 (93.1%)	95 (96.0%)

Data source: Table 11.4.1 in Section 11; Listing B.03 in Appendix B
* Subjects were considered to have completed the study if no reason for withdrawal was recorded on the CRF.
** Development of parasitaemia.
† Includes one subject who did not develop malaria but refused further medication because of abdominal pain (see Table 44).
†† Including non-compliance.

Almost all premature withdrawals from the study were as a result of subjects developing parasitaemia (see Section 6). There was only one subject recorded as withdrawn due to adverse experiences (030.001.00173). This is inconsistent with Table 44 where there are ten subjects reported to have withdrawn due to adverse experiences since Table 9 is taken from the Study Conclusion page of the CRF. Thus although a further 9 subjects were withdrawn due to adverse experiences this was not the reason given on the Conclusion page

The cumulative proportions of subjects withdrawn over time is summarised in Table 9.

Table 9 Cumulative proportion of subjects withdrawn from the study: intent-to-treat population

Visit	Placebo N=99				Tafenoquine 200 mg N=102				Mefloquine 250 mg N=99			
	AE	ITE	Oth	Tot	AE	ITE	Oth	Tot	AE	ITE	Oth	Tot
Week 1	0	1.0	0	1.0	0	2.9	0	2.9	0	0.0	1.0	1.0
Week 2	0	11.1	0	11.1	0	8.8	1.0	9.8	0	8.1	1.0	9.1
Week 3	0	16.2	0	16.2	0	13.7	2.0	15.7	0	10.1	1.0	11.1
Week 4	0	19.2	2.0	21.2	0	15.7	2.9	18.6	0	18.2	1.0	19.2
Week 5	0	37.4	4.0	41.4	0	21.6	2.9	24.5	0	26.3	1.0	27.3
Week 6	0	50.5	5.1	55.6	0	25.5	2.9	28.4	0	38.4	3.0	41.4
Week 7	0	65.7	5.1	70.7	1.0	37.3	3.9	42.2	0	52.5	3.0	55.6
Week 8	0	76.8	5.1	81.8	1.0	49.0	3.9	53.9	0	55.6	4.0	59.6
Week 9	0	83.8	5.1	88.9	1.0	55.9	3.9	60.8	0	62.6	4.0	66.7
Week 10	0	84.8	6.1	90.9	1.0	60.8	4.9	66.7	0	71.7	5.1	76.8
Week 11	0	87.9	6.1	93.9	1.0	62.7	4.9	68.6	0	76.8	5.1	81.8
Week 12	0	88.9	6.1	94.9	1.0	67.6	4.9	73.5	0	80.8	5.1	85.9
Week 13	0	89.9	6.1	96.0	1.0	72.5	4.9	78.4	0	82.8	5.1	87.9
Week 14	0	90.9	6.1	97.0	1.0	72.5	4.9	78.4	0	83.8	5.1	88.9
Week 15	0	90.9	6.1	97.0	1.0	73.5	4.9	79.4	0	83.8	5.1	88.9
Week 16	0	90.9	6.1	97.0	1.0	76.5	4.9	82.4	0	83.8	6.1	88.9
Week 17	0	90.9	6.1	97.0	1.0	78.4	5.9	85.3	0	83.8	6.1	89.9
Week 18	0	90.9	6.1	97.0	1.0	80.4	5.9	87.3	0	83.8	6.1	90.9
Week 19	0	91.9	6.1	98.0	1.0	82.4	5.9	89.2	0	84.8	6.1	90.9
Week 20	0	92.9	6.1	99.0	1.0	84.3	5.9	91.2	0	84.8	6.1	91.9
Week 21	0	92.9	6.1	99.0	1.0	86.3	5.9	93.1	0	85.9	6.1	91.9
Week 22	0	92.9	6.1	99.0	1.0	86.3	5.9	93.1	0	85.9	6.1	96.0
Week 23	0	92.9	6.1	99.0	1.0	86.3	5.9	93.1	0	89.9	6.1	96.0
Week 24	0	92.9	6.1	99.0	1.0	86.3	5.9	93.1	0	89.9	6.1	96.0
Week 25	0	92.9	6.1	99.0	1.0	86.3	5.9	93.1	0	89.9	6.1	96.0
Week 26	0	92.9	6.1	99.0	1.0	86.3	5.9	93.1	0	89.9	6.1	96.0
Week 27	0	92.9	6.1	99.0	1.0	86.3	5.9	93.1	0	89.9	6.1	96.0
Week 28	0	92.9	6.1	99.0	1.0	86.3	5.9	93.1	0	89.9	6.1	96.0
Post-Week 28	0	92.9	6.1	99.0	1.0	86.3	5.9	93.1	0	89.9	6.1	96.0

Data source: Table 11.4.2 in Section 11; Listing B.03 in Appendix B

NB AE=adverse experience; ITE=insufficient therapeutic effect; Oth=other (includes protocol deviations and lost to follow-up); Tot=total.

Withdrawal due to apparent development of parasitaemia (Insufficient Therapeutic Effect (ITE) occurred earlier in the placebo group than in the active treatment groups. In the placebo group, over 80% of subjects had withdrawn for this reason by Week 9, whereas withdrawals due to parasitaemia did not reach 80% until Week 18 in the tafenoquine group and Week 12 in the mefloquine group.

4.3 Protocol Violations

A total of 24 subjects (8.0%) had protocol violations which led to exclusion from the per-protocol population. The reasons for violation in each group are summarised for the intent-to-treat population in Table 10.

Table 10 Protocol violations leading to exclusion from the per-protocol population (number (%) of subjects)

Protocol Violation	Treatment Group		
	Placebo<br>N=99	Tafenoquine 200 mg<br>N=102	Mefloquine 250 mg<br>N=99
At least one violation	7 (7.1%)	8 (7.8%)	9 (9.1%)
Received other anti-malarial	1 (1.0%)	1 (1.0%)	1 (1.0%)
Non-compliance with halofantrine	0	0	1 (1.0%)
Non-compliance with loading medication	0	1 (1.0%)	1 (1.0%)
Did not complete prophylactic phase	6 (6.1%)	6 (5.9%)	7 (7.1%)

Data source: Table 11.5.2 in Section 11; Listing B.06 in Appendix B
NB Subjects could have more than one violation.

The most frequently reported protocol violation in each group was failure to complete the prophylactic phase ie did not complete the 24 week study and did not achieve the study endpoint, although the numbers were low.

A listing by subject of inclusion and exclusion criteria violations is presented in Listing B.07 in Appendix B.

4.4 Demographic and Baseline Characteristics

4.4.1 Demographic Characteristics

Demographic details for the intent-to-treat population are summarised in Table 11

Table 11 Demographic characteristics: intent-to-treat population

Demographic Characteristic	Treatment Group		
	Placebo<br>N=99	Tafenoquine 200 mg<br>N=102	Mefloquine 250 mg<br>N=99
**Sex n(%)**			
male	63 (63.6%)	66 (64.7%)	66 (66.7%)
female	36 (36.4%)	36 (35.3%)	33 (33.3%)
**Age (years)**			
<18	1 (1.0%)	2 (2.0%)	2 (2.0%)
18-25	40 (40.4%)	51 (50.0%)	43 (43.4%)
26-35	22 (22.2%)	20 (19.6%)	25 (25.3%)
36-45	14 (14.1%)	15 (14.7%)	22 (22.2%)
46-55	21 (21.2%)	14 (13.7%)	7 (7.1%)
>55	1 (1.0%)	0	0
mean (sd)	32.0 (11.9)	29.5 (11.2)	29.4 (10.4)
range	17 – 56	17 – 54	17 – 55
**Race n(%)**			
Black	99 (100%)	102 (100%)	99 (100%)
**Weight (kg)**			
mean (sd)	60.1 (7.9)	61.0 (8.5)	61.9 (10.0)
range	44.0 – 84.0	42.0 – 90.0	40.0 – 97.0

Data source: Tables 11.6.1a, 11.6.2a in Section 11; Listing B.08 in Appendix B

There were no marked differences between the groups in demographic characteristics. Approximately two thirds of subjects were male and all subjects were Black. The mean age in each group was between 29 and 32 years.

Demographic details for the per-protocol population are summarised in Table 12.

Table 12 Demographic characteristics: per-protocol population

Demographic Characteristic	Treatment Group		
	Placebo N=92	Tafenoquine 200 mg N=94	Mefloquine 250 mg N=90
**Sex n(%)**			
male	59 (64.1%)	61 (64.9%)	60 (66.7%)
female	33 (35.9%)	33 (35.1%)	30 (33.3%)
**Age (years)**			
<18	1 (1.1%)	2 (2.1%)	2 (2.2%)
18-25	37 (40.2%)	46 (48.9%)	39 (43.3%)
26-35	20 (21.7%)	18 (19.1%)	23 (25.6%)
36-45	14 (15.2%)	15 (16.0%)	20 (22.2%)
46-55	19 (20.7%)	13 (13.8%)	6 (6.7%)
>55	1 (1.1%)	0	0
mean (sd)	32.1 (11.9)	29.8 (11.2)	29.3 (10.4)
range	17 – 56	17 – 54	17 – 55
**Race n(%)**			
Black	92 (100%)	94 (100%)	90 (100%)
**Weight (kg)**			
mean (sd)	60.2 (7.9)	60.7 (8.5)	62.0 (10.3)
range	44.0 – 84.0	42.0 – 90.0	40.0 – 97.0

Data source: Tables 11.6.1b, 11.6.2b in Section 11; Listing B.08 in Appendix B

The demographic characteristics in the per-protocol population were similar to those in the intent-to-treat population.

4.4.2 Baseline Characteristics

4.4.2.1 Clinical Characteristics

Details of the subjects' malaria history are summarised in Table 13 for the intent-to-treat population.

Table 13 History of malaria: intent-to-treat population

Malaria History	Treatment Group		
	Placebo<br><br>N=99	Tafenoquine<br>200 mg<br>N=102	Mefloquine<br>250 mg<br>N=99
**History of malaria n(%)**			
yes	96 (97.0%)	100 (98.0%)	97 (98.0%)
no	3 (3.0%)	2 (2.0%)	1 (1.0%)
unknown	0	0	1 (1.0%)
**No. of attacks in last 6 months n(%)**			
0	25 (25.3%)	21 (20.6%)	32 (32.3%)
1	31 (31.3%)	36 (35.3%)	32 (32.3%)
2	19 (19.2%)	24 (23.5%)	15 (15.2%)
3	16 (16.2%)	7 (6.9%)	12 (12.1%)
4	7 (7.1%)	6 (5.9%)	3 (3.0%)
5	0	3 (2.9%)	2 (2.0%)
≥6	1 (1.0%)	5 (4.9%)	1 (1.0%)
unknown	0	0	2 (2.0%)
**Time since last attack (months)**			
subjects with data	91	98	93
median	4.0	4.0	5.0
range	1.0 – 88.0	1.0 – 70.0	1.0 – 124.0

Data source: Tables 11.8a, 11.9.1a in Section 11; Listing B.12 in Appendix B

There were no marked differences between the groups in the characteristics of the self-reported subjects' malaria history. Almost all subjects had a history of malaria with a median time in each group of either 4 or 5 months since the last attack

Details of the subjects' malaria history are summarised in Table 14 for the per-protocol population

Table 14 History of malaria: per-protocol population

Malaria History	Treatment Group		
	Placebo<br><br>N=92	Tafenoquine<br>200 mg<br>N=94	Mefloquine<br>250 mg<br>N=90
**History of malaria n(%)**			
yes	90 (97.8%)	92 (97.9%)	89 (98.9%)
no	2 (2.2%)	2 (2.1%)	1 (1.1%)
**No. of attacks in last 6 months n(%)**			
0	23 (25.0%)	20 (21.3%)	30 (33.3%)
1	28 (30.4%)	32 (34.0%)	30 (33.3%)
2	18 (19.6%)	21 (22.3%)	14 (15.6%)
3	15 (16.3%)	7 (7.4%)	9 (10.0%)
4	7 (7.6%)	6 (6.4%)	3 (3.3%)
5	0	3 (3.2%)	2 (2.2%)
≥6	1 (1.1%)	5 (5.3%)	1 (1.1%)
unknown	0	0	1 (1.1%)
**Time since last attack (months)**			
subjects with data	85	90	85
median	3.0	4.0	5.0
range	1.0 – 88.0	1.0 – 70.0	1.0 – 124.0

Data source: Tables 11.8b, 11.9.1b in Section 11; Listing B.12 in Appendix B

Characteristics of the subjects' malaria history in the per-protocol population were similar to those in the intent-to-treat population.

4.4.2.2 Parasitology

The number of subjects with malaria parasites present at screening and following treatment with halofantrine is shown by *Plasmodium* species in Table 15 for all randomised subjects.

Table 15 Baseline parasitology: all randomised subjects (number (%) of subjects)

***Plasmodium* species**	**Treatment Group**		
	**Placebo N=101**	**Tafenoquine 200 mg N=104**	**Mefloquine 250 mg N=101**
**Screening; Pre-halofantrine**			
P. falciparum	63 (62.4%)	50 (48.1%)	59 (58.4%)
P. vivax	0	0	0
P. ovale	1 (1.0%)	4 (3.8%)	2 (2.0%)
P. malariae	10 (9.9%)	8 (7.7%)	8 (7.9%)
Mixed species*	10 (9.9%)	11 (10.6%)	10 (9.9%)
No parasites	37 (36.6%)	53 (51.0%)	42 (41.6%)
**Post-halofantrine**			
P. falciparum	1 (1.0%)	0	0
P. vivax	0	0	0
P. ovale	0	0	0
P. malariae	0	0	0
Mixed species*	0	0	0
No parasites	100 (99.0%)	104 (100%)	101 (100%)

Data source: Tables 11.9.2, 11.9.4 in Section 11; Listing C.01 in Appendix C
* Indicates that more than one species was present.

At screening, between 36 and 51% of subjects had no malaria parasites. Of the subjects with a positive parasitology almost all were positive for *P. falciparum* and approximately 10% tested positive for more than one species. Other species reported were *P.ovale* and *P. malariae*

After clearance treatment with halofantrine all subjects but one in the placebo group tested negative for malaria parasites. The subject with a positive test was excluded from the ITT and per-protocol population.

Summary statistics for the parasite count at screening are provided in Table 16 for the intent-to-treat population.

Table 16 Parasite count at screening: intent-to-treat population

Parasite Count (per uL of blood)		Treatment Group		
		**Placebo N=99**	**Tafenoquine 200 mg N=102**	**Mefloquine 250 mg N=99**
P. falciparum	n	61	49	59
	median	73.3	41.8	47.3
	range	4.3 – 10654.0	3.0 – 17904.4	4.8 – 10331.9
P. ovale	n	1	3	2
	median	15.3	24.1	29.5
	range	-	9.1 – 43.9	28.7 – 30.2
P. malariae	n	9	8	8
	median	36.4	218.8	19.2
	range	10.1 – 297.6	11.0 – 338.0	5.2 – 27.8

Data source: Table 11.9.3a in Section 11; Listing C.01 in Appendix C
- Range not appropriate as only one subject tested positive.
NB *P. vivax* not included in this table as no subjects tested positive.

At screening, the median *P. falciparum* count in the placebo group was higher than the median count in the active treatment groups.

Results were similar in the per-protocol population (Table 11.9.3b in Section 11).

4.5 Presenting Conditions and Medical History

4.5.1 Medical/Surgical History

At screening, there were 174 subjects (58.0%) with prior significant medical/surgical history: 61 (61.6%) in the placebo group, 56 (54.9%) in the tafenoquine group and 57 (57.6%) in the mefloquine group (Tables 11.7.1.1 and 11.7.2.1 in Section 11; Listing B.11 in Appendix B). The most commonly reported conditions were malaria, headache and myalgia.

Active conditions at screening were reported for 202 subjects (67.3%) (Table 11.7.1.2 in Section 11). The most frequently occurring conditions (i.e. those occurring in at least 5% of subjects overall) are summarised in Table 17.

Table 17 Most commonly occurring (in ≥5% of subjects overall) active conditions at screening: intent-to-treat population (number (%) of subjects)

Active Condition	Treatment Group		
	Placebo<br>N=99	Tafenoquine 200 mg<br>N=102	Mefloquine 250 mg<br>N=99
At least one condition	66 (66.7%)	72 (70.6%)	64 (64.6%)
Serum bilirubin increased	21 (21.2%)	9 (8.8%)	13 (13.1%)
Headache	16 (16.2%)	12 (11.8%)	13 (13.1%)
Teeth disorder	9 (9.1%)	7 (6.9%)	11 (11.1%)
Abdomino-pelvic pain	12 (12.1%)	10 (9.8%)	2 (2.0%)
Back pain	10 (10.1%)	6 (5.9%)	6 (6.1%)
Joint pain	5 (5.1%)	8 (7.8%)	3 (3.0%)
Myalgia	5 (5.1%)	3 (2.9%)	8 (8.1%)

Data source: Table 11.7.2.2 in Section 11; Listing B.11 in Appendix B

In general, the treatment groups were similar at screening with respect to active conditions, although the placebo group did show higher frequencies of some conditions than the tafenoquine and/or mefloquine groups, such as increased bilirubin, abdomino-pelvic pain and back pain. None of these was felt to have clinical relevance on the conduct of the study.

4.5.2 Pregnancy Screen

All female subjects underwent a serum/urine pregnancy test at screening and also within 48 hours of the start of randomised study medication. There were no subjects with positive test results (Table 11.2; Listing B.03 in Appendix B) Listing B.09 in Appendix B).

4.5.3 G6PD Status

G6PD status was determined for all subjects on two occasions during the screening period and only subjects with a ‘normal’ reading on both occasions were eligible for the study. Forty five subjects (8.7%) of the 517 screened for the study were G6PD deficient. A further 5 subjects (1.0%) did not undergo the second G6PD test (Listing B.04 in Appendix B). These subjects were excluded from the study.

A listing by subject of G6PD test results for the intent-to-treat population is presented in Listing B.10 in Appendix B.

4.6 Baseline Signs and Symptoms

Baseline signs and symptoms were reported for 151 subjects (49.3%) (Table 11.10.1 in Section 11). The most frequently occurring conditions (i.e. those occurring in at least 2% of subjects overall) are summarised in Table 18.

Table 18 Most commonly occurring (in ≥2% of subjects overall) baseline signs and symptoms: safety population (number (%) of subjects)

Baseline Sign/Symptom	Treatment Group		
	Placebo<br>N=101	Tafenoquine 200 mg<br>N=104	Mefloquine 250 mg<br>N=101
At least one sign/symptom	51 (50.5%)	54 (51.9%)	46 (45.5%)
Headache	11 (10.9%)	12 (11.5%)	14 (13.9%)
Coughing	9 (8.9%)	7 (6.7%)	2 (2.0%)
Back pain	6 (5.9%)	3 (2.9%)	6 (5.9%)
Upper respiratory tract infection	5 (5.0%)	3 (2.9%)	7 (6.9%)
Abdominal pain	4 (4.0%)	6 (5.8%)	3 (3.0%)
Arthralgia	5 (5.0%)	3 (2.9%)	4 (4.0%)
Pruritus	5 (5.0%)	4 (3.8%)	3 (3.0%)
Myalgia	1 (1.0%)	5 (4.8%)	2 (2.0%)
Bronchitis	4 (4.0%)	1 (1.0%)	2 (2.0%)
Injury	1 (1.0%)	5 (4.8%)	1 (1.0%)
Chest pain	2 (2.0%)	3 (2.9%)	1 (1.0%)
Dyspepsia	4 (4.0%)	1 (1.0%)	1 (1.0%)

Data source: Table 11.10.2 in Section 11; Listing B.13 in Appendix B

In general, the treatment groups were similar at screening with respect to baseline signs and symptoms and the most commonly occurring event in all groups was headache.

There were no subjects with baseline signs and symptoms leading to withdrawal (Table 11.10.3 in Section 11).

4.7 Prior and Concomitant Medications

4.7.1 Prior Medication

A total of 236 subjects (78.7%) had received prior medications; 83 (83.8%) in the placebo group, 79 (77.5%) in the tafenoquine group and 74 (74.7%) in the mefloquine group (Tables 11.11.1.1 and 11.11.1.2 in Section 11; Listing B.14.1 in Appendix B). The most commonly received medications were paracetamol (47.3%) and ibuprofen (22.7%).

4.7.2 Concomitant Medication

A total of 276 subjects (92.0%) received concomitant medication during the study (Table 11.11.2.1 in Section 11). The most frequently received medications (i.e. those received by at least 10% of subjects in any treatment group) are summarised in Table 19.

Table 19 Most frequently received (by ≥10% of subjects in any treatment group) concomitant medication: intent-to-treat population (number (%) of subjects)

Concomitant Medication	Treatment Group		
	Placebo<br>N=99	Tafenoquine 200 mg<br>N=102	Mefloquine 250 mg<br>N=99
At least one medication	91 (91.9%)	95 (93.1%)	90 (90.9%)
Paracetamol	59 (59.6%)	65 (63.7%)	72 (72.7%)
Ibuprofen	51 (51.5%)	60 (58.8%)	52 (52.5%)
Cough syrup/medication	24 (24.2%)	34 (33.3%)	27 (27.3%)
Aluminium hydroxide	32 (32.3%)	34 (33.3%)	24 (24.2%)
Magnesium hydroxide	32 (32.3%)	34 (33.3%)	24 (24.2%)
Chlorphenamine	33 (33.3%)	28 (27.5%)	41 (41.4%)
Amoxycillin	27 (27.3%)	27 (26.5%)	25 (25.3%)
Indometacin	13 (13.1%)	19 (18.6%)	15 (15.2%)
Sodium citrate	12 (12.1%)	18 (17.6%)	9 (9.1%)
Menthol	4 (4.0%)	18 (17.6%)	5 (5.1%)
Methyl salicylate	4 (4.0%)	18 (17.6%)	5 (5.1%)
Camphor	3 (3.0%)	18 (17.6%)	5 (5.1%)
Capsicum	3 (3.0%)	18 (17.6%)	5 (5.1%)
Clove oil	3 (3.0%)	18 (17.6%)	5 (5.1%)
Eucalyptus oil	3 (3.0%)	18 (17.6%)	5 (5.1%)
Lemon grass	3 (3.0%)	18 (17.6%)	5 (5.1%)
Turpentine oil	3 (3.0%)	18 (17.6%)	5 (5.1%)
Ammonium chloride	12 (12.1%)	17 (16.7%)	9 (9.1%)
Pseudoephedrine	15 (15.2%)	15 (14.7%)	11 (11.1%)
Metronidazole	12 (12.1%)	15 (14.7%)	11 (11.1%)
Norfloxacin	7 (7.1%)	14 (13.7%)	10 (10.1%)
Clotrimazole	5 (5.1%)	14 (13.7%)	10 (10.1%)
Ciprofloxacin	11 (11.1%)	13 (12.7%)	8 (8.1%)
Benzylpenicillin	2 (2.0%)	11 (10.8%)	7 (7.1%)
Erythromycin	10 (10.1%)	11 (10.8%)	6 (6.1%)
Bisacodyl	3 (3.0%)	8 (7.8%)	10 (10.1%)
Lozenges	6 (6.1%)	3 (2.9%)	12 (12.1%)

Data source: Table 11.11.2.2 in Section 11; Listing B.14.2 in Appendix B

The most frequently received concomitant medications in all treatment groups were paracetamol and ibuprofen. In general, the groups were similar with respect

to concomitant medication, although a higher proportion of subjects in the tafenoquine group received treatment with aromatic oils. This was mostly in the form of a compound containing eight of these ingredients called Nopen Balm.

4.7.3 Follow-up Medication

A total of 272 subjects (90.7%) received medication prescribed during the follow-up period after the end of prophylaxis; 94 (94.9%) in the placebo group, 90 (88.2%) in the tafenoquine group and 88 (88.9%) in the mefloquine group (Tables 11.11.3.1 and 11.11.3.2 in Section 11; Listing B.14.3 in Appendix B). Table 20 shows that the most commonly received medications were paracetamol, ibuprofen, doxycycline and quinine. A higher proportion of the subjects in the placebo group went on to receive anti-malarial drugs than in the tafenoquine and mefloquine groups.

Table 20 Most Commonly Received Medication During Follow-up

Follow-up Medication	Treatment Group		
	Placebo<br><br>N=94	Tafenoquine<br>200 mg<br>N=90	Mefloquine<br>250 mg<br>N=88
Paracetamol	67 (59.6%)	62 (60.8%)	65 (65.7%)
Ibuprofen	41 (41.4%)	38 (37.3%)	39 (39.4%)
Doxyclycline	49 (49.5%)	34 (33.3%)	27 (27.3%)
Quinine	48 (48.5%)	31 (30.4%)	25 (25.3%)

Data source:Table 11.11.3..2 in Section 11

4.8 Treatment Compliance

At least 99% of subjects in each treatment group were compliant with halofantrine dosing and at least 99% of subjects in each treatment group were compliant with prophylactic study medication. Compliance is summarised for each treatment group in Table 21.

Table 21 Study medication compliance: intent-to-treat population (number (%) of subjects)

Compliance with Study Medication	Treatment Group		
	Placebo<br>N=99	Tafenoquine 200 mg<br>N=102	Mefloquine 250 mg<br>N=99
Compliant with halofantrine clearance medication	99 (100%)	102 (100%)	98 (99.0%)
Compliant with loading dose regimen	99 (100%)	101 (99.0%)	98 (99.0%)
Compliant with weekly dose regimen	99 (100%)	102 (100%)	99 (100%)
Compliant with overall prophylactic study medication	99 (100%)	101 (99.0%)	98 (99.0%)

Data source: Table 11.12 in Section 11; Listings B.15, B.16 in Appendix B

NB Compliant with clearance medication = received three or more halofantrine capsules on consecutive days.
Compliant with loading dose regimen = received three doses of prophylactic study medication with no more than one day between doses.
Compliant with weekly dose regimen = received weekly doses of prophylactic study medication with no dose gaps of >21 days and no more than two dose gaps of >14 days.
Compliant with overall prophylactic study medication = compliant with both loading dose and weekly dose regimen (see Section 3.13.4.1).

5 Efficacy Results

5.1 Efficacy Evaluation

5.1.1 Data Sets Analysed

The principal efficacy analysis was based on the intent-to-treat population and the per-protocol population was used to confirm the findings of the principal analysis.

5.2 Primary Efficacy Parameters

The primary efficacy variable was the prophylactic outcome based on a 'first positive' smear ie the first occurrence of a single positive smear at any time during the prophylactic treatment period and up to 7 days after the last dose. This was used to calculate protective efficacy.

It was noted early during the study that there was a higher than expected failure rate being seen and investigations were set up to determine the cause. These strongly indicated that the microscopists were reading false positive results (see Section 6.0 below). The following results cannot therefore be treated as an accurate reflection of the true chemoprophylactic efficacy of tafenoquine (or mefloquine).

5.2.1 Principal Analysis

Prophylactic outcome and protective efficacy based on first positive smear (all species) for all treatment groups during prophylactic treatment is summarised in Table 22 for the intent-to-treat population.

Table 22 Prophylactic outcome and protective efficacy during the prophylaxis treatment period: intent-to-treat population (number (%) of subjects)

Prophylactic Outcome	Treatment Group		
	Placebo<br><br>N=99	Tafenoquine 200 mg<br>N=102	Mefloquine 250 mg<br>N=99
Prophylactic failure	93 (93.9%)	90 (88.2%)	92 (92.9%)
Prophylactic success (total*)	6 (6.1%)	12 (11.8%)	7 (7.1%)
Prophylactic success (known**)	0	6 (5.9%)	0
Prophylactic success (assumed†)	6 (6.1%)	6 (5.9%)	7 (7.1%)
PE†† (%)	-	6.1%	1.1%
95.2% CI	-	-2.8%, 15.0%	-7.4%, 9.1%

Data source: Table 12.1a in Section 12; Listing C.02 in Appendix C
* Total success = known successes + assumed successes.
** Known success = subject had negative smears up to and including Week 24.
† Assumed success = subject had negative smears throughout the study but withdrew prior to Week 24.
†† Protective efficacy relative to placebo based on failure rate.

There was an apparent high failure rate in all treatment groups, although the lowest failure rate was seen in the tafenoquine group. In addition, the only known prophylactic successes occurred in the tafenoquine group. The observed protective efficacy of tafenoquine compared with placebo was 6.1%.

5.2.2 Confirmatory Analyses

Per-protocol Analysis

Prophylactic outcome based on first positive smear (all species) for all treatment groups during prophylactic treatment is summarised in Table 23 for the per-protocol population.

Table 23 Prophylactic outcome during the prophylaxis treatment period: per-protocol population (number (%) of subjects)

Prophylactic Outcome	Treatment Group		
	Placebo<br>N=92	Tafenoquine 200 mg<br>N=94	Mefloquine 250 mg<br>N=90
Prophylactic failure	92 (100%)	88 (93.6%)	90 (100%)
Prophylactic success (known*)	0	6 (6.4%)	0

Data source: Table 12.1b in Section 12; Listing C.02 in Appendix C
* All successes were known successes as withdrawals were removed from the per-protocol population.
NB Protective efficacy and associated confidence intervals are not presented due to the extreme nature of the data ie lack of treatment success in the placebo and mefloquine groups.

The results in the intent-to-treat population were similar to those in the per-protocol population

Incidence Density

The protective efficacies of tafenoquine and mefloquine during prophylactic treatment based on incidence density (see Section 3.13.4.1) are summarised in Table 24 for the intent-to-treat population.

Table 24 Protective efficacy based on incidence density during the prophylaxis treatment period: intent-to-treat population

Protective Efficacy (based on incidence density)	Treatment Group		
	Placebo<br>N=99	Tafenoquine 200 mg<br>N=102	Mefloquine 250 mg<br>N=99
Failures	93	90	92
Exposure*	12.5	19.6	16.7
Incidence density*	7.4	4.6	5.5
95% CI for incidence density	6.1, 9.1	3.7, 5.7	4.5, 6.8
PE** (%)	-	38.2%	25.8%
95.2% CI for PE	-	17.4%, 53.7%	1.1%, 44.3%

Data source: Table 12.2 in Section 12; Listing C.02 in Appendix C
* Expressed in person-years.
** Protective efficacy relative to placebo based on incidence density.

Based on incidence density, the observed protective efficacy of tafenoquine compared with placebo was 38.2%. This compares to a prophylactic efficacy of 25.8% on mefloquine.

Worst Case Analysis

Worst case prophylactic outcome (see Section 3.13.4.1) for all treatment groups during the prophylactic treatment period is summarised in Table 25 for the intent-to-treat population.

Table 25 Prophylactic outcome (worst case) during the prophylaxis treatment period: intent-to-treat population (number (%) of subjects)

**Prophylactic Outcome (worst case)**	**Treatment Group**		
	**Placebo N=99**	**Tafenoquine 200 mg N=102**	**Mefloquine 250 mg N=99**
Prophylactic failure*	99 (100%)	96 (94.1%)	99 (100%)
Prophylactic success	0	6 (5.9%)	0

Data source: Table 12.3 in Section 12; Listing C.02 in Appendix C
* Subjects classified as assumed successes (see Table 22) are classified in the worst case analysis as prophylactic failures.
NB Protective efficacy and associated confidence intervals are not presented due to the extreme nature of the data. ie lack of treatment success in the placebo and mefloquine groups.

In the worst case analysis the only prophylactic successes were in the tafenoquine group.

5.3 Secondary Efficacy Parameters

5.3.1 Two Consecutive Positive Smears During Prophylactic Treatment

Prophylactic outcome and protective efficacy based on two consecutive positive smears (all species) during the prophylactic treatment period is summarised in Table 26 for the intent-to-treat population.

Table 26 Prophylactic outcome (two consecutive positive smears) and protective efficacy during the prophylaxis treatment period: intent-to-treat population (number (%) of subjects)

**Prophylactic Outcome (two consecutive positive smears)**	**Treatment Group**		
	**Placebo N=99**	**Tafenoquine 200 mg N=102**	**Mefloquine 250 mg N=99**
Prophylactic failure	44 (44.4%)	10 (9.8%)	19 (19.2%)
Prophylactic success (total*)	55 (55.6%)	92 (90.2%)	80 (80.8%)
Prophylactic success (known**)	0	7 (6.9%)	5 (5.1%)
Prophylactic success (assumed†)	55 (55.6%)	85 (83.3%)	75 (75.8%)
PE†† (%)	-	77.9%	56.8%
95% CI	-	59.7%, 88.2%	32.5%, 72.9%

Data source: Table 12.4a in Section 12; Listing C.02 in Appendix C
* Total success = known successes + assumed successes.
** Known success = subject had negative smears up to and including Week 24.
† Assumed success = subject had negative smears throughout the study but withdrew prior to Week 24.
†† Protective efficacy relative to placebo based on failure rate.

Prophylactic outcome based on two consecutive positive smears showed a higher rate of prophylactic success compared to that based on a single positive smear. The rate of prophylactic success was 90.2% in the tafenoquine group and 80.8% in the mefloquine group, compared with 55.6% in the placebo group. Most of these prophylactic successes, however, were assumed successes (see Section 3.13.3.1) due to the high rate of premature withdrawals. The protective efficacy of tafenoquine compared with placebo was 77.9% with the lower limit of the 95% confidence interval being only 59.7%. In the mefloquine group, the protective efficacy compared with placebo was 56.8% (95% LLCI of 32.5%).

Results were similar in the per-protocol population (Table 12.4b in Section 12).

5.3.2 First Positive Smear (*P. falciparum* only) During Prophylactic Treatment

Prophylactic outcome based on first positive smear (*P. falciparum* only) and protective efficacy during prophylactic treatment is summarised in Table 27 for the intent-to-treat population.

Table 27 Prophylactic outcome (*P. falciparum* only) and protective efficacy during the prophylaxis treatment period: intent-to-treat population (number (%) of subjects)

**Prophylactic Outcome (*P. falciparum* only)**	**Treatment Group**		
	**Placebo N=99**	**Tafenoquine 200 mg N=102**	**Mefloquine 250 mg N=99**
Prophylactic failure	92 (92.9%)	89 (87.3%)	92 (92.9%)
Prophylactic success (total*)	7 (7.1%)	13 (12.7%)	7 (7.1%)
Prophylactic success (known**)	0	6 (5.9%)	0
Prophylactic success (assumed†)	7 (7.1%)	7 (6.9%)	7 (7.1%)
PE†† (%)	-	6.1%	0
95% CI	-	-3.2%, 15.3%	-8.9%, 8.2%

Data source: Table 12.5a in Section 12; Listing C.02 in Appendix C
* Total success = known successes + assumed successes.
** Known success = subject had negative smears up to and including Week 24.
† Assumed success = subject had negative smears throughout the study but withdrew prior to Week 24.
†† Protective efficacy relative to placebo based on failure rate.

Protective efficacy based on first smear positive for *P. falciparum* only was similar to protective efficacy based on first smear positive for any species (see Section 5.2.1). As before, the observed protective efficacy of tafenoquine was low.

Results were similar in the per-protocol population (Table 12.5b in Section 12).

5.3.3 Time to First Positive Smear

The time to first positive smear is summarised in Table 28 for the intent-to-treat population.

Table 28 Time to first positive smear: intent-to-treat population

**Time to First Positive Smear (days)**	**Treatment Group**		
	**Placebo N=99**	**Tafenoquine 200 mg N=102**	**Mefloquine 250 mg N=99**
Median	43	57	50
95% CI	42, 50	53, 70	44, 64

Data source: Table 4 in the Statistical Appendix in Appendix H

There was a statistically significant difference between the survival curves over the three treatment groups ($p<0.01$, Table 5 in the Statistical Appendix in

Appendix H), with Figure 2 suggesting that this might be due to the difference between tafenoquine and placebo (analysis not performed). However this result should be treated with caution as almost all subjects had a positive smear by the end of the study. Although in most survival analyses delaying the onset of the event is a successful outcome, for malaria prophylaxis the primary objective is to prevent people from experiencing the event at all. The survival curve is shown in Figure 2 below.

Figure 2 Time to first positive smear: intent -to- treat population

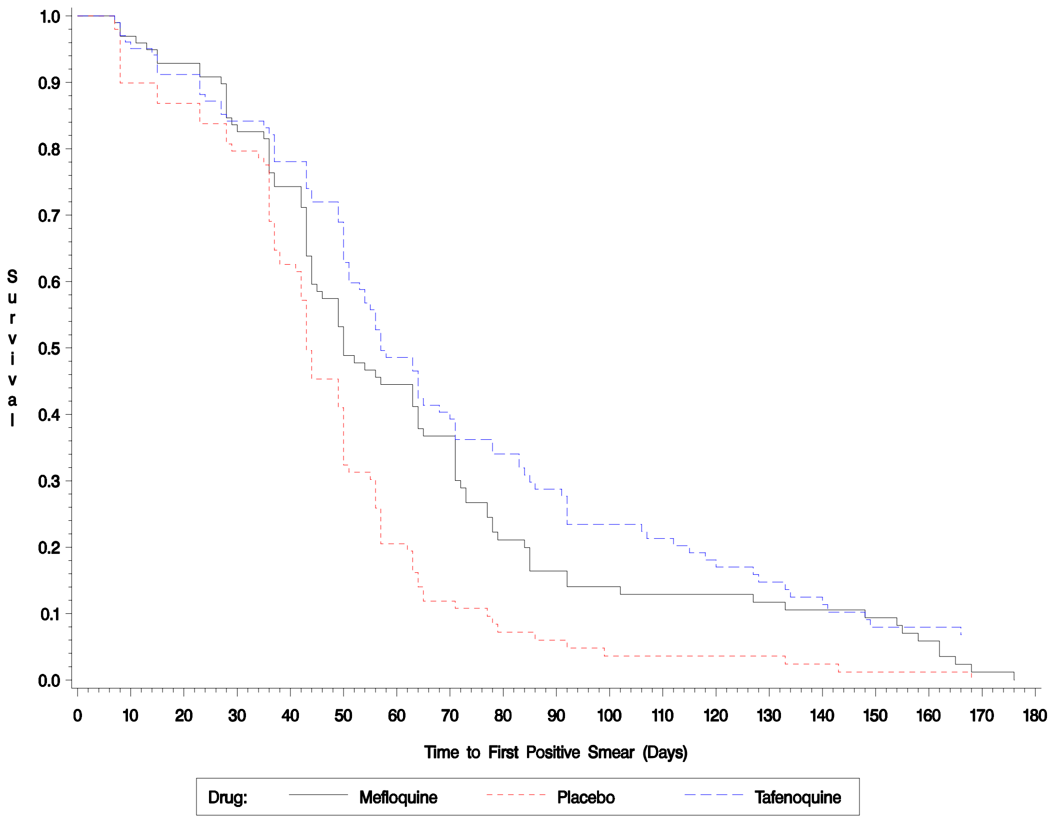

Results were similar in the per-protocol population (Tables 4 and 5 in the Statistical Appendix in Appendix H, and Figure 2 in Section 14).

5.3.4 Time to Two Consecutive Positive Smears

The time to two consecutive positive smears is summarised in Table 29 for the intent-to-treat population.

Table 29 Time to two consecutive positive smears: intent-to-treat population

Time to Two Consecutive Positive Smears (days)	Treatment Group		
	Placebo<br>N=99	Tafenoquine 200 mg<br>N=102	Mefloquine 250 mg<br>N=99
No. of subjects with positive smears	44	10	19
Median	63	NE	165
95% CI	57, 79	-	154, NE

Data source: Table 4 in the Statistical Appendix in Appendix H
NB NE = no estimate available

For the tafenoquine and placebo groups there was a statistically significant difference between the survival curves ($p<0.01$; Table 5 in the Statistical Appendix in Appendix H). As mentioned previously, although in most survival analyses delaying the onset of the event is a successful outcome, for malaria prophylaxis the primary objective is to prevent people from experiencing the event at all . The survival curve is provided in Figure 3 below.

Figure 3 Time to two consecutive positive smears: intent-to-treat population

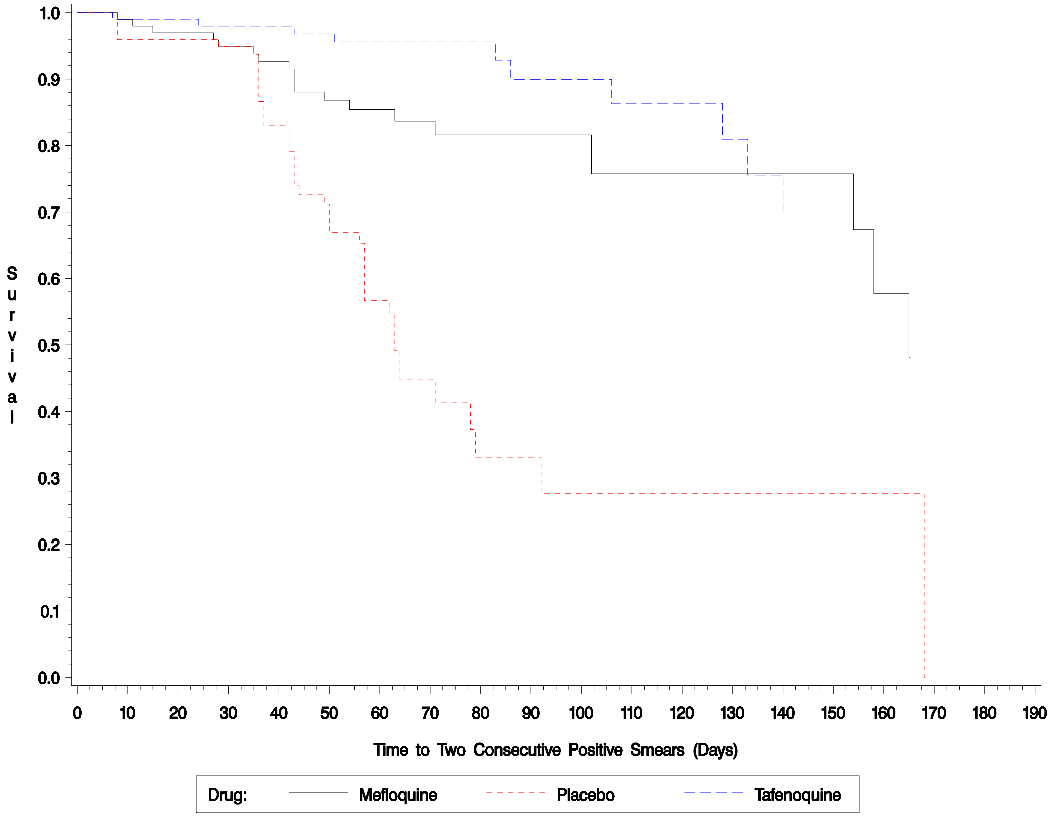

Results were similar in the per-protocol population (Tables 4 and 5 in the Statistical Appendix in Appendix H, and Figure 4 in Section 14).

The median time to two consecutive positive smears for those subjects who had two consecutive positive smears is provided in Tables 12.7a and 12.7b for the intent-to-treat and per-protocol populations, respectively.

5.4 Other Efficacy Variables

5.4.1 First Positive Smear by Species

The number of subjects with a first positive smear during prophylactic treatment is summarised by *Plasmodium* species in Table 30.

Table 30 First positive smear by *Plasmodium* species: intent-to-treat population (number of subjects)

Plasmodium species	Treatment Group		
	Placebo<br><br>N=99	Tafenoquine 200 mg<br>N=102	Mefloquine 250 mg<br>N=99
P. falciparum	92 (92.9%)	89 (87.3%)	92 (92.9%)
P. vivax	0	0	0
P. ovale	3 (3.0%)	3 (2.9%)	1 (1.0%)
P. malariae	0	1 (1.0%)	0
Mixed species*	2 (2.0%)	3 (2.9%)	1 (1.0%)
No parasites	6 (6.1%)	12 (11.8%)	7 (7.1%)

Data source: Table 12.8a in Section 12; Listing C.01 in Appendix C
* Indicates that more than one species was present.

Almost all subjects with a first positive smear were positive for *P. falciparum*, and only small numbers of subjects were positive for other *Plasmodium* species (*P.ovale* and *P.malariae*)

Results were similar in the per-protocol population (Table 12.8b in Section 12).

Summary statistics for the parasite count at first positive smear are provided in Table 31 for the intent-to-treat population.

Table 31 Parasite count at first positive smear: intent-to-treat population

Parasite Count (per uL of blood)		Treatment Group		
		Placebo<br><br>N=99	Tafenoquine 200 mg<br>N=102	Mefloquine 250 mg<br>N=99
P. falciparum	n	92	89	91*
	median	23.7	20.4	15.3
	range	1.1 – 10041.8	2.3 – 153.6	1.1 – 2194.9
P. ovale	n	3	3	0*
	mean (sd)	20.0	15.8	-
	range	13.3 – 26.0	13.8 – 153.3	-

Data source: Table 12.9a in Section 12; Listing C.01 in Appendix C
* Subject 030.001.00283 is not included because the parasite count could not be converted from ‘per 500 WBC’ to ‘per uL of blood’.

The median parasite count for *P. falciparum* was similar in all three treatment groups, although the maximum parasite count was considerably lower in the tafenoquine group than the other two groups.

Results were similar in the per-protocol population (Table 12.9b in Section 12).

5.4.2 Malaria Status

It had been the intention to tabulate the malaria status by severity to look for trends in the severity of the malaria experienced in each treatment group. However, due to the false positive slide readings it was not possible to draw any meaningful conclusions from the results. This table can be found in Table 12.10a in Section 12.

6 Investigations into High Failure Rate During Study

Following the high apparent prophylactic failure rate in the study, initially observed early July 2000, activities were set in motion to elucidate the cause. These involved set up of an IDMC and a series of investigations to eliminate possible technical problems. All these activities were conducted in parallel with one another.

6.1 IDMC Review of Safety and Efficacy

The IDMC met on 21st August 2000. They reviewed the protective efficacies of the two active therapies, tafenoquine and mefloquine, relative to placebo and found that both drugs were providing apparently low protective efficacies ie. it was not an issue with one of the treatment arms. At the time of their review, 75/101 (74.3%) of tafenoquine subjects, 85/98 (86.7%) of mefloquine subjects and 91/99 (91.9%) of placebo subjects had failed prophylaxis according to the USAMRU-K slide-reading results. This translated into protective efficacies relative to placebo of 19.2% (95% CI; 8.7%, 29.9%) for tafenoquine and 5.6% (95% CI; 4.2%,15.3%) for mefloquine.

They also reviewed safety information, in the form of the serious adverse experiences reported during the study.

The conclusion of the IDMC was that the study should continue without change to the current protocol.

6.2 Technical Investigations

6.2.1 Re-reading of Slides-during study

Whilst the study was still ongoing, 364 slide pairs were provided to NAMRU-2 in Jakarta for re-reading. These were all of the first positive slides (113 duplicate pairs) up to approximately week 12, plus a random selection of negative slides (251 duplicate pairs). The results are shown in Table 32.

Table 32 Comparison of USAMRU-K and NAMRU-2 slide readings

	NAMRU-2 Reading			
**USAMRU-K Reading**	**Positive**	**Negative**	**Unevaluable**	**Total**
Positive	19	89	5	113
Negative	0	245	6	251
Total	19	334	11	364

Data source: NAMRU-2 report in Appendix A

Of the 113 slides identified as positive by the local slide readers in Kenya, only 19 were also read positive by NAMRU-2.

The re-reading at NAMRU-2 resulted in an overall concordance rate (i.e. slides determined either as positive at both sites or negative at both sites) of 74.8% (264/353 not including unevaluable slides) and a positive concordance rate of 17.6% (19/108). Therefore, assuming that the NAMRU-2 reading is correct, only 17.6% of those slides read as positive by USAMRU-K were in fact true positives. In this initial set of slides the negative concordance was 100% ie USAMRU-K did not appear to be reading false negatives.

It was the results of this initial re-reading exercise that identified the most likely reason for the observed high failure rate in the study. The study was not stopped by the IDMC since it was felt that there was no safety issue for the subjects who remained ongoing and that subjects received benefit from the medical care provided during participation in the study.

This slide set was subsequently sent to Australia Malaria Institute (AMI), Gallipoli Barracks, Queensland, for a further re-read by Professor Rieckmann and his team. Their conclusion was also that only 19 of the 113 USAMRU-K positives contained malaria parasites. Of these 19, sixteen were the same slide pairs as the NAMRU-2 positive slides; AMI also reported a further 3 slide pairs to be positive which NAMRU-2 had reported as negative and 3 slide pairs as

negative which NAMRU-2 reported as positive. No statistical comparisons between the NAMRU-2 and AMI readings were made because it cannot be guaranteed that the identity of the NAMRU-2 positive slides were not disclosed to AMI..

Full details of these initial slide re-readings are provided in Appendix A.

6.2.2 Sensitivity and Specificity of Slide Readings

A comparison of the original slide reading as performed at USAMRU-K with the result of the re-reading performed at NAMRU-2 is shown in Table 33 for all re-read slides, including those re-read after study completion, irrespective of treatment group. The all re-read slide set included all slides which could be readily located which were diagnosed as positive by USAMRU-K plus a random selection of negative slides diagnosed by the USAMRU-K slide-readers.

Table 33 Comparison of USAMRU-K and NAMRU-2 slide readings: all re-read slides

	NAMRU-2 Reading		
**USAMRU-K Reading**	**Positive**	**Negative**	**Total**
Positive	31	219	250
Negative	5	507	512
Total	36	726	762

Data source: Table 12.12a in Section 12

Assuming that the NAMRU-2 readings were correct, for all re-read slides the sensitivity (the proportion of true positives correctly identified by USAMRU-K) was 86.1% (31/36) and the specificity (the proportion of true negatives correctly identified) was 69.8% (507/726). (Table 12.13 in Section 12). The overall concordance rate was 70.6% (538/762) and the negative concordance rate was 99% (507/512). By comparison, the positive concordance rate was only 12.4% (31/250). From all the re-read slides it appears that USAMRU-K read 219 false positives out of the total slide set of 762.

Comparison of the USAMRU-K and NAMRU-2 slide readings for individual treatment groups are given in Tables 12.12b, 12.12c and 12.12d in Section 12. Whereas in the total slide set made available for re-reading, USAMRU-K slide-readers identified 81 positive slides in placebo treated subjects, 85 in tafenoquine subjects and 84 in mefloquine subjects, the NAMRU-2 slide-readers identified 32, 2 and 2 subjects in the placebo, tafenoquine and mefloquine groups respectively.

6.2.3 Parasite Resistance

At the time of writing this report no results are available. Since the high failure rate was identified as being due to false positive slide reading these samples may not be tested.

6.2.4 Drug Packaging

An investigation of the packaging and stability of the drug supplies was carried out by the sponsor’s Clinical Trial Supplies Packaging (CTS-P) department. There was no evidence that the supplies were packed or randomised incorrectly. In addition, analysis of returned medication by the sponsor's Drug Analysis Department confirmed that the supplies were within specification in terms of chemical stability and dissolution release rate.

Details of the CTS-P and the Drug Analysis reports are provided in Appendix A.

6.2.5 Analysis of Pharmacokinetic Samples

The samples used were primarily those collected following the third loading dose and at 4 weeks (pre-dose) of the prophylactic treatment phase. The analysis was carried out by the sponsor’s Drug Metabolism and Pharmacokinetics (DMPK) department.

All tafenoquine plasma concentrations were >100 ng/mL (the target minimum concentration) following the loading doses and weekly dosing up to 4 weeks after the first loading dose. Plasma concentrations observed in subjects who failed on treatment (according to USAMRU-K-diagnosed parasiataemia) and those who did not fail were similar during both loading dosing and during prophylactic treatment.

Details of the DMPK report are provided in Appendix A.

6.2.6 Procedural Errors

Since all samples analysed contained plasma concentrations of tafenoquine above the target minimum concentration, it would not appear that non-compliance with study medication or field workers not dosing subjects according to the protocol was an issue during the study.

7 Safety Results

7.1 Extent of Exposure

Halofantrine Exposure

All subjects received three days of halofantrine treatment but one subject in the mefloquine group was not assessed as compliant because the three doses had not been taken on consecutive days. (Listing B.15 in Appendix B).

Prophylactic Study Medication Exposure

The extent of exposure to prophylactic study medication is summarised in Table 34.

Table 34 Extent of exposure to prophylactic study medication: safety population (number (%) of subjects)

Extent of Exposure	Treatment Group		
	Placebo<br><br>N=101	Tafenoquine<br>200 mg<br>N=104	Mefloquine<br>250 mg<br>N=101
No. of weeks on therapy			
1	2 (2.0%)	5 (4.8%)	3 (3.0%)
2	10 (9.9%)	7 (6.7%)	8 (7.9%)
3	5 (5.0%)	6 (5.8%)	2 (2.0%)
4	5 (5.0%)	3 (2.9%)	8 (7.9%)
5	20 (19.8%)	6 (5.8%)	8 (7.9%)
6	14 (13.9%)	4 (3.8%)	14 (13.9%)
7	15 (14.9%)	14 (13.5%)	14 (13.9%)
8	11 (10.9%)	12 (11.5%)	4 (4.0%)
9	7 (6.9%)	7 (6.7%)	7 (6.9%)
10	3 (3.0%)	6 (5.8%)	10 (9.9%)
11	3 (3.0%)	2 (1.9%)	5 (5.0%)
12	1 (1.0%)	5 (4.8%)	4 (4.0%)
13	1 (1.0%)	5 (4.8%)	2 (2.0%)
14	1 (1.0%)	0	1 (1.0%)
15	0	1 (1.0%)	0
16	0	3 (2.9%)	0
17	0	3 (2.9%)	0
18	0	2 (1.9%)	1 (1.0%)
19	1 (1.0%)	2 (1.9%)	1 (1.0%)
20	1 (1.0%)	2 (1.9%)	0
21	0	2 (1.9%)	1 (1.0%)
22	0	0	0
23	0	0	5 (5.0%)
24	1 (1.0%)	7 (6.7%)	3 (3.0%)
Mean no. of days (sd)	46.8 (25.7)	68.4 (44.0)	60.5 (39.9)
Range	3.0 – 168.0	3.0 – 169.0	2.0 – 169.0

Data source: Tables 13.1.1, 13.1.2 in Section 13; Listing B.02 in Appendix B

The mean length of exposure was shorter in the placebo group (47 days) than the tafenoquine and mefloquine groups (68 and 61 days, respectively). In the placebo group, 82 subjects (81.2%) received no more than 8 weeks of prophylactic treatment, compared with 57 subjects (54.8%) in the tafenoquine group and 61 subjects (60.4%) in the mefloquine group. Only 11 subjects overall received 24 weeks of therapy and 7 of these subjects were in the tafenoquine group.

7.2 Adverse Experiences

In this section, results for the incidence of adverse experiences are reported for the interval ‘prophylaxis treatment phase’ (see Section 3.13.6) unless otherwise stated.

7.2.1 Continuing Baseline Signs and Symptoms

There was only one baseline sign/symptom which was continuing at the start of study medication in more than 5% of subjects: coughing. The mean (sd) number of days this event continued beyond Day 0 was 19.5 (32.9) days in the placebo group, 23.8 (47.6) days in the tafenoquine group and 32.5 (26.2) days in the mefloquine group (Table 13.2 in Section 13).

7.2.2 Adverse Experiences by Frequency

A total of 92 subjects (91.1%) in the placebo group, 98 (94.2%) in the tafenoquine group and 97 (96.0%) in the mefloquine group had adverse experiences during the prophylaxis treatment phase. The most commonly reported experiences (i.e. those occurring in at least 10% of subjects in any treatment group) are summarised in Table 35.

Table 35 Number (%) of subjects with the most frequently reported adverse experiences (≥10% of subjects in any treatment group): safety population

Adverse Experience	Treatment Group		
	Placebo<br><br>N=101	Tafenoquine<br>200 mg<br>N=104	Mefloquine<br>250 mg<br>N=101
At least one AE	92 (91.1%)	98 (94.2%)	97 (96.0%)
Headache	37 (36.6%)	45 (43.3%)	49 (48.5%)
Upper respiratory tract infection	17 (16.8%)	30 (28.8%)	26 (25.7%)
Back pain	12 (11.9%)	28 (26.9%)	10 (9.9%)
Myalgia	25 (24.8%)	27 (26.0%)	29 (28.7%)
Abdominal pain	24 (23.8%)	25 (24.0%)	19 (18.8%)
Coughing	13 (12.9%)	24 (23.1%)	29 (28.7%)
Infection	20 (19.8%)	19 (18.3%)	14 (13.9%)
Injury	8 (7.9%)	18 (17.3%)	14 (13.9%)
Pharyngitis	12 (11.9%)	14 (13.5%)	14 (13.9%)
Gastroenteritis	9 (8.9%)	13 (12.5%)	14 (13.9%)
Pruritus	13 (12.9%)	13 (12.5%)	11 (10.9%)
Rhinitis	21 (20.8%)	12 (11.5%)	20 (19.8%)
Dizziness	7 (6.9%)	12 (11.5%)	14 (13.9%)
Arthralgia	8 (7.9%)	12 (11.5%)	10 (9.9%)
Conjunctivitis	13 (12.9%)	12 (11.5%)	7 (6.9%)
Viral infection	7 (6.9%)	5 (4.8%)	11 (10.9%)

Data source: Tables 13.3.1.1, 13.3.2.1 in Section 13; Listings D.01, D.02 in Appendix D

The most commonly occurring event in all treatment groups was headache. Most of the adverse experiences reported by at least 10% of subjects involved pain or infections. Although there were some differences between treatment groups in the incidence of individual events, there was no consistent pattern of an increased incidence in the tafenoquine or mefloquine groups, although back pain was reported slightly more frequently in the tafenoquine group. Coughing and upper respiratory tract infection appeared to occur more frequently in the tafenoquine and mefloquine groups compared to placebo. Abdominal pain was reported at a similar incidence in the tafenoquine and placebo groups.

Summaries of adverse experiences occurring during follow-up only are provided in Tables 13.3.1.2 and 13.3.2.2 in Section 13. There were no noticeable differences between treatment groups other than the placebo group appeared to have a higher incidence of infection (10 subjects) than tafenoquine (3 subjects) or mefloquine (5 subjects).

CNS/Psychiatric Adverse Experiences

A total of 44 subjects (43.6%) in the placebo group, 52 (50.0%) in the tafenoquine group and 57 (56.4%) in the mefloquine group had CNS/psychiatric adverse experiences during the prophylaxis treatment phase. These experiences are summarised in Table 36.

Table 36 Number (%) of subjects with CNS/psychiatric adverse experiences: safety population

Adverse Experience	Treatment Group		
	**Placebo** N=101	**Tafenoquine 200 mg** N=104	**Mefloquine 250 mg** N=101
At least one AE	44 (43.6%)	52 (50.0%)	57 (56.4%)
Headache	37 (36.6%)	45 (43.3%)	49 (48.5%)
Dizziness	7 (6.9%)	12 (11.5%)	14 (13.9%)
Insomnia	1 (1.0%)	1 (1.0%)	0
Somnolence	1 (1.0%)	1 (1.0%)	1 (1.0%)
Anxiety	0	0	2 (2.0%)

Data source: Table 13.3.3.1 in Section 13; Listings D.01, D.02 in Appendix D

Apart from headache and dizziness there were very few subjects with CNS/psychiatric events. Both headache and dizziness occurred in slightly higher proportions of subjects in the tafenoquine and mefloquine groups than in the placebo group, with the highest incidence in the mefloquine group. There were also two cases of anxiety reported on mefloquine (030.001.035 and 030.001.097) with none on either placebo or tafenoquine.

A summary of CNS/psychiatric adverse experiences occurring during follow-up only is provided in Table 13.3.3.2 in Section 13. Headache was experienced approximately equally in all three groups, and there was one incident of vertigo in the mefloquine group.

7.2.3 Adverse Experiences by Relationship to Study Medication

A total of 13 subjects (12.9%) in the placebo group, 20 (19.2%) in the tafenoquine group and 17 (16.8%) in the mefloquine group had adverse experiences during the prophylaxis treatment phase with a suspected/probable relationship to study treatment. The most commonly reported experiences related to study treatment (i.e. those occurring in at least two subjects in any treatment group) are summarised in Table 37.

Table 37 Number (%) of subjects with the most frequently reported adverse experiences (≥2 subjects in any treatment group) related to study treatment: safety population

Adverse Experience Related to Study Treatment	Treatment Group		
	Placebo N=101	Tafenoquine 200 mg N=104	Mefloquine 250 mg N=101
At least one AE	13 (12.9%)	20 (19.2%)	17 (16.8%)
Dizziness	2 (2.0%)	5 (4.8%)	7 (6.9%)
Headache	1 (1.0%)	4 (3.8%)	2 (2.0%)
Pruritus	1 (1.0%)	2 (1.9%)	2 (2.0%)
Anorexia	1 (1.0%)	2 (1.9%)	0
Dyspepsia	0	2 (1.9%)	0
Bilirubinaemia	0	2 (1.9%)	0
Fatigue	2 (2.0%)	1 (1.0%)	0
Maculo-papular rash	2 (2.0%)	0	0

Data source: Table 13.3.5.1 in Section 13; Listings D.01, D.02 in Appendix D
NB Includes adverse experiences with an unknown relationship to study treatment.

In the tafenoquine group, the most commonly reported events classified as related to study treatment were dizziness and headache, and in the mefloquine group the most commonly reported event was dizziness. The incidence of these events was higher than in the placebo group, but the numbers of subjects affected were low.

A summary of adverse experiences occurring during follow-up only with a suspected/probable relationship to study treatment is provided in Table 13.3.5.2 in Section 13. There were no trends noted.

7.2.4 Adverse Experiences by Severity

The intensity of the adverse experiences during the prophylaxis treatment phase is summarised in Table 38.

Table 38 Number (%) of subjects with adverse experiences by intensity: safety population

Intensity	Treatment Group		
	Placebo<br><br>N=101	Tafenoquine<br>200 mg<br>N=104	Mefloquine<br>250 mg<br>N=101
At least one AE	92 (91.1%)	98 (94.2%)	97 (96.0%)
Mild	91 (90.1%)	98 (94.2%)	96 (95.0%)
Moderate	18 (17.8%)	19 (18.3%)	23 (22.8%)
Severe	2 (2.0%)	4 (3.8%)	1 (1.0%)
Unknown	0	0	2 (2.0%)

Data source: Table 13.3.4.1 in Section 13; Listings D.01, D.02 in Appendix D

Most adverse experiences were of mild or moderate intensity and the distribution of mild/moderate adverse experiences was similar in all treatment groups.

There were few subjects with severe adverse experiences, these are summarised in Table 39.

Table 39 Number (%) of subjects with severe adverse experiences: safety population

Severe Adverse Experience	Treatment Group		
	Placebo<br><br>N=101	Tafenoquine<br>200 mg<br>N=104	Mefloquine<br>250 mg<br>N=101
At least one severe AE	2 (2.0%)	4 (3.8%)	1 (1.0%)
Infection	1 (1.0%)	2 (1.9%)	0
Cellulitis	0	1 (1.0%)	0
Headache	0	1 (1.0%)	0
Anxiety	0	0	1 (1.0%)
Vomiting	1 (1.0%)	0	0

Data source: Table 13.3.4.1 in Section 13; Listings D.01, D.02 in Appendix D

The severe anxiety adverse experience (030.001.0035) was also recorded as a Serious Adverse Experience and appears in Table 43.

A summary of adverse experiences by intensity occurring during follow-up only is provided in Table 13.3.4.2 in Section 13. There were only 5 subjects with severe adverse experiences during follow-up: three in the placebo group and one in each of the tafenoquine and mefloquine groups. The severe events were infections (cellulitis, moniliasis, oesophagitis and pneumonia) and headache.

7.2.5 Adverse Experiences by Time of First Occurrence

The time of first occurrence of the most frequently reported adverse experiences are shown in Table 40.

Headache appeared most frequently in all three groups during the first week. There appeared to be no trend for the time to first occurrence of the most frequently reported adverse experiences.

Table 40 Number (%) of subjects with most frequently reported adverse experiences by time of first occurrence: safety population

Adverse Experience	N*	Time Interval									
		Wk 1	Wk 2	Wk 3	Wk 4	Wk 5-8	Wk 9-12	Wk 13-16	Wk 17-20	Wk 21-24	Wk 25+
**Placebo**											
Headache	51	24 (47.1%)	4 (7.8%)	3 (5.9%)	4 (7.8%)	10 (19.6%)	4 (7.8%)	2 (3.9%)	0	0	0
URTI	29	3 (10.3%)	2 (6.9%)	2 (6.9%)	6 (20.7%)	7 (24.1%)	8 (27.6%)	1 (3.4%)	0	0	0
Back pain	18	5 (27.8%)	0	3 (16.7%)	2 (11.1%)	5 (27.8%)	1 (5.6%)	1 (5.6%)	1 (5.6%)	0	0
Myalgia	30	8 (26.7%)	9 (32.1%)	2 (6.7%)	2 (6.7%)	5 (16.7%)	3 (10.0%)	0	1 (3.3%)	0	0
Abdominal pain	33	8 (24.2%)	3 (9.1%)	2 (6.1%)	4 (12.1%)	8 (24.2%)	7 (21.2%)	1 (3.0%)	0	0	0
Coughing	25	3 (12.0%)	1 (4.0%)	2 (8.0%)	2 (8.0%)	10 (40.0%)	7 (28.0%)	0	0	0	0
Infection	28	4 (14.3%)	5 (17.9%)	1 (3.6%)	4 (14.3%)	10 (35.7%)	3 (10.7%)	0	1 (3.6%)	0	0
**Tafenoquine 200 mg**											
Headache	57	22 (38.6%)	5 (8.8%)	6 (10.5%)	2 (3.5%)	11 (19.3%)	3 (5.3%)	2 (3.5%)	3 (5.3%)	3 (5.3%)	0
URTI	37	10 (27.0%)	1 (2.7%)	2 (5.4%)	7 (18.9%)	12 (32.4%)	3 (8.1%)	1 (2.7%)	1 (2.7%)	0	0
Back pain	35	8 (22.9%)	2 (5.7%)	4 (11.4%)	1 (2.9%)	9 (25.7%)	6 (17.1%)	3 (8.6%)	1 (2.9%)	0	1 (2.9%)
Myalgia	34	7 (20.6%)	7 (20.6%)	1 (2.9%)	3 (8.8%)	8 (23.5%)	3 (8.8%)	2 (5.9%)	0	3 (8.8%)	0
Abdominal pain	33	7 (21.2%)	4 (12.1%)	2 (6.1%)	3 (9.1%)	7 (21.2%)	6 (18.2%)	2 (6.1%)	1 (3.0%)	1 (3.0%)	0
Coughing	33	6 (18.2%)	1 (3.0%)	4 (12.1%)	2 (6.1%)	6 (18.2%)	8 (24.2%)	3 (9.1%)	0	2 (6.1%)	1 (3.0%)
Infection	21	7 (33.3%)	2 (9.5%)	1 (4.8%)	2 (9.5%)	8 (38.1%)	1 (4.8%)	0	0	0	0
**Mefloquine 250 mg**											
Headache	58	21 (36.2%)	5 (8.6%)	3 (5.2%)	7 (12.1%)	14 (24.1%)	4 (6.9%)	3 (5.2%)	0	0	1 (1.7%)
URTI	42	2 (4.8%)	3 (7.1%)	2 (4.8%)	8 (19.0%)	17 (40.5%)	8 (19.0%)	2 (4.8%)	0	0	0
Back pain	17	5 (29.4%)	0	2 (11.8%)	1 (5.9%)	2 (11.8%)	4 (23.5%)	2 (11.8%)	0	0	1 (5.9%)
Myalgia	34	9 (26.5%)	10 (29.4%)	1 (2.9%)	2 (5.9%)	6 (17.6%)	5 (14.7%)	1 (2.9%)	0	0	0
Abdominal pain	26	9 (34.6%)	1 (3.8%)	3 (11.5%)	1 (3.8%)	6 (23.1%)	3 (11.5%)	1 (3.8%)	0	2 (7.7%)	0
Coughing	31	11 (35.5%)	3 (9.7%)	1 (3.2%)	3 (9.7%)	5 (16.1%)	5 (16.1%)	2 (6.5%)	0	1 (3.2%)	0
Infection	16	5 (31.3%)	1 (6.3%)	0	1 (6.3%)	7 (43.8%)	1 (6.3%)	0	0	1 (6.3%)	0

Data source: Table 13.3.8 in Section 13; Listings D.01, D.02 in Appendix D

* Number of subjects with a particular AEs. The percentage of subjects with the AE in a particular interval is expressed as a percentage of the total number of subjects in that group with the AE.

7.3 Deaths

There were no deaths reported during the study, either during the prophylaxis treatment phase or during follow-up (Listing D.03 in Appendix D).

7.4 Serious Adverse Experiences

Eleven subjects (3.6%) had serious adverse experiences in the on-treatment period and 5 subjects (1.6%) had serious adverse experiences in the follow-up period. In addition, there were four subjects with serious adverse experiences before the start of the prophylactic medication phase. All these are summarised in Table 41.

Table 41 Number (%) of subjects with serious adverse experiences: safety population

Serious Adverse Experience	Treatment Group		
	Placebo<br><br>N=101	Tafenoquine<br>200 mg<br>N=104	Mefloquine<br>250 mg<br>N=101
**Prior to Prophylactic Study Medication**			
At least one serious AE	1(1.0%)	1 (1.0%)	2 (2.0%)
Pelvic inflammation*	0	1 (1.0%)	0
Arthritis*	0	0	1 (1.0%)
Varicella*	0	0	1 (1.0%)
Bacterial infection*	1(1.0%)	0	0
**Prophylaxis Treatment Period**			
At least one serious AE	2 (2.0%)	8 (7.6%)	1 (1.0%)
Infection	1(1.0%)	2 (1.9%)	0
Haemolytic Anaemia **	0	1(1.0%)	0
Cellulitis	0	1(1.0%)	0
Injury	0	1(1.0%)	0
Headache	0	1(1.0%)	0
Enteritis	0	1(1.0%)	0
Unintended pregnancy	0	1(1.0%)	0
Anxiety	0	0	1(1.0%)
Pneumonia	1(1.0%)	0	0
**Follow-up Period**			
At least one serious AE	2 (2.0%)	2 (1.9%)	1 (1.0%)
Pneumonia	1(1.0%)	1(1.0%)	1(1.0%)
Unintended pregnancy	0	1(1.0%)	0
Moniliasis GI	1(1.0%)	0	0
Pharyngitis	1(1.0%)	0	0

Data source: Tables 13.3.7.1, 13.3.7.2, 13.3.7.3 in Section 13; Listing B.13 in Appendix B; Listing D.02 in Appendix D; Sponsor’s safety database. Two pregancies do not appear in this table since were not reported as SAEs: 00163 and 00289 both in mefloquine arm
** This event occurred in the prophylactic phase but is reported in the Follow-up phase in Sponsor's database.
* These events are recorded only on the Sponsor’s safety database.

Only two subjects had serious adverse experiences reported with a suspected relationship to study treatment. Brief details are given below.

Subject 030.001.00261 in the tafenoquine group was a 31 year old female who developed mild haemolytic anaemia on Day 3 of the study. Her G6PD status was recorded as normal on two occasions, using the same technique, prior to entering the study. At baseline her bilirubin was slightly elevated at 38.48 umol/L (reference range 0.0-34.2 umol/L) which had increased to 174.42 umol/L 3 days later. At baseline her haemoglobin (144 g/L) and haematocrit (44%) values were within reference range, six days later both had become out of range (haemoglobin 90 g/L (ref 100-180 g/L), haematocrit 28.1% (ref 31-51%). The investigator

initially suspected acute hepatitis but after additional examinations concluded the signs and symptoms were most consistent with haemolytic anaemia. She was treated with multivitamins and ferrous sulphate and the event resolved after 25 days. The investigator considered the event to have a suspected relationship to study treatment and the subject was withdrawn from the study.

Subject 030.001.00035 in the mefloquine group was an 18 year old male who had a severe anxiety reaction on Day 2 of the study. He received treatment with diazepam and chlorpheniramine and his symptoms resolved after 4 days. The subject was withdrawn from the study and the investigator reported the event with a suspected relationship to study treatment, although other possible causes were not ruled out and illicit use of cannabis was suspected.

Narratives for all subjects with serious adverse experiences are provided in Table 13.3.7.4 in Section 13, and copies of the CRFs for these subjects are provided in Appendix G.

7.5 Withdrawals Due to Adverse Experiences

Ten subjects (3.3%) had adverse experiences during the prophylaxis treatment phase recorded as leading to withdrawal, and 5 subjects (1.6%) had adverse experiences during follow-up recorded as leading to withdrawal (two subjects had events during both phases). These are summarised in Table 42.

Table 42 Number (%) of subjects with adverse experiences leading to withdrawal: safety population

Adverse Experience Leading to Withdrawal	Treatment Group		
	Placebo N=101	Tafenoquine 200 mg N=104	Mefloquine 250 mg N=101
**Prophylaxis treatment phase**			
At least one AE	3 (3.0%)	5 (4.8%)	2 (2.0%)
Infection**	3 (3.0%)	3 (2.9%)	0
Bilirubinaemia	0	1* (1.0%)	0
Cholestatic hepatitis	0	1* (1.0%)	0
Unintended pregnancy	0	1 (1.0%)	0
Haemolytic anaemia [&]	0	1* (1.0%)	0
Abdominal pain	0	0	1† (1.0%)
Anxiety	0	0	1* (1.0%)
**Follow-up**			
At least one AE	2 (2.0%)	2 (1.9%)	0
Infection**	2 (2.0%)	1 (1.0%)	0
Pneumonia	0	1†† (1.0%)	0

Data source: Tables 13.3.6.1, 13.3.6.2 in Section 13; Listings D.01, D.02 in Appendix D
* Subjects excluded from the intent-to-treat population.
** Subjects with malaria: reason for withdrawal recorded as ‘insufficient therapeutic effect’ (see Table 9).
& This event occurred in the prophylactic phase but is reported in the Follow-up phase in Sponsor's database.
† Subject with reason for withdrawal recorded as ‘insufficient therapeutic effect’ (see Table 9) although the subject did not develop malaria but refused further medication because of abdominal pain.
†† Secondary to malaria: reason for withdrawal recorded as ‘insufficient therapeutic effect’ (see Table 9).

Most of the premature withdrawals from the study were as a result of the subjects apparently developing parasitaemia ie denoted as Infection in Table 42. The cholestatic hepatitis and bilirubinaemia occurred in the same subject (0030.001.00261) but was reported as two separate events; this was later diagnosed as haemolytic anaemia and is described in further detail in Section 7.4. Therefore three of the events reported in Table 42 relate to the same adverse experience occuring to a single study subject.

Only two subjects were withdrawn due to adverse experiences with a suspected relationship to study drug treatment. These were the serious adverse experiences of haemolytic anaemia in the tafenoquine group and axiety in the mefloquine group, discussed in further detail under the previous Section 7.4.

Narratives for the subjects withdrawn due to serious adverse experiences are provided in Table 13.3.7.4 in Section 13. Copies of the CRFs for subjects withdrawn following adverse experiences other than infection (i.e. malaria) are provided in Appendix G.

7.6 Pregnancy

There were four pregnancies reported during the treatment and post-treatment periods.

Subject 030.001.00158 in the tafenoquine group was aged 20 years and had had two previous pregnancies with normal outcomes. She received study medication from 9 May 2000 to 29 May 2000. On 5 June 2000, 7 days after the last dose, the subject was diagnosed as pregnant; her last menstrual period had been on 16 April 2000. Relevant test results included positive beta HCG. The subject gave birth by spontaneous vertex delivery on 28 January 2001, at 41 weeks gestation. Post-partum was uneventful. The neonate weighed 2.5 kg and had an Apgar score of 10/10. The subject and neonate were reported to be doing well. The subject had experienced a serious adverse experience of pelvic inflammatory disease during the screening phase of the study.

Subject 030.001.00163 in the mefloquine group was aged 27 years. She received study medication from 9 May 2000 to 26 June 2000 when medication was stopped due to a positive malaria smear. On 8 August 2000, 43 days after the last dose, the subject was discovered to be pregnant following positive HCG urine and serum results. The subject was treated by a gynaecologist on 11 August 2000 for non-serious sexually transmitted infection and dysfunctional uterine bleeding.

On 26 August 2000, the subject experienced vaginal bleeding, low abdominal pain and weakness. Relevant test results included blood pressure 123/67 mmHg, temperature 36.8°C and pulse 77 bpm. Urine microscopy revealed Gram-positive bacilli, Gram-negative cocci and was positive for pus cells and epithelial cells. The subject was initially diagnosed as having acute pelvic inflammatory disorder and was treated with amoxycillin/clavulanate, paracetamol and diclofenac. Results of further investigations revealed a pseudo sac in the uterus and a left adnexial gestation sac with a foetus of 1.5 centimetres, equivalent to 8 weeks gestation. Cardiac activity was seen and free pelvic fluid noted. The subject underwent a left salpingo-oophorectomy with subsequent termination of the left fallopian tubal ectopic pregnancy and was treated with ibuprofen, ferrous sulphate, folic acid, glucose and saline, interferon alpha and pethidine. The investigator reported the ectopic pregnancy as not related to treatment with study medication.

Subject 030.001.00173 in the tafenoquine group was aged 19 years. She received study medication from 9 May 2000. On 26 June 2000, 48 days after the first dose, the subject presented with symptoms of pregnancy. The subject’s last

menstrual period had been on 26 May 2000 and she tested positive for HCG. Treatment was stopped due to the pregnancy on 26 June 2000. The subject gave birth by spontaneous vertex delivery on 19 February 2001 at 36 weeks gestation. The neonate had a birth weight of 3.3 kg and an Apgar score of 10. There were no gross abnormalities. The subject and neonate were both stable and discharged on 19 February 2001.

Subject 030.001.00289 in the mefloquine group was aged 19 years and had had one previous full-term pregnancy. She received study medication from 11 May 2000 to 28 June 2000 when medication was stopped due to a positive malaria smear. On 31 July 2000, 33 days after the last dose of study medication, she tested positive for HCG during a safety check. Her last menstrual period had been on 7 June 2000. She was given ferrous sulphate for 30 days due to low haemoglobin. The estimated delivery date was 14 March 2001. On 23 February 2001, the subject gave birth to a healthy male child. There were no post-partum complications.

7.7 Vital Signs

Mean vital signs values at baseline and mean changes from baseline at Weeks 4, 8, 16 and 24 are summarised in Table 43.

Table 43 Mean vital signs at baseline and mean change from baseline: safety population

Vital Sign Variable	Treatment Group					
	Placebo<br><br>N=101		Tafenoquine<br>200 mg<br>N=104		Mefloquine<br>250 mg<br>N=101	
	mean	n	mean	n	mean	n
**Systolic BP (mmHg)**						
Baseline	109	101	110	104	108	101
Change at Week 4	5	82	2	83	7	87
Change at Week 8	6	26	6	54	6	39
Change at Week 16	3	3	8	20	2	10
Change at Week 24	7	1	9	7	4	2
**Diastolic BP (mmHg)**						
Baseline	67	101	66	104	64	101
Change at Week 4	3	82	3	83	3	87
Change at Week 8	2	26	7	54	6	39
Change at Week 16	2	3	4	20	4	10
Change at Week 24	2	1	8	7	10	2
**Pulse rate (bpm)**						
Baseline	67	101	67	104	66	101
Change at Week 4	3	82	4	83	4	87
Change at Week 8	5	26	11	54	4	39
Change at Week 16	10	3	3	21	-1	10
Change at Week 24	27	1	12	7	9	2
**Temperature (°C)**						
Baseline	36.6	101	36.5	104	36.6	101
Change at Week 4	-0.1	83	-0.0	85	-0.0	88
Change at Week 8	0.2	28	-0.0	56	-0.1	40
Change at Week 16	0.0	3	0.1	21	-0.1	10
Change at Week 24	0.4	1	0.3	7	0.8	2

Data source: Table 13.4.1.1 in Section 13; Listing E.01 in Appendix E

In general, there were small increases in mean blood pressure and pulse rate during treatment, but these were similar in all treatment groups considering the small number of subjects remaining in the study after Week 8. Mean temperature was little changed during treatment.

Mean vital signs values at baseline and mean changes from baseline during the safety follow-up are summarised in Table 13.4.1.2 in Section 13. In general, mean changes were small and similar in all treatment groups up to Week 8 of follow-up. Thereafter there were too few subjects remaining in follow-up to make a meaningful comparison of the treatments.

The numbers of subjects with vital signs results of potential clinical concern at any time during the prophylactic treatment period are summarised in Table 44.

Table 44 Number (%) of subjects with vital signs of potential clinical concern: safety population

Vital Sign Variable	Treatment Group					
	Placebo N=101		Tafenoquine 200 mg N=104		Mefloquine 250 mg N=101	
		n		n		n
**Systolic BP (mmHg)**						
>140	7 (8.3%)	84	8 (8.6%)	93	8 (8.5%)	94
<80	0	84	2 (2.2%)	93	1 (1.1%)	94
**Diastolic BP (mmHg)**						
>100	3 (3.6%)	83	2 (2.3%)	86	2 (2.2%)	89
**Pulse rate (bpm)**						
>100	5 (5.7%)	87	10 (10.8%)	93	6 (6.4%)	94
<50	6 (6.9%)	87	2 (2.2%)	93	4 (4.3%)	94
**Temperature (°C)**						
>37.5	2 (2.4%)	84	1 (1.2%)	86	2 (2.2%)	89

Data source: Table 13.4.2.1 in Section 13; Listing E.01 in Appendix E

The number of subjects with vital signs values flagged of potential clinical concern was generally low, and there were no marked differences between the treatment groups.

The numbers of subjects with vital signs results of potential clinical concern at each week during the prophylactic treatment period are summarised in Table 13.4.2.1 in Section 13.

The numbers of subjects with vital signs results of potential clinical concern during the safety follow-up are summarised in Table 13.4.2.2 in Section 13. There were few subjects with vital signs values flagged of potential clinical concern and no marked differences between the treatment groups.

7.8 Laboratory Tests

In general, there were only small changes from baseline in mean laboratory values during the prophylactic treatment period and few marked differences between the treatment groups (Table 13.5.1.1 in Section 13; Listings F.01 and F.02 in Appendix F). However, there did appear to be limited differences between treatment groups for a number of parameters, summarised in Table 45. In the tafenoquine group there were small mean decreases in haemoglobin and haematocrit at Weeks 4 and 8 compared to the placebo and mefloquine groups; beyond week 8 the numbers at each time-point became small, so that the mean

changes have wide confidence intervals. Platelet counts at Week 4 in the tafenoquine group also seemed to show a small mean decrease from baseline compared to placebo and mefloquine groups.

Concerning clinical chemistry parameters, there appeared to be small mean increases from baseline in creatinine in both the tafenoquine and mefloquine groups at Week 4 compared to placebo; the shift seemed a little greater in the tafenoquine group than the mefloquine group, although after Week 4 there was a tendency to return back towards baseline values.

Table 45 Mean Change from Baseline by Visit

Laboratory Test	Units	Baseline	Week 4	Week 8	Week 16	Week 24
**Haemoglobin**	G/L					
Placebo		130.0	1.1	1.3	-2.0	8.0
Tafenoquine		130.7	-4.6	-3.9	-7.8	1.0
Mefloquine		131.1	0.3	-1.2	-5.4	-5.0
**Haematocrit**	%					
Placebo		41.0	0.6	0.7	-0.6	3.2
Tafenoquine		41.3	-1.2	-0.9	-2.6	0.8
Mefloquine		41.4	0.3	-0.0	-2.0	-0.9
**Platelets**	10^9/L					
Placebo		191.4	-2.5	-15.0	-32.5	-46.0
Tafenoquine		191.0	-15.8	-10.6	-3.4	-38.8
Mefloquine		188.4	11.5	21.1	-4.1	19.0
**Creatinine**	UMOL/L					
Placebo		68.9	0.4	-8.2	-8.8	-6.8
Tafenoquine		68.2	6.3	1.2	1.0	0.4
Mefloquine		70.0	3.2	-1.5	-7.0	3.7

Data source Table 13.5.1.1 in Section 13

Mean laboratory values during the safety follow-up are summarised in Table 13.5.1.2 in Section 13. Considering the small number of subjects at some timepoints there were no marked differences between the treatment groups.

The numbers of subjects with laboratory results which were F2 flagged (i.e. had increased or decreased from baseline by more than a specified amount, see Table 4 in Section 3.13.6) during the prophylactic period of the study are summarised in Table 46.

Table 46 Number (%) of subjects with F2 flagged laboratory values during the prophylactic period: safety population

Laboratory Variable		Treatment Group					
		Placebo<br><br>N=101		Tafenoquine<br>200 mg<br>N=104		Mefloquine<br>250 mg<br>N=101	
			n		n		n
**Haematology**							
Haemaglobin g/L	Low	0	82	8 (9.4%)	85	1 (1.1%)	88
Haematocrit %	Low	11 (13.4%)	82	36 (42.4%)	85	16 (18.2%)	88
WBC 10^9/L	High	3 (3.7%)	82	3 (3.5%)	85	5 (5.7%)	88
	Low	6 (7.1%)	82	9 (10.6%)	85	9 (10.2%)	88
Platelets 10^9/L	Low	10 (11.6%)	82	15 (17.7%)	85	8 (9.1%)	88
**Clinical Chemistry**							
Creatinine UMOL/L	High	2 (2.4%)	82	4 (4.7%)	85	2 (2.3%)	88
ALT IU/L	High	0	82	0	85	1 (1.4%)	88
Total bilirubin UMOL/L	High	1 (1.2%)	82	2 (2.4%)	85	0	88

Data source: Table 13.5.2.1 in Section 13; Listing F.01 in Appendix F

The proportions of subjects with haematocrit values which had decreased from baseline outside the F2 flag range were higher in the tafenoquine group (42.4%) than in the placebo (13.4%) and mefloquine groups (18.2%). The F2 flag for haematocrit was set at a reduction to <0.95 of baseline value, so relatively small decreases were required to produce an F2 flagged value. By comparison, the F2 flag for haemoglobin was set at a reduction of ≥20.0g/L from baseline value. Review of haemoglobin data shows that smaller proportions of tafenoquine subjects (9.4%) were outside the F2 flag using this parameter compared to haematocrit, but the values were still higher than in either the placebo (0%) or mefloquine (1.1%) groups. Platelets showed a greater proportion of tafenoquine subjects (17.7%) with decreases to below the F2 flag compared to placebo (11.6%) and mefloquine (9.1%) groups.

The proportions of subjects with F2 flags for the remainder of the laboratory parameters - white blood cells, creatinine, ALT and bilirubin - showed little of note between the treatment groups.

The numbers of subjects with laboratory values F2 flagged during the safety follow-up are summarised in Table 13.5.2.2 in Section 13. There were no marked clinically relevant differences between treatment groups in this period of the study.

There was only one subject with a laboratory results which was F3 flagged during the prophylactic treatment period (i.e. fell outside an extended normal range, see Table 4 in Section 3.13.6). This was a subject 030.001.00292 in the mefloquine group with a high ALT value (Table 13.5.3.1 in Section 13). During the safety follow-up there were three subjects with laboratory results F3 flagged. Subject 030.001.00131 in the placebo group with a low platelet value, subject 030.001.00035 in the mefloquine with a low platelet value and subject 030.001.00194 in the tafenoquine with a high ALT value of 115 IU/L at Week 4 (ref range 0-42 IU/L). This had been within range at screening and there were no further follow-up blood samples taken.

There were no subjects with laboratory results which were F2F3 flagged (i.e. had increased or decreased from baseline by more than a specified amount, and fell outside an extended normal range, see Table 4 in Section 3.13.6) during the prophylactic treatment period (Table 13.5.4.1 in Section 13). During the safety follow-up there were two subjects with laboratory results F2F3 flagged: subject 030.001.00131 and 030.001.0035 in the placebo and mefloquine groups respectively with low platelet values (Table 13.5.4.2 in Section 13).

8 Pharmacokinetic Evaluation

The results of the pharmacokinetic analysis are to be presented in a separate report.

9 Discussion

This study was designed to determine the prophylactic efficacy of tafenoquine compared with placebo in chemosuppression of *P. falciparum* in subjects aged between 18 and 55 years in Kombewa in Nyanza Province, western Kenya. In addition, the effectiveness of tafenoquine compared with mefloquine was investigated, together with the safety and tolerability of tafenoquine compared with both placebo and mefloquine. An earlier study in Kenya [13] had shown that tafenoquine, taken as a 3 day loading regimen followed by once weekly at either 200 mg or 400 mg, provided a protective efficacy of >86% in preventing malaria

parasitaemia in adult volunteers on the background of a 92% malaria attack rate in the placebo group. Similar load-plus weekly dosing regimens in Ghana, at doses of 50 –200 mg, had also shown a high degree of protective efficacy (84-87%) against high rates of *P.falciparum* transmission [14]**.**

In the current study, before starting malaria prophylaxis, study subjects received three days of treatment with halofantrine to clear any existing parasitaemia. Only those subjects whose thick blood smears showed absence of malaria parasitaemia were randomised to prophylactic treatment in a 1:1:1 ratio with tafenoquine, mefloquine or placebo.

In total, 306 subjects were randomised to prophylactic treatment and 300 were eligible for the intention-to-treat population. The three treatment groups were demographically well matched. Approximately two thirds of subjects were male and all subjects were Black. The mean age in each group ranged from 29 to 32 years.

The primary efficacy variable was the prophylactic outcome of treatment, with prophylactic failure based on the first, single blood smear positive for malaria parasites as assessed by two microscopists blinded to one another's assessments. This compared to the previous study in Kenya [13] where prophylactic failure was based on diagnosing malaria parasites in two consecutive blood smears (diagnosis in one smear lead to subject recall for a second smear, up to one week later). The Ghanaian study [14], on the other hand, utilised a single positive blood smear, confirmed by two separate microscopists, as its definition of prophylactic failure for the primary efficacy variable. The latter approach was also adopted for the current study since it was felt to be the most sensitive assessment of prophylactic failure in the semi-immune population assessed in the study; presence of naturally acquired immunity to malaria infection may lead to clearance of low levels of parasitaemia and an over-estimate of the protective efficacy of test therapy if using the two consecutive smear approach.

Early in the study it became apparent that the prophylactic failure rate was higher than would be expected from the previously observed protective efficacy of both tafenoquine and mefloquine. This lead to the initiation of a series of investigations into the cause, plus the set-up of an IDMC to assess whether it was appropriate to continue with the study. The IDMC reviewed safety data (serious adverse event reports) from the study and unblinded efficacy data so that protective efficacies could be calculated; at all stages, GSK staff were blinded to the individual subject data so the validity of the blinded design of the trial was not compromised. The IDMC found evidence of low protective efficacies in both the

tafenoquine and mefloquine arms. Since it affected both active arms, rather than one, this suggested a possible technical failure in the study conduct rather than drug failure. However, the IDMC recommended that the study continue to completion since it could still fulfill one of the study objectives of obtaining some long-term safety data on the trial medication, and since review of the safety data indicated that there were no risks associated with continued exposure of trial subjects to study medication.

Investigations into the possible cause of the high failure rate were conducted concurrent with the IDMC set-up and review. These investigations included re-reading a batch of 'first positive' slides and a selection of negative slides at a second experienced slide-reading site, NAMRU-2 in Jakarta, using the same slide-reading protocol as used at the USAMRU-K study site. NAMRU-2 used slide-readers who had passed exams as part of a Quality Assurance process designed to ensure a high degree of specificity and sensitivity were achieved and maintained with slide-reading personnel. Of the initial 113 slides read as positive by USAMRU-K slide readers only 19 were read positive by NAMRU-2. A second re-reading site, the Australian Army Malaria Institute (AMI), also found only 19 positive slides in this batch of 113 positives from USAMRU-K, with agreement on the identity of 16 of the 19 positive slides. This second AMI re-read result gives confidence in the NAMRU-2 re-read and, taken together, would tend to suggest that the USAMRU-K local study slide readers had been reporting false positive blood smear results.

In order to evaluate other factors which may have lead to such a high prophylactic failure rate, drug supplies were checked for stability and correct packaging, and blood samples were checked for the presence of tafenoquine to determine that the drug had been systemically absorbed. These investigations showed no evidence that any of these factors had contributed to the high failure rate reported. The conclusion must be that the high apparent failure rate was due to local study slide readers at USAMRU-K reading false positives blood smears.

For future studies in the tafenoquine program a number of measures could be usefully applied in order to prevent a similar experience. Of paramount importance is ensuring adequate training of slide-readers, since this must be presumed to be contributory in this instance. The site did have a slide-reading SOP and Quality Control (QC) procedure in place but this did not appear to function adequately. A thorough training procedure prior to the initiation of future studies would be considered useful, with an examination process to ensure that adequate quality of slide-readers are selected. Following this, an ongoing QC

procedure should ensure slide-reading quality is maintained throughout the study. In addition, although there is no direct evidence that the blinding of the two readers to one another's' results was at fault in the current study, this is another area where attention at future study sites would only be of benefit. Other areas worthy of consideration would be slide preparation, staining and storage techniques, to ensure standardised methodologies are used in order to optimise slide quality and make the job of slide reading more reliable and reproducible.

Sensitivity and specificity of slide reading were calculated for the USAMRU-K site assuming the NAMRU-2 diagnoses to be true – these were 86% and 70% respectively. The overall positive concordance rate (the proportion of slides read as positive by USAMRU-K which were also identified as positive by NAMRU-2) was only 12.4%. It is difficult to ascertain what is regarded as acceptable sensitivity and specificity for microscopy diagnoses in malaria prophylaxis studies, since microscopy is regarded as the 'gold standard' and calculation of sensitivity and specificity requires a reference diagnosis to be presumed to be correct. Ohrt et al [21], however, has modelled the effect of false positive diagnoses on protective efficacies in malaria prophylaxis studies and showed protective efficacy to be highly impacted by small over-estimations of false-positive smears - 0.5% false-positive smears (99.5% specificity) can lead to an underestimate of protective efficacy by 7-15%, dependent on malaria attack rate and duration of trial. This is clearly an important aspect to control in future studies if reliable estimates of the prophylactic efficacy of tafenoquine are to be demonstrated.

As a result of these observations in slide-reading, when considering the primary efficacy variable at the end of the study, there was a high failure rate in all treatment groups (88-94%). This translated into low protective efficacies compared with placebo at 6.1% for the tafenoquine group and 1.1% for the mefloquine group. However, due to failure of the fundamental diagnosis process, this cannot be considered to be a true reflection of the chemoprophylactic efficacy of tafenoquine in this study setting. Due to the failure of the slide-reading process, further discussion of the primary and secondary efficacy variables will not be conducted here. Demonstration of the chemoprophylactic efficacy of tafenoquine in a Phase III setting will have to be shown in further clinical studies.

In spite of these issues with slide-reading, and the resultant early loss of study subjects from the trial, it is still possible to draw conclusions about the safety and tolerability of tafenoquine from this study although overall exposure times are reduced compared to the originally planned 6 month duration of the study.

Overall, tafenoquine was well tolerated in this study setting. The incidence of adverse experiences during the prophylaxis treatment phase in the tafenoquine group (94.2%) was similar to the incidence in the placebo (91.1%) and mefloquine (96.0%) groups. Most adverse experiences were of mild or moderate intensity. The most commonly occurring event in all treatment groups was headache, and a majority of the most frequently reported events involved pain or infections. Only headache and dizziness appeared to be reported at somewhat higher incidences in the tafenoquine group compared to placebo (headache at 43.3% vs. 36.6% and dizziness at 11.5% vs. 6.9% respectively), these being at comparable rates to the mefloquine group (headache at 48.5% and dizziness at 13.9%).

Eleven subjects (3.6%) had serious adverse experiences during the prophylaxis treatment phase and 5 subjects (1.6%) had serious adverse experiences in the follow-up period. Only two subjects had serious adverse experiences reported with a suspected relationship to study treatment: a subject in the tafenoquine group with investigator-diagnosed haemolytic anaemia and a subject in the mefloquine group with anxiety. The haemolytic anaemia is unusual since, although this is known to be class effect of 8-aminoquinolines, this subject had tested normal for G6PD status on two separate occasions as part of the screening process, albeit using the same technique on both occasions. It would have been beneficial to have assessed G6PD status using more than one technique and to have determined the indivdual's genotype status with respect to G6PD in an effort to have understood this case more completely. Following withdrawal from the study, the subject recovered on treatment with multivitamins and ferrous sulphate without need for transfusion.

There were no deaths reported. Thirteen subjects had adverse experiences recorded as leading to withdrawal. Most of these involved malaria or associated events and the reason for withdrawal from the study was ‘insufficient therapeutic effect’.

Monitoring of laboratory results showed some evidence of mild decreases in haemoglobin, haematocrit and platelet values on tafenoquine treatment compared to placebo or mefloquine treated subjects. Additionally, there was some suggestion of mild and apparently transient decreases in creatinine on starting tafenoquine medication which then tended towards baseline after 4 weeks of therapy. It should be noted, however, that apart from the individual serious adverse experience of haemolytic anaemia noted above, none of these observations were considered clinically relevant.

Vitals signs assessments showed no results of clinical significance and no marked differences between the treatment groups.

10 Conclusions

- A failure of the slide-reading process in this study lead to an inability to assess the primary objective ie. determining the chemoprophylactic efficacy of tafenoquine against *P.falciparum* infection. Measures will have to be taken to ensure the quality of slide-reading in future Phase III studies.
- Tafenoquine was well tolerated with the most frequently reported adverse experience being mild or moderate headache.
- There was some evidence of small decreases in laboratory measures of haemoglobin, haematocrit and platelets on tafenoquine therapy, and small and transient increases in creatinine. None of these reached clinical relevance, apart from a single case of investigator-diagnosed haemolytic anaemia reported as a serious adverse experience; this in spite of the subject testing normal for G6PD status.

11 References

1. BRUCE-CHWATT LJ. 1987. *Ann Rev Public Health 8,* **75,** 110.

2. BRUCE-CHWATT LJ. 1971. Malaria and its prevention in military campaigns. *Z Tropenmed Parasitol,* **22,** 370-390.

3. BRUCE-CHWATT LJ 1991. Chemotherapy of malaria. *WHO Monograph Series,* **27.** Geneva: WHO.

4. COATNEY GR, COLLINS WE, WARREN MCW, CONTACOS PG. 1979. *The Primate Malarias,* USDHEW **273-274.** Government Printing Office.

5. STEFFEN R, FUCHS E, SCHILDKNECHT J ET AL. 1993. Mefloquine compared with other malaria chemoprophylactic regimens in tourists visiting east Africa. *Lancet,* **341,** 1299-1303.

6. FONTANET AL, JOHNSTON BD, WALDER AM ET AL. 1993. *Bull WHO,* **71,** 377-383.

7. SUKWA, TY,MULENGA M.CHISKA ET AL. 1999. A randomised, double blind,placebo controlled field trial to determine the efficacy and Safety of Malarone (atovaquone/proguanil) for the prophylaxis of malaria in Zambia. *Am J Trop Med Hyg,* **60,** 521-525.

8. SHANKS D, GORDON DM, KLOTZ FW ET AL. 1998. Efficacy and safety of atovaquone/proguanil for suppressive prophylaxis against *Plasmodium falciparum* malaria. *Clin Infect Dis,* **27,** 494-499.

9. LELL B, LUCKNER D, NDJAVE M,ET AL. 1998. Randomised placebo controlled study of atovaquone plus proguanil for malaria prophylaxis in children. *Lancet,* **351**, 709-713.

10. VENNERSTROM, JL, NUZUM EO, MILLER RE, ET AL. 1999. 8-Aminoquinolines active against blood stage *Plasmodium falciparum* in vitro inhibit hematin polymerisation. *Anti-Microb Agents Chemotherapy,* **43,** 598-602.

11. FISK TL, MILLET P COLLINS WE, NGUYEN-DINH P. 1989. In vitro activity of antimalarial compounds on the exoerthrocytic stages of Plasmodium cynomgi and P.knowlesi. *Am J Trop Med Hyg,* **40,** 235-239.

12. BRUECKNER RP, LASSETER KC, LIN ET, SCHUSTER BG,. 1998. First-time in humans safety and pharmacokinetics of WR 238605, a new antimalarial. *Am J Trop Med Hyg,* **58,** 645-649.

13. SHANKS DG, OLOO AJ, ALEMAN GM, OHRT C ET AL. 2001. A new primaquine analogue, tafenoquine (WR238605) for prophylaxis against Plasmodium falciparum. *Clinical Infectious Diseases,* **33,** 1968-1974.

14. SB-252263/RSD-101KVV/1. A randomized, double-blind, placebo-controlled evaluation of increasing doses of weekly tafenoquine for chemosuppression of Plasmodium falciparum in semi-immune adults living in the Kassena-Nankana district of Northern Ghana. Pickford P, Moate R, Whately-Smith C, McIntyre J. 2003.

15. BOUDREAU E, SCHUSTER B, SANCHEZ J ET AL. 1993. Tolerability of prophylactic Lariam regimens. *Trop Med Parasitol,* **44,** 257-265.

16. BOUDREAU E, personal communication.

17. OHRT C, RICHIE TL, WIDJAJA H ET AL. 1997. Mefloquine compared to doxycycline for the prophylaxis of malaria in Indonesian soldiers. *Ann Int Med,* **126,** 963-972.

18. FARRINGTON CP, MANNING G. 1990. Test statistics and sample size formulae for comparative binomial trials with null hypothesis of non-zero risk difference or non-unity relative risk. *Statistics in Medicine,* **9,** 1447-1454.

19. KOOPMAN PAR. 1984. Confidence intervals for the ratio of two binomial proportions. *Biometrics,* **40,** 513-517.

20. O'BRIEN PC, FLEMING TR. 1979. A multiple testing procedure for clinical trials. *Biometrics,* **35,** 549-556.

21. OHRT C, PURNOMO, AWALLUDIN SUTAMIHARDJA M ET AL. 2002. Impact of Microscopy Error on Estimates of Protective Efficacy in Malaria-Prevention Trials. *J Infect Dis,* **186,** 540-546.

12 Source Tables: Study Population

Table No.	Title	No. of pages
Table 11.1	Subject disposition: all screened subjects	1
Table 11.2	Reasons for exclusion from the randomised study: all screened subjects	1
Table 11.3.1a	Number of subjects at each visit (on-dose): intent-to-treat population	2
Table 11.3.1b	Number of subjects at each visit (on-dose): per protocol population	2
Table 11.3.2a	Number of subjects at each visit (post-dose): intent-to-treat population	2
Table 11.3.2b	Number of subjects at each visit (post-dose): per protocol population	2
Table 11.4.1	Subject withdrawals: intent-to-treat population	1
Table 11.4.2	Cumulative percentage of subject withdrawals over time: intent-to-treat population	2
Table 11.5.1	Subjects excluded from the intent-to-treat population: all randomised subjects	1
Table 11.5.2	Subjects excluded from the per protocol population: intent-to-treat population	1
Table 11.6.1a	Demographic characteristics – frequency distribution for gender, race and age: intent-to-treat population	1
Table 11.6.1b	Demographic characteristics – frequency distribution for gender, race and age: per protocol population	1
Table 11.6.2a	Demographic characteristics – summary statistics for age and weight: intent-to-treat population	1
Table 11.6.2b	Demographic characteristics – summary statistics for age and weight: per protocol population	1
Table 11.7.1.1	Significant past medical and surgical history by body system and preferred term: intent-to-treat population	2
Table 11.7.1.2	Significant ongoing medical and surgical history by body system and preferred term: intent-to-treat population	3
Table 11.7.2.1	Significant past medical and surgical history by preferred term ordered by decreasing frequency: intent-to-treat population	3
Table 11.7.2.2	Significant ongoing medical and surgical history by	5

Table No.	Title	No. of pages
	preferred term ordered by decreasing frequency: intent-to-treat population	
Table 11.8a	Number (%) of subjects with a history of malaria: intent-to-treat population	1
Table 11.8b	Number (%) of subjects with a history of malaria: per protocol population	1
Table 11.9.1a	Summary statistics for malaria history: intent-to-treat population	1
Table 11.9.1b	Summary statistics for malaria history: per protocol population	1
Table 11.9.2	Number (%) of subjects with malaria parasites at screening prior to halofantrine by species: all randomised subjects	1
Table 11.9.3a	Summary statistics for parasite count at screening (expressed as per UL of blood): intent-to-treat population	1
Table 11.9.3b	Summary statistics for parasite count at screening (expressed as per UL of blood): per protocol population	1
Table 11.9.4	Number (%) of subjects with malaria parasites following treatment with halofantrine by species: all randomised subjects	1
Table 11.10.1	Baseline signs and symptoms by body system and preferred term: safety population	2
Table 11.10.2	Baseline signs and symptoms by preferred term ordered by decreasing frequency: safety population	3
Table 11.10.3	Baseline signs and symptoms leading to withdrawal by body system and preferred term: safety population	1
Table 11.11.1.1	Prior medications by ATC classification and generic term: intent-to-treat population	3
Table 11.11.1.2	Prior medications by generic term ordered by decreasing frequency: intent-to-treat population	4
Table 11.11.2.1	Concomitant medications by ATC classification and generic term: intent-to-treat population	6
Table 11.11.2.2	Concomitant medications by generic term ordered by decreasing frequency: intent-to-treat population	7
Table 11.11.3.1	Follow-up medications by ATC classification and generic term: intent-to-treat population	6
Table 11.11.3.2	Follow-up medications by generic term ordered by decreasing frequency: intent-to-treat population	7
Table 11.12	Study medication compliance: intent-to-treat population	1

13 Source Tables: Efficacy Results

Table No.	Title	No. of pages
Table 12.1a	Prophylactic outcome and protective efficacy based on single positive smear (all species) during the prophylaxis treatment period: intent-to-treat population	1
Table 12.1b	Prophylactic outcome and protective efficacy based on single positive smear (all species) during the prophylaxis treatment period: per protocol population	1
Table 12.2	Incidence density and protective efficacy based on single positive smear (all species) during the prophylaxis treatment period: intent-to-treat population	1
Table 12.3	Worst case prophylactic outcome and protective efficacy based on single positive smear (all species) during the prophylaxis treatment period: intent-to-treat population	1
Table 12.4a	Prophylactic outcome and protective efficacy based on two consecutive positive smears (all species) during the prophylaxis treatment period: intent-to-treat population	1
Table 12.4b	Prophylactic outcome and protective efficacy based on two consecutive positive smears (all species) during the prophylaxis treatment period: per protocol population	1
Table 12.5a	Prophylactic outcome and protective efficacy based on single positive smear (*P. falciparum* only) during the prophylaxis treatment period: intent-to-treat population	1
Table 12.5b	Prophylactic outcome and protective efficacy based on single positive smear (*P. falciparum* only) during the prophylaxis treatment period: per protocol population	1
Table 12.6a	Summary statistics for time to single positive smear: intent-to-treat population	1
Table 12.6b	Summary statistics for time to single positive smear: per protocol population	1
Table 12.7a	Summary statistics for time to two consecutive positive smears: intent-to-treat population	1
Table 12.7b	Summary statistics for time to two consecutive	1

Table No.	Title	No. of pages
	positive smears: per protocol population	
Table 12.8a	Number (%) of subjects with a single positive smear by species during the prophylaxis treatment period: intent-to-treat population	1
Table 12.8b	Number (%) of subjects with a single positive smear by species during the prophylaxis treatment period: (per protocol population	1
Table 12.9a	Summary statistics for parasite count at single positive smear (expressed as per UL blood): intent-to-treat population	1
Table 12.9b	Summary statistics for parasite count at single positive smear (expressed as per UL blood): per protocol population	1
Table 12.10a	Malaria status of all subjects at the end of the prophylaxis treatment period: intent-to-treat population	1
Table 12.10b	Malaria status of all subjects at the end of the prophylaxis treatment period: per protocol population	1
Table 12.11a	Malaria status of subjects not treated for malaria after single positive smear at the end of the prophylaxis treatment period: intent-to-treat population	1
Table 12.11b	Malaria status of subjects not treated for malaria after single positive smear at the end of the prophylaxis treatment period: per protocol population	1
Table 12.12a	Comparison of the original USAMRU-K reading to the NAMRU-2 result (treatment=all): all re-read slides	1
Table 12.12b	Comparison of the original USAMRU-K reading to the NAMRU-2 result (treatment=placebo): all re-read slides	1
Table 12.12c	Comparison of the original USAMRU-K reading to the NAMRU-2 result (treatment=tafenoquine): all re-read slides	1
Table 12.12d	Comparison of the original USAMRU-K reading to the NAMRU-2 result (treatment=mefloquine): all re-read slides	1
Table 12.13	Sensitivity and specificity of USAMRU-K results compared to NAMRU-2 (treatment=all): all re-read slides	1

14 Source Tables: Safety Results

Table No.	Title	No. of pages
Table 13.1.1	Extent of exposure: number of weeks on prophylactic treatment: safety population	1
Table 13.1.2	Extent of exposure: summary statistics for number of weeks on prophylactic treatment: safety population)	1
Table 13.2	Summary statistics for duration of continuing baseline signs and symptoms at start of prophylactic medication: safety population	1
Table 13.3.1.1	Number (%) of subjects reporting adverse experiences by body system and preferred term: prophylaxis treatment phase: safety population	5
Table 13.3.1.2	Number (%) of subjects reporting adverse experiences by body system and preferred term: safety follow-up phase: safety population	3
Table 13.3.2.1	Number (%) of subjects reporting adverse experiences by preferred term ordered by decreasing frequency: prophylaxis treatment phase: safety population	6
Table 13.3.2.2	Number (%) of subjects reporting adverse experiences by preferred term ordered by decreasing frequency: safety follow-up phase: safety population	4
Table 13.3.3.1	Number (%) of subjects reporting adverse experiences (CNS/psychiatric only) by preferred term ordered by decreasing frequency: prophylaxis treatment phase: safety population	1
Table 13.3.3.2	Number (%) of subjects reporting adverse experiences (CNS/psychiatric only) by preferred term ordered by decreasing frequency: safety follow-up phase: safety population	1
Table 13.3.4.1	Number (%) of subjects reporting adverse experiences by severity, body system and preferred term: prophylaxis treatment phase: safety population	9
Table 13.3.4.2	Number (%) of subjects reporting adverse experiences by severity, body system and preferred term: safety follow-up phase: safety population	5
Table 13.3.5.1	Number (%) of subjects reporting suspected or probably related adverse experiences by body system and preferred term: prophylaxis treatment phase: safety population	2

Table No.	Title	No. of pages
Table 13.3.5.2	Number (%) of subjects reporting suspected or probably related adverse experiences by body system and preferred term: safety follow-up phase: safety population	1
Table 13.3.6.1	Number (%) of subjects reporting adverse experiences leading to withdrawal by body system and preferred term: prophylaxis treatment phase: safety population	1
Table 13.3.6.2	Number (%) of subjects reporting adverse experiences leading to withdrawal by body system and preferred term: safety follow-up phase: safety population	1
Table 13.3.7.1	Number (%) of subjects reporting serious adverse experiences by body system and preferred term: prior to prophylactic study medication phase: safety population	1
Table 13.3.7.2	Number (%) of subjects reporting serious adverse experiences by body system and preferred term: prophylaxis treatment phase: safety population	1
Table 13.3.7.3	Number (%) of subjects reporting serious adverse experiences by body system and preferred term: safety follow-up phase: safety population	1
Table 13.3.7.4	Serious adverse experiences: individual subject narrative	24
Table 13.3.8	Number (%) of subjects reporting adverse experiences by time of first occurrence: safety population	21
Table 13.4.1.1	Summary statistics for baseline vital signs and change in vital signs from baseline by visit: prophylaxis treatment phase: safety population	3
Table 13.4.1.2	Summary statistics for baseline vital signs and change in vital signs from baseline by visit: safety follow-up phase: safety population	3
Table 13.4.2.1	Number (%) of subjects with vital signs of potential clinical concern by visit: prophylaxis treatment phase: safety population	3
Table 13.4.2.2	Number (%) of subjects with vital signs of potential clinical concern by visit: safety follow-up phase: safety population	3
Table 13.5.1.1	Summary statistics for baseline laboratory parameters and change in laboratory parameters from baseline by visit: prophylaxis treatment phase: safety population	6

Table No.	Title	No. of pages
Table 13.5.1.2	Summary statistics for baseline laboratory parameters and change in laboratory parameters from baseline by visit: safety follow-up phase: safety population	6
Table 13.5.2.1	Number (%) of subjects with laboratory parameters outside the F2 range by visit: prophylaxis treatment phase: safety population	9
Table 13.5.2.2	Number (%) of subjects with laboratory parameters outside the F2 range by visit: safety follow-up phase: safety population	9
Table 13.5.3.1	Number (%) of subjects with laboratory parameters outside the F3 range by visit: prophylaxis treatment phase: safety population	9
Table 13.5.3.2	Number (%) of subjects with laboratory parameters outside the F3 range by visit: safety follow-up phase: safety population	9
Table 13.5.4.1	Number (%) of subjects with laboratory parameters outside the F2F3 range by visit: prophylaxis treatment phase: safety population	9
Table 13.5.4.2	Number (%) of subjects with laboratory parameters outside the F2F3 range by visit: safety follow-up phase: safety population	9

15 Source Figures

Figure No.	Title	No. of pages
Figure 1	Survival curve showing time to first positive smear: intent-to-treat population	1
Figure 2	Survival curve showing time to first positive smear: per protocol population	1
Figure 3	Survival curve showing time to two consecutive positive smears: intent-to-treat population	1
Figure 4	Survival curve showing time to two consecutive positive smears: per protocol population	1

Appendices

Appendix A: Protocol, Sample Blank CRF, Investigator CV, Randomisation Code, Certificates of Analysis

Item
Protocol incorporating amendments
Sample blank CRF
Investigator CV
Randomisation code
Certificates of analysis
CTS-P Report (Failure rate investigation)
DMPK (interim) Report (Failure rate investigation)
Drug Analysis Report (Failure rate investigation)
Australian Malaria Institute (AMI) Slide Reads
IDMC Charter
NAMRU-2 Microscopy training SOP
Re-Read Protocol for NAMRU and AMI
WHO codelist for CNS Adverse Experiences
NAMRU-2 Report

Appendix B: Subject Data Listings of Demographic and Baseline Characteristics

Listing No.	Title	No. of pages
Listing B.01	Treatment assignment	7
Listing B.02	Visit and dosing dates	175
Listing B.03	Subject withdrawals	9
Listing B.04	All non-randomised subjects: reasons for exclusion from the study	5
Listing B.05	All randomised subjects: reasons for exclusion from the intent-to-treat population	3
Listing B.06	All randomised subjects: reasons for exclusion from the per protocol population	10
Listing B.07	Inclusion and exclusion criteria violations	10
Listing B.08	Demographic characteristics	15
Listing B.09	Pregnancy test results	27
Listing B.10	G6PD test results	9
Listing B.11	Significant medical and surgical history	46
Listing B.12	History of malaria	15
Listing B.13	Baseline signs and symptoms	33
Listing B.14.1	Prior medications	57
Listing B.14.2	Concomitant medications	161
Listing B.14.3	Follow-up medications	136
Listing B.15	Halofantrine compliance	36
Listing B.16	Prophylactic medication compliance	9

Appendix C: Subject Data Listings of Efficacy

Listing No.	Title	No. of pages
Listing C.01	Results of blood smears by visit	321
Listing C.02	Prophylactic outcome (all endpoints)	42
Listing C.03	Time to parasitaemia	31
Listing C.04	Results of NAMRU-2 slide reading	52
Listing C.05	Consensus results of USAMRU-K and NAMRU-2 slide reading	19

Appendix D: Subject Data Listings of Adverse Experiences

**Listing No.**	**Title**	**No. of pages**
Listing D.01	Non-serious adverse experiences	273
Listing D.02	Serious adverse experiences	4
Listing D.03	Deaths	1

Appendix E: Subject Data Listings of Vital Signs

Listing No.	Title	No. of pages
Listing E.01	Vital signs	151

Appendix F: Subject Data Listings of Laboratory Tests

Listing No.	Title	No. of pages
Listing F.01	Laboratory values by subject	361
Listing F.02	Laboratory values by parameter	297

Appendix G: CRF Copies for Deaths, Serious Adverse Experiences and Withdrawals due to Adverse Experiences

Subjects who had events in more than one category are listed in each of the respective categories and are denoted by *.

Subjects With Serious (Non-Fatal) Adverse Experiences

Placebo

030.001.00012
030.001.00087
030.001.00101
030.001.00160
030.001.00256

Tafenoquine 200 mg

030.001.00043*
030.001.00109
030.001.00155
030.001.00158
030.001.00173*
030.001.00190
030.001.00233
030.001.00261*
030.001.00267
030.001.00282

Mefloquine 250 mg

030.001.00003
030.001.00035*
030.001.00036
030.001.00105

Subjects Who Were Withdrawn Due to Adverse Experiences

Tafenoquine 200 mg

030.001.00043*
030.001.00173*
030.001.00261*

Mefloquine 250 mg

030.001.00035*
030.001.00123

DEPARTMENT OF THE ARMY
US ARMY MEDICAL RESEARCH AND MATERIEL COMMAND
504 SCOTT STREET
FORT DETRICK, MARYLAND 21702-5012

REPLY TO
ATTENTION OF:

March 31, 2000

Office of the Deputy Chief of Staff
for Regulatory Compliance and Quality

SUBJECT: IND 38,503 - WR 238,605 Succinate (Serial No. 086)

Food and Drug Administration
Office of Drug Evaluation IV
Division of Anti-Infective Drug Products
9200 Corporate Boulevard, HFD-520
Rockville, Maryland 20850

Dear Sir or Madam:

Enclosed in triplicate is a new clinical protocol entitled, "A Randomized, Double-Blind, Placebo Controlled Evaluation of Weekly Tafenoquine (WR 23865/SB252263) Compared to Mefloquine for Chemosuppression of *Plasmodium falciparum* in Western Kenya" (HSRRB Log No. A-9467) that will be conducted by LTC Jose Stoute, MC, WRAIR/USAMRU-K, under the subject IND. This protocol was reviewed by the Human Subjects Research Review Board on March 8, 2000 and was approved for implementation on March 30, 2000.

Please contact Ms. Kathie Mantine at 301-619-2809 (alternate 7550), facsimile 301-619-7803, or electronic mail kathie.mantine@det.amedd.army.mil for regulatory questions concerning this submission. The U.S. Army Medical Materiel Development Activity point of contact for technical questions is LTC William Prescott at 301-619-7872.

Sincerely,

Julie K. Zadinsky

Julie K. Zadinsky
Colonel, Army Nurse Corps
Deputy Chief of Staff for
Regulatory Compliance and Quality

Enclosure

Copies Furnished (wo/enclosure):

U.S. Army Medical Research and Materiel Command, ATTN: MCMR-SGS
U.S. Army Medical Materiel Development Activity, ATTN: MCMR-UMP

DEPARTMENT OF HEALTH AND HUMAN SERVICES
PUBLIC HEALTH SERVICE
FOOD AND DRUG ADMINISTRATION

INVESTIGATIONAL NEW DRUG APPLICATION (IND)

(TITLE 21, CODE OF FEDERAL REGULATIONS (CFR) PART 312)

Form Approved: OMB No. 0910-0014.
Expiration Date: December 31, 1999
See OMB Statement on Reverse.

NOTE: No drug may be shipped or clinical investigation begun until an IND for that investigation is in effect (21 CFR 312.40).

1. NAME OF SPONSOR
Office of The Surgeon General, Department of the Army

2. DATE OF SUBMISSION
31 MAR 2000

3. ADDRESS *(Number, Street, City, State and Zip Code)*
Commanding General, U.S. Army Medical Research and Materiel Command
ATTN: MCMR-RCQ-RA
504 Scott Street
Fort Detrick, MD 21702-5012

4. TELEPHONE NUMBER *(Include Area Code)*
301-619-2165

5. NAME(S) OF DRUG *(Include all available names: Trade, Generic, Chemical, Code)*
WR 238,605 Succinate

6. IND NUMBER *(If previously assigned)*
IND 38,503

7. INDICATION(S) *(Covered by this submission)*
Antimalarial

8. PHASE(S) OF CLINICAL INVESTIGATION TO BE CONDUCTED:
☐ PHASE 1 ☐ PHASE 2 ☒ PHASE 3 ☐ OTHER ______ *(Specify)*

9. LIST NUMBERS OF ALL INVESTIGATIONAL NEW DRUG APPLICATIONS (21 CFR Part 312), NEW DRUG OR ANTIBIOTIC APPLICATIONS *(21 CFR Part 314)*, DRUG MASTER FILES *(21 CFR Part 314.420)*, AND PRODUCT LICENSE APPLICATIONS (21 CFR Part 601) REFERRED TO IN THIS APPLICATION.

10. ***IND submission should be consecutively numbered. The initial IND should be numbered "Serial number: 000." The next submission (e.g., amendment, report, or correspondence) should be numbered "Serial Number: 001." Subsequent submissions should be numbered consecutively in the order in which they are submitted.***

SERIAL NUMBER
086

11. THIS SUBMISSION CONTAINS THE FOLLOWING: *(Check all that apply)*
☐ INITIAL INVESTIGATIONAL NEW DRUG APPLICATION (IND)
☐ RESPONSE TO CLINICAL HOLD

PROTOCOL AMENDMENT(S):
☒ NEW PROTOCOL
☐ CHANGE IN PROTOCOL
☐ NEW INVESTIGATOR

INFORMATION AMENDMENT(S):
☐ CHEMISTRY/MICROBIOLOGY
☐ PHARMACOLOGY/TOXICOLOGY
☐ CLINICAL

IND SAFETY REPORT(S):
☐ INITIAL WRITTEN REPORT
☐ FOLLOW-UP TO A WRITTEN REPORT

☐ RESPONSE TO FDA REQUEST FOR INFORMATION
☐ ANNUAL REPORT
☐ GENERAL CORRESPONDENCE
☐ REQUEST FOR REINSTATEMENT OF IND THAT IS WITHDRAWN, INACTIVATED, TERMINATED OR DISCONTINUED
☐ OTHER ______ *(Specify)*

CHECK ONLY IF APPLICABLE

JUSTIFICATION STATEMENT MUST BE SUBMITTED WITH APPLICATION FOR ANY CHECKED BELOW. REFER TO THE CITED CFR SECTION FOR FURTHER INFORMATION.

☐ **TREATMENT IND 21 CFR 312.35(b)** ☐ **TREATMENT PROTOCOL 21 CFR 312.35(a)** ☐ **CHARGE REQUEST/NOTIFICATION 21 CFR312.7(d)**

FOR FDA USE ONLY

CDR/DBIND/DGD RECEIPT STAMP	DDR RECEIPT STAMP	DIVISION ASSIGNMENT:
		IND NUMBER ASSIGNED:

Created by: PSC Media Arts Branch (301) 443-2454 EF

12. **CONTENTS OF APPLICATION**

This application contains the following items: *(Check all that apply)*

- ☐ 1. Form FDA 1571 *[21 CFR 312.23(a)(1)]*
- ☐ 2. Table of Contents *[21 CFR 312.23(a)(2)]*
- ☐ 3. Introductory statement *[21 CFR 312.23(a)(3)]*
- ☐ 4. General Investigational plan *[21 CFR 312.23(a)(3)]*
- ☐ 5. Investigator's brochure *[21 CFR 312.23(a)(5)]*
- ☐ 6. Protocol(s) *[21 CFR 312.23(a)(6)]*
  - ☐ a. Study protocol(s) *[21 CFR 312.23(a)(6)]*
  - ☐ b. Investigator data *[21 CFR 312.23(a)(6)(iii)(b)]* or completed Form(s) FDA 1572
  - ☐ c. Facilities data *[21 CFR 312.23(a)(6)(iii)(b)]* or completed Form(s) FDA 1572
  - ☐ d. Institutional Review Board data *[21 CFR 312.23(a)(6)(iii)(b)]* or completed Form(s) FDA 1572
- ☐ 7. Chemistry, manufacturing, and control data *[21 CFR 312.23(a)(7)]*
  - ☐ Environmental assessment or claim for exclusion *[21 CFR 312.23(a)(7)(iv)(e)]*
- ☐ 8. Pharmacology and toxicology data *[21 CFR 312.23(a)(8)]*
- ☐ 9. Previous human experience *[21 CFR 312.23(a)(9)]*
- ☐ 10. Additional information *[21 CFR 312.23(a)(10)]*

13. IS ANY PART OF THE CLINICAL STUDY TO BE CONDUCTED BY A CONTRACT RESEARCH ORGANIZATION? ☐ YES ☐ NO

IF YES, WILL ANY SPONSOR OBLIGATIONS BE TRANSFERRED TO THE CONTRACT RESEARCH ORGANIZATION? ☐ YES ☐ NO

IF YES, ATTACH A STATEMENT CONTAINING THE NAME AND ADDRESS OF THE CONTRACT RESEARCH ORGANIZATION, IDENTIFICATION OF THE CLINICAL STUDY, AND A LISTING OF THE OBLIGATIONS TRANSFERRED.

14. NAME AND TITLE OF THE PERSON RESPONSIBLE FOR MONITORING THE CONDUCT AND PROGRESS OF THE CLINICAL INVESTIGATIONS

LTC William R. Prescott
Product Manager
USAMMDA
Fort Detrick, MD 21702-5009

15. NAME(S) AND TITLE(S) OF THE PERSON(S) RESPONSIBLE FOR REVIEW AND EVALUATION OF INFORMATION RELEVANT TO THE SAFETY OF THE DRUG

LTC Jose Stoute, MC
U.S. Army Medical Research Unit, Kenya
Unit 64109 Box 401
APO AE 09831-4109

I agree not to begin clinical investigations until 30 days after FDA's receipt of the IND unless I receive earlier notification by FDA that the studies may begin. I also agree not to begin or continue clinical investigations covered by the IND if those studies are placed on clinical hold. I agree that an Institutional Review Board (IRB) that complies with the requirements set fourth in 21 CFR Part 56 will be responsible for initial and continuing review and approval of each of the studies in the proposed clinical investigation. I agree to conduct the investigation in accordance with all other applicable regulatory requirements.

16. NAME OF SPONSOR OR SPONSOR'S AUTHORIZED REPRESENTATIVE	17. SIGNATURE OF SPONSOR OR SPONSOR'S AUTHORIZED REPRESENTATIVE	
Ronald E. Clawson, Ph.D., Project Manager<br>Pharmaceutical Systems Division, USAMMDA<br>622 Neiman Street, Fort Detrick, MD 21702-5009	[signature]	
18. ADDRESS *(Number, Street, City, State and Zip Code)*	19. TELEPHONE NUMBER *(Include Area Code)*	20. DATE
Commanding General, U.S. Army Medical Research and Materiel Command<br>ATTN: MCMR-RCQ-RA, 504 Scott Street<br>Fort Detrick, MD 21702-5012	301-619-2051	March 24, 2000

(WARNING: A willfully false statement is a criminal offense. U.S.C. Title 18, Sec. 1001.)

Public reporting burden for this collection of information is estimated to average 100 hours per response, including the time for reviewing instructions, searching existing data sources, gathering and maintaining the data needed, and completing reviewing the collection of information. Send comments regarding this burden estimate or any other aspect of this collection of information, including suggestions for reducing this burden to:

Food and Drug Administration
CBER (HFM-99)
1401 Rockville Pike
Rockville, MD 20852-1448

Food and Drug Administration
CDER (HFD-94)
5516 Nicholson Lane
Kensington, MD 20895

"An agency may not conduct or sponsor, and a person is not required to respond to, a collection of information unless it displays a currently valid OMB control number."

Please **DO NOT RETURN** this application to this address.

A Randomized, Double Blind, Placebo Controlled Evaluation of Weekly Tafenoquine (WR 238605/SB252263) Compared to Mefloquine for Chemosuppression of *Plasmodium falciparum* in Western Kenya

Protocol Number SB 252263/030

INSTITUTIONS/INVESTIGATORS

Principal Investigators

Dr Jose Stoute MD, LTC, MC, USAMRU-K

Investigators

US Army Medical Research Unit-Kenya, (USAMRU-K)
Dr. G. Dennis Shanks M.D., MPH, COL, MC Investigator, USAMRU-K
Dr. Carl Mason, M.D., LTC, MC, Investigator, USAMRU-K

Jon Davis, CPT, MS, Investigator

Walter Reed Army Institute of Research, Washington, DC
Doug Tang, Ph.D., Biostatistician

SmithKline Beecham UK
Dr. John Horton M.D.,
Matthew Truman, MSc.
Lorraine Martin, BSc.

US Army Medical Materiel Development Activity (USAMMDA)
William Prescott Ph.D., LTC, MS, Product Manager Antimalarial Drugs

Medical Monitor
Dr. Ambrose Misore

FINAL PROTOCOL
Amended 15 March 2000

1. SUMMARY **5**

2. INTRODUCTION/BACKGROUND **5**

A. THE MALARIA PROBLEM 5
B. CURRENT DRUGS FOR MALARIA PROPHYLAXIS 6
C. TEST ARTICLE: TAFENOQUINE 7
D. TEST ARTICLE: MEFLOQUINE 11
E. PRE-PROPHYLAXIS TREATMENT: HALOFANTRINE 13

3. JUSTIFICATION FOR INVESTIGATION **13**

4. GOALS AND OBJECTIVES **14**

A. PRIMARY OBJECTIVES 14
B. SECONDARY OBJECTIVES: 14

5. DESIGN AND METHODOLOGY **14**

A. STUDY SITE (GEOGRAPHICAL) 14
B. STUDY POPULATION 14
1. Malaria in western Kenya *14*
2. Recruitment Procedure *15*

6. INFORMED CONSENT **16**

6 A GENERAL CONSIDERATIONS 16
6 B THE INFORMED CONSENT PROCESS 17

7. INCLUSION CRITERIA **17**

8. EXCLUSION CRITERIA **17**

9. STUDY PROCEDURES **18**

A. GENERAL INSTRUCTIONS 18
B. SCHEDULE OF ASSESSMENTS 19
1. Screening: Day –21 to -6 ..... *20*
2. Parasitemia Clearance treatment : Day –5 to –3 ..... *20*
3. Start of randomized double-blind study medication: Day 0 ..... *21*
4. Initial loading of prophylaxis medication : Day 0 to Day 2 ..... *21*
5. Weekly medication visits: Day 7 (Week 1) to Week 24 ..... *21*
6. Safety Follow-up Visit: Week 28 ..... *22*
7. Immunologic follow-up *22*

10. SUBJECT IDENTIFICATION AND RANDOMISATION: **22**

11. DRUGS AND DOSAGE **23**

A. GENERAL 23
B. ADMINISTRATION OF STUDY DRUG 23
C. PACKAGING AND LABELLING 24

12. DRUG ACCOUNTABILITY **25**

13. CONDUCT OF STUDY **25**

A. EVALUATION OF MALARIA 25

B. MANAGEMENT OF MALARIA 26

14. ADVERSE EXPERIENCE REPORTING 26

A. DEFINITION 26
B. RECORDING OF INTENSITY 27
C. FOLLOW-UP OF ADVERSE EXPERIENCES 28

15. SERIOUS ADVERSE EXPERIENCES 28

A. DEFINITION 28
B. REPORTING SERIOUS ADVERSE EXPERIENCES 29

16. CONCOMITANT MEDICATION 30

17. LABORATORY ASSESSMENTS 30

A. SAFETY SAMPLES 30
B. PHARMACOKINETIC SAMPLING 31
C. SAMPLES FOR MEASUREMENT OF PLASMA MALARIAL ANTIBODIES 32
D. SAMPLES FOR PERIPHERAL MONONUCLEAR BLOOD CELLS 32

18. PHARMACOLOGY AND IMMUNOLOGY EVALUATION. 32

19. PARASITE DRUG SENSITIVITY 33

20. WITHDRAWAL FROM THE STUDY 33

A. REASONS FOR WITHDRAWAL 33
B. SAFETY FOLLOW UP VISIT AFTER WITHDRAWAL 34

21. DATA MANAGEMENT AND STATISTICAL ANALYSIS 34

A. TARGET SAMPLE SIZE 34
B. METHOD OF RANDOMIZATION 35
C. BREAKING THE STUDY BLIND 35
D. PLANNED EFFICACY EVALUATIONS 35
E. METHODS OF ANALYSIS 36
F. PLANNED SAFETY EVALUATIONS 37
G. PHARMACOKINETIC ANALYSIS 38
H. ANALYSIS OF IMMUNOLOGIC PARAMETERS: 38

22. RECORDING AND DISSEMINATION OF DATA 38

A. RECORDING OF DATA 38
B. DISSEMINATION OF DATA 39

23. ADMINISTRATIVE MATTERS 39

24. TIME FRAME 39

25. ETHICAL CONSIDERATIONS 39

26. REFERENCES 40

27. BUDGET 40

28. RESOURCES AND COORDINATION 40

29. IND INFORMATION 40

30. MODIFICATION OF PROTOCOL 40
31. COMPENSATION TO VOLUNTEERS 41
32. STATEMENT REGARDING POTENTIAL CONFLICT OF FINANCIAL INTEREST 41
33. ROLE OF INVESTIGATORS 41
34. SPECIAL FUNDING IMPLICATIONS 41
35. SIGNATURE OF PRINCIPAL INVESTIGATOR: 41
APPENDIX A – DECLARATION OF HELSINKI 43
APPENDIX B - INFORMED CONSENT FORM 46
APPENDIX D- PHARMACOKINETIC SAMPLING 60
APPENDIX F- REFERENCES 63

1. SUMMARY

This study is conducted in two parts. A 24 week placebo controlled, randomized, double-blinded, comparative study of the efficacy of weekly tafenoquine (WR 238605), a new primaquine analog, compared to weekly mefloquine and weekly placebo as chemosuppression of *P. falciparum* malaria in Nyanza Province, western Kenya comprises the first part. Subsequent to this, subjects will continue to be followed up for a further 46 weeks in an immunologic follow-up study to determine whether or not treatment with malaria prophylaxis has had an effect on the semi-immune status of the population. The 2 parts of the study will be reported separately; up to week 28 by SmithKline Beecham and from Week 29 onwards by Dr Jose Stoute, United States Army Medical Research Unit-Kenya

Tafenoquine is being co-developed by the United States Army Medical Research and Materiel Command (USAMRMC) and SmithKline Beecham, and are referred to in this protocol as, 'the sponsor.'

Major objectives are to determine the chemosuppressive efficacy of tafenoquine compared to both mefloquine and placebo in preventing malaria parasitemia and to obtain safety and tolerance data for a period of six months. An additional objective is to study the immunologic changes resulting from extended chemosuppression of malaria in an endemic population.

The prophylaxis and safety follow-up phase i.e. up to week 28, and the immunologic follow-up phase (week 28 -70) will be the subjects of independent reports.

Up to 300 adults in the study area will be enrolled after screening for good general health and giving Informed Consent, and then those qualifying for drug administration will be randomized into three groups to receive tafenoquine, mefloquine or placebo. The three separate groups will receive

1) 200 mg tafenoquine daily for 3 days followed by 200 mg/week, or
2) 250 mg mefloquine daily for 3 days followed by 250 mg weekly, or
3) placebo daily for 3 days followed by weekly placebo.

All prophylaxis treatment will be blinded throughout the period of drug administration. Prior to starting prophylaxis, volunteers will receive a treatment course of halofantrine in order to eliminate all pre-existing parasitemia. Volunteers will receive closely monitored weekly study medication for 24 weeks. There will be additional follow up visits up to 12 months after drug administration ends primarily for the immunology endpoints. Subjects will be evaluated for falciparum malaria parasitemia by weekly blood smears and for adverse events by questioning and periodic blood tests. At the end of the study, the code will be broken and the groups evaluated with respect to cumulative malaria incidence during drug administration and side effects. The study will be conducted according to Good Clinical Practice Guidelines and the Declaration of Helsinki , as amended (Appendix A) and U.S. 21 CFR Part 50 – Protection of Human Subjects, and Part 56 – Institutional Review Boards. All Investigators will complete and sign the form FDA 1572. It is estimated that at least 85 volunteers receiving tafenoquine will complete the study prophylaxis section of the study.

2. INTRODUCTION/BACKGROUND

A. The Malaria Problem

Malaria is a leading cause of morbidity and mortality in many developing countries with an estimated 100 to 300 million infections worldwide and 1.0-1.5 million deaths.[1] *P. falciparum* malaria is considered the most important malaria parasite because it causes high morbidity and mortality particularly in Africa, Southeast Asia, and the tropical zones of the Americas.[2] Malaria has been, and continues to be a disease of military concern throughout the history of the U.S. Armed Forces through to the present.[3]

The sporozoite form of the *P. falciparum* parasite is transmitted through the saliva of *Anopheles* mosquitoes as they feed on human blood during bites. Within minutes after inoculation, the sporozoites travel through the blood and invade the liver where they undergo asexual division and mature.[4] The pre-patent period (the time between the mosquito bite and the first appearance of plasmodia in the peripheral blood) for the *P. falciparum* parasite in man normally ranges between 9-12 days. The shortest reported pre-patent period in man is 5 days while the longest (in individuals not taking an antimalarial drug) is 25 days.[5] From the liver, merozoites are released into the blood and invade the erythrocytes, where they develop into schizonts. In the case of *P. falciparum* malaria, there are no residual parasites in the liver after the initial cycle of entry, division, maturation and release. Drugs active on the exoerythrocytic stage (i.e., the hepatic stage) of the parasite's life cycle are known as causal prophylactic drugs. They effectively disrupt the life-cycle of the parasites, preventing parasitemia, systemic illness and further transmission. Drugs active on the erythrocytic schizonts are called blood schizontocides. These drugs are used to treat clinically apparent malaria infections, or as a suppressive prophylactic drug, which prevents clinical symptoms by destroying schizonts before they can cause illness.

There are currently no effective vaccines to prevent malaria infections. For non-immune individuals, drugs have been used over the years to prevent malaria. Although drugs such as chloroquine, pyrimethamine-sulfadoxine, mefloquine, and doxycycline have been used successfully in the past to prevent malaria infections, the advent of chloroquine-resistant strains of *Plasmodium falciparum* and *Plasmodium vivax* malaria parasites has necessitated the development of new drugs which can be used prophylactically to prevent malaria in those exposed to the parasite.

B. Current Drugs for Malaria Prophylaxis

At present, the principal drugs available for malaria prophylaxis are mefloquine and doxycycline. Mefloquine can be given weekly and is the usual recommendation given for travelers to Sub-Saharan Africa because of its high efficacy and weekly dosage regimen which is thought to enhance compliance.[6] Mefloquine resistance is widespread in SE Asia and has been reported in Africa as well.[7,8] Mefloquine is used as suppressive prophylaxis against the erythrocytic stages of *P. falciparum* as well as *P. vivax* malaria. Doxycycline 100 mg daily has been shown to be effective in western Kenya but it cannot be given to children and pregnant women and it often has distressing gastrointestinal adverse effects. Proguanil combinations such as atovaquone/proguanil (Malarone®, Glaxo Wellcome) have been shown effective against intense *falciparum* malaria exposure in western Kenya and so has azithromycin (Zithromax®, Pfizer) but neither are registered yet for the prevention of falciparum malaria.[9,10] Both require daily

medication and thus have less than ideal compliance characteristics.

C. Test Article: Tafenoquine

Tafenoquine is an 8-aminoquinoline with an additional methoxy group at the 2 position, a methyl group at the 4 position, and a 3-trifluoromethylphenoxy substitution at the 5 position of the quinoline ring. It is "an excellent candidate for clinical evaluation as: (1) a causal prophylactic; and/or (2) a blood schizonticidal drug against human malaria parasites, including poly-resistant *P. falciparum* and chloroquine-resistant *P. vivax*".[11] It is administered as a succinate salt of the base.

Pre-Clinical Pharmacology

The pre-clinical efficacy, toxicology and pharmacokinetics are summarized in this section. For more complete information, please refer to the Investigator's Brochure.

Efficacy

The prophylactic activity of tafenoquine against *P. cynomolgi* was evaluated in rhesus monkeys.[12] Tafenoquine is seven to fourteen times more potent than primaquine in the rhesus monkey as a prophylactic agent.
Tafenoquine also had prophylactic activity when given up to 3 days before infection at a single dose of 5.68 mg/kg, whereas primaquine had no activity when 16 mg/kg was given 1 day before infection. This suggests that the activity of tafenoquine persists for days after drug administration, unlike primaquine, which requires daily administration.
The prophylactic activity of tafenoquine against *P. berghei* was evaluated in mice.[13] A single oral dose of 40 mg/kg effected 100% protection, and a dose of 80 mg/kg provided complete protection when administered 3 days before infection.
Using standard *in vitro* techniques, tafenoquine has also been shown to have blood schizonticidal activity.[14] On an equimolar basis, tafenoquine was found to be 4.5 and 15.2 times more active than primaquine against chloroquine sensitive and resistant clones, respectively.

Toxicology

In addition to its greater efficacy in both *in vitro* and *in vivo* models, tafenoquine is less toxic than primaquine (see below). In addition, several hematological and chemistry tests provide good indicators of lack of toxicity.

Acute Toxicology

The acute toxicity of tafenoquine has been studied in several animal species, and tafenoquine was found to be several times less toxic than primaquine.[15,16]

Sub-Chronic Toxicology

The sub-chronic toxicity of tafenoquine succinate was studied in Fischer 344 rats at daily oral doses of 0, 3, 9, 16, 27, and 30 mg/kg/day for 28 days.[17] All animals survived to scheduled necropsy. Significant dose-dependent clinical findings included reduction in weight gain and food consumption, anemia with reticulocytosis, methemoglobinemia, leukocytosis and elevation of liver transaminases. Pathological findings included increases in spleen and liver weights, and histopathological evidence of pulmonary inflammation.
Sub-chronic toxicology of tafenoquine was studied in beagle dogs at daily oral doses of 0.5, 3, 6, 12 and 16 mg/kg/d for 28 days.[18] All animals survived to scheduled necropsy. Significant, dose-dependent clinical findings included weight loss, anemia, methemoglobinemia, thrombocytopenia and hypoalbuminemia. Pathological findings included increased liver and spleen weights with extramedullary hematopoiesis, as well as liver and pulmonary inflammation. In contrast, when similar toxicology studies were performed with primaquine in beagles at doses of only 1, 3 and 9 mg/kg/d for 28 days, deaths resulted at 3 and 9 mg/kg/d. [19] Other findings included coma, cardiomyopathy, muscle necrosis and ventricular arrhythmias.

Chronic Toxicology

The chronic toxicology of tafenoquine was studied in rats at oral doses of 0, 0.5, 6 and 18 mg/kg/d for 90 days, with a 90-day recovery period.[20] Six out of 20 high dose rats died during the study. Three of these animals failed to recover after CO_2 anesthesia for blood collection. Clinical findings include decreased weight gain, decreased food consumption, anemia, leukocytosis, methemoglobinemia and transaminase elevation. Pathological findings included alveolar proteinosis. During the recovery period, all clinical and pathological findings resolved, with the exception of the lungs. During this recovery period, mild chronic pulmonary inflammation was seen, thought to be part of the process of resolution of the acute findings, and thus secondary to the direct treatment-related effect.

The chronic toxicology of tafenoquine was studied in beagle dogs at oral doses of 0, 0.1, 2 and 6 mg/kg/d for 90 days, with a 90-day recovery period.[21] All animals survived to scheduled necropsy. Clinical findings included decreased weight gain, anemia (thought to be secondary to hemolysis), thrombocytopenia and methemoglobinemia. Pathological findings included extramedullary hematopoiesis, hepatic and pulmonary inflammation with alveolar proteinosis. All clinical and pathological findings resolved during the recovery period, again with the exception of the lungs. Mild chronic pulmonary inflammation was also seen in the dogs during the recovery period.

These findings suggest that tafenoquine succinate has many potential advantages over primaquine. Although primaquine has been shown to be effective as a prophylactic drug, its toxicity and the requirement for frequent dosing limits its use in this setting. Based on the efficacy of tafenoquine in animal models, its long half-life and greater therapeutic index, it has potential as a significantly improved prophylactic drug.

Previous Human Experience

To date, tafenoquine has been administered to a total of approximately 250 healthy volunteers.

The first study in man was a rising, single oral-dose safety and tolerance study in 48 male volunteers receiving doses from 4 to 600 mg base fasting.[22] The subjects tolerated all doses well. Sporadic complaints of mild headache and gastrointestinal symptoms were noted. There were sporadic, borderline elevations of transaminases, although they occurred in placebo controls with the same frequency and degree. Preliminary kinetic analysis of plasma samples from this study indicated that peak plasma levels occur at about 12 hours and increase linearly with dose. The half-life was at least 14 days, and appeared to be independent of dose. Whole blood concentrations were 1.75 fold greater than plasma concentrations, indicating that tafenoquine accumulates inside the red cell

The second study investigated the pharmacokinetics, pharmacodynamics, safety and tolerance of a single oral dose of tafenoquine succinate in 18 male volunteers.[23] Three groups of six fasting volunteers received 100, 200 and 400 mg. Tafenoquine was well tolerated. Sporadic mild complaints of headache and abdominal distress (gas, loose stools) were reported. One individual had mild elevation of serum transaminase enzymes after drug administration. However, upon review of his past records, it was noted that he had chronic borderline liver function test abnormalities. His values returned to his baseline during the follow-up period. A one-compartment model with a mean elimination half-life of 14.8 +/- 2.0 days best described the plasma and blood tafenoquine levels from this study.

The third study was a placebo-controlled, randomized pilot study to evaluate whether a single 600 mg oral dose of tafenoquine, when administered 1 day before sporozoite inoculation, could prevent parasitemia in non-immune male and female volunteers.[24] In this study, four volunteers received drug and two received placebo while fasting. The subjects who received placebo developed parasitemia and clinical malaria ten days after inoculation. Of the four on drug, three were completely protected by the single dose, whereas the fourth subject developed malaria on day 30. The peak blood concentration of tafenoquine in this individual was 244 ng/ml; in contrast, the three who were completely protected had peak levels of 417-489 ng/ml. Two drug-treated individuals had loose stools within hours after receiving drug, and one of these two was the individual who developed malaria. This gastrointestinal side effect may have resulted in the reduced drug concentrations.

The fourth study was a randomized, double-blind, placebo-controlled, multiple-dose safety and tolerance.[25] Thirty-six male and female subjects were enrolled, divided into three groups of 12. The three groups received weekly oral doses of 200, 400 and 600 mg/week for 10 weeks, respectively, while fasting. In each group of 12, eight subjects were randomly assigned to receive drug and four to receive placebo. A study report has not yet been received. However, correspondence with the study investigator has revealed that side-effects possibly due to study drug have all been mild and include: gastrointestinal disturbances (nausea, vomiting, diarrhea), headache, lightheadedness and dysgeusia. Methaemoglobin levels of up to 5% have been seen, and there were no abnormal trends in the remaining clinical labs or pulmonary function tests. While all three regimens were safe, the high dose was not thought to be well tolerated on an empty stomach due to the frequency of the GI effects.

The fifth study was a randomized, double-blind, placebo-controlled multiple-dose prophylaxis

study against *P. falciparum.*[26] Five non-immune volunteers were randomized to receive a loading dose of 600 mg on days -3 and -2 before sporozoite inoculation of the NF54 strain of *P. falciparum* using a standard mosquito inoculation model (day 0), followed by an additional 300 mg dose on days 5, 12, 19 and 26 (suppressive arm). Five additional volunteers were randomized to receive the same loading dose regimen (days -3, -2 and 5) with placebos on days 12, 19 and 26 (causal arm). Two volunteers received placebos at all dosing times. All doses were administered with breakfast. When it became apparent that the proposed regimen was not completely causally prophylactic in this setting, those volunteers in the causal arm were changed to the suppressive regimen, and also received the 300 mg maintenance doses for the last three weeks. All but one of the volunteers had positive parasite cultures. Six out of the ten drug treated individuals developed asymptomatic parasitemia. Five of these six were treated with chloroquine. The sixth was not treated and the parasitemia cleared. *In vitro* blood schizonticidal sensitivity studies suggested that the NF54 parasite is several times less susceptible to tafenoquine than most others isolates - even new multi-drug isolates from southeast Asia. This would suggest that if tafenoquine has causal prophylaxis activity, the dosing regimen used may not protect against all strains. The fact that these non-immune individuals, infected with parasites that are relatively resistant to study drug, had low parasite counts (<25 / ul) and no symptoms suggests that the weekly 300 mg dose may be close to being a completely suppressive regimen, and doses close to this should be studied further. The volunteers complained of mild, transient episodes of abdominal cramping, diarrhea and headache, lasting for a few hours after drug administration. MHb levels of up to 8% were seen. Preliminary concentration data suggest that co-administering tafenoquine with food may increase bioavailability 50% compared to the fasting state.

The sixth study in Thailand evaluated the safety and efficacy of tafenoquine in the prevention of relapse of *vivax* malaria in male and female patients.[27] The study compared a number of dosing regimens of tafenoquine (300mg daily for 7 days; 500mg daily for 3 days followed by a repeat regime 1 week later; single dose of 500 mg) following chloroquine blood schizontocidal treatment to chloroquine alone and chloroquine followed by a standard eradication course of primaquine. Up to 30 patients received each of the dosing regimes. In patients completing >2 months of follow-up the incidence of cure (ie. lack of relapse) was 100% on the 7 day tafenoquine regime and 85% or greater on the other tafenoquine regimes, compared to 25% on chloroquine alone and 75% on primaquine. In this study there was evidence of some gastrointestinal side-effects (nausea, abdominal pain, diarrhea) associated with the administration of tafenoquine.

In 1997, 249 persons in the same region of Kenya proposed for this study participated in a phase II dose ranging study of weekly tafenoquine compared to placebo. The findings of this study were that in persons with a normal Glucose 6 Phosphate Dehydrogenase (G6PD), weekly tafenoquine in the regimen proposed in this protocol is safe and well-tolerated. Tafenoquine (either 200 or 400 mg base) given weekly was highly efficacious (approx. 90% protective) in preventing any malaria parasitemia in adult semi-immune volunteers compared to an 92% malaria attack rate in the placebo group. The one serious drug-related adverse event was hemolysis in a woman whose G6PD test was incorrectly recorded. Despite the very long half life of tafenoquine (>2 weeks), following a 2 unit blood transfusion to restore her erythrocyte volume

the volunteer did not experience any further drug-induced hemolysis on follow up of several months. One other asymptomatic hemolytic event was detected also in a person whose repeat G6PD value was found to be deficient. GI side effects were found to be higher in the tafenoquine groups than placebo and appeared to be higher on the 400mg than the 200mg weekly regime.

In 1998, 205 members of the Royal Thai Army stationed on the Cambodian border participated in a double-blind, randomized, placebo-controlled trial of tafenoquine prophylaxis. Approximately half of the volunteers received a 400 mg loading dose for 3 days followed by 400 mg monthly for 5 months; the remainder received an identical placebo. Twenty-seven cases of malaria (18 *P. vivax*, 1 *P. falciparum*, and 1 mixed) occurred in the placebo group versus just one case of *P. vivax* malaria in the tafenoquine group. The overall protective efficacy was >95%. Nausea and vomiting occurred in 5% and diarrhea in 6% of the tafenoquine group compared to 1% in the placebo group. Methemoglobin levels never rose above 5%.

In 1998/99, 513 immune adult volunteers were randomized and treated in a 12-week randomized, double-blind, placebo-controlled trial in northern Ghana. Following radical cure with quinine, doxycycline, and primaquine, volunteers were randomized to tafenoquine 25 mg, 50 mg, 100 mg, and 200 mg or placebo. Seventy percent of the volunteers in the placebo group became infected with *P. falciparum*, while protective efficacy was > 95% in the three highest dose tafenoquine groups. Some evidence of transiently increased serum transaminase enzymes was noted, but none were clinically significant.

A double-blind, randomized, placebo-controlled dose ranging study was carried out in Gabon in 1999. A total of 415 immune volunteers between 12 and 20 years of age were treated with halofantrine (250 mg/day for 3 days) to clear their parasitemia and then received a loading dose of 25 mg, 50 mg, 100 mg, or 200 mg base of tafenoquine daily for 3 days, or placebo treatment, and followed for 70 days. A placebo attack rate of 25% was seen over the duration of the study. At 70 days the 200 mg tafenoquine treatment, as a loading dose over 3 days, showed a protective efficacy of 100%. As the tafenoquine dose decreased the protective efficacy also decreased. Abdominal pain was the main adverse event reported by volunteers which was higher following treatment with tafenoquine (up to 12%) compared to the placebo group.

In 1998/99, 259 Australian Army members of a peace-keeping force on the island of Bougainville received tafenoquine, 400 mg daily (as a single or a split dose of 200 mg x 2) for 3 days, while another 219 soldiers received primaquine 22.5 mg daily for 14 days at the end of their tour. This was a randomized open-label design. There were 7 *P. vivax* eradication failures each in the tafenoquine and primaquine groups, occurring between 6 and 20 weeks after leaving Bougainville. Approximately 5% of the volunteers receiving primaquine complained of gastrointestinal side effects (abdominal pain, nausea, vomiting) compared with 15% in the tafenoquine group. Incidence of gastrointestinal events appeared to be reduced by splitting the daily dose of tafenoquine (M. Edstein, personal communication).

D. Test Article: Mefloquine

Mefloquine in recent years has replaced chloroquine as a single agent prophylactic against

chloroquine-resistant *P. falciparum.* Mefloquine, which has a long half-life, was used originally as an every other week dose. Since there were prophylactic failures of *P. falciparum* with this dosing regimen, once weekly dosing is now recommended. [28]
Although mefloquine is generally well tolerated at prophylactic doses, it also is not without side effects. Neuropsychiatric side effects (such as dysphoria, dizziness, and rarely seizures, psychosis) have been reported, but the incidence is not significantly different with respect to chloroquine when tested in a blinded manner.[29] Western travelers tend to have many complaints that often focus on their antimalarial prophylaxis. A recent survey of British tourists found that 40% complained of some problem that they related to malaria prophylaxis.[30] The complaints were not different between mefloquine or proguanil combined with chloroquine except for neuropsychiatric adverse events which were significantly more common in mefloquine users. Still, less than 1% of travelers taking mefloquine complained of disabling neuropsychiatric adverse events.

Although mefloquine resistance has been documented in *P. falciparum* on the Thai-Cambodian and Thai-Burmese borders, mefloquine continues to be effective elsewhere, including Vietnam.[31] The IC_{50} of mefloquine against a chloroquine-resistant, mefloquine-sensitive clone of *P. falciparum* (W2) is about 0.6 ng/ml; against a chloroquine-sensitive, mefloquine-resistant clone (D6), the IC_{50} is approximately 4 ng/ml. The metabolism of mefloquine is incompletely understood. However, it appears that one of its metabolites is 2,8-bis (triflouromethyl) -4-quinoline carboxylic acid is active; its antimalarial role, if any, has not been well established.

Mefloquine HCl is used for both treatment and prophylaxis. The treatment regimen as labeled (Lariam®) consists of a single dose of 10-25 mg/kg as a single or split dosage. For prophylaxis, mefloquine is given as a single dose of 250 mg weekly.

The pharmacokinetics of mefloquine has been reviewed.[32] Mefloquine is rapidly absorbed, with a bioavailability of around 85%. The volume of distribution is less than that of chloroquine and ranges between 9 and 50 l/kg. Following a single 250 mg dose of mefloquine HCl given to healthy males, the mean time to peak was 13.5 hours. [33] The mean half-life was 292 hours (range 155-503); it is modeled best using a one- or two-compartment model.[34]

The serum concentrations necessary for mefloquine to be effective have not been well-established. In a study in which Peace Corps volunteers' mefloquine serum concentrations were determined after their most recent mefloquine dose and correlated with *P. falciparum* breakthroughs, the failures occurred in the second week after their last dose; however, although serum mefloquine concentrations decreased gradually over the two week time period, the correlation of serum concentrations between failures and non-failures was poor.[35] Serum concentrations of mefloquine in those volunteers who failed prophylaxis ranged from 62-398 ng/ml (mean 262 ng/ml). However, there were other individuals who had similar concentrations in the first week.

In another study, an attempt was made to correlate mefloquine treatment failures with serum mefloquine concentrations and resistance patterns of *P. falciparum in vitro.*[36] The authors were

unable to find a positive correlation. In yet another study, blood concentrations were not associated with treatment outcome.[37] The reasons for the lack of pharmacodynamic-pharmacokinetic correlation are unknown.

E. Pre-prophylaxis treatment: Halofantrine

In holoendemic areas such as the proposed study site, all adults must be regarded as infected even if repeated blood smears are negative. When studying malaria in endemic populations the study end point is parasitemia rather than symptomatic malaria due to the ability of most adults to suppress clinical signs and symptoms of acute malaria infection. This necessitates a pre-prophylaxis treatment course with an effective antimalarial drug. Halofantrine has been shown safe and effective for this purpose and is registered in both Kenya and the USA for malaria treatment. Prior to starting prophylaxis, volunteers will receive 750mg of halofantrine divided into three doses over three days. This modification of the usual treatment regimen (given over 3 days instead of 1 day) has been used successfully western Kenya for the purpose of eliminating pre-existing malaria parasitemia.[38] EKG measurement will be done prior to study entry to eliminate rare individuals with congenitally long QT intervals that might be susceptible to dysrhythmias induced by halofantrine.

3. JUSTIFICATION FOR INVESTIGATION

The malaria parasite continues to develop resistance to common antimalarial drugs. Chloroquine is largely ineffective in East Africa and there are now questions about the evolution of pyrimethamine /sulfadoxine resistance.[39] The parasite is evolving new means of drug resistance faster than the pharmaceutical industry is developing new antimalarial drugs. The need to develop new drugs and new regimens of already licensed antimalarial drugs is driven by the introduction of large numbers of non-immune persons into areas of high malaria endemicity. Prophylaxis or the prevention of symptomatic malaria infections has an important role for the country of Kenya in that epidemics of falciparum malaria are now endangering a new non-immune population as previously non-malarious areas develop seasonal transmission.[40] Medications such as tafenoquine that have the ability to block the further transmission of malaria infections may have a particular role in combating epidemic malaria. The tourist industry of Kenya is an important source of foreign exchange earnings and it depends on being able to bring large numbers of tourists to malaria endemic areas safely.

The US military has recently been challenged by operations in three areas of the African continent: Liberia, Somalia, and Rwanda. Malaria casualties resulted from all three operations. In Somalia the need for a weekly regimen that could be enforced by unit leaders as opposed to a daily regimen taken by the individual soldier was demonstrated by the decreased failure rates of weekly mefloquine versus daily doxycycline.[41]

Long-term loading followed by weekly prophylaxis studies performed to date (Kenya and Ghana; see previous human experience) indicate that doses of tafenoquine of 100mg and above are highly efficacious in prevention of malaria in a semi-immune population in intensely endemic conditions. From a safety and tolerability standpoint, the main issue for tafenoquine

appears to be gastrointestinal side effects and there is evidence that these are dose related. It is therefore proposed to investigate a dosing regime of 200 mg of tafenoquine as a 3-day load followed by weekly treatment, as the optimal balance between efficacy and tolerability, in this semi-immune population. The mefloquine regimen (loading of 750 mg over 3 days and then 250 mg/week) is known to be safe and well tolerated from studies done in US Marines and Indonesian soldiers.[42, 43] The placebo group is to account for variations in malaria attack rates which occur based on time and place.

4. GOALS AND OBJECTIVES

A. Primary Objectives

- To determine the prophylactic chemosuppressive efficacy of weekly tafenoquine in preventing falciparum parasitemia compared to placebo

B. Secondary Objectives:

- To compare the safety and tolerability of weekly tafenoquine given for 24 weeks with that of placebo.
- To provide comparative data on the effectiveness, safety and tolerability of tafenoquine versus mefloquine
- Determine the impact of chemoprophylaxis with mefloquine and tafenoquine on the subsequent susceptibility to malaria.
- .If an increase in the susceptibility to malaria is observed following prophylaxis, identify the immune responses that are most affected by each regimen.
- To characterize the population pharmacokinetics of tafenoquine and evaluate the effects of various subject characteristics on tafenoquine pharmacokinetics.

5. DESIGN AND METHODOLOGY

A. Study site (geographical)

The anticipated study site is Kombewa village, in Nyanza Province, Kenya, about 25 km from the town of Kisumu on the shore of Lake Victoria in East Africa. Several previous field trials of malaria prophylaxis/treatment and immunity have been done at this site.

B. Study population

Volunteers will be male and female adults 18-65 years of age who are in good health. Only women who are neither pregnant nor breast-feeding will be eligible. In previous similar studies the proportion of female volunteers has varied from 30-50%.

1. Malaria in western Kenya

Epidemiology: Malaria in Western Kenya is holoendemic and representative of the general situation in sub-Saharan Africa. The chief characteristic is intense transmission via *Anopheles gambiae*. The villages in the study area have been the sites of many malaria epidemiology and chemotherapy studies for over a decade. Malaria transmission occurs at a low level all year but at a very high level during the two rainy seasons. The "long" rains from April through June produce intense transmission from May through July; the "short" rains of August through October produce another transmission season from September through November. Both of these transmission seasons can be predicted by the amount of rain seen in the preceding month and correlate with the entomological inoculation rate (EIR)[44], which is the number of infected mosquito bites per person per day as well as the total population of female anopheles. Cumulative malaria attack rates have been in the order of 95% during the "long" and 75% during the "short" rains. Over several years of study, >95% of adolescents ages 9-12 years have been found to develop patent parasitemia during a ten week study period. The adult population has also been the subject of several malaria chemotherapy and epidemiology studies.[45] These studies demonstrate about 90% incidence of malaria parasitemia over 10 weeks during a high transmission period. Approximately 25% of the infected adults will be symptomatic some time during that period. These studies show that approximately 80-90% of volunteers complete a study of 12 weeks duration i.e. there is a 10-20% dropout rate. Typically, chemosuppression studies have been initiated, executed and completed within 3-4 months.

Immune Status: Adults permanently living in the study area have all had multiple malaria infections and should be considered at least semi-immune. This relative immunity protects against clinical disease but does not appear to influence the rate of parasitemia appearing after initial clearance of parasites (1995-97 chemosuppression studies done at USAMRU-Kenya). We propose to study the efficacy of tafenoquine to prevent both patent infection measured by parasitemia and malaria disease measured by parasitemia in conjunction with symptoms consistent with malaria.
Serologic markers of malaria exposure as determined by ELISA (Enzyme Linked Immunosorbant Assay) to different stages of the parasite will be followed over time. Peripheral blood lymphocytes will be used for studies of malaria cellular immunity.
Drug resistance: Although not of the same order as multiple drug resistance seen in Southeast Asia, East Africa does have multiple drug resistance problems. Chloroquine fails to cure 80% of falciparum cases.[46,47] Pyrimethamine /sulfadoxine has at least a 20% failure rate during previous USAMRU-K field trials in the same area. Since mefloquine and artemisinin are not commonly used, it is difficult to give clinically relevant information on them. Tourists have contracted mefloquine-resistant malaria in East Africa.[48]

2. Recruitment Procedure

Volunteers will be recruited by non-coercive methods according to existing policies of the Kenya Medical Research Institute (KEMRI) and U.S. Army regulation and

USAMRMC reg. 70-25, (Use of Volunteers as Subjects of Research). Trained field workers from the region will speak with potential volunteers to inform them of the study and its aims. Meetings will be held to further inform interested individuals. Subjects will receive oral and written explanation of the study, after which written Informed Consent will be obtained. Witnessed oral consent will be accepted for illiterate persons. Up to 400 individuals will be screened. After screening for inclusion/exclusion criteria, 300 subjects will be enrolled and be randomized to the three separate arms of the study A log will be kept of all volunteers who were considered for entry into the study, and the reason for not entering, where applicable.
Screening of volunteers will occur in April 2000. A healthy volunteer is defined as one who is free of ailments that might cause difficulty in evaluating drug efficacy or adverse experiences.

6. INFORMED CONSENT

6 A General Considerations

The principles of informed consent in the current edition of the Declaration of Helsinki (Protocol Appendix A) will be implemented in each clinical study before protocol-specified procedures are carried out.

Informed Consent will be obtained in accordance with 21 CFR 50.25 and ICH GCP.

Information will be given in both oral and written form whenever possible and deemed appropriate by the IRB. Subjects, their relatives, guardians or, if necessary, legal representatives must be given ample opportunity to enquire about details of the study.

The consent form generated by the investigator will be approved (along with the protocol) by the IRB. Consent forms must be in a language fully comprehensible to the prospective subject. Informed Consent shall be documented by the use of a written consent form approved by the IRB and signed by the subject or the subject's legally authorised representative. In the event of illiteracy, a thumbprint will be acceptable in lieu of a signature and the written informed consent will be read and explained to the subject.. Witnesses will document this process by signing all consent forms.

The written consent document will embody the elements of informed consent as described in the Declaration of Helsinki and will also comply with local regulations. This form may be read to the subject or the subject's legally authorised representative, but, in any event, the investigator shall give either the subject or the representative adequate opportunity to read it before it is signed.

Consent will be documented either by the dated signature of the subject or the subject's legally authorised representative. The signature confirms that the consent is based on information that has been understood. Each subject's signed Informed Consent form must be kept on file by the investigator for possible inspection by Regulatory Authorities and/or the sponsor's professional

and Regulatory Compliance persons.

Full and comprehensive information must be communicated to the potential subject. If the subject is illiterate an impartial witness will be present during the entire Informed Consent procedure. The witness will be an independent third party i.e. not a nominated co-investigator. The witness will sign the Informed Consent form (testifying that informed consent has been given orally) along with the Investigator (or his/her nominated representative).

6B The Informed Consent Process

It is recognised that requesting female subjects not to become pregnant up until 2 weeks after the second course of halofantrine i.e. 39 weeks after entering the study has implications for their sexual partners. Therefore, female subjects will be asked to invite their partners to be present whilst giving Informed Consent, although they will not be prohibited from entering the study if they refuse to do so.

7. INCLUSION CRITERIA

1. Healthy volunteers (male or female) providing Informed Consent
2. Age: 18-55 years old
3. Planning to reside in the study area for the entire duration of the study of approximately 70 weeks

8. EXCLUSION CRITERIA

1. Positive parasitemia following halofantrine treatment for radical cure.
2. Any medical condition which in the opinion of the investigator makes the subject unsuitable to enter the study
3. Personal or family history of seizures
4. Positive serum β-HCG (to be tested during screening and within 48 hours of first drug administration and approximately monthly thereafter).

note: All female volunteers will have β-HCG testing. Those requiring repeat testing due to the lapse of >48 hrs will have their original negative test confirmed by an additional urine test within 48 hours of initiation of study medication.

5. Women who are pregnant or lactating or who in the opinion of the investigator are at risk of becoming pregnant.
6. Clinically significant abnormalities (to include but not limited to abnormal hepatic or renal function) as determined by history, physical and routine blood chemistries and hematology values.
7. Has demonstrated hypersensitivity to any of the study drugs especially to any other 8 - aminoquinolines
8. Unwilling to report for drug administration or blood drawing during the 70 week duration of the study

9. Glucose 6 Phosphate Dehydrogenase deficiency (G6PD)
10. Laboratory guideline values for exclusion are values Hb < 10 gm/dL, PLT < 80,000/mm3, WBC < 3000 l^3, Creatinine or ALT more than 2 times the upper limit of normal for age.
11. Abnormal EKG particularly an extended QTc interval >0.4 sec.
12. Subject is taking any other anti-malarial product , or has taken an antimalarial drug other than halofantrine within the previous 2 weeks
13. Subject has received an investigational drug (a new chemical entity not registered for use) within 30 days or 5 half-lives whichever is the longer.
14. Subject has a history of psychiatric disorder.

9. STUDY PROCEDURES

A. General Instructions

All subjects will be tested for G6PD deficiency during the screening process i.e. day –21 to –6. This will be done by using standard laboratory tests. Any subject whose result is 'Low' or 'Intermediate' after the qualitative test will be excluded from the study i.e. only those with a reading of 'Normal' are eligible. Subjects will undergo 2 tests from different blood samples and only those with a result of 'Normal' on both occasions will be eligible.

All female subjects must undergo serum or urine pregnancy test within 48 hours of first administration of the clearance drug, halofantrine. All female subjects will also be tested for pregnancy prior to commencing randomized study medication and at intervals of every 4 weeks throughout the prophylaxis treatment phase of the study. At visits where blood samples are taken for safety testing the serum test will be used; on other occasions the urine test will be used.

All eligible subjects will be treated initially with halofantrine 250 mg for 3 days (Days –5 to -3) in order to clear any pre-existing malaria parasites/parasitemia.

On Day -2, a thick blood smear will be performed. Subjects with demonstrated negative thick blood smear will be randomized to one of the three prophylaxis treatment groups on day 0. The prophylaxis treatment period will be for 24 weeks and will be monitored on a weekly basis by field workers. Subjects who still have a positive smear after halofantrine treatment are ineligible and will be withdrawn from the study.

The participating field workers will obtain all blood samples for the thick blood smears by finger prick method on the elected days. Venous blood sampling for clinical chemistry and hematology assessments, measurement of plasma drug levels and malaria antibodies will be made at intervals throughout the study and on these occasions subjects will be required to come into the clinic. Venous blood sampling on any other visit must be clinically justified e.g. adverse experience or concurrent illness requiring investigation.
After the 24 week treatment period subjects will attend a safety follow-up visit during week 28.

Subjects will then enter the immunological follow-up and continue to be monitored on a weekly basis for a further 9 weeks until week 37 as a washout period of study drug. At week 37,

subjects will be given a further treatment course of halofantrine 250mg for 3 consecutive days, and will be followed up until week 70. The 2 phases of the study will be the reported separately. The prophylaxis report will include the data from each subject only up until 4 weeks after his/her last dose of study medication.

For both phases of the study, prophylactic treatment and immunological follow-up, subjects will be visited weekly by health workers who will perform blood smears and follow-up assessments.

If a subject is found to have a demonstrable positive thick blood smear (thick blood smear with *Plasmodium spp.* as defined in section 13A) at any of the assessment visits they will be considered a prophylactic failure and will be withdrawn from the treatment part of the study. They will enter the four week post-treatment safety follow-up part before progressing into the immunologic follow-up phase of the study.

Withdrawn subjects may be treated with an appropriate anti-malarial therapy at the investigator's discretion depending upon whether or not the subject is suffering from malaria symptoms.
A Case Report Form (CRF) will be completed for each subject entered into the study.

The 2 phases of the study i.e. the prophylaxis phase up until week 28 and the subsequent immunological follow-up until week 70 will be reported separately.

B. Schedule of assessments

See also Study Assessment Schedule in Appendix E.

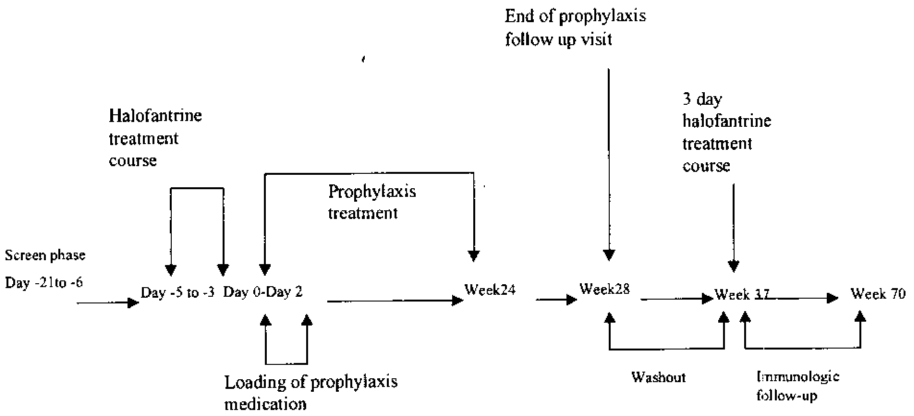

1. Screening: Day –21 to -6

The following procedures/data collection will be performed during the screening period

- Informed Consent
- Demographics: Date of birth, sex, weight, ethnicity,
- Medical History
- Physical Examination
- G-6PD status
- Blood sample taken for hematology and clinical chemistry
- Serum Pregnancy Test (women only)
- 12 lead EKG
- Vital signs

If the subject fulfills all of the entry criteria and the laboratory requirements are met, the subject will commence 3 days treatment of halofantrine

2. Parasitemia Clearance treatment : Day –5 to –3

All volunteers eligible to enter the study will receive 3 days treatment of halofantrine 250mg /day. If female subjects have not been tested for pregnancy within the previous 48 hours this will be repeated before halofantrine treatment is started on day –5.

At each of these visits information will be collected on medication compliance, adverse experiences, concomitant medication.
A blood smear will be taken on day –2 and reported prior to initiating study prophylaxis medication to ensure that there is no parasitemia.

3. Start of randomized double-blind study medication: Day 0

At Day 0, the subject will be randomized to receive study medication if study eligibility criteria are still met and the following procedures will be carried out:

- Collect information on Adverse Experiences
- Collection of information on concomitant therapy
- A sample of blood taken for laboratory safety assessments and pregnancy test
- A sample of blood taken for Peripheral Blood Mononuclear Cells (PBMCs) (120 volunteers only)
- Blood sample for malaria antibody measurement
- Measurement of vital signs (pulse and blood pressure)
- First study drug administration (if thick blood smear negative for parasitemia)

4. Initial loading of prophylaxis medication : Day 0 to Day 2

Subjects will receive 3 consecutive days treatment of their double-blind, randomized medication. On each of these occasions, subjects will be asked to provide details on any changes in concomitant medication, and will asked the non-leading question "Have you felt unwell since starting the new treatment/or since the last visit?"
One hundred subjects will be asked to provide an additional blood sample for analysis of plasma drug levels 6-12 hours after the third loading dose.

5. Weekly medication visits: Day 7 (Week 1) to Week 24

After the initial loading doses, volunteers will receive their double-blind medication on a weekly basis during visits from health workers. Health workers will also check for any adverse experiences by asking the non-leading question "Have you felt unwell since starting the new treatment/or since the last visit?" In addition, blood smears will be taken for parasitology, and any changes in concomitant medication will be recorded.

Vital signs and blood samples for routine clinical chemistry and hematology will be done at weeks 4,8,16, 24, and at safety follow-up (week 28 or 4 weeks post last dose of study medication). Blood samples for analysis of antibody levels will be taken at the randomisation visit, weeks 4, 8, 24 and 28.

Blood samples for pharmacokinetic testing will be taken at week 4, week 8, week 16 at the same times as the safety samples. In a further 100 subjects an additional pharmacokinetic sample will be taken at week 17 prior to taking study medication. These will be the same subjects who give

the sample of the loading dose on Day 3.
Subjects will be required to attend the clinic for visits where blood samples are taken.

6. Safety Follow-up Visit: Week 28

All subjects will attend the clinic for a safety follow-up visit 4 weeks after their last dose of study medication, even if they withdraw from the prophylaxis part of the study before the end of the 24 week treatment period.

At this visit the following procedures will be carried out:

- Physical examination (including vital signs)
- Collect information on Adverse Experiences
- Collection of information on concomitant therapy
- Blood sample taken for safety assessments
- Blood sample for pharmacokinetic sampling
- Blood sample for measurement of Peripheral Blood Mononuclear Cells (PBMCs) in the same 120 volunteers who gave samples at screening.

Subjects will continue to be followed up on a weekly basis in the immunologic follow-up.

7. Immunologic follow-up

The second follow-up period, the immunological follow-up, will commence after the completion of the prophylactic follow-up at week 28. Female subjects will have a pregnancy test performed within 3 days prior to starting the treatment course of halofantrine. Subjects will continue to have weekly smears and have free access to the Walter Reed Project Kombewa Clinic as above for a further period of 9 weeks as period of washout for study medication. At this point, approximately week 37, volunteers will be treated again with halofantrine 250mg for 3 days and observed until week 70 for the development of parasitemia in the same fashion as during the first follow-up period.
On the following occasions all subjects will be required to come to the clinic:

Weeks 37, 50, 60 and 70

A blood sample will be taken in the clinic for measurement of plasma malaria antibodies.

Week 38

The selected 120 subject subset will be required to come to the clinic to give a blood sample for measurement of Peripheral Blood Mononuclear Cells (PBMCs)

<u>10. SUBJECT IDENTIFICATION AND RANDOMISATION:</u>

Randomization will be by a predetermined randomization procedure. Volunteers will be randomized to one of the three treatment groups using blocked randomization. This will achieve balance in number of subjects assigned to each treatment arm and will control for possible time of entrance effects. The master code linking subject study number to treatment group will be kept by the sponsor. Individual code break envelopes will be provided to be held on site but will only be opened in the event of an emergency and after prior consultation with the study sponsor and medical monitor.

11. DRUGS AND DOSAGE

A. General

This will be a double-blind, double dummy, placebo controlled study. Drugs will be pre-packaged and pre-randomized as described above.
Tafenoquine will be supplied as Swedish Orange, size 1, hard gelatin capsules, each containing tafenoquine succinate equivalent to 200mg (pure free base). A matched placebo will be identical in external appearance to the active capsules.

Mefloquine will be supplied as white round tablets, each containing mefloquine 250mg (pure free base). A matched placebo will be identical in external appearance to the active tablets.

Tafenoquine: Volunteers randomized to tafenoquine will receive 200 mg of tafenoquine for 3 days as described above. Following that they will receive 200 mg of tafenoquine in the form of one 200 mg capsule once a week. They will also receive mefloquine placebo.

Mefloquine: Volunteers randomized to mefloquine will receive 250 mg of mefloquine (one 250 mg tablet) for 3 days as described above. Following that they will receive 250 mg of mefloquine once a week. They will also receive tafenoquine placebo.

Placebo: Volunteers randomized to placebo will receive weekly medication identical to the above tafenoquine capsules and mefloquine tablet except that no active drug will be contained in either. This will apply to the loading doses in the first three days and to the weekly medication.

Emergency drugs for the treatment of allergic reactions will be kept at the study site. Any allergic reactions will be treated promptly according to standard medical protocols.

B. Administration of study drug

Drug dosages will be administered under observation. The study subject will sign that medication was taken as well as the health worker administering the drug. The health worker will visit the volunteer the following day; if vomiting has occurred the dose will be re-administered. If the volunteer sequentially vomits two doses of medication he will be regarded as intolerant of study medication and withdrawn from the study.

To minimize gastrointestinal symptoms with drug administration, a small meal will be given to all participants to eat before ingesting their medication. Subjects who miss a once weekly dose of medication will be given the same dose the following day.

If a subject should miss 2 consecutive doses of study medication then he or she will be withdrawn from the study. If a subject should miss 2 non-consecutive doses the investigator should contact the monitor of the study to discuss whether or not the subject can remain in the prophylaxis part of the study i.e. on a case by case basis.

Drug will be administered under supervision for the 3 day loading dose and the 24 weeks as follows:

**Randomization group n=100each group**	**loading regimen (days 0 to 2)**	**weekly regimen (week 1 to week 24)**
tafenoquine 200mg (pfb)	tafenoquine one 200 mg capsule<br>mefloquine placebo one tablet	tafenoquine one 200 mg capsule<br>mefloquine placebo one tablet
mefloquine	tafenoquine placebo one capsule<br>mefloquine one tablet 250 mg	tafenoquine placebo one capsule<br>mefloquine one tablet 250 mg
Placebo	tafenoquine placebo one capsule<br>mefloquine placebo one tablet	tafenoquine placebo one capsule<br>mefloquine placebo one tablet

SmithKline Beecham, UK, will package the study medication.

C. Packaging and labelling

All study medication will be provided in white opaque high-density polyethylene (HDPE) bottles with white, opaque, plastic, child-resistant caps with coated polyester film bonded aluminum foil inner seal.

Each bottle will contain 30 dose units. The bottles will be labeled with a small plain white label. Each subjects supplies will comprise two bottles contained in a small carton, labeled with a small label with a tear-off portion.
The label text will contain the following information:

- Patient identification (initials and/or name) handwritten
- Job Number
- Protocol Number
- Medication Number
- Contents: i.e. quantity and description of dose form.
- Dosage Directions i.e. 'As directed'.
- Storage instructions
- "Keep out of reach of children"
- "FOR CLINICAL TRIAL USE ONLY"
- The sponsor address

- Any other information required by the regulatory authorities in the participating country.

The study medication should be protected from light and stored at room temperature (between 15° to 30° C) under secure conditions.

12. DRUG ACCOUNTABILITY

The investigator/pharmacist/nurse/field worker must sign that he/she received the clinical supplies for the study, confirming the quantity received. The pharmacist/nurse/field worker is also responsible for appropriate handling and storage of the study medication. The pharmacist/nurse/field worker has to ensure that the study medication is only dispensed to study subjects in accordance with the protocol and that a record is maintained of all study medication dispensed to each subject. All unused study medication must be returned to SB. Certificates of returns must be signed with the assurance from the study pharmacist/nurse/field worker that all part used and unused study medication packs for this study has been returned. At the end of the study it must be possible to reconcile delivery records with those of used and returned stocks. A written account must be given of any discrepancies

13. CONDUCT OF STUDY

A. Evaluation of malaria

Thick and thin smears for malaria will be obtained by finger prick during Screening prior to starting halofantrine (day –5), after clearance medication i.e. on Day –2, and then once a week during drug treatment (scheduled slides) and immunologic follow-up. Additional smears will be done any day a subject is ill or thought to have malaria (unscheduled slides). Every attempt will be made for the smears to be taken 7 days apart, but any smear obtained during the week will be counted as compliant with the parasitemia monitoring requirement.

Thick blood films will be stained and evaluated by standard techniques and read by 2 blinded microscopists. Duplicate thick and thin smears will be prepared and stained with Giemsa. Each slide will be read by a separate microscopist. Asexual stage parasites in 200 high power fields will be counted and reported as the number of parasites per 500 white blood cells. Negative/positive discrepancies will be resolved by a third microscopist (tie breaker) who will re-read both slides. The result of this re-read will be considered final.

Blood smears identified as positive for *Plasmodium spp.* through this procedure will result in the subject being considered a prophylactic failure (ie. have met the study endpoint) and will be termed a 'first positive' parasitaemia.

Subjects considered a prophylactic failure will be withdrawn from the treatment phase of the study, enter the post-treatment safety follow-up phase, and be given treatment according to local clinical practice. During the post-treatment safety follow-up subjects will continue to be monitored weekly for parasitemia. If, during the course of this follow-up, a subject has two

consecutive blood smears which are both positive (as outlined by the procedure above), this will be termed a 'confirmed' parasitaemia.

In addition to evaluation through blood smears, subjects will be assessed at visits for symptoms of malaria. For the purposes of data analysis only, a further definition of parasitemia will involve classification into 'symptomatic' and 'asymptomatic' parasitemia. Symptomatic malaria will be defined as either first positive or confirmed parasitemia (see above) plus the presence of clinical symptoms consistent with malaria, such as, fever, chills, headache, myalgias, nausea/vomiting, diarrhea not otherwise explained. Asymptomatic malaria will be defined as First Positive or Confirmed parasitemia with no associated symptoms.

Subjects are able to visit the clinic at any time if they are feeling unwell for 'unscheduled visits'.

B. Management of malaria

Field workers who dispense medicine and assess the health of the subjects will visit subjects on a scheduled weekly basis. A subject who becomes ill or is suspected of having malaria between scheduled visits will have additional ('unscheduled') smears performed and read immediately.

Subjects who are prophylactic failures will be withdrawn from the prophylaxis part of the study and given treatment according to local clinical practice, such as a single dose (three tablets) of pyrimethamine/ sulfadoxine or another treatment at the discretion of the investigator. Such treatments may include, for example, mefloquine, chloroquine, primaquine, halofantrine, or doxycycline. These subjects will be monitored for four weeks as part of the post-treatment safety follow-up and then continue into the immunological follow-up study.

14. ADVERSE EXPERIENCE REPORTING

The recording of adverse experiences is an important aspect of study documentation. Detailed guidelines are set out below.

It is the responsibility of the investigator to document all adverse experiences that occur during the investigation.

A. Definition

An Adverse Experience includes any noxious, pathological or unintended change in anatomical, physiological or metabolic functions as indicated by physical signs, symptoms and/or laboratory changes occurring in any phase of the clinical study whether associated with the study medication, active comparator or placebo and whether or not considered drug related. This includes an exacerbation of pre-existing conditions or events, concurrent illnesses, drug interaction or the significant worsening of the disease under investigation that is not recorded elsewhere in the case record form under specific efficacy assessments. Anticipated day-to-day fluctuations of pre-existing conditions, including the disease under study, that do not represent a clinically significant exacerbation or worsening need not be considered adverse experiences.

Discrete episodes of chronic conditions occurring during a study period should be reported as adverse experiences in order to assess changes in frequency or severity.
Adverse experiences should be documented in terms of a medical diagnosis(es). When this is not possible, the adverse experience should be documented in terms of signs and/or symptoms observed by the investigator or reported by the patient at each study visit.
Any pre-existing condition or signs and/or symptoms present in a subject/patient prior to the start of the study should be recorded on the Medical/ Surgical history form within the subject's/ patient's case report form (CRF).
Adverse experiences which occur after informed consent is obtained, but prior to starting active or randomised treatment will be documented on the 'Baseline signs and symptoms' report form as instructed by the SB clinical study monitor. All adverse experiences occurring after administration of the first dose of study medication (i.e., active drug, placebo, comparator drug) must be reported on the Adverse Experience form in the subject's CRF. All subsequent adverse experiences, whether no drug (i.e. during reference 'run-in' or 'wash-out' period) or when active drug or placebo is being administered, must be reported REGARDLESS OF WHETHER OR NOT THEY ARE CONSIDERED DRUG RELATED.
Serious adverse experiences which occur during the clinical study or within 3 months of receiving the last dose of study medication, whether or nor related to study drug, must be reported.
Instances of death, cancer or congenital abnormality if brought to the attention of the Investigator AT ANY TIME after cessation of study medication AND considered by the Investigator to be possibly related to study medication, should be reported to the Site Monitor and medical monitor.
At each visit/assessment, the investigator will evaluate adverse experiences. Adverse experiences not previously documented in the study will be recorded in the adverse experience record form within the patient's CRF. The nature of each experience, date and time (where appropriate) of onset, outcome, course (i.e. intermittent or constant), maximum intensity, action taken with respect to dosage and relationship to treatment should be established. Details of changes to the dosage schedule or any corrective treatment should be recorded on the appropriate pages of the CRF.
Adverse experiences already documented in the CRF (i.e., at a previous assessment) and designated as 'ongoing' should be reviewed at subsequent visits as necessary. If these have resolved, the documentation in the CRF should be completed. If an adverse experience increases in frequency or severity during a study period, a new record of the experience will be started.
As a consistent method of soliciting AEs, ask the subject a non-leading question such as:
"Have you felt unwell since starting the new treatment/or since the last visit?"

B. Recording of Intensity

Maximum intensity should be assigned to one of the following categories:

Mild: An adverse experience that is easily tolerated by the subject, causing minimal discomfort and not interfering with everyday activities.
Moderate: An adverse experience that is sufficiently discomforting to interfere with normal everyday activities.
Severe: An adverse experience that prevents normal everyday activities.

Every effort should be made by the investigator to explain each adverse experience and assess its relationship, if any, to study drug treatment. Causality should be assessed using the following categories: not related, unlikely, suspected *(reasonable possibility)*, probable.
Not related: The adverse experience is definitely not related to the test drug.
Unlikely: There are other, more likely causes and the drug is not suspected as a cause.
Suspected *(reasonable possibility)*: A direct cause and effect relationship between the drug and the adverse experience has not been demonstrated but *there is a reasonable possibility that the experience was caused by the drug.*
Probable: There *probably* is a direct cause and effect relationship between the adverse experience and the study drug.
The degree of certainty with which an adverse experience is attributed to drug treatment (or alternative causes, e.g. natural history of the underlying diseases, concomitant therapy, etc.) will be determined by how well the experience can be understood in terms of one or more of the following:

1. Known pharmacology of the drug
2. Reaction of similar nature being previously observed with this drug or class of drug
3. The experience having often been reported in literature for similar drugs as drug related e.g. skin rashes, blood dyscrasia
4. The experience being related by time to drug ingestion terminating with drug withdrawal (dechallenge) or reproduced on rechallenge

C. Follow-up of Adverse Experiences

Investigators should follow-up subjects with adverse experiences until the event has subsided (disappeared) or until the condition has stabilised. Reports relative to the subsequent course of an AE noted for any subject must be submitted to the clinical study monitor.

15. SERIOUS ADVERSE EXPERIENCES

A. Definition

A serious adverse experience is any event which is fatal, life threatening, disabling/incapacitating or results in hospitalisation, prolongs a hospital stay or is associated with congenital abnormality/birth defect, cancer or overdose (either accidental or intentional), or a persistent or significant disability/incapacity. In addition any experience which the investigator regards as serious or which would suggest any significant hazard, contraindication, side effect or precaution that may be associated with the use of the drug will be documented as a serious event.
The medical monitor must review all serious and unexpected adverse events associated with the protocol and provide an unbiased report of the event within 10 calendar days of the initial report. At a minimum the medical monitor should comment on the outcome of the event and relationship of the AE to test medication. The medical monitor should also indicate whether or not he concurs with the details of the report provided by the study investigator

Life threatening - definition:

An adverse experience is life threatening if the subject was at immediate risk of death from the event as it occurred (i.e., it does not include a reaction that if it had occurred in a more serious form might have caused death). For example, drug induced hepatitis that resolved without evidence of hepatic failure would not be considered life threatening even though drug induced hepatitis could be fatal.

Disability/incapacitating - definition:
An adverse experience is incapacitating or disabling if the experience results in a substantial and/or permanent disruption of the subject's ability to carry out normal life functions.

Hospitalisation - definition
AEs requiring hospitalisation should be considered serious. Hospitalisation for elective surgery or routine clinical procedures that are not the result of an AE (e.g., elective surgery for a pre-existing condition) need not be considered AEs and should be recorded on the medical/surgical procedures form. If anything untoward is reported during the procedure, that occurrence must be reported as and AE, either 'serious' or 'non-serious' according to the usual criteria.
In general, hospitalisation signifies that the subject has been detained (usually involving at least an overnight stay) at the hospital or emergency ward for observation and/or treatment that would not have been appropriate in the physician's office or out-patient setting. When in doubt as to whether 'hospitalisation' occurred or was necessary, the AE should be considered serious.

B. Reporting Serious Adverse Experiences

Any serious adverse experiences which occur at any time during the clinical study or within 12 weeks of the last dose of study medication, and whether or not considered related to the study medication, must be reported immediately following the procedure below.

The principal investigator will report any serious or unexpected adverse experience within 24 hours by telephone or FAX transmission to: the KEMRI Human Use Review Committee (phone 254 2 72251).
Serious and unexpected adverse experiences will be immediately reported by telephone to the USAMRMC Deputy Chief of Staff for Regulatory Compliance and Quality (301-619-2165) (non-duty hours call 301-619-2165 and send information by facsimile to 301-619-7803). A written report will follow the initial telephone call within 3 working days and information on the resolution when available. Address the written report to the U.S. Army Medical Research and Materiel Command, ATTN: MCMR-RCQ, 504 Scott Street, Fort Detrick, Maryland 21702-5012. The PI will also notify the commander of USAMRU-Kenya and the SmithKline Beecham Central Safety Group, by the most expedient means possible.

Instances of death, cancer or congenital abnormality if brought to the attention of the investigator AT ANY TIME after cessation of study medication AND considered by the investigator to be possibly related to study medication, should be reported to the site monitor, and medical monitor.

Reporting of Overdoses

Any instance of overdose (suspected or confirmed and irrespective of whether or not it involves study medication) must be fully documented as a serious adverse experience and reported as outlined above. Details of any signs or symptoms and their management should be recorded including details of any antidote(s) administered.

Reporting of pregnancy

Subjects who become pregnant during the study should discontinue the study medication immediately. Subjects should be instructed to notify the investigator if it is determined after completion of the study that they become pregnant either during the treatment phase of the study or within 12 weeks. Whenever possible a pregnancy should be followed to term, any premature terminations reported, and the status of the mother and child should be reported after delivery. In addition, any premature terminations, including elective abortions must be reported.

Breaking the study Blind

Only in the event of a serious adverse experience that the investigator feels cannot be adequately treated without knowing the identity of the study medication may the randomisation code be broken for a particular patient. Every effort must be made to contact the SB Medical Monitor prior to breaking the code. If this is not possible and the situation is an emergency the investigator may break the code and contact the Medical Monitor as soon as possible thereafter.

The SmithKline Beecham Medical Monitor for this study is:

Name:	Dr John Horton
Email	john.horton@sbcom
Telephone	+44 181-975-3638
Fax	+44-181-975-3514
Home/Emergency	+44-1462-62-4081 or +44-1462-45-1618

16. CONCOMITANT MEDICATION.

Use of antimalarial drugs are not permitted whilst the subject is in the 70 weeks of the study, or for the 2 weeks prior to starting study medication, with the exception of halofantrine used for parasite clearance.
The Principle Investigator will approve medication concurrently administered during the period of study medication administration. Trade name, dose, indication and duration of treatment will be recorded. Any indication should correspond to an adverse event in the adverse event section of the CRF.

17. LABORATORY ASSESSMENTS

A. Safety Samples

Blood samples of 2.5mls for routine hematology and clinical chemistry assessments will be taken during screening (one sample between day –21 and day 0), baseline (day 0), week 4, week 8, week 16, week 24, week 28.
The assessments to be carried out are:

- white blood cells with partial differential
- hematocrit
- hemoglobin
- platelets
- clinical chemistry:
- blood urea nitrogen (BUN)
- creatinine
- alanine transaminase (ALT)

B. Pharmacokinetic Sampling

Up to a total of 7 blood samples (2.5 ml in EDTA tubes) will be collected from patients for pharmacokinetic analysis during the double blind phase. Three post-dose samples will be collected within varying time windows: one between 6 and 12 hours following the third loading dose, one between 2 and 5 hours postdose on week 8, and one 48-120 hours (2-5 days) following the week 16 dose. Two samples will also be collected just prior to dosing of study medication on weeks 4 and 17 (trough) of the prophylactic phase of the study. Approximately one hundred subjects will be asked to consent to the Week 17 and post third loading dose samples due to the additional clinic visit involved.

Post third loading dose (6-12 h)	Wk 4* pre-dose	Wk 8* 2-5 h post dose	Wk 16* 2-5 days post-dose	Wk 17 pre-dose
X	X	X	X	X

* The safety blood draws can be coordinated with the time of PK sample draws on these visits.

The safety samples at week 4, week 8 and week 16 can be taken at the same time as the pharmacokinetic samples at these timepoints to avoid the need for 2 clinic visits. The post loading dose sample, and that at week 17 will require additional clinic visits and it is proposed that these samples will be taken in a subset of 100 patients.
Additional two samples will be collected from each patient at the time of parasitemia development and at the week 28 follow-up visit (2.5 ml into EDTA tubes).

No. Patients	Time of parasitemia	Week 28 follow-up
Any with parasitemia	X	X

See Appendix D for further details.

C. Samples for measurement of plasma malarial antibodies

A 5ml-blood sample will be taken in EDTA for measurement of malarial antibody levels at baseline (day 0), week 4, week 8, week 16, week 24, week 28. During the immunologic follow-up these samples will be taken at week 37, 50, 60 and week 70.

D. Samples for Peripheral Mononuclear Blood Cells

During the prophylaxis study a 20-ml sample will be taken from 120 subjects during screening and week 28. During the immunologic follow-up these samples will be taken at week 38 and week 70.

18. PHARMACOLOGY AND IMMUNOLOGY EVALUATION.

Peripheral blood mononuclear cells (PBMCs) and serum will be collected at the beginning and end of each follow-up period from 40 volunteers in each group. The 40 volunteers will consist of the first 40 to present on that day. Subsequent PBC collections will be limited to the same volunteers. The PBMCs and serum will be used to measure immune responses against pre-erythrocytic and erythrocytic antigens as described below.

a. Stimulation Antigens:

Cellular and humoral immune responses to the following antigens will be measured:

Pre-erythrocytic Antigens:
LSA-1: ERRAKEKLQEQRDLEQRKADTKK LSA-J
(LAKEKLQEQQSDLEQER)$_2$LAKEKLQ LSA-Rep
R32LR (recombinant)
Pf CS c-term (recombinant)
CS Th2 -PSDKHIKEYLNKIQNSLS
CS Th3 -IKPGSANKPKDELDYANDIE

Erythrocytic Antigens:
MSP-1_{19} Recombinant
MSP-1 LNDITKEYEKLLNE Peptide 1
Schizont Extract
Uninfected RBC extract

b. PBMC (Peripheral blood mononuclear cells) Isolation:

PBMCs will be isolated by standard Ficoll-hypaque gradient centrifugation (Boyum). Briefly whole blood collected in sterile heparinized tubes will be layered on a Ficoll-hypaque bed at the ratio of 3 parts Ficoll to 5 parts of whole blood. After centrifugation at 1,5000 rpm for 30 min, the mononuclear cell layer will be aspirated and washed twice with RPMI thus removing the

platelets. After counting and the determination of viability cells will be re-suspended in complete media containing at 10^6 cells/ ml in FCS containing 10% DMSO.

c. *IFN-(ELISPOT):*

The night before the assay Millipore cellulose MHA 96-well filter plates (Millipore Corp.) will be coated overnight at 4 °C with 10 µg/ml of monoclonal antibody against IFN-((Endogen, M-700A) in PBS. The next day, the plates will be washed and blocked with RPMI 1640 containing 10% fetal calf serum for 1 hour at room temperature. PBMCs will be re-suspended at a concentration of 10^6 cell/ml in complete medium (RPMI 1640 5% human AB+ serum, 5% Fetal Calf Serum) and 100 µl of cells added to each well. PHA and other stimulation antigens will be added to a concentration of 5-10 µg/ml. Schizont and uninfected RBC lysates will be added to 10% of total volume per well. The plates will be incubated at 37 °C in 5% CO_2 atmosphere for 48 hours, which is the optimal duration of incubation we have determined in preliminary studies. After incubation the contents of the wells will be discarded and the cells removed by washing with PBS 0.05% Tween 20. 100 µl of biotinylated MAb (Endogen, M-701-B), 2 µg/ml in PBS will be added to each well and the plates incubated for 2 hours at room temperature. After washing x 6 in PBS/Tween, 100 µl of streptavidin-alkaline phosphatase (0.5 ug/ml in PBS) will be added and the plates incubated for 1 hour at room temperature. After a final wash 100 µl of substrate (BCIP/NBT) will be added and the plates incubated at room temperature until spots appear (30 min to 1 hour). Stop color development by washing in tap water 5-6 times.

d. ELISA's:

Enzyme Linked Immunosorbant Assays (ELISA) using immobilized antigens R32LR and MSP-19 will be measured as previously described (Stoute). Briefly, wells of 96-well plates will be coated overnight with antigen at 0.1 µg/well. Following blocking for 1 hour with 5% casein serial dilutions of serum will be added beginning at 1:50. Total human IgG will be measured after washing using anti-human horseradish peroxidase for 1 hour followed by addition of substrate. The dilution that gives an OD_{415} = 1.0 will be reported. In some cases the antibody concentration will be measured in ug/ml by the use of a known standard if available.

19. PARASITE DRUG SENSITIVITY

Subjects with confirmed patent parasitemia will have blood collected for storage of parasites, malaria parasite culture and in vitro drug sensitivity by standard techniques. This will be a single 1ml sample and will be stored frozen at –20 degrees °C or lower.

These data will not form part of the primary efficacy analysis of the study.

20. WITHDRAWAL FROM THE STUDY

A. Reasons For Withdrawal

Subjects will be allowed to decline participation at any point in the study without prejudice.

Subjects may be withdrawn from the study without their consent if:

- they develop health conditions that would make continued participation in this study dangerous.
- other conditions occur that would make participation or continued drug administration detrimental to their health. These include physical or laboratory evidence of intolerance.
- subject misses two consecutive dose of study medication
- volunteer becomes pregnant.

B. Safety follow up visit after withdrawal

Subjects leaving the study early, whatever the reason, will be asked to return to the clinic for a safety follow-up visit 4 weeks later. This will involve taking blood samples a and a physical examination.

21. DATA MANAGEMENT AND STATISTICAL ANALYSIS

A. Target Sample Size

The protective efficacy of tafenoquine will be considered to be adequate if the lower limit of the 95% confidence interval is no lower than 70% (see section 19E)
123 evaluable subjects will be sufficient to give at least 90% power to reject Protective Efficacies of less than 70% using a two sided 95% confidence interval, given a placebo attack rate of 70% and a tafenoquine attack rate of 3.5% (estimated PE = 95%). This assumes a randomization ration of 1:1:1 for tafenoquine: placebo: mefloquine. Given a 15% attrition rate through the study 147 subjects would have to be randomized; 49 each to tafenoquine, mefloquine and placebo.

Due to the high placebo attack rate, and therefore low sample size, there is concern that accurate estimates for incidence rates of adverse events (AEs) will not be attainable; indeed with as few as 49 subjects randomized per treatment group certain common adverse experiences may not be observed. For this reason it is recommended that at least 100 subjects be randomized per treatment group in a 1:1:1 fashion so that AE incidence rates can be estimated using 95% confidence intervals.

With 100 randomized subjects per arm on tafenoquine, placebo and mefloquine the following incidence rates for AEs can be estimated using a 95% confidence interval with the following accuracy:

AE incidence Rate	Accuracy of 95% CI
5%	+/- 4.3 %

10%	+/- 5.9 %

Given 100 subjects randomized per group (85 evaluable) the study will have > 99% power to reject PEs of less than 70% under the above assumptions.

A sensitivity analysis of the power calculation shows that the study also has 90% power to reject the following PEs under various assumptions of placebo and tafenoquine attack rates:

Placebo Attack Rate	Tafenoquine Attack Rate	PE	Rule Out Values of PE less than:
70%	3.5%	95%	80%
63%	3.5%	94.4%	77%
56%	3.5%	93.75%	74%
70%	7%	90%	71%

Although no formal statistical comparison will be made between the PE for tafenoquine and that for mefloquine it is anticipated that the PEs will be within 5% of each other.

B. Method of Randomization

All subjects meeting the study criteria will be randomly assigned, in equal proportions, and in a double-blind, double dummy fashion, to receive one of the three treatment regimens according to a pre-determined code provided by SmithKline Beecham. The block size will remain confidential.

C. Breaking the Study Blind

A SB statistician will be responsible for breaking the blind at a time point after collection, databasing and cleaning of all data and after the assessment of the Per Protocol population.

D. Planned Efficacy Evaluations

Primary Efficacy Variable

Prophylactic outcome at study endpoint is the primary efficacy variable. The prophylactic outcome for each subject will be evaluated as follows using all available blood smears up to and including seven days after last dose of prophylactic study medication:

Prophylactic Failure: First positive parasitemia (as defined in section 13A) during prophylactic study drug administration up until last available blood smear up to and including seven days after last dose of prophylactic study medication.

Prophylactic Success: No parasitemia (as defined in section 13A) during prophylactic study drug administration up until last available blood smear up to and including seven days after last dose

of prophylactic study medication.

Secondary Efficacy Variables

The secondary efficacy variables will include number of subjects with confirmed parasitemia, number of subjects with symptomatic versus asymptomatic parasitemia and time to first positive parasitemia.

Subject Populations

Two populations are defined for the analysis of clinical efficacy data.

Intent-To-Treat (ITT) : All randomized subjects who took at least one dose of study medication (tafenoquine/mefloquine/placebo) and attended at least one follow-up efficacy assessment where assessment of a blood smear took place.

Per Protocol (PP): All randomized subjects who satisfied the inclusion/exclusion and subsequently adhered to the protocol. This is a subset of the ITT population.

Subjects who receive the wrong coded study medication will be analyzed according to the treatment they received.

Subjects will only be excluded from the PP population from the time that the violation occurs. If a subject is a prophylactic failure and then subsequently violates the protocol they will not be excluded from the PP population since they have already satisfied the criteria for failure prior to violation of the protocol.

The ITT population is used to address the question "How does the medication work in subjects who are prescribed the drug and who take at least one dose of the drug?" Subjects will be excluded from the ITT population if there is documented evidence that they have taken no study medication. The PP population is used to address the question "How does the medication work in subjects who are prescribed the medication and who take the medication as prescribed?". Thus, the ITT population may be thought of as assessing the drug's effectiveness and safety, whilst the PP population may be thought of as assessing the drug's efficacy and safety.

In trials designed to show the superiority of a new drug over placebo/ comparator, the ITT population is thought of as a conservative approach to the statistical analysis. For this reason, the ITT population is the population of primary interest here, whilst the PP population will be used to confirm the results.

All decisions on eligibility for inclusion in these populations will be made prior to code- break or any data evaluation.

E. Methods Of Analysis

The baseline demographic characteristics will be summarized to assess the comparability of the treatment groups at baseline. If large differences are observed between treatment groups for baseline or demographic characteristics then the effect of these will be investigated. No formal hypothesis testing or interval estimation will be applied to baseline or demographic characteristics.

The primary efficacy analysis will be based on a calculation of the protective efficacy of tafenoquine, for the intent-to treat population. Protective efficacy is defined as

$$PE(\%) = \frac{I_{placebo} - I_{drug}}{I_{placebo}} * 100$$

where $I_{placebo}$ = number of prophylactic failures / number of subjects at risk in the placebo group and I_{drug} = number of prophylactic failures / number of subjects at risk in the drug group. Corresponding 95% confidence limits for the protective efficacy will be calculated based on the method of Koopman. A conclusion of adequate efficacy of tafenoquine will be drawn if the lower limit of this 95% confidence interval is no lower than 70%. The protective efficacy of mefloquine will also be calculated and presented along with that of tafenoquine, however no formal statistical tests of comparison will be used as the study is not powered to detect differences between tafenoquine and mefloquine.

As confirmation of the primary analysis, the protective efficacy and 95% confidence interval will be calculated for the per protocol population. An additional analysis, to assess the robustness of the calculations of protective efficacy to dropouts, will be performed. This will involve calculating protective efficacy based on the incidence density of parasitemia in each treatment group, which takes into account the length of time each subject was observed in the study.

The secondary efficacy variables will be summarized and analyzed. A Kaplan-Meier curve will be produced showing the cumulative probability of remaining malaria free. If appropriate, further survival analysis will be performed.

F. Planned Safety Evaluations

There is one subject population defined for safety analyses in this study.

Safety: All subjects who took at least one dose of study medication (tafenoquine/ placebo/ mefloquine).

Subjects who receive the wrong study medication will be analyzed according to the medication they received.

Adverse Experience (AE) reporting will be performed using the World Health Organization (WHO) coding system. Each AE coded using the WHO system can be associated with up to

three body systems (e.g. Headache is associated with Central and Peripheral Nervous System and Body as a Whole General). An AE will be associated with the primary body system only.

Counting will be based on the number of subjects not the number of AEs, e.g. if a subject reports the same AE on three occasions within a time interval, that AE will only be counted once. Subjects reporting more than one AE in a body system will only be counted once in the body system total.

G. Pharmacokinetic Analysis

Plasma tafenoquine concentration-time data will be displayed in tables and/or graphs. Population PK and/or Bayesian estimation methods will be performed using software such as NONMEM or other currently acceptable methods to assess the pharmacokinetics of tafenoquine, if data are appropriate. To support the population PK analysis, the data from this study may be combined with data from other studies. Mean population parameters will be assessed taking into account demographic variables (such as age, weight, gender, race), concomitant medications and/or co-morbid diseases. If data permit, the relationship between tafenoquine concentrations and efficacy parameters and selected adverse events will be explored.

Pharmacokinetic analysis will be the responsibility of the Department of Pharmacokinetics, DMPK, SmithKline Beecham. All pharmacokinetic data will be stored in the Archives, SmithKline Beecham, Research and Development. The pharmacokinetic analysis may be reported separately.

H. Analysis of Immunologic Parameters:

The susceptibility to malaria of the three groups beginning 9 weeks after the end of prophylaxis will be compared by using Log-rank analysis of malaria free survival. Continuous numeric data of immune responses from the different groups prior to prophylaxis, at end of prophylaxis and after the third follow-up period will be compared using nonparametric methods of analysis of variance.

22. RECORDING AND DISSEMINATION OF DATA

A. Recording of data

Subject data up to and including week 28 will be recorded into Case Report Forms (CRFs). A copy of the CRFs and all original data will be kept on file at USAMRU-K (or a designated archive center) as required by US Army regulations.

This data will then be entered into a suitable database (to be decided) and will be cleaned at the study site. To assist this process SmithKline Beecham will provide the Data Checks and Field Code names to USAMRU and will approve the Data Entry Package and Data Validation Specification.

Access to the database will be limited and will be password controlled, and any changes to the data will be logged in an audit trail that will be retained as study documentation.

Prior to export of the trial data to SmithKline Beecham, a package of test data will be transferred to ensure that the transfer systems are adequate. The study will subsequently be analyzed and reported by SmithKline Beecham.

B. Dissemination of data

It is expected that these data will be reported in both scientific journals and scientific meetings, and be submitted to governmental medication regulatory authorities such as the U.S. Food and Drug Administration (FDA) for review. Confidentiality of subjects will be maintained. Volunteers will be informed in general terms of the results as soon as practical. All publications resulting from this study will be approved by KEMRI, the US Army Medical Research and Materiel Command, and SmithKline Beecham.

23. ADMINISTRATIVE MATTERS

To comply with Good Clinical Practice Guidelines, important administrative obligations relating to investigator responsibilities, monitoring, archiving data, audits, confidentiality, and publications must be fulfilled (see Appendix C).

24. TIME FRAME

A. Screening and selection of volunteers, April 2000.
B. Drug administration April to October 2000.
C. Data analysis, Nov-Dec 2000.
D. Preliminary Report, December 2000.

25. ETHICAL CONSIDERATIONS

All subjects will receive a medical examination and will be cleared of malaria parasitemia at the start of the study. They will also be treated for medical illnesses developing during the study. These benefits will apply to all subjects whether or not they are randomized to placebo or medication groups. Because symptomatic malaria is so common even among the semi-immune population, this should be a significant benefit, as the overall risk becoming severely ill with malaria should be much less for anyone involved in the study. Persons on placebo will benefit, as all volunteers will receive active antimalarial medication for at least part of the trial.

Currently prophylaxis is not given to adult Kenyans permanently residing in malaria endemic areas except pregnant women. With close follow-up and rapid treatment, it would be possible and ethical to enroll a placebo group in order to calculate the absolute as well as comparative efficacy to other antimalarial drugs.

It is possible that increased susceptibility to malaria may be observed following prophylaxis with

either tafenoquine and/or mefloquine. However, any risk will be off-set by the close follow-up that will be provided during the 70 weeks after randomization into the study consisting of active and passive case detection even after the prophylaxis period has ended.

26. REFERENCES

See Appendix D

27. BUDGET

The U. S. Army Medical Research and Materiel Command, through the US Army Medical Materiel Development Activity, will fund the study. Figures below are in $ US with an approximate conversion rate of 60 Kenyan shillings = 1 $ US.

Personnel/Salaries	30000
TDY personnel	30000
Field Support	40000
Volunteer Benefits	90000
Clinical Lab support	50000
Parasitology Lab support	10000
total	250000 $ US or about 19 million Kenyan shillings

28. RESOURCES AND COORDINATION

Laboratory: USAMRU-Kenya has an established laboratory both in Nairobi and Ndori capable of handling all the tests required for a chemosuppression study. HPLC analyses for mefloquine and tafenoquine concentrations are not available in Kenya and will be sent to contracting laboratories in the USA. USAMRU-Kenya has established a quality assurance (QA) program that will allow its laboratory to comply with Good Clinical Practice (GCP) standards of the US FDA.

Logistics: Both USAMRU-Kenya and KEMRI have extensive practical experience with the execution of chemotherapy and prophylaxis trials. Local employees, lab facilities, housing and transport are in place. The local population knows and trusts the study teams on the basis of their past successful study execution.

Project Management: USAMMDA and SmithKline Beecham will provide project management, monitoring, and external quality assurance audits. For the purposes of this protocol they are referred to as, 'the sponsor.'

29. IND INFORMATION

Tafenoquine: IND Number: 38503

30. MODIFICATION OF PROTOCOL

Any modifications will be approved by the appropriate Ethics Committees/ Institutional Review

Boards prior to implementation.

31. COMPENSATION TO VOLUNTEERS

In accordance with Kenyan practice, there will be no direct monetary compensation to the subjects.

32. STATEMENT REGARDING POTENTIAL CONFLICT OF FINANCIAL INTEREST

The Principal Investigators, Medical Monitor and the Co-Investigators have no financial interest in the drugs used in this study. Dr. John Horton is employed by SmithKline Beecham. As stated above, this study is funded by the USAMRMC and not by SmithKline Beecham.

33. ROLE OF INVESTIGATORS

Principal Investigator:
Jose Stoute, LTC, MC, US Army

Investigators:
US Army Medical Research Unit-Kenya, Nairobi, Kenya
Dr. G. Dennis Shanks M.D., MPH, COL, MC Commander AFRIMS/Investigator, USAMRU-K: Protocol design, analysis of data

Jon Davis, CPT, MS, Clinical laboratory

WRAIR
Doug Tang, Ph.D., Biostatistician

SmithKline Beecham
Dr John Horton MD, Analysis and reporting of data, preparation of clinical report.

USAMMDA
William Prescott, Ph.D. LTC, MS project design, management, auditing and review

Medical monitor
Dr Ambrose

34. SPECIAL FUNDING IMPLICATIONS

None.

35. SIGNATURE OF PRINCIPAL INVESTIGATOR:

I, the undersigned, have reviewed this protocol, including Appendices, and I will conduct this

clinical study as described and will adhere to the Ethical and Regulatory considerations stated. I have read and understood the Investigator Brochure.

Investigator Date
Jose Stoute, LTC, MC, US Army
US Army Medical Research Unit - Kenya

APPENDIX A – Declaration of Helsinki

World Medical Association
DECLARATION OF HELSINKI
Recommendations Guiding Physicians
in Biomedical Research Involving Human Subjects

Adopted by the 18th World Medical Assembly Helsinki, Finland, June 1964
and amended by the
29th World Medical Assembly Tokyo, Japan, October 1975
35th World Medical Assembly Venice, Italy, October 1983
41st World Medical Assembly Hong Kong, September 1989
and the
48th General Assembly Somerset West, Republic of South Africa, October 1996.

INTRODUCTION

It is the mission of the physician to safeguard the health of the people. His or her knowledge and conscience are dedicated to the fulfilment of this mission.

The Declaration of Geneva of the World Medical Association binds the physician with the words, "The health of my patient will be my first consideration," and the International Code of Medical Ethics declares that, "A physician shall act only in the patient's interest when providing medical care which might have the effect of weakening the physical and mental condition of the patient."

The purpose of biomedical research involving human subjects must be to improve diagnostic, therapeutic and prophylactic procedures and the understanding of the etiology and pathogenesis of disease.

In current medical practice most diagnostic, therapeutic or prophylactic procedures involve hazards. This applies especially to biomedical research.

Medical progress is based on research which ultimately must rest in part on experimentation involving human subjects.

In the field of biomedical research a fundamental distinction must be recognised between medical research in which the aim is essentially diagnostic or therapeutic for a patient, and medical research, the essential object of which is purely scientific and without implying direct diagnostic or therapeutic value to the person subjected to the research.

Special caution must be exercised in the conduct of research which may affect the environment, and the welfare of animals used for research must be respected.

Because it is essential that the results of laboratory experiments be applied to human beings to further scientific knowledge and to help suffering humanity, the World Medical Association has prepared the following recommendations as a guide to every physician in biomedical research involving human subjects. They should be kept under review in the future. It must be stressed

that the standards as drafted are only a guide to physicians all over the world. Physicians are not relieved from criminal, civil and ethical responsibilities under the laws of their own countries.

I. BASIC PRINCIPLES

1. Biomedical research involving human subjects must conform to generally accepted scientific principles and should be based on adequately performed laboratory and animal experimentation and on a thorough knowledge of the scientific literature.
2. The design and performance of each experimental procedure involving human subjects should be clearly formulated in an experimental protocol which should be transmitted for consideration, comment and guidance to a specially appointed committee independent of the investigator and the sponsor provided that this independent committee is in conformity with the laws and regulations of the country in which the research experiment is performed.
3. Biomedical research involving human subjects should be conducted only by scientifically qualified persons and under the supervision of a clinically competent medical person. The responsibility for the human subject must always rest with a medically qualified person and never rest on the subject of the research, even though the subject has given his or her consent.
4. Biomedical research involving human subjects cannot legitimately be carried out unless the importance of the objective is in proportion to the inherent risk to the subject.
5. Every biomedical research project involving human subjects should be preceded by careful assessment of predictable risks in comparison with foreseeable benefits to the subject or to others. Concern for the interests of the subject must always prevail over the interest of science and society.
6. The right of the research subject to safeguard his or her integrity must always be respected. Every precaution should be taken to respect the privacy of the subject and to minimize the impact of the study on the subject's physical and mental integrity and on the personality of the subject.
7. Physicians should abstain from engaging in research projects involving human subjects unless they are satisfied that the hazards involved are believed to be predictable. Physicians should cease any investigation if the hazards are found to outweigh the potential benefits.
8. In publication of the results of his or her research, the physician is obliged to preserve the accuracy of the results. Reports of experimentation not in accordance with the principles laid down in this Declaration should not be accepted for publication.
9. In any research on human beings, each potential subject must be adequately informed of the aims, methods, anticipated benefits and potential hazards of the study and the discomfort it may entail. He or she should be informed that he or she is at liberty to abstain from participation in the study and that he or she is free to withdraw his or her consent to participation at any time. The physician should then obtain the subject's freely-given informed consent, preferably in writing.
10. When obtaining informed consent for the research project the physician should be particularly cautious if the subject is in a dependent relationship to him or her or may consent under duress. In that case the informed consent should be obtained by a physician who is not engaged in the investigation and who is completely independent of this official relationship.

11. In case of legal incompetence, informed consent should be obtained from the legal guardian in accordance with national legislation. Where physical or mental incapacity make it impossible to obtain informed consent, or when the subject is a minor, permission from the responsible relative replaces that of the subject in accordance with national legislation. Whenever the minor child is in fact able to give a consent, the minor's consent must be obtained in addition to the consent of the minor's legal guardian.
12. The research protocol should always contain a statement of the ethical considerations involved and should indicate that the principles enunciated in the present Declaration are complied with.

II. MEDICAL RESEARCH COMBINED WITH PROFESSIONAL CARE (Clinical Research)

1. In the treatment of the sick person, the physician must be free to use a new diagnostic and therapeutic measure, if in his or her judgement it offers hope of saving life, re-establishing health or alleviating suffering.
2. The potential benefits, hazards and discomfort of a new method should be weighed against the advantages of the best current diagnostic and therapeutic methods.
3. In any medical study, every patient - including those of a control group, if any – should be assured of the best-proven diagnostic and therapeutic method. This does not exclude the use of inert placebo in studies where no proven diagnostic or therapeutic method exists.
4. The refusal of the patient to participate in a study must never interfere with the physician-patient relationship.
5. If the physician considers it essential not to obtain informed consent, the specific reasons for this proposal should be stated in the experimental protocol for transmission the independent committee (see I,2).
6. The physician can combine medical research with professional care, the objective being the acquisition of new medical knowledge, only to the extent that medical research is justified by its potential diagnostic or therapeutic value for the patient.

III. NON-THERAPEUTIC BIOMEDICAL RESEARCH INVOLVING HUMAN SUBJECTS (Non-Clinical Biomedical Research)

1. In the purely scientific application of medical research carried out on a human being, it is the duty of the physician to remain the protector of the life and health of that person on whom biomedical research is being carried out.
2. The subjects should be volunteers -- either healthy persons or volunteers for whom the experimental design is not related to the patient's illness.
3. The investigator of the investigation team should discontinue the research if in his/her or their judgement it may, if continued, be harmful to the individual.
4. In research on man, the interest of science and society should never take precedence over considerations related to the well being of the subject.

APPENDIX B - Informed Consent Form

A Randomized, Double Blind, Placebo Controlled Evaluation of Weekly Tafenoquine (WR 238605/SB252263) Compared to Mefloquine for Chemosuppression of *Plasmodium falciparum* in Western Kenya

You must understand the information in this document before you agree to participate in the study. You will be given a copy of this document to take away with you and keep.

Purpose of study

You are being asked to participate in a research study (clinical trial) of a new anti-malarial drug. This drug is called tafenoquine and has been proven to be very effective (90-95%) at preventing malaria in smaller clinical trials and at various different doses. It is not yet on the market since it is new and being investigated for safety and effectiveness, but Regulatory Authorities (Food and Drug Administration (FDA) in USA) have approved its use in this study.

The aim of the study is to confirm that the dose chosen (200mg) is as effective as we think it is in larger studies, and to compare it to a well-established drug on the market called mefloquine (250mg). Three hundred volunteers will be enrolled and receive study medication. Some of the volunteers (a third) will receive an inactive treatment called a placebo that will look exactly like the active drugs. This is to prove that malaria is in fact present in the area and that the active drugs are working.
You will have a one in three chance of taking either mefloquine, tafenoquine or the inactive placebo. Neither you nor you doctor will know what treatment you have been given until the study is finished.
Before you start taking any treatment you will also be given a 3-day treatment course of a drug called halofantrine. This is similar to mefloquine and is also marketed in Kenya. It has been shown to kill any malarial parasites that are in your liver, even if they cannot be seen in your blood. This is to make sure that you are well before the treatment is started.

Duration of study.

The study comprises a screening phase of about three weeks to see if you are fit and well, to check that you don't have malaria, and that you are suitable for the study. After this you will be given 3 days oral treatment with a drug called halofantrine to clear any malaria parasites that are already in your body and then asked to take your study (malaria prevention) medication for 24 weeks. At the end of your drug treatment you will have a safety follow-up as part of the drug study for a further 4 weeks.
At the end of this 28 week period, although you will no longer be taking any drugs, you will be followed-up by health workers to see if the treatment you have received has effected your resistance to malaria. Health workers will visit you once a week for 9 weeks to check that you are well and to see if you develop malaria. After this you will be given another treatment course

of halofantrine to kill any malaria parasites that may be in your liver and followed up for a further 33 weeks. The total duration of the study is therefore 70 weeks and you will be required to remain in the area and not take any other anti-malarial treatment for this time. During the study you will be required to come to the clinic approximately 10 times during the first 6 months of the study, and approximately a further 7 times for the remainder of the study. On any occasion during this period you will have free access to the clinic and are able to come in if you feel unwell.

Risks/Benefits of the study

If you decide to participate in the study, there is a 1 in 3 chance that you will be in the placebo group and therefore receive no active malaria protection
There is a risk of side effects with all types of drugs. The most common side effects of mefloquine are dizziness, poor sleep, strange dreams, vomiting, nausea; these side effects affect only about 1 in 5 people.
Tafenoquine can adversely effect the blood of people who have a type of blood called G6PD deficiency where it causes the blood to break up and may lead to the need for a blood transfusion. About 10% of the population have this condition. You will be tested for this type of blood and will not be able to participate in the study if you fall into this category.
Other side effects thought to be due to tafenoquine include nausea, vomiting and diarrhea in about 1 in 5 people, and occasionally headache, lightheadedness or unusual tastes in the mouth. It is possible that other side effects could be observed which are currently unknown. Should you have any side effects the severity will be evaluated and appropriate medical treatment will be given to you. The study medication may be stopped if necessary to treat any side-effects. You will be told about any significant findings that may affect your decision to continue to take part in the study.
It is possible that you may develop malaria parasites in your blood, even if you don't have symptoms. If this happens it means that the medication hasn't worked and your drug treatment will stop. Your doctor will decide whether or not you need treatment and decide on the best therapy for you. You will have your finger prick smear taken the following week and you will still be seen weekly by the health workers. If you have malaria symptoms your doctor will treat you as he feels is best.
It is possible that after stopping the medication you may be more likely to get malaria. However, you will continue to be closely monitored for malaria for almost one year after stopping and will continue to have free access to treatment at the Walter Reed Kombewa Clinic.
The benefits of the study are that you will be closely monitored for malaria, and given treatment if you need it. Complete medical care for all/any illnesses related to the study will be provided. The facilities of the Kombewa Health Clinic will be additionally available for general health care during the study period. The study results may help gain a better understanding of malaria.

Study design

The study will be in three phases; screening, prevention of malaria, and then follow-up. 300

volunteers will be selected for the study on a first come, first served basis.

Screening: Healthy adults from 18 to 65 years of age who will be available every week for up to 70 weeks may volunteer to enter the study, and will be screened over a period of about 3

weeks starting in April 2000. In order to be accepted for this research study, it is necessary to pass a physical examination and have some laboratory tests done on a sample of your blood drawn from your arm with a needle. The laboratory tests are done to make sure that your blood, liver, heart and kidneys are working properly and in order to minimize any risk of adverse side effects. You will be given a test called an EKG which looks to see if the heart is working properly; this involves lying down and having pads attached to your chest with wires for a few minutes. A blood smear will be taken to see if you have malaria parasites in your blood. This involves a tiny needle prick to the end of one of your fingers and a couple of drops of blood squeezed onto a glass slide.

If you are a female you will be tested to see if you are pregnant by urine or a blood test. If you are pregnant or if you are breast feeding you are not able to join the study. You will also be asked not to become pregnant up until 15 weeks after stopping study drug i.e. if you complete the study, until 39 weeks after starting treatment.

Any volunteers who take an antimalarial drug other than halofantrine up to two weeks before starting the study may also not be eligible

Your blood will not be measured for evidence of the HIV or AIDS virus since this is a malaria study and not one concerning AIDS. Persons found not to qualify for the study will be given a letter explaining this and invited to return to the clinic for an explanation from a doctor. Those then desiring a medical referral to the Kenyan health system will be given a written letter of referral to the nearest government hospital.

Prevention: Eligible subjects will then start the 24 week drug treatment phase after clearing any malaria parasites in the body with a three day treatment course of halofantrine. Following this, you will be allocated to receive either mefloquine, tafenoquine (the trial drug) or placebo. It will not be possible to tell what you are taking.

You will take your assigned study medication for 3 consecutive days to start with to reach effective levels quickly, which will be one capsule and one tablet per day taken orally. Every dose of trial medication will be taken with a small meal to help absorption.

Thereafter each week for a further 23 weeks, medication will be given once a week by health workers i.e. one tablet and one capsule taken with food. Any person developing malaria in their blood during the study will receive a known treatment when he feels ill and will be treated until he is free of malaria. Volunteers will continue to be followed up until the end of the 70 week period even if they develop malaria and are given alternative treatment. Approximately 100 subjects will have an additional blood sample taken.

Follow-up

Health workers will give volunteers a further treatment course (one tablet once a day for 3 days) of halofantrine during week 37, and then you will be followed up on a weekly basis until week 70. A finger prick smear will be done every week to look for malaria parasites. Blood samples will be taken on 4 occasions to see if you have been exposed to the malaria parasite at weeks 37, 50, 60 and week 70.

Study Tests/what is involved

You will be asked to give a finger prick of blood for malaria slides on a weekly basis for the duration of the study. You will also be asked to give a blood sample at screening and a maximum of 8 times during the prevention phase of the study for routine blood tests and drug measurement purposes. You will be asked to attend the clinic when the blood samples are to be taken i.e. at start of taking medication, week 4, 8, 16, 24 and 28. The purpose of taking these blood samples is: 1) to make sure that your blood, liver, heart and kidneys are working properly in order to minimize any risk of adverse side effects: 2) measure the amount of drug in your blood; and3) measure parasite antibodies. During the follow-up phase blood samples will be taken on a further 4 occasions, at weeks 37, 50, 60 and week 70 to see if you have been exposed to the malaria parasite (measure antibody levels). An additional sample may be requested if you develop malaria. There may be some discomfort and sometimes bruising occurs when blood is taken from a vein in the arm. Blood drawn from women during the study will be also be tested for pregnancy and pregnant women will no longer be allowed in the study.

During the prevention phase, approximately 100 volunteers will be asked to attend the clinic on additional days, on Day 3 and during week 17. This is to give an additional blood sample to look at drug levels at these time-points.

During the follow-up phase, approximately 120 subjects will be asked to give an additional blood sample at baseline and at week 28, 38, and 70 to look at how well the body's natural defences are working against the malaria parasite. For all but the week 38 sample, samples will be taken at the same time as the routine blood samples and no extra visits are needed.

Subjects for the additional blood draws will be asked to participate at random.

The maximum total amount of blood taken during the study is about 173mls (a cup full) over a period of 70 weeks.

On all other occasions trained health workers who will give you your study drug will visit you once a week. They will need to collect information on how you are so will ask you 'have you felt unwell at all since my last visit'.

If you feel unwell you can visit the clinic for treatment at any time.

Summary: Someone who volunteers for this research study should expect the following:

a. a physical exam and laboratory tests to show that he or she can safely receive the medicine

b. daily oral medicine given by a doctor or a field worker for the first three days of the study

c. weekly oral medicine for up to 24 weeks given by a field worker

d. answer questions weekly about any health problems that might have happened

e. report to the research clinic if any serious health problem develops while taking medicine

f. give blood from the arm 15 times during the study

g. have a finger prick done weekly in order to determine if malaria is in your blood

h. be followed up by health workers for 70 weeks

PRECAUTIONS

Chloroquine and other medicines can also affect malaria. If you join this study, you must agree not to take chloroquine or other anti-malarial medicines, unless the study doctors prescribe them. You should remain in the area during the entire period of the study.

 volunteer initials_______ witness initials ____________

PREGNANCY

Do not become pregnant.

All women volunteering for this study will have a blood pregnancy test done within 2 days before receiving the treatment drug, halofantrine, and repeated before you start study drug. Only those with negative tests will be able to participate. This is because it is possible, but unlikely, that the drugs used in this study could harm a developing baby. The pregnancy test will be repeated monthly during the drug administration period, by blood test in the clinic or urine test on other occasions. Any women who become pregnant will be not be allowed to continue taking study medication.

You should avoid becoming pregnant up until 2 weeks after the second treatment course of halofantrine i.e. 39 weeks after entering the study. Before the week 37 treatment course of halofantrine, a further pregnancy test will be performed. To avoid becoming preganant, you should either abstain from sexual relations or practice a method of birth control. Except for surgical removal of the uterus, birth control methods such as the use of condoms, a diaphram or cervical cap, birth control pills, IUD, or sperm killing products are not totally effective in preventing pregnancy.

CONFIDENTIALITY

All study documents will be given to the company manufacturing the medications but only in the form of anonymous reports; a number will be used to identify you. Your name will not appear on any publication or report from this study. Direct access to your medical records may be required by the drug manufacturing company representatives, Kenyan or United States government medical research or drug regulatory authorities. Representatives from the US Army Medical Research and Materiel Command may also inspect study records.

It is the policy of the U.S Army Medical and Material Command that data sheets are to be completed on all volunteers participating in research for entry into the Command's Volunteer Registry Database. This is a confidential database and the data entered includes name, address, social security number and details of the clinical trial you are involved in. This information is needed to answer questions concerning subjects participating in research sponsored by USAMRMC, and to ensure you can be contacted if there is new information on the study drug. The information is stored for 75 years.

COMPENSATION

You are authorized all necessary medical care for injury or disease which is the proximate result of your participation in this research study.

If you feel that you have been injured as a direct result of participating in this study you should contact Dr Stoute who will advise you what to do.

Other than the medical care provided, there is no other payment available for your participation in this research study; however this is not a waiver or release of your legal rights.

YOUR RIGHTS

If during the course of this study, you have questions concerning the nature of the research or you believe you have sustained a research-related injury, you should contact Dr. Stout or the doctor on duty at the study clinic. The Chairman of the Kenya National Ethical Review Committee, c/o Kenya Medical Research Institute, P.O. Box 54840, Nairobi, Kenya, tel. 02-

72251 can also be contacted concerning your rights as a research volunteer.

WITHDRAWAL FROM STUDY

You will be taken out of the study and receive no more medication without your consent in the following circumstances. 1) If you develop health conditions that would make continued participation in this study dangerous. 2) If you develop another illness or condition that would make continuing in the study bad for your health. These include physical or laboratory evidence of intolerance to the drug. 3) You miss more than one dose of study medication
4) You become pregnant.
If you are withdrawn from the study you will still be eligible for treatment at the clinic.
If you leave the study early, whatever the reason, you will be asked to return to the clinic for a safety follow-up visit 4 weeks later. This will involve taking blood samples as at other clinic visits, and a physical examination.

VOLUNTARY PARTICIPATION

Your decision to participate in the study is entirely voluntary and refusal to participate will involve no penalty or loss of benefits to which you otherwise would be entitled. In addition, you may withdraw from the study at any time without penalty or loss of benefits. If you choose to leave the study, you should return to the study doctor to inform him.
The doctor has the right to withdraw you from the study at any time should he feel it appropriate to do so.

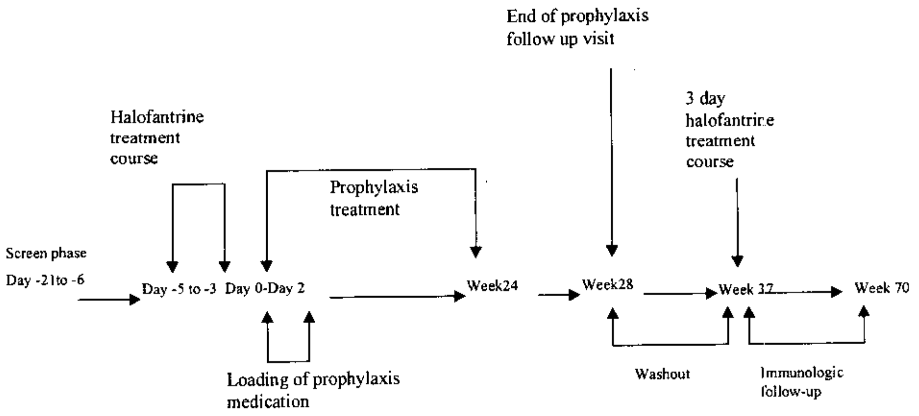

 volunteer initials_______ witness initials _______________

WRITTEN CONSENT

A Randomized, Double Blind, Placebo Controlled Evaluation of Weekly Tafenoquine (WR 238605/SB252263) Compared to Mefloquine for Chemosuppression of *Plasmodium falciparum* in Western Kenya

Volunteer's Printed Name____________________________________

I have carefully read the information provided to about the about study and understand what is involved. All questions raised by me have been answered to my satisfaction. I have been given a copy of the consent form. I understand that I am free to withdraw from the study at any time without it incurring any disadvantage to me in the future. I understand that identifying information will be used for location purposes. Information will be used to document the study and may be furnished to Federal and local agencies.
I consent to my participation in the study:

VOLUNTEER'S SIGNATURE__________________**DATE** ________________

Locating information (village) ______________________________

In addition, I am willing to attend for clinic visits to give a blood sample after the third loading dose and at week 17.
VOLUNTEER'S SIGNATURE__________________**DATE** ________________

In addition, I am willing to give a blood sample at baseline, week 38 and week 70 for assessment of malaria resistance
VOLUNTEER'S SIGNATURE___________________**DATE** _______________

STUDY DOCTOR'S SIGNATURE__________________________________

Printed name __________________________________

DATE ________________

WITNESS'S SIGNATURE__________________________________

Printed name__________________________________

DATE ________________

A Randomized, Double Blind, Placebo Controlled Evaluation of Weekly Tafenoquine (WR 238605/SB252263) Compared to Mefloquine for Chemosuppression of *Plasmodium falciparum* in Western Kenya

WITNESSED VERBAL CONSENT

I have been present during the Informed Consent process for ____________________ to participate in the above study. The doctor has explained the study procedures to him/her and the implications of participating are understood. All questions raised by him/her have been answered to his/her satisfaction and he/she has been given a copy of this consent form. The volunteer understands that he/she is free to withdraw from the study at any time without it incurring any disadvantage to him/her in the future.

WITNESS'S SIGNATURE__________________________

WITNESS'S Printed name__________________________

DATE ____________________

Thumbprint of **subject** indicating illiteracy

In addition, he /she is willing to attend for clinic visits to give a blood sample after the third loading dose and at week 17 .

WITNESS'S SIGNATURE____________________**DATE** ____________________

In addition, he/she is willing to give a blood sample at baseline, week 38 and week 70 for assessment of malaria resistance

WITNESS'S SIGNATURE______________________**DATE** __________________

I have carefully explained the study procedures to the volunteer and he/she understands all the points. All questions raised by him/her have been answered to his/her satisfaction. I have provided a copy of the consent form to the volunteer. The volunteer understands that he/she is free to withdraw from the study at any time without it incurring any disadvantage to him/her in the future.

STUDY DOCTOR'S SIGNATURE____

Printed name____

DATE ____________________

Appendix C – Administrative Matters

Administrative matters

I Responsibilities of the Investigator

To ensure that he/she has sufficient time to conduct and complete the study and has adequate staff and appropriate facilities which are available for the duration of the study and to ensure that other studies do not divert essential subjects or facilities away from the study at hand.
To submit an up-to-date curriculum vitae and other credentials (e.g., medical license number) to the sponsor and where required, to relevant authorities.
To acquire the normal ranges for laboratory tests performed locally and, if required by local regulations, obtain the Laboratory License or Certification.
To prepare and maintain adequate case histories designed to record observations and other data pertinent to the study.

II Protocol Amendments

No changes to the study protocol will be allowed unless discussed in detail with the sponsor and filed as an amendment/modification to this protocol.
Any amendment/modification to the protocol will be adhered to by the participating centre (or all participating centres) and will apply to all subjects following approval as appropriate by the Ethical Review Committee or Institutional Review Board.

III Case Report Form Instructions

Prior to Screening the first potential participant, the investigator will provide a list showing the signature and handwritten initials of all individuals authorised to make or change entries on case report forms (CRFs). If the authorised individuals should change during the study, the investigator is to inform SB.
CRFs will be produced with the assistance of SB for recording all data. It is the responsibility of the investigator and co-investigators to ensure that CRFs (and patient diary cards) are legible and completely filled in with a black ink ballpoint pen. Enter the patient's identification (2-3 alphabetic letters representing the patient's initials or first 2-3 letters of patient's first or last name), the patient identification number (PID) if not already pre-printed and the visit date, on the CRF Page Headers as required.
Errors must be corrected by drawing a single line through the incorrect entry and writing in the new value/data positioned as close to the original as possible. The correction must then be initialled, dated, and justified by the authorised individual making the change. Do not obliterate, write over, or erase the original entry when making a correction.
When a patient completes a visit, it is anticipated that relevant sections of the CRF will be completed by the investigator (or designated staff) within 24 hours of the last data becoming available, but in no case later than 5 days. Similarly, when a patient completes a study, it is anticipated that all relevant CRF pages will be completed within 24 hours of the last data becoming available, but in no case later than 5 days. This also applies to forms for potential study participants who were not randomised to a treatment group.
As soon as the patient has completed/withdrawn from the study and the CRF is completed the

principal investigator or designated physician(s) under his/her supervision will sign the study conclusion page of the CRF to confirm that they have reviewed the data and that the data are complete and accurate. An original (top copy) CRF must be submitted for all volunteers who have undergone protocol specific procedures, whether or not they completed the study.
Any questions or comments related to the CRF should be directed to the assigned study monitor.

IV Monitoring

Monitoring responsibilities for this protocol will be performed by USAMMDA's Quality Assurance Office and SmithKline Beecham. A Pre-Study/Initiation monitoring visit will be conducted with monitors from USAMMDA and SmithKline Beecham. A minimum of two periodic monitoring visits will be conducted. At least one of these visits will be conducted by USAMMDA's Quality Assurance Office and the rest will be conducted by monitors from SmithKline Beecham. The Close-Out monitoring visit will be conducted by monitors from both USAMMDA's Quality Assurance Office and SmithKline Beecham. Monitoring Reports will be provided to USAMMDA's Quality Assurance Office and SmithKline Beecham after each monitoring visit.

These visits are for the purpose of confirming that the study is being conducted in compliance with the relevant Good Clinical Practice regulations/guidelines, verifying adherence to the protocol and the completeness and exactness of data entered on the CRF and Drug Inventory Forms. The monitor will verify CRF entries by comparing them with the hospital/clinic/office records that will be made available for this purpose. The monitor will retrieve completed CRF sections at each visit. Adequate time and space for these visits must be made available by the investigator.
Investigators must ensure provision of reasonable space and adequate qualified personnel for monitoring visits.

V Archiving of Data

The investigator must retain patient records and CRFs as well as drug disposition records in an easily retrievable form until disposal has been agreed in writing with the sponsor. The investigator must have a 'key' linking the patient's identification code (i.e., treatment number) to the patient's clinical file. If the investigator moves or retires, he/she must nominate someone in writing to be responsible for record keeping. Archived data may be held on microfiche or electronic record, provided that a back-up exists and a hard copy can be obtained from it if required.
It is the policy of the U.S Army Medical and Material Command that data sheets are to be completed on all volunteers participating in research for entry into the Command's Volunteer Registry Database. This is a confidential database and the data entered includes name, address, social security number and details of the clinical. This information is needed to answer questions concerning subjects participating in research sponsored by USAMRMC, and to ensure that subjects can be contacted if there is new information on the study drug. The information should be stored for 75 years.

The sponsor agrees to retain a copy of the protocol, documentation, approvals and all other documents related to the study, including certificates that satisfactory audit and inspection procedures have been carried out.

VI Audits

For the purpose of compliance with Good Clinical Practice and Regulatory Agency Guidelines it may be necessary for the sponsor or a Drug Regulatory Agency to conduct a site audit. This may occur at any time from start to after conclusion of the study.

When an investigator signs the protocol, he agrees to allow the Drug Regulatory Agency and the sponsor's auditors to inspect his/her study records. Furthermore, if an investigator refuses an inspection, their data will not be accepted in support of a New Drug Registration and/or Application.

The sponsor has a substantial investment in clinical studies. Having the highest quality data and studies are essential aspects of drug development. The sponsor has a Regulatory Compliance staff who audit investigational sites. Regulatory Compliance assesses the quality of data with regard to accuracy, adequacy and consistency. In addition, Regulatory Compliance assures that the sponsor's studies are in accordance with the relevant Good Clinical Practices regulations/guidelines and are being followed.

To accomplish these functions, Regulatory Compliance selects investigational sites to audit. These audits usually take 1 to 2 days. The sponsor's audits entail review of source documents supporting the adequacy and accuracy of CRFs, review of documentation required to be maintained, and checks on drug accountability. The audit therefore helps prepare an investigator for a possible regulatory agency inspection as well as assuring the sponsor of the validity of the database across investigational sites.

The Inspector will be especially interested in the following items:

- Log of visits from the sponsor's representatives
- ERC/IRB approval
- Test article accountability
- Approved study protocol and amendments
- Informed consent of the volunteers (written or witnessed oral consent)
- Medical records supportive of CRF data
- Reports to the ERC/IRB and the sponsor
- Record retention

The sponsor will gladly help investigators prepare for an inspection.

VII Confidentiality and Publication

You agree that all information communicated to you by SB is the exclusive property of SB and you will ensure that the same shall be kept strictly confidential by you or other person connected with the work and shall not be disclosed by you or such person to any third party without the prior written consent of SB. You shall communicate the results of the work promptly to the sponsor.

We agree that you shall have the right to publish or permit the publication of any information or material relating to or arising out of the work after prior submission to us provided that if we shall so request, you will delay publication for a maximum of 6 months to enable us to protect our rights of such information or material. Any proposed publication or presentation (e.g., manuscript, abstract or poster) for submission to a journal or scientific meeting, should be sent to the study monitor prior to submission. The sponsor will undertake to comment on such documents within 4 weeks.

All rights and interests world-wide in any inventions, know-how or other intellectual or industrial property rights which arise during the course of and/or as a result of the clinical study which is the subject of this Protocol or which otherwise arise from the information or materials supplied under this Agreement, shall be assigned to, vested in and remain the property of the sponsor.

In drafting this section, additional stipulations can be included in accordance with local custom and practice but the above aspects must also be retained.

APPENDIX D- PHARMACOKINETIC SAMPLING

Up to a total of 7 blood samples (2.5 ml in EDTA tubes) will be collected from all patients for pharmacokinetic analysis during the double blind phase. Three post-dose samples will be collected within varying time windows: one between 6 and 12 hours following the third loading dose, one between 2 and 5 hours postdose on week 8, and one 48-120 hours (2-5 days) following the week 16 dose. Two samples will also be collected just prior to dosing of study medication on weeks 4 and 17 (trough) of the prophylactic phase of the study.

Post third loading dose (6-12 h)	Wk 4* pre-dose	Wk 8* 2-5 h post dose	Wk 16* 2-5 days post-dose	Wk 17 pre-dose
X	X	X	X	X

* The safety blood draws can be coordinated with the time of PK sample draws on these visits.

Additional two samples will be collected from each subject who develops parasitemia. One at the time of developing parastiemia, and the second at the week 28 follow-up visit (2.5 ml into EDTA tubes).

No. Patients	Time of parasitemia	Week 28 follow-up
Any with parasitemia	X	X

At the time of each scheduled clinic visit preceding each scheduled post-dose pharmacokinetic sample (e.g. Day 2, Weeks 8 and 16), the patient will be reminded to come to the clinic for the blood draw within the specified time window. *Attempts should be made to avoid all samples within a specific window to be at one of the extreme ends of the window (eg. 6-12 h window - not all samples at 6 h).* The **date** and **exact** time of each sample should be recorded on the CRF. The **exact** date and time of administration of the dose prior to the sample collection must also be recorded in the CRF (note: for the loading dose regimen, the exact date and time of all 3 doses should be recorded). It is very important that this information is accurately recorded.

Sample Preparation and Handling

Blood samples (2.5 ml) will be collected into EDTA tubes and will be chilled on crushed water ice. Plasma will be separated by centrifugation and will be transferred to appropriately labeled polypropylene tubes. Plasma samples will be stored frozen at approximately -20°C or colder until shipped to either Department of Drug Analysis, SmithKline Beecham, The Frythe, UK or its designee. Samples will be used solely for determination of tafenoquine plasma concentrations. SB or its designee will store (-20°C or colder) the frozen pharmacokinetic plasma samples until analyzed for tafenoquine concentrations using an approved assay technique. Samples should be shipped intermittently during the study period.

Drug Analysis

Plasma concentrations of tafenoquine will be determined using approved assay methodology for the quantification of tafenoquine in human plasma. These analyses will be performed intermittently throughout the study in order to provide Quality Assured analytical results for incorporation into the SB DMPK database in an ongoing fashion. The drug analysis aspects of this study will be done under the direction of the Department of Drug Analysis, DMPK, SmithKline Beecham Pharmaceuticals, UK. The randomization treatment code may be provided to the Drug Analysis Department in order to avoid analyzing samples from patients receiving placebo and/or active comparator. Prior to database freeze, this information will not be communicated to anyone outside of DMPK, SmithKline Beecham Pharmaceuticals. All drug analysis data will be stored in the Archives, SmithKline Beecham, Research and Development or its designee.

Pharmacokinetic Analysis

Plasma tafenoquine concentration-time data will be displayed in tables and/or graphs. Population PK and/or Bayesian estimation methods will be performed using software such as NONMEM or other currently acceptable methods to assess the pharmacokinetics of tafenoquine, if data are appropriate. To support the population PK analysis, the data from this study may be combined with data from other studies. Mean population parameters will be assessed taking into account demographic variables (such as age, weight, gender, race), concomitant medications and/or comorbid diseases. If data permit, the relationship between tafenoquine concentrations and efficacy parameters and selected adverse events will be explored.

Pharmacokinetic analysis will be the responsibility of the Department of Pharmacokinetics, DMPK, SmithKline Beecham. All pharmacokinetic data will be stored in the Archives, SmithKline Beecham, Research and Development. The pharmacokinetic analysis may be reported separately.

Appendix E- Study Schedule: Prophylaxis Phase

	SCREENING					PROPHYLAXIS																														
		Days							Weeks																											
PROCEDURE	-21to -6	-5	-4	-3	-2	0	1	2	1	2	3	4	5	6	7	8	9	10	11	12	13	14	15	16	17	18	19	20	21	22	23	24	25	26	27	28
Informed Consent	X																																			
G6PD	XX																																			
Eligibility Criteria	X					X																														
Demography	X																																			
Pregnancy test	X	X				X						X				X				X				X				X				X				
EKG	X																																			
Medical History	X																																			
Baseline Signs and Symptoms	X	X	X	X	X	X																														
Physical Examination	X																																			X
Prior/Concomitant Medication	X	X	X	X	X	X	X	X	X	X	X	X	X	X	X	X	X	X	X	X	X	X	X	X	X	X	X	X	X	X	X	X	X	X	X	X
Clearance Medication		X	X	X																																
Prophylaxis Medication						X	X	X	X	X	X	X	X	X	X	X	X	X	X	X	X	X	X	X	X	X	X	X	X	X	X	X				
Periph. Blood Mononuclear Cells (PBMCs) 20ml +						X																														X
Plasma Antibody levels 5ml						X						X				X								X									X			X
Safety blood samples 5ml	X					X						X				X								X								X				X
Pharmacokinetic sample* 2.5ml								X§				X				X								X	X§											£X
Adverse Experiences		X	X	X	X	X	X	X	X	X	X	X	X	X	X	X	X.	X	X	X	X	X	X	X	X	X	X	X	X	X	X	X	X	X	X	X
Vital signs	X					X						X				X								X								X				X
Parasite Assessment (Smear)	X				X						X	X	X	X	X	X	X	X	X	X	X	X	X	X	X	X	X	X	X	X	X	X	X	X	X	X

* For details of timing of PK samples see Appendix D; timing of routine hematology/clinical chemistry can be adapted around these visits;

+ 120 subjects only; £ for patients who have developed parasitemia § 100 subjects only

	Post-PROPHYLAXIS (Weeks)																		
PROCEDURE	29	30	31	32	33	34	35	36	37	38	39	40	41	42-49	50	51-59	60	61-69	70
Clearance Medication 3 days									X										
Weekly Parasitological Assessement (Blood Smear)	X	X	X	X	X	X	X	X	X	X	X	X	X	X	X	X	X	X	X
PBMC 20 ml +										X									X
Plasma antibody levels 5ml									X						X		X		X

APPENDIX F- REFERENCES

[1]Anonymous . 1990. World Health Stat Q. 43: 68-79.

[2]Bruce-Chwatt L. J. 1987. Annu Rev Public Health 8: 75: 110

[3]Bruce-Chwatt LJ, Malaria and its prevention in military campaigns. Z Tropenmed Parasitol 22:370-390, 1971

[4]Bruce-Chwatt LJ, (editor) Chemotherapy of malaria. WHO Monograph series. No. 27. 2nd ed. Geneva: World Health Organization, 1981.

[5]Coatney GR, Collins WE, Warren McW, Contacos PG. The Primate Malarias. USDHEW, Government Printing Office, 1979, pp 273-274.

[6] Centers for Disease Control. Health Information for International Travel 1995. US Dept Health and Human Services, Washington DC

[7]Fontanet AL, Johnston BD, Walder AM et al. 1993. Bull WHO 71:377-383

[8]Steffen R, Fuchs E,.Schildknecht J, Naef U, Funk M, Schlagenhauf P,.Phillips-Howard P, Nevill C, Sturchler D, 1993. Mefloquine compared with other malaria chemoprophylactic regimens in tourists visiting east Africa Lancet. 341(8856):1299-303.

[9] Shanks D, Oloo A, Gordon D, Klotz F, Aleman G, Sadie D, Scott T A randomized, double-blinded, placebo-controlled field trial of the chemosuppressive activity of atovaquone/ proguanil (Malarone®) for P. falciparum in Kenya. submitted

[10] Andersen SL, Oloo AJ, Gordon DM, Aleman G, Berman J, Tang , DB, Dunne MW, Shanks GD. A double blinded, placebo controlled trial of azithromycin compared to doxycycline for malaria prophylaxis in western Kenya. Clinical Inf Diseases (in press)

[11] Peters, W., Robinson, BL and Milhous, WK. The chemotherapy of rodent malaria. LI. Studies on a new 8-aminoquinoline, WR 238605. Ann Trop Med Parasitol 87:6, 547-552, 1993.

[12]IND # 38503 (tafenoquine), Surgeon General, U.S. Army, Section 8.1.1.5.

[13] IND # 38503 (WR 238605), Surgeon General, U.S. Army, Section 8.1.1.7.

[14] IND # 38503 (tafenoquine), Surgeon General, U.S. Army, Section 8.1.1.8.

[15] IND # 38503 (WR 238605), Surgeon General, U.S. Army, Section 8.1.1.10

[16] IND # 38503 (tafenoquine), Surgeon General, U.S. Army, Section 8.2.1.

[17] Lee, C.C., Hwang, S.W., Seifert, W.K., and Olson, T.W., (1975), Acute Oral Toxicity of Primaquine and 4-Methylprimaquine in Rats, Mice, Guinea Pigs, and Rabbits, USAMRDC Contract No. DAMD-17-74-C-4036, MRI, Kansas City, Missouri.

[18] IND # 38503 (tafenoquine), Surgeon General, U.S. Army, Section 8.2.1.1.

[19]IND # 38503 (WR 238605), Surgeon General, U.S. Army, Section 8.2.2.2.

[20] Lee, CC, Kinter, LD and Heiffer, MH. Subacute toxicity of primaquine in dogs, monkeys and rats. Bull WHO, 59(3):439-448 (1981).

[21] Levine, BS. (1994) Thirteen week Oral Toxicity Study of WR 238605 with a Thirteen week Recovery Period in Rats, USAMRDC Contract No. DAMD17-92-C2001, Task Order No. UIC-5B, Toxicology Research Laboratory, University of Chicago, Chicago, IL.

[22] A Multiple Dose Safety, Tolerance and Pharmacokinetic Study of tafenoquine when given to Healthy Male and Female Subjects", South Florida Drug Research Corporation, USAMRDC Task Order No. 94-07.

[23]"Rising, single oral dose safety and tolerance study of WR 238605 succinate", South Florida Drug Research Corporation, USAMRDC Task Order 92-04.

[24] Brueckner, RP, Wesche, D, Miller, R, Rieckmann, K, Coster, T and Shmuklarsky, M. Pharmacokinetics, Pharmacodynamics, Safety and Tolerance of a Single Oral Dose of tafenoquine Succinate, Draft Study Report, May 1995.

[25] A Multiple Dose Safety, Tolerance and Pharmacokinetic Study of WR 238605 when given to Healthy Male and Female Subjects", South Florida Drug Research Corporation, USAMRDC Task Order No. 94-07.

[26] Brueckner, RP, Coster, T, Wesche, DL, Shmuklarsky, M, Kim-Ahn, G, Kyle, D, Schneider, I. "Evaluation of tafenoquine as a Prophylactic Agent Against Induced P. falciparum Malaria Infection in Healthy Non-immune Subjects II: a Multiple-dose Causal Versus Suppressive Study"

[27] Sornchai Looareesuwan, et. al. "Dose-Ranging Study of the Safety and Efficacy of WR 238605 in the Prevention of Relapse of *Plasmodium vivax* Infection in Thailand" in progress

[28] CDC Health Information for International Travel 1992 Washington DC

[29] Boudreau E, Schuster B, Sanchez J et al. Tolerability of prophylactic Lariam regimens. Trop Med Parasitol 1993; 44: 257-265.

[30] Barrett PJ, Emmins PD, Clarke PD, Bradley DJ. Comparison of adverse events associated with use of mefloquine and combination of chlorquine and proguanil as antimalarial prophylaxis: postal and telephone survey of travellers. Brit Med J 1996; 313: 525-28.

[31] Nosten F, ter Kuile F, Chongsuphajaisiddhi T, et al. Mefloquine-resistant falciparum malaria on the Thai-Burmese border. Lancet 1991; 337: 1140-43

[32] World Health Organization Malaria Action Programme. Severe and complicated malaria. Trans R Soc Trop Med Hyg 1986; 80(suppl):1-50

[33] Desjardins RE, Pamplin III CI, von Bredow J, Barry KG, and Canfield CJ. Kinetics of a new antimalarial, mefloquine. Clin Pharm Ther 1979; 26:372-379

[34] Schwartz DE, Eckert G, Hartmann D, Weber B, Richard-Lenoble D, Ekue JMK, Gentilini M. Single dose kinetics of mefloquine in man. Plasma levels of the unchanged drug and of one of its metabolites. Chemother 1982; 28:70-84.

[35] Lobel HO, Bernard KW, Williams SL, Hightower AW, Patchen LC, Campbell CC. Effectiveness and tolerance of long-term malaria prophylaxis with mefloquine. JAMA 1991; 265:361-364.

[36] Childs GE, Boudreau EF, Wimonwattratee T, Pang L, Milhous WK. Am J Trop Med Hyg 1991; 44:553-559

[37] ter Kuile FO, Dolan G, Nosten F, Edstein MD, Luxemburger C, Phaipun L, Chongsuphajaisiddhi T, Webster HK, NJ White. Halofantrine versus mefloquine in treatment of multidrug-resistant falciparum malaria. Lancet 1993; 341:1044-49

[38] Shanks G D, Ragama B O, Oloo A J. Time to Reappearance of Malaria Parasites following Various Drug Treatment Regimens in a Holoendemic Area of Western Kenya Trans Royal Soc Trop Med Hyg 1999; in press

[39] Ronn AM, Msangeni HA, Mhina J et al. High level of resistance of Plasmodium falciparum to sulfadoxine-pyrimethamine in children in Tanzania. Trans Royal Soc Trop Med Hyg 1996; 90: 179-81.

[40] Boudreau E, Schuster B, Sanchez J et al. Tolerability of prophylactic Lariam regimens. Trop Med Parasitol 1993; 44: 257-265.

[41] Sanchez JL, DeFraites RF, Sharp TW, Hanson RK. Mefloquine or doxycycline prophylaxis in US troops in Somalia. Lancet 1993; 341:1021-22.

[42] Boudreau, personal communication

[43]Ohrt C, Richie TL, Widjaja H, Shanks GD et al. A double-blind placebo-controlled trial of mefloquine versus doxycycline for the prophylaxis of malaria in Indonesian soldiers. Ann Int Med 1997

[44]Beier JC, Oster CN, Onyango FK, Bales JD, et al Plasmodium falciparum incidence relative to entomologic inoculation rates at a site proposed for testing malaria vaccines in western Kenya. Am J Trop Med Hyg 1994 May Vol. 50 pg 529-36

[45]Hoffman SL, Oster CN, Plowe CV, et al. Naturally acquired antibodies to sporozoites do not prevent malaria: vaccine development implications. Science 1987; 237:639-642

[46]Bennett W, Oloo AJ, Watkins W, et al. Chloroquine treatment of falciparum malaria in an area of Kenya of intermediate chloroquine resistance. Trans Roy Soc Trop Med Hyg 1988; 82:833-837

[47]Oloo AJ, Adoyo A, Kariuki F et al. Sensitivity of P. falciparum malaria against chloroquine and amodiaquine in W. Kenya. East Africa Medical Journal 1991; 68:606-610

[48]Bygbjerg IC, Schapira A, Flachs H, et al. Mefloquine resistance of falciparum malaria from Tanzania enhanced by treatment. Lancet 1983; 1: 774-775.

7th draft

Protocol	Centre Number	Subject Number	Subject Initials
252263/030		0 0 0 0 0	

CONFIDENTIAL

PROTOCOL 252263/030

SCREENING TO WEEK 28

A RANDOMISED, DOUBLE BLIND, PLACEBO CONTROLLED EVALUATION OF WEEKLY TAFENOQUINE (WR 238605/SB252263) COMPARED TO MEFLOQUINE FOR CHEMOSUPPRESSION OF PLASMODIUM FALCIPARUM IN WESTERN KENYA

7th draft

SCREENING

(DAY -21 TO DAY -6)

Protocol 252263/030		Subject Number	Subject Initials	Visit Date (Day Month Year)	Screening (Day-21 to -6)
		0 0 0 0 0			

DEMOGRAPHY

Date of Birth Day Month Year

Gender
- ☐ Male
- ☐ Female

Race
- ☐ Black
- ☐ White
- ☐ Oriental
- ☐ Other → Specify : _______________

VITAL SIGNS

Weight (kg)	Pulse Rate beats/min	Sitting Blood Pressure *(after 5 minutes sitting)* (mmHg) Systolic / Diastolic	Temperature *(Oral)* °C
☐☐☐.☐	☐☐☐	☐☐☐/☐☐☐	☐☐.☐

PREGNANCY TEST

This test should be completed no more than 48 hours before starting Halofantrine treatment.

Is the patient a female of child-bearing potential?

- ☐ No
- ☐ Yes → *If 'Yes' please complete a pregnancy test (blood or urine) and record the results below*

Date performed : Day Month Yr

Is the patient pregnant?

- ☐ No
- ☐ Yes → *If 'Yes' please withdraw the patient from the study, enter details in the Adverse Experience section. Complete the Study Conclusion Section.*

G6PD - TEST 1

The patient should be tested for G6PD twice (from different blood samples) during the Screening period (day -21 to day -6). The patient must have a result of "Normal" on both occasions to be eligible for the study.

Test 1 : Date performed : Day Month Yr

Result :
- ☐ Low → *Patient is not eligible for the study*
- ☐ Intermediate → *Patient is not eligible for the study*
- ☐ Normal

Protocol 252263/030		Subject Number	Subject Initials		Screening (Day-21 to -6)
		0 0 0 0 0			

G6PD - TEST 2

The patient should be tested for G6PD twice (from different blood samples) during the Screening period (day -21 to day -6). The patient must have a result of "Normal" on both occasions to be eligible for the study.

Test 2 : Date performed : ☐☐ ☐☐☐ ☐☐ (Day Month Yr)

Result :
- ☐ Low → *Patient is not eligible for the study*
- ☐ Intermediate → *Patient is not eligible for the study*
- ☐ Normal

PARASITAEMIA

Was a finger prick smear taken ?

- ☐ No
- ☐ Yes → Sample Date ☐☐ ☐☐☐ ☐☐ (Day Month Yr)

Parasitaemia present ?

- ☐ No
- ☐ Yes → Results :
  - ☐ P. Falciparum
  - ☐ P. Malariae
  - ☐ P. Ovale

HISTORY OF MALARIA

Has the patient ever had malaria ?

- ☐ No
- ☐ Yes → Number of attacks in the last six months ☐☐

When was the last attack : ☐☐☐ ☐☐☐☐ (Month Year)

Protocol 252263/030		Subject Number	Subject Initials		Screening (Day-21 to -6)
		0 0 0 0 0			

SCREENING SAFETY BLOOD SAMPLE

**Visit Day**		Screening
**Sample Date**		Day Month Yr
Haematology	**For SB Lab Code Number**	
	**Test**	**Value** [1]
	White Blood Cells	
	Haematocrit	
	Haemoglobin	
	Platelets	
Clinical Chem.	**For SB Lab Code Number**	
	Blood Urea Nitrogen (BUN)	
	Creatinine	
	Alanine transaminase (ALT)	
Are there ***clinically significant abnormal*** laboratory values?		☐ No ☐ Yes*

[1] *Enter laboratory result or one of the following codes:* ***ND*** *=* **not done,** ***LE*** *=* **documented laboratory error**

* *Circle any significant laboratory value and record details on the Significant Medical / Surgical History and Physical Examination page.*

Protocol 252263/030		Subject Number	Subject Initials		Screening (Day-21 to -6)
		0 0 0 0 0			

PRIOR AND CONCOMITANT MEDICATION

Has the patient taken any medication in the **30 days** prior to starting the study or during the study ?

☐ No

☐ Yes ⟶ *If 'Yes', please record details below (Please print clearly)*

Drug Name *(Trade Name Preferred)*	Daily Dose (e.g. 500mg)	Medical Illness/ Diagnosis *(or symptom in absence of diagnosis)*	Start Date *(be as precise as possible)* Day Mth Yr	End Date *(or if Continuing mark box)* Day Mth Yr	
					☐
For SB					
					☐
For SB					
					☐
For SB					
					☐
For SB					
					☐
For SB					
					☐
For SB					
					☐
For SB					
					☐
For SB					
					☐
For SB					
					☐
For SB					
					☐
For SB					

Protocol		Subject Number	Subject Initials		Screening (Day-21 to -6)
252263/030		0 0 0 0 0			

SIGNIFICANT MEDICAL/SURGICAL HISTORY AND PHYSICAL EXAMINATION

Is the patient suffering from or has he/she ever suffered from any **clinically significant** medical or surgical condition?

☐ No

☐ Yes ⟶ *If 'Yes', please list below one diagnosis per line. (Please print clearly)*

Only in the absence of a diagnosis, record the signs and symptoms on separate lines.

Diagnosis	Year of first diagnosis	Past	Ongoing
		☐	☐
For SB			
		☐	☐
For SB			
		☐	☐
For SB			
		☐	☐
For SB			
		☐	☐
For SB			
		☐	☐
For SB			
		☐	☐
For SB			
		☐	☐
For SB			
		☐	☐
For SB			
		☐	☐
For SB			

Protocol 252263/030		Subject Number	Subject Initials		Screening (Day-21 to -6)
		0 0 0 0 0			

ELIGIBILITY CHECKLIST

Please complete the following inclusion criteria.

INCLUSION CRITERIA

	Yes	No
1. Subject is a volunteer (male or female) providing Informed Consent	☐	☐
2. Subject is aged between 18 and 55 years old.	☐	☐
3. Subject will be residing in the study area for the entire duration of the study (approximately 70 weeks).	☐	☐

Do not admit the Subject to this study if any "No" box has been marked.

Please complete the following exclusion criteria.

EXCLUSION CRITERIA

	Yes	No
1. Subject is unwilling to report for drug administration or blood drawing during the 70 week duration of the study.	☐	☐
2. Subject has any medical condition which in the opinion of the investigator makes the subject unsuitable to enter the study	☐	☐
3. Subject has clinically significant abnormalities (to include but not limited to abnormal hepatic or renal function) as determined by history, physical and routine blood chemistries and hematology values.	☐	☐
4. Subject has a personal or family history of seizures	☐	☐
5. Subject has a history of psychiatric disorders	☐	☐
6. Subject is female and is pregnant or lactating or who in the opinion of the investigator are at risk of becoming pregnant	☐	☐
7. Subject has a positive serum / plasma B-HCG (to be tested during screening and within 48 hours of first drug administration and approximately monthly thereafter).	☐	☐

note: All female volunteers will have B-HCG testing. Those requiring repeat testing due to the lapse of >48 hrs will have their original negative test confirmed by an additional urine test within 48 hours of initiation of study medication (halofantrine treatment).

	Yes	No
8. Subject has demonstrated hypersensitivity to any of the study drugs especially to any other 8 - aminoquinolines	☐	☐
9. Subject is taking any other anti-malarial product , or has taken another antimalarial drug other than halofantrine within the previous 2 weeks	☐	☐
10. Subject has abnormal EKG particularly an extended QTc interval >0.42 sec.	☐	☐
11. Subject has received an investigational drug (a new chemical entity not registered for use) within 30 days or 5 half-lives whichever is the longer.	☐	☐

Do not admit the Subject to this study if any "Yes" box has been marked.

CLEARANCE VISITS

(DAY -5 TO DAY -2)

Protocol 252263/030		Subject Number 0 0 0 0 0	Subject Initials		Clearance Day -5 to Day -2

DAY -5 TO DAY -3 : HALOFANTRINE CLEARANCE DOSES

Day	Dose received ?	Date dose received
Clearance dose 1 (Day -5)	☐ No ☐ Yes →	Day Month Yr
Clearance dose 2 (Day -4)	☐ No ☐ Yes →	Day Month Yr
Clearance dose 3 (Day -3)	☐ No ☐ Yes →	Day Month Yr

DAY -2 : PARASITAEMIA

Was a finger prick smear taken ?

☐ No

☐ Yes → Sample Date Day Month Yr

Parasitaemia present ?

☐ No

☐ Yes → Results :

☐ P. Falciparum
☐ P. Malariae
☐ P. Ovale

Protocol 252263/030		Subject Number: 0 0 0 0 0	Subject Initials		Clearance Day -5 to Day -2

BASELINE SIGNS AND SYMPTOMS (or events, or diagnoses)

*Record any baseline events (using **standard medical terminology**) observed or elicited by the following direct question to patient: "Have you felt different in any way since screening ?"*

*Provide the diagnosis, **not** symptoms where possible. One baseline event per column.*

If you consider this to be a serious baseline event (SBE), please do not enter on this page but enter in the Serious Adverse Experience section (SAE) (See opposite for definitions of an SBE)

If no baseline events since Screening, please sign form below and mark this box ☐

**Baseline Event** *(please print clearly)*		
**For SmithKline Beecham**		
**Onset Date and Time**	Day Month Yr 24hr:min	Day Month Yr 24hr:min
**End Date and Time** *(If ongoing please leave blank)*	Day Month Yr 24hr:min	Day Month Yr 24hr:min
**Outcome** *If patient died, STOP: go to SAE section and follow instructions given there*	☐ Resolved ☐ Ongoing ☐ Died	☐ Resolved ☐ Ongoing ☐ Died
**Event Course**	☐ Intermittent → No. of episodes ☐ ☐ Constant	☐ Intermittent → No. of episodes ☐ ☐ Constant
**Intensity** (maximum)	☐ Mild ☐ Moderate ☐ Severe	☐ Mild ☐ Moderate ☐ Severe
**Relationship to study procedures performed prior to randomisation**	☐ Not related ☐ Unlikely ☐ Suspected ☐ Probable (reasonable possibility)	☐ Not related ☐ Unlikely ☐ Suspected ☐ Probable (reasonable possibility)
**Corrective Therapy** *If 'Yes', record details in Prior and Concomitant Medication section and/or Resource Utilisation section if appropriate*	☐ Yes ☐ No	☐ Yes ☐ No
**Was patient withdrawn due to this specific event prior to randomisation?**	☐ Yes ☐ No	☐ Yes ☐ No

Investigator's Signature : ______________________ **Date :** Day Month Yr

RANDOMISATION

(DAY 0)

Protocol 252263/030		Subject Number 0 0 0 0 0	Subject Initials		Randomisation (Day 0)

ELIGIBILITY CHECKLIST

Please complete the following exclusion criteria.

EXCLUSION CRITERIA

		No	Yes
1.	Subject has positive parasitemia following halofantrine treatment for radical cure.	☐	☐
2.	Subject has Glucose 6 Phosphate Dehydrogenase deficiency (G6PD) *(NB. **Two normal** results are needed to be eligible for the study)*	☐	☐
3.	Subject's laboratory values are : Hb < 10 gm, PLT < 80,000/mm^3, WBC < 3000ul^3, Creatinine or ALT more than 2 times the upper limit of normal for age	☐	☐
4.	Subject has a positive serum / plasma B-HCG	☐	☐

Do not admit the patient to this study if any "Yes" box has been marked.

INVESTIGATOR INSTRUCTIONS

Is the patient continuing in the study ?

☐ No ⟶ *Withdraw the patient from the study. Complete the Study Conclusion section.*

☐ Yes ⟶ *Randomise the patient to study medication and give the first dose to the subject (Loading Dose 1)*

RANDOMISATION

Assign a medication pack to the patient and record details below :

Please attach label here

Medication Number : ☐☐☐☐☐

INVESTIGATOR INSTRUCTIONS

Please continue to record the randomisation visit results on page 10 under Visit "Load. dose 1".

RANDOMISATION TO WEEK 8

Protocol	Subject Number	Subject Initials
252263/030	0 0 0 0 0	

RANDOMISATION TO WEEK 3

Visit	Visit / Dosing Date and Time	Dose Taken[1]	Symptoms of Malaria[2]	Preg-nancy Test[3]	Concom. Med.[4]	Adverse Exp.[5]	Parasitaemia[7] (Sample Date)	Strain	Vital Signs	Safety Blood Samples	Date and time of PK blood sample[8]
Load Dose 1	Day Month Yr 24hr:min<br>Day Month Yr 24hr:min	No<br>Yes	No<br>Yes	No<br>Yes	No<br>Yes	No<br>Yes			Temp .<br>Pulse<br>BP /	No<br>Yes	
Load Dose 2	Day Month Yr 24hr:min<br>Day Month Yr 24hr:min	No<br>Yes	No<br>Yes		No<br>Yes	No<br>Yes	Para. present?<br>No<br>Yes→	P.Fal<br>P.Mal<br>P.Ov	Temp .		
Load Dose 3	Day Month Yr 24hr:min<br>Day Month Yr 24hr:min	No<br>Yes	No<br>Yes		No<br>Yes	No<br>Yes	Para. present?<br>No<br>Yes→	P.Fal<br>P.Mal<br>P.Ov	Temp .		6-12 hrs post-dose<br>Day Month Yr<br>24hr:min
Week 1	Day Month Yr 24hr:min<br>Day Month Yr 24hr:min	No<br>Yes	No<br>Yes		No<br>Yes	No<br>Yes	Para. present?<br>No<br>Yes→	P.Fal<br>P.Mal<br>P.Ov	Temp .		
Week 2	Day Month Yr 24hr:min<br>Day Month Yr 24hr:min	No<br>Yes	No<br>Yes		No<br>Yes	No<br>Yes	Para. present?<br>No<br>Yes→	P.Fal<br>P.Mal<br>P.Ov	Temp .		
Week 3	Day Month Yr 24hr:min<br>Day Month Yr 24hr:min	No<br>Yes	No<br>Yes		No<br>Yes	No<br>Yes	Para. present?<br>No<br>Yes→	P.Fal<br>P.Mal<br>P.Ov	Temp .		

Protocol	Subject Number	Subject Initials
252263/030	0 0 0 0 0	

WEEK 4 TO WEEK 8

Visit	Visit / Dosing Date and Time	Dose Taken[1]	Symptoms of Malaria[2]	Pregnancy Test[3]	Concom. Med.[4]	Adverse Exp.[5]	Parasitaemia[7] (Sample Date)	Strain	Vital Signs	Safety Blood Samples	Date and time of PK blood sample[8]
Week 4	Day Month Yr 24hr:min<br>Day Month Yr 24hr:min	No<br>Yes	No<br>Yes	No<br>Yes	No<br>Yes	No<br>Yes	Para. present?<br>No<br>Yes→	P.Fal<br>P.Mal<br>P.Ov	Temp .<br>Pulse<br>BP /	No<br>Yes	Pre-dose<br>Day Month Yr<br>24hr:min
Week 5	Day Month Yr 24hr:min<br>Day Month Yr 24hr:min	No<br>Yes	No<br>Yes		No<br>Yes	No<br>Yes	Para. present?<br>No<br>Yes→	P.Fal<br>P.Mal<br>P.Ov	Temp .		
Week 6	Day Month Yr 24hr:min<br>Day Month Yr 24hr:min	No<br>Yes	No<br>Yes		No<br>Yes	No<br>Yes	Para. present?<br>No<br>Yes→	P.Fal<br>P.Mal<br>P.Ov	Temp .		
Week 7	Day Month Yr 24hr:min<br>Day Month Yr 24hr:min	No<br>Yes	No<br>Yes		No<br>Yes	No<br>Yes	Para. present?<br>No<br>Yes→	P.Fal<br>P.Mal<br>P.Ov	Temp .		
Week 8	Day Month Yr 24hr:min<br>Day Month Yr 24hr:min	No<br>Yes	No<br>Yes	No<br>Yes	No<br>Yes	No<br>Yes	Para. present?<br>No<br>Yes→	P.Fal<br>P.Mal<br>P.Ov	Temp .<br>Pulse<br>BP /	No<br>Yes	2-5 hrs post-dose<br>Day Month Yr<br>24hr:min

Protocol	Subject Number	Subject Initials
252263/030	0 0 0 0 0	

UNSCHEDULED VISITS

Visit Date	Symptoms of Malaria[2]	Preg-nancy Test[3]	Concom. Med.[4]	Adverse Exp.[5]	Parasitaemia[7] (Sample Date)	Strain	Vital Signs	Safety Blood Samples	Date and time of PK blood sample[8]
Day Month Yr	No / Yes	No / Yes	No / Yes	No / Yes	Para. present? No / Yes→	P.Falciparum / P.Malariae / P.Ovale	Temp . / Pulse / BP /	No / Yes	PK sample taken if parasitaemia is positive. Day Month Yr 24hr:min
Day Month Yr	No / Yes	No / Yes	No / Yes	No / Yes	Para. present? No / Yes→	P.Falciparum / P.Malariae / P.Ovale	Temp . / Pulse / BP /	No / Yes	PK sample taken if parasitaemia is positive. Day Month Yr 24hr:min

SAFETY BLOOD SAMPLE RESULTS

	Visit Day	Randomisation	Week 4	Week 8	Unscheduled	Unscheduled
	Sample Date	Day Month Yr	Day Month Yr	Day Month Yr	Day Month Yr	Day Month Yr
Haematology	For SB Lab Code Number					
	Test	Value [1]	Value [1]	Value [1]	Value [1]	Value [1]
	White Blood Cells					
	Haematocrit					
	Haemoglobin					
	Platelets					
Clinical Chem.	For SB Lab Code Number					
	Blood Urea Nitrogen (BUN)					
	Creatinine					
	Alanine transaminase (ALT)					
	Are there ***clinically significant abnormal*** laboratory values?	No Yes*	No Yes*	No Yes*	No Yes*	No Yes*

1 *Enter laboratory result or one of the following codes:*
ND *=* **not done,**
LE = documented laboratory error

* *Circle any significant laboratory value and record details on the Adverse Experience page*

ADVERSE EXPERIENCES (Non-serious)

*Record any adverse experiences (using **standard medical terminology**) observed or elicited by the following direct question to patient:*

"Do you feel different in any way since starting the treatment or since the last visit?"

*Provide the diagnosis, **not** symptoms, where possible. One adverse experience per column.*

If you consider this to be a serious adverse experience (SAE), please do not enter on this page but enter in the SAE section (See opposite for definitions of an SAE).

Protocol	Subject Number	Subject Initials
252263/030	0 0 0 0 0	

7th draft

ADVERSE EXPERIENCES (Non-serious)

If no adverse experiences occured since Clearance, please mark this box. ☐

**Adverse Experience** *(please print clearly)*				
**For SmithKline Beecham**				
**Onset Date and Time**	Day Month Yr 24hr:min	Day Month Yr 24hr:min	Day Month Yr 24hr:min	Day Month Yr 24hr:min
**End Date and Time** *(If ongoing please leave blank)*	Day Month Yr 24hr:min	Day Month Yr 24hr:min	Day Month Yr 24hr:min	Day Month Yr 24hr:min
**Outcome** ***If patient died, STOP: go to SAE section Exacerbation pages***	☐ Resolved ☐ Ongoing ☐ Died	☐ Resolved ☐ Ongoing ☐ Died	☐ Resolved ☐ Ongoing ☐ Died	☐ Resolved ☐ Ongoing ☐ Died
**Experience Course**	☐ Intermittent → No. of episodes ☐ ☐ Constant	☐ Intermittent → No. of episodes ☐ ☐ Constant	☐ Intermittent → No. of episodes ☐ ☐ Constant	☐ Intermittent → No. of episodes ☐ ☐ Constant
**Intensity** (maximum) *See definitions page for Adverse Experience*	☐ Mild ☐ Moderate ☐ Severe	☐ Mild ☐ Moderate ☐ Severe	☐ Mild ☐ Moderate ☐ Severe	☐ Mild ☐ Moderate ☐ Severe
**Action Taken with Respect to Investigational Drug**	☐ None ☐ Dose reduced ☐ Dose increased ☐ Drug interrupted/restarted ☐ Drug stopped	☐ None ☐ Dose reduced ☐ Dose increased ☐ Drug interrupted/restarted ☐ Drug stopped	☐ None ☐ Dose reduced ☐ Dose increased ☐ Drug interrupted/restarted ☐ Drug stopped	☐ None ☐ Dose reduced ☐ Dose increased ☐ Drug interrupted/restarted ☐ Drug stopped
**Relationship to Investigational Drug**	☐ Not related ☐ Unlikely ☐ Suspected ☐ Probable (reasonable possibility)	☐ Not related ☐ Unlikely ☐ Suspected ☐ Probable (reasonable possibility)	☐ Not related ☐ Unlikely ☐ Suspected ☐ Probable (reasonable possibility)	☐ Not related ☐ Unlikely ☐ Suspected ☐ Probable (reasonable possibility)
**Corrective Therapy** *If 'Yes', record details in the Concomitant Medication section and/or Resource Utilisation section if appropriate*	☐ Yes ☐ No	☐ Yes ☐ No	☐ Yes ☐ No	☐ Yes ☐ No
**Was patient withdrawn due to this specific AE?**	☐ Yes ☐ No	☐ Yes ☐ No	☐ Yes ☐ No	☐ Yes ☐ No

Protocol	Subject Number	Subject Initials
252263/030	0 0 0 0 0	

CONCOMITANT MEDICATION

Record any changes in concomitant medication taken since Clearance

☐ No concomitant medication changes since Clearance

**Drug Name** *(Trade Name Preferred)*	**Daily Dose** (e.g. 500mg)	**Medical Illness/ Diagnosis** *(or symptom in absence of diagnosis)*	**Start Date** *(be as precise as possible)* Day Mth Yr	**End Date** Day Mth Yr	*(or if Continuing mark box)*
					☐
For SB					
					☐
For SB					
					☐
For SB					
					☐
For SB					
					☐
For SB					
					☐
For SB					
					☐
For SB					
					☐
For SB					
					☐
For SB					

7th draft

WEEK 9 TO WEEK 16

Protocol	Subject Number	Subject Initials
252263/030	0 0 0 0 0	

WEEK 9 TO WEEK 14

Visit	Visit / Dosing Date and Time	Dose Taken[1]	Symptoms of Malaria[2]	Pregnancy Test[3]	Concom. Med.[4]	Adverse Exp.[5]	Parasitaemia[7] (Sample Date)	Strain	Vital Signs	Safety Blood Samples	Date and time of PK blood sample[8]
Week 9	Day Month Yr 24hr:min<br>Day Month Yr 24hr:min	No<br>Yes	No<br>Yes		No<br>Yes	No<br>Yes	Para. present?<br>No<br>Yes→	P.Fal<br>P.Mal<br>P.Ov	Temp .		
Week 10	Day Month Yr 24hr:min<br>Day Month Yr 24hr:min	No<br>Yes	No<br>Yes		No<br>Yes	No<br>Yes	Para. present?<br>No<br>Yes→	P.Fal<br>P.Mal<br>P.Ov	Temp .		
Week 11	Day Month Yr 24hr:min<br>Day Month Yr 24hr:min	No<br>Yes	No<br>Yes		No<br>Yes	No<br>Yes	Para. present?<br>No<br>Yes→	P.Fal<br>P.Mal<br>P.Ov	Temp .		
Week 12	Day Month Yr 24hr:min<br>Day Month Yr 24hr:min	No<br>Yes	No<br>Yes	No<br>Yes	No<br>Yes	No<br>Yes	Para. present?<br>No<br>Yes→	P.Fal<br>P.Mal<br>P.Ov	Temp .		
Week 13	Day Month Yr 24hr:min<br>Day Month Yr 24hr:min	No<br>Yes	No<br>Yes		No<br>Yes	No<br>Yes	Para. present?<br>No<br>Yes→	P.Fal<br>P.Mal<br>P.Ov	Temp .		
Week 14	Day Month Yr 24hr:min<br>Day Month Yr 24hr:min	No<br>Yes	No<br>Yes		No<br>Yes	No<br>Yes	Para. present?<br>No<br>Yes→	P.Fal<br>P.Mal<br>P.Ov	Temp .		

Protocol	Subject Number	Subject Initials
252263/030	0 0 0 0 0	

WEEK 15 TO WEEK 16

Visit	Visit / Dosing Date and Time	Dose Taken[1]	Symptoms of Malaria[2]	Preg-nancy Test[3]	Concom. Med.[4]	Adverse Exp.[5]	Parasitaemia[7] (Sample Date)	Strain	Vital Signs	Safety Blood Samples	Date and time of PK blood sample[8]
Week 15	Day Month Yr 24hr:min<br>Day Month Yr 24hr:min	No<br>Yes	No<br>Yes		No<br>Yes	No<br>Yes	Para. present?<br>No<br>Yes→	P.Fal<br>P.Mal<br>P.Ov	Temp .		
Week 16	Day Month Yr 24hr:min<br>Day Month Yr 24hr:min	No<br>Yes	No<br>Yes	No<br>Yes	No<br>Yes	No<br>Yes	Para. present?<br>No<br>Yes→	P.Fal<br>P.Mal<br>P.Ov	Temp .<br>Pulse<br>BP /	No<br>Yes	2-5 days post-dose<br>Day Month Yr<br>24hr:min

UNSCHEDULED VISITS

Visit Date	Symptoms of Malaria[2]	Preg-nancy Test[3]	Concom. Med.[4]	Adverse Exp.[5]	Parasitaemia[7] (Sample Date)	Strain	Vital Signs	Safety Blood Samples	Date and time of PK blood sample[8]
Day Month Yr	No<br>Yes	No<br>Yes	No<br>Yes	No<br>Yes	Para. present?<br>No<br>Yes→	P.Falciparum<br>P.Malariae<br>P.Ovale	Temp .<br>Pulse<br>BP /	No<br>Yes	PK sample taken if parasitaemia is positive.<br>Day Month Yr 24hr:min
Day Month Yr	No<br>Yes	No<br>Yes	No<br>Yes	No<br>Yes	Para. present?<br>No<br>Yes→	P.Falciparum<br>P.Malariae<br>P.Ovale	Temp .<br>Pulse<br>BP /	No<br>Yes	PK sample taken if parasitaemia is positive.<br>Day Month Yr 24hr:min

Protocol	Subject Number	Subject Initials
252263/030	0 0 0 0 0	

SAFETY BLOOD SAMPLE RESULTS

		Week 16	Unscheduled	Unscheduled
**Visit Day**		Week 16	Unscheduled	Unscheduled
**Sample Date**		Day Month Yr	Day Month Yr	Day Month Yr
Haematology	**For SB Lab Code Number**			
	**Test**	**Value** [1]	**Value** [1]	**Value** [1]
	White Blood Cells			
	Haematocrit			
	Haemoglobin			
	Platelets			
Clinical Chem.	**For SB Lab Code Number**			
	Blood Urea Nitrogen (BUN)			
	Creatinine			
	Alanine transaminase (ALT)			
Are there ***clinically significant abnormal*** laboratory values?		☐ No ☐ Yes*	☐ No ☐ Yes*	☐ No ☐ Yes*

1 *Enter laboratory result or one of the following codes:*
ND = not done, LE = documented laboratory error

* *Circle any significant laboratory value and record details on the Adverse Experience page*

ADVERSE EXPERIENCES (Non-serious)

*Record any adverse experiences (using **standard medical terminology**) observed or elicited by the following direct question to patient:*<br><br>*"Do you feel different in any way since starting the treatment or since the last visit?"*<br><br>*Provide the diagnosis, **not** symptoms, where possible. One adverse experience per column.*
If you consider this to be a serious adverse experience (SAE), please do not enter on this page but enter in the SAE section (See opposite for definitions of an SAE).

Protocol	Subject Number	Subject Initials
252263/030	0 0 0 0 0	

ADVERSE EXPERIENCES (Non-serious)

If no adverse experiences occured since Week 8, please mark this box. ☐

**Adverse Experience** *(please print clearly)*				
**For SmithKline Beecham**				
**Onset Date and Time**	Day Month Yr 24hr:min	Day Month Yr 24hr:min	Day Month Yr 24hr:min	Day Month Yr 24hr:min
**End Date and Time** *(If ongoing please leave blank)*	Day Month Yr 24hr:min	Day Month Yr 24hr:min	Day Month Yr 24hr:min	Day Month Yr 24hr:min
**Outcome** ***If patient died, STOP: go to SAE section Exacerbation pages***	☐ Resolved<br>☐ Ongoing<br>☐ Died	☐ Resolved<br>☐ Ongoing<br>☐ Died	☐ Resolved<br>☐ Ongoing<br>☐ Died	☐ Resolved<br>☐ Ongoing<br>☐ Died
**Experience Course**	☐ Intermittent → No. of episodes ☐<br>☐ Constant	☐ Intermittent → No. of episodes ☐<br>☐ Constant	☐ Intermittent → No. of episodes ☐<br>☐ Constant	☐ Intermittent → No. of episodes ☐<br>☐ Constant
**Intensity** (maximum) *See definitions page for Adverse Experience*	☐ Mild<br>☐ Moderate<br>☐ Severe	☐ Mild<br>☐ Moderate<br>☐ Severe	☐ Mild<br>☐ Moderate<br>☐ Severe	☐ Mild<br>☐ Moderate<br>☐ Severe
**Action Taken with Respect to Investigational Drug**	☐ None<br>☐ Dose reduced<br>☐ Dose increased<br>☐ Drug interrupted/restarted<br>☐ Drug stopped	☐ None<br>☐ Dose reduced<br>☐ Dose increased<br>☐ Drug interrupted/restarted<br>☐ Drug stopped	☐ None<br>☐ Dose reduced<br>☐ Dose increased<br>☐ Drug interrupted/restarted<br>☐ Drug stopped	☐ None<br>☐ Dose reduced<br>☐ Dose increased<br>☐ Drug interrupted/restarted<br>☐ Drug stopped
**Relationship to Investigational Drug**	☐ Not related<br>☐ Unlikely<br>☐ Suspected (reasonable<br>☐ Probable possibility)	☐ Not related<br>☐ Unlikely<br>☐ Suspected (reasonable<br>☐ Probable possibility)	☐ Not related<br>☐ Unlikely<br>☐ Suspected (reasonable<br>☐ Probable possibility)	☐ Not related<br>☐ Unlikely<br>☐ Suspected (reasonable<br>☐ Probable possibility)
**Corrective Therapy** *If 'Yes', record details in the Concomitant Medication section and/or Resource Utilisation section if appropriate*	☐ Yes ☐ No	☐ Yes ☐ No	☐ Yes ☐ No	☐ Yes ☐ No
**Was patient withdrawn due to this specific AE?**	☐ Yes ☐ No	☐ Yes ☐ No	☐ Yes ☐ No	☐ Yes ☐ No

WEEK 17 TO WEEK 24

Protocol	Subject Number	Subject Initials
252263/030	0 0 0 0 0	

CONCOMITANT MEDICATION

Record any changes in concomitant medication taken since Week 8

☐ No concomitant medication changes since Week 8

**Drug Name** *(Trade Name Preferred)*	**Daily Dose** (e.g. 500mg)	**Medical Illness/ Diagnosis** *(or symptom in absence of diagnosis)*	**Start Date** *(be as precise as possible)* Day Mth Yr	**End Date** Day Mth Yr	*(or if Continuing mark box)*
					☐
For SB					
					☐
For SB					
					☐
For SB					
					☐
For SB					
					☐
For SB					
					☐
For SB					
					☐
For SB					
					☐
For SB					
					☐
For SB					

7th draft

Protocol	Subject Number	Subject Initials
252263/030	0 0 0 0 0	

WEEK 17 TO WEEK 22

Visit	Visit / Dosing Date and Time	Dose Taken[1]	Symptoms of Malaria[2]	Preg-nancy Test[3]	Concom. Med.[4]	Adverse Exp.[5]	Parasitaemia[7] (Sample Date)	Strain	Vital Signs	Safety Blood Samples	Date and time of PK blood sample[8]
Week 17	Day Month Yr 24hr:min<br>Day Month Yr 24hr:min	No<br>Yes	No<br>Yes		No<br>Yes	No<br>Yes	Para. present?<br>No<br>Yes →	P.Fal<br>P.Mal<br>P.Ov	Temp .		Pre-dose<br>Day Month Yr<br>24hr:min
Week 18	Day Month Yr 24hr:min<br>Day Month Yr 24hr:min	No<br>Yes	No<br>Yes		No<br>Yes	No<br>Yes	Para. present?<br>No<br>Yes →	P.Fal<br>P.Mal<br>P.Ov	Temp .		
Week 19	Day Month Yr 24hr:min<br>Day Month Yr 24hr:min	No<br>Yes	No<br>Yes		No<br>Yes	No<br>Yes	Para. present?<br>No<br>Yes →	P.Fal<br>P.Mal<br>P.Ov	Temp .		
Week 20	Day Month Yr 24hr:min<br>Day Month Yr 24hr:min	No<br>Yes	No<br>Yes	No<br>Yes	No<br>Yes	No<br>Yes	Para. present?<br>No<br>Yes →	P.Fal<br>P.Mal<br>P.Ov	Temp .		
Week 21	Day Month Yr 24hr:min<br>Day Month Yr 24hr:min	No<br>Yes	No<br>Yes		No<br>Yes	No<br>Yes	Para. present?<br>No<br>Yes →	P.Fal<br>P.Mal<br>P.Ov	Temp .		
Week 22	Day Month Yr 24hr:min<br>Day Month Yr 24hr:min	No<br>Yes	No<br>Yes		No<br>Yes	No<br>Yes	Para. present?<br>No<br>Yes →	P.Fal<br>P.Mal<br>P.Ov	Temp .		

Protocol	Subject Number	Subject Initials
252263/030	0 0 0 0 0	

WEEK 23 TO WEEK 24

Visit	Visit / Dosing Date and Time	Dose Taken[1]	Symptoms of Malaria[2]	Preg-nancy Test[3]	Concom. Med.[4]	Adverse Exp.[5]	Parasitaemia[7] (Sample Date)	Strain	Vital Signs	Safety Blood Samples	Date and time of PK blood sample[8]
Week 23	Day Month Yr 24hr:min<br>Day Month Yr 24hr:min	No<br>Yes	No<br>Yes		No<br>Yes	No<br>Yes	Para. present?<br>No<br>Yes→	P.Fal<br>P.Mal<br>P.Ov	Temp .		
Week 24	Day Month Yr 24hr:min<br>Day Month Yr 24hr:min	No<br>Yes	No<br>Yes	No<br>Yes	No<br>Yes	No<br>Yes	Para. present?<br>No<br>Yes→	P.Fal<br>P.Mal<br>P.Ov	Temp .<br>Pulse<br>BP /	No<br>Yes	

UNSCHEDULED VISITS

Visit Date	Symptoms of Malaria[2]	Preg-nancy Test[3]	Concom. Med.[4]	Adverse Exp.[5]	Parasitaemia[7] (Sample Date)	Strain	Vital Signs	Safety Blood Samples	Date and time of PK blood sample[8]
Day Month Yr	No<br>Yes	No<br>Yes	No<br>Yes	No<br>Yes	Para. present?<br>No<br>Yes→	P.Falciparum<br>P.Malariae<br>P.Ovale	Temp .<br>Pulse<br>BP /	No<br>Yes	PK sample taken if parasitaemia is positive.<br>Day Month Yr 24hr:min
Day Month Yr	No<br>Yes	No<br>Yes	No<br>Yes	No<br>Yes	Para. present?<br>No<br>Yes→	P.Falciparum<br>P.Malariae<br>P.Ovale	Temp .<br>Pulse<br>BP /	No<br>Yes	PK sample taken if parasitaemia is positive.<br>Day Month Yr 24hr:min

Protocol	Subject Number	Subject Initials
252263/030	0 0 0 0 0	

SAFETY BLOOD SAMPLE RESULTS

	Visit Day	Week 24	Unscheduled	Unscheduled
	**Sample Date**	Day Month Yr	Day Month Yr	Day Month Yr
**Haematology**	**For SB Lab Code Number**			
	**Test**	**Value** [1]	**Value** [1]	**Value** [1]
	White Blood Cells			
	Haematocrit			
	Haemoglobin			
	Platelets			
**Clinical Chem.**	**For SB Lab Code Number**			
	Blood Urea Nitrogen (BUN)			
	Creatinine			
	Alanine transaminase (ALT)			
	Are there ***clinically significant abnormal*** laboratory values?	☐ No ☐ Yes*	☐ No ☐ Yes*	☐ No ☐ Yes*

1 *Enter laboratory result or one of the following codes:*
ND = not done, LE = documented laboratory error

* *Circle any significant laboratory value and record details on the Adverse Experience page*

ADVERSE EXPERIENCES (Non-serious)

Record any adverse experiences (using ***standard medical terminology****) observed or elicited by the following direct question to patient:*

"Do you feel different in any way since starting the treatment or since the last visit?"

Provide the diagnosis, ***not*** *symptoms, where possible. One adverse experience per column.*

If you consider this to be a serious adverse experience (SAE), please do not enter on this page but enter in the SAE section (See opposite for definitions of an SAE).

7th draft

Protocol	Subject Number	Subject Initials
252263/030	0 0 0 0 0	

ADVERSE EXPERIENCES (Non-serious)

If no adverse experiences occured since Week 16, please mark this box. ☐

**Adverse Experience** *(please print clearly)*				
**For SmithKline Beecham**				
**Onset Date and Time**	Day Month Yr 24hr:min	Day Month Yr 24hr:min	Day Month Yr 24hr:min	Day Month Yr 24hr:min
**End Date and Time** *(If ongoing please leave blank)*	Day Month Yr 24hr:min	Day Month Yr 24hr:min	Day Month Yr 24hr:min	Day Month Yr 24hr:min
**Outcome** ***If patient died, STOP: go to SAE section Exacerbation pages***	☐ Resolved<br>☐ Ongoing<br>☐ Died	☐ Resolved<br>☐ Ongoing<br>☐ Died	☐ Resolved<br>☐ Ongoing<br>☐ Died	☐ Resolved<br>☐ Ongoing<br>☐ Died
**Experience Course**	☐ Intermittent →No. of episodes ☐<br>☐ Constant	☐ Intermittent →No. of episodes ☐<br>☐ Constant	☐ Intermittent →No. of episodes ☐<br>☐ Constant	☐ Intermittent →No. of episodes ☐<br>☐ Constant
**Intensity** (maximum) *See definitions page for Adverse Experience*	☐ Mild<br>☐ Moderate<br>☐ Severe	☐ Mild<br>☐ Moderate<br>☐ Severe	☐ Mild<br>☐ Moderate<br>☐ Severe	☐ Mild<br>☐ Moderate<br>☐ Severe
**Action Taken with Respect to Investigational Drug**	☐ None<br>☐ Dose reduced<br>☐ Dose increased<br>☐ Drug interrupted/restarted<br>☐ Drug stopped	☐ None<br>☐ Dose reduced<br>☐ Dose increased<br>☐ Drug interrupted/restarted<br>☐ Drug stopped	☐ None<br>☐ Dose reduced<br>☐ Dose increased<br>☐ Drug interrupted/restarted<br>☐ Drug stopped	☐ None<br>☐ Dose reduced<br>☐ Dose increased<br>☐ Drug interrupted/restarted<br>☐ Drug stopped
**Relationship to Investigational Drug**	☐ Not related<br>☐ Unlikely<br>☐ Suspected (reasonable possibility)<br>☐ Probable	☐ Not related<br>☐ Unlikely<br>☐ Suspected (reasonable possibility)<br>☐ Probable	☐ Not related<br>☐ Unlikely<br>☐ Suspected (reasonable possibility)<br>☐ Probable	☐ Not related<br>☐ Unlikely<br>☐ Suspected (reasonable possibility)<br>☐ Probable
**Corrective Therapy** *If 'Yes', record details in the Concomitant Medication section and/or Resource Utilisation section if appropriate*	☐ Yes ☐ No	☐ Yes ☐ No	☐ Yes ☐ No	☐ Yes ☐ No
**Was patient withdrawn due to this specific AE?**	☐ Yes ☐ No	☐ Yes ☐ No	☐ Yes ☐ No	☐ Yes ☐ No

Protocol	Subject Number	Subject Initials
252263/030	0 0 0 0 0	

7th draft

CONCOMITANT MEDICATION

Record any changes in concomitant medication taken since Week 16

☐ No concomitant medication changes since Week 16

Drug Name *(Trade Name Preferred)*	Daily Dose (e.g. 500mg)	Medical Illness/ Diagnosis *(or symptom in absence of diagnosis)*	Start Date *(be as precise as possible)* Day Mth Yr	End Date Day Mth Yr	*(or if Continuing mark box)*
					☐
For SB					
					☐
For SB					
					☐
For SB					
					☐
For SB					
					☐
For SB					
					☐
For SB					
					☐
For SB					
					☐
For SB					
					☐
For SB					

7th draft

SAFETY FOLLOW-UP VISITS

Protocol	Subject Number	Subject Initials
252263/030	0 0 0 0 0	

SAFETY FOLLOW-UP VISITS

Visit	Visit Date	Symptoms of Malaria[2]	Preg-nancy Test[3]	Concom. Med.[4]	Adverse Exp.[5]	Parasitaemia[7] (Sample Date)	Strain	Vital Signs	Safety Blood Samples	Date and time of PK blood sample[8]
**Safety Follow-up Visit 1**	Day Month Yr	No<br>Yes		No<br>Yes	No<br>Yes	Para. present?<br>No<br>Yes →	P.Falciparum<br>P.Malariae<br>P.Ovale	Temp .		
**Safety Follow-up Visit 2**	Day Month Yr	No<br>Yes		No<br>Yes	No<br>Yes	Para. present?<br>No<br>Yes →	P.Falciparum<br>P.Malariae<br>P.Ovale	Temp .		
**Safety Follow-up Visit 3**	Day Month Yr	No<br>Yes		No<br>Yes	No<br>Yes	Para. present?<br>No<br>Yes →	P.Falciparum<br>P.Malariae<br>P.Ovale	Temp .		
**Safety Follow-up Visit 4**	Day Month Yr	No<br>Yes		No<br>Yes	No<br>Yes	Para. present?<br>No<br>Yes →	P.Falciparum<br>P.Malariae<br>P.Ovale	Temp .<br>Pulse<br>BP /	No<br>Yes	Day Month Yr<br>24hr:min

Protocol	Subject Number	Subject Initials
252263/030	0 0 0 0 0	

7th draft

UNSCHEDULED VISITS

Visit Date	Symptoms of Malaria[2]	Preg-nancy Test[3]	Concom. Med.[4]	Adverse Exp.[5]	Parasitaemia[7] (Sample Date)	Strain	Vital Signs	Safety Blood Samples	Date and time of PK blood sample[8]
Day Month Yr<br>24hr:min	No<br>Yes	No<br>Yes	No<br>Yes	No<br>Yes	Para. present?<br>No<br>Yes →	P.Falciparum<br>P.Malariae<br>P.Ovale	Temp .<br>Pulse<br>BP /	No<br>Yes	PK sample taken if parasitaemia is positive.<br>Day Month Yr 24hr:min
Day Month Yr<br>24hr:min	No<br>Yes	No<br>Yes	No<br>Yes	No<br>Yes	Para. present?<br>No<br>Yes →	P.Falciparum<br>P.Malariae<br>P.Ovale	Temp .<br>Pulse<br>BP /	No<br>Yes	PK sample taken if parasitaemia is positive.<br>Day Month Yr 24hr:min

SAFETY BLOOD SAMPLE RESULTS

	Visit Day	Follow-up Visit 4	Unscheduled	Unscheduled
	Sample Date	Day Month Yr	Day Month Yr	Day Month Yr
Haematology	For SB Lab Code Number			
	Test	Value [1]	Value [1]	Value [1]
	White Blood Cells			
	Haematocrit			
	Haemoglobin			
	Platelets			
Clinical Chem.	For SB Lab Code Number			
	Blood Urea Nitrogen (BUN)			
	Creatinine			
	Alanine transaminase (ALT)			
	Are there ***clinically significant abnormal*** laboratory values?	No Yes*	No Yes*	No Yes*

1 *Enter laboratory result or one of the following codes:*
ND = not done, LE = documented laboratory error

* *Circle any significant laboratory value and record details on the Adverse Experience page*

ADVERSE EXPERIENCES (Non-serious)

*Record any adverse experiences (using **standard medical terminology**) observed or elicited by the following direct question to patient:*

"Do you feel different in any way since starting the treatment or since the last visit?"

*Provide the diagnosis, **not** symptoms, where possible. One adverse experience per column.*

If you consider this to be a serious adverse experience (SAE), please do not enter on this page but enter in the SAE section (See opposite for definitions of an SAE).

Protocol	Subject Number	Subject Initials
252263/030	0 0 0 0 0	

ADVERSE EXPERIENCES (Non-serious)

If no adverse experiences occured since Week 24, please mark this box. ☐

**Adverse Experience** *(please print clearly)*				
**For SmithKline Beecham**				
**Onset Date and Time**	Day Month Yr 24hr:min	Day Month Yr 24hr:min	Day Month Yr 24hr:min	Day Month Yr 24hr:min
**End Date and Time** *(If ongoing please leave blank)*	Day Month Yr 24hr:min	Day Month Yr 24hr:min	Day Month Yr 24hr:min	Day Month Yr 24hr:min
**Outcome** ***If patient died, STOP: go to SAE section Exacerbation pages***	☐ Resolved ☐ Ongoing ☐ Died	☐ Resolved ☐ Ongoing ☐ Died	☐ Resolved ☐ Ongoing ☐ Died	☐ Resolved ☐ Ongoing ☐ Died
**Experience Course**	☐ Intermittent → No. of episodes ☐ Constant	☐ Intermittent → No. of episodes ☐ Constant	☐ Intermittent → No. of episodes ☐ Constant	☐ Intermittent → No. of episodes ☐ Constant
**Intensity** (maximum) *See definitions page for Adverse Experience*	☐ Mild ☐ Moderate ☐ Severe	☐ Mild ☐ Moderate ☐ Severe	☐ Mild ☐ Moderate ☐ Severe	☐ Mild ☐ Moderate ☐ Severe
**Action Taken with Respect to Investigational Drug**	☐ None ☐ Dose reduced ☐ Dose increased ☐ Drug interrupted/restarted ☐ Drug stopped	☐ None ☐ Dose reduced ☐ Dose increased ☐ Drug interrupted/restarted ☐ Drug stopped	☐ None ☐ Dose reduced ☐ Dose increased ☐ Drug interrupted/restarted ☐ Drug stopped	☐ None ☐ Dose reduced ☐ Dose increased ☐ Drug interrupted/restarted ☐ Drug stopped
**Relationship to Investigational Drug**	☐ Not related ☐ Unlikely ☐ Suspected ☐ Probable (reasonable possibility)	☐ Not related ☐ Unlikely ☐ Suspected ☐ Probable (reasonable possibility)	☐ Not related ☐ Unlikely ☐ Suspected ☐ Probable (reasonable possibility)	☐ Not related ☐ Unlikely ☐ Suspected ☐ Probable (reasonable possibility)
**Corrective Therapy** *If 'Yes', record details in the Concomitant Medication section and/or Resource Utilisation section if appropriate*	☐ Yes ☐ No	☐ Yes ☐ No	☐ Yes ☐ No	☐ Yes ☐ No
**Was patient withdrawn due to this specific AE?**	☐ Yes ☐ No	☐ Yes ☐ No	☐ Yes ☐ No	☐ Yes ☐ No

Protocol	Subject Number	Subject Initials
252263/030	0 0 0 0 0	

7th draft

CONCOMITANT MEDICATION

Record any changes in concomitant medication taken since Week 24

☐ No concomitant medication changes since Week 24

Drug Name *(Trade Name Preferred)*	Daily Dose (e.g. 500mg)	Medical Illness/ Diagnosis *(or symptom in absence of diagnosis)*	Start Date *(be as precise as possible)* Day Mth Yr	End Date Day Mth Yr	*(or if Continuing mark box)*
					☐
For SB					
					☐
For SB					
					☐
For SB					
					☐
For SB					
					☐
For SB					
					☐
For SB					
					☐
For SB					
					☐
For SB					
					☐
For SB					

STUDY CONCLUSION

Protocol		Subject Number	Subject Initials		Study Conclusion
252263/030		0 0 0 0 0			

STUDY CONCLUSION

Did the subject complete the study as planned ?

☐ Yes

☐ No ⟶ *If 'No', mark the* ***primary*** *cause of withdrawal. (Mark one box only)*

- ☐ Adverse Experience (complete AE page)
- ☐ Insufficient therapeutic effect (Development of Parasitaemia)
- ☐ Protocol deviation(including non-compliance)
- ☐ Lost to follow-up
- ☐ Did not meet Eligibility criteria
- ☐ Other - specify: ____

I certify that I have reviewed the data on this case report form and that all information is complete and accurate.

Investigator's Signature ____ Day Month Yr

GENERAL INSTRUCTIONS

7th draft

- Write clearly in capital letters using a black ball point pen and press firmly so that all copies are legible
- Do not write in shaded areas
- Answer ALL questions on every page
- Enter "N/A" if information requested is not available
- Initial and date all changes, additions or deletions. Use a single pen stroke to remove an incorrect entry
- Enter the patient initials on all pages on which information has been entered and the patient number and centre number where required

Do not enter additional data/information after the set of CRF pages has been split/separated

The cover flap or plastic writing board should be used to divide each set of pages before writing on them with a ball point pen

DATE

Please use the following three-letter abbreviations for the months

January	JAN
February	FEB
March	MAR
April	APR
May	MAY
June	JUN
July	JUL
August	AUG
September	SEP
October	OCT
November	NOV
December	DEC

e.g. | 0 1 | J A N | 9 9 | *= 1st January 1999*

Day Month Year

TIME

Unless specified otherwise, please use the 24 hour clock

e.g. | 1 5 | 3 0 | *= 3.30 p.m*

24hr:min

PATIENT INITIALS

Please use the following format:

e.g. | J | P | A | S | *= John Peter Andrew Smith*

ROUTE KEY FOR PRIOR/CONCOMITANT MEDICATION PAGE

PO	=	*By mouth*	*SL*	=	*Sublingual*	*TD*	=	*Transdermal*
IV	=	*Intravenous*	*IT*	=	*Intrathecal*	*IH*	=	*Inhaled*
IM	=	*Intramuscular*	*VA*	=	*Vaginal*	*SC*	=	*Subcutaneous*
IAR	=	*Intra-arterial*	*NA*	=	*Nasal*	*PR*	=	*Rectal*
TO	=	*Topical*	*IA*	=	*Intra-articular injection*	*ID*	=	*Intradermal*

Study medication

If the subject reports vomiting within one hour of dosing, the same dose will be re-administerd the next day. If the subject sequentially vomits two doses of study drug, he / she will be withdrawn from the study as intolerant of medication and the Study Conclusion page will be completed.

1. Dose Taken

Where dose is subsequently re-administered due to vomiting, this refers to first dose only.

2. Symptoms of Malaria

Refer to the information on the field workers visit report form.

3. Pregnancy test

If positive for pregnancy, subject must be withdrawn from prophylaxis treatment. This must be reported immediately as a Serious Adverse Event

4. Concomitant Medication

Any changes in concomitant medication, including dose changes, should be recorded on the 'Prior and Concomitant Medication' page in the relevant section of the CRF.

5. Adverse Experiences

Elicit AEs by asking the standard non-leading question "Have you felt unwell at all since the last visit ?". Any reported should be recorded in detail on the AE pages in the relevant section of the CRF.

6. Parasitaemia

If the subject has a positive parasitaemia, withdraw them from study medication. Continue to follow-up the subject and complete the Safety Follow-Up Visits at the back of this book.

7. Safety Blood Samples

Record results in relevant section of CRF.

8. PK (Pharmacokinetic) Blood Sample

It is vital that the date and time of sample collection are noted and recorded as accurately as possible, using the 24 hour clock time Attempts should be made to avoid all samples within a specific window to be at one of the extreme ends of the window (eg. 6-12 h post-dose window - not all samples should be taken at 6 h).

Patient's withdrawing from study medication

If subject is to be withdrawn from study medication, they should complete the Safety Follow-Up visits and then the Study Conclusion page.

INSTRUCTIONS FOR REPORTING SERIOUS ADVERSE EXPERIENCES (SAE)

SAEs MUST BE REPORTED TO SMITHKLINE BEECHAM WITHIN 24 HOURS

COMPLETE THE SAE PAGES OPPOSITE

Please complete these pages as fully and accurately as possible in order to minimise the time you spend dealing with data queries

If the SAE is still ongoing at the time of reporting, please leave 'Experience Course' blank and update it later

SIGN AND DATE THE SAE PAGE

PLEASE ENSURE THAT ALL OF THE INFORMATION ON THE FOLLOWING CRF PAGES IS COMPLETE

- *Demography*
- *Significant Medical/Surgical History and Physical Examination*
- *Study Medication Record*
- *Concomitant Medication*
- *Form D (if applicable)*

PHOTOCOPY THE SAE PAGES AND THE CRF PAGES SPECIFIED ABOVE
(Do not separate the NCR pages)

FAX A COPY OF THE SAE PAGES AND ALL OF THE CRF PAGES SPECIFIED ABOVE TO:

Your local SB CRA/Medical Monitor (see Investigator Site File for appropriate fax number)

If no photocopier OR fax is available please telephone your local SB CRA/Medical monitor within 24 hours

Protocol		Subject Number	Subject Initials	SB Receipt Date Day Month Year	
252263/030		0 0 0 0 0			

SERIOUS ADVERSE EXPERIENCE (SAE)

Person Reporting SAE ______________________
(Please print clearly)

AEGIS Number

Serious Adverse Experience
(Please print clearly)

For SmithKline Beecham

**Onset Date and Time**	Day Month Yr 24hr:min
**End Date and Time** *(If ongoing please leave blank)*	Day Month Yr 24hr:min
**Outcome** *If patient died, please complete Form D*	☐ Resolved ☐ Ongoing ☐ Died
**Experience Course**	☐ Intermittent → No. of episodes ☐ ☐ Constant
**Intensity** *(maximum)*	☐ Mild ☐ Moderate ☐ Severe
**Action Taken with Respect to Investigational Drug**	☐ None ☐ Dose reduced ☐ Dose increased ☐ Drug interrupted/ restarted ☐ Drug stopped
**Relationship to Investigational Drug**	☐ Not related ☐ Unlikely ☐ Suspected (reasonable possibility) ☐ Probable
**Corrective Therapy** *If 'Yes', record details in the Concomitant Medication section*	☐ Yes ☐ No
**Was patient withdrawn due to this specific SAE ?**	☐ Yes ☐ No

→ *Specify reason(s) for considering this a serious AE. Mark all that apply.*

[1] ☐ fatal
[2] ☐ life threatening
[3] ☐ disabling/incapacitating
[4] ☐ results in hospitalisation (excluding elective surgery or routine clinical procedures)
[5] ☐ hospitalisation prolonged
[6] ☐ congenital abnormality
[7] ☐ cancer
[8] ☐ overdose
[9] ☐ Investigator considers serious or a significant hazard, contraindication, side effect or precaution

Did the SAE abate? ☐ Yes ☐ No

If study medication was interrupted, stopped or dose reduced:
Was study medication reintroduced (or dose increased)? ☐ Yes ☐ No

If yes, did SAE recur? ☐ Yes ☐ No

Assessment (Not related / Unlikely)
The SAE is probably associated with:

☐ Protocol design or procedures (but not to study drug)
Please specify ______________

☐ Another condition (eg, condition under study, intercurrent illness)
Please specify ______________

☐ Another drug
Please specify ______________

Protocol 252263/030		Subject Number	Subject Initials	SB Receipt Date Day Month Year	
		0 0 0 0 0			

SERIOUS ADVERSE EXPERIENCE (SAE) (cont)

Relevant Laboratory Data
Please provide relevant abnormal laboratory data below

Test	Date	Value	Units	Normal Range
	Day Month Yr			
	Day Month Yr			
	Day Month Yr			
	Day Month Yr			

Remarks *(Please provide a brief narrative description of the SAE, attaching extra pages eg. hospital discharge summary if necessary)*

If applicable, was randomisation code broken at investigational site? ☐ No ☐ Yes

Randomisation / Study Medication Number: ☐☐☐☐☐

Investigator's Signature: ------------------------------ Date Day Month Year
(confirming that the above data are accurate and complete)

Please PRINT Name ------------------------------

SB Medical Monitor's Signature: ------------------------------ Date Day Month Year

Please PRINT Name ------------------------------

00000

7th draft

Protocol 252263/030	Centre Number				

LABORATORY REFERENCE RANGES

Investigator ______________________ | For SB | Lab Number Code ________

Date for which these reference ranges become effective [Day | Month | Yr]

Laboratory Name ______________________

Address ______________________

☐ Male reference range ☐ Female reference range Age range [ ] - [ ] Years

Enter the reference range for each test. If the reference ranges for any test differ by sex or age, complete a separate form for each variation, e.g., males 0-16, males 17-99, females 0-16, females 17-99 years. Complete the units reported by this laboratory for each test.

	Test	Unit	Low Reference Value	High Reference Value
Haematology	White Blood Cells			
	Haematocrit			
	Haemoglobin			
	Platelets			
Clininall Chem.	Blood Urea Nitrogen (BUN)			
	Creatinine			
	Alanine transaminase (ALT)			

Protocol 252263/030		Subject Number	Subject Initials		Parasitaemia Testing
		0 0 0 0 0			

PARASITAEMIA TESTING

☐ Reader 1 ☐ Reader 2 ☐ Arbitrator reading

Visit	Sample Date	Results		Strain count (per mm^3)			Readers Initials
	Day Month Year	Pos	Neg	P. Falciparum	P. malariae	P. ovale	
Screen							
Day-2 Post-Halofantrine Clearance							
Loading 2							
Loading 3							
Week 1							
Week 2							
Week 3							
Week 4							
Week 5							
Week 6							
Week 7							
Week 8							
Week 9							
Week 10							
Week 11							
Week 12							
Week 13							
Week 14							
Week 15							
Week 16							
Week 17							

00000

7th draft

Protocol 252263/030		Subject Number	Subject Initials		Parasitaemia Testing
		0 0 0 0 0			

PARASITAEMIA TESTING

☐ Reader 1 ☐ Reader 2 ☐ Arbitrator reading

Visit	Sample Date	Results		Strain count (per mm³)			Readers Initials
	Day Month Year	Pos	Neg	P. Falciparum	P. malariae	P. ovale	
Week 18							
Week 19							
Week 20							
Week 21							
Week 22							
Week 23							
Week 24							
Week 25							
Week 26							
Week 27							
Week 28							

Protocol 252263/030		Subject Number	Subject Initials		Unscheduled Parasitaemia Testing
		0 0 0 0 0			

UNSCHEDULED PARASITAEMIA TESTING

☐ Reader 1 ☐ Reader 2 ☐ Arbitrator reading

Sample Date	Results		Strain count (per mm³)			Readers Initials
Day Month Year	Pos	Neg	P. Falciparum	P. malariae	P. ovale	

7th draft

Protocol 252263/030		Subject Number	Subject Initials		Parasitaemia Testing
		0 0 0 0 0			

PARASITAEMIA TESTING

☐ Reader 1 ☐ Reader 2 ☐ Arbitrator reading

Visit	Sample Date	Results		Strain count (per mm³)			Readers Initials
	Day Month Year	Pos	Neg	P. Falciparum	P. malariae	P. ovale	
Week 29							
Week 30							
Week 31							
Week 32							
Week 33							
Week 34							
Week 35							
Week 36							
Week 37							
Week 38							
Week 39							
Week 40							
Week 41							
Week 42							
Week 43							
Week 44							
Week 45							
Week 46							
Week 47							
Week 48							
Week 49							

00000

7th draft

Protocol		Subject Number	Subject Initials		Parasitaemia Testing
252263/030		0 0 0 0 0			

PARASITAEMIA TESTING

☐ Reader 1 ☐ Reader 2 ☐ Arbitrator reading

Visit	Sample Date	Results		Strain count (per mm³)			Readers Initials
	Day Month Year	Pos	Neg	P. Falciparum	P. malariae	P. ovale	
Week 50							
Week 51							
Week 52							
Week 53							
Week 54							
Week 55							
Week 56							
Week 57							
Week 58							
Week 59							
Week 60							
Week 61							
Week 62							
Week 63							
Week 64							
Week 65							
Week 66							
Week 67							
Week 68							
Week 69							
Week 70							

Protocol 252263/030		Subject Number	Subject Initials		FORM D
		0 0 0 0 0			

FORM D

Certified Cause of Death ____________________

Date of Death [_ _ | _ _ _ | _ _] Day Month Yr → *Complete Adverse Experience section*

Was a post-mortem Performed ?

- [ ] No
- [ ] Yes → *If 'Yes' please summarise findings (include diagnosis):*

____

____

____

____

Physician's Signature ____________________ ____________________ **Date**

7th draft

Protocol		Subject Number	Subject Initials	Investigator
252263/030		0 0 0 0 0		

CRF TRACKING/REVIEW FORM

Please submit COMPLETE Modules only. Do NOT submit whole blank modules.

Module	Module Description	Page Range	Modules: Sent to DM	Modules: Unused	Missing Pages (If Applicable)
MOD1	Screening to Randomisation	1 - 9	☐	☐	
MOD2	Randomisation to Week 8	10 - 14	☐	☐	
MOD3	Week 9 to 16	15 - 19	☐	☐	
MOD4	Week 17 to 24	20 - 24	☐	☐	
SFUP	Safety Follow-up Visits	25 - 28	☐	☐	
CONC1	Study Conclusion	29	☐	☐	
PARA1	Parasitaemia Results	n/a	☐	☐	
PARA2	Unscheduled Parasitaemia results	n/a	☐	☐	
SAE1	Serious Adverse Experience	30 - 31	☐	☐	
FORMD	Form D	n/a	☐	☐	

If multiple modules are submitted together, please ensure they are sorted according to the order above

Site Monitor's Comments (e.g. protocol violations or deviations)

Site Monitor's Signature ______________________ Date Submitted [| |] Day Month Yr

UHOBELAHOBELA, FICHO PANDE MBILI, VIBANDIKIZO VILIVYO LINGANISHA KATIKA UTAFITI WA TAFENOQUINE WA KILA JUMA (WR 238605/SB252263) KULINGANISHA NA MEFLOQUINE KAMA ANGAMIZO LA *PLASMODIUM FALCIPARUM* MAGHARIBI MWAKENYA

KISWAHILI CONSENT

APPENDIX B - FOMU YA MAELEZO JUU YA KUBALIANO.
MAELEZO JUU YA KUBALIANO

Ni lazima uelewe maneno yalioko katika kielezo hiki kabla kukubali kushiriki katika utafiti huu. Utapewa mfano wa kielezo cha kuchukua uende ukaweke wewe mwenyewe.

KUSUDIO LA UTAFITI

Unaombwa kushiriki katika utafiti wa jaribio la kiliniki la dawa kukinga malaria. Dawa hili linaitwa "Tafenoquine" na limekwisha dhibitiswa kua na uwezo wa asili mia 90-95% wa kuzuia homa ya malaria katika jaribio za kiliniki ndogo ndogo na katika utumishi wa dawa. Bado halijastawi katika masoko kwa vile ni jipya na bado linafanyiwa uchunguzi wa usalama na ubora wake, lakini tahasisi ya utumishi (Food and Drug Administration "FDA") ya marekani) wameidhinisha utumishi wake katik utafiti huu.

Lengo la utafiti huu ni kuhakikisha kwamba kipimo chenye kilipendekezwa cha (miligramu 200) kina uwezo vile tunavotarajia katika utafiti huu kubwa na kwa kulinganisha na dawa iitwayo mefloquine (miligramu 250) yenye imekwisha stawishwa kisoko. Wahisani miatatu wataandikishwa na kupata dawa kiutafiti. Kati ya wahisani (thuluthi moja) watapata utabibu wenye hauna nguvu za kutibu itwayo Placebo ambalo litafanana sawa sawa kama lile dawa lenye uwezo wakutibu. Hii ni kwa akikisho kwamba homa ya malaria kwa ukweli iko katika sehemu na ya kwamba madawa yalio na nguvu yana fanya kazi.
Utakua na moja kati ya bahati tatu ya kuchukua aitha mefloquine, tafenoquine au lile dawa lenye halina uwezo Placebo. Kati yako na daktari wako hakuna mwenye atajua ni dawa ipi iliokuwa ikitumika mpaka baada ya mwisho wa utafiti.
Kabla kuanza kuchukua matibabu yeyote utapewa na utabibu wa tiiba la siku 3 ya dawa liitwalo halofantrine. Hili linauwezo kama ule wa mefloquine nalo pia liko katika soko la hapa nchini Kenya. Imeonyesha kwamba linauwezekano wa kuuwa vijidudu vya aina yeyote vilioko katika maini yako, hata kama havionenkani katika damu yako. Hii ni ya ku akikisha kwamba wewe uko katika afya njema kabla ya kuanzishwa matibabu.

MUDA WA UTAFITI

Utafiti unahusika kipindi cha ukaguzi wa takribani wiki tatu kuona kama uko na afya njema kutizamwa kama huna homa ya malaria, na kwamba una stahili kuekwa katika utafiti. Baada ya hii utaombwa kuchukua dawa zako za utafiti kwa muda wa majuma 24. Mwisho wa tiiba la madawa utapata fatilio la usalama kama mojawapo wa utafiti kwa muda zaidi wa majuma 4. Mwisho wa muda huu wa majuma 28, ijapokuwa utahitajika kuwa hau chukua dawa lolote, utaangaliwa na maafisa wa ki afya kuona kama matibabu yenye unepatiwa yamedhuru kinga yako dhidi ya malaria. Watumishi wa ki afya watakutembelea mara moja kila juma kwa muda wa juma 9 kutizama kwamba hali yako ni nzuri nakuona kama pengine umepatikana na malaria. Baada ya hii utapatiwa kipindi cha utabibu cha halofantrine kuuwa viini vya malaria vyenye vinauwezekano wakukaa katika maini na kufatiliwa kwa muda wa majuma 33 zaidi. Kwa ujumla utafiti kwa hivyo ni wa majuma 70 na utahitajika kuishi katika sehemu hiyo ya utafiti kwa muda huo. Wakati wa utafiti utahitajika kuja katika kiliniki takribani mara 10 katika muda wa miezi 6 za kwanza, na takribani mara 7 zaidi kwa muda utakaobaki wa utafiti. Katika tokeo lolote katika muda huu utakua huru kwa kiliniki na unauwezo kuja kiliniki usikiapo huna afya ya kupendeza.

HATARI/MANUFAA YA UTAFITI

Unapoamua kushiriki katika utafiti huu, kuna dalili ya kupata 1 katiya bahati 3 kwamba utakuwa katika kundi la placebo na kwa hivyo kutopata kinga kamilifu.
Kuna hatari ya madawa kutoelewana na mwili wa mtu yeyote. Kawaida madhara ya mefloquine ni ukosefu wa nguvu, ukosefu wa usingizi, ndoto mbaya, kutapika, kusikia kuchafuka kwa roho, matokeo haya mabaya

Nambari ya mhisani: .... Ufupisho wa mhisani: ....

yanadhuru karibu 1 kati ya watu 5. Tafenoquine inaweza kudhuru watu wenye aina ya damu ijulikanayo kama G6PD kosefu ambao uleta kukatika kwa damu ambao mwisho uleta kuhitajii kuongezwa kwa damu. Takribani asili mia 10 wako katika hali hii. Utapimwa kwa ajili ya damu aina hii na hautaruhusiwa kushiriki katika utafiti huu kama uko katika kundi la watu walio na damu aina hii. Madhara mengine yenye yanafikiriwa kuwa kwa ajili ya tafenoquine inahusisha kuhisi kutapika, kuharisha katika mtu 1 kwa 5, na kwa wakati chache kuumwa kwa kichwa, wepesi wa kichwa au kutohisi ladha katika mdomo.

Kunauwezekano kuwa madhara mengine yanaweza kuonekana ambayo bado haijatambulika. Iwapo utapata madhara yeyote wingi wake utatizamwa na matibabu yatakayo lingana na madhara hayo yatapeanwa kwako. Madawa ya utafiti yanaweza kusimamishwa ikibidi ili kutibu madhara yeyote yatokeayo kwa ajili ya madawa haya. Utaelezwa kuhusu matokeo muhimu ambayo yanaweza ku adhiri uamuzi wako kuendelea kushiriki katika utafiti huu.

Kunauwezekano kwamba unaweza kupatikana na viini vya malaria katika damu yako, hata kama huna dalili ya hali hiyo. Hili likitokea ina maanisha kwamba dawa hazija fanya kazi vilivyo na matibabu kwako ya dawa hizo yatasimamishwa. Daktari wako ataamuwa kama unahitaji matibabu au la na kuamuwa matibabu yafaayo. Utapata kutobolewa kidole chako kiasi ili damu kidogo ipatikane ipakwayo kwenye ki glasi juma lifatalo na baadaye utatizamwa kila baada ya juma moja na watumishi fatilizi wa kiafya. Upatikanapo na dalili za malaria, daktari wako atakutibu vile atakavyo onelea ni vyema kwako

Kuna uwezekano kwamba baada ya kusimamisha matibabu inaweza kuwa rahisi kwako kupata malaria. Hata hivyo, utafatiliwa na kuangaliwa kuhusu malaria kwa muda usiopungua mwaka moja baada ya kusimaisha na utazidi kua huru kwa matibabu katika kiliniki ya Walter Reed Kombewa.

Manufaa ya utafiti ni kwamba utaangaliwa zaidi kuhusu maradhi ya malaria, na utapewa matibabu utakapo pendelea hivyo. Hali ya afya yako kuhusu maradhi yeyote wakati wa muda wa utafiti yatakua ni bure bila malipo kwako. Matokeo ya utafiti yanaweza kusaidia kupata kuelewa zaidi maradhi ya malaria.

MPANGILIO WA UTAFITI

Utafiti utakua katika vipindi vitatu, Ukaguzi, Uzuizi wa malaria na baadaye kufatiliwa. Wahisani 300 watachaguliwa katika utafiti kulingana na msemo wa "anaekuja mwanzo utumikiwa kwanza".

Uchunguzi: Watu wazima wenye umri kati ya miaka 18 na 55 na wenye hawatakosa kupatikana hata kwa juma moja kwa muda wa majuma 70 wanaweza kujitolea ili washiriki katika utafii huu.na wata kaguliwa kwa muda wa zaidi ya majuma 3 kuanzia Aprili 2000. Hili lina husisha kasindano kadogo kenye kantumiwa kutoboa kidole pahali padogo ili damu kidogo ipatikane baada ya kufinya ili idadi ya matone kadha yapatikane kuekwa kwenye glasi. Uchunguzi katika mahabara unafanywa kuhakikisha kwamba damu yako, maini na figo zinafanya katika njia inayofaa na kwa kujihadharisha kutokeapo madhara fulani. Utapatiwa pimo la EKG lenye uonekana kuangalia kama roho inafanya kazi vilivyo, hili linahusisha kulala chini na kupata vitambara kushikanishwa kwenye kifua chako na waya kwa muda wa dakika chache. Kama wewe ni mtu wakike utajaribiwa kuona kama wewe ni mja mzito kwa kutumia mkojo au jaribio la damu. Kama umja mzito ama kama unanyonyesha huna haki ya kujiunga katika utafiti huu. Vile vile utaombwa kutokuwa mjamzito mpaka baada ya majuma 15 baada ya kukamilisha dawa za utafiti yaani likikamilisha muda wa utafiti, mpaka ma juma 39 baada ya kuanza matibabu. Wahisani wote wanaotumia dawa zinazo tibu malaria badala ya halofantrine hadi majuma mawili kabla kuanza utafiti pia hawata husishwa.

Damu yako haitapimwa ili kuchunguzwa HIV au virushi vya AIDS kwa vile huu ni utafiti wa malaria siyo wenye unaohusu AIDS. Watu wenye watapatikana kwamba hawa kupata kujiunga na utafiti huu utapewa au mtapewa barua ya kujulisha jambo hili na kukaribishwa kurudi katika kiliniki ili kupata ufafanuzi kutoka kwa daktari. Hao halafu watakapo hitaji matibabu kutoka kwa vituo vya matibabu watapewa barua ya uhamisho ili wapate kuenda hospitali yeyote yaki serikali itakayo kuwa karibu nao ili watibiwe kulingana na desturi za kiafya hapa Kenya

UHOBELAHOBELA, FICHO PANDE MBILI, VIBANDIKIZO VILIVYO LINGANISHA KATIKA UTAFITI WA TAFENOQUINE WA KILA JUMA (WR 238605/SB252263) KULINGANISHA NA MEFLOQUINE KAMA ANGAMIZO LA *PLASMODIUM FALCIPARUM* MAGHARIBI MWAKENYA

Uzuwizi: Washiriki wenye kustahili kuanza baadaye wanaweza kuanza ule msimu wa kutibiwa wa juma 24 baada ya kukamilisha viini vya malaria katika mwili na utibabu wa Halofantrine kwa muda wa siku 3 kufuatia hili, utapangiwa kupata aitha mefloquine, tafenoquine (dawa la jaribio) au Placebo. Haitakuwa ni rahisi kwako kujua ni dawa ipi unaotumia.

Utachukua dawa yenye umepangiwa kwa muda wa siku 3 zitakazo fatana kuanza nazo ili kufika viwango vya nguvu zaidi haraka, ambao itakua ni capsule moja na tembe moja kila siku zitakazo mezwa. Kila kiimo cha madawa ya utafiti yatatumiwa na chakula kidogo kusaidia kuenea kwa maungo.

Hapo baadaye kila juma hadi juma 24, utabibu utapeanwa kula juma. Watu wengine watapatiwa capusels zenye zinafanana kama madawa ya utafiti huu kumbe hayana dawa kamilifu ndani yake. Mtu yeyote mwenye atapatikana na malaria katika damu yake wakati wa utafiti huu atapatiwa tiba ijulikanayo wakati akiwa mgonjwa na atatibiwa mpaka atakapo pona, ugonjwa huo wa malaria. Wahisani wataendelea kufuatiliwa mpaka mwisho wa juma la 70 hata kama watapatikana na malaria na kupewa utabibu tofauti. Takribani wahisani 100 watapata nyongeza kidogo ya damu kuchukuliwa.

Fatiliyo: Wafanya kazi fatilizi wa kiafya watapatia wahisani madawa kwa kipindi (Tembe moja kila siku kwa muda wa siku 3) ya halofantrine katika juma la 37, na baadaye kutazamiwa baada ya kila juma moja mpaka juma la 70. Kidole kitatobolewa pahali padogo ili kiasi kidogo cha damu kipatikane kila juma kuchunguza virushi vya malaria. Sampuli za damu zitachukuliwa katika vipindi 4 kuona kama umepatikana na virushi vya malaria. Katika juma la 37, 50, 60 na 70.

PIMO KATIKA UTAFITI/CHENYE KUHUSISWA

Utaombwa kupeana damu kidogo kutoka kwenye kidole ili pimola malaria litimizwe kwenye glasi ya kupima wiki baada ya juma kwa muda wote wa utafiti. Pia ili utoe sampuli kidogo ya damu wakati wa uchunguzi na kiwango cha juu mara 8 katika kipindi cha kuzuia cha utafiti kwa pimo za damu za kawaida na shughuli za viwango vya madawa. Utaombwa kuhudhuria kiliniki wakati sampuli za damu zinataka kuchukuliwa kwa mfano wakati wa kuchukuwa dawa, juma la 4, 8, 16, 24 na 28. Umuhimu wa kuchukua sampuli hizi za damu ni kuhakikisha kwamba damu, maini, moyo na figo za fanya kazi vilivyo ili kupunguza hatari za matokeo mabaya badaye. Kupima kiwango cha dawa katika damu, nakupima unyefu wa viini vya malaria hatari. Wakati wa kipindicha kufatiliwa sampuli za damu zitachukuliwa kwa tokeo 4 zaidi. Wakati wa juma la 37, 50, 60 na la70 kuoma kwamba umekwisha wahi ambukizwa na viini vya malaria (Kupima unyefu hatari ya viini). Sampuli ya ziada yaweza itajika kama umepatikaniwa na malaria kutizama kama umekwisha angaziwa ambukizo la viini vya malaria (kupima kiwango cha ambukizo). Kunaweza kua na umivu kidogo na kwa wakati mwingine kwaruzo kidogo kama damu imechukuliwa kutoka kwenye mshipa kuu wa mkono. Damu itakayo chukuliwa kutoka kwa akina mama katika utafiti pia itajaribiwa iwapo kuna ujauzito au la na akina mama waja wazito hawata husishwa kamwe katika utafiti. Wakati wa mhula wa kizuizi takribani wahisani 100 wataombwa kuhudhuria kiliniki kwa masiku kadha, kwenye siku ya 3 na katika juma la 17, Hili ni kuezesha kutolewa damu zaidi yakuongezea ili kutizama viwango vya dawa katika nyakati hizi.. Hii ni ya kuezesha kupeana sampuli za damu ili viwango vya madawa vitizamwe kwa nyakati hizi. Wakati wa mhula wa fatiliyo, takribani wahusika 120 wataombwa kutoa kiasi tena cha damu wakati wa kuanza na wakati wa juma la 28, 38 na 70 ili kutizama ubora wakimaumbile wa nguvu zipiganazo na maradhi ya malaria. Katika mwilini mwako kwa zote ila kwa sampuli ya juma la 38, sampuli zote zitachukuliwa kwa wakati moja kama desturi ya sampuli za damu ya kawaida na hakuna matembezi ya ziada yatahitajika.

Wahusika wenye watahitajika kutolewa damu ya zaidi wataombwa kushiriki kwa kulaza kiwango chote cha damu kitakacho chukuliwa wakati wa utafiti huu, hi ni mililita 173 (kikombe kizima) kwa zaidi ya muda wa majuma 70. Katika matokeo mengineo utatembelewa mara moja kwa juma na wasaidizi wa kiafya wenye wamehitimu ambao watashirikiana nawe ili upatiwe dawa za utafiti. Watahitaji kutafuta habari kujua hali yako hivyo wata ku uliza "Umekwisha jisikia vibaya tangu nije hapa mara ya mwisho".
Usipojisikia vyema unaweza kutembelea kiliniki kwa utabibu kwa wakati wowote.

Nambari ya mhisani: .... Ufupisho wa mhisani: ....

UHOBELAHOBELA, FICHO PANDE MBILI, VIBANDIKIZO VILIVYO LINGANISHA KATIKA UTAFITI WA TAFENOQUINE WA KILA JUMA (WR 238605/SB252263) KULINGANISHA NA MEFLOQUINE KAMA ANGAMIZO LA *PLASMODIUM FALCIPARUM* MAGHARIBI MWAKENYA

MUHTASARI

Mtu yeyote atakae kujitolea ni lazima atarijie mambo kama yafuatayo

a. Kuchunguzwa maungo na majibu utokeayo kwenye zahanati kuonyesha kwamba mtu huyo ana afya njema na kwamba anaweza kustahimili nguvu za dawa.
b. Kuna dawa ya kumeza upeanwa na daktari ama mmoja wa wafanya kazi wa utafiti huu kwa muda wa siku 3 za kwanza za utafiti.
c. Dawa za kila juma hadi juma 24 zipeanazwo na mfanye kazi wa utafiti huu.
d. Kuyajibu maswali baada ya kila juma ili kuchunguza kasoro kuhusiana na afya yako kujua ni jambo lipi linaweza kuwa limetokea.
e. Kujitokeza kwenye kiliniki ya utafiti kama pengine kuna madhara yeyote kuhusiana na afya yako wakati ukiyameza madawa.
f. Kujitolea kwa kutoa damu mara 15 katika utafiti wote ujumla.
g. Kuchukuliwa sampuli kidogo kutoka kwenye kidole kila juma ili kuamuwa kama malaria imo katika damu yako.
h. Kufuatiliwa na watumishi wa kiafya kwa muda wa majuma 70.

TAHADHARISHO

Chloroquine na madawa mengine yanaweza kudhuru malaria. Ukijiunga katika utafiti huu, ni lazima utakubali kutotumia chloroquine na madawa mengine isipokuwa kwa wakati huo wa utafiti kama daktari amekuandikia dawa hizo ili utumie. Ni lazima ubaki kwenye sehemu ya utafiti wakati wa utafiti huu.

UJA UZITO

Wanawake wote wenye wamependelea kujihusisha na utafiti huu, watapata kupimwa uwezekano wa uja uzito katika muda wa siku mbili za kwanza kabla ya kupewa dawa za tiiba, halofantrine, na kurudiwa kabla kuanza dawa ya utafiti. Wale wasio waja wazito ndio watakao husishwa. Kwa ajili hii inawezekana, lakini kwa bahati mbaya madawa haya yaweza kumdhuru mtoto anaendelea kukua tumboni. Pimo la uwezekano wa uja uzito utakuwepo kila mwezi wakati wa kupewa dawa, kwa kupimwa damu katika kiliniki au mkojo kwa wakati mwingine. Mwanamke yeyote atakae patikana kuwa mja mzito hataruhusiwa kuendelea kutumia madawa ya utafiti. Ni lazima kuepuka kupatikana na mimba hadi majuma 2 baada ya mhula wa pili wa halofantrine yaani majuma 39 baada ya kuhusishwa katika tafiti kabla muhula wa halofantrine, kabla juma ya 37 jaribio zaidi ya uja uzito litatekelizwa. Kuepuka kupata mimba, nilazima uepuke kutumia njia ya kupanga uzazi. Ijapokuwa lile la kuondoa kizazi, upanganji uzazi kama vile kutumia mpira, kizuizi cha diaphragm au kifuniko cha njia ya kizazi, tembe za kupanga uzazi, IUD au maji ya uzazi ya kiume kuuwawa, hizi sio vizuizi kamwe vya kutokupata mimba.

USIRI

Stakabadhi zote zitapewa kampuni ihusikanayo na utengenezaji wa madawa haya lakini kwa majina tofauti na majina zako. Nambari itatumika. Jina lako halitatumika wala halita onekana katika ripoti yeyote kutokana na utafiti huu. Kutizamwa kwa stakabadhi moja kwa moja yaweza kuhitajika na waakilishi wa kampuni ama serikali ya Kenya au serikali ya marekani ihusikanayo na utafiti ama watoa idhini ya uenezaji wa madawa.

Waakilishi watokao katika utafiti wa kimarekani wa jeshi na materiel command wanaweza kukagua stakabadhi za utafiti. Hua ni sheria kwa wa tafiti wa kiafya wajeshi wa marekani kwamba tarakimu zitengenezwe kwa wahisani washiriki, habari hii inahitajika ili kujibu maswali yanao wahusu wahisani washiriki ambao unadhaminiwa na (USAMRMC) na kuakikisha ya kwamba unaweza kujulishwa pindi habari mpya ipatikanapo kuhusu dawa ya utafiti. Hii ni tarakimu ya siri na taarifa iwekwao katika tarakimu hii ni kama ifuatayo;- Jina, anwani, nambari ya kitambulisho, taarifa kamilifu kuhusu utafiti wa kiliniki yenye umehusishwa. Kusudio ya kuweka tarakimu ni kuwezesha kujibiwa kwa maswali katika utafiti huu unaodhaminiwa na (USAMRMC) na

kuhakikisha ya kwamba waweza kupatikana pindi taarifa mpya ipatikanapo kutokana na utafiti huu. Taarifa hii inawekwa kwa muda usiopungua miaka 75.

RIDHAA

Umehalalishiwa matibabu yote muhimu ya kitiba kuhusu kuumia au ugonjwa wowote utokeao kwa ajili ya kushiriki katika utafiti huu.
Kama una hakika kwamba umeumizwa kama chombo cha utafiti ni lazima umuone Dr. Stoute. Atakupa mawaidha ni vipi utafanya na atawasiliana na kampuni ya madawa kwa niaba yako.

Ijapokuwa matibabu peke yanapeanwa, hakuna ridhaa nyingine ilioko kwa kushiriki kwako katika utafiti huu, hata hivyo hii sio nafasi ya kunyimwa ama kupuuzwa kwa haki zako za kisheria.

HAKI ZAKO

Kama wakati wa utafiti huu, una maswali kuhusiana na hali ya utafiti au umepata majeraha yanaohusiana na kushiriki kwako, unaweza kuwasiliana na Daktari Stoute au daktari wa zamu wa kliniki ya utafiti. Mwenyekiti wa Kenya National Ethical Review Committee c/o Kenya Medical Research Institute S.L.P 54840, Nairobi, Kenya, Simu 02 722541 unaweza kuwasiliana naye kuhusu haki zako kama mshiriki katika utafiti huu.

UKIONDOLEWA KUTOKA KATIKA UTAFITI

Utaondolewa kutoka katika utafiti na usimamishwe kutopata tiba bila hiyari yako kama mambo yafuatayo yametokea

1. Kama umepata hali yakutokua na afya njema ambapo kuendelea zaidi katika huu utafiti unaweza kuathirika.
2. Mambo mengine kutokea ambao yanaweza kufanya kushiriki kwako kuto ridhia afya yako. Kama ulemavu au ushahidi utokao katika mahabara kwamba maungo yako hayana nguvu za kustahimili.
3. Ukikosa zaidi ya mara moja ya dawa.
4. Ukiwa mja mzito.

Ukiondoka katika utafiti mapema kabla muda ulio kadiriwa, kulingana na sababu yeyote, utaombwa kurudi katika kliniki kwafatilio imara baada ya majuma 4 baadaye. Hililitahusishwa na kuchukuliwa damu kama vile ifanywayo katika kiliniki zinginezo zenye umekwisha wahi kuhusiswa wakati ukiwa katika utafiti huu, na kuchunguzwa maungo.

KUSHIRIKI KWA HIYARI

Wamuzi wako kushiriki katika utafiti huu ni kwa hiyari na kukataa kushiriki hautaadhibiwa au kupoteza manufaa ambazo ungeweza kujipatia. Kwa kuongezea, unaweza kujiondoa kutoka katika utafiti huu kwa wakati wowote bila kuadhibiwa au kupoteza manufaa ambazo ungeweza kuzipata. Ukiamua kutoka katika utafiti huu ni lazima kurejea kwa daktari ahusikanae na zamu umueleze. Daktari yuko na haki ya kukuondoa katika utafiti huu kwa wakati wowote akionelea ni vyema.

UHOBELAHOBELA, FICHO PANDE MBILI, VIBANDIKIZO VILIVYO LINGANISHA KATIKA UTAFITI WA TAFENOQUINE WA KILA JUMA (WR 238605/SB252263) KULINGANISHA NA MEFLOQUINE KAMA ANGAMIZO LA *PLASMODIUM FALCIPARUM* MAGHARIBI MWAKENYA

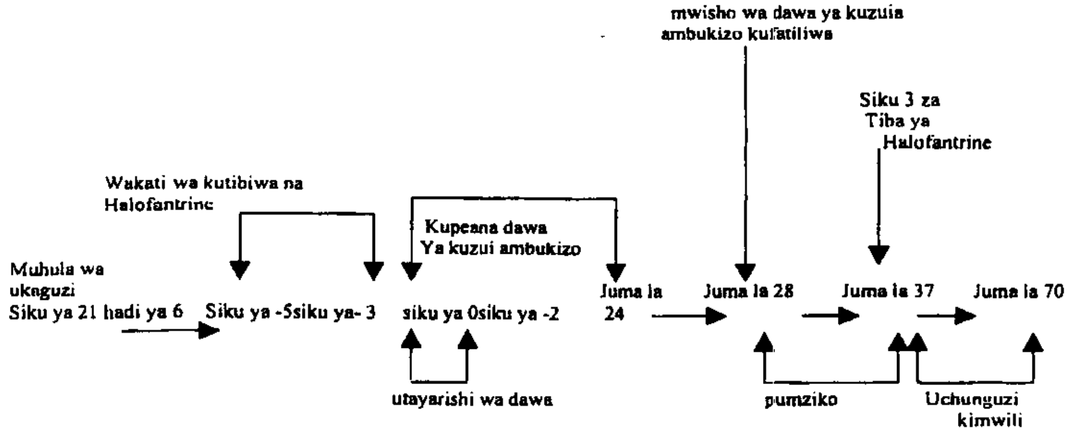

Nambari ya mhisani: .... Ufupisho wa mhisani: ....

UHOBELAHOBELA, FICHO PANDE MBILI, VIBANDIKIZO VILIVYO LINGANISHA KATIKA UTAFITI WA TAFENOQUINE WA KILA JUMA (WR 238605/SB252263) KULINGANISHA NA MEFLOQUINE KAMA ANGAMIZO LA *PLASMODIUM FALCIPARUM* MAGHARIBI MWAKENYA

ANDIKO LA KUKUBALIANA

Jina la Mhisani Kwa Kirefu: ....

Nimesoma habari kuhusu utafiti ambao nimepewa nakuyaelewa mambo muhimu yaliomo. Maswali yote nimeuliza yamejibiwa kikamilifu. Nimepewa mfano wa andiko hili. Nimelewa kwamba niko huru kujiondoa kama mshiriki wakati wowote nipendapo bila madhara yeyote kwa maisha yangu baadaye. Ninafahamu kwamba habari za kutambulisha zitatumiwa ili iwe ni rahisi kwangu kupatikana nitakikapo. Habari hizizaweza kutumiwa kama habari zitokanazo kwangu mimikama chombo cha utafiti na yaeza kusambazwa kwa Federal na local agencies.

Nina Kubali nishirikishwe katika utafiti huu.

SAHIHI YA MHISANI: .... **TAREHE:** ....

Habari za kupatikana (Kijiji): ....

Kwa kuongezaea, niko tayari kuhudhuria kiliniki ili nitolewe sampuli za damu baada ya bandikizo la dawa la tatu na wakati wa juma la 17.
SAHIHI YA MHISANI: .... **TAREHE:** ....

Kwa kuongezea, niko tayari kutoa sampuli za damu wakati wa mwanzo, juma la 38 na juma la 70 ili kupima usugu wa malaria.
SAHIHI YA MHISANI: .... **TAREHE:** ....

SAHIHI YA DAKTARI WA KLINIKI: ....

Jina kamilifu: ....

Tarehe: ....

Sahihi ya Shahidi: ....

Jina la shahidi; ....

Tarehe; ....

Nambari ya mhisani: .... Ufupisho wa mhisani: ....

UHOBELAHOBELA, FICHO PANDE MBILI, VIBANDIKIZO VILIVYO LINGANISHA KATIKA UTAFITI WA TAFENOQUINE WA KILA JUMA (WR 238605/SB252263) KULINGANISHA NA MEFLOQUINE KAMA ANGAMIZO LA *PLASMODIUM FALCIPARUM* MAGHARIBI MWAKENYA

KUBALIANO KWA SHAHIDI KWA KAULI

Nimehudhuria kipindi cha kupeana kubaliano baada ya kujulishwa kuhusu .... ili ahusiswe katika utafiti vile ime elezwa hapo juu. Daktari ameeleza utaratibu na umuhimu wa kwake kama mushiriki katika utafiti huu na ameelewa maswali yote yenye ameuliza yamejibiwa kwa kurithia kwakw na amepewa formu kama hii. Mhisani yuko huru kujiondoa wakati wowote bila madhara yeyote kwake katika maisha yake ya baadaye.

SAHIHI YA SHAHIDI: ....

Jina kwa kirefu: ....

Tarehe: ....

Gumba kuonyesha kutoelewa kusoma

Kwa kuongezea, yeye yuko tayari kuhudhuria kiliniki na kutoa sampuli ya damu baada ya Ubandikizo wa dawa ya tatu na katika juma ya 17

Sahihi ya shahidi: .... **Tarehe;** ....

Kwa kuongezea yeye yuko tayari kutoa sampuli ya damu wakati wa mwanzo, juma la 38 na juma la 70 ili kutizama usugu wa malaria.

Sahihi ya shahidi: .... **Tarehe;** ....

Nimefafanulia huyu mhisani vilivyo na ameelewa vipengele vyote. Maswali yote yenye ameuliza yamejibiwa kikamilifu. Nimempa mfano wa fomu ya ukubaliano. Mhisani anaelewa kwamba yeye yuko huru kujiondoa kutoka katika utafiti wakati wowote bila madhara kwake kwa maisha yake ya baadaye.

SAHIHI YA DAKTARI WA UTAFITI HUU: ....

Jina kwa Kirefu: ....

Tarehe: ....

DEPARTMENT OF HEALTH AND HUMAN SERVICES
PUBLIC HEALTH SERVICE
FOOD AND DRUG ADMINISTRATION

STATEMENT OF INVESTIGATOR

(TITLE 21, CODE OF FEDERAL REGULATIONS (CFR) PART 312)
(See instructions on reverse side.)

Form Approved: OMB No. 0910-0014.
Expiration Date: September 30, 2002.
See OMB Statement on Reverse.

NOTE: No investigator may participate in an investigation until he/she provides the sponsor with a completed, signed Statement of Investigator, Form FDA 1572 (21 CFR 312.53(c)).

1. NAME AND ADDRESS OF INVESTIGATOR

LTC Jose A. Stoute MC
U.S. Army Medical Research Unit, Kenya
Unit 64109, Box 401
APO AE 09831-4109

2. EDUCATION, TRAINING, AND EXPERIENCE THAT QUALIFIES THE INVESTIGATOR AS AN EXPERT IN THE CLINICAL INVESTIGATION OF THE DRUG FOR THE USE UNDER INVESTIGATION. ONE OF THE FOLLOWING IS ATTACHED.

☒ CURRICULUM VITAE ☐ OTHER STATEMENT OF QUALIFICATIONS

3. NAME AND ADDRESS OF ANY MEDICAL SCHOOL, HOSPITAL OR OTHER RESEARCH FACILITY WHERE THE CLINICAL INVESTIGATION(S) WILL BE CONDUCTED.

U.S. Army Medical Research Unit, Kenya
Unit 64109, Box 401
APO AE 09831-4109

International Address: American Embassy
ATTN: MRU Dr. Stoute
P.O. Box 30137
Nairobi, Kenya

4. NAME AND ADDRESS OF ANY CLINICAL LABORATORY FACILITIES TO BE USED IN THE STUDY.

Walter Reed Project Kombewa Clinic
P.O. Box 54
Kisumu, Kenya

5. NAME AND ADDRESS OF THE INSTITUTIONAL REVIEW BOARD (IRB) THAT IS RESPONSIBLE FOR REVIEW AND APPROVAL OF THE STUDY(IES).

Kenya Medical Research Institute
KEMRI/National Ethical Review Committee
P.O. Box 54840
Nairobi, Kenya

U.S. Army Human Subjects Research Review Board
504 Scott Street
Fort Detrick, MD 21702-5009

6. NAMES OF THE SUBINVESTIGATORS (*e.g., research fellows, residents, associates*) WHO WILL BE ASSISTING THE INVESTIGATOR IN THE CONDUCT OF THE INVESTIGATION(S).

Dr. G. Dennis Shanks
Dr. Carl Mason
Jon Davis
Dr. Robert Gasser
Dr. Kimberly Moran

7. NAME AND CODE NUMBER, IF ANY, OF THE PROTOCOL(S) IN THE IND FOR THE STUDY(IES) TO BE CONDUCTED BY THE INVESTIGATOR.

A Randomized, Double-Blind, Placebo Controlled Evaluation of Weekly Tafenoquine (WR 238605/SB252263) Compared to Mefloquine for Chemosuppression of Plasmodium falciparum in Western Kenya (Log No. A-9467)

Created by: PSC Media Arts Branch (301) 443-2454 EF

8. ATTACH THE FOLLOWING CLINICAL PROTOCOL INFORMATION:

☐ FOR PHASE 1 INVESTIGATIONS, A GENERAL OUTLINE OF THE PLANNED INVESTIGATION INCLUDING THE ESTIMATED DURATION OF THE STUDY AND THE MAXIMUM NUMBER OF SUBJECTS THAT WILL BE INVOLVED.

☐ FOR PHASE 2 OR 3 INVESTIGATIONS, AN OUTLINE OF THE STUDY PROTOCOL INCLUDING AN APPROXIMATION OF THE NUMBER OF SUBJECTS TO BE TREATED WITH THE DRUG AND THE NUMBER TO BE EMPLOYED AS CONTROLS, IF ANY; THE CLINICAL USES TO BE INVESTIGATED; CHARACTERISTICS OF SUBJECTS BY AGE, SEX, AND CONDITION; THE KIND OF CLINICAL OBSERVATIONS AND LABORATORY TESTS TO BE CONDUCTED; THE ESTIMATED DURATION OF THE STUDY; AND COPIES OR A DESCRIPTION OF CASE REPORT FORMS TO BE USED.

9. COMMITMENTS:

I agree to conduct the study(ies) in accordance with the relevant, current protocol(s) and will only make changes in a protocol after notifying the sponsor, except when necessary to protect the safety, rights, or welfare of subjects.

I agree to personally conduct or supervise the described investigation(s).

I agree to inform any patients, or any persons used as controls, that the drugs are being used for investigational purposes and I will ensure that the requirements relating to obtaining informed consent in 21 CFR Part 50 and institutional review board (IRB) review and approval in 21 CFR Part 56 are met.

I agree to report to the sponsor adverse experiences that occur in the course of the investigation(s) in accordance with 21 CFR 312.64.

I have read and understand the information in the investigator's brochure, including the potential risks and side effects of the drug.

I agree to ensure that all associates, colleagues, and employees assisting in the conduct of the study(ies) are informed about their obligations in meeting the above commitments.

I agree to maintain adequate and accurate records in accordance with 21 CFR 312.62 and to make those records available for inspection in accordance with 21 CFR 312.68.

I will ensure that an IRB that complies with the requirements of 21 CFR Part 56 will be responsible for the initial and continuing review and approval of the clinical investigation. I also agree to promptly report to the IRB all changes in the research activity and all unanticipated problems involving risks to human subjects or others. Additionally, I will not make any changes in the research without IRB approval, except where necessary to eliminate apparent immediate hazards to human subjects.

I agree to comply with all other requirements regarding the obligations of clinical investigators and all other pertinent requirements in 21 CFR Part 312.

INSTRUCTIONS FOR COMPLETING FORM FDA 1572
STATEMENT OF INVESTIGATOR:

1. Complete all sections. Attach a separate page if additional space is needed.
2. Attach curriculum vitae or other statement of qualifications as described in Section 2.
3. Attach protocol outline as described in Section 8.
4. Sign and date below.
5. FORWARD THE COMPLETED FORM AND ATTACHMENTS TO THE SPONSOR. The sponsor will incorporate this information along with other technical data into an Investigational New Drug Application (IND).

10. SIGNATURE OF INVESTIGATOR	11. DATE
[signature]	2/18/00

(WARNING: A willfully false statement is a criminal offense. U.S.C. Title 18, Sec. 1001.)

Public reporting burden for this collection of information is estimated to average 100 hours per response, including the time for reviewing instructions, searching existing data sources, gathering and maintaining the data needed, and completing reviewing the collection of information. Send comments regarding this burden estimate or any other aspect of this collection of information, including suggestions for reducing this burden, to:

Food and Drug Administration<br>CBER (HFM-99)<br>1401 Rockville Pike<br>Rockville, MD 20852-1448	Food and Drug Administration<br>CDER (HFD-94)<br>5516 Nicholson Lane<br>Kensington, MD 20895	"An agency may not conduct or sponsor, and a person is not required to respond to, a collection of information unless it displays a currently valid OMB control number."

Please DO NOT RETURN this application to this address.

Curriculum Vitae

Name: Jose' A. Stoute, M.D.

Current Position: Chief, Malaria Immunology and Vaccine Research
US Army Medical Research Unit
KEMRI
Unit 64109 Box 401
APO AE 09831-4109
Tel 254-35-22942
Fax 254-35-22903
e-mail: stoutej@net2000ke.com

Personal:
Date of Birth: 4 April 1961
SSN: 266-81-7794
Marital Status: Married
Spouse: Ellen J. Stoute
Children: Julian A. Stoute, Gabriel M. Stoute
Weight: 150 lbs.

Civilian and Professional Education:

1978-1981	B.A. in Biology, University of South Florida Tampa, Florida.
1981-1985	M.D. Degree, University of Miami School of Medicine, Miami, Florida.
1985-1988	Residency training in Internal Medicine Emory University of Medicine affiliated hospitals, Atlanta, Georgia.
1991-1994	Fellowship in Infectious Diseases, Walter Reed Army Medical Center, Washington, DC.

Military Education:

1982	AMMED Officer Basic Course, Ft. Jackson, SC.
1985	Airborne Course, Ft. Benning, GA.
1989	Expert Field Medical Badge, Ft. Hood, Texas.
1989	AMMED Advanced Officer Course, 9/89.

Military Decorations:

1991	Meritorious Service Medal for Exceptional Duty Performance while stationed at Ft. Hood, Texas, July, 1988 through June, 1991.
1991	Army Commendation Medal for Service in the Persian Gulf War, Sept. 1990-April, 1991.
1991	Saudi Arabia Defense Medal for service in the Persian Gulf War, Sept. 1990-April, 1991.
1991	Kuwait Liberation Medal for service in the Persian Gulf War, Sept. 1990-

	April,1991.
1991	Combat Field Medic Badge for service in the Persian Gulf War.
1994	Army Commendation Medal for outstanding research while assigned as Infectioius Disease Fellow, Walter Reed Army Medical Center.

Honors and Other Awards:

1980 - Received Dr. Sylvan Myer's Award for Outstanding Achievement in Physics and Mathematics, University of South Florida.
1981 - Finished undergraduate studies as Magna Cum Laude.
1985 - Received M.D. degree with an additional distinction for scientific research performed.
1994 - Bailey K. Ashford Clinical Research Award, Walter Reed Army Medical Center
1998 - WRAIR Association, Edward L. Buescher Young Scientist Award

Professional Societies:

Member of the American College of Physicians.
Member of the American Society of Tropical Medicine and Hygiene.

Professional Certifications:

1988 - Diplomate of the American Board of Internal Medicine.
1994- Diplomate of the American Board of Internal Medicine in the Subspecialty of Infectious Diseases.

Military and Professional Assignments:

1988-1991	Staff, General Internal Medicine, Darnall Army Community Hospital, Ft. Hood, TX. Chairman, Critical Care Committee and Member, Infection Control Committee.
1990-1991	Battalion Surgeon, Task Force 1-8, 1st Cavalry Division, Persian Gulf War.
1991-1994	Infectious Disease Fellow, Walter Reed Army Medical Center, Washington, DC.
1994-Present	Infectious Disease Officer, Department of Immunology, Walter Reed Army Institute of Research, Washington, DC.
1994-Present	Staff, Infectious Disease Service, Walter Reed Army Medical Center, Washington, DC.
1994-Present	Assistant Professor, Internal Medicine, Uniformed Services University of Health Sciences, Bethesda, Maryland.

Research Fellowships:

1979, 1980 - Recipient, American Cancer Society Summer Research Fellowship for undergraduate students.

Research Experience:

1979 - 1980 Isolation of Histone mRNA from HeLa cells. Isolation of globin mRNA from rabbit reticulocytes. University of South Florida, Research Advisor: Lee A. Weber, Ph.D.

1980-1981 Study of the rRNA transcriptional regulation by the DNA binding proteins HMG-1,2,14, and 17. Florida State University, Tallhassee, FL. Research Advisor: William F. Marzluff, PhD.

1981 Electron microscopy study of intracellular changes in HeLa cells during heat shock. University of South Florida, Research Director: Lee A. Weber, Ph.D.

1985 Development of novel technique for the characterization of HSP70 promoter binding factors. University of Miami, School of Medicine. Research Director: Richard Voellmy, Ph.D.

1991-Present: Use of phage epitope library for the discovery of B-cell epitopes of potential use in vaccine development.

1996-Present Identification of fibronectin binding activity of *Plasmodium falciparum* malaria.

Abstracts:

1981- **Jose' A. Stoute** and William F. Marzluff. HMG Proteins are Necessary for Transcription.. The 4th Annual Cancer Research Seminar, American Cancer Society and University of South Florida, College of Medicine, February 14, 1981, Tampa, Florida.

1982- **Jose' A. Stoute** and William F. Marzluff. HMG Proteins 1 and 2 are Required for Transcription. The 14th Miami Winter Symposium, 11-15 January, 1982, Miami, Florida.

1993- **J. A. Stoute**, W.R. Ballou, N. Kolodny, C. Deal R.A. Wirtz, and L.E. Lindler Use of a Phage Library to Identify Epitopes that Mimic a Native Antigen of *Plasmodium falciparum*: Utility in the Construction of a Potential Synthetic Malaria Vaccine.. Infectious Disease Society of America, Oct. 16-18, 1993, New Orleans, LA.

1993- Cohen SJ*, Lindler LE, **Stoute JA**, and Klotz FW. Epitope Specificity of a Mouse Monoclonal Antibody Against a 50 KDa Maurer's Cleft-Associated Antigen of *Plasmodium falciparum* malaria. Presented at the American Society of Tropical Medicine and Hygiene and the American Society of Parasitologists meeting, October 31 - November 4, 1993.

1994- Drabick JJ, **Stoute J**, Lindler L, Gordon D, Sadoff J, Ballou WR. Epitope mapping of a functional human Mab against Plasmodium falciparum using a random hexapeptide filamentous phage expression library. Clin Res 1994; 42(2): 283A.

1996- Palmer D, **Stoute J**, and Krzych U. T cells Responses in Malaria: Requirements for Maintenance of Memory, Proliferation and Cell Death. Presented at the the American Society of Tropical Medicine and Hygiene. December 1-5, 1996, Baltimore, MD.

1996- **Stoute JA**, Heppner DG, Schwenk R et al. Safety, Immunogenicity and Efficacy of a Plasmodium falciparum Circumsporozoite Protein-Hepatitis B Surface Antigen Subunit Vaccine (RTS,S). Presented at the American Society of Tropical Medicine and Hygiene. December 1-5, 1996, Baltimore, MD.

1996- Heppner DG, Ngampochjana M, Tongtane P, **Stoute J**, Kester K, Kyle DE, Slaou M, Voet P, Pall P, Ballou WR, and Cohen J. Use of a Rhesus Model to Optimize Formulations and Dosing Regimens of the Candidate Falciparum Malaria Vaccine RTS,S. Presented at the 45th Annual Meeting of the American Society of Tropical Medicine and Hygiene. December 1-5, 1996, Baltimore, MD.

1997- Palmer D, **Stoute J** and Krzych U. Vaccination with Plasmodium falciparum Irradiated Sporozoites Induces Memory T Cell Responses. Presented at the meeting Fundamental Issues in Vaccine Immunity, Jenner Institute for Vaccine Rsearch, April 1997, Oxford, UK.

1997- Evaluation of Cellular Responses Induced by Recombinant Plasmodium falciparum circumsporozoite protein RTS,S Vaccine. Krzych U, Schwenk R, White KL, Malik A, Sun P, Kolodny N, Lalvani A, Hill A V, **Stoute JA**, et al. 46th Annual Meeting of the American Society of Tropical Medicine and Hygiene. Orlando, Florida, 1997.

1997- Mapping of Fine Antigen Specificities of INFG-Producing T Lymphocytes Induced by Recombinant Plasmodium falciparum Circumsporozoite Protein RTS,S Vaccine. Sun P, Schwenk R, White KL, Lalvani A, Hill AV, Kolodny N, Stoute JA, et al. 46th Annual Meeting of the American Society of Tropical Medicine and Hygiene. Orlando, Florida, 1997.

1997- Binding of Fibronectin to Plasmodium falciparum-infected Red Cells. **Stoute JA**, Ware LA, Lanar DE, Ockenhouse CF. 46th Annual Meeting of the American Society of Tropical Medicine and Hygiene. Orlando, Florida, 1997.

1997- High Level Expression, Purification and Immunological Analysis of Plasmodium falciparum CS Protein. Lanar DE, Abbott MR, De Armas VM, Miller VL, **Stoute JA**, Kester KE, Sun P, and Krzych U. 46th Annual Meeting of the American Society of Tropical Medicine and Hygiene. Orlando, Florida, 1997.

1997- Fibronectin Binding Activity of Plasmodium falciparum GRP 78 Protein. Ware LA, Lanar DE, Hansen B, and **Stoute JA.** 46th Annual Meeting of the American Society of Tropical Medicine and Hygiene. Orlando, Florida, 1997.

02/17/00 THU 12:27 [TX/RX NO 7033]

1999 Safety and Immunogenicity of the lyophilized RTS,S/SBAS2 Malaria Vaccine in a Malaria-Experienced Adult Population of Western Kenya. **Stoute JA**, Mason C, Siangla J, Opollo M, Vigneron L, Walter M, Kester K, Heppner DG, Tornieporth N, Voss G, Ballou WR, Cohen J.

1999 In vitro cytokine stimulation profile in patients with *Plasmodium falciparum* infection and severe anemia compared to age-matched controls. Siangla J and **Stoute JA.** Multilateral Initiative on Malaria Meeting, Durban, South Africa, March 14-19, 1999.

1999 Measurement of Red Cell Surface IgG, CR1, CD55, and CD59 in Children with Severe Anemia Due to *Plasmodium falciparum* Malaria. Opollo M, Waitumbi JN and **Stoute JA.** Multilateral Initiative on Malaria Meeting, Durban, South Africa, March 14-19, 1999.

1999 Susceptibility of Red Blood Cells from Children with Severe Plasmodium falciparum Anemia and Age Matched Controls to Erythrophagocytosis. Waitumbi JN and Stoute JA. Multilateral Initiative on Malaria Meeting, Durban, South Africa, March 14-19, 1999.

Seminars and Symposia:

1992- Determination of Epitopes Using a Phage Library. US/Israeli Defense Forces Medical Research Conference, November 1-6,1992, Annapolis, Maryland.

1994- Searching for Peptides that are Antigenic and Immunogenic Mimics of Malarial Antigens by Use of a Phage Epitope Library. SmithKline Beecham, Pennsylvania, June 29, 1994.

1996- Evaluation of Candidate Pre-erythrocytic Malaria Vaccines. Given at the 45th Annual Meeting of the American Society of Tropical Medicine and Hygiene. December 1-5, 1996, Baltimore, MD.

1997- A New Vaccine for Malaria. Given at the XXXIII Congress of the Brazilian Society of Tropical Medicine, Belo Horizonte, Brazil 24-27 February, 1997.

Publications:

1. **Jose' A. Stoute** and William F. Marzluff. HMG-Proteins 1 and 2 are Required for Transcription of Chromatin by Endogenous RNA Polymerase. Biochemical and Biophysical Research Communications, 107(4),1279-1284,1982.

2. Michael Allon, Wallace G. Campbell, Sherif A. Nasr, Edmund Bourke, **Jose' Stoute**, and Jayarama Guntupalli. Minimal Change Glomerulonephropathy and Interstitial Infiltration with Mycosis Fungoides. American Journal of Medicine, 84,756-758, 1988.

3. **Jose' A. Stoute** and W. Dallas Hall. Treatment of Hypertension in the Psychiatric Population. Review. Emory Journal of Medicine. 4(1):19-24,1990.

4. Michael W.Cannon, **Jose' A. Stoute**, and Lanny Stone. The Battalion Medical Platoon: A Flawed Scalpel. Military Medicine 157(8):414-416,1992.

5. **Jose' A.Stoute,** W. Ripley Ballou, Robert A. Wirtz, Nelly Kolodny, Caroline D. Deal, and Luther Lindler. Induction of Humoral Immune Response Against *P. falciparum* Sporozoites by Immunization with a Peptide Mimotope Whose Sequence was Derived from Screening Screening a Phage Epitope Library. Infection and Immunity. 63(3):9334-939,1995.

6. **José A. Stoute** et al. A Preliminary Evaluation of a Recombinant Circumsporozoite Protein Vaccine Against *Plasmodium falciparum* Malaria. New England Journal of Medicine 336:86-91,1997.

7. Ockenhouse CF, Sun PF, Lanar DE, Wellde BT, Hall BT, **Stoute JA**, et al. Phase I/IIa Safety, Immunogenicity and Efficacy of NYVAC-Pf7, a Pox-vectored, multiantigen, multistage vaccine candidate for *Plasmodium falciparum* malaria. Submitted

8. **José A. Stoute** and W. Ripley Ballou Malaria Vaccines: Current Status. BioDrugs. 10(2):123-136, 1998..

9. **J. A. Stoute** et al. Long Term Efficacy and Immune Responses Following Immunization with the RTS,S Malaria Vaccine. JID 178(4):1139-1144.

10. Morikawa SJ, Cortese LM, Walker JS, Oster CN and **Stoute JA**. The Risk of Hypersensitivity Reactions to Trimethoprim/Sulfamethoxazole as a Function of the CD4+ Count in HIV-infected Patients. J Pharm Technol *In Press.*

11. Waitumbi JN, Opollo M, Misore A, Muga R, **Stoute JA.** Red Cell Surface Changes and Erythrophagocytosis in Children with Severe Plasmodium falciparum Anemia. Blood *In Press.*

Research Interests:

Malaria Molecular Biology:

Parasitization of the red cell by *P. falciparum* malaria leads to changes on the surface of the cell which are the result of the expression of parasite-derived antigens as well as the modification of host antigens by the organism. The identification of these antigens is important because they are likely vital for the survival of the parasite and its interaction with the host. These antigens may be important candidates for vaccine development. As part of this effort, I have recently identified a parasite antigen that binds to host fibronectin. Further studies in this area will concentrate in determining what role fibronectin has in the pathogenesis of malaria and identification of other malaria that may interact with fibronectin.

Malaria Pathogenesis:

Most of the mortality and morbidity from *P. falciparum* malaria is the result of complications such as cerebral malaria and malarial anemia. The pathogenesis of these syndromes is poorly understood. In particular, it is not clear why most individuals do not develop these complications while a significant minority does. I am interested in identifying the mechanisms accelerated destruction of uninfected red blood cells during malaria infection and determining whether these mechanisms are more active in severe malarial anemia patients than in milder cases of malaria. The identification of how severe anemia takes place during malaria infection could lead to new forms of treatment and prevention of complications.

KENYA MEDICAL RESEARCH INSTITUTE

P.O. Box 54840, Tel: (02) 722541, Fax: (02) 720030, KEMRI, NAIROBI, Kenya, E-mail: Kemrilib@ken.healthnet.org

KEMRI/RES/7/3/1

26th November, 1999

Dr. J. Stoute
WRP

Thro'
Director
WRP

RE: **SSC Protocol No. 462 "A Radomized Double Blind placebo controlled Evaluation of weekly Tafenoquine (WR 238605) compared to mefloquine for chemosuppression of *Plasmodium Falciparum* in Western Kenya" by J.Stoute et al**

This is to inform you that during the 80th meeting of the KEMRI/National Ethical Review Committee held on Tuesday 23rd November 1999, the above protocol was granted approval for you to embark on your studies.

G.A.O.SEKO
for: SECRETARY,
KEMRI/NATIONAL ETHICAL REVIEW COMMITTEE

5:29 November 1999

Date

ROUTING AND TRANSMITTAL SLIP

18 November 1999

TO: (Name, office symbol, room number, building, Agency/Post)	Initials	Date
1. Jonathan Berman, COL, MC, Chief, Department of Biology, Division of Experimental Therapeutics	JB	19 Nov 99
2. Sara W. Rothman, Ph.D., Director, Office of Research Management		
3.		
4.		
5.		

	Action		File		Note and Return
X	Approval		For Clearance		Per Conversation
	As Requested		For Correction		Prepare Reply
	Circulate		For Your Information		See Me
	Comment		Investigate		Signature
	Coordination		Justify		

REMARKS

As Chairman of the Scientific Review Committee for WRAIR human use protocol #663, please review the following modification and signify your approval/disapproval.

APPROVAL/DISAPPROVAL

JONATHAN BERMAN
COL, MC
Chief, Department of Biology
Division of Experimental Therapeutics

DO NOT use this form as a RECORD of approvals, concurrences, disposals, clearances, and similar actions

FROM: (Name, org. symbol, Agency/Post)	Room No.-Bldg.
for Denise Davis<br>Sara W. Rothman, Ph.D., Director, Office of Research Management	Rm. 1W30 Bldg. 503
	Phone No. (301) 319-9940/ (301) 319-9961 fax

DOD EXCEPTION TO OF41
APPROVED BY GSA/IRMS 4/89

OPTIONAL FORM 41 (Rev. 7-76)
Prescribed by GSA
FPMR (41 CFR) 101-11.206

USAPPC V3.00

12/14/99 TUE 15:31 [TX/RX NO 6606]

MEMORANDUM FOR OFFICE RESEARCH MANAGEMENT (DR ROTHMAN)

MEMORANDUM FROM CHAIR, SCIENTIFIC REVIEW COMMITTEE (COL BERMAN)

SUBJECT: Review of revised protocol WRAIR 663 27 Dec 99

["A Randomized, Double Blind, Placebo Controlled Evaluation of Weekly Tafenoquine (WR 238605/SB252263) Compared to Mefloquine for Chemosuppression of *Plasmodium falciparum* in Western Kenya"] submitted by Jose A. Stoute, LTC, MC.

1. Revised protocol has been reviewed.

2. Protocol is accepted.

J Berman
COL MC
Chair, Review Committee

DEPARTMENT OF THE ARMY
WALTER REED ARMY INSTITUTE OF RESEARCH
WALTER REED ARMY MEDICAL CENTER
WASHINGTON, D.C. 20307-5100

REPLY TO
ATTENTION OF

MCMR-UWZ-C (5-14a) 29 November 1999

MEMORANDUM THRU

Deputy Director, Walter Reed Army Institute of Research, Washington, D.C. 20307-5100

Commander, USAMRU-Kenya, Nairobi/MRU, APO AE 09831-4109

FOR G. Dennis Shanks, COL, MC, USAMRU-Kenya, Nairobi/MRU, APO AE 09831-4109

SUBJECT: Modification of a Human Use Protocol: "A Randomized Double Blind, Placebo Controlled Evaluation of Weekly Tafenoquine (WR 238605) Compared to Mefloquine for Chemosuppression of *P. falciparum* in Western Kenya" (WRAIR #663, Log #A-7944)

1. This modification to the referenced human use protocol and supporting information have been submitted and administratively reviewed in accordance with AR 70-25 and WRAIR Policy Letter 98-07.

2. This modification proposes to change the study site (20 km difference), update the investigator list, and lower the weekly dosage of Tafenoquine from 400 mg to 200 mg. This modification was scientifically reviewed and approved on 19 November 1999. The Chair of the WRAIR Human Use Review Committee recommended approval on 24 November 1999, as posing no greater risk to volunteers than the original protocol.

3. Authority is, therefore, granted to implement this modification.

MARTIN H. CRUMRINE
COL, MS
Director

CF:
HSP, MCMR-RCQ-HR

MCMR-UWZ-C 24 November 1999

MEMORANDUM FOR Office of Research Management, Walter Reed Army Institute of Research, ATTN: Dr. Sara Rothman, Washington, DC 20307-5100

SUBJECT: Human Use Protocol Modification (dated 18 October 1999)

I have read the modification of the human use protocol entitled, "A Randomized Double Blind, Placebo Controlled Evaluation of Weekly Tafenoquine (WR 238605) Compared to Mefloquine for Chemosuppression of *P. falciparum* in Western Kenya" (WRAIR #663, Log #A-7944), submitted by G. Dennis Shanks, COL, MC, USAMRU-Kenya, and recommend approval.

This modification proposes to change the study site (20 km difference), update the investigator list, and lower the weekly dosage of Tafenoquine from 400 mg to 200 mg. This modification poses no greater risk to volunteers than the original protocol.

Frank B. Johnson

FRANK B. JOHNSON, M.D.
Dept. Chemical Pathology, AFIP
Chairman, WRAIR Human Use
Review Committee

DEPARTMENT OF THE ARMY
WALTER REED ARMY INSTITUTE OF RESEARCH
UNITED STATES ARMY MEDICAL RESEARCH UNIT-KENYA
UNIT 64109 BOX 401, APO AE 09831-4109

RECEIVED NOV 3 [illegible]

18 Oct 99

MEMORANDUM FOR OFFICE OF RESEARCH MANAGEMENT, WRAIR

THRU: Commander, USAMRU-Kenya

SUBJECT: Modifications to WRAIR 663, HSRRB A-7944

1. Attached you will find a revised copy of WRAIR 663 "A Randomized, Double Blind, Placebo Controlled Evaluation of Weekly Tafenoquine(WR 238605) Compared to Mefloquine for Chemosuppression of *Plasmodium falciparum* in Western Kenya"

2. Although we were not able to execute the protocol in 1998 as planned, Dr Jose Stoute and I believe we will be able to execute the study in a similar study site in 2000.

3. This protocol has been updated to reflect the change in study site (20 km difference), and different investigators.

4. In all scientifically important respects, this is the same protocol. The drugs (tafenoquine is a name change but it is the same drug that was previously known as etaquine), comparators, placebo and route of administration have not changed. The proposed dose of tafenoquine to be used has been lowered from 400 mg base to 200 mg base weekly based on additional information available since the original protocol was written.

5. There are no significant changes in human use aspects. The amount of blood drawn is substantially the same. The major benefit to the volunteers (medical care) is continued for a much longer time than originally proposed. The major risk to the volunteers (use of an investigational drug) has decreased over time due to the extra 2000 person's experience with tafenoquine that has gained since the protocol was written in 1998.

6. Request that the attached protocol be administratively approved as an amendment to an existing approved protocol.

G. Dennis Shanks
COL, MC, US Army
US Army Medical Research Unit-Kenya

DEPARTMENT OF THE ARMY
OFFICE OF THE SURGEON GENERAL
5109 LEESBURG PIKE
FALLS CHURCH VA 22041-3258

REPLY TO
ATTENTION OF

MCMR-RCQ (70-1n) 30 March 2000

MEMORANDUM FOR Director, Walter Reed Army Institute of Research, ATTN:
MCMR-UWZ-C (Dr. Rothman), Forest Glen Annex,
Building 503, Room 1W30, Washington, DC 20307-5100

SUBJECT: Protocol Entitled, "A Randomized, Double-Blind, Placebo Controlled Evaluation of Weekly Tafenoquine (WR 23865/SB252263) Compared to Mefloquine for Chemosuppression of *Plasmodium falciparum* in Western Kenya" Submitted by Jose Stoute, LTC, MC, WRAIR/USAMRU-K, HSRRB Log No. A-9467

1. Revisions to the subject protocol received on 28 March 2000 have been reviewed and found to comply with recommendations made at the 8 March 2000 meeting of The Surgeon General's Human Subjects Research Review Board.

2. The study is approved for implementation. Approval is granted with the understanding that the certificate of translation for the consent form will be received in this office within one month from the date of this memorandum.

3. Point of contact for this action is Ms. Kathie Mantine at DSN 343-2809, facsimile DSN 343-7803, or e-mail kathie.mantine@det.amedd.army.mil.

Julie K. Zadinsky

JULIE K. ZADINSKY
COL, AN
Acting Chair, Human Subjects
Research Review Board

CF:
USAMRMC, ATTN: MCMR-SGS
WRAIR/USAMRU-K, ATTN: LTC Stoute
USAMMDA, ATTN: MCMR-UMP

Printed on Recycled Paper

Protocol	Centre Number	Subject Number	Subject Initials
252263/030		0 0 0 0 0	

CONFIDENTIAL

Annotated
CRF
030

PROTOCOL 252263/030

SCREENING TO WEEK 28

A RANDOMISED, DOUBLE BLIND, PLACEBO CONTROLLED EVALUATION OF WEEKLY TAFENOQUINE (WR 238605/SB252263) COMPARED TO MEFLOQUINE FOR CHEMOSUPPRESSION OF PLASMODIUM FALCIPARUM IN WESTERN KENYA

SCREENING

(DAY -21 TO DAY -6)

MODULE 1

OBS: 1000.SCR
VISIT: SCREENING

00000

Page	1

Protocol		Subject Number	Subject Initials	Visit Date (Day / Month / Year)	Screening (Day-21 to -6)
252263/030		0 0 0 0 0			

DEMOGRAPHY

DEMOG <DEMOG2>

Date of Birth: DOB (Day / Month / Year)

Gender SEX <XSEX>
- M Male
- F Female

Race RACE <XRACE>
- B Black
- C White
- E Oriental
- O Other → Specify : RACETXT

VITAL SIGNS

ANYYN <XYESNO> VITALS <VITALS5>

VPARAM <XVPARAM> VUNIT <XVUNIT>

Weight WT (kg) KG	Pulse Rate PUL beats/min BPM	Sitting Blood Pressure (mmHg) Systolic SYS / Diastolic DIA	Temperature (Oral) TMP °C C	
VALUE . VALUET		/	.	

PREGNANCY TEST

ANYYN <XYESNO> PSTAT <PSTAT1 PREG>

This test should be completed no more than 48 hours before starting Halofantrine treatment.

PREG1 Is the patient a female of child-bearing potential? PSTATQ <XPSTATAL>

- N No ANSYN <XYESNO>
- Y Yes → *If 'Yes' please complete a pregnancy test (serum or urine) and record the results below*

PREG2 Date performed : ANSDAT (Day Month Yr)

PREG3 Is the patient pregnant?

- N No ANSYN <XYESNO>
- Y Yes → *If 'Yes' please withdraw the patient from the study, enter details in the Adverse Experience section. Complete the Study Conclusion Section.*

G6PD - TEST 1

ANYYN <XYESNO> LAB <LAB7 T1>

The patient should be tested for G6PD twice (from two different blood samples) during the Screening period (day -21 to day -6). The patient must have a result of "Normal" on both occasions to be eligible for the study.

Test 1 : LPARAM <XLPARAM> = G6PD

Date performed : ASSYDAT (Day Month Yr)

Result : VALUET
- Low → *Patient is not eligible for the study*
- Intermediate → *Patient is not eligible for the study*
- Normal

OBS: 1000.SCR

VISIT: SCREENING

00000

Page	2

Protocol 252263/030		Subject Number	Subject Initials		Screening (Day-21 to -6)
		0 0 0 0 0			

G6PD - TEST 2

LAB <LAB7 T2> [SEE PAGE 1]

The patient should be tested for G6PD twice (from two different blood samples) during the Screening period (day -21 to day -6). The patient must have a result of "Normal" on both occasions to be eligible for the study.

Test 2 : Date performed : ☐☐/☐☐☐/☐☐ Day Month Yr

Result :
- ☐ Low ⟶ *Patient is not eligible for the study*
- ☐ Intermediate ⟶ *Patient is not eligible for the study*
- ☐ Normal

PARASITAEMIA

ANYYN <XYESNO> PARASIT <PARASIT2>

(2) Was a finger prick smear taken ? PARASQ <XPARASQ>

- N No PARASA <XYESNO> [PARASC] (<XPARASC> = XYESNO).
- Y Yes ⟶ Sample Date PERFDAT Day Month Yr

(3) Parasitaemia present ? PARASQ <XPARASQ>

- N No PARASA <XYESNO> [PARASC] <XPARASC= XYESNO>
- Y Yes ⟶ Results : (4) PARASQ
  - 1 P. Falciparum
  - 2 P. Malariae PARASA <XPARASC> [PARASC] <XPLASMOD>
  - 3 P. Ovale
  - 4 P. VIVAX

HISTORY OF MALARIA

ANYYN <XYESNO> PSTAT <PSTAT1HIS>

Has the patient ever had malaria ? PSTATQ (HISM1)

- N No ANSYN <XYESNO>
- Y Yes ⟶ (HISM2) Number of attacks in the last six months ☐☐ ANSTXT PSTATQ <XPSTATAL>

(HISM3) When was the last attack : ANSDAT Month Year PSTATQ <XPSTATAL>

OBS : 1000.SCR

VISIT : SCREENING

00000

Page	3

Protocol 252263/030		Subject Number	Subject Initials		Screening (Day-21 to -6)
		0 0 0 0 0			

SCREENING SAFETY BLOOD SAMPLE

ANYYN <XYESNO> LAB <LAB5>

LABCODE <XLABCODE> (= CODE FOR LAB USED)

NRSOURCE <XNSOURE> = P

LABGRP <XLABGRP> = HEMRE

	Visit Day	Screening
	Sample Date	ASSYDAT Day Month Yr
Haematology	For SB Lab Code Number	
	Test (LPARAM)	Value [1]
	White Blood Cells WBC	VALUE / VALUET
	Haematocrit HCT	LABUNITS <XLABUNITS>
	Haemoglobin HGB	
	Platelets PLATE	
Clini. Chem.	For SB Lab Code Number (LPARAM)	
	Creatinine CREAT	
	Alanine transaminase (ALT) ALAT	
	Bilirubin BILTOT	
	Are there ***clinically significant abnormal*** laboratory values? PSTATQ <XPSTATAL> LAB1	N No Y Yes* ANSYN

ANYYN <XYESNO> LAB <LAB6>

LABCODE <XLABCODE>

NRSOURCE <XNSOURC> = P

LABGROUP <XLABGRP> = CHEM

ANYYN <XYESNO> PSTAT <PSTAT2CS>

[1]*Enter laboratory result or one of the following codes: ND* = **not done,** *LE* = **documented laboratory error**

* *Circle any significant laboratory value and record details on the Significant Medical / Surgical History and Physical Examination page.*

OBS: 1900.PCMED

VISIT: SCREENING.

00000

Page	4

Protocol 252263/030		Subject Number 0 0 0 0 0	Subject Initials		Screening (Day-21 to -6)

PRIOR AND CONCOMITANT MEDICATION

PCMED <PCMED2>

Has the patient taken any medication in the **30 days** prior to starting the study or during the study ?

[N] No ANYYN <XYESNO>

[Y] Yes ⟶ *If 'Yes', please record details below (Please print clearly)*

**Drug Name** *(Trade Name Preferred)*	**Daily Dose** (e.g. 500mg)	**Medical Illness/ Diagnosis** *(or symptom in absence of diagnosis)*	**Start Date** *(be as precise as possible)* Day Mth Yr	**End Date** *(or if Continuing mark box)* Day Mth Yr	CONTYN
DRUGNAME	SDOSE	INDICAT	STARTDAT	ENDDATE	<XYESN[illegible]
For SB					
(NOTIFY AND WHODRUG FOR AUTOENCODER) <XNOTIFY = (1)>					
For SB					
For SB					
For SB					
For SB					
For SB					
For SB					
For SB					
For SB					
For SB					
For SB					

OBS: 1000.SCR

VISIT: SCREENING

00000

Page	5

Protocol 252263/030		Subject Number 0 0 0 0 0	Subject Initials		Screening (Day-21 to -6)

SIGNIFICANT MEDICAL/SURGICAL HISTORY AND PHYSICAL EXAMINATION

Is the patient suffering from or has he/she ever suffered from any **clinically significant** medical or surgical condition? HISEX <HISEX2>

N No ANYYN <XYESNO>

Y Yes → *If 'Yes', please list below one diagnosis per line. (Please print clearly)*

Only in the absence of a diagnosis, record the signs and symptoms on separate lines.

Diagnosis	Year of first diagnosis	Past	Ongoing
DIAG<br>For SB	DIAGYR	NCP <XNCP> 1 (3)	2
[+ NOTIFY AND MEDTERM FOR AUTOENCODER] <XNOTIFY = L><br>For SB			
For SB			
For SB			
For SB			
For SB			
For SB			
For SB			
For SB			
For SB			

OBS: 1000.SCR

VISIT: SCREENING

00000

Page	6

Protocol		Subject Number	Subject Initials		
252263/030		0 0 0 0 0			Screening (Day-21 to -6)

ELIGIBILITY CHECKLIST

ELIG <ELIG2INC>

Please complete the following inclusion criteria.

INCLUSION CRITERIA INEXQ <XINEXQAN>

		Yes	No
		ANSYN <XYESNO>	
1.	Subject is a volunteer (male or female) providing Informed Consent (INC5)	Y	N
2.	Subject is aged between 18 and 55 years old (INC6)	☐	☐
3.	Subject will be residing in the study area for the entire duration of the study (approximately 70 weeks). (INC7)	☐	☐

Do not admit the Subject to this study if any "No" box has been marked.

Please complete the following exclusion criteria. ELIG <ELIG3EXC>

EXCLUSION CRITERIA INEXQ <XINEXQAN>

		Yes	No
		ANSYN	
1.	Subject is unwilling to report for drug administration or blood drawing during the 70 week duration of the study. (EXC17)	Y	N
2.	Subject has any medical condition which in the opinion of the investigator makes the subject unsuitable to enter the study (EXC18)	☐	☐
3.	Subject has clinically significant abnormalities (to include but not limited to abnormal hepatic or renal function) as determined by history, physical and routine blood chemistries and hematology values. (EXC19)	☐	☐
4.	Subject has a personal or family history of seizures (EXC20)	☐	☐
5.	Subject has a history of psychiatric disorders (EXC21)	☐	☐
6.	Subject is female and is pregnant or lactating or who in the opinion of the investigator are at risk of becoming pregnant (EXC22)	☐	☐
7.	Subject has a positive serum or urine B-HCG (to be tested during screening and within 48 hours of first drug administration and approximately monthly thereafter). (EXC23)	☐	☐

note: All female volunteers will have B-HCG testing. Those requiring repeat testing due to the lapse of >48 hrs will have their original negative test confirmed by an additional urine test within 48 hours of initiation of study medication (halofantrine treatment).

		Yes	No
8.	Subject has demonstrated hypersensitivity to any of the study drugs especially to any other 8 - aminoquinolines (EXC1)	☐	☐
9.	Subject is taking any other anti-malarial product , or has taken another antimalarial drug other than halofantrine within the previous 2 weeks (EXC24)	☐	☐
10.	Subject has abnormal EKG particularly an extended QTc interval >0.4 sec. (EXC25)	☐	☐
11.	Subject has received an investigational drug (a new chemical entity not registered for use) within 30 days or 5 half-lives whichever is the longer. (EXC3[illegible])	☐	☐

Do not admit the Subject to this study if any "Yes" box has been marked.

MODULE 1

CLEARANCE VISITS

(DAY -5 TO DAY -2)

MODULE 1

OBS: 1040. D2CL
VISIT: (DAY -2) CLEARANCE

00000

Page	7

Protocol 252263/030		Subject Number 0 0 0 0 0	Subject Initials		Clearance Day -5 to Day -2

DAY -5 TO DAY -3 : HALOFANTRINE CLEARANCE DOSES

CRF <CRF5>

Day	Dose received ?	Date dose received
VISIT= DAY-5 Clearance dose 1 (Day -5) OBS= 1010.D5CL	N No ANYYN / Y Yes → <XYESNO>	STARTDT Day Month Yr
VISIT= DAY-4 Clearance dose 2 (Day -4) OBS= 1020.D4CL	☐ No / ☐ Yes →	Day Month Yr
VISIT=DAY-3 Clearance dose 3 (Day -3) OBS=1030.D3CL	☐ No / ☐ Yes →	Day Month Yr

DAY -2 : PARASITAEMIA

PARASIT <PARASIT4>
[SEE PAGE 2]

Was a finger prick smear taken ?

☐ No

☐ Yes → Sample Date Day Month Yr

Parasitaemia present ?

☐ No

☐ Yes → Results :

☐ P. Falciparum
☐ P. Malariae
☐ P. Ovale

OBS: 1800. ~~AE~~

VISIT: AE ~~CLEARANCE~~

00000

Page	8

Protocol		Subject Number	Subject Initials		Clearance
252263/030		0 0 0 0 0			Day -5 to Day -2

BASELINE SIGNS AND SYMPTOMS (or events, or diagnoses) ACE <ACE2BSS>

*Record any baseline events (using **standard medical terminology**) observed or elicited by the following direct question to patient: "Have you felt different in any way since screening ?"*

*Provide the diagnosis, **not** symptoms where possible. One baseline event per column.*

If you consider this to be a serious baseline event (SBE), please do not enter on this page but enter in the Serious Adverse Experience section (SAE) (See opposite for definitions of an SBE)

If no baseline events since Screening, please sign form below and mark this box ☐ ANYYN <XYESNO>

**Baseline Event** *(please print clearly)*	AE [+ NOTIFY, AEMOD, WHOAE FOR AUTOENCODER]	
**For SmithKline Beecham**	(XNOTIFY = ⓛ)	
**Onset Date and Time** ONSETD ONSETT	Day Month Yr 24hr:min	Day Month Yr 24hr:min
**End Date and Time** *(If ongoing please leave blank)* STOPDT STOPT	Day Month Yr 24hr:min	Day Month Yr 24hr:min
**Outcome** OUTCOME <XOUTCOME> *If patient died, STOP: go to SAE section and follow instructions given there*	(1) Resolved<br>(2) Ongoing<br>(3) Died	☐ Resolved<br>☐ Ongoing<br>☐ Died
**Event Course** COURSE1 (XCOURSE1)	(1) Intermittent → No. of episodes ☐ EPISODES<br>(2) Constant	☐ Intermittent → No. of episodes ☐<br>☐ Constant
**Intensity** (maximum) INTENSITY <XINTENSIT>	(1) Mild<br>(2) Moderate<br>(3) Severe	☐ Mild<br>☐ Moderate<br>☐ Severe
**Relationship to study procedures performed prior to randomisation** RELATION <XRELATN3>	(1) Not related<br>(2) Unlikely<br>(3) Suspected (reasonable possibility)<br>(4) Probable	☐ Not related<br>☐ Unlikely<br>☐ Suspected (reasonable possibility)<br>☐ Probable
**Corrective Therapy** *If 'Yes', record details in Prior and Concomitant Medication section and/or Resource Utilisation section if appropriate*	(Y) Yes (N) No TREATYN <XYESNO>	☐ Yes ☐ No
**Was patient withdrawn due to this specific event prior to randomisation?**	(Y) Yes (N) No WITHAE <XYESNO>	☐ Yes ☐ No

Investigator's Signature : ______________________ **Date :** Day Month Yr

MODULE 1

RANDOMISATION

(DAY 0)

MODULE 1

OBS: 1050.DØRND

VISIT: DAY Ø

00000

Page	9

Protocol		Subject Number	Subject Initials		Randomisation
252263/030		0 0 0 0 0			(Day 0)

ELIGIBILITY CHECKLIST

ELIG <ELIG4EXC>

Please complete the following exclusion criteria.

EXCLUSION CRITERIA INEXQ <XINEXQ>

		No	Yes
		ANSYN	
1.	Subject has positive parasitemia following halofantrine treatment for radical cure. EXC27	(N)	(Y)
2.	Subject has Glucose 6 Phosphate Dehydrogenase deficiency (G6PD) *(NB.* ***Two normal*** *results are needed to be eligible for the study).* EXC15	☐	☐
3.	Subject's laboratory values are : Hb < 10 gm, PLT < 80,000/mm³, WBC < 3000ul³, Creatinine or ALT more than 2 times the upper limit of normal for age. EXC28	☐	☐
4.	Subject has a positive serum or urine B-HCG. EXC23	☐	☐

Do not admit the patient to this study if any "Yes" box has been marked.

INVESTIGATOR INSTRUCTIONS

ANYYN <XYESNO> PSTAT <PSTAT1PC>

Is the patient continuing in the study ? PSTATQ <XPSTATAL> = (PC1)

(N) No ⟶ *Withdraw the patient from the study. Complete the Study Conclusion section.*

ANSYN

(Y) Yes ⟶ *Randomise the patient to study medication and give the first dose to the subject (Loading Dose 1)*

RANDOMISATION

ANYYN <XYESNO> CRF <CRF3>

Assign a medication pack to the patient and record details below : DRUGNAME (TAFENOQUINE)

Please attach label here

Medication Number : ☐☐☐☐☐ BOTLAB

INVESTIGATOR INSTRUCTIONS

Please continue to record the randomisation visit results on page 10 under Visit "Load. dose 1".

RANDOMISATION TO WEEK 8

MODULE 2

Protocol	Subject Number	Subject Initials
252263/030	0 0 0 0 0	

Page	10

RANDOMISATION TO WEEK 3

Visit	CRF <CRF4> Visit / Dosing Date and Time ANYYN<XYESNO> DAT	PSTATQ Dose Taken[1] DT1	ANYYN<XYESNO> <XPSTATAL> Symptoms of Malaria[2] SM1	Pregnancy Test[3] PREG3	PSTAT <PSTAT1PS> Concom. Med.[4] CM1	Adverse Exp.[5] AE2	DAT PARASIT <PARASIT3> Parasitaemia[6] (Sample Date) ANYYN <XYESNO>	Species	DAT ANYYN <XYESNO> Vital Signs VITALS <VITMLS6>	LABLINK <LABLINK2> Safety Blood Samples[7] DAT	LABLINK <LABLINK3> Date and time of PK blood sample[8] DAT
Load Dose 1	STARTDAT Day Month Yr / STARTTIME 24hr:min<br>Day Month Yr / 24hr:min<br>OBS = 1050.BORND	No / Yes	No / Yes	No / Yes<br>VISIT = LOAD DOSE 1	No / Yes	No / Yes			TMP Temp VALUE.VUNIT (C) <XVUNIT><br>PUL Pulse VALUET (Bpm)<br>BP BP / (mmHg)<br>VPARAM <XVPARAM>	ANYYN <XYESNO><br>N No<br>Y Yes<br>LABTYPE = (6)	ANYYN <XYESNO>
Load Dose 2	Day Month Yr / 24hr:min<br>Day Month Yr / 24hr:min<br>OBS = 2010.LD2 VISIT = LOAD DOSE 2	ANYYN <XYESNO> PSTATQ <XPSTATAL><br>No / Yes<br>DT1	No / Yes<br>SM1		PSTAT <PSTAT2PS><br>No / Yes<br>CM1	No / Yes<br>AE2			VITALS <VITALS7> ANYYN <XYESNO><br>Temp VALUE./VALUET<br>VPARAM <XVPARAM> = (TMP)<br>VUNIT <XVUNIT> = (C)		
Load Dose 3	Day Month Yr / 24hr:min<br>Day Month Yr / 24hr:min<br>OBS = 2020.LD3 VISIT = LOAD DOSE 3	No / Yes	No / Yes		No / Yes	No / Yes	(SDAT) sample date		Temp .		6-12 hrs post-dose<br>SAMPDAT Day Month Yr<br>SAMPTIM 24hr:min<br>LABTYPE = (3)
Week 1	DAT (visit date) Day Month Yr / 24hr:min<br>(dosing date) Day Month Yr / 24hr:min<br>OBS = 2030.W1 VISIT = WEEK 1	No / Yes	No / Yes		No / Yes	No / Yes	PERFDAT<br>PARAQ <XPARASQ><br>Para. present? (3) (4)<br>N No<br>Y Yes →	P.Fal<br>P.Mal<br>P.Ov	Temp .		
Week 2	Day Month Yr / 24hr:min<br>Day Month Yr / 24hr:min<br>OBS = 2040.W2 VISIT = WEEK 2	No / Yes	No / Yes		No / Yes	No / Yes	Para. present?<br>No<br>Yes →	P.Fal<br>P.Mal<br>P.Ov	Temp .		
Week 3	Day Month Yr / 24hr:min<br>Day Month Yr / 24hr:min<br>OBS = 2050.W3 VISIT = WEEK 3	No / Yes	No / Yes		No / Yes	No / Yes	Para. present?<br>No<br>Yes →	P.Fal<br>P.Mal<br>P.Ov	Temp .		

Protocol	Subject Number	Subject Initials
252263/030	0 0 0 0 0	

WEEK 4 TO WEEK 8

Visit	Visit / Dosing Date and Time	Dose Taken[1]	Symptoms of Malaria[2]	Preg-nancy Test[3]	Concom. Med.[4]	Adverse Exp.[5]	Parasitaemia[6] (Sample Date) / Species	Vital Signs	Safety Blood Samples[7] (LABLINK)	Date and time of PK blood sample[8]
Week 4	Day Month Yr 24hr:min<br>Day Month Yr 24hr:min<br>OBS: 2060.W4 VISIT:WEEK4	☐ No ☐ Yes	☐ No ☐ Yes	☐ No ☐ Yes	☐ No ☐ Yes	☐ No ☐ Yes	Para. present? ☐ No ☐ Yes → ☐ P.Fal ☐ P.Mal ☐ P.Ov	Temp __ __ . __<br>Pulse __ __ __<br>BP __ __ __ / __ __ __	<LABLINK(1)> ANYYN <X;YES;NO> N No Y Yes LABTYPE=6	Pre-dose<br>Day Month Yr<br>24hr:min
Week 5	Day Month Yr 24hr:min<br>Day Month Yr 24hr:min<br>OBS: 2070.W5 VISIT:WEEK5	☐ No ☐ Yes	☐ No ☐ Yes		☐ No ☐ Yes	☐ No ☐ Yes	Para. present? ☐ No ☐ Yes → ☐ P.Fal ☐ P.Mal ☐ P.Ov	Temp __ __ . __		
Week 6	Day Month Yr 24hr:min<br>Day Month Yr 24hr:min<br>OBS: 2080.W6 VISIT:WEEK6	☐ No ☐ Yes	☐ No ☐ Yes		☐ No ☐ Yes	☐ No ☐ Yes	Para. present? ☐ No ☐ Yes → ☐ P.Fal ☐ P.Mal ☐ P.Ov	Temp __ __ . __		
Week 7	Day Month Yr 24hr:min<br>Day Month Yr 24hr:min<br>OBS: 2090.W7 VISIT:WEEK7	☐ No ☐ Yes	☐ No ☐ Yes		☐ No ☐ Yes	☐ No ☐ Yes	Para. present? ☐ No ☐ Yes → ☐ P.Fal ☐ P.Mal ☐ P.Ov	Temp __ __ . __		
Week 8	Day Month Yr 24hr:min<br>Day Month Yr 24hr:min<br>OBS: 2100.W8 VISIT:WEEK8	☐ No ☐ Yes	☐ No ☐ Yes	☐ No ☐ Yes	☐ No ☐ Yes	☐ No ☐ Yes	Para. present? ☐ No ☐ Yes → ☐ P.Fal ☐ P.Mal ☐ P.Ov	Temp __ __ . __<br>Pulse __ __ __<br>BP __ __ __ / __ __ __	<LABLINK(2)> ☐ No ☐ Yes	2-5 hrs post-dose<br>Day Month Yr<br>24hr:min

Protocol	Subject Number	Subject Initials
252263/030	0 0 0 0 0	

UNSCHEDULED VISITS

PSTAT <PSTAT3PS> PARASIT <PARASIT3> VITALS <VITALS6> [SEE PAGE 10]

Visit Date CRF <CRF6>	Symptoms of Malaria[2] PSTATCO	Pregnancy Test[3] <XPSTATAL>	Concom. Med.[4]	Adverse Exp.[5]	Parasitaemia[6] (Sample Date)	Species	Vital Signs	Safety Blood Samples[7]	Date and time of PK blood sample[8]
DAT Day Month Yr<br>OBS: 2210.UN1	SM1 ANYYN <XYESNO> N No / Y Yes<br>VISIT: UNSCHED. VISIT1	PREG3 N No / Y Yes	CM1 N No / Y Yes	AE2 N No / Y Yes	Para. present? No / Yes →	P.Falciparum / P.Malariae / P.Ovale	Temp . / Pulse / BP /	LABLINK [LABLINK2A] No / Yes	LABLINK <LABLINK3> PK sample taken if parasitaemia is positive. Day Month Yr 24hr:min
Day Month Yr<br>OBS: 2220.UN2	No / Yes<br>VISIT: UNSCHED. VISIT2	No / Yes	No / Yes	No / Yes	Para. present? No / Yes →	P.Falciparum / P.Malariae / P.Ovale	Temp . / Pulse / BP /	No / Yes	PK sample taken if parasitaemia is positive. Day Month Yr 24hr:min
Day Month Yr<br>OBS: 2230.UN3	No / Yes<br>VISIT: UNSCHED. VISIT3	No / Yes	No / Yes	No / Yes	Para. present? No / Yes →	P.Falciparum / P.Malariae / P.Ovale	Temp . / Pulse / BP /	No / Yes	PK sample taken if parasitaemia is positive. Day Month Yr 24hr:min
Day Month Yr<br>OBS: 2240.UN4	No / Yes<br>VISIT: UNSCHED. VISIT4	No / Yes	No / Yes	No / Yes	Para. present? No / Yes →	P.Falciparum / P.Malariae / P.Ovale	Temp . / Pulse / BP /	No / Yes	PK sample taken if parasitaemia is positive. Day Month Yr 24hr:min
Day Month Yr<br>OBS: 2250.UN5	No / Yes<br>VISIT: UNSCHED. VISIT5	No / Yes	No / Yes	No / Yes	Para. present? No / Yes →	P.Falciparum / P.Malariae / P.Ovale	Temp . / Pulse / BP /	No / Yes	PK sample taken if parasitaemia is positive. Day Month Yr 24hr:min
Day Month Yr<br>OBS: 2260.UN6	No / Yes<br>VISIT: UNSCHED. VISIT6	No / Yes	No / Yes	No / Yes	Para. present? No / Yes →	P.Falciparum / P.Malariae / P.Ovale	Temp . / Pulse / BP /	No / Yes	PK sample taken if parasitaemia is positive. Day Month Yr 24hr:min

Protocol	Subject Number	Subject Initials
252263/030	0 0 0 0 0	

SAFETY BLOOD SAMPLE RESULTS

[SEE PAGE 3]

	Visit Day	Randomisation	Week 4	Week 8	Unscheduled	Unscheduled	Unscheduled	Unscheduled
	Sample Date	Day Month Yr	Day Month Yr	Day Month Yr	Day Month Yr	Day Month Yr	Day Month Yr	Day Month Yr
Haematology	For SB Lab Code Number	OBS: 1080.DØRND *VISIT: DAY Ø	OBS: 2060.W4 VISIT: WEEK4	OBS: 2100.W8 VISIT: WEEK8	OBS: 2210.UNS1 VISIT: UNSCHED. VISIT1	OBS: 2220.UNS2 VISIT: UNSCHED. VISIT2	OBS: 2230.UNS3 VISIT: UNSCHED. VISIT3	OBS: 2240.UNS4 VISIT: UNSCHED VISIT4
	Test	Value **	Value **	Value **	Value **	Value **	Value **	Value **
	White Blood Cells							
	Haematocrit							
	Haemoglobin							
	Platelets							
Clin. Chem.	For SB Lab Code Number							
	Creatinine							
	Alanine transaminase (ALT)							
	Bilirubin							
	Are there ***clinically significant abnormal*** laboratory values?	☐ No ☐ Yes*	☐ No ☐ Yes*	☐ No ☐ Yes*	☐ No ☐ Yes*	☐ No ☐ Yes*	☐ No ☐ Yes*	☐ No ☐ Yes*

** *Enter laboratory result or one of the following codes:*
ND **= not done,**
LE = documented laboratory error

* *Circle any significant laboratory value and record details on the Adverse Experience page*

* VISIT : LOAD ONE 1

ADVERSE EXPERIENCES (Non-serious)

*Record any adverse experiences (using **standard medical terminology**) observed or elicited by the following direct question to subject*

"Do you feel different in any way since starting the treatment or since the last visit?"

*Provide the diagnosis, **not** symptoms, where possible. One adverse experience per column.*

If you consider this to be a serious adverse experience (SAE), please do not enter on this page but enter in the SAE section (See opposite for definitions of an SAE).

Protocol	Subject Number	Subject Initials
252263/030	0 0 0 0 0	

OBS : 2800.AE

VISIT: ADVERSE EXPERIENCES (AE)

Page 14

ADVERSE EXPERIENCES (Non-serious)

ACE <ACE1NS> SERYN <XYESNO = (N)>

If no adverse experiences occured since Clearance, please mark this box. ☐ ANYYN <XYESNO>

**Adverse Experience** *(please print clearly)*	AE (AEMOD, WHOAE FOR AUTOENCODER)	NOTIFY <XNOTIFY> = (L)		
**For SmithKline Beecham**				
**Onset Date and Time** ONSETD ONSETT	Day Month Yr 24hr:min	Day Month Yr 24hr:min	Day Month Yr 24hr:min	Day Month Yr 24hr:min
**End Date and Time** *(If ongoing please leave blank)* STOPDT STOPT	Day Month Yr 24hr:min	Day Month Yr 24hr:min	Day Month Yr 24hr:min	Day Month Yr 24hr:min
**Outcome** OUTCOME <XOUTCOME> ***If subject died, STOP: go to SAE section Exacerbation pages***	(1) Resolved<br>(2) Ongoing<br>(3) Died	☐ Resolved<br>☐ Ongoing<br>☐ Died	☐ Resolved<br>☐ Ongoing<br>☐ Died	☐ Resolved<br>☐ Ongoing<br>☐ Died
**Experience Course** COURSE <XCOURSE1>	(1) Intermittent → No. of episodes ☐ EPISODES<br>(2) Constant	☐ Intermittent → No. of episodes ☐<br>☐ Constant	☐ Intermittent → No. of episodes ☐<br>☐ Constant	☐ Intermittent → No. of episodes ☐<br>☐ Constant
**Intensity** (maximum) INTENSIT <XINTENSIT> *See definitions page for Adverse Experience*	(1) Mild<br>(2) Moderate<br>(3) Severe	☐ Mild<br>☐ Moderate<br>☐ Severe	☐ Mild<br>☐ Moderate<br>☐ Severe	☐ Mild<br>☐ Moderate<br>☐ Severe
**Action Taken with Respect to Investigational Drug** ACTION <XACTION3>	(1) None<br>(2) Dose reduced<br>(3) Dose increased<br>(4) Drug interrupted/restarted<br>(5) Drug stopped	☐ None<br>☐ Dose reduced<br>☐ Dose increased<br>☐ Drug interrupted/restarted<br>☐ Drug stopped	☐ None<br>☐ Dose reduced<br>☐ Dose increased<br>☐ Drug interrupted/restarted<br>☐ Drug stopped	☐ None<br>☐ Dose reduced<br>☐ Dose increased<br>☐ Drug interrupted/restarted<br>☐ Drug stopped
**Relationship to Investigational Drug** RELATN3 <XRELATN3>	(1) Not related<br>(2) Unlikely<br>(3) Suspected (reasonable possibility)<br>(4) Probable	☐ Not related<br>☐ Unlikely<br>☐ Suspected (reasonable possibility)<br>☐ Probable	☐ Not related<br>☐ Unlikely<br>☐ Suspected (reasonable possibility)<br>☐ Probable	☐ Not related<br>☐ Unlikely<br>☐ Suspected (reasonable possibility)<br>☐ Probable
**Corrective Therapy** *If 'Yes', record details in the Concomitant Medication section and/or Resource Utilisation section if appropriate*	(Y) Yes (N) No TREATYN <XYESNO>	☐ Yes ☐ No	☐ Yes ☐ No	☐ Yes ☐ No
**Was subject withdrawn due to this specific AE?**	(Y) Yes (N) No WITHAE <XYESNO>	☐ Yes ☐ No	☐ Yes ☐ No	☐ Yes ☐ No

Protocol	Subject Number	Subject Initials
252263/030	0 0 0 0 0	

ADVERSE EXPERIENCES (Non-serious)

[SEE PAGE 14]

**Adverse Experience** *(please print clearly)*				
**For SmithKline Beecham**				
**Onset Date and Time**	Day Month Yr 24hr:min	Day Month Yr 24hr:min	Day Month Yr 24hr:min	Day Month Yr 24hr:min
**End Date and Time** *(If ongoing please leave blank)*	Day Month Yr 24hr:min	Day Month Yr 24hr:min	Day Month Yr 24hr:min	Day Month Yr 24hr:min
**Outcome** ***If subject died, STOP: go to SAE section Exacerbation pages***	☐ Resolved<br>☐ Ongoing<br>☐ Died	☐ Resolved<br>☐ Ongoing<br>☐ Died	☐ Resolved<br>☐ Ongoing<br>☐ Died	☐ Resolved<br>☐ Ongoing<br>☐ Died
**Experience Course**	☐ Intermittent → No. of episodes ☐<br>☐ Constant	☐ Intermittent → No. of episodes ☐<br>☐ Constant	☐ Intermittent → No. of episodes ☐<br>☐ Constant	☐ Intermittent → No. of episodes ☐<br>☐ Constant
**Intensity** (maximum) *See definitions page for Adverse Experience*	☐ Mild<br>☐ Moderate<br>☐ Severe	☐ Mild<br>☐ Moderate<br>☐ Severe	☐ Mild<br>☐ Moderate<br>☐ Severe	☐ Mild<br>☐ Moderate<br>☐ Severe
**Action Taken with Respect to Investigational Drug**	☐ None<br>☐ Dose reduced<br>☐ Dose increased<br>☐ Drug interrupted/restarted<br>☐ Drug stopped	☐ None<br>☐ Dose reduced<br>☐ Dose increased<br>☐ Drug interrupted/restarted<br>☐ Drug stopped	☐ None<br>☐ Dose reduced<br>☐ Dose increased<br>☐ Drug interrupted/restarted<br>☐ Drug stopped	☐ None<br>☐ Dose reduced<br>☐ Dose increased<br>☐ Drug interrupted/restarted<br>☐ Drug stopped
**Relationship to Investigational Drug**	☐ Not related<br>☐ Unlikely<br>☐ Suspected (reasonable<br>☐ Probable possibility)	☐ Not related<br>☐ Unlikely<br>☐ Suspected (reasonable<br>☐ Probable possibility)	☐ Not related<br>☐ Unlikely<br>☐ Suspected (reasonable<br>☐ Probable possibility)	☐ Not related<br>☐ Unlikely<br>☐ Suspected (reasonable<br>☐ Probable possibility)
**Corrective Therapy** *If 'Yes', record details in the Concomitant Medication section and/or Resource Utilisation section if appropriate*	☐ Yes ☐ No	☐ Yes ☐ No	☐ Yes ☐ No	☐ Yes ☐ No
**Was subject withdrawn due to this specific AE?**	☐ Yes ☐ No	☐ Yes ☐ No	☐ Yes ☐ No	☐ Yes ☐ No

Protocol	Subject Number	Subject Initials
252263/030	0 0 0 0 0	

ADVERSE EXPERIENCES (Non-serious)

[SEE PAGE 14]

**Adverse Experience** *(please print clearly)*				
**For SmithKline Beecham**				
**Onset Date and Time**	Day Month Yr 24hr:min	Day Month Yr 24hr:min	Day Month Yr 24hr:min	Day Month Yr 24hr:min
**End Date and Time** *(If ongoing please leave blank)*	Day Month Yr 24hr:min	Day Month Yr 24hr:min	Day Month Yr 24hr:min	Day Month Yr 24hr:min
**Outcome** ***If subject died, STOP: go to SAE section Exacerbation pages***	☐ Resolved<br>☐ Ongoing<br>☐ Died	☐ Resolved<br>☐ Ongoing<br>☐ Died	☐ Resolved<br>☐ Ongoing<br>☐ Died	☐ Resolved<br>☐ Ongoing<br>☐ Died
**Experience Course**	☐ Intermittent → No. of episodes ☐<br>☐ Constant	☐ Intermittent → No. of episodes ☐<br>☐ Constant	☐ Intermittent → No. of episodes ☐<br>☐ Constant	☐ Intermittent → No. of episodes ☐<br>☐ Constant
**Intensity** (maximum) *See definitions page for Adverse Experience*	☐ Mild<br>☐ Moderate<br>☐ Severe	☐ Mild<br>☐ Moderate<br>☐ Severe	☐ Mild<br>☐ Moderate<br>☐ Severe	☐ Mild<br>☐ Moderate<br>☐ Severe
**Action Taken with Respect to Investigational Drug**	☐ None<br>☐ Dose reduced<br>☐ Dose increased<br>☐ Drug interrupted/restarted<br>☐ Drug stopped	☐ None<br>☐ Dose reduced<br>☐ Dose increased<br>☐ Drug interrupted/restarted<br>☐ Drug stopped	☐ None<br>☐ Dose reduced<br>☐ Dose increased<br>☐ Drug interrupted/restarted<br>☐ Drug stopped	☐ None<br>☐ Dose reduced<br>☐ Dose increased<br>☐ Drug interrupted/restarted<br>☐ Drug stopped
**Relationship to Investigational Drug**	☐ Not related<br>☐ Unlikely<br>☐ Suspected (reasonable possibility)<br>☐ Probable	☐ Not related<br>☐ Unlikely<br>☐ Suspected (reasonable possibility)<br>☐ Probable	☐ Not related<br>☐ Unlikely<br>☐ Suspected (reasonable possibility)<br>☐ Probable	☐ Not related<br>☐ Unlikely<br>☐ Suspected (reasonable possibility)<br>☐ Probable
**Corrective Therapy** *If 'Yes', record details in the Concomitant Medication section and/or Resource Utilisation section if appropriate*	☐ Yes ☐ No	☐ Yes ☐ No	☐ Yes ☐ No	☐ Yes ☐ No
**Was subject withdrawn due to this specific AE?**	☐ Yes ☐ No	☐ Yes ☐ No	☐ Yes ☐ No	☐ Yes ☐ No

Protocol	Subject Number	Subject Initials
252263/030	0 0 0 0 0	

OBS: 2900. PCMED
VISIT : CONMED

CONCOMITANT MEDICATION

PCMED <PCMED3>

Record any changes in concomitant medication taken since Clearance

☐ No concomitant medication changes since Clearance ANYN ('N' IF TICKED, 'Y' IF NOT) <XYESNO>

Drug Name (Trade Name Preferred)	Daily Dose (e.g. 500mg)	Medical Illness/ Diagnosis (or symptom in absence of diagnosis)	Start Date (be as precise as possible) Day Mth Yr	End Date Day Mth Yr	(or if Continuing mark box) CONTYN
DRUGNAME	SDOSE	INDICAT	STARTDAT	ENDDATE	☐ <XCONTYN>
For SB					
[NOTIFY, WHODRUG FOR AUTOENCODER] <XNOTIFY = L>					☐
For SB					
					☐
For SB					
					☐
For SB					
					☐
For SB					
					☐
For SB					
					☐
For SB					
					☐
For SB					
					☐
For SB					

Protocol	Subject Number	Subject Initials
252263/030	0 0 0 0 0	

CONCOMITANT MEDICATION - Continued

[SEE PAGE 17]

Drug Name (Trade Name Preferred)	Daily Dose (e.g. 500mg)	Medical Illness/ Diagnosis (or symptom in absence of diagnosis)	Start Date (be as precise as possible) Day Mth Yr	End Date Day Mth Yr	(or if Continuing mark box)
					☐
For SB					
					☐
For SB					
					☐
For SB					
					☐
For SB					
					☐
For SB					
					☐
For SB					
					☐
For SB					
					☐
For SB					
					☐
For SB					
					☐
For SB					

WEEK 9 TO WEEK 16

MODULE 3

Protocol	Subject Number	Subject Initials
252263/030	0 0 0 0 0	

WEEK 9 TO WEEK 14

[SEE PAGE 10]

Visit	Visit / Dosing Date and Time	Dose Taken[1]	Symptoms of Malaria[2]	Pregnancy Test[3]	Concom. Med.[4]	Adverse Exp.[5]	Parasitaemia[6] (Sample Date)	Species	Vital Signs	Safety Blood Samples[7]	Date and time of PK blood sample[8]
Week 9	Day Month Yr 24hr:min<br>Day Month Yr 24hr:min<br>OBS: 3000.W9 VISIT: WEEK9	No<br>Yes	No<br>Yes		No<br>Yes	No<br>Yes	Para. present?<br>No<br>Yes →	P.Fal<br>P.Mal<br>P.Ov	Temp .		
Week 10	Day Month Yr 24hr:min<br>Day Month Yr 24hr:min<br>OBS: 3010.W10 VISIT: WEEK10	No<br>Yes	No<br>Yes		No<br>Yes	No<br>Yes	Para. present?<br>No<br>Yes →	P.Fal<br>P.Mal<br>P.Ov	Temp .		
Week 11	Day Month Yr 24hr:min<br>Day Month Yr 24hr:min<br>OBS: 3020.W11 VISIT: WEEK11	No<br>Yes	No<br>Yes		No<br>Yes	No<br>Yes	Para. present?<br>No<br>Yes →	P.Fal<br>P.Mal<br>P.Ov	Temp .		
Week 12	Day Month Yr 24hr:min<br>Day Month Yr 24hr:min<br>OBS: 3030.W12 VISIT: WEEK12	No<br>Yes	No<br>Yes	No<br>Yes	No<br>Yes	No<br>Yes	Para. present?<br>No<br>Yes →	P.Fal<br>P.Mal<br>P.Ov	Temp .		
Week 13	Day Month Yr 24hr:min<br>Day Month Yr 24hr:min<br>OBS: 3040.W13 VISIT: WEEK13	No<br>Yes	No<br>Yes		No<br>Yes	No<br>Yes	Para. present?<br>No<br>Yes →	P.Fal<br>P.Mal<br>P.Ov	Temp .		
Week 14	Day Month Yr 24hr:min<br>Day Month Yr 24hr:min<br>OBS: 3050.W14 VISIT: WEEK14	No<br>Yes	No<br>Yes		No<br>Yes	No<br>Yes	Para. present?<br>No<br>Yes →	P.Fal<br>P.Mal<br>P.Ov	Temp .		

Protocol	Subject Number	Subject Initials
252263/030	0 0 0 0 0	

WEEK 15 TO WEEK 16

[SEE PAGE 10]

Visit	Visit / Dosing Date and Time	Dose Taken[1]	Symptoms of Malaria[2]	Preg-nancy Test[3]	Concom. Med.[4]	Adverse Exp.[5]	Parasitaemia[6] (Sample Date) / Species	Vital Signs	Safety Blood Samples[7]	Date and time of PK blood sample[8]
Week 15	Day Month Yr 24hr:min<br>Day Month Yr 24hr:min<br>OBS: 3060.W15 VISIT: WEEK15	No<br>Yes	No<br>Yes		No<br>Yes	No<br>Yes	Para. present? No / Yes →<br>P.Fal / P.Mal / P.Ov	Temp .		
Week 16	Day Month Yr 24hr:min<br>Day Month Yr 24hr:min<br>OBS: 3070.W16 VISIT: WEEK16	No<br>Yes	No<br>Yes	No<br>Yes	No<br>Yes	No<br>Yes	Para. present? No / Yes →<br>P.Fal / P.Mal / P.Ov	Temp .<br>Pulse<br>BP /	No<br>Yes	2-5 days post-dose<br>Day Month Yr<br>24hr:min

Protocol	Subject Number	Subject Initials
252263/030	0 0 0 0 0	

UNSCHEDULED VISITS

[SEE PAGE 12]

Visit Date	Symptoms of Malaria[2]	Pregnancy Test[3]	Concom. Med.[4]	Adverse Exp.[5]	Parasitaemia[6] (Sample Date) / Species	Vital Signs	Safety Blood Samples[7]	Date and time of PK blood sample[8]
Day Month Yr	No / Yes	No / Yes	No / Yes	No / Yes	Para. present? No / Yes → ; P.Falciparum / P.Malariae / P.Ovale	Temp . ; Pulse ; BP /	No / Yes	PK sample taken if parasitaemia is positive. Day Month Yr 24hr:min
OBS: 3210.UNS1	VISIT: UNSCHED. VISIT1							
Day Month Yr	No / Yes	No / Yes	No / Yes	No / Yes	Para. present? No / Yes → ; P.Falciparum / P.Malariae / P.Ovale	Temp . ; Pulse ; BP /	No / Yes	PK sample taken if parasitaemia is positive. Day Month Yr 24hr:min
OBS: 3220.UNS2	VISIT: UNSCHED. VISIT2							
Day Month Yr	No / Yes	No / Yes	No / Yes	No / Yes	Para. present? No / Yes → ; P.Falciparum / P.Malariae / P.Ovale	Temp . ; Pulse ; BP /	No / Yes	PK sample taken if parasitaemia is positive. Day Month Yr 24hr:min
OBS: 3230.UNS3	VISIT: UNSCHED. VISIT3							
Day Month Yr	No / Yes	No / Yes	No / Yes	No / Yes	Para. present? No / Yes → ; P.Falciparum / P.Malariae / P.Ovale	Temp . ; Pulse ; BP /	No / Yes	PK sample taken if parasitaemia is positive. Day Month Yr 24hr:min
OBS: 3240.UNS4	VISIT: UNSCHED. VISIT4							
Day Month Yr	No / Yes	No / Yes	No / Yes	No / Yes	Para. present? No / Yes → ; P.Falciparum / P.Malariae / P.Ovale	Temp . ; Pulse ; BP /	No / Yes	PK sample taken if parasitaemia is positive. Day Month Yr 24hr:min
OBS: 3250.UNS5	VISIT: UNSCHED. VISIT5							
Day Month Yr	No / Yes	No / Yes	No / Yes	No / Yes	Para. present? No / Yes → ; P.Falciparum / P.Malariae / P.Ovale	Temp . ; Pulse ; BP /	No / Yes	PK sample taken if parasitaemia is positive. Day Month Yr 24hr:min
OBS: 3260.UNS6	VISIT: UNSCHED. VISIT6							

Protocol	Subject Number	Subject Initials
252263/030	0 0 0 0 0	

SAFETY BLOOD SAMPLE RESULTS

[SEE PAGE 3]

Visit Day		Week 16 OBS: 3070.W16	Unscheduled OBS: 3210.UNS1	Unscheduled OBS: 3220.UNS2	Unscheduled OBS: 3230.UNS3	Unscheduled OBS: 3240.UNS4	Unscheduled OBS: 3250.UNS5	Unscheduled OBS: 3260.UNS6
Sample Date		Day Month Yr	Day Month Yr	Day Month Yr	Day Month Yr	Day Month Yr	Day Month Yr	Day Month Yr
Haematology	For SB Lab Code Number	VISIT: WEEK 16	VISIT: UNSCHED.	VISIT: UNSCHED.	VISIT: UNSCHED. VISIT 3	VISIT: UNSCHED VISIT 4	VISIT: UNSCHED. VISIT 5	VISIT: UNSCHED. VISIT 6
	Test	Value **	Value ** VISIT 1	Value ** VISIT 2	Value **	Value **	Value **	Value **
	White Blood Cells							
	Haematocrit							
	Haemoglobin							
	Platelets							
Clin. Chem.	For SB Lab Code Number							
	Creatinine							
	Alanine transaminase (ALT)							
	Bilirubin							
Are there ***clinically significant abnormal*** laboratory values?		☐ No ☐ Yes*	☐ No ☐ Yes*	☐ No ☐ Yes*	☐ No ☐ Yes*	☐ No ☐ Yes*	☐ No ☐ Yes*	☐ No ☐ Yes*

** *Enter laboratory result or one of the following codes:*
ND **= not done,**
LE = documented laboratory error

* *Circle any significant laboratory value and record details on the Adverse Experience page*

ADVERSE EXPERIENCES (Non-serious)

*Record any adverse experiences (using **standard medical terminology**) observed or elicited by the following direct question to subject:*

"Do you feel different in any way since starting the treatment or since the last visit?"

*Provide the diagnosis, **not** symptoms, where possible. One adverse experience per column.*

If you consider this to be a serious adverse experience (SAE), please do not enter on this page but enter in the SAE section (See opposite for definitions of an SAE).

Protocol	Subject Number	Subject Initials
252263/030	0 0 0 0 0	

OBS: 3800.AE
VISIT: AE

[SEE PAGE 14]

ADVERSE EXPERIENCES (Non-serious)

If no adverse experiences occured since Week 8, please mark this box. ☐

**Adverse Experience** *(please print clearly)*				
**For SmithKline Beecham**				
**Onset Date and Time**	Day Month Yr 24hr:min	Day Month Yr 24hr:min	Day Month Yr 24hr:min	Day Month Yr 24hr:min
**End Date and Time** *(If ongoing please leave blank)*	Day Month Yr 24hr:min	Day Month Yr 24hr:min	Day Month Yr 24hr:min	Day Month Yr 24hr:min
**Outcome** ***If subject died, STOP: go to SAE section Exacerbation pages***	☐ Resolved ☐ Ongoing ☐ Died	☐ Resolved ☐ Ongoing ☐ Died	☐ Resolved ☐ Ongoing ☐ Died	☐ Resolved ☐ Ongoing ☐ Died
**Experience Course**	☐ Intermittent → No. of episodes ☐ Constant	☐ Intermittent → No. of episodes ☐ Constant	☐ Intermittent → No. of episodes ☐ Constant	☐ Intermittent → No. of episodes ☐ Constant
**Intensity** (maximum) *See definitions page for Adverse Experience*	☐ Mild ☐ Moderate ☐ Severe	☐ Mild ☐ Moderate ☐ Severe	☐ Mild ☐ Moderate ☐ Severe	☐ Mild ☐ Moderate ☐ Severe
**Action Taken with Respect to Investigational Drug**	☐ None ☐ Dose reduced ☐ Dose increased ☐ Drug interrupted/restarted ☐ Drug stopped	☐ None ☐ Dose reduced ☐ Dose increased ☐ Drug interrupted/restarted ☐ Drug stopped	☐ None ☐ Dose reduced ☐ Dose increased ☐ Drug interrupted/restarted ☐ Drug stopped	☐ None ☐ Dose reduced ☐ Dose increased ☐ Drug interrupted/restarted ☐ Drug stopped
**Relationship to Investigational Drug**	☐ Not related ☐ Unlikely ☐ Suspected ☐ Probable (reasonable possibility)	☐ Not related ☐ Unlikely ☐ Suspected ☐ Probable (reasonable possibility)	☐ Not related ☐ Unlikely ☐ Suspected ☐ Probable (reasonable possibility)	☐ Not related ☐ Unlikely ☐ Suspected ☐ Probable (reasonable possibility)
**Corrective Therapy** *If 'Yes', record details in the Concomitant Medication section and/or Resource Utilisation section if appropriate*	☐ Yes ☐ No	☐ Yes ☐ No	☐ Yes ☐ No	☐ Yes ☐ No
**Was subject withdrawn due to this specific AE?**	☐ Yes ☐ No	☐ Yes ☐ No	☐ Yes ☐ No	☐ Yes ☐ No

Protocol	Subject Number	Subject Initials
252263/030	0 0 0 0 0	

ADVERSE EXPERIENCES (Non-serious)

[SEE PAGE 14]

**Adverse Experience** *(please print clearly)*				
**For SmithKline Beecham**				
**Onset Date and Time**	Day Month Yr 24hr:min	Day Month Yr 24hr:min	Day Month Yr 24hr:min	Day Month Yr 24hr:min
**End Date and Time** *(If ongoing please leave blank)*	Day Month Yr 24hr:min	Day Month Yr 24hr:min	Day Month Yr 24hr:min	Day Month Yr 24hr:min
**Outcome** ***If subject died, STOP: go to SAE section Exacerbation pages***	☐ Resolved<br>☐ Ongoing<br>☐ Died	☐ Resolved<br>☐ Ongoing<br>☐ Died	☐ Resolved<br>☐ Ongoing<br>☐ Died	☐ Resolved<br>☐ Ongoing<br>☐ Died
**Experience Course**	☐ Intermittent → No. of episodes ☐<br>☐ Constant	☐ Intermittent → No. of episodes ☐<br>☐ Constant	☐ Intermittent → No. of episodes ☐<br>☐ Constant	☐ Intermittent → No. of episodes ☐<br>☐ Constant
**Intensity** (maximum) *See definitions page for Adverse Experience*	☐ Mild<br>☐ Moderate<br>☐ Severe	☐ Mild<br>☐ Moderate<br>☐ Severe	☐ Mild<br>☐ Moderate<br>☐ Severe	☐ Mild<br>☐ Moderate<br>☐ Severe
**Action Taken with Respect to Investigational Drug**	☐ None<br>☐ Dose reduced<br>☐ Dose increased<br>☐ Drug interrupted/restarted<br>☐ Drug stopped	☐ None<br>☐ Dose reduced<br>☐ Dose increased<br>☐ Drug interrupted/restarted<br>☐ Drug stopped	☐ None<br>☐ Dose reduced<br>☐ Dose increased<br>☐ Drug interrupted/restarted<br>☐ Drug stopped	☐ None<br>☐ Dose reduced<br>☐ Dose increased<br>☐ Drug interrupted/restarted<br>☐ Drug stopped
**Relationship to Investigational Drug**	☐ Not related<br>☐ Unlikely<br>☐ Suspected (reasonable possibility)<br>☐ Probable	☐ Not related<br>☐ Unlikely<br>☐ Suspected (reasonable possibility)<br>☐ Probable	☐ Not related<br>☐ Unlikely<br>☐ Suspected (reasonable possibility)<br>☐ Probable	☐ Not related<br>☐ Unlikely<br>☐ Suspected (reasonable possibility)<br>☐ Probable
**Corrective Therapy** *If 'Yes', record details in the Concomitant Medication section and/or Resource Utilisation section if appropriate*	☐ Yes ☐ No	☐ Yes ☐ No	☐ Yes ☐ No	☐ Yes ☐ No
**Was subject withdrawn due to this specific AE?**	☐ Yes ☐ No	☐ Yes ☐ No	☐ Yes ☐ No	☐ Yes ☐ No

Protocol	Subject Number	Subject Initials
252263/030	0 0 0 0 0	

ADVERSE EXPERIENCES (Non-serious)

[SEE PAGE 14]

Adverse Experience *(please print clearly)*				
**For SmithKline Beecham**				
**Onset Date and Time**	Day Month Yr 24hr:min	Day Month Yr 24hr:min	Day Month Yr 24hr:min	Day Month Yr 24hr:min
**End Date and Time** *(If ongoing please leave blank)*	Day Month Yr 24hr:min	Day Month Yr 24hr:min	Day Month Yr 24hr:min	Day Month Yr 24hr:min
**Outcome** ***If subject died, STOP: go to SAE section Exacerbation pages***	☐ Resolved<br>☐ Ongoing<br>☐ Died	☐ Resolved<br>☐ Ongoing<br>☐ Died	☐ Resolved<br>☐ Ongoing<br>☐ Died	☐ Resolved<br>☐ Ongoing<br>☐ Died
**Experience Course**	☐ Intermittent → No. of episodes ☐<br>☐ Constant	☐ Intermittent → No. of episodes ☐<br>☐ Constant	☐ Intermittent → No. of episodes ☐<br>☐ Constant	☐ Intermittent → No. of episodes ☐<br>☐ Constant
**Intensity** (maximum) *See definitions page for Adverse Experience*	☐ Mild<br>☐ Moderate<br>☐ Severe	☐ Mild<br>☐ Moderate<br>☐ Severe	☐ Mild<br>☐ Moderate<br>☐ Severe	☐ Mild<br>☐ Moderate<br>☐ Severe
**Action Taken with Respect to Investigational Drug**	☐ None<br>☐ Dose reduced<br>☐ Dose increased<br>☐ Drug interrupted/restarted<br>☐ Drug stopped	☐ None<br>☐ Dose reduced<br>☐ Dose increased<br>☐ Drug interrupted/restarted<br>☐ Drug stopped	☐ None<br>☐ Dose reduced<br>☐ Dose increased<br>☐ Drug interrupted/restarted<br>☐ Drug stopped	☐ None<br>☐ Dose reduced<br>☐ Dose increased<br>☐ Drug interrupted/restarted<br>☐ Drug stopped
**Relationship to Investigational Drug**	☐ Not related<br>☐ Unlikely<br>☐ Suspected (reasonable<br>☐ Probable possibility)	☐ Not related<br>☐ Unlikely<br>☐ Suspected (reasonable<br>☐ Probable possibility)	☐ Not related<br>☐ Unlikely<br>☐ Suspected (reasonable<br>☐ Probable possibility)	☐ Not related<br>☐ Unlikely<br>☐ Suspected (reasonable<br>☐ Probable possibility)
**Corrective Therapy** *If 'Yes', record details in the Concomitant Medication section and/or Resource Utilisation section if appropriate*	☐ Yes ☐ No	☐ Yes ☐ No	☐ Yes ☐ No	☐ Yes ☐ No
**Was subject withdrawn due to this specific AE?**	☐ Yes ☐ No	☐ Yes ☐ No	☐ Yes ☐ No	☐ Yes ☐ No

Protocol	Subject Number	Subject Initials
252263/030	0 0 0 0 0	

OBS: 3900.CONMED
VISIT: CONMED

[SEE PAGE 17]

CONCOMITANT MEDICATION

Record any changes in concomitant medication taken since Week 8

☐ No concomitant medication changes since Week 8

Drug Name *(Trade Name Preferred)*	Daily Dose (e.g. 500mg)	Medical Illness/ Diagnosis *(or symptom in absence of diagnosis)*	Start Date *(be as precise as possible)* Day Mth Yr	End Date Day Mth Yr	*(or if Continuing mark box)*
					☐
For SB					
					☐
For SB					
					☐
For SB					
					☐
For SB					
					☐
For SB					
					☐
For SB					
					☐
For SB					
					☐
For SB					
					☐
For SB					

Protocol	Subject Number	Subject Initials
252263/030	0 0 0 0 0	

CONCOMITANT MEDICATION - Continued

(SEE PAGE 7)

Drug Name (Trade Name Preferred)	Daily Dose (e.g. 500mg)	Medical Illness/ Diagnosis (or symptom in absence of diagnosis)	Start Date (be as precise as possible) Day Mth Yr	End Date Day Mth Yr	(or if Continuing mark box)
					☐
For SB					
					☐
For SB					
					☐
For SB					
					☐
For SB					
					☐
For SB					
					☐
For SB					
					☐
For SB					
					☐
For SB					
					☐
For SB					
					☐
For SB					

WEEK 17 TO WEEK 24

MODULE 4

Protocol	Subject Number	Subject Initials
252263/030	0 0 0 0 0	

WEEK 17 TO WEEK 22

[SEE PAGE 10]

Visit	Visit / Dosing Date and Time	Dose Taken[1]	Symptoms of Malaria[2]	Pregnancy Test[3]	Concom. Med.[4]	Adverse Exp.[5]	Parasitaemia[6] (Sample Date)	Species	Vital Signs	Safety Blood Samples[7]	Date and time of PK blood sample[8]
Week 17	Day Month Yr 24hr:min<br>Day Month Yr 24hr:min<br>OBS: 4000.W17 VISIT: WEEK 17	No<br>Yes	No<br>Yes		No<br>Yes	No<br>Yes	Para. present?<br>No<br>Yes →	P.Fal<br>P.Mal<br>P.Ov	Temp .		Pre-dose<br>Day Month Yr<br>24hr:min
Week 18	Day Month Yr 24hr:min<br>Day Month Yr 24hr:min<br>OBS: 4010.W18 VISIT: WEEK 18	No<br>Yes	No<br>Yes		No<br>Yes	No<br>Yes	Para. present?<br>No<br>Yes →	P.Fal<br>P.Mal<br>P.Ov	Temp .		
Week 19	Day Month Yr 24hr:min<br>Day Month Yr 24hr:min<br>OBS: 4020.W19 VISIT: WEEK 19	No<br>Yes	No<br>Yes		No<br>Yes	No<br>Yes	Para. present?<br>No<br>Yes →	P.Fal<br>P.Mal<br>P.Ov	Temp .		
Week 20	Day Month Yr 24hr:min<br>Day Month Yr 24hr:min<br>OBS: 4030.W20 VISIT: WEEK 20	No<br>Yes	No<br>Yes	No<br>Yes	No<br>Yes	No<br>Yes	Para. present?<br>No<br>Yes →	P.Fal<br>P.Mal<br>P.Ov	Temp .		
Week 21	Day Month Yr 24hr:min<br>Day Month Yr 24hr:min<br>OBS: 4040.W21 VISIT: WEEK 21	No<br>Yes	No<br>Yes		No<br>Yes	No<br>Yes	Para. present?<br>No<br>Yes →	P.Fal<br>P.Mal<br>P.Ov	Temp .		
Week 22	Day Month Yr 24hr:min<br>Day Month Yr 24hr:min<br>OBS: 4050.W22 VISIT: WEEK 22	No<br>Yes	No<br>Yes		No<br>Yes	No<br>Yes	Para. present?<br>No<br>Yes →	P.Fal<br>P.Mal<br>P.Ov	Temp .		

Protocol	Subject Number	Subject Initials
252263/030	0 0 0 0 0	

WEEK 23 TO WEEK 24

[SEE PAGE 10]

Visit	Visit / Dosing Date and Time	Dose Taken[1]	Symptoms of Malaria[2]	Preg-nancy Test[3]	Concom. Med.[4]	Adverse Exp.[5]	Parasitaemia[6] (Sample Date)	Species	Vital Signs	Safety Blood Samples[7]	Date and time of PK blood sample[8]
Week 23	Day Month Yr 24hr:min<br>Day Month Yr 24hr:min<br>OBS : 4060 . W23 VISIT: WEEK 23	No<br>Yes	No<br>Yes		No<br>Yes	No<br>Yes	Para. present?<br>No<br>Yes →	P.Fal<br>P.Mal<br>P.Ov	Temp .		
Week 24	Day Month Yr 24hr:min<br>Day Month Yr 24hr:min<br>OBS : 4070 . W24 VISIT: WEEK 24	No<br>Yes	No<br>Yes	No<br>Yes	No<br>Yes	No<br>Yes	Para. present?<br>No<br>Yes →	P.Fal<br>P.Mal<br>P.Ov	Temp .<br>Pulse<br>BP /	No<br>Yes	

Protocol	Subject Number	Subject Initials
252263/030	0 0 0 0 0	

UNSCHEDULED VISITS

[SEE PAGE 12]

Visit Date	Symptoms of Malaria[2]	Pregnancy Test[3]	Concom. Med.[4]	Adverse Exp.[5]	Parasitaemia[6] (Sample Date)	Species	Vital Signs	Safety Blood Samples[7]	Date and time of PK blood sample[8]
Day Month Yr	No / Yes	No / Yes	No / Yes	No / Yes	Para. present? No / Yes →	P.Falciparum / P.Malariae / P.Ovale	Temp . / Pulse / BP /	No / Yes	PK sample taken if parasitaemia is positive. Day Month Yr 24hr:min
OBS: 4210.UNS1 VISIT: UNSCHED. VISIT1									
Day Month Yr	No / Yes	No / Yes	No / Yes	No / Yes	Para. present? No / Yes →	P.Falciparum / P.Malariae / P.Ovale	Temp . / Pulse / BP /	No / Yes	PK sample taken if parasitaemia is positive. Day Month Yr 24hr:min
OBS: 4220.UNS2 VISIT: UNSCHED. VISIT2									
Day Month Yr	No / Yes	No / Yes	No / Yes	No / Yes	Para. present? No / Yes →	P.Falciparum / P.Malariae / P.Ovale	Temp . / Pulse / BP /	No / Yes	PK sample taken if parasitaemia is positive. Day Month Yr 24hr:min
OBS: 4230.UNS3 VISIT: UNSCHED. VISIT3									
Day Month Yr	No / Yes	No / Yes	No / Yes	No / Yes	Para. present? No / Yes →	P.Falciparum / P.Malariae / P.Ovale	Temp . / Pulse / BP /	No / Yes	PK sample taken if parasitaemia is positive. Day Month Yr 24hr:min
OBS: 4240.UNS4 VISIT: UNSCHED. VISIT4									
Day Month Yr	No / Yes	No / Yes	No / Yes	No / Yes	Para. present? No / Yes →	P.Falciparum / P.Malariae / P.Ovale	Temp . / Pulse / BP /	No / Yes	PK sample taken if parasitaemia is positive. Day Month Yr 24hr:min
OBS: 4250.UNS5 VISIT: UNSCHED. VISIT5									
Day Month Yr	No / Yes	No / Yes	No / Yes	No / Yes	Para. present? No / Yes →	P.Falciparum / P.Malariae / P.Ovale	Temp . / Pulse / BP /	No / Yes	PK sample taken if parasitaemia is positive. Day Month Yr 24hr:min
OBS: 4250.UNS6 VISIT: UNSCHED. VISIT6									

Protocol	Subject Number	Subject Initials
252263/030	0 0 0 0 0	

SAFETY BLOOD SAMPLE RESULTS

[SEE PAGE 3]

	Visit Day	Week 24	Unscheduled	Unscheduled	Unscheduled	Unscheduled	Unscheduled	Unscheduled
	Sample Date	Day Month Yr	Day Month Yr	Day Month Yr	Day Month Yr	Day Month Yr	Day Month Yr	Day Month Yr
Haematology	For SB Lab Code Number	OBS: 4070. W 24	OBS: 4210. UNS1	OBS: 4220. UNS 2	OBS: 4230. UNS3	OBS: 4240. UNS4	OBS: 4250. UNS5	OBS: 4260. UNS6
	Test	Value **	Value **	Value **	Value **	Value **	Value **	Value **
	White Blood Cells	VISIT: WEEK 24	VISIT: UNSCHED. VISIT 1	VISIT: UNSCHED. VISIT 2	VISIT: UNSCHED. VISIT 3	VISIT: UNSCHED. VISIT 4	VISIT: UNSCHED. VISIT 5	VISIT: UNSCHED. VISIT 6
	Haematocrit							
	Haemoglobin							
	Platelets							
Clin. Chem.	For SB Lab Code Number							
	Creatinine							
	Alanine transaminase (ALT)							
	Bilirubin							
	Are there ***clinically significant abnormal*** laboratory values?	☐ No ☐ Yes*	☐ No ☐ Yes*	☐ No ☐ Yes*	☐ No ☐ Yes*	☐ No ☐ Yes*	☐ No ☐ Yes*	☐ No ☐ Yes*

** *Enter laboratory result or one of the following codes:*
***ND* = not done,**
LE = documented laboratory error

* *Circle any significant laboratory value and record details on the Adverse Experience page*

ADVERSE EXPERIENCES (Non-serious)

*Record any adverse experiences (using **standard medical terminology**) observed or elicited by the following direct question to subject:*

"Do you feel different in any way since starting the treatment or since the last visit?"

*Provide the diagnosis, **not** symptoms, where possible. One adverse experience per column.*

If you consider this to be a serious adverse experience (SAE), please do not enter on this page but enter in the SAE section (See opposite for definitions of an SAE).

Protocol	Subject Number	Subject Initials
252263/030	0 0 0 0 0	

OBS: 4800. AE
VISIT: AE

ADVERSE EXPERIENCES (Non-serious)

[SEE PAGE 14]

If no adverse experiences occured since Week 16, please mark this box. ☐

**Adverse Experience** *(please print clearly)*				
**For SmithKline Beecham**				
**Onset Date and Time**	Day Month Yr 24hr:min	Day Month Yr 24hr:min	Day Month Yr 24hr:min	Day Month Yr 24hr:min
**End Date and Time** *(If ongoing please leave blank)*	Day Month Yr 24hr:min	Day Month Yr 24hr:min	Day Month Yr 24hr:min	Day Month Yr 24hr:min
**Outcome** ***If subject died, STOP: go to SAE section Exacerbation pages***	☐ Resolved ☐ Ongoing ☐ Died	☐ Resolved ☐ Ongoing ☐ Died	☐ Resolved ☐ Ongoing ☐ Died	☐ Resolved ☐ Ongoing ☐ Died
**Experience Course**	☐ Intermittent → No. of episodes ☐ ☐ Constant	☐ Intermittent → No. of episodes ☐ ☐ Constant	☐ Intermittent → No. of episodes ☐ ☐ Constant	☐ Intermittent → No. of episodes ☐ ☐ Constant
**Intensity** (maximum) *See definitions page for Adverse Experience*	☐ Mild ☐ Moderate ☐ Severe	☐ Mild ☐ Moderate ☐ Severe	☐ Mild ☐ Moderate ☐ Severe	☐ Mild ☐ Moderate ☐ Severe
**Action Taken with Respect to Investigational Drug**	☐ None ☐ Dose reduced ☐ Dose increased ☐ Drug interrupted/restarted ☐ Drug stopped	☐ None ☐ Dose reduced ☐ Dose increased ☐ Drug interrupted/restarted ☐ Drug stopped	☐ None ☐ Dose reduced ☐ Dose increased ☐ Drug interrupted/restarted ☐ Drug stopped	☐ None ☐ Dose reduced ☐ Dose increased ☐ Drug interrupted/restarted ☐ Drug stopped
**Relationship to Investigational Drug**	☐ Not related ☐ Unlikely ☐ Suspected ☐ Probable (reasonable possibility)	☐ Not related ☐ Unlikely ☐ Suspected ☐ Probable (reasonable possibility)	☐ Not related ☐ Unlikely ☐ Suspected ☐ Probable (reasonable possibility)	☐ Not related ☐ Unlikely ☐ Suspected ☐ Probable (reasonable possibility)
**Corrective Therapy** *If 'Yes', record details in the Concomitant Medication section and/or Resource Utilisation section if appropriate*	☐ Yes ☐ No	☐ Yes ☐ No	☐ Yes ☐ No	☐ Yes ☐ No
**Was subject withdrawn due to this specific AE?**	☐ Yes ☐ No	☐ Yes ☐ No	☐ Yes ☐ No	☐ Yes ☐ No

Protocol	Subject Number	Subject Initials
252263/030	0 0 0 0 0	

ADVERSE EXPERIENCES (Non-serious)

[SEE PAGE 14]

**Adverse Experience** *(please print clearly)*				
**For SmithKline Beecham**				
**Onset Date and Time**	Day Month Yr 24hr:min	Day Month Yr 24hr:min	Day Month Yr 24hr:min	Day Month Yr 24hr:min
**End Date and Time** *(If ongoing please leave blank)*	Day Month Yr 24hr:min	Day Month Yr 24hr:min	Day Month Yr 24hr:min	Day Month Yr 24hr:min
**Outcome** *If subject died, STOP: go to SAE section Exacerbation pages*	Resolved<br>Ongoing<br>Died	Resolved<br>Ongoing<br>Died	Resolved<br>Ongoing<br>Died	Resolved<br>Ongoing<br>Died
**Experience Course**	Intermittent → No. of episodes<br>Constant	Intermittent → No. of episodes<br>Constant	Intermittent → No. of episodes<br>Constant	Intermittent → No. of episodes<br>Constant
**Intensity** (maximum) *See definitions page for Adverse Experience*	Mild<br>Moderate<br>Severe	Mild<br>Moderate<br>Severe	Mild<br>Moderate<br>Severe	Mild<br>Moderate<br>Severe
**Action Taken with Respect to Investigational Drug**	None<br>Dose reduced<br>Dose increased<br>Drug interrupted/restarted<br>Drug stopped	None<br>Dose reduced<br>Dose increased<br>Drug interrupted/restarted<br>Drug stopped	None<br>Dose reduced<br>Dose increased<br>Drug interrupted/restarted<br>Drug stopped	None<br>Dose reduced<br>Dose increased<br>Drug interrupted/restarted<br>Drug stopped
**Relationship to Investigational Drug**	Not related<br>Unlikely<br>Suspected (reasonable possibility)<br>Probable	Not related<br>Unlikely<br>Suspected (reasonable possibility)<br>Probable	Not related<br>Unlikely<br>Suspected (reasonable possibility)<br>Probable	Not related<br>Unlikely<br>Suspected (reasonable possibility)<br>Probable
**Corrective Therapy** *If 'Yes', record details in the Concomitant Medication section and/or Resource Utilisation section if appropriate*	Yes No	Yes No	Yes No	Yes No
**Was subject withdrawn due to this specific AE?**	Yes No	Yes No	Yes No	Yes No

Protocol	Subject Number	Subject Initials
252263/030	0 0 0 0 0	

ADVERSE EXPERIENCES (Non-serious)

[SEE PAGE 14]

**Adverse Experience** *(please print clearly)*				
**For SmithKline Beecham**				
**Onset Date and Time**	Day Month Yr 24hr:min	Day Month Yr 24hr:min	Day Month Yr 24hr:min	Day Month Yr 24hr:min
**End Date and Time** *(If ongoing please leave blank)*	Day Month Yr 24hr:min	Day Month Yr 24hr:min	Day Month Yr 24hr:min	Day Month Yr 24hr:min
**Outcome** ***If subject died, STOP: go to SAE section Exacerbation pages***	☐ Resolved<br>☐ Ongoing<br>☐ Died	☐ Resolved<br>☐ Ongoing<br>☐ Died	☐ Resolved<br>☐ Ongoing<br>☐ Died	☐ Resolved<br>☐ Ongoing<br>☐ Died
**Experience Course**	☐ Intermittent → No. of episodes ☐<br>☐ Constant	☐ Intermittent → No. of episodes ☐<br>☐ Constant	☐ Intermittent → No. of episodes ☐<br>☐ Constant	☐ Intermittent → No. of episodes ☐<br>☐ Constant
**Intensity** (maximum) *See definitions page for Adverse Experience*	☐ Mild<br>☐ Moderate<br>☐ Severe	☐ Mild<br>☐ Moderate<br>☐ Severe	☐ Mild<br>☐ Moderate<br>☐ Severe	☐ Mild<br>☐ Moderate<br>☐ Severe
**Action Taken with Respect to Investigational Drug**	☐ None<br>☐ Dose reduced<br>☐ Dose increased<br>☐ Drug interrupted/restarted<br>☐ Drug stopped	☐ None<br>☐ Dose reduced<br>☐ Dose increased<br>☐ Drug interrupted/restarted<br>☐ Drug stopped	☐ None<br>☐ Dose reduced<br>☐ Dose increased<br>☐ Drug interrupted/restarted<br>☐ Drug stopped	☐ None<br>☐ Dose reduced<br>☐ Dose increased<br>☐ Drug interrupted/restarted<br>☐ Drug stopped
**Relationship to Investigational Drug**	☐ Not related<br>☐ Unlikely<br>☐ Suspected (reasonable<br>☐ Probable possibility)	☐ Not related<br>☐ Unlikely<br>☐ Suspected (reasonable<br>☐ Probable possibility)	☐ Not related<br>☐ Unlikely<br>☐ Suspected (reasonable<br>☐ Probable possibility)	☐ Not related<br>☐ Unlikely<br>☐ Suspected (reasonable<br>☐ Probable possibility)
**Corrective Therapy** *If 'Yes', record details in the Concomitant Medication section and/or Resource Utilisation section if appropriate*	☐ Yes ☐ No	☐ Yes ☐ No	☐ Yes ☐ No	☐ Yes ☐ No
**Was subject withdrawn due to this specific AE?**	☐ Yes ☐ No	☐ Yes ☐ No	☐ Yes ☐ No	☐ Yes ☐ No

Protocol	Subject Number	Subject Initials
252263/030	0 0 0 0 0	

OBS: 4900. CONMED
VISIT: CONMED

CONCOMITANT MEDICATION

[SEE PAGE 17]

Record any changes in concomitant medication taken since Week 16

☐ No concomitant medication changes since Week 16

Drug Name *(Trade Name Preferred)*	Daily Dose (e.g. 500mg)	Medical Illness/ Diagnosis *(or symptom in absence of diagnosis)*	Start Date *(be as precise as possible)* Day Mth Yr	End Date Day Mth Yr	*(or if Continuing mark box)*
					☐
For SB					
					☐
For SB					
					☐
For SB					
					☐
For SB					
					☐
For SB					
					☐
For SB					
					☐
For SB					
					☐
For SB					
					☐
For SB					

Protocol	Subject Number	Subject Initials
252263/030	0 0 0 0 0	

CONCOMITANT MEDICATION - Continued

[SEE PAGE 17]

Drug Name (Trade Name Preferred)	Daily Dose (e.g. 500mg)	Medical Illness/ Diagnosis (or symptom in absence of diagnosis)	Start Date (be as precise as possible) Day Mth Yr	End Date Day Mth Yr	(or if Continuing mark box)
					☐
For SB					
					☐
For SB					
					☐
For SB					
					☐
For SB					
					☐
For SB					
					☐
For SB					
					☐
For SB					
					☐
For SB					
					☐
For SB					
					☐
For SB					

WITHDRAWAL / SAFETY FOLLOW-UP VISITS

OR WEEK 25 - 28

SFUP

Protocol	Subject Number	Subject Initials
252263/030	0 0 0 0 0	

WITHDRAWAL / SAFETY FOLLOW-UP VISITS OR WEEK 25 - 28

[SEE PAGE 12]

Visit	Visit Date	Symptoms of Malaria[2]	Preg-nancy Test[3]	Concom. Med.[4]	Adverse Exp.[5]	Parasitaemia[6] (Sample Date)	Species	Vital Signs	Safety Blood Samples[7]	Date and time of PK blood sample[8]
**Safety Follow-up Visit 1**<br>OBS: 5000.SF1	Day Month Yr<br>VISIT: SFU_VISIT1	No<br>Yes		No<br>Yes	No<br>Yes	Para. present?<br>No<br>Yes →	P.Falciparum<br>P.Malariae<br>P.Ovale	Temp .		
**Safety Follow-up Visit 2**<br>OBS: 5010-SF2	Day Month Yr<br>VISIT: SFU_VISIT2	No<br>Yes		No<br>Yes	No<br>Yes	Para. present?<br>No<br>Yes →	P.Falciparum<br>P.Malariae<br>P.Ovale	Temp .		
**Safety Follow-up Visit 3**<br>OBS: 5020.SF3	Day Month Yr<br>VISIT: SFU_VISIT3	No<br>Yes		No<br>Yes	No<br>Yes	Para. present?<br>No<br>Yes →	P.Falciparum<br>P.Malariae<br>P.Ovale	Temp .		
**Safety Follow-up Visit 4**<br>OBS: 5030.SF4	Day Month Yr<br>VISIT: SFU_VISIT4	No<br>Yes		No<br>Yes	No<br>Yes	Para. present?<br>No<br>Yes →	P.Falciparum<br>P.Malariae<br>P.Ovale	Temp .<br>Pulse<br>BP /	No<br>Yes	Day Month Yr<br>24hr:min

Protocol	Subject Number	Subject Initials
252263/030	0 0 0 0 0	

UNSCHEDULED VISITS

<PSTAT30S>   [SEE PAGE 12]

Visit Date CRF <CRF4A>	Symptoms of Malaria[2]	Pregnancy Test[3]	Concom. Med.[4]	Adverse Exp.[5]	Parasitaemia[6] (Sample Date) / Species	Vital Signs	Safety Blood Samples[7]	Date and time of PK blood sample[8]
DATE Day Month Yr<br>STARTTIME 24hr:min<br>OBS: 5210.UNS1 VISIT: UNSCHED. VISIT1	No / Yes	No / Yes	No / Yes	No / Yes	Para. present? No / Yes →<br>P.Falciparum / P.Malariae / P.Ovale	Temp . / Pulse / BP /	No / Yes	PK sample taken if parasitaemia is positive.<br>Day Month Yr 24hr:min
Day Month Yr<br>24hr:min<br>OBS: 5220.UNS2 VISIT: UNSCHED. VISIT2	No / Yes	No / Yes	No / Yes	No / Yes	Para. present? No / Yes →<br>P.Falciparum / P.Malariae / P.Ovale	Temp . / Pulse / BP /	No / Yes	PK sample taken if parasitaemia is positive.<br>Day Month Yr 24hr:min
Day Month Yr<br>24hr:min<br>OBS: 5230.UNS3 VISIT: UNSCHED. VISIT3	No / Yes	No / Yes	No / Yes	No / Yes	Para. present? No / Yes →<br>P.Falciparum / P.Malariae / P.Ovale	Temp . / Pulse / BP /	No / Yes	PK sample taken if parasitaemia is positive.<br>Day Month Yr 24hr:min
Day Month Yr<br>24hr:min<br>OBS: 5240.UNS4 VISIT: UNSCHED. VISIT4	No / Yes	No / Yes	No / Yes	No / Yes	Para. present? No / Yes →<br>P.Falciparum / P.Malariae / P.Ovale	Temp . / Pulse / BP /	No / Yes	PK sample taken if parasitaemia is positive.<br>Day Month Yr 24hr:min
Day Month Yr<br>24hr:min<br>OBS: 5250.UNS5 VISIT: UNSCHED. VISIT5	No / Yes	No / Yes	No / Yes	No / Yes	Para. present? No / Yes →<br>P.Falciparum / P.Malariae / P.Ovale	Temp . / Pulse / BP /	No / Yes	PK sample taken if parasitaemia is positive.<br>Day Month Yr 24hr:min
Day Month Yr<br>24hr:min<br>OBS: 5260.UNS6 VISIT: UNSCHED. VISIT6	No / Yes	No / Yes	No / Yes	No / Yes	Para. present? No / Yes →<br>P.Falciparum / P.Malariae / P.Ovale	Temp . / Pulse / BP /	No / Yes	PK sample taken if parasitaemia is positive.<br>Day Month Yr 24hr:min

Protocol	Subject Number	Subject Initials
252263/030	0 0 0 0 0	

SAFETY BLOOD SAMPLE RESULTS

[SEE PAGE 3]

	Visit Day	Safety Follow-up Visit 4 OBS: 5030.SF4	Unscheduled OBS: 5210.UNS1	Unscheduled OBS: 5220.UNS2	Unscheduled OBS: 5230.UNS3	Unscheduled OBS: 5240.UNS4	Unscheduled OBS: 5250.UNS5	Unscheduled OBS: 5260.UNS6
	Sample Date	Day Month Yr	Day Month Yr	Day Month Yr	Day Month Yr	Day Month Yr	Day Month Yr	Day Month Yr
Haematology	For SB Lab Code Number							
	Test	Value **	Value **	Value **	Value **	Value **	Value **	Value **
	White Blood Cells	VISIT: SFU-VISIT4	VISIT: UNSCHED.	VISIT: UNSCHED.	VISIT: UNSCHED.	VISIT: UNSCHED.	VISIT: UNSCHED	VISIT: UNSCHED.
	Haematocrit		VISIT1	VISIT2	VISIT3	VISIT3	VISIT4	VISIT5
	Haemoglobin							
	Platelets							
Clin. Chem.	For SB Lab Code Number							
	Creatinine							
	Alanine transaminase (ALT)							
	Bilirubin							
	Are there ***clinically significant abnormal*** laboratory values?	☐ No ☐ Yes*	☐ No ☐ Yes*	☐ No ☐ Yes*	☐ No ☐ Yes*	☐ No ☐ Yes*	☐ No ☐ Yes*	☐ No ☐ Yes*

** *Enter laboratory result or one of the following codes:*
ND **= not done,**
LE = documented laboratory error

* *Circle any significant laboratory value and record details on the Adverse Experience page*

ADVERSE EXPERIENCES (Non-serious)

Record any adverse experiences (using ***standard medical terminology****) observed or elicited by the following direct question to subject:*

"Do you feel different in any way since starting the treatment or since the last visit?"

Provide the diagnosis, ***not*** *symptoms, where possible. One adverse experience per column.*

If you consider this to be a serious adverse experience (SAE), please do not enter on this page but enter in the SAE section (See opposite for definitions of an SAE).

Protocol	Subject Number	Subject Initials
252263/030	0 0 0 0 0	

OBS: 5800.AE
VISIT: AE

ADVERSE EXPERIENCES (Non-serious)

[SEE PAGE 14]

If no adverse experiences occured since Week 24, please mark this box. ☐

**Adverse Experience** *(please print clearly)*				
**For SmithKline Beecham**				
**Onset Date and Time**	Day Month Yr 24hr:min	Day Month Yr 24hr:min	Day Month Yr 24hr:min	Day Month Yr 24hr:min
**End Date and Time** *(If ongoing please leave blank)*	Day Month Yr 24hr:min	Day Month Yr 24hr:min	Day Month Yr 24hr:min	Day Month Yr 24hr:min
**Outcome** ***If subject died, STOP: go to SAE section Exacerbation pages***	☐ Resolved ☐ Ongoing ☐ Died	☐ Resolved ☐ Ongoing ☐ Died	☐ Resolved ☐ Ongoing ☐ Died	☐ Resolved ☐ Ongoing ☐ Died
**Experience Course**	☐ Intermittent ☐ Constant → No. of episodes ☐	☐ Intermittent ☐ Constant → No. of episodes ☐	☐ Intermittent ☐ Constant → No. of episodes ☐	☐ Intermittent ☐ Constant → No. of episodes ☐
**Intensity** (maximum) *See definitions page for Adverse Experience*	☐ Mild ☐ Moderate ☐ Severe	☐ Mild ☐ Moderate ☐ Severe	☐ Mild ☐ Moderate ☐ Severe	☐ Mild ☐ Moderate ☐ Severe
**Action Taken with Respect to Investigational Drug**	☐ None ☐ Dose reduced ☐ Dose increased ☐ Drug interrupted/restarted ☐ Drug stopped	☐ None ☐ Dose reduced ☐ Dose increased ☐ Drug interrupted/restarted ☐ Drug stopped	☐ None ☐ Dose reduced ☐ Dose increased ☐ Drug interrupted/restarted ☐ Drug stopped	☐ None ☐ Dose reduced ☐ Dose increased ☐ Drug interrupted/restarted ☐ Drug stopped
**Relationship to Investigational Drug**	☐ Not related ☐ Unlikely ☐ Suspected ☐ Probable (reasonable possibility)	☐ Not related ☐ Unlikely ☐ Suspected ☐ Probable (reasonable possibility)	☐ Not related ☐ Unlikely ☐ Suspected ☐ Probable (reasonable possibility)	☐ Not related ☐ Unlikely ☐ Suspected ☐ Probable (reasonable possibility)
**Corrective Therapy** *If 'Yes', record details in the Concomitant Medication section and/or Resource Utilisation section if appropriate*	☐ Yes ☐ No	☐ Yes ☐ No	☐ Yes ☐ No	☐ Yes ☐ No
**Was subject withdrawn due to this specific AE?**	☐ Yes ☐ No	☐ Yes ☐ No	☐ Yes ☐ No	☐ Yes ☐ No

Protocol	Subject Number	Subject Initials
252263/030	0 0 0 0 0	

ADVERSE EXPERIENCES (Non-serious)

[SEE PAGE 14]

Adverse Experience *(please print clearly)*				
For SmithKline Beecham				
Onset Date and Time	Day Month Yr 24hr:min	Day Month Yr 24hr:min	Day Month Yr 24hr:min	Day Month Yr 24hr:min
End Date and Time *(If ongoing please leave blank)*	Day Month Yr 24hr:min	Day Month Yr 24hr:min	Day Month Yr 24hr:min	Day Month Yr 24hr:min
Outcome *If subject died, STOP: go to SAE section Exacerbation pages*	Resolved<br>Ongoing<br>Died	Resolved<br>Ongoing<br>Died	Resolved<br>Ongoing<br>Died	Resolved<br>Ongoing<br>Died
Experience Course	Intermittent → No. of episodes<br>Constant	Intermittent → No. of episodes<br>Constant	Intermittent → No. of episodes<br>Constant	Intermittent → No. of episodes<br>Constant
Intensity (maximum) *See definitions page for Adverse Experience*	Mild<br>Moderate<br>Severe	Mild<br>Moderate<br>Severe	Mild<br>Moderate<br>Severe	Mild<br>Moderate<br>Severe
Action Taken with Respect to Investigational Drug	None<br>Dose reduced<br>Dose increased<br>Drug interrupted/restarted<br>Drug stopped	None<br>Dose reduced<br>Dose increased<br>Drug interrupted/restarted<br>Drug stopped	None<br>Dose reduced<br>Dose increased<br>Drug interrupted/restarted<br>Drug stopped	None<br>Dose reduced<br>Dose increased<br>Drug interrupted/restarted<br>Drug stopped
Relationship to Investigational Drug	Not related<br>Unlikely<br>Suspected (reasonable<br>Probable possibility)	Not related<br>Unlikely<br>Suspected (reasonable<br>Probable possibility)	Not related<br>Unlikely<br>Suspected (reasonable<br>Probable possibility)	Not related<br>Unlikely<br>Suspected (reasonable<br>Probable possibility)
Corrective Therapy *If 'Yes', record details in the Concomitant Medication section and/or Resource Utilisation section if appropriate*	Yes No	Yes No	Yes No	Yes No
Was subject withdrawn due to this specific AE?	Yes No	Yes No	Yes No	Yes No

Protocol	Subject Number	Subject Initials
252263/030	0 0 0 0 0	

OBS: 5900. CONMED
VISIT: CONMED.

CONCOMITANT MEDICATION

[SEE PAGE 17]

Record any changes in concomitant medication taken since Week 24

☐ No concomitant medication changes since Week 24

Drug Name *(Trade Name Preferred)*	Daily Dose (e.g. 500mg)	Medical Illness/ Diagnosis *(or symptom in absence of diagnosis)*	Start Date *(be as precise as possible)* Day Mth Yr	End Date Day Mth Yr	*(or if Continuing mark box)*
					☐
For SB					
					☐
For SB					
					☐
For SB					
					☐
For SB					
					☐
For SB					
					☐
For SB					
					☐
For SB					
					☐
For SB					
					☐
For SB					

Protocol	Subject Number	Subject Initials
252263/030	0 0 0 0 0	

CONCOMITANT MEDICATION - Continued

[SEE PAGE 17]

Drug Name (Trade Name Preferred)	Daily Dose (e.g. 500mg)	Medical Illness/ Diagnosis (or symptom in absence of diagnosis)	Start Date (be as precise as possible) Day Mth Yr	End Date Day Mth Yr	(or if Continuing mark box)
					☐
For SB					
					☐
For SB					
					☐
For SB					
					☐
For SB					
					☐
For SB					
					☐
For SB					
					☐
For SB					
					☐
For SB					
					☐
For SB					
					☐
For SB					

STUDY CONCLUSION

CONC1

OBS: 8000.END
VISIT: STUDY CONCLUSION

00000

Page	44

Protocol 252263/030		Subject Number 0 0 0 0 0	Subject Initials		Study Conclusion

STUDY CONCLUSION

PATEND <PATEND2>

Did the subject complete the study as planned ?

(Y) Yes ENDYN <XYESNO>

(N) No → *If 'No', mark the **primary** cause of withdrawal. (Mark one box only)*

DISCODE <XDISCODE> xdiscos 1

(1) Adverse Experience (complete AE page)

(2) Insufficient therapeutic effect (Development of Parasitaemia)

(4) Protocol deviation(including non-compliance)

(5) Lost to follow-up

(8) Did not meet Eligibility criteria

(7) Other - specify: ENDTEXT

I certify that I have reviewed the data on this case report form and that all information is complete and accurate.

Investigator's Signature NOT ENTERED

Day Month Yr

GENERAL INSTRUCTIONS

- Write clearly in capital letters using a black ball point pen and press firmly so that all copies are legible
- Do not write in shaded areas
- Answer ALL questions on every page
- Enter "N/A" if information requested is not available
- Initial and date all changes, additions or deletions. Use a single pen stroke to remove an incorrect entry
- Enter the subjectt initials on all pages on which information has been entered and the subject number and centre number where required

Do not enter additional data/information after the set of CRF pages has been split/separated

The cover flap or plastic writing board should be used to divide each set of pages before writing on them with a ball point pen

DATE

Please use the following three-letter abbreviations for the months

January	JAN
February	FEB
March	MAR
April	APR
May	MAY
June	JUN
July	JUL
August	AUG
September	SEP
October	OCT
November	NOV
December	DEC

e.g. | 0 1 | J A N | 9 9 | *= 1st January 1999*

Day Month Year

TIME

Unless specified otherwise, please use the 24 hour clock

e.g. | 1 5 | 3 0 | *= 3.30 p.m*

24hr:min

SUBJECT INITIALS

Please use the following format:

e.g. | J | P | A | S | *= John Peter Andrew Smith*

ROUTE KEY FOR PRIOR/CONCOMITANT MEDICATION PAGE

PO	=	*By mouth*	*SL*	=	*Sublingual*	*TD*	=	*Transdermal*
IV	=	*Intravenous*	*IT*	=	*Intrathecal*	*IH*	=	*Inhaled*
IM	=	*Intramuscular*	*VA*	=	*Vaginal*	*SC*	=	*Subcutaneous*
IAR	=	*Intra-arterial*	*NA*	=	*Nasal*	*PR*	=	*Rectal*
TO	=	*Topical*	*IA*	=	*Intra-articular injection*	*ID*	=	*Intradermal*

Study medication

If the subject reports vomiting within one hour of dosing, the same dose will be re-administered the next day. If the subject sequentially vomits two doses of study drug, he / she will be withdrawn from the study as intolerant of medication and the Study Conclusion page will be completed.

1. Dose Taken

Where dose is subsequently re-administered due to vomiting, this refers to first dose only.

2. Symptoms of Malaria

Refer to the information on the field workers visit report form.

3. Pregnancy test

If positive for pregnancy, subject must be withdrawn from prophylaxis treatment. This must be reported immediately as a Serious Adverse Event.

4. Concomitant Medication

Any changes in concomitant medication, including dose changes, should be recorded on the 'Prior and Concomitant Medication' page in the relevant section of the CRF.

5. Adverse Experiences

Elicit AEs by asking the standard non-leading question "Have you felt unwell at all since the last visit ?". Any reported should be recorded in detail on the AE pages in the relevant section of the CRF.

6. Parasitaemia

If the subject has a positive parasitaemia, withdraw subject from study medication. Continue to follow-up the subject and complete the Safety Follow-Up Visits at the back of this book.

7. Safety Blood Samples

Record results in relevant section of CRF.

8. PK (Pharmacokinetic) Blood Sample

It is vital that the date and time of sample collection are noted and recorded as accurately as possible, using the 24 hour clock time. Attempts should be made to avoid all samples within a specific window to be at one of the extreme ends of the window (eg. 6-12 h post-dose window - not all samples should be taken at 6 h).

Patient's withdrawing from study medication

If subject is to be withdrawn from study medication, complete the Safety Follow-Up visits and then the Study Conclusion page.

INSTRUCTIONS FOR REPORTING SERIOUS ADVERSE EXPERIENCES (SAE)

SAEs MUST BE REPORTED TO SMITHKLINE BEECHAM WITHIN 24 HOURS

COMPLETE THE SAE PAGES OPPOSITE

Please complete these pages as fully and accurately as possible in order to minimise the time you spend dealing with data queries

If the SAE is still ongoing at the time of reporting, please leave 'Experience Course' blank and update it later

SIGN AND DATE THE SAE PAGE

PLEASE ENSURE THAT ALL OF THE INFORMATION ON THE FOLLOWING CRF PAGES IS COMPLETE

- *Demography*
- *Significant Medical/Surgical History and Physical Examination*
- *Study Medication Record*
- *Concomitant Medication*
- *Form D (if applicable)*

PHOTOCOPY THE SAE PAGES AND THE CRF PAGES SPECIFIED ABOVE
(Do not separate the NCR pages)

FAX A COPY OF THE SAE PAGES AND ALL OF THE CRF PAGES SPECIFIED ABOVE TO:

Your local SB CRA/Medical Monitor (see Investigator Site File for appropriate fax number)

If no photocopier OR fax is available please telephone your local SB CRA/Medical monitor within 24 hours

OBS: 8000. SAE

VISIT: SAE

00000

Page	45

Protocol		Subject Number	Subject Initials	SB Receipt Date (Day Month Year)	
252263/030		0 0 0 0 0			

SERIOUS ADVERSE EXPERIENCE (SAE) ANYYN <XYESNO>

ACE <ACE1S> SERYN <XYESNO = Y>

Person Reporting SAE ~~(Please print clearly)~~	NOT ENTERED	AEGIS Number: AE_NO
Serious Adverse Experience (Please print clearly)	AE [AEMOD, WHOAE NOTIFY <XNOTIFY = L>]	→ *Specify reason(s) for considering this a serious AE. Mark all that apply.* SERCOD * <XSERCOD><br>(1) ☐ fatal<br>(2) ☐ life threatening<br>(3) ☐ disabling/incapacitating<br>(4) ☐ results in hospitalisation (excluding elective surgery or routine clinical procedures)<br>(5) ☐ hospitalisation prolonged<br>(6) ☐ congenital abnormality<br>(7) ☐ cancer<br>(8) ☐ overdose<br>(9) ☐ Investigator considers serious or a significant hazard, contraindication, side effect or precaution<br>* NB: CHECKLIST ∴ MORE 1 CODE CAN BE ENTERED - SEPARATED BY COMMA
For SmithKline Beecham		
Onset Date and Time<br>ONSETD, ONSETT	Day Month Yr 24hr:min	
End Date and Time (If ongoing please leave blank)<br>STOPD, STOPT	Day Month Yr 24hr:min	
Outcome<br>*If patient died, please complete Form D*<br>OUTCOME <XOUTCOME>	(1) Resolved<br>(2) Ongoing<br>(3) Died	
Experience Course COURSE <XCOURSE1>	(1) Intermittent → No. of episodes<br>(2) Constant	
Intensity (maximum) INTENSIT <XINTENSIT>	(1) Mild<br>(2) Moderate<br>(3) Severe	
Action Taken with Respect to Investigational Drug<br>ACTION <XACTION3>	(1) None<br>(2) Dose reduced<br>(3) Dose increased<br>(4) Drug interrupted/restarted<br>(5) Drug stopped	Did the SAE abate? ☐ Yes ☐ No<br>**If study medication was interrupted, stopped or dose reduced:**<br>Was study medication reintroduced (or dose increased)? ☐ Yes ☐ No<br>If yes, did SAE recur? ☐ Yes ☐ No
Relationship to Investigational Drug<br>RELATN3 <XRELATN3>	(1) Not related<br>(2) Unlikely<br>(3) Suspected (reasonable possibility)<br>(4) Probable	→ **Assessment**<br>The SAE is probably associated with:<br>☐ Protocol design or procedures (but not to study drug) NOT ENTERED<br>*Please specify* ______<br>☐ Another condition (eg, condition under study, intercurrent illness)<br>*Please specify* ______<br>☐ Another drug<br>*Please specify* ______
Corrective Therapy<br>*If 'Yes', record details in the Concomitant Medication section*	(Y) Yes (N) No<br>TREATYN <XYESNO>	
Was patient withdrawn due to this specific SAE ?	(Y) Yes (N) No<br>WITHAE <XYESNO>	

Protocol		Subject Number	Subject Initials	SB Receipt Date (Day Month Year)
252263/030		0 0 0 0 0		

SERIOUS ADVERSE EXPERIENCE (SAE) (cont)

Relevant Laboratory Data
Please provide relevant abnormal laboratory data below

Test	Date	Value	Units	Normal Range
	Day Month Yr			
	Day Month Yr			
	Day Month Yr			
	Day Month Yr			

Remarks *(Please provide a brief narrative description of the SAE, attaching extra pages eg. hospital discharge summary if necessary)*

NOT ENTERED

If applicable, was randomisation code broken at investigational site? ☐ No ☐ Yes

Randomisation / Study Medication Number:

Investigator's Signature: ______________________ Date (Day Month Year)
(confirming that the above data are accurate and complete)

Please PRINT Name ______________________

SB Medical Monitor's Signature: ______________________ Date (Day Month Year)

Please PRINT Name ______________________

Protocol		Subject Number	Subject Initials		Parasitaemia Slide Reading
252263/030		0 0 0 0 0			PAR1

PARASITAEMIA SLIDE READING - (Screening and Day -2)

PARASIT <PARASIT5>

1 Reader 1 2 Reader 2 9 Arbitrator reading READER <XREADER>

Visit	Sample Date	Results		Species count (per 500 WBC)			
	Day Month Year	Pos	Neg	P. falciparum (8)	P. malariae (9)	P. ovale (10)	P. vivax (11)
Screen OBS:1000.SCR	PERFDAT	1	2	PARASA			
Day-2 (Post-Halofantrine Clearance) OBS:1040.D2CL							
Unscheduled OBS:1210.UNS1							
Unscheduled OBS:1220.UNS2							
Unscheduled OBS:1230.UNS3							
Unscheduled OBS:1240.UNS4							

PARASQL <XPARAQS>=6

PARASA <XPOSNEG> <XNT CODED>

SDAT

Result

1, 2, 9

3, 4, 8

Protocol		Subject Number	Subject Initials		Parasitaemia Slide Reading PAR2
252263/030		0 0 0 0 0			

PARASITAEMIA SLIDE READING - (Week 1 to Week 8)

[SEE PAGE PAR1]

☐ Reader 1 ☐ Reader 2 ☐ Arbitrator reading

Visit	Sample Date Day Month Year	Results		Species count (per 500 WBC)			
		Pos	Neg	P. falciparum	P. malariae	P. ovale	P. vivax
Week 1 OBS: 2030.W1							
Week 2 OBS: 2040.W2							
Week 3 OBS: 2050.W3							
Week 4 OBS: 2060. W4							
Week 5 OBS: 2070. W5							
Week 6 OBS: 2080.W6							
Week 7 OBS: 2090.W7							
Week 8 OBS: 2100. W8							
Unscheduled OBS: 2210.UNS1							
Unscheduled OBS: 2220.UNS2							
Unscheduled OBS: 2230. UNS3							
Unscheduled OBS: 2240. UNS4							

Protocol		Subject Number	Subject Initials		Parasitaemia Slide Reading PAR3
252263/030		0 0 0 0 0			

PARASITAEMIA SLIDE READING - (Week 9 to Week 16)

[SEE PAGE PAR1]

☐ Reader 1 ☐ Reader 2 ☐ Arbitrator reading

Visit	Sample Date Day Month Year	Results Pos	Results Neg	Species count (per 500 WBC) P. falciparum	P. malariae	P. ovale	P. vivax
Week 9 OBS: 3000.W9							
Week 10 OBS: 3010.W10							
Week 11 OBS: 3020.W11							
Week 12 OBS: 3030.W12							
Week 13 OBS: 3040.W13							
Week 14 OBS: 3050.W14							
Week 15 OBS: 3060.W15							
Week 16 OBS: 3070.W16							
Unscheduled OBS: 3210.UNS1							
Unscheduled OBS: 3220.UNS2							
Unscheduled OBS: 3230.UNS3							
Unscheduled OBS: 3240.UNS4							

Protocol		Subject Number	Subject Initials		Parasitaemia Slide Reading PAR4
252263/030		0 0 0 0 0			

PARASITAEMIA SLIDE READING - (Week 17 to Week 24)

[SEE PAGE PAR1]

☐ Reader 1 ☐ Reader 2 ☐ Arbitrator reading

Visit	Sample Date	Results		Species count (per 500 WBC)			
	Day Month Year	Pos	Neg	P. falciparum	P. malariae	P. ovale	P. vivax
Week 17 OBS: 4000.W17		☐	☐				
Week 18 OBS: 4010.W18		☐	☐				
Week 19 OBS: 4020.W19		☐	☐				
Week 20 OBS: 4030.W20		☐	☐				
Week 21 OBS: 4040.W21		☐	☐				
Week 22 OBS: 4050.W22		☐	☐				
Week 23 OBS: 4060.W23		☐	☐				
Week 24 OBS: 4070.W24		☐	☐				
Unscheduled OBS: 4210.UNS1		☐	☐				
Unscheduled OBS: 4220.UNS2		☐	☐				
Unscheduled OBS: 4230.UNS3		☐	☐				
Unscheduled OBS: 4240.UNS4		☐	☐				

Protocol		Subject Number	Subject Initials		Parasitaemia Slide Reading PAR5
252263/030		0 0 0 0 0			

PARASITAEMIA SLIDE READING - (Safety Follow-up Visits)

[SEE PAGE PAR1]

☐ Reader 1 ☐ Reader 2 ☐ Arbitrator reading

Visit	Sample Date Day Month Year	Results		Species count (per 500 WBC)			
		Pos	Neg	P. falciparum	P. malariae	P. ovale	P. vivax
Safety Follow-Up Visit 1 (Week 25)	OBS: 5000.SF1						
Safety Follow-Up Visit 2 (Week 26)	OBS: 5010.SF2						
Safety Follow-Up Visit 3 (Week 27)	OBS: 5020.SF3						
Safety Follow-Up Visit 4 (Week 28)	OBS: 5030.SF4						
Unscheduled	OBS: 5210.UNS1						
Unscheduled	OBS: 5220.UNS2						
Unscheduled	OBS: 5230.UNS3						
Unscheduled	OBS: 5240.UNS4						

Protocol		Subject Number	Subject Initials	Investigator
252263/030		0 0 0 0 0		

CRF TRACKING/REVIEW FORM

Please submit COMPLETE Modules only. Do NOT submit whole blank modules.

☐ Screening Failure (archive only)

Module	Module Description	Page Range	Modules: Sent to DM	Modules: Unused	Missing Pages (if Applicable)
MOD1	Screening to Randomisation	1 - 9	☐	☐	
MOD2	Loading Dose 1 to Week 8	10 - 18	☐	☐	
MOD3	Week 9 to 16	19 - 27	☐	☐	
MOD4	Week 17 to 24	28 - 36	☐	☐	
SFUP	Safety Follow-up Visits	37 - 43	☐	☐	
CONC1	Study Conclusion	44	☐	☐	
PAR1R1	Parasite: Scrn & Day -2 Reader 1	n/a	☐	☐	
PAR1R2	Parasite: Scrn & Day -2 Reader 2	n/a	☐	☐	
PAR1AR	Parasite: Scrn & Day -2 Arbitrator	n/a	☐	☐	
PAR2R1	Parasite: Wk 1 - Wk 8 Reader 1	n/a	☐	☐	
PAR2R2	Parasite: Wk 1 - Wk 8 Reader 2	n/a	☐	☐	
PAR2AR	Parasite: Wk 1 - Wk 8 Arbitrator	n/a	☐	☐	
PAR3R1	Parasite: Wk 9 - Wk 16 Reader 1	n/a	☐	☐	
PAR3R2	Parasite: Wk 9 - Wk 16 Reader 2	n/a	☐	☐	
PAR3AR	Parasite: Wk 9 - Wk 16 Arbitrator	n/a	☐	☐	
PAR4R1	Parasite: Wk 17 - Wk 24 Reader 1	n/a	☐	☐	
PAR4R2	Parasite: Wk 17 - Wk 24 Reader 2	n/a	☐	☐	
PAR4AR	Parasite: Wk 17 - Wk 24 Arbitrator	n/a	☐	☐	

OBS: 9000. DIED
VISIT: DIED.

00000

Protocol 252263/030		Subject Number 0 0 0 0 0	Subject Initials		FORM D

FORM D

DIED <DIED1> [NB: ONLY ENTERED AS A RECORD IF PATIENT DIED]

Certified Cause of Death DEATHTXT

Date of Death DEATHDAT (Day Month Yr) → *Complete Adverse Experience section*

Was a post-mortem Performed ?

- [N] No ANSYN <XYESNO>
- [Y] Yes → *If 'Yes' please summarise findings (include diagnosis):*

ANSDT

Physician's Signature NOT ENTERED

Date

00000

Protocol 252263/030	Centre Number ☐☐☐				

LABORATORY REFERENCE RANGES

NR <NR>

Investigator ________________________________

For SB | Lab Number Code_ LABCODE_____

Date for which these reference ranges become effective DSTART (Day Month Yr)

Laboratory Name ________________________________

Address ________________________________ LOWAGE HIGHAGE ____

(M) Male reference range SEX <XSEX> (F) Female reference range Age range ☐☐ - ☐☐ Years AGEUNITS

Enter the reference range for each test. If the reference ranges for any test differ by sex or age, complete a separate form for each variation, e.g., males 0-16, males 17-99, females 0-16, females 17-99 years. Complete the units reported by this laboratory for each test.

	Test LPARAM <XLPARAM>	Unit UNITS <XUNITS	Low Reference Value LOW / LOWT	High Reference Value HIGH / HIGHT
Haematology	White Blood Cells WBC			
	Haematocrit HCT			
	Haemoglobin HGB			
	Platelets PLATE			
Clin. Chem.	Creatinine CREAT			
	Alanine transaminase (ALT) ALAT			
	BUN BUN			

Protocol		Subject Number	Subject Initials	Investigator
252263/030		0 0 0 0 0		________________________

CRF TRACKING/REVIEW FORM - Continued

Please submit COMPLETE Modules only. Do NOT submit whole blank modules.

Module	Module Description	Page Range	Modules		Missing Pages (if Applicable)
			Sent to DM	Unused	
PAR5R1	Parasite: Safety F-up Reader 1	n/a	☐	☐	________________
PAR5R2	Parasite: Safety F-up Reader 2	n/a	☐	☐	________________
PAR5AR	Parasite: Safety F-up Arbitrator	n/a	☐	☐	________________
SAE1	Serious Adverse Experience	45 - 46	☐	☐	________________
FORMD	Form D	n/a	☐	☐	________________

If multiple modules are submitted together, please ensure they are sorted according to the order above

Site Monitor's Comments (e.g. protocol violations or deviations)

Site Monitor's Signature ________________________________ Date Submitted [| |] Day Month Yr

Instructions for Parasitaemia Slide Reading

Tafenoquine Clinical Trial 252263/030

1) Introduction

Tafenoquine is an 8-aminoquinoline compound analagous to primaquine, being co-developed by the US Army (USAMRU) and SmithKline Beecham Pharmaceuticals (SB), with *in vitro* activity against multi-resistant *Plasmodium falciparum* and chloroquine-resistant *P.vivax* malarial parasites.

With the exception of long-term follow-up in one study, the phase II clinical programme is complete. Efficacy and safety in chemoprophylaxis, radical cure and post-exposure prophylaxis were evaluated and on the basis of these results the drug is now being progressed into larger phase III clinical trials.

Study 252263/030 is currently ongoing in Kenya and is the first of four planned phase IIIa clinical studies. The observed prohylaxis failure rate has been significantly higher than would be expected from the protective efficacies observed in Phase II studies, and by the 10 week asssessment visit 207 of the 306 subjects randomised to prophylactic study medication had been withdrawn due to positive parasitaemia. As a result of this an Independent Data Monitoring Committee has been put in place to recommend whether or not the study should be terminated early, and a series of investigations are being undertaken to try to determine the reasons for this unexpected high failure rate.

As part of these investigations, it has been decided that a selection of the blood smears will be re-read by a further two independent readers to confirm the initial results. This excersise will be undertaken by the laboratories of NAMRU I-2, Jakata.

In order maintain consistency of reporting it is essential that the methodology for the re-reads is the same as that used initially in Kenya. This document summarises the procedure that should be followed.

The USAMRU Standard Operating Procedure (SOP) detailing the whole process including smear taking and slide prepration is given in Appendix 1.

2) Numbers of Slides

After a single finger prick duplicate thick and thin slides were prepared.

Approximately 500 pairs of slides will be re-read, a mixture of positives and negatives in proportions unknown to the new readers.

3) Selection of slides

The slides will be randomly selected by a list generated by SB Biometrics Department. They will be packaged and shipped by the Investigator, Dr Jose Stoute at the study site in Kenya, and shipped to Jakarta for the purposes of this exercise. They will be returned to Dr Stoute when the re-reads are complete.

4) Slide reading

Asexual stage parasites and gametocytes in 200 high power fields will be counted and reported as the number of parasites per 500 white blood cells. If the slide obviously contains parasites only 50 fields may be counted.

A. Duplicate smears will be read by two separate microscopists and results recorded on a blood smear worksheet (provided). Each microscopist will have his own worksheet and be blinded to the others' results. A further individual will be assigned to collate the results of the two readers.

B. In the event of a positive/negative malarial smear discrepancy between two microscopists, a third microscopist will be utilized (preferably the senior technician). The third "tie breaker" slide reading will be used to confirm positive or negative parasitemia from the initial slide that was reported negative and this result will be the final diagnosis.

C. All three sets of results be will recorded on the forms provided, recording positive/negative, species, and numbers of parasites seen per 500 WBC, and will be entered into an Excel spreadsheet.

Methods

1) On the thick smear count the number of asexual parasites and WBCs seen in 50 HPFs. Keep separate counts of the WBCs and parasites. **A minimum of 200 high power fields must be viewed before a slide/patient can be resulted as negative**.

2) If more than 50 HPFs are scanned, calculate the number of WBCs in the total number of fields scan based on the number of WBCs seen in 50 HPFs (see calculation below).

3) Whenever there is less than 0.01 parasites per 500WBCs, then the parasitemia will be recorded as <0.01/500WBCs.

4) Gametocytes in the number of fields scanned will be reported separately.

5) Use the following formula to calculate parasitemia:

$$\frac{\text{\# parasites Counted}}{\text{\# WBC's counted}} \times 500 = \text{\# Parasites/500WBCs}$$

Where #WBCs counted = #WBCs/50HPF x #HPFs scanned

NOTE: If the senior microscopist is involved with the positive/negative discrepancy, another experienced microscopist will read the "tie breaker" slide.

5) Reporting of results

Results will be reported to Lorraine Martin and Philip Pickford at SmithKline Beecham, and also to Dr. Stoute, in the form of an Exel spreadsheet, at the earliest possible opportunity and within 2 weeks of receipt of the slides.

6) Discrepancies in results

The results of the slide readings from Kenya and the re-reads from NARU-2 will be compared and discrepancies identified by Lorraine Martin and Philip Pickford. PCR will be performed on these slides to determine the presence or absence of parasites.

Subsequent to this number of these discrepant slides, plus a selection of positive slides, may be shipped to Professor Reikman at AMI, Brisbane, for morphological assessment of any parasites found. The number and selection of these slides will be decided at a later date.
All slides will be returned to Dr Stoute as soon as investigations are completed.

APPENDIX A

LABORATORY SERVICES
UNITED STATES ARMY MEDICAL RESEARCH UNIT KENYA
UNIT 64109 BOX 401
APO AE 09831-4109

PREPARED BY	DATE ADOPTED	SUPERSEDES PROCEDURE #
Michael J. Walter, MT(ASCP)		N/A

REVIEW DATE	REVISION DATE	SIGNATURE

STANDARD OPERATING PROCEDURE

TECHNICAL PROCEDURE – HEMATOLOGY
MAY 1999

MALARIAL SMEARS

1. PRINCIPLE:

   The purpose of this SOP is to establish guidelines for the examination of blood smear for the presence of malaria parasites.

2. SPECIMEN:

FINGERSTICK (Follow Capillary Specimen Collection Procedure – Phlebotomy SOP)

A. Ensure that blood taken is free from grease, perspiration, or dirt which may be on the skin and that the alcohol used for cleaning never be allowed to mix with the blood.

B. Cleanse the area to be punctured with an alcohol prep pad or gauze/cotton soaked in alcohol. Allow to alcohol to evaporate before blood is taken for the smear.

C. Prick the skin deep enough to allow the blood to well up in a large drop under gentle pressure but not deep enough to cause excessive bleeding. Puncture is made with a sterile lancet. Lancets are for one time use only.

VENOUS COLLECTION EDTA BLOOD

Thick films from blood collected in EDTA or Heparin anticoagulant can be made in the same manner. Once prepared, however, they should be thoroughly dried at room temperature for at least 30 minutes.

Properly labeled slides will be stored in the slide folders or slide boxes. Handling of potentially infectious material should follow recommended universal precautions as outlined in the Laboratory Safety SOP.

3. EQUIPMENT & MATERIALS:

Stock Giemsa Stain
Buffered Water (pH 6.8-7.2)
Slides
Staining Jars

4. QUALITY CONTROL:

QC slides will be stained to make sure stain is appropriate and that the colors are distinctive. The slides will be scanned for the presence of debris. QC slides will read each day staining occurs.

5. PROCEDURE:

A. Performance Parameters

A well prepared thin film is thick at one end ad thin at the other (one layer of evenly distributed RBC's with no cell overlap). The thin feathered end should be at least 2 cm long, and the film should occupy the central area of the slide, with free margins on each side. Approximately 3 uL of blood is used to make this smear. Long streamers of blood indicate the spreader slide was chipped or dirty. Streaks in the blood film are usually caused by dirt, and holes indicate the presence of grease on the slide. Ensure the slides are labeled with a lead pencil with the date smear taken and the study number.

NOTE: Two malarial slides will be prepared at Kombewa for reading at the Kisian laboratory. The two slides made will be labeled A and B respectively.

Thick films should be about the size of a dime or nickel (1.5 to 2.0 cm in diameter and thick enough to just read newspaper print through). Approximately 10 uL of blood is used to make this type of smear.

Again, thick and thin films will be prepared in duplicate. In the case of an acute smear, an additional slide will be made and read at Kombewa.

B. Thick and Thin Films:

1) After a blood sample has been placed on the slide, the slide will be placed horizontally on a table and allowed to dry at room temperature for 10-15 minutes.

2) Once dry, the slides will be placed upright and the thin smear will be fixed by application of absolute methanol by submersion or by dropping methanol onto the smear placed at an angle of 30 to 40 degrees.

3) Allow films to dry. When dry, the slides will be subjected to a stream of warm air from a heat source for 10 minutes to further dry the thick blood smear. Ensure that the slide is thoroughly dry before staining. The introduction of even a small amount of methyl alcohol will interfere with the dehemoglobinization of the thick film.

4) Stain the films for 10 minutes in a 1:10 solution of Giemsa (10 mL Giemsa to 90 mL Buffered Water pH 6.8-7.2) for clinical purposes.

For research history slides, stain the films for 30-60 minutes in 3% or 5% dilution of Giemsa. If this stain is not available, proceed with the local Giemsa stain and the 1:10 dilution.

5) Wash the smears in tap water until remnants of excess stain are removed. Take care not to wash off the films.

6) Dry the films in a vertical position at room temperature for 10-15 minutes.

6. CALCULATIONS:

A. Qualitative method

1) Positive
2) Negative

B. Semiquantitative method

6) Once the thick smear is ready for reading, count the number of asexual parasites and WBCs seen in 50 HPFs. Keep separate counts of the WBCs and parasites. A minimum of 200 high power fields must be viewed before a slide/patient can be resulted as negative.

7) If more than 50 HPFs are scanned, calculate the number of WBCs in the total number of fields scan based on the number of WBCs seen in 50 HPFs (see calculation below).

8) Whenever there is less than 0.01 parasites per 500WBCs, then the parasitemia will be recorded as <0.01/500WBCs.

9) Gametocytes in the number of fields scanned will be reported separately.

10) Use the following formula to calculate parasitemia:

$$\frac{\text{\# parasites Counted}}{\text{\# WBC's counted}} \times 500 = \text{\# Parasites/500WBCs}$$

Where #WBCs counted = #WBCs/50HPF x #HPFs scanned

7. REPORTING RESULTS:

NOTE: AT LEAST TWO MICROSCOPISTS WILL READ A SMEAR FROM ONE VOLUNTEER SAME DATE TAKEN AT KISIAN. ACUTE SMEARS REQUIRE ONLY ONE MICROSCOPIST READ.

D. Positive or Negative for Parasites

NOTE: If Positive, quantitate in the following manner:

+ = few (0-10 parasites per 500 WBC's)
++ = moderate (11-20 parasites per 500 WBC's)
+++ = many (>20 parasites per 500 WBC's)

E. %Parasitemia

F. Preliminary reads (slides read at Kombewa laboratory) will be reported on a Blood Smear Worksheet (separate sheet for each microscopist). Preliminary reads will be conducted by one microscopist onsite. Final reads will be performed at Kisian and read by two separate microscopists and results recorded on a blood smear worksheet

G. Final results will be transcribed to the malarial diagnosis form. If final malarial smear results from Kisian microscopists agree, the 1st microscopic reading will be documented.

H. In the event of a positive/negative malarial smear discrepancy during the final read between two microscopists, a third microscopist will be utilized (preferably the senior technician). The third "tie breaker" slide reading will be used to confirm positive or negative parasitemia from the initial slide reported negative and this result will be recorded on the final malarial diagnosis form.

NOTE: If the senior microscopist is involved with the positive/negative discrepancy, another experienced microscopist will read the "tie breaker" slide.

NOTE: A SLIDE IS CONSIDERED NEGATIVE AFTER SCANNING THROUGH 200 OIL FIELDS.

8. PROCEDURAL NOTES:

A. Combination films are prepared by placing both thick and thin films on the same slide. Two slides are made per volunteer. The thin film may be smeared toward the thick film or away from it. There should be space between the thick and thin films so that the thin film can be fixed with methanol without fixing the thick smear. The films are dried for 5-10 minutes in a horizontal position at room temperature on slide racks or using a hair dryer.

B. After the initial screening on low power, both thick and thin films should be examined under oil immersion. It is recommended that thin films should have a minimum of 200 fields examined before submitting a negative report.

C. The maximum limit of malarial smears a microscopist will read in a day will be 50.

9. LIMITATIONS:

A. Sources of error:

1) The inexperienced microscopist may be confused in the examination of thick films by bacteria or dirt from the skin; by dust particles on the slide, by vegetable spores, yeast, fungi; and by bacteria, molds, protozoa and other contaminants from the distilled water used in staining. Probably the main source of debris on the thick film is an improperly cleaned slide.

2) Artifacts that may deceive the inexperienced microscopist will be elements found above the blood plane. Sometimes red dots appear in the preparation without any visible cytoplasm. It is possible that some of these are at times remnants of malaria parasites, but one should never call a slide positive on these dots alone. If the dots are evenly distributed throughout the smear, they are more likely to be associated with the cells than if they are found in clumps or only in a part of the smear. If a red-stained coccus from the skin or a small nuclear

remnant lies adjacent to a blue stained particle of cellular substance or fibrin, there may be a resemblance to a parasite. This rarely occurs more than once or twice on a single slide, and numbers of free cocci will usually be found also, whereas free chromatin dots are rarely found in well stained smears. As one becomes more familiar with the thick film, it is less likely to mistake a foreign body for a parasite. Thick films should not be diagnosed as positive on what appears to be only one parasite. The parasite should be unmistakable, or the search should be continued until others are found. If this is impossible, another smear should be requested.

10. REFERENCES:

Ash, L.R., Parasites: A Guide to Laboratory Procedures and Identification, ASCP Press Chicago, IL, pgs 99-108; 179-183.

Department of Army: Clinical Laboratory Procedures – Parasitology, AUG 84, pgs 11-30.

NCCLS Document M15-T Vol. 12 No. 15 Tentative Guidelines: section 6

Parasitaemia Slide Reading

Tafenoquine Clinical Trial 252263/030

Reader No. ________

				**Species Count per 500 WBCs**				
**PID**	**Date of Slide**	Pos	Neg	P. fal	P.mal	P. Ov	P. Viv	P.Gamet.

Kenya Tafenoquine Trial
U.S. NAMRU-2 Re-read

Slide ID Number	Date of Slide	KEMRI diagnosis	NAMRU-2 Diagnosis	
1013	9-Jul	pos	PF	1
1019	10-Jul	pos	PF	1
1020	12-Jun	pos	PF	1
1039	19-Jun	pos	PF	1
1044	26-Jun	pos	PF	1
1057	3-Jul	pos	PF	1
1063	26-Jun	pos	PF	1
1064	26-Jun	pos	PF	1
1072	12-Jun	pos	PF	1
1088	3-Jul	pos	PF	1
1101	19-Jun	pos	PF	1
1103	19-Jun	pos	PF	1
1160	18-Jun	pos	PF	1
1160	19-Jul	pos	PF	1
1177	21-Jun	pos	PF	1
1211	21-Jun	pos	PF	1
1222	12-Jul	pos	PF	1
1250	21-Jun	pos	PF	1
1260	26-Jul	pos	PF	1
1287	18-May	pos	neg	
1005	2-Jul	pos	neg	
1010	12-Jun	pos	neg	
1011	22-May	pos	neg	
1022	15-May	pos	neg	
1023	19-Jun	pos	neg	
1029	3-Jul	pos	neg	
1030	10-Jul	pos	neg	
1036	24-Jul	pos	neg	
1037	15-May	pos	neg	
1038	19-Jun	pos	neg	
1041	12-Jun	pos	neg	
1042	19-Jun	pos	neg	
1045	22-May	pos	neg	
1046	19-Jun	pos	neg	
1047	19-Jun	pos	neg	
1055	22-May	pos	neg	
1060	14-Jul	pos	neg	
1061	10-Jul	pos	neg	
1065	26-Jun	pos	neg	
1071	17-Jul	pos	neg	
1073	26-Jun	pos	neg	
1077	3-Jul	pos	neg	
1086	15-May	pos	neg	
1089	15-May	pos	neg	
1097	19-Jun	pos	neg	
1105	15-May	pos	neg	
1108	15-May	pos	neg	
1112	26-Jun	pos	neg	
1113	26-Jun	pos	neg	
1115	3-Jul	pos	neg	
1118	26-Jun	pos	neg	
1119	15-May	pos	neg	
1120	15-May	pos	neg	

Kenya Tafenoquine Trial
U.S. NAMRU-2 Re-read

Slide ID Number	Date of Slide	KEMRI diagnosis	NAMRU-2 Diagnosis
1123	10-Jul	pos	neg
1124	5-Jun	pos	neg
1131	26-Jun	pos	neg
1132	26-Jun	pos	neg
1135	26-Jun	pos	neg
1136	5-Jun	pos	neg
1143	26-Jun	pos	neg
1144	5-Jun	pos	neg
1147	31-Jul	pos	neg
1149	17-Jul	pos	neg
1152	19-Jun	pos	neg
1153	26-Jun	pos	neg
1155	31-Jul	pos	neg
1158	5-Jun	pos	neg
1161	3-Jul	pos	neg
1163	19-Jun	pos	neg
1165	5-Jun	pos	neg
1168	5-Jun	pos	neg
1169	19-Jun	pos	neg
1170	19-Jun	pos	neg
1186	14-Jun	pos	neg
1188	5-Jun	pos	neg
1197	31-May	pos	neg
1198	29-Jun	pos	neg
1201	14-Jun	pos	neg
1203	21-Jun	pos	neg
1209	5-Jul	pos	neg
1212	28-Jun	pos	neg
1217	21-Jun	pos	neg
1221	20-Jul	pos	neg
1225	14-Jun	pos	neg
1229	21-Jun	pos	neg
1230	4-Jul	pos	neg
1234	14-Jun	pos	neg
1237	6-Jun	pos	neg
1238	12-Jun	pos	neg
1239	14-Jun	pos	neg
1240	14-Jun	pos	neg
1248	3-Jul	pos	neg
1249	4-Jul	pos	neg
1253	28-Jun	pos	neg
1255	19-Jul	pos	neg
1268	24-May	pos	neg
1275	28-Jun	pos	neg
1280	27-Jun	pos	neg
1292	12-Jul	pos	neg
1294	12-Jul	pos	neg
1295	19-Jul	pos	neg
1299	24-May	pos	neg
1300	21-Jun	pos	neg
1301	4-Jul	pos	neg
1304	4-Jul	pos	neg
1306	2-Aug	pos	neg

Kenya Tafenoquine Trial
U.S. NAMRU-2 Re-read

Slide ID Number	Date of Slide	KEMRI diagnosis	NAMRU-2 Diagnosis
087	22-May	pos	neg
279 ~~or 267~~	19-May	pos	neg
1002	6-May	neg	neg
1064	7-May	neg	neg
1070	6-May	neg	neg
1228	7-May	neg	neg
105	6-May	neg	neg
138	7-May	neg	neg
149	7-May	neg	neg
163	7-May	neg	neg
169	7-May	neg	neg
181	24-May	neg	neg
184	7-May	neg	neg
191	8-May	neg	neg
195	8-May	neg	neg
222	8-May	neg	neg
230	7-May	neg	neg
250	8-May	neg	neg
255	8-May	neg	neg
258	7-May	neg	neg
265	7-May	neg	neg
269	7-May	neg	neg
363	15-May	neg	neg
451	15-May	neg	neg
487	22-May	neg	neg
491	15-May	neg	neg
1003	29-May	neg	neg
1005	15-May	neg	neg
1005	15-May	neg	neg
1006	22-May	neg	neg
1006	15-May	neg	neg
1008	22-May	neg	neg
1010	15-May	neg	neg
1010	22-May	neg	neg
1014	29-May	neg	neg
1014	29-May	neg	neg
1015	22-May	neg	neg
1021	13-May	neg	neg
1021	29-May	neg	neg
1022	12-Jun	neg	neg
1022	12-Jun	neg	neg
1023	22-May	neg	neg
1024	29-May	neg	neg
1025	29-May	neg	neg
1026	15-May	neg	neg
1027	22-May	neg	neg
1033	15-May	neg	neg
1040	19-Jun	neg	neg
1042	15-May	neg	neg
1044	15-May	neg	neg
1048	15-May	neg	neg
1049	22-May	neg	neg
1051	17-May	neg	neg

Kenya Tafenoquine Trial
U.S. NAMRU-2 Re-read

Slide ID Number	Date of Slide	KEMRI diagnosis	NAMRU-2 Diagnosis
1056	15-May	neg	neg
1058	15-May	neg	neg
1058	15-May	neg	neg
1060	22-May	neg	neg
1062	15-May	neg	neg
1063	22-May	neg	neg
1064	15-May	neg	neg
1064	18-Jun	neg	neg
1066	22-May	neg	neg
1066	15-May	neg	neg
1067	15-May	neg	neg
1070	15-May	neg	neg
1077	22-May	neg	neg
1078	22-May	neg	neg
1080	29-May	neg	neg
1085	29-May	neg	neg
1085	22-May	neg	neg
1086	22-May	neg	neg
1089	15-May	neg	neg
1089	22-May	neg	neg
1091	15-May	neg	neg
1092	15-May	neg	neg
1093	15-May	neg	neg
1093	22-May	neg	neg
1094	29-May	neg	neg
1097	29-May	neg	neg
1098	24-May	neg	neg
1104	15-May	neg	neg
1104	15-May	neg	neg
1105	29-May	neg	neg
1106	17-May	neg	neg
1107	22-May	neg	neg
1110	22-May	neg	neg
1116	15-May	neg	neg
1116	29-May	neg	neg
1117	15-May	neg	neg
1120	15-May	neg	neg
1122	15-May	neg	neg
1122	22-May	neg	neg
1130	29-May	neg	neg
1132	15-May	neg	neg
1133	22-May	neg	neg
1133	15-May	neg	neg
1133	15-May	neg	neg
1135	15-May	neg	neg
1135	15-May	neg	neg
1136	15-May	neg	neg
1141	15-May	neg	neg
1143	22-May	neg	neg
1147	15-May	neg	neg
1149	22-May	neg	neg
1150	29-May	neg	neg
1150	15-May	neg	neg

Kenya Tafenoquine Trial
U.S. NAMRU-2 Re-read

Slide ID Number	Date of Slide	KEMRI diagnosis	NAMRU-2 Diagnosis
1152	22-May	neg	neg
1152	15-May	neg	neg
1152	15-May	neg	neg
1154	31-Jul	neg	neg
1154	22-May	neg	neg
1159	15-May	neg	neg
1161	15-May	neg	neg
1161	17-May	neg	neg
1162	15-Jun	neg	neg
1164	17-May	neg	neg
1167	17-May	neg	neg
1174	17-May	neg	neg
1174	24-May	neg	neg
1179	17-May	neg	neg
1182	17-May	neg	neg
1183	17-May	neg	neg
1183	17-May	neg	neg
1185	17-May	neg	neg
1187	24-May	neg	neg
1190	17-May	neg	neg
1196	21-Jun	neg	neg
1204	24-May	neg	neg
1204	17-May	neg	neg
1207	24-May	neg	neg
1212	17-May	neg	neg
1216	17-May	neg	neg
1217	17-May	neg	neg
1217	24-May	neg	neg
1221	11-Jun	neg	neg
1224	24-May	neg	neg
1225	24-May	neg	neg
1225	17-May	neg	neg
1228	24-May	neg	neg
1229	14-Jun	neg	neg
1230	17-May	neg	neg
1231	24-May	neg	neg
1238	17-May	neg	neg
1238	24-May	neg	neg
1239	17-May	neg	neg
1239	24-May	neg	neg
1246	17-May	neg	neg
1246	24-May	neg	neg
1262	17-May	neg	neg
1262	24-May	neg	neg
1266	17-May	neg	neg
1266	24-May	neg	neg
1271	17-May	neg	neg
1271	29-May	neg	neg
1273	17-May	neg	neg
1273	14-Jun	neg	neg
1275	6-May	neg	neg
1279	15-May	neg	neg
1281	6-May	neg	neg

Kenya Tafenoquine Trial
U.S. NAMRU-2 Re-read

Slide ID Number	Date of Slide	KEMRI diagnosis	NAMRU-2 Diagnosis
1290	6-May	neg	neg
003	7-May	neg	neg
003	7-May	neg	neg
009	6-May	neg	neg
011	6-May	neg	neg
017	12-May	neg	neg
020	7-May	neg	neg
025	8-May	neg	neg
026	6-May	neg	neg
031	15-May	neg	neg
037	6-May	neg	neg
040	6-May	neg	neg
043	6-May	neg	neg
044	15-May	neg	neg
046	6-May	neg	neg
051	6-May	neg	neg
053	7-May	neg	neg
053	6-May	neg	neg
055	7-May	neg	neg
056	7-May	neg	neg
057	6-May	neg	neg
059	6-May	neg	neg
067	7-May	neg	neg
071	6-May	neg	neg
072	7-May	neg	neg
074	18-May	neg	neg
075	6-May	neg	neg
076	7-May	neg	neg
078	7-May	neg	neg
079	7-May	neg	neg
082	8-May	neg	neg
101	7-May	neg	neg
108	8-May	neg	neg
110	7-May	neg	neg
125	6-May	neg	neg
136	8-May	neg	neg
140	7-May	neg	neg
141	8-May	neg	neg
153	7-May	neg	neg
154	7-May	neg	neg
156	8-May	neg	neg
166	7-May	neg	neg
172	7-May	neg	neg
177	8-May	neg	neg
186	7-May	neg	neg
192	8-May	neg	neg
192	7-May	neg	neg
199	8-May	neg	neg
217	8-May	neg	neg
218	7-May	neg	neg
223	7-May	neg	neg
228	7-May	neg	neg
231	8-May	neg	neg

Kenya Tafenoquine Trial
U.S. NAMRU-2 Re-read

Slide ID Number	Date of Slide	KEMRI diagnosis	NAMRU-2 Diagnosis
236	7-May	neg	neg
244	8-May	neg	neg
247	7-May	neg	neg
254	7-May	neg	neg
264	8-May	neg	neg
266	7-May	neg	neg
267	7-May	neg	neg
285	7-May	neg	neg
290	7-May	neg	neg
304	8-May	neg	neg
313	8-May	neg	neg
320	7-May	neg	neg
321	7-May	neg	neg
334	7-May	neg	neg
336	8-May	neg	neg
339	8-May	neg	neg
353	7-May	neg	neg
354	7-May	neg	neg
358	8-May	neg	neg
368	7-May	neg	neg
370	8-May	neg	neg
378	8-May	neg	neg
390	7-May	neg	neg
392	7-May	neg	neg
393	7-May	neg	neg
401	8-May	neg	neg
403	7-May	neg	neg
412	7-May	neg	neg
415	6-May	neg	neg
420	7-May	neg	neg
424	8-May	neg	neg
427	7-May	neg	neg
428	7-May	neg	neg
429	7-May	neg	neg
473	8-May	neg	neg
489	19-Jun	pos	nd
493	26-Jun	pos	nd
505	19-Jun	pos	nd
519	14-Jun	pos	nd
146	6-May	neg	nd
1135	29-May	neg	nd
060	8-May	neg	nd
087	6-May	neg	nd
095	7-May	neg	nd
386	8-May	neg	nd
**278**	**14-Jun**	**?**	**nd**

REPORT ON EXAMINATION OF THICK BLOOD FILMS FROM KENYA

Tafenoquine Clinical Trial 252263/030

Introduction

AMI received 7 slide boxes containing slides from NAMRU 2, Jakarta, Indonesia, on 30 October 2000. These slides, containing thick and thin blood films, had previously been forwarded from USAMRU, Kenya to NAMRU 2 for examination of malaria parasites. Blood films had been made during the first clinical study in the Phase III development program for tafenoquine, study number SB-252263/030, which was initiated in Kenya in April 2000. Early in the study it became apparent that a higher than expected failure rate was occurring leading to subject withdrawal. This led to a series of investigations to determine the cause.

Randomly-selected slides were examined at NAMRU 2 by experienced malaria microscopists. Briefly, the re-read of 364 slides showed that only 19 of the 113 films found to be positive for *Plasmodium falciparum* in Kenya could be confirmed to be positive in Indonesia. The remaining 345 blood films were either negative (334) or unevaluable (11).

The 113 blood films found to be positive in Kenya were examined at AMI between 1-22 November 2000. I examined 81 blood films and my most trusted microscopist, Tom Travers, examined the remaining 32 films because I just didn't have the time to examine them all myself.

Results

Quality of thick blood films

In general, the blood films were made well and stained well. However, the results from 19 blood films are unclear (see table) because of poor staining, excessive debris or, mainly, insufficient blood on the slides (unable to examine 200 oil immersion fields with more than 1-2 white cells per field). This means that parasites may be missed because too little blood can be examined and counts of ?parasites or artefacts would have been expressed against fewer than 500 WBC's.

Microscopy

The findings showed that 19 of the blood films were positive for *P. falciparum.* (see table1). One blood film (no. 1169) could possibly also have *P. malariae.* On the whole, parasite densities were low.

Morphology of parasites

Parasites were all rings at various stages of development. Some blood films showed a preponderance of small rings while others had mostly mature forms. Parasites appeared normal; the influence of any drug effect could not be determined.

Comments

The results indicate that only 19 of 113 (16.8%) "positive" Kenyan slides were, in fact, positive for malaria parasites.

The reasons for this are one or more of the following:

1. Poor ability to recognise malaria parasites by the examiners due to lack of training, experience and/or motivation.
2. Inadequate supervision
3. Inadequate microscopes or microscopic illumination

The reasons for this are not one or more of the following:

1. Poor preparation and staining of slides
2. 'Over-reading' of slides. If anything, there was 'under-reading'.

I am very curious to find out how many of the positives were in the placebo group. If PCR analysis is needed to confirm microscopic results, this could be done at AMI.

Professor Karl H. Rieckmann
Director
Army Malaria Institute
Gallipoli Barracks
QLD 4051
Australia

Tel. 617 - 3332 4931 or 3332 4801
Fax. 617 - 3331 4800

23 November 2000

Table. Results of thick film examination of Kenyan blood slides

PID	Date	+	-	UNCLEAR	Count/ 500 WBC	Comment
1013	18-May		-	X		Partly understained
1019	09-Jul	+			120	
1020	10-Jul	+			6	
1039	12-Jun	+			14	
1044	19-Jun	+			23	
1057	26-Jun	+			4	
1063	03-Jul	+			9	
1064	26-Jun			X		No blood left on slide - see duplicate
1072	26-Jun	+			8	
1088	12-Jun	+			9	
1101	03-Jul	+			4	
1103	19-Jun	+			15	
1160	19-Jun	+			27	
1160	18-Jun	+			6	
1177	19-Jul		-			
1211	21-Jun		-			
1222	21-Jun	+			5	
1250	12-Jul	+			3	
1260	21-Jun	+			?	Understained
1287	26-Jul	+			14	
1005	02-Jul	+			2	
1010	12-Jun		-			
1011	22-May		-	X		Enough blood for 50 fields
1022	15-May		-			
1023	19-Jun		-			1 artefact
1029	03-Jul		-			
1030	10-Jul		-	X		Enough blood for 41 fields
1036	24-Jul		-			
1037	15-May		-			
1038	19-Jun		-			
1041	12-Jun		-			
1042	19-Jun			X		Only 30 fields available
1045	22-May		-			
1046	19-Jun		-			
1047	19-Jun		-			
1055	22-May		-			
1060	14-Jul		-			1 artefact
1061	10-Jul		-			
1065	26-Jun		-			
1071	17-Jul		-	X		1-2 WBC's/field

PID	Date	+	-	UNCLEAR	Count/ 500 WBC	Comment
1073	26-Jun		-			
1077	03-Jul		-	X		1-2WBC's/field; 1 artefact
1086	15-May		-			
1089	15-May		-			
1097	19-Jun		-			1 artefact
1105	15-May		-			
1108	15-May		-			
1112	26-Jun		-			
1113	26-Jun		-			
1115	03-Jul		-	X		Heavy debris
1118	26-Jun		-			
1119	15-May		-			
1120	15-May		-			
1123	10-Jul		-			
1124	05-Jun		-			
1131	26-Jun		-			
1132	26-Jun		-			
1135	26-Jun		-			
1136	05-Jun		-			1 artefact
1143	26-Jun		-			
1144	05-Jun		-	X		Heavy debris
1147	31-Jul		-			
1149	17-Jul		-			
1152	19-Jun		-			
1153	26-Jun		-			
1155	31-Jul		-			
1158	05-Jun		-			
1161	03-Jul		-	X		Not enough blood - 1-2 WBC's/field
1163	19-Jun		-			
1165	05-Jun		-			
1168	05-Jun		-			
1169	19-Jun	+			9	Possible P. malariae in addition to Pf
1170	19-Jun		-			
1186	14-Jun		-	X		Only 10 fields could be examined
1188	05-Jun		-			
1197	31-May		-			
1198	29-Jun		-			
1201	14-Jun		-			
1203	21-Jun		-			
1209	05-Jul		-			
1212	28-Jun		-			
1217	21-Jun		-			
1221	20-Jul		-			
1225	14-Jun		-			
1229	21-Jun		-			
1230	04-Jul		-			

PID	Date	+	-	UNCLEAR	Count/ 500 WBC	Comment
1234	14-Jun		-			
1237	06-Jun		-			
1238	12-Jun		-			
1239	14-Jun		-	X		Little blood - no WBC's most fields
1240	14-Jun		-			
1248	03-Jul		-			Many artefacts
1249	04-Jul		-			
1253	28-Jun		-			
1255	19-Jul		-			
1268	24-May		-			
1275	28-Jun		-			
1280	27-Jun		-	X		Debris
1292	12-Jul		-			
1294	12-Jul		-	X		Little blood and poor staining
1295	19-Jul		-			
1299	24-May		-	X		Much debris
1300	21-Jun		-			
1301	04-Jul		-	X		Much debris
1304	04-Jul		-			
1306	02-Aug		-			
087	22-May			X		Little blood
279	19-May			X		Poor staining
1002	19-Jun			X		0-1WBC/field
1064	26-Jun	+			**11**	Duplicate slide had no blood left
1070	19-Jun			X		Only 30 fields - 1-2 WBC's/field
1228	14-Jun		-			
278	14-Jun			X		Too little blood

**Name and address of Person Completing Report:** Nasreen Casoojee, CTS-P, Pharm Tech, NFSP-S, Harlow	**Date:** 25 July 2000
	**Study Protocol (if Relevant): 252263/030**

Investigation:

- Review of the packaging documentation, ADLS files and randomisation codes for the affected supplies.

- For further verification, the surplus patient packs have been returned from the centre in Kenya for evaluation. Samples of the study medication were tested for content, degradation and dissolution release rate.
  Twenty-four patient packs, medication numbers 01307 to 01330 inclusive, from Job No. 252263/030KE002 were returned.
  Each patient pack comprises of two 60 cc HDPE bottles, each containing 30 capsules. To ensure optimum data was available, a sample from each un-opened (i.e. tamper-evident seal intact) bottle within each patient pack was tested.

Impact on use of supplies: These are surplus patient packs which have been returned for evaluation. Any impact for this ongoing study will be assessed by the Drug Safety Monitoring Board.

Actions for Follow-up (to be completed by CTS):
No follow-up actions are required for CTS as the packaging is complete for the study.

Investigation Report:

Three hundred and thirty patient supplies (medication nos. 01001 to 01330) were packed under Job No. 252263/030KE002 as per CIS01/PLC02 (Version 01) and shipped to Kenya.

Following the review of the packaging documentation and ADLS files for the affected supplies shipped to Kenya (randomisation nos. 01307 - 01330 inc.), there is no evidence which suggests that the supplies were packed or randomised incorrectly.

Furthermore, analysis of the returned medication (medication numbers 01307 to 01330 inclusive) confirms that the correct treatment was assigned to the correct patient and that the supplies are within specification in terms of chemical stability and dissolution release rate. (Refer to Pharm. Tech. Report No.:

00PTHW_0253/01: Identification and Stability Evaluation of Clinical Supplies KE002 Returned from SB-252263/030 Clinical Study (Kenya.)

NB: The Pharm. Tech. Report No.: 00PTHW_0253/01 will be restricted in its circulation as full disclosure may break the blind.

Therefore, it can be concluded that the high rate of malaria failures observed in this study cannot be attributed to either the integrity of the packaging or the quality or poor stability of the drug products used.

Approval:________________________ **Date:_______________**
HEAD CTS PACKAGING

Approval:________________________ **Date:_______________**
TEAM LEADER
REGULATORY SUPPORT

To: Dr Keith F. Barker

From: George Okafo

Date: 19-Jun-2015

cc: Dr Dave Z Bell
Dr Ann K Miller
Mr Neil Mortimer
Dr Philip D Pickford
Dr Christopher Kell
Lorraine H Martin

Dr Fariba Koosha

SUBJECT: SUMMARY OF LC/MS AND NMR ANALYSIS OF TAFENOQUINE CAPSULES BATCH NUMBER 252263-/030/KEO USED IN STUDY 030 KENYA

As requested, we have analysed the Tafenoquine capsules used in Study 030 Kenya using NMR (F^{19} and H^{1}) and by LC/MS (ESI+ mode) to investigate the possibility that secondary manufacturing had altered/degraded the drug substance, SB-252263-AX. The results have been summarised below

LC/MS (ESI+ mode)
Two capsules (Batch number 252263-/030/KEO) were extracted according to the procedure reported in PDMH-2164/02 and analysed by HPLC according to AS report SB-252263/RSD-10116S/1. The Tafenoquine peak was analysed using positive ion electrospray ionisation (ESI+) on a Micromass Quattro mass spectrometer. The results show no evidence of any other components that could not be accounted for either as fragments or adducts. Additionally, there is no evidence of other components co-eluting with the
Tafenoquine peak.

NMR (F^{19} and H^{1})

Tafenoquine capsules (Batch number 252263-/030/KEO) contents were dissolved in deuterated DMSO and analysed on a Bruker DPX400MHz NMR spectrometer equipped with proton and fluorine –19 nuclei probes. Samples of SB-252263-AX drug substance, batch number TFN-A-02C2 were prepared and analysed similarly for comparison (analytical reference 0A10194). The results showed no evidence of any chemical change in the Tafenoquine drug substance present in the capsule formulation.

George Okafo

PK data report

Date: 18 August 2000

To: Dr. Duolao Wang

From: Ann Miller, DMPK, SB

RE: Study 252263/030 Data for IDMC

This document provides the following information for use at the IDMC meeting to be held on Monday, 21 August, 2000:

A. A summary of tafenoquine concentrations in patients randomized to receive tafenoquine. Data include primarily samples collected following third loading dose (6-12 h post-dose) and at 4 weeks (predose) of the double blind phase.

B. Description of tafenoquine drug analysis method with regard to specificity.

Other documents attached separately are as follows:

C. Summary of the data in support of tafenoquine dose selection for Phase III studies.

D. Excel table containing the raw concentration time data for tafenoquine (mefloquine not yet received).

Tafenoquine assay results:

Drug Assay:

The LC/MS/MS assay for tafenoquine (TQ) is based on a method known as selected reaction monitoring (SRM) detection which is probably the most selective and specific mode of operation used in mass spectrometry. Essentially SRM relies on the following series of events:

1) Isolation of a characteristic ion of the analyte by mass selection. This is a molecular weight/mass marker for the analyte.
2) Excitation of the mass selected species in order to promote decomposition. The resulting abundant fragment ion is essentially a structural marker.
3) Mass selection of the chosen characteristic fragment followed by detection.

In this instance TQ is observed as the protonated species in the gas phase at m/z 464 on the MS mass scale (Mol. weight 463). The fragment selected is m/z 379 which represents loss of the pentylamine moiety.

In order to detect a signal by SRM three acceptance criteria have to be fulfilled; (a) correct chromatographic retention time, (b) correct parent selected and (c) correct fragment ion selected. From work during the assay validation in four species and many studies performed thus far, there has been no indication of selectivity issues in the LC/MS/MS assays. The detection of other compounds (such as methaquine, Mol. weight 379) or metabolites of TQ are highly unlikely due to the fact that the difference in molecular weight will preclude selection in the first stage of the experiment. No peaks with different retention times to that of TQ have been observed in the chromatograms during assay.

The consensus in the drug analysis group is that there is a very miniscule chance that what was measured in these samples could be something other than tafenoquine after looking at the structure of TQ, the LC/MS/MS method used, the full mass spec of standard TQ used in the validation of the method, which showed only minor fragments outside of the parent ion, and the product ion full spectrum of standard TQ. Based on the structure of TQ, it does not appear that dimerization is possible (which could have possibly resulted in an inactive compound). Also based on the high concentrations observed in study 030, and the assay used, it is highly unlikely that anything endogenous could have interfered with the assay.

Tafenoquine is administered as a racemate. The LC/MS/MS assay used does not separate the two enantiomers. But, based on the fact that the *in vitro* drug susceptibility test (performed at Walter Reed) indicated both enantiomers have similar levels of antimalarial activity and the pharmacokinetic profiles of

the enantiomers are similar in healthy volunteer studies, a difference in the ratio of enantiomers in the current TQ formulation, should that have possibly occurred, would not be expected to result in any difference in activity in this study.

Tafenoquine concentration data showed the following:

1. All samples analyzed had quantifiable concentrations of tafenoquine (range: 40.6 - 612 ng/mL).

2. Plasma concentrations were >100 ng/mL (target minimum concentration) following loading dose and weekly dosing up to 4 weeks post first loading dose. The loading dose achieved concentrations ranging from 247-612 ng/mL. Following weekly dosing, concentrations at ca. 4 weeks ranged from 103-461 ng/mL. There is limited data beyond 4 weeks, thus, it is difficult to comment on the relevance of the concentrations observed.

3. Plasma concentrations in failed patients as of 1 (samples taken prior to positive smear) were randomly scattered across the same range as those in patients who did not fail (see table below). Within the 2 groups of data, there is no pattern to suggest that the failures are limited to those subjects for whom concentrations are at the low end of the range. Failures appear to have occurred in subjects with concentrations across the full range of concentrations observed.

Conclusion: Based on the data in hand, tafenoquine plasma concentrations were >100 ng/mL following the loading dose plus weekly dose regimen. The concentrations observed in those patients who eventually failed and those who did not fail were similar during the loading dose and at week 4 based on failure rate as of July 27th.

Mean (SD) tafenoquine concentrations at various times relative to first loading dose (LD)

**Time of sample**	**Non-failure**	**Failure (Prior)***
LD (0-4 days)	421 (105)	383 (86)
Week 4 (25-32 days)	249 (62)	259 (78)

* Samples taken prior to positive smear. Failures as of data received by 27 July.

Mean (SD) tafenoquine concentrations at various times relative to first loading dose by field worker (July 27 failure data)

Field Worker #		Loading Dose		***Week 4***	
		**Non-failure**	**Failure**	**Non-failure**	**Failure**
1	n<br>Mean(SD)	0<br>.	1<br>297	3<br>286 (79)	3<br>298 (17)
2	n<br>Mean(SD)	1<br>413	1<br>329	4<br>258 (47)	1<br>352
3	n<br>Mean(SD)	1<br>331	2<br>436 (217)	2<br>198 (70)	2<br>327 (191)
4	n<br>Mean(SD)	3<br>451 (132)	0<br>.	4<br>238 (26)	1<br>204
5	n<br>Mean(SD)	3<br>438 (127)	2<br>374 (89)	6<br>270 (58)	2<br>262 (0.3)
6	n<br>Mean(SD)	1<br>350.6	0<br>.	2<br>253 (66)	2<br>265 (98)
7	n<br>Mean(SD)	1<br>247	2<br>361 (42)	3<br>196 (19)	2<br>230 (10)
8	n<br>Mean(SD)	1<br>551	1<br>327	2<br>288 (13)	1<br>254
9	n<br>Mean(SD)	1<br>437	0<br>.	3<br>314 (52)	0<br>.
10	n<br>Mean(SD)	1<br>298	1<br>268	1<br>213	1<br>201
11	n<br>Mean(SD)	0<br>.	1<br>415	5<br>184 (53)	3<br>275 (76)
12	n<br>Mean(SD)	1<br>364.8	1<br>501.1	1<br>242.9	2<br>238 (48)
13	n<br>Mean(SD)	1<br>380.6	0<br>.	2<br>291 (15)	2<br>320 (156)
14	n<br>Mean(SD)	1<br>412	1<br>426	2<br>320 (129)	1<br>378
15	n<br>Mean(SD)	0<br>.	2<br>395 (98)	1<br>152	2<br>299 (102)
16	n<br>Mean(SD)	3<br>514.3	0<br>.	5<br>268 (41)	4<br>179 (58)
17	n<br>Mean(SD)	0<br>.	1<br>392	6<br>230 (69)	2<br>182 (36)

Mean (SD) tafenoquine concentrations at Week 4 post first loading dose (LD) by number of samples obtained per patient

Patient status	LD & Week 4	Week 4 only
Non-failures	273 (62) (n=17)	237 (60) (n=35)
Failures*	293 (90) (n=11)	242 (73) (n=20)

* Samples taken prior to positive smear. Failures as of data received by 27 July.

Tafenoquine concentrations following loading dose by field worker
(27 July failure data)

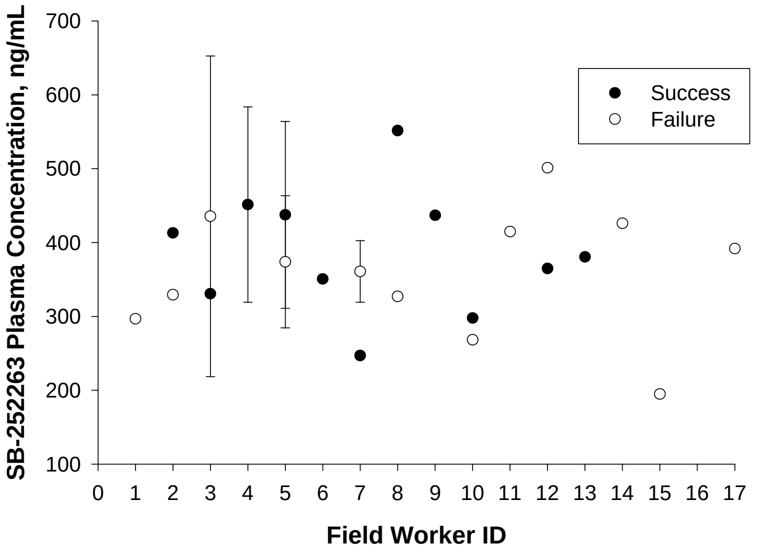

Tafenoquine concentrations at 4 weeks (predose)
(27 July failure data)

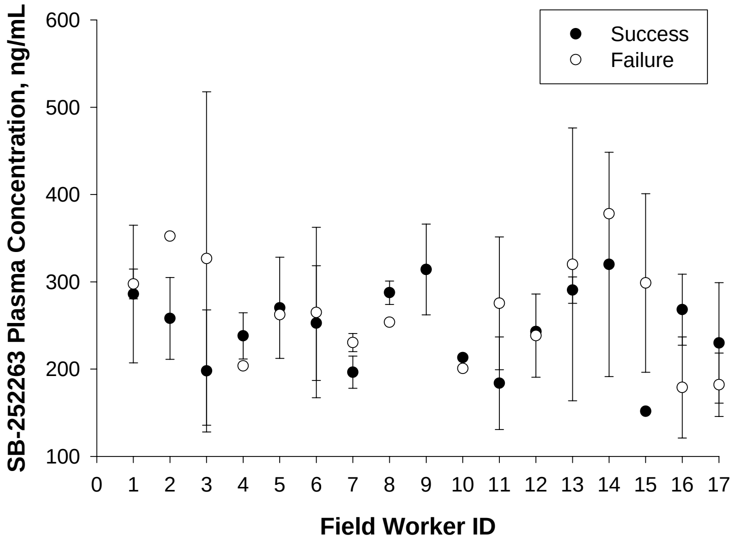

Tafenoquine concentrations relative to first loading dose

(27 July failure data)

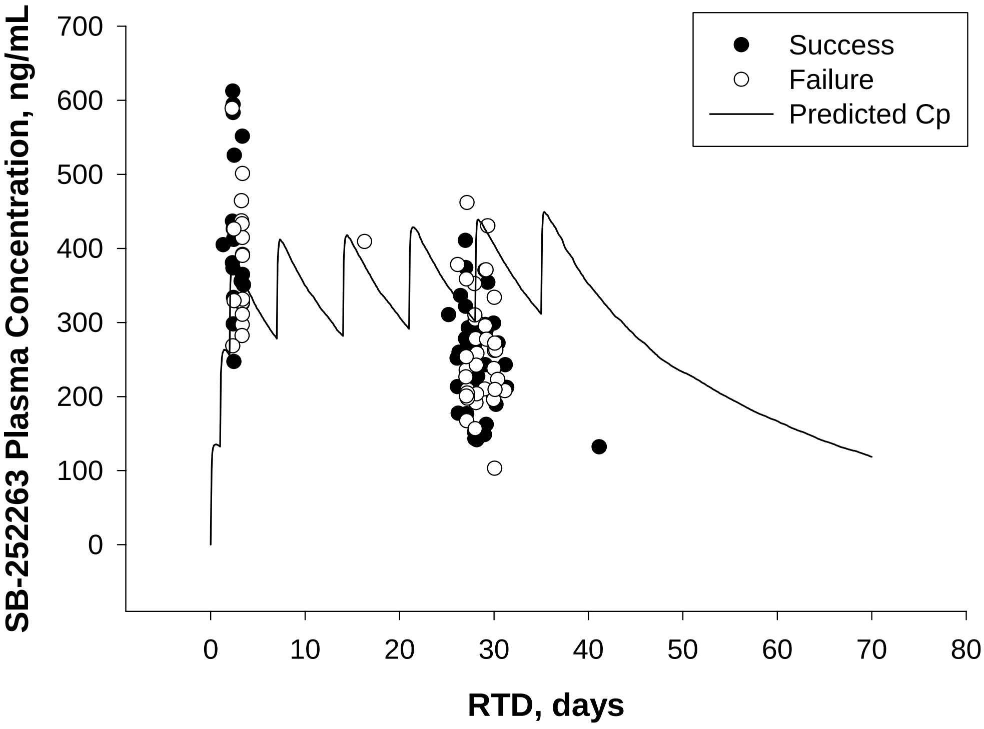

RTD = Relative Time to First Loading Dose

Predicted concentrations based on simulation of clinical dose regimen (200 mg TQ daily for 3 days followed by 200 mg weekly doses) from mean estimated population pharmacokinetic parameters from Phase I data.

SmithKline Beecham
Pharmaceuticals

Date: **18 August 2000**

To: **Dr. Duolao Wang**

From: **Ann Miller, DMPK, SB**

RE: **Study 252263/030 Data for IDMC**

This document provides the following information for use at the IDMC meeting to be held on Monday, 21 August, 2000:

A. A summary of tafenoquine concentrations in patients randomized to receive tafenoquine. Data include primarily samples collected following third loading dose (6-12 h post-dose) and at 4 weeks (predose) of the double blind phase.

B. Description of tafenoquine drug analysis method with regard to specificity.

Other documents attached separately are as follows:

C. Summary of the data in support of tafenoquine dose selection for Phase III studies.

D. Excel table containing the raw concentration time data for tafenoquine (mefloquine not yet received).

Tafenoquine assay results:

Drug Assay:

The LC/MS/MS assay for tafenoquine (TQ) is based on a method known as selected reaction monitoring (SRM) detection which is probably the most selective and specific mode of operation used in mass spectrometry. Essentially SRM relies on the following series of events:

1) Isolation of a characteristic ion of the analyte by mass selection. This is a molecular weight/mass marker for the analyte.
2) Excitation of the mass selected species in order to promote decomposition. The resulting abundant fragment ion is essentially a structural marker.
3) Mass selection of the chosen characteristic fragment followed by detection.

In this instance TQ is observed as the protonated species in the gas phase at m/z 464 on the MS mass scale (Mol. weight 463). The fragment selected is m/z 379 which represents loss of the pentylamine moiety.

In order to detect a signal by SRM three acceptance criteria have to be fulfilled; (a) correct chromatographic retention time, (b) correct parent selected and (c) correct fragment ion selected. From work during the assay validation in four species and many studies performed thus far, there has been no indication of selectivity issues in the LC/MS/MS assays. The detection of other compounds (such as methaquine, Mol. weight 379) or metabolites of TQ are highly unlikely due to the fact that the difference in molecular weight will preclude selection in the first stage of the experiment. No peaks with different retention times to that of TQ have been observed in the chromatograms during assay.

The consensus in the drug analysis group is that there is a very miniscule chance that what was measured in these samples could be something other than tafenoquine after looking at the structure of TQ, the LC/MS/MS method used, the full mass spec of standard TQ used in the validation of the method, which showed only minor fragments outside of the parent ion, and the product ion full spectrum of standard TQ. Based on the structure of TQ, it does not appear that dimerization is possible (which could have possibly resulted in an inactive compound). Also based on the high concentrations observed in study 030, and the assay used, it is highly unlikely that anything endogenous could have interfered with the assay.

Tafenoquine is administered as a racemate. The LC/MS/MS assay used does not separate the two enantiomers. But, based on the fact that the *in vitro* drug susceptibility test (performed at Walter Reed) indicated both enantiomers have similar levels of antimalarial activity and the pharmacokinetic profiles of

the enantiomers are similar in healthy volunteer studies, a difference in the ratio of enantiomers in the current TQ formulation, should that have possibly occurred, would not be expected to result in any difference in activity in this study.

Tafenoquine concentration data showed the following:

1. All samples analyzed had quantifiable concentrations of tafenoquine (range: 40.6 - 612 ng/mL).

2. Plasma concentrations were >100 ng/mL (target minimum concentration) following loading dose and weekly dosing up to 4 weeks post first loading dose. The loading dose achieved concentrations ranging from 247-612 ng/mL. Following weekly dosing, concentrations at ca. 4 weeks ranged from 103-461 ng/mL. There is limited data beyond 4 weeks, thus, it is difficult to comment on the relevance of the concentrations observed.

3. Plasma concentrations in failed patients as of 1 (samples taken prior to positive smear) were randomly scattered across the same range as those in patients who did not fail (see table below). Within the 2 groups of data, there is no pattern to suggest that the failures are limited to those subjects for whom concentrations are at the low end of the range. Failures appear to have occurred in subjects with concentrations across the full range of concentrations observed.

Conclusion: Based on the data in hand, tafenoquine plasma concentrations were >100 ng/mL following the loading dose plus weekly dose regimen. The concentrations observed in those patients who eventually failed and those who did not fail were similar during the loading dose and at week 4 based on failure rate as of July 27th.

Mean (SD) tafenoquine concentrations at various times relative to first loading dose (LD)

Time of sample	Non-failure	Failure (Prior)*
LD (0-4 days)	421 (105)	383 (86)
Week 4 (25-32 days)	249 (62)	259 (78)

* Samples taken prior to positive smear. Failures as of data received by 27 July.

Mean (SD) tafenoquine concentrations at various times relative to first loading dose by field worker (July 27 failure data)

Field Worker #		Loading Dose		Week 4	
		Non-failure	Failure	Non-failure	Failure
1	n	0	1	3	3
	Mean(SD)	.	297	286 (79)	298 (17)
2	n	1	1	4	1
	Mean(SD)	413	329	258 (47)	352
3	n	1	2	2	2
	Mean(SD)	331	436 (217)	198 (70)	327 (191)
4	n	3	0	4	1
	Mean(SD)	451 (132)	.	238 (26)	204
5	n	3	2	6	2
	Mean(SD)	438 (127)	374 (89)	270 (58)	262 (0.3)
6	n	1	0	2	2
	Mean(SD)	350.6	.	253 (66)	265 (98)
7	n	1	2	3	2
	Mean(SD)	247	361 (42)	196 (19)	230 (10)
8	n	1	1	2	1
	Mean(SD)	551	327	288 (13)	254
9	n	1	0	3	0
	Mean(SD)	437	.	314 (52)	.
10	n	1	1	1	1
	Mean(SD)	298	268	213	201
11	n	0	1	5	3
	Mean(SD)	.	415	184 (53)	275 (76)
12	n	1	1	1	2
	Mean(SD)	364.8	501.1	242.9	238 (48)
13	n	1	0	2	2
	Mean(SD)	380.6	.	291 (15)	320 (156)
14	n	1	1	2	1
	Mean(SD)	412	426	320 (129)	378
15	n	0	2	1	2
	Mean(SD)	.	395 (98)	152	299 (102)
16	n	3	0	5	4
	Mean(SD)	514.3	.	268 (41)	179 (58)
17	n	0	1	6	2
	Mean(SD)	.	392	230 (69)	182 (36)

Mean (SD) tafenoquine concentrations at Week 4 post first loading dose (LD) by number of samples obtained per patient

Patient status	LD & Week 4	Week 4 only
Non-failures	273 (62) (n=17)	237 (60) (n=35)
Failures*	293 (90) (n=11)	242 (73) (n=20)

* Samples taken prior to positive smear. Failures as of data received by 27 July.

Tafenoquine concentrations following loading dose by field worker
(27 July failure data)

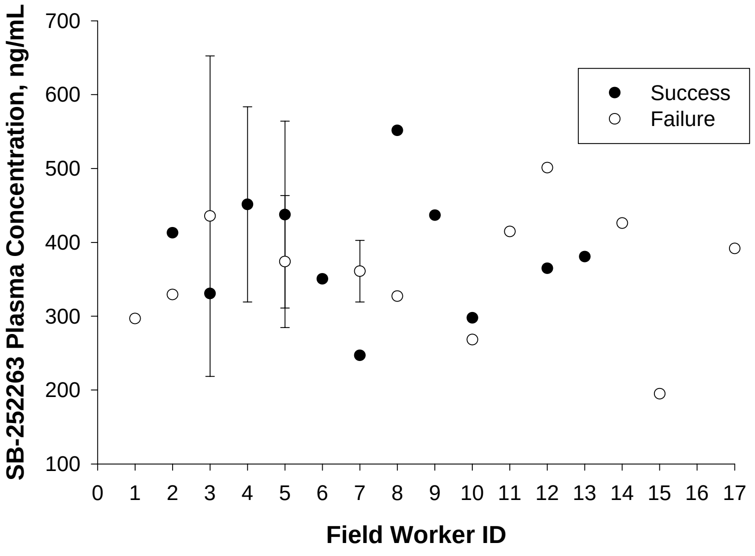

Tafenoquine concentrations at 4 weeks (predose)
(27 July failure data)

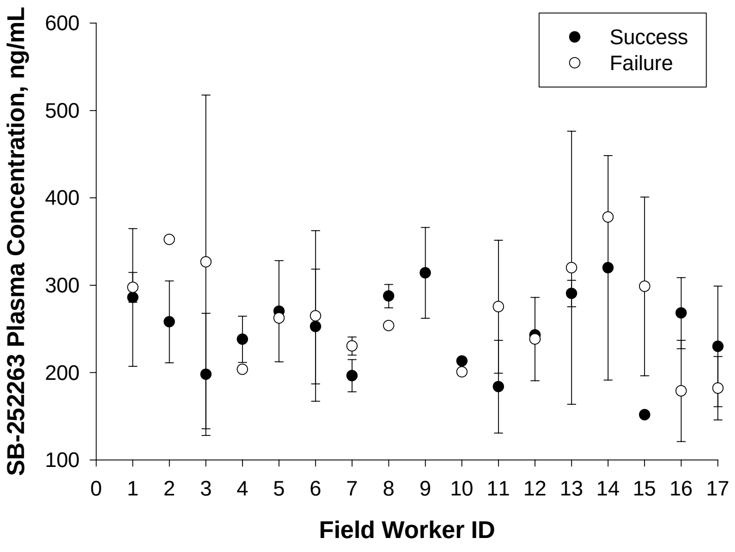

Tafenoquine concentrations relative to first loading dose
(27 July failure data)

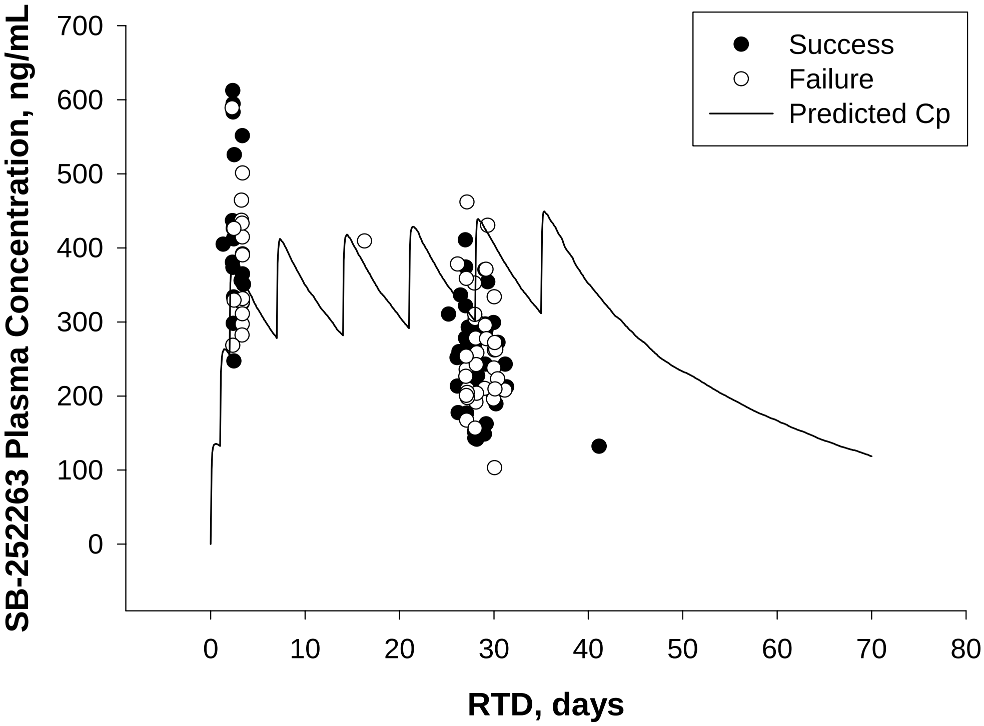

RTD = Relative Time to First Loading Dose

Predicted concentrations based on simulation of clinical dose regimen (200 mg TQ daily for 3 days followed by 200 mg weekly doses) from mean estimated population pharmacokinetic parameters from Phase I data.

SmithKline Beecham
Pharmaceuticals

M E M O R A N D U M

To: Dr Keith F. Barker

From: George Okafo

Date: 19-Jun-2015

cc: Dr Dave Z Bell
Dr Ann K Miller
Mr Neil Mortimer
Dr Philip D Pickford
Dr Christopher Kell
Lorraine H Martin
Dr Fariba Koosha

SUBJECT: **SUMMARY OF LC/MS AND NMR ANALYSIS OF TAFENOQUINE CAPSULES BATCH NUMBER 252263-/030/KEO USED IN STUDY 030 KENYA**

As requested, we have analysed the Tafenoquine capsules used in Study 030 Kenya using NMR (F^{19} and H^{1}) and by LC/MS (ESI+ mode) to investigate the possibility that secondary manufacturing had altered/degraded the drug substance, SB-252263-AX. The results have been summarised below

LC/MS (ESI+ mode)

Two capsules (Batch number 252263-/030/KEO) were extracted according to the procedure reported in PDMH-2164/02 and analysed by HPLC according to AS report SB-252263/RSD-10116S/1. The Tafenoquine peak was analysed using positive ion electrospray ionisation (ESI+) on a Micromass Quattro mass spectrometer. The results show no evidence of any other components that could not be accounted for either as fragments or adducts. Additionally, there is no evidence of other components co-eluting with the Tafenoquine peak.

New Frontiers Science Park, Third Avenue, Harlow, Essex, CM19 5AW
Telephone: 01279 622000 Fax: 01279 644100

SmithKline Beecham p.l.c. Registered in England and Wales, 2337959. Registered Office: New Horizons Court, Brentford, Middlesex TW8 9EP.

NMR (F^{19} and H^1)

Tafenoquine capsules (Batch number 252263-/030/KEO) contents were dissolved in deuterated DMSO and analysed on a Bruker DPX400MHz NMR spectrometer equipped with proton and fluorine –19 nuclei probes. Samples of SB-252263-AX drug substance, batch number TFN-A-02C2 were prepared and analysed similarly for comparison (analytical reference 0A10194). The results showed no evidence of any chemical change in the Tafenoquine drug substance present in the capsule formulation.

George Okafo

Standard Operating Procedures
Certification of Expert Microscopists in the Diagnosis of Malaria

1. Candidates are given a set of 25 slides for examination. Candidate has no time limit and may use bench aids or other materials to assist in reading slides. Candidate must not seek or receive help from any other person. Results are recorded and signed by the candidate.

2. The examination set is prepared by a senior expert microscopist and includes 25 slides with the following species and parasite densities:

8 negative slides
1 *P. falciparum* >50/200 wbc
3 *P. falciparum* <50/200 wbc
2 *P. falciparum* <5/200 wbc
1 *P. vivax* >50/200 wbc
3 *P. vivax* <50/200 wbc
2 *P. vivax* <5/200 wbc
4 *P. malariae* any density
1 *P. ovale* any density

3. The examination is scored in two components:

<u>Malaria Score</u>. Sensitivity and specificity for the diagnosis of "malaria." The outcome is either positive or negative for malaria parasites. Errors in species identification do not count. Specificity and sensitivity are calculated in the normal manner. Passing score is 100% specificity and 90% sensitivity.

<u>Species Score</u>. Reliability of species identification. Negative slides are removed from the analysis. A false negative/positive on the species score is the product of identifying a species incorrectly. Passing score is a minimum sum of specificity and sensitivity > 150% for each species. There is no requirement for *P. ovale* diagnosis.

4. Candidates with passing malaria and species scores are certified as “experts” in the microscopic diagnosis of malaria. The certification is valid for one year for the purposes of this trial.

Obs	WHOAE	PREF
1	0162	AGGRESSIVE REACTION
2	0163	AGITATION
3	0164	AMNESIA
4	0166	ANXIETY
5	0167	APATHY
6	1127	CONCENTRATION IMPAIRED
7	0092	CONFUSION
8	0099	DELIRIUM
9	0170	DELUSION
10	0171	DEPERSONALIZATION
11	0172	DEPRESSION
12	0967	DEPRESSION AGGRAVATED
13	0173	DEPRESSION PSYCHOTIC
14	1243	DREAMING ABNORMAL
15	0177	EMOTIONAL LABILITY
16	0178	EUPHORIA
17	0179	HALLUCINATION
18	0180	HYSTERIA
19	0181	ILLUSION
20	0183	INSOMNIA
21	0186	MANIC REACTION
22	0188	NERVOUSNESS
23	0189	NEUROSIS
24	0190	PARANOID REACTION
25	0191	PARONIRIA
26	0192	PERSONALITY DISORDER
27	0193	PSYCHOSIS
28	0194	SCHIZOPHRENIC REACTION
29	0195	SLEEP DISORDER
30	0196	SOMNAMBULISM
31	0197	SOMNOLENCE
32	0198	SUICIDE ATTEMPT
33	0199	THINKING ABNORMAL
34	0088	ATAXIA
35	0093	CONVULSIONS
36	0094	CONVULSIONS AGGRAVATED
37	0095	CONVULSIONS GRAND MAL
38	0096	CONVULSIONS LOCAL
39	0097	COORDINATION ABNORMAL
40	0101	DIZZINESS
41	0102	DYSKINESIA

42	0109	HEADACHE
43	0114	HYPERKINESIA
44	0151	STUPOR
45	0154	TREMOR
46	0158	VERTIGO

SB252263 - 030

Listing B.01

Treatment Assignment

Subject Number	Popn*	Screening Date	Randomisation Number	Treatment Group
030.001.00001	IPP	12APR2000	1001	Tafenoquine
030.001.00002	IPP	10APR2000	1002	Placebo
030.001.00003	I	10APR2000	1003	Mefloquine
030.001.00004	IPP	13APR2000	1004	Tafenoquine
030.001.00005	IPP	10APR2000	1005	Placebo
030.001.00006	IPP	10APR2000	1006	Tafenoquine
030.001.00007	IPP	11APR2000	1007	Mefloquine
030.001.00008	IPP	11APR2000	1008	Mefloquine
030.001.00009	IPP	12APR2000	1009	Tafenoquine
030.001.00010	IPP	11APR2000	1010	Mefloquine
030.001.00011	IPP	10APR2000	1011	Placebo
030.001.00012	IPP	10APR2000	1012	Placebo
030.001.00013	IPP	13APR2000	1013	Mefloquine
030.001.00014	IPP	10APR2000	1014	Tafenoquine
030.001.00015	I	10APR2000	1015	Placebo
030.001.00016	IPP	10APR2000	1016	Tafenoquine
030.001.00017	IPP	17APR2000	1017	Tafenoquine
030.001.00018	IPP	11APR2000	1018	Mefloquine
030.001.00019	IPP	10APR2000	1019	Placebo
030.001.00020	IPP	10APR2000	1020	Placebo
030.001.00021	IPP	10APR2000	1021	Mefloquine
030.001.00022	IPP	13APR2000	1022	Placebo
030.001.00023	I	10APR2000	1023	Mefloquine
030.001.00024	IPP	11APR2000	1024	Tafenoquine
030.001.00025	IPP	11APR2000	1025	Tafenoquine
030.001.00026	IPP	10APR2000	1026	Placebo
030.001.00027	IPP	12APR2000	1027	Placebo
030.001.00028	IPP	11APR2000	1028	Tafenoquine
030.001.00029	IPP	11APR2000	1029	Tafenoquine
030.001.00030	IPP	14APR2000	1030	Tafenoquine
030.001.00031	IPP	12APR2000	1031	Mefloquine
030.001.00032	IPP	13APR2000	1032	Placebo
030.001.00033	IPP	11APR2000	1033	Placebo
030.001.00034	IPP	17APR2000	1034	Mefloquine
030.001.00035		12APR2000	1035	Mefloquine
030.001.00036	I	13APR2000	1036	Mefloquine
030.001.00037	IPP	11APR2000	1037	Placebo
030.001.00038	IPP	18APR2000	1038	Mefloquine
030.001.00039	IPP	14APR2000	1039	Placebo
030.001.00040	IPP	13APR2000	1040	Tafenoquine
030.001.00041	IPP	10APR2000	1041	Placebo
030.001.00042	IPP	10APR2000	1042	Tafenoquine
030.001.00043	IPP	18APR2000	1043	Tafenoquine
030.001.00044	IPP	10APR2000	1044	Placebo
030.001.00045	IPP	14APR2000	1045	Mefloquine
030.001.00046	IPP	10APR2000	1046	Mefloquine
030.001.00047	IPP	12APR2000	1047	Mefloquine

*Popn=Population (I=Intent-To-Treat, PP=Per Protocol)
Tafenoquine = Tafenoquine 200mg, Mefloquine = Mefloquine 250mg

SB252263 - 030

Listing B.01

Treatment Assignment

Subject Number	Popn*	Screening Date	Randomisation Number	Treatment Group
030.001.00048	IPP	13APR2000	1048	Tafenoquine
030.001.00049	IPP	13APR2000	1049	Mefloquine
030.001.00050	IPP	10APR2000	1050	Tafenoquine
030.001.00051	IPP	11APR2000	1051	Tafenoquine
030.001.00052	IPP	10APR2000	1052	Mefloquine
030.001.00053	IPP	11APR2000	1053	Mefloquine
030.001.00054	IPP	10APR2000	1054	Mefloquine
030.001.00055	IPP	12APR2000	1055	Placebo
030.001.00056	I	11APR2000	1056	Placebo
030.001.00057	IPP	14APR2000	1057	Placebo
030.001.00058	IPP	17APR2000	1058	Tafenoquine
030.001.00059	IPP	13APR2000	1059	Placebo
030.001.00060	IPP	13APR2000	1060	Tafenoquine
030.001.00061	IPP	14APR2000	1061	Tafenoquine
030.001.00062	IPP	12APR2000	1062	Mefloquine
030.001.00063	IPP	11APR2000	1063	Placebo
030.001.00064	IPP	11APR2000	1064	Placebo
030.001.00065	IPP	11APR2000	1065	Tafenoquine
030.001.00066	IPP	14APR2000	1066	Mefloquine
030.001.00067	IPP	12APR2000	1067	Placebo
030.001.00068	IPP	14APR2000	1068	Tafenoquine
030.001.00069	I	10APR2000	1069	Mefloquine
030.001.00070	IPP	11APR2000	1070	Tafenoquine
030.001.00071	IPP	10APR2000	1071	Mefloquine
030.001.00072	IPP	12APR2000	1072	Placebo
030.001.00073	IPP	13APR2000	1073	Mefloquine
030.001.00074	IPP	10APR2000	1074	Mefloquine
030.001.00075	IPP	10APR2000	1075	Mefloquine
030.001.00076	IPP	11APR2000	1076	Placebo
030.001.00077	IPP	11APR2000	1077	Tafenoquine
030.001.00078	IPP	12APR2000	1078	Placebo
030.001.00079	IPP	14APR2000	1079	Tafenoquine
030.001.00080	IPP	12APR2000	1080	Placebo
030.001.00081	IPP	11APR2000	1081	Placebo
030.001.00082		18APR2000	1082	Tafenoquine
030.001.00083	IPP	13APR2000	1083	Mefloquine
030.001.00084	IPP	13APR2000	1084	Tafenoquine
030.001.00085	IPP	10APR2000	1085	Tafenoquine
030.001.00086	IPP	11APR2000	1086	Tafenoquine
030.001.00087	I	14APR2000	1087	Placebo
030.001.00088	IPP	14APR2000	1088	Placebo
030.001.00089	IPP	17APR2000	1089	Mefloquine
030.001.00090	IPP	12APR2000	1090	Placebo
030.001.00091	IPP	13APR2000	1091	Mefloquine
030.001.00092	IPP	12APR2000	1092	Placebo
030.001.00093	IPP	10APR2000	1093	Tafenoquine
030.001.00094	I	11APR2000	1094	Tafenoquine

*Popn=Population (I=Intent-To-Treat, PP=Per Protocol)
Tafenoquine = Tafenoquine 200mg, Mefloquine = Mefloquine 250mg

SB252263 - 030

Listing B.01

Treatment Assignment

Subject Number	Popn*	Screening Date	Randomisation Number	Treatment Group
030.001.00095	IPP	14APR2000	1095	Mefloquine
030.001.00096	IPP	12APR2000	1096	Mefloquine
030.001.00097	IPP	11APR2000	1097	Mefloquine
030.001.00098	IPP	12APR2000	1098	Mefloquine
030.001.00099	IPP	10APR2000	1099	Mefloquine
030.001.00100	IPP	12APR2000	1100	Tafenoquine
030.001.00101	IPP	14APR2000	1101	Placebo
030.001.00102	IPP	13APR2000	1102	Tafenoquine
030.001.00103	IPP	11APR2000	1103	Placebo
030.001.00104	IPP	14APR2000	1104	Tafenoquine
030.001.00105	IPP	11APR2000	1105	Mefloquine
030.001.00106	IPP	10APR2000	1106	Placebo
030.001.00107	IPP	12APR2000	1107	Tafenoquine
030.001.00108	IPP	14APR2000	1108	Placebo
030.001.00109	IPP	11APR2000	1109	Tafenoquine
030.001.00110	IPP	10APR2000	1110	Mefloquine
030.001.00111	IPP	11APR2000	1111	Tafenoquine
030.001.00112	IPP	13APR2000	1112	Tafenoquine
030.001.00113	IPP	17APR2000	1113	Mefloquine
030.001.00114	IPP	14APR2000	1114	Placebo
030.001.00115	IPP	17APR2000	1115	Placebo
030.001.00116	IPP	12APR2000	1116	Placebo
030.001.00117	IPP	11APR2000	1117	Mefloquine
030.001.00118	IPP	13APR2000	1118	Tafenoquine
030.001.00119	IPP	11APR2000	1119	Placebo
030.001.00120	IPP	10APR2000	1120	Mefloquine
030.001.00121	IPP	10APR2000	1121	Mefloquine
030.001.00122	IPP	12APR2000	1122	Tafenoquine
030.001.00123	I	10APR2000	1123	Mefloquine
030.001.00124	IPP	11APR2000	1124	Placebo
030.001.00125	IPP	10APR2000	1125	Placebo
030.001.00126	IPP	11APR2000	1126	Tafenoquine
030.001.00127	I	12APR2000	1127	Placebo
030.001.00128	IPP	10APR2000	1128	Tafenoquine
030.001.00129	IPP	12APR2000	1129	Tafenoquine
030.001.00130	IPP	10APR2000	1130	Mefloquine
030.001.00131	IPP	10APR2000	1131	Placebo
030.001.00132	IPP	13APR2000	1132	Mefloquine
030.001.00133	IPP	10APR2000	1133	Tafenoquine
030.001.00134	IPP	11APR2000	1134	Mefloquine
030.001.00135	IPP	11APR2000	1135	Tafenoquine
030.001.00136	IPP	11APR2000	1136	Placebo
030.001.00137	IPP	11APR2000	1137	Placebo
030.001.00138	IPP	12APR2000	1138	Tafenoquine
030.001.00139	IPP	10APR2000	1139	Tafenoquine
030.001.00140	IPP	10APR2000	1140	Placebo
030.001.00141	IPP	11APR2000	1141	Placebo

*Popn=Population (I=Intent-To-Treat, PP=Per Protocol)
Tafenoquine = Tafenoquine 200mg, Mefloquine = Mefloquine 250mg

SB252263 - 030

Listing B.01

Treatment Assignment

Subject Number	Popn*	Screening Date	Randomisation Number	Treatment Group
030.001.00142	IPP	14APR2000	1142	Mefloquine
030.001.00143	IPP	14APR2000	1143	Mefloquine
030.001.00144	IPP	11APR2000	1144	Mefloquine
030.001.00145	IPP	18APR2000	1145	Placebo
030.001.00146	I	13APR2000	1146	Mefloquine
030.001.00147	IPP	10APR2000	1147	Mefloquine
030.001.00148	IPP	10APR2000	1148	Tafenoquine
030.001.00149	IPP	13APR2000	1149	Tafenoquine
030.001.00150	IPP	12APR2000	1150	Tafenoquine
030.001.00151	IPP	11APR2000	1151	Placebo
030.001.00152	IPP	13APR2000	1152	Placebo
030.001.00153	IPP	12APR2000	1153	Mefloquine
030.001.00154	IPP	13APR2000	1154	Placebo
030.001.00155	IPP	11APR2000	1155	Tafenoquine
030.001.00156	IPP	14APR2000	1156	Mefloquine
030.001.00157	IPP	12APR2000	1157	Tafenoquine
030.001.00158	IPP	11APR2000	1158	Tafenoquine
030.001.00159	IPP	12APR2000	1159	Tafenoquine
030.001.00160	I	11APR2000	1160	Placebo
030.001.00161	IPP	10APR2000	1161	Tafenoquine
030.001.00162	IPP	11APR2000	1162	Placebo
030.001.00163	IPP	11APR2000	1163	Mefloquine
030.001.00164	IPP	14APR2000	1164	Placebo
030.001.00165	IPP	11APR2000	1165	Mefloquine
030.001.00166	IPP	10APR2000	1166	Placebo
030.001.00167	IPP	13APR2000	1167	Mefloquine
030.001.00168	IPP	14APR2000	1168	Mefloquine
030.001.00169	IPP	10APR2000	1169	Placebo
030.001.00170	IPP	12APR2000	1170	Mefloquine
030.001.00171	IPP	12APR2000	1171	Tafenoquine
030.001.00172	IPP	10APR2000	1172	Placebo
030.001.00173	I	14APR2000	1173	Tafenoquine
030.001.00174	IPP	11APR2000	1174	Placebo
030.001.00175	IPP	17APR2000	1175	Placebo
030.001.00176	IPP	13APR2000	1176	Tafenoquine
030.001.00177	IPP	13APR2000	1177	Mefloquine
030.001.00178	I	12APR2000	1178	Mefloquine
030.001.00179	I	12APR2000	1179	Tafenoquine
030.001.00180	IPP	17APR2000	1180	Mefloquine
030.001.00181	IPP	10APR2000	1181	Mefloquine
030.001.00182	I	10APR2000	1182	Placebo
030.001.00183	IPP	11APR2000	1183	Placebo
030.001.00184	IPP	17APR2000	1184	Tafenoquine
030.001.00185	IPP	18APR2000	1185	Mefloquine
030.001.00186	IPP	10APR2000	1186	Tafenoquine
030.001.00187	I	10APR2000	1187	Tafenoquine
030.001.00188	IPP	13APR2000	1188	Mefloquine

*Popn=Population (I=Intent-To-Treat, PP=Per Protocol)
Tafenoquine = Tafenoquine 200mg, Mefloquine = Mefloquine 250mg

SB252263 - 030

Listing B.01

Treatment Assignment

Subject Number	Popn*	Screening Date	Randomisation Number	Treatment Group
030.001.00189	I	10APR2000	1189	Mefloquine
030.001.00190	IPP	11APR2000	1190	Tafenoquine
030.001.00191	IPP	18APR2000	1191	Placebo
030.001.00192	IPP	11APR2000	1192	Placebo
030.001.00193	IPP	14APR2000	1193	Placebo
030.001.00194	IPP	14APR2000	1194	Tafenoquine
030.001.00195	IPP	18APR2000	1195	Tafenoquine
030.001.00196	IPP	11APR2000	1196	Mefloquine
030.001.00197	IPP	17APR2000	1197	Placebo
030.001.00198	IPP	12APR2000	1198	Mefloquine
030.001.00199	IPP	10APR2000	1199	Mefloquine
030.001.00200	IPP	17APR2000	1200	Mefloquine
030.001.00201	IPP	10APR2000	1201	Tafenoquine
030.001.00202		17APR2000	1202	Placebo
030.001.00203	IPP	13APR2000	1203	Tafenoquine
030.001.00204	I	14APR2000	1204	Placebo
030.001.00205	IPP	10APR2000	1205	Tafenoquine
030.001.00206	I	10APR2000	1206	Mefloquine
030.001.00207	IPP	13APR2000	1207	Tafenoquine
030.001.00208	IPP	11APR2000	1208	Placebo
030.001.00209	IPP	13APR2000	1209	Tafenoquine
030.001.00210	IPP	12APR2000	1210	Tafenoquine
030.001.00211	IPP	11APR2000	1211	Placebo
030.001.00212	IPP	13APR2000	1212	Placebo
030.001.00213	IPP	14APR2000	1213	Mefloquine
030.001.00214	IPP	13APR2000	1214	Mefloquine
030.001.00215	IPP	12APR2000	1215	Placebo
030.001.00216	IPP	11APR2000	1216	Mefloquine
030.001.00217	IPP	11APR2000	1217	Tafenoquine
030.001.00218	IPP	13APR2000	1218	Tafenoquine
030.001.00219	IPP	12APR2000	1219	Placebo
030.001.00220	IPP	13APR2000	1220	Tafenoquine
030.001.00221	IPP	17APR2000	1221	Mefloquine
030.001.00222	IPP	14APR2000	1222	Placebo
030.001.00223	IPP	14APR2000	1223	Mefloquine
030.001.00224	IPP	12APR2000	1224	Mefloquine
030.001.00225	IPP	10APR2000	1225	Placebo
030.001.00226	IPP	13APR2000	1226	Placebo
030.001.00227	IPP	14APR2000	1227	Mefloquine
030.001.00228	IPP	13APR2000	1228	Tafenoquine
030.001.00229	IPP	13APR2000	1229	Placebo
030.001.00230	IPP	13APR2000	1230	Tafenoquine
030.001.00231	IPP	12APR2000	1231	Tafenoquine
030.001.00232	IPP	18APR2000	1232	Mefloquine
030.001.00233	IPP	10APR2000	1233	Tafenoquine
030.001.00234	IPP	11APR2000	1234	Placebo
030.001.00235	IPP	10APR2000	1235	Mefloquine

*Popn=Population (I=Intent-To-Treat, PP=Per Protocol)
Tafenoquine = Tafenoquine 200mg, Mefloquine = Mefloquine 250mg

SB252263 - 030

Listing B.01

Treatment Assignment

Subject Number	Popn*	Screening Date	Randomisation Number	Treatment Group
030.001.00236	IPP	11APR2000	1236	Placebo
030.001.00237	IPP	17APR2000	1237	Mefloquine
030.001.00238	IPP	12APR2000	1238	Placebo
030.001.00239	IPP	10APR2000	1239	Mefloquine
030.001.00240	IPP	13APR2000	1240	Tafenoquine
030.001.00241	IPP	13APR2000	1241	Mefloquine
030.001.00242	IPP	14APR2000	1242	Placebo
030.001.00243	IPP	13APR2000	1243	Tafenoquine
030.001.00244	IPP	13APR2000	1244	Mefloquine
030.001.00245	IPP	11APR2000	1245	Mefloquine
030.001.00246	IPP	11APR2000	1246	Tafenoquine
030.001.00247	IPP	11APR2000	1247	Placebo
030.001.00248	IPP	13APR2000	1248	Placebo
030.001.00249	IPP	12APR2000	1249	Tafenoquine
030.001.00250		18APR2000	1250	Mefloquine
030.001.00251	IPP	12APR2000	1251	Placebo
030.001.00252	IPP	12APR2000	1252	Tafenoquine
030.001.00253	IPP	13APR2000	1253	Placebo
030.001.00254	I	12APR2000	1254	Tafenoquine
030.001.00255	IPP	12APR2000	1255	Mefloquine
030.001.00256	IPP	14APR2000	1256	Placebo
030.001.00257	IPP	14APR2000	1257	Placebo
030.001.00258	IPP	14APR2000	1258	Mefloquine
030.001.00259	IPP	12APR2000	1259	Tafenoquine
030.001.00260	IPP	17APR2000	1260	Placebo
030.001.00261		13APR2000	1261	Tafenoquine
030.001.00262	IPP	13APR2000	1262	Mefloquine
030.001.00263	IPP	17APR2000	1263	Mefloquine
030.001.00264	IPP	18APR2000	1264	Tafenoquine
030.001.00265	IPP	12APR2000	1265	Mefloquine
030.001.00266	I	12APR2000	1266	Tafenoquine
030.001.00267	IPP	11APR2000	1267	Tafenoquine
030.001.00268	IPP	14APR2000	1268	Placebo
030.001.00269	IPP	13APR2000	1269	Placebo
030.001.00270	IPP	13APR2000	1270	Placebo
030.001.00271	I	13APR2000	1271	Tafenoquine
030.001.00272	IPP	10APR2000	1272	Tafenoquine
030.001.00273	IPP	11APR2000	1273	Mefloquine
030.001.00274	IPP	11APR2000	1274	Mefloquine
030.001.00275	IPP	12APR2000	1275	Placebo
030.001.00276	IPP	12APR2000	1276	Mefloquine
030.001.00277	IPP	12APR2000	1277	Placebo
030.001.00278	IPP	12APR2000	1278	Placebo
030.001.00279	IPP	12APR2000	1279	Tafenoquine
030.001.00280	IPP	13APR2000	1280	Mefloquine
030.001.00281	IPP	12APR2000	1281	Mefloquine
030.001.00282	IPP	11APR2000	1282	Tafenoquine

*Popn=Population (I=Intent-To-Treat, PP=Per Protocol)
Tafenoquine = Tafenoquine 200mg, Mefloquine = Mefloquine 250mg

SB252263 - 030

Listing B.01

Treatment Assignment

Subject Number	Popn*	Screening Date	Randomisation Number	Treatment Group
030.001.00283	IPP	14APR2000	1283	Mefloquine
030.001.00284	IPP	10APR2000	1284	Tafenoquine
030.001.00285	IPP	18APR2000	1285	Placebo
030.001.00286	IPP	12APR2000	1286	Mefloquine
030.001.00287	IPP	10APR2000	1287	Placebo
030.001.00288	IPP	12APR2000	1288	Tafenoquine
030.001.00289	IPP	14APR2000	1289	Mefloquine
030.001.00290	IPP	12APR2000	1290	Mefloquine
030.001.00291	IPP	17APR2000	1291	Placebo
030.001.00292	IPP	12APR2000	1292	Mefloquine
030.001.00293	IPP	11APR2000	1293	Tafenoquine
030.001.00294	IPP	13APR2000	1294	Tafenoquine
030.001.00295		17APR2000	1295	Placebo
030.001.00296	IPP	14APR2000	1296	Tafenoquine
030.001.00297	IPP	18APR2000	1297	Placebo
030.001.00298	IPP	17APR2000	1298	Tafenoquine
030.001.00299	IPP	17APR2000	1299	Mefloquine
030.001.00300	IPP	11APR2000	1300	Placebo
030.001.00301	IPP	14APR2000	1301	Tafenoquine
030.001.00302	I	14APR2000	1302	Tafenoquine
030.001.00303	IPP	13APR2000	1303	Placebo
030.001.00304	IPP	13APR2000	1304	Tafenoquine
030.001.00305	IPP	13APR2000	1305	Mefloquine
030.001.00306	IPP	13APR2000	1306	Tafenoquine

*Popn=Population (I=Intent-To-Treat, PP=Per Protocol)
Tafenoquine = Tafenoquine 200mg, Mefloquine = Mefloquine 250mg

SB252263 - 030

Listing B.01

Treatment Assignment

Subject Number	Popn*	Screening Date	Randomisation Number	Treatment Group
030.001.00001	IPP	12APR2000	1001	Tafenoquine
030.001.00002	IPP	10APR2000	1002	Placebo
030.001.00003	I	10APR2000	1003	Mefloquine
030.001.00004	IPP	13APR2000	1004	Tafenoquine
030.001.00005	IPP	10APR2000	1005	Placebo
030.001.00006	IPP	10APR2000	1006	Tafenoquine
030.001.00007	IPP	11APR2000	1007	Mefloquine
030.001.00008	IPP	11APR2000	1008	Mefloquine
030.001.00009	IPP	12APR2000	1009	Tafenoquine
030.001.00010	IPP	11APR2000	1010	Mefloquine
030.001.00011	IPP	10APR2000	1011	Placebo
030.001.00012	IPP	10APR2000	1012	Placebo
030.001.00013	IPP	13APR2000	1013	Mefloquine
030.001.00014	IPP	10APR2000	1014	Tafenoquine
030.001.00015	I	10APR2000	1015	Placebo
030.001.00016	IPP	10APR2000	1016	Tafenoquine
030.001.00017	IPP	17APR2000	1017	Tafenoquine
030.001.00018	IPP	11APR2000	1018	Mefloquine
030.001.00019	IPP	10APR2000	1019	Placebo
030.001.00020	IPP	10APR2000	1020	Placebo
030.001.00021	IPP	10APR2000	1021	Mefloquine
030.001.00022	IPP	13APR2000	1022	Placebo
030.001.00023	I	10APR2000	1023	Mefloquine
030.001.00024	IPP	11APR2000	1024	Tafenoquine
030.001.00025	IPP	11APR2000	1025	Tafenoquine
030.001.00026	IPP	10APR2000	1026	Placebo
030.001.00027	IPP	12APR2000	1027	Placebo
030.001.00028	IPP	11APR2000	1028	Tafenoquine
030.001.00029	IPP	11APR2000	1029	Tafenoquine
030.001.00030	IPP	14APR2000	1030	Tafenoquine
030.001.00031	IPP	12APR2000	1031	Mefloquine
030.001.00032	IPP	13APR2000	1032	Placebo
030.001.00033	IPP	11APR2000	1033	Placebo
030.001.00034	IPP	17APR2000	1034	Mefloquine
030.001.00035		12APR2000	1035	Mefloquine
030.001.00036	I	13APR2000	1036	Mefloquine
030.001.00037	IPP	11APR2000	1037	Placebo
030.001.00038	IPP	18APR2000	1038	Mefloquine
030.001.00039	IPP	14APR2000	1039	Placebo
030.001.00040	IPP	13APR2000	1040	Tafenoquine
030.001.00041	IPP	10APR2000	1041	Placebo
030.001.00042	IPP	10APR2000	1042	Tafenoquine
030.001.00043	IPP	18APR2000	1043	Tafenoquine
030.001.00044	IPP	10APR2000	1044	Placebo
030.001.00045	IPP	14APR2000	1045	Mefloquine
030.001.00046	IPP	10APR2000	1046	Mefloquine
030.001.00047	IPP	12APR2000	1047	Mefloquine

*Popn=Population (I=Intent-To-Treat, PP=Per Protocol)
Tafenoquine = Tafenoquine 200mg, Mefloquine = Mefloquine 250mg

SB252263 - 030

Listing B.01

Treatment Assignment

Subject Number	Popn*	Screening Date	Randomisation Number	Treatment Group
030.001.00048	IPP	13APR2000	1048	Tafenoquine
030.001.00049	IPP	13APR2000	1049	Mefloquine
030.001.00050	IPP	10APR2000	1050	Tafenoquine
030.001.00051	IPP	11APR2000	1051	Tafenoquine
030.001.00052	IPP	10APR2000	1052	Mefloquine
030.001.00053	IPP	11APR2000	1053	Mefloquine
030.001.00054	IPP	10APR2000	1054	Mefloquine
030.001.00055	IPP	12APR2000	1055	Placebo
030.001.00056	I	11APR2000	1056	Placebo
030.001.00057	IPP	14APR2000	1057	Placebo
030.001.00058	IPP	17APR2000	1058	Tafenoquine
030.001.00059	IPP	13APR2000	1059	Placebo
030.001.00060	IPP	13APR2000	1060	Tafenoquine
030.001.00061	IPP	14APR2000	1061	Tafenoquine
030.001.00062	IPP	12APR2000	1062	Mefloquine
030.001.00063	IPP	11APR2000	1063	Placebo
030.001.00064	IPP	11APR2000	1064	Placebo
030.001.00065	IPP	11APR2000	1065	Tafenoquine
030.001.00066	IPP	14APR2000	1066	Mefloquine
030.001.00067	IPP	12APR2000	1067	Placebo
030.001.00068	IPP	14APR2000	1068	Tafenoquine
030.001.00069	I	10APR2000	1069	Mefloquine
030.001.00070	IPP	11APR2000	1070	Tafenoquine
030.001.00071	IPP	10APR2000	1071	Mefloquine
030.001.00072	IPP	12APR2000	1072	Placebo
030.001.00073	IPP	13APR2000	1073	Mefloquine
030.001.00074	IPP	10APR2000	1074	Mefloquine
030.001.00075	IPP	10APR2000	1075	Mefloquine
030.001.00076	IPP	11APR2000	1076	Placebo
030.001.00077	IPP	11APR2000	1077	Tafenoquine
030.001.00078	IPP	12APR2000	1078	Placebo
030.001.00079	IPP	14APR2000	1079	Tafenoquine
030.001.00080	IPP	12APR2000	1080	Placebo
030.001.00081	IPP	11APR2000	1081	Placebo
030.001.00082		18APR2000	1082	Tafenoquine
030.001.00083	IPP	13APR2000	1083	Mefloquine
030.001.00084	IPP	13APR2000	1084	Tafenoquine
030.001.00085	IPP	10APR2000	1085	Tafenoquine
030.001.00086	IPP	11APR2000	1086	Tafenoquine
030.001.00087	I	14APR2000	1087	Placebo
030.001.00088	IPP	14APR2000	1088	Placebo
030.001.00089	IPP	17APR2000	1089	Mefloquine
030.001.00090	IPP	12APR2000	1090	Placebo
030.001.00091	IPP	13APR2000	1091	Mefloquine
030.001.00092	IPP	12APR2000	1092	Placebo
030.001.00093	IPP	10APR2000	1093	Tafenoquine
030.001.00094	I	11APR2000	1094	Tafenoquine

*Popn=Population (I=Intent-To-Treat, PP=Per Protocol)
Tafenoquine = Tafenoquine 200mg, Mefloquine = Mefloquine 250mg

SB252263 - 030

Listing B.01

Treatment Assignment

Subject Number	Popn*	Screening Date	Randomisation Number	Treatment Group
030.001.00095	IPP	14APR2000	1095	Mefloquine
030.001.00096	IPP	12APR2000	1096	Mefloquine
030.001.00097	IPP	11APR2000	1097	Mefloquine
030.001.00098	IPP	12APR2000	1098	Mefloquine
030.001.00099	IPP	10APR2000	1099	Mefloquine
030.001.00100	IPP	12APR2000	1100	Tafenoquine
030.001.00101	IPP	14APR2000	1101	Placebo
030.001.00102	IPP	13APR2000	1102	Tafenoquine
030.001.00103	IPP	11APR2000	1103	Placebo
030.001.00104	IPP	14APR2000	1104	Tafenoquine
030.001.00105	IPP	11APR2000	1105	Mefloquine
030.001.00106	IPP	10APR2000	1106	Placebo
030.001.00107	IPP	12APR2000	1107	Tafenoquine
030.001.00108	IPP	14APR2000	1108	Placebo
030.001.00109	IPP	11APR2000	1109	Tafenoquine
030.001.00110	IPP	10APR2000	1110	Mefloquine
030.001.00111	IPP	11APR2000	1111	Tafenoquine
030.001.00112	IPP	13APR2000	1112	Tafenoquine
030.001.00113	IPP	17APR2000	1113	Mefloquine
030.001.00114	IPP	14APR2000	1114	Placebo
030.001.00115	IPP	17APR2000	1115	Placebo
030.001.00116	IPP	12APR2000	1116	Placebo
030.001.00117	IPP	11APR2000	1117	Mefloquine
030.001.00118	IPP	13APR2000	1118	Tafenoquine
030.001.00119	IPP	11APR2000	1119	Placebo
030.001.00120	IPP	10APR2000	1120	Mefloquine
030.001.00121	IPP	10APR2000	1121	Mefloquine
030.001.00122	IPP	12APR2000	1122	Tafenoquine
030.001.00123	I	10APR2000	1123	Mefloquine
030.001.00124	IPP	11APR2000	1124	Placebo
030.001.00125	IPP	10APR2000	1125	Placebo
030.001.00126	IPP	11APR2000	1126	Tafenoquine
030.001.00127	I	12APR2000	1127	Placebo
030.001.00128	IPP	10APR2000	1128	Tafenoquine
030.001.00129	IPP	12APR2000	1129	Tafenoquine
030.001.00130	IPP	10APR2000	1130	Mefloquine
030.001.00131	IPP	10APR2000	1131	Placebo
030.001.00132	IPP	13APR2000	1132	Mefloquine
030.001.00133	IPP	10APR2000	1133	Tafenoquine
030.001.00134	IPP	11APR2000	1134	Mefloquine
030.001.00135	IPP	11APR2000	1135	Tafenoquine
030.001.00136	IPP	11APR2000	1136	Placebo
030.001.00137	IPP	11APR2000	1137	Placebo
030.001.00138	IPP	12APR2000	1138	Tafenoquine
030.001.00139	IPP	10APR2000	1139	Tafenoquine
030.001.00140	IPP	10APR2000	1140	Placebo
030.001.00141	IPP	11APR2000	1141	Placebo

*Popn=Population (I=Intent-To-Treat, PP=Per Protocol)
Tafenoquine = Tafenoquine 200mg, Mefloquine = Mefloquine 250mg

SB252263 - 030

Listing B.01

Treatment Assignment

Subject Number	Popn*	Screening Date	Randomisation Number	Treatment Group
030.001.00142	IPP	14APR2000	1142	Mefloquine
030.001.00143	IPP	14APR2000	1143	Mefloquine
030.001.00144	IPP	11APR2000	1144	Mefloquine
030.001.00145	IPP	18APR2000	1145	Placebo
030.001.00146	I	13APR2000	1146	Mefloquine
030.001.00147	IPP	10APR2000	1147	Mefloquine
030.001.00148	IPP	10APR2000	1148	Tafenoquine
030.001.00149	IPP	13APR2000	1149	Tafenoquine
030.001.00150	IPP	12APR2000	1150	Tafenoquine
030.001.00151	IPP	11APR2000	1151	Placebo
030.001.00152	IPP	13APR2000	1152	Placebo
030.001.00153	IPP	12APR2000	1153	Mefloquine
030.001.00154	IPP	13APR2000	1154	Placebo
030.001.00155	IPP	11APR2000	1155	Tafenoquine
030.001.00156	IPP	14APR2000	1156	Mefloquine
030.001.00157	IPP	12APR2000	1157	Tafenoquine
030.001.00158	IPP	11APR2000	1158	Tafenoquine
030.001.00159	IPP	12APR2000	1159	Tafenoquine
030.001.00160	I	11APR2000	1160	Placebo
030.001.00161	IPP	10APR2000	1161	Tafenoquine
030.001.00162	IPP	11APR2000	1162	Placebo
030.001.00163	IPP	11APR2000	1163	Mefloquine
030.001.00164	IPP	14APR2000	1164	Placebo
030.001.00165	IPP	11APR2000	1165	Mefloquine
030.001.00166	IPP	10APR2000	1166	Placebo
030.001.00167	IPP	13APR2000	1167	Mefloquine
030.001.00168	IPP	14APR2000	1168	Mefloquine
030.001.00169	IPP	10APR2000	1169	Placebo
030.001.00170	IPP	12APR2000	1170	Mefloquine
030.001.00171	IPP	12APR2000	1171	Tafenoquine
030.001.00172	IPP	10APR2000	1172	Placebo
030.001.00173	I	14APR2000	1173	Tafenoquine
030.001.00174	IPP	11APR2000	1174	Placebo
030.001.00175	IPP	17APR2000	1175	Placebo
030.001.00176	IPP	13APR2000	1176	Tafenoquine
030.001.00177	IPP	13APR2000	1177	Mefloquine
030.001.00178	I	12APR2000	1178	Mefloquine
030.001.00179	I	12APR2000	1179	Tafenoquine
030.001.00180	IPP	17APR2000	1180	Mefloquine
030.001.00181	IPP	10APR2000	1181	Mefloquine
030.001.00182	I	10APR2000	1182	Placebo
030.001.00183	IPP	11APR2000	1183	Placebo
030.001.00184	IPP	17APR2000	1184	Tafenoquine
030.001.00185	IPP	18APR2000	1185	Mefloquine
030.001.00186	IPP	10APR2000	1186	Tafenoquine
030.001.00187	I	10APR2000	1187	Tafenoquine
030.001.00188	IPP	13APR2000	1188	Mefloquine

*Popn=Population (I=Intent-To-Treat, PP=Per Protocol)
Tafenoquine = Tafenoquine 200mg, Mefloquine = Mefloquine 250mg

SB252263 - 030

Listing B.01

Treatment Assignment

Subject Number	Popn*	Screening Date	Randomisation Number	Treatment Group
030.001.00189	I	10APR2000	1189	Mefloquine
030.001.00190	IPP	11APR2000	1190	Tafenoquine
030.001.00191	IPP	18APR2000	1191	Placebo
030.001.00192	IPP	11APR2000	1192	Placebo
030.001.00193	IPP	14APR2000	1193	Placebo
030.001.00194	IPP	14APR2000	1194	Tafenoquine
030.001.00195	IPP	18APR2000	1195	Tafenoquine
030.001.00196	IPP	11APR2000	1196	Mefloquine
030.001.00197	IPP	17APR2000	1197	Placebo
030.001.00198	IPP	12APR2000	1198	Mefloquine
030.001.00199	IPP	10APR2000	1199	Mefloquine
030.001.00200	IPP	17APR2000	1200	Mefloquine
030.001.00201	IPP	10APR2000	1201	Tafenoquine
030.001.00202		17APR2000	1202	Placebo
030.001.00203	IPP	13APR2000	1203	Tafenoquine
030.001.00204	I	14APR2000	1204	Placebo
030.001.00205	IPP	10APR2000	1205	Tafenoquine
030.001.00206	I	10APR2000	1206	Mefloquine
030.001.00207	IPP	13APR2000	1207	Tafenoquine
030.001.00208	IPP	11APR2000	1208	Placebo
030.001.00209	IPP	13APR2000	1209	Tafenoquine
030.001.00210	IPP	12APR2000	1210	Tafenoquine
030.001.00211	IPP	11APR2000	1211	Placebo
030.001.00212	IPP	13APR2000	1212	Placebo
030.001.00213	IPP	14APR2000	1213	Mefloquine
030.001.00214	IPP	13APR2000	1214	Mefloquine
030.001.00215	IPP	12APR2000	1215	Placebo
030.001.00216	IPP	11APR2000	1216	Mefloquine
030.001.00217	IPP	11APR2000	1217	Tafenoquine
030.001.00218	IPP	13APR2000	1218	Tafenoquine
030.001.00219	IPP	12APR2000	1219	Placebo
030.001.00220	IPP	13APR2000	1220	Tafenoquine
030.001.00221	IPP	17APR2000	1221	Mefloquine
030.001.00222	IPP	14APR2000	1222	Placebo
030.001.00223	IPP	14APR2000	1223	Mefloquine
030.001.00224	IPP	12APR2000	1224	Mefloquine
030.001.00225	IPP	10APR2000	1225	Placebo
030.001.00226	IPP	13APR2000	1226	Placebo
030.001.00227	IPP	14APR2000	1227	Mefloquine
030.001.00228	IPP	13APR2000	1228	Tafenoquine
030.001.00229	IPP	13APR2000	1229	Placebo
030.001.00230	IPP	13APR2000	1230	Tafenoquine
030.001.00231	IPP	12APR2000	1231	Tafenoquine
030.001.00232	IPP	18APR2000	1232	Mefloquine
030.001.00233	IPP	10APR2000	1233	Tafenoquine
030.001.00234	IPP	11APR2000	1234	Placebo
030.001.00235	IPP	10APR2000	1235	Mefloquine

*Popn=Population (I=Intent-To-Treat, PP=Per Protocol)
Tafenoquine = Tafenoquine 200mg, Mefloquine = Mefloquine 250mg

SB252263 - 030

Listing B.01

Treatment Assignment

Subject Number	Popn*	Screening Date	Randomisation Number	Treatment Group
030.001.00236	IPP	11APR2000	1236	Placebo
030.001.00237	IPP	17APR2000	1237	Mefloquine
030.001.00238	IPP	12APR2000	1238	Placebo
030.001.00239	IPP	10APR2000	1239	Mefloquine
030.001.00240	IPP	13APR2000	1240	Tafenoquine
030.001.00241	IPP	13APR2000	1241	Mefloquine
030.001.00242	IPP	14APR2000	1242	Placebo
030.001.00243	IPP	13APR2000	1243	Tafenoquine
030.001.00244	IPP	13APR2000	1244	Mefloquine
030.001.00245	IPP	11APR2000	1245	Mefloquine
030.001.00246	IPP	11APR2000	1246	Tafenoquine
030.001.00247	IPP	11APR2000	1247	Placebo
030.001.00248	IPP	13APR2000	1248	Placebo
030.001.00249	IPP	12APR2000	1249	Tafenoquine
030.001.00250		18APR2000	1250	Mefloquine
030.001.00251	IPP	12APR2000	1251	Placebo
030.001.00252	IPP	12APR2000	1252	Tafenoquine
030.001.00253	IPP	13APR2000	1253	Placebo
030.001.00254	I	12APR2000	1254	Tafenoquine
030.001.00255	IPP	12APR2000	1255	Mefloquine
030.001.00256	IPP	14APR2000	1256	Placebo
030.001.00257	IPP	14APR2000	1257	Placebo
030.001.00258	IPP	14APR2000	1258	Mefloquine
030.001.00259	IPP	12APR2000	1259	Tafenoquine
030.001.00260	IPP	17APR2000	1260	Placebo
030.001.00261		13APR2000	1261	Tafenoquine
030.001.00262	IPP	13APR2000	1262	Mefloquine
030.001.00263	IPP	17APR2000	1263	Mefloquine
030.001.00264	IPP	18APR2000	1264	Tafenoquine
030.001.00265	IPP	12APR2000	1265	Mefloquine
030.001.00266	I	12APR2000	1266	Tafenoquine
030.001.00267	IPP	11APR2000	1267	Tafenoquine
030.001.00268	IPP	14APR2000	1268	Placebo
030.001.00269	IPP	13APR2000	1269	Placebo
030.001.00270	IPP	13APR2000	1270	Placebo
030.001.00271	I	13APR2000	1271	Tafenoquine
030.001.00272	IPP	10APR2000	1272	Tafenoquine
030.001.00273	IPP	11APR2000	1273	Mefloquine
030.001.00274	IPP	11APR2000	1274	Mefloquine
030.001.00275	IPP	12APR2000	1275	Placebo
030.001.00276	IPP	12APR2000	1276	Mefloquine
030.001.00277	IPP	12APR2000	1277	Placebo
030.001.00278	IPP	12APR2000	1278	Placebo
030.001.00279	IPP	12APR2000	1279	Tafenoquine
030.001.00280	IPP	13APR2000	1280	Mefloquine
030.001.00281	IPP	12APR2000	1281	Mefloquine
030.001.00282	IPP	11APR2000	1282	Tafenoquine

*Popn=Population (I=Intent-To-Treat, PP=Per Protocol)
Tafenoquine = Tafenoquine 200mg, Mefloquine = Mefloquine 250mg

SB252263 - 030

Listing B.01

Treatment Assignment

Subject Number	Popn*	Screening Date	Randomisation Number	Treatment Group
030.001.00283	IPP	14APR2000	1283	Mefloquine
030.001.00284	IPP	10APR2000	1284	Tafenoquine
030.001.00285	IPP	18APR2000	1285	Placebo
030.001.00286	IPP	12APR2000	1286	Mefloquine
030.001.00287	IPP	10APR2000	1287	Placebo
030.001.00288	IPP	12APR2000	1288	Tafenoquine
030.001.00289	IPP	14APR2000	1289	Mefloquine
030.001.00290	IPP	12APR2000	1290	Mefloquine
030.001.00291	IPP	17APR2000	1291	Placebo
030.001.00292	IPP	12APR2000	1292	Mefloquine
030.001.00293	IPP	11APR2000	1293	Tafenoquine
030.001.00294	IPP	13APR2000	1294	Tafenoquine
030.001.00295		17APR2000	1295	Placebo
030.001.00296	IPP	14APR2000	1296	Tafenoquine
030.001.00297	IPP	18APR2000	1297	Placebo
030.001.00298	IPP	17APR2000	1298	Tafenoquine
030.001.00299	IPP	17APR2000	1299	Mefloquine
030.001.00300	IPP	11APR2000	1300	Placebo
030.001.00301	IPP	14APR2000	1301	Tafenoquine
030.001.00302	I	14APR2000	1302	Tafenoquine
030.001.00303	IPP	13APR2000	1303	Placebo
030.001.00304	IPP	13APR2000	1304	Tafenoquine
030.001.00305	IPP	13APR2000	1305	Mefloquine
030.001.00306	IPP	13APR2000	1306	Tafenoquine

*Popn=Population (I=Intent-To-Treat, PP=Per Protocol)
Tafenoquine = Tafenoquine 200mg, Mefloquine = Mefloquine 250mg

SB252263 - 030

Listing B.02

Visit And Dosing Dates

Treatment : Placebo

Subject Number	Popn*	Visit	Visit Date	Dose Taken ?	Date of Dose	Time of Dose
030.001.00002	IPP	Screening	10APR2000	No		
		Screening (Unsch.)	29APR2000	No		
		Clearance (Day -5)	03MAY2000	Yes	03MAY2000	
		Clearance (Day -4)	04MAY2000	Yes	04MAY2000	
		Clearance (Day -3)	05MAY2000	Yes	05MAY2000	
		Load Dose 1	08MAY2000	Yes	08MAY2000	6:21
		Load Dose 2	09MAY2000	Yes	09MAY2000	7:30
		Load Dose 3	10MAY2000	Yes	10MAY2000	9:00
		Week 1	15MAY2000	Yes	15MAY2000	11:13
		Week 1 (Unsch.)	18MAY2000	No		
		Week 2	22MAY2000	Yes	22MAY2000	6:35
		Week 3 (Unsch.)	26MAY2000	No		
		Week 3	29MAY2000	Yes	29MAY2000	8:09
		Week 3 (Unsch.)	31MAY2000	No		
		Week 4	05JUN2000	Yes	05JUN2000	6:40
		Week 5	12JUN2000	Yes	12JUN2000	9:15
		Week 5 (Unsch.)	12JUN2000	No		
		Week 6	19JUN2000	Yes	19JUN2000	11:20
		Week 6 (Unsch. Fol. Up)	22JUN2000	No		
		Week 7 (Fol. Up)	26JUN2000	No		
		Week 8 (Unsch. Fol. Up)	30JUN2000	No		
		Week 8 (Fol. Up)	03JUL2000	No		
		Week 8 (Unsch. Fol. Up)	03JUL2000	No		
		Week 9 (Fol. Up)	10JUL2000	No		
		Week 10 (Fol. Up)	17JUL2000	No		
030.001.00005	IPP	Screening	10APR2000	No		
		Screening (Unsch.)	25APR2000	No		
		Clearance (Day -5)	03MAY2000	Yes	03MAY2000	
		Clearance (Day -4)	04MAY2000	Yes	04MAY2000	
		Clearance (Day -3)	05MAY2000	Yes	05MAY2000	
		Load Dose 1	08MAY2000	Yes	08MAY2000	5:48
		Load Dose 2	09MAY2000	Yes	09MAY2000	11:35
		Load Dose 3	10MAY2000	Yes	10MAY2000	7:31
		Week 1 (Unsch.)	12MAY2000	No		
		Week 1	15MAY2000	Yes	15MAY2000	7:11
		Week 2	22MAY2000	Yes	22MAY2000	6:16
		Week 3	30MAY2000	Yes	30MAY2000	9:45
		Week 4	04JUN2000	Yes	04JUN2000	7:20
		Week 4 (Unsch.)	05JUN2000	No		
		Week 5	12JUN2000	Yes	12JUN2000	6:59
		Week 6 (Unsch.)	17JUN2000	No		
		Week 6	19JUN2000	Yes	19JUN2000	7:25

*Popn=Population (I=Intent-to-Treat, PP=Per Protocol)
Tafenoquine = Tafenoquine 200mg, Mefloquine = Mefloquine 250mg

SB252263 - 030

Listing B.02

Visit And Dosing Dates

Treatment : Placebo

Subject Number	Popn*	Visit	Visit Date	Dose Taken ?	Date of Dose	Time of Dose
030.001.00005	IPP	Week 6 (Unsch.)	19JUN2000	No		
		Week 6 (Unsch.)	22JUN2000	No		
		Week 7	26JUN2000	Yes	26JUN2000	6:25
		Week 8	03JUL2000	Yes	03JUL2000	6:10
		Week 9 (Fol. Up)	10JUL2000	No		
		Week 10 (Unsch. Fol. Up)	16JUL2000	No		
		Week 10 (Fol. Up)	17JUL2000	No		
		Week 12 (Fol. Up)	28JUL2000	No		
		Week 12 (Unsch. Fol. Up)	28JUL2000	No		
030.001.00011	IPP	Screening	10APR2000	No		
		Screening (Unsch.)	19APR2000	No		
		Clearance (Day -5)	03MAY2000	Yes	03MAY2000	
		Clearance (Day -4)	04MAY2000	Yes	04MAY2000	
		Clearance (Day -3)	05MAY2000	Yes	05MAY2000	
		Load Dose 1	08MAY2000	Yes	08MAY2000	6:59
		Load Dose 2	09MAY2000	Yes	09MAY2000	8:12
		Load Dose 3	10MAY2000	Yes	10MAY2000	11:42
		Week 1	15MAY2000	Yes	15MAY2000	13:12
		Week 2	22MAY2000	Yes	22MAY2000	8:28
		Week 3	29MAY2000	Yes	29MAY2000	7:55
		Week 3 (Unsch. Fol. Up)	30MAY2000	No		
		Week 4 (Fol. Up)	04JUN2000	No		
		Week 5 (Fol. Up)	12JUN2000	No		
		Week 6 (Unsch. Fol. Up)	16JUN2000	No		
		Week 6 (Fol. Up)	19JUN2000	No		
		Week 7 (Unsch. Fol. Up)	23JUN2000	No		
		Week 7 (Fol. Up)	26JUN2000	No		
030.001.00012	IPP	Screening	10APR2000	No		
		Screening (Unsch.)	17APR2000	No		
		Screening (Unsch.)	26APR2000	No		
		Clearance (Day -5)	03MAY2000	Yes	03MAY2000	
		Clearance (Day -4)	04MAY2000	Yes	04MAY2000	
		Clearance (Day -3)	05MAY2000	Yes	05MAY2000	
		Load Dose 1	08MAY2000	Yes	08MAY2000	7:08
		Load Dose 2	09MAY2000	Yes	09MAY2000	7:05
		Load Dose 3	10MAY2000	Yes	10MAY2000	8:20
		Load Dose (Unsch.)	10MAY2000	No		
		Week 1 (Unsch.)	11MAY2000	No		
		Week 1	15MAY2000	Yes	15MAY2000	9:00
		Week 2	23MAY2000	Yes	23MAY2000	12:00

*Popn=Population (I=Intent-to-Treat, PP=Per Protocol)
Tafenoquine = Tafenoquine 200mg, Mefloquine = Mefloquine 250mg

SB252263 - 030

Listing B.02

Visit And Dosing Dates

Treatment : Placebo

Subject Number	Popn*	Visit	Visit Date	Dose Taken ?	Date of Dose	Time of Dose
030.001.00012	IPP	Week 3	29MAY2000	Yes	29MAY2000	17:00
		Week 4	05JUN2000	Yes	05JUN2000	8:55
		Week 5	12JUN2000	Yes	12JUN2000	6:50
		Week 6	19JUN2000	Yes	19JUN2000	7:50
		Week 7	26JUN2000	Yes	26JUN2000	7:40
		Week 8 (Fol. Up)	03JUL2000	No		
		Week 9 (Unsch. Fol. Up)	08JUL2000	No		
		Week 9 (Unsch. Fol. Up)	09JUL2000	No		
		Week 9 (Fol. Up)	10JUL2000	No		
		Week 9 (Unsch. Fol. Up)	12JUL2000	No		
		Week 9 (Unsch. Fol. Up)	13JUL2000	No		
		Week 10 (Unsch. Fol. Up)	14JUL2000	No		
		Week 10 (Fol. Up)	17JUL2000	No		
		Week 11 (Unsch. Fol. Up)	21JUL2000	No		
		Week 11 (Fol. Up)	25JUL2000	No		
030.001.00015	I	Screening	10APR2000	No		
		Screening (Unsch.)	24APR2000	No		
		Clearance (Day -5)	03MAY2000	Yes	03MAY2000	
		Clearance (Day -4)	04MAY2000	Yes	04MAY2000	
		Clearance (Day -3)	05MAY2000	Yes	05MAY2000	
		Load Dose 1	08MAY2000	Yes	08MAY2000	7:35
		Load Dose 2	09MAY2000	Yes	09MAY2000	7:05
		Load Dose 3	10MAY2000	Yes	10MAY2000	8:55
		Week 1	15MAY2000	Yes	15MAY2000	8:33
		Week 1 (Unsch.)	18MAY2000	No		
		Week 2	22MAY2000	Yes	22MAY2000	8:00
		Week 3	01JUN2000	Yes	01JUN2000	9:30
		Week 4	05JUN2000	Yes	05JUN2000	17:20
		Week 4 (Unsch.)	08JUN2000	No		
		Week 5	12JUN2000	Yes	12JUN2000	8:13
		Week 5 (Unsch.)	15JUN2000	No		
		Week 6	19JUN2000	Yes	19JUN2000	7:02
		Week 7	26JUN2000	Yes	26JUN2000	9:52
		Week 8 (Unsch.)	30JUN2000	No		
		Week 8	03JUL2000	Yes	03JUL2000	6:46
		Week 9	10JUL2000	Yes	10JUL2000	8:30
		Week 10 (Unsch.)	14JUL2000	No		
		Week 10	17JUL2000	Yes	17JUL2000	8:00
		Week 10 (Unsch. Fol. Up)	18JUL2000	No		
030.001.00019	IPP	Screening	10APR2000	No		

*Popn=Population (I=Intent-to-Treat, PP=Per Protocol)
Tafenoquine = Tafenoquine 200mg, Mefloquine = Mefloquine 250mg

SB252263 - 030

Listing B.02

Visit And Dosing Dates

Treatment : Placebo

Subject Number	Popn*	Visit	Visit Date	Dose Taken ?	Date of Dose	Time of Dose
030.001.00019	IPP	Screening (Unsch.)	14APR2000	No		
		Screening (Unsch.)	20APR2000	No		
		Screening (Unsch.)	26APR2000	No		
		Screening (Unsch.)	02MAY2000	No		
		Clearance (Day -5)	03MAY2000	Yes	03MAY2000	
		Clearance (Day -4)	04MAY2000	Yes	04MAY2000	
		Clearance (Day -3)	05MAY2000	Yes	05MAY2000	
		Clearance (Unsch.)	03MAY2000	No		
		Load Dose 1	08MAY2000	Yes	08MAY2000	7:54
		Load Dose 2	09MAY2000	Yes	09MAY2000	7:25
		Load Dose 3	10MAY2000	Yes	10MAY2000	8:42
		Week 1 (Unsch.)	12MAY2000	No		
		Week 1	15MAY2000	Yes	15MAY2000	9:03
		Week 2	22MAY2000	Yes	22MAY2000	7:55
		Week 3	29MAY2000	Yes	29MAY2000	9:40
		Week 4	05JUN2000	Yes	05JUN2000	9:05
		Week 5	12JUN2000	Yes	12JUN2000	9:55
		Week 6	19JUN2000	Yes	19JUN2000	9:55
		Week 7	26JUN2000	Yes	26JUN2000	7:05
		Week 8	04JUL2000	Yes	04JUL2000	11:55
		Week 9 (Unsch.)	09JUL2000	No		
		Week 9	10JUL2000	Yes	10JUL2000	10:53
		Week 10 (Fol. Up)	17JUL2000	No		
		Week 11 (Fol. Up)	24JUL2000	No		
		Week 12 (Fol. Up)	31JUL2000	No		
030.001.00020	IPP	Screening	10APR2000	No		
		Screening (Unsch.)	01MAY2000	No		
		Clearance (Day -5)	03MAY2000	Yes	03MAY2000	
		Clearance (Day -4)	04MAY2000	Yes	04MAY2000	
		Clearance (Day -3)	05MAY2000	Yes	05MAY2000	
		Load Dose 1	08MAY2000	Yes	08MAY2000	7:55
		Load Dose 2	09MAY2000	Yes	09MAY2000	6:31
		Load Dose 3	10MAY2000	Yes	10MAY2000	11:08
		Week 1 (Unsch.)	13MAY2000	No		
		Week 1	15MAY2000	Yes	15MAY2000	10:05
		Week 2	22MAY2000	Yes	22MAY2000	8:45
		Week 3 (Unsch.)	26MAY2000	No		
		Week 3	29MAY2000	Yes	29MAY2000	8:00
		Week 4	05JUN2000	Yes	05JUN2000	11:19
		Week 5	12JUN2000	Yes	12JUN2000	6:50
		Week 6	19JUN2000	Yes	19JUN2000	7:27
		Week 7	26JUN2000	Yes	26JUN2000	7:30

*Popn=Population (I=Intent-to-Treat, PP=Per Protocol)
Tafenoquine = Tafenoquine 200mg, Mefloquine = Mefloquine 250mg

SB252263 - 030

Listing B.02

Visit And Dosing Dates

Treatment : Placebo

Subject Number	Popn*	Visit	Visit Date	Dose Taken ?	Date of Dose	Time of Dose
030.001.00020	IPP	Week 8	03JUL2000	Yes	03JUL2000	8:19
		Week 9	10JUL2000	Yes	10JUL2000	6:02
		Week 10 (Fol. Up)	17JUL2000	No		
		Week 11 (Fol. Up)	24JUL2000	No		
		Week 11 (Unsch. Fol. Up)	24JUL2000	No		
		Week 12 (Fol. Up)	31JUL2000	No		
		Week 12 (Unsch. Fol. Up)	31JUL2000	No		
030.001.00022	IPP	Screening	13APR2000	No		
		Screening (Unsch.)	01MAY2000	No		
		Clearance (Day -5)	03MAY2000	Yes	03MAY2000	
		Clearance (Day -4)	04MAY2000	Yes	04MAY2000	
		Clearance (Day -3)	05MAY2000	Yes	05MAY2000	
		Load Dose 1	08MAY2000	Yes	08MAY2000	7:40
		Load Dose 2	09MAY2000	Yes	09MAY2000	7:04
		Load Dose 3	10MAY2000	Yes	10MAY2000	10:45
		Week 1	15MAY2000	Yes	15MAY2000	10:40
		Week 2	22MAY2000	Yes	22MAY2000	9:11
		Week 2 (Unsch.)	22MAY2000	No		
		Week 3 (Unsch. Fol. Up)	26MAY2000	No		
		Week 3 (Fol. Up)	29MAY2000	No		
		Week 4 (Fol. Up)	05JUN2000	No		
		Week 5 (Fol. Up)	12JUN2000	No		
		Week 5 (Unsch. Fol. Up)	14JUN2000	No		
		Week 6 (Fol. Up)	19JUN2000	No		
		Week 6 (Unsch. Fol. Up)	20JUN2000	No		
030.001.00026	IPP	Screening	10APR2000	No		
		Screening (Unsch.)	16APR2000	No		
		Clearance (Day -5)	03MAY2000	Yes	03MAY2000	
		Clearance (Day -4)	04MAY2000	Yes	04MAY2000	
		Clearance (Day -3)	05MAY2000	Yes	05MAY2000	
		Load Dose 1	08MAY2000	Yes	08MAY2000	7:47
		Load Dose 2	09MAY2000	Yes	09MAY2000	6:12
		Load Dose 3	10MAY2000	Yes	10MAY2000	15:00
		Week 1 (Unsch.)	14MAY2000	No		
		Week 1	15MAY2000	Yes	15MAY2000	13:55
		Week 2	22MAY2000	Yes	22MAY2000	6:00
		Week 4 (Fol. Up)	03JUN2000	No		
		Week 4 (Unsch. Fol. Up)	03JUN2000	No		
		Week 4 (Fol. Up)	06JUN2000	No		
		Week 5 (Fol. Up)	12JUN2000	No		

*Popn=Population (I=Intent-to-Treat, PP=Per Protocol)
Tafenoquine = Tafenoquine 200mg, Mefloquine = Mefloquine 250mg

SB252263 - 030

Listing B.02

Visit And Dosing Dates

Treatment : Placebo

Subject Number	Popn*	Visit	Visit Date	Dose Taken ?	Date of Dose	Time of Dose
030.001.00026	IPP	Week 6 (Fol. Up)	19JUN2000	No		
		Week 6 (Unsch. Fol. Up)	19JUN2000	No		
030.001.00027	IPP	Screening	12APR2000	No		
		Clearance (Day -5)	03MAY2000	Yes	03MAY2000	
		Clearance (Day -4)	04MAY2000	Yes	04MAY2000	
		Clearance (Day -3)	05MAY2000	Yes	05MAY2000	
		Load Dose 1	08MAY2000	Yes	08MAY2000	8:01
		Load Dose 2	09MAY2000	Yes	09MAY2000	7:00
		Load Dose 3	10MAY2000	Yes	10MAY2000	13:47
		Load Dose (Unsch.)	09MAY2000	No		
		Week 1	15MAY2000	Yes	15MAY2000	12:21
		Week 2	22MAY2000	Yes	22MAY2000	6:20
		Week 2 (Unsch. Fol. Up)	24MAY2000	No		
		Week 3 (Fol. Up)	30MAY2000	No		
		Week 4 (Fol. Up)	05JUN2000	No		
		Week 5 (Fol. Up)	12JUN2000	No		
		Week 6 (Fol. Up)	19JUN2000	No		
030.001.00032	IPP	Screening	13APR2000	No		
		Screening (Unsch.)	28APR2000	No		
		Clearance (Day -5)	03MAY2000	Yes	03MAY2000	
		Clearance (Day -4)	04MAY2000	Yes	04MAY2000	
		Clearance (Day -3)	05MAY2000	Yes	05MAY2000	
		Load Dose 1	08MAY2000	Yes	08MAY2000	8:17
		Load Dose 2	09MAY2000	Yes	09MAY2000	7:43
		Load Dose 3	10MAY2000	Yes	10MAY2000	8:05
		Week 1	16MAY2000	Yes	16MAY2000	11:56
		Week 2	22MAY2000	Yes	22MAY2000	8:30
		Week 2 (Unsch.)	23MAY2000	No		
		Week 3	29MAY2000	Yes	29MAY2000	9:09
		Week 4	05JUN2000	Yes	05JUN2000	
		Week 4 (Unsch.)	06JUN2000	No		
		Week 5	12JUN2000	Yes	12JUN2000	8:36
		Week 6	19JUN2000	Yes	19JUN2000	8:51
		Week 7	26JUN2000	Yes	26JUN2000	9:11
		Week 8	03JUL2000	Yes	03JUL2000	8:56
		Week 9 (Fol. Up)	10JUL2000	No		
		Week 9 (Unsch. Fol. Up)	11JUL2000	No		
		Week 10 (Fol. Up)	17JUL2000	No		
		Week 11 (Fol. Up)	24JUL2000	No		

*Popn=Population (I=Intent-to-Treat, PP=Per Protocol)
Tafenoquine = Tafenoquine 200mg, Mefloquine = Mefloquine 250mg

SB252263 - 030

Listing B.02

Visit And Dosing Dates

Treatment : Placebo

Subject Number	Popn*	Visit	Visit Date	Dose Taken ?	Date of Dose	Time of Dose
030.001.00033	IPP	Screening	11APR2000	No		
		Screening (Unsch.)	14APR2000	No		
		Screening (Unsch.)	19APR2000	No		
		Clearance (Day -5)	03MAY2000	Yes	03MAY2000	
		Clearance (Day -4)	04MAY2000	Yes	04MAY2000	
		Clearance (Day -3)	05MAY2000	Yes	05MAY2000	
		Load Dose 1	08MAY2000	Yes	08MAY2000	8:26
		Load Dose 2	09MAY2000	Yes	09MAY2000	6:55
		Load Dose 3	10MAY2000	Yes	10MAY2000	7:41
		Week 1	15MAY2000	Yes	15MAY2000	7:00
		Week 2	22MAY2000	Yes	22MAY2000	7:22
		Week 2 (Unsch. Fol. Up)	24MAY2000	No		
		Week 3 (Unsch. Fol. Up)	26MAY2000	No		
		Week 3 (Fol. Up)	29MAY2000	No		
		Week 4 (Fol. Up)	04JUN2000	No		
		Week 5 (Fol. Up)	12JUN2000	No		
		Week 5 (Unsch. Fol. Up)	13JUN2000	No		
		Week 6 (Fol. Up)	19JUN2000	No		
		Week 6 (Unsch. Fol. Up)	19JUN2000	No		
030.001.00037	IPP	Screening	11APR2000	No		
		Screening (Unsch.)	21APR2000	No		
		Clearance (Day -5)	03MAY2000	Yes	03MAY2000	
		Clearance (Day -4)	04MAY2000	Yes	04MAY2000	
		Clearance (Day -3)	05MAY2000	Yes	05MAY2000	
		Load Dose 1	08MAY2000	Yes	08MAY2000	8:30
		Load Dose 2	09MAY2000	Yes	09MAY2000	6:50
		Load Dose 3	10MAY2000	Yes	10MAY2000	7:30
		Week 1	15MAY2000	Yes	15MAY2000	14:50
		Week 2	22MAY2000	Yes	22MAY2000	6:25
		Week 3 (Unsch.)	26MAY2000	No		
		Week 3	29MAY2000	Yes	29MAY2000	7:17
		Week 4 (Fol. Up)	05JUN2000	No		
		Week 5 (Fol. Up)	12JUN2000	No		
		Week 6 (Fol. Up)	19JUN2000	No		
		Week 7 (Unsch. Fol. Up)	23JUN2000	No		
		Week 7 (Fol. Up)	26JUN2000	No		
030.001.00039	IPP	Screening	14APR2000	No		
		Screening (Unsch.)	28APR2000	No		
		Clearance (Day -5)	03MAY2000	Yes	03MAY2000	
		Clearance (Day -4)	04MAY2000	Yes	04MAY2000	

*Popn=Population (I=Intent-to-Treat, PP=Per Protocol)
Tafenoquine = Tafenoquine 200mg, Mefloquine = Mefloquine 250mg

SB252263 - 030

Listing B.02

Visit And Dosing Dates

Treatment : Placebo

Subject Number	Popn*	Visit	Visit Date	Dose Taken ?	Date of Dose	Time of Dose
030.001.00039	IPP	Clearance (Day -3)	05MAY2000	Yes	05MAY2000	
		Load Dose 1	08MAY2000	Yes	08MAY2000	8:40
		Load Dose 2	09MAY2000	Yes	09MAY2000	7:20
		Load Dose 3	10MAY2000	Yes	10MAY2000	9:38
		Load Dose (Unsch.)	10MAY2000	No		
		Week 1	15MAY2000	Yes	15MAY2000	13:07
		Week 2	22MAY2000	Yes	22MAY2000	6:32
		Week 3	29MAY2000	Yes	29MAY2000	7:25
		Week 4	04JUN2000	Yes	04JUN2000	10:30
		Week 5	12JUN2000	Yes	12JUN2000	6:50
		Week 6 (Fol. Up)	19JUN2000	No		
		Week 7 (Fol. Up)	26JUN2000	No		
		Week 9 (Fol. Up)	07JUL2000	No		
		Week 9 (Fol. Up)	08JUL2000	No		
030.001.00041	IPP	Screening	10APR2000	No		
		Screening (Unsch.)	25APR2000	No		
		Clearance (Day -5)	03MAY2000	Yes	03MAY2000	
		Clearance (Day -4)	04MAY2000	Yes	04MAY2000	
		Clearance (Day -3)	05MAY2000	Yes	05MAY2000	
		Load Dose 1	08MAY2000	Yes	08MAY2000	8:39
		Load Dose 2	09MAY2000	Yes	09MAY2000	6:55
		Load Dose 3	10MAY2000	Yes	10MAY2000	7:35
		Week 1	16MAY2000	Yes	16MAY2000	12:14
		Week 2	22MAY2000	Yes	22MAY2000	7:10
		Week 2 (Unsch.)	22MAY2000	No		
		Week 3	29MAY2000	Yes	29MAY2000	8:00
		Week 4	05JUN2000	Yes	05JUN2000	12:45
		Week 4 (Unsch.)	06JUN2000	No		
		Week 5	12JUN2000	Yes	12JUN2000	7:53
		Week 6 (Fol. Up)	19JUN2000	No		
		Week 7 (Unsch. Fol. Up)	23JUN2000	No		
		Week 7 (Fol. Up)	26JUN2000	No		
		Week 8 (Fol. Up)	03JUL2000	No		
		Week 8 (Unsch. Fol. Up)	04JUL2000	No		
		Week 9 (Fol. Up)	10JUL2000	No		
030.001.00044	IPP	Screening	10APR2000	No		
		Clearance (Day -5)	03MAY2000	Yes	03MAY2000	
		Clearance (Day -4)	04MAY2000	Yes	04MAY2000	
		Clearance (Day -3)	05MAY2000	Yes	05MAY2000	
		Load Dose 1	08MAY2000	Yes	08MAY2000	8:45

*Popn=Population (I=Intent-to-Treat, PP=Per Protocol)
Tafenoquine = Tafenoquine 200mg, Mefloquine = Mefloquine 250mg

SB252263 - 030

Listing B.02

Visit And Dosing Dates

Treatment : Placebo

Subject Number	Popn*	Visit	Visit Date	Dose Taken ?	Date of Dose	Time of Dose
030.001.00044	IPP	Load Dose 2	09MAY2000	Yes	09MAY2000	6:35
		Load Dose 3	10MAY2000	Yes	10MAY2000	7:30
		Week 1 (Unsch.)	14MAY2000	No		
		Week 1	15MAY2000	Yes	15MAY2000	8:48
		Week 2	22MAY2000	Yes	22MAY2000	6:20
		Week 3	29MAY2000	Yes	29MAY2000	6:35
		Week 4 (Unsch.)	02JUN2000	No		
		Week 4	05JUN2000	Yes	05JUN2000	9:41
		Week 5 (Unsch.)	11JUN2000	No		
		Week 5	12JUN2000	Yes	12JUN2000	6:59
		Week 6	19JUN2000	Yes	19JUN2000	7:50
		Week 6 (Unsch. Fol. Up)	22JUN2000	No		
		Week 7 (Unsch. Fol. Up)	24JUN2000	No		
		Week 7 (Fol. Up)	26JUN2000	No		
		Week 7 (Unsch. Fol. Up)	26JUN2000	No		
		Week 7 (Unsch. Fol. Up)	29JUN2000	No		
		Week 8 (Fol. Up)	03JUL2000	No		
		Week 9 (Unsch. Fol. Up)	07JUL2000	No		
		Week 9 (Fol. Up)	10JUL2000	No		
		Week 10 (Unsch. Fol. Up)	17JUL2000	No		
		Week 10 (Unsch. Fol. Up)	18JUL2000	No		
030.001.00055	IPP	Screening	12APR2000	No		
		Clearance (Day -5)	03MAY2000	Yes	03MAY2000	
		Clearance (Day -4)	04MAY2000	Yes	04MAY2000	
		Clearance (Day -3)	05MAY2000	Yes	05MAY2000	
		Load Dose 1	08MAY2000	Yes	08MAY2000	9:40
		Load Dose 2	09MAY2000	Yes	09MAY2000	7:35
		Load Dose 3	10MAY2000	Yes	10MAY2000	8:32
		Week 1	15MAY2000	Yes	15MAY2000	7:40
		Week 2	22MAY2000	Yes	22MAY2000	6:40
		Week 3 (Fol. Up)	29MAY2000	No		
		Week 4 (Fol. Up)	05JUN2000	No		
		Week 5 (Fol. Up)	12JUN2000	No		
		Week 6 (Fol. Up)	19JUN2000	No		
030.001.00056	I	Screening	11APR2000	No		
		Screening (Unsch.)	18APR2000	No		
		Screening (Unsch.)	28APR2000	No		
		Clearance (Day -5)	03MAY2000	Yes	03MAY2000	
		Clearance (Day -4)	04MAY2000	Yes	04MAY2000	
		Clearance (Day -3)	05MAY2000	Yes	05MAY2000	

*Popn=Population (I=Intent-to-Treat, PP=Per Protocol)
Tafenoquine = Tafenoquine 200mg, Mefloquine = Mefloquine 250mg

SB252263 - 030

Listing B.02

Visit And Dosing Dates

Treatment : Placebo

Subject Number	Popn*	Visit	Visit Date	Dose Taken ?	Date of Dose	Time of Dose
030.001.00056	I	Clearance (Unsch.)	06MAY2000	No		
		Load Dose 1	08MAY2000	Yes	08MAY2000	9:38
		Load Dose 2	09MAY2000	Yes	09MAY2000	8:34
		Load Dose 3	10MAY2000	Yes	10MAY2000	8:47
		Week 1	15MAY2000	Yes	15MAY2000	8:51
		Week 1 (Unsch.)	16MAY2000	No		
		Week 2	22MAY2000	Yes	22MAY2000	9:00
		Week 3	29MAY2000	Yes	29MAY2000	10:50
		Week 4	05JUN2000	Yes	05JUN2000	14:38
		Week 4 (Unsch.)	06JUN2000	No		
		Week 5	12JUN2000	Yes	12JUN2000	9:08
		Week 6 (Fol. Up)	19JUN2000	No		
		Week 8 (Fol. Up)	30JUN2000	No		
		Week 8 (Unsch. Fol. Up)	01JUL2000	No		
		Week 8 (Fol. Up)	05JUL2000	No		
		Week 9 (Fol. Up)	10JUL2000	No		
030.001.00057	IPP	Screening	14APR2000	No		
		Clearance (Day -5)	03MAY2000	Yes	03MAY2000	
		Clearance (Day -4)	04MAY2000	Yes	04MAY2000	
		Clearance (Day -3)	05MAY2000	Yes	05MAY2000	
		Load Dose 1	08MAY2000	Yes	08MAY2000	9:31
		Load Dose 2	09MAY2000	Yes	09MAY2000	7:45
		Load Dose 3	10MAY2000	Yes	10MAY2000	9:05
		Load Dose (Unsch.)	10MAY2000	No		
		Week 1	15MAY2000	Yes	15MAY2000	9:15
		Week 1 (Unsch.)	18MAY2000	No		
		Week 2	22MAY2000	Yes	22MAY2000	7:30
		Week 3	29MAY2000	Yes	29MAY2000	7:30
		Week 4	05JUN2000	Yes	05JUN2000	
		Week 5	12JUN2000	Yes	12JUN2000	8:00
		Week 6	19JUN2000	Yes	19JUN2000	6:30
		Week 7	26JUN2000	Yes	26JUN2000	8:30
		Week 8 (Fol. Up)	03JUL2000	No		
		Week 9 (Fol. Up)	11JUL2000	No		
		Week 10 (Fol. Up)	17JUL2000	No		
		Week 11 (Fol. Up)	24JUL2000	No		
030.001.00059	IPP	Screening	13APR2000	No		
		Screening (Unsch.)	14APR2000	No		
		Screening (Unsch.)	26APR2000	No		
		Clearance (Day -5)	03MAY2000	Yes	03MAY2000	

*Popn=Population (I=Intent-to-Treat, PP=Per Protocol)
Tafenoquine = Tafenoquine 200mg, Mefloquine = Mefloquine 250mg

SB252263 - 030

Listing B.02

Visit And Dosing Dates

Treatment : Placebo

Subject Number	Popn*	Visit	Visit Date	Dose Taken ?	Date of Dose	Time of Dose
030.001.00059	IPP	Clearance (Day -4)	04MAY2000	Yes	04MAY2000	
		Clearance (Day -3)	05MAY2000	Yes	05MAY2000	
		Clearance (Unsch.)	06MAY2000	No		
		Load Dose 1	08MAY2000	Yes	08MAY2000	9:45
		Load Dose 2	09MAY2000	Yes	09MAY2000	8:02
		Load Dose 3	10MAY2000	Yes	10MAY2000	8:03
		Week 1	15MAY2000	Yes	15MAY2000	7:30
		Week 1 (Unsch.)	16MAY2000	No		
		Week 2	22MAY2000	Yes	22MAY2000	7:20
		Week 2 (Unsch. Fol. Up)	24MAY2000	No		
		Week 3 (Fol. Up)	29MAY2000	No		
		Week 3 (Unsch. Fol. Up)	29MAY2000	No		
		Week 4 (Fol. Up)	05JUN2000	No		
		Week 5 (Fol. Up)	12JUN2000	No		
		Week 6 (Fol. Up)	19JUN2000	No		
030.001.00063	IPP	Screening	11APR2000	No		
		Clearance (Day -5)	03MAY2000	Yes	03MAY2000	
		Clearance (Day -4)	04MAY2000	Yes	04MAY2000	
		Clearance (Day -3)	05MAY2000	Yes	05MAY2000	
		Load Dose 1	08MAY2000	Yes	08MAY2000	10:00
		Load Dose 2	09MAY2000	Yes	09MAY2000	7:30
		Load Dose 3	10MAY2000	Yes	10MAY2000	8:13
		Week 1 (Unsch.)	11MAY2000	No		
		Week 1	15MAY2000	Yes	15MAY2000	7:35
		Week 2	22MAY2000	Yes	22MAY2000	6:30
		Week 2 (Unsch.)	22MAY2000	No		
		Week 3	29MAY2000	Yes	29MAY2000	6:15
		Week 3 (Unsch.)	30MAY2000	No		
		Week 4	05JUN2000	Yes	05JUN2000	13:25
		Week 5	12JUN2000	Yes	12JUN2000	8:00
		Week 6	19JUN2000	Yes	19JUN2000	6:20
		Week 7	26JUN2000	Yes	26JUN2000	6:35
		Week 8	03JUL2000	Yes	03JUL2000	12:35
		Week 8 (Fol. Up)	05JUL2000	No		
		Week 9 (Fol. Up)	11JUL2000	No		
		Week 10 (Fol. Up)	17JUL2000	No		
		Week 11 (Fol. Up)	24JUL2000	No		
		Week 12 (Fol. Up)	31JUL2000	No		
030.001.00064	IPP	Screening	11APR2000	No		
		Screening (Unsch.)	30APR2000	No		

*Popn=Population (I=Intent-to-Treat, PP=Per Protocol)
Tafenoquine = Tafenoquine 200mg, Mefloquine = Mefloquine 250mg

SB252263 - 030

Listing B.02

Visit And Dosing Dates

Treatment : Placebo

Subject Number	Popn*	Visit	Visit Date	Dose Taken ?	Date of Dose	Time of Dose
030.001.00064	IPP	Clearance (Day -5)	03MAY2000	Yes	03MAY2000	
		Clearance (Day -4)	04MAY2000	Yes	04MAY2000	
		Clearance (Day -3)	05MAY2000	Yes	05MAY2000	
		Load Dose 1	08MAY2000	Yes	08MAY2000	9:35
		Load Dose 2	09MAY2000	Yes	09MAY2000	7:55
		Load Dose 3	10MAY2000	Yes	10MAY2000	9:15
		Week 1 (Unsch.)	13MAY2000	No		
		Week 1	15MAY2000	Yes	15MAY2000	8:24
		Week 2 (Unsch.)	21MAY2000	No		
		Week 2	22MAY2000	Yes	22MAY2000	8:05
		Week 3	29MAY2000	Yes	29MAY2000	8:50
		Week 4	04JUN2000	Yes	04JUN2000	10:36
		Week 5	12JUN2000	Yes	12JUN2000	9:35
		Week 6	19JUN2000	Yes	19JUN2000	9:30
		Week 7	26JUN2000	Yes	26JUN2000	9:10
		Week 8 (Fol. Up)	03JUL2000	No		
		Week 8 (Fol. Up)	05JUL2000	No		
		Week 8 (Unsch. Fol. Up)	05JUL2000	No		
		Week 9 (Fol. Up)	10JUL2000	No		
		Week 10 (Unsch. Fol. Up)	16JUL2000	No		
		Week 10 (Fol. Up)	17JUL2000	No		
		Week 11 (Fol. Up)	24JUL2000	No		
030.001.00067	IPP	Screening	12APR2000	No		
		Screening (Unsch.)	15APR2000	No		
		Screening (Unsch.)	18APR2000	No		
		Clearance (Day -5)	03MAY2000	Yes	03MAY2000	
		Clearance (Day -4)	04MAY2000	Yes	04MAY2000	
		Clearance (Day -3)	05MAY2000	Yes	05MAY2000	
		Load Dose 1	08MAY2000	Yes	08MAY2000	10:20
		Load Dose 2	09MAY2000	Yes	09MAY2000	7:28
		Load Dose 3	10MAY2000	Yes	10MAY2000	9:05
		Week 1 (Unsch.)	12MAY2000	No		
		Week 1 (Unsch.)	13MAY2000	No		
		Week 1	15MAY2000	Yes	15MAY2000	8:12
		Week 2	23MAY2000	Yes	23MAY2000	7:30
		Week 3	29MAY2000	Yes	29MAY2000	7:55
		Week 3 (Unsch.)	29MAY2000	No		
		Week 4	05JUN2000	Yes	05JUN2000	9:33
		Week 5	12JUN2000	Yes	12JUN2000	9:41
		Week 6	19JUN2000	Yes	19JUN2000	8:26
		Week 7	26JUN2000	Yes	26JUN2000	8:47
		Week 9	07JUL2000	No		

*Popn=Population (I=Intent-to-Treat, PP=Per Protocol)
Tafenoquine = Tafenoquine 200mg, Mefloquine = Mefloquine 250mg

SB252263 - 030

Listing B.02

Visit And Dosing Dates

Treatment : Placebo

Subject Number	Popn*	Visit	Visit Date	Dose Taken ?	Date of Dose	Time of Dose
030.001.00067	IPP	Week 9	10JUL2000	Yes	10JUL2000	8:18
		Week 9 (Unsch.)	13JUL2000	No		
		Week 10	20JUL2000	Yes	20JUL2000	9:35
		Week 11	24JUL2000	Yes	24JUL2000	8:25
		Week 12	31JUL2000	Yes	31JUL2000	10:05
		Week 13	07AUG2000	Yes	07AUG2000	9:07
		Week 13 (Unsch.)	07AUG2000	No		
		Week 14 (Fol. Up)	14AUG2000	No		
		Week 15 (Fol. Up)	21AUG2000	No		
		Week 15 (Unsch. Fol. Up)	24AUG2000	No		
		Week 16 (Fol. Up)	28AUG2000	No		
		Week 17 (Fol. Up)	04SEP2000	No		
030.001.00072	IPP	Screening	12APR2000	No		
		Screening (Unsch.)	27APR2000	No		
		Clearance (Day -5)	03MAY2000	Yes	03MAY2000	
		Clearance (Day -4)	04MAY2000	Yes	04MAY2000	
		Clearance (Day -3)	05MAY2000	Yes	05MAY2000	
		Load Dose 1	08MAY2000	Yes	08MAY2000	10:05
		Load Dose 2	09MAY2000	Yes	09MAY2000	6:40
		Load Dose 3	10MAY2000	Yes	10MAY2000	8:45
		Week 1 (Unsch.)	11MAY2000	No		
		Week 1	15MAY2000	Yes	15MAY2000	8:48
		Week 2	22MAY2000	Yes	22MAY2000	7:10
		Week 3	29MAY2000	Yes	29MAY2000	6:45
		Week 3 (Unsch.)	29MAY2000	No		
		Week 3 (Unsch.)	31MAY2000	No		
		Week 4	06JUN2000	Yes	06JUN2000	14:25
		Week 5	12JUN2000	Yes	12JUN2000	11:00
		Week 6	21JUN2000	Yes	21JUN2000	6:33
		Week 7	26JUN2000	Yes	26JUN2000	13:58
		Week 7 (Unsch.)	26JUN2000	No		
		Week 8 (Fol. Up)	03JUL2000	No		
		Week 8 (Fol. Up)	05JUL2000	No		
		Week 9 (Fol. Up)	10JUL2000	No		
		Week 10 (Fol. Up)	17JUL2000	No		
		Week 11 (Fol. Up)	24JUL2000	No		
030.001.00076	IPP	Screening	11APR2000	No		
		Screening (Unsch.)	26APR2000	No		
		Clearance (Day -5)	03MAY2000	Yes	03MAY2000	
		Clearance (Day -4)	04MAY2000	Yes	04MAY2000	

*Popn=Population (I=Intent-to-Treat, PP=Per Protocol)
Tafenoquine = Tafenoquine 200mg, Mefloquine = Mefloquine 250mg

SB252263 - 030

Listing B.02

Visit And Dosing Dates

Treatment : Placebo

Subject Number	Popn*	Visit	Visit Date	Dose Taken ?	Date of Dose	Time of Dose
030.001.00076	IPP	Clearance (Day -3)	05MAY2000	Yes	05MAY2000	
		Clearance (Unsch.)	06MAY2000	No		
		Load Dose 1	08MAY2000	Yes	08MAY2000	10:45
		Load Dose 2	09MAY2000	Yes	09MAY2000	8:06
		Load Dose 3	10MAY2000	Yes	10MAY2000	9:35
		Week 1	15MAY2000	Yes	15MAY2000	9:44
		Week 1 (Unsch.)	17MAY2000	No		
		Week 2	22MAY2000	Yes	22MAY2000	8:20
		Week 3	29MAY2000	Yes	29MAY2000	7:38
		Week 4	05JUN2000	Yes	05JUN2000	8:20
		Week 5	12JUN2000	Yes	12JUN2000	8:23
		Week 6 (Fol. Up)	19JUN2000	No		
		Week 7 (Fol. Up)	26JUN2000	No		
		Week 7 (Unsch. Fol. Up)	26JUN2000	No		
		Week 8 (Fol. Up)	03JUL2000	No		
		Week 9 (Fol. Up)	10JUL2000	No		
		Week 9 (Unsch. Fol. Up)	10JUL2000	No		
030.001.00078	IPP	Screening	12APR2000	No		
		Clearance (Day -5)	03MAY2000	Yes	03MAY2000	
		Clearance (Day -4)	04MAY2000	Yes	04MAY2000	
		Clearance (Day -3)	05MAY2000	Yes	05MAY2000	
		Clearance (Unsch.)	06MAY2000	No		
		Load Dose 1	08MAY2000	Yes	08MAY2000	10:12
		Load Dose 2	09MAY2000	Yes	09MAY2000	6:55
		Load Dose 3	10MAY2000	Yes	10MAY2000	8:22
		Week 1 (Unsch.)	11MAY2000	No		
		Week 1	15MAY2000	Yes	15MAY2000	8:25
		Week 2	22MAY2000	Yes	22MAY2000	7:39
		Week 2 (Unsch.)	22MAY2000	No		
		Week 3	29MAY2000	Yes	29MAY2000	8:00
		Week 4	05JUN2000	Yes	05JUN2000	10:50
		Week 5	12JUN2000	Yes	12JUN2000	7:38
		Week 5 (Unsch.)	12JUN2000	No		
		Week 6	19JUN2000	Yes	19JUN2000	8:10
		Week 7 (Fol. Up)	26JUN2000	No		
		Week 7 (Unsch. Fol. Up)	29JUN2000	No		
		Week 8 (Fol. Up)	03JUL2000	No		
		Week 8 (Fol. Up)	04JUL2000	No		
		Week 9 (Fol. Up)	10JUL2000	No		
		Week 9 (Unsch. Fol. Up)	10JUL2000	No		

*Popn=Population (I=Intent-to-Treat, PP=Per Protocol)
Tafenoquine = Tafenoquine 200mg, Mefloquine = Mefloquine 250mg

SB252263 - 030

Listing B.02

Visit And Dosing Dates

Treatment : Placebo

Subject Number	Popn*	Visit	Visit Date	Dose Taken ?	Date of Dose	Time of Dose
030.001.00080	IPP	Screening	12APR2000	No		
		Clearance (Day -5)	03MAY2000	Yes	03MAY2000	
		Clearance (Day -4)	04MAY2000	Yes	04MAY2000	
		Clearance (Day -3)	05MAY2000	Yes	05MAY2000	
		Load Dose 1	08MAY2000	Yes	08MAY2000	10:35
		Load Dose 2	09MAY2000	Yes	09MAY2000	13:30
		Load Dose 3	10MAY2000	Yes	10MAY2000	9:40
		Week 1 (Unsch.)	14MAY2000	No		
		Week 1	15MAY2000	Yes	15MAY2000	11:24
		Week 2	22MAY2000	Yes	22MAY2000	8:58
		Week 3	29MAY2000	Yes	29MAY2000	9:35
		Week 4	05JUN2000	Yes	05JUN2000	14:23
		Week 5	12JUN2000	Yes	12JUN2000	8:40
		Week 6	19JUN2000	Yes	19JUN2000	12:55
		Week 7	26JUN2000	Yes	26JUN2000	8:40
		Week 8	03JUL2000	Yes	03JUL2000	7:58
		Week 9	12JUL2000	Yes	12JUL2000	9:40
		Week 10	17JUL2000	Yes	17JUL2000	9:45
		Week 11	24JUL2000	Yes	24JUL2000	9:45
		Week 12	31JUL2000	Yes	31JUL2000	8:45
		Week 13	07AUG2000	Yes	07AUG2000	9:58
		Week 14	14AUG2000	Yes	14AUG2000	9:50
		Week 15 (Fol. Up)	21AUG2000	No		
		Week 16 (Fol. Up)	29AUG2000	No		
		Week 16 (Unsch. Fol. Up)	31AUG2000	No		
		Week 17 (Fol. Up)	04SEP2000	No		
		Week 17 (Unsch. Fol. Up)	06SEP2000	No		
030.001.00081	IPP	Screening	11APR2000	No		
		Screening (Unsch.)	20APR2000	No		
		Clearance (Day -5)	03MAY2000	Yes	03MAY2000	
		Clearance (Day -4)	04MAY2000	Yes	04MAY2000	
		Clearance (Day -3)	05MAY2000	Yes	05MAY2000	
		Load Dose 1	08MAY2000	Yes	08MAY2000	10:38
		Load Dose 2	09MAY2000	Yes	09MAY2000	7:32
		Load Dose 3	10MAY2000	Yes	10MAY2000	12:48
		Week 1	16MAY2000	Yes	16MAY2000	11:10
		Week 1 (Unsch.)	17MAY2000	No		
		Week 2	22MAY2000	Yes	22MAY2000	8:55
		Week 3	29MAY2000	Yes	29MAY2000	8:04
		Week 4	05JUN2000	Yes	05JUN2000	6:50
		Week 5	13JUN2000	Yes	13JUN2000	9:40
		Week 6 (Fol. Up)	19JUN2000	No		

*Popn=Population (I=Intent-to-Treat, PP=Per Protocol)
Tafenoquine = Tafenoquine 200mg, Mefloquine = Mefloquine 250mg

SB252263 - 030

Listing B.02

Visit And Dosing Dates

Treatment : Placebo

Subject Number	Popn*	Visit	Visit Date	Dose Taken ?	Date of Dose	Time of Dose
030.001.00081	IPP	Week 7 (Fol. Up)	26JUN2000	No		
		Week 7 (Unsch. Fol. Up)	26JUN2000	No		
		Week 8 (Fol. Up)	03JUL2000	No		
		Week 9 (Fol. Up)	10JUL2000	No		
030.001.00087	I	Screening	14APR2000	No		
		Screening (Unsch.)	23APR2000	No		
		Clearance (Day -5)	03MAY2000	Yes	03MAY2000	
		Clearance (Day -4)	04MAY2000	Yes	04MAY2000	
		Clearance (Day -3)	05MAY2000	Yes	05MAY2000	
		Clearance (Unsch.)	06MAY2000	No		
		Load Dose 1	08MAY2000	Yes	08MAY2000	11:20
		Load Dose 2	09MAY2000	Yes	09MAY2000	7:55
		Load Dose 3	10MAY2000	Yes	10MAY2000	8:47
		Week 1	15MAY2000	Yes	15MAY2000	8:50
		Week 2 (Unsch. Fol. Up)	19MAY2000	No		
		Week 2 (Unsch. Fol. Up)	20MAY2000	No		
		Week 2 (Unsch. Fol. Up)	21MAY2000	No		
		Week 2 (Unsch. Fol. Up)	22MAY2000	No		
		Week 2 (Fol. Up)	24MAY2000	No		
		Week 3 (Unsch. Fol. Up)	28MAY2000	No		
		Week 5 (Fol. Up)	11JUN2000	No		
		Week 5 (Fol. Up)	12JUN2000	No		
030.001.00088	IPP	Screening	14APR2000	No		
		Clearance (Day -5)	03MAY2000	Yes	03MAY2000	
		Clearance (Day -4)	04MAY2000	Yes	04MAY2000	
		Clearance (Day -3)	05MAY2000	Yes	05MAY2000	
		Load Dose 1	08MAY2000	Yes	08MAY2000	11:25
		Load Dose 2	09MAY2000	Yes	09MAY2000	7:45
		Load Dose 3	10MAY2000	Yes	10MAY2000	8:38
		Week 1	15MAY2000	Yes	15MAY2000	10:20
		Week 2	22MAY2000	Yes	22MAY2000	12:00
		Week 3	29MAY2000	Yes	29MAY2000	11:05
		Week 4	06JUN2000	Yes	06JUN2000	15:17
		Week 5	12JUN2000	Yes	12JUN2000	9:20
		Week 6 (Fol. Up)	19JUN2000	No		
		Week 7 (Fol. Up)	26JUN2000	No		
		Week 8 (Fol. Up)	03JUL2000	No		
030.001.00090	IPP	Screening	12APR2000	No		

*Popn=Population (I=Intent-to-Treat, PP=Per Protocol)
Tafenoquine = Tafenoquine 200mg, Mefloquine = Mefloquine 250mg

SB252263 - 030

Listing B.02

Visit And Dosing Dates

Treatment : Placebo

Subject Number	Popn*	Visit	Visit Date	Dose Taken ?	Date of Dose	Time of Dose
030.001.00090	IPP	Screening (Unsch.)	18APR2000	No		
		Screening (Unsch.)	29APR2000	No		
		Clearance (Day -5)	03MAY2000	Yes	03MAY2000	
		Clearance (Day -4)	04MAY2000	Yes	04MAY2000	
		Clearance (Day -3)	05MAY2000	Yes	05MAY2000	
		Load Dose 1	08MAY2000	Yes	08MAY2000	12:16
		Load Dose 2	09MAY2000	Yes	09MAY2000	6:39
		Load Dose 3	10MAY2000	Yes	10MAY2000	19:49
		Week 1	15MAY2000	Yes	15MAY2000	7:00
		Week 2	22MAY2000	Yes	22MAY2000	10:50
		Week 3	29MAY2000	Yes	29MAY2000	7:00
		Week 3 (Unsch.)	30MAY2000	No		
		Week 4	04JUN2000	Yes	04JUN2000	9:25
		Week 5	12JUN2000	Yes	12JUN2000	10:29
		Week 5 (Unsch.)	14JUN2000	No		
		Week 6	19JUN2000	Yes	19JUN2000	6:08
		Week 6 (Unsch.)	21JUN2000	No		
		Week 7	26JUN2000	Yes	26JUN2000	6:35
		Week 8	03JUL2000	Yes	03JUL2000	6:30
		Week 9	10JUL2000	Yes	10JUL2000	6:45
		Week 10	17JUL2000	Yes	17JUL2000	6:50
		Week 11	25JUL2000	Yes	25JUL2000	15:20
		Week 12 (Fol. Up)	31JUL2000	No		
		Week 14 (Fol. Up)	15AUG2000	No		
		Week 15 (Fol. Up)	21AUG2000	No		
030.001.00092	IPP	Screening	12APR2000	No		
		Screening (Unsch.)	18APR2000	No		
		Clearance (Day -5)	03MAY2000	Yes	03MAY2000	
		Clearance (Day -4)	04MAY2000	Yes	04MAY2000	
		Clearance (Day -3)	05MAY2000	Yes	05MAY2000	
		Clearance (Unsch.)	03MAY2000	No		
		Load Dose 1	08MAY2000	Yes	08MAY2000	12:32
		Load Dose 2	09MAY2000	Yes	09MAY2000	9:20
		Load Dose 3	10MAY2000	Yes	10MAY2000	14:55
		Week 1	15MAY2000	Yes	15MAY2000	7:45
		Week 2	22MAY2000	Yes	22MAY2000	8:20
		Week 3	29MAY2000	Yes	29MAY2000	9:00
		Week 4	05JUN2000	Yes	05JUN2000	12:05
		Week 4 (Unsch.)	07JUN2000	No		
		Week 5	12JUN2000	Yes	12JUN2000	10:05
		Week 6	19JUN2000	Yes	19JUN2000	8:20
		Week 6 (Unsch.)	22JUN2000	No		

*Popn=Population (I=Intent-to-Treat, PP=Per Protocol)
Tafenoquine = Tafenoquine 200mg, Mefloquine = Mefloquine 250mg

SB252263 - 030

Listing B.02

Visit And Dosing Dates

Treatment : Placebo

Subject Number	Popn*	Visit	Visit Date	Dose Taken ?	Date of Dose	Time of Dose
030.001.00092	IPP	Week 7	26JUN2000	Yes	26JUN2000	11:20
		Week 8	03JUL2000	Yes	03JUL2000	11:30
		Week 9 (Fol. Up)	10JUL2000	No		
		Week 9 (Unsch. Fol. Up)	11JUL2000	No		
		Week 10 (Fol. Up)	18JUL2000	No		
		Week 11 (Fol. Up)	24JUL2000	No		
		Week 12 (Fol. Up)	02AUG2000	No		
030.001.00101	IPP	Screening	14APR2000	No		
		Screening (Unsch.)	25APR2000	No		
		Screening (Unsch.)	28APR2000	No		
		Clearance (Day -5)	03MAY2000	Yes	03MAY2000	
		Clearance (Day -4)	04MAY2000	Yes	04MAY2000	
		Clearance (Day -3)	05MAY2000	Yes	05MAY2000	
		Load Dose 1	08MAY2000	Yes	08MAY2000	14:01
		Load Dose 2	09MAY2000	Yes	09MAY2000	8:19
		Load Dose 3	10MAY2000	Yes	10MAY2000	8:19
		Week 1	15MAY2000	Yes	15MAY2000	8:26
		Week 2	22MAY2000	Yes	22MAY2000	7:59
		Week 2 (Unsch.)	24MAY2000	No		
		Week 2 (Unsch.)	25MAY2000	No		
		Week 3 (Unsch.)	26MAY2000	No		
		Week 3 (Unsch.)	28MAY2000	No		
		Week 3	29MAY2000	Yes	29MAY2000	7:55
		Week 4	05JUN2000	Yes	05JUN2000	11:50
		Week 5	12JUN2000	Yes	12JUN2000	7:49
		Week 6	19JUN2000	Yes	19JUN2000	7:13
		Week 7	26JUN2000	Yes	26JUN2000	8:10
		Week 8	03JUL2000	Yes	03JUL2000	6:38
		Week 9 (Fol. Up)	10JUL2000	No		
		Week 9 (Unsch. Fol. Up)	11JUL2000	No		
		Week 10 (Unsch. Fol. Up)	14JUL2000	No		
		Week 10 (Fol. Up)	17JUL2000	No		
		Week 11 (Fol. Up)	24JUL2000	No		
		Week 12 (Fol. Up)	01AUG2000	No		
030.001.00103	IPP	Screening	11APR2000	No		
		Clearance (Day -5)	03MAY2000	Yes	03MAY2000	
		Clearance (Day -4)	04MAY2000	Yes	04MAY2000	
		Clearance (Day -3)	05MAY2000	Yes	05MAY2000	
		Load Dose 1	08MAY2000	Yes	08MAY2000	13:59
		Load Dose 2	09MAY2000	Yes	09MAY2000	7:00

*Popn=Population (I=Intent-to-Treat, PP=Per Protocol)
Tafenoquine = Tafenoquine 200mg, Mefloquine = Mefloquine 250mg

SB252263 - 030

Listing B.02

Visit And Dosing Dates

Treatment : Placebo

Subject Number	Popn*	Visit	Visit Date	Dose Taken ?	Date of Dose	Time of Dose
030.001.00103	IPP	Load Dose 3	10MAY2000	Yes	10MAY2000	7:49
		Week 1	15MAY2000	Yes	15MAY2000	7:20
		Week 2 (Unsch.)	19MAY2000	No		
		Week 2 (Unsch.)	20MAY2000	No		
		Week 2	22MAY2000	Yes	22MAY2000	7:09
		Week 3	29MAY2000	Yes	29MAY2000	6:53
		Week 4	06JUN2000	Yes	06JUN2000	10:46
		Week 5	12JUN2000	Yes	12JUN2000	6:49
		Week 6	19JUN2000	Yes	19JUN2000	7:16
		Week 7 (Unsch. Fol. Up)	23JUN2000	No		
		Week 7 (Fol. Up)	26JUN2000	No		
		Week 8 (Fol. Up)	06JUL2000	No		
		Week 9 (Fol. Up)	11JUL2000	No		
		Week 10 (Fol. Up)	17JUL2000	No		
030.001.00106	IPP	Screening	10APR2000	No		
		Clearance (Day -5)	03MAY2000	Yes	03MAY2000	
		Clearance (Day -4)	04MAY2000	Yes	04MAY2000	
		Clearance (Day -3)	05MAY2000	Yes	05MAY2000	
		Load Dose 1	08MAY2000	Yes	08MAY2000	14:34
		Load Dose 2	09MAY2000	Yes	09MAY2000	12:00
		Load Dose 3	10MAY2000	Yes	10MAY2000	12:55
		Week 1	15MAY2000	Yes	15MAY2000	11:30
		Week 2	24MAY2000	No		
		Week 3	28MAY2000	Yes	28MAY2000	13:15
		Week 3 (Unsch.)	28MAY2000	No		
		Week 4	07JUN2000	Yes	07JUN2000	18:46
		Week 5 (Unsch.)	10JUN2000	No		
		Week 5	13JUN2000	Yes	13JUN2000	16:20
		Week 6	19JUN2000	Yes	19JUN2000	18:05
		Week 8	02JUL2000	Yes	02JUL2000	
		Week 8 (Fol. Up)	04JUL2000	No		
		Week 8 (Unsch. Fol. Up)	05JUL2000	No		
		Week 9 (Fol. Up)	13JUL2000	No		
		Week 10 (Fol. Up)	17JUL2000	No		
		Week 11 (Unsch. Fol. Up)	25JUL2000	No		
030.001.00108	IPP	Screening	14APR2000	No		
		Clearance (Day -5)	03MAY2000	Yes	03MAY2000	
		Clearance (Day -4)	04MAY2000	Yes	04MAY2000	
		Clearance (Day -3)	05MAY2000	Yes	05MAY2000	
		Load Dose 1	08MAY2000	Yes	08MAY2000	14:55

*Popn=Population (I=Intent-to-Treat, PP=Per Protocol)
Tafenoquine = Tafenoquine 200mg, Mefloquine = Mefloquine 250mg

SB252263 - 030

Listing B.02

Visit And Dosing Dates

Treatment : Placebo

Subject Number	Popn*	Visit	Visit Date	Dose Taken ?	Date of Dose	Time of Dose
030.001.00108	IPP	Load Dose 2	09MAY2000	Yes	09MAY2000	7:17
		Load Dose 3	10MAY2000	Yes	10MAY2000	8:39
		Week 1 (Unsch.)	11MAY2000	No		
		Week 1	15MAY2000	Yes	15MAY2000	11:15
		Week 1 (Unsch.)	18MAY2000	No		
		Week 2 (Unsch.)	19MAY2000	No		
		Week 2	22MAY2000	Yes	22MAY2000	9:40
		Week 3 (Fol. Up)	29MAY2000	No		
		Week 4 (Fol. Up)	07JUN2000	No		
		Week 5 (Fol. Up)	12JUN2000	No		
		Week 6 (Fol. Up)	19JUN2000	No		
030.001.00114	IPP	Screening	14APR2000	No		
		Screening (Unsch.)	28APR2000	No		
		Clearance (Day -5)	03MAY2000	Yes	03MAY2000	
		Clearance (Day -4)	04MAY2000	Yes	04MAY2000	
		Clearance (Day -3)	05MAY2000	Yes	05MAY2000	
		Load Dose 1	08MAY2000	Yes	08MAY2000	16:00
		Load Dose 2	09MAY2000	Yes	09MAY2000	6:49
		Load Dose 3	10MAY2000	Yes	10MAY2000	13:50
		Week 1	15MAY2000	Yes	15MAY2000	7:53
		Week 2	22MAY2000	Yes	22MAY2000	7:00
		Week 3	29MAY2000	Yes	29MAY2000	6:43
		Week 4	05JUN2000	Yes	05JUN2000	15:01
		Week 5	12JUN2000	Yes	12JUN2000	6:35
		Week 6	19JUN2000	Yes	19JUN2000	6:45
		Week 6 (Unsch.)	19JUN2000	No		
		Week 6 (Unsch.)	20JUN2000	No		
		Week 7	26JUN2000	Yes	26JUN2000	7:14
		Week 8	03JUL2000	Yes	03JUL2000	6:21
		Week 9 (Fol. Up)	10JUL2000	No		
		Week 10 (Fol. Up)	17JUL2000	No		
		Week 11 (Fol. Up)	24JUL2000	No		
		Week 11 (Unsch. Fol. Up)	27JUL2000	No		
		Week 12 (Fol. Up)	31JUL2000	No		
030.001.00115	IPP	Screening	17APR2000	No		
		Clearance (Day -5)	03MAY2000	Yes	03MAY2000	
		Clearance (Day -4)	04MAY2000	Yes	04MAY2000	
		Clearance (Day -3)	05MAY2000	Yes	05MAY2000	
		Load Dose 1	08MAY2000	Yes	08MAY2000	16:35
		Load Dose 2	09MAY2000	Yes	09MAY2000	7:26

*Popn=Population (I=Intent-to-Treat, PP=Per Protocol)
Tafenoquine = Tafenoquine 200mg, Mefloquine = Mefloquine 250mg

SB252263 - 030

Listing B.02

Visit And Dosing Dates

Treatment : Placebo

Subject Number	Popn*	Visit	Visit Date	Dose Taken ?	Date of Dose	Time of Dose
030.001.00115	IPP	Load Dose 3	10MAY2000	Yes	10MAY2000	8:29
		Week 1	15MAY2000	Yes	15MAY2000	8:50
		Week 2	22MAY2000	Yes	22MAY2000	10:04
		Week 3 (Unsch.)	26MAY2000	No		
		Week 3	29MAY2000	Yes	29MAY2000	12:07
		Week 4	05JUN2000	Yes	05JUN2000	16:10
		Week 5 (Fol. Up)	12JUN2000	No		
		Week 6 (Fol. Up)	19JUN2000	No		
		Week 7 (Fol. Up)	26JUN2000	No		
		Week 8 (Fol. Up)	03JUL2000	No		
030.001.00116	IPP	Screening	12APR2000	No		
		Clearance (Day -5)	03MAY2000	Yes	03MAY2000	
		Clearance (Day -4)	04MAY2000	Yes	04MAY2000	
		Clearance (Day -3)	05MAY2000	Yes	05MAY2000	
		Load Dose 1	08MAY2000	Yes	08MAY2000	16:23
		Load Dose 2	09MAY2000	Yes	09MAY2000	13:30
		Load Dose 3	10MAY2000	Yes	10MAY2000	16:10
		Week 1	15MAY2000	Yes	15MAY2000	7:45
		Week 1 (Unsch.)	15MAY2000	No		
		Week 2	22MAY2000	Yes	22MAY2000	7:00
		Week 3 (Unsch. Fol. Up)	26MAY2000	No		
		Week 3 (Fol. Up)	29MAY2000	No		
		Week 3 (Unsch. Fol. Up)	30MAY2000	No		
		Week 4 (Fol. Up)	05JUN2000	No		
		Week 5 (Fol. Up)	13JUN2000	No		
		Week 6 (Fol. Up)	20JUN2000	No		
		Week 6 (Unsch. Fol. Up)	20JUN2000	No		
030.001.00119	IPP	Screening	11APR2000	No		
		Screening (Unsch.)	01MAY2000	No		
		Clearance (Day -5)	04MAY2000	Yes	04MAY2000	
		Clearance (Day -4)	05MAY2000	Yes	05MAY2000	
		Clearance (Day -3)	06MAY2000	Yes	06MAY2000	
		Load Dose 1	09MAY2000	Yes	09MAY2000	6:40
		Load Dose 2	10MAY2000	Yes	10MAY2000	14:10
		Load Dose 3	11MAY2000	Yes	11MAY2000	17:27
		Week 1	15MAY2000	Yes	15MAY2000	13:07
		Week 2	23MAY2000	Yes	23MAY2000	12:58
		Week 3 (Fol. Up)	31MAY2000	No		
		Week 4 (Fol. Up)	07JUN2000	No		
		Week 4 (Unsch. Fol. Up)	07JUN2000	No		

*Popn=Population (I=Intent-to-Treat, PP=Per Protocol)
Tafenoquine = Tafenoquine 200mg, Mefloquine = Mefloquine 250mg

SB252263 - 030

Listing B.02

Visit And Dosing Dates

Treatment : Placebo

Subject Number	Popn*	Visit	Visit Date	Dose Taken ?	Date of Dose	Time of Dose
030.001.00119	IPP	Week 5 (Fol. Up)	12JUN2000	No		
		Week 6 (Fol. Up)	19JUN2000	No		
030.001.00124	IPP	Screening	11APR2000	No		
		Clearance (Day -5)	04MAY2000	Yes	04MAY2000	
		Clearance (Day -4)	05MAY2000	Yes	05MAY2000	
		Clearance (Day -3)	06MAY2000	Yes	06MAY2000	
		Load Dose 1	09MAY2000	Yes	09MAY2000	7:01
		Load Dose 2	10MAY2000	Yes	10MAY2000	7:45
		Load Dose 3	11MAY2000	Yes	11MAY2000	8:08
		Week 1	15MAY2000	Yes	15MAY2000	7:41
		Week 2	22MAY2000	Yes	22MAY2000	7:30
		Week 3	29MAY2000	Yes	29MAY2000	9:27
		Week 3 (Unsch.)	30MAY2000	No		
		Week 4	05JUN2000	Yes	05JUN2000	6:14
		Week 4 (Unsch. Fol. Up)	09JUN2000	No		
		Week 5 (Unsch. Fol. Up)	13JUN2000	No		
		Week 5 (Fol. Up)	14JUN2000	No		
		Week 6 (Unsch. Fol. Up)	18JUN2000	No		
		Week 6 (Fol. Up)	19JUN2000	No		
		Week 7 (Fol. Up)	26JUN2000	No		
		Week 8 (Fol. Up)	03JUL2000	No		
030.001.00125	IPP	Screening	10APR2000	No		
		Screening (Unsch.)	02MAY2000	No		
		Clearance (Day -5)	04MAY2000	Yes	04MAY2000	
		Clearance (Day -4)	05MAY2000	Yes	05MAY2000	
		Clearance (Day -3)	06MAY2000	Yes	06MAY2000	
		Load Dose 1	09MAY2000	Yes	09MAY2000	8:33
		Load Dose 2	10MAY2000	Yes	10MAY2000	8:35
		Load Dose 3	11MAY2000	Yes	11MAY2000	6:58
		Week 1	15MAY2000	Yes	15MAY2000	8:21
		Week 2 (Unsch.)	20MAY2000	No		
		Week 2 (Unsch.)	21MAY2000	No		
		Week 2	22MAY2000	Yes	22MAY2000	7:40
		Week 2 (Unsch.)	24MAY2000	No		
		Week 3	29MAY2000	Yes	29MAY2000	9:41
		Week 3 (Unsch.)	29MAY2000	No		
		Week 4	05JUN2000	Yes	05JUN2000	12:16
		Week 4 (Unsch.)	07JUN2000	No		
		Week 5	12JUN2000	Yes	12JUN2000	8:21
		Week 6	20JUN2000	Yes	20JUN2000	8:24

*Popn=Population (I=Intent-to-Treat, PP=Per Protocol)
Tafenoquine = Tafenoquine 200mg, Mefloquine = Mefloquine 250mg

SB252263 - 030

Listing B.02

Visit And Dosing Dates

Treatment : Placebo

Subject Number	Popn*	Visit	Visit Date	Dose Taken ?	Date of Dose	Time of Dose
030.001.00125	IPP	Week 7	26JUN2000	Yes	26JUN2000	12:47
		Week 8	03JUL2000	Yes	03JUL2000	6:55
		Week 8 (Fol. Up)	05JUL2000	No		
		Week 9 (Fol. Up)	10JUL2000	No		
		Week 9 (Unsch. Fol. Up)	11JUL2000	No		
		Week 9 (Unsch. Fol. Up)	13JUL2000	No		
		Week 10 (Fol. Up)	17JUL2000	No		
		Week 11 (Fol. Up)	24JUL2000	No		
		Week 11 (Unsch. Fol. Up)	26JUL2000	No		
030.001.00127	I	Screening	12APR2000	No		
		Clearance (Day -5)	04MAY2000	Yes	04MAY2000	
		Clearance (Day -4)	05MAY2000	Yes	05MAY2000	
		Clearance (Day -3)	06MAY2000	Yes	06MAY2000	
		Load Dose 1	09MAY2000	Yes	09MAY2000	7:25
		Load Dose 2	10MAY2000	Yes	10MAY2000	7:04
		Load Dose 3	11MAY2000	Yes	11MAY2000	6:34
		Week 1 (Unsch.)	14MAY2000	No		
		Week 1	16MAY2000	Yes	16MAY2000	6:20
		Week 2 (Unsch.)	20MAY2000	No		
		Week 2	24MAY2000	Yes	24MAY2000	11:00
		Week 2 (Unsch.)	24MAY2000	No		
		Week 3	29MAY2000	Yes	29MAY2000	6:25
		Week 3 (Unsch.)	29MAY2000	No		
		Week 4	08JUN2000	Yes	08JUN2000	17:14
		Week 5	11JUN2000	Yes	11JUN2000	16:36
030.001.00131	IPP	Screening	10APR2000	No		
		Clearance (Day -5)	04MAY2000	Yes	04MAY2000	
		Clearance (Day -4)	05MAY2000	Yes	05MAY2000	
		Clearance (Day -3)	06MAY2000	Yes	06MAY2000	
		Load Dose 1	09MAY2000	Yes	09MAY2000	7:22
		Load Dose 2	10MAY2000	Yes	10MAY2000	18:37
		Load Dose 3	11MAY2000	Yes	11MAY2000	7:19
		Week 1	15MAY2000	Yes	15MAY2000	10:02
		Week 1 (Unsch.)	19MAY2000	No		
		Week 2	22MAY2000	Yes	22MAY2000	9:38
		Week 3	29MAY2000	Yes	29MAY2000	16:00
		Week 4	06JUN2000	Yes	06JUN2000	11:00
		Week 4 (Unsch.)	07JUN2000	No		
		Week 5	12JUN2000	Yes	12JUN2000	6:12
		Week 6	19JUN2000	Yes	19JUN2000	6:26

*Popn=Population (I=Intent-to-Treat, PP=Per Protocol)
Tafenoquine = Tafenoquine 200mg, Mefloquine = Mefloquine 250mg

SB252263 - 030

Listing B.02

Visit And Dosing Dates

Treatment : Placebo

Subject Number	Popn*	Visit	Visit Date	Dose Taken ?	Date of Dose	Time of Dose
030.001.00131	IPP	Week 6 (Unsch.)	21JUN2000	No		
		Week 7	26JUN2000	Yes	26JUN2000	6:50
		Week 8 (Fol. Up)	03JUL2000	No		
		Week 8 (Unsch. Fol. Up)	07JUL2000	No		
		Week 9 (Fol. Up)	10JUL2000	No		
		Week 10 (Fol. Up)	17JUL2000	No		
		Week 11 (Unsch. Fol. Up)	24JUL2000	No		
030.001.00136	IPP	Screening	11APR2000	No		
		Clearance (Day -5)	04MAY2000	Yes	04MAY2000	
		Clearance (Day -4)	05MAY2000	Yes	05MAY2000	
		Clearance (Day -3)	06MAY2000	Yes	06MAY2000	
		Load Dose 1	09MAY2000	Yes	09MAY2000	8:25
		Load Dose 2	10MAY2000	Yes	10MAY2000	10:04
		Load Dose 3	11MAY2000	Yes	11MAY2000	7:16
		Week 1	15MAY2000	Yes	15MAY2000	10:20
		Week 1 (Unsch.)	16MAY2000	No		
		Week 2	22MAY2000	Yes	22MAY2000	8:51
		Week 2 (Unsch.)	26MAY2000	No		
		Week 3	29MAY2000	Yes	29MAY2000	8:29
		Week 3 (Unsch.)	29MAY2000	No		
		Week 4	05JUN2000	Yes	05JUN2000	9:10
		Week 5 (Fol. Up)	12JUN2000	No		
		Week 5 (Unsch. Fol. Up)	13JUN2000	No		
		Week 6 (Fol. Up)	19JUN2000	No		
		Week 6 (Unsch. Fol. Up)	22JUN2000	No		
		Week 7 (Fol. Up)	26JUN2000	No		
		Week 7 (Unsch. Fol. Up)	26JUN2000	No		
		Week 8 (Fol. Up)	03JUL2000	No		
030.001.00137	IPP	Screening	11APR2000	No		
		Clearance (Day -5)	04MAY2000	Yes	04MAY2000	
		Clearance (Day -4)	05MAY2000	Yes	05MAY2000	
		Clearance (Day -3)	06MAY2000	Yes	06MAY2000	
		Load Dose 1	09MAY2000	Yes	09MAY2000	8:03
		Load Dose 2	10MAY2000	Yes	10MAY2000	14:15
		Load Dose 3	11MAY2000	Yes	11MAY2000	8:10
		Week 1	15MAY2000	Yes	15MAY2000	13:47
		Week 2	22MAY2000	Yes	22MAY2000	13:21
		Week 3	29MAY2000	Yes	29MAY2000	7:15
		Week 4	04JUN2000	Yes	04JUN2000	8:40
		Week 5	12JUN2000	Yes	12JUN2000	7:20

*Popn=Population (I=Intent-to-Treat, PP=Per Protocol)
Tafenoquine = Tafenoquine 200mg, Mefloquine = Mefloquine 250mg

SB252263 - 030

Listing B.02

Visit And Dosing Dates

Treatment : Placebo

Subject Number	Popn*	Visit	Visit Date	Dose Taken ?	Date of Dose	Time of Dose
030.001.00137	IPP	Week 6	19JUN2000	Yes	19JUN2000	7:15
		Week 7	26JUN2000	Yes	26JUN2000	7:25
		Week 7 (Unsch.)	30JUN2000	No		
		Week 8	03JUL2000	Yes	03JUL2000	7:34
		Week 9 (Unsch. Fol. Up)	08JUL2000	No		
		Week 9 (Fol. Up)	10JUL2000	No		
		Week 9 (Unsch. Fol. Up)	12JUL2000	No		
		Week 10 (Fol. Up)	17JUL2000	No		
		Week 11 (Fol. Up)	24JUL2000	No		
030.001.00140	IPP	Screening	10APR2000	No		
		Clearance (Day -5)	04MAY2000	Yes	04MAY2000	
		Clearance (Day -4)	05MAY2000	Yes	05MAY2000	
		Clearance (Day -3)	06MAY2000	Yes	06MAY2000	
		Load Dose 1	09MAY2000	Yes	09MAY2000	8:10
		Load Dose 2	10MAY2000	Yes	10MAY2000	8:00
		Load Dose 3	11MAY2000	Yes	11MAY2000	7:20
		Week 1	15MAY2000	Yes	15MAY2000	8:25
		Week 2	22MAY2000	Yes	22MAY2000	6:15
		Week 2 (Unsch.)	22MAY2000	No		
		Week 3	28MAY2000	Yes	28MAY2000	13:21
		Week 4	07JUN2000	Yes	07JUN2000	18:31
		Week 5	12JUN2000	Yes	12JUN2000	7:55
		Week 6	19JUN2000	Yes	19JUN2000	7:37
		Week 6 (Unsch. Fol. Up)	23JUN2000	No		
		Week 7 (Fol. Up)	26JUN2000	No		
		Week 8 (Fol. Up)	03JUL2000	No		
		Week 9 (Fol. Up)	10JUL2000	No		
		Week 10 (Fol. Up)	17JUL2000	No		
030.001.00141	IPP	Screening	11APR2000	No		
		Screening (Unsch.)	18APR2000	No		
		Clearance (Day -5)	04MAY2000	Yes	04MAY2000	
		Clearance (Day -4)	05MAY2000	Yes	05MAY2000	
		Clearance (Day -3)	06MAY2000	Yes	06MAY2000	
		Load Dose 1	09MAY2000	Yes	09MAY2000	9:04
		Load Dose 2	10MAY2000	Yes	10MAY2000	13:10
		Load Dose 3	11MAY2000	Yes	11MAY2000	7:35
		Week 1	15MAY2000	Yes	15MAY2000	7:35
		Week 2	22MAY2000	Yes	22MAY2000	7:20
		Week 3	29MAY2000	Yes	29MAY2000	8:30
		Week 4	06JUN2000	Yes	06JUN2000	8:05

*Popn=Population (I=Intent-to-Treat, PP=Per Protocol)
Tafenoquine = Tafenoquine 200mg, Mefloquine = Mefloquine 250mg

SB252263 - 030

Listing B.02

Visit And Dosing Dates

Treatment : Placebo

Subject Number	Popn*	Visit	Visit Date	Dose Taken ?	Date of Dose	Time of Dose
030.001.00141	IPP	Week 5	14JUN2000	Yes	14JUN2000	12:58
		Week 6	19JUN2000	Yes	19JUN2000	7:35
		Week 7	26JUN2000	Yes	26JUN2000	7:45
		Week 8 (Fol. Up)	03JUL2000	No		
		Week 9 (Fol. Up)	10JUL2000	No		
		Week 10 (Fol. Up)	17JUL2000	No		
030.001.00145	IPP	Screening	18APR2000	No		
		Clearance (Day -5)	04MAY2000	Yes	04MAY2000	
		Clearance (Day -4)	05MAY2000	Yes	05MAY2000	
		Clearance (Day -3)	06MAY2000	Yes	06MAY2000	
		Clearance (Unsch.)	04MAY2000	No		
		Load Dose 1	09MAY2000	Yes	09MAY2000	9:00
		Load Dose 2	10MAY2000	Yes	10MAY2000	10:30
		Load Dose 3	11MAY2000	Yes	11MAY2000	8:00
		Week 1	15MAY2000	Yes	15MAY2000	8:00
		Week 2	23MAY2000	Yes	23MAY2000	12:20
		Week 3	29MAY2000	Yes	29MAY2000	7:00
		Week 4	06JUN2000	Yes	06JUN2000	13:50
		Week 5	13JUN2000	Yes	13JUN2000	10:00
		Week 6 (Fol. Up)	23JUN2000	No		
		Week 7 (Fol. Up)	26JUN2000	No		
		Week 8 (Unsch. Fol. Up)	01JUL2000	No		
		Week 8 (Fol. Up)	04JUL2000	No		
		Week 9 (Fol. Up)	10JUL2000	No		
		Week 9 (Unsch. Fol. Up)	11JUL2000	No		
030.001.00151	IPP	Screening	11APR2000	No		
		Clearance (Day -5)	04MAY2000	Yes	04MAY2000	
		Clearance (Day -4)	05MAY2000	Yes	05MAY2000	
		Clearance (Day -3)	06MAY2000	Yes	06MAY2000	
		Load Dose 1	09MAY2000	Yes	09MAY2000	9:15
		Load Dose 2	10MAY2000	Yes	10MAY2000	8:34
		Load Dose 3	11MAY2000	Yes	11MAY2000	8:18
		Week 1	15MAY2000	Yes	15MAY2000	7:12
		Week 2	22MAY2000	Yes	22MAY2000	6:35
		Week 2 (Unsch.)	23MAY2000	No		
		Week 3	29MAY2000	Yes	29MAY2000	6:52
		Week 3 (Unsch.)	30MAY2000	No		
		Week 4	04JUN2000	Yes	04JUN2000	8:26
		Week 5	12JUN2000	Yes	12JUN2000	8:00
		Week 6	19JUN2000	Yes	19JUN2000	9:30

*Popn=Population (I=Intent-to-Treat, PP=Per Protocol)
Tafenoquine = Tafenoquine 200mg, Mefloquine = Mefloquine 250mg

SB252263 - 030

Listing B.02

Visit And Dosing Dates

Treatment : Placebo

Subject Number	Popn*	Visit	Visit Date	Dose Taken ?	Date of Dose	Time of Dose
030.001.00151	IPP	Week 7	26JUN2000	Yes	26JUN2000	6:20
		Week 8	03JUL2000	Yes	03JUL2000	6:40
		Week 9	10JUL2000	Yes	10JUL2000	7:00
		Week 10 (Fol. Up)	17JUL2000	No		
		Week 10 (Unsch. Fol. Up)	19JUL2000	No		
		Week 11 (Fol. Up)	24JUL2000	No		
		Week 12 (Fol. Up)	31JUL2000	No		
030.001.00152	IPP	Screening	13APR2000	No		
		Clearance (Day -5)	04MAY2000	Yes	04MAY2000	
		Clearance (Day -4)	05MAY2000	Yes	05MAY2000	
		Clearance (Day -3)	06MAY2000	Yes	06MAY2000	
		Load Dose 1	09MAY2000	Yes	09MAY2000	9:17
		Load Dose 2	10MAY2000	Yes	10MAY2000	7:15
		Load Dose 3	11MAY2000	Yes	11MAY2000	7:25
		Week 1	15MAY2000	Yes	15MAY2000	13:05
		Week 2 (Unsch.)	20MAY2000	No		
		Week 2	22MAY2000	Yes	22MAY2000	8:15
		Week 2 (Unsch.)	26MAY2000	No		
		Week 3 (Unsch.)	27MAY2000	No		
		Week 3	29MAY2000	Yes	29MAY2000	8:06
		Week 4	05JUN2000	Yes	05JUN2000	13:49
		Week 5	12JUN2000	Yes	12JUN2000	7:27
		Week 6	19JUN2000	Yes	19JUN2000	7:11
		Week 7	26JUN2000	Yes	26JUN2000	8:39
		Week 7 (Unsch. Fol. Up)	28JUN2000	No		
		Week 8 (Unsch. Fol. Up)	06JUL2000	No		
		Week 9 (Fol. Up)	10JUL2000	No		
		Week 9 (Unsch. Fol. Up)	13JUL2000	No		
		Week 10 (Fol. Up)	17JUL2000	No		
		Week 11 (Fol. Up)	24JUL2000	No		
030.001.00154	IPP	Screening	13APR2000	No		
		Clearance (Day -5)	04MAY2000	Yes	04MAY2000	
		Clearance (Day -4)	05MAY2000	Yes	05MAY2000	
		Clearance (Day -3)	06MAY2000	Yes	06MAY2000	
		Load Dose 1	09MAY2000	Yes	09MAY2000	10:00
		Load Dose 2	10MAY2000	Yes	10MAY2000	7:38
		Load Dose 3	11MAY2000	Yes	11MAY2000	7:25
		Week 1	15MAY2000	Yes	15MAY2000	8:43
		Week 2	22MAY2000	Yes	22MAY2000	8:20
		Week 3	29MAY2000	Yes	29MAY2000	8:14

*Popn=Population (I=Intent-to-Treat, PP=Per Protocol)
Tafenoquine = Tafenoquine 200mg, Mefloquine = Mefloquine 250mg

SB252263 - 030

Listing B.02

Visit And Dosing Dates

Treatment : Placebo

Subject Number	Popn*	Visit	Visit Date	Dose Taken ?	Date of Dose	Time of Dose
030.001.00154	IPP	Week 4	07JUN2000	Yes	07JUN2000	9:35
		Week 5	12JUN2000	Yes	12JUN2000	7:30
		Week 6 (Fol. Up)	19JUN2000	No		
		Week 7 (Fol. Up)	26JUN2000	No		
		Week 7 (Unsch. Fol. Up)	27JUN2000	No		
		Week 9 (Fol. Up)	09JUL2000	No		
		Week 9 (Fol. Up)	12JUL2000	No		
030.001.00160	I	Screening	11APR2000	No		
		Clearance (Day -5)	04MAY2000	Yes	04MAY2000	
		Clearance (Day -4)	05MAY2000	Yes	05MAY2000	
		Clearance (Day -3)	06MAY2000	Yes	06MAY2000	
		Load Dose 1	09MAY2000	Yes	09MAY2000	9:16
		Load Dose 2	10MAY2000	Yes	10MAY2000	8:22
		Load Dose 3	11MAY2000	Yes	11MAY2000	7:11
		Week 1 (Unsch.)	12MAY2000	No		
		Week 1	15MAY2000	Yes	15MAY2000	8:00
		Week 2	22MAY2000	Yes	22MAY2000	7:45
		Week 2 (Unsch.)	26MAY2000	No		
		Week 3	29MAY2000	Yes	29MAY2000	10:20
		Week 3 (Unsch.)	31MAY2000	No		
		Week 4	05JUN2000	Yes	05JUN2000	10:07
		Week 4 (Unsch.)	07JUN2000	No		
		Week 5	12JUN2000	Yes	12JUN2000	6:31
		Week 6	19JUN2000	Yes	19JUN2000	6:30
		Week 7	26JUN2000	Yes	26JUN2000	10:30
		Week 8 (Fol. Up)	05JUL2000	No		
		Week 8 (Unsch. Fol. Up)	05JUL2000	No		
		Week 9 (Fol. Up)	10JUL2000	No		
		Week 10 (Fol. Up)	17JUL2000	No		
		Week 11 (Fol. Up)	25JUL2000	No		
		Week 11 (Unsch. Fol. Up)	26JUL2000	No		
030.001.00162	IPP	Screening	11APR2000	No		
		Clearance (Day -5)	04MAY2000	Yes	04MAY2000	
		Clearance (Day -4)	05MAY2000	Yes	05MAY2000	
		Clearance (Day -3)	06MAY2000	Yes	06MAY2000	
		Load Dose 1	09MAY2000	Yes	09MAY2000	9:55
		Load Dose 2	10MAY2000	Yes	10MAY2000	18:01
		Load Dose 3	11MAY2000	Yes	11MAY2000	19:14
		Week 1	16MAY2000	Yes	16MAY2000	12:58
		Week 2 (Unsch.)	21MAY2000	No		

*Popn=Population (I=Intent-to-Treat, PP=Per Protocol)
Tafenoquine = Tafenoquine 200mg, Mefloquine = Mefloquine 250mg

SB252263 - 030

Listing B.02

Visit And Dosing Dates

Treatment : Placebo

Subject Number	Popn*	Visit	Visit Date	Dose Taken ?	Date of Dose	Time of Dose
030.001.00162	IPP	Week 2	22MAY2000	Yes	22MAY2000	6:50
		Week 3	29MAY2000	Yes	29MAY2000	15:30
		Week 4	05JUN2000	Yes	05JUN2000	13:06
		Week 5	12JUN2000	Yes	12JUN2000	8:00
		Week 6	19JUN2000	Yes	19JUN2000	17:45
		Week 7	26JUN2000	Yes	26JUN2000	9:17
		Week 7 (Unsch.)	27JUN2000	No		
		Week 8	03JUL2000	Yes	03JUL2000	7:23
		Week 9 (Fol. Up)	10JUL2000	No		
		Week 10 (Fol. Up)	17JUL2000	No		
		Week 11 (Fol. Up)	25JUL2000	No		
		Week 12 (Fol. Up)	01AUG2000	No		
030.001.00164	IPP	Screening	14APR2000	No		
		Clearance (Day -5)	04MAY2000	Yes	04MAY2000	
		Clearance (Day -4)	05MAY2000	Yes	05MAY2000	
		Clearance (Day -3)	06MAY2000	Yes	06MAY2000	
		Clearance (Unsch.)	08MAY2000	No		
		Load Dose 1	09MAY2000	Yes	09MAY2000	9:46
		Load Dose 2	10MAY2000	Yes	10MAY2000	9:43
		Load Dose 3	11MAY2000	Yes	11MAY2000	16:40
		Week 1	15MAY2000	Yes	15MAY2000	13:40
		Week 2	22MAY2000	Yes	22MAY2000	6:53
		Week 2 (Unsch.)	23MAY2000	No		
		Week 3	29MAY2000	Yes	29MAY2000	9:28
		Week 4	05JUN2000	Yes	05JUN2000	11:15
		Week 5	12JUN2000	Yes	12JUN2000	8:06
		Week 6 (Fol. Up)	19JUN2000	No		
		Week 7 (Unsch. Fol. Up)	26JUN2000	No		
		Week 7 (Fol. Up)	27JUN2000	No		
		Week 8 (Fol. Up)	03JUL2000	No		
		Week 9 (Fol. Up)	10JUL2000	No		
030.001.00166	IPP	Screening	10APR2000	No		
		Clearance (Day -5)	04MAY2000	Yes	04MAY2000	
		Clearance (Day -4)	05MAY2000	Yes	05MAY2000	
		Clearance (Day -3)	06MAY2000	Yes	06MAY2000	
		Load Dose 1	09MAY2000	Yes	09MAY2000	9:49
		Load Dose 2	10MAY2000	Yes	10MAY2000	12:07
		Load Dose 3	11MAY2000	Yes	11MAY2000	10:49
		Week 1	15MAY2000	Yes	15MAY2000	6:51
		Week 1 (Unsch.)	15MAY2000	No		

*Popn=Population (I=Intent-to-Treat, PP=Per Protocol)
Tafenoquine = Tafenoquine 200mg, Mefloquine = Mefloquine 250mg

SB252263 - 030

Listing B.02

Visit And Dosing Dates

Treatment : Placebo

Subject Number	Popn*	Visit	Visit Date	Dose Taken ?	Date of Dose	Time of Dose
030.001.00166	IPP	Week 2	22MAY2000	Yes	22MAY2000	10:31
		Week 2 (Unsch.)	22MAY2000	No		
		Week 2 (Unsch.)	24MAY2000	No		
		Week 3	29MAY2000	Yes	29MAY2000	11:07
		Week 4	05JUN2000	Yes	05JUN2000	8:29
		Week 5	12JUN2000	Yes	12JUN2000	11:39
		Week 6	19JUN2000	Yes	19JUN2000	12:12
		Week 7	26JUN2000	Yes	26JUN2000	11:37
		Week 8	04JUL2000	Yes	04JUL2000	14:28
		Week 8	05JUL2000	No		
		Week 9	10JUL2000	Yes	10JUL2000	9:28
		Week 10	17JUL2000	Yes	17JUL2000	7:55
		Week 10 (Unsch.)	19JUL2000	No		
		Week 11	25JUL2000	Yes	25JUL2000	12:08
		Week 11 (Unsch.)	26JUL2000	No		
		Week 12	31JUL2000	Yes	31JUL2000	7:40
		Week 13	07AUG2000	Yes	07AUG2000	8:29
		Week 14	14AUG2000	Yes	14AUG2000	8:40
		Week 14 (Unsch.)	16AUG2000	No		
		Week 15	22AUG2000	Yes	22AUG2000	11:00
		Week 15 (Unsch.)	22AUG2000	No		
		Week 16	28AUG2000	Yes	28AUG2000	9:10
		Week 16	30AUG2000	No		
		Week 17	04SEP2000	Yes	04SEP2000	11:25
		Week 17 (Unsch.)	07SEP2000	No		
		Week 18	11SEP2000	Yes	11SEP2000	8:35
		Week 18 (Unsch.)	12SEP2000	No		
		Week 19	20SEP2000	Yes	20SEP2000	12:27
		Week 19 (Unsch.)	21SEP2000	No		
		Week 20	25SEP2000	Yes	25SEP2000	7:59
		Week 20 (Unsch.)	26SEP2000	No		
		Week 21	04OCT2000	Yes	04OCT2000	9:55
		Week 22	09OCT2000	Yes	09OCT2000	10:35
		Week 22 (Unsch.)	09OCT2000	No		
		Week 22 (Unsch.)	10OCT2000	No		
		Week 23	16OCT2000	Yes	16OCT2000	8:58
		Week 24	23OCT2000	Yes	23OCT2000	11:09
		Week 24 (Unsch. Fol. Up)	26OCT2000	No		
		Week 25 (Fol. Up)	30OCT2000	No		
		Week 25 (Unsch. Fol. Up)	30OCT2000	No		
		Week 26 (Unsch. Fol. Up)	06NOV2000	No		
		Week 26 (Fol. Up)	07NOV2000	No		
		Week 27 (Fol. Up)	13NOV2000	No		
		Week 27 (Unsch. Fol. Up)	16NOV2000	No		

*Popn=Population (I=Intent-to-Treat, PP=Per Protocol)
Tafenoquine = Tafenoquine 200mg, Mefloquine = Mefloquine 250mg

SB252263 - 030

Listing B.02

Visit And Dosing Dates

Treatment : Placebo

Subject Number	Popn*	Visit	Visit Date	Dose Taken ?	Date of Dose	Time of Dose
030.001.00166	IPP	Week 28 (Fol. Up)	20NOV2000	No		
030.001.00169	IPP	Screening	10APR2000	No		
		Clearance (Day -5)	04MAY2000	Yes	04MAY2000	
		Clearance (Day -4)	05MAY2000	Yes	05MAY2000	
		Clearance (Day -3)	06MAY2000	Yes	06MAY2000	
		Load Dose 1	09MAY2000	Yes	09MAY2000	10:42
		Load Dose 2	10MAY2000	Yes	10MAY2000	13:33
		Load Dose 3	11MAY2000	Yes	11MAY2000	6:20
		Week 1 (Unsch.)	14MAY2000	No		
		Week 1	15MAY2000	Yes	15MAY2000	7:05
		Week 1 (Unsch.)	19MAY2000	No		
		Week 2	22MAY2000	Yes	22MAY2000	7:48
		Week 3	29MAY2000	Yes	29MAY2000	6:25
		Week 3 (Unsch.)	30MAY2000	No		
		Week 4 (Unsch.)	03JUN2000	No		
		Week 4	05JUN2000	Yes	05JUN2000	10:20
		Week 5	12JUN2000	Yes	12JUN2000	10:16
		Week 5 (Unsch.)	13JUN2000	No		
		Week 6	19JUN2000	Yes	19JUN2000	9:05
		Week 6 (Unsch.)	20JUN2000	No		
		Week 7	26JUN2000	Yes	26JUN2000	8:47
		Week 8 (Fol. Up)	03JUL2000	No		
		Week 9 (Fol. Up)	10JUL2000	No		
		Week 9 (Unsch. Fol. Up)	13JUL2000	No		
		Week 10 (Fol. Up)	17JUL2000	No		
		Week 10 (Unsch. Fol. Up)	17JUL2000	No		
		Week 11 (Unsch. Fol. Up)	24JUL2000	No		
030.001.00172	IPP	Screening	10APR2000	No		
		Clearance (Day -5)	04MAY2000	Yes	04MAY2000	
		Clearance (Day -4)	05MAY2000	Yes	05MAY2000	
		Clearance (Day -3)	06MAY2000	Yes	06MAY2000	
		Load Dose 1	09MAY2000	Yes	09MAY2000	10:09
		Load Dose 2	10MAY2000	Yes	10MAY2000	6:20
		Load Dose 3	11MAY2000	Yes	11MAY2000	6:30
		Week 1	15MAY2000	Yes	15MAY2000	7:40
		Week 2	22MAY2000	Yes	22MAY2000	6:10
		Week 3	29MAY2000	Yes	29MAY2000	6:55
		Week 4 (Unsch.)	04JUN2000	No		
		Week 4	06JUN2000	Yes	06JUN2000	8:07
		Week 5	13JUN2000	Yes	13JUN2000	11:10

*Popn=Population (I=Intent-to-Treat, PP=Per Protocol)
Tafenoquine = Tafenoquine 200mg, Mefloquine = Mefloquine 250mg

SB252263 - 030

Listing B.02

Visit And Dosing Dates

Treatment : Placebo

Subject Number	Popn*	Visit	Visit Date	Dose Taken ?	Date of Dose	Time of Dose
030.001.00172	IPP	Week 6	19JUN2000	Yes	19JUN2000	6:50
		Week 7	26JUN2000	Yes	26JUN2000	6:20
		Week 8	03JUL2000	Yes	03JUL2000	6:30
		Week 9	11JUL2000	Yes	11JUL2000	9:35
		Week 10	17JUL2000	Yes	17JUL2000	6:25
		Week 11	24JUL2000	Yes	24JUL2000	7:05
		Week 12	31JUL2000	Yes	31JUL2000	6:30
		Week 12 (Unsch.)	31JUL2000	No		
		Week 13	09AUG2000	Yes	09AUG2000	15:09
		Week 14	15AUG2000	Yes	15AUG2000	14:30
		Week 15	21AUG2000	Yes	21AUG2000	13:40
		Week 16	30AUG2000	Yes	30AUG2000	9:50
		Week 16	01SEP2000	No		
		Week 17	04SEP2000	Yes	04SEP2000	12:30
		Week 18	11SEP2000	Yes	11SEP2000	7:09
		Week 18 (Unsch.)	12SEP2000	No		
		Week 19	19SEP2000	Yes	19SEP2000	10:00
		Week 20	28SEP2000	Yes	28SEP2000	8:55
		Week 21 (Fol. Up)	02OCT2000	No		
		Week 22 (Fol. Up)	09OCT2000	No		
		Week 22 (Unsch. Fol. Up)	09OCT2000	No		
		Week 24 (Fol. Up)	27OCT2000	No		
030.001.00174	IPP	Screening	11APR2000	No		
		Clearance (Day -5)	04MAY2000	Yes	04MAY2000	
		Clearance (Day -4)	05MAY2000	Yes	05MAY2000	
		Clearance (Day -3)	06MAY2000	Yes	06MAY2000	
		Load Dose 1	09MAY2000	Yes	09MAY2000	10:25
		Load Dose 2	10MAY2000	Yes	10MAY2000	8:38
		Load Dose 3	11MAY2000	Yes	11MAY2000	8:10
		Week 1	17MAY2000	Yes	17MAY2000	8:40
		Week 2	24MAY2000	Yes	24MAY2000	8:28
		Week 3	30MAY2000	Yes	30MAY2000	15:11
		Week 4	06JUN2000	Yes	06JUN2000	14:25
		Week 5	15JUN2000	Yes	15JUN2000	7:38
		Week 6	22JUN2000	Yes	22JUN2000	12:10
		Week 7	28JUN2000	Yes	28JUN2000	8:35
		Week 7 (Unsch.)	30JUN2000	No		
		Week 8	04JUL2000	Yes	04JUL2000	8:25
		Week 8	05JUL2000	No		
		Week 8 (Unsch.)	05JUL2000	No		
		Week 9	12JUL2000	Yes	12JUL2000	8:07
		Week 10 (Fol. Up)	19JUL2000	No		

*Popn=Population (I=Intent-to-Treat, PP=Per Protocol)
Tafenoquine = Tafenoquine 200mg, Mefloquine = Mefloquine 250mg

SB252263 - 030

Listing B.02

Visit And Dosing Dates

Treatment : Placebo

Subject Number	Popn*	Visit	Visit Date	Dose Taken ?	Date of Dose	Time of Dose
030.001.00174	IPP	Week 10 (Unsch. Fol. Up)	19JUL2000	No		
		Week 11 (Fol. Up)	27JUL2000	No		
		Week 12 (Fol. Up)	02AUG2000	No		
		Week 12 (Unsch. Fol. Up)	03AUG2000	No		
030.001.00175	IPP	Screening	17APR2000	No		
		Clearance (Day -5)	04MAY2000	Yes	04MAY2000	
		Clearance (Day -4)	05MAY2000	Yes	05MAY2000	
		Clearance (Day -3)	06MAY2000	Yes	06MAY2000	
		Load Dose 1	09MAY2000	Yes	09MAY2000	10:20
		Load Dose 2	10MAY2000	Yes	10MAY2000	11:59
		Load Dose 3	11MAY2000	Yes	11MAY2000	
		Week 1	17MAY2000	Yes	17MAY2000	9:37
		Week 2	24MAY2000	Yes	24MAY2000	9:03
		Week 3	31MAY2000	Yes	31MAY2000	8:58
		Week 4	06JUN2000	Yes	06JUN2000	9:35
		Week 5	14JUN2000	Yes	14JUN2000	8:23
		Week 6	21JUN2000	Yes	21JUN2000	8:32
		Week 7	28JUN2000	Yes	28JUN2000	10:32
		Week 8	04JUL2000	Yes	04JUL2000	7:54
		Week 8 (Unsch.)	07JUL2000	No		
		Week 9	12JUL2000	Yes	12JUL2000	8:31
		Week 10 (Fol. Up)	19JUL2000	No		
		Week 11 (Fol. Up)	26JUL2000	No		
		Week 12 (Fol. Up)	02AUG2000	No		
		Week 13 (Unsch. Fol. Up)	09AUG2000	No		
030.001.00182	I	Screening	10APR2000	No		
		Clearance (Day -5)	04MAY2000	Yes	04MAY2000	
		Clearance (Day -4)	05MAY2000	Yes	05MAY2000	
		Clearance (Day -3)	06MAY2000	Yes	06MAY2000	
		Load Dose 1	09MAY2000	Yes	09MAY2000	11:20
		Load Dose 2	10MAY2000	Yes	10MAY2000	10:00
		Load Dose 3	11MAY2000	Yes	11MAY2000	7:10
		Week 1	17MAY2000	Yes	17MAY2000	6:40
		Week 2	24MAY2000	Yes	24MAY2000	8:06
		Week 3	31MAY2000	Yes	31MAY2000	6:20
		Week 4	06JUN2000	Yes	06JUN2000	13:39
		Week 5 (Unsch. Fol. Up)	12JUN2000	No		
		Week 5 (Fol. Up)	14JUN2000	No		
		Week 6 (Fol. Up)	19JUN2000	No		
		Week 7 (Fol. Up)	26JUN2000	No		

*Popn=Population (I=Intent-to-Treat, PP=Per Protocol)
Tafenoquine = Tafenoquine 200mg, Mefloquine = Mefloquine 250mg

SB252263 - 030

Listing B.02

Visit And Dosing Dates

Treatment : Placebo

Subject Number	Popn*	Visit	Visit Date	Dose Taken ?	Date of Dose	Time of Dose
030.001.00183	IPP	Screening	11APR2000	No		
		Screening (Unsch.)	20APR2000	No		
		Clearance (Day -5)	04MAY2000	Yes	04MAY2000	
		Clearance (Day -4)	05MAY2000	Yes	05MAY2000	
		Clearance (Day -3)	06MAY2000	Yes	06MAY2000	
		Clearance (Unsch.)	05MAY2000	No		
		Load Dose 1	09MAY2000	Yes	09MAY2000	11:24
		Load Dose 2	10MAY2000	Yes	10MAY2000	11:30
		Load Dose 3	11MAY2000	Yes	11MAY2000	8:49
		Week 1 (Unsch.)	14MAY2000	No		
		Week 1 (Unsch.)	15MAY2000	No		
		Week 1	17MAY2000	Yes	17MAY2000	7:50
		Week 2	24MAY2000	Yes	24MAY2000	7:13
		Week 3 (Unsch.)	28MAY2000	No		
		Week 3 (Unsch.)	30MAY2000	No		
		Week 3	31MAY2000	Yes	31MAY2000	7:50
		Week 4	06JUN2000	Yes	06JUN2000	12:01
		Week 5	14JUN2000	Yes	14JUN2000	7:50
		Week 6	21JUN2000	Yes	21JUN2000	7:43
		Week 7 (Unsch. Fol. Up)	27JUN2000	No		
		Week 7 (Fol. Up)	28JUN2000	No		
		Week 8 (Fol. Up)	03JUL2000	No		
		Week 8 (Unsch. Fol. Up)	03JUL2000	No		
		Week 9 (Fol. Up)	12JUL2000	No		
		Week 9 (Unsch. Fol. Up)	14JUL2000	No		
030.001.00191	IPP	Screening (Unsch.)	10APR2000	No		
		Screening	18APR2000	No		
		Screening (Unsch.)	18APR2000	No		
		Clearance (Day -5)	04MAY2000	Yes	04MAY2000	
		Clearance (Day -4)	05MAY2000	Yes	05MAY2000	
		Clearance (Day -3)	06MAY2000	Yes	06MAY2000	
		Load Dose 1	09MAY2000	Yes	09MAY2000	12:19
		Load Dose 2	10MAY2000	Yes	10MAY2000	12:21
		Load Dose 3	11MAY2000	Yes	11MAY2000	10:07
		Week 1	17MAY2000	Yes	17MAY2000	10:28
		Week 2	25MAY2000	Yes	25MAY2000	13:05
		Week 3	01JUN2000	Yes	01JUN2000	11:15
		Week 4	09JUN2000	Yes	09JUN2000	12:35
		Week 5	15JUN2000	Yes	15JUN2000	13:10
		Week 6 (Fol. Up)	21JUN2000	No		
		Week 7 (Fol. Up)	28JUN2000	No		
		Week 8 (Fol. Up)	04JUL2000	No		

*Popn=Population (I=Intent-to-Treat, PP=Per Protocol)
Tafenoquine = Tafenoquine 200mg, Mefloquine = Mefloquine 250mg

SB252263 - 030

Listing B.02

Visit And Dosing Dates

Treatment : Placebo

Subject Number	Popn*	Visit	Visit Date	Dose Taken ?	Date of Dose	Time of Dose
030.001.00191	IPP	Week 8 (Unsch. Fol. Up)	07JUL2000	No		
		Week 9 (Fol. Up)	12JUL2000	No		
030.001.00192	IPP	Screening	11APR2000	No		
		Clearance (Day -5)	04MAY2000	Yes	04MAY2000	
		Clearance (Day -4)	05MAY2000	Yes	05MAY2000	
		Clearance (Day -3)	06MAY2000	Yes	06MAY2000	
		Load Dose 1	09MAY2000	Yes	09MAY2000	11:55
		Load Dose 2	10MAY2000	Yes	10MAY2000	17:40
		Load Dose 3	11MAY2000	Yes	11MAY2000	17:30
		Week 1	17MAY2000	Yes	17MAY2000	12:00
		Week 2	25MAY2000	Yes	25MAY2000	18:20
		Week 3	31MAY2000	Yes	31MAY2000	9:00
		Week 4 (Fol. Up)	06JUN2000	No		
		Week 6 (Fol. Up)	17JUN2000	No		
		Week 6 (Unsch. Fol. Up)	19JUN2000	No		
		Week 6 (Fol. Up)	21JUN2000	No		
		Week 7 (Fol. Up)	29JUN2000	No		
030.001.00193	IPP	Screening	14APR2000	No		
		Clearance (Day -5)	04MAY2000	Yes	04MAY2000	
		Clearance (Day -4)	05MAY2000	Yes	05MAY2000	
		Clearance (Day -3)	06MAY2000	Yes	06MAY2000	
		Clearance (Unsch.)	08MAY2000	No		
		Load Dose 1	09MAY2000	Yes	09MAY2000	12:05
		Load Dose 2	10MAY2000	Yes	10MAY2000	16:20
		Load Dose 3	11MAY2000	Yes	11MAY2000	8:10
		Week 1 (Unsch.)	14MAY2000	No		
		Week 1	17MAY2000	Yes	17MAY2000	8:10
		Week 2 (Unsch.)	20MAY2000	No		
		Week 2	24MAY2000	Yes	24MAY2000	8:45
		Week 3	31MAY2000	Yes	31MAY2000	7:30
		Week 4 (Fol. Up)	07JUN2000	No		
		Week 5 (Fol. Up)	15JUN2000	No		
		Week 6 (Fol. Up)	21JUN2000	No		
030.001.00197	IPP	Screening	17APR2000	No		
		Clearance (Day -5)	04MAY2000	Yes	04MAY2000	
		Clearance (Day -4)	05MAY2000	Yes	05MAY2000	
		Clearance (Day -3)	06MAY2000	Yes	06MAY2000	
		Load Dose 1	09MAY2000	Yes	09MAY2000	12:45

*Popn=Population (I=Intent-to-Treat, PP=Per Protocol)
Tafenoquine = Tafenoquine 200mg, Mefloquine = Mefloquine 250mg

SB252263 - 030

Listing B.02

Visit And Dosing Dates

Treatment : Placebo

Subject Number	Popn*	Visit	Visit Date	Dose Taken ?	Date of Dose	Time of Dose
030.001.00197	IPP	Load Dose 2	10MAY2000	Yes	10MAY2000	11:00
		Load Dose 3	11MAY2000	Yes	11MAY2000	8:11
		Week 1 (Unsch.)	15MAY2000	No		
		Week 1	17MAY2000	Yes	17MAY2000	6:15
		Week 2	24MAY2000	Yes	24MAY2000	7:38
		Week 3	31MAY2000	Yes	31MAY2000	7:39
		Week 4 (Fol. Up)	06JUN2000	No		
		Week 5 (Fol. Up)	14JUN2000	No		
		Week 6 (Fol. Up)	21JUN2000	No		
		Week 7 (Fol. Up)	28JUN2000	No		
030.001.00202		Screening	17APR2000	No		
		Clearance (Day -5)	04MAY2000	Yes	04MAY2000	
		Clearance (Day -4)	05MAY2000	Yes	05MAY2000	
		Clearance (Day -3)	06MAY2000	Yes	06MAY2000	
		Load Dose 1	09MAY2000	Yes	09MAY2000	14:30
		Load Dose 2	10MAY2000	Yes	10MAY2000	8:45
		Load Dose 3	11MAY2000	Yes	11MAY2000	8:40
		Week 1 (Unsch. Fol. Up)	13MAY2000	No		
		Week 1 (Fol. Up)	17MAY2000	No		
		Week 2 (Fol. Up)	24MAY2000	No		
030.001.00204	I	Screening	14APR2000	No		
		Clearance (Day -5)	04MAY2000	Yes	04MAY2000	
		Clearance (Day -4)	05MAY2000	Yes	05MAY2000	
		Clearance (Day -3)	06MAY2000	Yes	06MAY2000	
		Load Dose 1	09MAY2000	Yes	09MAY2000	13:45
		Load Dose 2	10MAY2000	Yes	10MAY2000	9:27
		Load Dose 3	11MAY2000	Yes	11MAY2000	7:24
		Week 1	17MAY2000	Yes	17MAY2000	6:12
		Week 2	24MAY2000	Yes	24MAY2000	6:51
		Week 3	31MAY2000	Yes	31MAY2000	6:35
		Week 4	05JUN2000	Yes	05JUN2000	18:25
		Week 5 (Fol. Up)	14JUN2000	No		
		Week 6 (Fol. Up)	21JUN2000	No		
		Week 7 (Fol. Up)	28JUN2000	No		
		Week 8 (Fol. Up)	04JUL2000	No		
030.001.00208	IPP	Screening	11APR2000	No		
		Clearance (Day -5)	04MAY2000	Yes	04MAY2000	
		Clearance (Day -4)	05MAY2000	Yes	05MAY2000	

*Popn=Population (I=Intent-to-Treat, PP=Per Protocol)
Tafenoquine = Tafenoquine 200mg, Mefloquine = Mefloquine 250mg

SB252263 - 030

Listing B.02

Visit And Dosing Dates

Treatment : Placebo

Subject Number	Popn*	Visit	Visit Date	Dose Taken ?	Date of Dose	Time of Dose
030.001.00208	IPP	Clearance (Day -3)	06MAY2000	Yes	06MAY2000	
		Load Dose 1	09MAY2000	Yes	09MAY2000	13:52
		Load Dose 2	10MAY2000	Yes	10MAY2000	7:30
		Load Dose 3	11MAY2000	Yes	11MAY2000	6:38
		Load Dose (Unsch.)	11MAY2000	No		
		Week 1	17MAY2000	Yes	17MAY2000	6:40
		Week 2	24MAY2000	Yes	24MAY2000	6:30
		Week 3 (Unsch.)	29MAY2000	No		
		Week 3	31MAY2000	Yes	31MAY2000	6:40
		Week 4	07JUN2000	Yes	07JUN2000	18:05
		Week 5	14JUN2000	Yes	14JUN2000	6:50
		Week 6	21JUN2000	Yes	21JUN2000	6:55
		Week 7	28JUN2000	Yes	28JUN2000	6:30
		Week 8	04JUL2000	Yes	04JUL2000	6:40
		Week 9	12JUL2000	Yes	12JUL2000	6:30
		Week 10	19JUL2000	Yes	19JUL2000	6:49
		Week 11	26JUL2000	No		
		Week 12	02AUG2000	Yes	02AUG2000	7:05
		Week 13 (Fol. Up)	10AUG2000	No		
		Week 14 (Fol. Up)	17AUG2000	No		
		Week 15 (Fol. Up)	23AUG2000	No		
		Week 16 (Fol. Up)	28AUG2000	No		
030.001.00211	IPP	Screening	11APR2000	No		
		Clearance (Day -5)	04MAY2000	Yes	04MAY2000	
		Clearance (Day -4)	05MAY2000	Yes	05MAY2000	
		Clearance (Day -3)	06MAY2000	Yes	06MAY2000	
		Load Dose 1	09MAY2000	Yes	09MAY2000	14:15
		Load Dose 2	10MAY2000	Yes	10MAY2000	9:13
		Load Dose 3	11MAY2000	Yes	11MAY2000	7:45
		Week 1	17MAY2000	Yes	17MAY2000	7:20
		Week 1 (Unsch.)	18MAY2000	No		
		Week 2	24MAY2000	Yes	24MAY2000	7:27
		Week 2 (Unsch.)	26MAY2000	No		
		Week 3	31MAY2000	Yes	31MAY2000	7:41
		Week 4 (Unsch.)	04JUN2000	No		
		Week 4	06JUN2000	Yes	06JUN2000	15:09
		Week 5	14JUN2000	Yes	14JUN2000	6:30
		Week 6	21JUN2000	Yes	21JUN2000	6:18
		Week 6 (Unsch.)	21JUN2000	No		
		Week 7 (Fol. Up)	28JUN2000	No		
		Week 7 (Unsch. Fol. Up)	29JUN2000	No		
		Week 9 (Fol. Up)	09JUL2000	No		

*Popn=Population (I=Intent-to-Treat, PP=Per Protocol)
Tafenoquine = Tafenoquine 200mg, Mefloquine = Mefloquine 250mg

SB252263 - 030

Listing B.02

Visit And Dosing Dates

Treatment : Placebo

Subject Number	Popn*	Visit	Visit Date	Dose Taken ?	Date of Dose	Time of Dose
030.001.00211	IPP	Week 9 (Fol. Up)	12JUL2000	No		
030.001.00212	IPP	Screening	13APR2000	No		
		Clearance (Day -5)	04MAY2000	Yes	04MAY2000	
		Clearance (Day -4)	05MAY2000	Yes	05MAY2000	
		Clearance (Day -3)	06MAY2000	Yes	06MAY2000	
		Load Dose 1	09MAY2000	Yes	09MAY2000	14:16
		Load Dose 2	10MAY2000	Yes	10MAY2000	7:30
		Load Dose 3	11MAY2000	Yes	11MAY2000	6:44
		Week 1	17MAY2000	Yes	17MAY2000	6:45
		Week 2	24MAY2000	Yes	24MAY2000	6:25
		Week 3	31MAY2000	Yes	31MAY2000	6:15
		Week 4	07JUN2000	Yes	07JUN2000	18:36
		Week 5 (Unsch.)	13JUN2000	No		
		Week 5	14JUN2000	Yes	14JUN2000	6:45
		Week 6 (Unsch.)	18JUN2000	No		
		Week 6 (Unsch.)	20JUN2000	No		
		Week 6	21JUN2000	Yes	21JUN2000	6:45
		Week 7	28JUN2000	Yes	28JUN2000	7:15
		Week 7 (Unsch. Fol. Up)	30JUN2000	No		
		Week 8 (Fol. Up)	04JUL2000	No		
		Week 9 (Fol. Up)	12JUL2000	No		
		Week 10 (Fol. Up)	20JUL2000	No		
		Week 11 (Fol. Up)	26JUL2000	No		
030.001.00215	IPP	Screening	12APR2000	No		
		Clearance (Day -5)	04MAY2000	Yes	04MAY2000	
		Clearance (Day -4)	05MAY2000	Yes	05MAY2000	
		Clearance (Day -3)	06MAY2000	Yes	06MAY2000	
		Load Dose 1	09MAY2000	Yes	09MAY2000	15:10
		Load Dose 2	10MAY2000	Yes	10MAY2000	7:35
		Load Dose 3	11MAY2000	Yes	11MAY2000	7:31
		Week 1 (Unsch.)	12MAY2000	No		
		Week 1 (Unsch.)	14MAY2000	No		
		Week 1 (Unsch.)	15MAY2000	No		
		Week 1	17MAY2000	Yes	17MAY2000	6:50
		Week 2	24MAY2000	Yes	24MAY2000	7:30
		Week 3	31MAY2000	Yes	31MAY2000	6:49
		Week 4	06JUN2000	Yes	06JUN2000	14:45
		Week 5	14JUN2000	Yes	14JUN2000	9:45
		Week 7 (Fol. Up)	27JUN2000	No		
		Week 8 (Fol. Up)	04JUL2000	No		

*Popn=Population (I=Intent-to-Treat, PP=Per Protocol)
Tafenoquine = Tafenoquine 200mg, Mefloquine = Mefloquine 250mg

SB252263 - 030

Listing B.02

Visit And Dosing Dates

Treatment : Placebo

Subject Number	Popn*	Visit	Visit Date	Dose Taken ?	Date of Dose	Time of Dose
030.001.00215	IPP	Week 9 (Fol. Up)	10JUL2000	No		
030.001.00219	IPP	Screening	12APR2000	No		
		Clearance (Day -5)	04MAY2000	Yes	04MAY2000	
		Clearance (Day -4)	05MAY2000	Yes	05MAY2000	
		Clearance (Day -3)	06MAY2000	Yes	06MAY2000	
		Load Dose 1	09MAY2000	Yes	09MAY2000	15:42
		Load Dose 2	10MAY2000	Yes	10MAY2000	10:36
		Load Dose 3	11MAY2000	Yes	11MAY2000	9:30
		Week 1	17MAY2000	Yes	17MAY2000	7:41
		Week 2	24MAY2000	Yes	24MAY2000	7:26
		Week 3	31MAY2000	Yes	31MAY2000	7:34
		Week 4	06JUN2000	Yes	06JUN2000	10:40
		Week 5	14JUN2000	Yes	14JUN2000	7:31
		Week 6 (Fol. Up)	21JUN2000	No		
		Week 7 (Fol. Up)	30JUN2000	No		
		Week 9 (Fol. Up)	10JUL2000	No		
030.001.00222	IPP	Screening	14APR2000	No		
		Clearance (Day -5)	04MAY2000	Yes	04MAY2000	
		Clearance (Day -4)	05MAY2000	Yes	05MAY2000	
		Clearance (Day -3)	06MAY2000	Yes	06MAY2000	
		Load Dose 1	09MAY2000	Yes	09MAY2000	15:50
		Load Dose 2	10MAY2000	Yes	10MAY2000	11:57
		Load Dose 3	11MAY2000	Yes	11MAY2000	7:15
		Week 1 (Unsch.)	15MAY2000	No		
		Week 1	17MAY2000	Yes	17MAY2000	17:00
		Week 2	24MAY2000	Yes	24MAY2000	19:10
		Week 3	31MAY2000	Yes	31MAY2000	6:45
		Week 4	06JUN2000	Yes	06JUN2000	9:03
		Week 5	14JUN2000	Yes	14JUN2000	7:18
		Week 6	21JUN2000	Yes	21JUN2000	7:21
		Week 7 (Unsch. Fol. Up)	26JUN2000	No		
		Week 7 (Fol. Up)	28JUN2000	No		
		Week 8 (Fol. Up)	04JUL2000	No		
		Week 9 (Fol. Up)	12JUL2000	No		
		Week 10 (Fol. Up)	19JUL2000	No		
030.001.00225	IPP	Screening	10APR2000	No		
		Clearance (Day -5)	06MAY2000	Yes	06MAY2000	
		Clearance (Day -4)	07MAY2000	Yes	07MAY2000	

*Popn=Population (I=Intent-to-Treat, PP=Per Protocol)
Tafenoquine = Tafenoquine 200mg, Mefloquine = Mefloquine 250mg

SB252263 - 030

Listing B.02

Visit And Dosing Dates

Treatment : Placebo

Subject Number	Popn*	Visit	Visit Date	Dose Taken ?	Date of Dose	Time of Dose
030.001.00225	IPP	Clearance (Day -3)	08MAY2000	Yes	08MAY2000	
		Clearance (Unsch.)	06MAY2000	No		
		Load Dose 1	09MAY2000	Yes	09MAY2000	16:23
		Load Dose 2	10MAY2000	Yes	10MAY2000	10:30
		Load Dose 3	11MAY2000	Yes	11MAY2000	7:40
		Week 1	17MAY2000	Yes	17MAY2000	8:00
		Week 2	24MAY2000	Yes	24MAY2000	8:40
		Week 3 (Unsch.)	27MAY2000	No		
		Week 3	31MAY2000	Yes	31MAY2000	6:31
		Week 4	06JUN2000	Yes	06JUN2000	13:43
		Week 5	14JUN2000	Yes	14JUN2000	7:18
		Week 6 (Fol. Up)	21JUN2000	No		
		Week 6 (Unsch. Fol. Up)	21JUN2000	No		
		Week 7 (Unsch. Fol. Up)	26JUN2000	No		
		Week 7 (Fol. Up)	28JUN2000	No		
		Week 8 (Fol. Up)	04JUL2000	No		
		Week 9 (Fol. Up)	12JUL2000	No		
030.001.00226	IPP	Screening	13APR2000	No		
		Clearance (Day -5)	04MAY2000	Yes	04MAY2000	
		Clearance (Day -4)	05MAY2000	Yes	05MAY2000	
		Clearance (Day -3)	06MAY2000	Yes	06MAY2000	
		Load Dose 1	09MAY2000	Yes	09MAY2000	16:50
		Load Dose 2	10MAY2000	Yes	10MAY2000	11:00
		Load Dose 3	11MAY2000	Yes	11MAY2000	8:30
		Week 1	17MAY2000	Yes	17MAY2000	7:20
		Week 1 (Unsch.)	18MAY2000	No		
		Week 2	24MAY2000	Yes	24MAY2000	7:00
		Week 3	31MAY2000	Yes	31MAY2000	8:20
		Week 4	08JUN2000	Yes	08JUN2000	14:12
		Week 5	14JUN2000	Yes	14JUN2000	8:00
		Week 5	16JUN2000	No		
		Week 5 (Unsch.)	16JUN2000	No		
		Week 6	21JUN2000	Yes	21JUN2000	13:59
		Week 7 (Fol. Up)	28JUN2000	No		
		Week 8 (Fol. Up)	04JUL2000	No		
		Week 9 (Unsch. Fol. Up)	10JUL2000	No		
		Week 9 (Fol. Up)	12JUL2000	No		
		Week 10 (Fol. Up)	20JUL2000	No		
030.001.00229	IPP	Screening	13APR2000	No		
		Clearance (Day -5)	04MAY2000	Yes	04MAY2000	

*Popn=Population (I=Intent-to-Treat, PP=Per Protocol)
Tafenoquine = Tafenoquine 200mg, Mefloquine = Mefloquine 250mg

SB252263 - 030

Listing B.02

Visit And Dosing Dates

Treatment : Placebo

Subject Number	Popn*	Visit	Visit Date	Dose Taken ?	Date of Dose	Time of Dose
030.001.00229	IPP	Clearance (Day -4)	05MAY2000	Yes	05MAY2000	
		Clearance (Day -3)	06MAY2000	Yes	06MAY2000	
		Load Dose 1	09MAY2000	Yes	09MAY2000	17:06
		Load Dose 2	10MAY2000	Yes	10MAY2000	17:52
		Load Dose 3	11MAY2000	Yes	11MAY2000	13:30
		Week 1	18MAY2000	Yes	18MAY2000	20:00
		Week 2	24MAY2000	Yes	24MAY2000	9:50
		Week 3 (Unsch.)	28MAY2000	No		
		Week 3	31MAY2000	Yes	31MAY2000	10:59
		Week 4	07JUN2000	Yes	07JUN2000	10:36
		Week 4 (Unsch.)	09JUN2000	No		
		Week 5	15JUN2000	Yes	15JUN2000	18:47
		Week 6	21JUN2000	Yes	21JUN2000	7:35
		Week 7 (Fol. Up)	28JUN2000	No		
		Week 8 (Fol. Up)	05JUL2000	No		
		Week 9 (Fol. Up)	12JUL2000	No		
		Week 10 (Fol. Up)	19JUL2000	No		
		Week 10 (Unsch. Fol. Up)	21JUL2000	No		
030.001.00234	IPP	Screening	11APR2000	No		
		Clearance (Day -5)	05MAY2000	Yes	05MAY2000	
		Clearance (Day -4)	06MAY2000	Yes	06MAY2000	
		Clearance (Day -3)	07MAY2000	Yes	07MAY2000	
		Load Dose 1	10MAY2000	Yes	10MAY2000	7:03
		Load Dose 2	11MAY2000	Yes	11MAY2000	7:04
		Load Dose 3	12MAY2000	Yes	12MAY2000	6:44
		Week 1 (Unsch.)	13MAY2000	No		
		Week 1	17MAY2000	Yes	17MAY2000	6:47
		Week 2	24MAY2000	Yes	24MAY2000	9:00
		Week 3	31MAY2000	Yes	31MAY2000	6:10
		Week 4	06JUN2000	Yes	06JUN2000	13:31
		Week 5	14JUN2000	Yes	14JUN2000	6:20
		Week 6 (Fol. Up)	21JUN2000	No		
		Week 7 (Fol. Up)	28JUN2000	No		
		Week 8 (Fol. Up)	05JUL2000	No		
030.001.00236	IPP	Screening	11APR2000	No		
		Clearance (Day -5)	04MAY2000	Yes	04MAY2000	
		Clearance (Day -4)	05MAY2000	Yes	05MAY2000	
		Clearance (Day -3)	06MAY2000	Yes	06MAY2000	
		Load Dose 1	11MAY2000	Yes	11MAY2000	6:50
		Load Dose 2	12MAY2000	Yes	12MAY2000	6:30

*Popn=Population (I=Intent-to-Treat, PP=Per Protocol)
Tafenoquine = Tafenoquine 200mg, Mefloquine = Mefloquine 250mg

SB252263 - 030

Listing B.02

Visit And Dosing Dates

Treatment : Placebo

Subject Number	Popn*	Visit	Visit Date	Dose Taken ?	Date of Dose	Time of Dose
030.001.00236	IPP	Load Dose 3	13MAY2000	Yes	13MAY2000	15:31
		Week 1 (Unsch.)	14MAY2000	No		
		Week 1	18MAY2000	Yes	18MAY2000	6:55
		Week 2	24MAY2000	Yes	24MAY2000	7:10
		Week 3	01JUN2000	Yes	01JUN2000	14:51
		Week 4	09JUN2000	Yes	09JUN2000	11:05
		Week 5 (Unsch.)	12JUN2000	No		
		Week 5	14JUN2000	Yes	14JUN2000	8:10
		Week 6	21JUN2000	Yes	21JUN2000	9:09
		Week 7	30JUN2000	Yes	30JUN2000	10:15
		Week 8	04JUL2000	Yes	04JUL2000	9:22
		Week 9	12JUL2000	Yes	12JUL2000	15:00
		Week 10 (Fol. Up)	21JUL2000	No		
		Week 11 (Unsch. Fol. Up)	24JUL2000	No		
		Week 11 (Fol. Up)	26JUL2000	No		
		Week 12 (Fol. Up)	02AUG2000	No		
		Week 12 (Unsch. Fol. Up)	03AUG2000	No		
		Week 13 (Fol. Up)	09AUG2000	No		
030.001.00238	IPP	Screening	12APR2000	No		
		Clearance (Day -5)	05MAY2000	Yes	05MAY2000	
		Clearance (Day -4)	06MAY2000	Yes	06MAY2000	
		Clearance (Day -3)	07MAY2000	Yes	07MAY2000	
		Load Dose 1	10MAY2000	Yes	10MAY2000	7:47
		Load Dose 2	11MAY2000	Yes	11MAY2000	8:30
		Load Dose 3	12MAY2000	Yes	12MAY2000	6:35
		Week 1	17MAY2000	Yes	17MAY2000	7:10
		Week 2	24MAY2000	Yes	24MAY2000	7:30
		Week 2 (Unsch.)	25MAY2000	No		
		Week 3	31MAY2000	Yes	31MAY2000	8:10
		Week 5	12JUN2000	Yes	12JUN2000	
		Week 5 (Unsch.)	12JUN2000	No		
		Week 5	14JUN2000	Yes	14JUN2000	8:30
		Week 6 (Fol. Up)	21JUN2000	No		
		Week 7 (Unsch. Fol. Up)	26JUN2000	No		
		Week 7 (Unsch. Fol. Up)	27JUN2000	No		
		Week 7 (Fol. Up)	28JUN2000	No		
		Week 8 (Fol. Up)	04JUL2000	No		
		Week 8 (Unsch. Fol. Up)	05JUL2000	No		
		Week 9 (Fol. Up)	11JUL2000	No		
030.001.00242	IPP	Screening	14APR2000	No		

*Popn=Population (I=Intent-to-Treat, PP=Per Protocol)
Tafenoquine = Tafenoquine 200mg, Mefloquine = Mefloquine 250mg

SB252263 - 030

Listing B.02

Visit And Dosing Dates

Treatment : Placebo

Subject Number	Popn*	Visit	Visit Date	Dose Taken ?	Date of Dose	Time of Dose
030.001.00242	IPP	Clearance (Day -5)	05MAY2000	Yes	05MAY2000	
		Clearance (Day -4)	06MAY2000	Yes	06MAY2000	
		Clearance (Day -3)	07MAY2000	Yes	07MAY2000	
		Clearance (Unsch.)	08MAY2000	No		
		Load Dose 1	10MAY2000	Yes	10MAY2000	8:39
		Load Dose 2	11MAY2000	Yes	11MAY2000	6:35
		Load Dose 3	12MAY2000	Yes	12MAY2000	7:06
		Week 1 (Unsch.)	15MAY2000	No		
		Week 1	17MAY2000	Yes	17MAY2000	6:21
		Week 1 (Unsch.)	19MAY2000	No		
		Week 2	24MAY2000	Yes	24MAY2000	6:30
		Week 3	31MAY2000	Yes	31MAY2000	15:05
		Week 4	08JUN2000	Yes	08JUN2000	
		Week 5	14JUN2000	Yes	14JUN2000	12:55
		Week 6 (Fol. Up)	21JUN2000	No		
		Week 7 (Fol. Up)	30JUN2000	No		
		Week 8 (Fol. Up)	05JUL2000	No		
		Week 9 (Fol. Up)	10JUL2000	No		
030.001.00247	IPP	Screening	11APR2000	No		
		Clearance (Day -5)	05MAY2000	Yes	05MAY2000	
		Clearance (Day -4)	06MAY2000	Yes	06MAY2000	
		Clearance (Day -3)	07MAY2000	Yes	07MAY2000	
		Load Dose 1	10MAY2000	Yes	10MAY2000	8:55
		Load Dose 2	11MAY2000	Yes	11MAY2000	6:40
		Load Dose 3	12MAY2000	Yes	12MAY2000	6:55
		Week 1	17MAY2000	Yes	17MAY2000	6:42
		Week 1 (Unsch.)	20MAY2000	No		
		Week 2	24MAY2000	Yes	24MAY2000	6:20
		Week 3 (Unsch.)	29MAY2000	No		
		Week 3	31MAY2000	Yes	31MAY2000	6:10
		Week 4	06JUN2000	Yes	06JUN2000	10:51
		Week 5	14JUN2000	Yes	14JUN2000	7:32
		Week 5 (Unsch.)	14JUN2000	No		
		Week 6	21JUN2000	Yes	21JUN2000	8:14
		Week 7 (Unsch.)	26JUN2000	No		
		Week 7	28JUN2000	Yes	28JUN2000	8:11
		Week 8	04JUL2000	Yes	04JUL2000	7:30
		Week 8	05JUL2000	No		
		Week 9	14JUL2000	Yes	14JUL2000	7:37
		Week 10	19JUL2000	Yes	19JUL2000	8:31
		Week 11	26JUL2000	Yes	26JUL2000	8:45
		Week 12 (Fol. Up)	03AUG2000	No		

*Popn=Population (I=Intent-to-Treat, PP=Per Protocol)
Tafenoquine = Tafenoquine 200mg, Mefloquine = Mefloquine 250mg

SB252263 - 030

Listing B.02

Visit And Dosing Dates

Treatment : Placebo

Subject Number	Popn*	Visit	Visit Date	Dose Taken ?	Date of Dose	Time of Dose
030.001.00247	IPP	Week 13 (Fol. Up)	09AUG2000	No		
		Week 14 (Fol. Up)	19AUG2000	No		
		Week 15 (Fol. Up)	23AUG2000	No		
030.001.00248	IPP	Screening	13APR2000	No		
		Clearance (Day -5)	05MAY2000	Yes	05MAY2000	
		Clearance (Day -4)	06MAY2000	Yes	06MAY2000	
		Clearance (Day -3)	07MAY2000	Yes	07MAY2000	
		Load Dose 1	10MAY2000	Yes	10MAY2000	8:45
		Load Dose 2	11MAY2000	Yes	11MAY2000	8:15
		Load Dose 3	12MAY2000	Yes	12MAY2000	7:36
		Week 1 (Unsch.)	13MAY2000	No		
		Week 1 (Unsch.)	15MAY2000	No		
		Week 1	17MAY2000	Yes	17MAY2000	8:10
		Week 1 (Unsch.)	17MAY2000	No		
		Week 2	24MAY2000	Yes	24MAY2000	8:19
		Week 2 (Unsch.)	24MAY2000	No		
		Week 3	31MAY2000	Yes	31MAY2000	7:20
		Week 4	06JUN2000	Yes	06JUN2000	11:40
		Week 5	14JUN2000	Yes	14JUN2000	7:55
		Week 6	21JUN2000	Yes	21JUN2000	6:22
		Week 7	28JUN2000	Yes	28JUN2000	6:27
		Week 7 (Unsch.)	30JUN2000	No		
		Week 8	03JUL2000	Yes	03JUL2000	9:47
		Week 8 (Fol. Up)	04JUL2000	No		
		Week 9 (Fol. Up)	12JUL2000	No		
		Week 10 (Fol. Up)	20JUL2000	No		
		Week 11 (Fol. Up)	26JUL2000	No		
		Week 12 (Unsch. Fol. Up)	31JUL2000	No		
		Week 12 (Fol. Up)	02AUG2000	No		
030.001.00251	IPP	Screening	12APR2000	No		
		Clearance (Day -5)	05MAY2000	Yes	05MAY2000	
		Clearance (Day -4)	06MAY2000	Yes	06MAY2000	
		Clearance (Day -3)	07MAY2000	Yes	07MAY2000	
		Load Dose 1	10MAY2000	Yes	10MAY2000	9:42
		Load Dose 2	11MAY2000	Yes	11MAY2000	6:35
		Load Dose 3	12MAY2000	Yes	12MAY2000	6:40
		Week 1	17MAY2000	Yes	17MAY2000	10:23
		Week 2	24MAY2000	Yes	24MAY2000	6:35
		Week 3	31MAY2000	Yes	31MAY2000	6:02
		Week 4	06JUN2000	Yes	06JUN2000	11:41

*Popn=Population (I=Intent-to-Treat, PP=Per Protocol)
Tafenoquine = Tafenoquine 200mg, Mefloquine = Mefloquine 250mg

SB252263 - 030

Listing B.02

Visit And Dosing Dates

Treatment : Placebo

Subject Number	Popn*	Visit	Visit Date	Dose Taken ?	Date of Dose	Time of Dose
030.001.00251	IPP	Week 4 (Unsch.)	09JUN2000	No		
		Week 5	14JUN2000	Yes	14JUN2000	8:00
		Week 6 (Fol. Up)	21JUN2000	No		
		Week 7 (Unsch. Fol. Up)	25JUN2000	No		
		Week 7 (Fol. Up)	28JUN2000	No		
		Week 8 (Unsch. Fol. Up)	07JUL2000	No		
		Week 9 (Unsch. Fol. Up)	10JUL2000	No		
		Week 9 (Fol. Up)	12JUL2000	No		
030.001.00253	IPP	Screening	13APR2000	No		
		Clearance (Day -5)	05MAY2000	Yes	05MAY2000	
		Clearance (Day -4)	06MAY2000	Yes	06MAY2000	
		Clearance (Day -3)	07MAY2000	Yes	07MAY2000	
		Load Dose 1	10MAY2000	Yes	10MAY2000	9:45
		Load Dose 2	11MAY2000	Yes	11MAY2000	8:15
		Load Dose 3	12MAY2000	Yes	12MAY2000	9:30
		Week 1	17MAY2000	Yes	17MAY2000	10:05
		Week 2	24MAY2000	Yes	24MAY2000	8:22
		Week 3	31MAY2000	Yes	31MAY2000	8:20
		Week 4	06JUN2000	Yes	06JUN2000	10:10
		Week 5	14JUN2000	Yes	14JUN2000	11:33
		Week 6	21JUN2000	Yes	21JUN2000	7:30
		Week 7	28JUN2000	Yes	28JUN2000	8:38
		Week 8 (Unsch. Fol. Up)	03JUL2000	No		
		Week 8 (Fol. Up)	06JUL2000	No		
		Week 9 (Fol. Up)	12JUL2000	No		
		Week 10 (Fol. Up)	19JUL2000	No		
		Week 11 (Unsch. Fol. Up)	25JUL2000	No		
030.001.00256	IPP	Screening	14APR2000	No		
		Clearance (Day -5)	05MAY2000	Yes	05MAY2000	
		Clearance (Day -4)	06MAY2000	Yes	06MAY2000	
		Clearance (Day -3)	07MAY2000	Yes	07MAY2000	
		Load Dose 1	10MAY2000	Yes	10MAY2000	9:27
		Load Dose 2	11MAY2000	Yes	11MAY2000	8:37
		Load Dose 3	12MAY2000	Yes	12MAY2000	13:53
		Week 1	17MAY2000	Yes	17MAY2000	9:15
		Week 2	24MAY2000	Yes	24MAY2000	8:38
		Week 2 (Unsch.)	24MAY2000	No		
		Week 3	31MAY2000	Yes	31MAY2000	9:50
		Week 4	06JUN2000	Yes	06JUN2000	14:59
		Week 5	16JUN2000	Yes	16JUN2000	6:00

*Popn=Population (I=Intent-to-Treat, PP=Per Protocol)
Tafenoquine = Tafenoquine 200mg, Mefloquine = Mefloquine 250mg

SB252263 - 030

Listing B.02

Visit And Dosing Dates

Treatment : Placebo

Subject Number	Popn*	Visit	Visit Date	Dose Taken ?	Date of Dose	Time of Dose
030.001.00256	IPP	Week 5 (Unsch.)	16JUN2000	No		
		Week 5 (Unsch. Fol. Up)	17JUN2000	No		
		Week 6 (Fol. Up)	21JUN2000	No		
		Week 7 (Fol. Up)	29JUN2000	No		
		Week 8 (Fol. Up)	04JUL2000	No		
		Week 9 (Fol. Up)	10JUL2000	No		
030.001.00257	IPP	Screening	14APR2000	No		
		Clearance (Day -5)	05MAY2000	Yes	05MAY2000	
		Clearance (Day -4)	06MAY2000	Yes	06MAY2000	
		Clearance (Day -3)	07MAY2000	Yes	07MAY2000	
		Clearance (Unsch.)	08MAY2000	No		
		Load Dose 1	10MAY2000	No		
		Load Dose 1	12MAY2000	Yes	12MAY2000	14:50
		Load Dose 2	13MAY2000	Yes	13MAY2000	19:54
		Load Dose 3	14MAY2000	Yes	14MAY2000	9:30
		Week 1	18MAY2000	Yes	18MAY2000	6:50
		Week 2	25MAY2000	Yes	25MAY2000	14:11
		Week 3	01JUN2000	Yes	01JUN2000	14:00
		Week 4	06JUN2000	Yes	06JUN2000	14:05
		Week 4 (Unsch.)	11JUN2000	No		
		Week 5	16JUN2000	Yes	16JUN2000	7:10
		Week 6	23JUN2000	Yes	23JUN2000	17:26
		Week 7 (Fol. Up)	28JUN2000	No		
		Week 8 (Fol. Up)	05JUL2000	No		
		Week 8 (Unsch. Fol. Up)	10JUL2000	No		
		Week 9 (Fol. Up)	13JUL2000	No		
		Week 10 (Fol. Up)	20JUL2000	No		
030.001.00260	IPP	Screening	17APR2000	No		
		Clearance (Day -5)	05MAY2000	Yes	05MAY2000	
		Clearance (Day -4)	06MAY2000	Yes	06MAY2000	
		Clearance (Day -3)	07MAY2000	Yes	07MAY2000	
		Load Dose 1	10MAY2000	Yes	10MAY2000	9:52
		Load Dose 2	11MAY2000	Yes	11MAY2000	11:20
		Load Dose 3	12MAY2000	Yes	12MAY2000	10:04
		Week 1	17MAY2000	Yes	17MAY2000	7:35
		Week 1 (Unsch.)	18MAY2000	No		
		Week 2	24MAY2000	Yes	24MAY2000	7:30
		Week 2 (Unsch.)	27MAY2000	No		
		Week 3	31MAY2000	Yes	31MAY2000	7:40
		Week 4	06JUN2000	Yes	06JUN2000	9:10

*Popn=Population (I=Intent-to-Treat, PP=Per Protocol)
Tafenoquine = Tafenoquine 200mg, Mefloquine = Mefloquine 250mg

SB252263 - 030

Listing B.02

Visit And Dosing Dates

Treatment : Placebo

Subject Number	Popn*	Visit	Visit Date	Dose Taken ?	Date of Dose	Time of Dose
030.001.00260	IPP	Week 5	15JUN2000	Yes	15JUN2000	14:19
		Week 6	21JUN2000	Yes	21JUN2000	8:15
		Week 6 (Unsch. Fol. Up)	23JUN2000	No		
		Week 7 (Fol. Up)	28JUN2000	No		
		Week 7 (Unsch. Fol. Up)	01JUL2000	No		
		Week 8 (Fol. Up)	04JUL2000	No		
		Week 9 (Fol. Up)	12JUL2000	No		
		Week 10 (Unsch. Fol. Up)	19JUL2000	No		
030.001.00268	IPP	Screening	14APR2000	No		
		Clearance (Day -5)	05MAY2000	Yes	05MAY2000	
		Clearance (Day -4)	06MAY2000	Yes	06MAY2000	
		Clearance (Day -3)	07MAY2000	Yes	07MAY2000	
		Load Dose 1	10MAY2000	Yes	10MAY2000	12:27
		Load Dose 2	11MAY2000	Yes	11MAY2000	6:40
		Load Dose 3	12MAY2000	Yes	12MAY2000	6:50
		Week 1	17MAY2000	Yes	17MAY2000	6:40
		Week 1 (Unsch.)	18MAY2000	No		
		Week 2	24MAY2000	Yes	24MAY2000	6:20
		Week 3 (Fol. Up)	31MAY2000	No		
		Week 3 (Unsch. Fol. Up)	31MAY2000	No		
		Week 4 (Fol. Up)	06JUN2000	No		
		Week 4 (Unsch. Fol. Up)	09JUN2000	No		
		Week 5 (Unsch. Fol. Up)	13JUN2000	No		
		Week 5 (Fol. Up)	14JUN2000	No		
030.001.00269	IPP	Screening	13APR2000	No		
		Clearance (Day -5)	05MAY2000	Yes	05MAY2000	
		Clearance (Day -4)	06MAY2000	Yes	06MAY2000	
		Clearance (Day -3)	07MAY2000	Yes	07MAY2000	
		Load Dose 1	10MAY2000	Yes	10MAY2000	13:50
		Load Dose 2	11MAY2000	Yes	11MAY2000	13:18
		Load Dose 3	12MAY2000	Yes	12MAY2000	8:28
		Week 1	18MAY2000	Yes	18MAY2000	10:05
		Week 2	24MAY2000	Yes	24MAY2000	12:13
		Week 2 (Unsch.)	24MAY2000	No		
		Week 3	31MAY2000	Yes	31MAY2000	7:24
		Week 4	06JUN2000	Yes	06JUN2000	9:01
		Week 5	14JUN2000	Yes	14JUN2000	11:45
		Week 6	21JUN2000	Yes	21JUN2000	9:16
		Week 7 (Unsch. Fol. Up)	26JUN2000	No		
		Week 7 (Fol. Up)	28JUN2000	No		

*Popn=Population (I=Intent-to-Treat, PP=Per Protocol)
Tafenoquine = Tafenoquine 200mg, Mefloquine = Mefloquine 250mg

SB252263 - 030

Listing B.02

Visit And Dosing Dates

Treatment : Placebo

Subject Number	Popn*	Visit	Visit Date	Dose Taken ?	Date of Dose	Time of Dose
030.001.00269	IPP	Week 8 (Fol. Up)	06JUL2000	No		
		Week 9 (Fol. Up)	13JUL2000	No		
		Week 10 (Fol. Up)	20JUL2000	No		
030.001.00270	IPP	Screening	13APR2000	No		
		Clearance (Day -5)	04MAY2000	Yes	04MAY2000	
		Clearance (Day -4)	05MAY2000	Yes	05MAY2000	
		Clearance (Day -3)	06MAY2000	Yes	06MAY2000	
		Load Dose 1	10MAY2000	Yes	10MAY2000	14:06
		Load Dose 2	11MAY2000	Yes	11MAY2000	6:52
		Load Dose 3	12MAY2000	Yes	12MAY2000	6:35
		Week 1	17MAY2000	Yes	17MAY2000	5:30
		Week 2	24MAY2000	Yes	24MAY2000	6:00
		Week 3	31MAY2000	Yes	31MAY2000	6:00
		Week 4	07JUN2000	Yes	07JUN2000	
		Week 5	14JUN2000	Yes	14JUN2000	8:00
		Week 6	21JUN2000	Yes	21JUN2000	8:02
		Week 7	28JUN2000	Yes	28JUN2000	9:30
		Week 8 (Fol. Up)	04JUL2000	No		
		Week 8 (Unsch. Fol. Up)	05JUL2000	No		
		Week 9 (Fol. Up)	12JUL2000	No		
		Week 10 (Fol. Up)	19JUL2000	No		
030.001.00275	IPP	Screening	12APR2000	No		
		Clearance (Day -5)	05MAY2000	Yes	05MAY2000	
		Clearance (Day -4)	06MAY2000	Yes	06MAY2000	
		Clearance (Day -3)	07MAY2000	Yes	07MAY2000	
		Load Dose 1	10MAY2000	Yes	10MAY2000	16:42
		Load Dose 2	11MAY2000	Yes	11MAY2000	7:08
		Load Dose 3	12MAY2000	Yes	12MAY2000	7:10
		Week 1	17MAY2000	Yes	17MAY2000	8:13
		Week 2 (Unsch.)	22MAY2000	No		
		Week 2	24MAY2000	Yes	24MAY2000	6:52
		Week 2 (Unsch.)	27MAY2000	No		
		Week 3	31MAY2000	Yes	31MAY2000	6:32
		Week 4	06JUN2000	Yes	06JUN2000	8:54
		Week 5	14JUN2000	Yes	14JUN2000	13:30
		Week 6	21JUN2000	Yes	21JUN2000	7:09
		Week 7	28JUN2000	Yes	28JUN2000	6:47
		Week 8 (Unsch. Fol. Up)	05JUL2000	No		
		Week 9 (Fol. Up)	13JUL2000	No		
		Week 10 (Fol. Up)	19JUL2000	No		

*Popn=Population (I=Intent-to-Treat, PP=Per Protocol)
Tafenoquine = Tafenoquine 200mg, Mefloquine = Mefloquine 250mg

SB252263 - 030

Listing B.02

Visit And Dosing Dates

Treatment : Placebo

Subject Number	Popn*	Visit	Visit Date	Dose Taken ?	Date of Dose	Time of Dose
030.001.00275	IPP	Week 11 (Fol. Up)	26JUL2000	No		
030.001.00277	IPP	Screening	12APR2000	No		
		Screening	13APR2000	No		
		Clearance (Day -5)	05MAY2000	Yes	05MAY2000	
		Clearance (Day -4)	06MAY2000	Yes	06MAY2000	
		Clearance (Day -3)	07MAY2000	Yes	07MAY2000	
		Load Dose 1	10MAY2000	Yes	10MAY2000	17:50
		Load Dose 2	11MAY2000	Yes	11MAY2000	16:30
		Load Dose 3	12MAY2000	Yes	12MAY2000	7:33
		Week 1	17MAY2000	Yes	17MAY2000	6:40
		Week 2	24MAY2000	Yes	24MAY2000	7:53
		Week 3	31MAY2000	Yes	31MAY2000	7:30
		Week 4	07JUN2000	Yes	07JUN2000	5:09
		Week 5 (Unsch.)	13JUN2000	No		
		Week 5	14JUN2000	Yes	14JUN2000	8:00
		Week 6	21JUN2000	Yes	21JUN2000	9:46
		Week 7	28JUN2000	Yes	28JUN2000	9:13
		Week 8	04JUL2000	Yes	04JUL2000	13:04
		Week 8	05JUL2000	No		
		Week 9	12JUL2000	Yes	12JUL2000	7:10
		Week 10	19JUL2000	Yes	19JUL2000	6:45
		Week 11 (Fol. Up)	26JUL2000	No		
		Week 12 (Fol. Up)	02AUG2000	No		
		Week 13 (Fol. Up)	09AUG2000	No		
030.001.00278	IPP	Screening	12APR2000	No		
		Clearance (Day -5)	05MAY2000	Yes	05MAY2000	
		Clearance (Day -4)	06MAY2000	Yes	06MAY2000	
		Clearance (Day -3)	07MAY2000	Yes	07MAY2000	
		Clearance (Unsch.)	09MAY2000	No		
		Load Dose 1	10MAY2000	Yes	10MAY2000	17:43
		Load Dose 2	12MAY2000	Yes	12MAY2000	7:05
		Load Dose 3	13MAY2000	Yes	13MAY2000	15:16
		Week 1	17MAY2000	Yes	17MAY2000	7:12
		Week 2	24MAY2000	Yes	24MAY2000	7:18
		Week 3	31MAY2000	Yes	31MAY2000	6:30
		Week 4	07JUN2000	Yes	07JUN2000	17:42
		Week 5 (Unsch.)	13JUN2000	No		
		Week 5	14JUN2000	Yes	14JUN2000	17:45
		Week 6 (Unsch. Fol. Up)	18JUN2000	No		
		Week 6 (Fol. Up)	21JUN2000	No		

*Popn=Population (I=Intent-to-Treat, PP=Per Protocol)
Tafenoquine = Tafenoquine 200mg, Mefloquine = Mefloquine 250mg

SB252263 - 030

Listing B.02

Visit And Dosing Dates

Treatment : Placebo

Subject Number	Popn*	Visit	Visit Date	Dose Taken ?	Date of Dose	Time of Dose
030.001.00278	IPP	Week 7 (Fol. Up)	28JUN2000	No		
		Week 8 (Unsch. Fol. Up)	02JUL2000	No		
		Week 8 (Fol. Up)	04JUL2000	No		
030.001.00285	IPP	Screening	18APR2000	No		
		Clearance (Day -5)	05MAY2000	Yes	05MAY2000	
		Clearance (Day -4)	06MAY2000	Yes	06MAY2000	
		Clearance (Day -3)	07MAY2000	Yes	07MAY2000	
		Load Dose 1	11MAY2000	Yes	11MAY2000	9:02
		Load Dose 2	12MAY2000	Yes	12MAY2000	7:06
		Load Dose 3	13MAY2000	Yes	13MAY2000	10:26
		Week 1	18MAY2000	Yes	18MAY2000	7:30
		Week 2	24MAY2000	Yes	24MAY2000	7:00
		Week 3	31MAY2000	Yes	31MAY2000	6:41
		Week 4	06JUN2000	Yes	06JUN2000	11:15
		Week 5	14JUN2000	Yes	14JUN2000	7:15
		Week 6	21JUN2000	Yes	21JUN2000	8:26
		Week 7	28JUN2000	Yes	28JUN2000	11:38
		Week 8 (Fol. Up)	05JUL2000	No		
		Week 9 (Fol. Up)	13JUL2000	No		
		Week 10 (Fol. Up)	20JUL2000	No		
030.001.00287	IPP	Screening	10APR2000	No		
		Clearance (Day -5)	05MAY2000	Yes	05MAY2000	
		Clearance (Day -4)	06MAY2000	Yes	06MAY2000	
		Clearance (Day -3)	07MAY2000	Yes	07MAY2000	
		Clearance (Unsch.)	07MAY2000	No		
		Load Dose 1	11MAY2000	Yes	11MAY2000	9:25
		Load Dose 2	12MAY2000	Yes	12MAY2000	7:32
		Load Dose 3	13MAY2000	Yes	13MAY2000	11:30
		Week 1	18MAY2000	Yes	18MAY2000	8:10
		Week 1 (Unsch.)	19MAY2000	No		
		Week 2 (Unsch.)	23MAY2000	No		
		Week 2	24MAY2000	Yes	24MAY2000	7:45
		Week 2 (Unsch.)	24MAY2000	No		
		Week 3	31MAY2000	Yes	31MAY2000	7:20
		Week 4	06JUN2000	Yes	06JUN2000	8:45
		Week 5	14JUN2000	Yes	14JUN2000	9:25
		Week 6	21JUN2000	Yes	21JUN2000	6:11
		Week 7	28JUN2000	Yes	28JUN2000	11:13
		Week 8	04JUL2000	Yes	04JUL2000	7:46
		Week 9	12JUL2000	Yes	12JUL2000	7:14

*Popn=Population (I=Intent-to-Treat, PP=Per Protocol)
Tafenoquine = Tafenoquine 200mg, Mefloquine = Mefloquine 250mg

SB252263 - 030

Listing B.02

Visit And Dosing Dates

Treatment : Placebo

Subject Number	Popn*	Visit	Visit Date	Dose Taken ?	Date of Dose	Time of Dose
030.001.00287	IPP	Week 10 (Unsch.)	18JUL2000	No		
		Week 10	19JUL2000	Yes	19JUL2000	15:23
		Week 11	26JUL2000	Yes	26JUL2000	7:26
		Week 13 (Unsch. Fol. Up)	08AUG2000	No		
		Week 13 (Fol. Up)	09AUG2000	No		
		Week 14 (Fol. Up)	16AUG2000	No		
		Week 14 (Unsch. Fol. Up)	17AUG2000	No		
		Week 15 (Fol. Up)	23AUG2000	No		
030.001.00291	IPP	Screening	17APR2000	No		
		Clearance (Day -5)	05MAY2000	Yes	05MAY2000	
		Clearance (Day -4)	06MAY2000	Yes	06MAY2000	
		Clearance (Day -3)	07MAY2000	Yes	07MAY2000	
		Clearance (Unsch.)	08MAY2000	No		
		Load Dose 1	11MAY2000	Yes	11MAY2000	11:30
		Load Dose 2	12MAY2000	Yes	12MAY2000	6:48
		Load Dose 3	13MAY2000	Yes	13MAY2000	11:53
		Load Dose (Unsch.)	13MAY2000	No		
		Week 1	18MAY2000	Yes	18MAY2000	6:20
		Week 2	24MAY2000	Yes	24MAY2000	6:15
		Week 3	31MAY2000	Yes	31MAY2000	7:00
		Week 4	06JUN2000	Yes	06JUN2000	9:28
		Week 5	14JUN2000	Yes	14JUN2000	6:30
		Week 5 (Unsch.)	16JUN2000	No		
		Week 6	21JUN2000	Yes	21JUN2000	7:40
		Week 7	28JUN2000	Yes	28JUN2000	6:59
		Week 8 (Fol. Up)	04JUL2000	No		
		Week 9 (Fol. Up)	12JUL2000	No		
		Week 10 (Unsch. Fol. Up)	18JUL2000	No		
		Week 10 (Fol. Up)	19JUL2000	No		
030.001.00295		Screening	17APR2000	No		
		Clearance (Day -5)	05MAY2000	Yes	05MAY2000	
		Clearance (Day -4)	06MAY2000	Yes	06MAY2000	
		Clearance (Day -3)	07MAY2000	Yes	07MAY2000	
		Load Dose 1	11MAY2000	Yes	11MAY2000	15:35
		Load Dose 2	12MAY2000	Yes	12MAY2000	8:38
		Load Dose 3	13MAY2000	Yes	13MAY2000	9:00
		Week 1	18MAY2000	Yes	18MAY2000	9:03
		Week 2 (Unsch.)	23MAY2000	No		
		Week 2	24MAY2000	Yes	24MAY2000	7:21
		Week 3	31MAY2000	Yes	31MAY2000	9:00

*Popn=Population (I=Intent-to-Treat, PP=Per Protocol)
Tafenoquine = Tafenoquine 200mg, Mefloquine = Mefloquine 250mg

SB252263 - 030

Listing B.02

Visit And Dosing Dates

Treatment : Placebo

Subject Number	Popn*	Visit	Visit Date	Dose Taken ?	Date of Dose	Time of Dose
030.001.00295		Week 4	06JUN2000	Yes	06JUN2000	14:35
		Week 5	14JUN2000	Yes	14JUN2000	6:50
		Week 6	21JUN2000	Yes	21JUN2000	9:45
		Week 7	28JUN2000	Yes	28JUN2000	8:00
		Week 8	04JUL2000	Yes	04JUL2000	6:30
		Week 9	12JUL2000	Yes	12JUL2000	9:50
		Week 10	19JUL2000	Yes	19JUL2000	9:35
		Week 11 (Fol. Up)	26JUL2000	No		
		Week 12 (Fol. Up)	02AUG2000	No		
		Week 13 (Fol. Up)	09AUG2000	No		
		Week 14 (Fol. Up)	16AUG2000	No		
030.001.00297	IPP	Screening	18APR2000	No		
		Clearance (Day -5)	06MAY2000	Yes	06MAY2000	
		Clearance (Day -4)	07MAY2000	Yes	07MAY2000	
		Clearance (Day -3)	08MAY2000	Yes	08MAY2000	
		Load Dose 1	11MAY2000	Yes	11MAY2000	17:56
		Load Dose 2	12MAY2000	Yes	12MAY2000	6:43
		Load Dose 3	13MAY2000	Yes	13MAY2000	9:15
		Week 1	19MAY2000	Yes	19MAY2000	10:55
		Week 2	24MAY2000	Yes	24MAY2000	6:28
		Week 3	31MAY2000	Yes	31MAY2000	6:30
		Week 4	07JUN2000	Yes	07JUN2000	12:40
		Week 5	14JUN2000	Yes	14JUN2000	6:30
		Week 6	21JUN2000	Yes	21JUN2000	8:30
		Week 7	28JUN2000	Yes	28JUN2000	6:30
		Week 8	06JUL2000	Yes	06JUL2000	11:55
		Week 9	12JUL2000	Yes	12JUL2000	7:30
		Week 10	20JUL2000	Yes	20JUL2000	6:00
		Week 11	26JUL2000	Yes	26JUL2000	9:04
		Week 12	31JUL2000	Yes	31JUL2000	13:15
		Week 13	09AUG2000	Yes	09AUG2000	7:00
		Week 14	16AUG2000	Yes	16AUG2000	6:55
		Week 15	26AUG2000	Yes	26AUG2000	9:25
		Week 16	30AUG2000	Yes	30AUG2000	7:08
		Week 17	08SEP2000	Yes	08SEP2000	16:05
		Week 18	13SEP2000	Yes	13SEP2000	7:15
		Week 19	20SEP2000	Yes	20SEP2000	7:00
		Week 20 (Fol. Up)	26SEP2000	No		
		Week 21 (Fol. Up)	04OCT2000	No		
		Week 22 (Fol. Up)	12OCT2000	No		

*Popn=Population (I=Intent-to-Treat, PP=Per Protocol)
Tafenoquine = Tafenoquine 200mg, Mefloquine = Mefloquine 250mg

SB252263 - 030

Listing B.02

Visit And Dosing Dates

Treatment : Placebo

Subject Number	Popn*	Visit	Visit Date	Dose Taken ?	Date of Dose	Time of Dose
030.001.00300	IPP	Screening	11APR2000	No		
		Clearance (Day -5)	05MAY2000	Yes	05MAY2000	
		Clearance (Day -4)	06MAY2000	Yes	06MAY2000	
		Clearance (Day -3)	07MAY2000	Yes	07MAY2000	
		Load Dose 1	12MAY2000	Yes	12MAY2000	9:41
		Load Dose 2	13MAY2000	Yes	13MAY2000	15:53
		Load Dose 3	14MAY2000	Yes	14MAY2000	9:30
		Week 1	19MAY2000	Yes	19MAY2000	13:40
		Week 2	24MAY2000	Yes	24MAY2000	13:32
		Week 3	01JUN2000	Yes	01JUN2000	10:00
		Week 4	07JUN2000	Yes	07JUN2000	7:53
		Week 5	18JUN2000	Yes	18JUN2000	15:53
		Week 6	21JUN2000	Yes	21JUN2000	16:52
		Week 7 (Fol. Up)	01JUL2000	No		
		Week 8 (Fol. Up)	04JUL2000	No		
		Week 9 (Fol. Up)	12JUL2000	No		
030.001.00303	IPP	Screening	13APR2000	No		
		Clearance (Day -5)	05MAY2000	Yes	05MAY2000	
		Clearance (Day -4)	06MAY2000	Yes	06MAY2000	
		Clearance (Day -3)	07MAY2000	Yes	07MAY2000	
		Load Dose 1	12MAY2000	Yes	12MAY2000	13:55
		Load Dose 2	13MAY2000	Yes	13MAY2000	13:56
		Load Dose 3	14MAY2000	Yes	14MAY2000	10:10
		Load Dose (Unsch.)	13MAY2000	No		
		Week 1	18MAY2000	Yes	18MAY2000	12:55
		Week 2	24MAY2000	Yes	24MAY2000	6:40
		Week 3	31MAY2000	Yes	31MAY2000	6:40
		Week 3 (Unsch.)	04JUN2000	No		
		Week 4	06JUN2000	Yes	06JUN2000	13:40
		Week 4 (Unsch.)	11JUN2000	No		
		Week 5	14JUN2000	Yes	14JUN2000	6:45
		Week 6	21JUN2000	Yes	21JUN2000	6:53
		Week 7	28JUN2000	Yes	28JUN2000	6:10
		Week 7	03JUL2000	Yes	03JUL2000	14:15
		Week 9	12JUL2000	Yes	12JUL2000	6:33
		Week 10 (Fol. Up)	19JUL2000	No		
		Week 11 (Fol. Up)	26JUL2000	No		
		Week 12 (Fol. Up)	03AUG2000	No		
		Week 12 (Unsch. Fol. Up)	05AUG2000	No		
		Week 13 (Unsch. Fol. Up)	08AUG2000	No		

*Popn=Population (I=Intent-to-Treat, PP=Per Protocol)
Tafenoquine = Tafenoquine 200mg, Mefloquine = Mefloquine 250mg

SB252263 - 030

Listing B.02

Visit And Dosing Dates

Treatment : Tafenoquine

Subject Number	Popn*	Visit	Visit Date	Dose Taken ?	Date of Dose	Time of Dose
030.001.00001	IPP	Screening	12APR2000	No		
		Screening (Unsch.)	02MAY2000	No		
		Clearance (Day -5)	03MAY2000	Yes	03MAY2000	
		Clearance (Day -4)	04MAY2000	Yes	04MAY2000	
		Clearance (Day -3)	05MAY2000	Yes	05MAY2000	
		Load Dose 1	08MAY2000	Yes	08MAY2000	7:09
		Load Dose 2	09MAY2000	Yes	09MAY2000	7:35
		Load Dose 3	10MAY2000	Yes	10MAY2000	7:55
		Week 1	15MAY2000	Yes	15MAY2000	9:59
		Week 1 (Unsch. Fol. Up)	16MAY2000	No		
		Week 3 (Fol. Up)	26MAY2000	No		
		Week 3 (Fol. Up)	29MAY2000	No		
		Week 3 (Unsch. Fol. Up)	30MAY2000	No		
		Week 4 (Fol. Up)	05JUN2000	No		
		Week 5 (Fol. Up)	12JUN2000	No		
030.001.00004	IPP	Screening	13APR2000	No		
		Screening (Unsch.)	01MAY2000	No		
		Clearance (Day -5)	03MAY2000	Yes	03MAY2000	
		Clearance (Day -4)	04MAY2000	Yes	04MAY2000	
		Clearance (Day -3)	05MAY2000	Yes	05MAY2000	
		Clearance (Unsch.)	07MAY2000	No		
		Load Dose 1	08MAY2000	Yes	08MAY2000	7:48
		Load Dose 2	09MAY2000	Yes	09MAY2000	6:14
		Load Dose 3	10MAY2000	Yes	10MAY2000	13:55
		Week 1	15MAY2000	Yes	15MAY2000	6:57
		Week 2	22MAY2000	Yes	22MAY2000	6:00
		Week 3 (Fol. Up)	29MAY2000	No		
		Week 3 (Unsch. Fol. Up)	01JUN2000	No		
		Week 4 (Fol. Up)	04JUN2000	No		
		Week 5 (Fol. Up)	12JUN2000	No		
		Week 6 (Fol. Up)	19JUN2000	No		
		Week 6 (Unsch. Fol. Up)	19JUN2000	No		
		Week 6 (Unsch. Fol. Up)	21JUN2000	No		
030.001.00006	IPP	Screening	10APR2000	No		
		Clearance (Day -5)	03MAY2000	Yes	03MAY2000	
		Clearance (Day -4)	04MAY2000	Yes	04MAY2000	
		Clearance (Day -3)	05MAY2000	Yes	05MAY2000	
		Load Dose 1	08MAY2000	Yes	08MAY2000	7:20
		Load Dose 2	09MAY2000	Yes	09MAY2000	7:58
		Load Dose 3	10MAY2000	Yes	10MAY2000	7:24

*Popn=Population (I=Intent-to-Treat, PP=Per Protocol)
Tafenoquine = Tafenoquine 200mg, Mefloquine = Mefloquine 250mg

SB252263 - 030

Listing B.02

Visit And Dosing Dates

Treatment : Tafenoquine

Subject Number	Popn*	Visit	Visit Date	Dose Taken ?	Date of Dose	Time of Dose
030.001.00006	IPP	Week 1 (Unsch.)	14MAY2000	No		
		Week 1	15MAY2000	Yes	15MAY2000	7:06
		Week 2	22MAY2000	Yes	22MAY2000	6:40
		Week 3 (Unsch. Fol. Up)	26MAY2000	No		
		Week 3 (Fol. Up)	29MAY2000	No		
		Week 3 (Unsch. Fol. Up)	29MAY2000	No		
		Week 4 (Unsch. Fol. Up)	02JUN2000	No		
		Week 4 (Fol. Up)	05JUN2000	No		
		Week 5 (Fol. Up)	12JUN2000	No		
		Week 6 (Fol. Up)	19JUN2000	No		
		Week 6 (Unsch. Fol. Up)	20JUN2000	No		
030.001.00009	IPP	Screening	12APR2000	No		
		Clearance (Day -5)	03MAY2000	Yes	03MAY2000	
		Clearance (Day -4)	04MAY2000	Yes	04MAY2000	
		Clearance (Day -3)	05MAY2000	Yes	05MAY2000	
		Load Dose 1	08MAY2000	Yes	08MAY2000	7:05
		Load Dose 2	09MAY2000	Yes	09MAY2000	7:00
		Load Dose 3	10MAY2000	Yes	10MAY2000	7:35
		Week 1	15MAY2000	Yes	15MAY2000	6:52
		Week 2	22MAY2000	Yes	22MAY2000	7:01
		Week 3	29MAY2000	Yes	29MAY2000	6:11
		Week 4	04JUN2000	Yes	04JUN2000	9:36
		Week 5	12JUN2000	Yes	12JUN2000	6:47
		Week 6	19JUN2000	Yes	19JUN2000	6:18
		Week 7	26JUN2000	Yes	26JUN2000	6:48
		Week 8 (Fol. Up)	03JUL2000	No		
		Week 9 (Fol. Up)	10JUL2000	No		
		Week 10 (Fol. Up)	17JUL2000	No		
		Week 11 (Fol. Up)	24JUL2000	No		
030.001.00014	IPP	Screening	10APR2000	No		
		Clearance (Day -5)	03MAY2000	Yes	03MAY2000	
		Clearance (Day -4)	04MAY2000	Yes	04MAY2000	
		Clearance (Day -3)	05MAY2000	Yes	05MAY2000	
		Load Dose 1	08MAY2000	Yes	08MAY2000	7:23
		Load Dose 2	09MAY2000	Yes	09MAY2000	7:10
		Load Dose 3	10MAY2000	Yes	10MAY2000	8:05
		Week 1	15MAY2000	Yes	15MAY2000	8:30
		Week 2	22MAY2000	Yes	22MAY2000	6:30
		Week 3	29MAY2000	Yes	29MAY2000	7:50
		Week 4	05JUN2000	Yes	05JUN2000	8:00

*Popn=Population (I=Intent-to-Treat, PP=Per Protocol)
Tafenoquine = Tafenoquine 200mg, Mefloquine = Mefloquine 250mg

SB252263 - 030

Listing B.02

Visit And Dosing Dates

Treatment : Tafenoquine

Subject Number	Popn*	Visit	Visit Date	Dose Taken ?	Date of Dose	Time of Dose
030.001.00014	IPP	Week 4 (Unsch.)	07JUN2000	No		
		Week 5	12JUN2000	Yes	12JUN2000	7:00
		Week 6	19JUN2000	Yes	19JUN2000	9:30
		Week 6 (Unsch.)	19JUN2000	No		
		Week 7	26JUN2000	Yes	26JUN2000	7:48
		Week 8	03JUL2000	Yes	03JUL2000	6:50
		Week 9	10JUL2000	Yes	10JUL2000	7:10
		Week 9 (Unsch.)	10JUL2000	No		
		Week 10	17JUL2000	Yes	17JUL2000	7:40
		Week 11	24JUL2000	Yes	24JUL2000	8:59
		Week 12	31JUL2000	Yes	31JUL2000	5:58
		Week 12 (Unsch. Fol. Up)	01AUG2000	No		
		Week 13 (Fol. Up)	07AUG2000	No		
		Week 13 (Unsch. Fol. Up)	09AUG2000	No		
		Week 14 (Fol. Up)	14AUG2000	No		
		Week 15 (Fol. Up)	21AUG2000	No		
030.001.00016	IPP	Screening	10APR2000	No		
		Screening (Unsch.)	28APR2000	No		
		Clearance (Day -5)	03MAY2000	Yes	03MAY2000	
		Clearance (Day -4)	04MAY2000	Yes	04MAY2000	
		Clearance (Day -3)	05MAY2000	Yes	05MAY2000	
		Load Dose 1	08MAY2000	Yes	08MAY2000	7:31
		Load Dose 2	09MAY2000	Yes	09MAY2000	6:48
		Load Dose 3	10MAY2000	Yes	10MAY2000	10:14
		Load Dose (Unsch.)	10MAY2000	No		
		Week 1	15MAY2000	Yes	15MAY2000	11:13
		Week 1 (Unsch.)	18MAY2000	No		
		Week 2 (Unsch.)	19MAY2000	No		
		Week 2	22MAY2000	Yes	22MAY2000	11:17
		Week 3	31MAY2000	Yes	31MAY2000	7:50
		Week 4	05JUN2000	Yes	05JUN2000	6:58
		Week 5	12JUN2000	Yes	12JUN2000	7:14
		Week 6 (Unsch.)	17JUN2000	No		
		Week 6	19JUN2000	Yes	19JUN2000	7:27
		Week 7 (Unsch.)	24JUN2000	No		
		Week 7	26JUN2000	Yes	26JUN2000	7:09
		Week 8	03JUL2000	Yes	03JUL2000	9:01
		Week 9	10JUL2000	Yes	10JUL2000	6:00
		Week 10	18JUL2000	Yes	18JUL2000	11:05
		Week 11	24JUL2000	Yes	24JUL2000	8:20
		Week 12	31JUL2000	Yes	31JUL2000	6:30
		Week 13	07AUG2000	Yes	07AUG2000	8:03

*Popn=Population (I=Intent-to-Treat, PP=Per Protocol)
Tafenoquine = Tafenoquine 200mg, Mefloquine = Mefloquine 250mg

SB252263 - 030

Listing B.02

Visit And Dosing Dates

Treatment : Tafenoquine

Subject Number	Popn*	Visit	Visit Date	Dose Taken ?	Date of Dose	Time of Dose
030.001.00016	IPP	Week 14 (Fol. Up)	14AUG2000	No		
		Week 15 (Fol. Up)	23AUG2000	No		
		Week 16 (Fol. Up)	28AUG2000	No		
030.001.00017	IPP	Screening	17APR2000	No		
		Screening (Unsch.)	28APR2000	No		
		Clearance (Day -5)	03MAY2000	Yes	03MAY2000	
		Clearance (Day -4)	04MAY2000	Yes	04MAY2000	
		Clearance (Day -3)	05MAY2000	Yes	05MAY2000	
		Load Dose 1	08MAY2000	Yes	08MAY2000	10:10
		Load Dose 2	09MAY2000	Yes	09MAY2000	6:50
		Load Dose 3	10MAY2000	Yes	10MAY2000	7:40
		Week 1	17MAY2000	Yes	17MAY2000	8:20
		Week 2	23MAY2000	Yes	23MAY2000	13:30
		Week 3	29MAY2000	Yes	29MAY2000	7:10
		Week 3 (Unsch.)	30MAY2000	No		
		Week 4	05JUN2000	Yes	05JUN2000	13:35
		Week 5	13JUN2000	Yes	13JUN2000	10:30
		Week 6 (Unsch.)	16JUN2000	No		
		Week 6	18JUN2000	Yes	18JUN2000	11:11
		Week 6 (Unsch.)	18JUN2000	No		
		Week 7	26JUN2000	Yes	26JUN2000	6:35
		Week 8	05JUL2000	No		
		Week 9	07JUL2000	Yes	07JUL2000	11:00
		Week 9 (Unsch.)	07JUL2000	No		
		Week 9	11JUL2000	Yes	11JUL2000	6:30
		Week 9 (Unsch.)	11JUL2000	No		
		Week 10	17JUL2000	Yes	17JUL2000	6:45
		Week 11	24JUL2000	Yes	24JUL2000	7:30
		Week 11 (Unsch.)	24JUL2000	No		
		Week 12	01AUG2000	Yes	01AUG2000	14:20
		Week 12 (Unsch.)	01AUG2000	No		
		Week 14	11AUG2000	Yes	11AUG2000	15:00
		Week 14	14AUG2000	Yes	14AUG2000	11:15
		Week 14 (Unsch.)	14AUG2000	No		
		Week 15	21AUG2000	Yes	21AUG2000	17:40
		Week 16	28AUG2000	Yes	28AUG2000	14:40
		Week 16 (Unsch.)	28AUG2000	No		
		Week 16	30AUG2000	No		
		Week 17	04SEP2000	Yes	04SEP2000	11:14
		Week 18	11SEP2000	Yes	11SEP2000	9:40
		Week 19	18SEP2000	Yes	18SEP2000	15:40
		Week 20 (Fol. Up)	28SEP2000	No		

*Popn=Population (I=Intent-to-Treat, PP=Per Protocol)
Tafenoquine = Tafenoquine 200mg, Mefloquine = Mefloquine 250mg

SB252263 - 030

Listing B.02

Visit And Dosing Dates

Treatment : Tafenoquine

Subject Number	Popn*	Visit	Visit Date	Dose Taken ?	Date of Dose	Time of Dose
030.001.00017	IPP	Week 22 (Fol. Up)	08OCT2000	No		
		Week 22 (Unsch. Fol. Up)	08OCT2000	No		
		Week 22 (Fol. Up)	11OCT2000	No		
		Week 22 (Unsch. Fol. Up)	11OCT2000	No		
030.001.00024	IPP	Screening	11APR2000	No		
		Clearance (Day -5)	03MAY2000	Yes	03MAY2000	
		Clearance (Day -4)	04MAY2000	Yes	04MAY2000	
		Clearance (Day -3)	05MAY2000	Yes	05MAY2000	
		Load Dose 1	08MAY2000	Yes	08MAY2000	8:11
		Load Dose 2	09MAY2000	Yes	09MAY2000	7:15
		Load Dose 3	10MAY2000	Yes	10MAY2000	8:26
		Week 1	17MAY2000	Yes	17MAY2000	13:45
		Week 2	23MAY2000	Yes	23MAY2000	16:16
		Week 3	29MAY2000	Yes	29MAY2000	9:30
		Week 4	08JUN2000	Yes	08JUN2000	12:30
		Week 5	12JUN2000	Yes	12JUN2000	5:57
		Week 5 (Unsch.)	12JUN2000	No		
		Week 6	19JUN2000	Yes	19JUN2000	6:11
		Week 7 (Fol. Up)	26JUN2000	No		
		Week 7 (Unsch. Fol. Up)	27JUN2000	No		
		Week 8 (Fol. Up)	03JUL2000	No		
		Week 9 (Fol. Up)	13JUL2000	No		
030.001.00025	IPP	Screening	11APR2000	No		
		Clearance (Day -5)	03MAY2000	Yes	03MAY2000	
		Clearance (Day -4)	04MAY2000	Yes	04MAY2000	
		Clearance (Day -3)	05MAY2000	Yes	05MAY2000	
		Clearance (Unsch.)	03MAY2000	No		
		Load Dose 1	08MAY2000	Yes	08MAY2000	7:45
		Load Dose 2	09MAY2000	Yes	09MAY2000	6:30
		Load Dose 3	10MAY2000	Yes	10MAY2000	13:40
		Load Dose (Unsch.)	10MAY2000	No		
		Week 1 (Unsch.)	11MAY2000	No		
		Week 1	15MAY2000	Yes	15MAY2000	12:58
		Week 2	22MAY2000	Yes	22MAY2000	6:17
		Week 3 (Unsch.)	28MAY2000	No		
		Week 3	29MAY2000	Yes	29MAY2000	6:30
		Week 4	04JUN2000	Yes	04JUN2000	11:07
		Week 4 (Unsch.)	08JUN2000	No		
		Week 5	12JUN2000	Yes	12JUN2000	15:00
		Week 6	19JUN2000	Yes	19JUN2000	18:14

*Popn=Population (I=Intent-to-Treat, PP=Per Protocol)
Tafenoquine = Tafenoquine 200mg, Mefloquine = Mefloquine 250mg

SB252263 - 030

Listing B.02

Visit And Dosing Dates

Treatment : Tafenoquine

Subject Number	Popn*	Visit	Visit Date	Dose Taken ?	Date of Dose	Time of Dose
030.001.00025	IPP	Week 7 (Unsch.)	25JUN2000	No		
		Week 7	26JUN2000	Yes	26JUN2000	6:13
		Week 8	03JUL2000	Yes	03JUL2000	6:45
		Week 9	10JUL2000	Yes	10JUL2000	5:44
		Week 10	17JUL2000	Yes	17JUL2000	5:48
		Week 11	24JUL2000	Yes	24JUL2000	6:18
		Week 12	31JUL2000	Yes	31JUL2000	6:18
		Week 13	07AUG2000	Yes	07AUG2000	9:20
		Week 15 (Fol. Up)	24AUG2000	No		
		Week 16 (Fol. Up)	28AUG2000	No		
		Week 16 (Fol. Up)	31AUG2000	No		
030.001.00028	IPP	Screening	11APR2000	No		
		Screening (Unsch.)	20APR2000	No		
		Screening (Unsch.)	02MAY2000	No		
		Clearance (Day -5)	03MAY2000	Yes	03MAY2000	
		Clearance (Day -4)	04MAY2000	Yes	04MAY2000	
		Clearance (Day -3)	05MAY2000	Yes	05MAY2000	
		Load Dose 1	08MAY2000	Yes	08MAY2000	8:07
		Load Dose 2	09MAY2000	Yes	09MAY2000	7:39
		Load Dose 3	10MAY2000	Yes	10MAY2000	8:54
		Load Dose (Unsch.)	10MAY2000	No		
		Week 1	15MAY2000	Yes	15MAY2000	8:41
		Week 2 (Unsch.)	21MAY2000	No		
		Week 2	22MAY2000	Yes	22MAY2000	7:39
		Week 2 (Unsch.)	22MAY2000	No		
		Week 2 (Unsch.)	23MAY2000	No		
		Week 3	29MAY2000	Yes	29MAY2000	17:15
		Week 4	06JUN2000	Yes	06JUN2000	16:05
		Week 5	12JUN2000	Yes	12JUN2000	9:32
		Week 6	19JUN2000	Yes	19JUN2000	6:59
		Week 7	26JUN2000	Yes	26JUN2000	9:29
		Week 8	03JUL2000	Yes	03JUL2000	7:10
		Week 9	10JUL2000	Yes	10JUL2000	7:49
		Week 10	17JUL2000	Yes	17JUL2000	8:53
		Week 10 (Unsch.)	17JUL2000	No		
		Week 10 (Unsch. Fol. Up)	18JUL2000	No		
		Week 10 (Unsch. Fol. Up)	19JUL2000	No		
		Week 11 (Unsch. Fol. Up)	21JUL2000	No		
		Week 11 (Fol. Up)	24JUL2000	No		
		Week 12 (Fol. Up)	31JUL2000	No		
		Week 13 (Unsch. Fol. Up)	04AUG2000	No		
		Week 13 (Fol. Up)	07AUG2000	No		

*Popn=Population (I=Intent-to-Treat, PP=Per Protocol)
Tafenoquine = Tafenoquine 200mg, Mefloquine = Mefloquine 250mg

SB252263 - 030

Listing B.02

Visit And Dosing Dates

Treatment : Tafenoquine

Subject Number	Popn*	Visit	Visit Date	Dose Taken ?	Date of Dose	Time of Dose
030.001.00028	IPP	Week 14 (Unsch. Fol. Up)	14AUG2000	No		
030.001.00029	IPP	Screening	11APR2000	No		
		Clearance (Day -5)	03MAY2000	Yes	03MAY2000	
		Clearance (Day -4)	04MAY2000	Yes	04MAY2000	
		Clearance (Day -3)	05MAY2000	Yes	05MAY2000	
		Load Dose 1	08MAY2000	Yes	08MAY2000	8:10
		Load Dose 2	09MAY2000	Yes	09MAY2000	6:54
		Load Dose 3	10MAY2000	Yes	10MAY2000	13:28
		Week 1	15MAY2000	Yes	15MAY2000	13:13
		Week 2	22MAY2000	Yes	22MAY2000	6:14
		Week 3	29MAY2000	Yes	29MAY2000	6:33
		Week 4	05JUN2000	Yes	05JUN2000	8:20
		Week 5	12JUN2000	Yes	12JUN2000	6:10
		Week 6	19JUN2000	Yes	19JUN2000	5:49
		Week 6 (Unsch.)	19JUN2000	No		
		Week 7	26JUN2000	Yes	26JUN2000	6:11
		Week 8	03JUL2000	Yes	03JUL2000	5:18
		Week 9 (Fol. Up)	10JUL2000	No		
		Week 10 (Fol. Up)	18JUL2000	No		
		Week 11 (Fol. Up)	24JUL2000	No		
030.001.00030	IPP	Screening	14APR2000	No		
		Screening (Unsch.)	26APR2000	No		
		Clearance (Day -5)	03MAY2000	Yes	03MAY2000	
		Clearance (Day -4)	04MAY2000	Yes	04MAY2000	
		Clearance (Day -3)	05MAY2000	Yes	05MAY2000	
		Clearance (Unsch.)	04MAY2000	No		
		Clearance (Unsch.)	06MAY2000	No		
		Load Dose 1	08MAY2000	Yes	08MAY2000	8:27
		Load Dose 2	09MAY2000	Yes	09MAY2000	6:45
		Load Dose 3	10MAY2000	Yes	10MAY2000	10:20
		Week 1	15MAY2000	Yes	15MAY2000	9:15
		Week 2	22MAY2000	Yes	22MAY2000	7:40
		Week 2 (Unsch.)	22MAY2000	No		
		Week 3	29MAY2000	Yes	29MAY2000	8:20
		Week 3 (Unsch.)	29MAY2000	No		
		Week 4	05JUN2000	Yes	05JUN2000	10:16
		Week 5 (Unsch.)	09JUN2000	No		
		Week 5	12JUN2000	Yes	12JUN2000	8:10
		Week 6	19JUN2000	Yes	19JUN2000	16:27
		Week 7	26JUN2000	Yes	26JUN2000	9:18

*Popn=Population (I=Intent-to-Treat, PP=Per Protocol)
Tafenoquine = Tafenoquine 200mg, Mefloquine = Mefloquine 250mg

SB252263 - 030

Listing B.02

Visit And Dosing Dates

Treatment : Tafenoquine

Subject Number	Popn*	Visit	Visit Date	Dose Taken ?	Date of Dose	Time of Dose
030.001.00030	IPP	Week 8	03JUL2000	Yes	03JUL2000	7:40
		Week 9	10JUL2000	Yes	10JUL2000	8:20
		Week 10 (Fol. Up)	17JUL2000	No		
		Week 11 (Fol. Up)	24JUL2000	No		
		Week 11 (Unsch. Fol. Up)	24JUL2000	No		
		Week 12 (Fol. Up)	31JUL2000	No		
		Week 13 (Unsch. Fol. Up)	07AUG2000	No		
030.001.00040	IPP	Screening	13APR2000	No		
		Clearance (Day -5)	03MAY2000	Yes	03MAY2000	
		Clearance (Day -4)	04MAY2000	Yes	04MAY2000	
		Clearance (Day -3)	05MAY2000	Yes	05MAY2000	
		Clearance (Unsch.)	06MAY2000	No		
		Load Dose 1	08MAY2000	Yes	08MAY2000	9:01
		Load Dose 2	09MAY2000	Yes	09MAY2000	7:35
		Load Dose 3	10MAY2000	Yes	10MAY2000	13:12
		Week 1	15MAY2000	Yes	15MAY2000	9:53
		Week 2 (Unsch.)	21MAY2000	No		
		Week 2	22MAY2000	Yes	22MAY2000	13:02
		Week 3 (Fol. Up)	29MAY2000	No		
		Week 4 (Fol. Up)	04JUN2000	No		
		Week 5 (Fol. Up)	12JUN2000	No		
		Week 6 (Fol. Up)	19JUN2000	No		
		Week 6 (Unsch. Fol. Up)	21JUN2000	No		
030.001.00042	IPP	Screening	10APR2000	No		
		Screening (Unsch.)	17APR2000	No		
		Screening (Unsch.)	27APR2000	No		
		Clearance (Day -5)	03MAY2000	Yes	03MAY2000	
		Clearance (Day -4)	04MAY2000	Yes	04MAY2000	
		Clearance (Day -3)	05MAY2000	Yes	05MAY2000	
		Clearance (Unsch.)	05MAY2000	No		
		Load Dose 1	08MAY2000	Yes	08MAY2000	8:40
		Load Dose 2	09MAY2000	Yes	09MAY2000	7:48
		Load Dose 3	10MAY2000	Yes	10MAY2000	7:46
		Load Dose (Unsch.)	09MAY2000	No		
		Week 1 (Unsch.)	14MAY2000	No		
		Week 1	15MAY2000	Yes	15MAY2000	7:15
		Week 2	22MAY2000	Yes	22MAY2000	7:19
		Week 2 (Unsch.)	22MAY2000	No		
		Week 3	29MAY2000	Yes	29MAY2000	6:39
		Week 4	04JUN2000	Yes	04JUN2000	10:42

*Popn=Population (I=Intent-to-Treat, PP=Per Protocol)
Tafenoquine = Tafenoquine 200mg, Mefloquine = Mefloquine 250mg

SB252263 - 030

Listing B.02

Visit And Dosing Dates

Treatment : Tafenoquine

Subject Number	Popn*	Visit	Visit Date	Dose Taken ?	Date of Dose	Time of Dose
030.001.00042	IPP	Week 5	12JUN2000	Yes	12JUN2000	7:16
		Week 6	19JUN2000	Yes	19JUN2000	11:02
		Week 7	26JUN2000	Yes	26JUN2000	7:10
		Week 8 (Fol. Up)	03JUL2000	No		
		Week 8 (Fol. Up)	04JUL2000	No		
		Week 8 (Unsch. Fol. Up)	04JUL2000	No		
		Week 9 (Fol. Up)	10JUL2000	No		
		Week 10 (Fol. Up)	18JUL2000	No		
		Week 11 (Unsch. Fol. Up)	22JUL2000	No		
030.001.00043	IPP	Screening	18APR2000	No		
		Screening (Unsch.)	28APR2000	No		
		Clearance (Day -5)	03MAY2000	Yes	03MAY2000	
		Clearance (Day -4)	04MAY2000	Yes	04MAY2000	
		Clearance (Day -3)	05MAY2000	Yes	05MAY2000	
		Clearance (Unsch.)	03MAY2000	No		
		Clearance (Unsch.)	06MAY2000	No		
		Load Dose 1	08MAY2000	Yes	08MAY2000	8:50
		Load Dose 2	09MAY2000	Yes	09MAY2000	9:31
		Load Dose 3	10MAY2000	Yes	10MAY2000	6:40
		Load Dose (Unsch.)	09MAY2000	No		
		Week 1	15MAY2000	Yes	15MAY2000	11:28
		Week 2 (Unsch.)	21MAY2000	No		
		Week 2	22MAY2000	Yes	22MAY2000	6:00
		Week 2 (Unsch. Fol. Up)	23MAY2000	No		
		Week 2 (Unsch. Fol. Up)	25MAY2000	No		
		Week 3 (Fol. Up)	29MAY2000	No		
		Week 3 (Unsch. Fol. Up)	29MAY2000	No		
		Week 4 (Unsch. Fol. Up)	02JUN2000	No		
		Week 4 (Fol. Up)	05JUN2000	No		
		Week 5 (Fol. Up)	12JUN2000	No		
		Week 6 (Fol. Up)	19JUN2000	No		
		Week 6 (Unsch. Fol. Up)	20JUN2000	No		
030.001.00048	IPP	Screening	13APR2000	No		
		Clearance (Day -5)	03MAY2000	Yes	03MAY2000	
		Clearance (Day -4)	04MAY2000	Yes	04MAY2000	
		Clearance (Day -3)	05MAY2000	Yes	05MAY2000	
		Clearance (Unsch.)	05MAY2000	No		
		Load Dose 1	08MAY2000	Yes	08MAY2000	8:52
		Load Dose 2	09MAY2000	Yes	09MAY2000	10:55
		Load Dose 3	10MAY2000	Yes	10MAY2000	20:56

*Popn=Population (I=Intent-to-Treat, PP=Per Protocol)
Tafenoquine = Tafenoquine 200mg, Mefloquine = Mefloquine 250mg

SB252263 - 030

Listing B.02

Visit And Dosing Dates

Treatment : Tafenoquine

Subject Number	Popn*	Visit	Visit Date	Dose Taken ?	Date of Dose	Time of Dose
030.001.00048	IPP	Week 1	15MAY2000	Yes	15MAY2000	8:25
		Week 2 (Unsch.)	21MAY2000	No		
		Week 2	22MAY2000	Yes	22MAY2000	10:30
		Week 3	29MAY2000	Yes	29MAY2000	8:15
		Week 3 (Unsch.)	29MAY2000	No		
		Week 3 (Unsch.)	30MAY2000	No		
		Week 4	04JUN2000	Yes	04JUN2000	12:30
		Week 5	12JUN2000	Yes	12JUN2000	8:28
		Week 6	19JUN2000	Yes	19JUN2000	7:37
		Week 7	26JUN2000	Yes	26JUN2000	7:02
		Week 7 (Unsch. Fol. Up)	29JUN2000	No		
		Week 8 (Fol. Up)	03JUL2000	No		
030.001.00050	IPP	Screening	10APR2000	No		
		Screening (Unsch.)	19APR2000	No		
		Clearance (Day -5)	03MAY2000	Yes	03MAY2000	
		Clearance (Day -4)	04MAY2000	Yes	04MAY2000	
		Clearance (Day -3)	05MAY2000	Yes	05MAY2000	
		Load Dose 1	08MAY2000	Yes	08MAY2000	9:09
		Load Dose 2	09MAY2000	Yes	09MAY2000	7:45
		Load Dose 3	10MAY2000	Yes	10MAY2000	8:30
		Week 1	15MAY2000	Yes	15MAY2000	8:20
		Week 2	22MAY2000	Yes	22MAY2000	6:35
		Week 3 (Unsch.)	26MAY2000	No		
		Week 3	31MAY2000	Yes	31MAY2000	8:17
		Week 4 (Fol. Up)	07JUN2000	No		
		Week 5 (Unsch. Fol. Up)	09JUN2000	No		
		Week 5 (Fol. Up)	12JUN2000	No		
		Week 7 (Fol. Up)	23JUN2000	No		
		Week 7 (Fol. Up)	26JUN2000	No		
		Week 7 (Unsch. Fol. Up)	27JUN2000	No		
030.001.00051	IPP	Screening	11APR2000	No		
		Screening (Unsch.)	19APR2000	No		
		Clearance (Day -5)	03MAY2000	Yes	03MAY2000	
		Clearance (Day -4)	04MAY2000	Yes	04MAY2000	
		Clearance (Day -3)	05MAY2000	Yes	05MAY2000	
		Clearance (Unsch.)	06MAY2000	No		
		Load Dose 1	08MAY2000	Yes	08MAY2000	9:05
		Load Dose 2	09MAY2000	Yes	09MAY2000	6:56
		Load Dose 3	10MAY2000	Yes	10MAY2000	8:06
		Week 1	15MAY2000	Yes	15MAY2000	7:41

*Popn=Population (I=Intent-to-Treat, PP=Per Protocol)
Tafenoquine = Tafenoquine 200mg, Mefloquine = Mefloquine 250mg

SB252263 - 030

Listing B.02

Visit And Dosing Dates

Treatment : Tafenoquine

Subject Number	Popn*	Visit	Visit Date	Dose Taken ?	Date of Dose	Time of Dose
030.001.00051	IPP	Week 2 (Unsch.)	21MAY2000	No		
		Week 2	22MAY2000	Yes	22MAY2000	6:29
		Week 3 (Unsch.)	27MAY2000	No		
		Week 3	29MAY2000	Yes	29MAY2000	7:20
		Week 4	04JUN2000	Yes	04JUN2000	8:48
		Week 5	12JUN2000	Yes	12JUN2000	6:16
		Week 5 (Unsch.)	13JUN2000	No		
		Week 6	19JUN2000	Yes	19JUN2000	5:50
		Week 7	26JUN2000	Yes	26JUN2000	6:30
		Week 8	03JUL2000	Yes	03JUL2000	5:11
		Week 9	10JUL2000	Yes	10JUL2000	6:32
		Week 10	17JUL2000	Yes	17JUL2000	6:12
		Week 11	24JUL2000	Yes	24JUL2000	6:32
		Week 12	31JUL2000	Yes	31JUL2000	7:30
		Week 13 (Unsch.)	04AUG2000	No		
		Week 13	07AUG2000	Yes	07AUG2000	6:42
		Week 14 (Unsch.)	12AUG2000	No		
		Week 14	14AUG2000	Yes	14AUG2000	6:10
		Week 14 (Unsch.)	14AUG2000	No		
		Week 15	21AUG2000	Yes	21AUG2000	6:52
		Week 16	28AUG2000	Yes	28AUG2000	6:52
		Week 16	30AUG2000	No		
		Week 18	11SEP2000	Yes	11SEP2000	
		Week 19	18SEP2000	Yes	18SEP2000	6:24
		Week 20 (Unsch.)	24SEP2000	No		
		Week 20	25SEP2000	Yes	25SEP2000	6:09
		Week 20 (Unsch. Fol. Up)	28SEP2000	No		
		Week 21 (Fol. Up)	02OCT2000	No		
		Week 22 (Fol. Up)	09OCT2000	No		
		Week 22 (Unsch. Fol. Up)	11OCT2000	No		
		Week 23 (Fol. Up)	16OCT2000	No		
		Week 24 (Fol. Up)	23OCT2000	No		
030.001.00058	IPP	Screening	17APR2000	No		
		Clearance (Day -5)	03MAY2000	Yes	03MAY2000	
		Clearance (Day -4)	04MAY2000	Yes	04MAY2000	
		Clearance (Day -3)	05MAY2000	Yes	05MAY2000	
		Clearance (Unsch.)	06MAY2000	No		
		Load Dose 1	08MAY2000	Yes	08MAY2000	9:20
		Load Dose 2	09MAY2000	Yes	09MAY2000	7:57
		Load Dose 3	10MAY2000	Yes	10MAY2000	8:00
		Week 1	15MAY2000	Yes	15MAY2000	7:45
		Week 2	22MAY2000	Yes	22MAY2000	7:21

*Popn=Population (I=Intent-to-Treat, PP=Per Protocol)
Tafenoquine = Tafenoquine 200mg, Mefloquine = Mefloquine 250mg

SB252263 - 030

Listing B.02

Visit And Dosing Dates

Treatment : Tafenoquine

Subject Number	Popn*	Visit	Visit Date	Dose Taken ?	Date of Dose	Time of Dose
030.001.00058	IPP	Week 3	29MAY2000	Yes	29MAY2000	7:40
		Week 3 (Unsch.)	30MAY2000	No		
		Week 4	05JUN2000	Yes	05JUN2000	11:30
		Week 5 (Unsch.)	09JUN2000	No		
		Week 5	12JUN2000	Yes	12JUN2000	7:40
		Week 6	19JUN2000	Yes	19JUN2000	7:58
		Week 7 (Unsch.)	24JUN2000	No		
		Week 7	26JUN2000	Yes	26JUN2000	7:57
		Week 8	03JUL2000	Yes	03JUL2000	7:25
		Week 9	10JUL2000	Yes	10JUL2000	8:33
		Week 10	17JUL2000	Yes	17JUL2000	7:22
		Week 11 (Fol. Up)	24JUL2000	No		
		Week 12 (Fol. Up)	31JUL2000	No		
		Week 12 (Unsch. Fol. Up)	03AUG2000	No		
		Week 13 (Fol. Up)	07AUG2000	No		
		Week 13 (Unsch. Fol. Up)	08AUG2000	No		
		Week 14 (Fol. Up)	14AUG2000	No		
030.001.00060	IPP	Screening	13APR2000	No		
		Clearance (Day -5)	03MAY2000	Yes	03MAY2000	
		Clearance (Day -4)	04MAY2000	Yes	04MAY2000	
		Clearance (Day -3)	05MAY2000	Yes	05MAY2000	
		Clearance (Unsch.)	06MAY2000	No		
		Load Dose 1	08MAY2000	Yes	08MAY2000	9:54
		Load Dose 2	09MAY2000	Yes	09MAY2000	7:14
		Load Dose 3	10MAY2000	Yes	10MAY2000	12:24
		Week 1 (Unsch.)	16MAY2000	No		
		Week 1	17MAY2000	Yes	17MAY2000	6:33
		Week 2	22MAY2000	Yes	22MAY2000	7:22
		Week 3 (Unsch.)	26MAY2000	No		
		Week 3	29MAY2000	Yes	29MAY2000	6:38
		Week 4	05JUN2000	Yes	05JUN2000	14:30
		Week 5	12JUN2000	Yes	12JUN2000	12:10
		Week 6	19JUN2000	Yes	19JUN2000	6:41
		Week 6 (Unsch.)	20JUN2000	No		
		Week 7	26JUN2000	Yes	26JUN2000	7:07
		Week 8	03JUL2000	Yes	03JUL2000	6:08
		Week 9	10JUL2000	Yes	10JUL2000	7:19
		Week 10 (Unsch.)	14JUL2000	No		
		Week 10	17JUL2000	Yes	17JUL2000	7:05
		Week 11 (Fol. Up)	24JUL2000	No		
		Week 12 (Fol. Up)	31JUL2000	No		
		Week 12 (Unsch. Fol. Up)	01AUG2000	No		

*Popn=Population (I=Intent-to-Treat, PP=Per Protocol)
Tafenoquine = Tafenoquine 200mg, Mefloquine = Mefloquine 250mg

SB252263 - 030

Listing B.02

Visit And Dosing Dates

Treatment : Tafenoquine

Subject Number	Popn*	Visit	Visit Date	Dose Taken ?	Date of Dose	Time of Dose
030.001.00060	IPP	Week 13 (Fol. Up)	08AUG2000	No		
		Week 13 (Fol. Up)	10AUG2000	No		
		Week 14 (Fol. Up)	14AUG2000	No		
030.001.00061	IPP	Screening	14APR2000	No		
		Clearance (Day -5)	03MAY2000	Yes	03MAY2000	
		Clearance (Day -4)	04MAY2000	Yes	04MAY2000	
		Clearance (Day -3)	05MAY2000	Yes	05MAY2000	
		Load Dose 1	08MAY2000	Yes	08MAY2000	9:25
		Load Dose 2	09MAY2000	Yes	09MAY2000	6:11
		Load Dose 3	10MAY2000	Yes	10MAY2000	9:25
		Week 1	15MAY2000	Yes	15MAY2000	8:15
		Week 2	22MAY2000	Yes	22MAY2000	8:45
		Week 3 (Unsch.)	26MAY2000	No		
		Week 3	29MAY2000	Yes	29MAY2000	9:45
		Week 4	05JUN2000	Yes	05JUN2000	6:39
		Week 5	12JUN2000	Yes	12JUN2000	9:30
		Week 6	20JUN2000	Yes	20JUN2000	12:30
		Week 7	26JUN2000	Yes	26JUN2000	10:05
		Week 8	03JUL2000	Yes	03JUL2000	6:00
		Week 9	10JUL2000	Yes	10JUL2000	6:05
		Week 10 (Fol. Up)	17JUL2000	No		
		Week 10 (Unsch. Fol. Up)	19JUL2000	No		
		Week 11 (Fol. Up)	25JUL2000	No		
		Week 12 (Fol. Up)	31JUL2000	No		
030.001.00065	IPP	Screening	11APR2000	No		
		Screening (Unsch.)	25APR2000	No		
		Clearance (Day -5)	03MAY2000	Yes	03MAY2000	
		Clearance (Day -4)	04MAY2000	Yes	04MAY2000	
		Clearance (Day -3)	05MAY2000	Yes	05MAY2000	
		Load Dose 1	08MAY2000	Yes	08MAY2000	9:41
		Load Dose 2	09MAY2000	Yes	09MAY2000	8:14
		Load Dose 3	10MAY2000	Yes	10MAY2000	9:05
		Week 1	15MAY2000	Yes	15MAY2000	8:56
		Week 1 (Unsch.)	15MAY2000	No		
		Week 2	22MAY2000	Yes	22MAY2000	6:48
		Week 3	29MAY2000	Yes	29MAY2000	7:50
		Week 4	05JUN2000	Yes	05JUN2000	11:09
		Week 5	12JUN2000	Yes	12JUN2000	7:20
		Week 5 (Unsch.)	14JUN2000	No		
		Week 6	19JUN2000	Yes	19JUN2000	6:00

*Popn=Population (I=Intent-to-Treat, PP=Per Protocol)
Tafenoquine = Tafenoquine 200mg, Mefloquine = Mefloquine 250mg

SB252263 - 030

Listing B.02

Visit And Dosing Dates

Treatment : Tafenoquine

Subject Number	Popn*	Visit	Visit Date	Dose Taken ?	Date of Dose	Time of Dose
030.001.00065	IPP	Week 7	26JUN2000	Yes	26JUN2000	6:22
		Week 8 (Fol. Up)	03JUL2000	No		
		Week 9 (Fol. Up)	10JUL2000	No		
		Week 10 (Fol. Up)	17JUL2000	No		
		Week 11 (Fol. Up)	24JUL2000	No		
030.001.00068	IPP	Screening	14APR2000	No		
		Clearance (Day -5)	03MAY2000	Yes	03MAY2000	
		Clearance (Day -4)	04MAY2000	Yes	04MAY2000	
		Clearance (Day -3)	05MAY2000	Yes	05MAY2000	
		Clearance (Unsch.)	04MAY2000	No		
		Load Dose 1	08MAY2000	Yes	08MAY2000	9:55
		Load Dose 2	09MAY2000	Yes	09MAY2000	9:00
		Load Dose 3	10MAY2000	Yes	10MAY2000	8:19
		Week 1 (Unsch.)	11MAY2000	No		
		Week 1	15MAY2000	Yes	15MAY2000	9:26
		Week 1 (Unsch.)	16MAY2000	No		
		Week 2	23MAY2000	Yes	23MAY2000	10:10
		Week 3	30MAY2000	Yes	30MAY2000	14:40
		Week 4	06JUN2000	Yes	06JUN2000	8:01
		Week 5	13JUN2000	Yes	13JUN2000	12:35
		Week 6	22JUN2000	Yes	22JUN2000	11:58
		Week 7	27JUN2000	Yes	27JUN2000	12:01
		Week 8	04JUL2000	Yes	04JUL2000	6:05
		Week 9	11JUL2000	Yes	11JUL2000	12:01
		Week 10 (Fol. Up)	17JUL2000	No		
		Week 10 (Unsch. Fol. Up)	18JUL2000	No		
		Week 11 (Fol. Up)	25JUL2000	No		
		Week 12 (Fol. Up)	01AUG2000	No		
		Week 12 (Unsch. Fol. Up)	01AUG2000	No		
		Week 13 (Fol. Up)	07AUG2000	No		
030.001.00070	IPP	Screening	11APR2000	No		
		Clearance (Day -5)	03MAY2000	Yes	03MAY2000	
		Clearance (Day -4)	04MAY2000	Yes	04MAY2000	
		Clearance (Day -3)	05MAY2000	Yes	05MAY2000	
		Clearance (Unsch.)	06MAY2000	No		
		Load Dose 1	08MAY2000	Yes	08MAY2000	10:30
		Load Dose 2	09MAY2000	Yes	09MAY2000	6:50
		Load Dose 3	10MAY2000	Yes	10MAY2000	8:30
		Week 1	15MAY2000	Yes	15MAY2000	9:30
		Week 2	22MAY2000	Yes	22MAY2000	8:00

*Popn=Population (I=Intent-to-Treat, PP=Per Protocol)
Tafenoquine = Tafenoquine 200mg, Mefloquine = Mefloquine 250mg

SB252263 - 030

Listing B.02

Visit And Dosing Dates

Treatment : Tafenoquine

Subject Number	Popn*	Visit	Visit Date	Dose Taken ?	Date of Dose	Time of Dose
030.001.00070	IPP	Week 3	29MAY2000	Yes	29MAY2000	8:30
		Week 3 (Unsch.)	29MAY2000	No		
		Week 4	05JUN2000	Yes	05JUN2000	9:16
		Week 5	12JUN2000	Yes	12JUN2000	7:47
		Week 6	19JUN2000	Yes	19JUN2000	17:31
		Week 7	26JUN2000	Yes	26JUN2000	8:04
		Week 7 (Unsch.)	28JUN2000	No		
		Week 8	03JUL2000	Yes	03JUL2000	9:55
		Week 9 (Fol. Up)	10JUL2000	No		
		Week 10 (Fol. Up)	17JUL2000	No		
		Week 10 (Unsch. Fol. Up)	18JUL2000	No		
		Week 11 (Fol. Up)	24JUL2000	No		
		Week 11 (Unsch. Fol. Up)	27JUL2000	No		
		Week 12 (Fol. Up)	31JUL2000	No		
030.001.00077	IPP	Screening	11APR2000	No		
		Screening (Unsch.)	28APR2000	No		
		Clearance (Day -5)	03MAY2000	Yes	03MAY2000	
		Clearance (Day -4)	04MAY2000	Yes	04MAY2000	
		Clearance (Day -3)	05MAY2000	Yes	05MAY2000	
		Load Dose 1	08MAY2000	Yes	08MAY2000	10:15
		Load Dose 2	09MAY2000	Yes	09MAY2000	7:40
		Load Dose 3	10MAY2000	Yes	10MAY2000	11:55
		Week 1	15MAY2000	Yes	15MAY2000	8:15
		Week 2	22MAY2000	Yes	22MAY2000	6:50
		Week 3	29MAY2000	Yes	29MAY2000	7:40
		Week 4	05JUN2000	Yes	05JUN2000	8:05
		Week 5	12JUN2000	Yes	12JUN2000	16:20
		Week 5 (Unsch.)	15JUN2000	No		
		Week 6	19JUN2000	Yes	19JUN2000	17:21
		Week 7	26JUN2000	Yes	26JUN2000	6:19
		Week 8	03JUL2000	Yes	03JUL2000	7:52
		Week 9 (Fol. Up)	10JUL2000	No		
		Week 10 (Fol. Up)	17JUL2000	No		
		Week 11 (Fol. Up)	24JUL2000	No		
030.001.00079	IPP	Screening	14APR2000	No		
		Screening (Unsch.)	21APR2000	No		
		Screening (Unsch.)	24APR2000	No		
		Clearance (Day -5)	03MAY2000	Yes	03MAY2000	
		Clearance (Day -4)	04MAY2000	Yes	04MAY2000	
		Clearance (Day -3)	05MAY2000	Yes	05MAY2000	

*Popn=Population (I=Intent-to-Treat, PP=Per Protocol)
Tafenoquine = Tafenoquine 200mg, Mefloquine = Mefloquine 250mg

SB252263 - 030

Listing B.02

Visit And Dosing Dates

Treatment : Tafenoquine

Subject Number	Popn*	Visit	Visit Date	Dose Taken ?	Date of Dose	Time of Dose
030.001.00079	IPP	Clearance (Unsch.)	06MAY2000	No		
		Load Dose 1	08MAY2000	Yes	08MAY2000	10:25
		Load Dose 2	09MAY2000	Yes	09MAY2000	7:32
		Load Dose 3	10MAY2000	Yes	10MAY2000	8:26
		Load Dose (Unsch.)	10MAY2000	No		
		Week 1 (Unsch.)	13MAY2000	No		
		Week 1	18MAY2000	Yes	18MAY2000	13:42
		Week 2 (Unsch.)	21MAY2000	No		
		Week 2	22MAY2000	Yes	22MAY2000	7:55
		Week 3	29MAY2000	Yes	29MAY2000	7:39
		Week 4	06JUN2000	Yes	06JUN2000	9:00
		Week 5	12JUN2000	Yes	12JUN2000	8:05
		Week 6	19JUN2000	Yes	19JUN2000	7:25
		Week 7	26JUN2000	Yes	26JUN2000	8:20
		Week 8	03JUL2000	Yes	03JUL2000	6:31
		Week 9 (Fol. Up)	10JUL2000	No		
		Week 10 (Unsch. Fol. Up)	16JUL2000	No		
		Week 10 (Fol. Up)	17JUL2000	No		
		Week 11 (Fol. Up)	24JUL2000	No		
030.001.00082		Screening	18APR2000	No		
		Clearance (Day -5)	03MAY2000	Yes	03MAY2000	
		Clearance (Day -4)	04MAY2000	Yes	04MAY2000	
		Clearance (Day -3)	05MAY2000	Yes	05MAY2000	
		Load Dose 1	08MAY2000	Yes	08MAY2000	10:50
		Load Dose 2	09MAY2000	Yes	09MAY2000	10:33
		Load Dose 3	10MAY2000	Yes	10MAY2000	9:53
		Week 1 (Fol. Up)	15MAY2000	No		
		Week 2 (Fol. Up)	22MAY2000	No		
		Week 3 (Fol. Up)	29MAY2000	No		
030.001.00084	IPP	Screening	13APR2000	No		
		Clearance (Day -5)	03MAY2000	Yes	03MAY2000	
		Clearance (Day -4)	04MAY2000	Yes	04MAY2000	
		Clearance (Day -3)	05MAY2000	Yes	05MAY2000	
		Load Dose 1	08MAY2000	Yes	08MAY2000	10:55
		Load Dose 2	09MAY2000	Yes	09MAY2000	7:06
		Load Dose 3	10MAY2000	Yes	10MAY2000	11:01
		Load Dose (Unsch.)	10MAY2000	No		
		Week 1	15MAY2000	Yes	15MAY2000	7:20
		Week 2	22MAY2000	Yes	22MAY2000	7:25
		Week 2 (Unsch.)	22MAY2000	No		

*Popn=Population (I=Intent-to-Treat, PP=Per Protocol)
Tafenoquine = Tafenoquine 200mg, Mefloquine = Mefloquine 250mg

SB252263 - 030

Listing B.02

Visit And Dosing Dates

Treatment : Tafenoquine

Subject Number	Popn*	Visit	Visit Date	Dose Taken ?	Date of Dose	Time of Dose
030.001.00084	IPP	Week 3	29MAY2000	Yes	29MAY2000	7:38
		Week 4	05JUN2000	Yes	05JUN2000	9:48
		Week 5	12JUN2000	Yes	12JUN2000	9:45
		Week 6 (Unsch.)	16JUN2000	No		
		Week 6	19JUN2000	Yes	19JUN2000	7:13
		Week 6 (Unsch.)	19JUN2000	No		
		Week 7	26JUN2000	Yes	26JUN2000	8:20
		Week 7 (Unsch.)	29JUN2000	No		
		Week 8	03JUL2000	Yes	03JUL2000	6:48
		Week 9	10JUL2000	Yes	10JUL2000	8:20
		Week 9 (Unsch.)	10JUL2000	No		
		Week 10	17JUL2000	Yes	17JUL2000	11:40
		Week 10 (Unsch.)	18JUL2000	No		
		Week 11	24JUL2000	Yes	24JUL2000	13:00
		Week 12	31JUL2000	Yes	31JUL2000	9:00
		Week 13	07AUG2000	Yes	07AUG2000	6:50
		Week 14	14AUG2000	Yes	14AUG2000	7:10
		Week 15	21AUG2000	Yes	21AUG2000	7:00
		Week 16 (Unsch. Fol. Up)	25AUG2000	No		
		Week 16 (Fol. Up)	28AUG2000	No		
		Week 16 (Unsch. Fol. Up)	31AUG2000	No		
		Week 17 (Fol. Up)	05SEP2000	No		
		Week 18 (Fol. Up)	11SEP2000	No		
		Week 19 (Fol. Up)	19SEP2000	No		
030.001.00085	IPP	Screening	10APR2000	No		
		Clearance (Day -5)	03MAY2000	Yes	03MAY2000	
		Clearance (Day -4)	04MAY2000	Yes	04MAY2000	
		Clearance (Day -3)	05MAY2000	Yes	05MAY2000	
		Load Dose 1	08MAY2000	Yes	08MAY2000	11:29
		Load Dose 2	09MAY2000	Yes	09MAY2000	7:20
		Load Dose 3	10MAY2000	Yes	10MAY2000	8:48
		Load Dose (Unsch.)	10MAY2000	No		
		Week 1	15MAY2000	Yes	15MAY2000	8:24
		Week 1 (Unsch.)	15MAY2000	No		
		Week 2	22MAY2000	Yes	22MAY2000	7:13
		Week 2 (Unsch.)	22MAY2000	No		
		Week 3	29MAY2000	Yes	29MAY2000	9:32
		Week 4	05JUN2000	Yes	05JUN2000	9:09
		Week 5	12JUN2000	Yes	12JUN2000	9:59
		Week 6	19JUN2000	Yes	19JUN2000	8:39
		Week 7	26JUN2000	Yes	26JUN2000	6:51
		Week 8	03JUL2000	Yes	03JUL2000	8:47

*Popn=Population (I=Intent-to-Treat, PP=Per Protocol)
Tafenoquine = Tafenoquine 200mg, Mefloquine = Mefloquine 250mg

SB252263 - 030

Listing B.02

Visit And Dosing Dates

Treatment : Tafenoquine

Subject Number	Popn*	Visit	Visit Date	Dose Taken ?	Date of Dose	Time of Dose
030.001.00085	IPP	Week 9	10JUL2000	Yes	10JUL2000	8:11
		Week 10	17JUL2000	Yes	17JUL2000	8:36
		Week 10 (Unsch.)	20JUL2000	No		
		Week 11	24JUL2000	Yes	24JUL2000	8:09
		Week 12	01AUG2000	Yes	01AUG2000	9:03
		Week 13	07AUG2000	Yes	07AUG2000	7:51
		Week 14 (Fol. Up)	14AUG2000	No		
		Week 14 (Unsch. Fol. Up)	14AUG2000	No		
		Week 15 (Fol. Up)	21AUG2000	No		
		Week 16 (Fol. Up)	28AUG2000	No		
		Week 17 (Fol. Up)	04SEP2000	No		
		Week 17 (Unsch. Fol. Up)	04SEP2000	No		
030.001.00086	IPP	Screening	11APR2000	No		
		Clearance (Day -5)	03MAY2000	Yes	03MAY2000	
		Clearance (Day -4)	04MAY2000	Yes	04MAY2000	
		Clearance (Day -3)	05MAY2000	Yes	05MAY2000	
		Load Dose 1	08MAY2000	Yes	08MAY2000	11:13
		Load Dose 2	09MAY2000	Yes	09MAY2000	6:45
		Load Dose 3	10MAY2000	Yes	10MAY2000	16:13
		Week 1	15MAY2000	Yes	15MAY2000	9:44
		Week 2	22MAY2000	Yes	22MAY2000	6:49
		Week 2 (Unsch. Fol. Up)	23MAY2000	No		
		Week 3 (Fol. Up)	29MAY2000	No		
		Week 3 (Unsch. Fol. Up)	29MAY2000	No		
		Week 4 (Fol. Up)	05JUN2000	No		
		Week 5 (Fol. Up)	12JUN2000	No		
		Week 6 (Fol. Up)	19JUN2000	No		
		Week 6 (Unsch. Fol. Up)	21JUN2000	No		
030.001.00093	IPP	Screening	10APR2000	No		
		Clearance (Day -5)	03MAY2000	Yes	03MAY2000	
		Clearance (Day -4)	04MAY2000	Yes	04MAY2000	
		Clearance (Day -3)	05MAY2000	Yes	05MAY2000	
		Load Dose 1	08MAY2000	Yes	08MAY2000	12:35
		Load Dose 2	09MAY2000	Yes	09MAY2000	6:25
		Load Dose 3	10MAY2000	Yes	10MAY2000	7:35
		Week 1	15MAY2000	Yes	15MAY2000	7:25
		Week 2	22MAY2000	Yes	22MAY2000	7:35
		Week 3	29MAY2000	Yes	29MAY2000	5:40
		Week 3 (Unsch.)	30MAY2000	No		
		Week 4 (Unsch.)	03JUN2000	No		

*Popn=Population (I=Intent-to-Treat, PP=Per Protocol)
Tafenoquine = Tafenoquine 200mg, Mefloquine = Mefloquine 250mg

SB252263 - 030

Listing B.02

Visit And Dosing Dates

Treatment : Tafenoquine

Subject Number	Popn*	Visit	Visit Date	Dose Taken ?	Date of Dose	Time of Dose
030.001.00093	IPP	Week 4	06JUN2000	Yes	06JUN2000	14:12
		Week 5	12JUN2000	Yes	12JUN2000	6:09
		Week 6 (Unsch.)	16JUN2000	No		
		Week 6	19JUN2000	Yes	19JUN2000	5:27
		Week 7 (Unsch.)	26JUN2000	No		
		Week 7	27JUN2000	Yes	27JUN2000	11:20
		Week 8	03JUL2000	Yes	03JUL2000	6:25
		Week 9	10JUL2000	Yes	10JUL2000	6:10
		Week 9 (Unsch.)	11JUL2000	No		
		Week 9 (Unsch.)	12JUL2000	No		
		Week 10	17JUL2000	Yes	17JUL2000	6:12
		Week 11	24JUL2000	Yes	24JUL2000	6:05
		Week 12	31JUL2000	Yes	31JUL2000	15:00
		Week 13	10AUG2000	Yes	10AUG2000	9:10
		Week 14	15AUG2000	Yes	15AUG2000	12:50
		Week 15	21AUG2000	Yes	21AUG2000	10:30
		Week 16	29AUG2000	Yes	29AUG2000	14:35
		Week 16 (Unsch.)	31AUG2000	No		
		Week 17	04SEP2000	Yes	04SEP2000	6:45
		Week 18 (Fol. Up)	11SEP2000	No		
		Week 19 (Fol. Up)	20SEP2000	No		
		Week 20 (Fol. Up)	25SEP2000	No		
		Week 20 (Unsch. Fol. Up)	25SEP2000	No		
030.001.00094	I	Screening	11APR2000	No		
		Screening (Unsch.)	15APR2000	No		
		Clearance (Day -5)	03MAY2000	Yes	03MAY2000	
		Clearance (Day -4)	04MAY2000	Yes	04MAY2000	
		Clearance (Day -3)	05MAY2000	Yes	05MAY2000	
		Load Dose 1	08MAY2000	Yes	08MAY2000	12:20
		Load Dose 2	09MAY2000	Yes	09MAY2000	11:59
		Load Dose 3	10MAY2000	Yes	10MAY2000	16:00
		Week 1	15MAY2000	Yes	15MAY2000	12:00
		Week 2 (Unsch.)	21MAY2000	No		
		Week 2	22MAY2000	Yes	22MAY2000	9:55
		Week 3	29MAY2000	Yes	29MAY2000	10:00
		Week 4 (Unsch.)	03JUN2000	No		
		Week 4	07JUN2000	Yes	07JUN2000	
		Week 5 (Unsch.)	11JUN2000	No		
		Week 5	13JUN2000	Yes	13JUN2000	10:20
		Week 6	19JUN2000	Yes	19JUN2000	17:15
		Week 7	26JUN2000	Yes	26JUN2000	13:00
		Week 8 (Unsch.)	02JUL2000	No		

*Popn=Population (I=Intent-to-Treat, PP=Per Protocol)
Tafenoquine = Tafenoquine 200mg, Mefloquine = Mefloquine 250mg

SB252263 - 030

Listing B.02

Visit And Dosing Dates

Treatment : Tafenoquine

Subject Number	Popn*	Visit	Visit Date	Dose Taken ?	Date of Dose	Time of Dose
030.001.00094	I	Week 8	03JUL2000	Yes	03JUL2000	6:35
		Week 8	04JUL2000	No		
		Week 8 (Unsch.)	05JUL2000	No		
		Week 9	10JUL2000	Yes	10JUL2000	10:01
		Week 10	17JUL2000	Yes	17JUL2000	6:45
		Week 11	24JUL2000	Yes	24JUL2000	7:59
		Week 12	31JUL2000	Yes	31JUL2000	6:51
		Week 13	07AUG2000	Yes	07AUG2000	6:33
		Week 14 (Unsch.)	11AUG2000	No		
		Week 14	14AUG2000	Yes	14AUG2000	8:45
		Week 15	23AUG2000	Yes	23AUG2000	17:05
		Week 15 (Unsch.)	24AUG2000	No		
		Week 16	30AUG2000	Yes	30AUG2000	13:46
		Week 17 (Fol. Up)	01SEP2000	No		
		Week 18 (Fol. Up)	09SEP2000	No		
		Week 18 (Fol. Up)	14SEP2000	No		
		Week 19 (Fol. Up)	18SEP2000	No		
030.001.00100	IPP	Screening	12APR2000	No		
		Clearance (Day -5)	03MAY2000	Yes	03MAY2000	
		Clearance (Day -4)	04MAY2000	Yes	04MAY2000	
		Clearance (Day -3)	05MAY2000	Yes	05MAY2000	
		Load Dose 1	08MAY2000	Yes	08MAY2000	13:40
		Load Dose 2	09MAY2000	Yes	09MAY2000	6:30
		Load Dose 3	10MAY2000	Yes	10MAY2000	13:15
		Week 1	15MAY2000	Yes	15MAY2000	12:10
		Week 1 (Unsch.)	16MAY2000	No		
		Week 2	22MAY2000	Yes	22MAY2000	6:25
		Week 3	29MAY2000	Yes	29MAY2000	6:30
		Week 3 (Unsch.)	01JUN2000	No		
		Week 4	04JUN2000	Yes	04JUN2000	9:10
		Week 5	12JUN2000	Yes	12JUN2000	6:45
		Week 6	19JUN2000	Yes	19JUN2000	7:00
		Week 6 (Unsch.)	19JUN2000	No		
		Week 6 (Unsch.)	20JUN2000	No		
		Week 7	26JUN2000	Yes	26JUN2000	6:30
		Week 8 (Fol. Up)	03JUL2000	No		
		Week 8 (Fol. Up)	04JUL2000	No		
		Week 9 (Fol. Up)	10JUL2000	No		
		Week 10 (Fol. Up)	17JUL2000	No		
		Week 10 (Unsch. Fol. Up)	17JUL2000	No		
		Week 10 (Unsch. Fol. Up)	18JUL2000	No		
		Week 11 (Fol. Up)	25JUL2000	No		

*Popn=Population (I=Intent-to-Treat, PP=Per Protocol)
Tafenoquine = Tafenoquine 200mg, Mefloquine = Mefloquine 250mg

SB252263 - 030

Listing B.02

Visit And Dosing Dates

Treatment : Tafenoquine

Subject Number	Popn*	Visit	Visit Date	Dose Taken ?	Date of Dose	Time of Dose
030.001.00102	IPP	Screening	13APR2000	No		
		Clearance (Day -5)	03MAY2000	Yes	03MAY2000	
		Clearance (Day -4)	04MAY2000	Yes	04MAY2000	
		Clearance (Day -3)	05MAY2000	Yes	05MAY2000	
		Load Dose 1	08MAY2000	Yes	08MAY2000	13:37
		Load Dose 2	09MAY2000	Yes	09MAY2000	7:05
		Load Dose 3	10MAY2000	Yes	10MAY2000	19:50
		Week 1	15MAY2000	Yes	15MAY2000	7:20
		Week 2 (Unsch.)	19MAY2000	No		
		Week 2	22MAY2000	Yes	22MAY2000	7:00
		Week 3	29MAY2000	Yes	29MAY2000	7:12
		Week 4	04JUN2000	Yes	04JUN2000	13:00
		Week 5	12JUN2000	Yes	12JUN2000	7:06
		Week 5 (Unsch.)	15JUN2000	No		
		Week 6	19JUN2000	Yes	19JUN2000	7:19
		Week 7	26JUN2000	Yes	26JUN2000	7:05
		Week 8	03JUL2000	Yes	03JUL2000	7:30
		Week 9	10JUL2000	Yes	10JUL2000	7:30
		Week 10 (Fol. Up)	17JUL2000	No		
		Week 11 (Unsch. Fol. Up)	22JUL2000	No		
		Week 11 (Fol. Up)	25JUL2000	No		
		Week 12 (Unsch. Fol. Up)	29JUL2000	No		
		Week 12 (Fol. Up)	31JUL2000	No		
		Week 12 (Unsch. Fol. Up)	31JUL2000	No		
		Week 13 (Fol. Up)	07AUG2000	No		
030.001.00104	IPP	Screening	14APR2000	No		
		Clearance (Day -5)	03MAY2000	Yes	03MAY2000	
		Clearance (Day -4)	04MAY2000	Yes	04MAY2000	
		Clearance (Day -3)	05MAY2000	Yes	05MAY2000	
		Load Dose 1	08MAY2000	Yes	08MAY2000	14:15
		Load Dose 2	09MAY2000	Yes	09MAY2000	8:15
		Load Dose 3	10MAY2000	Yes	10MAY2000	9:55
		Week 1 (Unsch.)	15MAY2000	No		
		Week 1	16MAY2000	Yes	16MAY2000	8:49
		Week 2	22MAY2000	Yes	22MAY2000	16:20
		Week 2 (Unsch.)	24MAY2000	No		
		Week 3	29MAY2000	Yes	29MAY2000	17:31
		Week 4	05JUN2000	Yes	05JUN2000	13:55
		Week 5	12JUN2000	Yes	12JUN2000	13:55
		Week 6	19JUN2000	Yes	19JUN2000	16:20
		Week 7	26JUN2000	Yes	26JUN2000	12:30
		Week 7 (Unsch. Fol. Up)	28JUN2000	No		

*Popn=Population (I=Intent-to-Treat, PP=Per Protocol)
Tafenoquine = Tafenoquine 200mg, Mefloquine = Mefloquine 250mg

SB252263 - 030

Listing B.02

Visit And Dosing Dates

Treatment : Tafenoquine

Subject Number	Popn*	Visit	Visit Date	Dose Taken ?	Date of Dose	Time of Dose
030.001.00104	IPP	Week 8 (Fol. Up)	03JUL2000	No		
		Week 9 (Fol. Up)	12JUL2000	No		
		Week 10 (Fol. Up)	17JUL2000	No		
		Week 11 (Fol. Up)	25JUL2000	No		
030.001.00107	IPP	Screening	12APR2000	No		
		Screening (Unsch.)	21APR2000	No		
		Screening (Unsch.)	01MAY2000	No		
		Clearance (Day -5)	03MAY2000	Yes	03MAY2000	
		Clearance (Day -4)	04MAY2000	Yes	04MAY2000	
		Clearance (Day -3)	05MAY2000	Yes	05MAY2000	
		Clearance (Unsch.)	04MAY2000	No		
		Load Dose 1	08MAY2000	Yes	08MAY2000	15:30
		Load Dose 2	09MAY2000	Yes	09MAY2000	7:20
		Load Dose 3	10MAY2000	Yes	10MAY2000	8:55
		Week 1	15MAY2000	Yes	15MAY2000	9:30
		Week 2	22MAY2000	Yes	22MAY2000	9:00
		Week 3	29MAY2000	Yes	29MAY2000	8:00
		Week 4	06JUN2000	Yes	06JUN2000	11:25
		Week 5	12JUN2000	Yes	12JUN2000	9:45
		Week 6	19JUN2000	Yes	19JUN2000	10:10
		Week 6 (Unsch.)	20JUN2000	No		
		Week 7	26JUN2000	Yes	26JUN2000	7:40
		Week 8	04JUL2000	Yes	04JUL2000	12:32
		Week 9	10JUL2000	Yes	10JUL2000	7:22
		Week 10	17JUL2000	Yes	17JUL2000	6:52
		Week 11	24JUL2000	Yes	24JUL2000	7:05
		Week 12	01AUG2000	Yes	01AUG2000	11:15
		Week 13	07AUG2000	Yes	07AUG2000	7:02
		Week 15 (Fol. Up)	20AUG2000	No		
		Week 15 (Fol. Up)	21AUG2000	No		
		Week 15 (Unsch. Fol. Up)	22AUG2000	No		
		Week 16 (Fol. Up)	28AUG2000	No		
		Week 17 (Fol. Up)	04SEP2000	No		
030.001.00109	IPP	Screening	11APR2000	No		
		Clearance (Day -5)	03MAY2000	Yes	03MAY2000	
		Clearance (Day -4)	04MAY2000	Yes	04MAY2000	
		Clearance (Day -3)	05MAY2000	Yes	05MAY2000	
		Load Dose 1	08MAY2000	Yes	08MAY2000	15:00
		Load Dose 2	09MAY2000	Yes	09MAY2000	15:17
		Load Dose 3	10MAY2000	Yes	10MAY2000	8:25

*Popn=Population (I=Intent-to-Treat, PP=Per Protocol)
Tafenoquine = Tafenoquine 200mg, Mefloquine = Mefloquine 250mg

SB252263 - 030

Listing B.02

Visit And Dosing Dates

Treatment : Tafenoquine

Subject Number	Popn*	Visit	Visit Date	Dose Taken ?	Date of Dose	Time of Dose
030.001.00109	IPP	Week 1	16MAY2000	Yes	16MAY2000	11:00
		Week 2	22MAY2000	Yes	22MAY2000	8:50
		Week 3	29MAY2000	Yes	29MAY2000	6:50
		Week 4 (Unsch.)	02JUN2000	No		
		Week 4	07JUN2000	Yes	07JUN2000	8:00
		Week 5 (Unsch.)	11JUN2000	No		
		Week 5	12JUN2000	Yes	12JUN2000	6:41
		Week 6	19JUN2000	Yes	19JUN2000	7:45
		Week 6 (Unsch.)	19JUN2000	No		
		Week 7	26JUN2000	Yes	26JUN2000	6:55
		Week 8	03JUL2000	Yes	03JUL2000	6:49
		Week 9	10JUL2000	Yes	10JUL2000	6:54
		Week 9 (Unsch.)	10JUL2000	No		
		Week 10	17JUL2000	Yes	17JUL2000	7:11
		Week 11	24JUL2000	Yes	24JUL2000	7:05
		Week 12 (Fol. Up)	31JUL2000	No		
		Week 13 (Fol. Up)	07AUG2000	No		
		Week 13 (Unsch. Fol. Up)	10AUG2000	No		
		Week 14 (Fol. Up)	14AUG2000	No		
		Week 15 (Unsch. Fol. Up)	21AUG2000	No		
030.001.00111	IPP	Screening	11APR2000	No		
		Clearance (Day -5)	03MAY2000	Yes	03MAY2000	
		Clearance (Day -4)	04MAY2000	Yes	04MAY2000	
		Clearance (Day -3)	05MAY2000	Yes	05MAY2000	
		Load Dose 1	08MAY2000	Yes	08MAY2000	15:10
		Load Dose 2	09MAY2000	Yes	09MAY2000	13:12
		Load Dose 3	10MAY2000	Yes	10MAY2000	13:34
		Week 1	16MAY2000	Yes	16MAY2000	10:43
		Week 2	23MAY2000	Yes	23MAY2000	7:06
		Week 3	29MAY2000	Yes	29MAY2000	7:10
		Week 4	05JUN2000	Yes	05JUN2000	7:55
		Week 5 (Unsch.)	11JUN2000	No		
		Week 5	12JUN2000	Yes	12JUN2000	7:02
		Week 6	19JUN2000	Yes	19JUN2000	7:03
		Week 7	26JUN2000	Yes	26JUN2000	7:19
		Week 8	03JUL2000	Yes	03JUL2000	7:32
		Week 9	10JUL2000	Yes	10JUL2000	7:35
		Week 10	17JUL2000	Yes	17JUL2000	7:40
		Week 11	24JUL2000	Yes	24JUL2000	7:45
		Week 12	01AUG2000	Yes	01AUG2000	10:55
		Week 13	07AUG2000	Yes	07AUG2000	8:00
		Week 14	14AUG2000	Yes	14AUG2000	7:45

*Popn=Population (I=Intent-to-Treat, PP=Per Protocol)
Tafenoquine = Tafenoquine 200mg, Mefloquine = Mefloquine 250mg

SB252263 - 030

Listing B.02

Visit And Dosing Dates

Treatment : Tafenoquine

Subject Number	Popn*	Visit	Visit Date	Dose Taken ?	Date of Dose	Time of Dose
030.001.00111	IPP	Week 15	21AUG2000	Yes	21AUG2000	7:40
		Week 16	29AUG2000	Yes	29AUG2000	12:58
		Week 16	31AUG2000	No		
		Week 17	04SEP2000	Yes	04SEP2000	6:00
		Week 18	13SEP2000	Yes	13SEP2000	17:25
		Week 19	21SEP2000	Yes	21SEP2000	13:00
		Week 20	27SEP2000	Yes	27SEP2000	11:53
		Week 21	03OCT2000	Yes	03OCT2000	13:20
		Week 22 (Fol. Up)	11OCT2000	No		
		Week 22 (Unsch. Fol. Up)	12OCT2000	No		
		Week 23 (Fol. Up)	17OCT2000	No		
030.001.00112	IPP	Screening	13APR2000	No		
		Clearance (Day -5)	02MAY2000	Yes	02MAY2000	
		Clearance (Day -4)	03MAY2000	Yes	03MAY2000	
		Clearance (Day -3)	04MAY2000	Yes	04MAY2000	
		Load Dose 1	08MAY2000	Yes	08MAY2000	15:50
		Load Dose 2	09MAY2000	Yes	09MAY2000	6:57
		Load Dose 3	10MAY2000	Yes	10MAY2000	13:14
		Week 1	15MAY2000	Yes	15MAY2000	7:41
		Week 2	22MAY2000	Yes	22MAY2000	6:30
		Week 3	29MAY2000	Yes	29MAY2000	6:39
		Week 3 (Unsch.)	29MAY2000	No		
		Week 3 (Unsch.)	30MAY2000	No		
		Week 4	04JUN2000	Yes	04JUN2000	12:59
		Week 5	12JUN2000	Yes	12JUN2000	6:57
		Week 6	19JUN2000	Yes	19JUN2000	6:57
		Week 7	26JUN2000	Yes	26JUN2000	7:22
		Week 8 (Fol. Up)	03JUL2000	No		
		Week 9 (Fol. Up)	10JUL2000	No		
		Week 9 (Unsch. Fol. Up)	13JUL2000	No		
		Week 10 (Fol. Up)	17JUL2000	No		
030.001.00118	IPP	Screening	13APR2000	No		
		Clearance (Day -5)	04MAY2000	Yes	04MAY2000	
		Clearance (Day -4)	05MAY2000	Yes	05MAY2000	
		Clearance (Day -3)	06MAY2000	Yes	06MAY2000	
		Load Dose 1	09MAY2000	Yes	09MAY2000	10:50
		Load Dose 2	10MAY2000	Yes	10MAY2000	9:44
		Load Dose 3	11MAY2000	Yes	11MAY2000	6:23
		Week 1	15MAY2000	Yes	15MAY2000	12:18
		Week 2	22MAY2000	Yes	22MAY2000	11:09

*Popn=Population (I=Intent-to-Treat, PP=Per Protocol)
Tafenoquine = Tafenoquine 200mg, Mefloquine = Mefloquine 250mg

SB252263 - 030

Listing B.02

Visit And Dosing Dates

Treatment : Tafenoquine

Subject Number	Popn*	Visit	Visit Date	Dose Taken ?	Date of Dose	Time of Dose
030.001.00118	IPP	Week 3	29MAY2000	Yes	29MAY2000	11:38
		Week 4	07JUN2000	Yes	07JUN2000	9:25
		Week 4 (Unsch.)	07JUN2000	No		
		Week 5	12JUN2000	Yes	12JUN2000	10:30
		Week 6	19JUN2000	Yes	19JUN2000	5:52
		Week 7	26JUN2000	Yes	26JUN2000	10:21
		Week 8 (Fol. Up)	03JUL2000	No		
		Week 9 (Fol. Up)	10JUL2000	No		
		Week 10 (Fol. Up)	17JUL2000	No		
030.001.00122	IPP	Screening	12APR2000	No		
		Clearance (Day -5)	04MAY2000	Yes	04MAY2000	
		Clearance (Day -4)	05MAY2000	Yes	05MAY2000	
		Clearance (Day -3)	06MAY2000	Yes	06MAY2000	
		Load Dose 1	09MAY2000	Yes	09MAY2000	6:49
		Load Dose 2	10MAY2000	Yes	10MAY2000	7:15
		Load Dose 3	11MAY2000	Yes	11MAY2000	7:00
		Week 1	15MAY2000	Yes	15MAY2000	9:15
		Week 2	22MAY2000	Yes	22MAY2000	8:50
		Week 2 (Unsch. Fol. Up)	26MAY2000	No		
		Week 3 (Fol. Up)	29MAY2000	No		
		Week 4 (Fol. Up)	05JUN2000	No		
		Week 5 (Fol. Up)	12JUN2000	No		
		Week 6 (Fol. Up)	19JUN2000	No		
030.001.00126	IPP	Screening	11APR2000	No		
		Clearance (Day -5)	04MAY2000	Yes	04MAY2000	
		Clearance (Day -4)	05MAY2000	Yes	05MAY2000	
		Clearance (Day -3)	06MAY2000	Yes	06MAY2000	
		Clearance (Unsch.)	07MAY2000	No		
		Load Dose 1	09MAY2000	Yes	09MAY2000	7:05
		Load Dose 2	10MAY2000	Yes	10MAY2000	10:59
		Load Dose 3	11MAY2000	Yes	11MAY2000	8:35
		Week 1 (Unsch.)	12MAY2000	No		
		Week 1	15MAY2000	Yes	15MAY2000	9:45
		Week 2	22MAY2000	Yes	22MAY2000	8:30
		Week 3	29MAY2000	Yes	29MAY2000	10:00
		Week 4	08JUN2000	Yes	08JUN2000	
		Week 5	12JUN2000	Yes	12JUN2000	9:35
		Week 6	19JUN2000	Yes	19JUN2000	16:39
		Week 7	26JUN2000	Yes	26JUN2000	12:22
		Week 8	03JUL2000	Yes	03JUL2000	8:35

*Popn=Population (I=Intent-to-Treat, PP=Per Protocol)
Tafenoquine = Tafenoquine 200mg, Mefloquine = Mefloquine 250mg

SB252263 - 030

Listing B.02

Visit And Dosing Dates

Treatment : Tafenoquine

Subject Number	Popn*	Visit	Visit Date	Dose Taken ?	Date of Dose	Time of Dose
030.001.00126	IPP	Week 9	10JUL2000	Yes	10JUL2000	13:26
		Week 10	17JUL2000	Yes	17JUL2000	13:04
		Week 10 (Unsch.)	18JUL2000	No		
		Week 11	24JUL2000	Yes	24JUL2000	9:49
		Week 12	31JUL2000	Yes	31JUL2000	9:25
		Week 13 (Unsch.)	06AUG2000	No		
		Week 13	07AUG2000	Yes	07AUG2000	6:14
		Week 13 (Unsch.)	08AUG2000	No		
		Week 13 (Unsch.)	10AUG2000	No		
		Week 14	14AUG2000	Yes	14AUG2000	9:24
		Week 15	21AUG2000	Yes	21AUG2000	11:49
		Week 16	28AUG2000	Yes	28AUG2000	9:05
		Week 17	04SEP2000	Yes	04SEP2000	9:14
		Week 17	05SEP2000	No		
		Week 18	11SEP2000	Yes	11SEP2000	9:29
		Week 19	18SEP2000	Yes	18SEP2000	9:39
		Week 20	25SEP2000	Yes	25SEP2000	12:12
		Week 20 (Unsch.)	27SEP2000	No		
		Week 21	02OCT2000	Yes	02OCT2000	9:17
		Week 22	09OCT2000	Yes	09OCT2000	5:57
		Week 23	16OCT2000	Yes	16OCT2000	5:59
		Week 24	23OCT2000	Yes	23OCT2000	6:39
		Week 24 (Fol. Up)	24OCT2000	No		
		Week 24 (Unsch. Fol. Up)	26OCT2000	No		
		Week 25 (Fol. Up)	31OCT2000	No		
		Week 25 (Unsch. Fol. Up)	31OCT2000	No		
		Week 26 (Fol. Up)	08NOV2000	No		
		Week 27 (Fol. Up)	13NOV2000	No		
		Week 28 (Fol. Up)	20NOV2000	No		
030.001.00128	IPP	Screening	10APR2000	No		
		Clearance (Day -5)	04MAY2000	Yes	04MAY2000	
		Clearance (Day -4)	05MAY2000	Yes	05MAY2000	
		Clearance (Day -3)	06MAY2000	Yes	06MAY2000	
		Clearance (Unsch.)	05MAY2000	No		
		Load Dose 1	09MAY2000	Yes	09MAY2000	7:49
		Load Dose 2	10MAY2000	Yes	10MAY2000	7:55
		Load Dose 3	11MAY2000	Yes	11MAY2000	8:45
		Week 1	15MAY2000	Yes	15MAY2000	7:59
		Week 2	22MAY2000	Yes	22MAY2000	7:45
		Week 3 (Unsch.)	27MAY2000	No		
		Week 3	29MAY2000	Yes	29MAY2000	8:21
		Week 4	05JUN2000	Yes	05JUN2000	8:40

*Popn=Population (I=Intent-to-Treat, PP=Per Protocol)
Tafenoquine = Tafenoquine 200mg, Mefloquine = Mefloquine 250mg

SB252263 - 030

Listing B.02

Visit And Dosing Dates

Treatment : Tafenoquine

Subject Number	Popn*	Visit	Visit Date	Dose Taken ?	Date of Dose	Time of Dose
030.001.00128	IPP	Week 5	12JUN2000	Yes	12JUN2000	17:10
		Week 6	19JUN2000	Yes	19JUN2000	8:00
		Week 7	26JUN2000	Yes	26JUN2000	6:43
		Week 8 (Unsch.)	02JUL2000	No		
		Week 8	03JUL2000	Yes	03JUL2000	8:40
		Week 9	10JUL2000	Yes	10JUL2000	8:10
		Week 9 (Unsch.)	11JUL2000	No		
		Week 10	17JUL2000	Yes	17JUL2000	7:45
		Week 11	24JUL2000	Yes	24JUL2000	7:45
		Week 12	31JUL2000	Yes	31JUL2000	12:55
		Week 13	07AUG2000	Yes	07AUG2000	8:20
		Week 14	14AUG2000	Yes	14AUG2000	8:20
		Week 15	21AUG2000	Yes	21AUG2000	6:50
		Week 15 (Unsch.)	21AUG2000	No		
		Week 16	28AUG2000	Yes	28AUG2000	7:00
		Week 16	30AUG2000	No		
		Week 17	05SEP2000	Yes	05SEP2000	8:35
		Week 17 (Unsch.)	05SEP2000	No		
		Week 18	11SEP2000	Yes	11SEP2000	8:05
		Week 19	18SEP2000	Yes	18SEP2000	8:00
		Week 20	25SEP2000	Yes	25SEP2000	11:25
		Week 21	02OCT2000	Yes	02OCT2000	9:40
		Week 22	09OCT2000	Yes	09OCT2000	9:05
		Week 23	16OCT2000	Yes	16OCT2000	7:30
		Week 24	23OCT2000	Yes	23OCT2000	7:20
		Week 25 (Fol. Up)	30OCT2000	No		
		Week 26 (Fol. Up)	06NOV2000	No		
		Week 26 (Unsch. Fol. Up)	08NOV2000	No		
		Week 27 (Fol. Up)	13NOV2000	No		
		Week 28 (Fol. Up)	20NOV2000	No		
030.001.00129	IPP	Screening	12APR2000	No		
		Clearance (Day -5)	04MAY2000	Yes	04MAY2000	
		Clearance (Day -4)	05MAY2000	Yes	05MAY2000	
		Clearance (Day -3)	06MAY2000	Yes	06MAY2000	
		Load Dose 1	09MAY2000	Yes	09MAY2000	7:43
		Load Dose 2	10MAY2000	Yes	10MAY2000	11:05
		Load Dose 3	11MAY2000	Yes	11MAY2000	8:35
		Week 1	15MAY2000	Yes	15MAY2000	10:45
		Week 2 (Unsch.)	22MAY2000	No		
		Week 2	23MAY2000	Yes	23MAY2000	8:51
		Week 3	29MAY2000	Yes	29MAY2000	8:40
		Week 3 (Unsch.)	31MAY2000	No		

*Popn=Population (I=Intent-to-Treat, PP=Per Protocol)
Tafenoquine = Tafenoquine 200mg, Mefloquine = Mefloquine 250mg

SB252263 - 030

Listing B.02

Visit And Dosing Dates

Treatment : Tafenoquine

Subject Number	Popn*	Visit	Visit Date	Dose Taken ?	Date of Dose	Time of Dose
030.001.00129	IPP	Week 4	06JUN2000	Yes	06JUN2000	10:35
		Week 5	12JUN2000	Yes	12JUN2000	10:20
		Week 5 (Unsch.)	13JUN2000	No		
		Week 6	19JUN2000	Yes	19JUN2000	18:43
		Week 7	26JUN2000	Yes	26JUN2000	8:11
		Week 8	03JUL2000	Yes	03JUL2000	10:00
		Week 9	10JUL2000	Yes	10JUL2000	10:46
		Week 10	17JUL2000	Yes	17JUL2000	9:20
		Week 11	24JUL2000	Yes	24JUL2000	7:12
		Week 12	01AUG2000	Yes	01AUG2000	12:20
		Week 13	07AUG2000	Yes	07AUG2000	7:33
		Week 14 (Fol. Up)	14AUG2000	No		
		Week 15 (Fol. Up)	21AUG2000	No		
		Week 15 (Unsch. Fol. Up)	21AUG2000	No		
		Week 16 (Fol. Up)	28AUG2000	No		
030.001.00133	IPP	Screening	10APR2000	No		
		Screening (Unsch.)	28APR2000	No		
		Clearance (Day -5)	04MAY2000	Yes	04MAY2000	
		Clearance (Day -4)	05MAY2000	Yes	05MAY2000	
		Clearance (Day -3)	06MAY2000	Yes	06MAY2000	
		Load Dose 1	09MAY2000	Yes	09MAY2000	7:37
		Load Dose 2	10MAY2000	Yes	10MAY2000	8:27
		Load Dose 3	11MAY2000	Yes	11MAY2000	7:20
		Week 1	15MAY2000	Yes	15MAY2000	9:11
		Week 2	22MAY2000	Yes	22MAY2000	9:40
		Week 3	29MAY2000	Yes	29MAY2000	9:46
		Week 4	05JUN2000	Yes	05JUN2000	13:37
		Week 5	12JUN2000	Yes	12JUN2000	6:52
		Week 6	19JUN2000	Yes	19JUN2000	6:15
		Week 7	26JUN2000	Yes	26JUN2000	15:12
		Week 8	03JUL2000	Yes	03JUL2000	6:30
		Week 9	10JUL2000	Yes	10JUL2000	9:30
		Week 10 (Fol. Up)	17JUL2000	No		
		Week 11 (Fol. Up)	24JUL2000	No		
		Week 12 (Fol. Up)	01AUG2000	No		
		Week 13 (Fol. Up)	07AUG2000	No		
030.001.00135	IPP	Screening	11APR2000	No		
		Clearance (Day -5)	04MAY2000	Yes	04MAY2000	
		Clearance (Day -4)	05MAY2000	Yes	05MAY2000	
		Clearance (Day -3)	06MAY2000	Yes	06MAY2000	

*Popn=Population (I=Intent-to-Treat, PP=Per Protocol)
Tafenoquine = Tafenoquine 200mg, Mefloquine = Mefloquine 250mg

SB252263 - 030

Listing B.02

Visit And Dosing Dates

Treatment : Tafenoquine

Subject Number	Popn*	Visit	Visit Date	Dose Taken ?	Date of Dose	Time of Dose
030.001.00135	IPP	Clearance (Unsch.)	08MAY2000	No		
		Load Dose 1	09MAY2000	Yes	09MAY2000	7:40
		Load Dose 2	10MAY2000	Yes	10MAY2000	7:15
		Load Dose 3	11MAY2000	Yes	11MAY2000	6:55
		Week 1 (Unsch.)	12MAY2000	No		
		Week 1	15MAY2000	Yes	15MAY2000	12:40
		Week 2	22MAY2000	Yes	22MAY2000	7:15
		Week 3	29MAY2000	Yes	29MAY2000	19:07
		Week 3 (Unsch.)	01JUN2000	No		
		Week 4	05JUN2000	Yes	05JUN2000	13:10
		Week 5	12JUN2000	Yes	12JUN2000	10:40
		Week 6	19JUN2000	Yes	19JUN2000	8:10
		Week 7	26JUN2000	Yes	26JUN2000	10:34
		Week 7 (Unsch.)	26JUN2000	No		
		Week 8 (Fol. Up)	03JUL2000	No		
		Week 9 (Fol. Up)	10JUL2000	No		
		Week 10 (Unsch. Fol. Up)	15JUL2000	No		
		Week 10 (Fol. Up)	18JUL2000	No		
030.001.00138	IPP	Screening	12APR2000	No		
		Clearance (Day -5)	04MAY2000	Yes	04MAY2000	
		Clearance (Day -4)	05MAY2000	Yes	05MAY2000	
		Clearance (Day -3)	06MAY2000	Yes	06MAY2000	
		Load Dose 1	09MAY2000	Yes	09MAY2000	8:00
		Load Dose 2	10MAY2000	Yes	10MAY2000	17:22
		Load Dose 3	11MAY2000	Yes	11MAY2000	7:23
		Week 1	15MAY2000	Yes	15MAY2000	11:37
		Week 1 (Unsch.)	18MAY2000	No		
		Week 2	24MAY2000	Yes	24MAY2000	8:50
		Week 3	29MAY2000	Yes	29MAY2000	8:19
		Week 4	05JUN2000	Yes	05JUN2000	8:45
		Week 5	12JUN2000	Yes	12JUN2000	7:49
		Week 6	19JUN2000	Yes	19JUN2000	7:59
		Week 6 (Unsch.)	20JUN2000	No		
		Week 7	26JUN2000	Yes	26JUN2000	7:40
		Week 8 (Fol. Up)	03JUL2000	No		
		Week 8 (Unsch. Fol. Up)	03JUL2000	No		
		Week 9 (Fol. Up)	10JUL2000	No		
		Week 9 (Unsch. Fol. Up)	10JUL2000	No		
		Week 10 (Fol. Up)	17JUL2000	No		
		Week 10 (Unsch. Fol. Up)	17JUL2000	No		

*Popn=Population (I=Intent-to-Treat, PP=Per Protocol)
Tafenoquine = Tafenoquine 200mg, Mefloquine = Mefloquine 250mg

SB252263 - 030

Listing B.02

Visit And Dosing Dates

Treatment : Tafenoquine

Subject Number	Popn*	Visit	Visit Date	Dose Taken ?	Date of Dose	Time of Dose
030.001.00139	IPP	Screening	10APR2000	No		
		Clearance (Day -5)	04MAY2000	Yes	04MAY2000	
		Clearance (Day -4)	05MAY2000	Yes	05MAY2000	
		Clearance (Day -3)	06MAY2000	Yes	06MAY2000	
		Load Dose 1	09MAY2000	Yes	09MAY2000	8:05
		Load Dose 2	10MAY2000	Yes	10MAY2000	10:30
		Load Dose 3	11MAY2000	Yes	11MAY2000	6:15
		Week 1	15MAY2000	Yes	15MAY2000	7:05
		Week 1 (Unsch.)	15MAY2000	No		
		Week 2	22MAY2000	Yes	22MAY2000	10:46
		Week 3	29MAY2000	Yes	29MAY2000	6:50
		Week 4	07JUN2000	Yes	07JUN2000	11:11
		Week 5	12JUN2000	Yes	12JUN2000	10:27
		Week 6	19JUN2000	Yes	19JUN2000	6:05
		Week 7	26JUN2000	Yes	26JUN2000	6:25
		Week 8	04JUL2000	Yes	04JUL2000	8:50
		Week 8	06JUL2000	No		
		Week 9	10JUL2000	Yes	10JUL2000	6:18
		Week 10	17JUL2000	Yes	17JUL2000	6:30
		Week 11	24JUL2000	Yes	24JUL2000	7:20
		Week 12	31JUL2000	Yes	31JUL2000	6:40
		Week 13	07AUG2000	Yes	07AUG2000	6:30
		Week 14	14AUG2000	Yes	14AUG2000	6:40
		Week 15	21AUG2000	Yes	21AUG2000	6:40
		Week 16	28AUG2000	Yes	28AUG2000	6:40
		Week 16	30AUG2000	No		
		Week 17	04SEP2000	Yes	04SEP2000	6:40
		Week 18	11SEP2000	Yes	11SEP2000	6:58
		Week 19	18SEP2000	Yes	18SEP2000	6:05
		Week 19 (Unsch. Fol. Up)	22SEP2000	No		
		Week 20 (Fol. Up)	25SEP2000	No		
		Week 21 (Fol. Up)	04OCT2000	No		
		Week 22 (Unsch. Fol. Up)	08OCT2000	No		
		Week 22 (Fol. Up)	09OCT2000	No		
030.001.00148	IPP	Screening	10APR2000	No		
		Screening (Unsch.)	01MAY2000	No		
		Clearance (Day -5)	04MAY2000	Yes	04MAY2000	
		Clearance (Day -4)	05MAY2000	Yes	05MAY2000	
		Clearance (Day -3)	06MAY2000	Yes	06MAY2000	
		Load Dose 1	09MAY2000	Yes	09MAY2000	8:50
		Load Dose 2	10MAY2000	Yes	10MAY2000	8:10
		Load Dose 3	11MAY2000	Yes	11MAY2000	11:00

*Popn=Population (I=Intent-to-Treat, PP=Per Protocol)
Tafenoquine = Tafenoquine 200mg, Mefloquine = Mefloquine 250mg

SB252263 - 030

Listing B.02

Visit And Dosing Dates

Treatment : Tafenoquine

Subject Number	Popn*	Visit	Visit Date	Dose Taken ?	Date of Dose	Time of Dose
030.001.00148	IPP	Week 1 (Unsch.)	13MAY2000	No		
		Week 1	15MAY2000	Yes	15MAY2000	10:10
		Week 2	22MAY2000	Yes	22MAY2000	8:15
		Week 3 (Unsch.)	27MAY2000	No		
		Week 3	29MAY2000	Yes	29MAY2000	8:38
		Week 3 (Unsch.)	30MAY2000	No		
		Week 4	05JUN2000	Yes	05JUN2000	9:43
		Week 5	12JUN2000	Yes	12JUN2000	10:59
		Week 6 (Unsch.)	17JUN2000	No		
		Week 6	19JUN2000	Yes	19JUN2000	9:04
		Week 6 (Unsch.)	20JUN2000	No		
		Week 7	26JUN2000	Yes	26JUN2000	9:43
		Week 8	03JUL2000	Yes	03JUL2000	9:34
		Week 9	10JUL2000	Yes	10JUL2000	9:10
		Week 10	17JUL2000	Yes	17JUL2000	8:45
		Week 11	24JUL2000	Yes	24JUL2000	9:10
		Week 12	31JUL2000	Yes	31JUL2000	9:35
		Week 13	07AUG2000	Yes	07AUG2000	8:45
		Week 13 (Unsch.)	07AUG2000	No		
		Week 14	14AUG2000	Yes	14AUG2000	8:32
		Week 15	21AUG2000	Yes	21AUG2000	7:45
		Week 16	28AUG2000	Yes	28AUG2000	8:00
		Week 16 (Fol. Up)	30AUG2000	No		
		Week 17 (Fol. Up)	04SEP2000	No		
		Week 18 (Fol. Up)	11SEP2000	No		
		Week 18 (Unsch. Fol. Up)	12SEP2000	No		
		Week 19 (Fol. Up)	18SEP2000	No		
		Week 20 (Unsch. Fol. Up)	25SEP2000	No		
		Week 20 (Fol. Up)	26SEP2000	No		
030.001.00149	IPP	Screening	13APR2000	No		
		Clearance (Day -5)	04MAY2000	Yes	04MAY2000	
		Clearance (Day -4)	05MAY2000	Yes	05MAY2000	
		Clearance (Day -3)	06MAY2000	Yes	06MAY2000	
		Clearance (Unsch.)	08MAY2000	No		
		Load Dose 1	09MAY2000	Yes	09MAY2000	9:11
		Load Dose 2	10MAY2000	Yes	10MAY2000	14:33
		Load Dose 3	11MAY2000	Yes	11MAY2000	10:31
		Week 1	15MAY2000	Yes	15MAY2000	10:45
		Week 2	22MAY2000	Yes	22MAY2000	7:02
		Week 3	29MAY2000	Yes	29MAY2000	6:35
		Week 4	05JUN2000	Yes	05JUN2000	9:28
		Week 5	12JUN2000	Yes	12JUN2000	9:30

*Popn=Population (I=Intent-to-Treat, PP=Per Protocol)
Tafenoquine = Tafenoquine 200mg, Mefloquine = Mefloquine 250mg

SB252263 - 030

Listing B.02

Visit And Dosing Dates

Treatment : Tafenoquine

Subject Number	Popn*	Visit	Visit Date	Dose Taken ?	Date of Dose	Time of Dose
030.001.00149	IPP	Week 6	19JUN2000	Yes	19JUN2000	8:35
		Week 7	26JUN2000	Yes	26JUN2000	8:24
		Week 8	03JUL2000	Yes	03JUL2000	8:20
		Week 8	05JUL2000	No		
		Week 9	10JUL2000	Yes	10JUL2000	8:30
		Week 10	17JUL2000	Yes	17JUL2000	6:35
		Week 11 (Fol. Up)	24JUL2000	No		
		Week 12 (Fol. Up)	31JUL2000	No		
		Week 12 (Unsch. Fol. Up)	31JUL2000	No		
		Week 13 (Fol. Up)	07AUG2000	No		
		Week 13 (Unsch. Fol. Up)	09AUG2000	No		
030.001.00150	IPP	Screening	12APR2000	No		
		Clearance (Day -5)	04MAY2000	Yes	04MAY2000	
		Clearance (Day -4)	05MAY2000	Yes	05MAY2000	
		Clearance (Day -3)	06MAY2000	Yes	06MAY2000	
		Load Dose 1	09MAY2000	Yes	09MAY2000	9:35
		Load Dose 2	10MAY2000	Yes	10MAY2000	7:56
		Load Dose 3	11MAY2000	Yes	11MAY2000	7:01
		Week 1	15MAY2000	Yes	15MAY2000	7:39
		Week 1 (Unsch.)	15MAY2000	No		
		Week 2	22MAY2000	Yes	22MAY2000	12:05
		Week 3	29MAY2000	Yes	29MAY2000	6:59
		Week 4	07JUN2000	Yes	07JUN2000	10:48
		Week 5	12JUN2000	Yes	12JUN2000	7:36
		Week 5 (Unsch.)	13JUN2000	No		
		Week 5 (Unsch.)	14JUN2000	No		
		Week 6	21JUN2000	Yes	21JUN2000	7:36
		Week 7	28JUN2000	Yes	28JUN2000	7:34
		Week 8	03JUL2000	Yes	03JUL2000	7:30
		Week 9 (Fol. Up)	10JUL2000	No		
		Week 10 (Unsch. Fol. Up)	17JUL2000	No		
		Week 10 (Fol. Up)	18JUL2000	No		
		Week 11 (Fol. Up)	25JUL2000	No		
		Week 12 (Fol. Up)	01AUG2000	No		
030.001.00155	IPP	Screening	11APR2000	No		
		Clearance (Day -5)	04MAY2000	Yes	04MAY2000	
		Clearance (Day -4)	05MAY2000	Yes	05MAY2000	
		Clearance (Day -3)	06MAY2000	Yes	06MAY2000	
		Load Dose 1	09MAY2000	Yes	09MAY2000	9:59
		Load Dose 2	10MAY2000	Yes	10MAY2000	8:39

*Popn=Population (I=Intent-to-Treat, PP=Per Protocol)
Tafenoquine = Tafenoquine 200mg, Mefloquine = Mefloquine 250mg

SB252263 - 030

Listing B.02

Visit And Dosing Dates

Treatment : Tafenoquine

Subject Number	Popn*	Visit	Visit Date	Dose Taken ?	Date of Dose	Time of Dose
030.001.00155	IPP	Load Dose 3	11MAY2000	Yes	11MAY2000	8:21
		Week 1 (Unsch.)	12MAY2000	No		
		Week 1	15MAY2000	Yes	15MAY2000	8:36
		Week 2 (Unsch.)	21MAY2000	No		
		Week 2	22MAY2000	Yes	22MAY2000	8:02
		Week 3	29MAY2000	Yes	29MAY2000	7:03
		Week 3 (Unsch.)	01JUN2000	No		
		Week 4	05JUN2000	Yes	05JUN2000	13:35
		Week 5	12JUN2000	Yes	12JUN2000	9:06
		Week 6	19JUN2000	Yes	19JUN2000	10:10
		Week 7	26JUN2000	Yes	26JUN2000	9:00
		Week 8	03JUL2000	Yes	03JUL2000	6:50
		Week 9	14JUL2000	Yes	14JUL2000	6:50
		Week 10	17JUL2000	Yes	17JUL2000	9:32
		Week 11	24JUL2000	Yes	24JUL2000	6:49
		Week 12	31JUL2000	Yes	31JUL2000	13:00
		Week 13 (Fol. Up)	07AUG2000	No		
		Week 13 (Unsch. Fol. Up)	07AUG2000	No		
		Week 14 (Fol. Up)	14AUG2000	No		
		Week 15 (Fol. Up)	21AUG2000	No		
		Week 16 (Fol. Up)	28AUG2000	No		
030.001.00157	IPP	Screening	12APR2000	No		
		Clearance (Day -5)	04MAY2000	Yes	04MAY2000	
		Clearance (Day -4)	05MAY2000	Yes	05MAY2000	
		Clearance (Day -3)	06MAY2000	Yes	06MAY2000	
		Load Dose 1	09MAY2000	Yes	09MAY2000	9:37
		Load Dose 2	10MAY2000	Yes	10MAY2000	9:00
		Load Dose 3	11MAY2000	Yes	11MAY2000	7:43
		Week 1	15MAY2000	Yes	15MAY2000	6:58
		Week 2	22MAY2000	Yes	22MAY2000	6:00
		Week 3	29MAY2000	Yes	29MAY2000	6:02
		Week 4	04JUN2000	Yes	04JUN2000	12:52
		Week 5 (Fol. Up)	12JUN2000	No		
		Week 6 (Fol. Up)	19JUN2000	No		
		Week 7 (Fol. Up)	26JUN2000	No		
030.001.00158	IPP	Screening	11APR2000	No		
		Clearance (Day -5)	04MAY2000	Yes	04MAY2000	
		Clearance (Day -4)	05MAY2000	Yes	05MAY2000	
		Clearance (Day -3)	06MAY2000	Yes	06MAY2000	
		Load Dose 1	09MAY2000	Yes	09MAY2000	10:30

*Popn=Population (I=Intent-to-Treat, PP=Per Protocol)
Tafenoquine = Tafenoquine 200mg, Mefloquine = Mefloquine 250mg

SB252263 - 030

Listing B.02

Visit And Dosing Dates

Treatment : Tafenoquine

Subject Number	Popn*	Visit	Visit Date	Dose Taken ?	Date of Dose	Time of Dose
030.001.00158	IPP	Load Dose 2	10MAY2000	Yes	10MAY2000	7:55
		Load Dose 3	11MAY2000	Yes	11MAY2000	7:20
		Week 1	15MAY2000	Yes	15MAY2000	8:10
		Week 2	22MAY2000	Yes	22MAY2000	6:01
		Week 2 (Unsch.)	22MAY2000	No		
		Week 3	29MAY2000	Yes	29MAY2000	7:40
		Week 4 (Fol. Up)	05JUN2000	No		
		Week 5 (Fol. Up)	12JUN2000	No		
		Week 6 (Fol. Up)	20JUN2000	No		
		Week 7 (Fol. Up)	27JUN2000	No		
030.001.00159	IPP	Screening	12APR2000	No		
		Clearance (Day -5)	04MAY2000	Yes	04MAY2000	
		Clearance (Day -4)	05MAY2000	Yes	05MAY2000	
		Clearance (Day -3)	06MAY2000	Yes	06MAY2000	
		Load Dose 1	09MAY2000	Yes	09MAY2000	10:29
		Load Dose 2	10MAY2000	Yes	10MAY2000	8:16
		Load Dose 3	11MAY2000	Yes	11MAY2000	6:57
		Week 1	15MAY2000	Yes	15MAY2000	10:00
		Week 1 (Unsch.)	15MAY2000	No		
		Week 2	23MAY2000	Yes	23MAY2000	8:30
		Week 3	29MAY2000	Yes	29MAY2000	5:30
		Week 4	05JUN2000	Yes	05JUN2000	11:40
		Week 5	12JUN2000	Yes	12JUN2000	6:18
		Week 6	19JUN2000	Yes	19JUN2000	7:30
		Week 7	28JUN2000	Yes	28JUN2000	7:10
		Week 7 (Unsch.)	28JUN2000	No		
		Week 8	03JUL2000	Yes	03JUL2000	10:27
		Week 9	10JUL2000	Yes	10JUL2000	12:30
		Week 10	17JUL2000	Yes	17JUL2000	16:30
		Week 11	24JUL2000	Yes	24JUL2000	13:00
		Week 12	01AUG2000	Yes	01AUG2000	10:02
		Week 13	09AUG2000	Yes	09AUG2000	16:05
		Week 14	14AUG2000	Yes	14AUG2000	11:00
		Week 15	21AUG2000	Yes	21AUG2000	7:45
		Week 16	28AUG2000	Yes	28AUG2000	9:00
		Week 16	30AUG2000	No		
		Week 17	04SEP2000	Yes	04SEP2000	10:20
		Week 17 (Unsch.)	04SEP2000	No		
		Week 18	12SEP2000	Yes	12SEP2000	7:30
		Week 19	18SEP2000	Yes	18SEP2000	14:05
		Week 20	25SEP2000	Yes	25SEP2000	16:51
		Week 21 (Fol. Up)	02OCT2000	No		

*Popn=Population (I=Intent-to-Treat, PP=Per Protocol)
Tafenoquine = Tafenoquine 200mg, Mefloquine = Mefloquine 250mg

Listing B.02

Visit And Dosing Dates

Treatment : Tafenoquine

Subject Number	Popn*	Visit	Visit Date	Dose Taken ?	Date of Dose	Time of Dose
030.001.00159	IPP	Week 23 (Fol. Up)	17OCT2000	No		
		Week 23 (Unsch. Fol. Up)	17OCT2000	No		
		Week 24 (Fol. Up)	24OCT2000	No		
030.001.00161	IPP	Screening	10APR2000	No		
		Clearance (Day -5)	04MAY2000	Yes	04MAY2000	
		Clearance (Day -4)	05MAY2000	Yes	05MAY2000	
		Clearance (Day -3)	06MAY2000	Yes	06MAY2000	
		Load Dose 1	09MAY2000	Yes	09MAY2000	9:32
		Load Dose 2	10MAY2000	Yes	10MAY2000	14:30
		Load Dose 3	11MAY2000	Yes	11MAY2000	19:00
		Load Dose (Unsch.)	10MAY2000	No		
		Week 1	15MAY2000	Yes	15MAY2000	8:40
		Week 2	22MAY2000	Yes	22MAY2000	6:30
		Week 2 (Unsch.)	24MAY2000	No		
		Week 3	29MAY2000	Yes	29MAY2000	7:00
		Week 3 (Unsch.)	31MAY2000	No		
		Week 4 (Unsch.)	04JUN2000	No		
		Week 4	05JUN2000	Yes	05JUN2000	7:11
		Week 5	12JUN2000	Yes	12JUN2000	7:55
		Week 6	19JUN2000	Yes	19JUN2000	8:15
		Week 6 (Unsch.)	19JUN2000	No		
		Week 7	26JUN2000	Yes	26JUN2000	7:10
		Week 8	03JUL2000	Yes	03JUL2000	6:16
		Week 8 (Fol. Up)	05JUL2000	No		
		Week 9 (Fol. Up)	10JUL2000	No		
		Week 10 (Fol. Up)	18JUL2000	No		
		Week 11 (Fol. Up)	24JUL2000	No		
		Week 12 (Fol. Up)	31JUL2000	No		
030.001.00171	IPP	Screening	12APR2000	No		
		Clearance (Day -5)	04MAY2000	Yes	04MAY2000	
		Clearance (Day -4)	05MAY2000	Yes	05MAY2000	
		Clearance (Day -3)	06MAY2000	Yes	06MAY2000	
		Clearance (Unsch.)	06MAY2000	No		
		Load Dose 1	09MAY2000	Yes	09MAY2000	10:10
		Load Dose 2	10MAY2000	Yes	10MAY2000	10:10
		Load Dose 3	11MAY2000	Yes	11MAY2000	8:45
		Load Dose (Unsch.)	11MAY2000	No		
		Week 1	17MAY2000	Yes	17MAY2000	9:34
		Week 1 (Unsch.)	19MAY2000	No		
		Week 2	22MAY2000	Yes	22MAY2000	10:51

*Popn=Population (I=Intent-to-Treat, PP=Per Protocol)
Tafenoquine = Tafenoquine 200mg, Mefloquine = Mefloquine 250mg

SB252263 - 030

Listing B.02

Visit And Dosing Dates

Treatment : Tafenoquine

Subject Number	Popn*	Visit	Visit Date	Dose Taken ?	Date of Dose	Time of Dose
030.001.00171	IPP	Week 2 (Unsch.)	24MAY2000	No		
		Week 3	29MAY2000	Yes	29MAY2000	9:45
		Week 3 (Unsch.)	31MAY2000	No		
		Week 4	05JUN2000	Yes	05JUN2000	10:15
		Week 5	12JUN2000	Yes	12JUN2000	13:00
		Week 6 (Fol. Up)	23JUN2000	No		
		Week 7 (Fol. Up)	26JUN2000	No		
		Week 7 (Unsch. Fol. Up)	26JUN2000	No		
		Week 8 (Fol. Up)	03JUL2000	No		
		Week 8 (Unsch. Fol. Up)	04JUL2000	No		
		Week 9 (Fol. Up)	10JUL2000	No		
030.001.00173	I	Screening	14APR2000	No		
		Screening (Unsch.)	20APR2000	No		
		Clearance (Day -5)	04MAY2000	Yes	04MAY2000	
		Clearance (Day -4)	05MAY2000	Yes	05MAY2000	
		Clearance (Day -3)	06MAY2000	Yes	06MAY2000	
		Load Dose 1	09MAY2000	Yes	09MAY2000	11:01
		Load Dose 2	10MAY2000	Yes	10MAY2000	8:55
		Load Dose 3	11MAY2000	Yes	11MAY2000	8:55
		Week 1	15MAY2000	Yes	15MAY2000	7:55
		Week 2	22MAY2000	Yes	22MAY2000	7:00
		Week 3	29MAY2000	Yes	29MAY2000	7:35
		Week 4	04JUN2000	Yes	04JUN2000	10:49
		Week 5	12JUN2000	Yes	12JUN2000	7:10
		Week 6	19JUN2000	Yes	19JUN2000	7:19
		Week 7	26JUN2000	Yes	26JUN2000	8:10
		Week 8 (Fol. Up)	03JUL2000	No		
		Week 9 (Fol. Up)	10JUL2000	No		
		Week 10 (Fol. Up)	17JUL2000	No		
030.001.00176	IPP	Screening	13APR2000	No		
		Clearance (Day -5)	04MAY2000	Yes	04MAY2000	
		Clearance (Day -4)	05MAY2000	Yes	05MAY2000	
		Clearance (Day -3)	06MAY2000	Yes	06MAY2000	
		Load Dose 1	09MAY2000	Yes	09MAY2000	11:00
		Load Dose 2	10MAY2000	Yes	10MAY2000	9:05
		Load Dose 3	11MAY2000	Yes	11MAY2000	7:53
		Week 1	17MAY2000	Yes	17MAY2000	7:10
		Week 1 (Unsch.)	17MAY2000	No		
		Week 2	24MAY2000	Yes	24MAY2000	7:45
		Week 3	31MAY2000	Yes	31MAY2000	8:02

*Popn=Population (I=Intent-to-Treat, PP=Per Protocol)
Tafenoquine = Tafenoquine 200mg, Mefloquine = Mefloquine 250mg

SB252263 - 030

Listing B.02

Visit And Dosing Dates

Treatment : Tafenoquine

Subject Number	Popn*	Visit	Visit Date	Dose Taken ?	Date of Dose	Time of Dose
030.001.00176	IPP	Week 4 (Fol. Up)	06JUN2000	No		
		Week 5 (Unsch. Fol. Up)	12JUN2000	No		
		Week 5 (Fol. Up)	14JUN2000	No		
		Week 6 (Fol. Up)	21JUN2000	No		
030.001.00179	I	Screening	12APR2000	No		
		Clearance (Day -5)	04MAY2000	Yes	04MAY2000	
		Clearance (Day -4)	05MAY2000	Yes	05MAY2000	
		Clearance (Day -3)	06MAY2000	Yes	06MAY2000	
		Load Dose 1	09MAY2000	Yes	09MAY2000	10:39
		Load Dose 2	10MAY2000	Yes	10MAY2000	9:50
		Load Dose 3	11MAY2000	Yes	11MAY2000	7:40
		Load Dose (Unsch.)	11MAY2000	No		
		Week 1 (Unsch.)	14MAY2000	No		
		Week 1	17MAY2000	Yes	17MAY2000	12:18
		Week 2 (Unsch.)	22MAY2000	No		
		Week 2	24MAY2000	Yes	24MAY2000	7:03
		Week 2 (Unsch.)	26MAY2000	No		
		Week 3	31MAY2000	Yes	31MAY2000	9:38
		Week 4	06JUN2000	Yes	06JUN2000	12:25
		Week 5	14JUN2000	Yes	14JUN2000	7:30
		Week 6	21JUN2000	Yes	21JUN2000	7:30
		Week 7	28JUN2000	Yes	28JUN2000	8:30
		Week 8	04JUL2000	Yes	04JUL2000	8:45
		Week 9	12JUL2000	Yes	12JUL2000	9:28
		Week 10 (Unsch.)	18JUL2000	No		
		Week 10	19JUL2000	Yes	19JUL2000	9:30
		Week 11	26JUL2000	Yes	26JUL2000	8:30
		Week 12	02AUG2000	Yes	02AUG2000	9:30
		Week 13	09AUG2000	Yes	09AUG2000	7:46
		Week 14 (Unsch.)	12AUG2000	No		
		Week 14	16AUG2000	Yes	16AUG2000	8:58
		Week 15	23AUG2000	Yes	23AUG2000	9:35
		Week 16	28AUG2000	Yes	28AUG2000	9:25
		Week 16	01SEP2000	No		
		Week 17	06SEP2000	Yes	06SEP2000	8:50
030.001.00184	IPP	Screening	17APR2000	No		
		Clearance (Day -5)	04MAY2000	Yes	04MAY2000	
		Clearance (Day -4)	05MAY2000	Yes	05MAY2000	
		Clearance (Day -3)	06MAY2000	Yes	06MAY2000	
		Load Dose 1	09MAY2000	Yes	09MAY2000	11:09

*Popn=Population (I=Intent-to-Treat, PP=Per Protocol)
Tafenoquine = Tafenoquine 200mg, Mefloquine = Mefloquine 250mg

SB252263 - 030

Listing B.02

Visit And Dosing Dates

Treatment : Tafenoquine

Subject Number	Popn*	Visit	Visit Date	Dose Taken ?	Date of Dose	Time of Dose
030.001.00184	IPP	Load Dose 2	10MAY2000	Yes	10MAY2000	8:57
		Load Dose 3	11MAY2000	Yes	11MAY2000	7:41
		Load Dose (Unsch.)	11MAY2000	No		
		Week 1	17MAY2000	Yes	17MAY2000	7:10
		Week 2	24MAY2000	Yes	24MAY2000	8:10
		Week 3	31MAY2000	Yes	31MAY2000	7:51
		Week 4	07JUN2000	Yes	07JUN2000	9:45
		Week 5	14JUN2000	Yes	14JUN2000	9:55
		Week 6	21JUN2000	Yes	21JUN2000	9:13
		Week 6 (Unsch.)	21JUN2000	No		
		Week 7 (Unsch.)	26JUN2000	No		
		Week 7	28JUN2000	Yes	28JUN2000	8:16
		Week 8 (Fol. Up)	04JUL2000	No		
		Week 9 (Fol. Up)	12JUL2000	No		
		Week 10 (Fol. Up)	19JUL2000	No		
030.001.00186	IPP	Screening	10APR2000	No		
		Screening (Unsch.)	28APR2000	No		
		Clearance (Day -5)	04MAY2000	Yes	04MAY2000	
		Clearance (Day -4)	05MAY2000	Yes	05MAY2000	
		Clearance (Day -3)	06MAY2000	Yes	06MAY2000	
		Load Dose 1	09MAY2000	Yes	09MAY2000	11:50
		Load Dose 2	10MAY2000	Yes	10MAY2000	7:37
		Load Dose 3	11MAY2000	Yes	11MAY2000	9:05
		Week 1	17MAY2000	Yes	17MAY2000	12:44
		Week 1 (Unsch.)	19MAY2000	No		
		Week 2	24MAY2000	Yes	24MAY2000	10:09
		Week 3	02JUN2000	Yes	02JUN2000	6:45
		Week 4	07JUN2000	Yes	07JUN2000	7:20
		Week 5	14JUN2000	Yes	14JUN2000	7:07
		Week 5 (Unsch.)	14JUN2000	No		
		Week 6 (Fol. Up)	21JUN2000	No		
		Week 7 (Fol. Up)	28JUN2000	No		
		Week 7 (Unsch. Fol. Up)	29JUN2000	No		
		Week 8 (Fol. Up)	04JUL2000	No		
		Week 9 (Fol. Up)	12JUL2000	No		
030.001.00187	I	Screening	10APR2000	No		
		Clearance (Day -5)	04MAY2000	Yes	04MAY2000	
		Clearance (Day -4)	05MAY2000	Yes	05MAY2000	
		Clearance (Day -3)	06MAY2000	Yes	06MAY2000	
		Load Dose 1	09MAY2000	Yes	09MAY2000	11:14

*Popn=Population (I=Intent-to-Treat, PP=Per Protocol)
Tafenoquine = Tafenoquine 200mg, Mefloquine = Mefloquine 250mg

SB252263 - 030

Listing B.02

Visit And Dosing Dates

Treatment : Tafenoquine

Subject Number	Popn*	Visit	Visit Date	Dose Taken ?	Date of Dose	Time of Dose
030.001.00187	I	Load Dose 2	10MAY2000	Yes	10MAY2000	9:10
		Load Dose 3	11MAY2000	Yes	11MAY2000	8:30
		Week 1	17MAY2000	Yes	17MAY2000	7:07
		Week 1 (Unsch.)	19MAY2000	No		
		Week 2 (Unsch.)	22MAY2000	No		
		Week 2	24MAY2000	Yes	24MAY2000	10:21
		Week 3 (Unsch.)	30MAY2000	No		
		Week 3	02JUN2000	Yes	02JUN2000	9:10
		Week 4	07JUN2000	Yes	07JUN2000	7:10
		Week 6	22JUN2000	Yes	22JUN2000	17:30
		Week 8	01JUL2000	Yes	01JUL2000	9:30
		Week 8	04JUL2000	Yes	04JUL2000	13:13
		Week 9	12JUL2000	Yes	12JUL2000	6:40
		Week 10	19JUL2000	Yes	19JUL2000	7:00
030.001.00190	IPP	Screening	11APR2000	No		
		Clearance (Day -5)	04MAY2000	Yes	04MAY2000	
		Clearance (Day -4)	05MAY2000	Yes	05MAY2000	
		Clearance (Day -3)	06MAY2000	Yes	06MAY2000	
		Load Dose 1	09MAY2000	Yes	09MAY2000	11:34
		Load Dose 2	10MAY2000	Yes	10MAY2000	12:40
		Load Dose 3	11MAY2000	Yes	11MAY2000	7:30
		Week 1	17MAY2000	Yes	17MAY2000	7:29
		Week 2 (Unsch.)	20MAY2000	No		
		Week 2	24MAY2000	Yes	24MAY2000	7:33
		Week 2 (Unsch.)	24MAY2000	No		
		Week 3 (Unsch.)	27MAY2000	No		
		Week 3	31MAY2000	Yes	31MAY2000	7:57
		Week 3 (Unsch.)	31MAY2000	No		
		Week 4 (Unsch.)	03JUN2000	No		
		Week 4 (Unsch.)	04JUN2000	No		
		Week 4	07JUN2000	Yes	07JUN2000	8:37
		Week 4 (Unsch.)	09JUN2000	No		
		Week 5	14JUN2000	Yes	14JUN2000	10:40
		Week 6	21JUN2000	Yes	21JUN2000	13:43
		Week 7	28JUN2000	Yes	28JUN2000	7:46
		Week 8 (Fol. Up)	04JUL2000	No		
		Week 9 (Unsch. Fol. Up)	08JUL2000	No		
		Week 9 (Fol. Up)	12JUL2000	No		
		Week 9 (Unsch. Fol. Up)	12JUL2000	No		
		Week 10 (Unsch. Fol. Up)	18JUL2000	No		
		Week 10 (Fol. Up)	19JUL2000	No		
		Week 11 (Unsch. Fol. Up)	25JUL2000	No		

*Popn=Population (I=Intent-to-Treat, PP=Per Protocol)
Tafenoquine = Tafenoquine 200mg, Mefloquine = Mefloquine 250mg

SB252263 - 030

Listing B.02

Visit And Dosing Dates

Treatment : Tafenoquine

Subject Number	Popn*	Visit	Visit Date	Dose Taken ?	Date of Dose	Time of Dose
030.001.00194	IPP	Screening	14APR2000	No		
		Screening (Unsch.)	19APR2000	No		
		Screening (Unsch.)	27APR2000	No		
		Screening (Unsch.)	29APR2000	No		
		Screening (Unsch.)	02MAY2000	No		
		Clearance (Day -5)	04MAY2000	Yes	04MAY2000	
		Clearance (Day -4)	05MAY2000	Yes	05MAY2000	
		Clearance (Day -3)	06MAY2000	Yes	06MAY2000	
		Load Dose 1	09MAY2000	Yes	09MAY2000	12:23
		Load Dose 2	10MAY2000	Yes	10MAY2000	9:14
		Load Dose 3	11MAY2000	Yes	11MAY2000	8:12
		Week 1	17MAY2000	Yes	17MAY2000	7:10
		Week 1 (Unsch.)	17MAY2000	No		
		Week 1 (Unsch.)	19MAY2000	No		
		Week 2	24MAY2000	Yes	24MAY2000	7:50
		Week 3	31MAY2000	Yes	31MAY2000	7:12
		Week 4 (Fol. Up)	06JUN2000	No		
		Week 5 (Fol. Up)	14JUN2000	No		
		Week 6 (Fol. Up)	21JUN2000	No		
		Week 7 (Unsch. Fol. Up)	24JUN2000	No		
		Week 7 (Fol. Up)	28JUN2000	No		
030.001.00195	IPP	Screening	18APR2000	No		
		Clearance (Day -5)	04MAY2000	Yes	04MAY2000	
		Clearance (Day -4)	05MAY2000	Yes	05MAY2000	
		Clearance (Day -3)	06MAY2000	Yes	06MAY2000	
		Load Dose 1	09MAY2000	Yes	09MAY2000	12:35
		Load Dose 2	10MAY2000	Yes	10MAY2000	8:10
		Load Dose 3	11MAY2000	Yes	11MAY2000	7:59
		Week 1	17MAY2000	Yes	17MAY2000	8:30
		Week 1 (Unsch.)	17MAY2000	No		
		Week 2	24MAY2000	Yes	24MAY2000	6:49
		Week 3	31MAY2000	Yes	31MAY2000	7:10
		Week 4 (Unsch. Fol. Up)	04JUN2000	No		
		Week 4 (Fol. Up)	06JUN2000	No		
		Week 4 (Unsch. Fol. Up)	06JUN2000	No		
		Week 4 (Unsch. Fol. Up)	07JUN2000	No		
		Week 5 (Fol. Up)	14JUN2000	No		
		Week 6 (Unsch. Fol. Up)	20JUN2000	No		
		Week 6 (Fol. Up)	21JUN2000	No		
		Week 7 (Fol. Up)	28JUN2000	No		

*Popn=Population (I=Intent-to-Treat, PP=Per Protocol)
Tafenoquine = Tafenoquine 200mg, Mefloquine = Mefloquine 250mg

SB252263 - 030

Listing B.02

Visit And Dosing Dates

Treatment : Tafenoquine

Subject Number	Popn*	Visit	Visit Date	Dose Taken ?	Date of Dose	Time of Dose
030.001.00201	IPP	Screening	10APR2000	No		
		Clearance (Day -5)	04MAY2000	Yes	04MAY2000	
		Clearance (Day -4)	05MAY2000	Yes	05MAY2000	
		Clearance (Day -3)	06MAY2000	Yes	06MAY2000	
		Load Dose 1	09MAY2000	Yes	09MAY2000	13:25
		Load Dose 2	10MAY2000	Yes	10MAY2000	9:20
		Load Dose 3	11MAY2000	Yes	11MAY2000	8:24
		Week 1	17MAY2000	Yes	17MAY2000	7:29
		Week 2	24MAY2000	Yes	24MAY2000	8:33
		Week 3	31MAY2000	Yes	31MAY2000	7:26
		Week 4	06JUN2000	Yes	06JUN2000	14:50
		Week 5	14JUN2000	Yes	14JUN2000	7:24
		Week 6 (Fol. Up)	21JUN2000	No		
		Week 6 (Unsch. Fol. Up)	23JUN2000	No		
		Week 7 (Fol. Up)	28JUN2000	No		
		Week 8 (Fol. Up)	04JUL2000	No		
		Week 9 (Fol. Up)	10JUL2000	No		
030.001.00203	IPP	Screening	13APR2000	No		
		Clearance (Day -5)	03MAY2000	Yes	03MAY2000	
		Clearance (Day -4)	04MAY2000	Yes	04MAY2000	
		Clearance (Day -3)	05MAY2000	Yes	05MAY2000	
		Load Dose 1	09MAY2000	Yes	09MAY2000	13:30
		Load Dose 2	10MAY2000	Yes	10MAY2000	11:26
		Load Dose 3	11MAY2000	Yes	11MAY2000	7:57
		Week 1	17MAY2000	Yes	17MAY2000	7:38
		Week 2	24MAY2000	Yes	24MAY2000	7:17
		Week 3	31MAY2000	Yes	31MAY2000	7:22
		Week 4 (Unsch.)	05JUN2000	No		
		Week 4	07JUN2000	Yes	07JUN2000	14:10
		Week 5	14JUN2000	Yes	14JUN2000	7:29
		Week 6	21JUN2000	Yes	21JUN2000	7:10
		Week 7 (Fol. Up)	28JUN2000	No		
		Week 8 (Unsch. Fol. Up)	04JUL2000	No		
		Week 8 (Fol. Up)	05JUL2000	No		
		Week 9 (Fol. Up)	12JUL2000	No		
		Week 10 (Fol. Up)	19JUL2000	No		
030.001.00205	IPP	Screening	10APR2000	No		
		Clearance (Day -5)	06MAY2000	Yes	06MAY2000	
		Clearance (Day -4)	07MAY2000	Yes	07MAY2000	
		Clearance (Day -3)	08MAY2000	Yes	08MAY2000	

*Popn=Population (I=Intent-to-Treat, PP=Per Protocol)
Tafenoquine = Tafenoquine 200mg, Mefloquine = Mefloquine 250mg

SB252263 - 030

Listing B.02

Visit And Dosing Dates

Treatment : Tafenoquine

Subject Number	Popn*	Visit	Visit Date	Dose Taken ?	Date of Dose	Time of Dose
030.001.00205	IPP	Load Dose 1	09MAY2000	Yes	09MAY2000	14:04
		Load Dose 2	10MAY2000	Yes	10MAY2000	8:00
		Load Dose 3	11MAY2000	Yes	11MAY2000	7:01
		Week 1	17MAY2000	Yes	17MAY2000	6:00
		Week 1 (Unsch.)	17MAY2000	No		
		Week 2	24MAY2000	Yes	24MAY2000	6:59
		Week 3	31MAY2000	Yes	31MAY2000	6:05
		Week 4	06JUN2000	Yes	06JUN2000	13:59
		Week 5	14JUN2000	Yes	14JUN2000	7:30
		Week 6 (Fol. Up)	21JUN2000	No		
		Week 7 (Fol. Up)	28JUN2000	No		
		Week 8 (Fol. Up)	04JUL2000	No		
		Week 9 (Fol. Up)	11JUL2000	No		
030.001.00207	IPP	Screening	13APR2000	No		
		Clearance (Day -5)	04MAY2000	Yes	04MAY2000	
		Clearance (Day -4)	05MAY2000	Yes	05MAY2000	
		Clearance (Day -3)	06MAY2000	Yes	06MAY2000	
		Load Dose 1	09MAY2000	Yes	09MAY2000	13:38
		Load Dose 2	10MAY2000	Yes	10MAY2000	11:59
		Load Dose 3	11MAY2000	Yes	11MAY2000	7:10
		Week 1 (Unsch.)	12MAY2000	No		
		Week 1	17MAY2000	Yes	17MAY2000	8:42
		Week 2	24MAY2000	Yes	24MAY2000	7:38
		Week 2 (Unsch.)	24MAY2000	No		
		Week 3	31MAY2000	Yes	31MAY2000	8:53
		Week 4 (Unsch.)	04JUN2000	No		
		Week 4	08JUN2000	Yes	08JUN2000	13:15
		Week 5	14JUN2000	Yes	14JUN2000	6:34
		Week 6	21JUN2000	Yes	21JUN2000	7:32
		Week 7	28JUN2000	Yes	28JUN2000	6:52
		Week 8	03JUL2000	Yes	03JUL2000	8:00
		Week 8	07JUL2000	No		
		Week 9	12JUL2000	Yes	12JUL2000	6:49
		Week 9 (Unsch.)	12JUL2000	No		
		Week 10	19JUL2000	Yes	19JUL2000	7:50
		Week 10 (Unsch.)	19JUL2000	No		
		Week 11	26JUL2000	Yes	26JUL2000	8:15
		Week 12	02AUG2000	Yes	02AUG2000	7:24
		Week 13	09AUG2000	Yes	09AUG2000	7:53
		Week 14	17AUG2000	Yes	17AUG2000	11:52
		Week 15	23AUG2000	Yes	23AUG2000	7:32
		Week 15 (Unsch.)	24AUG2000	No		

*Popn=Population (I=Intent-to-Treat, PP=Per Protocol)
Tafenoquine = Tafenoquine 200mg, Mefloquine = Mefloquine 250mg

SB252263 - 030

Listing B.02

Visit And Dosing Dates

Treatment : Tafenoquine

Subject Number	Popn*	Visit	Visit Date	Dose Taken ?	Date of Dose	Time of Dose
030.001.00207	IPP	Week 16	28AUG2000	Yes	28AUG2000	7:36
		Week 17 (Fol. Up)	06SEP2000	No		
		Week 18 (Fol. Up)	14SEP2000	No		
		Week 18 (Unsch. Fol. Up)	14SEP2000	No		
		Week 19 (Fol. Up)	20SEP2000	No		
030.001.00209	IPP	Screening	13APR2000	No		
		Clearance (Day -5)	04MAY2000	Yes	04MAY2000	
		Clearance (Day -4)	05MAY2000	Yes	05MAY2000	
		Clearance (Day -3)	06MAY2000	Yes	06MAY2000	
		Load Dose 1	09MAY2000	Yes	09MAY2000	13:48
		Load Dose 2	10MAY2000	Yes	10MAY2000	9:10
		Load Dose 3	11MAY2000	Yes	11MAY2000	8:45
		Week 1 (Unsch.)	16MAY2000	No		
		Week 1	17MAY2000	Yes	17MAY2000	8:20
		Week 2	24MAY2000	Yes	24MAY2000	12:05
		Week 3	01JUN2000	Yes	01JUN2000	16:10
		Week 4	06JUN2000	Yes	06JUN2000	16:20
		Week 4 (Unsch.)	09JUN2000	No		
		Week 5	14JUN2000	Yes	14JUN2000	8:31
		Week 6	21JUN2000	Yes	21JUN2000	11:08
		Week 7	28JUN2000	Yes	28JUN2000	10:43
		Week 8	05JUL2000	Yes	05JUL2000	18:20
		Week 8 (Fol. Up)	06JUL2000	No		
		Week 9 (Fol. Up)	12JUL2000	No		
		Week 10 (Fol. Up)	20JUL2000	No		
		Week 11 (Fol. Up)	26JUL2000	No		
030.001.00210	IPP	Screening	12APR2000	No		
		Clearance (Day -5)	04MAY2000	Yes	04MAY2000	
		Clearance (Day -4)	05MAY2000	Yes	05MAY2000	
		Clearance (Day -3)	06MAY2000	Yes	06MAY2000	
		Load Dose 1	09MAY2000	Yes	09MAY2000	14:11
		Load Dose 2	10MAY2000	Yes	10MAY2000	14:08
		Load Dose 3	11MAY2000	Yes	11MAY2000	6:49
		Week 1	17MAY2000	Yes	17MAY2000	6:50
		Week 2	24MAY2000	Yes	24MAY2000	6:27
		Week 3	31MAY2000	Yes	31MAY2000	6:10
		Week 4	06JUN2000	Yes	06JUN2000	10:23
		Week 4 (Unsch.)	06JUN2000	No		
		Week 5	14JUN2000	Yes	14JUN2000	6:32
		Week 6	21JUN2000	Yes	21JUN2000	6:23

*Popn=Population (I=Intent-to-Treat, PP=Per Protocol)
Tafenoquine = Tafenoquine 200mg, Mefloquine = Mefloquine 250mg

SB252263 - 030

Listing B.02

Visit And Dosing Dates

Treatment : Tafenoquine

Subject Number	Popn*	Visit	Visit Date	Dose Taken ?	Date of Dose	Time of Dose
030.001.00210	IPP	Week 7	29JUN2000	Yes	29JUN2000	16:35
		Week 8	04JUL2000	Yes	04JUL2000	6:14
		Week 9	11JUL2000	No		
		Week 9	12JUL2000	Yes	12JUL2000	6:35
		Week 10	19JUL2000	Yes	19JUL2000	6:20
		Week 11	26JUL2000	Yes	26JUL2000	6:35
		Week 12	02AUG2000	Yes	02AUG2000	7:10
		Week 13 (Fol. Up)	09AUG2000	No		
		Week 14 (Fol. Up)	16AUG2000	No		
		Week 15 (Fol. Up)	23AUG2000	No		
030.001.00217	IPP	Screening	11APR2000	No		
		Clearance (Day -5)	04MAY2000	Yes	04MAY2000	
		Clearance (Day -4)	05MAY2000	Yes	05MAY2000	
		Clearance (Day -3)	06MAY2000	Yes	06MAY2000	
		Load Dose 1	09MAY2000	Yes	09MAY2000	15:00
		Load Dose 2	10MAY2000	Yes	10MAY2000	12:16
		Load Dose 3	11MAY2000	Yes	11MAY2000	8:23
		Week 1	17MAY2000	Yes	17MAY2000	6:58
		Week 2	24MAY2000	Yes	24MAY2000	7:14
		Week 3	31MAY2000	Yes	31MAY2000	7:36
		Week 4	07JUN2000	Yes	07JUN2000	17:39
		Week 5	14JUN2000	Yes	14JUN2000	7:40
		Week 6	21JUN2000	Yes	21JUN2000	15:40
		Week 7 (Fol. Up)	29JUN2000	No		
		Week 8 (Fol. Up)	04JUL2000	No		
		Week 9 (Fol. Up)	12JUL2000	No		
		Week 10 (Fol. Up)	19JUL2000	No		
030.001.00218	IPP	Screening	13APR2000	No		
		Clearance (Day -5)	04MAY2000	Yes	04MAY2000	
		Clearance (Day -4)	05MAY2000	Yes	05MAY2000	
		Clearance (Day -3)	06MAY2000	Yes	06MAY2000	
		Load Dose 1	09MAY2000	Yes	09MAY2000	15:23
		Load Dose 2	10MAY2000	Yes	10MAY2000	13:02
		Load Dose 3	11MAY2000	Yes	11MAY2000	9:34
		Week 1	17MAY2000	Yes	17MAY2000	12:25
		Week 2 (Unsch.)	23MAY2000	No		
		Week 2	24MAY2000	Yes	24MAY2000	7:49
		Week 3	31MAY2000	Yes	31MAY2000	9:35
		Week 4	06JUN2000	Yes	06JUN2000	10:24
		Week 5	14JUN2000	Yes	14JUN2000	12:44

*Popn=Population (I=Intent-to-Treat, PP=Per Protocol)
Tafenoquine = Tafenoquine 200mg, Mefloquine = Mefloquine 250mg

SB252263 - 030

Listing B.02

Visit And Dosing Dates

Treatment : Tafenoquine

Subject Number	Popn*	Visit	Visit Date	Dose Taken ?	Date of Dose	Time of Dose
030.001.00218	IPP	Week 6	21JUN2000	Yes	21JUN2000	11:05
		Week 7 (Unsch.)	24JUN2000	No		
		Week 7 (Unsch.)	27JUN2000	No		
		Week 7	28JUN2000	Yes	28JUN2000	14:02
		Week 8	04JUL2000	Yes	04JUL2000	8:30
		Week 9	12JUL2000	Yes	12JUL2000	11:15
		Week 10	19JUL2000	Yes	19JUL2000	7:36
		Week 11	26JUL2000	Yes	26JUL2000	8:05
		Week 12	02AUG2000	Yes	02AUG2000	8:30
		Week 13	09AUG2000	Yes	09AUG2000	7:35
		Week 14	17AUG2000	Yes	17AUG2000	8:30
		Week 15	23AUG2000	Yes	23AUG2000	8:09
		Week 16	28AUG2000	Yes	28AUG2000	15:07
		Week 18	09SEP2000	Yes	09SEP2000	10:40
		Week 18 (Unsch.)	11SEP2000	No		
		Week 18	13SEP2000	Yes	13SEP2000	7:40
		Week 19	20SEP2000	Yes	20SEP2000	7:42
		Week 19 (Unsch.)	22SEP2000	No		
		Week 20	27SEP2000	Yes	27SEP2000	7:30
		Week 21	04OCT2000	Yes	04OCT2000	14:54
		Week 22	13OCT2000	Yes	13OCT2000	16:20
		Week 23	17OCT2000	Yes	17OCT2000	10:06
		Week 23	19OCT2000	Yes	19OCT2000	7:40
		Week 24	24OCT2000	Yes	24OCT2000	13:57
030.001.00220	IPP	Screening	13APR2000	No		
		Clearance (Day -5)	04MAY2000	Yes	04MAY2000	
		Clearance (Day -4)	05MAY2000	Yes	05MAY2000	
		Clearance (Day -3)	06MAY2000	Yes	06MAY2000	
		Load Dose 1	09MAY2000	Yes	09MAY2000	15:15
		Load Dose 2	10MAY2000	Yes	10MAY2000	8:40
		Load Dose 3	11MAY2000	Yes	11MAY2000	8:05
		Week 1	17MAY2000	Yes	17MAY2000	7:05
		Week 2	24MAY2000	Yes	24MAY2000	7:40
		Week 3	31MAY2000	Yes	31MAY2000	6:55
		Week 4	07JUN2000	Yes	07JUN2000	10:30
		Week 5	14JUN2000	Yes	14JUN2000	7:30
		Week 6	21JUN2000	Yes	21JUN2000	7:17
		Week 7	28JUN2000	Yes	28JUN2000	7:01
		Week 8 (Fol. Up)	04JUL2000	No		
		Week 9 (Fol. Up)	12JUL2000	No		
		Week 10 (Fol. Up)	19JUL2000	No		
		Week 11 (Fol. Up)	26JUL2000	No		

*Popn=Population (I=Intent-to-Treat, PP=Per Protocol)
Tafenoquine = Tafenoquine 200mg, Mefloquine = Mefloquine 250mg

SB252263 - 030

Listing B.02

Visit And Dosing Dates

Treatment : Tafenoquine

Subject Number	Popn*	Visit	Visit Date	Dose Taken ?	Date of Dose	Time of Dose
030.001.00228	IPP	Screening	13APR2000	No		
		Clearance (Day -5)	04MAY2000	Yes	04MAY2000	
		Clearance (Day -4)	05MAY2000	Yes	05MAY2000	
		Clearance (Day -3)	06MAY2000	Yes	06MAY2000	
		Load Dose 1	09MAY2000	Yes	09MAY2000	16:37
		Load Dose 2	10MAY2000	Yes	10MAY2000	11:40
		Load Dose 3	11MAY2000	Yes	11MAY2000	6:48
		Week 1 (Unsch.)	12MAY2000	No		
		Week 1	17MAY2000	Yes	17MAY2000	9:08
		Week 2	24MAY2000	Yes	24MAY2000	7:26
		Week 2 (Unsch.)	24MAY2000	No		
		Week 3	31MAY2000	Yes	31MAY2000	7:34
		Week 4	07JUN2000	Yes	07JUN2000	10:20
		Week 4 (Unsch.)	09JUN2000	No		
		Week 5	14JUN2000	Yes	14JUN2000	7:27
		Week 6 (Fol. Up)	21JUN2000	No		
		Week 7 (Fol. Up)	28JUN2000	No		
		Week 8 (Fol. Up)	03JUL2000	No		
		Week 9 (Fol. Up)	12JUL2000	No		
030.001.00230	IPP	Screening	13APR2000	No		
		Clearance (Day -5)	04MAY2000	Yes	04MAY2000	
		Clearance (Day -4)	05MAY2000	Yes	05MAY2000	
		Clearance (Day -3)	06MAY2000	Yes	06MAY2000	
		Load Dose 1	09MAY2000	Yes	09MAY2000	17:15
		Load Dose 2	10MAY2000	Yes	10MAY2000	18:57
		Load Dose 3	11MAY2000	Yes	11MAY2000	6:26
		Week 1	18MAY2000	Yes	18MAY2000	8:06
		Week 2	24MAY2000	Yes	24MAY2000	6:44
		Week 3	31MAY2000	Yes	31MAY2000	6:30
		Week 4	07JUN2000	Yes	07JUN2000	18:16
		Week 5 (Unsch.)	10JUN2000	No		
		Week 5	14JUN2000	Yes	14JUN2000	6:23
		Week 6	21JUN2000	Yes	21JUN2000	6:15
		Week 7	28JUN2000	Yes	28JUN2000	6:00
		Week 8	04JUL2000	Yes	04JUL2000	7:06
		Week 8 (Fol. Up)	05JUL2000	No		
		Week 9 (Fol. Up)	12JUL2000	No		
		Week 9 (Unsch. Fol. Up)	12JUL2000	No		
		Week 10 (Fol. Up)	19JUL2000	No		
		Week 11 (Fol. Up)	26JUL2000	No		
		Week 12 (Unsch. Fol. Up)	29JUL2000	No		

*Popn=Population (I=Intent-to-Treat, PP=Per Protocol)
Tafenoquine = Tafenoquine 200mg, Mefloquine = Mefloquine 250mg

SB252263 - 030

Listing B.02

Visit And Dosing Dates

Treatment : Tafenoquine

Subject Number	Popn*	Visit	Visit Date	Dose Taken ?	Date of Dose	Time of Dose
030.001.00231	IPP	Screening	12APR2000	No		
		Clearance (Day -5)	04MAY2000	Yes	04MAY2000	
		Clearance (Day -4)	05MAY2000	Yes	05MAY2000	
		Clearance (Day -3)	06MAY2000	Yes	06MAY2000	
		Clearance (Unsch.)	05MAY2000	No		
		Load Dose 1	09MAY2000	Yes	09MAY2000	18:50
		Load Dose 2	10MAY2000	Yes	10MAY2000	15:21
		Load Dose 3	11MAY2000	Yes	11MAY2000	11:41
		Week 1 (Unsch.)	15MAY2000	No		
		Week 1 (Unsch.)	16MAY2000	No		
		Week 1	17MAY2000	Yes	17MAY2000	9:15
		Week 2	24MAY2000	Yes	24MAY2000	9:25
		Week 3	31MAY2000	Yes	31MAY2000	8:00
		Week 4	06JUN2000	Yes	06JUN2000	7:40
		Week 5	14JUN2000	Yes	14JUN2000	8:02
		Week 6	21JUN2000	Yes	21JUN2000	8:35
		Week 7	28JUN2000	Yes	28JUN2000	6:50
		Week 8	04JUL2000	Yes	04JUL2000	7:35
		Week 9	12JUL2000	Yes	12JUL2000	6:55
		Week 10	19JUL2000	Yes	19JUL2000	8:11
		Week 11 (Unsch.)	25JUL2000	No		
		Week 11	26JUL2000	Yes	26JUL2000	6:02
		Week 12	31JUL2000	Yes	31JUL2000	13:10
		Week 13	09AUG2000	Yes	09AUG2000	8:42
		Week 14	17AUG2000	Yes	17AUG2000	14:16
		Week 15 (Unsch.)	19AUG2000	No		
		Week 15	23AUG2000	Yes	23AUG2000	11:30
		Week 16	29AUG2000	Yes	29AUG2000	12:30
		Week 16	31AUG2000	No		
		Week 16 (Unsch.)	31AUG2000	No		
		Week 17	06SEP2000	Yes	06SEP2000	9:45
		Week 18	13SEP2000	Yes	13SEP2000	8:13
		Week 19 (Fol. Up)	18SEP2000	No		
		Week 20 (Unsch. Fol. Up)	25SEP2000	No		
		Week 21 (Fol. Up)	04OCT2000	No		
		Week 21 (Fol. Up)	05OCT2000	No		
030.001.00233	IPP	Screening	10APR2000	No		
		Clearance (Day -5)	05MAY2000	Yes	05MAY2000	
		Clearance (Day -4)	06MAY2000	Yes	06MAY2000	
		Clearance (Day -3)	07MAY2000	Yes	07MAY2000	
		Clearance (Unsch.)	09MAY2000	No		
		Load Dose 1	10MAY2000	Yes	10MAY2000	8:04

*Popn=Population (I=Intent-to-Treat, PP=Per Protocol)
Tafenoquine = Tafenoquine 200mg, Mefloquine = Mefloquine 250mg

SB252263 - 030

Listing B.02

Visit And Dosing Dates

Treatment : Tafenoquine

Subject Number	Popn*	Visit	Visit Date	Dose Taken ?	Date of Dose	Time of Dose
030.001.00233	IPP	Load Dose 2	11MAY2000	Yes	11MAY2000	8:25
		Load Dose 3	12MAY2000	Yes	12MAY2000	7:22
		Load Dose (Unsch.)	11MAY2000	No		
		Week 1 (Unsch.)	13MAY2000	No		
		Week 1 (Unsch.)	14MAY2000	No		
		Week 1	17MAY2000	Yes	17MAY2000	6:50
		Week 1 (Unsch.)	18MAY2000	No		
		Week 2	24MAY2000	Yes	24MAY2000	8:11
		Week 3	31MAY2000	Yes	31MAY2000	7:05
		Week 4	07JUN2000	Yes	07JUN2000	14:15
		Week 5	14JUN2000	Yes	14JUN2000	7:15
		Week 5 (Unsch.)	15JUN2000	No		
		Week 6	21JUN2000	Yes	21JUN2000	15:58
		Week 6 (Unsch.)	22JUN2000	No		
		Week 6 (Unsch.)	23JUN2000	No		
		Week 6 (Unsch.)	24JUN2000	No		
		Week 7 (Unsch.)	25JUN2000	No		
		Week 7 (Unsch.)	26JUN2000	No		
		Week 7 (Unsch.)	27JUN2000	No		
		Week 7 (Unsch.)	28JUN2000	No		
		Week 7 (Unsch.)	29JUN2000	No		
		Week 7	30JUN2000	Yes	30JUN2000	16:20
		Week 7 (Unsch.)	30JUN2000	No		
		Week 8	06JUL2000	Yes	06JUL2000	10:55
		Week 9 (Unsch.)	11JUL2000	No		
		Week 9	12JUL2000	Yes	12JUL2000	8:10
		Week 9 (Unsch.)	14JUL2000	No		
		Week 10	19JUL2000	Yes	19JUL2000	8:10
		Week 10 (Unsch.)	19JUL2000	No		
		Week 10 (Unsch.)	21JUL2000	No		
		Week 11	26JUL2000	Yes	26JUL2000	6:40
		Week 11 (Unsch.)	26JUL2000	No		
		Week 12	01AUG2000	Yes	01AUG2000	10:45
		Week 12 (Unsch.)	01AUG2000	No		
		Week 13	11AUG2000	Yes	11AUG2000	7:48
		Week 14 (Unsch.)	14AUG2000	No		
		Week 14	16AUG2000	Yes	16AUG2000	7:10
		Week 15	23AUG2000	Yes	23AUG2000	6:50
		Week 16	28AUG2000	Yes	28AUG2000	6:40
		Week 16	30AUG2000	No		
		Week 17	04SEP2000	Yes	04SEP2000	9:15
		Week 18 (Fol. Up)	13SEP2000	No		
		Week 19 (Fol. Up)	20SEP2000	No		
		Week 20 (Fol. Up)	25SEP2000	No		

*Popn=Population (I=Intent-to-Treat, PP=Per Protocol)
Tafenoquine = Tafenoquine 200mg, Mefloquine = Mefloquine 250mg

SB252263 - 030

Listing B.02

Visit And Dosing Dates

Treatment : Tafenoquine

Subject Number	Popn*	Visit	Visit Date	Dose Taken ?	Date of Dose	Time of Dose
030.001.00233	IPP	Week 21 (Fol. Up)	03OCT2000	No		
030.001.00240	IPP	Screening	13APR2000	No		
		Clearance (Day -5)	05MAY2000	Yes	05MAY2000	
		Clearance (Day -4)	06MAY2000	Yes	06MAY2000	
		Clearance (Day -3)	07MAY2000	Yes	07MAY2000	
		Load Dose 1	10MAY2000	Yes	10MAY2000	7:40
		Load Dose 2	11MAY2000	Yes	11MAY2000	13:06
		Load Dose 3	12MAY2000	Yes	12MAY2000	7:20
		Week 1	17MAY2000	Yes	17MAY2000	13:20
		Week 2	24MAY2000	Yes	24MAY2000	7:20
		Week 3	01JUN2000	Yes	01JUN2000	12:47
		Week 3 (Unsch.)	03JUN2000	No		
		Week 4	06JUN2000	Yes	06JUN2000	9:55
		Week 5	14JUN2000	Yes	14JUN2000	6:42
		Week 6 (Fol. Up)	21JUN2000	No		
		Week 7 (Fol. Up)	28JUN2000	No		
		Week 7 (Unsch. Fol. Up)	28JUN2000	No		
		Week 9 (Fol. Up)	11JUL2000	No		
030.001.00243	IPP	Screening	13APR2000	No		
		Clearance (Day -5)	05MAY2000	Yes	05MAY2000	
		Clearance (Day -4)	06MAY2000	Yes	06MAY2000	
		Clearance (Day -3)	07MAY2000	Yes	07MAY2000	
		Load Dose 1	10MAY2000	Yes	10MAY2000	8:25
		Load Dose 2	11MAY2000	Yes	11MAY2000	7:07
		Load Dose 3	12MAY2000	Yes	12MAY2000	7:50
		Week 1 (Unsch.)	15MAY2000	No		
		Week 1	17MAY2000	Yes	17MAY2000	6:55
		Week 2	24MAY2000	Yes	24MAY2000	7:05
		Week 3	31MAY2000	Yes	31MAY2000	7:15
		Week 4	06JUN2000	Yes	06JUN2000	13:19
		Week 5	14JUN2000	Yes	14JUN2000	7:10
		Week 6	21JUN2000	Yes	21JUN2000	7:00
		Week 7 (Unsch.)	26JUN2000	No		
		Week 7	28JUN2000	Yes	28JUN2000	7:25
		Week 8	04JUL2000	Yes	04JUL2000	7:10
		Week 9	12JUL2000	Yes	12JUL2000	7:25
		Week 10	19JUL2000	Yes	19JUL2000	6:40
		Week 11	26JUL2000	Yes	26JUL2000	7:30
		Week 12	02AUG2000	Yes	02AUG2000	8:30
		Week 13 (Unsch.)	07AUG2000	No		

*Popn=Population (I=Intent-to-Treat, PP=Per Protocol)
Tafenoquine = Tafenoquine 200mg, Mefloquine = Mefloquine 250mg

SB252263 - 030

Listing B.02

Visit And Dosing Dates

Treatment : Tafenoquine

Subject Number	Popn*	Visit	Visit Date	Dose Taken ?	Date of Dose	Time of Dose
030.001.00243	IPP	Week 13	09AUG2000	Yes	09AUG2000	8:20
		Week 14	16AUG2000	Yes	16AUG2000	7:40
		Week 16	27AUG2000	Yes	27AUG2000	15:34
		Week 16	29AUG2000	No		
		Week 16	30AUG2000	Yes	30AUG2000	12:30
		Week 17	06SEP2000	Yes	06SEP2000	7:15
		Week 18	13SEP2000	Yes	13SEP2000	6:50
		Week 19	20SEP2000	Yes	20SEP2000	6:58
		Week 20 (Unsch.)	25SEP2000	No		
		Week 20	27SEP2000	Yes	27SEP2000	6:55
		Week 21	04OCT2000	Yes	04OCT2000	7:10
		Week 22	11OCT2000	Yes	11OCT2000	8:15
		Week 23	18OCT2000	Yes	18OCT2000	9:00
		Week 24	24OCT2000	Yes	24OCT2000	9:15
		Week 25 (Fol. Up)	01NOV2000	No		
		Week 26 (Fol. Up)	08NOV2000	No		
		Week 27 (Fol. Up)	17NOV2000	No		
		Week 28 (Fol. Up)	20NOV2000	No		
		Week 28 (Fol. Up)	22NOV2000	No		
030.001.00246	IPP	Screening	11APR2000	No		
		Clearance (Day -5)	05MAY2000	Yes	05MAY2000	
		Clearance (Day -4)	06MAY2000	Yes	06MAY2000	
		Clearance (Day -3)	07MAY2000	Yes	07MAY2000	
		Load Dose 1	10MAY2000	Yes	10MAY2000	9:05
		Load Dose 2	11MAY2000	Yes	11MAY2000	7:01
		Load Dose 3	12MAY2000	Yes	12MAY2000	7:30
		Week 1	17MAY2000	Yes	17MAY2000	7:30
		Week 2	24MAY2000	Yes	24MAY2000	8:10
		Week 3	31MAY2000	Yes	31MAY2000	6:15
		Week 4	06JUN2000	No		
		Week 5	14JUN2000	Yes	14JUN2000	17:35
		Week 6	21JUN2000	Yes	21JUN2000	6:25
		Week 7	28JUN2000	Yes	28JUN2000	7:58
		Week 8	04JUL2000	Yes	04JUL2000	6:00
		Week 9	12JUL2000	Yes	12JUL2000	8:00
		Week 10 (Fol. Up)	19JUL2000	No		
		Week 10 (Unsch. Fol. Up)	21JUL2000	No		
		Week 11 (Fol. Up)	27JUL2000	No		
		Week 12 (Unsch. Fol. Up)	01AUG2000	No		
		Week 12 (Fol. Up)	02AUG2000	No		
		Week 13 (Fol. Up)	11AUG2000	No		

*Popn=Population (I=Intent-to-Treat, PP=Per Protocol)
Tafenoquine = Tafenoquine 200mg, Mefloquine = Mefloquine 250mg

Listing B.02

Visit And Dosing Dates

Treatment : Tafenoquine

Subject Number	Popn*	Visit	Visit Date	Dose Taken ?	Date of Dose	Time of Dose
030.001.00249	IPP	Screening	12APR2000	No		
		Clearance (Day -5)	05MAY2000	Yes	05MAY2000	
		Clearance (Day -4)	06MAY2000	Yes	06MAY2000	
		Clearance (Day -3)	07MAY2000	Yes	07MAY2000	
		Load Dose 1	10MAY2000	Yes	10MAY2000	8:50
		Load Dose 2	11MAY2000	Yes	11MAY2000	7:32
		Load Dose 3	12MAY2000	Yes	12MAY2000	8:00
		Week 1 (Unsch.)	15MAY2000	No		
		Week 1	17MAY2000	Yes	17MAY2000	7:05
		Week 2	24MAY2000	Yes	24MAY2000	7:57
		Week 3	31MAY2000	Yes	31MAY2000	7:20
		Week 4	06JUN2000	Yes	06JUN2000	7:51
		Week 5	14JUN2000	Yes	14JUN2000	7:50
		Week 6	21JUN2000	Yes	21JUN2000	7:53
		Week 7	28JUN2000	Yes	28JUN2000	7:40
		Week 8	04JUL2000	Yes	04JUL2000	6:45
		Week 8 (Unsch. Fol. Up)	05JUL2000	No		
		Week 9 (Fol. Up)	12JUL2000	No		
		Week 10 (Fol. Up)	19JUL2000	No		
		Week 11 (Fol. Up)	26JUL2000	No		
030.001.00252	IPP	Screening	12APR2000	No		
		Clearance (Day -5)	05MAY2000	Yes	05MAY2000	
		Clearance (Day -4)	06MAY2000	Yes	06MAY2000	
		Clearance (Day -3)	07MAY2000	Yes	07MAY2000	
		Load Dose 1	10MAY2000	Yes	10MAY2000	9:00
		Load Dose 2	11MAY2000	Yes	11MAY2000	9:17
		Load Dose 3	12MAY2000	Yes	12MAY2000	6:59
		Week 1	17MAY2000	Yes	17MAY2000	7:48
		Week 2	24MAY2000	Yes	24MAY2000	7:58
		Week 3	31MAY2000	Yes	31MAY2000	7:10
		Week 4	06JUN2000	Yes	06JUN2000	15:20
		Week 5	15JUN2000	Yes	15JUN2000	12:13
		Week 6	21JUN2000	Yes	21JUN2000	6:55
		Week 7	28JUN2000	Yes	28JUN2000	6:59
		Week 8	03JUL2000	Yes	03JUL2000	10:25
		Week 9	12JUL2000	Yes	12JUL2000	7:58
		Week 10	19JUL2000	Yes	19JUL2000	11:06
		Week 11	26JUL2000	Yes	26JUL2000	7:52
		Week 12	02AUG2000	Yes	02AUG2000	7:45
		Week 13	09AUG2000	Yes	09AUG2000	7:02
		Week 14	16AUG2000	Yes	16AUG2000	7:12
		Week 15	23AUG2000	Yes	23AUG2000	7:21

*Popn=Population (I=Intent-to-Treat, PP=Per Protocol)
Tafenoquine = Tafenoquine 200mg, Mefloquine = Mefloquine 250mg

SB252263 - 030

Listing B.02

Visit And Dosing Dates

Treatment : Tafenoquine

Subject Number	Popn*	Visit	Visit Date	Dose Taken ?	Date of Dose	Time of Dose
030.001.00252	IPP	Week 16	29AUG2000	Yes	29AUG2000	11:26
		Week 16	31AUG2000	No		
		Week 17	04SEP2000	Yes	04SEP2000	9:00
		Week 19	17SEP2000	Yes	17SEP2000	13:00
		Week 19	22SEP2000	Yes	22SEP2000	11:25
		Week 20	26SEP2000	Yes	26SEP2000	11:35
		Week 21	04OCT2000	Yes	04OCT2000	10:45
		Week 23 (Fol. Up)	19OCT2000	No		
		Week 25 (Fol. Up)	01NOV2000	No		
030.001.00254	I	Screening	12APR2000	No		
		Clearance (Day -5)	05MAY2000	Yes	05MAY2000	
		Clearance (Day -4)	06MAY2000	Yes	06MAY2000	
		Clearance (Day -3)	07MAY2000	Yes	07MAY2000	
		Clearance (Unsch.)	06MAY2000	No		
		Load Dose 1	10MAY2000	Yes	10MAY2000	9:37
		Load Dose 2	11MAY2000	Yes	11MAY2000	8:30
		Load Dose 3	12MAY2000	Yes	12MAY2000	9:40
		Week 1	17MAY2000	Yes	17MAY2000	12:10
		Week 2	24MAY2000	Yes	24MAY2000	8:14
		Week 2 (Unsch.)	27MAY2000	No		
		Week 3 (Unsch.)	29MAY2000	No		
		Week 3	31MAY2000	Yes	31MAY2000	8:00
		Week 4	08JUN2000	Yes	08JUN2000	6:38
		Week 5 (Fol. Up)	14JUN2000	No		
		Week 6 (Fol. Up)	19JUN2000	No		
		Week 7 (Fol. Up)	28JUN2000	No		
030.001.00259	IPP	Screening	12APR2000	No		
		Clearance (Day -5)	05MAY2000	Yes	05MAY2000	
		Clearance (Day -4)	06MAY2000	Yes	06MAY2000	
		Clearance (Day -3)	07MAY2000	Yes	07MAY2000	
		Load Dose 1	10MAY2000	Yes	10MAY2000	9:40
		Load Dose 2	11MAY2000	Yes	11MAY2000	6:55
		Load Dose 3	12MAY2000	Yes	12MAY2000	7:48
		Week 1	17MAY2000	Yes	17MAY2000	7:16
		Week 2	24MAY2000	Yes	24MAY2000	12:55
		Week 3	31MAY2000	Yes	31MAY2000	8:04
		Week 4	06JUN2000	Yes	06JUN2000	10:30
		Week 5	14JUN2000	Yes	14JUN2000	8:43
		Week 6	21JUN2000	Yes	21JUN2000	8:56
		Week 7	29JUN2000	Yes	29JUN2000	7:20

*Popn=Population (I=Intent-to-Treat, PP=Per Protocol)
Tafenoquine = Tafenoquine 200mg, Mefloquine = Mefloquine 250mg

SB252263 - 030

Listing B.02

Visit And Dosing Dates

Treatment : Tafenoquine

Subject Number	Popn*	Visit	Visit Date	Dose Taken ?	Date of Dose	Time of Dose
030.001.00259	IPP	Week 8	03JUL2000	Yes	03JUL2000	9:30
		Week 8 (Fol. Up)	04JUL2000	No		
		Week 9 (Unsch. Fol. Up)	09JUL2000	No		
		Week 10 (Fol. Up)	16JUL2000	No		
		Week 10 (Fol. Up)	20JUL2000	No		
		Week 11 (Fol. Up)	25JUL2000	No		
		Week 12 (Fol. Up)	02AUG2000	No		
030.001.00261		Screening	13APR2000	No		
		Clearance (Day -5)	05MAY2000	Yes	05MAY2000	
		Clearance (Day -4)	06MAY2000	Yes	06MAY2000	
		Clearance (Day -3)	07MAY2000	Yes	07MAY2000	
		Load Dose 1	10MAY2000	Yes	10MAY2000	16:21
		Load Dose 2	11MAY2000	Yes	11MAY2000	7:32
		Load Dose 3	12MAY2000	Yes	12MAY2000	8:20
		Week 1 (Unsch. Fol. Up)	13MAY2000	No		
		Week 1 (Unsch. Fol. Up)	15MAY2000	No		
		Week 1 (Unsch. Fol. Up)	16MAY2000	No		
		Week 1 (Fol. Up)	18MAY2000	No		
		Week 2 (Unsch. Fol. Up)	23MAY2000	No		
		Week 2 (Fol. Up)	24MAY2000	No		
		Week 3 (Fol. Up)	31MAY2000	No		
		Week 4 (Fol. Up)	06JUN2000	No		
030.001.00264	IPP	Screening	18APR2000	No		
		Clearance (Day -5)	05MAY2000	Yes	05MAY2000	
		Clearance (Day -4)	06MAY2000	Yes	06MAY2000	
		Clearance (Day -3)	07MAY2000	Yes	07MAY2000	
		Load Dose 1	10MAY2000	Yes	10MAY2000	13:07
		Load Dose 2	11MAY2000	Yes	11MAY2000	8:13
		Load Dose 3	12MAY2000	Yes	12MAY2000	6:25
		Week 1	17MAY2000	Yes	17MAY2000	7:55
		Week 1 (Unsch.)	17MAY2000	No		
		Week 2	24MAY2000	Yes	24MAY2000	9:29
		Week 3	31MAY2000	Yes	31MAY2000	7:20
		Week 4	06JUN2000	Yes	06JUN2000	8:55
		Week 5	14JUN2000	Yes	14JUN2000	7:16
		Week 6	21JUN2000	Yes	21JUN2000	7:30
		Week 7	28JUN2000	Yes	28JUN2000	6:57
		Week 7 (Unsch.)	29JUN2000	No		
		Week 8	04JUL2000	Yes	04JUL2000	6:55
		Week 9	12JUL2000	Yes	12JUL2000	7:05

*Popn=Population (I=Intent-to-Treat, PP=Per Protocol)
Tafenoquine = Tafenoquine 200mg, Mefloquine = Mefloquine 250mg

SB252263 - 030

Listing B.02

Visit And Dosing Dates

Treatment : Tafenoquine

Subject Number	Popn*	Visit	Visit Date	Dose Taken ?	Date of Dose	Time of Dose
030.001.00264	IPP	Week 10	19JUL2000	Yes	19JUL2000	7:55
		Week 11 (Fol. Up)	26JUL2000	No		
		Week 13 (Fol. Up)	11AUG2000	No		
		Week 14 (Fol. Up)	17AUG2000	No		
		Week 14 (Fol. Up)	18AUG2000	No		
030.001.00266	I	Screening	12APR2000	No		
		Clearance (Day -5)	05MAY2000	Yes	05MAY2000	
		Clearance (Day -4)	06MAY2000	Yes	06MAY2000	
		Clearance (Day -3)	07MAY2000	Yes	07MAY2000	
		Load Dose 1	10MAY2000	Yes	10MAY2000	11:52
		Load Dose 2	11MAY2000	Yes	11MAY2000	10:20
		Load Dose 3	12MAY2000	Yes	12MAY2000	8:34
		Week 1	17MAY2000	Yes	17MAY2000	8:50
		Week 2 (Unsch.)	22MAY2000	No		
		Week 2	24MAY2000	Yes	24MAY2000	8:31
		Week 5 (Fol. Up)	15JUN2000	No		
		Week 6 (Unsch. Fol. Up)	20JUN2000	No		
		Week 6 (Fol. Up)	21JUN2000	No		
030.001.00267	IPP	Screening	11APR2000	No		
		Clearance (Day -5)	05MAY2000	Yes	05MAY2000	
		Clearance (Day -4)	06MAY2000	Yes	06MAY2000	
		Clearance (Day -3)	07MAY2000	Yes	07MAY2000	
		Clearance (Unsch.)	08MAY2000	No		
		Load Dose 1	10MAY2000	Yes	10MAY2000	12:16
		Load Dose 2	11MAY2000	Yes	11MAY2000	11:43
		Load Dose 3	12MAY2000	Yes	12MAY2000	7:27
		Week 1 (Unsch.)	15MAY2000	No		
		Week 1	17MAY2000	Yes	17MAY2000	8:45
		Week 2	24MAY2000	Yes	24MAY2000	16:00
		Week 3	31MAY2000	Yes	31MAY2000	8:37
		Week 4	06JUN2000	Yes	06JUN2000	12:40
		Week 5	14JUN2000	Yes	14JUN2000	7:32
		Week 6	21JUN2000	Yes	21JUN2000	9:04
		Week 7 (Fol. Up)	28JUN2000	No		
		Week 8 (Fol. Up)	04JUL2000	No		
		Week 8 (Unsch. Fol. Up)	07JUL2000	No		
		Week 9 (Fol. Up)	13JUL2000	No		
		Week 10 (Fol. Up)	19JUL2000	No		

*Popn=Population (I=Intent-to-Treat, PP=Per Protocol)
Tafenoquine = Tafenoquine 200mg, Mefloquine = Mefloquine 250mg

SB252263 - 030

Listing B.02

Visit And Dosing Dates

Treatment : Tafenoquine

Subject Number	Popn*	Visit	Visit Date	Dose Taken ?	Date of Dose	Time of Dose
030.001.00271	I	Screening	13APR2000	No		
		Clearance (Day -5)	05MAY2000	Yes	05MAY2000	
		Clearance (Day -4)	06MAY2000	Yes	06MAY2000	
		Clearance (Day -3)	07MAY2000	Yes	07MAY2000	
		Load Dose 1	10MAY2000	Yes	10MAY2000	15:50
		Load Dose 2	11MAY2000	Yes	11MAY2000	6:25
		Load Dose 3	12MAY2000	Yes	12MAY2000	6:30
		Week 1	17MAY2000	Yes	17MAY2000	6:30
		Week 2	24MAY2000	Yes	24MAY2000	6:00
		Week 3	31MAY2000	Yes	31MAY2000	5:54
		Week 5 (Fol. Up)	11JUN2000	No		
		Week 5 (Fol. Up)	14JUN2000	No		
030.001.00272	IPP	Screening	10APR2000	No		
		Clearance (Day -5)	05MAY2000	Yes	05MAY2000	
		Clearance (Day -4)	06MAY2000	Yes	06MAY2000	
		Clearance (Day -3)	07MAY2000	Yes	07MAY2000	
		Load Dose 1	10MAY2000	Yes	10MAY2000	16:32
		Load Dose 2	11MAY2000	Yes	11MAY2000	7:48
		Load Dose 3	12MAY2000	Yes	12MAY2000	8:01
		Week 1	17MAY2000	Yes	17MAY2000	7:15
		Week 2	24MAY2000	Yes	24MAY2000	6:30
		Week 3	31MAY2000	Yes	31MAY2000	7:05
		Week 3 (Unsch.)	01JUN2000	No		
		Week 4	07JUN2000	Yes	07JUN2000	12:01
		Week 5 (Unsch.)	12JUN2000	No		
		Week 5	14JUN2000	Yes	14JUN2000	10:59
		Week 6	23JUN2000	Yes	23JUN2000	6:15
		Week 7	28JUN2000	Yes	28JUN2000	7:46
		Week 8	04JUL2000	Yes	04JUL2000	6:49
		Week 8	05JUL2000	No		
		Week 9	12JUL2000	Yes	12JUL2000	7:08
		Week 10	19JUL2000	Yes	19JUL2000	7:52
		Week 11	26JUL2000	Yes	26JUL2000	7:49
		Week 15 (Fol. Up)	23AUG2000	No		
030.001.00279	IPP	Screening	12APR2000	No		
		Clearance (Day -5)	04MAY2000	Yes	04MAY2000	
		Clearance (Day -4)	05MAY2000	Yes	05MAY2000	
		Clearance (Day -3)	06MAY2000	Yes	06MAY2000	
		Load Dose 1	11MAY2000	Yes	11MAY2000	8:47
		Load Dose 2	12MAY2000	Yes	12MAY2000	9:49

*Popn=Population (I=Intent-to-Treat, PP=Per Protocol)
Tafenoquine = Tafenoquine 200mg, Mefloquine = Mefloquine 250mg

Listing B.02

Visit And Dosing Dates

Treatment : Tafenoquine

Subject Number	Popn*	Visit	Visit Date	Dose Taken ?	Date of Dose	Time of Dose
030.001.00279	IPP	Load Dose 3	13MAY2000	Yes	13MAY2000	13:59
		Week 1	19MAY2000	Yes	19MAY2000	13:24
		Week 2 (Fol. Up)	25MAY2000	No		
		Week 3 (Fol. Up)	31MAY2000	No		
		Week 4 (Fol. Up)	06JUN2000	No		
		Week 5 (Unsch. Fol. Up)	12JUN2000	No		
		Week 5 (Fol. Up)	14JUN2000	No		
		Week 5 (Unsch. Fol. Up)	17JUN2000	No		
030.001.00282	IPP	Screening	11APR2000	No		
		Clearance (Day -5)	05MAY2000	Yes	05MAY2000	
		Clearance (Day -4)	06MAY2000	Yes	06MAY2000	
		Clearance (Day -3)	07MAY2000	Yes	07MAY2000	
		Load Dose 1	10MAY2000	Yes	10MAY2000	19:14
		Load Dose 2	11MAY2000	Yes	11MAY2000	10:10
		Load Dose 3	12MAY2000	Yes	12MAY2000	7:34
		Load Dose (Unsch.)	10MAY2000	No		
		Week 1	17MAY2000	Yes	17MAY2000	8:19
		Week 2 (Unsch.)	22MAY2000	No		
		Week 2	24MAY2000	Yes	24MAY2000	8:17
		Week 3	29MAY2000	Yes	29MAY2000	14:59
		Week 4	06JUN2000	Yes	06JUN2000	15:50
		Week 5 (Unsch.)	13JUN2000	No		
		Week 5	14JUN2000	Yes	14JUN2000	7:50
		Week 6 (Unsch.)	18JUN2000	No		
		Week 6	21JUN2000	Yes	21JUN2000	6:34
		Week 7	28JUN2000	Yes	28JUN2000	6:41
		Week 8	04JUL2000	Yes	04JUL2000	8:01
		Week 9	12JUL2000	Yes	12JUL2000	8:20
		Week 10	19JUL2000	Yes	19JUL2000	7:58
		Week 11	26JUL2000	Yes	26JUL2000	6:40
		Week 11 (Unsch.)	29JUL2000	No		
		Week 12	01AUG2000	Yes	01AUG2000	18:00
		Week 13	12AUG2000	Yes	12AUG2000	19:27
		Week 14	16AUG2000	Yes	16AUG2000	6:52
		Week 15	23AUG2000	Yes	23AUG2000	7:05
		Week 16	28AUG2000	Yes	28AUG2000	9:44
		Week 16	30AUG2000	No		
		Week 17	04SEP2000	Yes	04SEP2000	17:22
		Week 18	13SEP2000	Yes	13SEP2000	7:06
		Week 18	13SEP2000	Yes	13SEP2000	13:14
		Week 18 (Unsch.)	13SEP2000	No		
		Week 19 (Fol. Up)	20SEP2000	No		

*Popn=Population (I=Intent-to-Treat, PP=Per Protocol)
Tafenoquine = Tafenoquine 200mg, Mefloquine = Mefloquine 250mg

SB252263 - 030

Listing B.02

Visit And Dosing Dates

Treatment : Tafenoquine

Subject Number	Popn*	Visit	Visit Date	Dose Taken ?	Date of Dose	Time of Dose
030.001.00282	IPP	Week 20 (Fol. Up)	27SEP2000	No		
		Week 21 (Unsch. Fol. Up)	03OCT2000	No		
		Week 21 (Fol. Up)	04OCT2000	No		
		Week 22 (Fol. Up)	13OCT2000	No		
030.001.00284	IPP	Screening	10APR2000	No		
		Clearance (Day -5)	07MAY2000	Yes	07MAY2000	
		Clearance (Day -4)	08MAY2000	Yes	08MAY2000	
		Clearance (Day -3)	09MAY2000	Yes	09MAY2000	
		Load Dose 1	11MAY2000	Yes	11MAY2000	7:53
		Load Dose 2	12MAY2000	Yes	12MAY2000	12:18
		Load Dose 3	13MAY2000	Yes	13MAY2000	15:34
		Week 1 (Unsch.)	14MAY2000	No		
		Week 1	18MAY2000	Yes	18MAY2000	6:28
		Week 2	24MAY2000	Yes	24MAY2000	12:12
		Week 3	30MAY2000	Yes	30MAY2000	10:27
		Week 3 (Unsch.)	31MAY2000	No		
		Week 4	07JUN2000	Yes	07JUN2000	10:02
		Week 5	14JUN2000	Yes	14JUN2000	12:38
		Week 6	21JUN2000	Yes	21JUN2000	6:13
		Week 7	28JUN2000	Yes	28JUN2000	10:32
		Week 8	04JUL2000	Yes	04JUL2000	7:00
		Week 9	12JUL2000	Yes	12JUL2000	7:02
		Week 10	19JUL2000	Yes	19JUL2000	7:25
		Week 11	26JUL2000	Yes	26JUL2000	12:00
		Week 12	02AUG2000	Yes	02AUG2000	6:00
		Week 13	09AUG2000	Yes	09AUG2000	6:47
		Week 14	16AUG2000	Yes	16AUG2000	6:50
		Week 15	23AUG2000	Yes	23AUG2000	20:10
		Week 15 (Unsch.)	24AUG2000	No		
		Week 16	28AUG2000	Yes	28AUG2000	19:41
		Week 16	30AUG2000	No		
		Week 17	06SEP2000	Yes	06SEP2000	6:24
		Week 18	13SEP2000	Yes	13SEP2000	6:25
		Week 19	20SEP2000	Yes	20SEP2000	6:30
		Week 20	27SEP2000	Yes	27SEP2000	7:07
		Week 21	04OCT2000	Yes	04OCT2000	6:30
		Week 22	11OCT2000	Yes	11OCT2000	6:10
		Week 23	16OCT2000	Yes	16OCT2000	16:15
		Week 24	23OCT2000	Yes	23OCT2000	6:28
		Week 25 (Fol. Up)	01NOV2000	No		
		Week 26 (Fol. Up)	09NOV2000	No		
		Week 27 (Fol. Up)	15NOV2000	No		

*Popn=Population (I=Intent-to-Treat, PP=Per Protocol)
Tafenoquine = Tafenoquine 200mg, Mefloquine = Mefloquine 250mg

SB252263 - 030

Listing B.02

Visit And Dosing Dates

Treatment : Tafenoquine

Subject Number	Popn*	Visit	Visit Date	Dose Taken ?	Date of Dose	Time of Dose
030.001.00284	IPP	Week 28 (Fol. Up)	20NOV2000	No		
030.001.00288	IPP	Screening	12APR2000	No		
		Screening (Unsch.)	27APR2000	No		
		Clearance (Day -5)	05MAY2000	Yes	05MAY2000	
		Clearance (Day -4)	06MAY2000	Yes	06MAY2000	
		Clearance (Day -3)	07MAY2000	Yes	07MAY2000	
		Load Dose 1	11MAY2000	Yes	11MAY2000	9:38
		Load Dose 2	12MAY2000	Yes	12MAY2000	11:37
		Load Dose 3	13MAY2000	Yes	13MAY2000	8:40
		Week 1 (Unsch.)	17MAY2000	No		
		Week 1	18MAY2000	Yes	18MAY2000	7:15
		Week 2	24MAY2000	Yes	24MAY2000	10:36
		Week 3	31MAY2000	Yes	31MAY2000	9:48
		Week 4	06JUN2000	Yes	06JUN2000	12:10
		Week 4 (Unsch. Fol. Up)	09JUN2000	No		
		Week 5 (Fol. Up)	14JUN2000	No		
		Week 6 (Fol. Up)	21JUN2000	No		
		Week 7 (Fol. Up)	28JUN2000	No		
		Week 8 (Fol. Up)	04JUL2000	No		
030.001.00293	IPP	Screening	11APR2000	No		
		Screening (Unsch.)	29APR2000	No		
		Clearance (Day -5)	05MAY2000	Yes	05MAY2000	
		Clearance (Day -4)	06MAY2000	Yes	06MAY2000	
		Clearance (Day -3)	07MAY2000	Yes	07MAY2000	
		Load Dose 1	11MAY2000	Yes	11MAY2000	12:19
		Load Dose 2	12MAY2000	Yes	12MAY2000	12:00
		Load Dose 3	13MAY2000	Yes	13MAY2000	10:09
		Week 1	18MAY2000	Yes	18MAY2000	6:18
		Week 2	24MAY2000	Yes	24MAY2000	12:20
		Week 2 (Unsch.)	27MAY2000	No		
		Week 3	01JUN2000	Yes	01JUN2000	8:20
		Week 4	07JUN2000	Yes	07JUN2000	15:15
		Week 5	14JUN2000	Yes	14JUN2000	8:55
		Week 5 (Unsch.)	15JUN2000	No		
		Week 6 (Unsch.)	20JUN2000	No		
		Week 6	21JUN2000	Yes	21JUN2000	6:20
		Week 8	08JUL2000	Yes	08JUL2000	17:00
		Week 9	10JUL2000	No		
		Week 9 (Unsch.)	10JUL2000	No		
		Week 9	13JUL2000	Yes	13JUL2000	10:00

*Popn=Population (I=Intent-to-Treat, PP=Per Protocol)
Tafenoquine = Tafenoquine 200mg, Mefloquine = Mefloquine 250mg

SB252263 - 030

Listing B.02

Visit And Dosing Dates

Treatment : Tafenoquine

Subject Number	Popn*	Visit	Visit Date	Dose Taken ?	Date of Dose	Time of Dose
030.001.00293	IPP	Week 9 (Unsch.)	14JUL2000	No		
		Week 10	18JUL2000	Yes	18JUL2000	11:45
		Week 11	25JUL2000	Yes	25JUL2000	10:30
		Week 12	04AUG2000	Yes	04AUG2000	10:11
		Week 12 (Unsch.)	04AUG2000	No		
		Week 13	09AUG2000	Yes	09AUG2000	11:10
		Week 14	15AUG2000	Yes	15AUG2000	12:00
		Week 15	23AUG2000	Yes	23AUG2000	12:10
		Week 16	29AUG2000	Yes	29AUG2000	13:47
		Week 16	31AUG2000	No		
		Week 17	07SEP2000	Yes	07SEP2000	10:10
		Week 18	14SEP2000	Yes	14SEP2000	10:40
		Week 19	20SEP2000	Yes	20SEP2000	10:10
		Week 20	25SEP2000	Yes	25SEP2000	15:40
		Week 21	04OCT2000	Yes	04OCT2000	12:30
		Week 22	12OCT2000	Yes	12OCT2000	11:15
		Week 23	19OCT2000	Yes	19OCT2000	12:00
		Week 24	25OCT2000	Yes	25OCT2000	10:40
		Week 25 (Fol. Up)	01NOV2000	No		
		Week 27 (Fol. Up)	13NOV2000	No		
030.001.00294	IPP	Screening	13APR2000	No		
		Clearance (Day -5)	05MAY2000	Yes	05MAY2000	
		Clearance (Day -4)	06MAY2000	Yes	06MAY2000	
		Clearance (Day -3)	07MAY2000	Yes	07MAY2000	
		Load Dose 1	11MAY2000	Yes	11MAY2000	14:11
		Load Dose 2	12MAY2000	Yes	12MAY2000	6:58
		Load Dose 3	13MAY2000	Yes	13MAY2000	10:42
		Week 1 (Unsch.)	15MAY2000	No		
		Week 1	18MAY2000	Yes	18MAY2000	7:13
		Week 2	24MAY2000	Yes	24MAY2000	6:19
		Week 3	31MAY2000	Yes	31MAY2000	6:22
		Week 4	06JUN2000	Yes	06JUN2000	13:07
		Week 5	14JUN2000	Yes	14JUN2000	7:19
		Week 6	21JUN2000	Yes	21JUN2000	8:10
		Week 7	28JUN2000	Yes	28JUN2000	15:35
		Week 8	03JUL2000	Yes	03JUL2000	9:10
		Week 8 (Unsch.)	05JUL2000	No		
		Week 9	12JUL2000	Yes	12JUL2000	9:50
		Week 10 (Fol. Up)	19JUL2000	No		
		Week 11 (Fol. Up)	26JUL2000	No		
		Week 12 (Fol. Up)	01AUG2000	No		

*Popn=Population (I=Intent-to-Treat, PP=Per Protocol)
Tafenoquine = Tafenoquine 200mg, Mefloquine = Mefloquine 250mg

SB252263 - 030

Listing B.02

Visit And Dosing Dates

Treatment : Tafenoquine

Subject Number	Popn*	Visit	Visit Date	Dose Taken ?	Date of Dose	Time of Dose
030.001.00296	IPP	Screening	14APR2000	No		
		Clearance (Day -5)	08MAY2000	Yes	08MAY2000	
		Clearance (Day -4)	09MAY2000	Yes	09MAY2000	
		Clearance (Day -3)	10MAY2000	Yes	10MAY2000	
		Load Dose 1	11MAY2000	Yes	11MAY2000	16:20
		Load Dose 2	12MAY2000	Yes	12MAY2000	6:28
		Load Dose 3	13MAY2000	Yes	13MAY2000	11:27
		Week 1	18MAY2000	Yes	18MAY2000	6:48
		Week 2	24MAY2000	Yes	24MAY2000	7:08
		Week 2 (Unsch.)	24MAY2000	No		
		Week 3	31MAY2000	Yes	31MAY2000	6:51
		Week 4	06JUN2000	Yes	06JUN2000	12:20
		Week 5	14JUN2000	Yes	14JUN2000	7:00
		Week 5 (Unsch.)	15JUN2000	No		
		Week 6	21JUN2000	Yes	21JUN2000	7:13
		Week 7	28JUN2000	Yes	28JUN2000	7:15
		Week 8	03JUL2000	Yes	03JUL2000	10:13
		Week 9	12JUL2000	Yes	12JUL2000	7:34
		Week 10	19JUL2000	Yes	19JUL2000	7:20
		Week 11	26JUL2000	Yes	26JUL2000	7:20
		Week 12	31JUL2000	Yes	31JUL2000	9:15
		Week 13	09AUG2000	Yes	09AUG2000	7:20
		Week 14	16AUG2000	Yes	16AUG2000	7:31
		Week 15	23AUG2000	Yes	23AUG2000	8:34
		Week 16	28AUG2000	Yes	28AUG2000	8:21
		Week 16	30AUG2000	No		
		Week 17	04SEP2000	Yes	04SEP2000	10:30
		Week 18	13SEP2000	Yes	13SEP2000	7:31
		Week 19	20SEP2000	Yes	20SEP2000	7:15
		Week 20	27SEP2000	Yes	27SEP2000	10:40
		Week 20 (Unsch.)	27SEP2000	No		
		Week 21	04OCT2000	Yes	04OCT2000	7:35
		Week 22	11OCT2000	Yes	11OCT2000	8:15
		Week 23	17OCT2000	Yes	17OCT2000	10:50
		Week 24	23OCT2000	Yes	23OCT2000	11:07
		Week 25 (Fol. Up)	02NOV2000	No		
		Week 26 (Fol. Up)	08NOV2000	No		
		Week 27 (Fol. Up)	15NOV2000	No		
		Week 28 (Fol. Up)	20NOV2000	No		
030.001.00298	IPP	Screening	17APR2000	No		
		Clearance (Day -5)	05MAY2000	Yes	05MAY2000	
		Clearance (Day -4)	06MAY2000	Yes	06MAY2000	

*Popn=Population (I=Intent-to-Treat, PP=Per Protocol)
Tafenoquine = Tafenoquine 200mg, Mefloquine = Mefloquine 250mg

SB252263 - 030

Listing B.02

Visit And Dosing Dates

Treatment : Tafenoquine

Subject Number	Popn*	Visit	Visit Date	Dose Taken ?	Date of Dose	Time of Dose
030.001.00298	IPP	Clearance (Day -3)	07MAY2000	Yes	07MAY2000	
		Load Dose 1	12MAY2000	Yes	12MAY2000	7:30
		Load Dose 2	13MAY2000	Yes	13MAY2000	14:25
		Load Dose 3	14MAY2000	Yes	14MAY2000	18:20
		Week 1	18MAY2000	Yes	18MAY2000	6:10
		Week 1 (Unsch.)	18MAY2000	No		
		Week 2	24MAY2000	Yes	24MAY2000	6:20
		Week 2 (Unsch.)	25MAY2000	No		
		Week 3	31MAY2000	Yes	31MAY2000	6:53
		Week 3 (Unsch.)	01JUN2000	No		
		Week 4	06JUN2000	Yes	06JUN2000	14:41
		Week 5	14JUN2000	Yes	14JUN2000	6:50
		Week 6	21JUN2000	Yes	21JUN2000	6:50
		Week 7	28JUN2000	Yes	28JUN2000	6:53
		Week 8	04JUL2000	Yes	04JUL2000	6:31
		Week 9	12JUL2000	Yes	12JUL2000	7:00
		Week 9 (Unsch.)	12JUL2000	No		
		Week 10	19JUL2000	Yes	19JUL2000	6:45
		Week 10 (Unsch.)	20JUL2000	No		
		Week 11	26JUL2000	Yes	26JUL2000	19:54
		Week 12	02AUG2000	Yes	02AUG2000	6:46
		Week 13 (Fol. Up)	09AUG2000	No		
		Week 15 (Fol. Up)	24AUG2000	No		
		Week 16 (Fol. Up)	30AUG2000	No		
030.001.00301	IPP	Screening	14APR2000	No		
		Clearance (Day -5)	05MAY2000	Yes	05MAY2000	
		Clearance (Day -4)	06MAY2000	Yes	06MAY2000	
		Clearance (Day -3)	07MAY2000	Yes	07MAY2000	
		Load Dose 1	12MAY2000	Yes	12MAY2000	9:45
		Load Dose 2	13MAY2000	Yes	13MAY2000	9:26
		Load Dose 3	14MAY2000	Yes	14MAY2000	10:50
		Week 1	17MAY2000	Yes	17MAY2000	7:40
		Week 2	24MAY2000	Yes	24MAY2000	6:16
		Week 3	31MAY2000	Yes	31MAY2000	10:09
		Week 4	07JUN2000	Yes	07JUN2000	13:24
		Week 5	14JUN2000	Yes	14JUN2000	6:58
		Week 6	21JUN2000	Yes	21JUN2000	6:31
		Week 7	28JUN2000	Yes	28JUN2000	6:32
		Week 8	04JUL2000	Yes	04JUL2000	5:55
		Week 8 (Fol. Up)	06JUL2000	No		
		Week 9 (Fol. Up)	12JUL2000	No		
		Week 9 (Unsch. Fol. Up)	15JUL2000	No		

*Popn=Population (I=Intent-to-Treat, PP=Per Protocol)
Tafenoquine = Tafenoquine 200mg, Mefloquine = Mefloquine 250mg

SB252263 - 030

Listing B.02

Visit And Dosing Dates

Treatment : Tafenoquine

Subject Number	Popn*	Visit	Visit Date	Dose Taken ?	Date of Dose	Time of Dose
030.001.00301	IPP	Week 9 (Unsch. Fol. Up)	16JUL2000	No		
		Week 10 (Fol. Up)	19JUL2000	No		
		Week 11 (Fol. Up)	26JUL2000	No		
030.001.00302	I	Screening	14APR2000	No		
		Clearance (Day -5)	05MAY2000	Yes	05MAY2000	
		Clearance (Day -4)	06MAY2000	Yes	06MAY2000	
		Clearance (Day -3)	07MAY2000	Yes	07MAY2000	
		Load Dose 1	12MAY2000	Yes	12MAY2000	13:52
		Load Dose 2	13MAY2000	Yes	13MAY2000	13:24
		Week 1	21MAY2000	Yes	21MAY2000	8:55
		Week 2 (Fol. Up)	26MAY2000	No		
		Week 3 (Fol. Up)	31MAY2000	No		
		Week 4 (Fol. Up)	07JUN2000	No		
		Week 5 (Unsch. Fol. Up)	14JUN2000	No		
030.001.00304	IPP	Screening	13APR2000	No		
		Clearance (Day -5)	05MAY2000	Yes	05MAY2000	
		Clearance (Day -4)	06MAY2000	Yes	06MAY2000	
		Clearance (Day -3)	07MAY2000	Yes	07MAY2000	
		Load Dose 1	12MAY2000	Yes	12MAY2000	13:45
		Load Dose 2	13MAY2000	Yes	13MAY2000	13:49
		Load Dose 3	14MAY2000	Yes	14MAY2000	9:35
		Week 1	18MAY2000	Yes	18MAY2000	13:05
		Week 2	24MAY2000	Yes	24MAY2000	5:54
		Week 3	31MAY2000	Yes	31MAY2000	6:23
		Week 3 (Unsch.)	04JUN2000	No		
		Week 4	07JUN2000	Yes	07JUN2000	19:07
		Week 5	14JUN2000	Yes	14JUN2000	8:51
		Week 5 (Unsch.)	15JUN2000	No		
		Week 6	21JUN2000	Yes	21JUN2000	6:31
		Week 7	28JUN2000	Yes	28JUN2000	6:32
		Week 8	04JUL2000	Yes	04JUL2000	6:00
		Week 8 (Fol. Up)	05JUL2000	No		
		Week 9 (Fol. Up)	12JUL2000	No		
		Week 10 (Fol. Up)	19JUL2000	No		
		Week 11 (Fol. Up)	26JUL2000	No		
030.001.00306	IPP	Screening	13APR2000	No		
		Clearance (Day -5)	05MAY2000	Yes	05MAY2000	
		Clearance (Day -4)	06MAY2000	Yes	06MAY2000	

*Popn=Population (I=Intent-to-Treat, PP=Per Protocol)
Tafenoquine = Tafenoquine 200mg, Mefloquine = Mefloquine 250mg

SB252263 - 030

Listing B.02

Visit And Dosing Dates

Treatment : Tafenoquine

Subject Number	Popn*	Visit	Visit Date	Dose Taken ?	Date of Dose	Time of Dose
030.001.00306	IPP	Clearance (Day -3)	07MAY2000	Yes	07MAY2000	
		Load Dose 1	12MAY2000	Yes	12MAY2000	17:50
		Load Dose 2	13MAY2000	Yes	13MAY2000	10:55
		Load Dose 3	14MAY2000	Yes	14MAY2000	9:06
		Week 1	18MAY2000	Yes	18MAY2000	8:45
		Week 2	25MAY2000	Yes	25MAY2000	11:59
		Week 3	31MAY2000	Yes	31MAY2000	9:10
		Week 4	09JUN2000	Yes	09JUN2000	10:06
		Week 4 (Unsch.)	12JUN2000	No		
		Week 5	14JUN2000	Yes	14JUN2000	10:00
		Week 6	21JUN2000	Yes	21JUN2000	8:20
		Week 7	28JUN2000	Yes	28JUN2000	9:09
		Week 8	04JUL2000	Yes	04JUL2000	14:59
		Week 8 (Unsch.)	08JUL2000	No		
		Week 9	12JUL2000	Yes	12JUL2000	11:05
		Week 10	19JUL2000	Yes	19JUL2000	9:10
		Week 11	26JUL2000	Yes	26JUL2000	8:30
		Week 12	02AUG2000	Yes	02AUG2000	13:20
		Week 13 (Fol. Up)	10AUG2000	No		
		Week 14 (Fol. Up)	16AUG2000	No		
		Week 14 (Unsch. Fol. Up)	16AUG2000	No		
		Week 14 (Unsch. Fol. Up)	17AUG2000	No		
		Week 15 (Fol. Up)	23AUG2000	No		

*Popn=Population (I=Intent-to-Treat, PP=Per Protocol)
Tafenoquine = Tafenoquine 200mg, Mefloquine = Mefloquine 250mg

Listing B.02

Visit And Dosing Dates

Treatment : Mefloquine

Subject Number	Popn*	Visit	Visit Date	Dose Taken ?	Date of Dose	Time of Dose
030.001.00003	I	Screening	10APR2000	No		
		Clearance (Day -5)	03MAY2000	Yes	03MAY2000	
		Clearance (Day -4)	04MAY2000	Yes	04MAY2000	
		Clearance (Day -3)	05MAY2000	Yes	05MAY2000	
		Load Dose 1	08MAY2000	Yes	08MAY2000	7:17
		Load Dose 2	09MAY2000	Yes	09MAY2000	8:02
		Load Dose 3	10MAY2000	Yes	10MAY2000	6:59
		Load Dose (Unsch.)	10MAY2000	No		
		Week 1 (Unsch.)	12MAY2000	No		
		Week 1	15MAY2000	Yes	15MAY2000	7:30
		Week 1 (Unsch.)	15MAY2000	No		
		Week 2	22MAY2000	Yes	22MAY2000	8:05
		Week 3	29MAY2000	Yes	29MAY2000	8:15
		Week 3 (Unsch.)	31MAY2000	No		
		Week 4	05JUN2000	Yes	05JUN2000	14:10
		Week 6	16JUN2000	Yes	16JUN2000	18:30
		Week 6 (Fol. Up)	22JUN2000	No		
		Week 7 (Fol. Up)	26JUN2000	No		
		Week 8 (Fol. Up)	03JUL2000	No		
		Week 9 (Fol. Up)	10JUL2000	No		
030.001.00007	IPP	Screening	11APR2000	No		
		Clearance (Day -5)	03MAY2000	Yes	03MAY2000	
		Clearance (Day -4)	04MAY2000	Yes	04MAY2000	
		Clearance (Day -3)	05MAY2000	Yes	05MAY2000	
		Load Dose 1	08MAY2000	Yes	08MAY2000	7:30
		Load Dose 2	09MAY2000	Yes	09MAY2000	7:15
		Load Dose 3	10MAY2000	Yes	10MAY2000	7:32
		Week 1 (Unsch.)	11MAY2000	No		
		Week 1	15MAY2000	Yes	15MAY2000	6:55
		Week 2 (Unsch.)	19MAY2000	No		
		Week 2	22MAY2000	Yes	22MAY2000	6:56
		Week 3	29MAY2000	Yes	29MAY2000	7:30
		Week 4	05JUN2000	Yes	05JUN2000	8:30
		Week 5	12JUN2000	Yes	12JUN2000	6:29
		Week 6	19JUN2000	Yes	19JUN2000	7:30
		Week 7 (Unsch.)	23JUN2000	No		
		Week 7	26JUN2000	Yes	26JUN2000	6:20
		Week 8	03JUL2000	Yes	03JUL2000	7:50
		Week 9	10JUL2000	Yes	10JUL2000	7:30
		Week 9 (Unsch.)	13JUL2000	No		
		Week 10	17JUL2000	Yes	17JUL2000	7:46
		Week 11	24JUL2000	Yes	24JUL2000	7:50

*Popn=Population (I=Intent-to-Treat, PP=Per Protocol)
Tafenoquine = Tafenoquine 200mg, Mefloquine = Mefloquine 250mg

SB252263 - 030

Listing B.02

Visit And Dosing Dates

Treatment : Mefloquine

Subject Number	Popn*	Visit	Visit Date	Dose Taken ?	Date of Dose	Time of Dose
030.001.00007	IPP	Week 12 (Unsch.)	29JUL2000	No		
		Week 12	31JUL2000	Yes	31JUL2000	
		Week 13	07AUG2000	Yes	07AUG2000	10:08
		Week 14	14AUG2000	Yes	14AUG2000	10:05
		Week 15	21AUG2000	Yes	21AUG2000	8:45
		Week 16	28AUG2000	Yes	28AUG2000	10:00
		Week 16	30AUG2000	No		
		Week 17	04SEP2000	Yes	04SEP2000	10:30
		Week 18	11SEP2000	Yes	11SEP2000	9:30
		Week 19	18SEP2000	Yes	18SEP2000	9:53
		Week 20	25SEP2000	Yes	25SEP2000	14:20
		Week 21	02OCT2000	Yes	02OCT2000	9:45
		Week 22	09OCT2000	Yes	09OCT2000	10:44
		Week 23	16OCT2000	Yes	16OCT2000	15:15
		Week 23 (Unsch. Fol. Up)	19OCT2000	No		
		Week 24 (Fol. Up)	23OCT2000	No		
		Week 24 (Fol. Up)	24OCT2000	No		
		Week 24 (Unsch. Fol. Up)	24OCT2000	No		
		Week 25 (Fol. Up)	30OCT2000	No		
		Week 26 (Fol. Up)	06NOV2000	No		
		Week 27 (Fol. Up)	14NOV2000	No		
030.001.00008	IPP	Screening	11APR2000	No		
		Screening (Unsch.)	18APR2000	No		
		Screening (Unsch.)	23APR2000	No		
		Clearance (Day -5)	03MAY2000	Yes	03MAY2000	
		Clearance (Day -4)	04MAY2000	Yes	04MAY2000	
		Clearance (Day -3)	05MAY2000	Yes	05MAY2000	
		Clearance (Unsch.)	07MAY2000	No		
		Load Dose 1	08MAY2000	Yes	08MAY2000	6:55
		Load Dose 2	09MAY2000	Yes	09MAY2000	6:30
		Load Dose 3	10MAY2000	Yes	10MAY2000	7:15
		Week 1 (Unsch.)	14MAY2000	No		
		Week 1	15MAY2000	Yes	15MAY2000	11:30
		Week 2	22MAY2000	Yes	22MAY2000	6:15
		Week 3	29MAY2000	Yes	29MAY2000	7:01
		Week 4	05JUN2000	Yes	05JUN2000	9:54
		Week 5	12JUN2000	Yes	12JUN2000	7:17
		Week 6 (Unsch.)	17JUN2000	No		
		Week 6	19JUN2000	Yes	19JUN2000	7:50
		Week 7	26JUN2000	Yes	26JUN2000	7:28
		Week 8	03JUL2000	Yes	03JUL2000	6:35
		Week 9	10JUL2000	Yes	10JUL2000	6:45

*Popn=Population (I=Intent-to-Treat, PP=Per Protocol)
Tafenoquine = Tafenoquine 200mg, Mefloquine = Mefloquine 250mg

SB252263 - 030

Listing B.02

Visit And Dosing Dates

Treatment : Mefloquine

Subject Number	Popn*	Visit	Visit Date	Dose Taken ?	Date of Dose	Time of Dose
030.001.00008	IPP	Week 10	17JUL2000	Yes	17JUL2000	6:40
		Week 11	24JUL2000	Yes	24JUL2000	7:10
		Week 12 (Unsch.)	29JUL2000	No		
		Week 12	31JUL2000	Yes	31JUL2000	6:35
		Week 12 (Unsch.)	03AUG2000	No		
		Week 13	07AUG2000	Yes	07AUG2000	7:50
		Week 14	14AUG2000	Yes	14AUG2000	10:40
		Week 15	21AUG2000	Yes	21AUG2000	7:00
		Week 16	28AUG2000	Yes	28AUG2000	7:20
		Week 16	30AUG2000	No		
		Week 17	04SEP2000	Yes	04SEP2000	6:45
		Week 18	11SEP2000	Yes	11SEP2000	6:40
		Week 18 (Unsch.)	13SEP2000	No		
		Week 19	18SEP2000	Yes	18SEP2000	6:45
		Week 20	25SEP2000	Yes	25SEP2000	6:45
		Week 21	02OCT2000	Yes	02OCT2000	7:00
		Week 22	09OCT2000	Yes	09OCT2000	7:35
		Week 23	16OCT2000	Yes	16OCT2000	6:55
		Week 24 (Unsch. Fol. Up)	21OCT2000	No		
		Week 24 (Fol. Up)	23OCT2000	No		
		Week 24 (Unsch. Fol. Up)	23OCT2000	No		
		Week 26 (Fol. Up)	07NOV2000	No		
030.001.00010	IPP	Screening	11APR2000	No		
		Screening (Unsch.)	16APR2000	No		
		Clearance (Day -5)	03MAY2000	Yes	03MAY2000	
		Clearance (Day -4)	04MAY2000	Yes	04MAY2000	
		Clearance (Day -3)	05MAY2000	Yes	05MAY2000	
		Load Dose 1	08MAY2000	Yes	08MAY2000	7:25
		Load Dose 2	09MAY2000	Yes	09MAY2000	6:30
		Load Dose 3	10MAY2000	Yes	10MAY2000	7:50
		Week 1	15MAY2000	Yes	15MAY2000	9:15
		Week 2	22MAY2000	Yes	22MAY2000	8:50
		Week 2 (Unsch.)	22MAY2000	No		
		Week 3	29MAY2000	Yes	29MAY2000	5:40
		Week 4	05JUN2000	Yes	05JUN2000	13:45
		Week 5	12JUN2000	Yes	12JUN2000	6:04
		Week 5 (Unsch. Fol. Up)	13JUN2000	No		
		Week 6 (Fol. Up)	19JUN2000	No		
		Week 7 (Fol. Up)	26JUN2000	No		
		Week 8 (Fol. Up)	03JUL2000	No		
		Week 9 (Fol. Up)	10JUL2000	No		

*Popn=Population (I=Intent-to-Treat, PP=Per Protocol)
Tafenoquine = Tafenoquine 200mg, Mefloquine = Mefloquine 250mg

SB252263 - 030

Listing B.02

Visit And Dosing Dates

Treatment : Mefloquine

Subject Number	Popn*	Visit	Visit Date	Dose Taken ?	Date of Dose	Time of Dose
030.001.00013	IPP	Screening	13APR2000	No		
		Screening (Unsch.)	18APR2000	No		
		Screening (Unsch.)	21APR2000	No		
		Screening (Unsch.)	28APR2000	No		
		Clearance (Day -5)	03MAY2000	Yes	03MAY2000	
		Clearance (Day -4)	04MAY2000	Yes	04MAY2000	
		Clearance (Day -3)	05MAY2000	Yes	05MAY2000	
		Clearance (Unsch.)	06MAY2000	No		
		Load Dose 1	08MAY2000	Yes	08MAY2000	7:15
		Load Dose 2	09MAY2000	Yes	09MAY2000	9:52
		Load Dose 3	10MAY2000	Yes	10MAY2000	9:28
		Load Dose (Unsch.)	09MAY2000	No		
		Week 1 (Unsch.)	12MAY2000	No		
		Week 1	16MAY2000	Yes	16MAY2000	9:18
		Week 2	22MAY2000	Yes	22MAY2000	6:38
		Week 3 (Fol. Up)	29MAY2000	No		
		Week 4 (Fol. Up)	06JUN2000	No		
		Week 5 (Fol. Up)	13JUN2000	No		
		Week 6 (Fol. Up)	19JUN2000	No		
030.001.00018	IPP	Screening	11APR2000	No		
		Screening (Unsch.)	26APR2000	No		
		Clearance (Day -5)	03MAY2000	Yes	03MAY2000	
		Clearance (Day -4)	04MAY2000	Yes	04MAY2000	
		Clearance (Day -3)	05MAY2000	Yes	05MAY2000	
		Load Dose 1	08MAY2000	Yes	08MAY2000	8:00
		Load Dose 2	09MAY2000	Yes	09MAY2000	7:44
		Load Dose 3	10MAY2000	Yes	10MAY2000	9:00
		Load Dose (Unsch.)	09MAY2000	No		
		Week 1	15MAY2000	Yes	15MAY2000	8:48
		Week 1 (Unsch.)	15MAY2000	No		
		Week 2	22MAY2000	Yes	22MAY2000	7:55
		Week 3	29MAY2000	Yes	29MAY2000	17:20
		Week 4	06JUN2000	Yes	06JUN2000	17:25
		Week 5 (Unsch.)	09JUN2000	No		
		Week 5	12JUN2000	Yes	12JUN2000	9:40
		Week 5 (Unsch.)	13JUN2000	No		
		Week 6	19JUN2000	Yes	19JUN2000	7:00
		Week 7	26JUN2000	Yes	26JUN2000	9:31
		Week 8	03JUL2000	Yes	03JUL2000	7:14
		Week 9	10JUL2000	Yes	10JUL2000	7:55
		Week 9 (Unsch. Fol. Up)	11JUL2000	No		
		Week 10 (Fol. Up)	18JUL2000	No		

*Popn=Population (I=Intent-to-Treat, PP=Per Protocol)
Tafenoquine = Tafenoquine 200mg, Mefloquine = Mefloquine 250mg

SB252263 - 030

Listing B.02

Visit And Dosing Dates

Treatment : Mefloquine

Subject Number	Popn*	Visit	Visit Date	Dose Taken ?	Date of Dose	Time of Dose
030.001.00018	IPP	Week 11 (Fol. Up)	25JUL2000	No		
		Week 12 (Fol. Up)	31JUL2000	No		
		Week 13 (Unsch. Fol. Up)	08AUG2000	No		
030.001.00021	IPP	Screening	10APR2000	No		
		Screening (Unsch.)	17APR2000	No		
		Screening (Unsch.)	02MAY2000	No		
		Clearance (Day -5)	03MAY2000	Yes	03MAY2000	
		Clearance (Day -4)	04MAY2000	Yes	04MAY2000	
		Clearance (Day -3)	05MAY2000	Yes	05MAY2000	
		Clearance (Unsch.)	06MAY2000	No		
		Load Dose 1	08MAY2000	Yes	08MAY2000	7:50
		Load Dose 2	09MAY2000	Yes	09MAY2000	6:59
		Load Dose 3	10MAY2000	Yes	10MAY2000	9:19
		Week 1 (Unsch.)	11MAY2000	No		
		Week 1	15MAY2000	Yes	15MAY2000	9:16
		Week 1 (Unsch.)	16MAY2000	No		
		Week 2	22MAY2000	Yes	22MAY2000	6:40
		Week 3	29MAY2000	Yes	29MAY2000	7:18
		Week 4	05JUN2000	Yes	05JUN2000	8:21
		Week 5	12JUN2000	Yes	12JUN2000	8:37
		Week 6	19JUN2000	Yes	19JUN2000	6:23
		Week 7	27JUN2000	Yes	27JUN2000	11:46
		Week 8	03JUL2000	Yes	03JUL2000	6:20
		Week 9	10JUL2000	Yes	10JUL2000	7:16
		Week 10	17JUL2000	Yes	17JUL2000	6:33
		Week 11 (Unsch.)	21JUL2000	No		
		Week 11	24JUL2000	Yes	24JUL2000	6:17
		Week 12	31JUL2000	Yes	31JUL2000	11:45
		Week 13	07AUG2000	Yes	07AUG2000	6:14
		Week 14	14AUG2000	Yes	14AUG2000	7:25
		Week 15	24AUG2000	Yes	24AUG2000	9:15
		Week 16	28AUG2000	Yes	28AUG2000	7:37
		Week 16	30AUG2000	No		
		Week 17	04SEP2000	Yes	04SEP2000	9:00
		Week 18	11SEP2000	Yes	11SEP2000	7:00
		Week 19	18SEP2000	Yes	18SEP2000	7:03
		Week 20	25SEP2000	Yes	25SEP2000	11:26
		Week 21	02OCT2000	Yes	02OCT2000	6:58
		Week 21 (Unsch.)	02OCT2000	No		
		Week 22	09OCT2000	Yes	09OCT2000	7:25
		Week 23	16OCT2000	Yes	16OCT2000	7:29
		Week 24	23OCT2000	Yes	23OCT2000	7:23

*Popn=Population (I=Intent-to-Treat, PP=Per Protocol)
Tafenoquine = Tafenoquine 200mg, Mefloquine = Mefloquine 250mg

SB252263 - 030

Listing B.02

Visit And Dosing Dates

Treatment : Mefloquine

Subject Number	Popn*	Visit	Visit Date	Dose Taken ?	Date of Dose	Time of Dose
030.001.00021	IPP	Week 25 (Fol. Up)	30OCT2000	No		
		Week 26 (Fol. Up)	06NOV2000	No		
		Week 26 (Unsch. Fol. Up)	06NOV2000	No		
		Week 26 (Unsch. Fol. Up)	08NOV2000	No		
		Week 27 (Fol. Up)	13NOV2000	No		
		Week 28 (Fol. Up)	20NOV2000	No		
030.001.00023	I	Screening	10APR2000	No		
		Screening (Unsch.)	17APR2000	No		
		Screening (Unsch.)	26APR2000	No		
		Screening (Unsch.)	02MAY2000	No		
		Clearance (Day -5)	03MAY2000	Yes	03MAY2000	
		Clearance (Day -4)	04MAY2000	Yes	04MAY2000	
		Clearance (Day -3)	05MAY2000	Yes	05MAY2000	
		Clearance (Unsch.)	03MAY2000	No		
		Clearance (Unsch.)	06MAY2000	No		
		Load Dose 1	08MAY2000	Yes	08MAY2000	9:00
		Load Dose 2	09MAY2000	Yes	09MAY2000	8:35
		Load Dose 3	10MAY2000	Yes	10MAY2000	9:50
		Week 1 (Unsch.)	11MAY2000	No		
		Week 1	15MAY2000	Yes	15MAY2000	8:37
		Week 2	22MAY2000	Yes	22MAY2000	8:49
		Week 3 (Fol. Up)	29MAY2000	No		
		Week 4 (Fol. Up)	05JUN2000	No		
		Week 5 (Fol. Up)	12JUN2000	No		
		Week 6 (Fol. Up)	19JUN2000	No		
		Week 6 (Unsch. Fol. Up)	19JUN2000	No		
		Week 6 (Unsch. Fol. Up)	20JUN2000	No		
		Week 6 (Fol. Up)	21JUN2000	No		
030.001.00031	IPP	Screening	12APR2000	No		
		Clearance (Day -5)	03MAY2000	Yes	03MAY2000	
		Clearance (Day -4)	04MAY2000	Yes	04MAY2000	
		Clearance (Day -3)	05MAY2000	Yes	05MAY2000	
		Load Dose 1	08MAY2000	Yes	08MAY2000	8:19
		Load Dose 2	09MAY2000	Yes	09MAY2000	7:15
		Load Dose 3	10MAY2000	Yes	10MAY2000	9:00
		Week 1 (Unsch.)	12MAY2000	No		
		Week 1	15MAY2000	Yes	15MAY2000	9:05
		Week 1 (Unsch.)	16MAY2000	No		
		Week 2	22MAY2000	Yes	22MAY2000	7:15
		Week 3	29MAY2000	Yes	29MAY2000	7:00

*Popn=Population (I=Intent-to-Treat, PP=Per Protocol)
Tafenoquine = Tafenoquine 200mg, Mefloquine = Mefloquine 250mg

SB252263 - 030

Listing B.02

Visit And Dosing Dates

Treatment : Mefloquine

Subject Number	Popn*	Visit	Visit Date	Dose Taken ?	Date of Dose	Time of Dose
030.001.00031	IPP	Week 3 (Unsch.)	30MAY2000	No		
		Week 4	05JUN2000	Yes	05JUN2000	8:10
		Week 5	12JUN2000	Yes	12JUN2000	19:10
		Week 6 (Unsch.)	16JUN2000	No		
		Week 6	19JUN2000	Yes	19JUN2000	7:30
		Week 7	26JUN2000	Yes	26JUN2000	7:02
		Week 8	03JUL2000	Yes	03JUL2000	6:30
		Week 9	10JUL2000	Yes	10JUL2000	7:30
		Week 10	17JUL2000	Yes	17JUL2000	7:02
		Week 11	24JUL2000	Yes	24JUL2000	7:20
		Week 12	31JUL2000	Yes	31JUL2000	7:05
		Week 13	09AUG2000	Yes	09AUG2000	6:49
		Week 14	14AUG2000	Yes	14AUG2000	9:45
		Week 15	21AUG2000	Yes	21AUG2000	7:49
		Week 16	28AUG2000	Yes	28AUG2000	
		Week 16	30AUG2000	No		
		Week 17	04SEP2000	Yes	04SEP2000	6:51
		Week 18	11SEP2000	Yes	11SEP2000	9:11
		Week 19	18SEP2000	Yes	18SEP2000	7:12
		Week 20	25SEP2000	Yes	25SEP2000	6:58
		Week 21	02OCT2000	Yes	02OCT2000	6:51
		Week 22 (Fol. Up)	09OCT2000	No		
		Week 23 (Fol. Up)	16OCT2000	No		
		Week 24 (Fol. Up)	23OCT2000	No		
		Week 24 (Unsch. Fol. Up)	23OCT2000	No		
		Week 25 (Fol. Up)	31OCT2000	No		
030.001.00034	IPP	Screening	17APR2000	No		
		Screening (Unsch.)	20APR2000	No		
		Screening (Unsch.)	26APR2000	No		
		Clearance (Day -5)	03MAY2000	Yes	03MAY2000	
		Clearance (Day -4)	04MAY2000	Yes	04MAY2000	
		Clearance (Day -3)	05MAY2000	Yes	05MAY2000	
		Load Dose 1	08MAY2000	Yes	08MAY2000	8:21
		Load Dose 2	09MAY2000	Yes	09MAY2000	7:56
		Load Dose 3	10MAY2000	Yes	10MAY2000	11:42
		Week 1 (Unsch.)	14MAY2000	No		
		Week 1	15MAY2000	Yes	15MAY2000	10:18
		Week 2	22MAY2000	Yes	22MAY2000	6:30
		Week 2 (Unsch.)	23MAY2000	No		
		Week 3	29MAY2000	Yes	29MAY2000	6:55
		Week 4	05JUN2000	Yes	05JUN2000	13:26
		Week 4 (Unsch.)	06JUN2000	No		

*Popn=Population (I=Intent-to-Treat, PP=Per Protocol)
Tafenoquine = Tafenoquine 200mg, Mefloquine = Mefloquine 250mg

SB252263 - 030

Listing B.02

Visit And Dosing Dates

Treatment : Mefloquine

Subject Number	Popn*	Visit	Visit Date	Dose Taken ?	Date of Dose	Time of Dose
030.001.00034	IPP	Week 5	12JUN2000	Yes	12JUN2000	9:37
		Week 6	19JUN2000	Yes	19JUN2000	10:01
		Week 7	26JUN2000	Yes	26JUN2000	16:17
		Week 7 (Unsch.)	27JUN2000	No		
		Week 8	03JUL2000	Yes	03JUL2000	7:13
		Week 9	10JUL2000	Yes	10JUL2000	6:46
		Week 10	18JUL2000	Yes	18JUL2000	7:44
		Week 11	24JUL2000	Yes	24JUL2000	7:18
		Week 12	31JUL2000	Yes	31JUL2000	9:25
		Week 13	07AUG2000	Yes	07AUG2000	6:30
		Week 13 (Unsch.)	07AUG2000	No		
		Week 14 (Fol. Up)	14AUG2000	No		
		Week 15 (Fol. Up)	21AUG2000	No		
		Week 16 (Fol. Up)	28AUG2000	No		
030.001.00035		Screening	12APR2000	No		
		Screening (Unsch.)	02MAY2000	No		
		Clearance (Day -5)	03MAY2000	Yes	03MAY2000	
		Clearance (Day -4)	04MAY2000	Yes	04MAY2000	
		Clearance (Day -3)	05MAY2000	Yes	05MAY2000	
		Clearance (Unsch.)	04MAY2000	No		
		Load Dose 1	08MAY2000	Yes	08MAY2000	8:24
		Load Dose 2	09MAY2000	Yes	09MAY2000	6:35
		Load Dose 3	10MAY2000	Yes	10MAY2000	8:40
		Load Dose (Unsch.)	10MAY2000	No		
		Week 1 (Fol. Up)	18MAY2000	No		
		Week 2 (Fol. Up)	22MAY2000	No		
		Week 2 (Unsch. Fol. Up)	23MAY2000	No		
		Week 3 (Fol. Up)	29MAY2000	No		
		Week 3 (Unsch. Fol. Up)	01JUN2000	No		
		Week 4 (Fol. Up)	07JUN2000	No		
030.001.00036	I	Screening	13APR2000	No		
		Screening (Unsch.)	02MAY2000	No		
		Clearance (Day -5)	03MAY2000	Yes	03MAY2000	
		Clearance (Day -4)	04MAY2000	Yes	04MAY2000	
		Clearance (Day -3)	05MAY2000	Yes	05MAY2000	
		Load Dose 1	08MAY2000	Yes	08MAY2000	8:31
		Load Dose 2	09MAY2000	Yes	09MAY2000	6:15
		Load Dose 3	10MAY2000	Yes	10MAY2000	7:13
		Week 1	15MAY2000	Yes	15MAY2000	7:16
		Week 2	22MAY2000	Yes	22MAY2000	8:00

*Popn=Population (I=Intent-to-Treat, PP=Per Protocol)
Tafenoquine = Tafenoquine 200mg, Mefloquine = Mefloquine 250mg

SB252263 - 030

Listing B.02

Visit And Dosing Dates

Treatment : Mefloquine

Subject Number	Popn*	Visit	Visit Date	Dose Taken ?	Date of Dose	Time of Dose
030.001.00036	I	Week 3 (Unsch.)	28MAY2000	No		
		Week 3	29MAY2000	Yes	29MAY2000	8:00
		Week 4	05JUN2000	Yes	05JUN2000	15:35
		Week 5 (Unsch.)	10JUN2000	No		
		Week 5	12JUN2000	Yes	12JUN2000	11:05
		Week 6	19JUN2000	Yes	19JUN2000	12:52
		Week 6 (Unsch.)	22JUN2000	No		
		Week 7	28JUN2000	Yes	28JUN2000	8:08
		Week 8 (Unsch.)	30JUN2000	No		
		Week 8	03JUL2000	Yes	03JUL2000	7:15
		Week 9	10JUL2000	Yes	10JUL2000	8:15
		Week 9 (Unsch.)	10JUL2000	No		
		Week 10	17JUL2000	Yes	17JUL2000	7:30
		Week 11 (Fol. Up)	24JUL2000	No		
		Week 12 (Fol. Up)	31JUL2000	No		
		Week 14 (Fol. Up)	11AUG2000	No		
030.001.00038	IPP	Screening	18APR2000	No		
		Clearance (Day -5)	03MAY2000	Yes	03MAY2000	
		Clearance (Day -4)	04MAY2000	Yes	04MAY2000	
		Clearance (Day -3)	05MAY2000	Yes	05MAY2000	
		Clearance (Unsch.)	06MAY2000	No		
		Load Dose 1	08MAY2000	Yes	08MAY2000	8:41
		Load Dose 2	09MAY2000	Yes	09MAY2000	7:36
		Load Dose 3	10MAY2000	Yes	10MAY2000	11:04
		Week 1	15MAY2000	Yes	15MAY2000	10:07
		Week 2	22MAY2000	Yes	22MAY2000	6:48
		Week 3	29MAY2000	Yes	29MAY2000	6:40
		Week 4	04JUN2000	Yes	04JUN2000	8:06
		Week 5	12JUN2000	Yes	12JUN2000	7:10
		Week 6	19JUN2000	Yes	19JUN2000	6:22
		Week 7 (Fol. Up)	26JUN2000	No		
		Week 8 (Fol. Up)	03JUL2000	No		
		Week 9 (Fol. Up)	10JUL2000	No		
030.001.00045	IPP	Screening	14APR2000	No		
		Clearance (Day -5)	03MAY2000	Yes	03MAY2000	
		Clearance (Day -4)	04MAY2000	Yes	04MAY2000	
		Clearance (Day -3)	05MAY2000	Yes	05MAY2000	
		Load Dose 1	08MAY2000	Yes	08MAY2000	8:47
		Load Dose 2	09MAY2000	Yes	09MAY2000	7:00
		Load Dose 3	10MAY2000	Yes	10MAY2000	7:45

*Popn=Population (I=Intent-to-Treat, PP=Per Protocol)
Tafenoquine = Tafenoquine 200mg, Mefloquine = Mefloquine 250mg

SB252263 - 030

Listing B.02

Visit And Dosing Dates

Treatment : Mefloquine

Subject Number	Popn*	Visit	Visit Date	Dose Taken ?	Date of Dose	Time of Dose
030.001.00045	IPP	Load Dose (Unsch.)	10MAY2000	No		
		Week 1	16MAY2000	Yes	16MAY2000	8:30
		Week 1 (Unsch.)	17MAY2000	No		
		Week 1 (Unsch.)	18MAY2000	No		
		Week 2 (Unsch.)	21MAY2000	No		
		Week 2	22MAY2000	Yes	22MAY2000	7:00
		Week 3 (Fol. Up)	29MAY2000	No		
		Week 3 (Unsch. Fol. Up)	30MAY2000	No		
		Week 4 (Fol. Up)	05JUN2000	No		
		Week 5 (Fol. Up)	12JUN2000	No		
		Week 6 (Fol. Up)	19JUN2000	No		
		Week 6 (Unsch. Fol. Up)	19JUN2000	No		
		Week 6 (Unsch. Fol. Up)	21JUN2000	No		
030.001.00046	IPP	Screening	10APR2000	No		
		Clearance (Day -5)	03MAY2000	Yes	03MAY2000	
		Clearance (Day -4)	04MAY2000	Yes	04MAY2000	
		Clearance (Day -3)	05MAY2000	Yes	05MAY2000	
		Clearance (Unsch.)	03MAY2000	No		
		Load Dose 1	08MAY2000	Yes	08MAY2000	8:42
		Load Dose 2	09MAY2000	Yes	09MAY2000	6:59
		Load Dose 3	10MAY2000	Yes	10MAY2000	11:06
		Week 1	15MAY2000	Yes	15MAY2000	8:30
		Week 2 (Unsch.)	19MAY2000	No		
		Week 2	22MAY2000	Yes	22MAY2000	7:45
		Week 3	29MAY2000	Yes	29MAY2000	7:25
		Week 3 (Unsch.)	29MAY2000	No		
		Week 4	05JUN2000	Yes	05JUN2000	9:04
		Week 5	12JUN2000	Yes	12JUN2000	8:05
		Week 6	19JUN2000	Yes	19JUN2000	12:45
		Week 7	26JUN2000	Yes	26JUN2000	8:10
		Week 8 (Fol. Up)	03JUL2000	No		
		Week 9 (Fol. Up)	10JUL2000	No		
		Week 9 (Unsch. Fol. Up)	12JUL2000	No		
		Week 10 (Fol. Up)	17JUL2000	No		
		Week 10 (Unsch. Fol. Up)	18JUL2000	No		
030.001.00047	IPP	Screening	12APR2000	No		
		Screening (Unsch.)	17APR2000	No		
		Screening (Unsch.)	20APR2000	No		
		Clearance (Day -5)	03MAY2000	Yes	03MAY2000	
		Clearance (Day -4)	04MAY2000	Yes	04MAY2000	

*Popn=Population (I=Intent-to-Treat, PP=Per Protocol)
Tafenoquine = Tafenoquine 200mg, Mefloquine = Mefloquine 250mg

SB252263 - 030

Listing B.02

Visit And Dosing Dates

Treatment : Mefloquine

Subject Number	Popn*	Visit	Visit Date	Dose Taken ?	Date of Dose	Time of Dose
030.001.00047	IPP	Clearance (Day -3)	05MAY2000	Yes	05MAY2000	
		Clearance (Unsch.)	04MAY2000	No		
		Load Dose 1	08MAY2000	Yes	08MAY2000	8:59
		Load Dose 2	09MAY2000	Yes	09MAY2000	6:58
		Load Dose 3	10MAY2000	Yes	10MAY2000	8:20
		Week 1	15MAY2000	Yes	15MAY2000	9:00
		Week 1 (Unsch.)	17MAY2000	No		
		Week 2	22MAY2000	Yes	22MAY2000	7:59
		Week 3	29MAY2000	Yes	29MAY2000	6:10
		Week 3 (Unsch.)	01JUN2000	No		
		Week 4	07JUN2000	Yes	07JUN2000	11:29
		Week 5 (Unsch.)	09JUN2000	No		
		Week 5	12JUN2000	Yes	12JUN2000	13:15
		Week 6	19JUN2000	Yes	19JUN2000	8:30
		Week 7 (Fol. Up)	26JUN2000	No		
		Week 8 (Fol. Up)	03JUL2000	No		
		Week 9 (Fol. Up)	10JUL2000	No		
		Week 9 (Fol. Up)	11JUL2000	No		
		Week 10 (Unsch. Fol. Up)	18JUL2000	No		
		Week 10 (Unsch. Fol. Up)	19JUL2000	No		
030.001.00049	IPP	Screening	13APR2000	No		
		Clearance (Day -5)	03MAY2000	Yes	03MAY2000	
		Clearance (Day -4)	04MAY2000	Yes	04MAY2000	
		Clearance (Day -3)	05MAY2000	Yes	05MAY2000	
		Load Dose 1	08MAY2000	Yes	08MAY2000	8:55
		Load Dose 2	09MAY2000	Yes	09MAY2000	7:50
		Load Dose 3	10MAY2000	Yes	10MAY2000	10:20
		Week 1	15MAY2000	Yes	15MAY2000	7:50
		Week 2	22MAY2000	Yes	22MAY2000	8:50
		Week 3	29MAY2000	Yes	29MAY2000	7:30
		Week 4	05JUN2000	Yes	05JUN2000	10:58
		Week 5	12JUN2000	Yes	12JUN2000	7:15
		Week 6	19JUN2000	Yes	19JUN2000	9:20
		Week 7	26JUN2000	Yes	26JUN2000	6:02
		Week 8	03JUL2000	Yes	03JUL2000	7:50
		Week 8	04JUL2000	No		
		Week 9	10JUL2000	Yes	10JUL2000	10:10
		Week 10	17JUL2000	Yes	17JUL2000	6:50
		Week 11 (Fol. Up)	24JUL2000	No		
		Week 12 (Fol. Up)	31JUL2000	No		
		Week 13 (Fol. Up)	07AUG2000	No		

*Popn=Population (I=Intent-to-Treat, PP=Per Protocol)
Tafenoquine = Tafenoquine 200mg, Mefloquine = Mefloquine 250mg

SB252263 - 030

Listing B.02

Visit And Dosing Dates

Treatment : Mefloquine

Subject Number	Popn*	Visit	Visit Date	Dose Taken ?	Date of Dose	Time of Dose
030.001.00052	IPP	Screening	10APR2000	No		
		Screening (Unsch.)	26APR2000	No		
		Clearance (Day -5)	03MAY2000	Yes	03MAY2000	
		Clearance (Day -4)	04MAY2000	Yes	04MAY2000	
		Clearance (Day -3)	05MAY2000	Yes	05MAY2000	
		Clearance (Unsch.)	05MAY2000	No		
		Load Dose 1	08MAY2000	Yes	08MAY2000	9:11
		Load Dose 2	09MAY2000	Yes	09MAY2000	9:41
		Load Dose 3	10MAY2000	Yes	10MAY2000	9:40
		Week 1	15MAY2000	Yes	15MAY2000	1:40
		Week 1 (Unsch.)	16MAY2000	No		
		Week 2	22MAY2000	Yes	22MAY2000	9:18
		Week 3 (Unsch.)	28MAY2000	No		
		Week 3	29MAY2000	Yes	29MAY2000	8:30
		Week 4	05JUN2000	Yes	05JUN2000	11:59
		Week 5	12JUN2000	Yes	12JUN2000	6:45
		Week 5 (Unsch. Fol. Up)	13JUN2000	No		
		Week 6 (Fol. Up)	19JUN2000	No		
		Week 6 (Unsch. Fol. Up)	19JUN2000	No		
		Week 7 (Fol. Up)	26JUN2000	No		
		Week 8 (Fol. Up)	03JUL2000	No		
		Week 8 (Fol. Up)	04JUL2000	No		
030.001.00053	IPP	Screening	11APR2000	No		
		Screening (Unsch.)	19APR2000	No		
		Clearance (Day -5)	03MAY2000	Yes	03MAY2000	
		Clearance (Day -4)	04MAY2000	Yes	04MAY2000	
		Clearance (Day -3)	05MAY2000	Yes	05MAY2000	
		Load Dose 1	08MAY2000	Yes	08MAY2000	9:00
		Load Dose 2	09MAY2000	Yes	09MAY2000	6:59
		Load Dose 3	10MAY2000	Yes	10MAY2000	7:55
		Week 1	15MAY2000	Yes	15MAY2000	8:35
		Week 1 (Unsch.)	16MAY2000	No		
		Week 2	22MAY2000	Yes	22MAY2000	6:30
		Week 2 (Unsch.)	23MAY2000	No		
		Week 3	29MAY2000	Yes	29MAY2000	7:45
		Week 3 (Unsch.)	31MAY2000	No		
		Week 4	05JUN2000	Yes	05JUN2000	10:45
		Week 5	12JUN2000	Yes	12JUN2000	6:30
		Week 5 (Unsch.)	12JUN2000	No		
		Week 6	19JUN2000	Yes	19JUN2000	7:10
		Week 7	26JUN2000	Yes	26JUN2000	8:02
		Week 8	03JUL2000	Yes	03JUL2000	7:00

*Popn=Population (I=Intent-to-Treat, PP=Per Protocol)
Tafenoquine = Tafenoquine 200mg, Mefloquine = Mefloquine 250mg

SB252263 - 030

Listing B.02

Visit And Dosing Dates

Treatment : Mefloquine

Subject Number	Popn*	Visit	Visit Date	Dose Taken ?	Date of Dose	Time of Dose
030.001.00053	IPP	Week 8 (Unsch.)	05JUL2000	No		
		Week 9	10JUL2000	Yes	10JUL2000	7:02
		Week 10	17JUL2000	Yes	17JUL2000	10:05
		Week 10 (Unsch.)	19JUL2000	No		
		Week 11	24JUL2000	Yes	24JUL2000	8:10
		Week 12	31JUL2000	Yes	31JUL2000	7:30
		Week 13 (Fol. Up)	10AUG2000	No		
		Week 14 (Fol. Up)	17AUG2000	No		
		Week 15 (Unsch. Fol. Up)	18AUG2000	No		
		Week 16 (Fol. Up)	25AUG2000	No		
030.001.00054	IPP	Screening	10APR2000	No		
		Clearance (Day -5)	03MAY2000	Yes	03MAY2000	
		Clearance (Day -4)	04MAY2000	Yes	04MAY2000	
		Clearance (Day -3)	05MAY2000	Yes	05MAY2000	
		Load Dose 1	08MAY2000	Yes	08MAY2000	9:10
		Load Dose 2	09MAY2000	Yes	09MAY2000	7:20
		Load Dose 3	10MAY2000	Yes	10MAY2000	8:30
		Week 1	15MAY2000	Yes	15MAY2000	9:10
		Week 2	22MAY2000	Yes	22MAY2000	8:00
		Week 3	29MAY2000	Yes	29MAY2000	8:10
		Week 4	05JUN2000	Yes	05JUN2000	9:20
		Week 5	12JUN2000	Yes	12JUN2000	6:20
		Week 6	19JUN2000	Yes	19JUN2000	7:40
		Week 7	26JUN2000	Yes	26JUN2000	6:50
		Week 8 (Fol. Up)	03JUL2000	No		
		Week 9 (Fol. Up)	10JUL2000	No		
		Week 10 (Fol. Up)	17JUL2000	No		
		Week 11 (Fol. Up)	24JUL2000	No		
030.001.00062	IPP	Screening	12APR2000	No		
		Screening (Unsch.)	14APR2000	No		
		Screening (Unsch.)	21APR2000	No		
		Screening (Unsch.)	25APR2000	No		
		Clearance (Day -5)	03MAY2000	Yes	03MAY2000	
		Clearance (Day -4)	04MAY2000	Yes	04MAY2000	
		Clearance (Day -3)	05MAY2000	Yes	05MAY2000	
		Clearance (Unsch.)	05MAY2000	No		
		Load Dose 1	08MAY2000	Yes	08MAY2000	9:30
		Load Dose 2	09MAY2000	Yes	09MAY2000	14:30
		Load Dose 3	10MAY2000	Yes	10MAY2000	16:27
		Week 1 (Unsch.)	11MAY2000	No		

*Popn=Population (I=Intent-to-Treat, PP=Per Protocol)
Tafenoquine = Tafenoquine 200mg, Mefloquine = Mefloquine 250mg

SB252263 - 030

Listing B.02

Visit And Dosing Dates

Treatment : Mefloquine

Subject Number	Popn*	Visit	Visit Date	Dose Taken ?	Date of Dose	Time of Dose
030.001.00062	IPP	Week 1	15MAY2000	Yes	15MAY2000	11:40
		Week 1 (Unsch.)	17MAY2000	No		
		Week 2	22MAY2000	Yes	22MAY2000	9:16
		Week 3	29MAY2000	Yes	29MAY2000	7:37
		Week 4	05JUN2000	Yes	05JUN2000	8:26
		Week 5	12JUN2000	Yes	12JUN2000	7:46
		Week 6	19JUN2000	Yes	19JUN2000	7:25
		Week 6 (Unsch. Fol. Up)	22JUN2000	No		
		Week 7 (Fol. Up)	26JUN2000	No		
		Week 7 (Unsch. Fol. Up)	29JUN2000	No		
		Week 8 (Fol. Up)	03JUL2000	No		
		Week 9 (Fol. Up)	11JUL2000	No		
030.001.00066	IPP	Screening	14APR2000	No		
		Screening (Unsch.)	25APR2000	No		
		Clearance (Day -5)	03MAY2000	Yes	03MAY2000	
		Clearance (Day -4)	04MAY2000	Yes	04MAY2000	
		Clearance (Day -3)	05MAY2000	Yes	05MAY2000	
		Clearance (Unsch.)	06MAY2000	No		
		Load Dose 1	08MAY2000	Yes	08MAY2000	9:59
		Load Dose 2	09MAY2000	Yes	09MAY2000	7:40
		Load Dose 3	10MAY2000	Yes	10MAY2000	9:22
		Week 1	15MAY2000	Yes	15MAY2000	9:10
		Week 2 (Unsch.)	19MAY2000	No		
		Week 2	22MAY2000	Yes	22MAY2000	9:43
		Week 3 (Unsch.)	26MAY2000	No		
		Week 3	29MAY2000	Yes	29MAY2000	9:40
		Week 4	06JUN2000	Yes	06JUN2000	9:37
		Week 5 (Fol. Up)	12JUN2000	No		
		Week 5 (Unsch. Fol. Up)	13JUN2000	No		
		Week 6 (Fol. Up)	19JUN2000	No		
		Week 6 (Unsch. Fol. Up)	21JUN2000	No		
		Week 7 (Fol. Up)	26JUN2000	No		
		Week 8 (Fol. Up)	03JUL2000	No		
030.001.00069	I	Screening	10APR2000	No		
		Screening (Unsch.)	14APR2000	No		
		Screening (Unsch.)	02MAY2000	No		
		Clearance (Day -5)	03MAY2000	Yes	03MAY2000	
		Clearance (Day -4)	04MAY2000	Yes	04MAY2000	
		Clearance (Day -3)	05MAY2000	Yes	05MAY2000	
		Load Dose 1	08MAY2000	Yes	08MAY2000	9:50

*Popn=Population (I=Intent-to-Treat, PP=Per Protocol)
Tafenoquine = Tafenoquine 200mg, Mefloquine = Mefloquine 250mg

SB252263 - 030

Listing B.02

Visit And Dosing Dates

Treatment : Mefloquine

Subject Number	Popn*	Visit	Visit Date	Dose Taken ?	Date of Dose	Time of Dose
030.001.00069	I	Load Dose 2	09MAY2000	Yes	09MAY2000	7:31
		Load Dose 3	10MAY2000	Yes	10MAY2000	9:10
		Week 1	15MAY2000	Yes	15MAY2000	8:19
		Week 2	22MAY2000	Yes	22MAY2000	9:12
		Week 2 (Unsch.)	24MAY2000	No		
		Week 3	29MAY2000	Yes	29MAY2000	6:22
		Week 4	05JUN2000	Yes	05JUN2000	10:10
		Week 5	12JUN2000	Yes	12JUN2000	9:53
		Week 5 (Unsch.)	13JUN2000	No		
		Week 6	19JUN2000	Yes	19JUN2000	11:55
		Week 7	26JUN2000	Yes	26JUN2000	15:59
		Week 8	03JUL2000	Yes	03JUL2000	8:17
		Week 9	10JUL2000	Yes	10JUL2000	9:58
		Week 10	17JUL2000	Yes	17JUL2000	10:08
		Week 11 (Fol. Up)	25JUL2000	No		
		Week 11 (Unsch. Fol. Up)	27JUL2000	No		
		Week 12 (Fol. Up)	01AUG2000	No		
		Week 13 (Fol. Up)	07AUG2000	No		
030.001.00071	IPP	Screening	10APR2000	No		
		Screening (Unsch.)	27APR2000	No		
		Clearance (Day -5)	03MAY2000	Yes	03MAY2000	
		Clearance (Day -4)	04MAY2000	Yes	04MAY2000	
		Clearance (Day -3)	05MAY2000	Yes	05MAY2000	
		Clearance (Unsch.)	03MAY2000	No		
		Load Dose 1	08MAY2000	Yes	08MAY2000	10:16
		Load Dose 2	09MAY2000	Yes	09MAY2000	7:20
		Load Dose 3	10MAY2000	Yes	10MAY2000	10:25
		Week 1	15MAY2000	Yes	15MAY2000	10:15
		Week 2	22MAY2000	Yes	22MAY2000	8:20
		Week 3	29MAY2000	Yes	29MAY2000	9:30
		Week 4 (Unsch.)	02JUN2000	No		
		Week 4	06JUN2000	Yes	06JUN2000	11:25
		Week 4 (Unsch.)	06JUN2000	No		
		Week 5	12JUN2000	Yes	12JUN2000	9:55
		Week 6	19JUN2000	Yes	19JUN2000	16:59
		Week 7	26JUN2000	Yes	26JUN2000	12:45
		Week 8	03JUL2000	Yes	03JUL2000	10:31
		Week 9	10JUL2000	Yes	10JUL2000	6:51
		Week 10	17JUL2000	Yes	17JUL2000	10:48
		Week 11 (Fol. Up)	26JUL2000	No		
		Week 11 (Unsch. Fol. Up)	27JUL2000	No		
		Week 12 (Fol. Up)	31JUL2000	No		

*Popn=Population (I=Intent-to-Treat, PP=Per Protocol)
Tafenoquine = Tafenoquine 200mg, Mefloquine = Mefloquine 250mg

SB252263 - 030

Listing B.02

Visit And Dosing Dates

Treatment : Mefloquine

Subject Number	Popn*	Visit	Visit Date	Dose Taken ?	Date of Dose	Time of Dose
030.001.00071	IPP	Week 12 (Unsch. Fol. Up)	31JUL2000	No		
		Week 13 (Fol. Up)	07AUG2000	No		
		Week 14 (Fol. Up)	15AUG2000	No		
030.001.00073	IPP	Screening	13APR2000	No		
		Screening (Unsch.)	21APR2000	No		
		Screening (Unsch.)	29APR2000	No		
		Clearance (Day -5)	03MAY2000	Yes	03MAY2000	
		Clearance (Day -4)	04MAY2000	Yes	04MAY2000	
		Clearance (Day -3)	05MAY2000	Yes	05MAY2000	
		Load Dose 1	08MAY2000	Yes	08MAY2000	10:09
		Load Dose 2	09MAY2000	Yes	09MAY2000	6:30
		Load Dose 3	10MAY2000	Yes	10MAY2000	9:30
		Week 1 (Unsch.)	12MAY2000	No		
		Week 1	15MAY2000	Yes	15MAY2000	6:55
		Week 1 (Unsch.)	18MAY2000	No		
		Week 2	22MAY2000	Yes	22MAY2000	6:20
		Week 3	29MAY2000	Yes	29MAY2000	6:23
		Week 4	07JUN2000	Yes	07JUN2000	9:28
		Week 5	12JUN2000	Yes	12JUN2000	6:30
		Week 6	19JUN2000	Yes	19JUN2000	6:35
		Week 7	26JUN2000	Yes	26JUN2000	6:46
		Week 8 (Fol. Up)	04JUL2000	No		
		Week 9 (Fol. Up)	09JUL2000	No		
		Week 9 (Fol. Up)	10JUL2000	No		
		Week 10 (Fol. Up)	17JUL2000	No		
		Week 10 (Unsch. Fol. Up)	20JUL2000	No		
030.001.00074	IPP	Screening	10APR2000	No		
		Clearance (Day -5)	03MAY2000	Yes	03MAY2000	
		Clearance (Day -4)	04MAY2000	Yes	04MAY2000	
		Clearance (Day -3)	05MAY2000	Yes	05MAY2000	
		Load Dose 1	08MAY2000	Yes	08MAY2000	10:06
		Load Dose 2	09MAY2000	Yes	09MAY2000	6:50
		Load Dose 3	10MAY2000	Yes	10MAY2000	7:44
		Week 1	15MAY2000	Yes	15MAY2000	8:57
		Week 2	21MAY2000	Yes	21MAY2000	12:10
		Week 3 (Unsch.)	28MAY2000	No		
		Week 3	29MAY2000	Yes	29MAY2000	7:13
		Week 4	05JUN2000	Yes	05JUN2000	7:51
		Week 5	13JUN2000	Yes	13JUN2000	9:59
		Week 6	19JUN2000	Yes	19JUN2000	8:00

*Popn=Population (I=Intent-to-Treat, PP=Per Protocol)
Tafenoquine = Tafenoquine 200mg, Mefloquine = Mefloquine 250mg

SB252263 - 030

Listing B.02

Visit And Dosing Dates

Treatment : Mefloquine

Subject Number	Popn*	Visit	Visit Date	Dose Taken ?	Date of Dose	Time of Dose
030.001.00074	IPP	Week 7	26JUN2000	Yes	26JUN2000	7:15
		Week 7 (Unsch.)	29JUN2000	No		
		Week 8	03JUL2000	Yes	03JUL2000	6:40
		Week 9	10JUL2000	Yes	10JUL2000	6:50
		Week 10	17JUL2000	Yes	17JUL2000	7:15
		Week 11	24JUL2000	Yes	24JUL2000	7:00
		Week 12 (Fol. Up)	31JUL2000	No		
		Week 13 (Fol. Up)	07AUG2000	No		
		Week 14 (Fol. Up)	14AUG2000	No		
030.001.00075	IPP	Screening	10APR2000	No		
		Clearance (Day -5)	03MAY2000	Yes	03MAY2000	
		Clearance (Day -4)	04MAY2000	Yes	04MAY2000	
		Clearance (Day -3)	05MAY2000	Yes	05MAY2000	
		Clearance (Unsch.)	06MAY2000	No		
		Load Dose 1	08MAY2000	Yes	08MAY2000	10:41
		Load Dose 2	09MAY2000	Yes	09MAY2000	8:07
		Load Dose 3	10MAY2000	Yes	10MAY2000	9:00
		Week 1	15MAY2000	Yes	15MAY2000	7:40
		Week 2	22MAY2000	Yes	22MAY2000	8:15
		Week 3	29MAY2000	Yes	29MAY2000	7:30
		Week 4	06JUN2000	Yes	06JUN2000	12:15
		Week 5	12JUN2000	Yes	12JUN2000	7:13
		Week 6	19JUN2000	Yes	19JUN2000	6:58
		Week 7	26JUN2000	Yes	26JUN2000	6:20
		Week 8	03JUL2000	Yes	03JUL2000	7:26
		Week 9	10JUL2000	Yes	10JUL2000	6:40
		Week 10 (Fol. Up)	17JUL2000	No		
		Week 10 (Unsch. Fol. Up)	18JUL2000	No		
		Week 11 (Fol. Up)	24JUL2000	No		
		Week 12 (Fol. Up)	31JUL2000	No		
030.001.00083	IPP	Screening	13APR2000	No		
		Clearance (Day -5)	03MAY2000	Yes	03MAY2000	
		Clearance (Day -4)	04MAY2000	Yes	04MAY2000	
		Clearance (Day -3)	05MAY2000	Yes	05MAY2000	
		Load Dose 1	08MAY2000	Yes	08MAY2000	10:52
		Load Dose 2	09MAY2000	Yes	09MAY2000	6:55
		Load Dose 3	10MAY2000	Yes	10MAY2000	7:20
		Week 1	15MAY2000	Yes	15MAY2000	12:30
		Week 1 (Unsch.)	17MAY2000	No		
		Week 2	22MAY2000	Yes	22MAY2000	6:45

*Popn=Population (I=Intent-to-Treat, PP=Per Protocol)
Tafenoquine = Tafenoquine 200mg, Mefloquine = Mefloquine 250mg

SB252263 - 030

Listing B.02

Visit And Dosing Dates

Treatment : Mefloquine

Subject Number	Popn*	Visit	Visit Date	Dose Taken ?	Date of Dose	Time of Dose
030.001.00083	IPP	Week 2 (Unsch.)	25MAY2000	No		
		Week 3	29MAY2000	Yes	29MAY2000	6:45
		Week 4 (Unsch.)	03JUN2000	No		
		Week 4	05JUN2000	Yes	05JUN2000	10:00
		Week 5 (Unsch.)	10JUN2000	No		
		Week 5	12JUN2000	Yes	12JUN2000	6:05
		Week 6	19JUN2000	Yes	19JUN2000	7:30
		Week 6 (Unsch.)	19JUN2000	No		
		Week 7	26JUN2000	Yes	26JUN2000	7:50
		Week 8	03JUL2000	Yes	03JUL2000	6:59
		Week 9	10JUL2000	Yes	10JUL2000	7:30
		Week 10 (Fol. Up)	17JUL2000	No		
		Week 10 (Unsch. Fol. Up)	17JUL2000	No		
		Week 11 (Unsch. Fol. Up)	21JUL2000	No		
		Week 11 (Fol. Up)	24JUL2000	No		
		Week 12 (Fol. Up)	31JUL2000	No		
		Week 12 (Unsch. Fol. Up)	31JUL2000	No		
		Week 12 (Unsch. Fol. Up)	01AUG2000	No		
		Week 13 (Unsch. Fol. Up)	08AUG2000	No		
030.001.00089	IPP	Screening	17APR2000	No		
		Clearance (Day -5)	03MAY2000	Yes	03MAY2000	
		Clearance (Day -4)	04MAY2000	Yes	04MAY2000	
		Clearance (Day -3)	05MAY2000	Yes	05MAY2000	
		Load Dose 1	08MAY2000	Yes	08MAY2000	11:55
		Load Dose 2	09MAY2000	Yes	09MAY2000	9:10
		Load Dose 3	10MAY2000	Yes	10MAY2000	14:07
		Week 1	15MAY2000	Yes	15MAY2000	12:00
		Week 2	22MAY2000	Yes	22MAY2000	10:16
		Week 3 (Unsch. Fol. Up)	26MAY2000	No		
		Week 3 (Fol. Up)	29MAY2000	No		
		Week 4 (Fol. Up)	05JUN2000	No		
		Week 5 (Fol. Up)	13JUN2000	No		
		Week 6 (Fol. Up)	19JUN2000	No		
030.001.00091	IPP	Screening	13APR2000	No		
		Screening (Unsch.)	26APR2000	No		
		Clearance (Day -5)	03MAY2000	Yes	03MAY2000	
		Clearance (Day -4)	04MAY2000	Yes	04MAY2000	
		Clearance (Day -3)	05MAY2000	Yes	05MAY2000	
		Load Dose 1	08MAY2000	Yes	08MAY2000	12:00
		Load Dose 2	09MAY2000	Yes	09MAY2000	7:58

*Popn=Population (I=Intent-to-Treat, PP=Per Protocol)
Tafenoquine = Tafenoquine 200mg, Mefloquine = Mefloquine 250mg

SB252263 - 030

Listing B.02

Visit And Dosing Dates

Treatment : Mefloquine

Subject Number	Popn*	Visit	Visit Date	Dose Taken ?	Date of Dose	Time of Dose
030.001.00091	IPP	Load Dose 3	10MAY2000	Yes	10MAY2000	8:30
		Load Dose (Unsch.)	09MAY2000	No		
		Week 1 (Unsch.)	14MAY2000	No		
		Week 1	15MAY2000	Yes	15MAY2000	9:47
		Week 2	22MAY2000	Yes	22MAY2000	10:11
		Week 2 (Unsch.)	22MAY2000	No		
		Week 3	29MAY2000	Yes	29MAY2000	9:45
		Week 4 (Unsch.)	02JUN2000	No		
		Week 4	05JUN2000	Yes	05JUN2000	9:24
		Week 5	12JUN2000	Yes	12JUN2000	10:03
		Week 5 (Unsch.)	13JUN2000	No		
		Week 6	19JUN2000	Yes	19JUN2000	8:25
		Week 7	26JUN2000	Yes	26JUN2000	9:58
		Week 8	03JUL2000	Yes	03JUL2000	10:43
		Week 9	10JUL2000	Yes	10JUL2000	8:45
		Week 9 (Unsch.)	13JUL2000	No		
		Week 10	17JUL2000	Yes	17JUL2000	9:20
		Week 10 (Unsch.)	19JUL2000	No		
		Week 11	24JUL2000	Yes	24JUL2000	8:45
		Week 12	31JUL2000	Yes	31JUL2000	8:45
		Week 13	07AUG2000	Yes	07AUG2000	9:25
		Week 14	14AUG2000	Yes	14AUG2000	8:40
		Week 14 (Unsch. Fol. Up)	15AUG2000	No		
		Week 14 (Unsch. Fol. Up)	17AUG2000	No		
		Week 15 (Unsch. Fol. Up)	20AUG2000	No		
		Week 16 (Fol. Up)	26AUG2000	No		
		Week 16 (Fol. Up)	31AUG2000	No		
		Week 17 (Fol. Up)	04SEP2000	No		
030.001.00095	IPP	Screening	14APR2000	No		
		Screening (Unsch.)	20APR2000	No		
		Clearance (Day -5)	03MAY2000	Yes	03MAY2000	
		Clearance (Day -4)	04MAY2000	Yes	04MAY2000	
		Clearance (Day -3)	05MAY2000	Yes	05MAY2000	
		Clearance (Unsch.)	03MAY2000	No		
		Load Dose 1	08MAY2000	Yes	08MAY2000	12:40
		Load Dose 2	09MAY2000	Yes	09MAY2000	6:44
		Load Dose 3	10MAY2000	Yes	10MAY2000	8:11
		Week 1	17MAY2000	Yes	17MAY2000	15:25
		Week 1 (Unsch.)	17MAY2000	No		
		Week 2	22MAY2000	Yes	22MAY2000	8:50
		Week 3 (Unsch.)	26MAY2000	No		
		Week 3	29MAY2000	Yes	29MAY2000	6:30

*Popn=Population (I=Intent-to-Treat, PP=Per Protocol)
Tafenoquine = Tafenoquine 200mg, Mefloquine = Mefloquine 250mg

SB252263 - 030

Listing B.02

Visit And Dosing Dates

Treatment : Mefloquine

Subject Number	Popn*	Visit	Visit Date	Dose Taken ?	Date of Dose	Time of Dose
030.001.00095	IPP	Week 4	06JUN2000	Yes	06JUN2000	10:59
		Week 4 (Unsch.)	07JUN2000	No		
		Week 5	13JUN2000	Yes	13JUN2000	17:40
		Week 6	20JUN2000	Yes	20JUN2000	7:10
		Week 7 (Fol. Up)	27JUN2000	No		
		Week 8 (Fol. Up)	05JUL2000	No		
		Week 9 (Fol. Up)	08JUL2000	No		
		Week 9 (Fol. Up)	11JUL2000	No		
		Week 10 (Fol. Up)	19JUL2000	No		
030.001.00096	IPP	Screening	12APR2000	No		
		Screening (Unsch.)	25APR2000	No		
		Clearance (Day -5)	03MAY2000	Yes	03MAY2000	
		Clearance (Day -4)	04MAY2000	Yes	04MAY2000	
		Clearance (Day -3)	05MAY2000	Yes	05MAY2000	
		Load Dose 1	08MAY2000	Yes	08MAY2000	12:42
		Load Dose 2	09MAY2000	Yes	09MAY2000	7:28
		Load Dose 3	10MAY2000	Yes	10MAY2000	7:35
		Load Dose (Unsch.)	10MAY2000	No		
		Week 1	15MAY2000	Yes	15MAY2000	8:50
		Week 1 (Unsch.)	18MAY2000	No		
		Week 2	22MAY2000	Yes	22MAY2000	8:17
		Week 2 (Unsch.)	23MAY2000	No		
		Week 3	29MAY2000	Yes	29MAY2000	7:14
		Week 4	05JUN2000	Yes	05JUN2000	16:50
		Week 5	12JUN2000	Yes	12JUN2000	7:46
		Week 6	19JUN2000	Yes	19JUN2000	6:54
		Week 7	26JUN2000	Yes	26JUN2000	9:03
		Week 7 (Unsch.)	28JUN2000	No		
		Week 8	03JUL2000	Yes	03JUL2000	11:55
		Week 9 (Unsch. Fol. Up)	07JUL2000	No		
		Week 9 (Fol. Up)	10JUL2000	No		
		Week 10 (Fol. Up)	17JUL2000	No		
		Week 11 (Fol. Up)	24JUL2000	No		
		Week 12 (Fol. Up)	31JUL2000	No		
030.001.00097	IPP	Screening	11APR2000	No		
		Screening (Unsch.)	19APR2000	No		
		Clearance (Day -5)	03MAY2000	Yes	03MAY2000	
		Clearance (Day -4)	04MAY2000	Yes	04MAY2000	
		Clearance (Day -3)	05MAY2000	Yes	05MAY2000	
		Load Dose 1	08MAY2000	Yes	08MAY2000	13:10

*Popn=Population (I=Intent-to-Treat, PP=Per Protocol)
Tafenoquine = Tafenoquine 200mg, Mefloquine = Mefloquine 250mg

SB252263 - 030

Listing B.02

Visit And Dosing Dates

Treatment : Mefloquine

Subject Number	Popn*	Visit	Visit Date	Dose Taken ?	Date of Dose	Time of Dose
030.001.00097	IPP	Load Dose 2	09MAY2000	Yes	09MAY2000	7:50
		Load Dose 3	10MAY2000	Yes	10MAY2000	10:05
		Week 1	15MAY2000	Yes	15MAY2000	9:00
		Week 2	23MAY2000	Yes	23MAY2000	6:40
		Week 2 (Unsch.)	23MAY2000	No		
		Week 3	29MAY2000	Yes	29MAY2000	10:00
		Week 4	05JUN2000	Yes	05JUN2000	13:15
		Week 5 (Unsch.)	10JUN2000	No		
		Week 5	12JUN2000	Yes	12JUN2000	12:45
		Week 6	19JUN2000	Yes	19JUN2000	7:00
		Week 7 (Fol. Up)	26JUN2000	No		
		Week 7 (Unsch. Fol. Up)	28JUN2000	No		
		Week 8 (Fol. Up)	03JUL2000	No		
		Week 8 (Fol. Up)	05JUL2000	No		
		Week 8 (Unsch. Fol. Up)	05JUL2000	No		
		Week 9 (Fol. Up)	10JUL2000	No		
		Week 10 (Fol. Up)	17JUL2000	No		
030.001.00098	IPP	Screening	12APR2000	No		
		Screening (Unsch.)	13APR2000	No		
		Clearance (Day -5)	03MAY2000	Yes	03MAY2000	
		Clearance (Day -4)	04MAY2000	Yes	04MAY2000	
		Clearance (Day -3)	05MAY2000	Yes	05MAY2000	
		Load Dose 1	08MAY2000	Yes	08MAY2000	13:09
		Load Dose 2	09MAY2000	Yes	09MAY2000	6:35
		Load Dose 3	10MAY2000	Yes	10MAY2000	7:54
		Week 1	15MAY2000	Yes	15MAY2000	8:20
		Week 2	22MAY2000	Yes	22MAY2000	7:30
		Week 3	29MAY2000	Yes	29MAY2000	6:47
		Week 4	06JUN2000	Yes	06JUN2000	13:41
		Week 5	12JUN2000	Yes	12JUN2000	9:10
		Week 6	19JUN2000	Yes	19JUN2000	6:57
		Week 7	26JUN2000	Yes	26JUN2000	9:01
		Week 7 (Unsch.)	28JUN2000	No		
		Week 8	03JUL2000	Yes	03JUL2000	11:40
		Week 8 (Unsch.)	06JUL2000	No		
		Week 9 (Unsch.)	07JUL2000	No		
		Week 9	09JUL2000	Yes	09JUL2000	18:24
		Week 10 (Fol. Up)	17JUL2000	No		
		Week 11 (Fol. Up)	25JUL2000	No		
		Week 12 (Fol. Up)	01AUG2000	No		

*Popn=Population (I=Intent-to-Treat, PP=Per Protocol)
Tafenoquine = Tafenoquine 200mg, Mefloquine = Mefloquine 250mg

SB252263 - 030

Listing B.02

Visit And Dosing Dates

Treatment : Mefloquine

Subject Number	Popn*	Visit	Visit Date	Dose Taken ?	Date of Dose	Time of Dose
030.001.00099	IPP	Screening	10APR2000	No		
		Clearance (Day -5)	03MAY2000	Yes	03MAY2000	
		Clearance (Day -4)	04MAY2000	Yes	04MAY2000	
		Clearance (Day -3)	05MAY2000	Yes	05MAY2000	
		Load Dose 1	08MAY2000	Yes	08MAY2000	13:22
		Load Dose 2	09MAY2000	Yes	09MAY2000	9:58
		Load Dose 3	10MAY2000	Yes	10MAY2000	8:42
		Load Dose (Unsch.)	09MAY2000	No		
		Week 1	15MAY2000	Yes	15MAY2000	10:55
		Week 2	22MAY2000	Yes	22MAY2000	8:08
		Week 3	29MAY2000	Yes	29MAY2000	9:45
		Week 4	06JUN2000	Yes	06JUN2000	17:05
		Week 5	12JUN2000	Yes	12JUN2000	9:06
		Week 6 (Fol. Up)	19JUN2000	No		
		Week 7 (Fol. Up)	26JUN2000	No		
		Week 7 (Unsch. Fol. Up)	27JUN2000	No		
		Week 8 (Unsch. Fol. Up)	01JUL2000	No		
		Week 8 (Fol. Up)	05JUL2000	No		
		Week 8 (Unsch. Fol. Up)	05JUL2000	No		
		Week 8 (Fol. Up)	06JUL2000	No		
		Week 9 (Unsch. Fol. Up)	07JUL2000	No		
		Week 9 (Fol. Up)	10JUL2000	No		
030.001.00105	IPP	Screening	11APR2000	No		
		Clearance (Day -5)	03MAY2000	Yes	03MAY2000	
		Clearance (Day -4)	04MAY2000	Yes	04MAY2000	
		Clearance (Day -3)	05MAY2000	Yes	05MAY2000	
		Load Dose 1	08MAY2000	Yes	08MAY2000	14:29
		Load Dose 2	09MAY2000	Yes	09MAY2000	8:57
		Load Dose 3	10MAY2000	Yes	10MAY2000	11:00
		Week 1	15MAY2000	Yes	15MAY2000	13:11
		Week 2	22MAY2000	Yes	22MAY2000	12:07
		Week 3 (Fol. Up)	29MAY2000	No		
		Week 4 (Fol. Up)	05JUN2000	No		
		Week 4 (Unsch. Fol. Up)	05JUN2000	No		
		Week 5 (Fol. Up)	12JUN2000	No		
		Week 6 (Unsch. Fol. Up)	19JUN2000	No		
		Week 6 (Fol. Up)	20JUN2000	No		
030.001.00110	IPP	Screening	10APR2000	No		
		Clearance (Day -5)	03MAY2000	Yes	03MAY2000	
		Clearance (Day -4)	04MAY2000	Yes	04MAY2000	

*Popn=Population (I=Intent-to-Treat, PP=Per Protocol)
Tafenoquine = Tafenoquine 200mg, Mefloquine = Mefloquine 250mg

SB252263 - 030

Listing B.02

Visit And Dosing Dates

Treatment : Mefloquine

Subject Number	Popn*	Visit	Visit Date	Dose Taken ?	Date of Dose	Time of Dose
030.001.00110	IPP	Clearance (Day -3)	05MAY2000	Yes	05MAY2000	
		Clearance (Unsch.)	04MAY2000	No		
		Load Dose 1	08MAY2000	Yes	08MAY2000	15:04
		Load Dose 2	09MAY2000	Yes	09MAY2000	13:46
		Load Dose 3	10MAY2000	Yes	10MAY2000	10:30
		Week 1	15MAY2000	Yes	15MAY2000	9:44
		Week 2	22MAY2000	Yes	22MAY2000	6:38
		Week 3	29MAY2000	Yes	29MAY2000	5:52
		Week 4	04JUN2000	Yes	04JUN2000	10:11
		Week 5	12JUN2000	Yes	12JUN2000	6:35
		Week 6	19JUN2000	Yes	19JUN2000	6:08
		Week 6 (Unsch.)	20JUN2000	No		
		Week 7	27JUN2000	Yes	27JUN2000	9:37
		Week 8	03JUL2000	Yes	03JUL2000	5:34
		Week 9	10JUL2000	Yes	10JUL2000	6:50
		Week 9	13JUL2000	No		
		Week 9 (Unsch.)	13JUL2000	No		
		Week 10	17JUL2000	Yes	17JUL2000	6:33
		Week 11 (Fol. Up)	24JUL2000	No		
		Week 12 (Fol. Up)	31JUL2000	No		
		Week 13 (Unsch. Fol. Up)	06AUG2000	No		
		Week 13 (Fol. Up)	07AUG2000	No		
		Week 13 (Unsch. Fol. Up)	08AUG2000	No		
		Week 14 (Fol. Up)	14AUG2000	No		
030.001.00113	IPP	Screening	17APR2000	No		
		Clearance (Day -5)	03MAY2000	Yes	03MAY2000	
		Clearance (Day -4)	04MAY2000	Yes	04MAY2000	
		Clearance (Day -3)	05MAY2000	Yes	05MAY2000	
		Load Dose 1	08MAY2000	Yes	08MAY2000	15:54
		Load Dose 2	09MAY2000	Yes	09MAY2000	7:58
		Load Dose 3	10MAY2000	Yes	10MAY2000	9:01
		Week 1	15MAY2000	Yes	15MAY2000	9:30
		Week 1 (Unsch.)	15MAY2000	No		
		Week 2	22MAY2000	Yes	22MAY2000	8:45
		Week 2 (Unsch.)	24MAY2000	No		
		Week 3 (Unsch.)	27MAY2000	No		
		Week 3	29MAY2000	Yes	29MAY2000	8:03
		Week 4	05JUN2000	Yes	05JUN2000	15:25
		Week 4 (Unsch.)	07JUN2000	No		
		Week 5	12JUN2000	Yes	12JUN2000	7:41
		Week 6	19JUN2000	Yes	19JUN2000	7:48
		Week 6 (Unsch.)	20JUN2000	No		

*Popn=Population (I=Intent-to-Treat, PP=Per Protocol)
Tafenoquine = Tafenoquine 200mg, Mefloquine = Mefloquine 250mg

SB252263 - 030

Listing B.02

Visit And Dosing Dates

Treatment : Mefloquine

Subject Number	Popn*	Visit	Visit Date	Dose Taken ?	Date of Dose	Time of Dose
030.001.00113	IPP	Week 7	26JUN2000	Yes	26JUN2000	9:30
		Week 8 (Fol. Up)	03JUL2000	No		
		Week 8 (Fol. Up)	04JUL2000	No		
		Week 9 (Fol. Up)	10JUL2000	No		
		Week 9 (Unsch. Fol. Up)	10JUL2000	No		
		Week 10 (Fol. Up)	17JUL2000	No		
		Week 11 (Unsch. Fol. Up)	21JUL2000	No		
		Week 11 (Fol. Up)	24JUL2000	No		
030.001.00117	IPP	Screening	11APR2000	No		
		Clearance (Day -5)	03MAY2000	Yes	03MAY2000	
		Clearance (Day -4)	04MAY2000	Yes	04MAY2000	
		Clearance (Day -3)	05MAY2000	Yes	05MAY2000	
		Load Dose 1	08MAY2000	Yes	08MAY2000	
		Load Dose 2	09MAY2000	Yes	09MAY2000	8:24
		Load Dose 3	10MAY2000	Yes	10MAY2000	11:03
		Week 1 (Unsch.)	11MAY2000	No		
		Week 1 (Unsch.)	12MAY2000	No		
		Week 1	15MAY2000	Yes	15MAY2000	12:25
		Week 1 (Unsch.)	15MAY2000	No		
		Week 2	22MAY2000	Yes	22MAY2000	7:06
		Week 3	29MAY2000	Yes	29MAY2000	7:21
		Week 3 (Unsch.)	01JUN2000	No		
		Week 4	05JUN2000	Yes	05JUN2000	11:55
		Week 5	12JUN2000	Yes	12JUN2000	7:23
		Week 6	19JUN2000	Yes	19JUN2000	10:24
		Week 7	26JUN2000	Yes	26JUN2000	7:59
		Week 8 (Fol. Up)	03JUL2000	No		
		Week 9 (Fol. Up)	10JUL2000	No		
		Week 10 (Fol. Up)	17JUL2000	No		
030.001.00120	IPP	Screening	10APR2000	No		
		Clearance (Day -5)	04MAY2000	Yes	04MAY2000	
		Clearance (Day -4)	05MAY2000	Yes	05MAY2000	
		Clearance (Day -3)	06MAY2000	Yes	06MAY2000	
		Load Dose 1	09MAY2000	Yes	09MAY2000	6:45
		Load Dose 2	10MAY2000	Yes	10MAY2000	7:45
		Load Dose 3	11MAY2000	Yes	11MAY2000	7:50
		Week 1	15MAY2000	Yes	15MAY2000	9:20
		Week 2	22MAY2000	Yes	22MAY2000	7:20
		Week 2 (Unsch. Fol. Up)	25MAY2000	No		
		Week 2 (Unsch. Fol. Up)	26MAY2000	No		

*Popn=Population (I=Intent-to-Treat, PP=Per Protocol)
Tafenoquine = Tafenoquine 200mg, Mefloquine = Mefloquine 250mg

SB252263 - 030

Listing B.02

Visit And Dosing Dates

Treatment : Mefloquine

Subject Number	Popn*	Visit	Visit Date	Dose Taken ?	Date of Dose	Time of Dose
030.001.00120	IPP	Week 3 (Unsch. Fol. Up)	27MAY2000	No		
		Week 3 (Fol. Up)	29MAY2000	No		
		Week 4 (Fol. Up)	04JUN2000	No		
		Week 4 (Unsch. Fol. Up)	06JUN2000	No		
		Week 5 (Fol. Up)	12JUN2000	No		
		Week 6 (Fol. Up)	19JUN2000	No		
030.001.00121	IPP	Screening	10APR2000	No		
		Clearance (Day -5)	04MAY2000	Yes	04MAY2000	
		Clearance (Day -4)	05MAY2000	Yes	05MAY2000	
		Clearance (Day -3)	06MAY2000	Yes	06MAY2000	
		Load Dose 1	09MAY2000	Yes	09MAY2000	6:50
		Load Dose 2	10MAY2000	Yes	10MAY2000	17:02
		Load Dose 3	11MAY2000	Yes	11MAY2000	7:15
		Week 1	15MAY2000	Yes	15MAY2000	10:12
		Week 1 (Unsch.)	15MAY2000	No		
		Week 2	22MAY2000	Yes	22MAY2000	8:25
		Week 3	29MAY2000	Yes	29MAY2000	7:34
		Week 4	05JUN2000	Yes	05JUN2000	9:17
		Week 5	12JUN2000	Yes	12JUN2000	11:40
		Week 6	19JUN2000	Yes	19JUN2000	11:40
		Week 7	26JUN2000	Yes	26JUN2000	8:18
		Week 8 (Unsch. Fol. Up)	02JUL2000	No		
		Week 8 (Fol. Up)	03JUL2000	No		
		Week 9 (Fol. Up)	10JUL2000	No		
		Week 10 (Fol. Up)	17JUL2000	No		
030.001.00123	I	Screening	10APR2000	No		
		Screening (Unsch.)	14APR2000	No		
		Screening (Unsch.)	19APR2000	No		
		Screening (Unsch.)	27APR2000	No		
		Clearance (Day -5)	04MAY2000	Yes	04MAY2000	
		Clearance (Day -4)	05MAY2000	Yes	05MAY2000	
		Clearance (Day -3)	06MAY2000	Yes	06MAY2000	
		Load Dose 1	09MAY2000	Yes	09MAY2000	6:54
		Load Dose 2	10MAY2000	Yes	10MAY2000	10:50
		Load Dose 3	11MAY2000	Yes	11MAY2000	13:35
		Week 1	16MAY2000	Yes	16MAY2000	9:25
		Week 1 (Unsch.)	16MAY2000	No		
		Week 2	22MAY2000	Yes	22MAY2000	6:50
		Week 2 (Unsch.)	24MAY2000	No		
		Week 2 (Unsch.)	25MAY2000	No		

*Popn=Population (I=Intent-to-Treat, PP=Per Protocol)
Tafenoquine = Tafenoquine 200mg, Mefloquine = Mefloquine 250mg

SB252263 - 030

Listing B.02

Visit And Dosing Dates

Treatment : Mefloquine

Subject Number	Popn*	Visit	Visit Date	Dose Taken ?	Date of Dose	Time of Dose
030.001.00123	I	Week 3	29MAY2000	Yes	29MAY2000	15:45
		Week 4	05JUN2000	Yes	05JUN2000	9:47
		Week 5	12JUN2000	No		
		Week 6	21JUN2000	Yes	21JUN2000	13:54
		Week 7 (Fol. Up)	26JUN2000	No		
		Week 7 (Unsch. Fol. Up)	27JUN2000	No		
		Week 7 (Unsch. Fol. Up)	29JUN2000	No		
		Week 8 (Fol. Up)	05JUL2000	No		
		Week 9 (Fol. Up)	10JUL2000	No		
		Week 10 (Fol. Up)	17JUL2000	No		
		Week 10 (Unsch. Fol. Up)	20JUL2000	No		
030.001.00130	IPP	Screening	10APR2000	No		
		Screening (Unsch.)	20APR2000	No		
		Screening (Unsch.)	26APR2000	No		
		Clearance (Day -5)	04MAY2000	Yes	04MAY2000	
		Clearance (Day -4)	05MAY2000	Yes	05MAY2000	
		Clearance (Day -3)	06MAY2000	Yes	06MAY2000	
		Load Dose 1	09MAY2000	Yes	09MAY2000	7:29
		Load Dose 2	10MAY2000	Yes	10MAY2000	14:10
		Load Dose 3	11MAY2000	Yes	11MAY2000	8:40
		Week 1 (Unsch.)	13MAY2000	No		
		Week 1	15MAY2000	Yes	15MAY2000	11:35
		Week 1 (Unsch.)	18MAY2000	No		
		Week 2	22MAY2000	Yes	22MAY2000	10:40
		Week 2 (Unsch.)	25MAY2000	No		
		Week 3	29MAY2000	Yes	29MAY2000	10:30
		Week 3 (Unsch.)	31MAY2000	No		
		Week 4	07JUN2000	Yes	07JUN2000	
		Week 5	12JUN2000	Yes	12JUN2000	10:15
		Week 6	19JUN2000	Yes	19JUN2000	
		Week 7	26JUN2000	Yes	26JUN2000	9:50
		Week 8	03JUL2000	Yes	03JUL2000	8:55
		Week 9	10JUL2000	Yes	10JUL2000	10:19
		Week 10	17JUL2000	Yes	17JUL2000	8:55
		Week 11	24JUL2000	Yes	24JUL2000	9:47
		Week 12 (Fol. Up)	31JUL2000	No		
		Week 13 (Fol. Up)	07AUG2000	No		
		Week 13 (Unsch. Fol. Up)	10AUG2000	No		
		Week 14 (Fol. Up)	16AUG2000	No		
		Week 15 (Unsch. Fol. Up)	19AUG2000	No		

*Popn=Population (I=Intent-to-Treat, PP=Per Protocol)
Tafenoquine = Tafenoquine 200mg, Mefloquine = Mefloquine 250mg

SB252263 - 030

Listing B.02

Visit And Dosing Dates

Treatment : Mefloquine

Subject Number	Popn*	Visit	Visit Date	Dose Taken ?	Date of Dose	Time of Dose
030.001.00132	IPP	Screening	13APR2000	No		
		Screening (Unsch.)	02MAY2000	No		
		Clearance (Day -5)	04MAY2000	Yes	04MAY2000	
		Clearance (Day -4)	05MAY2000	Yes	05MAY2000	
		Clearance (Day -3)	06MAY2000	Yes	06MAY2000	
		Load Dose 1	09MAY2000	Yes	09MAY2000	7:30
		Load Dose 2	10MAY2000	Yes	10MAY2000	11:00
		Load Dose 3	11MAY2000	Yes	11MAY2000	7:00
		Week 1 (Unsch.)	12MAY2000	No		
		Week 1	15MAY2000	Yes	15MAY2000	7:00
		Week 2	22MAY2000	Yes	22MAY2000	6:00
		Week 2 (Unsch.)	23MAY2000	No		
		Week 3	29MAY2000	Yes	29MAY2000	7:00
		Week 3 (Unsch.)	29MAY2000	No		
		Week 3 (Unsch.)	30MAY2000	No		
		Week 4	05JUN2000	Yes	05JUN2000	9:30
		Week 5	12JUN2000	Yes	12JUN2000	5:40
		Week 5 (Unsch.)	12JUN2000	No		
		Week 6	19JUN2000	Yes	19JUN2000	7:15
		Week 7	26JUN2000	Yes	26JUN2000	5:50
		Week 8 (Fol. Up)	03JUL2000	No		
		Week 9 (Fol. Up)	09JUL2000	No		
		Week 10 (Fol. Up)	17JUL2000	No		
		Week 11 (Unsch. Fol. Up)	22JUL2000	No		
030.001.00134	IPP	Screening	11APR2000	No		
		Clearance (Day -5)	04MAY2000	Yes	04MAY2000	
		Clearance (Day -4)	05MAY2000	Yes	05MAY2000	
		Clearance (Day -3)	06MAY2000	Yes	06MAY2000	
		Load Dose 1	09MAY2000	Yes	09MAY2000	8:07
		Load Dose 2	10MAY2000	Yes	10MAY2000	17:20
		Load Dose 3	11MAY2000	Yes	11MAY2000	8:20
		Week 1	16MAY2000	Yes	16MAY2000	9:00
		Week 2	23MAY2000	Yes	23MAY2000	8:45
		Week 3	29MAY2000	Yes	29MAY2000	14:43
		Week 4	06JUN2000	Yes	06JUN2000	7:15
		Week 5	12JUN2000	Yes	12JUN2000	16:40
		Week 5 (Unsch.)	13JUN2000	No		
		Week 6	19JUN2000	Yes	19JUN2000	12:35
		Week 7	26JUN2000	Yes	26JUN2000	11:55
		Week 7 (Unsch.)	27JUN2000	No		
		Week 8	04JUL2000	Yes	04JUL2000	6:25
		Week 9	10JUL2000	Yes	10JUL2000	13:47

*Popn=Population (I=Intent-to-Treat, PP=Per Protocol)
Tafenoquine = Tafenoquine 200mg, Mefloquine = Mefloquine 250mg

SB252263 - 030

Listing B.02

Visit And Dosing Dates

Treatment : Mefloquine

Subject Number	Popn*	Visit	Visit Date	Dose Taken ?	Date of Dose	Time of Dose
030.001.00134	IPP	Week 10	17JUL2000	Yes	17JUL2000	10:00
		Week 11	26JUL2000	Yes	26JUL2000	14:35
		Week 12 (Fol. Up)	02AUG2000	No		
		Week 13 (Fol. Up)	08AUG2000	No		
		Week 14 (Fol. Up)	15AUG2000	No		
		Week 15 (Unsch. Fol. Up)	22AUG2000	No		
		Week 15 (Fol. Up)	24AUG2000	No		
030.001.00142	IPP	Screening	14APR2000	No		
		Clearance (Day -5)	04MAY2000	Yes	04MAY2000	
		Clearance (Day -4)	05MAY2000	Yes	05MAY2000	
		Clearance (Day -3)	06MAY2000	Yes	06MAY2000	
		Load Dose 1	09MAY2000	Yes	09MAY2000	8:15
		Load Dose 2	10MAY2000	Yes	10MAY2000	13:15
		Load Dose 3	11MAY2000	Yes	11MAY2000	7:30
		Week 1	15MAY2000	Yes	15MAY2000	9:40
		Week 2	22MAY2000	Yes	22MAY2000	12:56
		Week 3	29MAY2000	Yes	29MAY2000	6:55
		Week 4	04JUN2000	Yes	04JUN2000	9:50
		Week 5	12JUN2000	Yes	12JUN2000	6:35
		Week 6	19JUN2000	Yes	19JUN2000	6:35
		Week 7	26JUN2000	Yes	26JUN2000	6:35
		Week 8 (Unsch.)	01JUL2000	No		
		Week 8	03JUL2000	Yes	03JUL2000	6:30
		Week 9	10JUL2000	Yes	10JUL2000	7:30
		Week 10	17JUL2000	Yes	17JUL2000	17:30
		Week 11	24JUL2000	Yes	24JUL2000	6:45
		Week 12 (Unsch.)	30JUL2000	No		
		Week 12	31JUL2000	Yes	31JUL2000	7:10
		Week 13	07AUG2000	Yes	07AUG2000	7:20
		Week 14	14AUG2000	Yes	14AUG2000	10:35
		Week 15	21AUG2000	Yes	21AUG2000	7:25
		Week 16	28AUG2000	Yes	28AUG2000	7:25
		Week 16	30AUG2000	No		
		Week 17	04SEP2000	Yes	04SEP2000	7:35
		Week 18	11SEP2000	Yes	11SEP2000	6:55
		Week 19	18SEP2000	Yes	18SEP2000	6:45
		Week 20 (Fol. Up)	25SEP2000	No		
		Week 21 (Fol. Up)	02OCT2000	No		
		Week 22 (Fol. Up)	09OCT2000	No		
030.001.00143	IPP	Screening	14APR2000	No		

*Popn=Population (I=Intent-to-Treat, PP=Per Protocol)
Tafenoquine = Tafenoquine 200mg, Mefloquine = Mefloquine 250mg

SB252263 - 030

Listing B.02

Visit And Dosing Dates

Treatment : Mefloquine

Subject Number	Popn*	Visit	Visit Date	Dose Taken ?	Date of Dose	Time of Dose
030.001.00143	IPP	Screening (Unsch.)	19APR2000	No		
		Screening (Unsch.)	23APR2000	No		
		Clearance (Day -5)	04MAY2000	Yes	04MAY2000	
		Clearance (Day -4)	05MAY2000	Yes	05MAY2000	
		Clearance (Day -3)	06MAY2000	Yes	06MAY2000	
		Load Dose 1	09MAY2000	Yes	09MAY2000	8:45
		Load Dose 2	10MAY2000	Yes	10MAY2000	8:07
		Load Dose 3	11MAY2000	Yes	11MAY2000	6:57
		Week 1	15MAY2000	Yes	15MAY2000	7:51
		Week 2	22MAY2000	Yes	22MAY2000	7:49
		Week 3	29MAY2000	Yes	29MAY2000	6:56
		Week 4	04JUN2000	Yes	04JUN2000	8:46
		Week 4 (Unsch.)	04JUN2000	No		
		Week 5	12JUN2000	Yes	12JUN2000	7:55
		Week 6	19JUN2000	Yes	19JUN2000	6:58
		Week 7	26JUN2000	Yes	26JUN2000	7:50
		Week 7 (Unsch.)	26JUN2000	No		
		Week 8 (Fol. Up)	03JUL2000	No		
		Week 9 (Fol. Up)	11JUL2000	No		
		Week 9 (Unsch. Fol. Up)	12JUL2000	No		
		Week 10 (Fol. Up)	17JUL2000	No		
030.001.00144	IPP	Screening	11APR2000	No		
		Clearance (Day -5)	04MAY2000	Yes	04MAY2000	
		Clearance (Day -4)	05MAY2000	Yes	05MAY2000	
		Clearance (Day -3)	06MAY2000	Yes	06MAY2000	
		Load Dose 1	09MAY2000	Yes	09MAY2000	8:51
		Load Dose 2	10MAY2000	Yes	10MAY2000	8:15
		Load Dose 3	11MAY2000	Yes	11MAY2000	6:58
		Week 1	16MAY2000	Yes	16MAY2000	7:50
		Week 2	23MAY2000	Yes	23MAY2000	10:00
		Week 3	29MAY2000	Yes	29MAY2000	6:00
		Week 4	05JUN2000	Yes	05JUN2000	17:40
		Week 5 (Fol. Up)	12JUN2000	No		
		Week 5 (Unsch. Fol. Up)	12JUN2000	No		
		Week 6 (Fol. Up)	19JUN2000	No		
		Week 7 (Fol. Up)	28JUN2000	No		
		Week 8 (Fol. Up)	03JUL2000	No		
030.001.00146	I	Screening	13APR2000	No		
		Clearance (Day -5)	04MAY2000	Yes	04MAY2000	
		Clearance (Day -4)	05MAY2000	Yes	05MAY2000	

*Popn=Population (I=Intent-to-Treat, PP=Per Protocol)
Tafenoquine = Tafenoquine 200mg, Mefloquine = Mefloquine 250mg

SB252263 - 030

Listing B.02

Visit And Dosing Dates

Treatment : Mefloquine

Subject Number	Popn*	Visit	Visit Date	Dose Taken ?	Date of Dose	Time of Dose
030.001.00146	I	Clearance (Day -3)	06MAY2000	Yes	06MAY2000	
		Load Dose 1	09MAY2000	Yes	09MAY2000	9:06
		Load Dose 2	10MAY2000	Yes	10MAY2000	8:12
		Load Dose 3	11MAY2000	Yes	11MAY2000	16:54
		Week 1	16MAY2000	Yes	16MAY2000	10:48
		Week 1 (Unsch.)	18MAY2000	No		
		Week 2	23MAY2000	Yes	23MAY2000	8:03
		Week 3	29MAY2000	Yes	29MAY2000	14:29
		Week 3 (Unsch.)	29MAY2000	No		
		Week 4	07JUN2000	Yes	07JUN2000	8:45
		Week 5	12JUN2000	Yes	12JUN2000	8:15
		Week 6	20JUN2000	Yes	20JUN2000	10:45
		Week 7 (Fol. Up)	26JUN2000	No		
030.001.00147	IPP	Screening	10APR2000	No		
		Screening (Unsch.)	22APR2000	No		
		Clearance (Day -5)	04MAY2000	Yes	04MAY2000	
		Clearance (Day -4)	05MAY2000	Yes	05MAY2000	
		Clearance (Day -3)	06MAY2000	Yes	06MAY2000	
		Load Dose 1	09MAY2000	Yes	09MAY2000	8:35
		Load Dose 2	10MAY2000	Yes	10MAY2000	7:25
		Load Dose 3	11MAY2000	Yes	11MAY2000	7:55
		Week 1	15MAY2000	Yes	15MAY2000	7:25
		Week 2	22MAY2000	Yes	22MAY2000	7:00
		Week 3	29MAY2000	Yes	29MAY2000	7:42
		Week 4	05JUN2000	Yes	05JUN2000	9:26
		Week 5	12JUN2000	Yes	12JUN2000	7:47
		Week 6	19JUN2000	Yes	19JUN2000	7:40
		Week 7	27JUN2000	Yes	27JUN2000	9:40
		Week 8	04JUL2000	Yes	04JUL2000	10:09
		Week 8	05JUL2000	No		
		Week 9	10JUL2000	Yes	10JUL2000	16:16
		Week 10	18JUL2000	Yes	18JUL2000	7:55
		Week 11	24JUL2000	Yes	24JUL2000	8:20
		Week 12	31JUL2000	Yes	31JUL2000	7:20
		Week 13 (Fol. Up)	07AUG2000	No		
		Week 14 (Fol. Up)	15AUG2000	No		
		Week 15 (Unsch. Fol. Up)	19AUG2000	No		
		Week 15 (Fol. Up)	21AUG2000	No		
030.001.00153	IPP	Screening	12APR2000	No		
		Clearance (Day -5)	04MAY2000	Yes	04MAY2000	

*Popn=Population (I=Intent-to-Treat, PP=Per Protocol)
Tafenoquine = Tafenoquine 200mg, Mefloquine = Mefloquine 250mg

SB252263 - 030

Listing B.02

Visit And Dosing Dates

Treatment : Mefloquine

Subject Number	Popn*	Visit	Visit Date	Dose Taken ?	Date of Dose	Time of Dose
030.001.00153	IPP	Clearance (Day -4)	05MAY2000	Yes	05MAY2000	
		Clearance (Day -3)	06MAY2000	Yes	06MAY2000	
		Load Dose 1	09MAY2000	Yes	09MAY2000	9:20
		Load Dose 2	10MAY2000	Yes	10MAY2000	10:25
		Load Dose 3	11MAY2000	Yes	11MAY2000	10:03
		Week 1	15MAY2000	Yes	15MAY2000	6:57
		Week 2	22MAY2000	Yes	22MAY2000	6:00
		Week 3	29MAY2000	Yes	29MAY2000	6:00
		Week 4	04JUN2000	Yes	04JUN2000	12:34
		Week 5	12JUN2000	Yes	12JUN2000	6:10
		Week 6	19JUN2000	Yes	19JUN2000	6:04
		Week 7	26JUN2000	Yes	26JUN2000	6:12
		Week 8	05JUL2000	Yes	05JUL2000	12:25
		Week 9	10JUL2000	Yes	10JUL2000	7:05
		Week 10	17JUL2000	Yes	17JUL2000	8:45
		Week 11	24JUL2000	Yes	24JUL2000	6:45
		Week 12	31JUL2000	Yes	31JUL2000	7:20
		Week 13	11AUG2000	Yes	11AUG2000	9:57
		Week 14	14AUG2000	Yes	14AUG2000	8:06
		Week 15	21AUG2000	Yes	21AUG2000	11:10
		Week 16	28AUG2000	Yes	28AUG2000	6:45
		Week 16	30AUG2000	No		
		Week 17	04SEP2000	Yes	04SEP2000	7:10
		Week 18	11SEP2000	Yes	11SEP2000	7:28
		Week 19	18SEP2000	Yes	18SEP2000	7:12
		Week 20	29SEP2000	Yes	29SEP2000	17:37
		Week 21	05OCT2000	Yes	05OCT2000	8:50
		Week 22	10OCT2000	Yes	10OCT2000	11:02
		Week 23	16OCT2000	Yes	16OCT2000	7:30
		Week 24 (Fol. Up)	23OCT2000	No		
		Week 24 (Unsch. Fol. Up)	23OCT2000	No		
		Week 25 (Fol. Up)	01NOV2000	No		
		Week 26 (Fol. Up)	08NOV2000	No		
		Week 27 (Fol. Up)	14NOV2000	No		
030.001.00156	IPP	Screening	14APR2000	No		
		Screening (Unsch.)	02MAY2000	No		
		Clearance (Day -5)	04MAY2000	Yes	04MAY2000	
		Clearance (Day -4)	05MAY2000	Yes	05MAY2000	
		Clearance (Day -3)	06MAY2000	Yes	06MAY2000	
		Clearance (Unsch.)	04MAY2000	No		
		Load Dose 1	09MAY2000	Yes	09MAY2000	10:25
		Load Dose 2	10MAY2000	Yes	10MAY2000	10:20

*Popn=Population (I=Intent-to-Treat, PP=Per Protocol)
Tafenoquine = Tafenoquine 200mg, Mefloquine = Mefloquine 250mg

SB252263 - 030

Listing B.02

Visit And Dosing Dates

Treatment : Mefloquine

Subject Number	Popn*	Visit	Visit Date	Dose Taken ?	Date of Dose	Time of Dose
030.001.00156	IPP	Load Dose 3	11MAY2000	Yes	11MAY2000	7:35
		Week 1	15MAY2000	Yes	15MAY2000	8:00
		Week 2	22MAY2000	Yes	22MAY2000	18:06
		Week 2 (Unsch.)	24MAY2000	No		
		Week 3	29MAY2000	Yes	29MAY2000	14:10
		Week 4	04JUN2000	Yes	04JUN2000	12:10
		Week 5	12JUN2000	Yes	12JUN2000	13:45
		Week 6 (Unsch.)	18JUN2000	No		
		Week 6	19JUN2000	Yes	19JUN2000	16:58
		Week 6 (Unsch.)	20JUN2000	No		
		Week 7	26JUN2000	Yes	26JUN2000	12:30
		Week 7 (Unsch.)	26JUN2000	No		
		Week 8	03JUL2000	Yes	03JUL2000	17:55
		Week 8 (Fol. Up)	04JUL2000	No		
		Week 9 (Fol. Up)	10JUL2000	No		
		Week 10 (Fol. Up)	18JUL2000	No		
		Week 11 (Fol. Up)	25JUL2000	No		
030.001.00163	IPP	Screening	11APR2000	No		
		Screening (Unsch.)	20APR2000	No		
		Clearance (Day -5)	04MAY2000	Yes	04MAY2000	
		Clearance (Day -4)	05MAY2000	Yes	05MAY2000	
		Clearance (Day -3)	06MAY2000	Yes	06MAY2000	
		Load Dose 1	09MAY2000	Yes	09MAY2000	10:04
		Load Dose 2	10MAY2000	Yes	10MAY2000	8:38
		Load Dose 3	11MAY2000	Yes	11MAY2000	7:05
		Week 1	15MAY2000	Yes	15MAY2000	7:49
		Week 1 (Unsch.)	15MAY2000	No		
		Week 2	22MAY2000	Yes	22MAY2000	8:16
		Week 3	29MAY2000	Yes	29MAY2000	9:00
		Week 4	05JUN2000	Yes	05JUN2000	12:50
		Week 5	12JUN2000	Yes	12JUN2000	8:17
		Week 6	19JUN2000	Yes	19JUN2000	11:59
		Week 7	26JUN2000	Yes	26JUN2000	8:15
		Week 8 (Fol. Up)	03JUL2000	No		
		Week 8 (Unsch. Fol. Up)	03JUL2000	No		
		Week 9 (Fol. Up)	11JUL2000	No		
		Week 10 (Fol. Up)	17JUL2000	No		
		Week 11 (Unsch. Fol. Up)	26JUL2000	No		
030.001.00165	IPP	Screening	11APR2000	No		
		Clearance (Day -5)	04MAY2000	Yes	04MAY2000	

*Popn=Population (I=Intent-to-Treat, PP=Per Protocol)
Tafenoquine = Tafenoquine 200mg, Mefloquine = Mefloquine 250mg

SB252263 - 030

Listing B.02

Visit And Dosing Dates

Treatment : Mefloquine

Subject Number	Popn*	Visit	Visit Date	Dose Taken ?	Date of Dose	Time of Dose
030.001.00165	IPP	Clearance (Day -4)	05MAY2000	Yes	05MAY2000	
		Clearance (Day -3)	06MAY2000	Yes	06MAY2000	
		Load Dose 1	09MAY2000	Yes	09MAY2000	9:25
		Load Dose 2	10MAY2000	Yes	10MAY2000	10:53
		Load Dose 3	11MAY2000	Yes	11MAY2000	8:00
		Week 1	16MAY2000	Yes	16MAY2000	20:09
		Week 2	22MAY2000	Yes	22MAY2000	8:34
		Week 3	29MAY2000	Yes	29MAY2000	9:55
		Week 4	05JUN2000	Yes	05JUN2000	13:45
		Week 5 (Fol. Up)	12JUN2000	No		
		Week 5 (Unsch. Fol. Up)	12JUN2000	No		
		Week 6 (Fol. Up)	19JUN2000	No		
		Week 7 (Unsch. Fol. Up)	25JUN2000	No		
		Week 7 (Fol. Up)	26JUN2000	No		
		Week 8 (Fol. Up)	03JUL2000	No		
030.001.00167	IPP	Screening	13APR2000	No		
		Screening (Unsch.)	30APR2000	No		
		Clearance (Day -5)	04MAY2000	Yes	04MAY2000	
		Clearance (Day -4)	05MAY2000	Yes	05MAY2000	
		Clearance (Day -3)	06MAY2000	Yes	06MAY2000	
		Clearance (Unsch.)	06MAY2000	No		
		Load Dose 1	09MAY2000	Yes	09MAY2000	10:44
		Load Dose 2	10MAY2000	Yes	10MAY2000	14:42
		Load Dose 3	11MAY2000	Yes	11MAY2000	12:00
		Week 1	15MAY2000	Yes	15MAY2000	7:33
		Week 2	22MAY2000	Yes	22MAY2000	6:50
		Week 3	29MAY2000	Yes	29MAY2000	6:50
		Week 4	04JUN2000	Yes	04JUN2000	
		Week 5	12JUN2000	Yes	12JUN2000	8:13
		Week 6 (Fol. Up)	19JUN2000	No		
		Week 6 (Unsch. Fol. Up)	19JUN2000	No		
		Week 7 (Fol. Up)	26JUN2000	No		
		Week 8 (Fol. Up)	03JUL2000	No		
		Week 8 (Fol. Up)	05JUL2000	No		
		Week 8 (Unsch. Fol. Up)	05JUL2000	No		
		Week 9 (Fol. Up)	10JUL2000	No		
030.001.00168	IPP	Screening	14APR2000	No		
		Clearance (Day -5)	04MAY2000	Yes	04MAY2000	
		Clearance (Day -4)	05MAY2000	Yes	05MAY2000	
		Clearance (Day -3)	06MAY2000	Yes	06MAY2000	

*Popn=Population (I=Intent-to-Treat, PP=Per Protocol)
Tafenoquine = Tafenoquine 200mg, Mefloquine = Mefloquine 250mg

SB252263 - 030

Listing B.02

Visit And Dosing Dates

Treatment : Mefloquine

Subject Number	Popn*	Visit	Visit Date	Dose Taken ?	Date of Dose	Time of Dose
030.001.00168	IPP	Load Dose 1	09MAY2000	Yes	09MAY2000	10:40
		Load Dose 2	10MAY2000	Yes	10MAY2000	7:20
		Load Dose 3	11MAY2000	Yes	11MAY2000	7:20
		Week 1	15MAY2000	Yes	15MAY2000	8:32
		Week 2	22MAY2000	Yes	22MAY2000	8:00
		Week 3	29MAY2000	Yes	29MAY2000	7:48
		Week 3 (Unsch.)	31MAY2000	No		
		Week 4	05JUN2000	Yes	05JUN2000	14:45
		Week 5 (Fol. Up)	12JUN2000	No		
		Week 6 (Fol. Up)	19JUN2000	No		
		Week 7 (Fol. Up)	26JUN2000	No		
030.001.00170	IPP	Screening	12APR2000	No		
		Clearance (Day -5)	04MAY2000	Yes	04MAY2000	
		Clearance (Day -4)	05MAY2000	Yes	05MAY2000	
		Clearance (Day -3)	06MAY2000	Yes	06MAY2000	
		Load Dose 1	09MAY2000	Yes	09MAY2000	9:52
		Load Dose 2	10MAY2000	Yes	10MAY2000	14:51
		Load Dose 3	11MAY2000	Yes	11MAY2000	7:59
		Week 1 (Unsch.)	13MAY2000	No		
		Week 1	16MAY2000	Yes	16MAY2000	10:20
		Week 2	22MAY2000	Yes	22MAY2000	7:32
		Week 3	29MAY2000	Yes	29MAY2000	7:10
		Week 4	06JUN2000	Yes	06JUN2000	9:45
		Week 5	12JUN2000	Yes	12JUN2000	7:50
		Week 6	19JUN2000	Yes	19JUN2000	9:22
		Week 7	26JUN2000	Yes	26JUN2000	7:30
		Week 7 (Unsch.)	26JUN2000	No		
		Week 8 (Fol. Up)	03JUL2000	No		
		Week 9 (Fol. Up)	10JUL2000	No		
		Week 10 (Fol. Up)	17JUL2000	No		
030.001.00177	IPP	Screening	13APR2000	No		
		Clearance (Day -5)	04MAY2000	Yes	04MAY2000	
		Clearance (Day -4)	05MAY2000	Yes	05MAY2000	
		Clearance (Day -3)	06MAY2000	Yes	06MAY2000	
		Load Dose 1	09MAY2000	Yes	09MAY2000	10:55
		Load Dose 2	10MAY2000	Yes	10MAY2000	9:05
		Load Dose 3	11MAY2000	Yes	11MAY2000	7:55
		Week 1	17MAY2000	Yes	17MAY2000	7:11
		Week 2	24MAY2000	Yes	24MAY2000	10:05
		Week 2 (Unsch.)	24MAY2000	No		

*Popn=Population (I=Intent-to-Treat, PP=Per Protocol)
Tafenoquine = Tafenoquine 200mg, Mefloquine = Mefloquine 250mg

SB252263 - 030

Listing B.02

Visit And Dosing Dates

Treatment : Mefloquine

Subject Number	Popn*	Visit	Visit Date	Dose Taken ?	Date of Dose	Time of Dose
030.001.00177	IPP	Week 3	31MAY2000	Yes	31MAY2000	9:35
		Week 4	06JUN2000	Yes	06JUN2000	15:14
		Week 5	14JUN2000	Yes	14JUN2000	8:30
		Week 6	21JUN2000	Yes	21JUN2000	9:30
		Week 7	28JUN2000	Yes	28JUN2000	10:30
		Week 8	04JUL2000	Yes	04JUL2000	8:00
		Week 9	12JUL2000	Yes	12JUL2000	8:15
		Week 10	19JUL2000	Yes	19JUL2000	9:30
		Week 11 (Fol. Up)	26JUL2000	No		
		Week 11 (Unsch. Fol. Up)	27JUL2000	No		
		Week 12 (Fol. Up)	02AUG2000	No		
		Week 13 (Fol. Up)	09AUG2000	No		
030.001.00178	I	Screening	12APR2000	No		
		Clearance (Day -5)	04MAY2000	Yes	04MAY2000	
		Clearance (Day -4)	05MAY2000	Yes	05MAY2000	
		Clearance (Day -3)	06MAY2000	Yes	06MAY2000	
		Clearance (Unsch.)	05MAY2000	No		
		Load Dose 1	09MAY2000	Yes	09MAY2000	10:34
		Load Dose 2	10MAY2000	Yes	10MAY2000	12:08
		Load Dose 3	14MAY2000	Yes	14MAY2000	8:56
		Load Dose (Unsch.)	14MAY2000	No		
		Week 1	17MAY2000	Yes	17MAY2000	12:41
		Week 2	24MAY2000	Yes	24MAY2000	11:30
		Week 3	31MAY2000	Yes	31MAY2000	9:00
		Week 4	06JUN2000	Yes	06JUN2000	
		Week 5	15JUN2000	Yes	15JUN2000	10:00
		Week 6	21JUN2000	Yes	21JUN2000	9:20
		Week 7	28JUN2000	Yes	28JUN2000	9:37
		Week 8	04JUL2000	Yes	04JUL2000	9:45
		Week 8 (Fol. Up)	06JUL2000	No		
030.001.00180	IPP	Screening	17APR2000	No		
		Clearance (Day -5)	04MAY2000	Yes	04MAY2000	
		Clearance (Day -4)	05MAY2000	Yes	05MAY2000	
		Clearance (Day -3)	06MAY2000	Yes	06MAY2000	
		Clearance (Unsch.)	04MAY2000	No		
		Load Dose 1	09MAY2000	Yes	09MAY2000	11:10
		Load Dose 2	10MAY2000	Yes	10MAY2000	7:18
		Load Dose 3	11MAY2000	Yes	11MAY2000	7:54
		Week 1	17MAY2000	Yes	17MAY2000	9:30
		Week 1 (Unsch.)	19MAY2000	No		

*Popn=Population (I=Intent-to-Treat, PP=Per Protocol)
Tafenoquine = Tafenoquine 200mg, Mefloquine = Mefloquine 250mg

Listing B.02

Visit And Dosing Dates

Treatment : Mefloquine

Subject Number	Popn*	Visit	Visit Date	Dose Taken ?	Date of Dose	Time of Dose
030.001.00180	IPP	Week 2	24MAY2000	Yes	24MAY2000	9:34
		Week 3 (Unsch.)	30MAY2000	No		
		Week 3	31MAY2000	Yes	31MAY2000	10:17
		Week 4	05JUN2000	Yes	05JUN2000	13:23
		Week 4 (Unsch.)	07JUN2000	No		
		Week 5 (Unsch.)	12JUN2000	No		
		Week 5	14JUN2000	Yes	14JUN2000	13:13
		Week 6	21JUN2000	Yes	21JUN2000	7:00
		Week 7	28JUN2000	Yes	28JUN2000	6:27
		Week 8	04JUL2000	Yes	04JUL2000	7:10
		Week 9	12JUL2000	Yes	12JUL2000	7:15
		Week 10	19JUL2000	Yes	19JUL2000	9:55
		Week 11	26JUL2000	Yes	26JUL2000	13:30
		Week 11 (Unsch.)	28JUL2000	No		
		Week 12	01AUG2000	Yes	01AUG2000	8:05
		Week 12 (Unsch.)	01AUG2000	No		
		Week 13 (Unsch.)	05AUG2000	No		
		Week 13	09AUG2000	Yes	09AUG2000	8:23
		Week 14	16AUG2000	Yes	16AUG2000	8:13
		Week 15	23AUG2000	Yes	23AUG2000	8:13
		Week 16	28AUG2000	Yes	28AUG2000	8:01
		Week 16	30AUG2000	No		
		Week 17	04SEP2000	Yes	04SEP2000	10:41
		Week 17 (Unsch.)	05SEP2000	No		
		Week 17 (Unsch.)	07SEP2000	No		
		Week 18	13SEP2000	Yes	13SEP2000	7:45
		Week 19	20SEP2000	Yes	20SEP2000	7:52
		Week 19 (Unsch.)	21SEP2000	No		
		Week 19 (Unsch.)	22SEP2000	No		
		Week 20	25SEP2000	Yes	25SEP2000	11:20
		Week 20 (Unsch.)	26SEP2000	No		
		Week 21	04OCT2000	Yes	04OCT2000	7:40
		Week 22	11OCT2000	Yes	11OCT2000	7:48
		Week 22 (Unsch.)	13OCT2000	No		
		Week 23	16OCT2000	Yes	16OCT2000	16:00
		Week 24 (Fol. Up)	23OCT2000	No		
		Week 24 (Unsch. Fol. Up)	23OCT2000	No		
		Week 24 (Unsch. Fol. Up)	24OCT2000	No		
		Week 25 (Unsch. Fol. Up)	03NOV2000	No		
		Week 26 (Unsch. Fol. Up)	08NOV2000	No		
		Week 27 (Unsch. Fol. Up)	11NOV2000	No		
		Week 27 (Fol. Up)	14NOV2000	No		

*Popn=Population (I=Intent-to-Treat, PP=Per Protocol)
Tafenoquine = Tafenoquine 200mg, Mefloquine = Mefloquine 250mg

SB252263 - 030

Listing B.02

Visit And Dosing Dates

Treatment : Mefloquine

Subject Number	Popn*	Visit	Visit Date	Dose Taken ?	Date of Dose	Time of Dose
030.001.00181	IPP	Screening	10APR2000	No		
		Clearance (Day -5)	04MAY2000	Yes	04MAY2000	
		Clearance (Day -4)	05MAY2000	Yes	05MAY2000	
		Clearance (Day -3)	06MAY2000	Yes	06MAY2000	
		Load Dose 1	09MAY2000	Yes	09MAY2000	10:45
		Load Dose 2	10MAY2000	Yes	10MAY2000	13:00
		Load Dose 3	11MAY2000	Yes	11MAY2000	11:10
		Week 1 (Unsch.)	13MAY2000	No		
		Week 1	17MAY2000	Yes	17MAY2000	6:49
		Week 1 (Unsch.)	17MAY2000	No		
		Week 2 (Unsch.)	23MAY2000	No		
		Week 2	24MAY2000	Yes	24MAY2000	6:48
		Week 2 (Unsch.)	25MAY2000	No		
		Week 2 (Unsch.)	26MAY2000	No		
		Week 3	31MAY2000	Yes	31MAY2000	6:51
		Week 4	06JUN2000	Yes	06JUN2000	12:59
		Week 4 (Unsch.)	08JUN2000	No		
		Week 5	14JUN2000	Yes	14JUN2000	6:45
		Week 6 (Fol. Up)	21JUN2000	No		
		Week 6 (Unsch. Fol. Up)	22JUN2000	No		
		Week 7 (Fol. Up)	28JUN2000	No		
		Week 7 (Unsch. Fol. Up)	28JUN2000	No		
		Week 8 (Fol. Up)	04JUL2000	No		
030.001.00185	IPP	Screening	18APR2000	No		
		Clearance (Day -5)	04MAY2000	Yes	04MAY2000	
		Clearance (Day -4)	05MAY2000	Yes	05MAY2000	
		Clearance (Day -3)	06MAY2000	Yes	06MAY2000	
		Load Dose 1	09MAY2000	Yes	09MAY2000	11:49
		Load Dose 2	10MAY2000	Yes	10MAY2000	12:30
		Load Dose 3	11MAY2000	Yes	11MAY2000	12:45
		Week 1	17MAY2000	Yes	17MAY2000	7:35
		Week 2	25MAY2000	Yes	25MAY2000	13:10
		Week 3	31MAY2000	Yes	31MAY2000	9:17
		Week 4	06JUN2000	Yes	06JUN2000	14:40
		Week 5	15JUN2000	Yes	15JUN2000	15:01
		Week 6 (Unsch.)	21JUN2000	No		
		Week 6	22JUN2000	Yes	22JUN2000	13:40
		Week 7	28JUN2000	Yes	28JUN2000	9:40
		Week 8	06JUL2000	Yes	06JUL2000	8:04
		Week 8	07JUL2000	No		
		Week 9	12JUL2000	Yes	12JUL2000	9:30
		Week 10 (Unsch.)	18JUL2000	No		

*Popn=Population (I=Intent-to-Treat, PP=Per Protocol)
Tafenoquine = Tafenoquine 200mg, Mefloquine = Mefloquine 250mg

SB252263 - 030

Listing B.02

Visit And Dosing Dates

Treatment : Mefloquine

Subject Number	Popn*	Visit	Visit Date	Dose Taken ?	Date of Dose	Time of Dose
030.001.00185	IPP	Week 10	19JUL2000	Yes	19JUL2000	9:30
		Week 11	26JUL2000	Yes	26JUL2000	8:00
		Week 12	03AUG2000	Yes	03AUG2000	10:00
		Week 13	09AUG2000	Yes	09AUG2000	7:11
		Week 14	16AUG2000	Yes	16AUG2000	11:25
		Week 15	23AUG2000	Yes	23AUG2000	7:00
		Week 16	28AUG2000	Yes	28AUG2000	11:47
		Week 17	06SEP2000	Yes	06SEP2000	9:35
		Week 17 (Unsch.)	08SEP2000	No		
		Week 18	13SEP2000	Yes	13SEP2000	10:00
		Week 19	20SEP2000	Yes	20SEP2000	10:11
		Week 20	26SEP2000	Yes	26SEP2000	10:53
		Week 21	05OCT2000	Yes	05OCT2000	10:16
		Week 22	09OCT2000	Yes	09OCT2000	11:24
		Week 23	16OCT2000	Yes	16OCT2000	6:37
		Week 24 (Fol. Up)	23OCT2000	No		
		Week 24 (Unsch. Fol. Up)	23OCT2000	No		
		Week 26 (Fol. Up)	07NOV2000	No		
030.001.00188	IPP	Screening	13APR2000	No		
		Clearance (Day -5)	04MAY2000	Yes	04MAY2000	
		Clearance (Day -4)	05MAY2000	Yes	05MAY2000	
		Clearance (Day -3)	06MAY2000	Yes	06MAY2000	
		Load Dose 1	09MAY2000	Yes	09MAY2000	11:51
		Load Dose 2	10MAY2000	Yes	10MAY2000	8:35
		Load Dose 3	11MAY2000	Yes	11MAY2000	7:10
		Load Dose (Unsch.)	11MAY2000	No		
		Week 1	17MAY2000	Yes	17MAY2000	11:45
		Week 2 (Unsch.)	23MAY2000	No		
		Week 2	25MAY2000	Yes	25MAY2000	12:46
		Week 2 (Unsch.)	25MAY2000	No		
		Week 3	31MAY2000	Yes	31MAY2000	9:50
		Week 4	05JUN2000	Yes	05JUN2000	15:56
		Week 5 (Fol. Up)	14JUN2000	No		
		Week 6 (Fol. Up)	21JUN2000	No		
		Week 6 (Unsch. Fol. Up)	21JUN2000	No		
		Week 7 (Unsch. Fol. Up)	26JUN2000	No		
		Week 7 (Unsch. Fol. Up)	27JUN2000	No		
		Week 7 (Fol. Up)	29JUN2000	No		
		Week 8 (Fol. Up)	05JUL2000	No		
030.001.00189	I	Screening	10APR2000	No		

*Popn=Population (I=Intent-to-Treat, PP=Per Protocol)
Tafenoquine = Tafenoquine 200mg, Mefloquine = Mefloquine 250mg

SB252263 - 030

Listing B.02

Visit And Dosing Dates

Treatment : Mefloquine

Subject Number	Popn*	Visit	Visit Date	Dose Taken ?	Date of Dose	Time of Dose
030.001.00189	I	Clearance (Day -5)	04MAY2000	Yes	04MAY2000	
		Clearance (Day -4)	06MAY2000	Yes	06MAY2000	
		Clearance (Day -3)	07MAY2000	Yes	07MAY2000	
		Load Dose 1	09MAY2000	Yes	09MAY2000	12:10
		Load Dose 2	10MAY2000	Yes	10MAY2000	10:10
		Load Dose 3	11MAY2000	Yes	11MAY2000	7:20
		Week 1	17MAY2000	Yes	17MAY2000	8:20
		Week 2	24MAY2000	Yes	24MAY2000	8:30
		Week 3	31MAY2000	Yes	31MAY2000	6:40
		Week 4 (Fol. Up)	07JUN2000	No		
		Week 5 (Fol. Up)	14JUN2000	No		
		Week 6 (Fol. Up)	21JUN2000	No		
		Week 7 (Fol. Up)	30JUN2000	No		
030.001.00196	IPP	Screening	11APR2000	No		
		Screening (Unsch.)	18APR2000	No		
		Clearance (Day -5)	04MAY2000	Yes	04MAY2000	
		Clearance (Day -4)	05MAY2000	Yes	05MAY2000	
		Clearance (Day -3)	06MAY2000	Yes	06MAY2000	
		Load Dose 1	09MAY2000	Yes	09MAY2000	12:25
		Load Dose 2	10MAY2000	Yes	10MAY2000	11:37
		Load Dose 3	11MAY2000	Yes	11MAY2000	8:36
		Week 1	17MAY2000	Yes	17MAY2000	7:15
		Week 2	24MAY2000	Yes	24MAY2000	8:44
		Week 3	31MAY2000	Yes	31MAY2000	8:10
		Week 4	06JUN2000	Yes	06JUN2000	
		Week 5	15JUN2000	Yes	15JUN2000	12:24
		Week 6	22JUN2000	Yes	22JUN2000	8:30
		Week 7	28JUN2000	Yes	28JUN2000	9:15
		Week 8	04JUL2000	Yes	04JUL2000	10:15
		Week 9	12JUL2000	Yes	12JUL2000	9:00
		Week 10 (Fol. Up)	20JUL2000	No		
		Week 11 (Fol. Up)	26JUL2000	No		
		Week 12 (Fol. Up)	02AUG2000	No		
		Week 13 (Fol. Up)	09AUG2000	No		
030.001.00198	IPP	Screening	12APR2000	No		
		Clearance (Day -5)	04MAY2000	Yes	04MAY2000	
		Clearance (Day -4)	05MAY2000	Yes	05MAY2000	
		Clearance (Day -3)	06MAY2000	Yes	06MAY2000	
		Clearance (Unsch.)	04MAY2000	No		
		Load Dose 1	09MAY2000	Yes	09MAY2000	12:55

*Popn=Population (I=Intent-to-Treat, PP=Per Protocol)
Tafenoquine = Tafenoquine 200mg, Mefloquine = Mefloquine 250mg

SB252263 - 030

Listing B.02

Visit And Dosing Dates

Treatment : Mefloquine

Subject Number	Popn*	Visit	Visit Date	Dose Taken ?	Date of Dose	Time of Dose
030.001.00198	IPP	Load Dose 2	10MAY2000	Yes	10MAY2000	8:01
		Load Dose 3	11MAY2000	Yes	11MAY2000	6:59
		Week 1	17MAY2000	Yes	17MAY2000	6:30
		Week 1 (Unsch.)	18MAY2000	No		
		Week 2	24MAY2000	Yes	24MAY2000	7:14
		Week 3	31MAY2000	Yes	31MAY2000	7:51
		Week 4	06JUN2000	Yes	06JUN2000	13:00
		Week 5	14JUN2000	Yes	14JUN2000	9:15
		Week 6 (Unsch.)	18JUN2000	No		
		Week 6	21JUN2000	Yes	21JUN2000	9:00
		Week 7	28JUN2000	Yes	28JUN2000	8:20
		Week 7 (Unsch. Fol. Up)	29JUN2000	No		
		Week 8 (Fol. Up)	04JUL2000	No		
		Week 9 (Unsch. Fol. Up)	10JUL2000	No		
		Week 9 (Fol. Up)	12JUL2000	No		
		Week 10 (Fol. Up)	19JUL2000	No		
		Week 11 (Unsch. Fol. Up)	26JUL2000	No		
030.001.00199	IPP	Screening	10APR2000	No		
		Clearance (Day -5)	04MAY2000	Yes	04MAY2000	
		Clearance (Day -4)	05MAY2000	Yes	05MAY2000	
		Clearance (Day -3)	06MAY2000	Yes	06MAY2000	
		Load Dose 1	09MAY2000	Yes	09MAY2000	12:40
		Load Dose 2	10MAY2000	Yes	10MAY2000	11:53
		Load Dose 3	11MAY2000	Yes	11MAY2000	9:18
		Week 1	17MAY2000	Yes	17MAY2000	7:03
		Week 2	24MAY2000	Yes	24MAY2000	6:50
		Week 2 (Unsch.)	24MAY2000	No		
		Week 3	30MAY2000	Yes	30MAY2000	14:06
		Week 4	06JUN2000	Yes	06JUN2000	
		Week 5	14JUN2000	Yes	14JUN2000	7:05
		Week 6	21JUN2000	Yes	21JUN2000	7:01
		Week 7 (Fol. Up)	28JUN2000	No		
		Week 8 (Fol. Up)	04JUL2000	No		
		Week 8 (Unsch. Fol. Up)	04JUL2000	No		
		Week 9 (Fol. Up)	12JUL2000	No		
		Week 10 (Fol. Up)	19JUL2000	No		
030.001.00200	IPP	Screening	17APR2000	No		
		Clearance (Day -5)	04MAY2000	Yes	04MAY2000	
		Clearance (Day -4)	05MAY2000	Yes	05MAY2000	
		Clearance (Day -3)	06MAY2000	Yes	06MAY2000	

*Popn=Population (I=Intent-to-Treat, PP=Per Protocol)
Tafenoquine = Tafenoquine 200mg, Mefloquine = Mefloquine 250mg

SB252263 - 030

Listing B.02

Visit And Dosing Dates

Treatment : Mefloquine

Subject Number	Popn*	Visit	Visit Date	Dose Taken ?	Date of Dose	Time of Dose
030.001.00200	IPP	Load Dose 1	09MAY2000	Yes	09MAY2000	12:43
		Load Dose 2	10MAY2000	Yes	10MAY2000	10:22
		Load Dose 3	11MAY2000	Yes	11MAY2000	9:08
		Week 1	17MAY2000	Yes	17MAY2000	8:16
		Week 2	24MAY2000	Yes	24MAY2000	7:50
		Week 3	31MAY2000	Yes	31MAY2000	8:03
		Week 4 (Fol. Up)	06JUN2000	No		
		Week 5 (Fol. Up)	14JUN2000	No		
		Week 6 (Fol. Up)	21JUN2000	No		
		Week 7 (Fol. Up)	28JUN2000	No		
030.001.00206	I	Screening	10APR2000	No		
		Screening (Unsch.)	26APR2000	No		
		Clearance (Day -5)	04MAY2000	Yes	04MAY2000	
		Clearance (Day -4)	05MAY2000	Yes	05MAY2000	
		Clearance (Day -3)	06MAY2000	Yes	06MAY2000	
		Load Dose 1	09MAY2000	Yes	09MAY2000	13:55
		Load Dose 2	10MAY2000	Yes	10MAY2000	9:37
		Load Dose 3	11MAY2000	Yes	11MAY2000	8:16
		Week 1	17MAY2000	Yes	17MAY2000	8:00
		Week 2 (Fol. Up)	24MAY2000	No		
		Week 3 (Fol. Up)	31MAY2000	No		
030.001.00213	IPP	Screening	14APR2000	No		
		Clearance (Day -5)	04MAY2000	Yes	04MAY2000	
		Clearance (Day -4)	05MAY2000	Yes	05MAY2000	
		Clearance (Day -3)	06MAY2000	Yes	06MAY2000	
		Load Dose 1	09MAY2000	Yes	09MAY2000	14:39
		Load Dose 2	10MAY2000	Yes	10MAY2000	12:20
		Load Dose 3	11MAY2000	Yes	11MAY2000	10:00
		Week 1	17MAY2000	Yes	17MAY2000	10:20
		Week 2	24MAY2000	Yes	24MAY2000	11:20
		Week 3	31MAY2000	Yes	31MAY2000	16:30
		Week 3 (Unsch.)	01JUN2000	No		
		Week 4	06JUN2000	Yes	06JUN2000	13:25
		Week 5	14JUN2000	Yes	14JUN2000	10:00
		Week 6	21JUN2000	Yes	21JUN2000	13:45
		Week 6 (Unsch.)	22JUN2000	No		
		Week 7	28JUN2000	Yes	28JUN2000	11:25
		Week 8	04JUL2000	Yes	04JUL2000	10:45
		Week 9	11JUL2000	Yes	11JUL2000	14:21
		Week 10	19JUL2000	Yes	19JUL2000	17:29

*Popn=Population (I=Intent-to-Treat, PP=Per Protocol)
Tafenoquine = Tafenoquine 200mg, Mefloquine = Mefloquine 250mg

SB252263 - 030

Listing B.02

Visit And Dosing Dates

Treatment : Mefloquine

Subject Number	Popn*	Visit	Visit Date	Dose Taken ?	Date of Dose	Time of Dose
030.001.00213	IPP	Week 11	25JUL2000	Yes	25JUL2000	16:09
		Week 12	01AUG2000	Yes	01AUG2000	16:30
		Week 14 (Fol. Up)	17AUG2000	No		
		Week 14 (Unsch. Fol. Up)	17AUG2000	No		
		Week 15 (Fol. Up)	22AUG2000	No		
		Week 15 (Unsch. Fol. Up)	24AUG2000	No		
030.001.00214	IPP	Screening	13APR2000	No		
		Clearance (Day -5)	04MAY2000	Yes	04MAY2000	
		Clearance (Day -4)	05MAY2000	Yes	05MAY2000	
		Clearance (Day -3)	06MAY2000	Yes	06MAY2000	
		Load Dose 1	09MAY2000	Yes	09MAY2000	15:21
		Load Dose 2	10MAY2000	Yes	10MAY2000	10:05
		Load Dose 3	11MAY2000	Yes	11MAY2000	7:55
		Week 1	17MAY2000	Yes	17MAY2000	8:22
		Week 2	24MAY2000	Yes	24MAY2000	8:16
		Week 3	31MAY2000	Yes	31MAY2000	7:11
		Week 4	07JUN2000	Yes	07JUN2000	17:38
		Week 5 (Unsch.)	11JUN2000	No		
		Week 5	14JUN2000	Yes	14JUN2000	8:35
		Week 6	21JUN2000	Yes	21JUN2000	7:00
		Week 7 (Fol. Up)	28JUN2000	No		
		Week 8 (Fol. Up)	04JUL2000	No		
		Week 9 (Fol. Up)	12JUL2000	No		
030.001.00216	IPP	Screening	11APR2000	No		
		Clearance (Day -5)	04MAY2000	Yes	04MAY2000	
		Clearance (Day -4)	05MAY2000	Yes	05MAY2000	
		Clearance (Day -3)	06MAY2000	Yes	06MAY2000	
		Load Dose 1	09MAY2000	Yes	09MAY2000	15:11
		Load Dose 2	10MAY2000	Yes	10MAY2000	10:00
		Load Dose 3	11MAY2000	Yes	11MAY2000	9:00
		Week 1	17MAY2000	Yes	17MAY2000	7:46
		Week 2	24MAY2000	Yes	24MAY2000	6:20
		Week 3	31MAY2000	Yes	31MAY2000	6:20
		Week 4	06JUN2000	Yes	06JUN2000	14:16
		Week 5 (Unsch.)	10JUN2000	No		
		Week 5 (Unsch.)	13JUN2000	No		
		Week 5	14JUN2000	Yes	14JUN2000	6:30
		Week 6	21JUN2000	Yes	21JUN2000	7:21
		Week 7	28JUN2000	Yes	28JUN2000	15:09
		Week 9	09JUL2000	Yes	09JUL2000	14:05

*Popn=Population (I=Intent-to-Treat, PP=Per Protocol)
Tafenoquine = Tafenoquine 200mg, Mefloquine = Mefloquine 250mg

Listing B.02

Visit And Dosing Dates

Treatment : Mefloquine

Subject Number	Popn*	Visit	Visit Date	Dose Taken ?	Date of Dose	Time of Dose
030.001.00216	IPP	Week 10	16JUL2000	Yes	16JUL2000	13:24
		Week 10	17JUL2000	No		
		Week 10	20JUL2000	Yes	20JUL2000	16:15
		Week 11 (Fol. Up)	26JUL2000	No		
		Week 12 (Fol. Up)	02AUG2000	No		
		Week 13 (Fol. Up)	10AUG2000	No		
		Week 14 (Fol. Up)	16AUG2000	No		
		Week 14 (Unsch. Fol. Up)	16AUG2000	No		
030.001.00221	IPP	Screening	17APR2000	No		
		Clearance (Day -5)	04MAY2000	Yes	04MAY2000	
		Clearance (Day -4)	05MAY2000	Yes	05MAY2000	
		Clearance (Day -3)	06MAY2000	Yes	06MAY2000	
		Load Dose 1	09MAY2000	Yes	09MAY2000	15:45
		Load Dose 2	10MAY2000	Yes	10MAY2000	10:35
		Load Dose 3	11MAY2000	Yes	11MAY2000	12:11
		Week 1	17MAY2000	Yes	17MAY2000	12:36
		Week 2	25MAY2000	Yes	25MAY2000	13:40
		Week 2 (Unsch.)	25MAY2000	No		
		Week 3	31MAY2000	Yes	31MAY2000	6:25
		Week 4 (Unsch.)	03JUN2000	No		
		Week 4	06JUN2000	Yes	06JUN2000	15:05
		Week 5	16JUN2000	Yes	16JUN2000	10:58
		Week 6 (Unsch.)	17JUN2000	No		
		Week 6	21JUN2000	Yes	21JUN2000	7:39
		Week 7	28JUN2000	Yes	28JUN2000	8:23
		Week 8	04JUL2000	Yes	04JUL2000	8:28
		Week 8	05JUL2000	No		
		Week 9	12JUL2000	Yes	12JUL2000	7:15
		Week 9 (Unsch.)	13JUL2000	No		
		Week 10	20JUL2000	Yes	20JUL2000	10:15
		Week 11 (Fol. Up)	27JUL2000	No		
		Week 12 (Fol. Up)	03AUG2000	No		
		Week 13 (Unsch. Fol. Up)	07AUG2000	No		
		Week 13 (Fol. Up)	09AUG2000	No		
		Week 14 (Unsch. Fol. Up)	15AUG2000	No		
		Week 14 (Fol. Up)	18AUG2000	No		
030.001.00223	IPP	Screening	14APR2000	No		
		Clearance (Day -5)	04MAY2000	Yes	04MAY2000	
		Clearance (Day -4)	05MAY2000	Yes	05MAY2000	
		Clearance (Day -3)	06MAY2000	Yes	06MAY2000	

*Popn=Population (I=Intent-to-Treat, PP=Per Protocol)
Tafenoquine = Tafenoquine 200mg, Mefloquine = Mefloquine 250mg

Listing B.02

Visit And Dosing Dates

Treatment : Mefloquine

Subject Number	Popn*	Visit	Visit Date	Dose Taken ?	Date of Dose	Time of Dose
030.001.00223	IPP	Load Dose 1	09MAY2000	Yes	09MAY2000	16:15
		Load Dose 2	10MAY2000	Yes	10MAY2000	7:22
		Load Dose 3	11MAY2000	Yes	11MAY2000	6:47
		Week 1	17MAY2000	Yes	17MAY2000	6:50
		Week 2	24MAY2000	Yes	24MAY2000	6:24
		Week 3	31MAY2000	Yes	31MAY2000	6:12
		Week 4	06JUN2000	Yes	06JUN2000	13:10
		Week 4 (Unsch.)	07JUN2000	No		
		Week 5	14JUN2000	Yes	14JUN2000	6:40
		Week 6 (Unsch. Fol. Up)	18JUN2000	No		
		Week 6 (Fol. Up)	21JUN2000	No		
		Week 7 (Unsch. Fol. Up)	24JUN2000	No		
		Week 7 (Fol. Up)	28JUN2000	No		
		Week 8 (Fol. Up)	03JUL2000	No		
		Week 9 (Fol. Up)	10JUL2000	No		
030.001.00224	IPP	Screening	12APR2000	No		
		Clearance (Day -5)	04MAY2000	Yes	04MAY2000	
		Clearance (Day -4)	05MAY2000	Yes	05MAY2000	
		Clearance (Day -3)	06MAY2000	Yes	06MAY2000	
		Load Dose 1	09MAY2000	Yes	09MAY2000	16:01
		Load Dose 2	10MAY2000	Yes	10MAY2000	7:22
		Load Dose 3	11MAY2000	Yes	11MAY2000	6:35
		Week 1	17MAY2000	Yes	17MAY2000	6:39
		Week 2	24MAY2000	Yes	24MAY2000	6:13
		Week 3	31MAY2000	Yes	31MAY2000	6:05
		Week 4	06JUN2000	Yes	06JUN2000	14:29
		Week 5 (Fol. Up)	14JUN2000	No		
		Week 6 (Fol. Up)	21JUN2000	No		
		Week 7 (Unsch. Fol. Up)	25JUN2000	No		
		Week 7 (Fol. Up)	28JUN2000	No		
030.001.00227	IPP	Screening	14APR2000	No		
		Clearance (Day -5)	04MAY2000	Yes	04MAY2000	
		Clearance (Day -4)	05MAY2000	Yes	05MAY2000	
		Clearance (Day -3)	06MAY2000	Yes	06MAY2000	
		Clearance (Unsch.)	08MAY2000	No		
		Load Dose 1	09MAY2000	Yes	09MAY2000	17:27
		Load Dose 2	10MAY2000	Yes	10MAY2000	11:52
		Load Dose 3	11MAY2000	Yes	11MAY2000	11:12
		Week 1	17MAY2000	Yes	17MAY2000	6:30
		Week 2	24MAY2000	Yes	24MAY2000	10:07

*Popn=Population (I=Intent-to-Treat, PP=Per Protocol)
Tafenoquine = Tafenoquine 200mg, Mefloquine = Mefloquine 250mg

SB252263 - 030

Listing B.02

Visit And Dosing Dates

Treatment : Mefloquine

Subject Number	Popn*	Visit	Visit Date	Dose Taken ?	Date of Dose	Time of Dose
030.001.00227	IPP	Week 3	31MAY2000	Yes	31MAY2000	6:20
		Week 4	07JUN2000	Yes	07JUN2000	13:16
		Week 5	15JUN2000	Yes	15JUN2000	8:55
		Week 6	21JUN2000	Yes	21JUN2000	15:52
		Week 6 (Unsch.)	21JUN2000	No		
		Week 6 (Unsch. Fol. Up)	22JUN2000	No		
		Week 7 (Fol. Up)	28JUN2000	No		
		Week 7 (Unsch. Fol. Up)	29JUN2000	No		
		Week 8 (Unsch. Fol. Up)	03JUL2000	No		
		Week 8 (Fol. Up)	04JUL2000	No		
		Week 9 (Unsch. Fol. Up)	10JUL2000	No		
		Week 9 (Unsch. Fol. Up)	11JUL2000	No		
		Week 9 (Fol. Up)	12JUL2000	No		
		Week 9 (Unsch. Fol. Up)	13JUL2000	No		
		Week 10 (Fol. Up)	20JUL2000	No		
030.001.00232	IPP	Screening	18APR2000	No		
		Clearance (Day -5)	05MAY2000	Yes	05MAY2000	
		Clearance (Day -4)	06MAY2000	Yes	06MAY2000	
		Clearance (Day -3)	07MAY2000	Yes	07MAY2000	
		Load Dose 1	10MAY2000	Yes	10MAY2000	14:50
		Load Dose 2	11MAY2000	Yes	11MAY2000	6:10
		Load Dose 3	12MAY2000	Yes	12MAY2000	6:35
		Week 1 (Unsch.)	15MAY2000	No		
		Week 1	17MAY2000	Yes	17MAY2000	6:35
		Week 2	24MAY2000	Yes	24MAY2000	6:10
		Week 3 (Unsch. Fol. Up)	29MAY2000	No		
		Week 3 (Fol. Up)	31MAY2000	No		
		Week 3 (Unsch. Fol. Up)	03JUN2000	No		
		Week 4 (Unsch. Fol. Up)	05JUN2000	No		
		Week 4 (Fol. Up)	07JUN2000	No		
		Week 5 (Fol. Up)	14JUN2000	No		
		Week 6 (Fol. Up)	21JUN2000	No		
030.001.00235	IPP	Screening	10APR2000	No		
		Screening (Unsch.)	02MAY2000	No		
		Clearance (Day -5)	05MAY2000	Yes	05MAY2000	
		Clearance (Day -4)	06MAY2000	Yes	06MAY2000	
		Clearance (Day -3)	07MAY2000	Yes	07MAY2000	
		Clearance (Unsch.)	06MAY2000	No		
		Load Dose 1	10MAY2000	Yes	10MAY2000	8:15
		Load Dose 2	11MAY2000	Yes	11MAY2000	8:00

*Popn=Population (I=Intent-to-Treat, PP=Per Protocol)
Tafenoquine = Tafenoquine 200mg, Mefloquine = Mefloquine 250mg

SB252263 - 030

Listing B.02

Visit And Dosing Dates

Treatment : Mefloquine

Subject Number	Popn*	Visit	Visit Date	Dose Taken ?	Date of Dose	Time of Dose
030.001.00235	IPP	Load Dose 3	12MAY2000	Yes	12MAY2000	6:40
		Week 1	17MAY2000	Yes	17MAY2000	7:00
		Week 2	24MAY2000	Yes	24MAY2000	7:40
		Week 2 (Unsch.)	24MAY2000	No		
		Week 3	31MAY2000	Yes	31MAY2000	7:00
		Week 4	08JUN2000	Yes	08JUN2000	
		Week 5	14JUN2000	Yes	14JUN2000	8:35
		Week 5 (Unsch.)	14JUN2000	No		
		Week 6 (Fol. Up)	21JUN2000	No		
		Week 7 (Fol. Up)	29JUN2000	No		
		Week 8 (Fol. Up)	04JUL2000	No		
		Week 8 (Fol. Up)	05JUL2000	No		
030.001.00237	IPP	Screening	17APR2000	No		
		Clearance (Day -5)	05MAY2000	Yes	05MAY2000	
		Clearance (Day -4)	06MAY2000	Yes	06MAY2000	
		Clearance (Day -3)	07MAY2000	Yes	07MAY2000	
		Load Dose 1	10MAY2000	Yes	10MAY2000	7:34
		Load Dose 2	12MAY2000	Yes	12MAY2000	18:56
		Load Dose 3	13MAY2000	Yes	13MAY2000	8:30
		Week 1	17MAY2000	Yes	17MAY2000	6:51
		Week 2	24MAY2000	Yes	24MAY2000	19:50
		Week 3	31MAY2000	Yes	31MAY2000	6:32
		Week 4	06JUN2000	Yes	06JUN2000	15:10
		Week 5 (Fol. Up)	13JUN2000	No		
		Week 5 (Unsch. Fol. Up)	13JUN2000	No		
		Week 5 (Unsch. Fol. Up)	14JUN2000	No		
		Week 5 (Unsch. Fol. Up)	16JUN2000	No		
		Week 6 (Fol. Up)	22JUN2000	No		
		Week 7 (Fol. Up)	29JUN2000	No		
		Week 8 (Fol. Up)	05JUL2000	No		
030.001.00239	IPP	Screening	10APR2000	No		
		Clearance (Day -5)	05MAY2000	Yes	05MAY2000	
		Clearance (Day -4)	06MAY2000	Yes	06MAY2000	
		Clearance (Day -3)	07MAY2000	Yes	07MAY2000	
		Load Dose 1	10MAY2000	Yes	10MAY2000	7:54
		Load Dose 2	11MAY2000	Yes	11MAY2000	7:54
		Load Dose 3	12MAY2000	Yes	12MAY2000	6:25
		Week 1	17MAY2000	Yes	17MAY2000	12:58
		Week 2	24MAY2000	Yes	24MAY2000	1:10
		Week 3	30MAY2000	Yes	30MAY2000	10:55

*Popn=Population (I=Intent-to-Treat, PP=Per Protocol)
Tafenoquine = Tafenoquine 200mg, Mefloquine = Mefloquine 250mg

SB252263 - 030

Listing B.02

Visit And Dosing Dates

Treatment : Mefloquine

Subject Number	Popn*	Visit	Visit Date	Dose Taken ?	Date of Dose	Time of Dose
030.001.00239	IPP	Week 4 (Unsch.)	05JUN2000	No		
		Week 4	06JUN2000	Yes	06JUN2000	17:30
		Week 5	14JUN2000	Yes	14JUN2000	6:55
		Week 5 (Unsch. Fol. Up)	17JUN2000	No		
		Week 6 (Fol. Up)	21JUN2000	No		
		Week 7 (Fol. Up)	28JUN2000	No		
		Week 7 (Unsch. Fol. Up)	30JUN2000	No		
		Week 8 (Fol. Up)	04JUL2000	No		
		Week 9 (Unsch. Fol. Up)	11JUL2000	No		
		Week 9 (Fol. Up)	12JUL2000	No		
		Week 9 (Unsch. Fol. Up)	12JUL2000	No		
030.001.00241	IPP	Screening	13APR2000	No		
		Clearance (Day -5)	05MAY2000	Yes	05MAY2000	
		Clearance (Day -4)	06MAY2000	Yes	06MAY2000	
		Clearance (Day -3)	07MAY2000	Yes	07MAY2000	
		Load Dose 1	10MAY2000	Yes	10MAY2000	8:00
		Load Dose 2	11MAY2000	Yes	11MAY2000	11:00
		Load Dose 3	12MAY2000	Yes	12MAY2000	10:30
		Week 1	17MAY2000	Yes	17MAY2000	10:00
		Week 2	24MAY2000	Yes	24MAY2000	7:00
		Week 2 (Unsch.)	24MAY2000	No		
		Week 3 (Unsch.)	29MAY2000	No		
		Week 3	01JUN2000	Yes	01JUN2000	8:00
		Week 4	06JUN2000	Yes	06JUN2000	13:08
		Week 5	12JUN2000	Yes	12JUN2000	16:50
		Week 6	20JUN2000	Yes	20JUN2000	16:16
		Week 7	28JUN2000	Yes	28JUN2000	8:35
		Week 8	04JUL2000	Yes	04JUL2000	6:37
		Week 9	12JUL2000	Yes	12JUL2000	6:43
		Week 10	19JUL2000	Yes	19JUL2000	7:03
		Week 10 (Unsch.)	20JUL2000	No		
		Week 11 (Unsch.)	24JUL2000	No		
		Week 11	26JUL2000	Yes	26JUL2000	5:45
		Week 12	03AUG2000	Yes	03AUG2000	10:55
		Week 13	11AUG2000	Yes	11AUG2000	13:45
		Week 14	16AUG2000	Yes	16AUG2000	7:30
		Week 15 (Unsch.)	21AUG2000	No		
		Week 15	23AUG2000	Yes	23AUG2000	15:00
		Week 16	29AUG2000	Yes	29AUG2000	9:00
		Week 16 (Unsch.)	01SEP2000	No		
		Week 16	02SEP2000	No		
		Week 17	06SEP2000	Yes	06SEP2000	10:23

*Popn=Population (I=Intent-to-Treat, PP=Per Protocol)
Tafenoquine = Tafenoquine 200mg, Mefloquine = Mefloquine 250mg

SB252263 - 030

Listing B.02

Visit And Dosing Dates

Treatment : Mefloquine

Subject Number	Popn*	Visit	Visit Date	Dose Taken ?	Date of Dose	Time of Dose
030.001.00241	IPP	Week 18	13SEP2000	Yes	13SEP2000	13:05
		Week 19	21SEP2000	Yes	21SEP2000	10:16
		Week 20	27SEP2000	Yes	27SEP2000	14:53
		Week 21	05OCT2000	Yes	05OCT2000	10:25
		Week 22	11OCT2000	Yes	11OCT2000	11:07
		Week 23	17OCT2000	Yes	17OCT2000	10:02
		Week 24	24OCT2000	Yes	24OCT2000	10:46
		Week 25 (Fol. Up)	02NOV2000	No		
		Week 28 (Fol. Up)	23NOV2000	No		
030.001.00244	IPP	Screening	13APR2000	No		
		Clearance (Day -5)	05MAY2000	Yes	05MAY2000	
		Clearance (Day -4)	06MAY2000	Yes	06MAY2000	
		Clearance (Day -3)	07MAY2000	Yes	07MAY2000	
		Load Dose 1	10MAY2000	Yes	10MAY2000	8:30
		Load Dose 2	11MAY2000	Yes	11MAY2000	6:20
		Load Dose 3	12MAY2000	Yes	12MAY2000	6:20
		Week 1	17MAY2000	Yes	17MAY2000	6:10
		Week 1 (Unsch.)	19MAY2000	No		
		Week 2	24MAY2000	Yes	24MAY2000	8:10
		Week 3	31MAY2000	Yes	31MAY2000	8:00
		Week 4 (Unsch.)	05JUN2000	No		
		Week 4	06JUN2000	Yes	06JUN2000	11:04
		Week 5	14JUN2000	Yes	14JUN2000	8:20
		Week 6	21JUN2000	Yes	21JUN2000	8:02
		Week 7	28JUN2000	Yes	28JUN2000	8:34
		Week 8	04JUL2000	Yes	04JUL2000	7:50
		Week 9	12JUL2000	Yes	12JUL2000	7:40
		Week 10	19JUL2000	Yes	19JUL2000	10:25
		Week 11	26JUL2000	Yes	26JUL2000	8:25
		Week 12	02AUG2000	Yes	02AUG2000	7:45
		Week 12 (Unsch. Fol. Up)	05AUG2000	No		
		Week 13 (Fol. Up)	09AUG2000	No		
		Week 13 (Unsch. Fol. Up)	10AUG2000	No		
		Week 14 (Fol. Up)	18AUG2000	No		
		Week 15 (Fol. Up)	23AUG2000	No		
030.001.00245	IPP	Screening	11APR2000	No		
		Clearance (Day -5)	05MAY2000	Yes	05MAY2000	
		Clearance (Day -4)	06MAY2000	Yes	06MAY2000	
		Clearance (Day -3)	07MAY2000	Yes	07MAY2000	
		Load Dose 1	10MAY2000	Yes	10MAY2000	8:35

*Popn=Population (I=Intent-to-Treat, PP=Per Protocol)
Tafenoquine = Tafenoquine 200mg, Mefloquine = Mefloquine 250mg

SB252263 - 030

Listing B.02

Visit And Dosing Dates

Treatment : Mefloquine

Subject Number	Popn*	Visit	Visit Date	Dose Taken ?	Date of Dose	Time of Dose
030.001.00245	IPP	Load Dose 2	11MAY2000	Yes	11MAY2000	10:10
		Load Dose 3	12MAY2000	Yes	12MAY2000	8:58
		Week 1	17MAY2000	Yes	17MAY2000	10:30
		Week 2	24MAY2000	Yes	24MAY2000	8:31
		Week 3	31MAY2000	Yes	31MAY2000	8:35
		Week 4	07JUN2000	Yes	07JUN2000	7:27
		Week 5	14JUN2000	Yes	14JUN2000	7:30
		Week 6	21JUN2000	Yes	21JUN2000	7:42
		Week 7	28JUN2000	Yes	28JUN2000	6:55
		Week 8	04JUL2000	Yes	04JUL2000	9:35
		Week 8	05JUL2000	No		
		Week 9	12JUL2000	Yes	12JUL2000	7:00
		Week 9 (Unsch.)	13JUL2000	No		
		Week 10	19JUL2000	Yes	19JUL2000	7:00
		Week 11	26JUL2000	Yes	26JUL2000	7:10
		Week 12 (Fol. Up)	02AUG2000	No		
		Week 13 (Fol. Up)	10AUG2000	No		
		Week 14 (Fol. Up)	16AUG2000	No		
030.001.00250		Screening	18APR2000	No		
		Clearance (Day -5)	05MAY2000	Yes	05MAY2000	
		Clearance (Day -4)	06MAY2000	Yes	06MAY2000	
		Clearance (Day -3)	07MAY2000	Yes	07MAY2000	
		Load Dose 1	10MAY2000	Yes	10MAY2000	8:53
		Load Dose 2	11MAY2000	Yes	11MAY2000	7:15
		Week 1 (Fol. Up)	12MAY2000	No		
		Week 1 (Fol. Up)	19MAY2000	No		
		Week 2 (Fol. Up)	24MAY2000	No		
		Week 2 (Unsch. Fol. Up)	24MAY2000	No		
		Week 3 (Fol. Up)	31MAY2000	No		
		Week 4 (Fol. Up)	06JUN2000	No		
		Week 4 (Unsch. Fol. Up)	07JUN2000	No		
030.001.00255	IPP	Screening	12APR2000	No		
		Clearance (Day -5)	05MAY2000	Yes	05MAY2000	
		Clearance (Day -4)	06MAY2000	Yes	06MAY2000	
		Clearance (Day -3)	07MAY2000	Yes	07MAY2000	
		Load Dose 1	10MAY2000	Yes	10MAY2000	9:21
		Load Dose 2	11MAY2000	Yes	11MAY2000	6:45
		Load Dose 3	12MAY2000	Yes	12MAY2000	8:26
		Week 1	17MAY2000	Yes	17MAY2000	10:00
		Week 2	24MAY2000	Yes	24MAY2000	6:48

*Popn=Population (I=Intent-to-Treat, PP=Per Protocol)
Tafenoquine = Tafenoquine 200mg, Mefloquine = Mefloquine 250mg

SB252263 - 030

Listing B.02

Visit And Dosing Dates

Treatment : Mefloquine

Subject Number	Popn*	Visit	Visit Date	Dose Taken ?	Date of Dose	Time of Dose
030.001.00255	IPP	Week 3	31MAY2000	Yes	31MAY2000	8:12
		Week 4	06JUN2000	Yes	06JUN2000	10:45
		Week 5	14JUN2000	Yes	14JUN2000	16:17
		Week 6	21JUN2000	Yes	21JUN2000	8:33
		Week 7	28JUN2000	Yes	28JUN2000	9:08
		Week 8	04JUL2000	Yes	04JUL2000	6:10
		Week 9	12JUL2000	Yes	12JUL2000	6:28
		Week 10	19JUL2000	Yes	19JUL2000	6:19
		Week 11 (Fol. Up)	26JUL2000	No		
		Week 12 (Fol. Up)	02AUG2000	No		
		Week 13 (Fol. Up)	09AUG2000	No		
030.001.00258	IPP	Screening	14APR2000	No		
		Clearance (Day -5)	05MAY2000	Yes	05MAY2000	
		Clearance (Day -4)	06MAY2000	Yes	06MAY2000	
		Clearance (Day -3)	07MAY2000	Yes	07MAY2000	
		Load Dose 1	10MAY2000	Yes	10MAY2000	9:50
		Load Dose 2	11MAY2000	Yes	11MAY2000	10:45
		Load Dose 3	12MAY2000	Yes	12MAY2000	12:58
		Week 1 (Unsch.)	15MAY2000	No		
		Week 1	17MAY2000	Yes	17MAY2000	9:40
		Week 2	25MAY2000	Yes	25MAY2000	15:07
		Week 3	31MAY2000	Yes	31MAY2000	8:55
		Week 4	06JUN2000	Yes	06JUN2000	15:30
		Week 5	14JUN2000	Yes	14JUN2000	8:30
		Week 6	21JUN2000	Yes	21JUN2000	11:17
		Week 7	28JUN2000	Yes	28JUN2000	8:46
		Week 8	04JUL2000	Yes	04JUL2000	9:11
		Week 9	12JUL2000	Yes	12JUL2000	9:15
		Week 10	19JUL2000	Yes	19JUL2000	8:30
		Week 11 (Fol. Up)	26JUL2000	No		
		Week 12 (Unsch. Fol. Up)	31JUL2000	No		
		Week 12 (Fol. Up)	02AUG2000	No		
		Week 14 (Fol. Up)	16AUG2000	No		
		Week 14 (Unsch. Fol. Up)	17AUG2000	No		
030.001.00262	IPP	Screening	13APR2000	No		
		Clearance (Day -5)	05MAY2000	Yes	05MAY2000	
		Clearance (Day -4)	06MAY2000	Yes	06MAY2000	
		Clearance (Day -3)	07MAY2000	Yes	07MAY2000	
		Load Dose 1	10MAY2000	Yes	10MAY2000	10:21
		Load Dose 2	11MAY2000	Yes	11MAY2000	7:50

*Popn=Population (I=Intent-to-Treat, PP=Per Protocol)
Tafenoquine = Tafenoquine 200mg, Mefloquine = Mefloquine 250mg

SB252263 - 030

Listing B.02

Visit And Dosing Dates

Treatment : Mefloquine

Subject Number	Popn*	Visit	Visit Date	Dose Taken ?	Date of Dose	Time of Dose
030.001.00262	IPP	Load Dose 3	12MAY2000	Yes	12MAY2000	6:39
		Week 1	17MAY2000	Yes	17MAY2000	7:28
		Week 2	24MAY2000	Yes	24MAY2000	7:37
		Week 3	01JUN2000	Yes	01JUN2000	10:49
		Week 4	06JUN2000	Yes	06JUN2000	11:50
		Week 5	14JUN2000	Yes	14JUN2000	6:58
		Week 6	23JUN2000	Yes	23JUN2000	12:05
		Week 7 (Fol. Up)	28JUN2000	No		
		Week 8 (Fol. Up)	05JUL2000	No		
		Week 9 (Fol. Up)	12JUL2000	No		
030.001.00263	IPP	Screening	17APR2000	No		
		Screening (Unsch.)	02MAY2000	No		
		Clearance (Day -5)	05MAY2000	Yes	05MAY2000	
		Clearance (Day -4)	06MAY2000	Yes	06MAY2000	
		Clearance (Day -3)	07MAY2000	Yes	07MAY2000	
		Load Dose 1	10MAY2000	No		
		Load Dose 1	12MAY2000	Yes	12MAY2000	11:21
		Load Dose 2	13MAY2000	Yes	13MAY2000	16:19
		Load Dose 3	14MAY2000	Yes	14MAY2000	9:15
		Week 1	18MAY2000	Yes	18MAY2000	7:43
		Week 2	24MAY2000	Yes	24MAY2000	8:43
		Week 3	30MAY2000	Yes	30MAY2000	9:16
		Week 4	07JUN2000	Yes	07JUN2000	15:04
		Week 4 (Unsch.)	09JUN2000	No		
		Week 5	14JUN2000	Yes	14JUN2000	7:02
		Week 6	22JUN2000	Yes	22JUN2000	11:30
		Week 6 (Unsch.)	23JUN2000	No		
		Week 6 (Unsch.)	24JUN2000	No		
		Week 7	28JUN2000	Yes	28JUN2000	6:32
		Week 7	03JUL2000	Yes	03JUL2000	8:11
		Week 9	12JUL2000	Yes	12JUL2000	5:45
		Week 10 (Unsch.)	18JUL2000	No		
		Week 10	19JUL2000	Yes	19JUL2000	10:30
		Week 11	26JUL2000	Yes	26JUL2000	8:00
		Week 12	02AUG2000	Yes	02AUG2000	7:20
		Week 12 (Unsch.)	04AUG2000	No		
		Week 13	09AUG2000	Yes	09AUG2000	9:42
		Week 13 (Unsch.)	11AUG2000	No		
		Week 14	16AUG2000	Yes	16AUG2000	7:42
		Week 15	23AUG2000	Yes	23AUG2000	7:21
		Week 15	28AUG2000	Yes	28AUG2000	8:18
		Week 16	30AUG2000	No		

*Popn=Population (I=Intent-to-Treat, PP=Per Protocol)
Tafenoquine = Tafenoquine 200mg, Mefloquine = Mefloquine 250mg

SB252263 - 030

Listing B.02

Visit And Dosing Dates

Treatment : Mefloquine

Subject Number	Popn*	Visit	Visit Date	Dose Taken ?	Date of Dose	Time of Dose
030.001.00263	IPP	Week 17	06SEP2000	Yes	06SEP2000	7:17
		Week 17 (Unsch.)	07SEP2000	No		
		Week 18 (Unsch.)	12SEP2000	No		
		Week 18	13SEP2000	Yes	13SEP2000	7:11
		Week 19	20SEP2000	Yes	20SEP2000	7:27
		Week 19 (Unsch.)	20SEP2000	No		
		Week 20	29SEP2000	Yes	29SEP2000	6:41
		Week 21	04OCT2000	Yes	04OCT2000	7:11
		Week 22	11OCT2000	Yes	11OCT2000	7:07
		Week 22 (Unsch.)	11OCT2000	No		
		Week 22	16OCT2000	Yes	16OCT2000	16:30
		Week 23	23OCT2000	Yes	23OCT2000	7:33
		Week 25 (Fol. Up)	01NOV2000	No		
		Week 26 (Fol. Up)	08NOV2000	No		
		Week 26 (Unsch. Fol. Up)	10NOV2000	No		
		Week 27 (Fol. Up)	15NOV2000	No		
		Week 27 (Fol. Up)	20NOV2000	No		
030.001.00265	IPP	Screening	12APR2000	No		
		Clearance (Day -5)	05MAY2000	Yes	05MAY2000	
		Clearance (Day -4)	06MAY2000	Yes	06MAY2000	
		Clearance (Day -3)	07MAY2000	Yes	07MAY2000	
		Load Dose 1	10MAY2000	Yes	10MAY2000	12:00
		Load Dose 2	11MAY2000	Yes	11MAY2000	6:35
		Load Dose 3	12MAY2000	Yes	12MAY2000	7:04
		Week 1	17MAY2000	Yes	17MAY2000	10:30
		Week 1 (Unsch.)	20MAY2000	No		
		Week 2	24MAY2000	Yes	24MAY2000	7:30
		Week 2 (Unsch.)	24MAY2000	No		
		Week 3	31MAY2000	Yes	31MAY2000	7:35
		Week 4	07JUN2000	Yes	07JUN2000	
		Week 5	14JUN2000	Yes	14JUN2000	7:20
		Week 5 (Unsch.)	15JUN2000	No		
		Week 6	21JUN2000	Yes	21JUN2000	7:17
		Week 7	28JUN2000	Yes	28JUN2000	6:12
		Week 8	03JUL2000	Yes	03JUL2000	8:35
		Week 9	12JUL2000	Yes	12JUL2000	7:48
		Week 9 (Unsch.)	13JUL2000	No		
		Week 10	19JUL2000	Yes	19JUL2000	7:17
		Week 11	26JUL2000	Yes	26JUL2000	8:56
		Week 11 (Unsch.)	26JUL2000	No		
		Week 12	02AUG2000	Yes	02AUG2000	6:56
		Week 14	13AUG2000	Yes	13AUG2000	9:16

*Popn=Population (I=Intent-to-Treat, PP=Per Protocol)
Tafenoquine = Tafenoquine 200mg, Mefloquine = Mefloquine 250mg

SB252263 - 030

Listing B.02

Visit And Dosing Dates

Treatment : Mefloquine

Subject Number	Popn*	Visit	Visit Date	Dose Taken ?	Date of Dose	Time of Dose
030.001.00265	IPP	Week 14	17AUG2000	Yes	17AUG2000	14:50
		Week 16	27AUG2000	Yes	27AUG2000	8:18
		Week 16 (Unsch.)	27AUG2000	No		
		Week 16	02SEP2000	Yes	02SEP2000	9:05
		Week 17	06SEP2000	Yes	06SEP2000	12:00
		Week 18	13SEP2000	Yes	13SEP2000	10:20
		Week 21 (Fol. Up)	01OCT2000	No		
030.001.00273	IPP	Screening	11APR2000	No		
		Clearance (Day -5)	05MAY2000	Yes	05MAY2000	
		Clearance (Day -4)	06MAY2000	Yes	06MAY2000	
		Clearance (Day -3)	07MAY2000	Yes	07MAY2000	
		Load Dose 1	10MAY2000	Yes	10MAY2000	16:37
		Load Dose 2	11MAY2000	Yes	11MAY2000	7:02
		Load Dose 3	12MAY2000	Yes	12MAY2000	6:43
		Week 1	17MAY2000	Yes	17MAY2000	7:23
		Week 2	24MAY2000	Yes	24MAY2000	7:31
		Week 3 (Unsch.)	29MAY2000	No		
		Week 3	31MAY2000	Yes	31MAY2000	7:20
		Week 4	06JUN2000	Yes	06JUN2000	10:00
		Week 5	14JUN2000	Yes	14JUN2000	6:35
		Week 6	21JUN2000	Yes	21JUN2000	6:33
		Week 7	28JUN2000	Yes	28JUN2000	8:13
		Week 8	04JUL2000	Yes	04JUL2000	6:53
		Week 9	12JUL2000	Yes	12JUL2000	6:58
		Week 10	19JUL2000	Yes	19JUL2000	7:58
		Week 11	26JUL2000	Yes	26JUL2000	8:09
		Week 12	01AUG2000	Yes	01AUG2000	12:30
		Week 12 (Unsch.)	01AUG2000	No		
		Week 13	09AUG2000	Yes	09AUG2000	9:20
		Week 14 (Fol. Up)	16AUG2000	No		
		Week 14 (Unsch. Fol. Up)	16AUG2000	No		
		Week 15 (Fol. Up)	23AUG2000	No		
		Week 16 (Fol. Up)	01SEP2000	No		
030.001.00274	IPP	Screening	11APR2000	No		
		Clearance (Day -5)	05MAY2000	Yes	05MAY2000	
		Clearance (Day -4)	06MAY2000	Yes	06MAY2000	
		Clearance (Day -3)	07MAY2000	Yes	07MAY2000	
		Clearance (Unsch.)	06MAY2000	No		
		Load Dose 1	10MAY2000	Yes	10MAY2000	16:45
		Load Dose 2	11MAY2000	Yes	11MAY2000	10:24

*Popn=Population (I=Intent-to-Treat, PP=Per Protocol)
Tafenoquine = Tafenoquine 200mg, Mefloquine = Mefloquine 250mg

SB252263 - 030

Listing B.02

Visit And Dosing Dates

Treatment : Mefloquine

Subject Number	Popn*	Visit	Visit Date	Dose Taken ?	Date of Dose	Time of Dose
030.001.00274	IPP	Load Dose 3	12MAY2000	Yes	12MAY2000	7:35
		Week 1 (Unsch.)	13MAY2000	No		
		Week 1	17MAY2000	Yes	17MAY2000	11:59
		Week 1 (Unsch.)	19MAY2000	No		
		Week 2	24MAY2000	Yes	24MAY2000	6:30
		Week 3	31MAY2000	Yes	31MAY2000	6:30
		Week 4	06JUN2000	Yes	06JUN2000	9:55
		Week 4 (Unsch.)	10JUN2000	No		
		Week 5	14JUN2000	Yes	14JUN2000	6:40
		Week 6	21JUN2000	Yes	21JUN2000	6:00
		Week 7	28JUN2000	Yes	28JUN2000	6:30
		Week 8 (Fol. Up)	04JUL2000	No		
		Week 9 (Fol. Up)	12JUL2000	No		
		Week 10 (Fol. Up)	19JUL2000	No		
		Week 11 (Fol. Up)	26JUL2000	No		
030.001.00276	IPP	Screening	12APR2000	No		
		Clearance (Day -5)	05MAY2000	Yes	05MAY2000	
		Clearance (Day -4)	06MAY2000	Yes	06MAY2000	
		Clearance (Day -3)	07MAY2000	Yes	07MAY2000	
		Load Dose 1	10MAY2000	Yes	10MAY2000	17:36
		Load Dose 2	11MAY2000	Yes	11MAY2000	6:05
		Load Dose 3	12MAY2000	Yes	12MAY2000	6:35
		Week 1	17MAY2000	Yes	17MAY2000	6:30
		Week 2	24MAY2000	Yes	24MAY2000	6:00
		Week 3	31MAY2000	Yes	31MAY2000	6:05
		Week 4	07JUN2000	Yes	07JUN2000	7:01
		Week 5	14JUN2000	Yes	14JUN2000	6:20
		Week 6	21JUN2000	Yes	21JUN2000	6:20
		Week 7 (Fol. Up)	28JUN2000	No		
		Week 7 (Unsch. Fol. Up)	29JUN2000	No		
		Week 8 (Fol. Up)	04JUL2000	No		
		Week 8 (Unsch. Fol. Up)	08JUL2000	No		
		Week 9 (Fol. Up)	12JUL2000	No		
030.001.00280	IPP	Screening	13APR2000	No		
		Clearance (Day -5)	05MAY2000	Yes	05MAY2000	
		Clearance (Day -4)	06MAY2000	Yes	06MAY2000	
		Clearance (Day -3)	07MAY2000	Yes	07MAY2000	
		Load Dose 1	10MAY2000	Yes	10MAY2000	18:35
		Load Dose 2	11MAY2000	Yes	11MAY2000	11:11
		Load Dose 3	12MAY2000	Yes	12MAY2000	10:35

*Popn=Population (I=Intent-to-Treat, PP=Per Protocol)
Tafenoquine = Tafenoquine 200mg, Mefloquine = Mefloquine 250mg

SB252263 - 030

Listing B.02

Visit And Dosing Dates

Treatment : Mefloquine

Subject Number	Popn*	Visit	Visit Date	Dose Taken ?	Date of Dose	Time of Dose
030.001.00280	IPP	Week 1	17MAY2000	Yes	17MAY2000	11:15
		Week 2	24MAY2000	Yes	24MAY2000	6:00
		Week 3	31MAY2000	Yes	31MAY2000	6:10
		Week 4	06JUN2000	Yes	06JUN2000	13:26
		Week 5	15JUN2000	Yes	15JUN2000	11:24
		Week 6	21JUN2000	Yes	21JUN2000	8:10
		Week 7 (Unsch.)	27JUN2000	No		
		Week 7	28JUN2000	Yes	28JUN2000	15:25
		Week 8 (Fol. Up)	05JUL2000	No		
		Week 9 (Fol. Up)	12JUL2000	No		
		Week 10 (Fol. Up)	21JUL2000	No		
030.001.00281	IPP	Screening	12APR2000	No		
		Clearance (Day -5)	05MAY2000	Yes	05MAY2000	
		Clearance (Day -4)	06MAY2000	Yes	06MAY2000	
		Clearance (Day -3)	07MAY2000	Yes	07MAY2000	
		Load Dose 1	10MAY2000	Yes	10MAY2000	18:30
		Load Dose 2	12MAY2000	Yes	12MAY2000	13:14
		Load Dose 3	13MAY2000	Yes	13MAY2000	14:08
		Week 1	17MAY2000	Yes	17MAY2000	12:54
		Week 2	24MAY2000	Yes	24MAY2000	8:00
		Week 3	31MAY2000	Yes	31MAY2000	8:28
		Week 4	07JUN2000	Yes	07JUN2000	17:07
		Week 5	14JUN2000	Yes	14JUN2000	8:47
		Week 6	21JUN2000	Yes	21JUN2000	9:10
		Week 6 (Unsch. Fol. Up)	23JUN2000	No		
		Week 7 (Unsch. Fol. Up)	26JUN2000	No		
		Week 7 (Fol. Up)	28JUN2000	No		
		Week 8 (Fol. Up)	03JUL2000	No		
		Week 9 (Fol. Up)	12JUL2000	No		
030.001.00283	IPP	Screening	14APR2000	No		
		Screening (Unsch.)	27APR2000	No		
		Clearance (Day -5)	05MAY2000	Yes	05MAY2000	
		Clearance (Day -4)	06MAY2000	Yes	06MAY2000	
		Clearance (Day -3)	07MAY2000	Yes	07MAY2000	
		Load Dose 1	11MAY2000	Yes	11MAY2000	12:31
		Load Dose 2	12MAY2000	Yes	12MAY2000	12:15
		Load Dose 3	13MAY2000	Yes	13MAY2000	7:30
		Week 1	18MAY2000	Yes	18MAY2000	6:10
		Week 2	24MAY2000	Yes	24MAY2000	7:15
		Week 3	31MAY2000	Yes	31MAY2000	7:00

*Popn=Population (I=Intent-to-Treat, PP=Per Protocol)
Tafenoquine = Tafenoquine 200mg, Mefloquine = Mefloquine 250mg

SB252263 - 030

Listing B.02

Visit And Dosing Dates

Treatment : Mefloquine

Subject Number	Popn*	Visit	Visit Date	Dose Taken ?	Date of Dose	Time of Dose
030.001.00283	IPP	Week 3 (Unsch.)	31MAY2000	No		
		Week 4	08JUN2000	Yes	08JUN2000	12:00
		Week 5	14JUN2000	Yes	14JUN2000	18:30
		Week 6	21JUN2000	Yes	21JUN2000	8:42
		Week 7	28JUN2000	Yes	28JUN2000	6:13
		Week 7 (Unsch.)	28JUN2000	No		
		Week 7 (Unsch.)	01JUL2000	No		
		Week 8	04JUL2000	Yes	04JUL2000	6:10
		Week 9	12JUL2000	Yes	12JUL2000	6:20
		Week 10 (Fol. Up)	19JUL2000	No		
		Week 11 (Fol. Up)	27JUL2000	No		
		Week 12 (Fol. Up)	02AUG2000	No		
		Week 13 (Unsch. Fol. Up)	07AUG2000	No		
030.001.00286	IPP	Screening	12APR2000	No		
		Clearance (Day -5)	05MAY2000	Yes	05MAY2000	
		Clearance (Day -4)	06MAY2000	Yes	06MAY2000	
		Clearance (Day -3)	07MAY2000	Yes	07MAY2000	
		Clearance (Unsch.)	08MAY2000	No		
		Load Dose 1	11MAY2000	Yes	11MAY2000	9:09
		Load Dose 2	12MAY2000	Yes	12MAY2000	7:01
		Load Dose 3	13MAY2000	Yes	13MAY2000	13:19
		Load Dose (Unsch.)	12MAY2000	No		
		Week 1	18MAY2000	Yes	18MAY2000	7:20
		Week 2 (Unsch.)	22MAY2000	No		
		Week 2	24MAY2000	Yes	24MAY2000	6:45
		Week 2 (Unsch.)	25MAY2000	No		
		Week 3 (Unsch.)	30MAY2000	No		
		Week 3	31MAY2000	Yes	31MAY2000	6:45
		Week 4 (Unsch.)	05JUN2000	No		
		Week 4	06JUN2000	Yes	06JUN2000	9:15
		Week 5	14JUN2000	Yes	14JUN2000	7:04
		Week 6	21JUN2000	Yes	21JUN2000	8:10
		Week 7	29JUN2000	Yes	29JUN2000	15:20
		Week 8	03JUL2000	Yes	03JUL2000	10:39
		Week 8	06JUL2000	No		
		Week 9	12JUL2000	Yes	12JUL2000	17:52
		Week 10	19JUL2000	Yes	19JUL2000	7:15
		Week 11	26JUL2000	Yes	26JUL2000	9:40
		Week 12 (Fol. Up)	02AUG2000	No		
		Week 13 (Unsch. Fol. Up)	07AUG2000	No		
		Week 13 (Fol. Up)	09AUG2000	No		
		Week 14 (Fol. Up)	16AUG2000	No		

*Popn=Population (I=Intent-to-Treat, PP=Per Protocol)
Tafenoquine = Tafenoquine 200mg, Mefloquine = Mefloquine 250mg

SB252263 - 030

Listing B.02

Visit And Dosing Dates

Treatment : Mefloquine

Subject Number	Popn*	Visit	Visit Date	Dose Taken ?	Date of Dose	Time of Dose
030.001.00286	IPP	Week 15 (Unsch. Fol. Up)	21AUG2000	No		
030.001.00289	IPP	Screening	14APR2000	No		
		Clearance (Day -5)	05MAY2000	Yes	05MAY2000	
		Clearance (Day -4)	06MAY2000	Yes	06MAY2000	
		Clearance (Day -3)	07MAY2000	Yes	07MAY2000	
		Load Dose 1	11MAY2000	Yes	11MAY2000	10:35
		Load Dose 2	12MAY2000	Yes	12MAY2000	7:59
		Load Dose 3	13MAY2000	Yes	13MAY2000	12:21
		Week 1	18MAY2000	Yes	18MAY2000	7:00
		Week 1 (Unsch.)	21MAY2000	No		
		Week 2	24MAY2000	Yes	24MAY2000	7:50
		Week 3 (Unsch.)	29MAY2000	No		
		Week 3	31MAY2000	Yes	31MAY2000	6:58
		Week 3 (Unsch.)	31MAY2000	No		
		Week 4	06JUN2000	Yes	06JUN2000	15:33
		Week 5	14JUN2000	Yes	14JUN2000	7:30
		Week 6	21JUN2000	Yes	21JUN2000	7:29
		Week 7	28JUN2000	Yes	28JUN2000	7:40
		Week 7 (Unsch.)	28JUN2000	No		
		Week 8 (Fol. Up)	04JUL2000	No		
		Week 9 (Fol. Up)	12JUL2000	No		
		Week 10 (Unsch. Fol. Up)	18JUL2000	No		
		Week 10 (Fol. Up)	19JUL2000	No		
		Week 11 (Unsch. Fol. Up)	27JUL2000	No		
030.001.00290	IPP	Screening	12APR2000	No		
		Clearance (Day -5)	05MAY2000	Yes	05MAY2000	
		Clearance (Day -4)	06MAY2000	Yes	06MAY2000	
		Clearance (Day -3)	07MAY2000	Yes	07MAY2000	
		Load Dose 1	11MAY2000	Yes	11MAY2000	11:20
		Load Dose 2	12MAY2000	Yes	12MAY2000	13:10
		Load Dose 3	13MAY2000	Yes	13MAY2000	9:32
		Week 1	18MAY2000	Yes	18MAY2000	6:58
		Week 1 (Unsch.)	18MAY2000	No		
		Week 2	24MAY2000	Yes	24MAY2000	7:22
		Week 3	01JUN2000	Yes	01JUN2000	16:59
		Week 4	06JUN2000	Yes	06JUN2000	14:03
		Week 5 (Fol. Up)	15JUN2000	No		
		Week 6 (Fol. Up)	21JUN2000	No		
		Week 7 (Fol. Up)	28JUN2000	No		
		Week 8 (Fol. Up)	05JUL2000	No		

*Popn=Population (I=Intent-to-Treat, PP=Per Protocol)
Tafenoquine = Tafenoquine 200mg, Mefloquine = Mefloquine 250mg

SB252263 - 030

Listing B.02

Visit And Dosing Dates

Treatment : Mefloquine

Subject Number	Popn*	Visit	Visit Date	Dose Taken ?	Date of Dose	Time of Dose
030.001.00292	IPP	Screening	12APR2000	No		
		Clearance (Day -5)	05MAY2000	Yes	05MAY2000	
		Clearance (Day -4)	06MAY2000	Yes	06MAY2000	
		Clearance (Day -3)	07MAY2000	Yes	07MAY2000	
		Load Dose 1	11MAY2000	Yes	11MAY2000	11:47
		Load Dose 2	12MAY2000	Yes	12MAY2000	13:31
		Load Dose 3	13MAY2000	Yes	13MAY2000	9:48
		Week 1	18MAY2000	Yes	18MAY2000	6:28
		Week 2	24MAY2000	Yes	24MAY2000	7:00
		Week 3 (Unsch.)	29MAY2000	No		
		Week 3	31MAY2000	Yes	31MAY2000	6:58
		Week 3 (Unsch.)	04JUN2000	No		
		Week 4	07JUN2000	Yes	07JUN2000	6:49
		Week 5	14JUN2000	Yes	14JUN2000	6:50
		Week 6 (Unsch.)	19JUN2000	No		
		Week 6	21JUN2000	Yes	21JUN2000	6:50
		Week 7	28JUN2000	Yes	28JUN2000	7:06
		Week 8	03JUL2000	Yes	03JUL2000	10:47
		Week 8 (Unsch.)	05JUL2000	No		
		Week 9	12JUL2000	Yes	12JUL2000	6:45
		Week 9 (Unsch. Fol. Up)	14JUL2000	No		
		Week 10 (Fol. Up)	19JUL2000	No		
		Week 11 (Unsch. Fol. Up)	25JUL2000	No		
		Week 11 (Fol. Up)	26JUL2000	No		
		Week 12 (Fol. Up)	02AUG2000	No		
030.001.00299	IPP	Screening	17APR2000	No		
		Clearance (Day -5)	05MAY2000	Yes	05MAY2000	
		Clearance (Day -4)	06MAY2000	Yes	06MAY2000	
		Clearance (Day -3)	07MAY2000	Yes	07MAY2000	
		Load Dose 1	12MAY2000	Yes	12MAY2000	7:35
		Load Dose 2	13MAY2000	Yes	13MAY2000	11:39
		Load Dose 3	14MAY2000	Yes	14MAY2000	9:15
		Week 1	18MAY2000	Yes	18MAY2000	6:00
		Week 2	24MAY2000	Yes	24MAY2000	6:26
		Week 3 (Fol. Up)	31MAY2000	No		
		Week 4 (Fol. Up)	06JUN2000	No		
		Week 5 (Fol. Up)	14JUN2000	No		
030.001.00305	IPP	Screening	13APR2000	No		
		Clearance (Day -5)	09MAY2000	Yes	09MAY2000	
		Clearance (Day -4)	10MAY2000	Yes	10MAY2000	

*Popn=Population (I=Intent-to-Treat, PP=Per Protocol)
Tafenoquine = Tafenoquine 200mg, Mefloquine = Mefloquine 250mg

SB252263 - 030

Listing B.02

Visit And Dosing Dates

Treatment : Mefloquine

Subject Number	Popn*	Visit	Visit Date	Dose Taken ?	Date of Dose	Time of Dose
030.001.00305	IPP	Clearance (Day -3)	11MAY2000	Yes	11MAY2000	
		Load Dose 1	12MAY2000	Yes	12MAY2000	16:45
		Load Dose 2	13MAY2000	Yes	13MAY2000	6:24
		Load Dose 3	14MAY2000	Yes	14MAY2000	18:55
		Week 1 (Unsch.)	15MAY2000	No		
		Week 1	17MAY2000	Yes	17MAY2000	8:40
		Week 1 (Unsch.)	17MAY2000	No		
		Week 2	24MAY2000	Yes	24MAY2000	13:00
		Week 3	01JUN2000	Yes	01JUN2000	8:49
		Week 4	06JUN2000	Yes	06JUN2000	
		Week 5	15JUN2000	Yes	15JUN2000	10:39
		Week 5 (Unsch.)	16JUN2000	No		
		Week 6	21JUN2000	Yes	21JUN2000	9:40
		Week 7	28JUN2000	Yes	28JUN2000	10:00
		Week 7 (Unsch.)	29JUN2000	No		
		Week 8	04JUL2000	Yes	04JUL2000	6:32
		Week 9 (Fol. Up)	12JUL2000	No		
		Week 9 (Fol. Up)	17JUL2000	No		
		Week 11 (Fol. Up)	26JUL2000	No		

*Popn=Population (I=Intent-to-Treat, PP=Per Protocol)
Tafenoquine = Tafenoquine 200mg, Mefloquine = Mefloquine 250mg

SB252263 - 030

Listing B.03

Subject Withdrawals

Treatment : Placebo

Subject Number	Popn *	Completed Trial	Date Of Last Dose	Reason For Withdrawal	Comments
030.001.00002	IPP	No	19JUN2000	Insufficient Therapeutic Effect (Development of Parasitemia)	
030.001.00005	IPP	No	03JUL2000	Insufficient Therapeutic Effect (Development of Parasitemia)	
030.001.00011	IPP	No	29MAY2000	Insufficient Therapeutic Effect (Development of Parasitemia)	
030.001.00012	IPP	No	26JUN2000	Insufficient Therapeutic Effect (Development of Parasitemia)	
030.001.00015	I	No	17JUL2000	Lost to Follow-up	
030.001.00019	IPP	No	10JUL2000	Insufficient Therapeutic Effect (Development of Parasitemia)	
030.001.00020	IPP	No	10JUL2000	Insufficient Therapeutic Effect (Development of Parasitemia)	
030.001.00022	IPP	No	22MAY2000	Insufficient Therapeutic Effect (Development of Parasitemia)	
030.001.00026	IPP	No	22MAY2000	Insufficient Therapeutic Effect (Development of Parasitemia)	
030.001.00027	IPP	No	22MAY2000	Insufficient Therapeutic Effect (Development of Parasitemia)	
030.001.00032	IPP	No	03JUL2000	Insufficient Therapeutic Effect (Development of Parasitemia)	
030.001.00033	IPP	No	22MAY2000	Insufficient Therapeutic Effect (Development of Parasitemia)	
030.001.00037	IPP	No	29MAY2000	Insufficient Therapeutic Effect (Development of Parasitemia)	
030.001.00039	IPP	No	12JUN2000	Insufficient Therapeutic Effect (Development of Parasitemia)	
030.001.00041	IPP	No	12JUN2000	Insufficient Therapeutic Effect (Development of Parasitemia)	
030.001.00044	IPP	No	19JUN2000	Insufficient Therapeutic Effect (Development of Parasitemia)	
030.001.00055	IPP	No	22MAY2000	Insufficient Therapeutic Effect (Development of Parasitemia)	
030.001.00056	I	No	12JUN2000	Protocol Deviation (including non-compliance)	
030.001.00057	IPP	No	26JUN2000	Insufficient Therapeutic Effect (Development of Parasitemia)	
030.001.00059	IPP	No	22MAY2000	Insufficient Therapeutic Effect (Development of Parasitemia)	
030.001.00063	IPP	No	03JUL2000	Insufficient Therapeutic Effect (Development of Parasitemia)	
030.001.00064	IPP	No	26JUN2000	Insufficient Therapeutic Effect (Development of Parasitemia)	
030.001.00067	IPP	No	07AUG2000	Insufficient Therapeutic Effect (Development of Parasitemia)	
030.001.00072	IPP	No	26JUN2000	Insufficient Therapeutic Effect (Development of Parasitemia)	
030.001.00076	IPP	No	12JUN2000	Insufficient Therapeutic Effect (Development of Parasitemia)	
030.001.00078	IPP	No	19JUN2000	Insufficient Therapeutic Effect (Development of Parasitemia)	
030.001.00080	IPP	No	14AUG2000	Insufficient Therapeutic Effect (Development of Parasitemia)	
030.001.00081	IPP	No	13JUN2000	Insufficient Therapeutic Effect (Development of Parasitemia)	
030.001.00087	I	No	15MAY2000	Insufficient Therapeutic Effect (Development of Parasitemia)	
030.001.00088	IPP	No	12JUN2000	Insufficient Therapeutic Effect (Development of Parasitemia)	
030.001.00090	IPP	No	25JUL2000	Insufficient Therapeutic Effect (Development of Parasitemia)	
030.001.00092	IPP	No	03JUL2000	Insufficient Therapeutic Effect (Development of Parasitemia)	
030.001.00101	IPP	No	03JUL2000	Insufficient Therapeutic Effect (Development of Parasitemia)	
030.001.00103	IPP	No	19JUN2000	Insufficient Therapeutic Effect (Development of Parasitemia)	
030.001.00106	IPP	No	02JUL2000	Insufficient Therapeutic Effect (Development of Parasitemia)	
030.001.00108	IPP	No	22MAY2000	Insufficient Therapeutic Effect (Development of Parasitemia)	
030.001.00114	IPP	No	03JUL2000	Insufficient Therapeutic Effect (Development of Parasitemia)	
030.001.00115	IPP	No	05JUN2000	Insufficient Therapeutic Effect (Development of Parasitemia)	
030.001.00116	IPP	No	22MAY2000	Insufficient Therapeutic Effect (Development of Parasitemia)	
030.001.00119	IPP	No	23MAY2000	Insufficient Therapeutic Effect (Development of Parasitemia)	
030.001.00124	IPP	No	05JUN2000	Insufficient Therapeutic Effect (Development of Parasitemia)	
030.001.00125	IPP	No	03JUL2000	Insufficient Therapeutic Effect (Development of Parasitemia)	
030.001.00127	I	No	11JUN2000	Lost to Follow-up	
030.001.00131	IPP	No	26JUN2000	Insufficient Therapeutic Effect (Development of Parasitemia)	

*Popn=Population (I=Intent-to-Treat, PP=Per Protocol)
Tafenoquine = Tafenoquine 200mg, Mefloquine = Mefloquine 250mg

SB252263 - 030

Listing B.03

Subject Withdrawals

Treatment : Placebo

Subject Number	Popn *	Completed Trial	Date Of Last Dose	Reason For Withdrawal	Comments
030.001.00136	IPP	No	05JUN2000	Insufficient Therapeutic Effect (Development of Parasitemia)	
030.001.00137	IPP	No	03JUL2000	Insufficient Therapeutic Effect (Development of Parasitemia)	
030.001.00140	IPP	No	19JUN2000	Insufficient Therapeutic Effect (Development of Parasitemia)	
030.001.00141	IPP	No	26JUN2000	Insufficient Therapeutic Effect (Development of Parasitemia)	
030.001.00145	IPP	No	13JUN2000	Insufficient Therapeutic Effect (Development of Parasitemia)	
030.001.00151	IPP	No	10JUL2000	Insufficient Therapeutic Effect (Development of Parasitemia)	
030.001.00152	IPP	No	26JUN2000	Insufficient Therapeutic Effect (Development of Parasitemia)	
030.001.00154	IPP	No	12JUN2000	Insufficient Therapeutic Effect (Development of Parasitemia)	
030.001.00160	I	No	26JUN2000	Insufficient Therapeutic Effect (Development of Parasitemia)	
030.001.00162	IPP	No	03JUL2000	Insufficient Therapeutic Effect (Development of Parasitemia)	
030.001.00164	IPP	No	12JUN2000	Insufficient Therapeutic Effect (Development of Parasitemia)	
030.001.00166	IPP	Yes	23OCT2000	Completed	
030.001.00169	IPP	No	26JUN2000	Insufficient Therapeutic Effect (Development of Parasitemia)	
030.001.00172	IPP	No	28SEP2000	Insufficient Therapeutic Effect (Development of Parasitemia)	
030.001.00174	IPP	No	12JUL2000	Insufficient Therapeutic Effect (Development of Parasitemia)	
030.001.00175	IPP	No	12JUL2000	Insufficient Therapeutic Effect (Development of Parasitemia)	
030.001.00182	I	No	06JUN2000	Other	WITHDREW CONSENT
030.001.00183	IPP	No	21JUN2000	Insufficient Therapeutic Effect (Development of Parasitemia)	
030.001.00191	IPP	No	15JUN2000	Insufficient Therapeutic Effect (Development of Parasitemia)	
030.001.00192	IPP	No	31MAY2000	Insufficient Therapeutic Effect (Development of Parasitemia)	
030.001.00193	IPP	No	31MAY2000	Insufficient Therapeutic Effect (Development of Parasitemia)	
030.001.00197	IPP	No	31MAY2000	Insufficient Therapeutic Effect (Development of Parasitemia)	
030.001.00202		No	11MAY2000	Lost to Follow-up	
030.001.00204	I	No	05JUN2000	Protocol Deviation (including non-compliance)	
030.001.00208	IPP	No	02AUG2000	Insufficient Therapeutic Effect (Development of Parasitemia)	
030.001.00211	IPP	No	21JUN2000	Insufficient Therapeutic Effect (Development of Parasitemia)	
030.001.00212	IPP	No	28JUN2000	Insufficient Therapeutic Effect (Development of Parasitemia)	
030.001.00215	IPP	No	14JUN2000	Insufficient Therapeutic Effect (Development of Parasitemia)	
030.001.00219	IPP	No	14JUN2000	Insufficient Therapeutic Effect (Development of Parasitemia)	
030.001.00222	IPP	No	21JUN2000	Insufficient Therapeutic Effect (Development of Parasitemia)	
030.001.00225	IPP	No	14JUN2000	Insufficient Therapeutic Effect (Development of Parasitemia)	
030.001.00226	IPP	No	21JUN2000	Other	WITHDREW CONSENT
030.001.00229	IPP	No	21JUN2000	Insufficient Therapeutic Effect (Development of Parasitemia)	
030.001.00234	IPP	No	14JUN2000	Insufficient Therapeutic Effect (Development of Parasitemia)	
030.001.00236	IPP	No	12JUL2000	Insufficient Therapeutic Effect (Development of Parasitemia)	
030.001.00238	IPP	No	14JUN2000	Insufficient Therapeutic Effect (Development of Parasitemia)	
030.001.00242	IPP	No	14JUN2000	Insufficient Therapeutic Effect (Development of Parasitemia)	
030.001.00247	IPP	No	26JUL2000	Insufficient Therapeutic Effect (Development of Parasitemia)	
030.001.00248	IPP	No	03JUL2000	Insufficient Therapeutic Effect (Development of Parasitemia)	
030.001.00251	IPP	No	14JUN2000	Insufficient Therapeutic Effect (Development of Parasitemia)	
030.001.00253	IPP	No	28JUN2000	Insufficient Therapeutic Effect (Development of Parasitemia)	
030.001.00256	IPP	No	16JUN2000	Insufficient Therapeutic Effect (Development of Parasitemia)	

*Popn=Population (I=Intent-to-Treat, PP=Per Protocol)
Tafenoquine = Tafenoquine 200mg, Mefloquine = Mefloquine 250mg

SB252263 - 030

Listing B.03

Subject Withdrawals

Treatment : Placebo

Subject Number	Popn *	Completed Trial	Date Of Last Dose	Reason For Withdrawal	Comments
030.001.00257	IPP	No	23JUN2000	Insufficient Therapeutic Effect (Development of Parasitemia)	
030.001.00260	IPP	No	21JUN2000	Insufficient Therapeutic Effect (Development of Parasitemia)	
030.001.00268	IPP	No	24MAY2000	Insufficient Therapeutic Effect (Development of Parasitemia)	
030.001.00269	IPP	No	21JUN2000	Insufficient Therapeutic Effect (Development of Parasitemia)	
030.001.00270	IPP	No	28JUN2000	Insufficient Therapeutic Effect (Development of Parasitemia)	
030.001.00275	IPP	No	28JUN2000	Insufficient Therapeutic Effect (Development of Parasitemia)	
030.001.00277	IPP	No	19JUL2000	Insufficient Therapeutic Effect (Development of Parasitemia)	
030.001.00278	IPP	No	14JUN2000	Insufficient Therapeutic Effect (Development of Parasitemia)	
030.001.00285	IPP	No	28JUN2000	Insufficient Therapeutic Effect (Development of Parasitemia)	
030.001.00287	IPP	No	26JUL2000	Insufficient Therapeutic Effect (Development of Parasitemia)	
030.001.00291	IPP	No	28JUN2000	Insufficient Therapeutic Effect (Development of Parasitemia)	
030.001.00295		No	19JUL2000	Insufficient Therapeutic Effect (Development of Parasitemia)	
030.001.00297	IPP	No	20SEP2000	Insufficient Therapeutic Effect (Development of Parasitemia)	
030.001.00300	IPP	No	21JUN2000	Insufficient Therapeutic Effect (Development of Parasitemia)	
030.001.00303	IPP	No	12JUL2000	Insufficient Therapeutic Effect (Development of Parasitemia)	

*Popn=Population (I=Intent-to-Treat, PP=Per Protocol)
Tafenoquine = Tafenoquine 200mg, Mefloquine = Mefloquine 250mg

SB252263 - 030

Listing B.03

Subject Withdrawals

Treatment : Tafenoquine

Subject Number	Popn *	Completed Trial	Date Of Last Dose	Reason For Withdrawal	Comments
030.001.00001	IPP	No	15MAY2000	Insufficient Therapeutic Effect (Development of Parasitemia)	
030.001.00004	IPP	No	22MAY2000	Insufficient Therapeutic Effect (Development of Parasitemia)	
030.001.00006	IPP	No	22MAY2000	Insufficient Therapeutic Effect (Development of Parasitemia)	
030.001.00009	IPP	No	26JUN2000	Insufficient Therapeutic Effect (Development of Parasitemia)	
030.001.00014	IPP	No	31JUL2000	Insufficient Therapeutic Effect (Development of Parasitemia)	
030.001.00016	IPP	No	07AUG2000	Insufficient Therapeutic Effect (Development of Parasitemia)	
030.001.00017	IPP	No	18SEP2000	Insufficient Therapeutic Effect (Development of Parasitemia)	
030.001.00024	IPP	No	19JUN2000	Insufficient Therapeutic Effect (Development of Parasitemia)	
030.001.00025	IPP	No	07AUG2000	Insufficient Therapeutic Effect (Development of Parasitemia)	
030.001.00028	IPP	No	17JUL2000	Insufficient Therapeutic Effect (Development of Parasitemia)	
030.001.00029	IPP	No	03JUL2000	Insufficient Therapeutic Effect (Development of Parasitemia)	
030.001.00030	IPP	No	10JUL2000	Insufficient Therapeutic Effect (Development of Parasitemia)	
030.001.00040	IPP	No	22MAY2000	Insufficient Therapeutic Effect (Development of Parasitemia)	
030.001.00042	IPP	No	26JUN2000	Insufficient Therapeutic Effect (Development of Parasitemia)	
030.001.00043	IPP	No	22MAY2000	Insufficient Therapeutic Effect (Development of Parasitemia)	
030.001.00048	IPP	No	26JUN2000	Lost to Follow-up	
030.001.00050	IPP	No	31MAY2000	Insufficient Therapeutic Effect (Development of Parasitemia)	
030.001.00051	IPP	No	25SEP2000	Insufficient Therapeutic Effect (Development of Parasitemia)	
030.001.00058	IPP	No	17JUL2000	Insufficient Therapeutic Effect (Development of Parasitemia)	
030.001.00060	IPP	No	17JUL2000	Insufficient Therapeutic Effect (Development of Parasitemia)	
030.001.00061	IPP	No	10JUL2000	Insufficient Therapeutic Effect (Development of Parasitemia)	
030.001.00065	IPP	No	26JUN2000	Insufficient Therapeutic Effect (Development of Parasitemia)	
030.001.00068	IPP	No	11JUL2000	Insufficient Therapeutic Effect (Development of Parasitemia)	
030.001.00070	IPP	No	03JUL2000	Insufficient Therapeutic Effect (Development of Parasitemia)	
030.001.00077	IPP	No	03JUL2000	Insufficient Therapeutic Effect (Development of Parasitemia)	
030.001.00079	IPP	No	03JUL2000	Insufficient Therapeutic Effect (Development of Parasitemia)	
030.001.00082		No	10MAY2000	Other	WITHDREW CONSENT
030.001.00084	IPP	No	21AUG2000	Insufficient Therapeutic Effect (Development of Parasitemia)	
030.001.00085	IPP	No	07AUG2000	Insufficient Therapeutic Effect (Development of Parasitemia)	
030.001.00086	IPP	No	22MAY2000	Insufficient Therapeutic Effect (Development of Parasitemia)	
030.001.00093	IPP	No	04SEP2000	Insufficient Therapeutic Effect (Development of Parasitemia)	
030.001.00094	I	No	30AUG2000	Insufficient Therapeutic Effect (Development of Parasitemia)	
030.001.00100	IPP	No	26JUN2000	Insufficient Therapeutic Effect (Development of Parasitemia)	
030.001.00102	IPP	No	10JUL2000	Insufficient Therapeutic Effect (Development of Parasitemia)	
030.001.00104	IPP	No	26JUN2000	Insufficient Therapeutic Effect (Development of Parasitemia)	
030.001.00107	IPP	No	07AUG2000	Insufficient Therapeutic Effect (Development of Parasitemia)	
030.001.00109	IPP	No	24JUL2000	Insufficient Therapeutic Effect (Development of Parasitemia)	
030.001.00111	IPP	No	03OCT2000	Insufficient Therapeutic Effect (Development of Parasitemia)	
030.001.00112	IPP	No	26JUN2000	Insufficient Therapeutic Effect (Development of Parasitemia)	
030.001.00118	IPP	No	26JUN2000	Insufficient Therapeutic Effect (Development of Parasitemia)	
030.001.00122	IPP	No	22MAY2000	Insufficient Therapeutic Effect (Development of Parasitemia)	
030.001.00126	IPP	Yes	23OCT2000	Completed	
030.001.00128	IPP	Yes	23OCT2000	Completed	

*Popn=Population (I=Intent-to-Treat, PP=Per Protocol)
Tafenoquine = Tafenoquine 200mg, Mefloquine = Mefloquine 250mg

SB252263 - 030

Listing B.03

Subject Withdrawals

Treatment : Tafenoquine

Subject Number	Popn *	Completed Trial	Date Of Last Dose	Reason For Withdrawal	Comments
030.001.00129	IPP	No	07AUG2000	Insufficient Therapeutic Effect (Development of Parasitemia)	
030.001.00133	IPP	No	10JUL2000	Insufficient Therapeutic Effect (Development of Parasitemia)	
030.001.00135	IPP	No	26JUN2000	Insufficient Therapeutic Effect (Development of Parasitemia)	
030.001.00138	IPP	No	26JUN2000	Insufficient Therapeutic Effect (Development of Parasitemia)	
030.001.00139	IPP	No	18SEP2000	Insufficient Therapeutic Effect (Development of Parasitemia)	
030.001.00148	IPP	No	28AUG2000	Insufficient Therapeutic Effect (Development of Parasitemia)	
030.001.00149	IPP	No	17JUL2000	Insufficient Therapeutic Effect (Development of Parasitemia)	
030.001.00150	IPP	No	03JUL2000	Insufficient Therapeutic Effect (Development of Parasitemia)	
030.001.00155	IPP	No	31JUL2000	Insufficient Therapeutic Effect (Development of Parasitemia)	
030.001.00157	IPP	No	04JUN2000	Insufficient Therapeutic Effect (Development of Parasitemia)	
030.001.00158	IPP	No	29MAY2000	Insufficient Therapeutic Effect (Development of Parasitemia)	
030.001.00159	IPP	No	25SEP2000	Insufficient Therapeutic Effect (Development of Parasitemia)	
030.001.00161	IPP	No	03JUL2000	Insufficient Therapeutic Effect (Development of Parasitemia)	
030.001.00171	IPP	No	12JUN2000	Insufficient Therapeutic Effect (Development of Parasitemia)	
030.001.00173	I	No	26JUN2000	Adverse Experience	
030.001.00176	IPP	No	31MAY2000	Insufficient Therapeutic Effect (Development of Parasitemia)	
030.001.00179	I	No	06SEP2000	Lost to Follow-up	
030.001.00184	IPP	No	28JUN2000	Insufficient Therapeutic Effect (Development of Parasitemia)	
030.001.00186	IPP	No	14JUN2000	Insufficient Therapeutic Effect (Development of Parasitemia)	
030.001.00187	I	No	19JUL2000	Lost to Follow-up	
030.001.00190	IPP	No	28JUN2000	Insufficient Therapeutic Effect (Development of Parasitemia)	
030.001.00194	IPP	No	31MAY2000	Insufficient Therapeutic Effect (Development of Parasitemia)	
030.001.00195	IPP	No	31MAY2000	Insufficient Therapeutic Effect (Development of Parasitemia)	
030.001.00201	IPP	No	14JUN2000	Insufficient Therapeutic Effect (Development of Parasitemia)	
030.001.00203	IPP	No	21JUN2000	Insufficient Therapeutic Effect (Development of Parasitemia)	
030.001.00205	IPP	No	14JUN2000	Insufficient Therapeutic Effect (Development of Parasitemia)	
030.001.00207	IPP	No	28AUG2000	Insufficient Therapeutic Effect (Development of Parasitemia)	
030.001.00209	IPP	No	05JUL2000	Insufficient Therapeutic Effect (Development of Parasitemia)	
030.001.00210	IPP	No	02AUG2000	Insufficient Therapeutic Effect (Development of Parasitemia)	
030.001.00217	IPP	No	21JUN2000	Insufficient Therapeutic Effect (Development of Parasitemia)	
030.001.00218	IPP	Yes	24OCT2000	Completed	
030.001.00220	IPP	No	28JUN2000	Insufficient Therapeutic Effect (Development of Parasitemia)	
030.001.00228	IPP	No	14JUN2000	Insufficient Therapeutic Effect (Development of Parasitemia)	
030.001.00230	IPP	No	04JUL2000	Insufficient Therapeutic Effect (Development of Parasitemia)	
030.001.00231	IPP	No	13SEP2000	Insufficient Therapeutic Effect (Development of Parasitemia)	
030.001.00233	IPP	No	04SEP2000	Insufficient Therapeutic Effect (Development of Parasitemia)	
030.001.00240	IPP	No	14JUN2000	Insufficient Therapeutic Effect (Development of Parasitemia)	
030.001.00243	IPP	Yes	24OCT2000	Completed	
030.001.00246	IPP	No	12JUL2000	Insufficient Therapeutic Effect (Development of Parasitemia)	
030.001.00249	IPP	No	04JUL2000	Insufficient Therapeutic Effect (Development of Parasitemia)	
030.001.00252	IPP	No	04OCT2000	Insufficient Therapeutic Effect (Development of Parasitemia)	
030.001.00254	I	No	08JUN2000	Other	WITHDREW CONSENT
030.001.00259	IPP	No	03JUL2000	Insufficient Therapeutic Effect (Development of Parasitemia)	

*Popn=Population (I=Intent-to-Treat, PP=Per Protocol)
Tafenoquine = Tafenoquine 200mg, Mefloquine = Mefloquine 250mg

SB252263 - 030

Listing B.03

Subject Withdrawals

Treatment : Tafenoquine

Subject Number	Popn *	Completed Trial	Date Of Last Dose	Reason For Withdrawal	Comments
030.001.00261		No	12MAY2000	Adverse Experience	
030.001.00264	IPP	No	19JUL2000	Insufficient Therapeutic Effect (Development of Parasitemia)	
030.001.00266	I	No	24MAY2000	Protocol Deviation (including non-compliance)	
030.001.00267	IPP	No	21JUN2000	Insufficient Therapeutic Effect (Development of Parasitemia)	
030.001.00271	I	No	31MAY2000	Other	WITHDREW CONSENT
030.001.00272	IPP	No	26JUL2000	Insufficient Therapeutic Effect (Development of Parasitemia)	
030.001.00279	IPP	No	19MAY2000	Insufficient Therapeutic Effect (Development of Parasitemia)	
030.001.00282	IPP	No	13SEP2000	Insufficient Therapeutic Effect (Development of Parasitemia)	
030.001.00284	IPP	Yes	23OCT2000	Completed	
030.001.00288	IPP	No	06JUN2000	Insufficient Therapeutic Effect (Development of Parasitemia)	
030.001.00293	IPP	Yes	25OCT2000	Completed	
030.001.00294	IPP	No	12JUL2000	Insufficient Therapeutic Effect (Development of Parasitemia)	
030.001.00296	IPP	Yes	23OCT2000	Completed	
030.001.00298	IPP	No	02AUG2000	Insufficient Therapeutic Effect (Development of Parasitemia)	
030.001.00301	IPP	No	04JUL2000	Insufficient Therapeutic Effect (Development of Parasitemia)	
030.001.00302	I	No	21MAY2000	Insufficient Therapeutic Effect (Development of Parasitemia)	
030.001.00304	IPP	No	04JUL2000	Insufficient Therapeutic Effect (Development of Parasitemia)	
030.001.00306	IPP	No	02AUG2000	Insufficient Therapeutic Effect (Development of Parasitemia)	

*Popn=Population (I=Intent-to-Treat, PP=Per Protocol)
Tafenoquine = Tafenoquine 200mg, Mefloquine = Mefloquine 250mg

SB252263 - 030

Listing B.03

Subject Withdrawals

Treatment : Mefloquine

Subject Number	Popn *	Completed Trial	Date Of Last Dose	Reason For Withdrawal	Comments
030.001.00003	I	No	16JUN2000	Other	WITHDREW CONSENT
030.001.00007	IPP	No	16OCT2000	Insufficient Therapeutic Effect (Development of Parasitemia)	
030.001.00008	IPP	No	16OCT2000	Insufficient Therapeutic Effect (Development of Parasitemia)	
030.001.00010	IPP	No	12JUN2000	Insufficient Therapeutic Effect (Development of Parasitemia)	
030.001.00013	IPP	No	22MAY2000	Insufficient Therapeutic Effect (Development of Parasitemia)	
030.001.00018	IPP	No	10JUL2000	Insufficient Therapeutic Effect (Development of Parasitemia)	
030.001.00021	IPP	Yes	23OCT2000	Completed	
030.001.00023	I	No	22MAY2000	Insufficient Therapeutic Effect (Development of Parasitemia)	
030.001.00031	IPP	No	02OCT2000	Insufficient Therapeutic Effect (Development of Parasitemia)	
030.001.00034	IPP	No	07AUG2000	Insufficient Therapeutic Effect (Development of Parasitemia)	
030.001.00035		No	10MAY2000	Adverse Experience	
030.001.00036	I	No	17JUL2000	Protocol Deviation (including non-compliance)	
030.001.00038	IPP	No	19JUN2000	Insufficient Therapeutic Effect (Development of Parasitemia)	
030.001.00045	IPP	No	22MAY2000	Insufficient Therapeutic Effect (Development of Parasitemia)	
030.001.00046	IPP	No	26JUN2000	Insufficient Therapeutic Effect (Development of Parasitemia)	
030.001.00047	IPP	No	19JUN2000	Insufficient Therapeutic Effect (Development of Parasitemia)	
030.001.00049	IPP	No	17JUL2000	Insufficient Therapeutic Effect (Development of Parasitemia)	
030.001.00052	IPP	No	12JUN2000	Insufficient Therapeutic Effect (Development of Parasitemia)	
030.001.00053	IPP	No	31JUL2000	Insufficient Therapeutic Effect (Development of Parasitemia)	
030.001.00054	IPP	No	26JUN2000	Insufficient Therapeutic Effect (Development of Parasitemia)	
030.001.00062	IPP	No	19JUN2000	Insufficient Therapeutic Effect (Development of Parasitemia)	
030.001.00066	IPP	No	06JUN2000	Insufficient Therapeutic Effect (Development of Parasitemia)	
030.001.00069	I	No	17JUL2000	Insufficient Therapeutic Effect (Development of Parasitemia)	
030.001.00071	IPP	No	17JUL2000	Insufficient Therapeutic Effect (Development of Parasitemia)	
030.001.00073	IPP	No	26JUN2000	Insufficient Therapeutic Effect (Development of Parasitemia)	
030.001.00074	IPP	No	24JUL2000	Insufficient Therapeutic Effect (Development of Parasitemia)	
030.001.00075	IPP	No	10JUL2000	Insufficient Therapeutic Effect (Development of Parasitemia)	
030.001.00083	IPP	No	10JUL2000	Insufficient Therapeutic Effect (Development of Parasitemia)	
030.001.00089	IPP	No	22MAY2000	Insufficient Therapeutic Effect (Development of Parasitemia)	
030.001.00091	IPP	No	14AUG2000	Insufficient Therapeutic Effect (Development of Parasitemia)	
030.001.00095	IPP	No	20JUN2000	Insufficient Therapeutic Effect (Development of Parasitemia)	
030.001.00096	IPP	No	03JUL2000	Insufficient Therapeutic Effect (Development of Parasitemia)	
030.001.00097	IPP	No	19JUN2000	Insufficient Therapeutic Effect (Development of Parasitemia)	
030.001.00098	IPP	No	09JUL2000	Insufficient Therapeutic Effect (Development of Parasitemia)	
030.001.00099	IPP	No	12JUN2000	Insufficient Therapeutic Effect (Development of Parasitemia)	
030.001.00105	IPP	No	22MAY2000	Insufficient Therapeutic Effect (Development of Parasitemia)	
030.001.00110	IPP	No	17JUL2000	Insufficient Therapeutic Effect (Development of Parasitemia)	
030.001.00113	IPP	No	26JUN2000	Insufficient Therapeutic Effect (Development of Parasitemia)	
030.001.00117	IPP	No	26JUN2000	Insufficient Therapeutic Effect (Development of Parasitemia)	
030.001.00120	IPP	No	22MAY2000	Insufficient Therapeutic Effect (Development of Parasitemia)	
030.001.00121	IPP	No	26JUN2000	Insufficient Therapeutic Effect (Development of Parasitemia)	
030.001.00123	I	No	21JUN2000	Insufficient Therapeutic Effect (Development of Parasitemia)	
030.001.00130	IPP	No	24JUL2000	Insufficient Therapeutic Effect (Development of Parasitemia)	

*Popn=Population (I=Intent-to-Treat, PP=Per Protocol)
Tafenoquine = Tafenoquine 200mg, Mefloquine = Mefloquine 250mg

SB252263 - 030

Listing B.03

Subject Withdrawals

Treatment : Mefloquine

Subject Number	Popn *	Completed Trial	Date Of Last Dose	Reason For Withdrawal	Comments
030.001.00132	IPP	No	26JUN2000	Insufficient Therapeutic Effect (Development of Parasitemia)	
030.001.00134	IPP	No	26JUL2000	Insufficient Therapeutic Effect (Development of Parasitemia)	
030.001.00142	IPP	No	18SEP2000	Insufficient Therapeutic Effect (Development of Parasitemia)	
030.001.00143	IPP	No	26JUN2000	Insufficient Therapeutic Effect (Development of Parasitemia)	
030.001.00144	IPP	No	05JUN2000	Insufficient Therapeutic Effect (Development of Parasitemia)	
030.001.00146	I	No	20JUN2000	Other	WITHDREW CONSENT
030.001.00147	IPP	No	31JUL2000	Insufficient Therapeutic Effect (Development of Parasitemia)	
030.001.00153	IPP	No	16OCT2000	Insufficient Therapeutic Effect (Development of Parasitemia)	
030.001.00156	IPP	No	03JUL2000	Insufficient Therapeutic Effect (Development of Parasitemia)	
030.001.00163	IPP	No	26JUN2000	Insufficient Therapeutic Effect (Development of Parasitemia)	
030.001.00165	IPP	No	05JUN2000	Insufficient Therapeutic Effect (Development of Parasitemia)	
030.001.00167	IPP	No	12JUN2000	Insufficient Therapeutic Effect (Development of Parasitemia)	
030.001.00168	IPP	No	05JUN2000	Insufficient Therapeutic Effect (Development of Parasitemia)	
030.001.00170	IPP	No	26JUN2000	Insufficient Therapeutic Effect (Development of Parasitemia)	
030.001.00177	IPP	No	19JUL2000	Insufficient Therapeutic Effect (Development of Parasitemia)	
030.001.00178	I	No	04JUL2000	Other	WITHDREW CONSENT
030.001.00180	IPP	Yes	16OCT2000	Completed	
030.001.00181	IPP	No	14JUN2000	Insufficient Therapeutic Effect (Development of Parasitemia)	
030.001.00185	IPP	No	16OCT2000	Insufficient Therapeutic Effect (Development of Parasitemia)	
030.001.00188	IPP	No	05JUN2000	Insufficient Therapeutic Effect (Development of Parasitemia)	
030.001.00189	I	No	31MAY2000	Insufficient Therapeutic Effect (Development of Parasitemia)	
030.001.00196	IPP	No	12JUL2000	Insufficient Therapeutic Effect (Development of Parasitemia)	
030.001.00198	IPP	No	28JUN2000	Insufficient Therapeutic Effect (Development of Parasitemia)	
030.001.00199	IPP	No	21JUN2000	Insufficient Therapeutic Effect (Development of Parasitemia)	
030.001.00200	IPP	No	31MAY2000	Insufficient Therapeutic Effect (Development of Parasitemia)	
030.001.00206	I	No	17MAY2000	Lost to Follow-up	
030.001.00213	IPP	No	01AUG2000	Insufficient Therapeutic Effect (Development of Parasitemia)	
030.001.00214	IPP	No	21JUN2000	Insufficient Therapeutic Effect (Development of Parasitemia)	
030.001.00216	IPP	No	20JUL2000	Insufficient Therapeutic Effect (Development of Parasitemia)	
030.001.00221	IPP	No	20JUL2000	Insufficient Therapeutic Effect (Development of Parasitemia)	
030.001.00223	IPP	No	14JUN2000	Insufficient Therapeutic Effect (Development of Parasitemia)	
030.001.00224	IPP	No	06JUN2000	Insufficient Therapeutic Effect (Development of Parasitemia)	
030.001.00227	IPP	No	21JUN2000	Insufficient Therapeutic Effect (Development of Parasitemia)	
030.001.00232	IPP	No	24MAY2000	Insufficient Therapeutic Effect (Development of Parasitemia)	
030.001.00235	IPP	No	14JUN2000	Insufficient Therapeutic Effect (Development of Parasitemia)	
030.001.00237	IPP	No	06JUN2000	Insufficient Therapeutic Effect (Development of Parasitemia)	
030.001.00239	IPP	No	14JUN2000	Insufficient Therapeutic Effect (Development of Parasitemia)	
030.001.00241	IPP	Yes	24OCT2000	Completed	
030.001.00244	IPP	No	02AUG2000	Insufficient Therapeutic Effect (Development of Parasitemia)	
030.001.00245	IPP	No	26JUL2000	Insufficient Therapeutic Effect (Development of Parasitemia)	
030.001.00250		No	11MAY2000	Protocol Deviation (including non-compliance)	
030.001.00255	IPP	No	19JUL2000	Insufficient Therapeutic Effect (Development of Parasitemia)	

*Popn=Population (I=Intent-to-Treat, PP=Per Protocol)
Tafenoquine = Tafenoquine 200mg, Mefloquine = Mefloquine 250mg

SB252263 - 030

Listing B.03

Subject Withdrawals

Treatment : Mefloquine

Subject Number	Popn *	Completed Trial	Date Of Last Dose	Reason For Withdrawal	Comments
030.001.00258	IPP	No	19JUL2000	Insufficient Therapeutic Effect (Development of Parasitemia)	
030.001.00262	IPP	No	23JUN2000	Insufficient Therapeutic Effect (Development of Parasitemia)	
030.001.00263	IPP	Yes	23OCT2000	Completed	
030.001.00265	IPP	No	13SEP2000	Protocol Deviation (including non-compliance)	
030.001.00273	IPP	No	09AUG2000	Insufficient Therapeutic Effect (Development of Parasitemia)	
030.001.00274	IPP	No	28JUN2000	Insufficient Therapeutic Effect (Development of Parasitemia)	
030.001.00276	IPP	No	21JUN2000	Insufficient Therapeutic Effect (Development of Parasitemia)	
030.001.00280	IPP	No	28JUN2000	Insufficient Therapeutic Effect (Development of Parasitemia)	
030.001.00281	IPP	No	21JUN2000	Insufficient Therapeutic Effect (Development of Parasitemia)	
030.001.00283	IPP	No	12JUL2000	Insufficient Therapeutic Effect (Development of Parasitemia)	
030.001.00286	IPP	No	26JUL2000	Insufficient Therapeutic Effect (Development of Parasitemia)	
030.001.00289	IPP	No	28JUN2000	Insufficient Therapeutic Effect (Development of Parasitemia)	
030.001.00290	IPP	No	06JUN2000	Insufficient Therapeutic Effect (Development of Parasitemia)	
030.001.00292	IPP	No	12JUL2000	Insufficient Therapeutic Effect (Development of Parasitemia)	
030.001.00299	IPP	No	24MAY2000	Insufficient Therapeutic Effect (Development of Parasitemia)	
030.001.00305	IPP	No	04JUL2000	Insufficient Therapeutic Effect (Development of Parasitemia)	

*Popn=Population (I=Intent-to-Treat, PP=Per Protocol)
Tafenoquine = Tafenoquine 200mg, Mefloquine = Mefloquine 250mg

Listing B.04

SB252263 - 030

Listing B.04

All Non- Randomised Subjects : Reasons For Exclusion From The Study

Subject Number	G6PD deficient	Parasitemia clearance failure	Abnormal QTc	Abnormal Laboratory Results	Other
030.001.99006	X				
030.001.99008	X				
030.001.99010				X	
030.001.99014	X				
030.001.99015					ABNORMAL EKG
030.001.99018				X	
030.001.99019		X			
030.001.99021					ABNORMAL EKG
030.001.99023	X				
030.001.99029	X				
030.001.99032	X				
					ABNORMAL EKG
030.001.99033	X				
030.001.99034	X				
030.001.99035					HISTORY OF SEIZURES
030.001.99036				X	
030.001.99039	X				
030.001.99041					ABNORMAL EKG
030.001.99042					ABNORMAL EKG
030.001.99047	X				
030.001.99048	X				
030.001.99050					ABNORMAL EKG
030.001.99058					SIGNIFICANT ABNORMALITY OF LUNGS
030.001.99061					DID NOT BEGIN CLEARANCE
030.001.99062				X	
030.001.99063				X	
030.001.99064					HISTORY OF SEIZURES
030.001.99065					ABNORMAL EKG
030.001.99066					HISTORY OF SEIZURES
030.001.99068					ABNORMAL EKG
030.001.99069				X	
030.001.99070				X	
030.001.99073				X	
030.001.99077					DID NOT TAKE SECOND G6PD TEST
030.001.99080	X				
030.001.99084				X	
030.001.99085	X				
030.001.99089				X	
030.001.99091					SEPTIC ANTHRITIS
030.001.99093					
030.001.99097	X				
030.001.99098					HISTORY OF SEIZURES
030.001.99100				X	
030.001.99104	X				
030.001.99106	X				
030.001.99107					DID NOT TAKE SECOND G6PD TEST
030.001.99109					HISTORY OF SEIZURES
030.001.99111					POSITIVE HCG

SB252263 - 030

Listing B.04

All Non- Randomised Subjects : Reasons For Exclusion From The Study

Subject Number	G6PD deficient	Parasitemia clearance failure	Abnormal QTc	Abnormal Laboratory Results	Other
030.001.99112				X	
030.001.99117					ABNORMAL EKG
030.001.99121	X				
030.001.99122					ABNORMAL EKG
030.001.99126				X	
030.001.99129					ABNORMAL EKG
030.001.99130				X	
030.001.99132				X	
030.001.99143				X	
030.001.99147	X				
030.001.99150				X	
					DID NOT BEGIN LOADING
030.001.99152				X	
030.001.99155	X				
030.001.99158					DID NOT COMPLETE CLEARANCE
030.001.99160				X	
			X		
030.001.99161					ABNORMAL EKG
030.001.99162					B-HCG POSITIVE
030.001.99164					CLINICAL SIGNIFICANT ABNORMALITY
030.001.99168			X		
030.001.99171					ABNORMAL EKG
030.001.99173					HISTORY OF SEIZURES
030.001.99174	X				
030.001.99175					DID NOT BEGIN CLEARANCE
030.001.99176				X	
030.001.99179					BREAST FEEDING
030.001.99183				X	
030.001.99185	X				
					ABNORMAL EKG
030.001.99187	X				
030.001.99190					ABNORMAL EKG
030.001.99193				X	
030.001.99194					PLANNING TO BECOME PREGNANT
030.001.99196				X	
030.001.99197					ABNORMAL EKG
030.001.99204					ASTHMATIC
030.001.99207					ABNORMAL EKG
030.001.99210	X				
030.001.99213				X	
030.001.99220				X	
030.001.99222					DID NOT TAKE SECOND G6PD TEST
030.001.99225				X	
030.001.99226	X				
030.001.99227					ABNORMAL EKG
030.001.99229					B-HCG POSITIVE
030.001.99232					DID NOT START LOADING
030.001.99239					HISTORY OF SEIZURES

SB252263 - 030

Listing B.04

All Non- Randomised Subjects : Reasons For Exclusion From The Study

Subject Number	G6PD deficient	Parasitemia clearance failure	Abnormal QTc	Abnormal Laboratory Results	Other
030.001.99245				X	
030.001.99249					DID NOT PRESENT FOR RANDOMIZATION
030.001.99252					ABNORMAL EKG
030.001.99253					ABNORMAL EKG
030.001.99256					PLANNING TO BECOME PREGNANT
030.001.99257	X				
030.001.99260	X				
030.001.99262				X	
030.001.99263					DID NOT BEGIN LOADING
030.001.99271					PARTIAL HYSTERECTOMY
030.001.99272				X	
030.001.99275					DID NOT COMPLETE CLEARANCE
030.001.99276					DID NOT BEGIN CLEARANCE
030.001.99277					B-HCG POSITIVE
030.001.99278					B-HCG POSITIVE
030.001.99280	X				
030.001.99281					B-HCG POSITIVE
030.001.99283					B-HCG POSITIVE
030.001.99288					POSITIVE HCG
030.001.99289					HISTORY OF SEIZURES
030.001.99294		X			
030.001.99296	X				
030.001.99298	X				
030.001.99306					ABNORMAL
030.001.99308	X				
030.001.99309					HISTORY OF SEIZURES
030.001.99310				X	
030.001.99311					CELLUTIS OF RT HAND
030.001.99312				X	
030.001.99314				X	
030.001.99315					HAD SPLEEN REMOVED
030.001.99316	X				
030.001.99317					CLINICAL SIGNIFICANT ABNORMALITY
030.001.99326				X	
030.001.99329				X	
030.001.99335	X				
030.001.99337				X	
030.001.99340					INTRAUTERINE MASS
030.001.99343				X	
030.001.99346					HISTORY OF SEIZURES
030.001.99347					B-HCG POSITIVE
030.001.99348					HISTORY OF SEIZURES
030.001.99349	X				
030.001.99350				X	
030.001.99351	X				
030.001.99352	X				
030.001.99356				X	

Listing B.04

All Non- Randomised Subjects : Reasons For Exclusion From The Study

Subject Number	G6PD deficient	Parasitemia clearance failure	Abnormal QTc	Abnormal Laboratory Results	Other
030.001.99357					B-HCG POSITIVE
030.001.99359	X				
030.001.99360					ABNORMAL EKG
030.001.99362					DID NOT BEGIN LOADING
030.001.99364					DID NOT BEGIN HALFAN
030.001.99366				X	
030.001.99369					ABNORMAL EKG
030.001.99371					ABNORMAL EKG
030.001.99372					ABNORMAL EKG
030.001.99373	X				
030.001.99375					ABNORMAL EKG
030.001.99382				X	
030.001.99383				X	
030.001.99385	X				
030.001.99388				X	
030.001.99389					PLANNING TO BECOME PREGNANT
030.001.99391					HISTORY OF PSYCHIATRIC DISORDER
030.001.99395				X	
030.001.99397	X				
030.001.99399					ABNORMAL EKG
030.001.99402					ABNORMAL EKG
030.001.99404					DID NOT BEGIN CLEARANCE
030.001.99405					TOOK FANSIDAR
030.001.99406				X	
030.001.99408				X	
030.001.99411				X	
					PREGNANT
030.001.99413					ABNORMAL EKG
030.001.99414					ABNORMAL EKG
030.001.99418				X	
030.001.99419				X	
030.001.99422				X	
030.001.99425					LEFT THE STUDY AREA
030.001.99430				X	
030.001.99431				X	
030.001.99433					DID NOT BEGIN CLEARANCE
030.001.99434				X	
030.001.99435					HAND CELLULITIS
030.001.99436				X	
030.001.99437					HISTORY OF SEIZURES
030.001.99443					DID NOT COME FOR LOADING
030.001.99444					ABNORMAL EKG
030.001.99447					VAGINAL BLEEDING
030.001.99450				X	
030.001.99452	X				
030.001.99454	X				
030.001.99455				X	
030.001.99457	X				

SB252263 - 030

Listing B.04

All Non- Randomised Subjects : Reasons For Exclusion From The Study

Subject Number	G6PD deficient	Parasitemia clearance failure	Abnormal QTc	Abnormal Laboratory Results	Other
030.001.99458					PSYCHIATRIC DISORDER
030.001.99459				X	
030.001.99462					DID NOT TAKE SECOND G6PD TEST
030.001.99463					PSYCHIATRIC DISORDER
030.001.99465				X	
030.001.99466					HISTORY OF SEIZURES
030.001.99469					PULMONARY TUBERCLOSIS
030.001.99471					ABNORMAL EKG
030.001.99476	X				
030.001.99477					ABNORMAL EKG
030.001.99479				X	ABNORMAL EKG
030.001.99480			X		
030.001.99484					ABNORMAL EKG
030.001.99485				X	
030.001.99490					CLINICAL SIGNIFICANT ABNORMALITY
030.001.99494					HISTORY OF SEIZURES
030.001.99495				X	
030.001.99496					PLANNING TO GOT TO MOMBASA
030.001.99497	X				
030.001.99498	X				
030.001.99500					BREAST FEEDING
030.001.99502				X	
030.001.99507					HISTORY OF SEIZURES
030.001.99512					DID NOT TAKE SECOND G6PD TEST
030.001.99514				X	

SB252263 - 030

Listing B.05

All Randomised Subjects: Reasons For Exclusion From The Intent-To-Treat Population

Treatment: Placebo

Subject Number	Age (years)	Gender	Eligible For ITT	Eligible For Safety	Reason For Exclusion
030.001.00202	21	Female	No	Yes	Subject does not have at least one smear performed post first dose
030.001.00295	48	Female	No	Yes	Subject has no negative smears prior to first dose

Tafenoquine = Tafenoquine 200mg, Mefloquine = Mefloquine 250mg

SB252263 - 030

Listing B.05

All Randomised Subjects: Reasons For Exclusion From The Intent-To-Treat Population

Treatment: Tafenoquine

Subject Number	Age (years)	Gender	Eligible For ITT	Eligible For Safety	Reason For Exclusion
030.001.00082	36	Male	No	Yes	Subject does not have at least one smear performed post first dose
030.001.00261	31	Female	No	Yes	Subject does not have at least one smear performed post first dose

Tafenoquine = Tafenoquine 200mg, Mefloquine = Mefloquine 250mg

SB252263 - 030

Listing B.05

All Randomised Subjects: Reasons For Exclusion From The Intent-To-Treat Population

Treatment: Mefloquine

Subject Number	Age (years)	Gender	Eligible For ITT	Eligible For Safety	Reason For Exclusion
030.001.00035	18	Male	No	Yes	Subject does not have at least one smear performed post first dose
030.001.00250	18	Male	No	Yes	Subject does not have at least one smear performed post first dose

Tafenoquine = Tafenoquine 200mg, Mefloquine = Mefloquine 250mg

SB252263 - 030

Listing B.06

All Randomised Subjects: Reasons For Exclusion From The Per Protocol Population

Protocol Violation (PV) Codelist

PV Code	PV Description
1	Subject has taken another antimalarial product
2	Subject has received an investigational drug
3	Subject has a medical condition with the potential to bias efficacy
4	Subject was non-compliant with the halofantrine clearance medication
5	Subject was non-compliant with the loading medication
6	Subject was non-compliant with the randomised prophylactic medication
7	Subject was non-compliant with the parasitemia monitoring requirement
8	Subject did not complete the prophylactic phase

SB252263 - 030

Listing B.06

All Randomised Subjects: Reasons For Exclusion From The Per Protocol Population

Treatment : Placebo

Subject Number	Age (years)	Gender	Intent-To-Treat	Per Protcol	1	2	3	4	5	6	7	8
030.001.00002	18	Male	Yes	Yes	.	.	.	.	.	.	.	.
030.001.00005	42	Male	Yes	Yes	.	.	.	.	.	.	.	.
030.001.00011	45	Male	Yes	Yes	.	.	.	.	.	.	.	.
030.001.00012	19	Male	Yes	Yes	.	.	.	.	.	.	.	.
030.001.00015	23	Male	Yes	No	.	.	.	.	.	.	.	X
030.001.00019	33	Female	Yes	Yes	.	.	.	.	.	.	.	.
030.001.00020	40	Female	Yes	Yes	.	.	.	.	.	.	.	.
030.001.00022	36	Male	Yes	Yes	.	.	.	.	.	.	.	.
030.001.00026	32	Male	Yes	Yes	.	.	.	.	.	.	.	.
030.001.00027	22	Male	Yes	Yes	.	.	.	.	.	.	.	.
030.001.00032	20	Male	Yes	Yes	.	.	.	.	.	.	.	.
030.001.00033	30	Male	Yes	Yes	.	.	.	.	.	.	.	.
030.001.00037	32	Male	Yes	Yes	.	.	.	.	.	.	.	.
030.001.00039	18	Male	Yes	Yes	.	.	.	.	.	.	.	.
030.001.00041	20	Male	Yes	Yes	.	.	.	.	.	.	.	.
030.001.00044	25	Male	Yes	Yes	.	.	.	.	.	.	.	.
030.001.00055	55	Female	Yes	Yes	.	.	.	.	.	.	.	.
030.001.00056	48	Female	Yes	No	.	.	.	.	.	.	.	X
030.001.00057	37	Female	Yes	Yes	.	.	.	.	.	.	.	.
030.001.00059	47	Female	Yes	Yes	.	.	.	.	.	.	.	.
030.001.00063	20	Female	Yes	Yes	.	.	.	.	.	.	.	.
030.001.00064	18	Male	Yes	Yes	.	.	.	.	.	.	.	.
030.001.00067	47	Female	Yes	Yes	.	.	.	.	.	.	.	.
030.001.00072	22	Male	Yes	Yes	.	.	.	.	.	.	.	.
030.001.00076	37	Female	Yes	Yes	.	.	.	.	.	.	.	.
030.001.00078	31	Male	Yes	Yes	.	.	.	.	.	.	.	.
030.001.00080	19	Male	Yes	Yes	.	.	.	.	.	.	.	.
030.001.00081	38	Male	Yes	Yes	.	.	.	.	.	.	.	.
030.001.00087	20	Male	Yes	No	.	.	.	.	.	.	.	X
030.001.00088	22	Male	Yes	Yes	.	.	.	.	.	.	.	.
030.001.00090	48	Female	Yes	Yes	.	.	.	.	.	.	.	.
030.001.00092	41	Female	Yes	Yes	.	.	.	.	.	.	.	.
030.001.00101	33	Female	Yes	Yes	.	.	.	.	.	.	.	.
030.001.00103	52	Female	Yes	Yes	.	.	.	.	.	.	.	.
030.001.00106	19	Male	Yes	Yes	.	.	.	.	.	.	.	.
030.001.00108	31	Male	Yes	Yes	.	.	.	.	.	.	.	.
030.001.00114	47	Female	Yes	Yes	.	.	.	.	.	.	.	.
030.001.00115	51	Female	Yes	Yes	.	.	.	.	.	.	.	.
030.001.00116	52	Male	Yes	Yes	.	.	.	.	.	.	.	.
030.001.00119	20	Male	Yes	Yes	.	.	.	.	.	.	.	.
030.001.00124	29	Male	Yes	Yes	.	.	.	.	.	.	.	.
030.001.00125	22	Male	Yes	Yes	.	.	.	.	.	.	.	.
030.001.00127	19	Male	Yes	No	.	.	.	.	.	.	.	X
030.001.00131	50	Male	Yes	Yes	.	.	.	.	.	.	.	.

Note: Protocol Violations: X = Fail
Tafenoquine = Tafenoquine 200mg, Mefloquine = Mefloquine 250mg

SB252263 - 030

Listing B.06

All Randomised Subjects: Reasons For Exclusion From The Per Protocol Population

Treatment : Placebo

Subject Number	Age (years)	Gender	Intent-To-Treat	Per Protcol	1	2	3	4	5	6	7	8
030.001.00136	51	Female	Yes	Yes	.	.	.	.	.	.	.	.
030.001.00137	33	Male	Yes	Yes	.	.	.	.	.	.	.	.
030.001.00140	23	Male	Yes	Yes	.	.	.	.	.	.	.	.
030.001.00141	51	Female	Yes	Yes	.	.	.	.	.	.	.	.
030.001.00145	45	Female	Yes	Yes	.	.	.	.	.	.	.	.
030.001.00151	53	Male	Yes	Yes	.	.	.	.	.	.	.	.
030.001.00152	50	Male	Yes	Yes	.	.	.	.	.	.	.	.
030.001.00154	46	Female	Yes	Yes	.	.	.	.	.	.	.	.
030.001.00160	30	Male	Yes	No	X	.	.	.	.	.	.	.
030.001.00162	29	Female	Yes	Yes	.	.	.	.	.	.	.	.
030.001.00164	22	Male	Yes	Yes	.	.	.	.	.	.	.	.
030.001.00166	18	Male	Yes	Yes	.	.	.	.	.	.	.	.
030.001.00169	19	Female	Yes	Yes	.	.	.	.	.	.	.	.
030.001.00172	30	Male	Yes	Yes	.	.	.	.	.	.	.	.
030.001.00174	30	Male	Yes	Yes	.	.	.	.	.	.	.	.
030.001.00175	47	Male	Yes	Yes	.	.	.	.	.	.	.	.
030.001.00182	28	Female	Yes	No	.	.	.	.	.	.	.	X
030.001.00183	25	Female	Yes	Yes	.	.	.	.	.	.	.	.
030.001.00191	21	Female	Yes	Yes	.	.	.	.	.	.	.	.
030.001.00192	19	Male	Yes	Yes	.	.	.	.	.	.	.	.
030.001.00193	19	Male	Yes	Yes	.	.	.	.	.	.	.	.
030.001.00197	31	Female	Yes	Yes	.	.	.	.	.	.	.	.
030.001.00202	21	Female	No	No	.	.	.	.	.	.	.	.
030.001.00204	48	Female	Yes	No	.	.	.	.	.	.	.	X
030.001.00208	19	Male	Yes	Yes	.	.	.	.	.	.	.	.
030.001.00211	18	Male	Yes	Yes	.	.	.	.	.	.	.	.
030.001.00212	28	Male	Yes	Yes	.	.	.	.	.	.	.	.
030.001.00215	32	Female	Yes	Yes	.	.	.	.	.	.	.	.
030.001.00219	41	Female	Yes	Yes	.	.	.	.	.	.	.	.
030.001.00222	30	Male	Yes	Yes	.	.	.	.	.	.	.	.
030.001.00225	41	Female	Yes	Yes	.	.	.	.	.	.	.	.
030.001.00226	46	Female	Yes	Yes	.	.	.	.	.	.	.	.
030.001.00229	24	Male	Yes	Yes	.	.	.	.	.	.	.	.
030.001.00234	17	Male	Yes	Yes	.	.	.	.	.	.	.	.
030.001.00236	35	Male	Yes	Yes	.	.	.	.	.	.	.	.
030.001.00238	20	Male	Yes	Yes	.	.	.	.	.	.	.	.
030.001.00242	30	Male	Yes	Yes	.	.	.	.	.	.	.	.
030.001.00247	21	Female	Yes	Yes	.	.	.	.	.	.	.	.
030.001.00248	18	Male	Yes	Yes	.	.	.	.	.	.	.	.
030.001.00251	49	Female	Yes	Yes	.	.	.	.	.	.	.	.
030.001.00253	56	Female	Yes	Yes	.	.	.	.	.	.	.	.
030.001.00256	37	Male	Yes	Yes	.	.	.	.	.	.	.	.
030.001.00257	24	Male	Yes	Yes	.	.	.	.	.	.	.	.
030.001.00260	19	Male	Yes	Yes	.	.	.	.	.	.	.	.

Note: Protocol Violations: X = Fail
Tafenoquine = Tafenoquine 200mg, Mefloquine = Mefloquine 250mg

SB252263 - 030

Listing B.06

All Randomised Subjects: Reasons For Exclusion From The Per Protocol Population

Treatment : Placebo

Subject Number	Age (years)	Gender	Intent-To-Treat	Per Protcol	1	2	3	4	5	6	7	8
030.001.00268	24	Male	Yes	Yes	.	.	.	.	.	.	.	.
030.001.00269	19	Male	Yes	Yes	.	.	.	.	.	.	.	.
030.001.00270	22	Male	Yes	Yes	.	.	.	.	.	.	.	.
030.001.00275	50	Female	Yes	Yes	.	.	.	.	.	.	.	.
030.001.00277	31	Male	Yes	Yes	.	.	.	.	.	.	.	.
030.001.00278	32	Male	Yes	Yes	.	.	.	.	.	.	.	.
030.001.00285	39	Male	Yes	Yes	.	.	.	.	.	.	.	.
030.001.00287	48	Male	Yes	Yes	.	.	.	.	.	.	.	.
030.001.00291	18	Male	Yes	Yes	.	.	.	.	.	.	.	.
030.001.00295	48	Female	No	No	.	.	.	.	.	.	.	.
030.001.00297	43	Female	Yes	Yes	.	.	.	.	.	.	.	.
030.001.00300	21	Male	Yes	Yes	.	.	.	.	.	.	.	.
030.001.00303	18	Female	Yes	Yes	.	.	.	.	.	.	.	.

Note: Protocol Violations: X = Fail
Tafenoquine = Tafenoquine 200mg, Mefloquine = Mefloquine 250mg

SB252263 - 030

Listing B.06

All Randomised Subjects: Reasons For Exclusion From The Per Protocol Population

Treatment : Tafenoquine

Subject Number	Age (years)	Gender	Intent-To-Treat	Per Protcol	1	2	3	4	5	6	7	8
030.001.00001	39	Female	Yes	Yes	.	.	.	.	.	.	.	.
030.001.00004	18	Female	Yes	Yes	.	.	.	.	.	.	.	.
030.001.00006	33	Female	Yes	Yes	.	.	.	.	.	.	.	.
030.001.00009	45	Male	Yes	Yes	.	.	.	.	.	.	.	.
030.001.00014	20	Male	Yes	Yes	.	.	.	.	.	.	.	.
030.001.00016	52	Male	Yes	Yes	.	.	.	.	.	.	.	.
030.001.00017	26	Male	Yes	Yes	.	.	.	.	.	.	.	.
030.001.00024	48	Female	Yes	Yes	.	.	.	.	.	.	.	.
030.001.00025	18	Male	Yes	Yes	.	.	.	.	.	.	.	.
030.001.00028	38	Male	Yes	Yes	.	.	.	.	.	.	.	.
030.001.00029	18	Male	Yes	Yes	.	.	.	.	.	.	.	.
030.001.00030	42	Female	Yes	Yes	.	.	.	.	.	.	.	.
030.001.00040	18	Female	Yes	Yes	.	.	.	.	.	.	.	.
030.001.00042	19	Male	Yes	Yes	.	.	.	.	.	.	.	.
030.001.00043	25	Male	Yes	Yes	.	.	.	.	.	.	.	.
030.001.00048	40	Male	Yes	Yes	.	.	.	.	.	.	.	.
030.001.00050	26	Male	Yes	Yes	.	.	.	.	.	.	.	.
030.001.00051	18	Male	Yes	Yes	.	.	.	.	.	.	.	.
030.001.00058	51	Male	Yes	Yes	.	.	.	.	.	.	.	.
030.001.00060	44	Female	Yes	Yes	.	.	.	.	.	.	.	.
030.001.00061	40	Male	Yes	Yes	.	.	.	.	.	.	.	.
030.001.00065	38	Male	Yes	Yes	.	.	.	.	.	.	.	.
030.001.00068	20	Male	Yes	Yes	.	.	.	.	.	.	.	.
030.001.00070	32	Female	Yes	Yes	.	.	.	.	.	.	.	.
030.001.00077	43	Male	Yes	Yes	.	.	.	.	.	.	.	.
030.001.00079	21	Male	Yes	Yes	.	.	.	.	.	.	.	.
030.001.00082	36	Male	No	No	.	.	.	.	.	.	.	.
030.001.00084	42	Male	Yes	Yes	.	.	.	.	.	.	.	.
030.001.00085	51	Female	Yes	Yes	.	.	.	.	.	.	.	.
030.001.00086	26	Male	Yes	Yes	.	.	.	.	.	.	.	.
030.001.00093	25	Female	Yes	Yes	.	.	.	.	.	.	.	.
030.001.00094	27	Male	Yes	No	X	.	.	.	.	.	.	.
030.001.00100	17	Female	Yes	Yes	.	.	.	.	.	.	.	.
030.001.00102	33	Male	Yes	Yes	.	.	.	.	.	.	.	.
030.001.00104	36	Male	Yes	Yes	.	.	.	.	.	.	.	.
030.001.00107	51	Female	Yes	Yes	.	.	.	.	.	.	.	.
030.001.00109	33	Male	Yes	Yes	.	.	.	.	.	.	.	.
030.001.00111	27	Male	Yes	Yes	.	.	.	.	.	.	.	.
030.001.00112	26	Female	Yes	Yes	.	.	.	.	.	.	.	.
030.001.00118	25	Male	Yes	Yes	.	.	.	.	.	.	.	.
030.001.00122	36	Male	Yes	Yes	.	.	.	.	.	.	.	.
030.001.00126	24	Male	Yes	Yes	.	.	.	.	.	.	.	.
030.001.00128	25	Female	Yes	Yes	.	.	.	.	.	.	.	.
030.001.00129	50	Female	Yes	Yes	.	.	.	.	.	.	.	.

Note: Protocol Violations: X = Fail
Tafenoquine = Tafenoquine 200mg, Mefloquine = Mefloquine 250mg

SB252263 - 030

Listing B.06

All Randomised Subjects: Reasons For Exclusion From The Per Protocol Population

Treatment : Tafenoquine

Subject Number	Age (years)	Gender	Intent-To-Treat	Per Protcol	1	2	3	4	5	6	7	8
030.001.00133	19	Male	Yes	Yes	.	.	.	.	.	.	.	.
030.001.00135	23	Male	Yes	Yes	.	.	.	.	.	.	.	.
030.001.00138	31	Male	Yes	Yes	.	.	.	.	.	.	.	.
030.001.00139	22	Male	Yes	Yes	.	.	.	.	.	.	.	.
030.001.00148	22	Male	Yes	Yes	.	.	.	.	.	.	.	.
030.001.00149	20	Male	Yes	Yes	.	.	.	.	.	.	.	.
030.001.00150	36	Female	Yes	Yes	.	.	.	.	.	.	.	.
030.001.00155	49	Female	Yes	Yes	.	.	.	.	.	.	.	.
030.001.00157	19	Male	Yes	Yes	.	.	.	.	.	.	.	.
030.001.00158	20	Female	Yes	Yes	.	.	.	.	.	.	.	.
030.001.00159	19	Female	Yes	Yes	.	.	.	.	.	.	.	.
030.001.00161	34	Female	Yes	Yes	.	.	.	.	.	.	.	.
030.001.00171	54	Male	Yes	Yes	.	.	.	.	.	.	.	.
030.001.00173	19	Female	Yes	No	.	.	.	.	.	.	.	X
030.001.00176	22	Female	Yes	Yes	.	.	.	.	.	.	.	.
030.001.00179	23	Male	Yes	No	.	.	.	.	.	.	.	X
030.001.00184	20	Male	Yes	Yes	.	.	.	.	.	.	.	.
030.001.00186	24	Male	Yes	Yes	.	.	.	.	.	.	.	.
030.001.00187	20	Male	Yes	No	.	.	.	.	.	.	.	X
030.001.00190	22	Male	Yes	Yes	.	.	.	.	.	.	.	.
030.001.00194	50	Male	Yes	Yes	.	.	.	.	.	.	.	.
030.001.00195	21	Male	Yes	Yes	.	.	.	.	.	.	.	.
030.001.00201	50	Female	Yes	Yes	.	.	.	.	.	.	.	.
030.001.00203	32	Female	Yes	Yes	.	.	.	.	.	.	.	.
030.001.00205	44	Female	Yes	Yes	.	.	.	.	.	.	.	.
030.001.00207	19	Male	Yes	Yes	.	.	.	.	.	.	.	.
030.001.00209	30	Male	Yes	Yes	.	.	.	.	.	.	.	.
030.001.00210	21	Male	Yes	Yes	.	.	.	.	.	.	.	.
030.001.00217	23	Male	Yes	Yes	.	.	.	.	.	.	.	.
030.001.00218	24	Male	Yes	Yes	.	.	.	.	.	.	.	.
030.001.00220	30	Male	Yes	Yes	.	.	.	.	.	.	.	.
030.001.00228	22	Male	Yes	Yes	.	.	.	.	.	.	.	.
030.001.00230	19	Male	Yes	Yes	.	.	.	.	.	.	.	.
030.001.00231	25	Female	Yes	Yes	.	.	.	.	.	.	.	.
030.001.00233	48	Female	Yes	Yes	.	.	.	.	.	.	.	.
030.001.00240	18	Male	Yes	Yes	.	.	.	.	.	.	.	.
030.001.00243	26	Male	Yes	Yes	.	.	.	.	.	.	.	.
030.001.00246	51	Female	Yes	Yes	.	.	.	.	.	.	.	.
030.001.00249	24	Male	Yes	Yes	.	.	.	.	.	.	.	.
030.001.00252	18	Male	Yes	Yes	.	.	.	.	.	.	.	.
030.001.00254	19	Female	Yes	No	.	.	.	.	.	.	.	X
030.001.00259	26	Male	Yes	Yes	.	.	.	.	.	.	.	.
030.001.00261	31	Female	No	No	.	.	.	.	.	.	.	.
030.001.00264	24	Female	Yes	Yes	.	.	.	.	.	.	.	.

Note: Protocol Violations: X = Fail
Tafenoquine = Tafenoquine 200mg, Mefloquine = Mefloquine 250mg

SB252263 - 030

Listing B.06

All Randomised Subjects: Reasons For Exclusion From The Per Protocol Population

Treatment : Tafenoquine

Subject Number	Age (years)	Gender	Intent-To-Treat	Per Protcol	1	2	3	4	5	6	7	8
030.001.00266	30	Male	Yes	No	.	.	.	.	.	.	.	X
030.001.00267	22	Male	Yes	Yes	.	.	.	.	.	.	.	.
030.001.00271	53	Male	Yes	No	.	.	.	.	.	.	.	X
030.001.00272	35	Female	Yes	Yes	.	.	.	.	.	.	.	.
030.001.00279	29	Male	Yes	Yes	.	.	.	.	.	.	.	.
030.001.00282	50	Female	Yes	Yes	.	.	.	.	.	.	.	.
030.001.00284	18	Male	Yes	Yes	.	.	.	.	.	.	.	.
030.001.00288	22	Male	Yes	Yes	.	.	.	.	.	.	.	.
030.001.00293	21	Female	Yes	Yes	.	.	.	.	.	.	.	.
030.001.00294	24	Female	Yes	Yes	.	.	.	.	.	.	.	.
030.001.00296	44	Female	Yes	Yes	.	.	.	.	.	.	.	.
030.001.00298	17	Male	Yes	Yes	.	.	.	.	.	.	.	.
030.001.00301	18	Male	Yes	Yes	.	.	.	.	.	.	.	.
030.001.00302	18	Female	Yes	No	.	.	.	.	X	.	.	.
030.001.00304	19	Male	Yes	Yes	.	.	.	.	.	.	.	.
030.001.00306	24	Male	Yes	Yes	.	.	.	.	.	.	.	.

Note: Protocol Violations: X = Fail
Tafenoquine = Tafenoquine 200mg, Mefloquine = Mefloquine 250mg

SB252263 - 030

Listing B.06

All Randomised Subjects: Reasons For Exclusion From The Per Protocol Population

Treatment : Mefloquine

Subject Number	Age (years)	Gender	Intent-To-Treat	Per Protcol	1	2	3	4	5	6	7	8
030.001.00003	33	Female	Yes	No	.	.	.	.	.	.	.	X
030.001.00007	24	Female	Yes	Yes	.	.	.	.	.	.	.	.
030.001.00008	40	Male	Yes	Yes	.	.	.	.	.	.	.	.
030.001.00010	51	Female	Yes	Yes	.	.	.	.	.	.	.	.
030.001.00013	30	Male	Yes	Yes	.	.	.	.	.	.	.	.
030.001.00018	26	Female	Yes	Yes	.	.	.	.	.	.	.	.
030.001.00021	41	Female	Yes	Yes	.	.	.	.	.	.	.	.
030.001.00023	44	Female	Yes	No	.	.	.	.	.	.	.	X
030.001.00031	41	Male	Yes	Yes	.	.	.	.	.	.	.	.
030.001.00034	20	Male	Yes	Yes	.	.	.	.	.	.	.	.
030.001.00035	18	Male	No	No	.	.	.	.	.	.	.	.
030.001.00036	36	Male	Yes	No	.	.	.	.	.	.	.	X
030.001.00038	18	Male	Yes	Yes	.	.	.	.	.	.	.	.
030.001.00045	38	Male	Yes	Yes	.	.	.	.	.	.	.	.
030.001.00046	26	Male	Yes	Yes	.	.	.	.	.	.	.	.
030.001.00047	18	Male	Yes	Yes	.	.	.	.	.	.	.	.
030.001.00049	34	Male	Yes	Yes	.	.	.	.	.	.	.	.
030.001.00052	44	Female	Yes	Yes	.	.	.	.	.	.	.	.
030.001.00053	27	Male	Yes	Yes	.	.	.	.	.	.	.	.
030.001.00054	18	Male	Yes	Yes	.	.	.	.	.	.	.	.
030.001.00062	19	Male	Yes	Yes	.	.	.	.	.	.	.	.
030.001.00066	52	Female	Yes	Yes	.	.	.	.	.	.	.	.
030.001.00069	18	Male	Yes	No	X	.	.	.	.	.	.	.
030.001.00071	55	Female	Yes	Yes	.	.	.	.	.	.	.	.
030.001.00073	48	Male	Yes	Yes	.	.	.	.	.	.	.	.
030.001.00074	25	Male	Yes	Yes	.	.	.	.	.	.	.	.
030.001.00075	43	Female	Yes	Yes	.	.	.	.	.	.	.	.
030.001.00083	29	Male	Yes	Yes	.	.	.	.	.	.	.	.
030.001.00089	19	Male	Yes	Yes	.	.	.	.	.	.	.	.
030.001.00091	30	Male	Yes	Yes	.	.	.	.	.	.	.	.
030.001.00095	42	Male	Yes	Yes	.	.	.	.	.	.	.	.
030.001.00096	18	Male	Yes	Yes	.	.	.	.	.	.	.	.
030.001.00097	41	Female	Yes	Yes	.	.	.	.	.	.	.	.
030.001.00098	20	Male	Yes	Yes	.	.	.	.	.	.	.	.
030.001.00099	22	Male	Yes	Yes	.	.	.	.	.	.	.	.
030.001.00105	26	Male	Yes	Yes	.	.	.	.	.	.	.	.
030.001.00110	20	Male	Yes	Yes	.	.	.	.	.	.	.	.
030.001.00113	24	Female	Yes	Yes	.	.	.	.	.	.	.	.
030.001.00117	40	Female	Yes	Yes	.	.	.	.	.	.	.	.
030.001.00120	37	Male	Yes	Yes	.	.	.	.	.	.	.	.
030.001.00121	38	Male	Yes	Yes	.	.	.	.	.	.	.	.
030.001.00123	24	Male	Yes	No	.	.	.	.	.	.	.	X
030.001.00130	25	Male	Yes	Yes	.	.	.	.	.	.	.	.
030.001.00132	19	Male	Yes	Yes	.	.	.	.	.	.	.	.

Note: Protocol Violations: X = Fail
Tafenoquine = Tafenoquine 200mg, Mefloquine = Mefloquine 250mg

SB252263 - 030

Listing B.06

All Randomised Subjects: Reasons For Exclusion From The Per Protocol Population

Treatment : Mefloquine

Subject Number	Age (years)	Gender	Intent-To-Treat	Per Protcol	1	2	3	4	5	6	7	8
030.001.00134	27	Female	Yes	Yes	.	.	.	.	.	.	.	.
030.001.00142	18	Male	Yes	Yes	.	.	.	.	.	.	.	.
030.001.00143	17	Male	Yes	Yes	.	.	.	.	.	.	.	.
030.001.00144	21	Male	Yes	Yes	.	.	.	.	.	.	.	.
030.001.00146	18	Male	Yes	No	.	.	.	.	.	.	.	X
030.001.00147	33	Male	Yes	Yes	.	.	.	.	.	.	.	.
030.001.00153	20	Male	Yes	Yes	.	.	.	.	.	.	.	.
030.001.00156	20	Female	Yes	Yes	.	.	.	.	.	.	.	.
030.001.00163	27	Female	Yes	Yes	.	.	.	.	.	.	.	.
030.001.00165	38	Male	Yes	Yes	.	.	.	.	.	.	.	.
030.001.00167	38	Female	Yes	Yes	.	.	.	.	.	.	.	.
030.001.00168	28	Female	Yes	Yes	.	.	.	.	.	.	.	.
030.001.00170	34	Male	Yes	Yes	.	.	.	.	.	.	.	.
030.001.00177	18	Female	Yes	Yes	.	.	.	.	.	.	.	.
030.001.00178	27	Male	Yes	No	.	.	.	.	X	.	.	X
030.001.00180	55	Female	Yes	Yes	.	.	.	.	.	.	.	.
030.001.00181	18	Male	Yes	Yes	.	.	.	.	.	.	.	.
030.001.00185	18	Female	Yes	Yes	.	.	.	.	.	.	.	.
030.001.00188	35	Female	Yes	Yes	.	.	.	.	.	.	.	.
030.001.00189	50	Female	Yes	No	.	.	.	X	.	.	.	.
030.001.00196	37	Male	Yes	Yes	.	.	.	.	.	.	.	.
030.001.00198	52	Female	Yes	Yes	.	.	.	.	.	.	.	.
030.001.00199	20	Male	Yes	Yes	.	.	.	.	.	.	.	.
030.001.00200	18	Male	Yes	Yes	.	.	.	.	.	.	.	.
030.001.00206	23	Male	Yes	No	.	.	.	.	.	.	.	X
030.001.00213	28	Male	Yes	Yes	.	.	.	.	.	.	.	.
030.001.00214	43	Female	Yes	Yes	.	.	.	.	.	.	.	.
030.001.00216	21	Male	Yes	Yes	.	.	.	.	.	.	.	.
030.001.00221	41	Male	Yes	Yes	.	.	.	.	.	.	.	.
030.001.00223	23	Female	Yes	Yes	.	.	.	.	.	.	.	.
030.001.00224	41	Female	Yes	Yes	.	.	.	.	.	.	.	.
030.001.00227	29	Female	Yes	Yes	.	.	.	.	.	.	.	.
030.001.00232	34	Female	Yes	Yes	.	.	.	.	.	.	.	.
030.001.00235	45	Female	Yes	Yes	.	.	.	.	.	.	.	.
030.001.00237	25	Male	Yes	Yes	.	.	.	.	.	.	.	.
030.001.00239	25	Male	Yes	Yes	.	.	.	.	.	.	.	.
030.001.00241	20	Male	Yes	Yes	.	.	.	.	.	.	.	.
030.001.00244	23	Male	Yes	Yes	.	.	.	.	.	.	.	.
030.001.00245	24	Male	Yes	Yes	.	.	.	.	.	.	.	.
030.001.00250	18	Male	No	No	.	.	.	.	.	.	.	.
030.001.00255	19	Male	Yes	Yes	.	.	.	.	.	.	.	.
030.001.00258	24	Male	Yes	Yes	.	.	.	.	.	.	.	.
030.001.00262	29	Male	Yes	Yes	.	.	.	.	.	.	.	.
030.001.00263	45	Male	Yes	Yes	.	.	.	.	.	.	.	.

Note: Protocol Violations: X = Fail
Tafenoquine = Tafenoquine 200mg, Mefloquine = Mefloquine 250mg

SB252263 - 030

Listing B.06

All Randomised Subjects: Reasons For Exclusion From The Per Protocol Population

Treatment : Mefloquine

Subject Number	Age (years)	Gender	Intent-To-Treat	Per Protcol	1	2	3	4	5	6	7	8
030.001.00265	19	Male	Yes	Yes	.	.	.	.	.	.	.	.
030.001.00273	20	Female	Yes	Yes	.	.	.	.	.	.	.	.
030.001.00274	35	Female	Yes	Yes	.	.	.	.	.	.	.	.
030.001.00276	36	Male	Yes	Yes	.	.	.	.	.	.	.	.
030.001.00280	18	Male	Yes	Yes	.	.	.	.	.	.	.	.
030.001.00281	31	Male	Yes	Yes	.	.	.	.	.	.	.	.
030.001.00283	31	Male	Yes	Yes	.	.	.	.	.	.	.	.
030.001.00286	28	Male	Yes	Yes	.	.	.	.	.	.	.	.
030.001.00289	19	Female	Yes	Yes	.	.	.	.	.	.	.	.
030.001.00290	18	Male	Yes	Yes	.	.	.	.	.	.	.	.
030.001.00292	19	Male	Yes	Yes	.	.	.	.	.	.	.	.
030.001.00299	17	Male	Yes	Yes	.	.	.	.	.	.	.	.
030.001.00305	31	Male	Yes	Yes	.	.	.	.	.	.	.	.

Note: Protocol Violations: X = Fail
Tafenoquine = Tafenoquine 200mg, Mefloquine = Mefloquine 250mg

SB252263 - 030

Listing B.07

Inclusion And Exclusion Criteria Violations

Inclusion/Exclusion Criteria Codelist

Code	Description
1	Subj is volunteer(male or female)providing informed consent
2	Subj is aged between 18 and 55 years old
3	Subject will be residing in study area for study duration
4	Subj unwilling to report drug admin.or blood drawing during 70wk study
5	Subj has med.cond.in opinion of invest.makes subj unsuitable to enter study
6	Clin.sign.abnormalities as specified by protocol
7	Subject has a personal or family history of seizures
8	Subject has a history of Psychiatric disorders
9	Subj is female & is pregnant or lactating or who is at risk of pregnancy
10	Subject has a positive serum or Urine B-HCG (Screening)
11	Subject has a positive serum or Urine B-HCG (Randomisation)
12	Has subject demonstrated prev.hypersens.to any drug, esp. 8-aminoquinolines?
13	Subj taking/taken other anti-malarial product/drug except Halofantrine
14	Subj has abnormal EKG particular an extended QTC interval > 0.42 sec
15	Has subject rec. invest.drug within 30 days or 5 half-lives (longer)?
16	Subj has pos. parasitemia following Halofantrine treatment for radical cure
17	Is subject Glucose-6-phosphate dehydrogenase (G6PD) deficient?
18	Subj lab val are: Hb<10gm, PLT<80000mm3, WBC<3000ul, CREAT or ALT >2X upper NR

SB252263 - 030

Listing B.07

Inclusion And Exclusion Criteria Violations

Treatment : Placebo

Subject Number	Popn*	1	2	3	4	5	6	7	8	9	10	11	12	13	14	15	16	17	18
030.001.00002	IPP	.	.	.	.	.	.	.	.	.	.	.	.	.	.	.	.	.	.
030.001.00005	IPP	.	.	.	.	.	.	.	.	.	.	.	.	.	.	.	.	.	.
030.001.00011	IPP	.	.	.	.	.	.	.	.	.	.	.	.	.	.	.	.	.	.
030.001.00012	IPP	.	.	.	.	.	.	.	.	.	.	.	.	.	.	.	.	.	.
030.001.00015	I	.	.	.	.	.	.	.	.	.	.	.	.	.	.	.	.	.	.
030.001.00019	IPP	.	.	.	.	.	.	.	.	.	.	.	.	.	.	.	.	.	.
030.001.00020	IPP	.	.	.	.	.	.	.	.	.	.	.	.	.	.	.	.	.	.
030.001.00022	IPP	.	.	.	.	.	.	.	.	.	.	.	.	.	.	.	.	.	.
030.001.00026	IPP	.	.	.	.	.	.	.	.	.	.	.	.	.	.	.	.	.	.
030.001.00027	IPP	.	.	.	.	.	.	.	.	.	.	.	.	.	.	.	.	.	.
030.001.00032	IPP	.	.	.	.	.	.	.	.	.	.	.	.	.	.	.	.	.	.
030.001.00033	IPP	.	.	.	.	.	.	.	.	.	.	.	.	.	.	.	.	.	.
030.001.00037	IPP	.	.	.	.	.	.	.	.	.	.	.	.	.	.	.	.	.	.
030.001.00039	IPP	.	.	.	.	.	.	.	.	.	.	.	.	.	.	.	.	.	.
030.001.00041	IPP	.	.	.	.	.	.	.	.	.	.	.	.	.	.	.	.	.	.
030.001.00044	IPP	.	.	.	.	.	.	.	.	.	.	.	.	.	.	.	.	.	.
030.001.00055	IPP	.	.	.	.	.	.	.	.	.	.	.	.	.	.	.	.	.	.
030.001.00056	I	.	.	.	.	.	.	.	.	.	.	.	.	.	.	.	.	.	.
030.001.00057	IPP	.	.	.	.	.	.	.	.	.	.	.	.	.	.	.	.	.	.
030.001.00059	IPP	.	.	.	.	.	.	.	.	.	.	.	.	.	.	.	.	.	.
030.001.00063	IPP	.	.	.	.	.	.	.	.	.	.	.	.	.	.	.	.	.	.
030.001.00064	IPP	.	.	.	.	.	.	.	.	.	.	.	.	.	.	.	.	.	.
030.001.00067	IPP	.	.	.	.	.	.	.	.	.	.	.	.	.	.	.	.	.	.
030.001.00072	IPP	.	.	.	.	.	.	.	.	.	.	.	.	.	.	.	.	.	.
030.001.00076	IPP	.	.	.	.	.	.	.	.	.	.	.	.	.	.	.	.	.	.
030.001.00078	IPP	.	.	.	.	.	.	.	.	.	.	.	.	.	.	.	.	.	.
030.001.00080	IPP	.	.	.	.	.	.	.	.	.	.	.	.	.	.	.	.	.	.
030.001.00081	IPP	.	.	.	.	.	.	.	.	.	.	.	.	.	.	.	.	.	.
030.001.00087	I	.	.	.	.	.	.	.	.	.	.	.	.	.	.	.	.	.	.
030.001.00088	IPP	.	.	.	.	.	.	.	.	.	.	.	.	.	.	.	.	.	.
030.001.00090	IPP	.	.	.	.	.	.	.	.	.	.	.	.	.	.	.	.	.	.
030.001.00092	IPP	.	.	.	.	.	.	.	.	.	.	.	.	.	.	.	.	.	.
030.001.00101	IPP	.	.	.	.	.	.	.	.	.	.	.	.	.	.	.	.	.	.
030.001.00103	IPP	.	.	.	.	.	.	.	.	.	.	.	.	.	.	.	.	.	.
030.001.00106	IPP	.	.	.	.	.	.	.	.	.	.	.	.	.	.	.	.	.	.
030.001.00108	IPP	.	.	.	.	.	.	.	.	.	.	.	.	.	.	.	.	.	.
030.001.00114	IPP	.	.	.	.	.	.	.	.	.	.	.	.	.	.	.	.	.	.
030.001.00115	IPP	.	.	.	.	.	.	.	.	.	.	.	.	.	.	.	.	.	.
030.001.00116	IPP	.	.	.	.	.	.	.	.	.	.	.	.	.	.	.	.	.	.
030.001.00119	IPP	.	.	.	.	.	.	.	.	.	.	.	.	.	.	.	.	.	.
030.001.00124	IPP	.	.	.	.	.	.	.	.	.	.	.	.	.	.	.	.	.	.
030.001.00125	IPP	.	.	.	.	.	.	.	.	.	.	.	.	.	.	.	.	.	.
030.001.00127	I	.	.	.	.	.	.	.	.	.	.	.	.	.	.	.	.	.	.
030.001.00131	IPP	.	.	.	.	.	.	.	.	.	.	.	.	.	.	.	.	.	.

*Popn=Population (I = Intent-To-Treat, PP = Per Protocol)
Note: X = Violation
Tafenoquine = Tafenoquine 200mg, Mefloquine = Mefloquine 250mg

SB252263 - 030

Listing B.07

Inclusion And Exclusion Criteria Violations

Treatment : Placebo

Subject Number	Popn*	1	2	3	4	5	6	7	8	9	10	11	12	13	14	15	16	17	18
030.001.00136	IPP	.	.	.	.	.	.	.	.	.	.	.	.	.	.	.	.	.	.
030.001.00137	IPP	.	.	.	.	.	.	.	.	.	.	.	.	.	.	.	.	.	.
030.001.00140	IPP	.	.	.	.	.	.	.	.	.	.	.	.	.	.	.	.	.	.
030.001.00141	IPP	.	.	.	.	.	.	.	.	.	.	.	.	.	.	.	.	.	.
030.001.00145	IPP	.	.	.	.	.	.	.	.	.	.	.	.	.	.	.	.	.	.
030.001.00151	IPP	.	.	.	.	.	.	.	.	.	.	.	.	.	.	.	.	.	.
030.001.00152	IPP	.	.	.	.	.	.	.	.	.	.	.	.	.	.	.	.	.	.
030.001.00154	IPP	.	.	.	.	.	.	.	.	.	.	.	.	.	.	.	.	.	.
030.001.00160	I	.	.	.	.	.	.	.	.	.	.	.	.	.	.	.	.	.	.
030.001.00162	IPP	.	.	.	.	.	.	.	.	.	.	.	.	.	.	.	.	.	.
030.001.00164	IPP	.	.	.	.	.	.	.	.	.	.	.	.	.	.	.	.	.	.
030.001.00166	IPP	.	.	.	.	.	.	.	.	.	.	.	.	.	.	.	.	.	.
030.001.00169	IPP	.	.	.	.	.	.	.	.	.	.	.	.	.	.	.	.	.	.
030.001.00172	IPP	.	.	.	.	.	.	.	.	.	.	.	.	.	.	.	.	.	.
030.001.00174	IPP	.	.	.	.	.	.	.	.	.	.	.	.	.	.	.	.	.	.
030.001.00175	IPP	.	.	.	.	.	.	.	.	.	.	.	.	.	.	.	.	.	.
030.001.00182	I	.	.	.	.	.	.	.	.	.	.	.	.	.	.	.	.	.	.
030.001.00183	IPP	.	.	.	.	.	.	.	.	.	.	.	.	.	.	.	.	.	.
030.001.00191	IPP	.	.	.	.	.	.	.	.	.	.	.	.	.	.	.	.	.	.
030.001.00192	IPP	.	.	.	.	.	.	.	.	.	.	.	.	.	.	.	.	.	.
030.001.00193	IPP	.	.	.	.	.	.	.	.	.	.	.	.	.	.	.	.	.	.
030.001.00197	IPP	.	.	.	.	.	.	.	.	.	.	.	.	.	.	.	.	.	.
030.001.00202		.	.	.	.	.	.	.	.	.	.	.	.	.	.	.	.	.	.
030.001.00204	I	.	.	.	.	.	.	.	.	.	.	.	.	.	.	.	.	.	.
030.001.00208	IPP	.	.	.	.	.	.	.	.	.	.	.	.	.	.	.	.	.	.
030.001.00211	IPP	.	.	.	.	.	.	.	.	.	.	.	.	.	.	.	.	.	.
030.001.00212	IPP	.	.	.	.	.	.	.	.	.	.	.	.	.	.	.	.	.	.
030.001.00215	IPP	.	.	.	.	.	.	.	.	.	.	.	.	.	.	.	.	.	.
030.001.00219	IPP	.	.	.	.	.	.	.	.	.	.	.	.	.	.	.	.	.	.
030.001.00222	IPP	.	.	.	.	.	.	.	.	.	.	.	.	.	.	.	.	.	.
030.001.00225	IPP	.	.	.	.	.	.	.	.	.	.	.	.	.	.	.	.	.	.
030.001.00226	IPP	.	.	.	.	.	.	.	.	.	.	.	.	.	.	.	.	.	.
030.001.00229	IPP	.	.	.	.	.	.	.	.	.	.	.	.	.	.	.	.	.	.
030.001.00234	IPP	.	.	.	.	.	.	.	.	.	.	.	.	.	.	.	.	.	.
030.001.00236	IPP	.	.	.	.	.	.	.	.	.	.	.	.	.	.	.	.	.	.
030.001.00238	IPP	.	.	.	.	.	.	.	.	.	.	.	.	.	.	.	.	.	.
030.001.00242	IPP	.	.	.	.	.	.	.	.	.	.	.	.	.	.	.	.	.	.
030.001.00247	IPP	.	.	.	.	.	.	.	.	.	.	.	.	.	.	.	.	.	.
030.001.00248	IPP	.	.	.	.	.	.	.	.	.	.	.	.	.	.	.	.	.	.
030.001.00251	IPP	.	.	.	.	.	.	.	.	.	.	.	.	.	.	.	.	.	.
030.001.00253	IPP	.	.	.	.	.	.	.	.	.	.	.	.	.	.	.	.	.	.
030.001.00256	IPP	.	.	.	.	.	.	.	.	.	.	.	.	.	.	.	.	.	.
030.001.00257	IPP	.	.	.	.	.	.	.	.	.	.	.	.	.	.	.	.	.	.
030.001.00260	IPP	.	.	.	.	.	.	.	.	.	.	.	.	.	.	.	.	.	.

*Popn=Population (I = Intent-To-Treat, PP = Per Protocol)
Note: X = Violation
Tafenoquine = Tafenoquine 200mg, Mefloquine = Mefloquine 250mg

SB252263 - 030

Listing B.07

Inclusion And Exclusion Criteria Violations

Treatment : Placebo

Subject Number	Popn*	1	2	3	4	5	6	7	8	9	10	11	12	13	14	15	16	17	18
030.001.00268	IPP	.	.	.	.	.	.	.	.	.	.	.	.	.	.	.	.	.	.
030.001.00269	IPP	.	.	.	.	.	.	.	.	.	.	.	.	.	.	.	.	.	.
030.001.00270	IPP	.	.	.	.	.	.	.	.	.	.	.	.	.	.	.	.	.	.
030.001.00275	IPP	.	.	.	.	.	.	.	.	.	.	.	.	.	.	.	.	.	.
030.001.00277	IPP	.	.	.	.	.	.	.	.	.	.	.	.	.	.	.	.	.	.
030.001.00278	IPP	.	.	.	.	.	.	.	.	.	.	.	.	.	.	.	.	.	.
030.001.00285	IPP	.	.	.	.	.	.	.	.	.	.	.	.	.	.	.	.	.	.
030.001.00287	IPP		.	.	.	.	.	.	.	.	.	.	.	.	.	.	.	.	.
030.001.00291	IPP	.	.	.	.	.	.	.	.	.	.	.	.	.	.	.	.	.	.
030.001.00295		.	.	.	.	.	.	.	.	.	.	.	.	.	.	.	.	.	.
030.001.00297	IPP	.	.	.	.	.	.	.	.	.	.	.	.	.	.	.	.	.	.
030.001.00300	IPP	.	.	.	.	.	.	.	.	.	.	.	.	.	.	.	.	.	.
030.001.00303	IPP	.	.	.	.	.	.	.	.	.	.	.	.	.	.	.	.	.	.

*Popn=Population (I = Intent-To-Treat, PP = Per Protocol)
Note: X = Violation
Tafenoquine = Tafenoquine 200mg, Mefloquine = Mefloquine 250mg

SB252263 - 030

Listing B.07

Inclusion And Exclusion Criteria Violations

Treatment : Tafenoquine

Subject Number	Popn*	1	2	3	4	5	6	7	8	9	10	11	12	13	14	15	16	17	18
030.001.00001	IPP	.	.	.	.	.	.	.	.	.	.	.	.	.	.	.	.	.	.
030.001.00004	IPP	.	.	.	.	.	.	.	.	.	.	.	.	.	.	.	.	.	.
030.001.00006	IPP	.	.	.	.	.	.	.	.	.	.	.	.	.	.	.	.	.	.
030.001.00009	IPP	.	.	.	.	.	.	.	.	.	.	.	.	.	.	.	.	.	.
030.001.00014	IPP	.	.	.	.	.	.	.	.	.	.	.	.	.	.	.	.	.	.
030.001.00016	IPP	.	.	.	.	.	.	.	.	.	.	.	.	.	.	.	.	.	.
030.001.00017	IPP	.	.	.	.	.	.	.	.	.	.	.	.	.	.	.	.	.	.
030.001.00024	IPP	.	.	.	.	.	.	.	.	.	.	.	.	.	.	.	.	.	.
030.001.00025	IPP	.	.	.	.	.	.	.	.	.	.	.	.	.	.	.	.	.	.
030.001.00028	IPP	.	.	.	.	.	.	.	.	.	.	.	.	.	.	.	.	.	.
030.001.00029	IPP	.	.	.	.	.	.	.	.	.	.	.	.	.	.	.	.	.	.
030.001.00030	IPP	.	.	.	.	.	.	.	.	.	.	.	.	.	.	.	.	.	.
030.001.00040	IPP	.	.	.	.	.	.	.	.	.	.	.	.	.	.	.	.	.	.
030.001.00042	IPP	.	.	.	.	.	.	.	.	.	.	.	.	.	.	.	.	.	.
030.001.00043	IPP	.	.	.	.	.	.	.	.	.	.	.	.	.	.	.	.	.	.
030.001.00048	IPP	.	.	.	.	.	.	.	.	.	.	.	.	.	.	.	.	.	.
030.001.00050	IPP	.	.	.	.	.	.	.	.	.	.	.	.	.	.	.	.	.	.
030.001.00051	IPP	.	.	.	.	.	.	.	.	.	.	.	.	.	.	.	.	.	.
030.001.00058	IPP	.	.	.	.	.	.	.	.	.	.	.	.	.	.	.	.	.	.
030.001.00060	IPP	.	.	.	.	.	.	.	.	.	.	.	.	.	.	.	.	.	.
030.001.00061	IPP	.	.	.	.	.	.	.	.	.	.	.	.	.	.	.	.	.	.
030.001.00065	IPP	.	.	.	.	.	.	.	.	.	.	.	.	.	.	.	.	.	.
030.001.00068	IPP	.	.	.	.	.	.	.	.	.	.	.	.	.	.	.	.	.	.
030.001.00070	IPP	.	.	.	.	.	.	.	.	.	.	.	.	.	.	.	.	.	.
030.001.00077	IPP	.	.	.	.	.	.	.	.	.	.	.	.	.	.	.	.	.	.
030.001.00079	IPP	.	.	.	.	.	.	.	.	.	.	.	.	.	.	.	.	.	.
030.001.00082		.	.	.	.	.	.	.	.	.	.	.	.	.	.	.	.	.	.
030.001.00084	IPP	.	.	.	.	.	.	.	.	.	.	.	.	.	.	.	.	.	.
030.001.00085	IPP	.	.	.	.	.	.	.	.	.	.	.	.	.	.	.	.	.	.
030.001.00086	IPP	.	.	.	.	.	.	.	.	.	.	.	.	.	.	.	.	.	.
030.001.00093	IPP	.	.	.	.	.	.	.	.	.	.	.	.	.	.	.	.	.	.
030.001.00094	I	.	.	.	.	.	.	.	.	.	.	.	.	.	.	.	.	.	.
030.001.00100	IPP	.	X	.	.	.	.	.	.	.	.	.	.	.	.	.	.	.	.
030.001.00102	IPP	.	.	.	.	.	.	.	.	.	.	.	.	.	.	.	.	.	.
030.001.00104	IPP	.	.	.	.	.	.	.	.	.	.	.	.	.	.	.	.	.	.
030.001.00107	IPP	.	.	.	.	.	.	.	.	.	.	.	.	.	.	.	.	.	.
030.001.00109	IPP	.	.	.	.	.	.	.	.	.	.	.	.	.	.	.	.	.	.
030.001.00111	IPP	.	.	.	.	.	.	.	.	.	.	.	.	.	.	.	.	.	.
030.001.00112	IPP	.	.	.	.	.	.	.	.	.	.	.	.	.	.	.	.	.	.
030.001.00118	IPP	.	.	.	.	.	.	.	.	.	.	.	.	.	.	.	.	.	.
030.001.00122	IPP	.	.	.	.	.	.	.	.	.	.	.	.	.	.	.	.	.	.
030.001.00126	IPP	.	.	.	.	.	.	.	.	.	.	.	.	.	.	.	.	.	.
030.001.00128	IPP	.	.	.	.	.	.	.	.	.	.	.	.	.	.	.	.	.	.
030.001.00129	IPP	.	.	.	.	.	.	.	.	.	.	.	.	.	.	.	.	.	.

*Popn=Population (I = Intent-To-Treat, PP = Per Protocol)
Note: X = Violation
Tafenoquine = Tafenoquine 200mg, Mefloquine = Mefloquine 250mg

SB252263 - 030

Listing B.07

Inclusion And Exclusion Criteria Violations

Treatment : Tafenoquine

Subject Number	Popn*	1	2	3	4	5	6	7	8	9	10	11	12	13	14	15	16	17	18
030.001.00133	IPP	.	.	.	.	.	.	.	.	.	.	.	.	.	.	.	.	.	.
030.001.00135	IPP	.	.	.	.	.	.	.	.	.	.	.	.	.	.	.	.	.	.
030.001.00138	IPP	.	.	.	.	.	.	.	.	.	.	.	.	.	.	.	.	.	.
030.001.00139	IPP	.	.	.	.	.	.	.	.	.	.	.	.	.	.	.	.	.	.
030.001.00148	IPP	.	.	.	.	.	.	.	.	.	.	.	.	.	.	.	.	.	.
030.001.00149	IPP	.	.	.	.	.	.	.	.	.	.	.	.	.	.	.	.	.	.
030.001.00150	IPP	.	.	.	.	.	.	.	.	.	.	.	.	.	.	.	.	.	.
030.001.00155	IPP	.	.	.	.	.	.	.	.	.	.	.	.	.	.	.	.	.	.
030.001.00157	IPP	.	.	.	.	.	.	.	.	.	.	.	.	.	.	.	.	.	.
030.001.00158	IPP	.	.	.	.	.	.	.	.	.	.	.	.	.	.	.	.	.	.
030.001.00159	IPP	.	.	.	.	.	.	.	.	.	.	.	.	.	.	.	.	.	.
030.001.00161	IPP	.	.	.	.	.	.	.	.	.	.	.	.	.	.	.	.	.	.
030.001.00171	IPP	.	.	.	.	.	.	.	.	.	.	.	.	.	.	.	.	.	.
030.001.00173	I	.	.	.	.	.	.	.	.	.	.	.	.	.	.	.	.	.	.
030.001.00176	IPP	.	.	.	.	.	.	.	.	.	.	.	.	.	.	.	.	.	.
030.001.00179	I	.	.	.	.	.	.	.	.	.	.	.	.	.	.	.	.	.	.
030.001.00184	IPP	.	.	.	.	.	.	.	.	.	.	.	.	.	.	.	.	.	.
030.001.00186	IPP	.	.	.	.	.	.	.	.	.	.	.	.	.	.	.	.	.	.
030.001.00187	I	.	.	.	.	.	.	.	.	.	.	.	.	.	.	.	.	.	.
030.001.00190	IPP	.	.	.	.	.	.	.	.	.	.	.	.	.	.	.	.	.	.
030.001.00194	IPP	.	.	.	.	.	.	.	.	.	.	.	.	.	.	.	.	.	.
030.001.00195	IPP	.	.	.	.	.	.	.	.	.	.	.	.	.	.	.	.	.	.
030.001.00201	IPP	.	.	.	.	.	.	.	.	.	.	.	.	.	.	.	.	.	.
030.001.00203	IPP	.	.	.	.	.	.	.	.	.	.	.	.	.	.	.	.	.	.
030.001.00205	IPP	.	.	.	.	.	.	.	.	.	.	.	.	.	.	.	.	.	.
030.001.00207	IPP	.	.	.	.	.	.	.	.	.	.	.	.	.	.	.	.	.	.
030.001.00209	IPP	.	.	.	.	.	.	.	.	.	.	.	.	.	.	.	.	.	.
030.001.00210	IPP	.	.	.	.	.	.	.	.	.	.	.	.	.	.	.	.	.	.
030.001.00217	IPP	.	.	.	.	.	.	.	.	.	.	.	.	.	.	.	.	.	.
030.001.00218	IPP	.	.	.	.	.	.	.	.	.	.	.	.	.	.	.	.	.	.
030.001.00220	IPP	.	.	.	.	.	.	.	.	.	.	.	.	.	.	.	.	.	.
030.001.00228	IPP	.	.	.	.	.	.	.	.	.	.	.	.	.	.	.	.	.	.
030.001.00230	IPP	.	.	.	.	.	.	.	.	.	.	.	.	.	.	.	.	.	.
030.001.00231	IPP	.	.	.	.	.	.	.	.	.	.	.	.	.	.	.	.	.	.
030.001.00233	IPP	.	.	.	.	.	.	.	.	.	.	.	.	.	.	.	.	.	.
030.001.00240	IPP	.	.	.	.	.	.	.	.	.	.	.	.	.	.	.	.	.	.
030.001.00243	IPP	.	.	.	.	.	.	.	.	.	.	.	.	.	.	.	.	.	.
030.001.00246	IPP	.	.	.	.	.	.	.	.	.	.	.	.	.	.	.	.	.	.
030.001.00249	IPP	.	.	.	.	.	.	.	.	.	.	.	.	.	.	.	.	.	.
030.001.00252	IPP	.	.	.	.	.	.	.	.	.	.	.	.	.	.	.	.	.	.
030.001.00254	I	.	.	.	.	.	.	.	.	.	.	.	.	.	.	.	.	.	.
030.001.00259	IPP		.	.	.	.	.	.	.	.	.	.	.	.	.	.	.	.	.
030.001.00261		.	.	.	.	.	.	.	.	.	.	.	.	.	.	.	.	.	.
030.001.00264	IPP	.	.	.	.	.	.	.	.	.	.	.	.	.	.	.	.	.	.

*Popn=Population (I = Intent-To-Treat, PP = Per Protocol)
Note: X = Violation
Tafenoquine = Tafenoquine 200mg, Mefloquine = Mefloquine 250mg

SB252263 - 030

Listing B.07

Inclusion And Exclusion Criteria Violations

Treatment : Tafenoquine

Subject Number	Popn*	1	2	3	4	5	6	7	8	9	10	11	12	13	14	15	16	17	18
030.001.00266	I	.	.	.	.	.	.	.	.	.	.	.	.	.	.	.	.	.	.
030.001.00267	IPP	.	.	.	.	.	.	.	.	.	.	.	.	.	.	.	.	.	.
030.001.00271	I	.	.	.	.	.	.	.	.	.	.	.	.	.	.	.	.	.	.
030.001.00272	IPP	.	.	.	.	.	.	.	.	.	.	.	.	.	.	.	.	.	.
030.001.00279	IPP	.	.	.	.	.	.	.	.	.	.	.	.	.	.	.	.	.	.
030.001.00282	IPP	.	.	.	.	.	.	.	.	.	.	.	.	.	.	.	.	.	.
030.001.00284	IPP	.	.	.	.	.	.	.	.	.	.	.	.	.	.	.	.	.	.
030.001.00288	IPP	.	.	.	.	.	.	.	.	.	.	.	.	.	.	.	.	.	.
030.001.00293	IPP	.	.	.	.	.	.	.	.	.	.	.	.	.	.	.	.	.	.
030.001.00294	IPP	.	.	.	.	.	.	.	.	.	.	.	.	.	.	.	.	.	.
030.001.00296	IPP	.	.	.	.	.	.	.	.	.	.	.	.	.	.	.	.	.	.
030.001.00298	IPP	.	X	.	.	.	.	.	.	.	.	.	.	.	.	.	.	.	.
030.001.00301	IPP	.	.	.	.	.	.	.	.	.	.	.	.	.	.	.	.	.	.
030.001.00302	I	.	.	.	.	.	.	.	.	.	.	.	.	.	.	.	.	.	.
030.001.00304	IPP	.	.	.	.	.	.	.	.	.	.	.	.	.	.	.	.	.	.
030.001.00306	IPP	.	.	.	.	.	.	.	.	.	.	.	.	.	.	.	.	.	.

*Popn=Population (I = Intent-To-Treat, PP = Per Protocol)
Note: X = Violation
Tafenoquine = Tafenoquine 200mg, Mefloquine = Mefloquine 250mg

SB252263 - 030

Listing B.07

Inclusion And Exclusion Criteria Violations

Treatment : Mefloquine

Subject Number	Popn*	1	2	3	4	5	6	7	8	9	10	11	12	13	14	15	16	17	18
030.001.00003	I	.	.	.	.	.	.	.	.	.	.	.	.	.	.	.	.	.	.
030.001.00007	IPP	.	.	.	.	.	.	.	.	.	.	.	.	.	.	.	.	.	.
030.001.00008	IPP	.	.	.	.	.	.	.	.	.	.	.	.	.	.	.	.	.	.
030.001.00010	IPP	.	.	.	.	.	.	.	.	.	.	.	.	.	.	.	.	.	.
030.001.00013	IPP	.	.	.	.	.	.	.	.	.	.	.	.	.	.	.	.	.	.
030.001.00018	IPP	.	.	.	.	.	.	.	.	.	.	.	.	.	.	.	.	.	.
030.001.00021	IPP	.	.	.	.	.	.	.	.	.	.	.	.	.	.	.	.	.	.
030.001.00023	I	.	.	.	.	.	.	.	.	.	.	.	.	.	.	.	.	.	.
030.001.00031	IPP	.	.	.	.	.	.	.	.	.	.	.	.	.	.	.	.	.	.
030.001.00034	IPP	.	.	.	.	.	.	.	.	.	.	.	.	.	.	.	.	.	.
030.001.00035		.	.	.	.	.	.	.	.	.	.	.	.	.	.	.	.	.	.
030.001.00036	I	.	.	.	.	.	.	.	.	.	.	.	.	.	.	.	.	.	.
030.001.00038	IPP	.	.	.	.	.	.	.	.	.	.	.	.	.	.	.	.	.	.
030.001.00045	IPP	.	.	.	.	.	.	.	.	.	.	.	.	.	.	.	.	.	.
030.001.00046	IPP	.	.	.	.	.	.	.	.	.	.	.	.	.	.	.	.	.	.
030.001.00047	IPP	.	.	.	.	.	.	.	.	.	.	.	.	.	.	.	.	.	.
030.001.00049	IPP	.	.	.	.	.	.	.	.	.	.	.	.	.	.	.	.	.	.
030.001.00052	IPP	.	.	.	.	.	.	.	.	.	.	.	.	.	.	.	.	.	.
030.001.00053	IPP	.	.	.	.	.	.	.	.	.	.	.	.	.	.	.	.	.	.
030.001.00054	IPP	.	.	.	.	.	.	.	.	.	.	.	.	.	.	.	.	.	.
030.001.00062	IPP	.	.	.	.	.	.	.	.	.	.	.	.	.	.	.	.	.	.
030.001.00066	IPP	.	.	.	.	.	.	.	.	.	.	.	.	.	.	.	.	.	.
030.001.00069	I	.	.	.	.	.	.	.	.	.	.	.	.	.	.	.	.	.	.
030.001.00071	IPP	.	.	.	.	.	.	.	.	.	.	.	.	.	.	.	.	.	.
030.001.00073	IPP	.	.	.	.	.	.	.	.	.	.	.	.	.	.	.	.	.	.
030.001.00074	IPP	.	.	.	.	.	.	.	.	.	.	.	.	.	.	.	.	.	.
030.001.00075	IPP	.	.	.	.	.	.	.	.	.	.	.	.	.	.	.	.	.	.
030.001.00083	IPP	.	.	.	.	.	.	.	.	.	.	.	.	.	.	.	.	.	.
030.001.00089	IPP	.	.	.	.	.	.	.	.	.	.	.	.	.	.	.	.	.	.
030.001.00091	IPP	.	.	.	.	.	.	.	.	.	.	.	.	.	.	.	.	.	.
030.001.00095	IPP	.	.	.	.	.	.	.	.	.	.	.	.	.	.	.	.	.	.
030.001.00096	IPP	.	.	.	.	.	.	.	.	.	.	.	.	.	.	.	.	.	.
030.001.00097	IPP	.	.	.	.	.	.	.	.	.	.	.	.	.	.	.	.	.	.
030.001.00098	IPP	.	.	.	.	.	.	.	.	.	.	.	.	.	.	.	.	.	.
030.001.00099	IPP	.	.	.	.	.	.	.	.	.	.	.	.	.	.	.	.	.	.
030.001.00105	IPP	.	.	.	.	.	.	.	.	.	.	.	.	.	.	.	.	.	.
030.001.00110	IPP	.	.	.	.	.	.	.	.	.	.	.	.	.	.	.	.	.	.
030.001.00113	IPP	.	.	.	.	.	.	.	.	.	.	.	.	.	.	.	.	.	.
030.001.00117	IPP	.	.	.	.	.	.	.	.	.	.	.	.	.	.	.	.	.	.
030.001.00120	IPP	.	.	.	.	.	.	.	.	.	.	.	.	.	.	.	.	.	.
030.001.00121	IPP	.	.	.	.	.	.	.	.	.	.	.	.	.	.	.	.	.	.
030.001.00123	I	.	.	.	.	.	.	.	.	.	.	.	.	.	.	.	.	.	.
030.001.00130	IPP	.	.	.	.	.	.	.	.	.	.	.	.	.	.	.	.	.	.
030.001.00132	IPP	.	.	.	.	.	.	.	.	.	.	.	.	.	.	.	.	.	.

*Popn=Population (I = Intent-To-Treat, PP = Per Protocol)
Note: X = Violation
Tafenoquine = Tafenoquine 200mg, Mefloquine = Mefloquine 250mg

SB252263 - 030

Listing B.07

Inclusion And Exclusion Criteria Violations

Treatment : Mefloquine

Subject Number	Popn*	1	2	3	4	5	6	7	8	9	10	11	12	13	14	15	16	17	18
030.001.00134	IPP	.	.	.	.	.	.	.	.	.	.	.	.	.	.	.	.	.	.
030.001.00142	IPP	.	.	.	.	.	.	.	.	.	.	.	.	.	.	.	.	.	.
030.001.00143	IPP	.	.	.	.	.	.	.	.	.	.	.	.	.	.	.	.	.	.
030.001.00144	IPP	.	.	.	.	.	.	.	.	.	.	.	.	.	.	.	.	.	.
030.001.00146	I	.	.	.	.	.	.	.	.	.	.	.	.	.	.	.	.	.	.
030.001.00147	IPP	.	.	.	.	.	.	.	.	.	.	.	.	.	.	.	.	.	.
030.001.00153	IPP	.	.	.	.	.	.	.	.	.	.	.	.	.	.	.	.	.	.
030.001.00156	IPP	.	.	.	.	.	.	.	.	.	.	.	.	.	.	.	.	.	.
030.001.00163	IPP	.	.	.	.	.	.	.	.	.	.	.	.	.	.	.	.	.	.
030.001.00165	IPP	.	.	.	.	.	.	.	.	.	.	.	.	.	.	.	.	.	.
030.001.00167	IPP	.	.	.	.	.	.	.	.	.	.	.	.	.	.	.	.	.	.
030.001.00168	IPP	.	.	.	.	.	.	.	.	.	.	.	.	.	.	.	.	.	.
030.001.00170	IPP	.	.	.	.	.	.	.	.	.	.	.	.	.	.	.	.	.	.
030.001.00177	IPP	.	.	.	.	.	.	.	.	.	.	.	.	.	.	.	.	.	.
030.001.00178	I	.	.	.	.	.	.	.	.	.	.	.	.	.	.	.	.	.	.
030.001.00180	IPP	.	.	.	.	.	.	.	.	.	.	.	.	.	.	.	.	.	.
030.001.00181	IPP	.	.	.	.	.	.	.	.	.	.	.	.	.	.	.	.	.	.
030.001.00185	IPP	.	.	.	.	.	.	.	.	.	.	.	.	.	.	.	.	.	.
030.001.00188	IPP	.	.	.	.	.	.	.	.	.	.	.	.	.	.	.	.	.	.
030.001.00189	I	.	.	.	.	.	.	.	.	.	.	.	.	.	.	.	.	.	.
030.001.00196	IPP	.	.	.	.	.	.	.	.	.	.	.	.	.	.	.	.	.	.
030.001.00198	IPP	.	.	.	.	.	.	.	.	.	.	.	.	.	.	.	.	.	.
030.001.00199	IPP	.	.	.	.	.	.	.	.	.	.	.	.	.	.	.	.	.	.
030.001.00200	IPP	.	.	.	.	.	.	.	.	.	.	.	.	.	.	.	.	.	.
030.001.00206	I	.	.	.	.	.	.	.	.	.	.	.	.	.	.	.	.	.	.
030.001.00213	IPP	.	.	.	.	.	.	.	.	.	.	.	.	.	.	.	.	.	.
030.001.00214	IPP	.	.	.	.	.	.	.	.	.	.	.	.	.	.	.	.	.	.
030.001.00216	IPP	.	.	.	.	.	.	.	.	.	.	.	.	.	.	.	.	.	.
030.001.00221	IPP	.	.	.	.	.	.	.	.	.	.	.	.	.	.	.	.	.	.
030.001.00223	IPP	.	.	.	.	.	.	.	.	.	.	.	.	.	.	.	.	.	.
030.001.00224	IPP	.	.	.	.	.	.	.	.	.	.	.	.	.	.	.	.	.	.
030.001.00227	IPP	.	.	.	.	.	.	.	.	.	.	.	.	.	.	.	.	.	.
030.001.00232	IPP	.	.	.	.	.	.	.	.	.	.	.	.	.	.	.	.	.	.
030.001.00235	IPP	.	.	.	.	.	.	.	.	.	.	.	.	.	.	.	.	.	.
030.001.00237	IPP	.	.	.	.	.	.	.	.	.	.	.	.	.	.	.	.	.	.
030.001.00239	IPP	.	.	.	.	.	.	.	.	.	.	.	.	.	.	.	.	.	.
030.001.00241	IPP	.	.	.	.	.	.	.	.	.	.	.	.	.	.	.	.	.	.
030.001.00244	IPP	.	.	.	.	.	.	.	.	.	.	.	.	.	.	.	.	.	.
030.001.00245	IPP	.	.	.	.	.	.	.	.	.	.	.	.	.	.	.	.	.	.
030.001.00250		.	.	.	.	.	.	.	.	.	.	.	.	.	.	.	.	.	.
030.001.00255	IPP	.	.	.	.	.	.	.	.	.	.	.	.	.	.	.	.	.	.
030.001.00258	IPP	.	.	.	.	.	.	.	.	.	.	.	.	.	.	.	.	.	.
030.001.00262	IPP	.	.	.	.	.	.	.	.	.	.	.	.	.	.	.	.	.	.
030.001.00263	IPP	.	.	.	.	.	.	.	.	.	.	.	.	.	.	.	.	.	.

*Popn=Population (I = Intent-To-Treat, PP = Per Protocol)
Note: X = Violation
Tafenoquine = Tafenoquine 200mg, Mefloquine = Mefloquine 250mg

SB252263 - 030

Listing B.07

Inclusion And Exclusion Criteria Violations

Treatment : Mefloquine

Subject Number	Popn*	1	2	3	4	5	6	7	8	9	10	11	12	13	14	15	16	17	18
030.001.00265	IPP	.	.	.	.	.	.	.	.	.	.	.	.	.	.	.	.	.	.
030.001.00273	IPP	.	.	.	.	.	.	.	.	.	.	.	.	.	.	.	.	.	.
030.001.00274	IPP	.	.	.	.	.	.	.	.	.	.	.	.	.	.	.	.	.	.
030.001.00276	IPP	.	.	.	.	.	.	.	.	.	.	.	.	.	.	.	.	.	.
030.001.00280	IPP	.	.	.	.	.	.	.	.	.	.	.	.	.	.	.	.	.	.
030.001.00281	IPP	.	.	.	.	.	.	.	.	.	.	.	.	.	.	.	.	.	.
030.001.00283	IPP	.	.	.	.	.	.	.	.	.	.	.	.	.	.	.	.	.	.
030.001.00286	IPP	.	.	.	.	.	.	.	.	.	.	.	.	.	.	.	.	.	.
030.001.00289	IPP	.	.	.	.	.	.	.	.	.	.	.	.	.	.	.	.	.	.
030.001.00290	IPP	.	.	.	.	.	.	.	.	.	.	.	.	.	.	.	.	.	.
030.001.00292	IPP	.	.	.	.	.	.	.	.	.	.	.	.	.	.	.	.	.	.
030.001.00299	IPP	.	.	.	.	.	.	.	.	.	.	.	.	.	.	.	.	.	.
030.001.00305	IPP	.	.	.	.	.	.	.	.	.	.	.	.	.	.	.	.	.	.

*Popn=Population (I = Intent-To-Treat, PP = Per Protocol)
Note: X = Violation
Tafenoquine = Tafenoquine 200mg, Mefloquine = Mefloquine 250mg

SB252263 - 030

Listing B.08

Demographic Characteristics

Treatment: Placebo

Subject Number	Popn*	Screening Date	Gender	Date Of Birth	Age(years)	Weight(kg)	Race
030.001.00002	IPP	10APR2000	Male	01JAN1982	18	51.0	Black
030.001.00005	IPP	10APR2000	Male	01JAN1958	42	66.0	Black
030.001.00011	IPP	10APR2000	Male	01JAN1955	45	61.0	Black
030.001.00012	IPP	10APR2000	Male	01JAN1981	19	61.0	Black
030.001.00015	I	10APR2000	Male	01JAN1977	23	65.0	Black
030.001.00019	IPP	10APR2000	Female	01JAN1967	33	60.0	Black
030.001.00020	IPP	10APR2000	Female	01JAN1960	40	84.0	Black
030.001.00022	IPP	13APR2000	Male	01FEB1964	36	55.0	Black
030.001.00026	IPP	10APR2000	Male	01JAN1968	32	60.0	Black
030.001.00027	IPP	12APR2000	Male	01OCT1977	22	56.0	Black
030.001.00032	IPP	13APR2000	Male	01AUG1979	20	61.0	Black
030.001.00033	IPP	11APR2000	Male	10APR1970	30	66.0	Black
030.001.00037	IPP	11APR2000	Male	12DEC1967	32	64.0	Black
030.001.00039	IPP	14APR2000	Male	01JAN1982	18	51.0	Black
030.001.00041	IPP	10APR2000	Male	01JAN1980	20	67.0	Black
030.001.00044	IPP	10APR2000	Male	01JAN1975	25	61.0	Black
030.001.00055	IPP	12APR2000	Female	01JAN1945	55	55.0	Black
030.001.00056	I	11APR2000	Female	01FEB1952	48	51.0	Black
030.001.00057	IPP	14APR2000	Female	21JUL1962	37	57.0	Black
030.001.00059	IPP	13APR2000	Female	01JAN1953	47	46.0	Black
030.001.00063	IPP	11APR2000	Female	20APR1979	20	62.0	Black
030.001.00064	IPP	11APR2000	Male	20FEB1982	18	56.0	Black

*Popn=Population (I=Intent-to-Treat, PP=Per Protocol)

Tafenoquine = Tafenoquine 200mg, Mefloquine = Mefloquine 250mg

SB252263 - 030

Listing B.08

Demographic Characteristics

Treatment: Placebo

Subject Number	Popn*	Screening Date	Gender	Date Of Birth	Age(years)	Weight(kg)	Race
030.001.00067	IPP	12APR2000	Female	10AUG1952	47	54.0	Black
030.001.00072	IPP	12APR2000	Male	01JAN1978	22	74.0	Black
030.001.00076	IPP	11APR2000	Female	08JUN1962	37	57.0	Black
030.001.00078	IPP	12APR2000	Male	05MAY1968	31	65.0	Black
030.001.00080	IPP	12APR2000	Male	01FEB1981	19	62.0	Black
030.001.00081	IPP	11APR2000	Male	01AUG1961	38	67.0	Black
030.001.00087	I	14APR2000	Male	01JAN1980	20	56.0	Black
030.001.00088	IPP	14APR2000	Male	01JAN1978	22	76.0	Black
030.001.00090	IPP	12APR2000	Female	01JAN1952	48	60.0	Black
030.001.00092	IPP	12APR2000	Female	01JAN1959	41	60.0	Black
030.001.00101	IPP	14APR2000	Female	01JAN1967	33	49.0	Black
030.001.00103	IPP	11APR2000	Female	01JAN1948	52	57.0	Black
030.001.00106	IPP	10APR2000	Male	01JAN1981	19	60.0	Black
030.001.00108	IPP	14APR2000	Male	27MAR1969	31	75.0	Black
030.001.00114	IPP	14APR2000	Female	01JAN1953	47	50.0	Black
030.001.00115	IPP	17APR2000	Female	01MAY1948	51	65.0	Black
030.001.00116	IPP	12APR2000	Male	01JAN1948	52	59.0	Black
030.001.00119	IPP	11APR2000	Male	01JAN1980	20	60.0	Black
030.001.00124	IPP	11APR2000	Male	10OCT1970	29	65.0	Black
030.001.00125	IPP	10APR2000	Male	01JAN1978	22	53.0	Black
030.001.00127	I	12APR2000	Male	01MAY1980	19	59.0	Black
030.001.00131	IPP	10APR2000	Male	01JAN1950	50	60.0	Black

*Popn=Population (I=Intent-to-Treat, PP=Per Protocol)

Tafenoquine = Tafenoquine 200mg, Mefloquine = Mefloquine 250mg

SB252263 - 030

Listing B.08

Demographic Characteristics

Treatment: Placebo

Subject Number	Popn*	Screening Date	Gender	Date Of Birth	Age(years)	Weight(kg)	Race
030.001.00136	IPP	11APR2000	Female	28DEC1948	51	56.0	Black
030.001.00137	IPP	11APR2000	Male	25DEC1966	33	52.0	Black
030.001.00140	IPP	10APR2000	Male	06JUN1976	23	60.0	Black
030.001.00141	IPP	11APR2000	Female	06MAY1948	51	50.0	Black
030.001.00145	IPP	18APR2000	Female	01JAN1955	45	50.0	Black
030.001.00151	IPP	11APR2000	Male	01JUL1946	53	59.0	Black
030.001.00152	IPP	13APR2000	Male	01JAN1950	50	54.0	Black
030.001.00154	IPP	13APR2000	Female	01JAN1954	46	58.0	Black
030.001.00160	I	11APR2000	Male	01AUG1969	30	60.0	Black
030.001.00162	IPP	11APR2000	Female	05AUG1970	29	61.0	Black
030.001.00164	IPP	14APR2000	Male	01FEB1978	22	63.0	Black
030.001.00166	IPP	10APR2000	Male	01JAN1982	18	57.0	Black
030.001.00169	IPP	10APR2000	Female	01JAN1981	19	60.0	Black
030.001.00172	IPP	10APR2000	Male	01APR1970	30	59.0	Black
030.001.00174	IPP	11APR2000	Male	16APR1969	30	53.0	Black
030.001.00175	IPP	17APR2000	Male	12JUL1952	47	71.0	Black
030.001.00182	I	10APR2000	Female	01JAN1972	28	69.0	Black
030.001.00183	IPP	11APR2000	Female	15AUG1974	25	51.0	Black
030.001.00191	IPP	18APR2000	Female	04JUN1978	21	66.0	Black
030.001.00192	IPP	11APR2000	Male	10DEC1980	19	51.0	Black
030.001.00193	IPP	14APR2000	Male	01JAN1981	19	59.0	Black
030.001.00197	IPP	17APR2000	Female	18AUG1968	31	48.0	Black

*Popn=Population (I=Intent-to-Treat, PP=Per Protocol)

Tafenoquine = Tafenoquine 200mg, Mefloquine = Mefloquine 250mg

SB252263 - 030

Listing B.08

Demographic Characteristics

Treatment: Placebo

Subject Number	Popn*	Screening Date	Gender	Date Of Birth	Age(years)	Weight(kg)	Race
030.001.00202		17APR2000	Female	01JAN1979	21	65.0	Black
030.001.00204	I	14APR2000	Female	01JAN1952	48	50.0	Black
030.001.00208	IPP	11APR2000	Male	01JAN1981	19	71.0	Black
030.001.00211	IPP	11APR2000	Male	11AUG1981	18	55.0	Black
030.001.00212	IPP	13APR2000	Male	30MAY1971	28	68.0	Black
030.001.00215	IPP	12APR2000	Female	02NOV1967	32	48.0	Black
030.001.00219	IPP	12APR2000	Female	01JAN1959	41	44.0	Black
030.001.00222	IPP	14APR2000	Male	01JAN1970	30	66.0	Black
030.001.00225	IPP	10APR2000	Female	01JAN1959	41	81.0	Black
030.001.00226	IPP	13APR2000	Female	01JAN1954	46	52.0	Black
030.001.00229	IPP	13APR2000	Male	26APR1975	24	75.0	Black
030.001.00234	IPP	11APR2000	Male	05JUN1982	17	51.0	Black
030.001.00236	IPP	11APR2000	Male	01JUN1964	35	65.0	Black
030.001.00238	IPP	12APR2000	Male	01APR1980	20	62.0	Black
030.001.00242	IPP	14APR2000	Male	01JUL1969	30	57.0	Black
030.001.00247	IPP	11APR2000	Female	12JUN1978	21	54.0	Black
030.001.00248	IPP	13APR2000	Male	28MAR1982	18	51.0	Black
030.001.00251	IPP	12APR2000	Female	01JAN1951	49	51.0	Black
030.001.00253	IPP	13APR2000	Female	01JAN1944	56	60.0	Black
030.001.00256	IPP	14APR2000	Male	01JAN1963	37	60.0	Black
030.001.00257	IPP	14APR2000	Male	12DEC1975	24	61.0	Black
030.001.00260	IPP	17APR2000	Male	01JUL1980	19	65.0	Black

*Popn=Population (I=Intent-to-Treat, PP=Per Protocol)

Tafenoquine = Tafenoquine 200mg, Mefloquine = Mefloquine 250mg

SB252263 - 030

Listing B.08

Demographic Characteristics

Treatment: Placebo

Subject Number	Popn*	Screening Date	Gender	Date Of Birth	Age(years)	Weight(kg)	Race
030.001.00268	IPP	14APR2000	Male	01JAN1976	24	66.0	Black
030.001.00269	IPP	13APR2000	Male	01MAY1980	19	74.0	Black
030.001.00270	IPP	13APR2000	Male	14APR1977	22	69.0	Black
030.001.00275	IPP	12APR2000	Female	01JAN1950	50	57.0	Black
030.001.00277	IPP	12APR2000	Male	21NOV1968	31	70.0	Black
030.001.00278	IPP	12APR2000	Male	01AUG1967	32	63.0	Black
030.001.00285	IPP	18APR2000	Male	01JAN1961	39	59.0	Black
030.001.00287	IPP	10APR2000	Male	01JAN1952	48	80.0	Black
030.001.00291	IPP	17APR2000	Male	12APR1982	18	57.0	Black
030.001.00295		17APR2000	Female	17AUG1951	48	50.0	Black
030.001.00297	IPP	18APR2000	Female	01JAN1957	43	63.0	Black
030.001.00300	IPP	11APR2000	Male	01OCT1978	21	61.0	Black
030.001.00303	IPP	13APR2000	Female	01DEC1981	18	55.0	Black

*Popn=Population (I=Intent-to-Treat, PP=Per Protocol)

Tafenoquine = Tafenoquine 200mg, Mefloquine = Mefloquine 250mg

SB252263 - 030

Listing B.08

Demographic Characteristics

Treatment: Tafenoquine

Subject Number	Popn*	Screening Date	Gender	Date Of Birth	Age(years)	Weight(kg)	Race
030.001.00001	IPP	12APR2000	Female	01JAN1961	39	49.0	Black
030.001.00004	IPP	13APR2000	Female	14OCT1981	18	60.0	Black
030.001.00006	IPP	10APR2000	Female	01JAN1967	33	59.0	Black
030.001.00009	IPP	12APR2000	Male	01JAN1955	45	82.0	Black
030.001.00014	IPP	10APR2000	Male	01JAN1980	20	69.0	Black
030.001.00016	IPP	10APR2000	Male	01JAN1948	52	73.0	Black
030.001.00017	IPP	17APR2000	Male	11AUG1973	26	64.0	Black
030.001.00024	IPP	11APR2000	Female	04APR1952	48	46.0	Black
030.001.00025	IPP	11APR2000	Male	26DEC1981	18	61.0	Black
030.001.00028	IPP	11APR2000	Male	01DEC1961	38	50.0	Black
030.001.00029	IPP	11APR2000	Male	10FEB1982	18	51.0	Black
030.001.00030	IPP	14APR2000	Female	01AUG1957	42	60.0	Black
030.001.00040	IPP	13APR2000	Female	01FEB1982	18	60.0	Black
030.001.00042	IPP	10APR2000	Male	01JAN1981	19	61.0	Black
030.001.00043	IPP	18APR2000	Male	01APR1975	25	57.0	Black
030.001.00048	IPP	13APR2000	Male	01JAN1960	40	53.0	Black
030.001.00050	IPP	10APR2000	Male	01JAN1974	26	53.0	Black
030.001.00051	IPP	11APR2000	Male	01APR1982	18	55.0	Black
030.001.00058	IPP	17APR2000	Male	01SEP1948	51	62.0	Black
030.001.00060	IPP	13APR2000	Female	07OCT1955	44	60.0	Black
030.001.00061	IPP	14APR2000	Male	01JAN1960	40	61.0	Black
030.001.00065	IPP	11APR2000	Male	02MAR1962	38	59.0	Black

*Popn=Population (I=Intent-to-Treat, PP=Per Protocol)

Tafenoquine = Tafenoquine 200mg, Mefloquine = Mefloquine 250mg

SB252263 - 030

Listing B.08

Demographic Characteristics

Treatment: Tafenoquine

Subject Number	Popn*	Screening Date	Gender	Date Of Birth	Age(years)	Weight(kg)	Race
030.001.00068	IPP	14APR2000	Male	01JAN1980	20	61.0	Black
030.001.00070	IPP	11APR2000	Female	01JAN1968	32	47.0	Black
030.001.00077	IPP	11APR2000	Male	21OCT1956	43	67.0	Black
030.001.00079	IPP	14APR2000	Male	01JAN1979	21	80.0	Black
030.001.00082		18APR2000	Male	01JAN1964	36	56.0	Black
030.001.00084	IPP	13APR2000	Male	01AUG1957	42	61.0	Black
030.001.00085	IPP	10APR2000	Female	01JAN1949	51	54.0	Black
030.001.00086	IPP	11APR2000	Male	28DEC1973	26	66.0	Black
030.001.00093	IPP	10APR2000	Female	01JAN1975	25	56.0	Black
030.001.00094	I	11APR2000	Male	06OCT1972	27	74.0	Black
030.001.00100	IPP	12APR2000	Female	24DEC1982	17	54.0	Black
030.001.00102	IPP	13APR2000	Male	04JAN1967	33	80.0	Black
030.001.00104	IPP	14APR2000	Male	01JAN1964	36	74.0	Black
030.001.00107	IPP	12APR2000	Female	30OCT1948	51	59.0	Black
030.001.00109	IPP	11APR2000	Male	14JUN1966	33	63.0	Black
030.001.00111	IPP	11APR2000	Male	18JUN1972	27	64.0	Black
030.001.00112	IPP	13APR2000	Female	01SEP1973	26	46.0	Black
030.001.00118	IPP	13APR2000	Male	01MAR1975	25	51.0	Black
030.001.00122	IPP	12APR2000	Male	01NOV1963	36	68.0	Black
030.001.00126	IPP	11APR2000	Male	13MAY1975	24	60.0	Black
030.001.00128	IPP	10APR2000	Female	01JAN1975	25	50.0	Black
030.001.00129	IPP	12APR2000	Female	01JAN1950	50	42.0	Black

*Popn=Population (I=Intent-to-Treat, PP=Per Protocol)

Tafenoquine = Tafenoquine 200mg, Mefloquine = Mefloquine 250mg

SB252263 - 030

Listing B.08

Demographic Characteristics

Treatment: Tafenoquine

Subject Number	Popn*	Screening Date	Gender	Date Of Birth	Age(years)	Weight(kg)	Race
030.001.00133	IPP	10APR2000	Male	01OCT1980	19	62.0	Black
030.001.00135	IPP	11APR2000	Male	23OCT1976	23	64.0	Black
030.001.00138	IPP	12APR2000	Male	15JUL1968	31	61.0	Black
030.001.00139	IPP	10APR2000	Male	01JAN1978	22	64.0	Black
030.001.00148	IPP	10APR2000	Male	01JAN1978	22	57.0	Black
030.001.00149	IPP	13APR2000	Male	08SEP1979	20	56.0	Black
030.001.00150	IPP	12APR2000	Female	01DEC1963	36	47.0	Black
030.001.00155	IPP	11APR2000	Female	02AUG1950	49	56.0	Black
030.001.00157	IPP	12APR2000	Male	26DEC1980	19	59.0	Black
030.001.00158	IPP	11APR2000	Female	26JUN1979	20	53.0	Black
030.001.00159	IPP	12APR2000	Female	01NOV1980	19	60.0	Black
030.001.00161	IPP	10APR2000	Female	01JAN1966	34	51.0	Black
030.001.00171	IPP	12APR2000	Male	01JAN1946	54	57.0	Black
030.001.00173	I	14APR2000	Female	01MAY1980	19	73.0	Black
030.001.00176	IPP	13APR2000	Female	01SEP1977	22	66.0	Black
030.001.00179	I	12APR2000	Male	01OCT1976	23	67.0	Black
030.001.00184	IPP	17APR2000	Male	01SEP1979	20	56.0	Black
030.001.00186	IPP	10APR2000	Male	01JAN1976	24	73.0	Black
030.001.00187	I	10APR2000	Male	01JAN1980	20	49.0	Black
030.001.00190	IPP	11APR2000	Male	01JAN1978	22	60.0	Black
030.001.00194	IPP	14APR2000	Male	12DEC1949	50	60.0	Black
030.001.00195	IPP	18APR2000	Male	01NOV1978	21	64.0	Black

*Popn=Population (I=Intent-to-Treat, PP=Per Protocol)

Tafenoquine = Tafenoquine 200mg, Mefloquine = Mefloquine 250mg

SB252263 - 030

Listing B.08

Demographic Characteristics

Treatment: Tafenoquine

Subject Number	Popn*	Screening Date	Gender	Date Of Birth	Age(years)	Weight(kg)	Race
030.001.00201	IPP	10APR2000	Female	01JAN1950	50	57.0	Black
030.001.00203	IPP	13APR2000	Female	17APR1967	32	73.0	Black
030.001.00205	IPP	10APR2000	Female	01JAN1956	44	70.0	Black
030.001.00207	IPP	13APR2000	Male	04AUG1980	19	65.0	Black
030.001.00209	IPP	13APR2000	Male	24MAR1970	30	58.0	Black
030.001.00210	IPP	12APR2000	Male	01APR1979	21	60.0	Black
030.001.00217	IPP	11APR2000	Male	01JAN1977	23	63.0	Black
030.001.00218	IPP	13APR2000	Male	01DEC1975	24	69.0	Black
030.001.00220	IPP	13APR2000	Male	01JAN1970	30	56.0	Black
030.001.00228	IPP	13APR2000	Male	01OCT1977	22	64.0	Black
030.001.00230	IPP	13APR2000	Male	19DEC1980	19	64.0	Black
030.001.00231	IPP	12APR2000	Female	01MAR1975	25	61.0	Black
030.001.00233	IPP	10APR2000	Female	01JAN1952	48	50.0	Black
030.001.00240	IPP	13APR2000	Male	01DEC1981	18	59.0	Black
030.001.00243	IPP	13APR2000	Male	01MAR1974	26	64.0	Black
030.001.00246	IPP	11APR2000	Female	01JAN1949	51	54.0	Black
030.001.00249	IPP	12APR2000	Male	01JAN1976	24	54.0	Black
030.001.00252	IPP	12APR2000	Male	01AUG1981	18	59.0	Black
030.001.00254	I	12APR2000	Female	15JUL1980	19	55.0	Black
030.001.00259	IPP	12APR2000	Male	01JAN1974	26	70.0	Black
030.001.00261		13APR2000	Female	01FEB1969	31	50.0	Black
030.001.00264	IPP	18APR2000	Female	01JAN1976	24	63.0	Black

*Popn=Population (I=Intent-to-Treat, PP=Per Protocol)

Tafenoquine = Tafenoquine 200mg, Mefloquine = Mefloquine 250mg

SB252263 - 030

Listing B.08

Demographic Characteristics

Treatment: Tafenoquine

Subject Number	Popn*	Screening Date	Gender	Date Of Birth	Age(years)	Weight(kg)	Race
030.001.00266	I	12APR2000	Male	01JAN1970	30	57.0	Black
030.001.00267	IPP	11APR2000	Male	01AUG1977	22	53.0	Black
030.001.00271	I	13APR2000	Male	01JAN1947	53	73.0	Black
030.001.00272	IPP	10APR2000	Female	01JAN1965	35	90.0	Black
030.001.00279	IPP	12APR2000	Male	26MAY1970	29	82.0	Black
030.001.00282	IPP	11APR2000	Female	01JAN1950	50	51.0	Black
030.001.00284	IPP	10APR2000	Male	01JAN1982	18	57.0	Black
030.001.00288	IPP	12APR2000	Male	01JAN1978	22	57.0	Black
030.001.00293	IPP	11APR2000	Female	12JUL1978	21	68.0	Black
030.001.00294	IPP	13APR2000	Female	10APR1976	24	66.0	Black
030.001.00296	IPP	14APR2000	Female	01JAN1956	44	58.0	Black
030.001.00298	IPP	17APR2000	Male	01JUN1982	17	59.0	Black
030.001.00301	IPP	14APR2000	Male	27JAN1982	18	69.0	Black
030.001.00302	I	14APR2000	Female	03JAN1982	18	65.0	Black
030.001.00304	IPP	13APR2000	Male	01JUL1980	19	64.0	Black
030.001.00306	IPP	13APR2000	Male	01SEP1975	24	69.0	Black

*Popn=Population (I=Intent-to-Treat, PP=Per Protocol)

Tafenoquine = Tafenoquine 200mg, Mefloquine = Mefloquine 250mg

SB252263 - 030

Listing B.08

Demographic Characteristics

Treatment: Mefloquine

Subject Number	Popn*	Screening Date	Gender	Date Of Birth	Age(years)	Weight(kg)	Race
030.001.00003	I	10APR2000	Female	01JAN1967	33	64.0	Black
030.001.00007	IPP	11APR2000	Female	01APR1976	24	58.0	Black
030.001.00008	IPP	11APR2000	Male	03JAN1960	40	75.0	Black
030.001.00010	IPP	11APR2000	Female	20MAR1949	51	66.0	Black
030.001.00013	IPP	13APR2000	Male	05FEB1970	30	65.0	Black
030.001.00018	IPP	11APR2000	Female	11APR1974	26	53.0	Black
030.001.00021	IPP	10APR2000	Female	01JAN1959	41	45.0	Black
030.001.00023	I	10APR2000	Female	01JAN1956	44	55.0	Black
030.001.00031	IPP	12APR2000	Male	20APR1958	41	55.0	Black
030.001.00034	IPP	17APR2000	Male	01AUG1979	20	63.0	Black
030.001.00035		12APR2000	Male	01FEB1982	18	57.0	Black
030.001.00036	I	13APR2000	Male	01JAN1964	36	65.0	Black
030.001.00038	IPP	18APR2000	Male	01JAN1982	18	72.0	Black
030.001.00045	IPP	14APR2000	Male	01JAN1962	38	58.0	Black
030.001.00046	IPP	10APR2000	Male	01JAN1974	26	66.0	Black
030.001.00047	IPP	12APR2000	Male	01FEB1982	18	49.0	Black
030.001.00049	IPP	13APR2000	Male	01DEC1965	34	52.0	Black
030.001.00052	IPP	10APR2000	Female	01JAN1956	44	59.0	Black
030.001.00053	IPP	11APR2000	Male	01JAN1973	27	61.0	Black
030.001.00054	IPP	10APR2000	Male	01JAN1982	18	57.0	Black
030.001.00062	IPP	12APR2000	Male	01DEC1980	19	52.0	Black
030.001.00066	IPP	14APR2000	Female	01JAN1948	52	40.0	Black

*Popn=Population (I=Intent-to-Treat, PP=Per Protocol)

Tafenoquine = Tafenoquine 200mg, Mefloquine = Mefloquine 250mg

SB252263 - 030

Listing B.08

Demographic Characteristics

Treatment: Mefloquine

Subject Number	Popn*	Screening Date	Gender	Date Of Birth	Age(years)	Weight(kg)	Race
030.001.00069	I	10APR2000	Male	01JAN1982	18	64.0	Black
030.001.00071	IPP	10APR2000	Female	01JAN1945	55	65.0	Black
030.001.00073	IPP	13APR2000	Male	01JAN1952	48	62.0	Black
030.001.00074	IPP	10APR2000	Male	01JAN1975	25	67.0	Black
030.001.00075	IPP	10APR2000	Female	27DEC1956	43	56.0	Black
030.001.00083	IPP	13APR2000	Male	13AUG1970	29	53.0	Black
030.001.00089	IPP	17APR2000	Male	01JAN1981	19	52.0	Black
030.001.00091	IPP	13APR2000	Male	01APR1970	30	56.0	Black
030.001.00095	IPP	14APR2000	Male	01SEP1957	42	57.0	Black
030.001.00096	IPP	12APR2000	Male	01JUN1981	18	55.0	Black
030.001.00097	IPP	11APR2000	Female	01APR1959	41	60.0	Black
030.001.00098	IPP	12APR2000	Male	13APR1979	20	50.0	Black
030.001.00099	IPP	10APR2000	Male	01JAN1978	22	73.0	Black
030.001.00105	IPP	11APR2000	Male	01JAN1974	26	72.0	Black
030.001.00110	IPP	10APR2000	Male	01JAN1980	20	59.0	Black
030.001.00113	IPP	17APR2000	Female	23NOV1975	24	45.0	Black
030.001.00117	IPP	11APR2000	Female	01JAN1960	40	50.0	Black
030.001.00120	IPP	10APR2000	Male	01JAN1963	37	66.0	Black
030.001.00121	IPP	10APR2000	Male	01JAN1962	38	69.0	Black
030.001.00123	I	10APR2000	Male	25MAY1975	24	71.0	Black
030.001.00130	IPP	10APR2000	Male	01JAN1975	25	71.0	Black
030.001.00132	IPP	13APR2000	Male	01JUN1980	19	70.0	Black

*Popn=Population (I=Intent-to-Treat, PP=Per Protocol)

Tafenoquine = Tafenoquine 200mg, Mefloquine = Mefloquine 250mg

SB252263 - 030

Listing B.08

Demographic Characteristics

Treatment: Mefloquine

Subject Number	Popn*	Screening Date	Gender	Date Of Birth	Age(years)	Weight(kg)	Race
030.001.00134	IPP	11APR2000	Female	01SEP1972	27	65.0	Black
030.001.00142	IPP	14APR2000	Male	01FEB1982	18	60.0	Black
030.001.00143	IPP	14APR2000	Male	01JUL1982	17	55.0	Black
030.001.00144	IPP	11APR2000	Male	01AUG1978	21	64.0	Black
030.001.00146	I	13APR2000	Male	18FEB1982	18	56.0	Black
030.001.00147	IPP	10APR2000	Male	01JAN1967	33	90.0	Black
030.001.00153	IPP	12APR2000	Male	08MAY1979	20	62.0	Black
030.001.00156	IPP	14APR2000	Female	10SEP1979	20	50.0	Black
030.001.00163	IPP	11APR2000	Female	10AUG1972	27	62.0	Black
030.001.00165	IPP	11APR2000	Male	20AUG1961	38	55.0	Black
030.001.00167	IPP	13APR2000	Female	01JAN1962	38	58.0	Black
030.001.00168	IPP	14APR2000	Female	01FEB1972	28	64.0	Black
030.001.00170	IPP	12APR2000	Male	01JAN1966	34	60.0	Black
030.001.00177	IPP	13APR2000	Female	12MAR1982	18	66.0	Black
030.001.00178	I	12APR2000	Male	01SEP1972	27	59.0	Black
030.001.00180	IPP	17APR2000	Female	01AUG1944	55	52.0	Black
030.001.00181	IPP	10APR2000	Male	01JAN1982	18	59.0	Black
030.001.00185	IPP	18APR2000	Female	01FEB1982	18	59.0	Black
030.001.00188	IPP	13APR2000	Female	01APR1965	35	57.0	Black
030.001.00189	I	10APR2000	Female	01JAN1950	50	55.0	Black
030.001.00196	IPP	11APR2000	Male	04MAY1962	37	57.0	Black
030.001.00198	IPP	12APR2000	Female	01JAN1948	52	50.0	Black

*Popn=Population (I=Intent-to-Treat, PP=Per Protocol)

Tafenoquine = Tafenoquine 200mg, Mefloquine = Mefloquine 250mg

SB252263 - 030

Listing B.08

Demographic Characteristics

Treatment: Mefloquine

Subject Number	Popn*	Screening Date	Gender	Date Of Birth	Age(years)	Weight(kg)	Race
030.001.00199	IPP	10APR2000	Male	01JAN1980	20	80.0	Black
030.001.00200	IPP	17APR2000	Male	02JAN1982	18	51.0	Black
030.001.00206	I	10APR2000	Male	01JAN1977	23	65.0	Black
030.001.00213	IPP	14APR2000	Male	01JUN1971	28	71.0	Black
030.001.00214	IPP	13APR2000	Female	01DEC1956	43	97.0	Black
030.001.00216	IPP	11APR2000	Male	12JUN1978	21	85.0	Black
030.001.00221	IPP	17APR2000	Male	10APR1959	41	61.0	Black
030.001.00223	IPP	14APR2000	Female	01FEB1977	23	55.0	Black
030.001.00224	IPP	12APR2000	Female	01AUG1958	41	68.0	Black
030.001.00227	IPP	14APR2000	Female	01DEC1970	29	67.0	Black
030.001.00232	IPP	18APR2000	Female	01JAN1966	34	77.0	Black
030.001.00235	IPP	10APR2000	Female	01JAN1955	45	49.0	Black
030.001.00237	IPP	17APR2000	Male	12MAY1974	25	70.0	Black
030.001.00239	IPP	10APR2000	Male	01JAN1975	25	58.0	Black
030.001.00241	IPP	13APR2000	Male	01OCT1979	20	72.0	Black
030.001.00244	IPP	13APR2000	Male	05DEC1976	23	62.0	Black
030.001.00245	IPP	11APR2000	Male	01FEB1976	24	44.0	Black
030.001.00250		18APR2000	Male	01APR1982	18	47.0	Black
030.001.00255	IPP	12APR2000	Male	01DEC1980	19	61.0	Black
030.001.00258	IPP	14APR2000	Male	01AUG1975	24	70.0	Black
030.001.00262	IPP	13APR2000	Male	31OCT1970	29	65.0	Black
030.001.00263	IPP	17APR2000	Male	01FEB1955	45	76.0	Black

*Popn=Population (I=Intent-to-Treat, PP=Per Protocol)

Tafenoquine = Tafenoquine 200mg, Mefloquine = Mefloquine 250mg

SB252263 - 030

Listing B.08

Demographic Characteristics

Treatment: Mefloquine

Subject Number	Popn*	Screening Date	Gender	Date Of Birth	Age(years)	Weight(kg)	Race
030.001.00265	IPP	12APR2000	Male	01MAY1980	19	65.0	Black
030.001.00273	IPP	11APR2000	Female	01JAN1980	20	51.0	Black
030.001.00274	IPP	11APR2000	Female	01JAN1965	35	71.0	Black
030.001.00276	IPP	12APR2000	Male	01JAN1964	36	78.0	Black
030.001.00280	IPP	13APR2000	Male	01OCT1981	18	65.0	Black
030.001.00281	IPP	12APR2000	Male	02APR1969	31	61.0	Black
030.001.00283	IPP	14APR2000	Male	20SEP1968	31	82.0	Black
030.001.00286	IPP	12APR2000	Male	01JAN1972	28	66.0	Black
030.001.00289	IPP	14APR2000	Female	22MAR1981	19	41.0	Black
030.001.00290	IPP	12APR2000	Male	01MAR1982	18	61.0	Black
030.001.00292	IPP	12APR2000	Male	13SEP1980	19	71.0	Black
030.001.00299	IPP	17APR2000	Male	15DEC1982	17	57.0	Black
030.001.00305	IPP	13APR2000	Male	09SEP1968	31	70.0	Black

*Popn=Population (I=Intent-to-Treat, PP=Per Protocol)

Tafenoquine = Tafenoquine 200mg, Mefloquine = Mefloquine 250mg

SB252263 - 030

Listing B.09

Pregnancy Test Results

Treatment: Placebo

Subject Number	Popn*	Visit	Test Done	Date of Test /Visit Date	Relative Day **	Result
030.001.00019	IPP	Screening		10APR2000	-28	Negative
		Screening (Unsch.)	No	14APR2000	-24	
		Screening (Unsch.)	No	20APR2000	-18	
		Screening (Unsch.)	No	26APR2000	-12	
		Screening (Unsch.)	No	02MAY2000	-6	
		Clearance (Unsch.)	No	03MAY2000	-5	
		Load Dose 1	Yes	08MAY2000	0	
		Week 1 (Unsch.)	No	12MAY2000	4	
		Week 4	Yes	05JUN2000	28	
		Week 8	Yes	04JUL2000	57	
		Week 9 (Unsch.)	No	09JUL2000	62	
030.001.00020	IPP	Screening		10APR2000	-28	Negative
		Screening (Unsch.)	No	01MAY2000	-7	
		Load Dose 1	Yes	08MAY2000	0	
		Week 1 (Unsch.)	No	13MAY2000	5	
		Week 3 (Unsch.)	No	26MAY2000	18	
		Week 4	Yes	05JUN2000	28	
		Week 8	Yes	03JUL2000	56	
		Week 11 (Unsch. Fol. Up)	No	24JUL2000	77	
		Week 12 (Unsch. Fol. Up)	Yes	31JUL2000	84	
030.001.00055	IPP	Screening		12APR2000	-26	Negative
		Load Dose 1	Yes	08MAY2000	0	
		Week 4 (Fol. Up)	Yes	05JUN2000	28	
030.001.00056	I	Screening (Unsch.)	No	18APR2000	-20	
		Screening (Unsch.)	No	28APR2000	-10	
		Screening		03MAY2000	-5	Negative
		Clearance (Unsch.)	No	06MAY2000	-2	
		Load Dose 1	Yes	08MAY2000	0	
		Week 1 (Unsch.)	No	16MAY2000	8	
		Week 4	Yes	05JUN2000	28	
		Week 4 (Unsch.)	No	06JUN2000	29	
		Week 8 (Unsch. Fol. Up)	No	01JUL2000	54	
030.001.00057	IPP	Screening		03MAY2000	-5	Negative
		Load Dose 1	No	08MAY2000	0	
		Load Dose (Unsch.)	No	10MAY2000	2	
		Week 1 (Unsch.)	No	18MAY2000	10	
		Week 4	Yes	05JUN2000	28	

*Popn=Population (I=Intent-to-Treat, PP=Per Protocol)
**Day of test relative to start of prophylactic medication
Note: Pregnancy test data is listed for female subjects only.
Note: The date of test and result were recorded only at screening. Where test date is not recorded, it has been replaced with the visit date.
Tafenoquine = Tafenoquine 200mg, Mefloquine = Mefloquine 250mg

SB252263 - 030

Listing B.09

Pregnancy Test Results

Treatment: Placebo

Subject Number	Popn*	Visit	Test Done	Date of Test /Visit Date	Relative Day **	Result
030.001.00057	IPP	Week 8 (Fol. Up)	Yes	03JUL2000	56	
030.001.00059	IPP	Screening		13APR2000	-25	Negative
		Screening (Unsch.)	No	14APR2000	-24	
		Screening (Unsch.)	No	26APR2000	-12	
		Clearance (Unsch.)	No	06MAY2000	-2	
		Load Dose 1	Yes	08MAY2000	0	
		Week 1 (Unsch.)	No	16MAY2000	8	
		Week 2 (Unsch. Fol. Up)	No	24MAY2000	16	
		Week 3 (Unsch. Fol. Up)	No	29MAY2000	21	
		Week 4 (Fol. Up)	Yes	05JUN2000	28	
		Week 8 (Fol. Up)	Yes	03JUL2000	56	
		Week 10 (Unsch. Fol. Up)	No	19JUL2000	72	
		Week 12 (Fol. Up)	No	31JUL2000	84	
		Week 14 (Unsch. Fol. Up)	No	15AUG2000	99	
030.001.00063	IPP	Screening		11APR2000	-27	Negative
		Load Dose 1	Yes	08MAY2000	0	
		Week 1 (Unsch.)	No	11MAY2000	3	
		Week 2 (Unsch.)	No	22MAY2000	14	
		Week 3 (Unsch.)	No	30MAY2000	22	
		Week 4	Yes	05JUN2000	28	
		Week 8	Yes	03JUL2000	56	
030.001.00067	IPP	Screening (Unsch.)	No	15APR2000	-23	
		Screening (Unsch.)	No	18APR2000	-20	
		Screening		03MAY2000	-5	Negative
		Load Dose 1	Yes	08MAY2000	0	
		Week 1 (Unsch.)	No	12MAY2000	4	
		Week 1 (Unsch.)	No	13MAY2000	5	
		Week 3 (Unsch.)	No	29MAY2000	21	
		Week 4	Yes	05JUN2000	28	
		Week 9	No	07JUL2000	60	
		Week 9 (Unsch.)	No	13JUL2000	66	
		Week 12	Yes	31JUL2000	84	
		Week 13 (Unsch.)	No	07AUG2000	91	
		Week 15 (Unsch. Fol. Up)	No	24AUG2000	108	
030.001.00076	IPP	Screening		11APR2000	-27	Negative
		Screening (Unsch.)	No	26APR2000	-12	
		Clearance (Unsch.)	No	06MAY2000	-2	

*Popn=Population (I=Intent-to-Treat, PP=Per Protocol)
**Day of test relative to start of prophylactic medication
Note: Pregnancy test data is listed for female subjects only.
Note: The date of test and result were recorded only at screening. Where test date is not recorded, it has been replaced with the visit date.
Tafenoquine = Tafenoquine 200mg, Mefloquine = Mefloquine 250mg

SB252263 - 030

Listing B.09

Pregnancy Test Results

Treatment: Placebo

Subject Number	Popn*	Visit	Test Done	Date of Test /Visit Date	Relative Day **	Result
030.001.00076	IPP	Load Dose 1	Yes	08MAY2000	0	
		Week 1 (Unsch.)	No	17MAY2000	9	
		Week 4	Yes	05JUN2000	28	
		Week 7 (Unsch. Fol. Up)	No	26JUN2000	49	
		Week 8 (Fol. Up)	Yes	03JUL2000	56	
		Week 9 (Unsch. Fol. Up)		10JUL2000	63	
		Week 10 (Unsch. Fol. Up)		17JUL2000	70	
030.001.00090	IPP	Screening		12APR2000	-26	Negative
		Screening (Unsch.)	No	18APR2000	-20	
		Screening (Unsch.)	No	29APR2000	-9	
		Load Dose 1	Yes	08MAY2000	0	
		Week 3 (Unsch.)	No	30MAY2000	22	
		Week 4	Yes	04JUN2000	27	
		Week 5 (Unsch.)	No	14JUN2000	37	
		Week 6 (Unsch.)	No	21JUN2000	44	
		Week 8	Yes	03JUL2000	56	
030.001.00092	IPP	Screening (Unsch.)	No	18APR2000	-20	
		Screening		03MAY2000	-5	Negative
		Clearance (Unsch.)	No	03MAY2000	-5	
		Load Dose 1	Yes	08MAY2000	0	
		Week 4	Yes	05JUN2000	28	
		Week 4 (Unsch.)	No	07JUN2000	30	
		Week 6 (Unsch.)	No	22JUN2000	45	
		Week 8	Yes	03JUL2000	56	
		Week 9 (Unsch. Fol. Up)	No	11JUL2000	64	
030.001.00101	IPP	Screening (Unsch.)	No	25APR2000	-13	
		Screening (Unsch.)	No	28APR2000	-10	
		Screening		03MAY2000	-5	Negative
		Load Dose 1	Yes	08MAY2000	0	
		Week 2 (Unsch.)	No	24MAY2000	16	
		Week 2 (Unsch.)	No	25MAY2000	17	
		Week 3 (Unsch.)	No	26MAY2000	18	
		Week 3 (Unsch.)	No	28MAY2000	20	
		Week 4	Yes	05JUN2000	28	
		Week 8	Yes	03JUL2000	56	
		Week 9 (Unsch. Fol. Up)	No	11JUL2000	64	
		Week 10 (Unsch. Fol. Up)	No	14JUL2000	67	

*Popn=Population (I=Intent-to-Treat, PP=Per Protocol)
**Day of test relative to start of prophylactic medication
Note: Pregnancy test data is listed for female subjects only.
Note: The date of test and result were recorded only at screening. Where test date is not recorded, it has been replaced with the visit date.
Tafenoquine = Tafenoquine 200mg, Mefloquine = Mefloquine 250mg

SB252263 - 030

Listing B.09

Pregnancy Test Results

Treatment: Placebo

Subject Number	Popn*	Visit	Test Done	Date of Test /Visit Date	Relative Day **	Result
030.001.00103	IPP	Screening		03MAY2000	-5	Negative
		Load Dose 1	Yes	08MAY2000	0	
		Week 2 (Unsch.)	No	19MAY2000	11	
		Week 2 (Unsch.)	No	20MAY2000	12	
		Week 4	Yes	06JUN2000	29	
		Week 7 (Unsch. Fol. Up)	No	23JUN2000	46	
		Week 8 (Fol. Up)	Yes	06JUL2000	59	
		Week 15 (Unsch. Fol. Up)	No	22AUG2000	106	
030.001.00114	IPP	Screening (Unsch.)	No	28APR2000	-10	
		Screening		03MAY2000	-5	Negative
		Load Dose 1	Yes	08MAY2000	0	
		Week 4	Yes	05JUN2000	28	
		Week 6 (Unsch.)	No	19JUN2000	42	
		Week 6 (Unsch.)	No	20JUN2000	43	
		Week 8	Yes	03JUL2000	56	
		Week 11 (Unsch. Fol. Up)	No	27JUL2000	80	
030.001.00115	IPP	Screening		03MAY2000	-5	Negative
		Load Dose 1	Yes	08MAY2000	0	
		Week 3 (Unsch.)	No	26MAY2000	18	
		Week 4	Yes	05JUN2000	28	
030.001.00136	IPP	Screening		04MAY2000	-5	Negative
		Load Dose 1	Yes	09MAY2000	0	
		Week 1 (Unsch.)	No	16MAY2000	7	
		Week 2 (Unsch.)	No	26MAY2000	17	
		Week 3 (Unsch.)	No	29MAY2000	20	
		Week 4	Yes	05JUN2000	27	
		Week 5 (Unsch. Fol. Up)	No	13JUN2000	35	
		Week 6 (Unsch. Fol. Up)	No	22JUN2000	44	
		Week 7 (Unsch. Fol. Up)	No	26JUN2000	48	
		Week 8 (Fol. Up)	Yes	03JUL2000	55	
		Week 10 (Unsch. Fol. Up)	No	17JUL2000	69	
		Week 12 (Fol. Up)	No	31JUL2000	83	
		Week 14 (Unsch. Fol. Up)	No	14AUG2000	97	
030.001.00141	IPP	Screening (Unsch.)	No	18APR2000	-21	
		Screening		04MAY2000	-5	Negative
		Load Dose 1	Yes	09MAY2000	0	
		Week 4	Yes	06JUN2000	28	

*Popn=Population (I=Intent-to-Treat, PP=Per Protocol)
**Day of test relative to start of prophylactic medication
Note: Pregnancy test data is listed for female subjects only.
Note: The date of test and result were recorded only at screening. Where test date is not recorded, it has been replaced with the visit date.
Tafenoquine = Tafenoquine 200mg, Mefloquine = Mefloquine 250mg

SB252263 - 030

Listing B.09

Pregnancy Test Results

Treatment: Placebo

Subject Number	Popn*	Visit	Test Done	Date of Test /Visit Date	Relative Day **	Result
030.001.00145	IPP	Screening		04MAY2000	-5	Negative
		Clearance (Unsch.)	Yes	04MAY2000	-5	
		Load Dose 1	Yes	09MAY2000	0	
		Week 4	Yes	06JUN2000	28	
		Week 8 (Unsch. Fol. Up)	No	01JUL2000	53	
		Week 9 (Unsch. Fol. Up)	No	11JUL2000	63	
030.001.00154	IPP	Screening		04MAY2000	-5	Negative
		Load Dose 1	Yes	09MAY2000	0	
		Week 4	Yes	07JUN2000	29	
		Week 7 (Unsch. Fol. Up)	No	27JUN2000	49	
030.001.00162	IPP	Screening		04MAY2000	-5	Negative
		Load Dose 1	Yes	09MAY2000	0	
		Week 2 (Unsch.)	No	21MAY2000	12	
		Week 4	Yes	05JUN2000	27	
		Week 7 (Unsch.)	No	27JUN2000	49	
		Week 8	Yes	03JUL2000	55	
030.001.00169	IPP	Screening		04MAY2000	-5	Negative
		Load Dose 1	Yes	09MAY2000	0	
		Week 1 (Unsch.)	No	14MAY2000	5	
		Week 1 (Unsch.)	No	19MAY2000	10	
		Week 3 (Unsch.)	No	30MAY2000	21	
		Week 4 (Unsch.)	No	03JUN2000	25	
		Week 4	Yes	05JUN2000	27	
		Week 5 (Unsch.)	No	13JUN2000	35	
		Week 6 (Unsch.)	No	20JUN2000	42	
		Week 8 (Fol. Up)	Yes	03JUL2000	55	
		Week 9 (Unsch. Fol. Up)	No	13JUL2000	65	
		Week 10 (Unsch. Fol. Up)	No	17JUL2000	69	
		Week 11 (Unsch. Fol. Up)	No	24JUL2000	76	
030.001.00182	I	Screening		04MAY2000	-5	Negative
		Load Dose 1	Yes	09MAY2000	0	
		Week 4	Yes	06JUN2000	28	
		Week 5 (Unsch. Fol. Up)	No	12JUN2000	34	
030.001.00183	IPP	Screening (Unsch.)	No	20APR2000	-19	
		Screening		04MAY2000	-5	Negative
		Clearance (Unsch.)	No	05MAY2000	-4	

*Popn=Population (I=Intent-to-Treat, PP=Per Protocol)
**Day of test relative to start of prophylactic medication
Note: Pregnancy test data is listed for female subjects only.
Note: The date of test and result were recorded only at screening. Where test date is not recorded, it has been replaced with the visit date.
Tafenoquine = Tafenoquine 200mg, Mefloquine = Mefloquine 250mg

SB252263 - 030

Listing B.09

Pregnancy Test Results

Treatment: Placebo

Subject Number	Popn*	Visit	Test Done	Date of Test /Visit Date	Relative Day **	Result
030.001.00183	IPP	Load Dose 1	Yes	09MAY2000	0	
		Week 1 (Unsch.)	No	14MAY2000	5	
		Week 1 (Unsch.)	No	15MAY2000	6	
		Week 3 (Unsch.)	No	28MAY2000	19	
		Week 3 (Unsch.)	No	30MAY2000	21	
		Week 4	Yes	06JUN2000	28	
		Week 7 (Unsch. Fol. Up)	No	27JUN2000	49	
		Week 8 (Unsch. Fol. Up)	No	03JUL2000	55	
		Week 9 (Unsch. Fol. Up)	No	14JUL2000	66	
030.001.00191	IPP	Screening (Unsch.)	No	10APR2000	-29	
		Screening (Unsch.)	No	18APR2000	-21	
		Screening		04MAY2000	-5	Negative
		Load Dose 1	Yes	09MAY2000	0	
		Week 4	Yes	09JUN2000	31	
		Week 8 (Unsch. Fol. Up)	Yes	07JUL2000	59	
030.001.00197	IPP	Screening		04MAY2000	-5	Negative
		Load Dose 1	Yes	09MAY2000	0	
		Week 1 (Unsch.)	No	15MAY2000	6	
		Week 4 (Fol. Up)	Yes	06JUN2000	28	
		Week 8 (Unsch. Fol. Up)		04JUL2000	56	
		Week 8 (Unsch. Fol. Up)		05JUL2000	57	
030.001.00202		Screening		17APR2000	-22	Negative
		Load Dose 1	Yes	09MAY2000	0	
		Week 1 (Unsch. Fol. Up)	No	13MAY2000	4	
030.001.00204	I	Screening		04MAY2000	-5	Negative
		Load Dose 1	Yes	09MAY2000	0	
		Week 4	Yes	05JUN2000	27	
		Week 9 (Unsch. Fol. Up)	No	11JUL2000	63	
		Week 12 (Unsch. Fol. Up)	No	31JUL2000	83	
030.001.00215	IPP	Screening		04MAY2000	-5	Negative
		Load Dose 1	Yes	09MAY2000	0	
		Week 1 (Unsch.)	No	12MAY2000	3	
		Week 1 (Unsch.)	No	14MAY2000	5	
		Week 1 (Unsch.)	No	15MAY2000	6	
		Week 4	Yes	06JUN2000	28	

*Popn=Population (I=Intent-to-Treat, PP=Per Protocol)
**Day of test relative to start of prophylactic medication
Note: Pregnancy test data is listed for female subjects only.
Note: The date of test and result were recorded only at screening. Where test date is not recorded, it has been replaced with the visit date.
Tafenoquine = Tafenoquine 200mg, Mefloquine = Mefloquine 250mg

SB252263 - 030

Listing B.09

Pregnancy Test Results

Treatment: Placebo

Subject Number	Popn*	Visit	Test Done	Date of Test /Visit Date	Relative Day **	Result
030.001.00219	IPP	Screening		04MAY2000	-5	Negative
		Load Dose 1	Yes	09MAY2000	0	
		Week 4	Yes	06JUN2000	28	
030.001.00225	IPP	Screening		06MAY2000	-3	Negative
		Clearance (Unsch.)	No	06MAY2000	-3	
		Load Dose 1	No	09MAY2000	0	
		Week 3 (Unsch.)	No	27MAY2000	18	
		Week 4	No	06JUN2000	28	
		Week 6 (Unsch. Fol. Up)	No	21JUN2000	43	
		Week 6 (Unsch. Fol. Up)	No	21JUN2000	43	
		Week 7 (Unsch. Fol. Up)	No	26JUN2000	48	
		Week 7 (Unsch. Fol. Up)	No	26JUN2000	48	
030.001.00226	IPP	Screening		04MAY2000	-5	Negative
		Load Dose 1	Yes	09MAY2000	0	
		Week 1 (Unsch.)	No	18MAY2000	9	
		Week 4	Yes	08JUN2000	30	
		Week 5 (Unsch.)	No	16JUN2000	38	
		Week 9 (Unsch. Fol. Up)	Yes	10JUL2000	62	
030.001.00247	IPP	Screening		05MAY2000	-5	Negative
		Load Dose 1	Yes	10MAY2000	0	
		Week 1 (Unsch.)	No	20MAY2000	10	
		Week 3 (Unsch.)	No	29MAY2000	19	
		Week 4	Yes	06JUN2000	27	
		Week 5 (Unsch.)	No	14JUN2000	35	
		Week 7 (Unsch.)	No	26JUN2000	47	
		Week 8	No	04JUL2000	55	
030.001.00251	IPP	Screening		05MAY2000	-5	Negative
		Load Dose 1	Yes	10MAY2000	0	
		Week 4	Yes	06JUN2000	27	
		Week 4 (Unsch.)	No	09JUN2000	30	
		Week 7 (Unsch. Fol. Up)	No	25JUN2000	46	
		Week 8 (Unsch. Fol. Up)	No	07JUL2000	58	
		Week 9 (Unsch. Fol. Up)	No	10JUL2000	61	
030.001.00253	IPP	Screening		05MAY2000	-5	Negative
		Load Dose 1	Yes	10MAY2000	0	
		Week 4	Yes	06JUN2000	27	

*Popn=Population (I=Intent-to-Treat, PP=Per Protocol)
**Day of test relative to start of prophylactic medication
Note: Pregnancy test data is listed for female subjects only.
Note: The date of test and result were recorded only at screening. Where test date is not recorded, it has been replaced with the visit date.
Tafenoquine = Tafenoquine 200mg, Mefloquine = Mefloquine 250mg

SB252263 - 030

Listing B.09

Pregnancy Test Results

Treatment: Placebo

Subject Number	Popn*	Visit	Test Done	Date of Test /Visit Date	Relative Day **	Result
030.001.00253	IPP	Week 8 (Unsch. Fol. Up)	No	03JUL2000	54	
		Week 11 (Unsch. Fol. Up)	No	25JUL2000	76	
030.001.00275	IPP	Screening		05MAY2000	-5	Negative
		Load Dose 1	Yes	10MAY2000	0	
		Week 2 (Unsch.)	No	22MAY2000	12	
		Week 2 (Unsch.)	No	27MAY2000	17	
		Week 4	Yes	06JUN2000	27	
		Week 8 (Unsch. Fol. Up)	Yes	05JUL2000	56	
030.001.00295		Screening		05MAY2000	-6	Negative
		Load Dose 1	Yes	11MAY2000	0	
		Week 2 (Unsch.)	No	23MAY2000	12	
		Week 4	Yes	06JUN2000	26	
		Week 8	Yes	04JUL2000	54	
030.001.00297	IPP	Screening		06MAY2000	-5	Negative
		Load Dose 1	Yes	11MAY2000	0	
		Week 4	Yes	07JUN2000	27	
		Week 8	Yes	06JUL2000	56	
		Week 12	Yes	31JUL2000	81	
		Week 16	No	30AUG2000	111	
030.001.00303	IPP	Screening		05MAY2000	-7	Negative
		Load Dose 1	Yes	12MAY2000	0	
		Load Dose (Unsch.)	No	13MAY2000	1	
		Week 3 (Unsch.)	No	04JUN2000	23	
		Week 4	Yes	06JUN2000	25	
		Week 4 (Unsch.)	No	11JUN2000	30	
		Week 7	Yes	03JUL2000	52	
		Week 12 (Unsch. Fol. Up)	Yes	05AUG2000	85	
		Week 13 (Unsch. Fol. Up)	No	08AUG2000	88	
		Week 13 (Unsch. Fol. Up)	No	14AUG2000	94	

*Popn=Population (I=Intent-to-Treat, PP=Per Protocol)
**Day of test relative to start of prophylactic medication
Note: Pregnancy test data is listed for female subjects only.
Note: The date of test and result were recorded only at screening. Where test date is not recorded, it has been replaced with the visit date.
Tafenoquine = Tafenoquine 200mg, Mefloquine = Mefloquine 250mg

SB252263 - 030

Listing B.09

Pregnancy Test Results

Treatment: Tafenoquine

Subject Number	Popn*	Visit	Test Done	Date of Test /Visit Date	Relative Day **	Result
030.001.00001	IPP	Screening		12APR2000	-26	Negative
		Screening (Unsch.)	No	02MAY2000	-6	
		Load Dose 1	Yes	08MAY2000	0	
		Week 1 (Unsch. Fol. Up)	No	16MAY2000	8	
		Week 3 (Unsch. Fol. Up)	No	30MAY2000	22	
		Week 4 (Fol. Up)	Yes	05JUN2000	28	
		Week 6 (Unsch. Fol. Up)		20JUN2000	43	
		Week 8 (Fol. Up)	No	03JUL2000	56	
		Week 9 (Unsch. Fol. Up)	No	07JUL2000	60	
		Week 9 (Unsch. Fol. Up)	No	11JUL2000	64	
		Week 9 (Unsch. Fol. Up)	No	13JUL2000	66	
030.001.00004	IPP	Screening		13APR2000	-25	Negative
		Screening (Unsch.)	No	01MAY2000	-7	
		Clearance (Unsch.)	No	07MAY2000	-1	
		Load Dose 1	Yes	08MAY2000	0	
		Week 3 (Unsch. Fol. Up)	No	01JUN2000	24	
		Week 4 (Fol. Up)	Yes	04JUN2000	27	
		Week 6 (Unsch. Fol. Up)	No	19JUN2000	42	
		Week 6 (Unsch. Fol. Up)	No	21JUN2000	44	
		Week 8 (Fol. Up)	Yes	03JUL2000	56	
		Week 10 (Unsch. Fol. Up)	No	14JUL2000	67	
030.001.00006	IPP	Screening		10APR2000	-28	Negative
		Load Dose 1	Yes	08MAY2000	0	
		Week 1 (Unsch.)	No	14MAY2000	6	
		Week 3 (Unsch. Fol. Up)	No	26MAY2000	18	
		Week 3 (Unsch. Fol. Up)	No	29MAY2000	21	
		Week 4 (Unsch. Fol. Up)	No	02JUN2000	25	
		Week 4 (Fol. Up)	Yes	05JUN2000	28	
		Week 7 (Unsch. Fol. Up)	No	28JUN2000	51	
		Week 8 (Fol. Up)	Yes	03JUL2000	56	
		Week 10 (Unsch. Fol. Up)	No	19JUL2000	72	
030.001.00024	IPP	Screening		11APR2000	-27	Negative
		Load Dose 1	Yes	08MAY2000	0	
		Week 4	Yes	08JUN2000	31	
		Week 5 (Unsch.)	No	12JUN2000	35	
		Week 7 (Unsch. Fol. Up)	No	27JUN2000	50	
		Week 8 (Fol. Up)	Yes	03JUL2000	56	

*Popn=Population (I=Intent-to-Treat, PP=Per Protocol)
**Day of test relative to start of prophylactic medication
Note: Pregnancy test data is listed for female subjects only.
Note: The date of test and result were recorded only at screening. Where test date is not recorded, it has been replaced with the visit date.
Tafenoquine = Tafenoquine 200mg, Mefloquine = Mefloquine 250mg

SB252263 - 030

Listing B.09

Pregnancy Test Results

Treatment: Tafenoquine

Subject Number	Popn*	Visit	Test Done	Date of Test /Visit Date	Relative Day **	Result
030.001.00030	IPP	Screening		14APR2000	-24	Negative
		Screening (Unsch.)	No	26APR2000	-12	
		Clearance (Unsch.)	No	04MAY2000	-4	
		Clearance (Unsch.)	No	06MAY2000	-2	
		Load Dose 1	Yes	08MAY2000	0	
		Week 2 (Unsch.)	No	22MAY2000	14	
		Week 3 (Unsch.)	No	29MAY2000	21	
		Week 4	Yes	05JUN2000	28	
		Week 5 (Unsch.)	No	09JUN2000	32	
		Week 8	Yes	03JUL2000	56	
		Week 11 (Unsch. Fol. Up)	No	24JUL2000	77	
		Week 13 (Unsch. Fol. Up)	No	07AUG2000	91	
		Week 14 (Unsch. Fol. Up)	No	14AUG2000	98	
		Week 14 (Unsch. Fol. Up)	No	17AUG2000	101	
030.001.00040	IPP	Screening		13APR2000	-25	Negative
		Clearance (Unsch.)	No	06MAY2000	-2	
		Load Dose 1	Yes	08MAY2000	0	
		Week 2 (Unsch.)	No	21MAY2000	13	
		Week 4 (Fol. Up)	Yes	04JUN2000	27	
		Week 6 (Unsch. Fol. Up)		21JUN2000	44	
		Week 8 (Fol. Up)	No	06JUL2000	59	
		Week 10 (Unsch. Fol. Up)	No	15JUL2000	68	
030.001.00060	IPP	Screening		13APR2000	-25	Negative
		Clearance (Unsch.)	No	06MAY2000	-2	
		Load Dose 1	Yes	08MAY2000	0	
		Week 1 (Unsch.)	No	16MAY2000	8	
		Week 3 (Unsch.)	No	26MAY2000	18	
		Week 4	Yes	05JUN2000	28	
		Week 6 (Unsch.)	No	20JUN2000	43	
		Week 8	Yes	03JUL2000	56	
		Week 10 (Unsch.)	No	14JUL2000	67	
		Week 12 (Unsch. Fol. Up)	No	01AUG2000	85	
030.001.00070	IPP	Screening		03MAY2000	-5	Negative
		Clearance (Unsch.)	No	06MAY2000	-2	
		Load Dose 1	Yes	08MAY2000	0	
		Week 3 (Unsch.)	No	29MAY2000	21	
		Week 4	Yes	05JUN2000	28	
		Week 7 (Unsch.)	No	28JUN2000	51	

*Popn=Population (I=Intent-to-Treat, PP=Per Protocol)
**Day of test relative to start of prophylactic medication
Note: Pregnancy test data is listed for female subjects only.
Note: The date of test and result were recorded only at screening. Where test date is not recorded, it has been replaced with the visit date.
Tafenoquine = Tafenoquine 200mg, Mefloquine = Mefloquine 250mg

SB252263 - 030

Listing B.09

Pregnancy Test Results

Treatment: Tafenoquine

Subject Number	Popn*	Visit	Test Done	Date of Test /Visit Date	Relative Day **	Result
030.001.00070	IPP	Week 8	Yes	03JUL2000	56	
		Week 10 (Unsch. Fol. Up)	No	18JUL2000	71	
		Week 11 (Unsch. Fol. Up)	No	27JUL2000	80	
030.001.00085	IPP	Screening		03MAY2000	-5	Negative
		Load Dose 1	Yes	08MAY2000	0	
		Load Dose (Unsch.)	No	10MAY2000	2	
		Week 1 (Unsch.)	No	15MAY2000	7	
		Week 2 (Unsch.)	No	22MAY2000	14	
		Week 4	Yes	05JUN2000	28	
		Week 8	Yes	03JUL2000	56	
		Week 10 (Unsch.)	No	20JUL2000	73	
		Week 12	Yes	01AUG2000	85	
		Week 14 (Unsch. Fol. Up)	No	14AUG2000	98	
		Week 17 (Unsch. Fol. Up)	No	04SEP2000	119	
030.001.00093	IPP	Screening		03MAY2000	-5	Negative
		Load Dose 1	Yes	08MAY2000	0	
		Week 3 (Unsch.)	No	30MAY2000	22	
		Week 4 (Unsch.)	No	03JUN2000	26	
		Week 4	Yes	06JUN2000	29	
		Week 6 (Unsch.)	No	16JUN2000	39	
		Week 7 (Unsch.)	No	26JUN2000	49	
		Week 8	Yes	03JUL2000	56	
		Week 9 (Unsch.)	No	11JUL2000	64	
		Week 9 (Unsch.)	No	12JUL2000	65	
		Week 12	Yes	31JUL2000	84	
		Week 16	No	29AUG2000	113	
		Week 16 (Unsch.)	Yes	31AUG2000	115	
		Week 20 (Unsch. Fol. Up)	Yes	25SEP2000	140	
030.001.00100	IPP	Screening		03MAY2000	-5	Negative
		Load Dose 1	Yes	08MAY2000	0	
		Week 1 (Unsch.)	No	16MAY2000	8	
		Week 3 (Unsch.)	No	01JUN2000	24	
		Week 4	Yes	04JUN2000	27	
		Week 6 (Unsch.)	No	19JUN2000	42	
		Week 6 (Unsch.)	No	20JUN2000	43	
		Week 8 (Fol. Up)	Yes	03JUL2000	56	
		Week 10 (Unsch. Fol. Up)	No	17JUL2000	70	
		Week 10 (Unsch. Fol. Up)	No	18JUL2000	71	

*Popn=Population (I=Intent-to-Treat, PP=Per Protocol)
**Day of test relative to start of prophylactic medication
Note: Pregnancy test data is listed for female subjects only.
Note: The date of test and result were recorded only at screening. Where test date is not recorded, it has been replaced with the visit date.
Tafenoquine = Tafenoquine 200mg, Mefloquine = Mefloquine 250mg

SB252263 - 030

Listing B.09

Pregnancy Test Results

Treatment: Tafenoquine

Subject Number	Popn*	Visit	Test Done	Date of Test /Visit Date	Relative Day **	Result
030.001.00100	IPP	Week 13 (Unsch. Fol. Up)	No	09AUG2000	93	
		Week 14 (Unsch. Fol. Up)	No	16AUG2000	100	
030.001.00107	IPP	Screening (Unsch.)	No	21APR2000	-17	
		Screening (Unsch.)	No	01MAY2000	-7	
		Screening		03MAY2000	-5	Negative
		Clearance (Unsch.)	No	04MAY2000	-4	
		Load Dose 1	Yes	08MAY2000	0	
		Week 4	Yes	06JUN2000	29	
		Week 6 (Unsch.)	No	20JUN2000	43	
		Week 8	Yes	04JUL2000	57	
		Week 12	Yes	01AUG2000	85	
		Week 15 (Unsch. Fol. Up)	No	22AUG2000	106	
030.001.00112	IPP	Screening		03MAY2000	-5	Negative
		Load Dose 1	Yes	08MAY2000	0	
		Week 3 (Unsch.)	No	29MAY2000	21	
		Week 3 (Unsch.)	No	30MAY2000	22	
		Week 4	Yes	04JUN2000	27	
		Week 8 (Fol. Up)	Yes	03JUL2000	56	
		Week 9 (Unsch. Fol. Up)	No	13JUL2000	66	
030.001.00128	IPP	Screening		04MAY2000	-5	Negative
		Clearance (Unsch.)	No	05MAY2000	-4	
		Load Dose 1	Yes	09MAY2000	0	
		Week 3 (Unsch.)	No	27MAY2000	18	
		Week 4	Yes	05JUN2000	27	
		Week 8 (Unsch.)	No	02JUL2000	54	
		Week 8	Yes	03JUL2000	55	
		Week 9 (Unsch.)	No	11JUL2000	63	
		Week 12	Yes	31JUL2000	83	
		Week 15 (Unsch.)	No	21AUG2000	104	
		Week 16	No	28AUG2000	111	
		Week 17 (Unsch.)	No	05SEP2000	119	
		Week 20	Yes	25SEP2000	139	
		Week 24	Yes	23OCT2000	167	
		Week 26 (Unsch. Fol. Up)	No	08NOV2000	183	
030.001.00129	IPP	Screening		04MAY2000	-5	Negative
		Load Dose 1	Yes	09MAY2000	0	
		Week 2 (Unsch.)	No	22MAY2000	13	

*Popn=Population (I=Intent-to-Treat, PP=Per Protocol)
**Day of test relative to start of prophylactic medication
Note: Pregnancy test data is listed for female subjects only.
Note: The date of test and result were recorded only at screening. Where test date is not recorded, it has been replaced with the visit date.
Tafenoquine = Tafenoquine 200mg, Mefloquine = Mefloquine 250mg

SB252263 - 030

Listing B.09

Pregnancy Test Results

Treatment: Tafenoquine

Subject Number	Popn*	Visit	Test Done	Date of Test /Visit Date	Relative Day **	Result
030.001.00129	IPP	Week 3 (Unsch.)	No	31MAY2000	22	
		Week 4	Yes	06JUN2000	28	
		Week 5 (Unsch.)	No	13JUN2000	35	
		Week 8	Yes	03JUL2000	55	
		Week 12	Yes	01AUG2000	84	
		Week 15 (Unsch. Fol. Up)	No	21AUG2000	104	
030.001.00150	IPP	Screening		04MAY2000	-5	Negative
		Load Dose 1	Yes	09MAY2000	0	
		Week 1 (Unsch.)	No	15MAY2000	6	
		Week 4	Yes	07JUN2000	29	
		Week 5 (Unsch.)	No	13JUN2000	35	
		Week 5 (Unsch.)	No	14JUN2000	36	
		Week 8	No	03JUL2000	55	
		Week 10 (Unsch. Fol. Up)	No	17JUL2000	69	
030.001.00155	IPP	Screening		04MAY2000	-5	Negative
		Load Dose 1	Yes	09MAY2000	0	
		Week 1 (Unsch.)	No	12MAY2000	3	
		Week 2 (Unsch.)	No	21MAY2000	12	
		Week 3 (Unsch.)	No	01JUN2000	23	
		Week 4	Yes	05JUN2000	27	
		Week 8	Yes	03JUL2000	55	
		Week 12	Yes	31JUL2000	83	
		Week 13 (Unsch. Fol. Up)	No	07AUG2000	90	
030.001.00158	IPP	Screening		04MAY2000	-5	Negative
		Load Dose 1	Yes	09MAY2000	0	
		Week 2 (Unsch.)	No	22MAY2000	13	
		Week 4 (Fol. Up)	Yes	05JUN2000	27	
		Week 8 (Fol. Up)	Yes	05JUL2000	57	
		Week 9 (Unsch. Fol. Up)	No	10JUL2000	62	
		Week 10 (Unsch. Fol. Up)	No	17JUL2000	69	
030.001.00159	IPP	Screening		12APR2000	-27	Negative
		Load Dose 1	Yes	09MAY2000	0	
		Week 1 (Unsch.)	No	15MAY2000	6	
		Week 4	Yes	05JUN2000	27	
		Week 7 (Unsch.)	No	28JUN2000	50	
		Week 8	No	03JUL2000	55	
		Week 12	Yes	01AUG2000	84	

*Popn=Population (I=Intent-to-Treat, PP=Per Protocol)
**Day of test relative to start of prophylactic medication
Note: Pregnancy test data is listed for female subjects only.
Note: The date of test and result were recorded only at screening. Where test date is not recorded, it has been replaced with the visit date.
Tafenoquine = Tafenoquine 200mg, Mefloquine = Mefloquine 250mg

SB252263 - 030

Listing B.09

Pregnancy Test Results

Treatment: Tafenoquine

Subject Number	Popn*	Visit	Test Done	Date of Test /Visit Date	Relative Day **	Result
030.001.00159	IPP	Week 16	Yes	28AUG2000	111	
		Week 17 (Unsch.)	No	04SEP2000	118	
		Week 20	Yes	25SEP2000	139	
		Week 23 (Unsch. Fol. Up)	No	17OCT2000	161	
030.001.00161	IPP	Screening		04MAY2000	-5	Negative
		Load Dose 1	Yes	09MAY2000	0	
		Load Dose (Unsch.)	No	10MAY2000	1	
		Week 2 (Unsch.)	No	24MAY2000	15	
		Week 3 (Unsch.)	No	31MAY2000	22	
		Week 4 (Unsch.)	No	04JUN2000	26	
		Week 4	Yes	05JUN2000	27	
		Week 6 (Unsch.)	No	19JUN2000	41	
		Week 8	Yes	03JUL2000	55	
030.001.00173	I	Screening (Unsch.)	No	20APR2000	-19	
		Screening		04MAY2000	-5	Negative
		Load Dose 1	Yes	09MAY2000	0	
		Week 4	Yes	04JUN2000	26	
		Week 8 (Fol. Up)	Yes	03JUL2000	55	
030.001.00176	IPP	Screening		04MAY2000	-5	Negative
		Load Dose 1	Yes	09MAY2000	0	
		Week 1 (Unsch.)	No	17MAY2000	8	
		Week 5 (Unsch. Fol. Up)	No	12JUN2000	34	
		Week 5 (Unsch. Fol. Up)	No	12JUN2000	34	
030.001.00201	IPP	Screening		03MAY2000	-6	Negative
		Load Dose 1	Yes	09MAY2000	0	
		Week 4	Yes	06JUN2000	28	
		Week 6 (Unsch. Fol. Up)	No	23JUN2000	45	
030.001.00203	IPP	Screening		03MAY2000	-6	Negative
		Load Dose 1	Yes	09MAY2000	0	
		Week 4 (Unsch.)	No	05JUN2000	27	
		Week 4	Yes	07JUN2000	29	
		Week 11 (Unsch. Fol. Up)	No	26JUL2000	78	
030.001.00205	IPP	Screening		06MAY2000	-3	Negative
		Load Dose 1	Yes	09MAY2000	0	
		Week 1 (Unsch.)	No	17MAY2000	8	

*Popn=Population (I=Intent-to-Treat, PP=Per Protocol)
**Day of test relative to start of prophylactic medication
Note: Pregnancy test data is listed for female subjects only.
Note: The date of test and result were recorded only at screening. Where test date is not recorded, it has been replaced with the visit date.
Tafenoquine = Tafenoquine 200mg, Mefloquine = Mefloquine 250mg

SB252263 - 030

Listing B.09

Pregnancy Test Results

Treatment: Tafenoquine

Subject Number	Popn*	Visit	Test Done	Date of Test /Visit Date	Relative Day **	Result
030.001.00205	IPP	Week 4	Yes	06JUN2000	28	
030.001.00231	IPP	Screening		04MAY2000	-5	Negative
		Clearance (Unsch.)	No	05MAY2000	-4	
		Load Dose 1	Yes	09MAY2000	0	
		Week 1 (Unsch.)	No	15MAY2000	6	
		Week 1 (Unsch.)	No	16MAY2000	7	
		Week 4	Yes	06JUN2000	28	
		Week 8	Yes	04JUL2000	56	
		Week 11 (Unsch.)	No	25JUL2000	77	
		Week 12	Yes	31JUL2000	83	
		Week 15 (Unsch.)	No	19AUG2000	102	
		Week 16	Yes	29AUG2000	112	
		Week 16 (Unsch.)	No	31AUG2000	114	
		Week 20 (Unsch. Fol. Up)	Yes	25SEP2000	139	
030.001.00233	IPP	Screening		05MAY2000	-5	Negative
		Clearance (Unsch.)	No	09MAY2000	-1	
		Load Dose 1	Yes	10MAY2000	0	
		Load Dose (Unsch.)	No	11MAY2000	1	
		Week 1 (Unsch.)	No	13MAY2000	3	
		Week 1 (Unsch.)	No	14MAY2000	4	
		Week 1 (Unsch.)	No	18MAY2000	8	
		Week 4	Yes	07JUN2000	28	
		Week 5 (Unsch.)	No	15JUN2000	36	
		Week 6 (Unsch.)	No	22JUN2000	43	
		Week 6 (Unsch.)	No	23JUN2000	44	
		Week 6 (Unsch.)	No	24JUN2000	45	
		Week 7 (Unsch.)	No	25JUN2000	46	
		Week 7 (Unsch.)	No	26JUN2000	47	
		Week 7 (Unsch.)	No	27JUN2000	48	
		Week 7 (Unsch.)	No	28JUN2000	49	
		Week 7 (Unsch.)	No	29JUN2000	50	
		Week 7 (Unsch.)	No	30JUN2000	51	
		Week 8	No	06JUL2000	57	
		Week 9 (Unsch.)	No	11JUL2000	62	
		Week 9 (Unsch.)	No	14JUL2000	65	
		Week 10 (Unsch.)	No	19JUL2000	70	
		Week 10 (Unsch.)	No	21JUL2000	72	
		Week 11 (Unsch.)	No	26JUL2000	77	
		Week 12	Yes	01AUG2000	83	

*Popn=Population (I=Intent-to-Treat, PP=Per Protocol)
**Day of test relative to start of prophylactic medication
Note: Pregnancy test data is listed for female subjects only.
Note: The date of test and result were recorded only at screening. Where test date is not recorded, it has been replaced with the visit date.
Tafenoquine = Tafenoquine 200mg, Mefloquine = Mefloquine 250mg

SB252263 - 030

Listing B.09

Pregnancy Test Results

Treatment: Tafenoquine

Subject Number	Popn*	Visit	Test Done	Date of Test /Visit Date	Relative Day **	Result
030.001.00233	IPP	Week 12 (Unsch.)	No	01AUG2000	83	
		Week 14 (Unsch.)	No	14AUG2000	96	
		Week 16	Yes	28AUG2000	110	
030.001.00246	IPP	Screening		05MAY2000	-5	Negative
		Load Dose 1	Yes	10MAY2000	0	
		Week 4	Yes	06JUN2000	27	
		Week 8	Yes	04JUL2000	55	
		Week 10 (Unsch. Fol. Up)	No	21JUL2000	72	
		Week 12 (Unsch. Fol. Up)	Yes	01AUG2000	83	
030.001.00254	I	Screening		05MAY2000	-5	Negative
		Clearance (Unsch.)	No	06MAY2000	-4	
		Load Dose 1	Yes	10MAY2000	0	
		Week 2 (Unsch.)	No	27MAY2000	17	
		Week 3 (Unsch.)	No	29MAY2000	19	
		Week 4	No	08JUN2000	29	
030.001.00261		Screening		05MAY2000	-5	Negative
		Load Dose 1	Yes	10MAY2000	0	
		Week 1 (Unsch. Fol. Up)	No	13MAY2000	3	
		Week 1 (Unsch. Fol. Up)	No	15MAY2000	5	
		Week 1 (Unsch. Fol. Up)	No	16MAY2000	6	
		Week 2 (Unsch. Fol. Up)	No	23MAY2000	13	
030.001.00264	IPP	Screening		05MAY2000	-5	Negative
		Load Dose 1	Yes	10MAY2000	0	
		Week 1 (Unsch.)	No	17MAY2000	7	
		Week 4	Yes	06JUN2000	27	
		Week 7 (Unsch.)	No	29JUN2000	50	
		Week 8	Yes	04JUL2000	55	
030.001.00272	IPP	Screening		05MAY2000	-5	Negative
		Load Dose 1	Yes	10MAY2000	0	
		Week 3 (Unsch.)	No	01JUN2000	22	
		Week 4	Yes	07JUN2000	28	
		Week 5 (Unsch.)	No	12JUN2000	33	
		Week 8	Yes	04JUL2000	55	
030.001.00282	IPP	Screening		05MAY2000	-5	Negative
		Load Dose 1	Yes	10MAY2000	0	

*Popn=Population (I=Intent-to-Treat, PP=Per Protocol)
**Day of test relative to start of prophylactic medication
Note: Pregnancy test data is listed for female subjects only.
Note: The date of test and result were recorded only at screening. Where test date is not recorded, it has been replaced with the visit date.
Tafenoquine = Tafenoquine 200mg, Mefloquine = Mefloquine 250mg

Listing B.09

Pregnancy Test Results

Treatment: Tafenoquine

Subject Number	Popn*	Visit	Test Done	Date of Test /Visit Date	Relative Day **	Result
030.001.00282	IPP	Load Dose (Unsch.)	No	10MAY2000	0	
		Week 2 (Unsch.)	No	22MAY2000	12	
		Week 4	Yes	06JUN2000	27	
		Week 5 (Unsch.)	No	13JUN2000	34	
		Week 6 (Unsch.)	No	18JUN2000	39	
		Week 8	Yes	04JUL2000	55	
		Week 11 (Unsch.)	No	29JUL2000	80	
		Week 12	Yes	01AUG2000	83	
		Week 16	Yes	28AUG2000	110	
		Week 18 (Unsch.)	No	13SEP2000	126	
		Week 21 (Unsch. Fol. Up)	No	03OCT2000	146	
030.001.00293	IPP	Screening (Unsch.)	No	29APR2000	-12	
		Screening		05MAY2000	-6	Negative
		Load Dose 1	No	11MAY2000	0	
		Week 2 (Unsch.)	No	27MAY2000	16	
		Week 4	No	07JUN2000	27	
		Week 5 (Unsch.)	No	15JUN2000	35	
		Week 6 (Unsch.)	No	20JUN2000	40	
		Week 8	Yes	08JUL2000	58	
		Week 9 (Unsch.)	No	10JUL2000	60	
		Week 9 (Unsch.)	No	14JUL2000	64	
		Week 12	No	04AUG2000	85	
		Week 12 (Unsch.)	No	04AUG2000	85	
		Week 16	Yes	29AUG2000	110	
		Week 20	Yes	25SEP2000	137	
		Week 24	Yes	25OCT2000	167	
030.001.00294	IPP	Screening		05MAY2000	-6	Negative
		Load Dose 1	Yes	11MAY2000	0	
		Week 1 (Unsch.)	No	15MAY2000	4	
		Week 4	Yes	06JUN2000	26	
		Week 8	Yes	03JUL2000	53	
		Week 8 (Unsch.)	No	05JUL2000	55	
030.001.00296	IPP	Screening		07MAY2000	-4	Negative
		Load Dose 1	Yes	11MAY2000	0	
		Week 2 (Unsch.)	No	24MAY2000	13	
		Week 4	Yes	06JUN2000	26	
		Week 5 (Unsch.)	No	15JUN2000	35	
		Week 8	Yes	03JUL2000	53	

*Popn=Population (I=Intent-to-Treat, PP=Per Protocol)
**Day of test relative to start of prophylactic medication
Note: Pregnancy test data is listed for female subjects only.
Note: The date of test and result were recorded only at screening. Where test date is not recorded, it has been replaced with the visit date.
Tafenoquine = Tafenoquine 200mg, Mefloquine = Mefloquine 250mg

SB252263 - 030

Listing B.09

Pregnancy Test Results

Treatment: Tafenoquine

Subject Number	Popn*	Visit	Test Done	Date of Test /Visit Date	Relative Day **	Result
030.001.00296	IPP	Week 12	Yes	31JUL2000	81	
		Week 16	Yes	28AUG2000	109	
		Week 20	Yes	27SEP2000	139	
		Week 20 (Unsch.)	Yes	27SEP2000	139	
		Week 24	Yes	23OCT2000	165	
030.001.00302	I	Screening		05MAY2000	-7	Negative
		Load Dose 1	Yes	12MAY2000	0	
		Week 5 (Unsch. Fol. Up)	Yes	14JUN2000	33	

*Popn=Population (I=Intent-to-Treat, PP=Per Protocol)
**Day of test relative to start of prophylactic medication
Note: Pregnancy test data is listed for female subjects only.
Note: The date of test and result were recorded only at screening. Where test date is not recorded, it has been replaced with the visit date.
Tafenoquine = Tafenoquine 200mg, Mefloquine = Mefloquine 250mg

SB252263 - 030

Listing B.09

Pregnancy Test Results

Treatment: Mefloquine

Subject Number	Popn*	Visit	Test Done	Date of Test /Visit Date	Relative Day **	Result
030.001.00003	I	Screening		10APR2000	-28	Negative
		Load Dose 1	Yes	08MAY2000	0	
		Load Dose (Unsch.)	No	10MAY2000	2	
		Week 1 (Unsch.)	No	12MAY2000	4	
		Week 1 (Unsch.)	No	15MAY2000	7	
		Week 3 (Unsch.)	No	31MAY2000	23	
		Week 4	Yes	05JUN2000	28	
		Week 8 (Fol. Up)	No	03JUL2000	56	
030.001.00007	IPP	Screening		11APR2000	-27	Negative
		Load Dose 1	Yes	08MAY2000	0	
		Week 1 (Unsch.)	No	11MAY2000	3	
		Week 2 (Unsch.)	No	19MAY2000	11	
		Week 4	Yes	05JUN2000	28	
		Week 7 (Unsch.)	No	23JUN2000	46	
		Week 8	Yes	03JUL2000	56	
		Week 9 (Unsch.)	No	13JUL2000	66	
		Week 12 (Unsch.)	No	29JUL2000	82	
		Week 12	Yes	31JUL2000	84	
		Week 16	Yes	28AUG2000	112	
		Week 20	Yes	25SEP2000	140	
		Week 23 (Unsch. Fol. Up)	No	19OCT2000	164	
		Week 24 (Fol. Up)	Yes	23OCT2000	168	
		Week 24 (Unsch. Fol. Up)	No	24OCT2000	169	
030.001.00010	IPP	Screening		11APR2000	-27	Negative
		Screening (Unsch.)	No	16APR2000	-22	
		Load Dose 1	Yes	08MAY2000	0	
		Week 2 (Unsch.)	No	22MAY2000	14	
		Week 4	Yes	05JUN2000	28	
		Week 5 (Unsch. Fol. Up)	No	13JUN2000	36	
		Week 11 (Unsch. Fol. Up)	No	25JUL2000	78	
030.001.00018	IPP	Screening		11APR2000	-27	Negative
		Screening (Unsch.)	No	26APR2000	-12	
		Load Dose 1	Yes	08MAY2000	0	
		Load Dose (Unsch.)	No	09MAY2000	1	
		Week 1 (Unsch.)	No	15MAY2000	7	
		Week 4	Yes	06JUN2000	29	
		Week 5 (Unsch.)	No	09JUN2000	32	
		Week 5 (Unsch.)	No	13JUN2000	36	

*Popn=Population (I=Intent-to-Treat, PP=Per Protocol)
**Day of test relative to start of prophylactic medication
Note: Pregnancy test data is listed for female subjects only.
Note: The date of test and result were recorded only at screening. Where test date is not recorded, it has been replaced with the visit date.
Tafenoquine = Tafenoquine 200mg, Mefloquine = Mefloquine 250mg

SB252263 - 030

Listing B.09

Pregnancy Test Results

Treatment: Mefloquine

Subject Number	Popn*	Visit	Test Done	Date of Test /Visit Date	Relative Day **	Result
030.001.00018	IPP	Week 8	Yes	03JUL2000	56	
		Week 9 (Unsch. Fol. Up)	No	11JUL2000	64	
		Week 13 (Unsch. Fol. Up)	No	08AUG2000	92	
030.001.00021	IPP	Screening		10APR2000	-28	Negative
		Screening (Unsch.)	No	17APR2000	-21	
		Screening (Unsch.)	No	02MAY2000	-6	
		Clearance (Unsch.)	No	06MAY2000	-2	
		Load Dose 1	Yes	08MAY2000	0	
		Week 1 (Unsch.)	No	11MAY2000	3	
		Week 1 (Unsch.)	No	16MAY2000	8	
		Week 4	Yes	05JUN2000	28	
		Week 8	No	03JUL2000	56	
		Week 11 (Unsch.)	No	21JUL2000	74	
		Week 12	Yes	31JUL2000	84	
		Week 16	Yes	28AUG2000	112	
		Week 20	Yes	25SEP2000	140	
		Week 21 (Unsch.)	No	02OCT2000	147	
		Week 24	Yes	23OCT2000	168	
		Week 26 (Unsch. Fol. Up)	No	06NOV2000	182	
		Week 26 (Unsch. Fol. Up)	No	08NOV2000	184	
030.001.00023	I	Screening		10APR2000	-28	Negative
		Screening (Unsch.)	No	17APR2000	-21	
		Screening (Unsch.)	No	26APR2000	-12	
		Screening (Unsch.)	No	02MAY2000	-6	
		Clearance (Unsch.)	No	03MAY2000	-5	
		Clearance (Unsch.)	No	06MAY2000	-2	
		Load Dose 1	Yes	08MAY2000	0	
		Week 1 (Unsch.)	No	11MAY2000	3	
		Week 4 (Fol. Up)	Yes	05JUN2000	28	
		Week 6 (Unsch. Fol. Up)	No	19JUN2000	42	
		Week 6 (Unsch. Fol. Up)	No	20JUN2000	43	
		Week 8 (Fol. Up)	No	03JUL2000	56	
030.001.00052	IPP	Screening (Unsch.)	No	26APR2000	-12	
		Screening		03MAY2000	-5	Negative
		Clearance (Unsch.)	No	05MAY2000	-3	
		Load Dose 1	Yes	08MAY2000	0	
		Week 1 (Unsch.)	No	16MAY2000	8	
		Week 3 (Unsch.)	No	28MAY2000	20	

*Popn=Population (I=Intent-to-Treat, PP=Per Protocol)
**Day of test relative to start of prophylactic medication
Note: Pregnancy test data is listed for female subjects only.
Note: The date of test and result were recorded only at screening. Where test date is not recorded, it has been replaced with the visit date.
Tafenoquine = Tafenoquine 200mg, Mefloquine = Mefloquine 250mg

SB252263 - 030

Listing B.09

Pregnancy Test Results

Treatment: Mefloquine

Subject Number	Popn*	Visit	Test Done	Date of Test /Visit Date	Relative Day **	Result
030.001.00052	IPP	Week 4	Yes	05JUN2000	28	
		Week 5 (Unsch. Fol. Up)	No	13JUN2000	36	
		Week 6 (Unsch. Fol. Up)	No	19JUN2000	42	
		Week 6 (Unsch. Fol. Up)	No	19JUN2000	42	
		Week 10 (Unsch. Fol. Up)	No	14JUL2000	67	
		Week 12 (Unsch. Fol. Up)	No	31JUL2000	84	
		Week 15 (Unsch. Fol. Up)	No	22AUG2000	106	
030.001.00066	IPP	Screening (Unsch.)	No	25APR2000	-13	
		Screening		03MAY2000	-5	Negative
		Clearance (Unsch.)	No	06MAY2000	-2	
		Load Dose 1	Yes	08MAY2000	0	
		Week 2 (Unsch.)	No	19MAY2000	11	
		Week 3 (Unsch.)	No	26MAY2000	18	
		Week 4	Yes	06JUN2000	29	
		Week 5 (Unsch. Fol. Up)	No	13JUN2000	36	
		Week 6 (Unsch. Fol. Up)	No	21JUN2000	44	
		Week 9 (Unsch. Fol. Up)	Yes	11JUL2000	64	
030.001.00071	IPP	Screening (Unsch.)	No	27APR2000	-11	
		Screening		03MAY2000	-5	Negative
		Clearance (Unsch.)	No	03MAY2000	-5	
		Load Dose 1	Yes	08MAY2000	0	
		Week 4 (Unsch.)	No	02JUN2000	25	
		Week 4	Yes	06JUN2000	29	
		Week 4 (Unsch.)	No	06JUN2000	29	
		Week 8	Yes	03JUL2000	56	
		Week 11 (Unsch. Fol. Up)	No	27JUL2000	80	
		Week 12 (Unsch. Fol. Up)	Yes	31JUL2000	84	
030.001.00075	IPP	Screening		10APR2000	-28	Negative
		Clearance (Unsch.)	No	06MAY2000	-2	
		Load Dose 1	Yes	08MAY2000	0	
		Week 4	Yes	06JUN2000	29	
		Week 8	Yes	03JUL2000	56	
		Week 10 (Unsch. Fol. Up)	No	18JUL2000	71	
030.001.00097	IPP	Screening		11APR2000	-27	Negative
		Screening (Unsch.)	No	19APR2000	-19	
		Load Dose 1	Yes	08MAY2000	0	
		Week 2 (Unsch.)	No	23MAY2000	15	

*Popn=Population (I=Intent-to-Treat, PP=Per Protocol)
**Day of test relative to start of prophylactic medication
Note: Pregnancy test data is listed for female subjects only.
Note: The date of test and result were recorded only at screening. Where test date is not recorded, it has been replaced with the visit date.
Tafenoquine = Tafenoquine 200mg, Mefloquine = Mefloquine 250mg

SB252263 - 030

Listing B.09

Pregnancy Test Results

Treatment: Mefloquine

Subject Number	Popn*	Visit	Test Done	Date of Test /Visit Date	Relative Day **	Result
030.001.00097	IPP	Week 4	Yes	05JUN2000	28	
		Week 5 (Unsch.)	No	10JUN2000	33	
		Week 7 (Unsch. Fol. Up)	No	28JUN2000	51	
		Week 8 (Fol. Up)	Yes	03JUL2000	56	
		Week 8 (Unsch. Fol. Up)	No	05JUL2000	58	
		Week 12 (Unsch. Fol. Up)		01AUG2000	85	
030.001.00113	IPP	Screening		03MAY2000	-5	Negative
		Load Dose 1	Yes	08MAY2000	0	
		Week 1 (Unsch.)	No	15MAY2000	7	
		Week 2 (Unsch.)	No	24MAY2000	16	
		Week 3 (Unsch.)	No	27MAY2000	19	
		Week 4	Yes	05JUN2000	28	
		Week 4 (Unsch.)	No	07JUN2000	30	
		Week 6 (Unsch.)	No	20JUN2000	43	
		Week 8 (Fol. Up)	Yes	03JUL2000	56	
		Week 9 (Unsch. Fol. Up)	No	10JUL2000	63	
		Week 11 (Unsch. Fol. Up)	No	21JUL2000	74	
030.001.00117	IPP	Screening		03MAY2000	-5	Negative
		Load Dose 1	Yes	08MAY2000	0	
		Week 1 (Unsch.)	No	11MAY2000	3	
		Week 1 (Unsch.)	No	12MAY2000	4	
		Week 1 (Unsch.)	No	15MAY2000	7	
		Week 3 (Unsch.)	No	01JUN2000	24	
		Week 4	Yes	05JUN2000	28	
		Week 8 (Fol. Up)	Yes	03JUL2000	56	
		Week 12 (Unsch. Fol. Up)	No	28JUL2000	81	
030.001.00134	IPP	Screening		04MAY2000	-5	Negative
		Load Dose 1	Yes	09MAY2000	0	
		Week 4	No	06JUN2000	28	
		Week 5 (Unsch.)	No	13JUN2000	35	
		Week 7 (Unsch.)	No	27JUN2000	49	
		Week 8	Yes	04JUL2000	56	
		Week 15 (Unsch. Fol. Up)	No	22AUG2000	105	
030.001.00156	IPP	Screening (Unsch.)	No	02MAY2000	-7	
		Screening		04MAY2000	-5	Negative
		Clearance (Unsch.)	Yes	04MAY2000	-5	
		Load Dose 1	Yes	09MAY2000	0	

*Popn=Population (I=Intent-to-Treat, PP=Per Protocol)
**Day of test relative to start of prophylactic medication
Note: Pregnancy test data is listed for female subjects only.
Note: The date of test and result were recorded only at screening. Where test date is not recorded, it has been replaced with the visit date.
Tafenoquine = Tafenoquine 200mg, Mefloquine = Mefloquine 250mg

SB252263 - 030

Listing B.09

Pregnancy Test Results

Treatment: Mefloquine

Subject Number	Popn*	Visit	Test Done	Date of Test /Visit Date	Relative Day **	Result
030.001.00156	IPP	Week 2 (Unsch.)	No	24MAY2000	15	
		Week 4	Yes	04JUN2000	26	
		Week 6 (Unsch.)	No	18JUN2000	40	
		Week 6 (Unsch.)	No	20JUN2000	42	
		Week 7 (Unsch.)	No	26JUN2000	48	
		Week 8	Yes	03JUL2000	55	
030.001.00163	IPP	Screening (Unsch.)	No	20APR2000	-19	
		Screening		04MAY2000	-5	Negative
		Load Dose 1	Yes	09MAY2000	0	
		Week 1 (Unsch.)	No	15MAY2000	6	
		Week 4	Yes	05JUN2000	27	
		Week 11 (Unsch. Fol. Up)	No	26JUL2000	78	
030.001.00167	IPP	Screening (Unsch.)	No	30APR2000	-9	
		Screening		04MAY2000	-5	Negative
		Clearance (Unsch.)	No	06MAY2000	-3	
		Load Dose 1	Yes	09MAY2000	0	
		Week 4	Yes	04JUN2000	26	
		Week 6 (Unsch. Fol. Up)	No	19JUN2000	41	
		Week 8 (Fol. Up)	Yes	03JUL2000	55	
		Week 8 (Unsch. Fol. Up)	No	05JUL2000	57	
		Week 12 (Fol. Up)	No	31JUL2000	83	
		Week 17 (Unsch. Fol. Up)	No	05SEP2000	119	
030.001.00168	IPP	Screening		04MAY2000	-5	Negative
		Load Dose 1	Yes	09MAY2000	0	
		Week 3 (Unsch.)	No	31MAY2000	22	
		Week 4	Yes	05JUN2000	27	
030.001.00177	IPP	Screening		04MAY2000	-5	Negative
		Load Dose 1	Yes	09MAY2000	0	
		Week 2 (Unsch.)	No	24MAY2000	15	
		Week 4	Yes	06JUN2000	28	
		Week 8	Yes	04JUL2000	56	
		Week 11 (Unsch. Fol. Up)	No	27JUL2000	79	
030.001.00180	IPP	Screening		04MAY2000	-5	Negative
		Clearance (Unsch.)	No	04MAY2000	-5	
		Load Dose 1	Yes	09MAY2000	0	
		Week 1 (Unsch.)	No	19MAY2000	10	

*Popn=Population (I=Intent-to-Treat, PP=Per Protocol)
**Day of test relative to start of prophylactic medication
Note: Pregnancy test data is listed for female subjects only.
Note: The date of test and result were recorded only at screening. Where test date is not recorded, it has been replaced with the visit date.
Tafenoquine = Tafenoquine 200mg, Mefloquine = Mefloquine 250mg

SB252263 - 030

Listing B.09

Pregnancy Test Results

Treatment: Mefloquine

Subject Number	Popn*	Visit	Test Done	Date of Test /Visit Date	Relative Day **	Result
030.001.00180	IPP	Week 3 (Unsch.)	No	30MAY2000	21	
		Week 4	Yes	05JUN2000	27	
		Week 4 (Unsch.)	No	07JUN2000	29	
		Week 5 (Unsch.)	No	12JUN2000	34	
		Week 8	Yes	04JUL2000	56	
		Week 11 (Unsch.)	No	28JUL2000	80	
		Week 12	Yes	01AUG2000	84	
		Week 12 (Unsch.)	Yes	01AUG2000	84	
		Week 13 (Unsch.)	No	05AUG2000	88	
		Week 16	Yes	28AUG2000	111	
		Week 17 (Unsch.)	No	05SEP2000	119	
		Week 17 (Unsch.)	No	07SEP2000	121	
		Week 19 (Unsch.)	No	21SEP2000	135	
		Week 19 (Unsch.)	No	22SEP2000	136	
		Week 20	Yes	25SEP2000	139	
		Week 20 (Unsch.)	No	26SEP2000	140	
		Week 22 (Unsch.)	No	13OCT2000	157	
		Week 24 (Unsch. Fol. Up)	No	24OCT2000	168	
		Week 25 (Unsch. Fol. Up)	No	03NOV2000	178	
		Week 26 (Unsch. Fol. Up)	No	08NOV2000	183	
		Week 27 (Unsch. Fol. Up)	No	11NOV2000	186	
030.001.00185	IPP	Screening		04MAY2000	-5	Negative
		Load Dose 1	Yes	09MAY2000	0	
		Week 4	Yes	06JUN2000	28	
		Week 6 (Unsch.)	No	21JUN2000	43	
		Week 8	No	06JUL2000	58	
		Week 10 (Unsch.)	No	18JUL2000	70	
		Week 12	Yes	03AUG2000	86	
		Week 16	Yes	28AUG2000	111	
		Week 17 (Unsch.)	No	08SEP2000	122	
		Week 20	Yes	26SEP2000	140	
		Week 24 (Unsch. Fol. Up)	Yes	23OCT2000	167	
030.001.00188	IPP	Screening		04MAY2000	-5	Negative
		Load Dose 1	Yes	09MAY2000	0	
		Load Dose (Unsch.)	No	11MAY2000	2	
		Week 2 (Unsch.)	No	23MAY2000	14	
		Week 2 (Unsch.)	No	25MAY2000	16	
		Week 4	Yes	05JUN2000	27	
		Week 6 (Unsch. Fol. Up)	No	21JUN2000	43	

*Popn=Population (I=Intent-to-Treat, PP=Per Protocol)
**Day of test relative to start of prophylactic medication
Note: Pregnancy test data is listed for female subjects only.
Note: The date of test and result were recorded only at screening. Where test date is not recorded, it has been replaced with the visit date.
Tafenoquine = Tafenoquine 200mg, Mefloquine = Mefloquine 250mg

SB252263 - 030

Listing B.09

Pregnancy Test Results

Treatment: Mefloquine

Subject Number	Popn*	Visit	Test Done	Date of Test /Visit Date	Relative Day **	Result
030.001.00188	IPP	Week 7 (Unsch. Fol. Up)	No	26JUN2000	48	
		Week 7 (Unsch. Fol. Up)	No	27JUN2000	49	
030.001.00189	I	Screening		04MAY2000	-5	Negative
		Load Dose 1	Yes	09MAY2000	0	
		Week 4 (Fol. Up)	Yes	07JUN2000	29	
		Week 8 (Fol. Up)	Yes	05JUL2000	57	
		Week 10 (Unsch. Fol. Up)	No	18JUL2000	70	
030.001.00198	IPP	Screening		04MAY2000	-5	Negative
		Clearance (Unsch.)	Yes	04MAY2000	-5	
		Load Dose 1	Yes	09MAY2000	0	
		Week 1 (Unsch.)	No	18MAY2000	9	
		Week 4	Yes	06JUN2000	28	
		Week 6 (Unsch.)	No	18JUN2000	40	
		Week 7 (Unsch. Fol. Up)	No	29JUN2000	51	
		Week 8 (Fol. Up)	Yes	04JUL2000	56	
		Week 9 (Unsch. Fol. Up)	No	10JUL2000	62	
		Week 9 (Unsch. Fol. Up)	No	10JUL2000	62	
		Week 11 (Unsch. Fol. Up)		26JUL2000	78	
030.001.00214	IPP	Screening		04MAY2000	-5	Negative
		Load Dose 1	Yes	09MAY2000	0	
		Week 4	Yes	07JUN2000	29	
		Week 5 (Unsch.)	No	11JUN2000	33	
		Week 12 (Unsch. Fol. Up)	No	30JUL2000	82	
030.001.00223	IPP	Screening		04MAY2000	-5	Negative
		Load Dose 1	Yes	09MAY2000	0	
		Week 4	Yes	06JUN2000	28	
		Week 4 (Unsch.)	No	07JUN2000	29	
		Week 6 (Unsch. Fol. Up)	No	18JUN2000	40	
		Week 7 (Unsch. Fol. Up)	No	24JUN2000	46	
030.001.00224	IPP	Screening		04MAY2000	-5	Negative
		Load Dose 1	Yes	09MAY2000	0	
		Week 4	Yes	06JUN2000	28	
		Week 7 (Unsch. Fol. Up)	No	25JUN2000	47	
030.001.00227	IPP	Screening		04MAY2000	-5	Negative
		Clearance (Unsch.)	No	08MAY2000	-1	

*Popn=Population (I=Intent-to-Treat, PP=Per Protocol)
**Day of test relative to start of prophylactic medication
Note: Pregnancy test data is listed for female subjects only.
Note: The date of test and result were recorded only at screening. Where test date is not recorded, it has been replaced with the visit date.
Tafenoquine = Tafenoquine 200mg, Mefloquine = Mefloquine 250mg

SB252263 - 030

Listing B.09

Pregnancy Test Results

Treatment: Mefloquine

Subject Number	Popn*	Visit	Test Done	Date of Test /Visit Date	Relative Day **	Result
030.001.00227	IPP	Load Dose 1	Yes	09MAY2000	0	
		Week 4	No	07JUN2000	29	
		Week 6 (Unsch.)	No	21JUN2000	43	
		Week 6 (Unsch. Fol. Up)	No	22JUN2000	44	
		Week 7 (Unsch. Fol. Up)	No	29JUN2000	51	
		Week 8 (Unsch. Fol. Up)	No	03JUL2000	55	
		Week 9 (Unsch. Fol. Up)	No	10JUL2000	62	
		Week 9 (Unsch. Fol. Up)	No	11JUL2000	63	
		Week 9 (Unsch. Fol. Up)	No	13JUL2000	65	
030.001.00232	IPP	Screening		05MAY2000	-5	Negative
		Load Dose 1	Yes	10MAY2000	0	
		Week 1 (Unsch.)	No	15MAY2000	5	
		Week 3 (Unsch. Fol. Up)	No	29MAY2000	19	
		Week 3 (Unsch. Fol. Up)	No	03JUN2000	24	
		Week 4 (Unsch. Fol. Up)	No	05JUN2000	26	
030.001.00235	IPP	Screening (Unsch.)	No	02MAY2000	-8	
		Screening		05MAY2000	-5	Negative
		Clearance (Unsch.)	No	06MAY2000	-4	
		Load Dose 1	Yes	10MAY2000	0	
		Week 2 (Unsch.)	No	24MAY2000	14	
		Week 4	Yes	08JUN2000	29	
		Week 5 (Unsch.)	No	14JUN2000	35	
030.001.00273	IPP	Screening		05MAY2000	-5	Negative
		Load Dose 1	Yes	10MAY2000	0	
		Week 3 (Unsch.)	No	29MAY2000	19	
		Week 4	Yes	06JUN2000	27	
		Week 8	Yes	04JUL2000	55	
		Week 12	Yes	01AUG2000	83	
		Week 12 (Unsch.)	Yes	01AUG2000	83	
		Week 14 (Unsch. Fol. Up)	No	16AUG2000	98	
030.001.00274	IPP	Screening		05MAY2000	-5	Negative
		Clearance (Unsch.)	No	06MAY2000	-4	
		Load Dose 1	Yes	10MAY2000	0	
		Week 1 (Unsch.)	No	13MAY2000	3	
		Week 1 (Unsch.)	No	19MAY2000	9	
		Week 4	Yes	06JUN2000	27	
		Week 4 (Unsch.)	No	10JUN2000	31	

*Popn=Population (I=Intent-to-Treat, PP=Per Protocol)
**Day of test relative to start of prophylactic medication
Note: Pregnancy test data is listed for female subjects only.
Note: The date of test and result were recorded only at screening. Where test date is not recorded, it has been replaced with the visit date.
Tafenoquine = Tafenoquine 200mg, Mefloquine = Mefloquine 250mg

SB252263 - 030

Listing B.09

Pregnancy Test Results

Treatment: Mefloquine

Subject Number	Popn*	Visit	Test Done	Date of Test /Visit Date	Relative Day **	Result
030.001.00289	IPP	Screening		05MAY2000	-6	Negative
		Load Dose 1	Yes	11MAY2000	0	
		Week 1 (Unsch.)	No	21MAY2000	10	
		Week 3 (Unsch.)	No	29MAY2000	18	
		Week 3 (Unsch.)	No	31MAY2000	20	
		Week 4	Yes	06JUN2000	26	
		Week 7 (Unsch.)	No	28JUN2000	48	
		Week 10 (Unsch. Fol. Up)	No	18JUL2000	68	
		Week 11 (Unsch. Fol. Up)	No	27JUL2000	77	

*Popn=Population (I=Intent-to-Treat, PP=Per Protocol)
**Day of test relative to start of prophylactic medication
Note: Pregnancy test data is listed for female subjects only.
Note: The date of test and result were recorded only at screening. Where test date is not recorded, it has been replaced with the visit date.
Tafenoquine = Tafenoquine 200mg, Mefloquine = Mefloquine 250mg

SB252263 - 030

Listing B.10

G6PD Test Results

Treatment : Placebo

Subject Number	Popn*	Date of First Test	Relative Days **	Result	Date of Second Test	Relative Days **	Result	Eligible For Study
030.001.00002	IPP	10APR2000	-28	Normal	18APR2000	-20	Normal	Yes
030.001.00005	IPP	10APR2000	-28	Normal	17APR2000	-21	Normal	Yes
030.001.00011	IPP	10APR2000	-28	Normal	17APR2000	-21	Normal	Yes
030.001.00012	IPP	10APR2000	-28	Normal	17APR2000	-21	Normal	Yes
030.001.00015	I	10APR2000	-28	Normal	20APR2000	-18	Normal	Yes
030.001.00019	IPP	10APR2000	-28	Normal	17APR2000	-21	Normal	Yes
030.001.00020	IPP	10APR2000	-28	Normal	17APR2000	-21	Normal	Yes
030.001.00022	IPP	13APR2000	-25	Normal	17APR2000	-21	Normal	Yes
030.001.00026	IPP	10APR2000	-28	Normal	17APR2000	-21	Normal	Yes
030.001.00027	IPP	12APR2000	-26	Normal	19APR2000	-19	Normal	Yes
030.001.00032	IPP	13APR2000	-25	Normal	17APR2000	-21	Normal	Yes
030.001.00033	IPP	11APR2000	-27	Normal	18APR2000	-20	Normal	Yes
030.001.00037	IPP	11APR2000	-27	Normal	17APR2000	-21	Normal	Yes
030.001.00039	IPP	14APR2000	-24	Normal	17APR2000	-21	Normal	Yes
030.001.00041	IPP	10APR2000	-28	Normal	17APR2000	-21	Normal	Yes
030.001.00044	IPP	10APR2000	-28	Normal	17APR2000	-21	Normal	Yes
030.001.00055	IPP	12APR2000	-26	Normal	18APR2000	-20	Normal	Yes
030.001.00056	I	11APR2000	-27	Normal	17APR2000	-21	Normal	Yes
030.001.00057	IPP	14APR2000	-24	Normal	18APR2000	-20	Normal	Yes
030.001.00059	IPP	13APR2000	-25	Normal	17APR2000	-21	Normal	Yes
030.001.00063	IPP	11APR2000	-27	Normal	18APR2000	-20	Normal	Yes
030.001.00064	IPP	11APR2000	-27	Normal	18APR2000	-20	Normal	Yes
030.001.00067	IPP	12APR2000	-26	Normal	18APR2000	-20	Normal	Yes
030.001.00072	IPP	12APR2000	-26	Normal	18APR2000	-20	Normal	Yes
030.001.00076	IPP	11APR2000	-27	Normal	17APR2000	-21	Normal	Yes
030.001.00078	IPP	12APR2000	-26	Normal	18APR2000	-20	Normal	Yes
030.001.00080	IPP	12APR2000	-26	Normal	19APR2000	-19	Normal	Yes
030.001.00081	IPP	11APR2000	-27	Normal	17APR2000	-21	Normal	Yes
030.001.00087	I	14APR2000	-24	Normal	18APR2000	-20	Normal	Yes
030.001.00088	IPP	14APR2000	-24	Normal	18APR2000	-20	Normal	Yes
030.001.00090	IPP	12APR2000	-26	Normal	18APR2000	-20	Normal	Yes
030.001.00092	IPP	12APR2000	-26	Normal	18APR2000	-20	Normal	Yes
030.001.00101	IPP	14APR2000	-24	Normal	17APR2000	-21	Normal	Yes
030.001.00103	IPP	11APR2000	-27	Normal	17APR2000	-21	Normal	Yes
030.001.00106	IPP	10APR2000	-28	Normal	18APR2000	-20	Normal	Yes
030.001.00108	IPP	14APR2000	-24	Normal	19APR2000	-19	Normal	Yes
030.001.00114	IPP	14APR2000	-24	Normal	17APR2000	-21	Normal	Yes
030.001.00115	IPP	17APR2000	-21	Normal	19APR2000	-19	Normal	Yes
030.001.00116	IPP	12APR2000	-26	Normal	18APR2000	-20	Normal	Yes
030.001.00119	IPP	11APR2000	-28	Normal	19APR2000	-20	Normal	Yes
030.001.00124	IPP	11APR2000	-28	Normal	17APR2000	-22	Normal	Yes
030.001.00125	IPP	10APR2000	-29	Normal	17APR2000	-22	Normal	Yes
030.001.00127	I	12APR2000	-27	Normal	17APR2000	-22	Normal	Yes

*Popn=Population (I=Intent-to-Treat, PP=Per Protocol)
**Day of test relative to start of prophylactic medication
Tafenoquine = Tafenoquine 200mg, Mefloquine = Mefloquine 250mg

SB252263 - 030

Listing B.10

G6PD Test Results

Treatment : Placebo

Subject Number	Popn*	Date of First Test	Relative Days **	Result	Date of Second Test	Relative Days **	Result	Eligible For Study
030.001.00131	IPP	10APR2000	-29	Normal	17APR2000	-22	Normal	Yes
030.001.00136	IPP	11APR2000	-28	Normal	18APR2000	-21	Normal	Yes
030.001.00137	IPP	11APR2000	-28	Normal	18APR2000	-21	Normal	Yes
030.001.00140	IPP	10APR2000	-29	Normal	17APR2000	-22	Normal	Yes
030.001.00141	IPP	11APR2000	-28	Normal	18APR2000	-21	Normal	Yes
030.001.00145	IPP	18APR2000	-21	Normal	19APR2000	-20	Normal	Yes
030.001.00151	IPP	11APR2000	-28	Normal	17APR2000	-22	Normal	Yes
030.001.00152	IPP	13APR2000	-26	Normal	17APR2000	-22	Normal	Yes
030.001.00154	IPP	13APR2000	-26	Normal	17APR2000	-22	Normal	Yes
030.001.00160	I	11APR2000	-28	Normal	20APR2000	-19	Normal	Yes
030.001.00162	IPP	11APR2000	-28	Normal	17APR2000	-22	Normal	Yes
030.001.00164	IPP	14APR2000	-25	Normal	17APR2000	-22	Normal	Yes
030.001.00166	IPP	10APR2000	-29	Normal	17APR2000	-22	Normal	Yes
030.001.00169	IPP	10APR2000	-29	Normal	17APR2000	-22	Normal	Yes
030.001.00172	IPP	10APR2000	-29	Normal	17APR2000	-22	Normal	Yes
030.001.00174	IPP	11APR2000	-28	Normal	11APR2000	-28	Normal	Yes
030.001.00175	IPP	17APR2000	-22	Normal	20APR2000	-19	Normal	Yes
030.001.00182	I	10APR2000	-29	Normal	18APR2000	-21	Normal	Yes
030.001.00183	IPP	11APR2000	-28	Normal	17APR2000	-22	Normal	Yes
030.001.00191	IPP	18APR2000	-21	Normal	20APR2000	-19	Normal	Yes
030.001.00192	IPP	11APR2000	-28	Normal	17APR2000	-22	Normal	Yes
030.001.00193	IPP	14APR2000	-25	Normal	18APR2000	-21	Normal	Yes
030.001.00197	IPP	17APR2000	-22	Normal	20APR2000	-19	Normal	Yes
030.001.00204	I	14APR2000	-25	Normal	18APR2000	-21	Normal	Yes
030.001.00208	IPP	11APR2000	-28	Normal	17APR2000	-22	Normal	Yes
030.001.00211	IPP	11APR2000	-28	Normal	17APR2000	-22	Normal	Yes
030.001.00212	IPP	13APR2000	-26	Normal	20APR2000	-19	Normal	Yes
030.001.00215	IPP	12APR2000	-27	Normal	17APR2000	-22	Normal	Yes
030.001.00219	IPP	12APR2000	-27	Normal	17APR2000	-22	Normal	Yes
030.001.00222	IPP	14APR2000	-25	Normal	20APR2000	-19	Normal	Yes
030.001.00225	IPP	10APR2000	-29	Normal	17APR2000	-22	Normal	Yes
030.001.00226	IPP	13APR2000	-26	Normal	18APR2000	-21	Normal	Yes
030.001.00229	IPP	13APR2000	-26	Normal	18APR2000	-21	Normal	Yes
030.001.00234	IPP	11APR2000	-29	Normal	19APR2000	-21	Normal	Yes
030.001.00236	IPP	11APR2000	-30	Normal	17APR2000	-24	Normal	Yes
030.001.00238	IPP	12APR2000	-28	Normal	18APR2000	-22	Normal	Yes
030.001.00242	IPP	14APR2000	-26	Normal	19APR2000	-21	Normal	Yes
030.001.00247	IPP	11APR2000	-29	Normal	18APR2000	-22	Normal	Yes
030.001.00248	IPP	13APR2000	-27	Normal	18APR2000	-22	Normal	Yes
030.001.00251	IPP	12APR2000	-28	Normal	18APR2000	-22	Normal	Yes
030.001.00253	IPP	13APR2000	-27	Normal	18APR2000	-22	Normal	Yes
030.001.00256	IPP	14APR2000	-26	Normal	18APR2000	-22	Normal	Yes
030.001.00257	IPP	14APR2000	-28	Normal	17APR2000	-25	Normal	Yes

*Popn=Population (I=Intent-to-Treat, PP=Per Protocol)
**Day of test relative to start of prophylactic medication
Tafenoquine = Tafenoquine 200mg, Mefloquine = Mefloquine 250mg

SB252263 - 030

Listing B.10

G6PD Test Results

Treatment : Placebo

Subject Number	Popn*	Date of First Test	Relative Days **	Result	Date of Second Test	Relative Days **	Result	Eligible For Study
030.001.00260	IPP	17APR2000	-23	Normal	20APR2000	-20	Normal	Yes
030.001.00268	IPP	14APR2000	-26	Normal	18APR2000	-22	Normal	Yes
030.001.00269	IPP	13APR2000	-27	Normal	18APR2000	-22	Normal	Yes
030.001.00270	IPP	13APR2000	-27	Normal	17APR2000	-23	Normal	Yes
030.001.00275	IPP	12APR2000	-28	Normal	18APR2000	-22	Normal	Yes
030.001.00277	IPP	13APR2000	-27	Normal	19APR2000	-21	Normal	Yes
030.001.00278	IPP	12APR2000	-28	Normal	17APR2000	-23	Normal	Yes
030.001.00285	IPP	18APR2000	-23	Normal	19APR2000	-22	Normal	Yes
030.001.00287	IPP	10APR2000	-31	Normal	17APR2000	-24	Normal	Yes
030.001.00291	IPP	17APR2000	-24	Normal	19APR2000	-22	Normal	Yes
030.001.00297	IPP	18APR2000	-23	Normal	19APR2000	-22	Normal	Yes
030.001.00300	IPP	11APR2000	-31	Normal	18APR2000	-24	Normal	Yes
030.001.00303	IPP	13APR2000	-29	Normal	19APR2000	-23	Normal	Yes

*Popn=Population (I=Intent-to-Treat, PP=Per Protocol)
**Day of test relative to start of prophylactic medication
Tafenoquine = Tafenoquine 200mg, Mefloquine = Mefloquine 250mg

SB252263 - 030

Listing B.10

G6PD Test Results

Treatment : Tafenoquine

Subject Number	Popn*	Date of First Test	Relative Days **	Result	Date of Second Test	Relative Days **	Result	Eligible For Study
030.001.00001	IPP	12APR2000	-26	Normal	18APR2000	-20	Normal	Yes
030.001.00004	IPP	13APR2000	-25	Normal	18APR2000	-20	Normal	Yes
030.001.00006	IPP	10APR2000	-28	Normal	17APR2000	-21	Normal	Yes
030.001.00009	IPP	12APR2000	-26	Normal	18APR2000	-20	Normal	Yes
030.001.00014	IPP	10APR2000	-28	Normal	17APR2000	-21	Normal	Yes
030.001.00016	IPP	10APR2000	-28	Normal	17APR2000	-21	Normal	Yes
030.001.00017	IPP	17APR2000	-21	Normal	19APR2000	-19	Normal	Yes
030.001.00024	IPP	11APR2000	-27	Normal	18APR2000	-20	Normal	Yes
030.001.00025	IPP	11APR2000	-27	Normal	18APR2000	-20	Normal	Yes
030.001.00028	IPP	11APR2000	-27	Normal	17APR2000	-21	Normal	Yes
030.001.00029	IPP	11APR2000	-27	Normal	19APR2000	-19	Normal	Yes
030.001.00030	IPP	14APR2000	-24	Normal	18APR2000	-20	Normal	Yes
030.001.00040	IPP	13APR2000	-25	Normal	19APR2000	-19	Normal	Yes
030.001.00042	IPP	10APR2000	-28	Normal	17APR2000	-21	Normal	Yes
030.001.00043	IPP	18APR2000	-20	Normal	19APR2000	-19	Normal	Yes
030.001.00048	IPP	13APR2000	-25	Normal	17APR2000	-21	Normal	Yes
030.001.00050	IPP	10APR2000	-28	Normal	18APR2000	-20	Normal	Yes
030.001.00051	IPP	11APR2000	-27	Normal	19APR2000	-19	Normal	Yes
030.001.00058	IPP	17APR2000	-21	Normal	19APR2000	-19	Normal	Yes
030.001.00060	IPP	13APR2000	-25	Normal	17APR2000	-21	Normal	Yes
030.001.00061	IPP	14APR2000	-24	Normal	20APR2000	-18	Normal	Yes
030.001.00065	IPP	11APR2000	-27	Normal	18APR2000	-20	Normal	Yes
030.001.00068	IPP	14APR2000	-24	Normal	18APR2000	-20	Normal	Yes
030.001.00070	IPP	11APR2000	-27	Normal	17APR2000	-21	Normal	Yes
030.001.00077	IPP	11APR2000	-27	Normal	18APR2000	-20	Normal	Yes
030.001.00079	IPP	14APR2000	-24	Normal	17APR2000	-21	Normal	Yes
030.001.00084	IPP	13APR2000	-25	Normal	17APR2000	-21	Normal	Yes
030.001.00085	IPP	10APR2000	-28	Normal	18APR2000	-20	Normal	Yes
030.001.00086	IPP	11APR2000	-27	Normal	17APR2000	-21	Normal	Yes
030.001.00093	IPP	10APR2000	-28	Normal	17APR2000	-21	Normal	Yes
030.001.00094	I	11APR2000	-27	Normal	17APR2000	-21	Normal	Yes
030.001.00100	IPP	12APR2000	-26	Normal	17APR2000	-21	Normal	Yes
030.001.00102	IPP	13APR2000	-25	Normal	18APR2000	-20	Normal	Yes
030.001.00104	IPP	14APR2000	-24	Normal	17APR2000	-21	Normal	Yes
030.001.00107	IPP	12APR2000	-26	Normal	18APR2000	-20	Normal	Yes
030.001.00109	IPP	11APR2000	-27	Normal	18APR2000	-20	Normal	Yes
030.001.00111	IPP	11APR2000	-27	Normal	17APR2000	-21	Normal	Yes
030.001.00112	IPP	13APR2000	-25	Normal	17APR2000	-21	Normal	Yes
030.001.00118	IPP	13APR2000	-26	Normal	19APR2000	-20	Normal	Yes
030.001.00122	IPP	12APR2000	-27	Normal	20APR2000	-19	Normal	Yes
030.001.00126	IPP	11APR2000	-28	Normal	18APR2000	-21	Normal	Yes
030.001.00128	IPP	10APR2000	-29	Normal	17APR2000	-22	Normal	Yes
030.001.00129	IPP	12APR2000	-27	Normal	18APR2000	-21	Normal	Yes

*Popn=Population (I=Intent-to-Treat, PP=Per Protocol)
**Day of test relative to start of prophylactic medication
Tafenoquine = Tafenoquine 200mg, Mefloquine = Mefloquine 250mg

SB252263 - 030

Listing B.10

G6PD Test Results

Treatment : Tafenoquine

Subject Number	Popn*	Date of First Test	Relative Days **	Result	Date of Second Test	Relative Days **	Result	Eligible For Study
030.001.00133	IPP	10APR2000	-29	Normal	17APR2000	-22	Normal	Yes
030.001.00135	IPP	11APR2000	-28	Normal	18APR2000	-21	Normal	Yes
030.001.00138	IPP	12APR2000	-27	Normal	18APR2000	-21	Normal	Yes
030.001.00139	IPP	10APR2000	-29	Normal	18APR2000	-21	Normal	Yes
030.001.00148	IPP	10APR2000	-29	Normal	17APR2000	-22	Normal	Yes
030.001.00149	IPP	13APR2000	-26	Normal	17APR2000	-22	Normal	Yes
030.001.00150	IPP	12APR2000	-27	Normal	18APR2000	-21	Normal	Yes
030.001.00155	IPP	11APR2000	-28	Normal	19APR2000	-20	Normal	Yes
030.001.00157	IPP	12APR2000	-27	Normal	17APR2000	-22	Normal	Yes
030.001.00158	IPP	11APR2000	-28	Normal	18APR2000	-21	Normal	Yes
030.001.00159	IPP	12APR2000	-27	Normal	19APR2000	-20	Normal	Yes
030.001.00161	IPP	10APR2000	-29	Normal	17APR2000	-22	Normal	Yes
030.001.00171	IPP	12APR2000	-27	Normal	17APR2000	-22	Normal	Yes
030.001.00173	I	14APR2000	-25	Normal	18APR2000	-21	Normal	Yes
030.001.00176	IPP	13APR2000	-26	Normal	20APR2000	-19	Normal	Yes
030.001.00179	I	12APR2000	-27	Normal	18APR2000	-21	Normal	Yes
030.001.00184	IPP	17APR2000	-22	Normal	19APR2000	-20	Normal	Yes
030.001.00186	IPP	10APR2000	-29	Normal	17APR2000	-22	Normal	Yes
030.001.00187	I	10APR2000	-29	Normal	17APR2000	-22	Normal	Yes
030.001.00190	IPP	11APR2000	-28	Normal	18APR2000	-21	Normal	Yes
030.001.00194	IPP	14APR2000	-25	Normal	19APR2000	-20	Normal	Yes
030.001.00195	IPP	18APR2000	-21	Normal	20APR2000	-19	Normal	Yes
030.001.00201	IPP	10APR2000	-29	Normal	18APR2000	-21	Normal	Yes
030.001.00203	IPP	13APR2000	-26	Normal	17APR2000	-22	Normal	Yes
030.001.00205	IPP	10APR2000	-29	Normal	18APR2000	-21	Normal	Yes
030.001.00207	IPP	13APR2000	-26	Normal	17APR2000	-22	Normal	Yes
030.001.00209	IPP	13APR2000	-26	Normal	19APR2000	-20	Normal	Yes
030.001.00210	IPP	12APR2000	-27	Normal	18APR2000	-21	Normal	Yes
030.001.00217	IPP	11APR2000	-28	Normal	17APR2000	-22	Normal	Yes
030.001.00218	IPP	13APR2000	-26	Normal	18APR2000	-21	Normal	Yes
030.001.00220	IPP	13APR2000	-26	Normal	17APR2000	-22	Normal	Yes
030.001.00228	IPP	13APR2000	-26	Normal	17APR2000	-22	Normal	Yes
030.001.00230	IPP	13APR2000	-26	Normal	18APR2000	-21	Normal	Yes
030.001.00231	IPP	12APR2000	-27	Normal	18APR2000	-21	Normal	Yes
030.001.00233	IPP	10APR2000	-30	Normal	17APR2000	-23	Normal	Yes
030.001.00240	IPP	13APR2000	-27	Normal	18APR2000	-22	Normal	Yes
030.001.00243	IPP	13APR2000	-27	Normal	18APR2000	-22	Normal	Yes
030.001.00246	IPP	11APR2000	-29	Normal	18APR2000	-22	Normal	Yes
030.001.00249	IPP	12APR2000	-28	Normal	17APR2000	-23	Normal	Yes
030.001.00252	IPP	12APR2000	-28	Normal	18APR2000	-22	Normal	Yes
030.001.00254	I	12APR2000	-28	Normal	18APR2000	-22	Normal	Yes
030.001.00259	IPP	12APR2000	-28	Normal	18APR2000	-22	Normal	Yes
030.001.00264	IPP	18APR2000	-22	Normal	19APR2000	-21	Normal	Yes

*Popn=Population (I=Intent-to-Treat, PP=Per Protocol)
**Day of test relative to start of prophylactic medication
Tafenoquine = Tafenoquine 200mg, Mefloquine = Mefloquine 250mg

SB252263 - 030

Listing B.10

G6PD Test Results

Treatment : Tafenoquine

Subject Number	Popn*	Date of First Test	Relative Days **	Result	Date of Second Test	Relative Days **	Result	Eligible For Study
030.001.00266	I	12APR2000	-28	Normal	04MAY2000	-6	Normal	Yes
030.001.00267	IPP	11APR2000	-29	Normal	17APR2000	-23	Normal	Yes
030.001.00271	I	13APR2000	-27	Normal	19APR2000	-21	Normal	Yes
030.001.00272	IPP	10APR2000	-30	Normal	17APR2000	-23	Normal	Yes
030.001.00279	IPP	12APR2000	-29	Normal	18APR2000	-23	Normal	Yes
030.001.00282	IPP	11APR2000	-29	Normal	18APR2000	-22	Normal	Yes
030.001.00284	IPP	10APR2000	-31	Normal	19APR2000	-22	Normal	Yes
030.001.00288	IPP	12APR2000	-29	Normal	18APR2000	-23	Normal	Yes
030.001.00293	IPP	11APR2000	-30	Normal	17APR2000	-24	Normal	Yes
030.001.00294	IPP	13APR2000	-28	Normal	19APR2000	-22	Normal	Yes
030.001.00296	IPP	14APR2000	-27	Normal	18APR2000	-23	Normal	Yes
030.001.00298	IPP	17APR2000	-25	Normal	19APR2000	-23	Normal	Yes
030.001.00301	IPP	14APR2000	-28	Normal	19APR2000	-23	Normal	Yes
030.001.00302	I	14APR2000	-28	Normal	18APR2000	-24	Normal	Yes
030.001.00304	IPP	13APR2000	-29	Normal	18APR2000	-24	Normal	Yes
030.001.00306	IPP	13APR2000	-29	Normal	25APR2000	-17	Normal	Yes

*Popn=Population (I=Intent-to-Treat, PP=Per Protocol)
**Day of test relative to start of prophylactic medication
Tafenoquine = Tafenoquine 200mg, Mefloquine = Mefloquine 250mg

SB252263 - 030

Listing B.10

G6PD Test Results

Treatment : Mefloquine

Subject Number	Popn*	Date of First Test	Relative Days **	Result	Date of Second Test	Relative Days **	Result	Eligible For Study
030.001.00003	I	10APR2000	-28	Normal	17APR2000	-21	Normal	Yes
030.001.00007	IPP	11APR2000	-27	Normal	18APR2000	-20	Normal	Yes
030.001.00008	IPP	11APR2000	-27	Normal	17APR2000	-21	Normal	Yes
030.001.00010	IPP	11APR2000	-27	Normal	17APR2000	-21	Normal	Yes
030.001.00013	IPP	13APR2000	-25	Normal	18APR2000	-20	Normal	Yes
030.001.00018	IPP	11APR2000	-27	Normal	17APR2000	-21	Normal	Yes
030.001.00021	IPP	10APR2000	-28	Normal	17APR2000	-21	Normal	Yes
030.001.00023	I	10APR2000	-28	Normal	17APR2000	-21	Normal	Yes
030.001.00031	IPP	12APR2000	-26	Normal	17APR2000	-21	Normal	Yes
030.001.00034	IPP	17APR2000	-21	Normal	19APR2000	-19	Normal	Yes
030.001.00036	I	13APR2000	-25	Normal	17APR2000	-21	Normal	Yes
030.001.00038	IPP	18APR2000	-20	Normal	19APR2000	-19	Normal	Yes
030.001.00045	IPP	14APR2000	-24	Normal	17APR2000	-21	Normal	Yes
030.001.00046	IPP	10APR2000	-28	Normal	17APR2000	-21	Normal	Yes
030.001.00047	IPP	12APR2000	-26	Normal	17APR2000	-21	Normal	Yes
030.001.00049	IPP	13APR2000	-25	Normal	18APR2000	-20	Normal	Yes
030.001.00052	IPP	10APR2000	-28	Normal	17APR2000	-21	Normal	Yes
030.001.00053	IPP	11APR2000	-27	Normal	17APR2000	-21	Normal	Yes
030.001.00054	IPP	10APR2000	-28	Normal	17APR2000	-21	Normal	Yes
030.001.00062	IPP	12APR2000	-26	Normal	17APR2000	-21	Normal	Yes
030.001.00066	IPP	14APR2000	-24	Normal	19APR2000	-19	Normal	Yes
030.001.00069	I	10APR2000	-28	Normal	17APR2000	-21	Normal	Yes
030.001.00071	IPP	10APR2000	-28	Normal	17APR2000	-21	Normal	Yes
030.001.00073	IPP	13APR2000	-25	Normal	19APR2000	-19	Normal	Yes
030.001.00074	IPP	10APR2000	-28	Normal	17APR2000	-21	Normal	Yes
030.001.00075	IPP	10APR2000	-28	Normal	17APR2000	-21	Normal	Yes
030.001.00083	IPP	13APR2000	-25	Normal	17APR2000	-21	Normal	Yes
030.001.00089	IPP	17APR2000	-21	Normal	20APR2000	-18	Normal	Yes
030.001.00091	IPP	13APR2000	-25	Normal	18APR2000	-20	Normal	Yes
030.001.00095	IPP	14APR2000	-24	Normal	20APR2000	-18	Normal	Yes
030.001.00096	IPP	12APR2000	-26	Normal	17APR2000	-21	Normal	Yes
030.001.00097	IPP	11APR2000	-27	Normal	19APR2000	-19	Normal	Yes
030.001.00098	IPP	12APR2000	-26	Normal	17APR2000	-21	Normal	Yes
030.001.00099	IPP	10APR2000	-28	Normal	18APR2000	-20	Normal	Yes
030.001.00105	IPP	11APR2000	-27	Normal	19APR2000	-19	Normal	Yes
030.001.00110	IPP	10APR2000	-28	Normal	17APR2000	-21	Normal	Yes
030.001.00113	IPP	17APR2000	-21	Normal	20APR2000	-18	Normal	Yes
030.001.00117	IPP	11APR2000	-27	Normal	18APR2000	-20	Normal	Yes
030.001.00120	IPP	10APR2000	-29	Normal	17APR2000	-22	Normal	Yes
030.001.00121	IPP	10APR2000	-29	Normal	17APR2000	-22	Normal	Yes
030.001.00123	I	10APR2000	-29	Normal	17APR2000	-22	Normal	Yes
030.001.00130	IPP	10APR2000	-29	Normal	17APR2000	-22	Normal	Yes
030.001.00132	IPP	13APR2000	-26	Normal	17APR2000	-22	Normal	Yes

*Popn=Population (I=Intent-to-Treat, PP=Per Protocol)
**Day of test relative to start of prophylactic medication
Tafenoquine = Tafenoquine 200mg, Mefloquine = Mefloquine 250mg

SB252263 - 030

Listing B.10

G6PD Test Results

Treatment : Mefloquine

Subject Number	Popn*	Date of First Test	Relative Days **	Result	Date of Second Test	Relative Days **	Result	Eligible For Study
030.001.00134	IPP	11APR2000	-28	Normal	18APR2000	-21	Normal	Yes
030.001.00142	IPP	14APR2000	-25	Normal	17APR2000	-22	Normal	Yes
030.001.00143	IPP	14APR2000	-25	Normal	18APR2000	-21	Normal	Yes
030.001.00144	IPP	11APR2000	-28	Normal	18APR2000	-21	Normal	Yes
030.001.00146	I	13APR2000	-26	Normal	17APR2000	-22	Normal	Yes
030.001.00147	IPP	10APR2000	-29	Normal	17APR2000	-22	Normal	Yes
030.001.00153	IPP	12APR2000	-27	Normal	17APR2000	-22	Normal	Yes
030.001.00156	IPP	14APR2000	-25	Normal	18APR2000	-21	Normal	Yes
030.001.00163	IPP	11APR2000	-28	Normal	18APR2000	-21	Normal	Yes
030.001.00165	IPP	11APR2000	-28	Normal	18APR2000	-21	Normal	Yes
030.001.00167	IPP	13APR2000	-26	Normal	17APR2000	-22	Normal	Yes
030.001.00168	IPP	14APR2000	-25	Normal	19APR2000	-20	Normal	Yes
030.001.00170	IPP	12APR2000	-27	Normal	18APR2000	-21	Normal	Yes
030.001.00177	IPP	13APR2000	-26	Normal	18APR2000	-21	Normal	Yes
030.001.00178	I	12APR2000	-27	Normal	18APR2000	-21	Normal	Yes
030.001.00180	IPP	17APR2000	-22	Normal	19APR2000	-20	Normal	Yes
030.001.00181	IPP	10APR2000	-29	Normal	17APR2000	-22	Normal	Yes
030.001.00185	IPP	18APR2000	-21	Normal	19APR2000	-20	Normal	Yes
030.001.00188	IPP	13APR2000	-26	Normal	19APR2000	-20	Normal	Yes
030.001.00189	I	10APR2000	-29	Normal	17APR2000	-22	Normal	Yes
030.001.00196	IPP	11APR2000	-28	Normal	17APR2000	-22	Normal	Yes
030.001.00198	IPP	12APR2000	-27	Normal	18APR2000	-21	Normal	Yes
030.001.00199	IPP	10APR2000	-29	Normal	17APR2000	-22	Normal	Yes
030.001.00200	IPP	17APR2000	-22	Normal	19APR2000	-20	Normal	Yes
030.001.00206	I	10APR2000	-29	Normal	17APR2000	-22	Normal	Yes
030.001.00213	IPP	14APR2000	-25	Normal	18APR2000	-21	Normal	Yes
030.001.00214	IPP	13APR2000	-26	Normal	18APR2000	-21	Normal	Yes
030.001.00216	IPP	11APR2000	-28	Normal	18APR2000	-21	Normal	Yes
030.001.00221	IPP	17APR2000	-22	Normal	19APR2000	-20	Normal	Yes
030.001.00223	IPP	14APR2000	-25	Normal	18APR2000	-21	Normal	Yes
030.001.00224	IPP	12APR2000	-27	Normal	18APR2000	-21	Normal	Yes
030.001.00227	IPP	14APR2000	-25	Normal	19APR2000	-20	Normal	Yes
030.001.00232	IPP	18APR2000	-22	Normal	19APR2000	-21	Normal	Yes
030.001.00235	IPP	10APR2000	-30	Normal	18APR2000	-22	Normal	Yes
030.001.00237	IPP	17APR2000	-23	Normal	19APR2000	-21	Normal	Yes
030.001.00239	IPP	10APR2000	-30	Normal	18APR2000	-22	Normal	Yes
030.001.00241	IPP	13APR2000	-27	Normal	18APR2000	-22	Normal	Yes
030.001.00244	IPP	13APR2000	-27	Normal	18APR2000	-22	Normal	Yes
030.001.00245	IPP	11APR2000	-29	Normal	18APR2000	-22	Normal	Yes
030.001.00255	IPP	12APR2000	-28	Normal	18APR2000	-22	Normal	Yes
030.001.00258	IPP	14APR2000	-26	Normal	19APR2000	-21	Normal	Yes
030.001.00262	IPP	13APR2000	-27	Normal	18APR2000	-22	Normal	Yes
030.001.00263	IPP	17APR2000	-25	Normal	19APR2000	-23	Normal	Yes

*Popn=Population (I=Intent-to-Treat, PP=Per Protocol)
**Day of test relative to start of prophylactic medication
Tafenoquine = Tafenoquine 200mg, Mefloquine = Mefloquine 250mg

SB252263 - 030

Listing B.10

G6PD Test Results

Treatment : Mefloquine

Subject Number	Popn*	Date of First Test	Relative Days **	Result	Date of Second Test	Relative Days **	Result	Eligible For Study
030.001.00265	IPP	12APR2000	-28	Normal	17APR2000	-23	Normal	Yes
030.001.00273	IPP	11APR2000	-29	Normal	18APR2000	-22	Normal	Yes
030.001.00274	IPP	11APR2000	-29	Normal	17APR2000	-23	Normal	Yes
030.001.00276	IPP	12APR2000	-28	Normal	17APR2000	-23	Normal	Yes
030.001.00280	IPP	13APR2000	-27	Normal	18APR2000	-22	Normal	Yes
030.001.00281	IPP	12APR2000	-28	Normal	18APR2000	-22	Normal	Yes
030.001.00283	IPP	14APR2000	-27	Normal	17APR2000	-24	Normal	Yes
030.001.00286	IPP	12APR2000	-29	Normal	18APR2000	-23	Normal	Yes
030.001.00289	IPP	14APR2000	-27	Normal	19APR2000	-22	Normal	Yes
030.001.00290	IPP	12APR2000	-29	Normal	19APR2000	-22	Normal	Yes
030.001.00292	IPP	12APR2000	-29	Normal	17APR2000	-24	Normal	Yes
030.001.00299	IPP	17APR2000	-25	Normal	20APR2000	-22	Normal	Yes
030.001.00305	IPP	13APR2000	-29	Normal	18APR2000	-24	Normal	Yes

*Popn=Population (I=Intent-to-Treat, PP=Per Protocol)
**Day of test relative to start of prophylactic medication
Tafenoquine = Tafenoquine 200mg, Mefloquine = Mefloquine 250mg

SB252263 - 030

Listing B.11

Significant Medical And Surgical History

Treatment : Placebo

Subject Number	Popn*	WHO Body System	Preferred Term	Diagnosis Text	Year of First Diagnosis	Status
030.001.00005	IPP	GENERAL BODY OR SYS UNSPEC	HEADACHE	HEADACHE	2000	Past
030.001.00011	IPP	DIGESTIVE	ENTERITIS COLITIS	GASTROENTERITIS	2000	Past
		MUSCULOSKELETAL	PAIN, JOINT	JOINT PAIN	2000	Past
030.001.00012	IPP	DIGESTIVE	TEETH DISORD	DENTAL CARIES	2000	Ongoing
		GENERAL BODY OR SYS UNSPEC	HEADACHE	HEADACHE	2000	Past
		METABOLIC NUTRITIONAL IMMUNE	SERUM BILIRUBIN, INCREASED	ABNORMAL BILIRUBIN {HYPERBILIRUBINAEMIA }	2000	Ongoing
030.001.00015	I	RESPIRATORY	UPPER RESP INFECT, ACUTE	UPPER RESPIRATORY TRACT INFECTION	2000	Past
030.001.00019	IPP	DIGESTIVE	CONSTIPATION	CONSTIPATION	2000	Past
		DIGESTIVE	DYSPEPSIA	DYSPEPSIA	2000	Past
		GENERAL BODY OR SYS UNSPEC	ALLERGY, NEC	ALLERGY	2000	Past
		HEMATIC HEMATOPOIETIC LYMPH	ARTHROPOD-BORNE DIS, OTHER	MALARIA	2000	Past
		MUSCULOSKELETAL	MYALGIA	MUSCULOSKELETAL PAIN	2000	Ongoing
		RESPIRATORY	COUGH	COUGH	2000	Past
030.001.00020	IPP	GENERAL BODY OR SYS UNSPEC	BACK PAIN	CHRONIC BACK PAIN	2000	Ongoing
		MUSCULOSKELETAL	PAIN, JOINT	ARTHRALGIAS	2000	Past
		RESPIRATORY	ASTHMA	ASTHMA	1998	Ongoing
		RESPIRATORY	RHINITIS, ALLERGIC	ALLERGIC RHINITIS	1993	Ongoing
030.001.00022	IPP	DIGESTIVE	TEETH DISORD	DENTAL CARIES	2000	Ongoing
		MUSCULOSKELETAL	PAIN, JOINT	ARTHRALGIA	2000	Ongoing

*Popn=Population (I=Intent-to-Treat, PP=Per Protocol)
Tafenoquine = Tafenoquine 200mg, Mefloquine = Mefloquine 250mg

SB252263 - 030

Listing B.11

Significant Medical And Surgical History

Treatment : Placebo

Subject Number	Popn*	WHO Body System	Preferred Term	Diagnosis Text	Year of First Diagnosis	Status
030.001.00026	IPP	GENERAL BODY OR SYS UNSPEC	HEADACHE	PAIN & HEADACHE	2000	Past
030.001.00027	IPP	GENERAL BODY OR SYS UNSPEC	PAIN, ABDOMINO-PELVIC	LEFT UPPER QUADRANT TENDERNESS	2000	Ongoing
030.001.00033	IPP	METABOLIC NUTRITIONAL IMMUNE	MALNUTRITION	MALNUTRITION	2000	Past
		RESPIRATORY	COUGH	COUGH	2000	Past
		RESPIRATORY	RHINITIS, ALLERGIC	ALLERGIC RHINITIS	2000	Ongoing
		RESPIRATORY	UPPER RESP INFECT, ACUTE	UPPER RESPIRATORY TRACT INFECTION	2000	Past
030.001.00037	IPP	DIGESTIVE	CONSTIPATION	CONSTIPATION	2000	Past
		METABOLIC NUTRITIONAL IMMUNE	SERUM BILIRUBIN, INCREASED	HYPERBILIRUBINAEMIA	2000	Ongoing
		METABOLIC NUTRITIONAL IMMUNE	TRANSAMINASE LDH, ELEVATION	ELEVATED SERUM ALT	2000	Past
		MUSCULOSKELETAL	MYALGIA	MUSCULOSKELETAL PAIN	2000	Past
030.001.00039	IPP	INTEGUMENTARY	RASH OTHER SKIN ERUPTION	SKIN RASH	2000	Past
030.001.00041	IPP	METABOLIC NUTRITIONAL IMMUNE	TRANSAMINASE LDH, ELEVATION	ELEVATED ALT	2000	Ongoing
		NERVOUS SENSE ORGANS	CONJUNCTIVAL DISORD	EYES IRRITATION (ALLERGIC CONJUNCTIVITIS)	2000	Ongoing
		RESPIRATORY	ASTHMA	ASTHMA	2000	Ongoing
		RESPIRATORY	RHINITIS, ALLERGIC	ALLERGIC RHINITIS	2000	Ongoing
030.001.00044	IPP	INTEGUMENTARY	MYCOSES	FUNGAL SKIN INFECTION	2000	Ongoing

*Popn=Population (I=Intent-to-Treat, PP=Per Protocol)
Tafenoquine = Tafenoquine 200mg, Mefloquine = Mefloquine 250mg

SB252263 - 030

Listing B.11

Significant Medical And Surgical History

Treatment : Placebo

Subject Number	Popn*	WHO Body System	Preferred Term	Diagnosis Text	Year of First Diagnosis	Status
030.001.00044	IPP	METABOLIC NUTRITIONAL IMMUNE	SERUM BILIRUBIN, INCREASED	ELEVATED BILIRUBIN VALUE	2000	Ongoing
030.001.00056	I	DIGESTIVE	HELMINTHIASIS	ASCARIASIS	2000	Ongoing
		DIGESTIVE	HELMINTHIASIS	HOOKWORM	2000	Ongoing
		DIGESTIVE	INTEST INFECT DIS	AMOEBIASIS	2000	Ongoing
		GENERAL BODY OR SYS UNSPEC	BACK PAIN	BACK PAIN	2000	Ongoing
		GENERAL BODY OR SYS UNSPEC	PAIN, ABDOMINO-PELVIC	ABDOMINAL PAIN	2000	Past
		NERVOUS SENSE ORGANS	VISION ABNORMAL	POOR VISION	2000	Past
030.001.00057	IPP	GENERAL BODY OR SYS UNSPEC	HEADACHE	HEADACHE	2000	Ongoing
		MUSCULOSKELETAL	PAIN, JOINT	ARTHRALGIA	2000	Past
030.001.00059	IPP	DIGESTIVE	CONSTIPATION	CONSTIPATION	2000	Past
		DIGESTIVE	TEETH DISORD	DENTAL CARIES	2000	Ongoing
		GENERAL BODY OR SYS UNSPEC	BACK PAIN	BACKACHE	2000	Ongoing
		GENERAL BODY OR SYS UNSPEC	HEADACHE	HEADACHE	2000	Ongoing
		GENERAL BODY OR SYS UNSPEC	PAIN, ABDOMINO-PELVIC	ABDOMINAL PAIN	2000	Ongoing
		NERVOUS SENSE ORGANS	EYE DISORD, OTHER	EYE PAIN	2000	Ongoing
030.001.00063	IPP	GENERAL BODY OR SYS UNSPEC	ALLERGY, NEC	ALLERGY	2000	Ongoing
		METABOLIC NUTRITIONAL IMMUNE	MALNUTRITION	MALNUTRITION	2000	Ongoing
		MUSCULOSKELETAL	MYALGIA	MYALGIA	2000	Ongoing
		RESPIRATORY	ASTHMA	ASTHMA	2000	Ongoing
		RESPIRATORY	BRONCHITIS, OTHER	COUGH BRONCHITIS	2000	Ongoing

*Popn=Population (I=Intent-to-Treat, PP=Per Protocol)
Tafenoquine = Tafenoquine 200mg, Mefloquine = Mefloquine 250mg

SB252263 - 030

Listing B.11

Significant Medical And Surgical History

Treatment : Placebo

Subject Number	Popn*	WHO Body System	Preferred Term	Diagnosis Text	Year of First Diagnosis	Status
030.001.00067	IPP	DIGESTIVE	DYSPEPSIA	DYSPEPSIA	2000	Ongoing
		DIGESTIVE	INTEST INFECT DIS	AMOEBIASIS	2000	Past
		DIGESTIVE	INTEST INFECT DIS	INTESTINAL INFECTION	2000	Past
		GENERAL BODY OR SYS UNSPEC	PAIN, ABDOMINO-PELVIC	LEFT LOWER QUADRANT ABDOMINAL PAIN	2000	Ongoing
		RESPIRATORY	BRONCHITIS, OTHER	BRONCHITIS	2000	Past
		RESPIRATORY	COUGH	COUGH	2000	Past
		RESPIRATORY	RESP DIS, OTHER	BRONCHIAL SPASM	2000	Past
030.001.00072	IPP	GENERAL BODY OR SYS UNSPEC	CELLULITIS ABSCESS	CELLULITIS	2000	Ongoing
		GENERAL BODY OR SYS UNSPEC	HEADACHE	HEADACHE	2000	Ongoing
		METABOLIC NUTRITIONAL IMMUNE	BLOOD EXAM, ABN	ABNORMAL WBC	2000	Ongoing
030.001.00076	IPP	GENERAL BODY OR SYS UNSPEC	BACK PAIN	BACKACHE	2000	Past
		GENERAL BODY OR SYS UNSPEC	PAIN, ABDOMINO-PELVIC	ABDOMINAL PAIN	2000	Ongoing
		RESPIRATORY	COUGH	COUGH	2000	Past
030.001.00078	IPP	GENERAL BODY OR SYS UNSPEC	VIRAL INFECTION	VIRAL SYNDROME	2000	Ongoing
		RESPIRATORY	NASAL POLYPS	NASAL POLYPS	2000	Ongoing
030.001.00081	IPP	MUSCULOSKELETAL	PAIN, JOINT	JOINT PAIN	2000	Past
030.001.00087	I	GENERAL BODY OR SYS UNSPEC	HEADACHE	HEADACHE	2000	Ongoing
030.001.00088	IPP	GENERAL BODY OR SYS UNSPEC	TRAUMA INJURIES, UNSPEC	SOFT TISSUE INJURY	2000	Ongoing
030.001.00090	IPP	DIGESTIVE	INTEST INFECT DIS	INTESTINAL INFECTION	2000	Ongoing

*Popn=Population (I=Intent-to-Treat, PP=Per Protocol)
Tafenoquine = Tafenoquine 200mg, Mefloquine = Mefloquine 250mg

SB252263 - 030

Listing B.11

Significant Medical And Surgical History

Treatment : Placebo

Subject Number	Popn*	WHO Body System	Preferred Term	Diagnosis Text	Year of First Diagnosis	Status
030.001.00090	IPP	GENERAL BODY OR SYS UNSPEC	HEADACHE	HEADACHE	2000	Ongoing
		GENERAL BODY OR SYS UNSPEC	PAIN, ABDOMINO-PELVIC	RECURRENT ABDOMINAL PAIN	1999	Ongoing
		GENERAL BODY OR SYS UNSPEC	PAIN, ABDOMINO-PELVIC	RIGHT LOWER QUADRANT ABDOMINAL TENDERNESS	1999	Ongoing
		METABOLIC NUTRITIONAL IMMUNE	SERUM BILIRUBIN, INCREASED	HYPERBILIRUBINAEMIA	2000	Ongoing
		RESPIRATORY	COUGH	CHRONIC COUGH	1999	Ongoing
030.001.00092	IPP	DIGESTIVE	DIARRHEA	DIARRHOEA	2000	Past
		GENERAL BODY OR SYS UNSPEC	HEADACHE	HEADACHE	2000	Ongoing
		HEMATIC HEMATOPOIETIC LYMPH	LYMPHOCYTOSIS	LYMPHOCYTOSIS	2000	Ongoing
		MUSCULOSKELETAL	MYALGIA	MUSCULOSKELETAL PAIN	1998	Ongoing
		MUSCULOSKELETAL	RHEUMATIC DISORD	RIGHT ACHILLES TENDONITIS	1998	Ongoing
		NERVOUS SENSE ORGANS	VISION ABNORMAL	POOR VISION	1998	Ongoing
030.001.00101	IPP	DIGESTIVE	ENTERITIS COLITIS	GASTROENTERITIS	2000	Past
		GENERAL BODY OR SYS UNSPEC	HEADACHE	HEADACHE	2000	Past
		GENERAL BODY OR SYS UNSPEC	PAIN, ABDOMINO-PELVIC	ABDOMINAL PAIN	2000	Past
		GENITOURINARY	INFERTILITY, FEMALE	SECONDARY INFERTILITY {FEMALE}	2000	Ongoing
		METABOLIC NUTRITIONAL IMMUNE	SERUM BILIRUBIN, INCREASED	HYPERBILIRUBINEMIA	2000	Ongoing
030.001.00103	IPP	DIGESTIVE	INTEST INFECT DIS	AMOEBIASIS	2000	Past
		METABOLIC NUTRITIONAL IMMUNE	ANOREXIA	ANOREXIA	2000	Past

*Popn=Population (I=Intent-to-Treat, PP=Per Protocol)
Tafenoquine = Tafenoquine 200mg, Mefloquine = Mefloquine 250mg

SB252263 - 030

Listing B.11

Significant Medical And Surgical History

Treatment : Placebo

Subject Number	Popn*	WHO Body System	Preferred Term	Diagnosis Text	Year of First Diagnosis	Status
030.001.00103	IPP	MUSCULOSKELETAL	MYALGIA	MYALGIA	2000	Past
030.001.00106	IPP	GENERAL BODY OR SYS UNSPEC	PAIN, LIMB	ELBOW PAIN	2000	Ongoing
030.001.00108	IPP	GENERAL BODY OR SYS UNSPEC	HEADACHE	HEADACHE	2000	Ongoing
		MUSCULOSKELETAL	MYALGIA	MYALGIAS	2000	Past
		MUSCULOSKELETAL	PAIN, JOINT	ARTHRALGIA	2000	Ongoing
030.001.00114	IPP	GENERAL BODY OR SYS UNSPEC	BACK PAIN	CHRONIC BACK PAIN	1999	Ongoing
		GENERAL BODY OR SYS UNSPEC	PAIN, ABDOMINO-PELVIC	ABDOMINAL PAIN	2000	Past
		HEMATIC HEMATOPOIETIC LYMPH	RBC'S, ABN	MICROCYTOSIS	2000	Ongoing
		MUSCULOSKELETAL	MYALGIA	MUSCULOSKELETAL PAIN	2000	Past
030.001.00115	IPP	GENERAL BODY OR SYS UNSPEC	HEADACHE	HEADACHE	2000	Ongoing
		GENERAL BODY OR SYS UNSPEC	PAIN UNSP, CHEST	CHEST PAIN	2000	Ongoing
		MUSCULOSKELETAL	MYALGIA	MYALGIA	2000	Past
		RESPIRATORY	UPPER RESP INFECT, ACUTE	UPPER RESPIRATORY TRACT INFECTION	2000	Past
030.001.00116	IPP	DIGESTIVE	CONSTIPATION	CONSTIPATION	2000	Past
		GENERAL BODY OR SYS UNSPEC	ALLERGY, NEC	ALLERGY	1999	Ongoing
		INTEGUMENTARY	URTICARIA	URTICARIAL RASHES	1999	Ongoing
		NERVOUS SENSE ORGANS	CONJUNCTIVAL DISORD	CONJUNCTIVITIS	1999	Ongoing
030.001.00119	IPP	GENERAL BODY OR SYS UNSPEC	HEADACHE	PAIN (HEADACHE)	2000	Past

*Popn=Population (I=Intent-to-Treat, PP=Per Protocol)
Tafenoquine = Tafenoquine 200mg, Mefloquine = Mefloquine 250mg

SB252263 - 030

Listing B.11

Significant Medical And Surgical History

Treatment : Placebo

Subject Number	Popn*	WHO Body System	Preferred Term	Diagnosis Text	Year of First Diagnosis	Status
030.001.00124	IPP	METABOLIC NUTRITIONAL IMMUNE	SERUM BILIRUBIN, INCREASED	HYPERBILIRUBINAEMIA	2000	Ongoing
		MUSCULOSKELETAL	MYALGIA	MUSCULOSKELETAL PAIN	2000	Past
030.001.00125	IPP	GENERAL BODY OR SYS UNSPEC	BACK PAIN	CHRONIC LEFT LUMBAR PAIN	1998	Ongoing
		NERVOUS SENSE ORGANS	CONJUNCTIVAL DISORD	ALLERGIC CONJUNCTIVITIS	1995	Ongoing
030.001.00127	I	GENERAL BODY OR SYS UNSPEC	CELLULITIS ABSCESS	CELLULITIS	2000	Past
030.001.00131	IPP	GENERAL BODY OR SYS UNSPEC	BACK PAIN	BACKACHE	2000	Ongoing
030.001.00136	IPP	CARDIOVASCULAR	CARDIAC MURMURS	BENIGN MURMUR	2000	Ongoing
		DIGESTIVE	CONSTIPATION	CONSTIPATION	1992	Past
		DIGESTIVE	HEARTBURN	RECURRENT HEARTBURN	1992	Ongoing
		DIGESTIVE	INTEST INFECT DIS	AMOEBIASIS	2000	Past
		GENERAL BODY OR SYS UNSPEC	HEADACHE	HEADACHE	2000	Ongoing
		HEMATIC HEMATOPOIETIC LYMPH	SPLENOMEGALY	SPLENOMEGALY	2000	Ongoing
		METABOLIC NUTRITIONAL IMMUNE	SERUM BILIRUBIN, INCREASED	HYPERBILIRUBINEMIA	2000	Ongoing
		MUSCULOSKELETAL	PAIN, JOINT	ARTHRALGIAS	2000	Ongoing
		NERVOUS SENSE ORGANS	NEURALGIA, BACK	RIGHT SCIATICA	2000	Ongoing
030.001.00137	IPP	MUSCULOSKELETAL	MYALGIA	MYALGIA	2000	Past
		RESPIRATORY	COUGH	COUGH	2000	Past
030.001.00140	IPP	NERVOUS SENSE ORGANS	CONJUNCTIVAL DISORD	CONJUNCTIVITIS	2000	Past

*Popn=Population (I=Intent-to-Treat, PP=Per Protocol)
Tafenoquine = Tafenoquine 200mg, Mefloquine = Mefloquine 250mg

SB252263 - 030

Listing B.11

Significant Medical And Surgical History

Treatment : Placebo

Subject Number	Popn*	WHO Body System	Preferred Term	Diagnosis Text	Year of First Diagnosis	Status
030.001.00140	IPP	RESPIRATORY	COUGH	COUGH	2000	Past
030.001.00141	IPP	GENERAL BODY OR SYS UNSPEC	HEADACHE	HEADACHE	2000	Past
		MUSCULOSKELETAL	MYALGIA	MUSCULOSKELETAL PAIN	2000	Ongoing
030.001.00145	IPP	DIGESTIVE	TEETH DISORD	DENTAL CARIES	2000	Ongoing
		GENERAL BODY OR SYS UNSPEC	HEADACHE	HEADACHE	2000	Ongoing
		GENERAL BODY OR SYS UNSPEC	PAIN, ABDOMINO-PELVIC	ABDOMINAL PAIN	2000	Ongoing
		METABOLIC NUTRITIONAL IMMUNE	SERUM BILIRUBIN, INCREASED	HYPERBILIRUBINAEMIA	2000	Ongoing
030.001.00151	IPP	DIGESTIVE	INTEST INFECT DIS	INTESTINAL INFECTION	2000	Past
		GENERAL BODY OR SYS UNSPEC	HEADACHE	HEADACHE	2000	Past
		GENERAL BODY OR SYS UNSPEC	PAIN, ABDOMINO-PELVIC	ABDOMINAL PAIN	2000	Past
030.001.00152	IPP	GENERAL BODY OR SYS UNSPEC	PAIN UNSP, CHEST	CHEST PAIN	2000	Past
		GENERAL BODY OR SYS UNSPEC	PAIN, ABDOMINO-PELVIC	ABDOMINAL PAIN	1999	Ongoing
		MUSCULOSKELETAL	MYALGIA	MUSCULOSKELETAL PAIN	2000	Past
		RESPIRATORY	COUGH	COUGH	2000	Past
030.001.00154	IPP	GENERAL BODY OR SYS UNSPEC	PAIN, ABDOMINO-PELVIC	ABDOMINAL PAIN	2000	Ongoing
		GENITOURINARY	NEOPLASMS BENIGN	FIBROID UTERUS	2000	Ongoing
		HEMATIC HEMATOPOIETIC LYMPH	ARTHROPOD-BORNE DIS, OTHER	MALARIA (ACUTE)	2000	Ongoing
		METABOLIC NUTRITIONAL IMMUNE	SERUM BILIRUBIN, INCREASED	HYPERBILIRUBINAEMIA	2000	Ongoing

*Popn=Population (I=Intent-to-Treat, PP=Per Protocol)
Tafenoquine = Tafenoquine 200mg, Mefloquine = Mefloquine 250mg

Listing B.11

Significant Medical And Surgical History

Treatment : Placebo

Subject Number	Popn*	WHO Body System	Preferred Term	Diagnosis Text	Year of First Diagnosis	Status
030.001.00154	IPP	METABOLIC NUTRITIONAL IMMUNE	TRANSAMINASE LDH, ELEVATION	ELEVATED SERUM SGOT ALT	2000	Ongoing
		MUSCULOSKELETAL	OSTEOARTHROSIS	OSTEOARTHRITIS OF THE RIGHT KNEE	2000	Ongoing
030.001.00160	I	DIGESTIVE	MYCOSES	RECURRENT THRUSH	1999	Ongoing
		DIGESTIVE	TEETH DISORD	DENTAL CARIES	2000	Ongoing
		GENERAL BODY OR SYS UNSPEC	ALLERGY, NEC	ALLERGY	2000	Past
		GENERAL BODY OR SYS UNSPEC	CELLULITIS ABSCESS	CELLULITIS	2000	Past
		GENERAL BODY OR SYS UNSPEC	PAIN, ABDOMINO-PELVIC	RECURRENT ABDOMINAL PAIN	1999	Ongoing
		HEMATIC HEMATOPOIETIC LYMPH	ARTHROPOD-BORNE DIS, OTHER	MALARIA	2000	Past
030.001.00162	IPP	DIGESTIVE	GASTRITIS DUODENITIS	PAIN (GASTRITIS)	2000	Past
		DIGESTIVE	INTEST INFECT DIS	AMOEBIASIS	2000	Ongoing
		FACTORS INFLUENCING HEALTH	MANAGEMENT, CONTRACEPTIVE	CONTRACEPTIVE	1997	Ongoing
030.001.00164	IPP	GENERAL BODY OR SYS UNSPEC	PAIN, ABDOMINO-PELVIC	ABDOMINAL PAIN	2000	Past
030.001.00166	IPP	DIGESTIVE	TEETH DISORD	DENTAL CARIES	2000	Ongoing
		GENERAL BODY OR SYS UNSPEC	PAIN, ABDOMINO-PELVIC	ABDOMINAL PAIN	2000	Past
		MUSCULOSKELETAL	PAIN, JOINT	ARTHRALGIA	2000	Past
		RESPIRATORY	TONSILLITIS, ACUTE	TONSILLITIS	2000	Past
030.001.00172	IPP	GENERAL BODY OR SYS UNSPEC	HEADACHE	HEADACHE	1999	Ongoing
		HEMATIC HEMATOPOIETIC LYMPH	SPLENOMEGALY	SPLENOMEGALY	2000	Ongoing

*Popn=Population (I=Intent-to-Treat, PP=Per Protocol)
Tafenoquine = Tafenoquine 200mg, Mefloquine = Mefloquine 250mg

SB252263 - 030

Listing B.11

Significant Medical And Surgical History

Treatment : Placebo

Subject Number	Popn*	WHO Body System	Preferred Term	Diagnosis Text	Year of First Diagnosis	Status
030.001.00172	IPP	METABOLIC NUTRITIONAL IMMUNE	SERUM BILIRUBIN, INCREASED	HYPERBILIRUBINAEMIA	2000	Ongoing
030.001.00174	IPP	GENERAL BODY OR SYS UNSPEC	HEADACHE	HEADACHE	2000	Past
		INTEGUMENTARY	PRURITUS DISORD, UNSPEC	PRURITIS	2000	Past
		MUSCULOSKELETAL	MYALGIA	MYALGIA	2000	Past
030.001.00175	IPP	METABOLIC NUTRITIONAL IMMUNE	SERUM BILIRUBIN, INCREASED	HYPERBILIRUBINAEMIA	2000	Ongoing
		NERVOUS SENSE ORGANS	CATARACT	CATARACT RIGHT EYE	1955	Past
030.001.00182	I	HEMATIC HEMATOPOIETIC LYMPH	ARTHROPOD-BORNE DIS, OTHER	MALARIA	2000	Past
		METABOLIC NUTRITIONAL IMMUNE	SERUM BILIRUBIN, INCREASED	HYPERBILIRUBINAEMIA	2000	Ongoing
		RESPIRATORY	UPPER RESP INFECT, ACUTE	UPPER RESPIRATORY TRACT INFECTION	2000	Past
030.001.00183	IPP	CARDIOVASCULAR	MITRAL VALVE DISORD	MITRAL VALVE REGURGITATION	2000	Ongoing
		HEMATIC HEMATOPOIETIC LYMPH	ARTHROPOD-BORNE DIS, OTHER	MALARIA	2000	Past
030.001.00191	IPP	GENERAL BODY OR SYS UNSPEC	PAIN, ABDOMINO-PELVIC	ABDOMINAL PAIN	2000	Past
		HEMATIC HEMATOPOIETIC LYMPH	ANEMIA, IRON DEFIC	IRON DEFICIENCY-ANAEMIA	2000	Ongoing
		HEMATIC HEMATOPOIETIC LYMPH	ARTHROPOD-BORNE DIS, OTHER	MALARIA	2000	Past

*Popn=Population (I=Intent-to-Treat, PP=Per Protocol)
Tafenoquine = Tafenoquine 200mg, Mefloquine = Mefloquine 250mg

SB252263 - 030

Listing B.11

Significant Medical And Surgical History

Treatment : Placebo

Subject Number	Popn*	WHO Body System	Preferred Term	Diagnosis Text	Year of First Diagnosis	Status
030.001.00191	IPP	RESPIRATORY	TONSILLITIS, ACUTE	TONSILLITIS	2000	Past
030.001.00193	IPP	HEMATIC HEMATOPOIETIC LYMPH	ARTHROPOD-BORNE DIS, OTHER	MALARIA	2000	Past
		MUSCULOSKELETAL	MYALGIA	MUSCULOSKELETAL PAIN	2000	Past
030.001.00197	IPP	GENERAL BODY OR SYS UNSPEC	BACK PAIN	CHRONIC LOW BACK PAIN	1996	Ongoing
		GENERAL BODY OR SYS UNSPEC	PAIN, ABDOMINO-PELVIC	INTERMITTENT ABDOMINAL PAIN	1996	Ongoing
030.001.00204	I	DIGESTIVE	TEETH DISORD	DENTAL CARIES	1999	Ongoing
030.001.00211	IPP	GENERAL BODY OR SYS UNSPEC	BACK PAIN	BACKACHE	2000	Past
		GENERAL BODY OR SYS UNSPEC	HEADACHE	CHRONIC HEADACHE	2000	Ongoing
		MUSCULOSKELETAL	MYALGIA	MYALGIAS	2000	Past
030.001.00215	IPP	GENERAL BODY OR SYS UNSPEC	BACK PAIN	CHRONIC LOW BACK ACHE	2000	Ongoing
		GENERAL BODY OR SYS UNSPEC	HEADACHE	HEADACHE	2000	Past
030.001.00219	IPP	GENERAL BODY OR SYS UNSPEC	BACK PAIN	CHRONIC LOW BACK PAIN	2000	Ongoing
		GENERAL BODY OR SYS UNSPEC	HEADACHE	HEADACHE	2000	Past
		MUSCULOSKELETAL	JOINT DISORD, OTHER	RIGHT KNEE PAIN-BAKER'S CYST	1998	Ongoing
		NERVOUS SENSE ORGANS	DIZZINESS AND GIDDINESS	DIZZINESS	1998	Ongoing
030.001.00226	IPP	GENERAL BODY OR SYS UNSPEC	HEADACHE	RECURRENT HEADACHE	1999	Ongoing

*Popn=Population (I=Intent-to-Treat, PP=Per Protocol)
Tafenoquine = Tafenoquine 200mg, Mefloquine = Mefloquine 250mg

SB252263 - 030

Listing B.11

Significant Medical And Surgical History

Treatment : Placebo

Subject Number	Popn*	WHO Body System	Preferred Term	Diagnosis Text	Year of First Diagnosis	Status
030.001.00226	IPP	GENERAL BODY OR SYS UNSPEC	PAIN, ABDOMINO-PELVIC	ABDOMINAL PAIN	2000	Past
		GENERAL BODY OR SYS UNSPEC	PAIN, RESP	RECURRENT CHEST PAIN MUSCULOSKELETAL	1999	Ongoing
		METABOLIC NUTRITIONAL IMMUNE	SERUM BILIRUBIN, INCREASED	HYPERBILIRUBINAEMIA	2000	Ongoing
		MUSCULOSKELETAL	PAIN, JOINT	DIFFUSE ARTHRALGIA	1999	Ongoing
030.001.00229	IPP	HEMATIC HEMATOPOIETIC LYMPH	ARTHROPOD-BORNE DIS, OTHER	MALARIA	2000	Ongoing
		METABOLIC NUTRITIONAL IMMUNE	SERUM BILIRUBIN, INCREASED	HYPERBILIRUBINAEMIA	2000	Ongoing
		MUSCULOSKELETAL	JOINT DISORD, OTHER	GANGLION RIGHT HAND	1998	Ongoing
		MUSCULOSKELETAL	MYALGIA	MYALGIA	2000	Past
030.001.00234	IPP	GENERAL BODY OR SYS UNSPEC	HEADACHE	HEADACHE	2000	Ongoing
030.001.00236	IPP	DIGESTIVE	INTEST INFECT DIS	AMOEBIASIS	2000	Ongoing
		HEMATIC HEMATOPOIETIC LYMPH	ARTHROPOD-BORNE DIS, OTHER	MALARIA	2000	Ongoing
030.001.00238	IPP	DIGESTIVE	DYSPEPSIA	DYSPEPSIA	2000	Past
		DIGESTIVE	HELMINTHIASIS	ASCARIASIS	2000	Past
		GENERAL BODY OR SYS UNSPEC	ALLERGY, NEC	ALLERGY	2000	Past
		GENERAL BODY OR SYS UNSPEC	HEADACHE	HEADACHE	2000	Ongoing
		GENERAL BODY OR SYS UNSPEC	PAIN, ABDOMINO-PELVIC	ABDOMINAL PAIN	2000	Ongoing
		HEMATIC HEMATOPOIETIC LYMPH	ARTHROPOD-BORNE DIS, OTHER	MALARIA	2000	Past

*Popn=Population (I=Intent-to-Treat, PP=Per Protocol)
Tafenoquine = Tafenoquine 200mg, Mefloquine = Mefloquine 250mg

SB252263 - 030

Listing B.11

Significant Medical And Surgical History

Treatment : Placebo

Subject Number	Popn*	WHO Body System	Preferred Term	Diagnosis Text	Year of First Diagnosis	Status
030.001.00238	IPP	METABOLIC NUTRITIONAL IMMUNE	SERUM BILIRUBIN, INCREASED	HYPERBILIRUBINAEMIA	2000	Ongoing
		MUSCULOSKELETAL	MYALGIA	MYALGIA	2000	Ongoing
030.001.00242	IPP	GENERAL BODY OR SYS UNSPEC	ALLERGY, NEC	ALLERGY	2000	Past
		GENERAL BODY OR SYS UNSPEC	BACK PAIN	BACKACHE	2000	Past
		GENERAL BODY OR SYS UNSPEC	VIRAL DIS EXANTHEM	INFECTIVE HERPES LABIALIS	2000	Past
		HEMATIC HEMATOPOIETIC LYMPH	ARTHROPOD-BORNE DIS, OTHER	MALARIA	2000	Past
		METABOLIC NUTRITIONAL IMMUNE	TRANSAMINASE LDH, ELEVATION	ELEVATED SERUM SGPT	2000	Past
		MUSCULOSKELETAL	MYALGIA	MYALGIA	2000	Past
030.001.00247	IPP	HEMATIC HEMATOPOIETIC LYMPH	ARTHROPOD-BORNE DIS, OTHER	MALARIA	2000	Past
		MUSCULOSKELETAL	MYALGIA	MYALGIA	2000	Past
030.001.00248	IPP	GENERAL BODY OR SYS UNSPEC	HEADACHE	HEADACHE	2000	Past
		METABOLIC NUTRITIONAL IMMUNE	SERUM BILIRUBIN, INCREASED	HYPERBILIRUBINAEMIA	2000	Ongoing
030.001.00251	IPP	GENERAL BODY OR SYS UNSPEC	HEADACHE	HEADACHE	2000	Past
		GENITOURINARY	GENITAL FEMALE DISORD, OTHER	UTERINE PROLAPSE	1999	Ongoing
		INTEGUMENTARY	PRURITUS DISORD, UNSPEC	PRURITIS	2000	Ongoing
		METABOLIC NUTRITIONAL IMMUNE	SERUM BILIRUBIN, INCREASED	HYPERBILIRUBINAEMIA	2000	Ongoing
		MUSCULOSKELETAL	MYALGIA	MYALGIAS	2000	Past

*Popn=Population (I=Intent-to-Treat, PP=Per Protocol)
Tafenoquine = Tafenoquine 200mg, Mefloquine = Mefloquine 250mg

SB252263 - 030

Listing B.11

Significant Medical And Surgical History

Treatment : Placebo

Subject Number	Popn*	WHO Body System	Preferred Term	Diagnosis Text	Year of First Diagnosis	Status
030.001.00256	IPP	DIGESTIVE	DIARRHEA	DIARRHOEA	2000	Past
		GENERAL BODY OR SYS UNSPEC	PAIN, ABDOMINO-PELVIC	ABDOMINAL PAIN	2000	Past
		PSYCHOLOGICAL DISORDERS	ALCOHOLIC DEPEND	ALCOHOLISM	2000	Ongoing
030.001.00257	IPP	DIGESTIVE	DYSPEPSIA	DYSPEPSIA (WITH CERTAIN FOODS ONLY)	1999	Ongoing
		GENERAL BODY OR SYS UNSPEC	VIRAL INFECTION	VIRAL SYNDROME	2000	Past
030.001.00268	IPP	DIGESTIVE	ORAL SOFT TISSUE DIS	DENTAL CELLULITIS	2000	Past
		DIGESTIVE	TEETH DISORD	POOR DENTITION	2000	Ongoing
		INTEGUMENTARY	MYCOSES	TINEA CORPORIS	2000	Past
		METABOLIC NUTRITIONAL IMMUNE	SERUM BILIRUBIN, INCREASED	HYPERBILIRUBINAEMIA	2000	Ongoing
030.001.00269	IPP	HEMATIC HEMATOPOIETIC LYMPH	LEUCOCYTOSIS	LEUKOCYTOSIS	2000	Ongoing
030.001.00275	IPP	CARDIOVASCULAR	HYPERTENSION	HYPERTENSION	2000	Ongoing
		DIGESTIVE	INTEST INFECT DIS	INTESTINAL INFECTION	2000	Ongoing
		GENERAL BODY OR SYS UNSPEC	BACK PAIN	BACK PAIN	1999	Ongoing
		GENERAL BODY OR SYS UNSPEC	HEADACHE	HEADACHE	2000	Past
		GENERAL BODY OR SYS UNSPEC	PAIN UNSP, CHEST	CHEST PAIN	2000	Ongoing
		GENERAL BODY OR SYS UNSPEC	PAIN, ABDOMINO-PELVIC	ABDOMINAL PAIN	2000	Ongoing
		HEMATIC HEMATOPOIETIC LYMPH	ARTHROPOD-BORNE DIS, OTHER	MALARIA	2000	Past
		MUSCULOSKELETAL	PAIN, JOINT	ARTHRALGIAS	1999	Ongoing

*Popn=Population (I=Intent-to-Treat, PP=Per Protocol)
Tafenoquine = Tafenoquine 200mg, Mefloquine = Mefloquine 250mg

SB252263 - 030

Listing B.11

Significant Medical And Surgical History

Treatment : Placebo

Subject Number	Popn*	WHO Body System	Preferred Term	Diagnosis Text	Year of First Diagnosis	Status
030.001.00277	IPP	HEMATIC HEMATOPOIETIC LYMPH	ARTHROPOD-BORNE DIS, OTHER	MALARIA	2000	Past
		METABOLIC NUTRITIONAL IMMUNE	SERUM BILIRUBIN, INCREASED	ABNORMAL BILIRUBIN (HYPERBILIRUBINAEMIA)	2000	Ongoing
		MUSCULOSKELETAL	MYALGIA	MUSCULOSKELETAL PAIN	2000	Past
030.001.00278	IPP	DIGESTIVE	TEETH DISORD	DENTAL CARIES	2000	Ongoing
		HEMATIC HEMATOPOIETIC LYMPH	ARTHROPOD-BORNE DIS, OTHER	MALARIA	2000	Past
		INTEGUMENTARY	MYCOSES	FUNGAL SKIN CONDITION {NOS}	2000	Past
		METABOLIC NUTRITIONAL IMMUNE	SERUM BILIRUBIN, INCREASED	HYPERBILIRUBINAEMIA	2000	Ongoing
030.001.00287	IPP	GENERAL BODY OR SYS UNSPEC	HEADACHE	HEADACHE	2000	Past
		HEMATIC HEMATOPOIETIC LYMPH	ARTHROPOD-BORNE DIS, OTHER	MALARIA	2000	Past
		METABOLIC NUTRITIONAL IMMUNE	SERUM BILIRUBIN, INCREASED	HYPERBILIRUBINAEMIA	2000	Ongoing
		METABOLIC NUTRITIONAL IMMUNE	TRANSAMINASE LDH, ELEVATION	INCREASED ALT	2000	Ongoing
		MUSCULOSKELETAL	ARTHROPATHY	BILATERAL SHOULDER ARTHRITIS	1999	Ongoing
		MUSCULOSKELETAL	MYALGIA	MYALGIA	2000	Past
		MUSCULOSKELETAL	SPRAINS STRAINS	SPRAIN RIGHT HIP JOINT	2000	Past
030.001.00291	IPP	CARDIOVASCULAR	VARICES, LOWER EXTREMITIES	CHRONIC STASIS ULCER LEFT LEG	1999	Ongoing
		DIGESTIVE	MYCOSES	ORAL THRUSH	1999	Ongoing

*Popn=Population (I=Intent-to-Treat, PP=Per Protocol)
Tafenoquine = Tafenoquine 200mg, Mefloquine = Mefloquine 250mg

SB252263 - 030

Listing B.11

Significant Medical And Surgical History

Treatment : Placebo

Subject Number	Popn*	WHO Body System	Preferred Term	Diagnosis Text	Year of First Diagnosis	Status
030.001.00295		DIGESTIVE	TEETH DISORD	DENTAL CARIES	2000	Ongoing
		GENERAL BODY OR SYS UNSPEC	BACK PAIN	CHRONIC BACK PAIN	2000	Ongoing
		HEMATIC HEMATOPOIETIC LYMPH	THROMBOCYTOPENIA	THROMBOCYTOPENIA	2000	Ongoing
		METABOLIC NUTRITIONAL IMMUNE	SERUM BILIRUBIN, INCREASED	HYPERBILIRUBINAEMIA	2000	Ongoing
030.001.00297	IPP	HEMATIC HEMATOPOIETIC LYMPH	ARTHROPOD-BORNE DIS, OTHER	MALARIA	2000	Past
		MUSCULOSKELETAL	PAIN, JOINT	ARTHRALGIA LEFT KNEE	2000	Past
		RESPIRATORY	RHINITIS, ALLERGIC	SEASONAL ALLERGIES	2000	Past
030.001.00300	IPP	GENERAL BODY OR SYS UNSPEC	HEADACHE	HEADACHE	2000	Ongoing
		GENITOURINARY	URINARY TRACT INFECTION	URINARY TRACT INFECTION	2000	Ongoing
030.001.00303	IPP	GENERAL BODY OR SYS UNSPEC	PAIN, ABDOMINO-PELVIC	ABDOMINAL PAIN	2000	Past
		GENITOURINARY	URINARY TRACT INFECTION	URINARY TRACT INFECTION	2000	Ongoing
		HEMATIC HEMATOPOIETIC LYMPH	ARTHROPOD-BORNE DIS, OTHER	MALARIA	2000	Past
		HEMATIC HEMATOPOIETIC LYMPH	RBC'S, ABN	MICROCYTOSIS	2000	Ongoing
		RESPIRATORY	NASOPHARYNGITIS, ACUTE	ACUTE RHINITIS	2000	Past

*Popn=Population (I=Intent-to-Treat, PP=Per Protocol)
Tafenoquine = Tafenoquine 200mg, Mefloquine = Mefloquine 250mg

SB252263 - 030

Listing B.11

Significant Medical And Surgical History

Treatment : Tafenoquine

Subject Number	Popn*	WHO Body System	Preferred Term	Diagnosis Text	Year of First Diagnosis	Status
030.001.00001	IPP	GENERAL BODY OR SYS UNSPEC	HEADACHE	HEADACHE	2000	Ongoing
		GENERAL BODY OR SYS UNSPEC	MALAISE AND FATIGUE	MALAISE	2000	Ongoing
		GENITOURINARY	AMENORRHEA	AMENORRHEA	2000	Ongoing
		METABOLIC NUTRITIONAL IMMUNE	TRANSAMINASE LDH, ELEVATION	ABNORMAL ALT {ELEVATED}	2000	Ongoing
		MUSCULOSKELETAL	PAIN, JOINT	ARTHRALGIAS	2000	Ongoing
030.001.00004	IPP	GENERAL BODY OR SYS UNSPEC	HEADACHE	HEADACHE	2000	Past
		HEMATIC HEMATOPOIETIC LYMPH	ANEMIA, IRON DEFIC	MICROCYTIC ANAEMIA	2000	Ongoing
		INTEGUMENTARY	MYCOSES	TINEA CORPORIS	1998	Ongoing
		MUSCULOSKELETAL	MYALGIA	MUSCULOSKELETAL PAIN	2000	Past
		RESPIRATORY	RHINITIS, ALLERGIC	ALLERGIC RHINITIS	2000	Ongoing
		RESPIRATORY	UPPER RESP DIS, OTHER	HYPERTROPHIED NASAL TURBINATES	2000	Ongoing
030.001.00009	IPP	METABOLIC NUTRITIONAL IMMUNE	SERUM BILIRUBIN, INCREASED	ABNORMAL BILIRUBIN {ELEVATED}	2000	Ongoing
030.001.00014	IPP	METABOLIC NUTRITIONAL IMMUNE	TRANSAMINASE LDH, ELEVATION	ABNORMAL ALT {ELEVATED}	2000	Ongoing
030.001.00016	IPP	GENERAL BODY OR SYS UNSPEC	PAIN, ABDOMINO-PELVIC	RECURRENT ABDOMINAL PAIN	2000	Ongoing
030.001.00017	IPP	DIGESTIVE	TEETH DISORD	DENTAL CARIES	2000	Ongoing
		RESPIRATORY	TONSILLITIS, ACUTE	TONSILLITIS	2000	Past
030.001.00024	IPP	DIGESTIVE	ENTERITIS COLITIS	GASTRO ENTERITIS	2000	Past

*Popn=Population (I=Intent-to-Treat, PP=Per Protocol)
Tafenoquine = Tafenoquine 200mg, Mefloquine = Mefloquine 250mg

SB252263 - 030

Listing B.11

Significant Medical And Surgical History

Treatment : Tafenoquine

Subject Number	Popn*	WHO Body System	Preferred Term	Diagnosis Text	Year of First Diagnosis	Status
030.001.00024	IPP	GENERAL BODY OR SYS UNSPEC	HEADACHE	HEADACHE	2000	Ongoing
		GENERAL BODY OR SYS UNSPEC	PAIN UNSP, CHEST	CHEST PAIN	2000	Ongoing
		HEMATIC HEMATOPOIETIC LYMPH	SPLENOMEGALY	SPLENOMEGALY	2000	Ongoing
		METABOLIC NUTRITIONAL IMMUNE	ANOREXIA	ANOREXIA	2000	Past
		MUSCULOSKELETAL	MYALGIA	MYALGIA	2000	Past
		NERVOUS SENSE ORGANS	VISUAL DISTURB	MYOPIA	2000	Ongoing
030.001.00025	IPP	DIGESTIVE	DYSPEPSIA	DYSPEPSIA	2000	Ongoing
		NERVOUS SENSE ORGANS	EYE DISORD, OTHER	EYE DISCOMFORT	1999	Ongoing
030.001.00028	IPP	DIGESTIVE	DYSPEPSIA	DYSPEPSIA	2000	Past
		DIGESTIVE	TEETH DISORD	DENTAL CARIES	2000	Ongoing
		GENERAL BODY OR SYS UNSPEC	HEADACHE	HEADACHE	2000	Ongoing
		GENERAL BODY OR SYS UNSPEC	PAIN, ABDOMINO-PELVIC	STOMACH PAIN	2000	Past
		INTEGUMENTARY	INFLAM SKIN SUBCUT	DANDRUFF	1999	Ongoing
030.001.00029	IPP	INTEGUMENTARY	MYCOSES	TINEA CAPITIS	2000	Ongoing
030.001.00030	IPP	DIGESTIVE	DYSPEPSIA	DYSPEPSIA	2000	Past
		GENERAL BODY OR SYS UNSPEC	BACK PAIN	INTERMITTENT LOW BACK PAIN	1999	Ongoing
		METABOLIC NUTRITIONAL IMMUNE	TRANSAMINASE LDH, ELEVATION	ABNORMAL ALT VALUE {INCREASED}	2000	Ongoing
		MUSCULOSKELETAL	MYALGIA	MUSCULOSKELETAL PAIN	2000	Ongoing
		NERVOUS SENSE ORGANS	VISION ABNORMAL	DEFECTIVE DISTANT VISION	2000	Ongoing

*Popn=Population (I=Intent-to-Treat, PP=Per Protocol)
Tafenoquine = Tafenoquine 200mg, Mefloquine = Mefloquine 250mg

SB252263 - 030

Listing B.11

Significant Medical And Surgical History

Treatment : Tafenoquine

Subject Number	Popn*	WHO Body System	Preferred Term	Diagnosis Text	Year of First Diagnosis	Status
030.001.00040	IPP	GENERAL BODY OR SYS UNSPEC	HEADACHE	FRONTAL HEADACHE	1999	Ongoing
030.001.00042	IPP	DIGESTIVE	ENTERITIS COLITIS	ENTERITIS	2000	Past
		HEMATIC HEMATOPOIETIC LYMPH	LEUCOCYTOSIS	ABNORMAL WHITE BLOOD CELLS {ELEVATED}	2000	Ongoing
		INTEGUMENTARY	MYCOSES	TINEA CORPORIS AT COLLAR LINE	2000	Ongoing
		MUSCULOSKELETAL	ARTHROPATHY	PAIN IN RIGHT KNEE (POST ARTHRITIS)	1991	Ongoing
030.001.00043	IPP	INTEGUMENTARY	MYCOSES	FUNGAL SKIN INFECTION	2000	Past
		RESPIRATORY	UPPER RESP INFECT, ACUTE	URI	2000	Ongoing
030.001.00048	IPP	HEMATIC HEMATOPOIETIC LYMPH	ANEMIA, OTHER	LOW HAEMATOCRIT	2000	Ongoing
		HEMATIC HEMATOPOIETIC LYMPH	RBC'S, ABN	MICROCYTOSIS	2000	Ongoing
		HEMATIC HEMATOPOIETIC LYMPH	SPLENOMEGALY	SPLENOMEGALY	2000	Ongoing
030.001.00051	IPP	CARDIOVASCULAR	VARICES	VARICOSE VEINS	2000	Ongoing
		GENERAL BODY OR SYS UNSPEC	PAIN UNSP, CHEST	CHRONIC CHEST PAIN	1992	Ongoing
		GENERAL BODY OR SYS UNSPEC	TRAUMA INJURIES, UNSPEC	CHEST TRAUMA	1992	Past
		RESPIRATORY	COUGH	COUGH	2000	Past
		RESPIRATORY	UPPER RESP INFECT, ACUTE	UPPER RESPIRATORY TRACT INFECTION	2000	Past
030.001.00058	IPP	DIGESTIVE	INTEST INFECT DIS	AMOEBIASIS	2000	Past

*Popn=Population (I=Intent-to-Treat, PP=Per Protocol)
Tafenoquine = Tafenoquine 200mg, Mefloquine = Mefloquine 250mg

SB252263 - 030

Listing B.11

Significant Medical And Surgical History

Treatment : Tafenoquine

Subject Number	Popn*	WHO Body System	Preferred Term	Diagnosis Text	Year of First Diagnosis	Status
030.001.00058	IPP	GENERAL BODY OR SYS UNSPEC	BACK PAIN	CHRONIC BACKACHE	2000	Ongoing
030.001.00060	IPP	DIGESTIVE	DYSPEPSIA	DYSPEPSIA	2000	Past
		HEMATIC HEMATOPOIETIC LYMPH	LEUCOCYTOSIS	LEUCOCYTOSIS	2000	Past
		MUSCULOSKELETAL	DEFORMITY, ACQUIRED	LEG DEFORMITY SECONDARY TO TRAUMA	2000	Ongoing
		RESPIRATORY	COUGH	COUGH	2000	Ongoing
030.001.00065	IPP	GENITOURINARY	URINARY TRACT INFECTION	URINARY TRACT INFECTION	2000	Past
030.001.00068	IPP	NERVOUS SENSE ORGANS	HEARING LOSS	DEAF	1981	Ongoing
		RESPIRATORY	BRONCHITIS, OTHER	BRONCHITIS	2000	Past
030.001.00070	IPP	GENERAL BODY OR SYS UNSPEC	CELLULITIS ABSCESS	LEFT THIGH CELLULITIS	2000	Ongoing
		MUSCULOSKELETAL	PAIN, JOINT	ARTHRALGIA	2000	Past
		RESPIRATORY	COUGH	COUGH	2000	Past
030.001.00077	IPP	RESPIRATORY	BRONCHITIS, OTHER	BRONCHITIS	2000	Past
		RESPIRATORY	COUGH	COUGH	2000	Past
		RESPIRATORY	RESP DIS, OTHER	BRONCHIAL SPASM	2000	Past
030.001.00079	IPP	INTEGUMENTARY	MYCOSES	TINEA CORPORIS	1999	Ongoing
		METABOLIC NUTRITIONAL IMMUNE	BLOOD EXAM, ABN	ABNORMAL PLATELETS	2000	Ongoing
		RESPIRATORY	UPPER RESP INFECT, ACUTE	CONGESTION {UPPER RESPIRATORY TRACT INFECTION}	2000	Past
030.001.00084	IPP	CARDIOVASCULAR	HEMORRHOIDS	HAEMORRHOIDS	2000	Ongoing

*Popn=Population (I=Intent-to-Treat, PP=Per Protocol)
Tafenoquine = Tafenoquine 200mg, Mefloquine = Mefloquine 250mg

SB252263 - 030

Listing B.11

Significant Medical And Surgical History

Treatment : Tafenoquine

Subject Number	Popn*	WHO Body System	Preferred Term	Diagnosis Text	Year of First Diagnosis	Status
030.001.00084	IPP	GENERAL BODY OR SYS UNSPEC	BACK PAIN	RECURRENT BACK PAIN	2000	Ongoing
		GENERAL BODY OR SYS UNSPEC	PAIN, ABDOMINO-PELVIC	ABDOMINAL PAIN	2000	Ongoing
		INTEGUMENTARY	PRURITUS DISORD, UNSPEC	PRURITUS	2000	Ongoing
030.001.00085	IPP	GENERAL BODY OR SYS UNSPEC	BACK PAIN	BACKACHE	2000	Past
		GENERAL BODY OR SYS UNSPEC	HEADACHE	HEADACHE	2000	Ongoing
		GENERAL BODY OR SYS UNSPEC	PAIN UNSP, CHEST	CHEST PAIN	2000	Ongoing
		METABOLIC NUTRITIONAL IMMUNE	VITAMIN DEFIC, OTHER	AVITAMINOSIS	2000	Ongoing
		MUSCULOSKELETAL	PAIN, JOINT	JOINT PAIN	2000	Ongoing
030.001.00093	IPP	DIGESTIVE	ENTERITIS COLITIS	ENTERITIS	2000	Past
		DIGESTIVE	INTEST INFECT DIS	AMOEBIASIS	2000	Past
		DIGESTIVE	TEETH DISORD	DENTAL CARIES	2000	Ongoing
		GENERAL BODY OR SYS UNSPEC	HEADACHE	HEADACHE	2000	Past
		HEMATIC HEMATOPOIETIC LYMPH	ANEMIA, HEMOLYT, HERED	ABNORMAL HAEMOGLOBIN	2000	Ongoing
		MUSCULOSKELETAL	MYALGIA	MUSCULOSKELETAL PAIN	2000	Past
030.001.00094	I	GENITOURINARY	VENEREAL DIS	NONGONOCOCCAL URETHRITIS	2000	Ongoing
		MUSCULOSKELETAL	MYALGIA	MUSCULOSKELETAL PAIN	2000	Ongoing
030.001.00100	IPP	DIGESTIVE	TEETH DISORD	DENTAL CARIES	2000	Ongoing
		GENERAL BODY OR SYS UNSPEC	HEADACHE	HEADACHE	2000	Past
		INTEGUMENTARY	PRURITUS DISORD, UNSPEC	PRURITUS CHRONIC	2000	Ongoing
030.001.00102	IPP	CARDIOVASCULAR	HEMORRHOIDS	HAEMORRHOIDS	2000	Ongoing

*Popn=Population (I=Intent-to-Treat, PP=Per Protocol)
Tafenoquine = Tafenoquine 200mg, Mefloquine = Mefloquine 250mg

SB252263 - 030

Listing B.11

Significant Medical And Surgical History

Treatment : Tafenoquine

Subject Number	Popn*	WHO Body System	Preferred Term	Diagnosis Text	Year of First Diagnosis	Status
030.001.00102	IPP	DIGESTIVE	ENTERITIS COLITIS	GASTROENTERITIS	2000	Past
		METABOLIC NUTRITIONAL IMMUNE	SERUM BILIRUBIN, INCREASED	HYPERBILIRUBINAEMIA	2000	Ongoing
		MUSCULOSKELETAL	MYALGIA	MYOSITIS	2000	Past
030.001.00104	IPP	DIGESTIVE	GASTRITIS DUODENITIS	GASTRITIS	2000	Ongoing
		DIGESTIVE	ULCER, DUODENAL	CHRONIC DUODENAL ULCER	2000	Ongoing
		GENERAL BODY OR SYS UNSPEC	HEADACHE	HEADACHE RECURRENT	2000	Ongoing
		HEMATIC HEMATOPOIETIC LYMPH	THROMBOCYTOPENIA	THROMBOCYTOPENIA	2000	Ongoing
		METABOLIC NUTRITIONAL IMMUNE	SERUM BILIRUBIN, INCREASED	HYPERBILIRUBINEMIA	2000	Ongoing
030.001.00107	IPP	GENERAL BODY OR SYS UNSPEC	HEADACHE	HEADACHE	2000	Past
		HEMATIC HEMATOPOIETIC LYMPH	RBC'S, ABN	MICROCYTOSIS	2000	Ongoing
		INTEGUMENTARY	SKIN SUBCUT DISORD, OTHER	HYPERKERATOSIS LOWER LEGS	2000	Ongoing
		MUSCULOSKELETAL	PAIN, JOINT	JOINT PAIN	2000	Past
		RESPIRATORY	RHINITIS, ALLERGIC	ALLERGIC RHINITIS	1997	Ongoing
030.001.00109	IPP	DIGESTIVE	ENTERITIS COLITIS	GASTROENTERITIS	2000	Past
		GENERAL BODY OR SYS UNSPEC	MALAISE AND FATIGUE	MALAISE	2000	Past
		GENERAL BODY OR SYS UNSPEC	PAIN, ABDOMINO-PELVIC	EPIGASTRIC PAIN	2000	Ongoing
		INTEGUMENTARY	PRURITUS DISORD, UNSPEC	PRURITIS	2000	Past
		MUSCULOSKELETAL	PAIN, JOINT	ARTHRALGIA	2000	Past

*Popn=Population (I=Intent-to-Treat, PP=Per Protocol)
Tafenoquine = Tafenoquine 200mg, Mefloquine = Mefloquine 250mg

SB252263 - 030

Listing B.11

Significant Medical And Surgical History

Treatment : Tafenoquine

Subject Number	Popn*	WHO Body System	Preferred Term	Diagnosis Text	Year of First Diagnosis	Status
030.001.00111	IPP	NERVOUS SENSE ORGANS	CONJUNCTIVAL DISORD	CONJUNCTIVITIS	2000	Past
030.001.00112	IPP	GENERAL BODY OR SYS UNSPEC	MALAISE AND FATIGUE	MALAISE	2000	Ongoing
030.001.00118	IPP	NERVOUS SENSE ORGANS	CONJUNCTIVAL DISORD	CONJUNCTIVITIS	2000	Ongoing
030.001.00122	IPP	HEMATIC HEMATOPOIETIC LYMPH	THROMBOCYTOPENIA	THROMBOCYTOPENIA	2000	Ongoing
030.001.00126	IPP	GENITOURINARY	CYSTITIS	CYSTITIS	2000	Past
		NERVOUS SENSE ORGANS	NEURALGIA, BACK	LEFT LEG SCIATICA	2000	Past
030.001.00129	IPP	GENERAL BODY OR SYS UNSPEC	BACK PAIN	BACKACHE	2000	Ongoing
		MUSCULOSKELETAL	PAIN, JOINT	DIFFUSE ARTHRALGIAS	2000	Ongoing
030.001.00133	IPP	METABOLIC NUTRITIONAL IMMUNE	SERUM BILIRUBIN, INCREASED	ABNORMAL BILIRUBIN {INCREASE}	2000	Ongoing
		RESPIRATORY	RHINITIS, ALLERGIC	RHINITIS (ALLERGIC)	2000	Ongoing
		RESPIRATORY	TONSILLITIS, ACUTE	FOLLICULAR TONSILLITIS	2000	Ongoing
030.001.00135	IPP	GENERAL BODY OR SYS UNSPEC	HEADACHE	HEADACHE	2000	Ongoing
		MUSCULOSKELETAL	PAIN, JOINT	ARTHRALGIA	2000	Past
030.001.00138	IPP	GENERAL BODY OR SYS UNSPEC	CELLULITIS ABSCESS	PAIN (SWOLLEN LEG CELLULITIS)	2000	Past

*Popn=Population (I=Intent-to-Treat, PP=Per Protocol)
Tafenoquine = Tafenoquine 200mg, Mefloquine = Mefloquine 250mg

SB252263 - 030

Listing B.11

Significant Medical And Surgical History

Treatment : Tafenoquine

Subject Number	Popn*	WHO Body System	Preferred Term	Diagnosis Text	Year of First Diagnosis	Status
030.001.00138	IPP	GENERAL BODY OR SYS UNSPEC	CELLULITIS ABSCESS	PAIN CELLULITIS	2000	Past
		MUSCULOSKELETAL	PAIN, JOINT	ARTHRALGIAS	2000	Past
030.001.00148	IPP	GENERAL BODY OR SYS UNSPEC	VENEREAL DIS	SYPHILIS	1999	Past
		HEMATIC HEMATOPOIETIC LYMPH	LEUCOCYTOSIS	LEUCOCYTOSIS	2000	Ongoing
		HEMATIC HEMATOPOIETIC LYMPH	POLYCYTHEMIA	POLYCYTHEMIA	2000	Ongoing
		NERVOUS SENSE ORGANS	CONJUNCTIVAL DISORD	ALLERGIC CONJUNCTIVITIS	2000	Ongoing
030.001.00149	IPP	GENITOURINARY	URETHRITIS	URETHRITIS	2000	Ongoing
		INTEGUMENTARY	VIRUS CHLAMYD DIS, OTHER	GENITAL WARTS	2000	Ongoing
		MUSCULOSKELETAL	PAIN, JOINT	ARTHRALGIA	2000	Ongoing
030.001.00150	IPP	GENERAL BODY OR SYS UNSPEC	HEADACHE	HEADACHE	2000	Past
		INTEGUMENTARY	RASH OTHER SKIN ERUPTION	RASH	2000	Ongoing
		MUSCULOSKELETAL	MYALGIA	MYALGIAS	2000	Ongoing
		MUSCULOSKELETAL	PAIN, JOINT	ARTHRALGIAS	2000	Ongoing
		NERVOUS SENSE ORGANS	CONJUNCTIVAL DISORD	CONJUNCTIVITIS	2000	Past
030.001.00155	IPP	DIGESTIVE	HEARTBURN	HEARTBURN	2000	Ongoing
		MUSCULOSKELETAL	PAIN, JOINT	CHRONIC ARTHRALGIAS (SHOULDER,BACK,KNEES)	1999	Ongoing
		NERVOUS SENSE ORGANS	VISUAL DISTURB	DIFFICULTY WITH NEAR VISION {MYOPIA}	2000	Ongoing
		RESPIRATORY	COUGH	COUGH	2000	Ongoing
		RESPIRATORY	UPPER RESP INFECT, ACUTE	UPPER RESPIRATORY TRACT INFECTION	2000	Past

*Popn=Population (I=Intent-to-Treat, PP=Per Protocol)
Tafenoquine = Tafenoquine 200mg, Mefloquine = Mefloquine 250mg

SB252263 - 030

Listing B.11

Significant Medical And Surgical History

Treatment : Tafenoquine

Subject Number	Popn*	WHO Body System	Preferred Term	Diagnosis Text	Year of First Diagnosis	Status
030.001.00157	IPP	GENERAL BODY OR SYS UNSPEC	HEADACHE	HEADACHE	2000	Ongoing
		RESPIRATORY	ASTHMA	ASTHMA	2000	Ongoing
		RESPIRATORY	PHARYNGITIS, ACUTE	SORE THROAT	2000	Ongoing
030.001.00158	IPP	GENITOURINARY	AMENORRHEA	AMENORRHOEA	2000	Ongoing
		HEMATIC HEMATOPOIETIC LYMPH	ARTHROPOD-BORNE DIS, OTHER	MALARIA	2000	Past
030.001.00159	IPP	HEMATIC HEMATOPOIETIC LYMPH	ARTHROPOD-BORNE DIS, OTHER	MALARIA	2000	Past
		MUSCULOSKELETAL	MYALGIA	MYALGIA	2000	Past
030.001.00161	IPP	DIGESTIVE	DYSPEPSIA	DYSPEPSIA	2000	Ongoing
		INTEGUMENTARY	RASH OTHER SKIN ERUPTION	ALLERGIC PAPULAR RASH	2000	Past
		METABOLIC NUTRITIONAL IMMUNE	WEIGHT LOSS	WEIGHT LOSS	2000	Ongoing
		NERVOUS SENSE ORGANS	CONJUNCTIVAL DISORD	ALLERGIC CONJUNCTIVITIS	2000	Past
030.001.00171	IPP	GENERAL BODY OR SYS UNSPEC	HEADACHE	HEADACHE	2000	Ongoing
		GENERAL BODY OR SYS UNSPEC	HEADACHE	POST TRAUMATIC HEADACHES	1995	Ongoing
		GENERAL BODY OR SYS UNSPEC	PAIN, ABDOMINO-PELVIC	ABDOMINAL PAIN	2000	Ongoing
		HEMATIC HEMATOPOIETIC LYMPH	ARTHROPOD-BORNE DIS, OTHER	MALARIA	2000	Past
		MUSCULOSKELETAL	MYALGIA	MYALGIA	2000	Past
		MUSCULOSKELETAL	RHEUMATIC DISORD	LEFT SHOULDER ADHESIVE CAPSULITIS	2000	Ongoing

*Popn=Population (I=Intent-to-Treat, PP=Per Protocol)
Tafenoquine = Tafenoquine 200mg, Mefloquine = Mefloquine 250mg

SB252263 - 030

Listing B.11

Significant Medical And Surgical History

Treatment : Tafenoquine

Subject Number	Popn*	WHO Body System	Preferred Term	Diagnosis Text	Year of First Diagnosis	Status
030.001.00173	I	METABOLIC NUTRITIONAL IMMUNE	ANOREXIA	ANOREXIA	2000	Past
030.001.00176	IPP	GENERAL BODY OR SYS UNSPEC	CELLULITIS ABSCESS	CELLULITIS	2000	Past
		HEMATIC HEMATOPOIETIC LYMPH	LEUCOCYTOSIS	LEUCOCYTOSIS	2000	Ongoing
030.001.00179	I	RESPIRATORY	UPPER RESP INFECT, ACUTE	UPPER RESPIRATORY TRACT INFECTION	2000	Past
030.001.00184	IPP	METABOLIC NUTRITIONAL IMMUNE	SERUM BILIRUBIN, INCREASED	ABNORMAL BILIRUBIN VALUE (HYPERBILIRUBINAEMIA)	2000	Ongoing
030.001.00187	I	DIGESTIVE	TEETH DISORD	DENTAL CARIES	2000	Ongoing
		NERVOUS SENSE ORGANS	EYELID DISORD	PTOSIS LEFT EYE	1999	Past
030.001.00190	IPP	GENERAL BODY OR SYS UNSPEC	PAIN, ABDOMINO-PELVIC	RECURRENT ABDOMINAL PAIN	2000	Ongoing
		GENITOURINARY	DYSURIA	DYSURIA	1999	Ongoing
		METABOLIC NUTRITIONAL IMMUNE	SERUM BILIRUBIN, INCREASED	HYPERBILIRUBINAEMIA	2000	Ongoing
030.001.00194	IPP	DIGESTIVE	INTEST INFECT DIS	AMOEBIASIS	2000	Past
		GENERAL BODY OR SYS UNSPEC	PAIN, ABDOMINO-PELVIC	RECURRENT ABDOMINAL PAIN	2000	Ongoing
		METABOLIC NUTRITIONAL IMMUNE	SERUM BILIRUBIN, INCREASED	HYPERBILIRUBINAEMIA	2000	Ongoing
		MUSCULOSKELETAL	MYALGIA	MUSCULOSKELETAL PAIN	2000	Past

*Popn=Population (I=Intent-to-Treat, PP=Per Protocol)
Tafenoquine = Tafenoquine 200mg, Mefloquine = Mefloquine 250mg

Listing B.11

Significant Medical And Surgical History

Treatment : Tafenoquine

Subject Number	Popn*	WHO Body System	Preferred Term	Diagnosis Text	Year of First Diagnosis	Status
030.001.00195	IPP	GENERAL BODY OR SYS UNSPEC	PAIN, ABDOMINO-PELVIC	ABDOMINAL PAIN	2000	Past
		GENITOURINARY	VIRAL DIS EXANTHEM	GENITAL HERPES	2000	Ongoing
030.001.00201	IPP	METABOLIC NUTRITIONAL IMMUNE	CREATININE, INCREASED	ELEVATED CREATININE	2000	Past
030.001.00203	IPP	GENERAL BODY OR SYS UNSPEC	HEADACHE	HEADACHE	2000	Past
		MUSCULOSKELETAL	PAIN, JOINT	ARTHRALGIAS	2000	Past
		RESPIRATORY	RHINITIS,NOS	RHINITIS	2000	Past
030.001.00205	IPP	DIGESTIVE	DYSPEPSIA	DYSPEPSIA	2000	Past
		GENERAL BODY OR SYS UNSPEC	BACK PAIN	CHRONIC BACK PAIN	2000	Ongoing
		GENERAL BODY OR SYS UNSPEC	HEADACHE	HEADACHE	2000	Past
030.001.00207	IPP	GENERAL BODY OR SYS UNSPEC	HEADACHE	CHRONIC HEADACHE	2000	Ongoing
		GENERAL BODY OR SYS UNSPEC	PAIN, ABDOMINO-PELVIC	EPIGASTRIC PAIN	2000	Past
		MUSCULOSKELETAL	PAIN, JOINT	CHRONIC ARTHRALGIAS	2000	Ongoing
		NERVOUS SENSE ORGANS	VISUAL DISTURB	MYOPIA	2000	Ongoing
		RESPIRATORY	UPPER RESP DISORD, OTHER	NASAL CONGESTION	2000	Past
		RESPIRATORY	UPPER RESP INFECT, ACUTE	UPPER RESPIRATORY TRACT INFECTION	2000	Past
030.001.00210	IPP	RESPIRATORY	COUGH	COUGH	2000	Ongoing
030.001.00218	IPP	DIGESTIVE	CONSTIPATION	CONSTIPATION	2000	Past
		GENERAL BODY OR SYS UNSPEC	PAIN UNSP, CHEST	RECURRENT CHEST PAIN	1998	Ongoing

*Popn=Population (I=Intent-to-Treat, PP=Per Protocol)
Tafenoquine = Tafenoquine 200mg, Mefloquine = Mefloquine 250mg

SB252263 - 030

Listing B.11

Significant Medical And Surgical History

Treatment : Tafenoquine

Subject Number	Popn*	WHO Body System	Preferred Term	Diagnosis Text	Year of First Diagnosis	Status
030.001.00218	IPP	GENERAL BODY OR SYS UNSPEC	PAIN, ABDOMINO-PELVIC	ABDOMINAL PAIN	1999	Ongoing
		GENERAL BODY OR SYS UNSPEC	VIRAL INFECTION	VIRAL SYNDROME	2000	Past
030.001.00228	IPP	GENERAL BODY OR SYS UNSPEC	HEADACHE	HEADACHE	2000	Past
		GENITOURINARY	VENEREAL DIS	GONOCOCCAL URETHRITIS	2000	Past
		METABOLIC NUTRITIONAL IMMUNE	SERUM BILIRUBIN, INCREASED	HYPERBILIRUBINAEMIA	2000	Ongoing
030.001.00230	IPP	RESPIRATORY	ASTHMA	ASTHMA	1990	Ongoing
030.001.00231	IPP	GENERAL BODY OR SYS UNSPEC	PAIN, ABDOMINO-PELVIC	INTERMITTENT ABDOMINAL PAIN	1999	Ongoing
		HEMATIC HEMATOPOIETIC LYMPH	ANEMIA, OTHER	ANAEMIA	2000	Ongoing
		INTEGUMENTARY	SKIN SUBCUT DISORD, OTHER	FOLLICULITIS	1999	Ongoing
		RESPIRATORY	COUGH	COUGH	2000	Past
030.001.00233	IPP	DIGESTIVE	TEETH DISORD	DENTAL CARIES	1998	Ongoing
030.001.00240	IPP	HEMATIC HEMATOPOIETIC LYMPH	ERYTHROCYTOSIS	ABNORMAL HEMATOCRIT VALUE INCREASED	2000	Ongoing
030.001.00243	IPP	GENERAL BODY OR SYS UNSPEC	BACK PAIN	CHRONIC BACK PAIN	2000	Ongoing
030.001.00246	IPP	INTEGUMENTARY	SKIN SUBCUT DISORD, OTHER	HYPERKERATOSIS LEFT NECK AREA	2000	Ongoing
		RESPIRATORY	UPPER RESP INFECT, ACUTE	URI	2000	Past

*Popn=Population (I=Intent-to-Treat, PP=Per Protocol)
Tafenoquine = Tafenoquine 200mg, Mefloquine = Mefloquine 250mg

SB252263 - 030

Listing B.11

Significant Medical And Surgical History

Treatment : Tafenoquine

Subject Number	Popn*	WHO Body System	Preferred Term	Diagnosis Text	Year of First Diagnosis	Status
030.001.00249	IPP	GENERAL BODY OR SYS UNSPEC	HEADACHE	HEADACHE	2000	Past
		RESPIRATORY	RHINITIS,NOS	RHINITIS	2000	Past
030.001.00254	I	METABOLIC NUTRITIONAL IMMUNE	ANOREXIA	ANOREXIA	2000	Past
030.001.00261		GENITOURINARY	GENITAL FEMALE DISORD, OTHER	DYSMENORRHEA	2000	Ongoing
		GENITOURINARY	GENITAL FEMALE DISORD, OTHER	MENORRHAGIA	2000	Ongoing
		GENITOURINARY	GENITAL FEMALE DISORD, OTHER	METRORRHAGIA	2000	Ongoing
		GENITOURINARY	INFERTILITY, FEMALE	SECONDARY INFERTILITY {FEMALE}	1991	Ongoing
030.001.00264	IPP	GENERAL BODY OR SYS UNSPEC	HEADACHE	HEADACHE	2000	Ongoing
		MUSCULOSKELETAL	PAIN, JOINT	JOINT PAIN	2000	Ongoing
030.001.00267	IPP	DIGESTIVE	ENTERITIS COLITIS	GASTROENTERITIS	2000	Past
		DIGESTIVE	TEETH DISORD	DENTAL CARIES	2000	Ongoing
		HEMATIC HEMATOPOIETIC LYMPH	ARTHROPOD-BORNE DIS, OTHER	MALARIA	2000	Past
		METABOLIC NUTRITIONAL IMMUNE	BLOOD EXAM, ABN	BILIRUBINEMIA	2000	Ongoing
030.001.00271	I	METABOLIC NUTRITIONAL IMMUNE	SERUM BILIRUBIN, INCREASED	HYPERBILIRUBINEMIA	2000	Ongoing
030.001.00272	IPP	GENITOURINARY	INFLAM PELVIC DIS	PELVIC INFLAMMATORY DISEASE	2000	Ongoing

*Popn=Population (I=Intent-to-Treat, PP=Per Protocol)
Tafenoquine = Tafenoquine 200mg, Mefloquine = Mefloquine 250mg

SB252263 - 030

Listing B.11

Significant Medical And Surgical History

Treatment : Tafenoquine

Subject Number	Popn*	WHO Body System	Preferred Term	Diagnosis Text	Year of First Diagnosis	Status
030.001.00279	IPP	DIGESTIVE	DYSPEPSIA	DYSPEPSIA	2000	Past
		GENERAL BODY OR SYS UNSPEC	ALLERGY, NEC	ALLERGY	2000	Past
		HEMATIC HEMATOPOIETIC LYMPH	ARTHROPOD-BORNE DIS, OTHER	MALARIA	2000	Past
		METABOLIC NUTRITIONAL IMMUNE	CREATININE, INCREASED	ELEVATED CREATININE	2000	Ongoing
		METABOLIC NUTRITIONAL IMMUNE	MALNUTRITION	MALNUTRITION	2000	Past
030.001.00282	IPP	GENERAL BODY OR SYS UNSPEC	PAIN, ABDOMINO-PELVIC	ABDOMINAL PAIN	2000	Ongoing
030.001.00288	IPP	GENERAL BODY OR SYS UNSPEC	PAIN, ABDOMINO-PELVIC	ABDOMINAL COLIC	1998	Past
030.001.00293	IPP	DIGESTIVE	HELMINTHIASIS	STRONGYLOIDIASIS	2000	Past
		HEMATIC HEMATOPOIETIC LYMPH	ARTHROPOD-BORNE DIS, OTHER	MALARIA	2000	Past
		HEMATIC HEMATOPOIETIC LYMPH	RBC'S, ABN	MICROCYTOSIS	2000	Ongoing
		RESPIRATORY	RHINITIS, ALLERGIC	ALLERGIC RHINITIS	2000	Ongoing
030.001.00296	IPP	DIGESTIVE	DIARRHEA	INTERMITTENT DIARRHOEA	1998	Ongoing
		GENERAL BODY OR SYS UNSPEC	PAIN, ABDOMINO-PELVIC	ABDOMINAL PAIN	1997	Ongoing
030.001.00298	IPP	DIGESTIVE	NAUSEA	RECURRENT NAUSEA	1985	Ongoing
		GENERAL BODY OR SYS UNSPEC	HEADACHE	RECURRENT HEADACHE	1985	Ongoing
		NERVOUS SENSE ORGANS	INJURY, SUPERFICIAL	CORNEAL ABRASION	1989	Past

*Popn=Population (I=Intent-to-Treat, PP=Per Protocol)
Tafenoquine = Tafenoquine 200mg, Mefloquine = Mefloquine 250mg

SB252263 - 030

Listing B.11

Significant Medical And Surgical History

Treatment : Tafenoquine

Subject Number	Popn*	WHO Body System	Preferred Term	Diagnosis Text	Year of First Diagnosis	Status
030.001.00301	IPP	INTEGUMENTARY	INFLAM SKIN SUBCUT	DERMATITIS	2000	Ongoing
		RESPIRATORY	UPPER RESP INFECT, ACUTE	UPPER RESPIRATORY TRACT INFECTION	2000	Past
030.001.00302	I	GENERAL BODY OR SYS UNSPEC	VIRAL INFECTION	VIRAL SYNDROME	2000	Past
		HEMATIC HEMATOPOIETIC LYMPH	ARTHROPOD-BORNE DIS, OTHER	MALARIA	2000	Past
		METABOLIC NUTRITIONAL IMMUNE	MALNUTRITION	MALNUTRITION	2000	Past
030.001.00306	IPP	DIGESTIVE	DYSPEPSIA	DYSPEPSIA	2000	Past
		METABOLIC NUTRITIONAL IMMUNE	TRANSAMINASE LDH, ELEVATION	RAISED ALT	2000	Ongoing
		RESPIRATORY	UPPER RESP INFECT, ACUTE	UPPER RESPIRATORY TRACT INFECTION	2000	Past

*Popn=Population (I=Intent-to-Treat, PP=Per Protocol)
Tafenoquine = Tafenoquine 200mg, Mefloquine = Mefloquine 250mg

SB252263 - 030

Listing B.11

Significant Medical And Surgical History

Treatment : Mefloquine

Subject Number	Popn*	WHO Body System	Preferred Term	Diagnosis Text	Year of First Diagnosis	Status
030.001.00007	IPP	HEMATIC HEMATOPOIETIC LYMPH	RBC'S, ABN	MICROCYTOSIS	2000	Ongoing
030.001.00008	IPP	RESPIRATORY	RHINITIS, ALLERGIC	ALLERGIC RHINITIS	2000	Past
030.001.00010	IPP	GENERAL BODY OR SYS UNSPEC	BACK PAIN	LUMBAGO PAIN	2000	Past
		GENERAL BODY OR SYS UNSPEC	HEADACHE	HEADACHE	2000	Past
030.001.00013	IPP	GENERAL BODY OR SYS UNSPEC	PAIN, GENERAL	PAIN	2000	Past
		INTEGUMENTARY	MYCOSES	TINEA CORPORIS	2000	Ongoing
		METABOLIC NUTRITIONAL IMMUNE	ANOREXIA	LOSS OF APPETITE	2000	Past
		RESPIRATORY	COUGH	COUGH	2000	Past
030.001.00018	IPP	DIGESTIVE	DYSPEPSIA	DYSPEPSIA	2000	Ongoing
030.001.00021	IPP	GENERAL BODY OR SYS UNSPEC	MALAISE AND FATIGUE	MALAISE	2000	Past
		MUSCULOSKELETAL	PAIN, JOINT	JOINT PAIN	2000	Past
		RESPIRATORY	BRONCHITIS, OTHER	BRONCHITIS	2000	Ongoing
030.001.00023	I	CARDIOVASCULAR	HYPOTENSION, OTHER	HYPOTENSION	2000	Ongoing
		DIGESTIVE	DYSPEPSIA	DYSPEPSIA	2000	Past
		DIGESTIVE	TEETH DISORD	DENTAL CARIES	2000	Ongoing
		GENERAL BODY OR SYS UNSPEC	ALLERGY, NEC	ALLERGY	2000	Past
		GENERAL BODY OR SYS UNSPEC	HEADACHE	HEADACHE	2000	Past
		MUSCULOSKELETAL	MYALGIA	MUSCULOSKELETAL PAIN	2000	Past
		RESPIRATORY	COUGH	COUGH	2000	Ongoing

*Popn=Population (I=Intent-to-Treat, PP=Per Protocol)
Tafenoquine = Tafenoquine 200mg, Mefloquine = Mefloquine 250mg

SB252263 - 030

Listing B.11

Significant Medical And Surgical History

Treatment : Mefloquine

Subject Number	Popn*	WHO Body System	Preferred Term	Diagnosis Text	Year of First Diagnosis	Status
030.001.00034	IPP	DIGESTIVE	DYSPEPSIA	DYSPEPSIA	2000	Past
		METABOLIC NUTRITIONAL IMMUNE	TRANSAMINASE LDH, ELEVATION	ABNORMAL ALT {ELEVATED}	2000	Ongoing
		MUSCULOSKELETAL	MYALGIA	MUSCULOSKELETAL PAIN	2000	Ongoing
030.001.00035		GENITOURINARY	URINARY TRACT INFECTION	URINARY TRACT INFECTION	2000	Past
		MUSCULOSKELETAL	MYALGIA	MYALGIA	2000	Past
030.001.00036	I	GENERAL BODY OR SYS UNSPEC	ALLERGY, NEC	ALLERGY	2000	Past
030.001.00047	IPP	DIGESTIVE	TEETH DISORD	DENTAL CARIES	1996	Ongoing
		GENERAL BODY OR SYS UNSPEC	HEADACHE	HEADACHE	2000	Past
		HEMATIC HEMATOPOIETIC LYMPH	LYMPHADENOPATHY	POST AURICULAR LYMPHADENOPATHY	2000	Ongoing
		INTEGUMENTARY	MYCOSES	FUNGAL SKIN CONDITION	2000	Past
030.001.00052	IPP	GENERAL BODY OR SYS UNSPEC	BACK PAIN	BACKACHE	2000	Ongoing
		GENERAL BODY OR SYS UNSPEC	VIRAL INFECTION	VIRAL SYNDROME	2000	Ongoing
		MUSCULOSKELETAL	MYALGIA	MYALGIAS	2000	Past
030.001.00053	IPP	METABOLIC NUTRITIONAL IMMUNE	BLOOD EXAM, ABN	BILIRUBINAEMIA	2000	Ongoing
030.001.00054	IPP	HEMATIC HEMATOPOIETIC LYMPH	ARTHROPOD-BORNE DIS, OTHER	MALARIA	2000	Past

*Popn=Population (I=Intent-to-Treat, PP=Per Protocol)
Tafenoquine = Tafenoquine 200mg, Mefloquine = Mefloquine 250mg

SB252263 - 030

Listing B.11

Significant Medical And Surgical History

Treatment : Mefloquine

Subject Number	Popn*	WHO Body System	Preferred Term	Diagnosis Text	Year of First Diagnosis	Status
030.001.00054	IPP	METABOLIC NUTRITIONAL IMMUNE	SERUM BILIRUBIN, INCREASED	ABNORMAL BILIRUBIN {INCREASED}	2000	Ongoing
		RESPIRATORY	UPPER RESP INFECT, ACUTE	UPPER RESPIRATORY TRACT INFECTION	2000	Past
030.001.00062	IPP	DIGESTIVE	TEETH DISORD	DENTAL CARIES	2000	Ongoing
		GENERAL BODY OR SYS UNSPEC	OPEN WOUND	WOUND LEFT FOOT	2000	Ongoing
		GENITOURINARY	URINARY TRACT INFECTION	URINARY TRACT INFECTION	2000	Past
		INTEGUMENTARY	ULCER, SKIN	TROPICAL ULCER	2000	Ongoing
030.001.00066	IPP	DIGESTIVE	DYSPEPSIA	DYSPEPSIA	2000	Past
		GENERAL BODY OR SYS UNSPEC	HEADACHE	HEADACHE	2000	Past
		METABOLIC NUTRITIONAL IMMUNE	BLOOD EXAM, ABN	ABNORMAL PLATELETS	2000	Ongoing
030.001.00069	I	GENERAL BODY OR SYS UNSPEC	HEADACHE	HEADACHE	2000	Ongoing
		GENERAL BODY OR SYS UNSPEC	PAIN, ABDOMINO-PELVIC	ABDOMINAL PAIN	2000	Past
		INTEGUMENTARY	SKIN INFECT, LOCAL, OTHER	SEPTIC SKIN CONDITION	2000	Ongoing
		METABOLIC NUTRITIONAL IMMUNE	SERUM BILIRUBIN, INCREASED	HYPERBILIRUBINEMIA	2000	Ongoing
030.001.00071	IPP	GENERAL BODY OR SYS UNSPEC	BACK PAIN	CHRONIC BACK PAIN	2000	Ongoing
		GENERAL BODY OR SYS UNSPEC	HEADACHE	HEADACHE	2000	Ongoing
		METABOLIC NUTRITIONAL IMMUNE	SERUM BILIRUBIN, INCREASED	HYPERBILIRUBINEMIA	2000	Ongoing
		NERVOUS SENSE ORGANS	NEURALGIA, BACK	LEFT SCIATICA	2000	Ongoing

*Popn=Population (I=Intent-to-Treat, PP=Per Protocol)
Tafenoquine = Tafenoquine 200mg, Mefloquine = Mefloquine 250mg

SB252263 - 030

Listing B.11

Significant Medical And Surgical History

Treatment : Mefloquine

Subject Number	Popn*	WHO Body System	Preferred Term	Diagnosis Text	Year of First Diagnosis	Status
030.001.00073	IPP	GENERAL BODY OR SYS UNSPEC	CELLULITIS ABSCESS	LYMPHANGITIS CELLULITIS {LEFT LEG}	2000	Past
		GENERAL BODY OR SYS UNSPEC	CELLULITIS ABSCESS	PAIN (CELLULITIS)	2000	Past
		GENERAL BODY OR SYS UNSPEC	PAIN, ABDOMINO-PELVIC	ABDOMINAL PAIN	2000	Past
030.001.00075	IPP	GENERAL BODY OR SYS UNSPEC	HEADACHE	HEADACHE	2000	Ongoing
		GENERAL BODY OR SYS UNSPEC	VIRAL INFECTION	VIRAL SYNDROME	2000	Ongoing
030.001.00083	IPP	METABOLIC NUTRITIONAL IMMUNE	TRANSAMINASE LDH, ELEVATION	ELEVATED ALT	2000	Ongoing
030.001.00089	IPP	GENERAL BODY OR SYS UNSPEC	HEADACHE	HEADACHE	2000	Past
030.001.00091	IPP	DIGESTIVE	DIARRHEA	DIARRHOEA	2000	Past
		DIGESTIVE	DYSPEPSIA	DYSPEPSIA	2000	Ongoing
		HEMATIC HEMATOPOIETIC LYMPH	SPLENOMEGALY	SPLENOMEGALY	2000	Ongoing
		RESPIRATORY	RHINITIS, ALLERGIC	ALLERGIC RHINITIS	2000	Ongoing
030.001.00095	IPP	DIGESTIVE	ENTERITIS COLITIS	GASTROENTERITIS	2000	Past
		GENERAL BODY OR SYS UNSPEC	PAIN UNSP, CHEST	CHEST PAIN	2000	Ongoing
		INTEGUMENTARY	INFLAM SKIN SUBCUT	ALLERGIC SKIN RASH	2000	Ongoing
		NERVOUS SENSE ORGANS	CONJUNCTIVAL DISORD	CONJUNCTIVITIS	2000	Ongoing
030.001.00096	IPP	GENITOURINARY	VENEREAL DIS	CHANCROID	2000	Ongoing

*Popn=Population (I=Intent-to-Treat, PP=Per Protocol)
Tafenoquine = Tafenoquine 200mg, Mefloquine = Mefloquine 250mg

SB252263 - 030

Listing B.11

Significant Medical And Surgical History

Treatment : Mefloquine

Subject Number	Popn*	WHO Body System	Preferred Term	Diagnosis Text	Year of First Diagnosis	Status
030.001.00096	IPP	METABOLIC NUTRITIONAL IMMUNE	SERUM BILIRUBIN, INCREASED	HYPERBILIRUBINAEMIA	2000	Ongoing
		MUSCULOSKELETAL	MYALGIA	MYOSITIS	2000	Past
030.001.00097	IPP	GENERAL BODY OR SYS UNSPEC	HEADACHE	HEADACHE	2000	Past
		HEMATIC HEMATOPOIETIC LYMPH	ANEMIA, IRON DEFIC	MICROCYTIC ANAEMIA	2000	Ongoing
		MUSCULOSKELETAL	PAIN, JOINT	ARTHRALGIA	2000	Past
		RESPIRATORY	COUGH	COUGH	2000	Past
030.001.00098	IPP	DIGESTIVE	DIARRHEA	DIARRHOEA	2000	Past
		DIGESTIVE	DYSPEPSIA	DYSPEPSIA	2000	Past
		GENERAL BODY OR SYS UNSPEC	PAIN, ABDOMINO-PELVIC	ABDOMINAL PAIN	2000	Past
030.001.00099	IPP	DIGESTIVE	NAUSEA	NAUSEA	2000	Past
		GENERAL BODY OR SYS UNSPEC	HEADACHE	HEADACHE	2000	Ongoing
		GENERAL BODY OR SYS UNSPEC	PAIN, ABDOMINO-PELVIC	ABDOMINAL PAIN	2000	Past
		GENITOURINARY	VIRAL DIS EXANTHEM	GENITAL HERPES	2000	Ongoing
		MUSCULOSKELETAL	MYALGIA	MUSCULOSKELETAL PAIN	2000	Ongoing
030.001.00105	IPP	DIGESTIVE	ENTERITIS COLITIS	GASTROENTERITIS	2000	Past
		DIGESTIVE	STOMACH DUODENUM DISORD	HYPEREMESIS	2000	Past
		GENERAL BODY OR SYS UNSPEC	PAIN UNSP, CHEST	PAIN (CHEST)	2000	Past
		GENERAL BODY OR SYS UNSPEC	PAIN, ABDOMINO-PELVIC	PAIN (ABDOMEN)	2000	Past
		GENERAL BODY OR SYS UNSPEC	SEPSIS	SEPSIS	2000	Past
		GENERAL BODY OR SYS UNSPEC	VIRAL DIS EXANTHEM	CHICKEN POX RASH	2000	Past

*Popn=Population (I=Intent-to-Treat, PP=Per Protocol)
Tafenoquine = Tafenoquine 200mg, Mefloquine = Mefloquine 250mg

SB252263 - 030

Listing B.11

Significant Medical And Surgical History

Treatment : Mefloquine

Subject Number	Popn*	WHO Body System	Preferred Term	Diagnosis Text	Year of First Diagnosis	Status
030.001.00105	IPP	HEMATIC HEMATOPOIETIC LYMPH	ARTHROPOD-BORNE DIS, OTHER	MALARIA	2000	Past
		HEMATIC HEMATOPOIETIC LYMPH	SPLENOMEGALY	SPLENOMEGALY	2000	Ongoing
		METABOLIC NUTRITIONAL IMMUNE	SERUM BILIRUBIN, INCREASED	HIGH BILIRUBIN INCREASE	2000	Ongoing
030.001.00110	IPP	GENERAL BODY OR SYS UNSPEC	HEADACHE	HEADACHE	2000	Ongoing
		HEMATIC HEMATOPOIETIC LYMPH	RBC'S, ABN	MICROCYTOSIS	2000	Ongoing
		METABOLIC NUTRITIONAL IMMUNE	SERUM BILIRUBIN, INCREASED	HYPERBILIRUBINAEMIA	2000	Past
		MUSCULOSKELETAL	PAIN, JOINT	ARTHRALGIAS	2000	Ongoing
030.001.00113	IPP	DIGESTIVE	TEETH DISORD	DENTAL CARIES	2000	Ongoing
		HEMATIC HEMATOPOIETIC LYMPH	ANEMIA, IRON DEFIC	MICROCYTIC ANEMIA	2000	Past
		NERVOUS SENSE ORGANS	CATARACT	RIGHT OCULAR PROBLEM (CATARACT)	2000	Past
030.001.00117	IPP	GENERAL BODY OR SYS UNSPEC	HEADACHE	RECURRENT HEADACHES	2000	Ongoing
		GENERAL BODY OR SYS UNSPEC	PAIN, ABDOMINO-PELVIC	ABDOMINAL PAIN	2000	Ongoing
		GENITOURINARY	CONDITIONS, PERINATAL	STILLBIRTH	1995	Past
		GENITOURINARY	NEOPLASMS BENIGN	UTERINE FIBROIDS	1994	Ongoing
		MUSCULOSKELETAL	MYALGIA	MUSCULOSKELETAL PAIN	2000	Past
		MUSCULOSKELETAL	PAIN, JOINT	ARTHRALGIAS	1999	Ongoing
030.001.00120	IPP	METABOLIC NUTRITIONAL IMMUNE	SERUM BILIRUBIN, INCREASED	HYPERBILIRUBINAEMIA	2000	Ongoing

*Popn=Population (I=Intent-to-Treat, PP=Per Protocol)
Tafenoquine = Tafenoquine 200mg, Mefloquine = Mefloquine 250mg

SB252263 - 030

Listing B.11

Significant Medical And Surgical History

Treatment : Mefloquine

Subject Number	Popn*	WHO Body System	Preferred Term	Diagnosis Text	Year of First Diagnosis	Status
030.001.00121	IPP	GENERAL BODY OR SYS UNSPEC	HEADACHE	HEADACHE	2000	Past
		HEMATIC HEMATOPOIETIC LYMPH	SPLENOMEGALY	SPLENOMEGALY	2000	Ongoing
030.001.00123	I	GENERAL BODY OR SYS UNSPEC	CELLULITIS ABSCESS	PARONYCHIA	2000	Past
		GENERAL BODY OR SYS UNSPEC	PAIN, ABDOMINO-PELVIC	ABDOMINAL PAIN	2000	Past
		GENITOURINARY	URETHRITIS	URETHRITIS	2000	Past
		GENITOURINARY	URINARY TRACT INFECTION	URINARY TRACT INFECTION	2000	Past
		HEMATIC HEMATOPOIETIC LYMPH	ARTHROPOD-BORNE DIS, OTHER	MALARIA	2000	Past
		INTEGUMENTARY	MYCOSES	TINEA CAPITIS	2000	Ongoing
		INTEGUMENTARY	MYCOSES	TINEA CORPORIS	2000	Ongoing
		METABOLIC NUTRITIONAL IMMUNE	SERUM BILIRUBIN, INCREASED	HYPERBILIRUBINAEMIA	2000	Ongoing
		MUSCULOSKELETAL	MYALGIA	MYALGIA	2000	Ongoing
030.001.00130	IPP	GENERAL BODY OR SYS UNSPEC	ALLERGY, NEC	ALLERGY	2000	Ongoing
		GENERAL BODY OR SYS UNSPEC	HEADACHE	HEADACHE	2000	Ongoing
		MUSCULOSKELETAL	MYALGIA	MYOSITIS	2000	Ongoing
030.001.00132	IPP	INTEGUMENTARY	MYCOSES	TINEA VERSICOLOR	1998	Ongoing
		RESPIRATORY	EPISTAXIS	EPISTAXIS	1998	Ongoing
		RESPIRATORY	RHINITIS, ALLERGIC	ALLERGIC RHINITIS	1999	Ongoing
030.001.00134	IPP	DIGESTIVE	TEETH DISORD	DENTAL CARIES	2000	Ongoing
		DIGESTIVE	TEETH DISORD	TOOTHACHE	2000	Ongoing
		GENITOURINARY	INFLAM PELVIC DIS	PELVIC INFLAMMATORY DISEASE	2000	Ongoing

*Popn=Population (I=Intent-to-Treat, PP=Per Protocol)
Tafenoquine = Tafenoquine 200mg, Mefloquine = Mefloquine 250mg

SB252263 - 030

Listing B.11

Significant Medical And Surgical History

Treatment : Mefloquine

Subject Number	Popn*	WHO Body System	Preferred Term	Diagnosis Text	Year of First Diagnosis	Status
030.001.00134	IPP	HEMATIC HEMATOPOIETIC LYMPH	ARTHROPOD-BORNE DIS, OTHER	MALARIA	2000	Past
		MUSCULOSKELETAL	ARTHROPATHY	RIGHT KNEE ARTHRITIS	2000	Ongoing
030.001.00142	IPP	DIGESTIVE	TEETH DISORD	DENTAL CARIES	1999	Ongoing
		METABOLIC NUTRITIONAL IMMUNE	SERUM BILIRUBIN, INCREASED	HYPERBILIRUBINAEMIA	2000	Ongoing
		MUSCULOSKELETAL	MYALGIA	MUSCULOSKELETAL PAIN	2000	Ongoing
030.001.00143	IPP	DIGESTIVE	ENTERITIS COLITIS	GASTROENTERITIS	2000	Past
030.001.00144	IPP	DIGESTIVE	TEETH DISORD	DENTAL PAIN	2000	Ongoing
		GENERAL BODY OR SYS UNSPEC	HEADACHE	HEADACHE	2000	Past
030.001.00147	IPP	METABOLIC NUTRITIONAL IMMUNE	CREATININE, INCREASED	HIGH SERUM CREATININE	2000	Ongoing
		MUSCULOSKELETAL	MYALGIA	MYALGIA	2000	Past
		RESPIRATORY	UPPER RESP INFECT, ACUTE	UPPER RESPIRATORY TRACT INFECTION	2000	Past
030.001.00156	IPP	METABOLIC NUTRITIONAL IMMUNE	SERUM BILIRUBIN, INCREASED	HYPERBILIRUBINAEMIA	2000	Ongoing
		RESPIRATORY	RHINITIS, ALLERGIC	ALLERGIC RHINITIS	2000	Ongoing
030.001.00163	IPP	DIGESTIVE	TEETH DISORD	DENTAL CARIES	2000	Ongoing
		HEMATIC HEMATOPOIETIC LYMPH	ARTHROPOD-BORNE DIS, OTHER	MALARIA	2000	Past

*Popn=Population (I=Intent-to-Treat, PP=Per Protocol)
Tafenoquine = Tafenoquine 200mg, Mefloquine = Mefloquine 250mg

Listing B.11

Significant Medical And Surgical History

Treatment : Mefloquine

Subject Number	Popn*	WHO Body System	Preferred Term	Diagnosis Text	Year of First Diagnosis	Status
030.001.00167	IPP	GENERAL BODY OR SYS UNSPEC	BACK PAIN	CHRONIC BACKACHE	2000	Ongoing
		GENITOURINARY	URINARY TRACT INFECTION	URINARY TRACT INFECTION	2000	Past
		GENITOURINARY	VAGINITIS	VAGINITIS	2000	Ongoing
030.001.00168	IPP	GENITOURINARY	GENITAL FEMALE DISORD, OTHER	DYSMENORRHOEA	1996	Ongoing
		NERVOUS SENSE ORGANS	OTITIS MEDIA	OTITIS MEDIA, RECURRENT	1972	Ongoing
030.001.00170	IPP	DIGESTIVE	INTEST INFECT DIS	AMOEBIASIS	2000	Ongoing
		GENERAL BODY OR SYS UNSPEC	MYCOSES	FUNGAL INFECTION	2000	Past
		METABOLIC NUTRITIONAL IMMUNE	SERUM BILIRUBIN, INCREASED	HYPERBILIRUBINAEMIA	2000	Past
		METABOLIC NUTRITIONAL IMMUNE	TRANSAMINASE LDH, ELEVATION	ELEVATED SERUM SGPT ALT	2000	Past
030.001.00178	I	GENERAL BODY OR SYS UNSPEC	BACK PAIN	LOWER BACK PAIN	2000	Ongoing
		HEMATIC HEMATOPOIETIC LYMPH	ARTHROPOD-BORNE DIS, OTHER	MALARIA	2000	Past
		METABOLIC NUTRITIONAL IMMUNE	TRANSAMINASE LDH, ELEVATION	ELEVATED SERUM SGPT (ALT)	2000	Past
		MUSCULOSKELETAL	MYALGIA	MYALGIA	2000	Ongoing
030.001.00180	IPP	HEMATIC HEMATOPOIETIC LYMPH	ARTHROPOD-BORNE DIS, OTHER	MALARIA	2000	Past
		RESPIRATORY	UPPER RESP INFECT, ACUTE	UPPER RESPIRATORY TRACT INFECTION	2000	Past

*Popn=Population (I=Intent-to-Treat, PP=Per Protocol)
Tafenoquine = Tafenoquine 200mg, Mefloquine = Mefloquine 250mg

SB252263 - 030

Listing B.11

Significant Medical And Surgical History

Treatment : Mefloquine

Subject Number	Popn*	WHO Body System	Preferred Term	Diagnosis Text	Year of First Diagnosis	Status
030.001.00181	IPP	GENERAL BODY OR SYS UNSPEC	TYPHOID INFECT	TYPHOID FEVER	1999	Past
030.001.00185	IPP	DIGESTIVE	DYSPEPSIA	DYSPEPSIA	2000	Past
		GENERAL BODY OR SYS UNSPEC	HEADACHE	CHRONIC HEADACHE	2000	Ongoing
		GENITOURINARY	URINARY TRACT INFECTION	URINARY TRACT INFECTION	2000	Past
		HEMATIC HEMATOPOIETIC LYMPH	LEUCOCYTOSIS	LEUCOCYTOSIS	2000	Ongoing
		HEMATIC HEMATOPOIETIC LYMPH	RBC'S, ABN	MICROCYTOSIS	2000	Ongoing
		NERVOUS SENSE ORGANS	VISUAL DISTURB	PHOTOPHOBIA	2000	Ongoing
030.001.00188	IPP	DIGESTIVE	CONSTIPATION	CONSTIPATION	2000	Past
		DIGESTIVE	ENTERITIS COLITIS	GASTROENTERITIS	2000	Past
		DIGESTIVE	GASTRITIS DUODENITIS	ABDOMINAL PAIN EPIGASTRIC PAIN {GASTRITIS}	1998	Past
		INTEGUMENTARY	PRURITUS DISORD, UNSPEC	PRURITUS	2000	Past
030.001.00189	I	GENERAL BODY OR SYS UNSPEC	HEADACHE	HEADACHE	2000	Ongoing
		HEMATIC HEMATOPOIETIC LYMPH	SPLENOMEGALY	SPLENOMEGALY	2000	Ongoing
		HEMATIC HEMATOPOIETIC LYMPH	THROMBOCYTOPENIA	THROMBOCYTOPENIA	2000	Ongoing
		MUSCULOSKELETAL	PAIN, JOINT	ARTHRALGIAS	2000	Ongoing
030.001.00196	IPP	GENERAL BODY OR SYS UNSPEC	ALLERGY, NEC	ALLERGY	2000	Past

*Popn=Population (I=Intent-to-Treat, PP=Per Protocol)
Tafenoquine = Tafenoquine 200mg, Mefloquine = Mefloquine 250mg

SB252263 - 030

Listing B.11

Significant Medical And Surgical History

Treatment : Mefloquine

Subject Number	Popn*	WHO Body System	Preferred Term	Diagnosis Text	Year of First Diagnosis	Status
030.001.00196	IPP	HEMATIC HEMATOPOIETIC LYMPH	SPLENOMEGALY	SPLENOMEGALY	2000	Ongoing
		RESPIRATORY	UPPER RESP INFECT, ACUTE	UPPER RESPIRATORY TRACT INFECTION	2000	Past
030.001.00198	IPP	GENERAL BODY OR SYS UNSPEC	HEADACHE	HEADACHE	2000	Ongoing
		MUSCULOSKELETAL	MYALGIA	MYALGIAS	2000	Ongoing
030.001.00199	IPP	HEMATIC HEMATOPOIETIC LYMPH	SPLENOMEGALY	SPLENOMEGALY	2000	Ongoing
030.001.00200	IPP	GENERAL BODY OR SYS UNSPEC	ALLERGY, NEC	ALLERGY	2000	Past
030.001.00206	I	RESPIRATORY	NASOPHARYNGITIS, ACUTE	ACUTE RHINITIS	2000	Past
030.001.00213	IPP	METABOLIC NUTRITIONAL IMMUNE	SERUM BILIRUBIN, INCREASED	HYPERBILIRUBINAEMIA	2000	Ongoing
030.001.00214	IPP	GENERAL BODY OR SYS UNSPEC	HEADACHE	HEADACHE	2000	Ongoing
		INTEGUMENTARY	INFLAM SKIN SUBCUT	DERMATITIS	2000	Ongoing
		MUSCULOSKELETAL	OSTEOARTHROSIS	ARTHRITIS RIGHT ANKLE JOINT	2000	Ongoing
		RESPIRATORY	UPPER RESP INFECT, ACUTE	UPPER RESPIRATORY TRACT INFECTION	2000	Past
030.001.00216	IPP	GENERAL BODY OR SYS UNSPEC	BACK PAIN	LUMBAGO	2000	Ongoing

*Popn=Population (I=Intent-to-Treat, PP=Per Protocol)
Tafenoquine = Tafenoquine 200mg, Mefloquine = Mefloquine 250mg

SB252263 - 030

Listing B.11

Significant Medical And Surgical History

Treatment : Mefloquine

Subject Number	Popn*	WHO Body System	Preferred Term	Diagnosis Text	Year of First Diagnosis	Status
030.001.00221	IPP	DIGESTIVE	GASTRITIS DUODENITIS	GASTRITIS	2000	Ongoing
		GENERAL BODY OR SYS UNSPEC	BACK PAIN	CHRONIC BACK PAIN	2000	Ongoing
		METABOLIC NUTRITIONAL IMMUNE	SERUM BILIRUBIN, INCREASED	HYPERBILIRUBINAEMIA	2000	Past
		MUSCULOSKELETAL	PAIN, JOINT	PAIN (JOINT)	2000	Past
030.001.00224	IPP	DIGESTIVE	INTEST INFECT DIS	INTESTINAL INFECTION	2000	Past
		DIGESTIVE	NAUSEA	CHRONIC NAUSEA	2000	Ongoing
		GENERAL BODY OR SYS UNSPEC	HEADACHE	CHRONIC HEADACHE	2000	Ongoing
		GENERAL BODY OR SYS UNSPEC	PAIN UNSP, CHEST	CHRONIC CHEST PAIN (NON-CARDIAC)	1999	Ongoing
		GENERAL BODY OR SYS UNSPEC	PAIN, ABDOMINO-PELVIC	CHRONIC ABDOMINAL PAIN (RIB) {NOS}	2000	Ongoing
		NERVOUS SENSE ORGANS	CONJUNCTIVAL DISORD	ALLERGIC CONJUNCTIVITIS	2000	Ongoing
030.001.00227	IPP	DIGESTIVE	DYSPEPSIA	DYSPEPSIA	2000	Past
		DIGESTIVE	TEETH DISORD	DENTAL CARIES	2000	Ongoing
030.001.00232	IPP	DIGESTIVE	ENTERITIS COLITIS	GASTROENTERITIS	2000	Past
		METABOLIC NUTRITIONAL IMMUNE	ANOREXIA	ANOREXIA	2000	Past
030.001.00235	IPP	DIGESTIVE	ENTERITIS COLITIS	GASTROENTERITIS	2000	Past
		GENERAL BODY OR SYS UNSPEC	PAIN UNSP, CHEST	CHEST PAIN SYNDROME	2000	Ongoing
		GENERAL BODY OR SYS UNSPEC	PAIN, LIMB	KNEE PAIN	2000	Ongoing
		GENITOURINARY	INFLAM PELVIC DIS	PELVIC INFLAMMATORY DISEASE	2000	Past
		MUSCULOSKELETAL	MYALGIA	MUSCULOSKELETAL PAIN	2000	Ongoing

*Popn=Population (I=Intent-to-Treat, PP=Per Protocol)
Tafenoquine = Tafenoquine 200mg, Mefloquine = Mefloquine 250mg

SB252263 - 030

Listing B.11

Significant Medical And Surgical History

Treatment : Mefloquine

Subject Number	Popn*	WHO Body System	Preferred Term	Diagnosis Text	Year of First Diagnosis	Status
030.001.00237	IPP	HEMATIC HEMATOPOIETIC LYMPH	POLYCYTHEMIA	POLYCYTHAEMIA	2000	Ongoing
030.001.00241	IPP	HEMATIC HEMATOPOIETIC LYMPH	ARTHROPOD-BORNE DIS, OTHER	MALARIA	2000	Past
		MUSCULOSKELETAL	PAIN, JOINT	JOINT PAIN	2000	Past
030.001.00245	IPP	DIGESTIVE	INTEST INFECT DIS	AMOEBIASIS	2000	Past
030.001.00250		DIGESTIVE	INTEST INFECT DIS	AMOEBIC DYSENTERY	2000	Past
		DIGESTIVE	INTEST INFECT DIS	INTESTINAL INFECTION	2000	Past
		GENERAL BODY OR SYS UNSPEC	PAIN, GENERAL	PAIN	2000	Past
		HEMATIC HEMATOPOIETIC LYMPH	ARTHROPOD-BORNE DIS, OTHER	MALARIA	2000	Past
		MUSCULOSKELETAL	BONE CARTIL DISORD, OTHER	COSTAL CHONDRITIS	1999	Ongoing
030.001.00255	IPP	HEMATIC HEMATOPOIETIC LYMPH	ARTHROPOD-BORNE DIS, OTHER	MALARIA	2000	Past
		RESPIRATORY	UPPER RESP DIS, OTHER	ENLARGED TURBINATES {NASAL}	2000	Ongoing
030.001.00258	IPP	HEMATIC HEMATOPOIETIC LYMPH	THROMBOCYTOPENIA	ABNORMAL PLATELETS VALUE {DECREASE}	2000	Ongoing
030.001.00263	IPP	GENERAL BODY OR SYS UNSPEC	HEADACHE	HEADACHE	2000	Ongoing
		GENERAL BODY OR SYS UNSPEC	HERNIA, ABDOMINAL	LEFT INGUINAL HERNIA	2000	Ongoing
		INTEGUMENTARY	MYCOSES	FUNGAL SKIN INFECTION	2000	Ongoing

*Popn=Population (I=Intent-to-Treat, PP=Per Protocol)
Tafenoquine = Tafenoquine 200mg, Mefloquine = Mefloquine 250mg

SB252263 - 030

Listing B.11

Significant Medical And Surgical History

Treatment : Mefloquine

Subject Number	Popn*	WHO Body System	Preferred Term	Diagnosis Text	Year of First Diagnosis	Status
030.001.00263	IPP	METABOLIC NUTRITIONAL IMMUNE	SERUM BILIRUBIN, INCREASED	HYPERBILIRUBINAEMIA	2000	Ongoing
030.001.00265	IPP	GENERAL BODY OR SYS UNSPEC	PAIN UNSP, CHEST	CHEST PAIN	2000	Past
		HEMATIC HEMATOPOIETIC LYMPH	ARTHROPOD-BORNE DIS, OTHER	MALARIA	2000	Past
		MUSCULOSKELETAL	PAIN, JOINT	JOINT PAIN	2000	Past
		RESPIRATORY	RESP DIS, OTHER	BRONCHIAL SPASM	2000	Past
		RESPIRATORY	RHINITIS, ALLERGIC	ALLERGIC RHINITIS	2000	Ongoing
		RESPIRATORY	UPPER RESP INFECT, ACUTE	ACUTE UPPER RESPIRATORY TRACT INFECTION	2000	Past
030.001.00274	IPP	GENERAL BODY OR SYS UNSPEC	HEADACHE	HEADACHE	2000	Past
		HEMATIC HEMATOPOIETIC LYMPH	ARTHROPOD-BORNE DIS, OTHER	MALARIA	2000	Past
030.001.00280	IPP	DIGESTIVE	TEETH DISORD	PAINFUL TOOTH	2000	Ongoing
		HEMATIC HEMATOPOIETIC LYMPH	ARTHROPOD-BORNE DIS, OTHER	MALARIA	2000	Past
		INTEGUMENTARY	RASH OTHER SKIN ERUPTION	ALLERGIC RASH	2000	Past
		RESPIRATORY	COUGH	COUGH	2000	Past
030.001.00281	IPP	METABOLIC NUTRITIONAL IMMUNE	CREATININE, INCREASED	RAISED CREATININE	2000	Ongoing
030.001.00283	IPP	DIGESTIVE	HELMINTHIASIS	TAENIASIS	2000	Past
		INTEGUMENTARY	PRURITUS DISORD, UNSPEC	PRURITIS	2000	Ongoing

*Popn=Population (I=Intent-to-Treat, PP=Per Protocol)
Tafenoquine = Tafenoquine 200mg, Mefloquine = Mefloquine 250mg

SB252263 - 030

Listing B.11

Significant Medical And Surgical History

Treatment : Mefloquine

Subject Number	Popn*	WHO Body System	Preferred Term	Diagnosis Text	Year of First Diagnosis	Status
030.001.00286	IPP	DIGESTIVE	TEETH DISORD	DENTAL CARIES	1999	Ongoing
030.001.00289	IPP	GENERAL BODY OR SYS UNSPEC	HEADACHE	HEADACHE	2000	Past
		RESPIRATORY	UPPER RESP INFECT, ACUTE	URI	2000	Past
030.001.00290	IPP	METABOLIC NUTRITIONAL IMMUNE	SERUM BILIRUBIN, INCREASED	HYPERBILIRUBINAEMIA	2000	Ongoing
030.001.00292	IPP	DIGESTIVE	ENTERITIS COLITIS	ACUTE GASTROENTERITIS	2000	Past
		METABOLIC NUTRITIONAL IMMUNE	TRANSAMINASE LDH, ELEVATION	ELEVATED ALT	2000	Ongoing
		NERVOUS SENSE ORGANS	EYE DISORD, OTHER	CORNEAL SCAR	1997	Ongoing
030.001.00305	IPP	METABOLIC NUTRITIONAL IMMUNE	SERUM BILIRUBIN, INCREASED	HYPERBILIRUBINAEMIA	2000	Ongoing

*Popn=Population (I=Intent-to-Treat, PP=Per Protocol)
Tafenoquine = Tafenoquine 200mg, Mefloquine = Mefloquine 250mg

Listing B.12

SB252263 - 030

Listing B.12

History Of Malaria

Treatment : Placebo

Subject Number	Popn*	History of Malaria	Date of last Attack	Time Since Last Attack**	No. attacks in last 6 months
030.001.00002	IPP	Yes	01APR2000	1	4.00
030.001.00005	IPP	Yes	01FEB2000	3	2.00
030.001.00011	IPP	Yes	01FEB2000	3	2.00
030.001.00012	IPP	Yes	01DEC1999	5	1.00
030.001.00015	I	Yes	01MAR2000	2	3.00
030.001.00019	IPP	Yes	01MAR2000	2	1.00
030.001.00020	IPP	Yes			0.00
030.001.00022	IPP	Yes	01MAR2000	2	3.00
030.001.00026	IPP	Yes	01DEC1999	5	1.00
030.001.00027	IPP	Yes	01DEC1999	5	3.00
030.001.00032	IPP	Yes	01DEC1994	65	0.00
030.001.00033	IPP	Yes	01MAR2000	2	2.00
030.001.00037	IPP	Yes	01DEC1998	17	0.00
030.001.00039	IPP	Yes	01APR1997	37	0.00
030.001.00041	IPP	Yes	01JAN1993	88	0.00
030.001.00044	IPP	Yes	01AUG1999	9	0.00
030.001.00055	IPP	Yes	01APR2000	1	2.00
030.001.00056	I	Yes	01DEC1999	5	1.00
030.001.00057	IPP	Yes	01FEB2000	3	2.00
030.001.00059	IPP	Yes	01DEC1999	5	1.00
030.001.00063	IPP	Yes	01AUG1999	9	0.00

*Popn=Population (I=Intent-to-Treat, PP=Per Protocol)
Blank = UNKNOWN entered on CRF
** Time since last attack= Date of screening visit - Date of last attack
Tafenoquine = Tafenoquine 200mg, Mefloquine = Mefloquine 250mg

SB252263 - 030

Listing B.12

History Of Malaria

Treatment : Placebo

Subject Number	Popn*	History of Malaria	Date of last Attack	Time Since Last Attack**	No. attacks in last 6 months
030.001.00064	IPP	Yes			0.00
030.001.00067	IPP	Yes	01DEC1999	5	3.00
030.001.00072	IPP	Yes	01AUG1999	9	0.00
030.001.00076	IPP	Yes	01APR2000	1	4.00
030.001.00078	IPP	Yes	01JUL1999	10	0.00
030.001.00080	IPP	Yes	01MAR2000	2	2.00
030.001.00081	IPP	Yes	01FEB2000	3	4.00
030.001.00087	I	Yes	01FEB1999	15	0.00
030.001.00088	IPP	Yes	01DEC1999	5	1.00
030.001.00090	IPP	Yes	01FEB2000	3	1.00
030.001.00092	IPP	Yes	01MAR2000	2	2.00
030.001.00101	IPP	Yes	01FEB2000	3	1.00
030.001.00103	IPP	Yes	01APR2000	1	1.00
030.001.00106	IPP	Yes	01FEB2000	3	2.00
030.001.00108	IPP	Yes	01JAN2000	4	3.00
030.001.00114	IPP	Yes	01APR2000	1	2.00
030.001.00115	IPP	Yes	01JAN2000	4	2.00
030.001.00116	IPP	Yes	01MAR2000	2	3.00
030.001.00119	IPP	Yes	01MAY1999	12	0.00
030.001.00124	IPP	Yes	01DEC1999	5	1.00
030.001.00125	IPP	No			

*Popn=Population (I=Intent-to-Treat, PP=Per Protocol)
Blank = UNKNOWN entered on CRF
** Time since last attack= Date of screening visit - Date of last attack
Tafenoquine = Tafenoquine 200mg, Mefloquine = Mefloquine 250mg

SB252263 - 030

Listing B.12

History Of Malaria

Treatment : Placebo

Subject Number	Popn*	History of Malaria	Date of last Attack	Time Since Last Attack**	No. attacks in last 6 months
030.001.00127	I	Yes	01DEC1999	5	2.00
030.001.00131	IPP	Yes	01MAR2000	2	2.00
030.001.00136	IPP	Yes	01APR1999	13	0.00
030.001.00137	IPP	Yes	01APR2000	1	4.00
030.001.00140	IPP	Yes	01FEB2000	3	1.00
030.001.00141	IPP	Yes	01FEB2000	3	3.00
030.001.00145	IPP	Yes	01OCT1999	7	1.00
030.001.00151	IPP	Yes	01JAN2000	4	1.00
030.001.00152	IPP	Yes	01MAR2000	2	3.00
030.001.00154	IPP	Yes	01APR2000	1	1.00
030.001.00160	I	Yes	01DEC1999	5	1.00
030.001.00162	IPP	Yes	01AUG1999	9	0.00
030.001.00164	IPP	Yes	01JAN2000	4	1.00
030.001.00166	IPP	Yes	01DEC1996	41	0.00
030.001.00169	IPP	Yes			0.00
030.001.00172	IPP	Yes	01JAN1995	64	0.00
030.001.00174	IPP	Yes	01FEB2000	3	1.00
030.001.00175	IPP	Yes	01APR2000	1	3.00
030.001.00182	I	Yes	01DEC1999	5	1.00
030.001.00183	IPP	Yes	01JAN2000	4	1.00
030.001.00191	IPP	Yes	01FEB2000	3	1.00

*Popn=Population (I=Intent-to-Treat, PP=Per Protocol)
Blank = UNKNOWN entered on CRF
** Time since last attack= Date of screening visit - Date of last attack
Tafenoquine = Tafenoquine 200mg, Mefloquine = Mefloquine 250mg

SB252263 - 030

Listing B.12

History Of Malaria

Treatment : Placebo

Subject Number	Popn*	History of Malaria	Date of last Attack	Time Since Last Attack**	No. attacks in last 6 months
030.001.00192	IPP	Yes	01MAR2000	2	4.00
030.001.00193	IPP	Yes	01APR2000	1	4.00
030.001.00197	IPP	Yes	01DEC1999	5	1.00
030.001.00202		Yes	01JAN2000	4	2.00
030.001.00204	I	No			
030.001.00208	IPP	Yes			0.00
030.001.00211	IPP	Yes	01MAR2000	2	3.00
030.001.00212	IPP	Yes	01FEB2000	3	3.00
030.001.00215	IPP	Yes	01APR2000	1	1.00
030.001.00219	IPP	Yes	01MAR2000	2	2.00
030.001.00222	IPP	Yes	01JUL1999	10	1.00
030.001.00225	IPP	No			
030.001.00226	IPP	Yes	01JAN2000	4	1.00
030.001.00229	IPP	Yes	01MAR2000	2	6.00
030.001.00234	IPP	Yes	01DEC1999	5	1.00
030.001.00236	IPP	Yes	01JAN2000	4	1.00
030.001.00238	IPP	Yes	01DEC1999	5	1.00
030.001.00242	IPP	Yes	01MAR2000	2	2.00
030.001.00247	IPP	Yes	01DEC1999	5	1.00
030.001.00248	IPP	Yes	01MAR2000	2	2.00
030.001.00251	IPP	Yes	01MAR2000	2	3.00

*Popn=Population (I=Intent-to-Treat, PP=Per Protocol)
Blank = UNKNOWN entered on CRF
** Time since last attack= Date of screening visit - Date of last attack
Tafenoquine = Tafenoquine 200mg, Mefloquine = Mefloquine 250mg

SB252263 - 030

Listing B.12

History Of Malaria

Treatment : Placebo

Subject Number	Popn*	History of Malaria	Date of last Attack	Time Since Last Attack**	No. attacks in last 6 months
030.001.00253	IPP	Yes			3.00
030.001.00256	IPP	Yes	01JAN2000	4	2.00
030.001.00257	IPP	Yes	01APR2000	1	4.00
030.001.00260	IPP	Yes	01MAR2000	2	2.00
030.001.00268	IPP	Yes	01SEP1999	8	0.00
030.001.00269	IPP	Yes	01JUN1999	11	0.00
030.001.00270	IPP	Yes	01OCT1999	7	0.00
030.001.00275	IPP	Yes	01MAR2000	2	3.00
030.001.00277	IPP	Yes	01FEB2000	3	2.00
030.001.00278	IPP	Yes	01MAR2000	2	3.00
030.001.00285	IPP	Yes	01NOV1999	6	1.00
030.001.00287	IPP	Yes	01FEB2000	3	3.00
030.001.00291	IPP	Yes	01NOV1999	6	2.00
030.001.00295		Yes	01MAR2000	2	2.00
030.001.00297	IPP	Yes	01APR1999	13	0.00
030.001.00300	IPP	Yes	01DEC1999	5	1.00
030.001.00303	IPP	Yes	01FEB2000	3	1.00

*Popn=Population (I=Intent-to-Treat, PP=Per Protocol)
Blank = UNKNOWN entered on CRF
** Time since last attack= Date of screening visit - Date of last attack
Tafenoquine = Tafenoquine 200mg, Mefloquine = Mefloquine 250mg

SB252263 - 030

Listing B.12

History Of Malaria

Treatment : Tafenoquine

Subject Number	Popn*	History of Malaria	Date of last Attack	Time Since Last Attack**	No. attacks in last 6 months
030.001.00001	IPP	Yes	01APR2000	1	4.00
030.001.00004	IPP	Yes	01JAN2000	4	1.00
030.001.00006	IPP	Yes	01JAN2000	4	1.00
030.001.00009	IPP	Yes	01NOV1999	6	2.00
030.001.00014	IPP	Yes	01JAN2000	4	3.00
030.001.00016	IPP	Yes	01MAR2000	2	4.00
030.001.00017	IPP	Yes	01MAR2000	2	6.00
030.001.00024	IPP	Yes	01JAN2000	4	2.00
030.001.00025	IPP	Yes	01MAR2000	2	2.00
030.001.00028	IPP	Yes	01DEC1998	17	0.00
030.001.00029	IPP	Yes	01MAR2000	2	2.00
030.001.00030	IPP	Yes	01DEC1999	5	2.00
030.001.00040	IPP	Yes	01FEB2000	3	1.00
030.001.00042	IPP	Yes	01MAY1999	12	0.00
030.001.00043	IPP	Yes	01DEC1999	5	1.00
030.001.00048	IPP	Yes	01MAR2000	2	1.00
030.001.00050	IPP	No			
030.001.00051	IPP	Yes	01AUG1999	9	2.00
030.001.00058	IPP	Yes	01JAN2000	4	1.00
030.001.00060	IPP	Yes	01APR2000	1	6.00
030.001.00061	IPP	Yes	01FEB2000	3	2.00

*Popn=Population (I=Intent-to-Treat, PP=Per Protocol)
Blank = UNKNOWN entered on CRF
** Time since last attack= Date of screening visit - Date of last attack
Tafenoquine = Tafenoquine 200mg, Mefloquine = Mefloquine 250mg

SB252263 - 030

Listing B.12

History Of Malaria

Treatment : Tafenoquine

Subject Number	Popn*	History of Malaria	Date of last Attack	Time Since Last Attack**	No. attacks in last 6 months
030.001.00065	IPP	Yes	01JAN2000	4	1.00
030.001.00068	IPP	Yes	01APR2000	1	2.00
030.001.00070	IPP	Yes	01DEC1999	5	1.00
030.001.00077	IPP	No			
030.001.00079	IPP	Yes	01APR2000	1	1.00
030.001.00082		Yes	01OCT1999	7	0.00
030.001.00084	IPP	Yes	01MAR2000	2	1.00
030.001.00085	IPP	Yes	01MAR2000	2	1.00
030.001.00086	IPP	Yes	01NOV1999	6	1.00
030.001.00093	IPP	Yes			0.00
030.001.00094	I	Yes	01MAR2000	2	1.00
030.001.00100	IPP	Yes	01JAN2000	4	4.00
030.001.00102	IPP	Yes	01MAR2000	2	3.00
030.001.00104	IPP	Yes	01JAN2000	4	1.00
030.001.00107	IPP	Yes	01MAR2000	2	1.00
030.001.00109	IPP	Yes	01AUG1999	9	0.00
030.001.00111	IPP	Yes	01OCT1999	7	0.00
030.001.00112	IPP	Yes	01DEC1999	5	1.00
030.001.00118	IPP	Yes	01FEB2000	3	5.00
030.001.00122	IPP	Yes	01JAN2000	4	10.00
030.001.00126	IPP	Yes	01MAR2000	2	3.00

*Popn=Population (I=Intent-to-Treat, PP=Per Protocol)
Blank = UNKNOWN entered on CRF
** Time since last attack= Date of screening visit - Date of last attack
Tafenoquine = Tafenoquine 200mg, Mefloquine = Mefloquine 250mg

SB252263 - 030

Listing B.12

History Of Malaria

Treatment : Tafenoquine

Subject Number	Popn*	History of Malaria	Date of last Attack	Time Since Last Attack**	No. attacks in last 6 months
030.001.00128	IPP	Yes	01JAN2000	4	2.00
030.001.00129	IPP	Yes	01JAN2000	4	1.00
030.001.00133	IPP	Yes	01FEB2000	3	1.00
030.001.00135	IPP	Yes	01AUG1999	9	0.00
030.001.00138	IPP	Yes	01JUL1994	70	0.00
030.001.00139	IPP	Yes			0.00
030.001.00148	IPP	Yes	01MAY1999	12	0.00
030.001.00149	IPP	Yes	01SEP1999	8	0.00
030.001.00150	IPP	Yes	01APR2000	1	1.00
030.001.00155	IPP	Yes	01MAR2000	2	1.00
030.001.00157	IPP	Yes	01JAN2000	4	2.00
030.001.00158	IPP	Yes	01DEC1999	5	7.00
030.001.00159	IPP	Yes	01JUL1998	22	0.00
030.001.00161	IPP	Yes	01FEB2000	3	2.00
030.001.00171	IPP	Yes	01JAN2000	4	3.00
030.001.00173	I	Yes	01MAR2000	2	2.00
030.001.00176	IPP	Yes	01MAR1999	14	0.00
030.001.00179	I	Yes	01DEC1999	5	2.00
030.001.00184	IPP	Yes	01DEC1999	5	1.00
030.001.00186	IPP	Yes	01MAR2000	2	2.00
030.001.00187	I	Yes	01FEB2000	3	1.00

*Popn=Population (I=Intent-to-Treat, PP=Per Protocol)
Blank = UNKNOWN entered on CRF
** Time since last attack= Date of screening visit - Date of last attack
Tafenoquine = Tafenoquine 200mg, Mefloquine = Mefloquine 250mg

SB252263 - 030

Listing B.12

History Of Malaria

Treatment : Tafenoquine

Subject Number	Popn*	History of Malaria	Date of last Attack	Time Since Last Attack**	No. attacks in last 6 months
030.001.00190	IPP	Yes	01JAN2000	4	2.00
030.001.00194	IPP	Yes	01DEC1999	5	1.00
030.001.00195	IPP	Yes	01AUG1998	21	0.00
030.001.00201	IPP	Yes	01NOV1999	6	1.00
030.001.00203	IPP	Yes	01MAR2000	2	2.00
030.001.00205	IPP	Yes	01APR2000	1	2.00
030.001.00207	IPP	Yes	01DEC1999	5	1.00
030.001.00209	IPP	Yes	01NOV1999	6	1.00
030.001.00210	IPP	Yes	01JAN2000	4	1.00
030.001.00217	IPP	Yes	01MAR2000	2	5.00
030.001.00218	IPP	Yes	01MAR2000	2	3.00
030.001.00220	IPP	Yes	01APR2000	1	2.00
030.001.00228	IPP	Yes	01MAY1998	24	0.00
030.001.00230	IPP	Yes	01MAR2000	2	2.00
030.001.00231	IPP	Yes	01APR2000	1	4.00
030.001.00233	IPP	Yes	01NOV1999	6	1.00
030.001.00240	IPP	Yes	01MAR2000	2	1.00
030.001.00243	IPP	Yes	01MAR2000	2	3.00
030.001.00246	IPP	Yes	01DEC1999	5	1.00
030.001.00249	IPP	Yes	01JAN1998	28	0.00
030.001.00252	IPP	Yes	01NOV1998	18	0.00

*Popn=Population (I=Intent-to-Treat, PP=Per Protocol)
Blank = UNKNOWN entered on CRF
** Time since last attack= Date of screening visit - Date of last attack
Tafenoquine = Tafenoquine 200mg, Mefloquine = Mefloquine 250mg

SB252263 - 030

Listing B.12

History Of Malaria

Treatment : Tafenoquine

Subject Number	Popn*	History of Malaria	Date of last Attack	Time Since Last Attack**	No. attacks in last 6 months
030.001.00254	I	Yes	01DEC1999	5	1.00
030.001.00259	IPP	Yes	01DEC1999	5	1.00
030.001.00261		Yes	01MAR2000	2	5.00
030.001.00264	IPP	Yes	01APR2000	1	10.00
030.001.00266	I	Yes	01JAN1999	16	0.00
030.001.00267	IPP	Yes	01FEB2000	3	2.00
030.001.00271	I	Yes	01MAR2000	2	2.00
030.001.00272	IPP	Yes	01OCT1999	7	0.00
030.001.00279	IPP	Yes	01APR2000	1	4.00
030.001.00282	IPP	Yes	01OCT1999	7	1.00
030.001.00284	IPP	Yes	01JAN2000	4	3.00
030.001.00288	IPP	Yes	01MAR2000	2	1.00
030.001.00293	IPP	Yes	01JAN2000	4	2.00
030.001.00294	IPP	Yes	01JAN2000	4	2.00
030.001.00296	IPP	Yes	01MAR2000	2	4.00
030.001.00298	IPP	Yes	01JAN2000	4	1.00
030.001.00301	IPP	Yes	01MAR2000	2	5.00
030.001.00302	I	Yes	01JAN2000	4	1.00
030.001.00304	IPP	Yes	01JUL1999	10	0.00
030.001.00306	IPP	Yes	01APR2000	1	2.00

*Popn=Population (I=Intent-to-Treat, PP=Per Protocol)
Blank = UNKNOWN entered on CRF
** Time since last attack= Date of screening visit - Date of last attack
Tafenoquine = Tafenoquine 200mg, Mefloquine = Mefloquine 250mg

SB252263 - 030

Listing B.12

History Of Malaria

Treatment : Mefloquine

Subject Number	Popn*	History of Malaria	Date of last Attack	Time Since Last Attack**	No. attacks in last 6 months
030.001.00003	I	Yes	01MAR2000	2	1.00
030.001.00007	IPP	No			
030.001.00008	IPP	Yes	01MAR2000	2	2.00
030.001.00010	IPP	Yes	01MAR2000	2	1.00
030.001.00013	IPP	Yes	02FEB2000	3	1.00
030.001.00018	IPP	Yes	01APR2000	1	2.00
030.001.00021	IPP	Yes	01APR2000	1	4.00
030.001.00023	I	Yes	01AUG1999	9	0.00
030.001.00031	IPP	Yes	01DEC1999	5	1.00
030.001.00034	IPP	Yes	01JUL1999	10	0.00
030.001.00035		Yes	01APR1999	13	0.00
030.001.00036	I	Yes	01SEP1999	8	3.00
030.001.00038	IPP	Yes	01JUN1999	11	0.00
030.001.00045	IPP	Yes	01NOV1995	54	0.00
030.001.00046	IPP	Yes			0.00
030.001.00047	IPP	Yes	01MAR2000	2	6.00
030.001.00049	IPP	Yes	01JUN1999	11	0.00
030.001.00052	IPP	Yes	01FEB2000	3	1.00
030.001.00053	IPP	Yes	01DEC1999	5	1.00
030.001.00054	IPP	Yes			0.00
030.001.00062	IPP	Yes	01APR2000	1	2.00

*Popn=Population (I=Intent-to-Treat, PP=Per Protocol)
Blank = UNKNOWN entered on CRF
** Time since last attack= Date of screening visit - Date of last attack
Tafenoquine = Tafenoquine 200mg, Mefloquine = Mefloquine 250mg

SB252263 - 030

Listing B.12

History Of Malaria

Treatment : Mefloquine

Subject Number	Popn*	History of Malaria	Date of last Attack	Time Since Last Attack**	No. attacks in last 6 months
030.001.00066	IPP	Yes	01AUG1999	9	0.00
030.001.00069	I	Yes	01FEB2000	3	2.00
030.001.00071	IPP	Yes	01AUG1999	9	0.00
030.001.00073	IPP	Yes	01DEC1999	5	1.00
030.001.00074	IPP	Yes	01MAR2000	2	3.00
030.001.00075	IPP	Yes	01NOV1999	6	1.00
030.001.00083	IPP	Yes	01FEB2000	3	2.00
030.001.00089	IPP	Yes			0.00
030.001.00091	IPP	Yes	01MAR2000	2	1.00
030.001.00095	IPP	Yes	01SEP1997	32	0.00
030.001.00096	IPP	Yes	01MAR1996	50	0.00
030.001.00097	IPP	Yes	01JAN2000	4	3.00
030.001.00098	IPP	Yes	01APR2000	1	1.00
030.001.00099	IPP	Yes	01SEP1999	8	0.00
030.001.00105	IPP	Yes	01JAN2000	4	2.00
030.001.00110	IPP	Yes	01MAR2000	2	2.00
030.001.00113	IPP	Yes	01FEB2000	3	1.00
030.001.00117	IPP	Yes	01JAN1999	16	0.00
030.001.00120	IPP	Yes	01JAN1996	52	0.00
030.001.00121	IPP	Yes	01MAR2000	2	5.00
030.001.00123	I	Yes	01JUN1999	11	0.00

*Popn=Population (I=Intent-to-Treat, PP=Per Protocol)
Blank = UNKNOWN entered on CRF
** Time since last attack= Date of screening visit - Date of last attack
Tafenoquine = Tafenoquine 200mg, Mefloquine = Mefloquine 250mg

SB252263 - 030

Listing B.12

History Of Malaria

Treatment : Mefloquine

Subject Number	Popn*	History of Malaria	Date of last Attack	Time Since Last Attack**	No. attacks in last 6 months
030.001.00130	IPP	Yes	01JUN1999	11	0.00
030.001.00132	IPP	Yes	01APR2000	1	2.00
030.001.00134	IPP	Yes			
030.001.00142	IPP	Yes	01JAN2000	4	1.00
030.001.00143	IPP	Yes	01FEB2000	3	5.00
030.001.00144	IPP	Yes	01DEC1999	5	2.00
030.001.00147	IPP	Yes	01NOV1999	6	1.00
030.001.00153	IPP	Yes	01FEB1999	15	0.00
030.001.00156	IPP	Yes	01FEB2000	3	1.00
030.001.00163	IPP	Yes	01MAR2000	2	3.00
030.001.00165	IPP	Yes	01JAN2000	4	2.00
030.001.00167	IPP	Yes	01DEC1999	5	1.00
030.001.00168	IPP	Yes	01FEB2000	3	4.00
030.001.00170	IPP	Yes	01AUG1999	9	0.00
030.001.00177	IPP	Yes	01DEC1999	5	1.00
030.001.00178	I	Yes	01MAR2000	2	1.00
030.001.00180	IPP	Yes	01FEB2000	3	2.00
030.001.00181	IPP	Yes	01NOV1998	18	0.00
030.001.00185	IPP	Yes	01DEC1999	5	1.00
030.001.00188	IPP	Yes	01JAN2000	4	1.00
030.001.00189	I	Yes	01AUG1999	9	3.00

*Popn=Population (I=Intent-to-Treat, PP=Per Protocol)
Blank = UNKNOWN entered on CRF
** Time since last attack= Date of screening visit - Date of last attack
Tafenoquine = Tafenoquine 200mg, Mefloquine = Mefloquine 250mg

SB252263 - 030

Listing B.12

History Of Malaria

Treatment : Mefloquine

Subject Number	Popn*	History of Malaria	Date of last Attack	Time Since Last Attack**	No. attacks in last 6 months
030.001.00196	IPP	Yes	01JUN1999	11	0.00
030.001.00198	IPP	Yes	01JAN2000	4	1.00
030.001.00199	IPP	Yes	01MAR2000	2	2.00
030.001.00200	IPP	Yes	01NOV1999	6	1.00
030.001.00206	I	Yes	01FEB2000	3	3.00
030.001.00213	IPP	Yes	01DEC1999	5	3.00
030.001.00214	IPP	Yes	01DEC1999	5	1.00
030.001.00216	IPP	Yes	01APR1999	13	0.00
030.001.00221	IPP	Yes	01DEC1999	5	3.00
030.001.00223	IPP	Yes	01JAN1990	124	0.00
030.001.00224	IPP	Yes	01MAR2000	2	4.00
030.001.00227	IPP	Yes	01NOV1999	6	2.00
030.001.00232	IPP	Yes	01APR1999	13	0.00
030.001.00235	IPP	Yes	01AUG1999	9	0.00
030.001.00237	IPP	Yes	01JAN2000	4	1.00
030.001.00239	IPP	Yes	01JAN2000	4	3.00
030.001.00241	IPP	Yes	01APR2000	1	1.00
030.001.00244	IPP	Yes	01DEC1999	5	1.00
030.001.00245	IPP	Yes	01MAR2000	2	1.00
030.001.00250		Yes	01FEB2000	3	2.00
030.001.00255	IPP	Yes	01JAN1999	16	0.00

*Popn=Population (I=Intent-to-Treat, PP=Per Protocol)
Blank = UNKNOWN entered on CRF
** Time since last attack= Date of screening visit - Date of last attack
Tafenoquine = Tafenoquine 200mg, Mefloquine = Mefloquine 250mg

SB252263 - 030

Listing B.12

History Of Malaria

Treatment : Mefloquine

Subject Number	Popn*	History of Malaria	Date of last Attack	Time Since Last Attack**	No. attacks in last 6 months
030.001.00258	IPP	Yes	01JAN1998	28	0.00
030.001.00262	IPP	Yes	01MAR2000	2	3.00
030.001.00263	IPP	Yes	01DEC1999	5	1.00
030.001.00265	IPP	Yes	01JAN1997	40	0.00
030.001.00273	IPP	Yes	01NOV1999	6	1.00
030.001.00274	IPP	Yes	01DEC1999	5	1.00
030.001.00276	IPP	Yes	01MAR1999	14	0.00
030.001.00280	IPP	Yes	01MAR2000	2	3.00
030.001.00281	IPP	Yes	01DEC1999	5	2.00
030.001.00283	IPP	Yes	01DEC1999	5	1.00
030.001.00286	IPP	Yes	01JAN2000	4	2.00
030.001.00289	IPP	Yes	01MAR2000	2	1.00
030.001.00290	IPP	Yes	01FEB2000	3	3.00
030.001.00292	IPP	Yes	01OCT1998	19	0.00
030.001.00299	IPP	Yes	01FEB2000	3	1.00
030.001.00305	IPP	Yes	01JUL1994	70	0.00

*Popn=Population (I=Intent-to-Treat, PP=Per Protocol)
Blank = UNKNOWN entered on CRF
** Time since last attack= Date of screening visit - Date of last attack
Tafenoquine = Tafenoquine 200mg, Mefloquine = Mefloquine 250mg

Listing B.13

SB252263 - 030

Listing B.13

Baseline Signs And Symptoms

Treatment : Placebo

Subject Number	Popn*	Verbatim Term / PREFERRED TERM	Primary Body System	Onset Date Time	End Date Time	Relative Day of Onset (End)**	Dur+	Out++	Epi#	Max int##	Rel^	Corr ther^^	Wd AE$
030.001.00002	IPP	ARTHRALGIAS / ARTHRALGIA	MUSCULOSKELETAL SYSTEM	03MAY2000	04MAY2000	-5 (-4)	2 DAYS	RESO	CON	MIL	NOT	No	No
		HEADACHE / HEADACHE	CENTRAL AND PERIPHERAL NERVOUS SYSTEM	03MAY2000	04MAY2000	-5 (-4)	2 DAYS	RESO	CON	MIL	NOT	No	No
030.001.00005	IPP	HEADACHE / HEADACHE	CENTRAL AND PERIPHERAL NERVOUS SYSTEM	03MAY2000	04MAY2000	-5 (-4)	2 DAYS	RESO	CON	MIL	NOT	No	No
030.001.00022	IPP	ARTHRALGIAS / ARTHRALGIA	MUSCULOSKELETAL SYSTEM	29APR2000	04MAY2000	-9 (-4)	6 DAYS	RESO	CON	MIL	NOT	Yes	No
		HEADACHE / HEADACHE	CENTRAL AND PERIPHERAL NERVOUS SYSTEM	03MAY2000	04MAY2000	-5 (-4)	2 DAYS	RESO	CON	MIL	NOT	No	No
030.001.00032	IPP	COUGH / COUGHING	RESPIRATORY SYSTEM	03MAY2000	22MAY2000	-5 (14)	20 DAYS	RESO	3	MIL	NOT	Yes	No
030.001.00041	IPP	COUGH / COUGHING	RESPIRATORY SYSTEM	03MAY2000	15MAY2000	-5 (7)	13 DAYS	RESO	CON	MOD	NOT	No	No
		ITCHY EYES / CONJUNCTIVITIS	VISION	03MAY2000	06MAY2000	-5 (-2)	4 DAYS	RESO	CON	MIL	NOT	No	No

*Popn=Population (I=Intent-to-Treat, PP=Per Protocol)
**No. of days relative to start of prophylactic medication
+Dur=Duration : CONT=Continuing
++Out=Outcome : RESO=Resolved, ONGO=Ongoing
#Epi=Episode: n=No. of Episodes, CON= Constant, ITE= Intermittent
##Max Int = Intensity (Maximum): MIL=Mild, MOD=Moderate, SEV=Severe
^REL=Relationship To Study Procedures: NOT=Not related, UNL= Unlikely, SUS= Suspected, PRB=Probable
^^Corr Ther= Corrective Therapy
$WD AE: Withdrawn Due To A Specific Event
Tafenoquine = Tafenoquine 200mg, Mefloquine = Mefloquine 250mg

SB252263 - 030

Listing B.13

Baseline Signs And Symptoms

Treatment : Placebo

Subject Number	Popn*	Verbatim Term / PREFERRED TERM	Primary Body System	Onset Date Time	End Date Time	Relative Day of Onset (End)**	Dur+	Out++	Epi#	Max int##	Rel^	Corr ther^^	Wd AE$
030.001.00044	IPP	URTI / UPPER RESP TRACT INFECTION	RESISTANCE MECHANISM	06MAY2000	08MAY2000	-2 (0)	3 DAYS	RESO	CON	MIL	SUS	Yes	No
030.001.00056	I	BACKACHE / BACK PAIN	MUSCULOSKELETAL SYSTEM	04MAY2000	08MAY2000	-4 (0)	5 DAYS	RESO	CON	MIL	NOT	Yes	No
		STOMACH ACHE / ABDOMINAL PAIN	GASTROINTESTINAL SYSTEM	06MAY2000	08MAY2000	-2 (0)	3 DAYS	RESO	CON	MIL	UNL	No	No
030.001.00057	IPP	GENERALIZED PRURITIS / PRURITUS	SKIN AND APPENDAGES	04MAY2000	08MAY2000	-4 (0)	5 DAYS	RESO	CON	MOD	PRB	No	No
		HEADACHE / HEADACHE	CENTRAL AND PERIPHERAL NERVOUS SYSTEM	03MAY2000	04MAY2000	-5 (-4)	2 DAYS	RESO	CON	MIL	NOT	No	No
030.001.00063	IPP	BRONCHITIS/ASTHMA / BRONCHITIS	RESPIRATORY SYSTEM	05MAY2000	15MAY2000	-3 (7)	11 DAYS	RESO	CON	MIL	NOT	Yes	No
030.001.00064	IPP	SCROTAL CELLULITIS / CELLULITIS	BODY AS A WHOLE GENERAL	30APR2000	08MAY2000	-8 (0)	9 DAYS	RESO	CON	MOD	NOT	Yes	No

*Popn=Population (I=Intent-to-Treat, PP=Per Protocol)
**No. of days relative to start of prophylactic medication
+Dur=Duration : CONT=Continuing
++Out=Outcome : RESO=Resolved, ONGO=Ongoing
#Epi=Episode: n=No. of Episodes, CON= Constant, ITE= Intermittent
##Max Int = Intensity (Maximum): MIL=Mild, MOD=Moderate, SEV=Severe
^REL=Relationship To Study Procedures: NOT=Not related, UNL= Unlikely, SUS= Suspected, PRB=Probable
^^Corr Ther= Corrective Therapy
$WD AE: Withdrawn Due To A Specific Event
Tafenoquine = Tafenoquine 200mg, Mefloquine = Mefloquine 250mg

SB252263 - 030

Listing B.13

Baseline Signs And Symptoms

Treatment : Placebo

Subject Number	Popn*	Verbatim Term / PREFERRED TERM	Primary Body System	Onset Date Time	End Date Time	Relative Day of Onset (End)**	Dur+	Out++	Epi#	Max int##	Rel^	Corr ther^^	Wd AE$
030.001.00067	IPP	BACKACHE / BACK PAIN	MUSCULOSKELETAL SYSTEM	04MAY2000	05MAY2000	-4 (-3)	2 DAYS	RESO	CON	MIL	NOT	No	No
		COUGH / COUGHING	RESPIRATORY SYSTEM	05MAY2000	08MAY2000	-3 (0)	4 DAYS	RESO	CON	MIL	UNL	No	No
030.001.00072	IPP	BACKACHE / BACK PAIN	MUSCULOSKELETAL SYSTEM	05MAY2000	19MAY2000	-3 (11)	15 DAYS	RESO	4	MIL	NOT	Yes	No
030.001.00076	IPP	DIZZINESS / DIZZINESS	CENTRAL AND PERIPHERAL NERVOUS SYSTEM	06MAY2000	08MAY2000	-2 (0)	3 DAYS	RESO	CON	MIL	SUS	No	No
		HEADACHE / HEADACHE	CENTRAL AND PERIPHERAL NERVOUS SYSTEM	06MAY2000	08MAY2000	-2 (0)	3 DAYS	RESO	CON	MIL	SUS	No	No
030.001.00080	IPP	COLD / UPPER RESP TRACT INFECTION	RESISTANCE MECHANISM	03MAY2000	11MAY2000	-5 (3)	9 DAYS	RESO	CON	MIL	NOT	No	No
		CUT TOE / INJURY	BODY AS A WHOLE GENERAL	06MAY2000	20MAY2000	-2 (12)	15 DAYS	RESO	CON	MIL	NOT	No	No
030.001.00087	I	MYALGIA / MYALGIA	MUSCULOSKELETAL SYSTEM	06MAY2000	08MAY2000	-2 (0)	3 DAYS	RESO	CON	MIL	NOT	Yes	No

*Popn=Population (I=Intent-to-Treat, PP=Per Protocol)
**No. of days relative to start of prophylactic medication
+Dur=Duration : CONT=Continuing
++Out=Outcome : RESO=Resolved, ONGO=Ongoing
#Epi=Episode: n=No. of Episodes, CON= Constant, ITE= Intermittent
##Max Int = Intensity (Maximum): MIL=Mild, MOD=Moderate, SEV=Severe
^REL=Relationship To Study Procedures: NOT=Not related, UNL= Unlikely, SUS= Suspected, PRB=Probable
^^Corr Ther= Corrective Therapy
$WD AE: Withdrawn Due To A Specific Event
Tafenoquine = Tafenoquine 200mg, Mefloquine = Mefloquine 250mg

SB252263 - 030

Listing B.13

Baseline Signs And Symptoms

Treatment : Placebo

Subject Number	Popn*	Verbatim Term / PREFERRED TERM	Primary Body System	Onset Date Time	End Date Time	Relative Day of Onset (End)**	Dur+	Out++	Epi#	Max int##	Rel^	Corr ther^^	Wd AE$
030.001.00088	IPP	COUGH / COUGHING	RESPIRATORY SYSTEM	04MAY2000	10MAY2000	-4 (2)	7 DAYS	RESO	CON	MIL	NOT	No	No
030.001.00090	IPP	COUGH / COUGHING	RESPIRATORY SYSTEM	04MAY2000		-4 ()	CONT	ONGO	CON	MIL	UNL	Yes	No
		HEADACHE / HEADACHE	CENTRAL AND PERIPHERAL NERVOUS SYSTEM	03MAY2000	04MAY2000	-5 (-4)	2 DAYS	RESO	CON	MIL	NOT	Yes	No
030.001.00101	IPP	CHEST PAIN / CHEST PAIN	BODY AS A WHOLE GENERAL	05MAY2000	06MAY2000	-3 (-2)	2 DAYS	RESO	CON	MIL	UNL	No	No
		RECURRENT ABDOMINAL PAIN / ABDOMINAL PAIN	GASTROINTESTIN AL SYSTEM	03MAY2000	04SEP2000	-5 (119)	125 DAYS	RESO	6	MIL	NOT	Yes	No
030.001.00115	IPP	COUGH / COUGHING	RESPIRATORY SYSTEM	04MAY2000	02AUG2000	-4 (86)	91 DAYS	RESO	CON	MIL	NOT	Yes	No
030.001.00124	IPP	ARTHRALGIA / ARTHRALGIA	MUSCULOSKELETA L SYSTEM	05MAY2000	06MAY2000	-4 (-3)	2 DAYS	RESO	CON	MIL	NOT	No	No

*Popn=Population (I=Intent-to-Treat, PP=Per Protocol)
**No. of days relative to start of prophylactic medication
+Dur=Duration : CONT=Continuing
++Out=Outcome : RESO=Resolved, ONGO=Ongoing
#Epi=Episode: n=No. of Episodes, CON= Constant, ITE= Intermittent
##Max Int = Intensity (Maximum): MIL=Mild, MOD=Moderate, SEV=Severe
^REL=Relationship To Study Procedures: NOT=Not related, UNL= Unlikely, SUS= Suspected, PRB=Probable
^^Corr Ther= Corrective Therapy
$WD AE: Withdrawn Due To A Specific Event
Tafenoquine = Tafenoquine 200mg, Mefloquine = Mefloquine 250mg

SB252263 - 030

Listing B.13

Baseline Signs And Symptoms

Treatment : Placebo

Subject Number	Popn*	Verbatim Term / PREFERRED TERM	Primary Body System	Onset Date Time	End Date Time	Relative Day of Onset (End)**	Dur+	Out++	Epi#	Max int##	Rel^	Corr ther^^	Wd AE$
030.001.00127	I	HEADACHE / HEADACHE	CENTRAL AND PERIPHERAL NERVOUS SYSTEM	02MAY2000	07JUN2000	-7 (29)	37 DAYS	RESO	ITE	MIL	NOT	Yes	No
030.001.00131	IPP	PRURITUS / PRURITUS	SKIN AND APPENDAGES	06MAY2000	26JUN2000	-3 (48)	52 DAYS	RESO	2	MIL	UNL	No	No
030.001.00140	IPP	COUGH / COUGHING	RESPIRATORY SYSTEM	06MAY2000	07MAY2000	-3 (-2)	2 DAYS	RESO	CON	MIL	NOT	No	No
030.001.00141	IPP	HEADACHE / HEADACHE	CENTRAL AND PERIPHERAL NERVOUS SYSTEM	06MAY2000	07MAY2000	-3 (-2)	2 DAYS	RESO	CON	MIL	UNL	No	No
030.001.00145	IPP	DYSPEPSIA / DYSPEPSIA	GASTROINTESTIN AL SYSTEM	04MAY2000	11MAY2000	-5 (2)	8 DAYS	RESO	CON	MIL	NOT	Yes	No
030.001.00152	IPP	VIRAL SYNDROME / INFECTION VIRAL	RESISTANCE MECHANISM	04MAY2000	05MAY2000	-5 (-4)	2 DAYS	RESO	ITE	MIL	NOT	Yes	No
030.001.00160	I	PRURITIS / PRURITUS	SKIN AND APPENDAGES	05MAY2000		-4 ()	CONT	ONGO	CON	MIL	NOT	Yes	No

*Popn=Population (I=Intent-to-Treat, PP=Per Protocol)
**No. of days relative to start of prophylactic medication
+Dur=Duration : CONT=Continuing
++Out=Outcome : RESO=Resolved, ONGO=Ongoing
#Epi=Episode: n=No. of Episodes, CON= Constant, ITE= Intermittent
##Max Int = Intensity (Maximum): MIL=Mild, MOD=Moderate, SEV=Severe
^REL=Relationship To Study Procedures: NOT=Not related, UNL= Unlikely, SUS= Suspected, PRB=Probable
^^Corr Ther= Corrective Therapy
$WD AE: Withdrawn Due To A Specific Event
Tafenoquine = Tafenoquine 200mg, Mefloquine = Mefloquine 250mg

SB252263 - 030

Listing B.13

Baseline Signs And Symptoms

Treatment : Placebo

Subject Number	Popn*	Verbatim Term / PREFERRED TERM	Primary Body System	Onset Date Time	End Date Time	Relative Day of Onset (End)**	Dur+	Out++	Epi#	Max int##	Rel^	Corr ther^^	Wd AE$
030.001.00164	IPP	HEADACHE / HEADACHE	CENTRAL AND PERIPHERAL NERVOUS SYSTEM	05MAY2000	09MAY2000	-4 (0)	5 DAYS	RESO	2	MIL	SUS	Yes	No
030.001.00166	IPP	UPPER RESPIRATORY INFECTION / UPPER RESP TRACT INFECTION	RESISTANCE MECHANISM	06MAY2000	07MAY2000	-3 (-2)	2 DAYS	RESO	CON	MIL	NOT	Yes	No
030.001.00169	IPP	BRONCHITIS / BRONCHITIS	RESPIRATORY SYSTEM	04MAY2000	07MAY2000	-5 (-2)	4 DAYS	RESO	CON	MIL	NOT	Yes	No
		HEADACHE / HEADACHE	CENTRAL AND PERIPHERAL NERVOUS SYSTEM	05MAY2000	06MAY2000	-4 (-3)	2 DAYS	RESO	CON	MIL	NOT	Yes	No
030.001.00172	IPP	BACKACHE / BACK PAIN	MUSCULOSKELETA L SYSTEM	05MAY2000	07MAY2000	-4 (-2)	3 DAYS	RESO	CON	MIL	NOT	Yes	No
		HEADACHE / HEADACHE	CENTRAL AND PERIPHERAL NERVOUS SYSTEM	05MAY2000	07MAY2000	-4 (-2)	3 DAYS	RESO	CON	MIL	NOT	Yes	No

*Popn=Population (I=Intent-to-Treat, PP=Per Protocol)
**No. of days relative to start of prophylactic medication
+Dur=Duration : CONT=Continuing
++Out=Outcome : RESO=Resolved, ONGO=Ongoing
#Epi=Episode: n=No. of Episodes, CON= Constant, ITE= Intermittent
##Max Int = Intensity (Maximum): MIL=Mild, MOD=Moderate, SEV=Severe
^REL=Relationship To Study Procedures: NOT=Not related, UNL= Unlikely, SUS= Suspected, PRB=Probable
^^Corr Ther= Corrective Therapy
$WD AE: Withdrawn Due To A Specific Event
Tafenoquine = Tafenoquine 200mg, Mefloquine = Mefloquine 250mg

SB252263 - 030

Listing B.13

Baseline Signs And Symptoms

Treatment : Placebo

Subject Number	Popn*	Verbatim Term / PREFERRED TERM	Primary Body System	Onset Date Time	End Date Time	Relative Day of Onset (End)**	Dur+	Out++	Epi#	Max int##	Rel^	Corr ther^^	Wd AE$
030.001.00175	IPP	ABDOMINAL PAIN / ABDOMINAL PAIN	GASTROINTESTINAL SYSTEM	04MAY2000	11MAY2000	-5 (2)	8 DAYS	RESO	CON	MIL	NOT	Yes	No
		COUGH / COUGHING	RESPIRATORY SYSTEM	04MAY2000	13MAY2000	-5 (4)	10 DAYS	RESO	CON	MIL	NOT	No	No
030.001.00183	IPP	PRURITIS / PRURITUS	SKIN AND APPENDAGES	05MAY2000	06MAY2000	-4 (-3)	2 DAYS	RESO	CON	MIL	PRB	No	No
		VIRAL SYNDROME / INFECTION VIRAL	RESISTANCE MECHANISM	05MAY2000	14MAY2000	-4 (5)	10 DAYS	RESO	CON	MIL	UNL	Yes	No
030.001.00193	IPP	BRONCHITIS / BRONCHITIS	RESPIRATORY SYSTEM	04MAY2000	22MAY2000	-5 (13)	19 DAYS	RESO	4	MIL	NOT	Yes	No
		COUGH / COUGHING	RESPIRATORY SYSTEM	07MAY2000	13MAY2000	-2 (4)	7 DAYS	RESO	CON	MIL	NOT	No	No
030.001.00197	IPP	RHINITIS / RHINITIS	RESPIRATORY SYSTEM	08MAY2000 20:00	18MAY2000	-1 (9)	11 DAYS	RESO	CON	MIL	NOT	Yes	No

*Popn=Population (I=Intent-to-Treat, PP=Per Protocol)
**No. of days relative to start of prophylactic medication
+Dur=Duration : CONT=Continuing
++Out=Outcome : RESO=Resolved, ONGO=Ongoing
#Epi=Episode: n=No. of Episodes, CON= Constant, ITE= Intermittent
##Max Int = Intensity (Maximum): MIL=Mild, MOD=Moderate, SEV=Severe
^REL=Relationship To Study Procedures: NOT=Not related, UNL= Unlikely, SUS= Suspected, PRB=Probable
^^Corr Ther= Corrective Therapy
$WD AE: Withdrawn Due To A Specific Event
Tafenoquine = Tafenoquine 200mg, Mefloquine = Mefloquine 250mg

SB252263 - 030

Listing B.13

Baseline Signs And Symptoms

Treatment : Placebo

Subject Number	Popn*	Verbatim Term / PREFERRED TERM	Primary Body System	Onset Date Time	End Date Time	Relative Day of Onset (End)**	Dur+	Out++	Epi#	Max int##	Rel^	Corr ther^^	Wd AE$
030.001.00204	I	ABDOMINAL PAIN / ABDOMINAL PAIN	GASTROINTESTIN AL SYSTEM	07MAY2000	11MAY2000	-2 (2)	5 DAYS	RESO	CON	MIL	SUS	No	No
030.001.00208	IPP	BACK ACHE / BACK PAIN	MUSCULOSKELETA L SYSTEM	08MAY2000	17MAY2000	-1 (8)	10 DAYS	RESO	ITE	MIL	NOT	Yes	No
		CHEST PAIN / CHEST PAIN	BODY AS A WHOLE GENERAL	08MAY2000	17MAY2000	-1 (8)	10 DAYS	RESO	4	MIL	NOT	Yes	No
030.001.00215	IPP	ANOREXIA / ANOREXIA	GASTROINTESTIN AL SYSTEM	07MAY2000	05AUG2000	-2 (88)	91 DAYS	RESO	2	MIL	PRB	No	No
		VIRAL SYNDROME / INFECTION VIRAL	RESISTANCE MECHANISM	04MAY2000	05MAY2000	-5 (-4)	2 DAYS	RESO	CON	MIL	NOT	No	No
030.001.00225	IPP	BRONCHITIS / BRONCHITIS	RESPIRATORY SYSTEM	06MAY2000	26MAY2000	-3 (17)	21 DAYS	RESO	CON	MIL	NOT	Yes	No
		DYSPEPSIA / DYSPEPSIA	GASTROINTESTIN AL SYSTEM	06MAY2000	13MAY2000	-3 (4)	8 DAYS	RESO	CON	MIL	NOT	Yes	No
		SORE THROAT / PHARYNGITIS	RESPIRATORY SYSTEM	03MAY2000	08MAY2000	-6 (-1)	6 DAYS	RESO	CON	MIL	NOT	No	No

*Popn=Population (I=Intent-to-Treat, PP=Per Protocol)
**No. of days relative to start of prophylactic medication
+Dur=Duration : CONT=Continuing
++Out=Outcome : RESO=Resolved, ONGO=Ongoing
#Epi=Episode: n=No. of Episodes, CON= Constant, ITE= Intermittent
##Max Int = Intensity (Maximum): MIL=Mild, MOD=Moderate, SEV=Severe
^REL=Relationship To Study Procedures: NOT=Not related, UNL= Unlikely, SUS= Suspected, PRB=Probable
^^Corr Ther= Corrective Therapy
$WD AE: Withdrawn Due To A Specific Event
Tafenoquine = Tafenoquine 200mg, Mefloquine = Mefloquine 250mg

SB252263 - 030

Listing B.13

Baseline Signs And Symptoms

Treatment : Placebo

Subject Number	Popn*	Verbatim Term / PREFERRED TERM	Primary Body System	Onset Date Time	End Date Time	Relative Day of Onset (End)**	Dur+	Out++	Epi#	Max int##	Rel^	Corr ther^^	Wd AE$
030.001.00229	IPP	DYSPEPSIA SECONDARY TO INDOLID / DYSPEPSIA	GASTROINTESTINAL SYSTEM	04MAY2000	09MAY2000	-5 (0)	6 DAYS	RESO	CON	MIL	NOT	Yes	No
030.001.00238	IPP	COUGH / URTI / UPPER RESP TRACT INFECTION	RESISTANCE MECHANISM	07MAY2000	08MAY2000	-3 (-2)	2 DAYS	RESO	CON	MIL	UNL	Yes	No
030.001.00242	IPP	CONSTIPATION / CONSTIPATION	GASTROINTESTINAL SYSTEM	08MAY2000	12MAY2000	-2 (2)	5 DAYS	RESO	CON	MIL	NOT	Yes	No
030.001.00248	IPP	RECURRENT RHINITIS / RHINITIS	RESPIRATORY SYSTEM	06MAY2000		-4 ()	CONT	ONGO	CON	MIL	NOT	No	No
030.001.00251	IPP	SORE THROAT / PHARYNGITIS	RESPIRATORY SYSTEM	05MAY2000	06MAY2000	-5 (-4)	2 DAYS	RESO	CON	MIL	NOT	No	No
030.001.00257	IPP	(MUSCULOSKELETAL PAIN) POLYARTHRALGIA / ARTHRALGIA	MUSCULOSKELETAL SYSTEM	09MAY2000	31AUG2000	-3 (111)	115 DAYS	RESO	CON	MIL	NOT	Yes	No

*Popn=Population (I=Intent-to-Treat, PP=Per Protocol)
**No. of days relative to start of prophylactic medication
+Dur=Duration : CONT=Continuing
++Out=Outcome : RESO=Resolved, ONGO=Ongoing
#Epi=Episode: n=No. of Episodes, CON= Constant, ITE= Intermittent
##Max Int = Intensity (Maximum): MIL=Mild, MOD=Moderate, SEV=Severe
^REL=Relationship To Study Procedures: NOT=Not related, UNL= Unlikely, SUS= Suspected, PRB=Probable
^^Corr Ther= Corrective Therapy
$WD AE: Withdrawn Due To A Specific Event
Tafenoquine = Tafenoquine 200mg, Mefloquine = Mefloquine 250mg

SB252263 - 030

Listing B.13

Baseline Signs And Symptoms

Treatment : Placebo

Subject Number	Popn*	Verbatim Term / PREFERRED TERM	Primary Body System	Onset Date Time	End Date Time	Relative Day of Onset (End)**	Dur+	Out++	Epi#	Max int##	Rel^	Corr ther^^	Wd AE$
030.001.00257	IPP	URI / UPPER RESP TRACT INFECTION	RESISTANCE MECHANISM	08MAY2000	10MAY2000	-4 (-2)	3 DAYS	RESO	ITE	MIL	SUS	Yes	No
030.001.00260	IPP	PRURITUS / PRURITUS	SKIN AND APPENDAGES	06MAY2000	13MAY2000	-4 (3)	8 DAYS	RESO	CON	MIL	NOT	No	No
		TINEA CORPORIS / DERMATITIS FUNGAL	SKIN AND APPENDAGES	26APR2000	26SEP2000	-14 (139)	154 DAYS	RESO	CON	MIL	NOT	Yes	No
030.001.00269	IPP	CORYZA / RHINITIS	RESPIRATORY SYSTEM	13APR2000	15APR2000	-27 (-25)	3 DAYS	RESO	CON	MIL	NOT	Yes	No
030.001.00275	IPP	ARTHRALGIA / ARTHRALGIA	MUSCULOSKELETA L SYSTEM	07MAY2000	11MAY2000	-3 (1)	5 DAYS	RESO	CON	MIL	NOT	No	No
		DYSPEPSIA / DYSPEPSIA	GASTROINTESTIN AL SYSTEM	08MAY2000	11MAY2000	-2 (1)	4 DAYS	RESO	CON	MIL	NOT	Yes	No
030.001.00287	IPP	ATHLETES FOOT / DERMATITIS FUNGAL	SKIN AND APPENDAGES	09MAY2000	20JUN2000	-2 (40)	43 DAYS	RESO	CON	MIL	NOT	Yes	No

*Popn=Population (I=Intent-to-Treat, PP=Per Protocol)
**No. of days relative to start of prophylactic medication
+Dur=Duration : CONT=Continuing
++Out=Outcome : RESO=Resolved, ONGO=Ongoing
#Epi=Episode: n=No. of Episodes, CON= Constant, ITE= Intermittent
##Max Int = Intensity (Maximum): MIL=Mild, MOD=Moderate, SEV=Severe
^REL=Relationship To Study Procedures: NOT=Not related, UNL= Unlikely, SUS= Suspected, PRB=Probable
^^Corr Ther= Corrective Therapy
$WD AE: Withdrawn Due To A Specific Event
Tafenoquine = Tafenoquine 200mg, Mefloquine = Mefloquine 250mg

SB252263 - 030

Listing B.13

Baseline Signs And Symptoms

Treatment : Placebo

Subject Number	Popn*	Verbatim Term / PREFERRED TERM	Primary Body System	Onset Date Time	End Date Time	Relative Day of Onset (End)**	Dur+	Out++	Epi#	Max int##	Rel^	Corr ther^^	Wd AE$
030.001.00287	IPP	SEXUAL DYSFUNCTION / IMPOTENCE	REPRODUCTIVE MALE	01MAY2000		-10 ()	CONT	ONGO	CON	MIL	UNL	Yes	No
030.001.00297	IPP	BACKACHE / BACK PAIN	MUSCULOSKELETAL SYSTEM	09MAY2000	11MAY2000	-2 (0)	3 DAYS	RESO	CON	MIL	NOT	No	No
030.001.00303	IPP	ENTERITIS / ENTERITIS	GASTROINTESTINAL SYSTEM	11MAY2000 13:00	20MAY2000	-1 (8)	10 DAYS	RESO	3	MIL	UNL	Yes	No

*Popn=Population (I=Intent-to-Treat, PP=Per Protocol)
**No. of days relative to start of prophylactic medication
+Dur=Duration : CONT=Continuing
++Out=Outcome : RESO=Resolved, ONGO=Ongoing
#Epi=Episode: n=No. of Episodes, CON= Constant, ITE= Intermittent
##Max Int = Intensity (Maximum): MIL=Mild, MOD=Moderate, SEV=Severe
^REL=Relationship To Study Procedures: NOT=Not related, UNL= Unlikely, SUS= Suspected, PRB=Probable
^^Corr Ther= Corrective Therapy
$WD AE: Withdrawn Due To A Specific Event
Tafenoquine = Tafenoquine 200mg, Mefloquine = Mefloquine 250mg

SB252263 - 030

Listing B.13

Baseline Signs And Symptoms

Treatment : Tafenoquine

Subject Number	Popn*	Verbatim Term / PREFERRED TERM	Primary Body System	Onset Date Time	End Date Time	Relative Day of Onset (End)**	Dur+	Out++	Epi#	Max int##	Rel^	Corr ther^^	Wd AE$
030.001.00004	IPP	SPRAIN LEFT ANKLE JOINT / INJURY	BODY AS A WHOLE GENERAL	02MAY2000	19MAY2000	-6 (11)	18 DAYS	RESO	CON	MIL	NOT	Yes	No
030.001.00014	IPP	HEADACHE / HEADACHE	CENTRAL AND PERIPHERAL NERVOUS SYSTEM	03MAY2000	04MAY2000	-5 (-4)	2 DAYS	RESO	CON	MIL	NOT	No	No
		MYALGIA / MYALGIA	MUSCULOSKELETAL SYSTEM	03MAY2000	04MAY2000	-5 (-4)	2 DAYS	RESO	CON	MIL	NOT	No	No
030.001.00016	IPP	HEADACHE / HEADACHE	CENTRAL AND PERIPHERAL NERVOUS SYSTEM	03MAY2000	04MAY2000	-5 (-4)	2 DAYS	RESO	CON	MIL	NOT	Yes	No
		JOINT PAIN / ARTHRALGIA	MUSCULOSKELETAL SYSTEM	03MAY2000	04MAY2000	-5 (-4)	2 DAYS	RESO	CON	MIL	NOT	No	No
030.001.00024	IPP	HEADACHE / HEADACHE	CENTRAL AND PERIPHERAL NERVOUS SYSTEM	03MAY2000	04MAY2000	-5 (-4)	2 DAYS	RESO	CON	MIL	NOT	No	No
030.001.00028	IPP	HEADACHE / HEADACHE	CENTRAL AND PERIPHERAL NERVOUS SYSTEM	03MAY2000	04MAY2000	-5 (-4)	2 DAYS	RESO	CON	MIL	NOT	Yes	No

*Popn=Population (I=Intent-to-Treat, PP=Per Protocol)
**No. of days relative to start of prophylactic medication
+Dur=Duration : CONT=Continuing
++Out=Outcome : RESO=Resolved, ONGO=Ongoing
#Epi=Episode: n=No. of Episodes, CON= Constant, ITE= Intermittent
##Max Int = Intensity (Maximum): MIL=Mild, MOD=Moderate, SEV=Severe
^REL=Relationship To Study Procedures: NOT=Not related, UNL= Unlikely, SUS= Suspected, PRB=Probable
^^Corr Ther= Corrective Therapy
$WD AE: Withdrawn Due To A Specific Event
Tafenoquine = Tafenoquine 200mg, Mefloquine = Mefloquine 250mg

SB252263 - 030

Listing B.13

Baseline Signs And Symptoms

Treatment : Tafenoquine

Subject Number	Popn*	Verbatim Term / PREFERRED TERM	Primary Body System	Onset Date Time	End Date Time	Relative Day of Onset (End)**	Dur+	Out++	Epi#	Max int##	Rel^	Corr ther^^	Wd AE$
030.001.00028	IPP	STOMACH PAIN / ABDOMINAL PAIN	GASTROINTESTIN AL SYSTEM	03MAY2000	04MAY2000	-5 (-4)	2 DAYS	RESO	CON	MIL	NOT	Yes	No
030.001.00030	IPP	ORBITAL PAIN / EYE PAIN	VISION	06MAY2000	08MAY2000	-2 (0)	3 DAYS	RESO	CON	MIL	NOT	No	No
030.001.00040	IPP	PLASMODIUM FALCIPARUM MALARIA / INFECTION	RESISTANCE MECHANISM	02MAY2000		-6 ()	CONT	ONGO	CON	MIL	NOT	No	No
030.001.00042	IPP	COUGH / COUGHING	RESPIRATORY SYSTEM	03MAY2000	10MAY2000	-5 (2)	8 DAYS	RESO	CON	MOD	NOT	No	No
		ENTERITIS / ENTERITIS	GASTROINTESTIN AL SYSTEM	05MAY2000	08MAY2000	-3 (0)	4 DAYS	RESO	CON	MIL	NOT	Yes	No
030.001.00043	IPP	BACK PAIN / BACK PAIN	MUSCULOSKELETA L SYSTEM	03MAY2000	04MAY2000	-5 (-4)	2 DAYS	RESO	CON	MIL	UNL	Yes	No
		CHEST PAIN / CHEST PAIN	BODY AS A WHOLE GENERAL	03MAY2000	05MAY2000	-5 (-3)	3 DAYS	RESO	CON	MIL	NOT	Yes	No
030.001.00048	IPP	LOW HAEMATOCRIT / ANEMIA	RED BLOOD CELL	13APR2000	08MAY2000	-25 (0)	26 DAYS	RESO	CON	MIL	NOT	No	No

*Popn=Population (I=Intent-to-Treat, PP=Per Protocol)
**No. of days relative to start of prophylactic medication
+Dur=Duration : CONT=Continuing
++Out=Outcome : RESO=Resolved, ONGO=Ongoing
#Epi=Episode: n=No. of Episodes, CON= Constant, ITE= Intermittent
##Max Int = Intensity (Maximum): MIL=Mild, MOD=Moderate, SEV=Severe
^REL=Relationship To Study Procedures: NOT=Not related, UNL= Unlikely, SUS= Suspected, PRB=Probable
^^Corr Ther= Corrective Therapy
$WD AE: Withdrawn Due To A Specific Event
Tafenoquine = Tafenoquine 200mg, Mefloquine = Mefloquine 250mg

SB252263 - 030

Listing B.13

Baseline Signs And Symptoms

Treatment : Tafenoquine

Subject Number	Popn*	Verbatim Term / PREFERRED TERM	Primary Body System	Onset Date Time	End Date Time	Relative Day of Onset (End)**	Dur+	Out++	Epi#	Max int##	Rel^	Corr ther^^	Wd AE$
030.001.00048	IPP	NECK MUSCULOSKELETAL PAIN / MYALGIA	MUSCULOSKELETAL SYSTEM	16APR2000	31MAY2000	-22 (23)	46 DAYS	RESO	CON	MOD	NOT	Yes	No
030.001.00050	IPP	MYALGIA / MYALGIA	MUSCULOSKELETAL SYSTEM	07MAY2000	08MAY2000	-1 (0)	2 DAYS	RESO	CON	MIL	UNL	No	No
030.001.00051	IPP	LOWER RESPIRATORY TRACT INFECTION / RESPIRATORY DISORDER	RESPIRATORY SYSTEM	06MAY2000	13MAY2000	-2 (5)	8 DAYS	RESO	CON	MIL	NOT	Yes	No
030.001.00058	IPP	COUGH / COUGHING	RESPIRATORY SYSTEM	06MAY2000	07MAY2000	-2 (-1)	2 DAYS	RESO	CON	MIL	NOT	Yes	No
030.001.00060	IPP	ABDOMINAL PAIN / ABDOMINAL PAIN	GASTROINTESTINAL SYSTEM	03MAY2000	08MAY2000	-5 (0)	6 DAYS	RESO	CON	MIL	NOT	No	No
		BRONCHITIS / BRONCHITIS	RESPIRATORY SYSTEM	04MAY2000	10MAY2000	-4 (2)	7 DAYS	RESO	CON	MIL	NOT	Yes	No
		HELMINTHIASIS / INFECTION	RESISTANCE MECHANISM	06MAY2000	08MAY2000	-2 (0)	3 DAYS	RESO	CON	MIL	NOT	Yes	No

*Popn=Population (I=Intent-to-Treat, PP=Per Protocol)
**No. of days relative to start of prophylactic medication
+Dur=Duration : CONT=Continuing
++Out=Outcome : RESO=Resolved, ONGO=Ongoing
#Epi=Episode: n=No. of Episodes, CON= Constant, ITE= Intermittent
##Max Int = Intensity (Maximum): MIL=Mild, MOD=Moderate, SEV=Severe
^REL=Relationship To Study Procedures: NOT=Not related, UNL= Unlikely, SUS= Suspected, PRB=Probable
^^Corr Ther= Corrective Therapy
$WD AE: Withdrawn Due To A Specific Event
Tafenoquine = Tafenoquine 200mg, Mefloquine = Mefloquine 250mg

SB252263 - 030

Listing B.13

Baseline Signs And Symptoms

Treatment : Tafenoquine

Subject Number	Popn*	Verbatim Term / PREFERRED TERM	Primary Body System	Onset Date Time	End Date Time	Relative Day of Onset (End)**	Dur+	Out++	Epi#	Max int##	Rel^	Corr ther^^	Wd AE$
030.001.00060	IPP	PRURITUS / PRURITUS	SKIN AND APPENDAGES	03MAY2000		-5 ()	CONT	ONGO	2	MIL	NOT	Yes	No
030.001.00068	IPP	COUGH / COUGHING	RESPIRATORY SYSTEM	04MAY2000	25AUG2000	-4 (109)	114 DAYS	RESO	4	MIL	NOT	Yes	No
030.001.00070	IPP	CHEST PAIN / CHEST PAIN	BODY AS A WHOLE GENERAL	06MAY2000	08MAY2000	-2 (0)	3 DAYS	RESO	CON	MIL	SUS	Yes	No
		HEADACHE / HEADACHE	CENTRAL AND PERIPHERAL NERVOUS SYSTEM	06MAY2000	08MAY2000	-2 (0)	3 DAYS	RESO	CON	MIL	SUS	No	No
030.001.00079	IPP	DIAPHORESIS / SWEATING INCREASED	AUTONOMIC NERVOUS SYSTEM	07MAY2000	08MAY2000	-1 (0)	2 DAYS	RESO	CON	MIL	NOT	No	No
		HEADACHE / HEADACHE	CENTRAL AND PERIPHERAL NERVOUS SYSTEM	06MAY2000	08MAY2000	-2 (0)	3 DAYS	RESO	CON	MIL	SUS	No	No
		MALAISE / MALAISE	BODY AS A WHOLE GENERAL	03MAY2000	10MAY2000	-5 (2)	8 DAYS	RESO	CON	MIL	NOT	Yes	No
030.001.00104	IPP	NECK PAIN / BACK PAIN	MUSCULOSKELETAL SYSTEM	05MAY2000	05MAY2000	-3 (-3)	1 DAY	RESO	CON	MIL	NOT	No	No

*Popn=Population (I=Intent-to-Treat, PP=Per Protocol)
**No. of days relative to start of prophylactic medication
+Dur=Duration : CONT=Continuing
++Out=Outcome : RESO=Resolved, ONGO=Ongoing
#Epi=Episode: n=No. of Episodes, CON= Constant, ITE= Intermittent
##Max Int = Intensity (Maximum): MIL=Mild, MOD=Moderate, SEV=Severe
^REL=Relationship To Study Procedures: NOT=Not related, UNL= Unlikely, SUS= Suspected, PRB=Probable
^^Corr Ther= Corrective Therapy
$WD AE: Withdrawn Due To A Specific Event
Tafenoquine = Tafenoquine 200mg, Mefloquine = Mefloquine 250mg

SB252263 - 030

Listing B.13

Baseline Signs And Symptoms

Treatment : Tafenoquine

Subject Number	Popn*	Verbatim Term / PREFERRED TERM	Primary Body System	Onset Date Time	End Date Time	Relative Day of Onset (End)**	Dur+	Out++	Epi#	Max int##	Rel^	Corr ther^^	Wd AE$
030.001.00104	IPP	PRURITIS / PRURITUS	SKIN AND APPENDAGES	04MAY2000	07MAY2000	-4 (-1)	4 DAYS	RESO	CON	MIL	SUS	No	No
030.001.00109	IPP	UPPER RESPIRATORY TRACT INFECTION / UPPER RESP TRACT INFECTION	RESISTANCE MECHANISM	03MAY2000	08MAY2000	-5 (0)	6 DAYS	RESO	CON	MIL	NOT	No	No
030.001.00111	IPP	CONJUNCTIVITIS / CONJUNCTIVITIS	VISION	01APR2000	21APR2000	-37 (-17)	21 DAYS	RESO	CON	MOD	NOT	Yes	No
		LYMPHOCYTOSIS / LYMPHOCYTOSIS	WHITE CELL AND RETICULOENDOTHELIAL SYSTE	11APR2000	05JUN2000	-27 (28)	56 DAYS	RESO	CON	MIL	NOT	No	No
030.001.00126	IPP	DYSPEPSIA / DYSPEPSIA	GASTROINTESTINAL SYSTEM	07MAY2000	10MAY2000	-2 (1)	4 DAYS	RESO	4	MIL	SUS	Yes	No
030.001.00129	IPP	MUSCULOSKELETAL PAIN / MYALGIA	MUSCULOSKELETAL SYSTEM	01MAY2000	11MAY2000	-8 (2)	11 DAYS	RESO	4	MIL	NOT	Yes	No

*Popn=Population (I=Intent-to-Treat, PP=Per Protocol)
**No. of days relative to start of prophylactic medication
+Dur=Duration : CONT=Continuing
++Out=Outcome : RESO=Resolved, ONGO=Ongoing
#Epi=Episode: n=No. of Episodes, CON= Constant, ITE= Intermittent
##Max Int = Intensity (Maximum): MIL=Mild, MOD=Moderate, SEV=Severe
^REL=Relationship To Study Procedures: NOT=Not related, UNL= Unlikely, SUS= Suspected, PRB=Probable
^^Corr Ther= Corrective Therapy
$WD AE: Withdrawn Due To A Specific Event
Tafenoquine = Tafenoquine 200mg, Mefloquine = Mefloquine 250mg

SB252263 - 030

Listing B.13

Baseline Signs And Symptoms

Treatment : Tafenoquine

Subject Number	Popn*	Verbatim Term / PREFERRED TERM	Primary Body System	Onset Date Time	End Date Time	Relative Day of Onset (End)**	Dur+	Out++	Epi#	Max int##	Rel^	Corr ther^^	Wd AE$
030.001.00133	IPP	TONSILLITIS / PHARYNGITIS	RESPIRATORY SYSTEM	28APR2000	02MAY2000	-11 (-7)	5 DAYS	RESO	CON	MIL	NOT	Yes	No
030.001.00135	IPP	COUGH / COUGHING	RESPIRATORY SYSTEM	04MAY2000	09MAY2000	-5 (0)	6 DAYS	RESO	CON	MIL	NOT	Yes	No
030.001.00138	IPP	DENTAL PAIN/TOOTHACHE / TOOTH ACHE	GASTROINTESTINAL SYSTEM	07MAY2000	15MAY2000	-2 (6)	9 DAYS	RESO	CON	MIL	NOT	Yes	No
030.001.00139	IPP	COUGH / COUGHING	RESPIRATORY SYSTEM	06MAY2000	11MAY2000	-3 (2)	6 DAYS	RESO	CON	MOD	NOT	Yes	No
030.001.00155	IPP	ABDOMINAL PAIN / ABDOMINAL PAIN	GASTROINTESTINAL SYSTEM	04MAY2000	05MAY2000	-5 (-4)	2 DAYS	RESO	CON	MIL	NOT	Yes	No
		PRURITUS / PRURITUS	SKIN AND APPENDAGES	04MAY2000	08JUL2000	-5 (60)	66 DAYS	RESO	2	MIL	NOT	Yes	No
030.001.00158	IPP	PELVIC INFLAMMATORY DISEASE / PELVIC INFLAMMATION	REPRODUCTIVE FEMALE	19APR2000	25APR2000	-20 (-14)	7 DAYS	RESO	CON	SEV	NOT	Yes	No

*Popn=Population (I=Intent-to-Treat, PP=Per Protocol)
**No. of days relative to start of prophylactic medication
+Dur=Duration : CONT=Continuing
++Out=Outcome : RESO=Resolved, ONGO=Ongoing
#Epi=Episode: n=No. of Episodes, CON= Constant, ITE= Intermittent
##Max Int = Intensity (Maximum): MIL=Mild, MOD=Moderate, SEV=Severe
^REL=Relationship To Study Procedures: NOT=Not related, UNL= Unlikely, SUS= Suspected, PRB=Probable
^^Corr Ther= Corrective Therapy
$WD AE: Withdrawn Due To A Specific Event
Tafenoquine = Tafenoquine 200mg, Mefloquine = Mefloquine 250mg

SB252263 - 030

Listing B.13

Baseline Signs And Symptoms

Treatment : Tafenoquine

Subject Number	Popn*	Verbatim Term / PREFERRED TERM	Primary Body System	Onset Date Time	End Date Time	Relative Day of Onset (End)**	Dur+	Out++	Epi#	Max int##	Rel^	Corr ther^^	Wd AE$
030.001.00161	IPP	FATIGUE / FATIGUE	BODY AS A WHOLE GENERAL	05MAY2000	06MAY2000	-4 (-3)	2 DAYS	RESO	CON	MIL	UNL	No	No
030.001.00171	IPP	ABDOMINAL PAIN / ABDOMINAL PAIN	GASTROINTESTINAL SYSTEM	02MAY2000	11MAY2000	-7 (2)	10 DAYS	RESO	ITE	MIL	NOT	Yes	No
		WOUND ON THE FOREHEAD / INJURY	BODY AS A WHOLE GENERAL	06MAY2000	09MAY2000	-3 (0)	4 DAYS	RESO	CON	MIL	NOT	Yes	No
030.001.00176	IPP	HEADACHE / HEADACHE	CENTRAL AND PERIPHERAL NERVOUS SYSTEM	08MAY2000	10MAY2000	-1 (1)	3 DAYS	RESO	2	MIL	NOT	Yes	No
		TRICHOMONAS VAGINITIS / VAGINITIS	REPRODUCTIVE FEMALE	05MAY2000	09MAY2000	-4 (0)	5 DAYS	RESO	CON	MIL	NOT	Yes	No
030.001.00179	I	PEPTIC ULCER DISEASE / PEPTIC ULCER	GASTROINTESTINAL SYSTEM	05MAY2000		-4 ()	CONT	ONGO	CON	MIL	NOT	Yes	No
030.001.00186	IPP	MYALGIA / MYALGIA	MUSCULOSKELETAL SYSTEM	07MAY2000	10MAY2000	-2 (1)	4 DAYS	RESO	CON	MIL	NOT	Yes	No

*Popn=Population (I=Intent-to-Treat, PP=Per Protocol)
**No. of days relative to start of prophylactic medication
+Dur=Duration : CONT=Continuing
++Out=Outcome : RESO=Resolved, ONGO=Ongoing
#Epi=Episode: n=No. of Episodes, CON= Constant, ITE= Intermittent
##Max Int = Intensity (Maximum): MIL=Mild, MOD=Moderate, SEV=Severe
^REL=Relationship To Study Procedures: NOT=Not related, UNL= Unlikely, SUS= Suspected, PRB=Probable
^^Corr Ther= Corrective Therapy
$WD AE: Withdrawn Due To A Specific Event
Tafenoquine = Tafenoquine 200mg, Mefloquine = Mefloquine 250mg

SB252263 - 030

Listing B.13

Baseline Signs And Symptoms

Treatment : Tafenoquine

Subject Number	Popn*	Verbatim Term / PREFERRED TERM	Primary Body System	Onset Date Time	End Date Time	Relative Day of Onset (End)**	Dur+	Out++	Epi#	Max int##	Rel^	Corr ther^^	Wd AE$
030.001.00190	IPP	ABDOMINAL DISTENSION / ABDOMEN ENLARGED	BODY AS A WHOLE GENERAL	05MAY2000	09MAY2000	-4 (0)	5 DAYS	RESO	CON	MIL	NOT	No	No
030.001.00194	IPP	ARTHRALGIA / ARTHRALGIA	MUSCULOSKELETAL SYSTEM	04MAY2000	09MAY2000	-5 (0)	6 DAYS	RESO	CON	MIL	NOT	No	No
		HEADACHE / HEADACHE	CENTRAL AND PERIPHERAL NERVOUS SYSTEM	04MAY2000	13MAY2000	-5 (4)	10 DAYS	RESO	CON	MIL	NOT	Yes	No
030.001.00201	IPP	(L) SHOULDER ARTHRALGIA / ARTHRALGIA	MUSCULOSKELETAL SYSTEM	06MAY2000	13MAY2000	-3 (4)	8 DAYS	RESO	CON	MOD	NOT	Yes	No
		COUGH / COUGHING	RESPIRATORY SYSTEM	06MAY2000	13MAY2000	-3 (4)	8 DAYS	RESO	CON	MIL	NOT	Yes	No
030.001.00203	IPP	UPPER RESPIRATORY INFECTION / UPPER RESP TRACT INFECTION	RESISTANCE MECHANISM	04MAY2000	20MAY2000	-5 (11)	17 DAYS	RESO	CON	MIL	NOT	Yes	No

*Popn=Population (I=Intent-to-Treat, PP=Per Protocol)
**No. of days relative to start of prophylactic medication
+Dur=Duration : CONT=Continuing
++Out=Outcome : RESO=Resolved, ONGO=Ongoing
#Epi=Episode: n=No. of Episodes, CON= Constant, ITE= Intermittent
##Max Int = Intensity (Maximum): MIL=Mild, MOD=Moderate, SEV=Severe
^REL=Relationship To Study Procedures: NOT=Not related, UNL= Unlikely, SUS= Suspected, PRB=Probable
^^Corr Ther= Corrective Therapy
$WD AE: Withdrawn Due To A Specific Event
Tafenoquine = Tafenoquine 200mg, Mefloquine = Mefloquine 250mg

SB252263 - 030

Listing B.13

Baseline Signs And Symptoms

Treatment : Tafenoquine

Subject Number	Popn*	Verbatim Term / PREFERRED TERM	Primary Body System	Onset Date Time	End Date Time	Relative Day of Onset (End)**	Dur+	Out++	Epi#	Max int##	Rel^	Corr ther^^	Wd AE$
030.001.00205	IPP	HEADACHE / HEADACHE	CENTRAL AND PERIPHERAL NERVOUS SYSTEM	06MAY2000	07MAY2000	-3 (-2)	2 DAYS	RESO	CON	MIL	SUS	No	No
030.001.00231	IPP	CELLULITIS / CELLULITIS	BODY AS A WHOLE GENERAL	05MAY2000	12MAY2000	-4 (3)	8 DAYS	RESO	CON	MIL	NOT	Yes	No
		DYSPNEA / DYSPNEA	RESPIRATORY SYSTEM	04MAY2000	06MAY2000	-5 (-3)	3 DAYS	RESO	CON	MIL	NOT	No	No
030.001.00233	IPP	CHEST PAIN / CHEST PAIN	BODY AS A WHOLE GENERAL	08MAY2000	10MAY2000	-2 (0)	3 DAYS	RESO	2	MIL	PRB	Yes	No
		NAIL PRICK WOUND / INJURY	BODY AS A WHOLE GENERAL	08MAY2000	03JUL2000	-2 (54)	57 DAYS	RESO	CON	MOD	NOT	Yes	No
030.001.00243	IPP	BACKACHE / BACK PAIN	MUSCULOSKELETA L SYSTEM	05MAY2000	19MAY2000	-5 (9)	15 DAYS	RESO	CON	MIL	NOT	Yes	No
030.001.00254	I	ABDOMINAL PAIN / ABDOMINAL PAIN	GASTROINTESTIN AL SYSTEM	03MAY2000 18:00		-7 ()	CONT	ONGO	ITE	MIL	NOT	Yes	No

*Popn=Population (I=Intent-to-Treat, PP=Per Protocol)
**No. of days relative to start of prophylactic medication
+Dur=Duration : CONT=Continuing
++Out=Outcome : RESO=Resolved, ONGO=Ongoing
#Epi=Episode: n=No. of Episodes, CON= Constant, ITE= Intermittent
##Max Int = Intensity (Maximum): MIL=Mild, MOD=Moderate, SEV=Severe
^REL=Relationship To Study Procedures: NOT=Not related, UNL= Unlikely, SUS= Suspected, PRB=Probable
^^Corr Ther= Corrective Therapy
$WD AE: Withdrawn Due To A Specific Event
Tafenoquine = Tafenoquine 200mg, Mefloquine = Mefloquine 250mg

SB252263 - 030

Listing B.13

Baseline Signs And Symptoms

Treatment : Tafenoquine

Subject Number	Popn*	Verbatim Term / PREFERRED TERM	Primary Body System	Onset Date Time	End Date Time	Relative Day of Onset (End)**	Dur+	Out++	Epi#	Max int##	Rel^	Corr ther^^	Wd AE$
030.001.00254	I	UTI / URINARY TRACT INFECTION	URINARY SYSTEM	14APR2000	13MAY2000	-26 (3)	30 DAYS	RESO	CON	MIL	NOT	Yes	No
030.001.00259	IPP	FUNGAL SKIN INFECTION / DERMATITIS FUNGAL	SKIN AND APPENDAGES	08MAY2000	15MAY2000	-2 (5)	8 DAYS	RESO	CON	MIL	NOT	Yes	No
030.001.00266	I	PENILE INJURY / INJURY	BODY AS A WHOLE GENERAL	03MAY2000	07MAY2000	-7 (-3)	5 DAYS	RESO	CON	MIL	NOT	Yes	No
030.001.00279	IPP	SUNBURN / PHOTOSENSITIV ITY REACTION	SKIN AND APPENDAGES	04MAY2000	06MAY2000	-7 (-5)	3 DAYS	RESO	CON	MIL	NOT	No	No
		URI / UPPER RESP TRACT INFECTION	RESISTANCE MECHANISM	04MAY2000	06MAY2000	-7 (-5)	3 DAYS	RESO	CON	MIL	NOT	No	No
030.001.00282	IPP	SINUS CONGESTION / SINUSITIS	RESPIRATORY SYSTEM	06MAY2000	13JUN2000	-4 (34)	39 DAYS	RESO	ITE	MIL	NOT	Yes	No

*Popn=Population (I=Intent-to-Treat, PP=Per Protocol)
**No. of days relative to start of prophylactic medication
+Dur=Duration : CONT=Continuing
++Out=Outcome : RESO=Resolved, ONGO=Ongoing
#Epi=Episode: n=No. of Episodes, CON= Constant, ITE= Intermittent
##Max Int = Intensity (Maximum): MIL=Mild, MOD=Moderate, SEV=Severe
^REL=Relationship To Study Procedures: NOT=Not related, UNL= Unlikely, SUS= Suspected, PRB=Probable
^^Corr Ther= Corrective Therapy
$WD AE: Withdrawn Due To A Specific Event
Tafenoquine = Tafenoquine 200mg, Mefloquine = Mefloquine 250mg

Listing B.13

Baseline Signs And Symptoms

Treatment : Tafenoquine

Subject Number	Popn*	Verbatim Term / PREFERRED TERM	Primary Body System	Onset Date Time	End Date Time	Relative Day of Onset (End)**	Dur+	Out++	Epi#	Max int##	Rel^	Corr ther^^	Wd AE$
030.001.00282	IPP	VIRAL SYNDROME / INFECTION VIRAL	RESISTANCE MECHANISM	05MAY2000	06MAY2000	-5 (-4)	2 DAYS	RESO	CON	MIL	NOT	No	No
030.001.00284	IPP	HEADACHE / HEADACHE	CENTRAL AND PERIPHERAL NERVOUS SYSTEM	02MAY2000		-9 ()	CONT	ONGO	ITE	MIL	SUS	Yes	No
030.001.00288	IPP	COUGH / COUGHING	RESPIRATORY SYSTEM	08MAY2000	13MAY2000	-3 (2)	6 DAYS	RESO	CON	MIL	NOT	No	No
		HEADACHE / HEADACHE	CENTRAL AND PERIPHERAL NERVOUS SYSTEM	23APR2000	13MAY2000	-18 (2)	21 DAYS	RESO	CON	MIL	NOT	Yes	No
030.001.00293	IPP	FLU / INFECTION VIRAL	RESISTANCE MECHANISM	07MAY2000		-4 ()	CONT	ONGO	CON	MIL	NOT	No	No
030.001.00296	IPP	ABDOMINAL PAIN / ABDOMINAL PAIN	GASTROINTESTIN AL SYSTEM	07MAY2000	08MAY2000	-4 (-3)	2 DAYS	RESO	CON	MIL	SUS	No	No
030.001.00298	IPP	HOOKWORM / INFECTION	RESISTANCE MECHANISM	28APR2000	30APR2000	-14 (-12)	3 DAYS	RESO	CON	MIL	NOT	Yes	No

*Popn=Population (I=Intent-to-Treat, PP=Per Protocol)
**No. of days relative to start of prophylactic medication
+Dur=Duration : CONT=Continuing
++Out=Outcome : RESO=Resolved, ONGO=Ongoing
#Epi=Episode: n=No. of Episodes, CON= Constant, ITE= Intermittent
##Max Int = Intensity (Maximum): MIL=Mild, MOD=Moderate, SEV=Severe
^REL=Relationship To Study Procedures: NOT=Not related, UNL= Unlikely, SUS= Suspected, PRB=Probable
^^Corr Ther= Corrective Therapy
$WD AE: Withdrawn Due To A Specific Event
Tafenoquine = Tafenoquine 200mg, Mefloquine = Mefloquine 250mg

SB252263 - 030

Listing B.13

Baseline Signs And Symptoms

Treatment : Tafenoquine

Subject Number	Popn*	Verbatim Term / PREFERRED TERM	Primary Body System	Onset Date Time	End Date Time	Relative Day of Onset (End)**	Dur+	Out++	Epi#	Max int##	Rel^	Corr ther^^	Wd AE$
030.001.00301	IPP	DERMATITIS / DERMATITIS	SKIN AND APPENDAGES	07MAY2000	12MAY2000	-5 (0)	6 DAYS	RESO	CON	MIL	NOT	Yes	No
		SOFT TISSUE INJURY / INJURY	BODY AS A WHOLE GENERAL	07MAY2000	12MAY2000	-5 (0)	6 DAYS	RESO	CON	MIL	NOT	Yes	No
030.001.00302	I	HEADACHE / HEADACHE	CENTRAL AND PERIPHERAL NERVOUS SYSTEM	06MAY2000		-6 ()	CONT	ONGO	ITE	MIL	NOT	Yes	No
030.001.00306	IPP	PRURITUS / PRURITUS	SKIN AND APPENDAGES	10MAY2000	18MAY2000	-2 (6)	9 DAYS	RESO	ITE	MIL	NOT	Yes	No

*Popn=Population (I=Intent-to-Treat, PP=Per Protocol)
**No. of days relative to start of prophylactic medication
+Dur=Duration : CONT=Continuing
++Out=Outcome : RESO=Resolved, ONGO=Ongoing
#Epi=Episode: n=No. of Episodes, CON= Constant, ITE= Intermittent
##Max Int = Intensity (Maximum): MIL=Mild, MOD=Moderate, SEV=Severe
^REL=Relationship To Study Procedures: NOT=Not related, UNL= Unlikely, SUS= Suspected, PRB=Probable
^^Corr Ther= Corrective Therapy
$WD AE: Withdrawn Due To A Specific Event
Tafenoquine = Tafenoquine 200mg, Mefloquine = Mefloquine 250mg

SB252263 - 030

Listing B.13

Baseline Signs And Symptoms

Treatment : Mefloquine

Subject Number	Popn*	Verbatim Term / PREFERRED TERM	Primary Body System	Onset Date Time	End Date Time	Relative Day of Onset (End)**	Dur+	Out++	Epi#	Max int##	Rel^	Corr ther^^	Wd AE$
030.001.00008	IPP	URTI (URI) / UPPER RESP TRACT INFECTION	RESISTANCE MECHANISM	01MAY2000	20JUN2000	-7 (43)	51 DAYS	RESO	3	MIL	NOT	Yes	No
030.001.00010	IPP	BACKACHE / BACK PAIN	MUSCULOSKELETAL SYSTEM	06MAY2000	08MAY2000	-2 (0)	3 DAYS	RESO	CON	MIL	UNL	No	No
030.001.00013	IPP	LOW BACK PAIN / BACK PAIN	MUSCULOSKELETAL SYSTEM	06MAY2000	09MAY2000	-2 (1)	4 DAYS	RESO	CON	MIL	NOT	Yes	No
030.001.00021	IPP	BRONCHITIS COUGH / BRONCHITIS	RESPIRATORY SYSTEM	03MAY2000	08MAY2000	-5 (0)	6 DAYS	RESO	CON	MIL	NOT	Yes	No
		PHARYNGITIS SORE THROAT / PHARYNGITIS	RESPIRATORY SYSTEM	03MAY2000	08MAY2000	-5 (0)	6 DAYS	RESO	CON	MIL	NOT	Yes	No
030.001.00023	I	BACKACHE / BACK PAIN	MUSCULOSKELETAL SYSTEM	06MAY2000	08MAY2000	-2 (0)	3 DAYS	RESO	CON	MIL	UNL	No	No
		TOOTHACHE / TOOTH ACHE	GASTROINTESTINAL SYSTEM	04MAY2000	05MAY2000	-4 (-3)	2 DAYS	RESO	CON	MIL	NOT	Yes	No

*Popn=Population (I=Intent-to-Treat, PP=Per Protocol)
**No. of days relative to start of prophylactic medication
+Dur=Duration : CONT=Continuing
++Out=Outcome : RESO=Resolved, ONGO=Ongoing
#Epi=Episode: n=No. of Episodes, CON= Constant, ITE= Intermittent
##Max Int = Intensity (Maximum): MIL=Mild, MOD=Moderate, SEV=Severe
^REL=Relationship To Study Procedures: NOT=Not related, UNL= Unlikely, SUS= Suspected, PRB=Probable
^^Corr Ther= Corrective Therapy
$WD AE: Withdrawn Due To A Specific Event
Tafenoquine = Tafenoquine 200mg, Mefloquine = Mefloquine 250mg

SB252263 - 030

Listing B.13

Baseline Signs And Symptoms

Treatment : Mefloquine

Subject Number	Popn*	Verbatim Term / PREFERRED TERM	Primary Body System	Onset Date Time	End Date Time	Relative Day of Onset (End)**	Dur+	Out++	Epi#	Max int##	Rel^	Corr ther^^	Wd AE$
030.001.00031	IPP	BACKACHE / BACK PAIN	MUSCULOSKELETAL SYSTEM	03MAY2000		-5 ()	CONT	ONGO	CON	MIL	NOT	No	No
030.001.00035		GASTROENTERITIS / GASTROENTERITIS	GASTROINTESTINAL SYSTEM	04MAY2000	05MAY2000	-4 (-3)	2 DAYS	RESO	CON	MIL	UNL	No	No
		HEADACHE / HEADACHE	CENTRAL AND PERIPHERAL NERVOUS SYSTEM	04MAY2000	05MAY2000	-4 (-3)	2 DAYS	RESO	CON	MIL	UNL	Yes	No
030.001.00036	I	ARTHRALGIA / ARTHRALGIA	MUSCULOSKELETAL SYSTEM	03MAY2000	04MAY2000	-5 (-4)	2 DAYS	RESO	CON	MIL	NOT	Yes	No
		HEADACHE / HEADACHE	CENTRAL AND PERIPHERAL NERVOUS SYSTEM	03MAY2000	04MAY2000	-5 (-4)	2 DAYS	RESO	CON	MIL	NOT	Yes	No
030.001.00038	IPP	AMOEBIASIS / INFECTION	RESISTANCE MECHANISM	06MAY2000	13MAY2000	-2 (5)	8 DAYS	RESO	CON	MIL	NOT	Yes	No
		HEADACHE / HEADACHE	CENTRAL AND PERIPHERAL NERVOUS SYSTEM	06MAY2000	08MAY2000	-2 (0)	3 DAYS	RESO	CON	MIL	SUS	No	No

*Popn=Population (I=Intent-to-Treat, PP=Per Protocol)
**No. of days relative to start of prophylactic medication
+Dur=Duration : CONT=Continuing
++Out=Outcome : RESO=Resolved, ONGO=Ongoing
#Epi=Episode: n=No. of Episodes, CON= Constant, ITE= Intermittent
##Max Int = Intensity (Maximum): MIL=Mild, MOD=Moderate, SEV=Severe
^REL=Relationship To Study Procedures: NOT=Not related, UNL= Unlikely, SUS= Suspected, PRB=Probable
^^Corr Ther= Corrective Therapy
$WD AE: Withdrawn Due To A Specific Event
Tafenoquine = Tafenoquine 200mg, Mefloquine = Mefloquine 250mg

SB252263 - 030

Listing B.13

Baseline Signs And Symptoms

Treatment : Mefloquine

Subject Number	Popn*	Verbatim Term / PREFERRED TERM	Primary Body System	Onset Date Time	End Date Time	Relative Day of Onset (End)**	Dur+	Out++	Epi#	Max int##	Rel^	Corr ther^^	Wd AE$
030.001.00046	IPP	HEADACHE / HEADACHE	CENTRAL AND PERIPHERAL NERVOUS SYSTEM	04MAY2000	05MAY2000	-4 (-3)	2 DAYS	RESO	CON	MIL	UNL	Yes	No
		MALARIA / INFECTION	RESISTANCE MECHANISM	03MAY2000	05MAY2000	-5 (-3)	3 DAYS	RESO	CON	MIL	UNL	Yes	No
030.001.00047	IPP	BACKPAIN / BACK PAIN	MUSCULOSKELETA L SYSTEM	03MAY2000	05MAY2000	-5 (-3)	3 DAYS	RESO	CON	MIL	NOT	No	No
030.001.00052	IPP	HEADACHE / HEADACHE	CENTRAL AND PERIPHERAL NERVOUS SYSTEM	03MAY2000	06MAY2000	-5 (-2)	4 DAYS	RESO	CON	MIL	NOT	Yes	No
		LYMPHOCYTOSIS / LYMPHOCYTOSIS	WHITE CELL AND RETICULOENDOTH ELIAL SYSTE	10APR2000	08MAY2000	-28 (0)	29 DAYS	RESO	CON	MIL	NOT	No	No
030.001.00053	IPP	HEADACHE / HEADACHE	CENTRAL AND PERIPHERAL NERVOUS SYSTEM	19APR2000	22APR2000	-19 (-16)	4 DAYS	RESO	CON	MIL	NOT	Yes	No
		NAUSEA / NAUSEA	GASTROINTESTIN AL SYSTEM	04MAY2000	05MAY2000	-4 (-3)	2 DAYS	RESO	CON	MIL	UNL	No	No

*Popn=Population (I=Intent-to-Treat, PP=Per Protocol)
**No. of days relative to start of prophylactic medication
+Dur=Duration : CONT=Continuing
++Out=Outcome : RESO=Resolved, ONGO=Ongoing
#Epi=Episode: n=No. of Episodes, CON= Constant, ITE= Intermittent
##Max Int = Intensity (Maximum): MIL=Mild, MOD=Moderate, SEV=Severe
^REL=Relationship To Study Procedures: NOT=Not related, UNL= Unlikely, SUS= Suspected, PRB=Probable
^^Corr Ther= Corrective Therapy
$WD AE: Withdrawn Due To A Specific Event
Tafenoquine = Tafenoquine 200mg, Mefloquine = Mefloquine 250mg

SB252263 - 030

Listing B.13

Baseline Signs And Symptoms

Treatment : Mefloquine

Subject Number	Popn*	Verbatim Term / PREFERRED TERM	Primary Body System	Onset Date Time	End Date Time	Relative Day of Onset (End)**	Dur+	Out++	Epi#	Max int##	Rel^	Corr ther^^	Wd AE$
030.001.00062	IPP	MYALGIA / MYALGIA	MUSCULOSKELETAL SYSTEM	07MAY2000	14MAY2000	-1 (6)	8 DAYS	RESO	CON	MIL	NOT	No	No
030.001.00066	IPP	HEADACHE / HEADACHE	CENTRAL AND PERIPHERAL NERVOUS SYSTEM	03MAY2000	06MAY2000	-5 (-2)	4 DAYS	RESO	3	MIL	NOT	Yes	No
030.001.00075	IPP	PRURITIS / PRURITUS	SKIN AND APPENDAGES	05MAY2000	07MAY2000	-3 (-1)	3 DAYS	RESO	CON	MIL	PRB	Yes	No
		URI / UPPER RESP TRACT INFECTION	RESISTANCE MECHANISM	03MAY2000	04MAY2000	-5 (-4)	2 DAYS	RESO	CON	MIL	NOT	No	No
030.001.00099	IPP	STOMACH PAIN / ABDOMINAL PAIN	GASTROINTESTINAL SYSTEM	04MAY2000	11MAY2000	-4 (3)	8 DAYS	RESO	3	MIL	NOT	Yes	No
030.001.00105	IPP	CHICKEN POX / VARICELLA	SECONDARY TERM	13APR2000	19APR2000	-25 (-19)	7 DAYS	RESO	CON	SEV	NOT	Yes	No
030.001.00113	IPP	DENTAL CARIES / TOOTH CARIES	GASTROINTESTINAL SYSTEM	07MAY2000		-1 ()	CONT	ONGO	3	MIL	NOT	Yes	No

*Popn=Population (I=Intent-to-Treat, PP=Per Protocol)
**No. of days relative to start of prophylactic medication
+Dur=Duration : CONT=Continuing
++Out=Outcome : RESO=Resolved, ONGO=Ongoing
#Epi=Episode: n=No. of Episodes, CON= Constant, ITE= Intermittent
##Max Int = Intensity (Maximum): MIL=Mild, MOD=Moderate, SEV=Severe
^REL=Relationship To Study Procedures: NOT=Not related, UNL= Unlikely, SUS= Suspected, PRB=Probable
^^Corr Ther= Corrective Therapy
$WD AE: Withdrawn Due To A Specific Event
Tafenoquine = Tafenoquine 200mg, Mefloquine = Mefloquine 250mg

SB252263 - 030

Listing B.13

Baseline Signs And Symptoms

Treatment : Mefloquine

Subject Number	Popn*	Verbatim Term / PREFERRED TERM	Primary Body System	Onset Date Time	End Date Time	Relative Day of Onset (End)**	Dur+	Out++	Epi#	Max int##	Rel^	Corr ther^^	Wd AE$
030.001.00113	IPP	RECURRENT BACKACHE / BACK PAIN	MUSCULOSKELETAL SYSTEM	07MAY2000		-1 ()	CONT	ONGO	CON	MIL	NOT	No	No
030.001.00121	IPP	URTI / UPPER RESP TRACT INFECTION	RESISTANCE MECHANISM	04MAY2000	09MAY2000	-5 (0)	6 DAYS	RESO	CON	MIL	NOT	Yes	No
030.001.00156	IPP	HEADACHE / HEADACHE	CENTRAL AND PERIPHERAL NERVOUS SYSTEM	04MAY2000	08MAY2000	-5 (-1)	5 DAYS	RESO	CON	MIL	NOT	Yes	No
030.001.00165	IPP	PRURITIS / PRURITUS	SKIN AND APPENDAGES	06MAY2000	13MAY2000	-3 (4)	8 DAYS	RESO	CON	MIL	SUS	No	No
030.001.00167	IPP	HEADACHE / HEADACHE	CENTRAL AND PERIPHERAL NERVOUS SYSTEM	06MAY2000	07MAY2000	-3 (-2)	2 DAYS	RESO	CON	MIL	SUS	No	No
		JOINT PAIN / ARTHRALGIA	MUSCULOSKELETAL SYSTEM	06MAY2000	07MAY2000	-3 (-2)	2 DAYS	RESO	CON	MIL	SUS	No	No
030.001.00170	IPP	BRONCHITIS / BRONCHITIS	RESPIRATORY SYSTEM	03MAY2000	09MAY2000	-6 (0)	7 DAYS	RESO	CON	MIL	NOT	Yes	No

*Popn=Population (I=Intent-to-Treat, PP=Per Protocol)
**No. of days relative to start of prophylactic medication
+Dur=Duration : CONT=Continuing
++Out=Outcome : RESO=Resolved, ONGO=Ongoing
#Epi=Episode: n=No. of Episodes, CON= Constant, ITE= Intermittent
##Max Int = Intensity (Maximum): MIL=Mild, MOD=Moderate, SEV=Severe
^REL=Relationship To Study Procedures: NOT=Not related, UNL= Unlikely, SUS= Suspected, PRB=Probable
^^Corr Ther= Corrective Therapy
$WD AE: Withdrawn Due To A Specific Event
Tafenoquine = Tafenoquine 200mg, Mefloquine = Mefloquine 250mg

SB252263 - 030

Listing B.13

Baseline Signs And Symptoms

Treatment : Mefloquine

Subject Number	Popn*	Verbatim Term / PREFERRED TERM	Primary Body System	Onset Date Time	End Date Time	Relative Day of Onset (End)**	Dur+	Out++	Epi#	Max int##	Rel^	Corr ther^^	Wd AE$
030.001.00177	IPP	HEADACHE / HEADACHE	CENTRAL AND PERIPHERAL NERVOUS SYSTEM	05MAY2000	06MAY2000	-4 (-3)	2 DAYS	RESO	CON	MIL	SUS	No	No
030.001.00178	I	ABDOMINAL PAIN / ABDOMINAL PAIN	GASTROINTESTIN AL SYSTEM	05MAY2000	13MAY2000	-4 (4)	9 DAYS	RESO	CON	MIL	UNL	No	No
		ARTHRALGIA / ARTHRALGIA	MUSCULOSKELETA L SYSTEM	02MAY2000	14MAY2000	-7 (5)	13 DAYS	RESO	CON	MIL	UNL	Yes	No
		STOMACH ACHE / ABDOMINAL PAIN	GASTROINTESTIN AL SYSTEM	05MAY2000	06MAY2000	-4 (-3)	2 DAYS	RESO	CON	MIL	UNL	No	No
030.001.00180	IPP	URTI / UPPER RESP TRACT INFECTION	RESISTANCE MECHANISM	01MAY2000	09MAY2000	-8 (0)	9 DAYS	RESO	2	MIL	NOT	Yes	No
030.001.00181	IPP	PRURITIS / PRURITUS	SKIN AND APPENDAGES	01MAY2000		-8 ()	CONT	ONGO	CON	MIL	NOT	Yes	No
030.001.00188	IPP	HEADACHE / HEADACHE	CENTRAL AND PERIPHERAL NERVOUS SYSTEM	06MAY2000	07MAY2000	-3 (-2)	2 DAYS	RESO	CON	MIL	UNL	No	No

*Popn=Population (I=Intent-to-Treat, PP=Per Protocol)
**No. of days relative to start of prophylactic medication
+Dur=Duration : CONT=Continuing
++Out=Outcome : RESO=Resolved, ONGO=Ongoing
#Epi=Episode: n=No. of Episodes, CON= Constant, ITE= Intermittent
##Max Int = Intensity (Maximum): MIL=Mild, MOD=Moderate, SEV=Severe
^REL=Relationship To Study Procedures: NOT=Not related, UNL= Unlikely, SUS= Suspected, PRB=Probable
^^Corr Ther= Corrective Therapy
$WD AE: Withdrawn Due To A Specific Event
Tafenoquine = Tafenoquine 200mg, Mefloquine = Mefloquine 250mg

SB252263 - 030

Listing B.13

Baseline Signs And Symptoms

Treatment : Mefloquine

Subject Number	Popn*	Verbatim Term / PREFERRED TERM	Primary Body System	Onset Date Time	End Date Time	Relative Day of Onset (End)**	Dur+	Out++	Epi#	Max int##	Rel^	Corr ther^^	Wd AE$
030.001.00188	IPP	RHINITIS / RHINITIS	RESPIRATORY SYSTEM	08MAY2000	23MAY2000	-1 (14)	16 DAYS	RESO	3	MIL	NOT	Yes	No
030.001.00189	I	HEADACHE / HEADACHE	CENTRAL AND PERIPHERAL NERVOUS SYSTEM	04MAY2000	06MAY2000	-5 (-3)	3 DAYS	RESO	2	MIL	NOT	No	No
		JOINT PAIN / ARTHRALGIA	MUSCULOSKELETA L SYSTEM	04MAY2000	20JUL2000	-5 (72)	78 DAYS	RESO	CON	MIL	NOT	No	No
030.001.00198	IPP	CONJUNCTIVITI S / CONJUNCTIVITI S	VISION	05MAY2000	08MAY2000	-4 (-1)	4 DAYS	RESO	CON	MIL	NOT	No	No
		HEADACHE / HEADACHE	CENTRAL AND PERIPHERAL NERVOUS SYSTEM	04MAY2000	07MAY2000	-5 (-2)	4 DAYS	RESO	CON	MIL	NOT	Yes	No
		VERTIGO / VERTIGO	CENTRAL AND PERIPHERAL NERVOUS SYSTEM	07MAY2000	10MAY2000	-2 (1)	4 DAYS	RESO	ITE	MIL	NOT	No	No
030.001.00214	IPP	CHEST PAIN / CHEST PAIN	BODY AS A WHOLE GENERAL	07MAY2000		-2 ()	CONT	ONGO	CON	MIL	NOT	Yes	No

*Popn=Population (I=Intent-to-Treat, PP=Per Protocol)
**No. of days relative to start of prophylactic medication
+Dur=Duration : CONT=Continuing
++Out=Outcome : RESO=Resolved, ONGO=Ongoing
#Epi=Episode: n=No. of Episodes, CON= Constant, ITE= Intermittent
##Max Int = Intensity (Maximum): MIL=Mild, MOD=Moderate, SEV=Severe
^REL=Relationship To Study Procedures: NOT=Not related, UNL= Unlikely, SUS= Suspected, PRB=Probable
^^Corr Ther= Corrective Therapy
$WD AE: Withdrawn Due To A Specific Event
Tafenoquine = Tafenoquine 200mg, Mefloquine = Mefloquine 250mg

SB252263 - 030

Listing B.13

Baseline Signs And Symptoms

Treatment : Mefloquine

Subject Number	Popn*	Verbatim Term / PREFERRED TERM	Primary Body System	Onset Date Time	End Date Time	Relative Day of Onset (End)**	Dur+	Out++	Epi#	Max int##	Rel^	Corr ther^^	Wd AE$
030.001.00214	IPP	COUGH / COUGHING	RESPIRATORY SYSTEM	05MAY2000	23MAY2000	-4 (14)	19 DAYS	RESO	CON	MIL	NOT	No	No
030.001.00224	IPP	SKIN CONDITION / ECZEMA	SKIN AND APPENDAGES	07MAY2000	12MAY2000	-2 (3)	6 DAYS	RESO	CON	MIL	NOT	Yes	No
030.001.00232	IPP	EYE PROBLEM / EYE ABNORMALITY	VISION	05MAY2000	17MAY2000	-5 (7)	13 DAYS	RESO	CON	MIL	NOT	No	No
		LYMPHOCYTOSIS / LYMPHOCYTOSIS	WHITE CELL AND RETICULOENDOTHELIAL SYSTE	18APR2000	10MAY2000	-22 (0)	23 DAYS	RESO	CON	MIL	NOT	No	No
030.001.00237	IPP	MYALGIA / MYALGIA	MUSCULOSKELETAL SYSTEM	07MAY2000	09MAY2000	-3 (-1)	3 DAYS	RESO	CON	MIL	PRB	Yes	No
030.001.00239	IPP	COUGH / COUGHING	RESPIRATORY SYSTEM	05MAY2000	30JUN2000	-5 (51)	57 DAYS	RESO	3	MIL	NOT	No	No
030.001.00244	IPP	ACUTE RHINITIS / RHINITIS	RESPIRATORY SYSTEM	15APR2000	03MAY2000	-25 (-7)	19 DAYS	RESO	2	MIL	NOT	Yes	No

*Popn=Population (I=Intent-to-Treat, PP=Per Protocol)
**No. of days relative to start of prophylactic medication
+Dur=Duration : CONT=Continuing
++Out=Outcome : RESO=Resolved, ONGO=Ongoing
#Epi=Episode: n=No. of Episodes, CON= Constant, ITE= Intermittent
##Max Int = Intensity (Maximum): MIL=Mild, MOD=Moderate, SEV=Severe
^REL=Relationship To Study Procedures: NOT=Not related, UNL= Unlikely, SUS= Suspected, PRB=Probable
^^Corr Ther= Corrective Therapy
$WD AE: Withdrawn Due To A Specific Event
Tafenoquine = Tafenoquine 200mg, Mefloquine = Mefloquine 250mg

SB252263 - 030

Listing B.13

Baseline Signs And Symptoms

Treatment : Mefloquine

Subject Number	Popn*	Verbatim Term / PREFERRED TERM	Primary Body System	Onset Date Time	End Date Time	Relative Day of Onset (End)**	Dur+	Out++	Epi#	Max int##	Rel^	Corr ther^^	Wd AE$
030.001.00255	IPP	URTI / UPPER RESP TRACT INFECTION	RESISTANCE MECHANISM	04MAY2000	18MAY2000	-6 (8)	15 DAYS	RESO	CON	MIL	NOT	Yes	No
030.001.00258	IPP	HEADACHE / HEADACHE	CENTRAL AND PERIPHERAL NERVOUS SYSTEM	16APR2000	22APR2000	-24 (-18)	7 DAYS	RESO	CON	MIL	NOT	Yes	No
030.001.00263	IPP	ABDOMINAL PAIN / ABDOMINAL PAIN	GASTROINTESTIN AL SYSTEM	06MAY2000		-6 ()	CONT	ONGO	2	MIL	SUS	Yes	No
030.001.00274	IPP	CERVICAL STRAIN / INJURY	BODY AS A WHOLE GENERAL	06MAY2000	24MAY2000	-4 (14)	19 DAYS	RESO	2	MIL	NOT	Yes	No
030.001.00281	IPP	UPPER RESPIRATORY TRACT INFECTION / UPPER RESP TRACT INFECTION	RESISTANCE MECHANISM	05MAY2000	07MAY2000	-5 (-3)	3 DAYS	RESO	CON	MIL	NOT	No	No
030.001.00283	IPP	FATIGUE / FATIGUE	BODY AS A WHOLE GENERAL	06MAY2000	17MAY2000	-5 (6)	12 DAYS	RESO	CON	MIL	UNL	No	No

*Popn=Population (I=Intent-to-Treat, PP=Per Protocol)
**No. of days relative to start of prophylactic medication
+Dur=Duration : CONT=Continuing
++Out=Outcome : RESO=Resolved, ONGO=Ongoing
#Epi=Episode: n=No. of Episodes, CON= Constant, ITE= Intermittent
##Max Int = Intensity (Maximum): MIL=Mild, MOD=Moderate, SEV=Severe
^REL=Relationship To Study Procedures: NOT=Not related, UNL= Unlikely, SUS= Suspected, PRB=Probable
^^Corr Ther= Corrective Therapy
$WD AE: Withdrawn Due To A Specific Event
Tafenoquine = Tafenoquine 200mg, Mefloquine = Mefloquine 250mg

SB252263 - 030

Listing B.13

Baseline Signs And Symptoms

Treatment : Mefloquine

Subject Number	Popn*	Verbatim Term / PREFERRED TERM	Primary Body System	Onset Date Time	End Date Time	Relative Day of Onset (End)**	Dur+	Out++	Epi#	Max int##	Rel^	Corr ther^^	Wd AE$
030.001.00286	IPP	CANDIDA BALANITIS / MONILIASIS GENITAL	RESISTANCE MECHANISM	05MAY2000	09MAY2000	-6 (-2)	5 DAYS	RESO	CON	MIL	NOT	No	No
030.001.00289	IPP	UPPER RESPIRATORY TRACT INFECTION / UPPER RESP TRACT INFECTION	RESISTANCE MECHANISM	10MAY2000	15MAY2000	-1 (4)	6 DAYS	RESO	CON	MIL	NOT	Yes	No
030.001.00305	IPP	DYSPEPSIA / DYSPEPSIA	GASTROINTESTINAL SYSTEM	10MAY2000	16MAY2000	-2 (4)	7 DAYS	RESO	CON	MIL	NOT	No	No

*Popn=Population (I=Intent-to-Treat, PP=Per Protocol)
**No. of days relative to start of prophylactic medication
+Dur=Duration : CONT=Continuing
++Out=Outcome : RESO=Resolved, ONGO=Ongoing
#Epi=Episode: n=No. of Episodes, CON= Constant, ITE= Intermittent
##Max Int = Intensity (Maximum): MIL=Mild, MOD=Moderate, SEV=Severe
^REL=Relationship To Study Procedures: NOT=Not related, UNL= Unlikely, SUS= Suspected, PRB=Probable
^^Corr Ther= Corrective Therapy
$WD AE: Withdrawn Due To A Specific Event
Tafenoquine = Tafenoquine 200mg, Mefloquine = Mefloquine 250mg

SB252263 - 030

Listing B.15

Halofantrine Compliance

Treatment : Placebo

Subject Number	Popn*	Date of Dose	Relative Day+	Halofantrine Compliant
030.001.00002	IPP	03MAY2000	-5	Yes
		04MAY2000	-4	
		05MAY2000	-3	
030.001.00005	IPP	03MAY2000	-5	Yes
		04MAY2000	-4	
		05MAY2000	-3	
030.001.00011	IPP	03MAY2000	-5	Yes
		04MAY2000	-4	
		05MAY2000	-3	
030.001.00012	IPP	03MAY2000	-5	Yes
		04MAY2000	-4	
		05MAY2000	-3	
030.001.00015	I	03MAY2000	-5	Yes
		04MAY2000	-4	
		05MAY2000	-3	
030.001.00019	IPP	03MAY2000	-5	Yes
		04MAY2000	-4	
		05MAY2000	-3	
030.001.00020	IPP	03MAY2000	-5	Yes
		04MAY2000	-4	
		05MAY2000	-3	
030.001.00022	IPP	03MAY2000	-5	Yes
		04MAY2000	-4	
		05MAY2000	-3	
030.001.00026	IPP	03MAY2000	-5	Yes
		04MAY2000	-4	
		05MAY2000	-3	

*Popn=Population (I=Intent-To-Treat, PP=Per Protocol)
+Relative to start of prophylactic medication
Tafenoquine = Tafenoquine 200mg, Mefloquine = Mefloquine 250mg

SB252263 - 030

Listing B.15

Halofantrine Compliance

Treatment : Placebo

Subject Number	Popn*	Date of Dose	Relative Day+	Halofantrine Compliant
030.001.00027	IPP	03MAY2000	-5	Yes
		04MAY2000	-4	
		05MAY2000	-3	
030.001.00032	IPP	03MAY2000	-5	Yes
		04MAY2000	-4	
		05MAY2000	-3	
030.001.00033	IPP	03MAY2000	-5	Yes
		04MAY2000	-4	
		05MAY2000	-3	
030.001.00037	IPP	03MAY2000	-5	Yes
		04MAY2000	-4	
		05MAY2000	-3	
030.001.00039	IPP	03MAY2000	-5	Yes
		04MAY2000	-4	
		05MAY2000	-3	
030.001.00041	IPP	03MAY2000	-5	Yes
		04MAY2000	-4	
		05MAY2000	-3	
030.001.00044	IPP	03MAY2000	-5	Yes
		04MAY2000	-4	
		05MAY2000	-3	
030.001.00055	IPP	03MAY2000	-5	Yes
		04MAY2000	-4	
		05MAY2000	-3	
030.001.00056	I	03MAY2000	-5	Yes
		04MAY2000	-4	
		05MAY2000	-3	

*Popn=Population (I=Intent-To-Treat, PP=Per Protocol)
+Relative to start of prophylactic medication
Tafenoquine = Tafenoquine 200mg, Mefloquine = Mefloquine 250mg

SB252263 - 030

Listing B.15

Halofantrine Compliance

Treatment : Placebo

Subject Number	Popn*	Date of Dose	Relative Day+	Halofantrine Compliant
030.001.00057	IPP	03MAY2000	-5	Yes
		04MAY2000	-4	
		05MAY2000	-3	
030.001.00059	IPP	03MAY2000	-5	Yes
		04MAY2000	-4	
		05MAY2000	-3	
030.001.00063	IPP	03MAY2000	-5	Yes
		04MAY2000	-4	
		05MAY2000	-3	
030.001.00064	IPP	03MAY2000	-5	Yes
		04MAY2000	-4	
		05MAY2000	-3	
030.001.00067	IPP	03MAY2000	-5	Yes
		04MAY2000	-4	
		05MAY2000	-3	
030.001.00072	IPP	03MAY2000	-5	Yes
		04MAY2000	-4	
		05MAY2000	-3	
030.001.00076	IPP	03MAY2000	-5	Yes
		04MAY2000	-4	
		05MAY2000	-3	
030.001.00078	IPP	03MAY2000	-5	Yes
		04MAY2000	-4	
		05MAY2000	-3	
030.001.00080	IPP	03MAY2000	-5	Yes
		04MAY2000	-4	
		05MAY2000	-3	

*Popn=Population (I=Intent-To-Treat, PP=Per Protocol)
+Relative to start of prophylactic medication
Tafenoquine = Tafenoquine 200mg, Mefloquine = Mefloquine 250mg

SB252263 - 030

Listing B.15

Halofantrine Compliance

Treatment : Placebo

Subject Number	Popn*	Date of Dose	Relative Day+	Halofantrine Compliant
030.001.00081	IPP	03MAY2000	-5	Yes
		04MAY2000	-4	
		05MAY2000	-3	
030.001.00087	I	03MAY2000	-5	Yes
		04MAY2000	-4	
		05MAY2000	-3	
030.001.00088	IPP	03MAY2000	-5	Yes
		04MAY2000	-4	
		05MAY2000	-3	
030.001.00090	IPP	03MAY2000	-5	Yes
		04MAY2000	-4	
		05MAY2000	-3	
030.001.00092	IPP	03MAY2000	-5	Yes
		04MAY2000	-4	
		05MAY2000	-3	
030.001.00101	IPP	03MAY2000	-5	Yes
		04MAY2000	-4	
		05MAY2000	-3	
030.001.00103	IPP	03MAY2000	-5	Yes
		04MAY2000	-4	
		05MAY2000	-3	
030.001.00106	IPP	03MAY2000	-5	Yes
		04MAY2000	-4	
		05MAY2000	-3	
030.001.00108	IPP	03MAY2000	-5	Yes
		04MAY2000	-4	
		05MAY2000	-3	

*Popn=Population (I=Intent-To-Treat, PP=Per Protocol)
+Relative to start of prophylactic medication
Tafenoquine = Tafenoquine 200mg, Mefloquine = Mefloquine 250mg

SB252263 - 030

Listing B.15

Halofantrine Compliance

Treatment : Placebo

Subject Number	Popn*	Date of Dose	Relative Day+	Halofantrine Compliant
030.001.00114	IPP	03MAY2000	-5	Yes
		04MAY2000	-4	
		05MAY2000	-3	
030.001.00115	IPP	03MAY2000	-5	Yes
		04MAY2000	-4	
		05MAY2000	-3	
030.001.00116	IPP	03MAY2000	-5	Yes
		04MAY2000	-4	
		05MAY2000	-3	
030.001.00119	IPP	04MAY2000	-5	Yes
		05MAY2000	-4	
		06MAY2000	-3	
030.001.00124	IPP	04MAY2000	-5	Yes
		05MAY2000	-4	
		06MAY2000	-3	
030.001.00125	IPP	04MAY2000	-5	Yes
		05MAY2000	-4	
		06MAY2000	-3	
030.001.00127	I	04MAY2000	-5	Yes
		05MAY2000	-4	
		06MAY2000	-3	
030.001.00131	IPP	04MAY2000	-5	Yes
		05MAY2000	-4	
		06MAY2000	-3	
030.001.00136	IPP	04MAY2000	-5	Yes
		05MAY2000	-4	
		06MAY2000	-3	

*Popn=Population (I=Intent-To-Treat, PP=Per Protocol)
+Relative to start of prophylactic medication
Tafenoquine = Tafenoquine 200mg, Mefloquine = Mefloquine 250mg

SB252263 - 030

Listing B.15

Halofantrine Compliance

Treatment : Placebo

Subject Number	Popn*	Date of Dose	Relative Day+	Halofantrine Compliant
030.001.00137	IPP	04MAY2000	-5	Yes
		05MAY2000	-4	
		06MAY2000	-3	
030.001.00140	IPP	04MAY2000	-5	Yes
		05MAY2000	-4	
		06MAY2000	-3	
030.001.00141	IPP	04MAY2000	-5	Yes
		05MAY2000	-4	
		06MAY2000	-3	
030.001.00145	IPP	04MAY2000	-5	Yes
		05MAY2000	-4	
		06MAY2000	-3	
030.001.00151	IPP	04MAY2000	-5	Yes
		05MAY2000	-4	
		06MAY2000	-3	
030.001.00152	IPP	04MAY2000	-5	Yes
		05MAY2000	-4	
		06MAY2000	-3	
030.001.00154	IPP	04MAY2000	-5	Yes
		05MAY2000	-4	
		06MAY2000	-3	
030.001.00160	I	04MAY2000	-5	Yes
		05MAY2000	-4	
		06MAY2000	-3	
030.001.00162	IPP	04MAY2000	-5	Yes
		05MAY2000	-4	
		06MAY2000	-3	

*Popn=Population (I=Intent-To-Treat, PP=Per Protocol)
+Relative to start of prophylactic medication
Tafenoquine = Tafenoquine 200mg, Mefloquine = Mefloquine 250mg

SB252263 - 030

Listing B.15

Halofantrine Compliance

Treatment : Placebo

Subject Number	Popn*	Date of Dose	Relative Day+	Halofantrine Compliant
030.001.00164	IPP	04MAY2000	-5	Yes
		05MAY2000	-4	
		06MAY2000	-3	
030.001.00166	IPP	04MAY2000	-5	Yes
		05MAY2000	-4	
		06MAY2000	-3	
030.001.00169	IPP	04MAY2000	-5	Yes
		05MAY2000	-4	
		06MAY2000	-3	
030.001.00172	IPP	04MAY2000	-5	Yes
		05MAY2000	-4	
		06MAY2000	-3	
030.001.00174	IPP	04MAY2000	-5	Yes
		05MAY2000	-4	
		06MAY2000	-3	
030.001.00175	IPP	04MAY2000	-5	Yes
		05MAY2000	-4	
		06MAY2000	-3	
030.001.00182	I	04MAY2000	-5	Yes
		05MAY2000	-4	
		06MAY2000	-3	
030.001.00183	IPP	04MAY2000	-5	Yes
		05MAY2000	-4	
		06MAY2000	-3	
030.001.00191	IPP	04MAY2000	-5	Yes
		05MAY2000	-4	
		06MAY2000	-3	

*Popn=Population (I=Intent-To-Treat, PP=Per Protocol)
+Relative to start of prophylactic medication
Tafenoquine = Tafenoquine 200mg, Mefloquine = Mefloquine 250mg

SB252263 - 030

Listing B.15

Halofantrine Compliance

Treatment : Placebo

Subject Number	Popn*	Date of Dose	Relative Day+	Halofantrine Compliant
030.001.00192	IPP	04MAY2000	-5	Yes
		05MAY2000	-4	
		06MAY2000	-3	
030.001.00193	IPP	04MAY2000	-5	Yes
		05MAY2000	-4	
		06MAY2000	-3	
030.001.00197	IPP	04MAY2000	-5	Yes
		05MAY2000	-4	
		06MAY2000	-3	
030.001.00202		04MAY2000	-5	Yes
		05MAY2000	-4	
		06MAY2000	-3	
030.001.00204	I	04MAY2000	-5	Yes
		05MAY2000	-4	
		06MAY2000	-3	
030.001.00208	IPP	04MAY2000	-5	Yes
		05MAY2000	-4	
		06MAY2000	-3	
030.001.00211	IPP	04MAY2000	-5	Yes
		05MAY2000	-4	
		06MAY2000	-3	
030.001.00212	IPP	04MAY2000	-5	Yes
		05MAY2000	-4	
		06MAY2000	-3	
030.001.00215	IPP	04MAY2000	-5	Yes
		05MAY2000	-4	
		06MAY2000	-3	

*Popn=Population (I=Intent-To-Treat, PP=Per Protocol)
+Relative to start of prophylactic medication
Tafenoquine = Tafenoquine 200mg, Mefloquine = Mefloquine 250mg

SB252263 - 030

Listing B.15

Halofantrine Compliance

Treatment : Placebo

Subject Number	Popn*	Date of Dose	Relative Day+	Halofantrine Compliant
030.001.00219	IPP	04MAY2000	-5	Yes
		05MAY2000	-4	
		06MAY2000	-3	
030.001.00222	IPP	04MAY2000	-5	Yes
		05MAY2000	-4	
		06MAY2000	-3	
030.001.00225	IPP	06MAY2000	-3	Yes
		07MAY2000	-2	
		08MAY2000	-1	
030.001.00226	IPP	04MAY2000	-5	Yes
		05MAY2000	-4	
		06MAY2000	-3	
030.001.00229	IPP	04MAY2000	-5	Yes
		05MAY2000	-4	
		06MAY2000	-3	
030.001.00234	IPP	05MAY2000	-5	Yes
		06MAY2000	-4	
		07MAY2000	-3	
030.001.00236	IPP	04MAY2000	-7	Yes
		05MAY2000	-6	
		06MAY2000	-5	
030.001.00238	IPP	05MAY2000	-5	Yes
		06MAY2000	-4	
		07MAY2000	-3	
030.001.00242	IPP	05MAY2000	-5	Yes
		06MAY2000	-4	
		07MAY2000	-3	

*Popn=Population (I=Intent-To-Treat, PP=Per Protocol)
+Relative to start of prophylactic medication
Tafenoquine = Tafenoquine 200mg, Mefloquine = Mefloquine 250mg

SB252263 - 030

Listing B.15

Halofantrine Compliance

Treatment : Placebo

Subject Number	Popn*	Date of Dose	Relative Day+	Halofantrine Compliant
030.001.00247	IPP	05MAY2000	-5	Yes
		06MAY2000	-4	
		07MAY2000	-3	
030.001.00248	IPP	05MAY2000	-5	Yes
		06MAY2000	-4	
		07MAY2000	-3	
030.001.00251	IPP	05MAY2000	-5	Yes
		06MAY2000	-4	
		07MAY2000	-3	
030.001.00253	IPP	05MAY2000	-5	Yes
		06MAY2000	-4	
		07MAY2000	-3	
030.001.00256	IPP	05MAY2000	-5	Yes
		06MAY2000	-4	
		07MAY2000	-3	
030.001.00257	IPP	05MAY2000	-7	Yes
		06MAY2000	-6	
		07MAY2000	-5	
030.001.00260	IPP	05MAY2000	-5	Yes
		06MAY2000	-4	
		07MAY2000	-3	
030.001.00268	IPP	05MAY2000	-5	Yes
		06MAY2000	-4	
		07MAY2000	-3	
030.001.00269	IPP	05MAY2000	-5	Yes
		06MAY2000	-4	
		07MAY2000	-3	

*Popn=Population (I=Intent-To-Treat, PP=Per Protocol)
+Relative to start of prophylactic medication
Tafenoquine = Tafenoquine 200mg, Mefloquine = Mefloquine 250mg

SB252263 - 030

Listing B.15

Halofantrine Compliance

Treatment : Placebo

Subject Number	Popn*	Date of Dose	Relative Day+	Halofantrine Compliant
030.001.00270	IPP	04MAY2000	-6	Yes
		05MAY2000	-5	
		06MAY2000	-4	
030.001.00275	IPP	05MAY2000	-5	Yes
		06MAY2000	-4	
		07MAY2000	-3	
030.001.00277	IPP	05MAY2000	-5	Yes
		06MAY2000	-4	
		07MAY2000	-3	
030.001.00278	IPP	05MAY2000	-5	Yes
		06MAY2000	-4	
		07MAY2000	-3	
030.001.00285	IPP	05MAY2000	-6	Yes
		06MAY2000	-5	
		07MAY2000	-4	
030.001.00287	IPP	05MAY2000	-6	Yes
		06MAY2000	-5	
		07MAY2000	-4	
030.001.00291	IPP	05MAY2000	-6	Yes
		06MAY2000	-5	
		07MAY2000	-4	
030.001.00295		05MAY2000	-6	Yes
		06MAY2000	-5	
		07MAY2000	-4	
030.001.00297	IPP	06MAY2000	-5	Yes
		07MAY2000	-4	
		08MAY2000	-3	

*Popn=Population (I=Intent-To-Treat, PP=Per Protocol)
+Relative to start of prophylactic medication
Tafenoquine = Tafenoquine 200mg, Mefloquine = Mefloquine 250mg

SB252263 - 030

Listing B.15

Halofantrine Compliance

Treatment : Placebo

Subject Number	Popn*	Date of Dose	Relative Day+	Halofantrine Compliant
030.001.00300	IPP	05MAY2000	-7	Yes
		06MAY2000	-6	
		07MAY2000	-5	
030.001.00303	IPP	05MAY2000	-7	Yes
		06MAY2000	-6	
		07MAY2000	-5	

*Popn=Population (I=Intent-To-Treat, PP=Per Protocol)
+Relative to start of prophylactic medication
Tafenoquine = Tafenoquine 200mg, Mefloquine = Mefloquine 250mg

SB252263 - 030

Listing B.15

Halofantrine Compliance

Treatment : Tafenoquine

Subject Number	Popn*	Date of Dose	Relative Day+	Halofantrine Compliant
030.001.00001	IPP	03MAY2000	-5	Yes
		04MAY2000	-4	
		05MAY2000	-3	
030.001.00004	IPP	03MAY2000	-5	Yes
		04MAY2000	-4	
		05MAY2000	-3	
030.001.00006	IPP	03MAY2000	-5	Yes
		04MAY2000	-4	
		05MAY2000	-3	
030.001.00009	IPP	03MAY2000	-5	Yes
		04MAY2000	-4	
		05MAY2000	-3	
030.001.00014	IPP	03MAY2000	-5	Yes
		04MAY2000	-4	
		05MAY2000	-3	
030.001.00016	IPP	03MAY2000	-5	Yes
		04MAY2000	-4	
		05MAY2000	-3	
030.001.00017	IPP	03MAY2000	-5	Yes
		04MAY2000	-4	
		05MAY2000	-3	
030.001.00024	IPP	03MAY2000	-5	Yes
		04MAY2000	-4	
		05MAY2000	-3	
030.001.00025	IPP	03MAY2000	-5	Yes
		04MAY2000	-4	
		05MAY2000	-3	

*Popn=Population (I=Intent-To-Treat, PP=Per Protocol)
+Relative to start of prophylactic medication
Tafenoquine = Tafenoquine 200mg, Mefloquine = Mefloquine 250mg

SB252263 - 030

Listing B.15

Halofantrine Compliance

Treatment : Tafenoquine

Subject Number	Popn*	Date of Dose	Relative Day+	Halofantrine Compliant
030.001.00028	IPP	03MAY2000	-5	Yes
		04MAY2000	-4	
		05MAY2000	-3	
030.001.00029	IPP	03MAY2000	-5	Yes
		04MAY2000	-4	
		05MAY2000	-3	
030.001.00030	IPP	03MAY2000	-5	Yes
		04MAY2000	-4	
		05MAY2000	-3	
030.001.00040	IPP	03MAY2000	-5	Yes
		04MAY2000	-4	
		05MAY2000	-3	
030.001.00042	IPP	03MAY2000	-5	Yes
		04MAY2000	-4	
		05MAY2000	-3	
030.001.00043	IPP	03MAY2000	-5	Yes
		04MAY2000	-4	
		05MAY2000	-3	
030.001.00048	IPP	03MAY2000	-5	Yes
		04MAY2000	-4	
		05MAY2000	-3	
030.001.00050	IPP	03MAY2000	-5	Yes
		04MAY2000	-4	
		05MAY2000	-3	
030.001.00051	IPP	03MAY2000	-5	Yes
		04MAY2000	-4	
		05MAY2000	-3	

*Popn=Population (I=Intent-To-Treat, PP=Per Protocol)
+Relative to start of prophylactic medication
Tafenoquine = Tafenoquine 200mg, Mefloquine = Mefloquine 250mg

SB252263 - 030

Listing B.15

Halofantrine Compliance

Treatment : Tafenoquine

Subject Number	Popn*	Date of Dose	Relative Day+	Halofantrine Compliant
030.001.00058	IPP	03MAY2000	-5	Yes
		04MAY2000	-4	
		05MAY2000	-3	
030.001.00060	IPP	03MAY2000	-5	Yes
		04MAY2000	-4	
		05MAY2000	-3	
030.001.00061	IPP	03MAY2000	-5	Yes
		04MAY2000	-4	
		05MAY2000	-3	
030.001.00065	IPP	03MAY2000	-5	Yes
		04MAY2000	-4	
		05MAY2000	-3	
030.001.00068	IPP	03MAY2000	-5	Yes
		04MAY2000	-4	
		05MAY2000	-3	
030.001.00070	IPP	03MAY2000	-5	Yes
		04MAY2000	-4	
		05MAY2000	-3	
030.001.00077	IPP	03MAY2000	-5	Yes
		04MAY2000	-4	
		05MAY2000	-3	
030.001.00079	IPP	03MAY2000	-5	Yes
		04MAY2000	-4	
		05MAY2000	-3	
030.001.00082		03MAY2000	-5	Yes
		04MAY2000	-4	
		05MAY2000	-3	

*Popn=Population (I=Intent-To-Treat, PP=Per Protocol)
+Relative to start of prophylactic medication
Tafenoquine = Tafenoquine 200mg, Mefloquine = Mefloquine 250mg

SB252263 - 030

Listing B.15

Halofantrine Compliance

Treatment : Tafenoquine

Subject Number	Popn*	Date of Dose	Relative Day+	Halofantrine Compliant
030.001.00084	IPP	03MAY2000	-5	Yes
		04MAY2000	-4	
		05MAY2000	-3	
030.001.00085	IPP	03MAY2000	-5	Yes
		04MAY2000	-4	
		05MAY2000	-3	
030.001.00086	IPP	03MAY2000	-5	Yes
		04MAY2000	-4	
		05MAY2000	-3	
030.001.00093	IPP	03MAY2000	-5	Yes
		04MAY2000	-4	
		05MAY2000	-3	
030.001.00094	I	03MAY2000	-5	Yes
		04MAY2000	-4	
		05MAY2000	-3	
030.001.00100	IPP	03MAY2000	-5	Yes
		04MAY2000	-4	
		05MAY2000	-3	
030.001.00102	IPP	03MAY2000	-5	Yes
		04MAY2000	-4	
		05MAY2000	-3	
030.001.00104	IPP	03MAY2000	-5	Yes
		04MAY2000	-4	
		05MAY2000	-3	
030.001.00107	IPP	03MAY2000	-5	Yes
		04MAY2000	-4	
		05MAY2000	-3	

*Popn=Population (I=Intent-To-Treat, PP=Per Protocol)
+Relative to start of prophylactic medication
Tafenoquine = Tafenoquine 200mg, Mefloquine = Mefloquine 250mg

SB252263 - 030

Listing B.15

Halofantrine Compliance

Treatment : Tafenoquine

Subject Number	Popn*	Date of Dose	Relative Day+	Halofantrine Compliant
030.001.00109	IPP	03MAY2000	-5	Yes
		04MAY2000	-4	
		05MAY2000	-3	
030.001.00111	IPP	03MAY2000	-5	Yes
		04MAY2000	-4	
		05MAY2000	-3	
030.001.00112	IPP	02MAY2000	-6	Yes
		03MAY2000	-5	
		04MAY2000	-4	
030.001.00118	IPP	04MAY2000	-5	Yes
		05MAY2000	-4	
		06MAY2000	-3	
030.001.00122	IPP	04MAY2000	-5	Yes
		05MAY2000	-4	
		06MAY2000	-3	
030.001.00126	IPP	04MAY2000	-5	Yes
		05MAY2000	-4	
		06MAY2000	-3	
030.001.00128	IPP	04MAY2000	-5	Yes
		05MAY2000	-4	
		06MAY2000	-3	
030.001.00129	IPP	04MAY2000	-5	Yes
		05MAY2000	-4	
		06MAY2000	-3	
030.001.00133	IPP	04MAY2000	-5	Yes
		05MAY2000	-4	
		06MAY2000	-3	

*Popn=Population (I=Intent-To-Treat, PP=Per Protocol)
+Relative to start of prophylactic medication
Tafenoquine = Tafenoquine 200mg, Mefloquine = Mefloquine 250mg

SB252263 - 030

Listing B.15

Halofantrine Compliance

Treatment : Tafenoquine

Subject Number	Popn*	Date of Dose	Relative Day+	Halofantrine Compliant
030.001.00135	IPP	04MAY2000	-5	Yes
		05MAY2000	-4	
		06MAY2000	-3	
030.001.00138	IPP	04MAY2000	-5	Yes
		05MAY2000	-4	
		06MAY2000	-3	
030.001.00139	IPP	04MAY2000	-5	Yes
		05MAY2000	-4	
		06MAY2000	-3	
030.001.00148	IPP	04MAY2000	-5	Yes
		05MAY2000	-4	
		06MAY2000	-3	
030.001.00149	IPP	04MAY2000	-5	Yes
		05MAY2000	-4	
		06MAY2000	-3	
030.001.00150	IPP	04MAY2000	-5	Yes
		05MAY2000	-4	
		06MAY2000	-3	
030.001.00155	IPP	04MAY2000	-5	Yes
		05MAY2000	-4	
		06MAY2000	-3	
030.001.00157	IPP	04MAY2000	-5	Yes
		05MAY2000	-4	
		06MAY2000	-3	
030.001.00158	IPP	04MAY2000	-5	Yes
		05MAY2000	-4	
		06MAY2000	-3	

*Popn=Population (I=Intent-To-Treat, PP=Per Protocol)
+Relative to start of prophylactic medication
Tafenoquine = Tafenoquine 200mg, Mefloquine = Mefloquine 250mg

SB252263 - 030

Listing B.15

Halofantrine Compliance

Treatment : Tafenoquine

Subject Number	Popn*	Date of Dose	Relative Day+	Halofantrine Compliant
030.001.00159	IPP	04MAY2000	-5	Yes
		05MAY2000	-4	
		06MAY2000	-3	
030.001.00161	IPP	04MAY2000	-5	Yes
		05MAY2000	-4	
		06MAY2000	-3	
030.001.00171	IPP	04MAY2000	-5	Yes
		05MAY2000	-4	
		06MAY2000	-3	
030.001.00173	I	04MAY2000	-5	Yes
		05MAY2000	-4	
		06MAY2000	-3	
030.001.00176	IPP	04MAY2000	-5	Yes
		05MAY2000	-4	
		06MAY2000	-3	
030.001.00179	I	04MAY2000	-5	Yes
		05MAY2000	-4	
		06MAY2000	-3	
030.001.00184	IPP	04MAY2000	-5	Yes
		05MAY2000	-4	
		06MAY2000	-3	
030.001.00186	IPP	04MAY2000	-5	Yes
		05MAY2000	-4	
		06MAY2000	-3	
030.001.00187	I	04MAY2000	-5	Yes
		05MAY2000	-4	
		06MAY2000	-3	

*Popn=Population (I=Intent-To-Treat, PP=Per Protocol)
+Relative to start of prophylactic medication
Tafenoquine = Tafenoquine 200mg, Mefloquine = Mefloquine 250mg

SB252263 - 030

Listing B.15

Halofantrine Compliance

Treatment : Tafenoquine

Subject Number	Popn*	Date of Dose	Relative Day+	Halofantrine Compliant
030.001.00190	IPP	04MAY2000	-5	Yes
		05MAY2000	-4	
		06MAY2000	-3	
030.001.00194	IPP	04MAY2000	-5	Yes
		05MAY2000	-4	
		06MAY2000	-3	
030.001.00195	IPP	04MAY2000	-5	Yes
		05MAY2000	-4	
		06MAY2000	-3	
030.001.00201	IPP	04MAY2000	-5	Yes
		05MAY2000	-4	
		06MAY2000	-3	
030.001.00203	IPP	03MAY2000	-6	Yes
		04MAY2000	-5	
		05MAY2000	-4	
030.001.00205	IPP	06MAY2000	-3	Yes
		07MAY2000	-2	
		08MAY2000	-1	
030.001.00207	IPP	04MAY2000	-5	Yes
		05MAY2000	-4	
		06MAY2000	-3	
030.001.00209	IPP	04MAY2000	-5	Yes
		05MAY2000	-4	
		06MAY2000	-3	
030.001.00210	IPP	04MAY2000	-5	Yes
		05MAY2000	-4	
		06MAY2000	-3	

*Popn=Population (I=Intent-To-Treat, PP=Per Protocol)
+Relative to start of prophylactic medication
Tafenoquine = Tafenoquine 200mg, Mefloquine = Mefloquine 250mg

SB252263 - 030

Listing B.15

Halofantrine Compliance

Treatment : Tafenoquine

Subject Number	Popn*	Date of Dose	Relative Day+	Halofantrine Compliant
030.001.00217	IPP	04MAY2000	-5	Yes
		05MAY2000	-4	
		06MAY2000	-3	
030.001.00218	IPP	04MAY2000	-5	Yes
		05MAY2000	-4	
		06MAY2000	-3	
030.001.00220	IPP	04MAY2000	-5	Yes
		05MAY2000	-4	
		06MAY2000	-3	
030.001.00228	IPP	04MAY2000	-5	Yes
		05MAY2000	-4	
		06MAY2000	-3	
030.001.00230	IPP	04MAY2000	-5	Yes
		05MAY2000	-4	
		06MAY2000	-3	
030.001.00231	IPP	04MAY2000	-5	Yes
		05MAY2000	-4	
		06MAY2000	-3	
030.001.00233	IPP	05MAY2000	-5	Yes
		06MAY2000	-4	
		07MAY2000	-3	
030.001.00240	IPP	05MAY2000	-5	Yes
		06MAY2000	-4	
		07MAY2000	-3	
030.001.00243	IPP	05MAY2000	-5	Yes
		06MAY2000	-4	
		07MAY2000	-3	

*Popn=Population (I=Intent-To-Treat, PP=Per Protocol)
+Relative to start of prophylactic medication
Tafenoquine = Tafenoquine 200mg, Mefloquine = Mefloquine 250mg

SB252263 - 030

Listing B.15

Halofantrine Compliance

Treatment : Tafenoquine

Subject Number	Popn*	Date of Dose	Relative Day+	Halofantrine Compliant
030.001.00246	IPP	05MAY2000	-5	Yes
		06MAY2000	-4	
		07MAY2000	-3	
030.001.00249	IPP	05MAY2000	-5	Yes
		06MAY2000	-4	
		07MAY2000	-3	
030.001.00252	IPP	05MAY2000	-5	Yes
		06MAY2000	-4	
		07MAY2000	-3	
030.001.00254	I	05MAY2000	-5	Yes
		06MAY2000	-4	
		07MAY2000	-3	
030.001.00259	IPP	05MAY2000	-5	Yes
		06MAY2000	-4	
		07MAY2000	-3	
030.001.00261		05MAY2000	-5	Yes
		06MAY2000	-4	
		07MAY2000	-3	
030.001.00264	IPP	05MAY2000	-5	Yes
		06MAY2000	-4	
		07MAY2000	-3	
030.001.00266	I	05MAY2000	-5	Yes
		06MAY2000	-4	
		07MAY2000	-3	
030.001.00267	IPP	05MAY2000	-5	Yes
		06MAY2000	-4	
		07MAY2000	-3	

*Popn=Population (I=Intent-To-Treat, PP=Per Protocol)
+Relative to start of prophylactic medication
Tafenoquine = Tafenoquine 200mg, Mefloquine = Mefloquine 250mg

SB252263 - 030

Listing B.15

Halofantrine Compliance

Treatment : Tafenoquine

Subject Number	Popn*	Date of Dose	Relative Day+	Halofantrine Compliant
030.001.00271	I	05MAY2000	-5	Yes
		06MAY2000	-4	
		07MAY2000	-3	
030.001.00272	IPP	05MAY2000	-5	Yes
		06MAY2000	-4	
		07MAY2000	-3	
030.001.00279	IPP	04MAY2000	-7	Yes
		05MAY2000	-6	
		06MAY2000	-5	
030.001.00282	IPP	05MAY2000	-5	Yes
		06MAY2000	-4	
		07MAY2000	-3	
030.001.00284	IPP	07MAY2000	-4	Yes
		08MAY2000	-3	
		09MAY2000	-2	
030.001.00288	IPP	05MAY2000	-6	Yes
		06MAY2000	-5	
		07MAY2000	-4	
030.001.00293	IPP	05MAY2000	-6	Yes
		06MAY2000	-5	
		07MAY2000	-4	
030.001.00294	IPP	05MAY2000	-6	Yes
		06MAY2000	-5	
		07MAY2000	-4	
030.001.00296	IPP	08MAY2000	-3	Yes
		09MAY2000	-2	
		10MAY2000	-1	

*Popn=Population (I=Intent-To-Treat, PP=Per Protocol)
+Relative to start of prophylactic medication
Tafenoquine = Tafenoquine 200mg, Mefloquine = Mefloquine 250mg

SB252263 - 030

Listing B.15

Halofantrine Compliance

Treatment : Tafenoquine

Subject Number	Popn*	Date of Dose	Relative Day+	Halofantrine Compliant
030.001.00298	IPP	05MAY2000	-7	Yes
		06MAY2000	-6	
		07MAY2000	-5	
030.001.00301	IPP	05MAY2000	-7	Yes
		06MAY2000	-6	
		07MAY2000	-5	
030.001.00302	I	05MAY2000	-7	Yes
		06MAY2000	-6	
		07MAY2000	-5	
030.001.00304	IPP	05MAY2000	-7	Yes
		06MAY2000	-6	
		07MAY2000	-5	
030.001.00306	IPP	05MAY2000	-7	Yes
		06MAY2000	-6	
		07MAY2000	-5	

*Popn=Population (I=Intent-To-Treat, PP=Per Protocol)
+Relative to start of prophylactic medication
Tafenoquine = Tafenoquine 200mg, Mefloquine = Mefloquine 250mg

SB252263 - 030

Listing B.15

Halofantrine Compliance

Treatment : Mefloquine

Subject Number	Popn*	Date of Dose	Relative Day+	Halofantrine Compliant
030.001.00003	I	03MAY2000	-5	Yes
		04MAY2000	-4	
		05MAY2000	-3	
030.001.00007	IPP	03MAY2000	-5	Yes
		04MAY2000	-4	
		05MAY2000	-3	
030.001.00008	IPP	03MAY2000	-5	Yes
		04MAY2000	-4	
		05MAY2000	-3	
030.001.00010	IPP	03MAY2000	-5	Yes
		04MAY2000	-4	
		05MAY2000	-3	
030.001.00013	IPP	03MAY2000	-5	Yes
		04MAY2000	-4	
		05MAY2000	-3	
030.001.00018	IPP	03MAY2000	-5	Yes
		04MAY2000	-4	
		05MAY2000	-3	
030.001.00021	IPP	03MAY2000	-5	Yes
		04MAY2000	-4	
		05MAY2000	-3	
030.001.00023	I	03MAY2000	-5	Yes
		04MAY2000	-4	
		05MAY2000	-3	
030.001.00031	IPP	03MAY2000	-5	Yes
		04MAY2000	-4	
		05MAY2000	-3	

*Popn=Population (I=Intent-To-Treat, PP=Per Protocol)
+Relative to start of prophylactic medication
Tafenoquine = Tafenoquine 200mg, Mefloquine = Mefloquine 250mg

Listing B.15

Halofantrine Compliance

Treatment : Mefloquine

Subject Number	Popn*	Date of Dose	Relative Day+	Halofantrine Compliant
030.001.00034	IPP	03MAY2000	-5	Yes
		04MAY2000	-4	
		05MAY2000	-3	
030.001.00035		03MAY2000	-5	Yes
		04MAY2000	-4	
		05MAY2000	-3	
030.001.00036	I	03MAY2000	-5	Yes
		04MAY2000	-4	
		05MAY2000	-3	
030.001.00038	IPP	03MAY2000	-5	Yes
		04MAY2000	-4	
		05MAY2000	-3	
030.001.00045	IPP	03MAY2000	-5	Yes
		04MAY2000	-4	
		05MAY2000	-3	
030.001.00046	IPP	03MAY2000	-5	Yes
		04MAY2000	-4	
		05MAY2000	-3	
030.001.00047	IPP	03MAY2000	-5	Yes
		04MAY2000	-4	
		05MAY2000	-3	
030.001.00049	IPP	03MAY2000	-5	Yes
		04MAY2000	-4	
		05MAY2000	-3	
030.001.00052	IPP	03MAY2000	-5	Yes
		04MAY2000	-4	
		05MAY2000	-3	

*Popn=Population (I=Intent-To-Treat, PP=Per Protocol)
+Relative to start of prophylactic medication
Tafenoquine = Tafenoquine 200mg, Mefloquine = Mefloquine 250mg

SB252263 - 030

Listing B.15

Halofantrine Compliance

Treatment : Mefloquine

Subject Number	Popn*	Date of Dose	Relative Day+	Halofantrine Compliant
030.001.00053	IPP	03MAY2000	-5	Yes
		04MAY2000	-4	
		05MAY2000	-3	
030.001.00054	IPP	03MAY2000	-5	Yes
		04MAY2000	-4	
		05MAY2000	-3	
030.001.00062	IPP	03MAY2000	-5	Yes
		04MAY2000	-4	
		05MAY2000	-3	
030.001.00066	IPP	03MAY2000	-5	Yes
		04MAY2000	-4	
		05MAY2000	-3	
030.001.00069	I	03MAY2000	-5	Yes
		04MAY2000	-4	
		05MAY2000	-3	
030.001.00071	IPP	03MAY2000	-5	Yes
		04MAY2000	-4	
		05MAY2000	-3	
030.001.00073	IPP	03MAY2000	-5	Yes
		04MAY2000	-4	
		05MAY2000	-3	
030.001.00074	IPP	03MAY2000	-5	Yes
		04MAY2000	-4	
		05MAY2000	-3	
030.001.00075	IPP	03MAY2000	-5	Yes
		04MAY2000	-4	
		05MAY2000	-3	

*Popn=Population (I=Intent-To-Treat, PP=Per Protocol)
+Relative to start of prophylactic medication
Tafenoquine = Tafenoquine 200mg, Mefloquine = Mefloquine 250mg

SB252263 - 030

Listing B.15

Halofantrine Compliance

Treatment : Mefloquine

Subject Number	Popn*	Date of Dose	Relative Day+	Halofantrine Compliant
030.001.00083	IPP	03MAY2000	-5	Yes
		04MAY2000	-4	
		05MAY2000	-3	
030.001.00089	IPP	03MAY2000	-5	Yes
		04MAY2000	-4	
		05MAY2000	-3	
030.001.00091	IPP	03MAY2000	-5	Yes
		04MAY2000	-4	
		05MAY2000	-3	
030.001.00095	IPP	03MAY2000	-5	Yes
		04MAY2000	-4	
		05MAY2000	-3	
030.001.00096	IPP	03MAY2000	-5	Yes
		04MAY2000	-4	
		05MAY2000	-3	
030.001.00097	IPP	03MAY2000	-5	Yes
		04MAY2000	-4	
		05MAY2000	-3	
030.001.00098	IPP	03MAY2000	-5	Yes
		04MAY2000	-4	
		05MAY2000	-3	
030.001.00099	IPP	03MAY2000	-5	Yes
		04MAY2000	-4	
		05MAY2000	-3	
030.001.00105	IPP	03MAY2000	-5	Yes
		04MAY2000	-4	
		05MAY2000	-3	

*Popn=Population (I=Intent-To-Treat, PP=Per Protocol)
+Relative to start of prophylactic medication
Tafenoquine = Tafenoquine 200mg, Mefloquine = Mefloquine 250mg

SB252263 - 030

Listing B.15

Halofantrine Compliance

Treatment : Mefloquine

Subject Number	Popn*	Date of Dose	Relative Day+	Halofantrine Compliant
030.001.00110	IPP	03MAY2000	-5	Yes
		04MAY2000	-4	
		05MAY2000	-3	
030.001.00113	IPP	03MAY2000	-5	Yes
		04MAY2000	-4	
		05MAY2000	-3	
030.001.00117	IPP	03MAY2000	-5	Yes
		04MAY2000	-4	
		05MAY2000	-3	
030.001.00120	IPP	04MAY2000	-5	Yes
		05MAY2000	-4	
		06MAY2000	-3	
030.001.00121	IPP	04MAY2000	-5	Yes
		05MAY2000	-4	
		06MAY2000	-3	
030.001.00123	I	04MAY2000	-5	Yes
		05MAY2000	-4	
		06MAY2000	-3	
030.001.00130	IPP	04MAY2000	-5	Yes
		05MAY2000	-4	
		06MAY2000	-3	
030.001.00132	IPP	04MAY2000	-5	Yes
		05MAY2000	-4	
		06MAY2000	-3	
030.001.00134	IPP	04MAY2000	-5	Yes
		05MAY2000	-4	
		06MAY2000	-3	

*Popn=Population (I=Intent-To-Treat, PP=Per Protocol)
+Relative to start of prophylactic medication
Tafenoquine = Tafenoquine 200mg, Mefloquine = Mefloquine 250mg

SB252263 - 030

Listing B.15

Halofantrine Compliance

Treatment : Mefloquine

Subject Number	Popn*	Date of Dose	Relative Day+	Halofantrine Compliant
030.001.00142	IPP	04MAY2000	-5	Yes
		05MAY2000	-4	
		06MAY2000	-3	
030.001.00143	IPP	04MAY2000	-5	Yes
		05MAY2000	-4	
		06MAY2000	-3	
030.001.00144	IPP	04MAY2000	-5	Yes
		05MAY2000	-4	
		06MAY2000	-3	
030.001.00146	I	04MAY2000	-5	Yes
		05MAY2000	-4	
		06MAY2000	-3	
030.001.00147	IPP	04MAY2000	-5	Yes
		05MAY2000	-4	
		06MAY2000	-3	
030.001.00153	IPP	04MAY2000	-5	Yes
		05MAY2000	-4	
		06MAY2000	-3	
030.001.00156	IPP	04MAY2000	-5	Yes
		05MAY2000	-4	
		06MAY2000	-3	
030.001.00163	IPP	04MAY2000	-5	Yes
		05MAY2000	-4	
		06MAY2000	-3	
030.001.00165	IPP	04MAY2000	-5	Yes
		05MAY2000	-4	
		06MAY2000	-3	

*Popn=Population (I=Intent-To-Treat, PP=Per Protocol)
+Relative to start of prophylactic medication
Tafenoquine = Tafenoquine 200mg, Mefloquine = Mefloquine 250mg

SB252263 - 030

Listing B.15

Halofantrine Compliance

Treatment : Mefloquine

Subject Number	Popn*	Date of Dose	Relative Day+	Halofantrine Compliant
030.001.00167	IPP	04MAY2000	-5	Yes
		05MAY2000	-4	
		06MAY2000	-3	
030.001.00168	IPP	04MAY2000	-5	Yes
		05MAY2000	-4	
		06MAY2000	-3	
030.001.00170	IPP	04MAY2000	-5	Yes
		05MAY2000	-4	
		06MAY2000	-3	
030.001.00177	IPP	04MAY2000	-5	Yes
		05MAY2000	-4	
		06MAY2000	-3	
030.001.00178	I	04MAY2000	-5	Yes
		05MAY2000	-4	
		06MAY2000	-3	
030.001.00180	IPP	04MAY2000	-5	Yes
		05MAY2000	-4	
		06MAY2000	-3	
030.001.00181	IPP	04MAY2000	-5	Yes
		05MAY2000	-4	
		06MAY2000	-3	
030.001.00185	IPP	04MAY2000	-5	Yes
		05MAY2000	-4	
		06MAY2000	-3	
030.001.00188	IPP	04MAY2000	-5	Yes
		05MAY2000	-4	
		06MAY2000	-3	

*Popn=Population (I=Intent-To-Treat, PP=Per Protocol)
+Relative to start of prophylactic medication
Tafenoquine = Tafenoquine 200mg, Mefloquine = Mefloquine 250mg

SB252263 - 030

Listing B.15

Halofantrine Compliance

Treatment : Mefloquine

Subject Number	Popn*	Date of Dose	Relative Day+	Halofantrine Compliant
030.001.00189	I	04MAY2000	-5	No
		06MAY2000	-3	
		07MAY2000	-2	
030.001.00196	IPP	04MAY2000	-5	Yes
		05MAY2000	-4	
		06MAY2000	-3	
030.001.00198	IPP	04MAY2000	-5	Yes
		05MAY2000	-4	
		06MAY2000	-3	
030.001.00199	IPP	04MAY2000	-5	Yes
		05MAY2000	-4	
		06MAY2000	-3	
030.001.00200	IPP	04MAY2000	-5	Yes
		05MAY2000	-4	
		06MAY2000	-3	
030.001.00206	I	04MAY2000	-5	Yes
		05MAY2000	-4	
		06MAY2000	-3	
030.001.00213	IPP	04MAY2000	-5	Yes
		05MAY2000	-4	
		06MAY2000	-3	
030.001.00214	IPP	04MAY2000	-5	Yes
		05MAY2000	-4	
		06MAY2000	-3	
030.001.00216	IPP	04MAY2000	-5	Yes
		05MAY2000	-4	
		06MAY2000	-3	

*Popn=Population (I=Intent-To-Treat, PP=Per Protocol)
+Relative to start of prophylactic medication
Tafenoquine = Tafenoquine 200mg, Mefloquine = Mefloquine 250mg

SB252263 - 030

Listing B.15

Halofantrine Compliance

Treatment : Mefloquine

Subject Number	Popn*	Date of Dose	Relative Day+	Halofantrine Compliant
030.001.00221	IPP	04MAY2000	-5	Yes
		05MAY2000	-4	
		06MAY2000	-3	
030.001.00223	IPP	04MAY2000	-5	Yes
		05MAY2000	-4	
		06MAY2000	-3	
030.001.00224	IPP	04MAY2000	-5	Yes
		05MAY2000	-4	
		06MAY2000	-3	
030.001.00227	IPP	04MAY2000	-5	Yes
		05MAY2000	-4	
		06MAY2000	-3	
030.001.00232	IPP	05MAY2000	-5	Yes
		06MAY2000	-4	
		07MAY2000	-3	
030.001.00235	IPP	05MAY2000	-5	Yes
		06MAY2000	-4	
		07MAY2000	-3	
030.001.00237	IPP	05MAY2000	-5	Yes
		06MAY2000	-4	
		07MAY2000	-3	
030.001.00239	IPP	05MAY2000	-5	Yes
		06MAY2000	-4	
		07MAY2000	-3	
030.001.00241	IPP	05MAY2000	-5	Yes
		06MAY2000	-4	
		07MAY2000	-3	

*Popn=Population (I=Intent-To-Treat, PP=Per Protocol)
+Relative to start of prophylactic medication
Tafenoquine = Tafenoquine 200mg, Mefloquine = Mefloquine 250mg

SB252263 - 030

Listing B.15

Halofantrine Compliance

Treatment : Mefloquine

Subject Number	Popn*	Date of Dose	Relative Day+	Halofantrine Compliant
030.001.00244	IPP	05MAY2000	-5	Yes
		06MAY2000	-4	
		07MAY2000	-3	
030.001.00245	IPP	05MAY2000	-5	Yes
		06MAY2000	-4	
		07MAY2000	-3	
030.001.00250		05MAY2000	-5	Yes
		06MAY2000	-4	
		07MAY2000	-3	
030.001.00255	IPP	05MAY2000	-5	Yes
		06MAY2000	-4	
		07MAY2000	-3	
030.001.00258	IPP	05MAY2000	-5	Yes
		06MAY2000	-4	
		07MAY2000	-3	
030.001.00262	IPP	05MAY2000	-5	Yes
		06MAY2000	-4	
		07MAY2000	-3	
030.001.00263	IPP	05MAY2000	-7	Yes
		06MAY2000	-6	
		07MAY2000	-5	
030.001.00265	IPP	05MAY2000	-5	Yes
		06MAY2000	-4	
		07MAY2000	-3	
030.001.00273	IPP	05MAY2000	-5	Yes
		06MAY2000	-4	
		07MAY2000	-3	

*Popn=Population (I=Intent-To-Treat, PP=Per Protocol)
+Relative to start of prophylactic medication
Tafenoquine = Tafenoquine 200mg, Mefloquine = Mefloquine 250mg

SB252263 - 030

Listing B.15

Halofantrine Compliance

Treatment : Mefloquine

Subject Number	Popn*	Date of Dose	Relative Day+	Halofantrine Compliant
030.001.00274	IPP	05MAY2000	-5	Yes
		06MAY2000	-4	
		07MAY2000	-3	
030.001.00276	IPP	05MAY2000	-5	Yes
		06MAY2000	-4	
		07MAY2000	-3	
030.001.00280	IPP	05MAY2000	-5	Yes
		06MAY2000	-4	
		07MAY2000	-3	
030.001.00281	IPP	05MAY2000	-5	Yes
		06MAY2000	-4	
		07MAY2000	-3	
030.001.00283	IPP	05MAY2000	-6	Yes
		06MAY2000	-5	
		07MAY2000	-4	
030.001.00286	IPP	05MAY2000	-6	Yes
		06MAY2000	-5	
		07MAY2000	-4	
030.001.00289	IPP	05MAY2000	-6	Yes
		06MAY2000	-5	
		07MAY2000	-4	
030.001.00290	IPP	05MAY2000	-6	Yes
		06MAY2000	-5	
		07MAY2000	-4	
030.001.00292	IPP	05MAY2000	-6	Yes
		06MAY2000	-5	
		07MAY2000	-4	

*Popn=Population (I=Intent-To-Treat, PP=Per Protocol)
+Relative to start of prophylactic medication
Tafenoquine = Tafenoquine 200mg, Mefloquine = Mefloquine 250mg

SB252263 - 030

Listing B.15

Halofantrine Compliance

Treatment : Mefloquine

Subject Number	Popn*	Date of Dose	Relative Day+	Halofantrine Compliant
030.001.00299	IPP	05MAY2000	-7	Yes
		06MAY2000	-6	
		07MAY2000	-5	
030.001.00305	IPP	09MAY2000	-3	Yes
		10MAY2000	-2	
		11MAY2000	-1	

*Popn=Population (I=Intent-To-Treat, PP=Per Protocol)
+Relative to start of prophylactic medication
Tafenoquine = Tafenoquine 200mg, Mefloquine = Mefloquine 250mg

SB252263 - 030

Listing B.16

Prophylactic Medication Compliance

Treatment : Placebo

Subject Number	Popn*	First Dose Prophylactic Medication	Last Dose Prophylactic Medication	Number of Loading Doses	Loading Dose Compliant**	Number of Weekly Doses	Weekly Dosing Compliant+	Prophylactic Medication Compliant++
030.001.00002	IPP	08MAY2000	19JUN2000	3	Yes	6	Yes	Yes
030.001.00005	IPP	08MAY2000	03JUL2000	3	Yes	8	Yes	Yes
030.001.00011	IPP	08MAY2000	29MAY2000	3	Yes	3	Yes	Yes
030.001.00012	IPP	08MAY2000	26JUN2000	3	Yes	7	Yes	Yes
030.001.00015	I	08MAY2000	17JUL2000	3	Yes	10	Yes	Yes
030.001.00019	IPP	08MAY2000	10JUL2000	3	Yes	9	Yes	Yes
030.001.00020	IPP	08MAY2000	10JUL2000	3	Yes	9	Yes	Yes
030.001.00022	IPP	08MAY2000	22MAY2000	3	Yes	2	Yes	Yes
030.001.00026	IPP	08MAY2000	22MAY2000	3	Yes	2	Yes	Yes
030.001.00027	IPP	08MAY2000	22MAY2000	3	Yes	2	Yes	Yes
030.001.00032	IPP	08MAY2000	03JUL2000	3	Yes	8	Yes	Yes
030.001.00033	IPP	08MAY2000	22MAY2000	3	Yes	2	Yes	Yes
030.001.00037	IPP	08MAY2000	29MAY2000	3	Yes	3	Yes	Yes
030.001.00039	IPP	08MAY2000	12JUN2000	3	Yes	5	Yes	Yes
030.001.00041	IPP	08MAY2000	12JUN2000	3	Yes	5	Yes	Yes
030.001.00044	IPP	08MAY2000	19JUN2000	3	Yes	6	Yes	Yes
030.001.00055	IPP	08MAY2000	22MAY2000	3	Yes	2	Yes	Yes
030.001.00056	I	08MAY2000	12JUN2000	3	Yes	5	Yes	Yes
030.001.00057	IPP	08MAY2000	26JUN2000	3	Yes	7	Yes	Yes
030.001.00059	IPP	08MAY2000	22MAY2000	3	Yes	2	Yes	Yes
030.001.00063	IPP	08MAY2000	03JUL2000	3	Yes	8	Yes	Yes
030.001.00064	IPP	08MAY2000	26JUN2000	3	Yes	7	Yes	Yes
030.001.00067	IPP	08MAY2000	07AUG2000	3	Yes	12	Yes	Yes
030.001.00072	IPP	08MAY2000	26JUN2000	3	Yes	7	Yes	Yes
030.001.00076	IPP	08MAY2000	12JUN2000	3	Yes	5	Yes	Yes
030.001.00078	IPP	08MAY2000	19JUN2000	3	Yes	6	Yes	Yes
030.001.00080	IPP	08MAY2000	14AUG2000	3	Yes	14	Yes	Yes
030.001.00081	IPP	08MAY2000	13JUN2000	3	Yes	5	Yes	Yes
030.001.00087	I	08MAY2000	15MAY2000	3	Yes	1	Yes	Yes
030.001.00088	IPP	08MAY2000	12JUN2000	3	Yes	5	Yes	Yes
030.001.00090	IPP	08MAY2000	25JUL2000	3	Yes	11	Yes	Yes
030.001.00092	IPP	08MAY2000	03JUL2000	3	Yes	8	Yes	Yes
030.001.00101	IPP	08MAY2000	03JUL2000	3	Yes	8	Yes	Yes
030.001.00103	IPP	08MAY2000	19JUN2000	3	Yes	6	Yes	Yes
030.001.00106	IPP	08MAY2000	02JUL2000	3	Yes	6	Yes	Yes
030.001.00108	IPP	08MAY2000	22MAY2000	3	Yes	2	Yes	Yes
030.001.00114	IPP	08MAY2000	03JUL2000	3	Yes	8	Yes	Yes
030.001.00115	IPP	08MAY2000	05JUN2000	3	Yes	4	Yes	Yes
030.001.00116	IPP	08MAY2000	22MAY2000	3	Yes	2	Yes	Yes

*Popn=Population (I=Intent-To-Treat, PP=Per Protocol)
**Loading Dose Regimen Compliant = Received 3 prophylactic medication capsules with no more than 1 day between doses
+Weekly Dosing Regimen Compliant = Subject received weekly doses of prophylactic medication, with no dose gaps of greater than 18 days, and no more than three dose gaps of greater than 14 days
++Prophylactic Medication Compliant = Compliant with loading dose and weekly regimen
Tafenoquine = Tafenoquine 200mg, Mefloquine = Mefloquine 250mg

SB252263 - 030

Listing B.16

Prophylactic Medication Compliance

Treatment : Placebo

Subject Number	Popn*	First Dose Prophylactic Medication	Last Dose Prophylactic Medication	Number of Loading Doses	Loading Dose Compliant**	Number of Weekly Doses	Weekly Dosing Compliant+	Prophylactic Medication Compliant++
030.001.00119	IPP	09MAY2000	23MAY2000	3	Yes	2	Yes	Yes
030.001.00124	IPP	09MAY2000	05JUN2000	3	Yes	4	Yes	Yes
030.001.00125	IPP	09MAY2000	03JUL2000	3	Yes	8	Yes	Yes
030.001.00127	I	09MAY2000	11JUN2000	3	Yes	5	Yes	Yes
030.001.00131	IPP	09MAY2000	26JUN2000	3	Yes	7	Yes	Yes
030.001.00136	IPP	09MAY2000	05JUN2000	3	Yes	4	Yes	Yes
030.001.00137	IPP	09MAY2000	03JUL2000	3	Yes	8	Yes	Yes
030.001.00140	IPP	09MAY2000	19JUN2000	3	Yes	6	Yes	Yes
030.001.00141	IPP	09MAY2000	26JUN2000	3	Yes	7	Yes	Yes
030.001.00145	IPP	09MAY2000	13JUN2000	3	Yes	5	Yes	Yes
030.001.00151	IPP	09MAY2000	10JUL2000	3	Yes	9	Yes	Yes
030.001.00152	IPP	09MAY2000	26JUN2000	3	Yes	7	Yes	Yes
030.001.00154	IPP	09MAY2000	12JUN2000	3	Yes	5	Yes	Yes
030.001.00160	I	09MAY2000	26JUN2000	3	Yes	7	Yes	Yes
030.001.00162	IPP	09MAY2000	03JUL2000	3	Yes	8	Yes	Yes
030.001.00164	IPP	09MAY2000	12JUN2000	3	Yes	5	Yes	Yes
030.001.00166	IPP	09MAY2000	23OCT2000	3	Yes	24	Yes	Yes
030.001.00169	IPP	09MAY2000	26JUN2000	3	Yes	7	Yes	Yes
030.001.00172	IPP	09MAY2000	28SEP2000	3	Yes	20	Yes	Yes
030.001.00174	IPP	09MAY2000	12JUL2000	3	Yes	9	Yes	Yes
030.001.00175	IPP	09MAY2000	12JUL2000	3	Yes	9	Yes	Yes
030.001.00182	I	09MAY2000	06JUN2000	3	Yes	4	Yes	Yes
030.001.00183	IPP	09MAY2000	21JUN2000	3	Yes	6	Yes	Yes
030.001.00191	IPP	09MAY2000	15JUN2000	3	Yes	5	Yes	Yes
030.001.00192	IPP	09MAY2000	31MAY2000	3	Yes	3	Yes	Yes
030.001.00193	IPP	09MAY2000	31MAY2000	3	Yes	3	Yes	Yes
030.001.00197	IPP	09MAY2000	31MAY2000	3	Yes	3	Yes	Yes
030.001.00202		09MAY2000	11MAY2000	3	Yes	0	No	No
030.001.00204	I	09MAY2000	05JUN2000	3	Yes	4	Yes	Yes
030.001.00208	IPP	09MAY2000	02AUG2000	3	Yes	12	Yes	Yes
030.001.00211	IPP	09MAY2000	21JUN2000	3	Yes	6	Yes	Yes
030.001.00212	IPP	09MAY2000	28JUN2000	3	Yes	7	Yes	Yes
030.001.00215	IPP	09MAY2000	14JUN2000	3	Yes	5	Yes	Yes
030.001.00219	IPP	09MAY2000	14JUN2000	3	Yes	5	Yes	Yes
030.001.00222	IPP	09MAY2000	21JUN2000	3	Yes	6	Yes	Yes
030.001.00225	IPP	09MAY2000	14JUN2000	3	Yes	5	Yes	Yes
030.001.00226	IPP	09MAY2000	21JUN2000	3	Yes	6	Yes	Yes
030.001.00229	IPP	09MAY2000	21JUN2000	3	Yes	6	Yes	Yes
030.001.00234	IPP	10MAY2000	14JUN2000	3	Yes	5	Yes	Yes

*Popn=Population (I=Intent-To-Treat, PP=Per Protocol)
**Loading Dose Regimen Compliant = Received 3 prophylactic medication capsules with no more than 1 day between doses
+Weekly Dosing Regimen Compliant = Subject received weekly doses of prophylactic medication, with no dose gaps of greater than 18 days, and no more than three dose gaps of greater than 14 days
++Prophylactic Medication Compliant = Compliant with loading dose and weekly regimen
Tafenoquine = Tafenoquine 200mg, Mefloquine = Mefloquine 250mg

SB252263 - 030

Listing B.16

Prophylactic Medication Compliance

Treatment : Placebo

Subject Number	Popn*	First Dose Prophylactic Medication	Last Dose Prophylactic Medication	Number of Loading Doses	Loading Dose Compliant**	Number of Weekly Doses	Weekly Dosing Compliant+	Prophylactic Medication Compliant++
030.001.00236	IPP	11MAY2000	12JUL2000	3	Yes	9	Yes	Yes
030.001.00238	IPP	10MAY2000	14JUN2000	3	Yes	5	Yes	Yes
030.001.00242	IPP	10MAY2000	14JUN2000	3	Yes	5	Yes	Yes
030.001.00247	IPP	10MAY2000	26JUL2000	3	Yes	11	Yes	Yes
030.001.00248	IPP	10MAY2000	03JUL2000	3	Yes	8	Yes	Yes
030.001.00251	IPP	10MAY2000	14JUN2000	3	Yes	5	Yes	Yes
030.001.00253	IPP	10MAY2000	28JUN2000	3	Yes	7	Yes	Yes
030.001.00256	IPP	10MAY2000	16JUN2000	3	Yes	5	Yes	Yes
030.001.00257	IPP	12MAY2000	23JUN2000	3	Yes	6	Yes	Yes
030.001.00260	IPP	10MAY2000	21JUN2000	3	Yes	6	Yes	Yes
030.001.00268	IPP	10MAY2000	24MAY2000	3	Yes	2	Yes	Yes
030.001.00269	IPP	10MAY2000	21JUN2000	3	Yes	6	Yes	Yes
030.001.00270	IPP	10MAY2000	28JUN2000	3	Yes	7	Yes	Yes
030.001.00275	IPP	10MAY2000	28JUN2000	3	Yes	7	Yes	Yes
030.001.00277	IPP	10MAY2000	19JUL2000	3	Yes	10	Yes	Yes
030.001.00278	IPP	10MAY2000	14JUN2000	3	Yes	5	Yes	Yes
030.001.00285	IPP	11MAY2000	28JUN2000	3	Yes	7	Yes	Yes
030.001.00287	IPP	11MAY2000	26JUL2000	3	Yes	11	Yes	Yes
030.001.00291	IPP	11MAY2000	28JUN2000	3	Yes	7	Yes	Yes
030.001.00295		11MAY2000	19JUL2000	3	Yes	10	Yes	Yes
030.001.00297	IPP	11MAY2000	20SEP2000	3	Yes	19	Yes	Yes
030.001.00300	IPP	12MAY2000	21JUN2000	3	Yes	6	Yes	Yes
030.001.00303	IPP	12MAY2000	12JUL2000	3	Yes	9	Yes	Yes

*Popn=Population (I=Intent-To-Treat, PP=Per Protocol)
**Loading Dose Regimen Compliant = Received 3 prophylactic medication capsules with no more than 1 day between doses
+Weekly Dosing Regimen Compliant = Subject received weekly doses of prophylactic medication, with no dose gaps of greater than 18 days, and no more than three dose gaps of greater than 14 days
++Prophylactic Medication Compliant = Compliant with loading dose and weekly regimen
Tafenoquine = Tafenoquine 200mg, Mefloquine = Mefloquine 250mg

SB252263 - 030

Listing B.16

Prophylactic Medication Compliance

Treatment : Tafenoquine

Subject Number	Popn*	First Dose Prophylactic Medication	Last Dose Prophylactic Medication	Number of Loading Doses	Loading Dose Compliant**	Number of Weekly Doses	Weekly Dosing Compliant+	Prophylactic Medication Compliant++
030.001.00001	IPP	08MAY2000	15MAY2000	3	Yes	1	Yes	Yes
030.001.00004	IPP	08MAY2000	22MAY2000	3	Yes	2	Yes	Yes
030.001.00006	IPP	08MAY2000	22MAY2000	3	Yes	2	Yes	Yes
030.001.00009	IPP	08MAY2000	26JUN2000	3	Yes	7	Yes	Yes
030.001.00014	IPP	08MAY2000	31JUL2000	3	Yes	12	Yes	Yes
030.001.00016	IPP	08MAY2000	07AUG2000	3	Yes	13	Yes	Yes
030.001.00017	IPP	08MAY2000	18SEP2000	3	Yes	19	Yes	Yes
030.001.00024	IPP	08MAY2000	19JUN2000	3	Yes	6	Yes	Yes
030.001.00025	IPP	08MAY2000	07AUG2000	3	Yes	13	Yes	Yes
030.001.00028	IPP	08MAY2000	17JUL2000	3	Yes	10	Yes	Yes
030.001.00029	IPP	08MAY2000	03JUL2000	3	Yes	8	Yes	Yes
030.001.00030	IPP	08MAY2000	10JUL2000	3	Yes	9	Yes	Yes
030.001.00040	IPP	08MAY2000	22MAY2000	3	Yes	2	Yes	Yes
030.001.00042	IPP	08MAY2000	26JUN2000	3	Yes	7	Yes	Yes
030.001.00043	IPP	08MAY2000	22MAY2000	3	Yes	2	Yes	Yes
030.001.00048	IPP	08MAY2000	26JUN2000	3	Yes	7	Yes	Yes
030.001.00050	IPP	08MAY2000	31MAY2000	3	Yes	3	Yes	Yes
030.001.00051	IPP	08MAY2000	25SEP2000	3	Yes	19	Yes	Yes
030.001.00058	IPP	08MAY2000	17JUL2000	3	Yes	10	Yes	Yes
030.001.00060	IPP	08MAY2000	17JUL2000	3	Yes	10	Yes	Yes
030.001.00061	IPP	08MAY2000	10JUL2000	3	Yes	9	Yes	Yes
030.001.00065	IPP	08MAY2000	26JUN2000	3	Yes	7	Yes	Yes
030.001.00068	IPP	08MAY2000	11JUL2000	3	Yes	9	Yes	Yes
030.001.00070	IPP	08MAY2000	03JUL2000	3	Yes	8	Yes	Yes
030.001.00077	IPP	08MAY2000	03JUL2000	3	Yes	8	Yes	Yes
030.001.00079	IPP	08MAY2000	03JUL2000	3	Yes	8	Yes	Yes
030.001.00082		08MAY2000	10MAY2000	3	Yes	0	No	No
030.001.00084	IPP	08MAY2000	21AUG2000	3	Yes	15	Yes	Yes
030.001.00085	IPP	08MAY2000	07AUG2000	3	Yes	13	Yes	Yes
030.001.00086	IPP	08MAY2000	22MAY2000	3	Yes	2	Yes	Yes
030.001.00093	IPP	08MAY2000	04SEP2000	3	Yes	17	Yes	Yes
030.001.00094	I	08MAY2000	30AUG2000	3	Yes	16	Yes	Yes
030.001.00100	IPP	08MAY2000	26JUN2000	3	Yes	7	Yes	Yes
030.001.00102	IPP	08MAY2000	10JUL2000	3	Yes	9	Yes	Yes
030.001.00104	IPP	08MAY2000	26JUN2000	3	Yes	7	Yes	Yes
030.001.00107	IPP	08MAY2000	07AUG2000	3	Yes	13	Yes	Yes
030.001.00109	IPP	08MAY2000	24JUL2000	3	Yes	11	Yes	Yes
030.001.00111	IPP	08MAY2000	03OCT2000	3	Yes	21	Yes	Yes
030.001.00112	IPP	08MAY2000	26JUN2000	3	Yes	7	Yes	Yes

*Popn=Population (I=Intent-To-Treat, PP=Per Protocol)
**Loading Dose Regimen Compliant = Received 3 prophylactic medication capsules with no more than 1 day between doses
+Weekly Dosing Regimen Compliant = Subject received weekly doses of prophylactic medication, with no dose gaps of greater than 18 days, and no more than three dose gaps of greater than 14 days
++Prophylactic Medication Compliant = Compliant with loading dose and weekly regimen
Tafenoquine = Tafenoquine 200mg, Mefloquine = Mefloquine 250mg

SB252263 - 030

Listing B.16

Prophylactic Medication Compliance

Treatment : Tafenoquine

Subject Number	Popn*	First Dose Prophylactic Medication	Last Dose Prophylactic Medication	Number of Loading Doses	Loading Dose Compliant**	Number of Weekly Doses	Weekly Dosing Compliant+	Prophylactic Medication Compliant++
030.001.00118	IPP	09MAY2000	26JUN2000	3	Yes	7	Yes	Yes
030.001.00122	IPP	09MAY2000	22MAY2000	3	Yes	2	Yes	Yes
030.001.00126	IPP	09MAY2000	23OCT2000	3	Yes	24	Yes	Yes
030.001.00128	IPP	09MAY2000	23OCT2000	3	Yes	24	Yes	Yes
030.001.00129	IPP	09MAY2000	07AUG2000	3	Yes	13	Yes	Yes
030.001.00133	IPP	09MAY2000	10JUL2000	3	Yes	9	Yes	Yes
030.001.00135	IPP	09MAY2000	26JUN2000	3	Yes	7	Yes	Yes
030.001.00138	IPP	09MAY2000	26JUN2000	3	Yes	7	Yes	Yes
030.001.00139	IPP	09MAY2000	18SEP2000	3	Yes	19	Yes	Yes
030.001.00148	IPP	09MAY2000	28AUG2000	3	Yes	16	Yes	Yes
030.001.00149	IPP	09MAY2000	17JUL2000	3	Yes	10	Yes	Yes
030.001.00150	IPP	09MAY2000	03JUL2000	3	Yes	8	Yes	Yes
030.001.00155	IPP	09MAY2000	31JUL2000	3	Yes	12	Yes	Yes
030.001.00157	IPP	09MAY2000	04JUN2000	3	Yes	4	Yes	Yes
030.001.00158	IPP	09MAY2000	29MAY2000	3	Yes	3	Yes	Yes
030.001.00159	IPP	09MAY2000	25SEP2000	3	Yes	20	Yes	Yes
030.001.00161	IPP	09MAY2000	03JUL2000	3	Yes	8	Yes	Yes
030.001.00171	IPP	09MAY2000	12JUN2000	3	Yes	5	Yes	Yes
030.001.00173	I	09MAY2000	26JUN2000	3	Yes	7	Yes	Yes
030.001.00176	IPP	09MAY2000	31MAY2000	3	Yes	3	Yes	Yes
030.001.00179	I	09MAY2000	06SEP2000	3	Yes	17	Yes	Yes
030.001.00184	IPP	09MAY2000	28JUN2000	3	Yes	7	Yes	Yes
030.001.00186	IPP	09MAY2000	14JUN2000	3	Yes	5	Yes	Yes
030.001.00187	I	09MAY2000	19JUL2000	3	Yes	9	Yes	Yes
030.001.00190	IPP	09MAY2000	28JUN2000	3	Yes	7	Yes	Yes
030.001.00194	IPP	09MAY2000	31MAY2000	3	Yes	3	Yes	Yes
030.001.00195	IPP	09MAY2000	31MAY2000	3	Yes	3	Yes	Yes
030.001.00201	IPP	09MAY2000	14JUN2000	3	Yes	5	Yes	Yes
030.001.00203	IPP	09MAY2000	21JUN2000	3	Yes	6	Yes	Yes
030.001.00205	IPP	09MAY2000	14JUN2000	3	Yes	5	Yes	Yes
030.001.00207	IPP	09MAY2000	28AUG2000	3	Yes	16	Yes	Yes
030.001.00209	IPP	09MAY2000	05JUL2000	3	Yes	8	Yes	Yes
030.001.00210	IPP	09MAY2000	02AUG2000	3	Yes	12	Yes	Yes
030.001.00217	IPP	09MAY2000	21JUN2000	3	Yes	6	Yes	Yes
030.001.00218	IPP	09MAY2000	24OCT2000	3	Yes	25	Yes	Yes
030.001.00220	IPP	09MAY2000	28JUN2000	3	Yes	7	Yes	Yes
030.001.00228	IPP	09MAY2000	14JUN2000	3	Yes	5	Yes	Yes
030.001.00230	IPP	09MAY2000	04JUL2000	3	Yes	8	Yes	Yes
030.001.00231	IPP	09MAY2000	13SEP2000	3	Yes	18	Yes	Yes

*Popn=Population (I=Intent-To-Treat, PP=Per Protocol)
**Loading Dose Regimen Compliant = Received 3 prophylactic medication capsules with no more than 1 day between doses
+Weekly Dosing Regimen Compliant = Subject received weekly doses of prophylactic medication, with no dose gaps of greater than 18 days, and no more than three dose gaps of greater than 14 days
++Prophylactic Medication Compliant = Compliant with loading dose and weekly regimen
Tafenoquine = Tafenoquine 200mg, Mefloquine = Mefloquine 250mg

SB252263 - 030

Listing B.16

Prophylactic Medication Compliance

Treatment : Tafenoquine

Subject Number	Popn*	First Dose Prophylactic Medication	Last Dose Prophylactic Medication	Number of Loading Doses	Loading Dose Compliant**	Number of Weekly Doses	Weekly Dosing Compliant+	Prophylactic Medication Compliant++
030.001.00233	IPP	10MAY2000	04SEP2000	3	Yes	17	Yes	Yes
030.001.00240	IPP	10MAY2000	14JUN2000	3	Yes	5	Yes	Yes
030.001.00243	IPP	10MAY2000	24OCT2000	3	Yes	24	Yes	Yes
030.001.00246	IPP	10MAY2000	12JUL2000	3	Yes	8	Yes	Yes
030.001.00249	IPP	10MAY2000	04JUL2000	3	Yes	8	Yes	Yes
030.001.00252	IPP	10MAY2000	04OCT2000	3	Yes	21	Yes	Yes
030.001.00254	I	10MAY2000	08JUN2000	3	Yes	4	Yes	Yes
030.001.00259	IPP	10MAY2000	03JUL2000	3	Yes	8	Yes	Yes
030.001.00261		10MAY2000	12MAY2000	3	Yes	0	No	No
030.001.00264	IPP	10MAY2000	19JUL2000	3	Yes	10	Yes	Yes
030.001.00266	I	10MAY2000	24MAY2000	3	Yes	2	Yes	Yes
030.001.00267	IPP	10MAY2000	21JUN2000	3	Yes	6	Yes	Yes
030.001.00271	I	10MAY2000	31MAY2000	3	Yes	3	Yes	Yes
030.001.00272	IPP	10MAY2000	26JUL2000	3	Yes	11	Yes	Yes
030.001.00279	IPP	11MAY2000	19MAY2000	3	Yes	1	Yes	Yes
030.001.00282	IPP	10MAY2000	13SEP2000	3	Yes	19	Yes	Yes
030.001.00284	IPP	11MAY2000	23OCT2000	3	Yes	24	Yes	Yes
030.001.00288	IPP	11MAY2000	06JUN2000	3	Yes	4	Yes	Yes
030.001.00293	IPP	11MAY2000	25OCT2000	3	Yes	23	Yes	Yes
030.001.00294	IPP	11MAY2000	12JUL2000	3	Yes	9	Yes	Yes
030.001.00296	IPP	11MAY2000	23OCT2000	3	Yes	24	Yes	Yes
030.001.00298	IPP	12MAY2000	02AUG2000	3	Yes	12	Yes	Yes
030.001.00301	IPP	12MAY2000	04JUL2000	3	Yes	8	Yes	Yes
030.001.00302	I	12MAY2000	21MAY2000	2	No	1	Yes	No
030.001.00304	IPP	12MAY2000	04JUL2000	3	Yes	8	Yes	Yes
030.001.00306	IPP	12MAY2000	02AUG2000	3	Yes	12	Yes	Yes

*Popn=Population (I=Intent-To-Treat, PP=Per Protocol)
**Loading Dose Regimen Compliant = Received 3 prophylactic medication capsules with no more than 1 day between doses
+Weekly Dosing Regimen Compliant = Subject received weekly doses of prophylactic medication, with no dose gaps of greater than 18 days, and no more than three dose gaps of greater than 14 days
++Prophylactic Medication Compliant = Compliant with loading dose and weekly regimen
Tafenoquine = Tafenoquine 200mg, Mefloquine = Mefloquine 250mg

SB252263 - 030

Listing B.16

Prophylactic Medication Compliance

Treatment : Mefloquine

Subject Number	Popn*	First Dose Prophylactic Medication	Last Dose Prophylactic Medication	Number of Loading Doses	Loading Dose Compliant**	Number of Weekly Doses	Weekly Dosing Compliant+	Prophylactic Medication Compliant++
030.001.00003	I	08MAY2000	16JUN2000	3	Yes	5	Yes	Yes
030.001.00007	IPP	08MAY2000	16OCT2000	3	Yes	23	Yes	Yes
030.001.00008	IPP	08MAY2000	16OCT2000	3	Yes	23	Yes	Yes
030.001.00010	IPP	08MAY2000	12JUN2000	3	Yes	5	Yes	Yes
030.001.00013	IPP	08MAY2000	22MAY2000	3	Yes	2	Yes	Yes
030.001.00018	IPP	08MAY2000	10JUL2000	3	Yes	9	Yes	Yes
030.001.00021	IPP	08MAY2000	23OCT2000	3	Yes	24	Yes	Yes
030.001.00023	I	08MAY2000	22MAY2000	3	Yes	2	Yes	Yes
030.001.00031	IPP	08MAY2000	02OCT2000	3	Yes	21	Yes	Yes
030.001.00034	IPP	08MAY2000	07AUG2000	3	Yes	13	Yes	Yes
030.001.00035		08MAY2000	10MAY2000	3	Yes	0	No	No
030.001.00036	I	08MAY2000	17JUL2000	3	Yes	10	Yes	Yes
030.001.00038	IPP	08MAY2000	19JUN2000	3	Yes	6	Yes	Yes
030.001.00045	IPP	08MAY2000	22MAY2000	3	Yes	2	Yes	Yes
030.001.00046	IPP	08MAY2000	26JUN2000	3	Yes	7	Yes	Yes
030.001.00047	IPP	08MAY2000	19JUN2000	3	Yes	6	Yes	Yes
030.001.00049	IPP	08MAY2000	17JUL2000	3	Yes	10	Yes	Yes
030.001.00052	IPP	08MAY2000	12JUN2000	3	Yes	5	Yes	Yes
030.001.00053	IPP	08MAY2000	31JUL2000	3	Yes	12	Yes	Yes
030.001.00054	IPP	08MAY2000	26JUN2000	3	Yes	7	Yes	Yes
030.001.00062	IPP	08MAY2000	19JUN2000	3	Yes	6	Yes	Yes
030.001.00066	IPP	08MAY2000	06JUN2000	3	Yes	4	Yes	Yes
030.001.00069	I	08MAY2000	17JUL2000	3	Yes	10	Yes	Yes
030.001.00071	IPP	08MAY2000	17JUL2000	3	Yes	10	Yes	Yes
030.001.00073	IPP	08MAY2000	26JUN2000	3	Yes	7	Yes	Yes
030.001.00074	IPP	08MAY2000	24JUL2000	3	Yes	11	Yes	Yes
030.001.00075	IPP	08MAY2000	10JUL2000	3	Yes	9	Yes	Yes
030.001.00083	IPP	08MAY2000	10JUL2000	3	Yes	9	Yes	Yes
030.001.00089	IPP	08MAY2000	22MAY2000	3	Yes	2	Yes	Yes
030.001.00091	IPP	08MAY2000	14AUG2000	3	Yes	14	Yes	Yes
030.001.00095	IPP	08MAY2000	20JUN2000	3	Yes	6	Yes	Yes
030.001.00096	IPP	08MAY2000	03JUL2000	3	Yes	8	Yes	Yes
030.001.00097	IPP	08MAY2000	19JUN2000	3	Yes	6	Yes	Yes
030.001.00098	IPP	08MAY2000	09JUL2000	3	Yes	9	Yes	Yes
030.001.00099	IPP	08MAY2000	12JUN2000	3	Yes	5	Yes	Yes
030.001.00105	IPP	08MAY2000	22MAY2000	3	Yes	2	Yes	Yes
030.001.00110	IPP	08MAY2000	17JUL2000	3	Yes	10	Yes	Yes
030.001.00113	IPP	08MAY2000	26JUN2000	3	Yes	7	Yes	Yes
030.001.00117	IPP	08MAY2000	26JUN2000	3	Yes	7	Yes	Yes

*Popn=Population (I=Intent-To-Treat, PP=Per Protocol)
**Loading Dose Regimen Compliant = Received 3 prophylactic medication capsules with no more than 1 day between doses
+Weekly Dosing Regimen Compliant = Subject received weekly doses of prophylactic medication, with no dose gaps of greater than 18 days, and no more than three dose gaps of greater than 14 days
++Prophylactic Medication Compliant = Compliant with loading dose and weekly regimen
Tafenoquine = Tafenoquine 200mg, Mefloquine = Mefloquine 250mg

SB252263 - 030

Listing B.16

Prophylactic Medication Compliance

Treatment : Mefloquine

Subject Number	Popn*	First Dose Prophylactic Medication	Last Dose Prophylactic Medication	Number of Loading Doses	Loading Dose Compliant**	Number of Weekly Doses	Weekly Dosing Compliant+	Prophylactic Medication Compliant++
030.001.00120	IPP	09MAY2000	22MAY2000	3	Yes	2	Yes	Yes
030.001.00121	IPP	09MAY2000	26JUN2000	3	Yes	7	Yes	Yes
030.001.00123	I	09MAY2000	21JUN2000	3	Yes	5	Yes	Yes
030.001.00130	IPP	09MAY2000	24JUL2000	3	Yes	11	Yes	Yes
030.001.00132	IPP	09MAY2000	26JUN2000	3	Yes	7	Yes	Yes
030.001.00134	IPP	09MAY2000	26JUL2000	3	Yes	11	Yes	Yes
030.001.00142	IPP	09MAY2000	18SEP2000	3	Yes	19	Yes	Yes
030.001.00143	IPP	09MAY2000	26JUN2000	3	Yes	7	Yes	Yes
030.001.00144	IPP	09MAY2000	05JUN2000	3	Yes	4	Yes	Yes
030.001.00146	I	09MAY2000	20JUN2000	3	Yes	6	Yes	Yes
030.001.00147	IPP	09MAY2000	31JUL2000	3	Yes	12	Yes	Yes
030.001.00153	IPP	09MAY2000	16OCT2000	3	Yes	23	Yes	Yes
030.001.00156	IPP	09MAY2000	03JUL2000	3	Yes	8	Yes	Yes
030.001.00163	IPP	09MAY2000	26JUN2000	3	Yes	7	Yes	Yes
030.001.00165	IPP	09MAY2000	05JUN2000	3	Yes	4	Yes	Yes
030.001.00167	IPP	09MAY2000	12JUN2000	3	Yes	5	Yes	Yes
030.001.00168	IPP	09MAY2000	05JUN2000	3	Yes	4	Yes	Yes
030.001.00170	IPP	09MAY2000	26JUN2000	3	Yes	7	Yes	Yes
030.001.00177	IPP	09MAY2000	19JUL2000	3	Yes	10	Yes	Yes
030.001.00178	I	09MAY2000	04JUL2000	3	No	8	Yes	No
030.001.00180	IPP	09MAY2000	16OCT2000	3	Yes	23	Yes	Yes
030.001.00181	IPP	09MAY2000	14JUN2000	3	Yes	5	Yes	Yes
030.001.00185	IPP	09MAY2000	16OCT2000	3	Yes	23	Yes	Yes
030.001.00188	IPP	09MAY2000	05JUN2000	3	Yes	4	Yes	Yes
030.001.00189	I	09MAY2000	31MAY2000	3	Yes	3	Yes	Yes
030.001.00196	IPP	09MAY2000	12JUL2000	3	Yes	9	Yes	Yes
030.001.00198	IPP	09MAY2000	28JUN2000	3	Yes	7	Yes	Yes
030.001.00199	IPP	09MAY2000	21JUN2000	3	Yes	6	Yes	Yes
030.001.00200	IPP	09MAY2000	31MAY2000	3	Yes	3	Yes	Yes
030.001.00206	I	09MAY2000	17MAY2000	3	Yes	1	Yes	Yes
030.001.00213	IPP	09MAY2000	01AUG2000	3	Yes	12	Yes	Yes
030.001.00214	IPP	09MAY2000	21JUN2000	3	Yes	6	Yes	Yes
030.001.00216	IPP	09MAY2000	20JUL2000	3	Yes	10	Yes	Yes
030.001.00221	IPP	09MAY2000	20JUL2000	3	Yes	10	Yes	Yes
030.001.00223	IPP	09MAY2000	14JUN2000	3	Yes	5	Yes	Yes
030.001.00224	IPP	09MAY2000	06JUN2000	3	Yes	4	Yes	Yes
030.001.00227	IPP	09MAY2000	21JUN2000	3	Yes	6	Yes	Yes
030.001.00232	IPP	10MAY2000	24MAY2000	3	Yes	2	Yes	Yes
030.001.00235	IPP	10MAY2000	14JUN2000	3	Yes	5	Yes	Yes

*Popn=Population (I=Intent-To-Treat, PP=Per Protocol)
**Loading Dose Regimen Compliant = Received 3 prophylactic medication capsules with no more than 1 day between doses
+Weekly Dosing Regimen Compliant = Subject received weekly doses of prophylactic medication, with no dose gaps of greater than 18 days, and no more than three dose gaps of greater than 14 days
++Prophylactic Medication Compliant = Compliant with loading dose and weekly regimen
Tafenoquine = Tafenoquine 200mg, Mefloquine = Mefloquine 250mg

SB252263 - 030

Listing B.16

Prophylactic Medication Compliance

Treatment : Mefloquine

Subject Number	Popn*	First Dose Prophylactic Medication	Last Dose Prophylactic Medication	Number of Loading Doses	Loading Dose Compliant**	Number of Weekly Doses	Weekly Dosing Compliant+	Prophylactic Medication Compliant++
030.001.00237	IPP	10MAY2000	06JUN2000	3	Yes	4	Yes	Yes
030.001.00239	IPP	10MAY2000	14JUN2000	3	Yes	5	Yes	Yes
030.001.00241	IPP	10MAY2000	24OCT2000	3	Yes	24	Yes	Yes
030.001.00244	IPP	10MAY2000	02AUG2000	3	Yes	12	Yes	Yes
030.001.00245	IPP	10MAY2000	26JUL2000	3	Yes	11	Yes	Yes
030.001.00250		10MAY2000	11MAY2000	2	No	0	No	No
030.001.00255	IPP	10MAY2000	19JUL2000	3	Yes	10	Yes	Yes
030.001.00258	IPP	10MAY2000	19JUL2000	3	Yes	10	Yes	Yes
030.001.00262	IPP	10MAY2000	23JUN2000	3	Yes	6	Yes	Yes
030.001.00263	IPP	12MAY2000	23OCT2000	3	Yes	24	Yes	Yes
030.001.00265	IPP	10MAY2000	13SEP2000	3	Yes	18	Yes	Yes
030.001.00273	IPP	10MAY2000	09AUG2000	3	Yes	13	Yes	Yes
030.001.00274	IPP	10MAY2000	28JUN2000	3	Yes	7	Yes	Yes
030.001.00276	IPP	10MAY2000	21JUN2000	3	Yes	6	Yes	Yes
030.001.00280	IPP	10MAY2000	28JUN2000	3	Yes	7	Yes	Yes
030.001.00281	IPP	10MAY2000	21JUN2000	3	Yes	6	Yes	Yes
030.001.00283	IPP	11MAY2000	12JUL2000	3	Yes	9	Yes	Yes
030.001.00286	IPP	11MAY2000	26JUL2000	3	Yes	11	Yes	Yes
030.001.00289	IPP	11MAY2000	28JUN2000	3	Yes	7	Yes	Yes
030.001.00290	IPP	11MAY2000	06JUN2000	3	Yes	4	Yes	Yes
030.001.00292	IPP	11MAY2000	12JUL2000	3	Yes	9	Yes	Yes
030.001.00299	IPP	12MAY2000	24MAY2000	3	Yes	2	Yes	Yes
030.001.00305	IPP	12MAY2000	04JUL2000	3	Yes	8	Yes	Yes

*Popn=Population (I=Intent-To-Treat, PP=Per Protocol)
**Loading Dose Regimen Compliant = Received 3 prophylactic medication capsules with no more than 1 day between doses
+Weekly Dosing Regimen Compliant = Subject received weekly doses of prophylactic medication, with no dose gaps of greater than 18 days, and no more than three dose gaps of greater than 14 days
++Prophylactic Medication Compliant = Compliant with loading dose and weekly regimen
Tafenoquine = Tafenoquine 200mg, Mefloquine = Mefloquine 250mg

Listing C.01

SB252263 - 030

Listing C.01

Results Of Blood Smears By Visit

Treatment : Placebo

Subject Number	Popn *	Visit	Visit Date	Sample Date	Rel Day**	Symp ?	Rd+	Res +++	Spec #	Spec Count W	Spec Count B	Mean	Diff	Gam Count
030.001.00002	IPP	Screening	10APR2000	10APR2000	-28		1	Pos	P.f	0.8	9.7	7.0	5.6	
		Screening	10APR2000	10APR2000			2	Pos	P.f	0.4	4.2			
		Screening (Unsch.)	29APR2000			Yes								
		Post Clearance	06MAY2000	06MAY2000	-2		1	Neg						
		Post Clearance	06MAY2000	06MAY2000			2	Neg						
		Load Dose 1	08MAY2000			No								
		Load Dose 2	09MAY2000			No								
		Load Dose 3	10MAY2000			No								
		Week 1	15MAY2000	15MAY2000	7	Yes	1	Neg						
		Week 1	15MAY2000	15MAY2000		Yes	2	Neg						
		Week 1 (Unsch.)	18MAY2000			Yes								
		Week 2	22MAY2000	22MAY2000	14	Yes	1	Neg						
		Week 2	22MAY2000	22MAY2000		Yes	2	Neg						
		Week 3 (Unsch.)	26MAY2000			No								
		Week 3	29MAY2000	29MAY2000	21	Yes	1	Neg						
		Week 3	29MAY2000	29MAY2000		Yes	2	Neg						
		Week 3 (Unsch.)	31MAY2000			Yes								
		Week 4	05JUN2000	05JUN2000	28	Yes	1	Neg						
		Week 4	05JUN2000	05JUN2000		Yes	2	Neg						
		Week 5	12JUN2000	12JUN2000	35	Yes	1	Neg						
		Week 5	12JUN2000	12JUN2000		Yes	2	Neg						
		Week 5 (Unsch.)	12JUN2000			Yes								
		Week 6	19JUN2000	19JUN2000	42	Yes	1	Pos	P.f	1.1	14.7	18.1	-6.9	
		Week 6	19JUN2000	19JUN2000		Yes	2	Pos	P.f	1.7	21.6			
		Week 6 (Unsch. Fol. Up)	22JUN2000			Yes								
		Week 7 (Fol. Up)	26JUN2000	26JUN2000	49	No	1	Pos	P.f	177.3	2234.0	3068.6	-1669.2	
		Week 7 (Fol. Up)	26JUN2000	26JUN2000		No	2	Pos	P.f	309.8	3903.2			
		Week 8 (Unsch. Fol. Up)	30JUN2000			Yes								
		Week 8 (Fol. Up)	03JUL2000	03JUL2000	56	No	1	Neg						
		Week 8 (Fol. Up)	03JUL2000	03JUL2000		No	2	Neg						
		Week 9 (Fol. Up)	10JUL2000	10JUL2000	63	No	1	Neg						
		Week 9 (Fol. Up)	10JUL2000	10JUL2000		No	2	Neg						
		Week 10 (Fol. Up)	17JUL2000	17JUL2000	70	No	1	Neg						
		Week 10 (Fol. Up)	17JUL2000	17JUL2000		No	2	Neg						
030.001.00005	IPP	Screening	10APR2000	10APR2000	-28		1	Neg						
		Screening	10APR2000	10APR2000			2	Neg						

*Popn=Population (I=Intent-To-Treat, PP=Per Protocol)
**Rel Day=Number of days relative to start of prophylactic medication
Symp = Symptoms, Rd = Reader, Res = Result, Spec = Species, Diff = Difference
Spec Count W = Species Count recorded per 500 White Blood Cells
Spec Count B = Species Count converted to per microlitre of blood
Gam = Gametocyte
+Arb=Arbitrator, +++Pos=Positive, Neg=Negative
#P. f = P.falciparum, P. v = P.vivax, P. o = P.ovale, P. m = P.malariae
Tafenoquine = Tafenoquine 200mg, Mefloquine = Mefloquine 250mg

SB252263 - 030

Listing C.01

Results Of Blood Smears By Visit

Treatment : Placebo

Subject Number	Popn *	Visit	Visit Date	Sample Date	Rel Day**	Symp ?	Rd+	Res +++	Spec #	Spec Count W	Spec Count B	Mean	Diff	Gam Count
030.001.00005	IPP	Screening (Unsch.)	25APR2000			Yes								
		Post Clearance	06MAY2000	06MAY2000	-2		1	Neg						
		Post Clearance	06MAY2000	06MAY2000			2	Neg						
		Load Dose 1	08MAY2000			No								
		Load Dose 2	09MAY2000			No								
		Load Dose 3	10MAY2000			No								
		Week 1 (Unsch.)	12MAY2000			Yes								
		Week 1	15MAY2000	15MAY2000	7	No	1	Neg						
		Week 1	15MAY2000	15MAY2000		No	2	Neg						
		Week 2	22MAY2000	22MAY2000	14	No	1	Neg						
		Week 2	22MAY2000	22MAY2000		No	2	Neg						
		Week 3	30MAY2000	30MAY2000	22	No	1	Neg						
		Week 3	30MAY2000	30MAY2000		No	2	Neg						
		Week 4	04JUN2000	04JUN2000	27	No	1	Neg						
		Week 4	04JUN2000	04JUN2000		No	2	Neg						
		Week 4 (Unsch.)	05JUN2000			Yes								
		Week 5	12JUN2000	12JUN2000	35	No	1	Neg						
		Week 5	12JUN2000	12JUN2000		No	2	Neg						
		Week 6 (Unsch.)	17JUN2000			Yes								
		Week 6	19JUN2000	19JUN2000	42	Yes	1	Neg						
		Week 6	19JUN2000	19JUN2000		Yes	2	Neg						
		Week 6 (Unsch.)	19JUN2000			No								
		Week 6 (Unsch.)	22JUN2000			Yes								
		Week 7	26JUN2000	26JUN2000	49	No	1	Neg						
		Week 7	26JUN2000	26JUN2000		No	2	Pos	P.f	0.4	4.2			
		Week 7	26JUN2000	26JUN2000		No	Arb	Neg						
		Week 8	03JUL2000	03JUL2000	56	No	1	Neg						
		Week 8	03JUL2000	03JUL2000		No	2	Neg						
		Week 9 (Fol. Up)	10JUL2000	10JUL2000	63	No	1	Pos	P.f	3.3	38.0	26.0	23.9	
		Week 9 (Fol. Up)	10JUL2000	10JUL2000			1		P.o	3.3	38.0	26.0	23.9	
		Week 9 (Fol. Up)	10JUL2000	10JUL2000			2	Pos	P.o	1.2	14.0			
		Week 9 (Fol. Up)	10JUL2000	10JUL2000		No	Arb	Pos	P.f	1.2	14.0			
		Week 9 (Fol. Up)	10JUL2000	10JUL2000			Arb		P.o	1.2	14.0			
		Week 10 (Unsch. Fol. Up)	16JUL2000			Yes								
		Week 10 (Fol. Up)	17JUL2000	17JUL2000	70	No	1	Pos	P.f	14.6	178.0	195.2	-34.4	
		Week 10 (Fol. Up)	17JUL2000	17JUL2000		No	2	Pos	P.f	17.4	212.4			
		Week 12 (Fol. Up)	28JUL2000	28JUL2000	81	Yes	1	Pos	P.f	2.0	24.9	14.4	21.0	

*Popn=Population (I=Intent-To-Treat, PP=Per Protocol)
**Rel Day=Number of days relative to start of prophylactic medication
Symp = Symptoms, Rd = Reader, Res = Result, Spec = Species, Diff = Difference
Spec Count W = Species Count recorded per 500 White Blood Cells
Spec Count B = Species Count converted to per microlitre of blood
Gam = Gametocyte
+Arb=Arbitrator, +++Pos=Positive, Neg=Negative
#P. f = P.falciparum, P. v = P.vivax, P. o = P.ovale, P. m = P.malariae
Tafenoquine = Tafenoquine 200mg, Mefloquine = Mefloquine 250mg

SB252263 - 030

Listing C.01

Results Of Blood Smears By Visit

Treatment : Placebo

Subject Number	Popn *	Visit	Visit Date	Sample Date	Rel Day**	Symp ?	Rd+	Res +++	Spec #	Spec Count W	Spec Count B	Mean	Diff	Gam Count
030.001.00005	IPP	Week 12 (Fol. Up)	28JUL2000	28JUL2000		Yes	2	Pos	P.f	0.3	3.9			
		Week 12 (Unsch. Fol. Up)	28JUL2000			Yes								
030.001.00011	IPP	Screening	10APR2000	10APR2000	-28		1	Neg						
		Screening	10APR2000	10APR2000			2	Neg						
		Screening (Unsch.)	19APR2000	19APR2000	-19	Yes		Pos						
		Post Clearance	06MAY2000	06MAY2000	-2		1	Neg						
		Post Clearance	06MAY2000	06MAY2000			2	Neg						
		Load Dose 1	08MAY2000			No								
		Load Dose 2	09MAY2000			No								
		Load Dose 3	10MAY2000			No								
		Week 1	15MAY2000	15MAY2000	7	No	1	Neg						
		Week 1	15MAY2000	15MAY2000		No	2	Neg						
		Week 2	22MAY2000	22MAY2000	14	No	1	Pos	P.f	3.0	24.6	17.0	15.3	
		Week 2	22MAY2000	22MAY2000		No	2	Neg						
		Week 2	22MAY2000	22MAY2000		No	Arb	Pos	P.f	1.1	9.3			
		Week 3	29MAY2000	29MAY2000	21	No	1	Neg						
		Week 3	29MAY2000	29MAY2000		No	2	Neg						
		Week 3 (Unsch. Fol. Up)	30MAY2000			Yes								
		Week 4 (Fol. Up)	04JUN2000	04JUN2000	27	No	1	Neg						
		Week 4 (Fol. Up)	04JUN2000	04JUN2000		No	2	Neg						
		Week 5 (Fol. Up)	12JUN2000	12JUN2000	35	No	1	Neg						
		Week 5 (Fol. Up)	12JUN2000	12JUN2000		No	2	Neg						
		Week 6 (Unsch. Fol. Up)	16JUN2000			No								
		Week 6 (Fol. Up)	19JUN2000	19JUN2000	42	No	1	Pos	P.f	0.3	2.1	8.5	-12.8	
		Week 6 (Fol. Up)	19JUN2000	19JUN2000		No	2	Pos	P.f	2.0	14.9			
		Week 7 (Unsch. Fol. Up)	23JUN2000			No								
		Week 7 (Fol. Up)	26JUN2000	26JUN2000	49	No	1	Neg						
		Week 7 (Fol. Up)	26JUN2000	26JUN2000		No	2	Neg						
030.001.00012	IPP	Screening	10APR2000	10APR2000	-28		1	Neg						
		Screening	10APR2000	10APR2000			2	Neg						
		Screening (Unsch.)	17APR2000	17APR2000	-21	Yes	1	Neg						
		Screening (Unsch.)	17APR2000	17APR2000		Yes	2	Neg						
		Screening (Unsch.)	26APR2000			Yes								
		Post Clearance	06MAY2000	06MAY2000	-2		1	Neg						
		Post Clearance	06MAY2000	06MAY2000			2	Neg						

*Popn=Population (I=Intent-To-Treat, PP=Per Protocol)
**Rel Day=Number of days relative to start of prophylactic medication
Symp = Symptoms, Rd = Reader, Res = Result, Spec = Species, Diff = Difference
Spec Count W = Species Count recorded per 500 White Blood Cells
Spec Count B = Species Count converted to per microlitre of blood
Gam = Gametocyte
+Arb=Arbitrator, +++Pos=Positive, Neg=Negative
#P. f = P.falciparum, P. v = P.vivax, P. o = P.ovale, P. m = P.malariae
Tafenoquine = Tafenoquine 200mg, Mefloquine = Mefloquine 250mg

SB252263 - 030

Listing C.01

Results Of Blood Smears By Visit

Treatment : Placebo

Subject Number	Popn *	Visit	Visit Date	Sample Date	Rel Day**	Symp ?	Rd+	Res +++	Spec #	Spec Count W	Spec Count B	Mean	Diff	Gam Count
030.001.00012	IPP	Load Dose 1	08MAY2000			No								
		Load Dose 2	09MAY2000			No								
		Load Dose 3	10MAY2000			No								
		Load Dose (Unsch.)	10MAY2000			Yes								
		Week 1 (Unsch.)	11MAY2000			Yes								
		Week 1	15MAY2000	15MAY2000	7	No	1	Neg						
		Week 1	15MAY2000	15MAY2000		No	2	Neg						
		Week 2	23MAY2000	23MAY2000	15	No	1	Neg						
		Week 2	23MAY2000	23MAY2000		No	2	Neg						
		Week 3	29MAY2000	29MAY2000	21	No	1	Neg						
		Week 3	29MAY2000	29MAY2000		No	2	Neg						
		Week 4	05JUN2000	05JUN2000	28	No	1	Neg						
		Week 4	05JUN2000	05JUN2000		No	2	Neg						
		Week 5	12JUN2000	12JUN2000	35	No	1	Neg						
		Week 5	12JUN2000	12JUN2000		No	2	Neg						
		Week 6	19JUN2000	19JUN2000	42	No	1	Neg						
		Week 6	19JUN2000	19JUN2000		No	2	Neg						
		Week 7	26JUN2000	26JUN2000	49	No	1	Pos	P.f	3.6	39.3	22.3	34.0	
		Week 7	26JUN2000	26JUN2000		No	2	Neg						
		Week 7	26JUN2000	26JUN2000		No	Arb	Pos	P.f	0.5	5.3			
		Week 8 (Fol. Up)	03JUL2000	03JUL2000	56	No	1	Neg						
		Week 8 (Fol. Up)	03JUL2000	03JUL2000		No	2	Neg						
		Week 9 (Unsch. Fol. Up)	08JUL2000			Yes								
		Week 9 (Unsch. Fol. Up)	09JUL2000	09JUL2000	62	Yes	1	Neg						
		Week 9 (Unsch. Fol. Up)	09JUL2000	09JUL2000		Yes	2	Neg						
		Week 9 (Fol. Up)	10JUL2000	10JUL2000	63	Yes	1	Neg						
		Week 9 (Fol. Up)	10JUL2000	10JUL2000		Yes	2	Neg						
		Week 9 (Unsch. Fol. Up)	12JUL2000	12JUL2000	65	Yes	1	Neg						
		Week 9 (Unsch. Fol. Up)	12JUL2000	12JUL2000		Yes	2	Neg						
		Week 9 (Unsch. Fol. Up)	13JUL2000			Yes								
		Week 10 (Unsch. Fol. Up)	14JUL2000			Yes								
		Week 10 (Fol. Up)	17JUL2000	17JUL2000	70	Yes	1	Neg						
		Week 10 (Fol. Up)	17JUL2000	17JUL2000		Yes	2	Neg						
		Week 10 (Unsch. Fol. Up)	17JUL2000	17JUL2000			1	Neg						
		Week 10 (Unsch. Fol. Up)	17JUL2000	17JUL2000			2	Neg						
		Week 11 (Unsch. Fol. Up)	21JUL2000			No								
		Week 11 (Fol. Up)	25JUL2000	25JUL2000	78	No	1	Neg						

*Popn=Population (I=Intent-To-Treat, PP=Per Protocol)
**Rel Day=Number of days relative to start of prophylactic medication
Symp = Symptoms, Rd = Reader, Res = Result, Spec = Species, Diff = Difference
Spec Count W = Species Count recorded per 500 White Blood Cells
Spec Count B = Species Count converted to per microlitre of blood
Gam = Gametocyte
+Arb=Arbitrator, +++Pos=Positive, Neg=Negative
#P. f = P.falciparum, P. v = P.vivax, P. o = P.ovale, P. m = P.malariae
Tafenoquine = Tafenoquine 200mg, Mefloquine = Mefloquine 250mg

SB252263 - 030

Listing C.01

Results Of Blood Smears By Visit

Treatment : Placebo

Subject Number	Popn *	Visit	Visit Date	Sample Date	Rel Day**	Symp ?	Rd+	Res +++	Spec #	Spec Count W	Spec Count B	Mean	Diff	Gam Count
030.001.00012	IPP	Week 11 (Fol. Up)	25JUL2000	25JUL2000		No	2	Neg						
030.001.00015	I	Screening	10APR2000	10APR2000	-28		1	Neg						
		Screening	10APR2000	10APR2000			2	Neg						
		Screening (Unsch.)	24APR2000	24APR2000	-14	Yes	1	Neg						
		Screening (Unsch.)	24APR2000	24APR2000		Yes	2	Neg						
		Post Clearance	07MAY2000	07MAY2000	-1		1	Neg						
		Post Clearance	07MAY2000	07MAY2000			2	Neg						
		Load Dose 1	08MAY2000			No								
		Load Dose 2	09MAY2000			No								
		Load Dose 3	10MAY2000			No								
		Week 1	15MAY2000	15MAY2000	7	No	1	Neg						
		Week 1	15MAY2000	15MAY2000		No	2	Neg						
		Week 1 (Unsch.)	18MAY2000			No								
		Week 2	22MAY2000	22MAY2000	14	No	1	Neg						
		Week 2	22MAY2000	22MAY2000		No	2	Neg						
		Week 3	01JUN2000	01JUN2000	24	No	1	Neg						
		Week 3	01JUN2000	01JUN2000		No	2	Neg						
		Week 4	05JUN2000	05JUN2000	28	No	1	Neg						
		Week 4	05JUN2000	05JUN2000		No	2	Neg						
		Week 4 (Unsch.)	08JUN2000			No								
		Week 5	12JUN2000	12JUN2000	35	No	1	Neg						
		Week 5	12JUN2000	12JUN2000		No	2	Neg						
		Week 5 (Unsch.)	15JUN2000			No								
		Week 6	19JUN2000	19JUN2000	42	No	1	Neg						
		Week 6	19JUN2000	19JUN2000		No	2	Neg						
		Week 7	26JUN2000	26JUN2000	49	No	1	Neg						
		Week 7	26JUN2000	26JUN2000		No	2	Neg						
		Week 8 (Unsch.)	30JUN2000			No								
		Week 8	03JUL2000	03JUL2000	56	No	1	Neg						
		Week 8	03JUL2000	03JUL2000		No	2	Neg						
		Week 9	10JUL2000	10JUL2000	63	No	1	Neg						
		Week 9	10JUL2000	10JUL2000		No	2	Neg						
		Week 10 (Unsch.)	14JUL2000			No								
		Week 10	17JUL2000	17JUL2000	70	No	1	Neg						
		Week 10	17JUL2000	17JUL2000		No	2	Neg						
		Week 10 (Unsch. Fol. Up)	18JUL2000			No								

*Popn=Population (I=Intent-To-Treat, PP=Per Protocol)
**Rel Day=Number of days relative to start of prophylactic medication
Symp = Symptoms, Rd = Reader, Res = Result, Spec = Species, Diff = Difference
Spec Count W = Species Count recorded per 500 White Blood Cells
Spec Count B = Species Count converted to per microlitre of blood
Gam = Gametocyte
+Arb=Arbitrator, +++Pos=Positive, Neg=Negative
#P. f = P.falciparum, P. v = P.vivax, P. o = P.ovale, P. m = P.malariae
Tafenoquine = Tafenoquine 200mg, Mefloquine = Mefloquine 250mg

SB252263 - 030

Listing C.01

Results Of Blood Smears By Visit

Treatment : Placebo

Subject Number	Popn *	Visit	Visit Date	Sample Date	Rel Day**	Symp ?	Rd+	Res +++	Spec #	Spec Count W	Spec Count B	Mean	Diff	Gam Count
030.001.00019	IPP	Screening	10APR2000	10APR2000	-28		1	Neg						
		Screening	10APR2000	10APR2000			2	Neg						
		Screening (Unsch.)	14APR2000			Yes								
		Screening (Unsch.)	20APR2000			Yes								
		Screening (Unsch.)	26APR2000			Yes								
		Screening (Unsch.)	02MAY2000	02MAY2000	-6	Yes		Pos						
		Screening (Unsch.)	03MAY2000			Yes								
		Post Clearance	06MAY2000	06MAY2000	-2		1	Neg						
		Post Clearance	06MAY2000	06MAY2000			2	Neg						
		Load Dose 1	08MAY2000			No								
		Load Dose (Unsch.)	08MAY2000	08MAY2000	0		1	Neg						
		Load Dose (Unsch.)	08MAY2000	08MAY2000			2	Neg						
		Load Dose 2	09MAY2000			Yes								
		Load Dose 3	10MAY2000			No								
		Week 1 (Unsch.)	12MAY2000			Yes								
		Week 1	15MAY2000	15MAY2000	7	No	1	Neg						
		Week 1	15MAY2000	15MAY2000		No	2	Neg						
		Week 2	22MAY2000	22MAY2000	14	No	1	Neg						
		Week 2	22MAY2000	22MAY2000		No	2	Neg						
		Week 3	29MAY2000	29MAY2000	21	No	1	Neg						
		Week 3	29MAY2000	29MAY2000		No	2	Neg						
		Week 4	05JUN2000	05JUN2000	28	No	1	Neg						
		Week 4	05JUN2000	05JUN2000		No	2	Neg						
		Week 5	12JUN2000	12JUN2000	35	Yes	1	Neg						
		Week 5	12JUN2000	12JUN2000		Yes	2	Neg						
		Week 6	19JUN2000	19JUN2000	42	No	1	Neg						
		Week 6	19JUN2000	19JUN2000		No	2	Neg						
		Week 7	26JUN2000	26JUN2000	49	No	1	Neg						
		Week 7	26JUN2000	26JUN2000		No	2	Neg						
		Week 8	04JUL2000	04JUL2000	57	No	1	Neg						
		Week 8	04JUL2000	04JUL2000		No	2	Neg						
		Week 9 (Unsch.)	09JUL2000	09JUL2000	62	Yes	1	Pos	P.f	169.5	2135.6	2010.5	250.1	
		Week 9 (Unsch.)	09JUL2000	09JUL2000		Yes	2	Pos	P.f	149.6	1885.5			
		Week 9	10JUL2000	10JUL2000	63	Yes	1	Pos	P.f	61.0	768.2	1100.0	-663.5	
		Week 9	10JUL2000	10JUL2000		Yes	2	Pos	P.f	113.6	1431.7			
		Week 10 (Fol. Up)	17JUL2000	17JUL2000	70	No	1	Neg						
		Week 10 (Fol. Up)	17JUL2000	17JUL2000		No	2	Neg						

*Popn=Population (I=Intent-To-Treat, PP=Per Protocol)
**Rel Day=Number of days relative to start of prophylactic medication
Symp = Symptoms, Rd = Reader, Res = Result, Spec = Species, Diff = Difference
Spec Count W = Species Count recorded per 500 White Blood Cells
Spec Count B = Species Count converted to per microlitre of blood
Gam = Gametocyte
+Arb=Arbitrator, +++Pos=Positive, Neg=Negative
#P. f = P.falciparum, P. v = P.vivax, P. o = P.ovale, P. m = P.malariae
Tafenoquine = Tafenoquine 200mg, Mefloquine = Mefloquine 250mg

SB252263 - 030

Listing C.01

Results Of Blood Smears By Visit

Treatment : Placebo

Subject Number	Popn *	Visit	Visit Date	Sample Date	Rel Day**	Symp ?	Rd+	Res +++	Spec #	Spec Count W	Spec Count B	Mean	Diff	Gam Count
030.001.00019	IPP	Week 11 (Fol. Up)	24JUL2000	24JUL2000	77	No	1	Neg						
		Week 11 (Fol. Up)	24JUL2000	24JUL2000		No	2	Neg						
		Week 12 (Fol. Up)	31JUL2000	31JUL2000	84	No	1	Neg						
		Week 12 (Fol. Up)	31JUL2000	31JUL2000		No	2	Neg						
		Week 13 (Fol. Up)	07AUG2000	07AUG2000	91		1	Pos	P.f	1.0	9.6	5.4	8.5	
		Week 13 (Fol. Up)	07AUG2000	07AUG2000			2	Neg						
		Week 13 (Fol. Up)	07AUG2000	07AUG2000			Arb	Pos	P.f	0.1	1.1			
030.001.00020	IPP	Screening	10APR2000	10APR2000	-28		1	Pos	P.f	2.0	19.2	12.1	14.2	
		Screening	10APR2000	10APR2000			2	Neg						
		Screening	10APR2000	10APR2000			Arb	Pos	P.f	0.5	5.0			
		Screening (Unsch.)	01MAY2000			Yes								
		Post Clearance	06MAY2000	06MAY2000	-2		1	Neg						
		Post Clearance	06MAY2000	06MAY2000			2	Neg						
		Load Dose 1	08MAY2000			No								
		Load Dose 2	09MAY2000			No								
		Load Dose 3	10MAY2000			No								
		Week 1 (Unsch.)	13MAY2000			No								
		Week 1	15MAY2000	15MAY2000	7	No	1	Neg						
		Week 1	15MAY2000	15MAY2000		No	2	Neg						
		Week 2	22MAY2000	22MAY2000	14	Yes	1	Neg						
		Week 2	22MAY2000	22MAY2000		Yes	2	Neg						
		Week 3 (Unsch.)	26MAY2000			Yes								
		Week 3	29MAY2000	29MAY2000	21	No	1	Neg						
		Week 3	29MAY2000	29MAY2000		No	2	Neg						
		Week 4	05JUN2000	05JUN2000	28	No	1	Neg						
		Week 4	05JUN2000	05JUN2000		No	2	Neg						
		Week 5	12JUN2000	12JUN2000	35	No	1	Neg						
		Week 5	12JUN2000	12JUN2000		No	2	Neg						
		Week 6	19JUN2000	19JUN2000	42	No	1	Neg						
		Week 6	19JUN2000	19JUN2000		No	2	Neg						
		Week 7	26JUN2000	26JUN2000	49	No	1	Neg						
		Week 7	26JUN2000	26JUN2000		No	2	Neg						
		Week 8	03JUL2000	03JUL2000	56	No	1	Neg						
		Week 8	03JUL2000	03JUL2000		No	2	Neg						
		Week 9	10JUL2000	10JUL2000	63	No	1	Pos	P.f	14.5	153.6	123.2	60.8	
		Week 9	10JUL2000	10JUL2000		No	2	Neg						

*Popn=Population (I=Intent-To-Treat, PP=Per Protocol)
**Rel Day=Number of days relative to start of prophylactic medication
Symp = Symptoms, Rd = Reader, Res = Result, Spec = Species, Diff = Difference
Spec Count W = Species Count recorded per 500 White Blood Cells
Spec Count B = Species Count converted to per microlitre of blood
Gam = Gametocyte
+Arb=Arbitrator, +++Pos=Positive, Neg=Negative
#P. f = P.falciparum, P. v = P.vivax, P. o = P.ovale, P. m = P.malariae
Tafenoquine = Tafenoquine 200mg, Mefloquine = Mefloquine 250mg

SB252263 - 030

Listing C.01

Results Of Blood Smears By Visit

Treatment : Placebo

Subject Number	Popn *	Visit	Visit Date	Sample Date	Rel Day**	Symp ?	Rd+	Res +++	Spec #	Spec Count W	Spec Count B	Mean	Diff	Gam Count
030.001.00020	IPP	Week 9	10JUL2000	10JUL2000		No	Arb	Pos	P.f	8.8	92.8			
		Week 10 (Fol. Up)	17JUL2000	17JUL2000	70	No	1	Pos	P.f	4.9	40.3	29.8	21.0	
		Week 10 (Fol. Up)	17JUL2000	17JUL2000		No	2	Pos	P.f	2.4	19.4			
		Week 11 (Fol. Up)	24JUL2000	24JUL2000	77	No	1	Neg						
		Week 11 (Fol. Up)	24JUL2000	24JUL2000		No	2	Pos	P.f	2.8	22.7	22.5	0.5	
		Week 11 (Fol. Up)	24JUL2000	24JUL2000		No	Arb	Pos	P.f	2.7	22.2			
		Week 11 (Unsch. Fol. Up)	24JUL2000			No								
		Week 12 (Fol. Up)	31JUL2000	31JUL2000	84	Yes	1	Pos	P.f	6.5	53.2	59.8	-13.2	
		Week 12 (Fol. Up)	31JUL2000	31JUL2000		Yes	2	Pos	P.f	8.1	66.4			
		Week 12 (Unsch. Fol. Up)	31JUL2000			Yes								
		Week 13 (Fol. Up)	07AUG2000	07AUG2000	91		1	Neg						
		Week 13 (Fol. Up)	07AUG2000	07AUG2000			2	Neg						
030.001.00022	IPP	Screening	13APR2000	13APR2000	-25		1	Neg						
		Screening	13APR2000	13APR2000			2	Neg						
		Screening (Unsch.)	01MAY2000			Yes								
		Post Clearance	06MAY2000	06MAY2000	-2		1	Neg						
		Post Clearance	06MAY2000	06MAY2000			2	Neg						
		Load Dose 1	08MAY2000			Yes								
		Load Dose 2	09MAY2000			No								
		Load Dose 3	10MAY2000			No								
		Week 1	15MAY2000	15MAY2000	7	No	1	Pos	P.f	3.5	39.6	21.4	36.3	
		Week 1	15MAY2000	15MAY2000		No	2	Pos	P.f	0.3	3.3			
		Week 2	22MAY2000	22MAY2000	14	No	1	Neg						
		Week 2	22MAY2000	22MAY2000		No	2	Neg						
		Week 2 (Unsch.)	22MAY2000	22MAY2000		Yes		Neg						
		Week 3 (Unsch. Fol. Up)	26MAY2000			No								
		Week 3 (Fol. Up)	29MAY2000	29MAY2000	21	No	1	Neg						
		Week 3 (Fol. Up)	29MAY2000	29MAY2000		No	2	Neg						
		Week 4 (Fol. Up)	05JUN2000	05JUN2000	28	No	1	Neg						
		Week 4 (Fol. Up)	05JUN2000	05JUN2000		No	2	Neg						
		Week 5 (Fol. Up)	12JUN2000	12JUN2000	35	No	1	Neg						
		Week 5 (Fol. Up)	12JUN2000	12JUN2000		No	2	Neg						
		Week 5 (Unsch. Fol. Up)	14JUN2000			No								
		Week 6 (Fol. Up)	19JUN2000	19JUN2000	42	No	1	Neg						
		Week 6 (Fol. Up)	19JUN2000	19JUN2000		No	2	Neg						
		Week 6 (Fol. Up)	19JUN2000	19JUN2000		No	Arb	Neg						

*Popn=Population (I=Intent-To-Treat, PP=Per Protocol)
**Rel Day=Number of days relative to start of prophylactic medication
Symp = Symptoms, Rd = Reader, Res = Result, Spec = Species, Diff = Difference
Spec Count W = Species Count recorded per 500 White Blood Cells
Spec Count B = Species Count converted to per microlitre of blood
Gam = Gametocyte
+Arb=Arbitrator, +++Pos=Positive, Neg=Negative
#P. f = P.falciparum, P. v = P.vivax, P. o = P.ovale, P. m = P.malariae
Tafenoquine = Tafenoquine 200mg, Mefloquine = Mefloquine 250mg

SB252263 - 030

Listing C.01

Results Of Blood Smears By Visit

Treatment : Placebo

Subject Number	Popn *	Visit	Visit Date	Sample Date	Rel Day**	Symp ?	Rd+	Res +++	Spec #	Spec Count W	Spec Count B	Mean	Diff	Gam Count
030.001.00026	IPP	Screening	10APR2000	10APR2000	-28		1	Neg						
		Screening	10APR2000	10APR2000			2	Neg						
		Screening (Unsch.)	16APR2000	16APR2000	-22	Yes	1	Neg						
		Screening (Unsch.)	16APR2000	16APR2000		Yes	2	Neg						
		Post Clearance	06MAY2000	06MAY2000	-2		1	Neg						
		Post Clearance	06MAY2000	06MAY2000			2	Neg						
		Load Dose 1	08MAY2000			No								
		Load Dose 2	09MAY2000			No								
		Load Dose 3	10MAY2000			No								
		Week 1 (Unsch.)	14MAY2000			Yes								
		Week 1	15MAY2000	15MAY2000	7	No	1	Pos	P.f	2.2	16.3	22.3	-11.9	
		Week 1	15MAY2000	15MAY2000		No	2	Pos	P.f	3.7	28.2			
		Week 2	22MAY2000	22MAY2000	14	No	1	Neg						
		Week 2	22MAY2000	22MAY2000		No	2	Neg						
		Week 4 (Fol. Up)	03JUN2000	03JUN2000	26	Yes	1	Neg						
		Week 4 (Fol. Up)	03JUN2000	03JUN2000		Yes	2	Neg						
		Week 4 (Unsch. Fol. Up)	03JUN2000			Yes								
		Week 4 (Fol. Up)	06JUN2000	06JUN2000	29	No	1	Pos	P.f	54.5	522.8	472.8	100.0	
		Week 4 (Fol. Up)	06JUN2000	06JUN2000		No	2	Pos	P.f	44.0	422.8			
		Week 5 (Fol. Up)	12JUN2000	12JUN2000	35	Yes	1	Neg						
		Week 5 (Fol. Up)	12JUN2000	12JUN2000		Yes	2	Neg						
		Week 6 (Fol. Up)	19JUN2000	19JUN2000	42	Yes	1	Neg						
		Week 6 (Fol. Up)	19JUN2000	19JUN2000		Yes	2	Neg						
		Week 6 (Unsch. Fol. Up)	19JUN2000	19JUN2000		Yes	1	Pos	P.f	0.6	4.7	3.5	2.4	
		Week 6 (Unsch. Fol. Up)	19JUN2000	19JUN2000		Yes	2	Pos	P.f	0.3	2.3			
030.001.00027	IPP	Screening	12APR2000	12APR2000	-26		1	Pos	P.f	3.6	35.6	22.5	26.1	
		Screening	12APR2000	12APR2000			2	Neg						
		Screening	12APR2000	12APR2000			Arb	Pos	P.f	1.0	9.5			
		Post Clearance	06MAY2000	06MAY2000	-2		1	Neg						
		Post Clearance	06MAY2000	06MAY2000			2	Neg						
		Load Dose 1	08MAY2000			No								
		Load Dose 2	09MAY2000			No								
		Load Dose (Unsch.)	09MAY2000			No								
		Load Dose 3	10MAY2000			No								
		Week 1	15MAY2000	15MAY2000	7	No	1	Pos	P.f	0.6	5.8	9.3	-7.0	0.3
		Week 1	15MAY2000	15MAY2000		No	2	Pos	P.f	1.2	12.8			0.6

*Popn=Population (I=Intent-To-Treat, PP=Per Protocol)
**Rel Day=Number of days relative to start of prophylactic medication
Symp = Symptoms, Rd = Reader, Res = Result, Spec = Species, Diff = Difference
Spec Count W = Species Count recorded per 500 White Blood Cells
Spec Count B = Species Count converted to per microlitre of blood
Gam = Gametocyte
+Arb=Arbitrator, +++Pos=Positive, Neg=Negative
#P. f = P.falciparum, P. v = P.vivax, P. o = P.ovale, P. m = P.malariae
Tafenoquine = Tafenoquine 200mg, Mefloquine = Mefloquine 250mg

Listing C.01

Results Of Blood Smears By Visit

Treatment : Placebo

Subject Number	Popn *	Visit	Visit Date	Sample Date	Rel Day**	Symp ?	Rd+	Res +++	Spec #	Spec Count W	Spec Count B	Mean	Diff	Gam Count
030.001.00027	IPP	Week 2	22MAY2000	22MAY2000	14	No	1	Neg						
		Week 2	22MAY2000	22MAY2000		No	2	Neg						
		Week 2 (Unsch. Fol. Up)	24MAY2000			No								
		Week 3 (Fol. Up)	30MAY2000	30MAY2000	22	No	1	Neg						
		Week 3 (Fol. Up)	30MAY2000	30MAY2000		No	2	Neg						
		Week 4 (Fol. Up)	05JUN2000	05JUN2000	28	No	1	Neg						
		Week 4 (Fol. Up)	05JUN2000	05JUN2000		No	2	Neg						
		Week 5 (Fol. Up)	12JUN2000	12JUN2000	35	No	1	Pos	P.f	0.5	6.7	8.7	-4.0	
		Week 5 (Fol. Up)	12JUN2000	12JUN2000		No	2	Pos	P.f	0.8	10.7			
		Week 6 (Fol. Up)	19JUN2000	19JUN2000	42	No	1	Neg						
		Week 6 (Fol. Up)	19JUN2000	19JUN2000		No	2	Neg						
030.001.00032	IPP	Screening	13APR2000	13APR2000	-25		1	Pos	P.f	22.4	260.0	396.2	-272.5	
		Screening	13APR2000	13APR2000			1		P.m	6.9	79.9	163.6	-167.3	
		Screening	13APR2000	13APR2000			2	Pos	P.f	45.9	532.4			
		Screening	13APR2000	13APR2000			2		P.m	21.3	247.2			
		Screening (Unsch.)	28APR2000			No								
		Post Clearance	06MAY2000	06MAY2000	-2		1	Neg						
		Post Clearance	06MAY2000	06MAY2000			2	Neg						
		Load Dose 1	08MAY2000			No								
		Load Dose 2	09MAY2000			No								
		Load Dose 3	10MAY2000			No								
		Week 1	16MAY2000	16MAY2000	8	No	1	Neg						
		Week 1	16MAY2000	16MAY2000		No	2	Neg						
		Week 2	22MAY2000	22MAY2000	14	No	1	Neg						
		Week 2	22MAY2000	22MAY2000		No	2	Neg						
		Week 2 (Unsch.)	23MAY2000			No								
		Week 3	29MAY2000	29MAY2000	21	No	1	Neg						
		Week 3	29MAY2000	29MAY2000		No	2	Neg						
		Week 4	05JUN2000	05JUN2000	28	No	1	Neg						
		Week 4	05JUN2000	05JUN2000		No	2	Neg						
		Week 4 (Unsch.)	06JUN2000			No								
		Week 5	12JUN2000	12JUN2000	35	No	1	Neg						
		Week 5	12JUN2000	12JUN2000		No	2	Neg						
		Week 6	19JUN2000	19JUN2000	42	No	1	Neg						
		Week 6	19JUN2000	19JUN2000		No	2	Neg						
		Week 7	26JUN2000	26JUN2000	49	No	1	Neg						

*Popn=Population (I=Intent-To-Treat, PP=Per Protocol)
**Rel Day=Number of days relative to start of prophylactic medication
Symp = Symptoms, Rd = Reader, Res = Result, Spec = Species, Diff = Difference
Spec Count W = Species Count recorded per 500 White Blood Cells
Spec Count B = Species Count converted to per microlitre of blood
Gam = Gametocyte
+Arb=Arbitrator, +++Pos=Positive, Neg=Negative
#P. f = P.falciparum, P. v = P.vivax, P. o = P.ovale, P. m = P.malariae
Tafenoquine = Tafenoquine 200mg, Mefloquine = Mefloquine 250mg

Listing C.01

Results Of Blood Smears By Visit

Treatment : Placebo

Subject Number	Popn *	Visit	Visit Date	Sample Date	Rel Day**	Symp ?	Rd+	Res +++	Spec #	Spec Count W	Spec Count B	Mean	Diff	Gam Count
030.001.00032	IPP	Week 7	26JUN2000	26JUN2000		No	2	Pos	P.f	1.1	13.4			
		Week 7	26JUN2000	26JUN2000		No	Arb	Neg						
		Week 8	03JUL2000	03JUL2000	56	No	1	Pos	P.f	11.7	140.4	106.2	68.4	
		Week 8	03JUL2000	03JUL2000		No	2	Pos	P.f	6.0	72.0			
		Week 9 (Fol. Up)	10JUL2000	10JUL2000	63	No	1	Pos	P.f	584.3	7012.0	7948.7	-1873.4	
		Week 9 (Fol. Up)	10JUL2000	10JUL2000		No	2	Pos	P.f	740.5	8885.4			
		Week 9 (Unsch. Fol. Up)	11JUL2000	11JUL2000	64	No	1	Pos	P.f	681.8	8862.8	8397.9	929.6	
		Week 9 (Unsch. Fol. Up)	11JUL2000	11JUL2000		No	2	Pos	P.f	610.2	7933.1			
		Week 10 (Fol. Up)	17JUL2000	17JUL2000	70	No	1	Neg						
		Week 10 (Fol. Up)	17JUL2000	17JUL2000		No	2	Neg						
		Week 11 (Fol. Up)	24JUL2000	24JUL2000	77	No	1	Neg						
		Week 11 (Fol. Up)	24JUL2000	24JUL2000		No	2	Neg						
		Week 12 (Fol. Up)	31JUL2000	31JUL2000	84		1	Neg						
		Week 12 (Fol. Up)	31JUL2000	31JUL2000			2	Neg						
030.001.00033	IPP	Screening	11APR2000	11APR2000	-27		1	Neg						
		Screening	11APR2000	11APR2000			2	Neg						
		Screening (Unsch.)	14APR2000			No								
		Screening (Unsch.)	19APR2000			No								
		Post Clearance	06MAY2000	06MAY2000	-2		1	Neg						
		Post Clearance	06MAY2000	06MAY2000			2	Neg						
		Load Dose 1	08MAY2000			No								
		Load Dose 2	09MAY2000			No								
		Load Dose 3	10MAY2000			No								
		Week 1	15MAY2000	15MAY2000	7	No	1	Pos	P.f	0.3	2.2	12.3	-20.2	
		Week 1	15MAY2000	15MAY2000		No	2	Pos	P.f	2.6	22.4			
		Week 2	22MAY2000	22MAY2000	14	No		Pos						
		Week 2 (Unsch. Fol. Up)	24MAY2000			No								
		Week 3 (Unsch. Fol. Up)	26MAY2000			No								
		Week 3 (Fol. Up)	29MAY2000	29MAY2000	21	No	1	Neg						
		Week 3 (Fol. Up)	29MAY2000	29MAY2000		No	2	Neg						
		Week 4 (Fol. Up)	04JUN2000	04JUN2000	27	No	1	Neg						
		Week 4 (Fol. Up)	04JUN2000	04JUN2000		No	2	Neg						
		Week 5 (Fol. Up)	12JUN2000	12JUN2000	35	No	1	Neg						
		Week 5 (Fol. Up)	12JUN2000	12JUN2000		No	2	Neg						
		Week 5 (Unsch. Fol. Up)	13JUN2000			Yes								
		Week 6 (Fol. Up)	19JUN2000	19JUN2000	42	No	1	Pos	P.f	2.0	17.2	17.1	0.1	

*Popn=Population (I=Intent-To-Treat, PP=Per Protocol)
**Rel Day=Number of days relative to start of prophylactic medication
Symp = Symptoms, Rd = Reader, Res = Result, Spec = Species, Diff = Difference
Spec Count W = Species Count recorded per 500 White Blood Cells
Spec Count B = Species Count converted to per microlitre of blood
Gam = Gametocyte
+Arb=Arbitrator, +++Pos=Positive, Neg=Negative
#P. f = P.falciparum, P. v = P.vivax, P. o = P.ovale, P. m = P.malariae
Tafenoquine = Tafenoquine 200mg, Mefloquine = Mefloquine 250mg

SB252263 - 030

Listing C.01

Results Of Blood Smears By Visit

Treatment : Placebo

Subject Number	Popn *	Visit	Visit Date	Sample Date	Rel Day**	Symp ?	Rd+	Res +++	Spec #	Spec Count W	Spec Count B	Mean	Diff	Gam Count
030.001.00033	IPP	Week 6 (Fol. Up)	19JUN2000	19JUN2000			1		P.o	1.0	8.5	9.9	-2.8	
		Week 6 (Fol. Up)	19JUN2000	19JUN2000		No	2	Pos	P.f	1.9	17.1			
		Week 6 (Fol. Up)	19JUN2000	19JUN2000			2		P.o	1.3	11.4			
		Week 6 (Unsch. Fol. Up)	19JUN2000			Yes								
030.001.00037	IPP	Screening	11APR2000	11APR2000	-27		1	Neg						
		Screening	11APR2000	11APR2000			2	Neg						
		Screening (Unsch.)	21APR2000			Yes								
		Post Clearance	06MAY2000	06MAY2000	-2		1	Neg						
		Post Clearance	06MAY2000	06MAY2000			2	Neg						
		Load Dose 1	08MAY2000			No								
		Load Dose 2	09MAY2000			No								
		Load Dose 3	10MAY2000			No								
		Week 1	15MAY2000	15MAY2000	7	No	1	Pos	P.f	0.2	1.9	2.7	-1.5	
		Week 1	15MAY2000	15MAY2000		No	2	Pos	P.f	0.4	3.4			
		Week 2	22MAY2000	22MAY2000	14	No	1	Pos	P.f	4.3	36.0	41.1	-10.1	
		Week 2	22MAY2000	22MAY2000		No	2	Pos	P.f	5.5	46.1			
		Week 3 (Unsch.)	26MAY2000			No								
		Week 3	29MAY2000	29MAY2000	21	No	1	Neg						
		Week 3	29MAY2000	29MAY2000		No	2	Neg						
		Week 4 (Fol. Up)	05JUN2000	05JUN2000	28	No	1	Neg						
		Week 4 (Fol. Up)	05JUN2000	05JUN2000		No	2	Neg						
		Week 5 (Fol. Up)	12JUN2000	12JUN2000	35	No	1	Neg						
		Week 5 (Fol. Up)	12JUN2000	12JUN2000		No	2	Neg						
		Week 6 (Fol. Up)	19JUN2000	19JUN2000	42	No	1	Neg						
		Week 6 (Fol. Up)	19JUN2000	19JUN2000		No	2	Neg						
		Week 7 (Unsch. Fol. Up)	23JUN2000											
		Week 7 (Fol. Up)	26JUN2000	26JUN2000	49		1	Pos	P.o	2.4	18.5	18.5	0.0	
		Week 7 (Fol. Up)	26JUN2000	26JUN2000			2	Pos	P.o	2.4	18.5			
030.001.00039	IPP	Screening	14APR2000	14APR2000	-24		1	Pos	P.f	10.9	80.7	95.5	-29.7	
		Screening	14APR2000	14APR2000			2	Pos	P.f	14.9	110.4			
		Screening (Unsch.)	28APR2000			No								
		Post Clearance	06MAY2000	06MAY2000	-2		1	Neg						
		Post Clearance	06MAY2000	06MAY2000			2	Neg						
		Load Dose 1	08MAY2000			No								
		Load Dose 2	09MAY2000			No								

*Popn=Population (I=Intent-To-Treat, PP=Per Protocol)
**Rel Day=Number of days relative to start of prophylactic medication
Symp = Symptoms, Rd = Reader, Res = Result, Spec = Species, Diff = Difference
Spec Count W = Species Count recorded per 500 White Blood Cells
Spec Count B = Species Count converted to per microlitre of blood
Gam = Gametocyte
+Arb=Arbitrator, +++Pos=Positive, Neg=Negative
#P. f = P.falciparum, P. v = P.vivax, P. o = P.ovale, P. m = P.malariae
Tafenoquine = Tafenoquine 200mg, Mefloquine = Mefloquine 250mg

SB252263 - 030

Listing C.01

Results Of Blood Smears By Visit

Treatment : Placebo

Subject Number	Popn *	Visit	Visit Date	Sample Date	Rel Day**	Symp ?	Rd+	Res +++	Spec #	Spec Count W	Spec Count B	Mean	Diff	Gam Count
030.001.00039	IPP	Load Dose 3	10MAY2000			No								
		Load Dose (Unsch.)	10MAY2000			Yes								
		Week 1	15MAY2000	15MAY2000	7	No	1	Neg						
		Week 1	15MAY2000	15MAY2000		No	2	Neg						
		Week 2	22MAY2000	22MAY2000	14	No	1	Neg						
		Week 2	22MAY2000	22MAY2000		No	2	Neg						
		Week 3	29MAY2000	29MAY2000	21	No	1	Neg						
		Week 3	29MAY2000	29MAY2000		No	2	Neg						
		Week 4	04JUN2000	04JUN2000	27	No	1	Neg						
		Week 4	04JUN2000	04JUN2000		No	2	Neg						
		Week 5	12JUN2000	12JUN2000	35	No	1	Pos	P.f	2.6	33.3	95.8	-125.0	
		Week 5	12JUN2000	12JUN2000		No	2	Pos	P.f	12.6	158.3			
		Week 6 (Fol. Up)	19JUN2000	19JUN2000	42	No	1	Pos	P.f	12.7	160.4	111.1	98.7	
		Week 6 (Fol. Up)	19JUN2000	19JUN2000		No	2	Pos	P.f	4.9	61.7			
		Week 7 (Fol. Up)	26JUN2000	26JUN2000	49	No	1	Pos	P.f	106.0	1335.6	767.0	1137.2	
		Week 7 (Fol. Up)	26JUN2000	26JUN2000		No	2	Pos	P.f	15.8	198.5			
		Week 9 (Fol. Up)	07JUL2000			No								
		Week 9 (Fol. Up)	08JUL2000	08JUL2000	61	No	1	Pos	P.f	61.9	494.8	411.5	166.6	
		Week 9 (Fol. Up)	08JUL2000	08JUL2000		No	2	Pos	P.f	41.0	328.2			
		Week 9 (Fol. Up)	10JUL2000	10JUL2000	63		1	Pos	P.f	5.0	40.0	43.4	-6.8	
		Week 9 (Fol. Up)	10JUL2000	10JUL2000			2	Pos	P.f	5.9	46.8			
030.001.00041	IPP	Screening	10APR2000	10APR2000	-28		1	Pos	P.f	4.0	43.2	25.5	35.4	
		Screening	10APR2000	10APR2000			2	Neg						
		Screening	10APR2000	10APR2000			Arb	Pos	P.f	0.7	7.8			
		Screening (Unsch.)	25APR2000			Yes								
		Post Clearance	06MAY2000	06MAY2000	-2		1	Neg						
		Post Clearance	06MAY2000	06MAY2000			2	Neg						
		Load Dose 1	08MAY2000			No								
		Load Dose 2	09MAY2000			No								
		Load Dose 3	10MAY2000			No								
		Week 1	16MAY2000	16MAY2000	8	No	1	Neg						
		Week 1	16MAY2000	16MAY2000		No	2	Neg						
		Week 2	22MAY2000	22MAY2000	14	No	1	Neg						
		Week 2	22MAY2000	22MAY2000		No	2	Neg						
		Week 2 (Unsch.)	22MAY2000			No								
		Week 3	29MAY2000	29MAY2000	21	No	1	Neg						

*Popn=Population (I=Intent-To-Treat, PP=Per Protocol)
**Rel Day=Number of days relative to start of prophylactic medication
Symp = Symptoms, Rd = Reader, Res = Result, Spec = Species, Diff = Difference
Spec Count W = Species Count recorded per 500 White Blood Cells
Spec Count B = Species Count converted to per microlitre of blood
Gam = Gametocyte
+Arb=Arbitrator, +++Pos=Positive, Neg=Negative
#P. f = P.falciparum, P. v = P.vivax, P. o = P.ovale, P. m = P.malariae
Tafenoquine = Tafenoquine 200mg, Mefloquine = Mefloquine 250mg

SB252263 - 030

Listing C.01

Results Of Blood Smears By Visit

Treatment : Placebo

Subject Number	Popn *	Visit	Visit Date	Sample Date	Rel Day**	Symp ?	Rd+	Res +++	Spec #	Spec Count W	Spec Count B	Mean	Diff	Gam Count
030.001.00041	IPP	Week 3	29MAY2000	29MAY2000		No	2	Neg						
		Week 4	05JUN2000	05JUN2000	28	No	1	Neg						
		Week 4	05JUN2000	05JUN2000		No	2	Neg						
		Week 4 (Unsch.)	06JUN2000			No								
		Week 5	12JUN2000	12JUN2000	35	No	1	Neg						
		Week 5	12JUN2000	12JUN2000		No	2	Pos	P.f	0.2	6.0	6.8	-1.5	
		Week 5	12JUN2000	12JUN2000		No	Arb	Pos	P.f	0.3	7.5			
		Week 6 (Fol. Up)	19JUN2000	19JUN2000	42	No	1	Neg						
		Week 6 (Fol. Up)	19JUN2000	19JUN2000		No	2	Neg						
		Week 7 (Unsch. Fol. Up)	23JUN2000	23JUN2000	46	Yes	1	Neg						
		Week 7 (Unsch. Fol. Up)	23JUN2000	23JUN2000		Yes	2	Neg						
		Week 7 (Fol. Up)	26JUN2000	26JUN2000	49	No	1	Pos	P.f	9.0	130.2			
		Week 7 (Fol. Up)	26JUN2000	26JUN2000		No	2	Pos	P.f	3.5	50.4			
		Week 7 (Fol. Up)	26JUN2000	26JUN2000		No	Arb	Pos	P.f	9.0	130.2			
		Week 8 (Fol. Up)	03JUL2000	03JUL2000	56	No	1	Neg						
		Week 8 (Fol. Up)	03JUL2000	03JUL2000		No	2	Neg						
		Week 8 (Unsch. Fol. Up)	04JUL2000			No								
		Week 9 (Fol. Up)	10JUL2000	10JUL2000	63	No	1	Neg						
		Week 9 (Fol. Up)	10JUL2000	10JUL2000		No	2	Neg						
030.001.00044	IPP	Screening	10APR2000	10APR2000	-28		1	Pos	P.f	3.3	38.5	39.8	-2.7	
		Screening	10APR2000	10APR2000			1		P.m	1.4	16.4	16.4	0.0	
		Screening	10APR2000	10APR2000			2	Pos	P.f	3.5	41.2			
		Screening	10APR2000	10APR2000			2		P.m	1.4	16.4			
		Post Clearance	06MAY2000	06MAY2000	-2		1	Neg						
		Post Clearance	06MAY2000	06MAY2000			2	Neg						
		Load Dose 1	08MAY2000			No								
		Load Dose 2	09MAY2000			No								
		Load Dose 3	10MAY2000			No								
		Week 1 (Unsch.)	14MAY2000			Yes								
		Week 1	15MAY2000	15MAY2000	7	No	1	Neg						
		Week 1	15MAY2000	15MAY2000		No	2	Neg						
		Week 2	22MAY2000	22MAY2000	14	No	1	Neg						
		Week 2	22MAY2000	22MAY2000		No	2	Neg						
		Week 3	29MAY2000	29MAY2000	21	No	1	Neg						
		Week 3	29MAY2000	29MAY2000		No	2	Neg						
		Week 4 (Unsch.)	02JUN2000			Yes								

*Popn=Population (I=Intent-To-Treat, PP=Per Protocol)
**Rel Day=Number of days relative to start of prophylactic medication
Symp = Symptoms, Rd = Reader, Res = Result, Spec = Species, Diff = Difference
Spec Count W = Species Count recorded per 500 White Blood Cells
Spec Count B = Species Count converted to per microlitre of blood
Gam = Gametocyte
+Arb=Arbitrator, +++Pos=Positive, Neg=Negative
#P. f = P.falciparum, P. v = P.vivax, P. o = P.ovale, P. m = P.malariae
Tafenoquine = Tafenoquine 200mg, Mefloquine = Mefloquine 250mg

SB252263 - 030

Listing C.01

Results Of Blood Smears By Visit

Treatment : Placebo

Subject Number	Popn *	Visit	Visit Date	Sample Date	Rel Day**	Symp ?	Rd+	Res +++	Spec #	Spec Count W	Spec Count B	Mean	Diff	Gam Count
030.001.00044	IPP	Week 4	05JUN2000	05JUN2000	28	No	1	Neg						
		Week 4	05JUN2000	05JUN2000		No	2	Neg						
		Week 5 (Unsch.)	11JUN2000			No								
		Week 5	12JUN2000	12JUN2000	35	Yes	1	Neg						
		Week 5	12JUN2000	12JUN2000		Yes	2	Neg						
		Week 6	19JUN2000	19JUN2000	42	Yes	1	Pos	P.f	33.1	383.4	254.5	257.8	
		Week 6	19JUN2000	19JUN2000		Yes	2	Pos	P.f	10.8	125.6			
		Week 6 (Unsch. Fol. Up)	22JUN2000			Yes								
		Week 7 (Unsch. Fol. Up)	24JUN2000			Yes								
		Week 7 (Fol. Up)	26JUN2000	26JUN2000	49	No	1	Neg						
		Week 7 (Fol. Up)	26JUN2000	26JUN2000		No	2	Neg						
		Week 7 (Unsch. Fol. Up)	26JUN2000			Yes								
		Week 7 (Unsch. Fol. Up)	29JUN2000			No								
		Week 8 (Fol. Up)	03JUL2000	03JUL2000	56	No	1	Neg						
		Week 8 (Fol. Up)	03JUL2000	03JUL2000		No	2	Neg						
		Week 9 (Unsch. Fol. Up)	07JUL2000			No								
		Week 9 (Fol. Up)	10JUL2000	10JUL2000	63	Yes	1	Pos	P.f	1.8	20.7	14.8	11.8	
		Week 9 (Fol. Up)	10JUL2000	10JUL2000		Yes	2	Pos	P.f	0.8	8.9			
		Week 10 (Fol. Up)	17JUL2000	17JUL2000	70		1	Neg						
		Week 10 (Fol. Up)	17JUL2000	17JUL2000			2	Neg						
		Week 10 (Unsch. Fol. Up)	17JUL2000			No								
		Week 10 (Unsch. Fol. Up)	18JUL2000			No								
030.001.00055	IPP	Screening	12APR2000	12APR2000	-26		1	Pos	P.f	3.1	32.7	25.4	14.5	
		Screening	12APR2000	12APR2000			2	Pos	P.f	1.8	18.2			
		Post Clearance	06MAY2000	06MAY2000	-2		1	Neg						
		Post Clearance	06MAY2000	06MAY2000			2	Neg						
		Load Dose 1	08MAY2000			No								
		Load Dose 2	09MAY2000			No								
		Load Dose 3	10MAY2000			No								
		Week 1	15MAY2000	15MAY2000	7	No	1	Neg						
		Week 1	15MAY2000	15MAY2000		No	2	Neg						
		Week 2	22MAY2000	22MAY2000	14	No	1	Pos	P.f	2.5	20.3	11.2	18.2	
		Week 2	22MAY2000	22MAY2000		No	2	Pos	P.f	0.3	2.1			
		Week 3 (Fol. Up)	29MAY2000	29MAY2000	21	No	1	Neg						
		Week 3 (Fol. Up)	29MAY2000	29MAY2000		No	2	Neg						
		Week 4 (Fol. Up)	05JUN2000	05JUN2000	28	Yes	1	Neg						

*Popn=Population (I=Intent-To-Treat, PP=Per Protocol)
**Rel Day=Number of days relative to start of prophylactic medication
Symp = Symptoms, Rd = Reader, Res = Result, Spec = Species, Diff = Difference
Spec Count W = Species Count recorded per 500 White Blood Cells
Spec Count B = Species Count converted to per microlitre of blood
Gam = Gametocyte
+Arb=Arbitrator, +++Pos=Positive, Neg=Negative
#P. f = P.falciparum, P. v = P.vivax, P. o = P.ovale, P. m = P.malariae
Tafenoquine = Tafenoquine 200mg, Mefloquine = Mefloquine 250mg

SB252263 - 030

Listing C.01

Results Of Blood Smears By Visit

Treatment : Placebo

Subject Number	Popn *	Visit	Visit Date	Sample Date	Rel Day**	Symp ?	Rd+	Res +++	Spec #	Spec Count W	Spec Count B	Mean	Diff	Gam Count
030.001.00055	IPP	Week 4 (Fol. Up)	05JUN2000	05JUN2000		Yes	2	Neg						
		Week 5 (Fol. Up)	12JUN2000	12JUN2000	35	No	1	Neg						
		Week 5 (Fol. Up)	12JUN2000	12JUN2000		No	2	Neg						
		Week 6 (Fol. Up)	19JUN2000	19JUN2000	42	No	1	Neg						
		Week 6 (Fol. Up)	19JUN2000	19JUN2000		No	2	Neg						
030.001.00056	I	Screening	11APR2000	11APR2000	-27		1	Neg						
		Screening	11APR2000	11APR2000			2	Neg						
		Screening (Unsch.)	18APR2000			Yes								
		Screening (Unsch.)	28APR2000			Yes								
		Post Clearance	06MAY2000	06MAY2000	-2		1	Neg						
		Post Clearance	06MAY2000	06MAY2000			2	Neg						
		Post Clearance (Unsch.)	06MAY2000			Yes								
		Load Dose 1	08MAY2000			No								
		Load Dose 2	09MAY2000			No								
		Load Dose 3	10MAY2000			No								
		Week 1	15MAY2000	15MAY2000	7	No	1	Neg						
		Week 1	15MAY2000	15MAY2000		No	2	Neg						
		Week 1 (Unsch.)	16MAY2000			Yes								
		Week 2	22MAY2000	22MAY2000	14	Yes	1	Neg						
		Week 2	22MAY2000	22MAY2000		Yes	2	Neg						
		Week 3	29MAY2000	29MAY2000	21	No	1	Neg						
		Week 3	29MAY2000	29MAY2000		No	2	Neg						
		Week 4	05JUN2000	05JUN2000	28	Yes	1	Neg						
		Week 4	05JUN2000	05JUN2000		Yes	2	Neg						
		Week 4 (Unsch.)	06JUN2000	06JUN2000	29	Yes	1	Neg						
		Week 4 (Unsch.)	06JUN2000	06JUN2000		Yes	2	Neg						
		Week 5	12JUN2000	12JUN2000	35	Yes	1	Neg						
		Week 5	12JUN2000	12JUN2000		Yes	2	Neg						
		Week 6 (Fol. Up)	19JUN2000			No								
		Week 8 (Fol. Up)	30JUN2000	30JUN2000	53	No	1	Pos	P.f	13.8	195.8	210.6	-29.5	
		Week 8 (Fol. Up)	30JUN2000	30JUN2000			1		P.o	3.4	48.8	58.2	-18.7	
		Week 8 (Fol. Up)	30JUN2000	30JUN2000		No	2	Pos	P.f	15.9	225.4			
		Week 8 (Fol. Up)	30JUN2000	30JUN2000			2		P.o	4.8	67.6			
		Week 8 (Unsch. Fol. Up)	01JUL2000			No								
		Week 8 (Fol. Up)	05JUL2000	05JUL2000	58	No	1	Pos	P.f	3.4	39.9	32.6	14.6	
		Week 8 (Fol. Up)	05JUL2000	05JUL2000			1		P.o	1.7	19.9			

*Popn=Population (I=Intent-To-Treat, PP=Per Protocol)
**Rel Day=Number of days relative to start of prophylactic medication
Symp = Symptoms, Rd = Reader, Res = Result, Spec = Species, Diff = Difference
Spec Count W = Species Count recorded per 500 White Blood Cells
Spec Count B = Species Count converted to per microlitre of blood
Gam = Gametocyte
+Arb=Arbitrator, +++Pos=Positive, Neg=Negative
#P. f = P.falciparum, P. v = P.vivax, P. o = P.ovale, P. m = P.malariae
Tafenoquine = Tafenoquine 200mg, Mefloquine = Mefloquine 250mg

Listing C.01

Results Of Blood Smears By Visit

Treatment : Placebo

Subject Number	Popn *	Visit	Visit Date	Sample Date	Rel Day**	Symp ?	Rd+	Res +++	Spec #	Spec Count W	Spec Count B	Mean	Diff	Gam Count
030.001.00056	I	Week 8 (Fol. Up)	05JUL2000	05JUL2000		No	2	Pos	P.f	2.1	25.3			
		Week 9 (Fol. Up)	10JUL2000	10JUL2000	63	No	1	Neg						
		Week 9 (Fol. Up)	10JUL2000	10JUL2000		No	2	Neg						
030.001.00057	IPP	Screening	14APR2000	14APR2000	-24		1	Pos	P.f	6.1	81.5	54.2	54.5	
		Screening	14APR2000	14APR2000			2	Pos	P.f	2.0	26.9			
		Post Clearance	06MAY2000	06MAY2000	-2		1	Neg						
		Post Clearance	06MAY2000	06MAY2000			2	Neg						
		Load Dose 1	08MAY2000			No								
		Load Dose 2	09MAY2000			No								
		Load Dose 3	10MAY2000			Yes								
		Load Dose (Unsch.)	10MAY2000			Yes								
		Week 1	15MAY2000	15MAY2000	7	Yes	1	Neg						
		Week 1	15MAY2000	15MAY2000		Yes	2	Neg						
		Week 1 (Unsch.)	18MAY2000			Yes								
		Week 2	22MAY2000	22MAY2000	14	No	1	Neg						
		Week 2	22MAY2000	22MAY2000		No	2	Neg						
		Week 3	29MAY2000	29MAY2000	21	No	1	Neg						
		Week 3	29MAY2000	29MAY2000		No	2	Neg						
		Week 4	05JUN2000	05JUN2000	28	Yes	1	Neg						
		Week 4	05JUN2000	05JUN2000		Yes	2	Neg						
		Week 5	12JUN2000	12JUN2000	35	No	1	Neg						
		Week 5	12JUN2000	12JUN2000		No	2	Neg						
		Week 6	19JUN2000	19JUN2000	42	No	1	Neg						
		Week 6	19JUN2000	19JUN2000		No	2	Neg						
		Week 7	26JUN2000	26JUN2000	49	No	1	Pos	P.f	18.1	205.8	147.4	116.7	
		Week 7	26JUN2000	26JUN2000		No	2	Pos	P.f	7.8	89.0			
		Week 8 (Fol. Up)	03JUL2000	03JUL2000	56	No	1	Neg						
		Week 8 (Fol. Up)	03JUL2000	03JUL2000		No	2	Neg						
		Week 9 (Fol. Up)	11JUL2000	11JUL2000	64	No	1	Pos	P.f	9.9	114.3	108.9	10.8	
		Week 9 (Fol. Up)	11JUL2000	11JUL2000		No	2	Pos	P.f	8.9	103.5			
		Week 10 (Fol. Up)	17JUL2000	17JUL2000	70	No	1	Neg						
		Week 10 (Fol. Up)	17JUL2000	17JUL2000		No	2	Neg						
		Week 10 (Unsch. Fol. Up)	19JUL2000	19JUL2000	72		1	Neg						
		Week 10 (Unsch. Fol. Up)	19JUL2000	19JUL2000			2	Neg						
		Week 10 (Unsch. Fol. Up)	20JUL2000	20JUL2000	73		1	Pos	P.f	0.3	3.7	34.4	-61.4	
		Week 10 (Unsch. Fol. Up)	20JUL2000	20JUL2000			2	Pos	P.f	5.6	65.1			

*Popn=Population (I=Intent-To-Treat, PP=Per Protocol)
**Rel Day=Number of days relative to start of prophylactic medication
Symp = Symptoms, Rd = Reader, Res = Result, Spec = Species, Diff = Difference
Spec Count W = Species Count recorded per 500 White Blood Cells
Spec Count B = Species Count converted to per microlitre of blood
Gam = Gametocyte
+Arb=Arbitrator, +++Pos=Positive, Neg=Negative
#P. f = P.falciparum, P. v = P.vivax, P. o = P.ovale, P. m = P.malariae
Tafenoquine = Tafenoquine 200mg, Mefloquine = Mefloquine 250mg

SB252263 - 030

Listing C.01

Results Of Blood Smears By Visit

Treatment : Placebo

Subject Number	Popn *	Visit	Visit Date	Sample Date	Rel Day**	Symp ?	Rd+	Res +++	Spec #	Spec Count W	Spec Count B	Mean	Diff	Gam Count
030.001.00057	IPP	Week 11 (Fol. Up)	24JUL2000	24JUL2000	77	No	1	Neg						
		Week 11 (Fol. Up)	24JUL2000	24JUL2000		No	2	Neg						
030.001.00059	IPP	Screening	13APR2000	13APR2000	-25		1	Pos	P.f	2.0	16.4	11.0	10.8	
		Screening	13APR2000	13APR2000			2	Neg						
		Screening	13APR2000	13APR2000			Arb	Pos	P.f	0.7	5.6			
		Screening (Unsch.)	14APR2000			Yes								
		Screening (Unsch.)	26APR2000			Yes								
		Post Clearance	06MAY2000	06MAY2000	-2		1	Neg						
		Post Clearance	06MAY2000	06MAY2000			2	Neg						
		Post Clearance (Unsch.)	06MAY2000	06MAY2000		No		Neg						
		Load Dose 1	08MAY2000			No								
		Load Dose 2	09MAY2000			No								
		Load Dose 3	10MAY2000			No								
		Week 1	15MAY2000	15MAY2000	7	Yes	1	Pos	P.f	0.3	2.8	15.8	-26.0	
		Week 1	15MAY2000	15MAY2000		Yes	2	Pos	P.f	3.2	28.8			
		Week 1 (Unsch.)	16MAY2000			Yes								
		Week 2	22MAY2000	22MAY2000	14	No	1	Neg						
		Week 2	22MAY2000	22MAY2000		No	2	Neg						
		Week 2 (Unsch. Fol. Up)	24MAY2000			Yes								
		Week 3 (Fol. Up)	29MAY2000	29MAY2000	21	Yes	1	Neg						
		Week 3 (Fol. Up)	29MAY2000	29MAY2000		Yes	2	Neg						
		Week 3 (Unsch. Fol. Up)	29MAY2000			Yes								
		Week 4 (Fol. Up)	05JUN2000	05JUN2000	28	No	1	Pos	P.f	4.5	47.9	47.6	0.6	
		Week 4 (Fol. Up)	05JUN2000	05JUN2000		No	2	Pos	P.f	4.5	47.3			
		Week 5 (Fol. Up)	12JUN2000	12JUN2000	35	Yes	1	Pos	P.f	0.4	4.0	9.6	-11.2	
		Week 5 (Fol. Up)	12JUN2000	12JUN2000		Yes	2	Pos	P.f	1.4	15.3			
		Week 6 (Fol. Up)	19JUN2000	19JUN2000	42	No	1	Pos	P.f	3.7	33.3	29.9	6.8	
		Week 6 (Fol. Up)	19JUN2000	19JUN2000		No	2	Neg						
		Week 6 (Fol. Up)	19JUN2000	19JUN2000		No	Arb	Pos	P.f	3.0	26.6			
030.001.00063	IPP	Screening	11APR2000	11APR2000	-27		1	Pos	P.f	14.2	119.2	162.6	-86.8	
		Screening	11APR2000	11APR2000			2	Pos	P.f	24.5	206.0			
		Post Clearance	06MAY2000	06MAY2000	-2		1	Neg						
		Post Clearance	06MAY2000	06MAY2000			2	Neg						
		Load Dose 1	08MAY2000			No								
		Load Dose 2	09MAY2000			No								

*Popn=Population (I=Intent-To-Treat, PP=Per Protocol)
**Rel Day=Number of days relative to start of prophylactic medication
Symp = Symptoms, Rd = Reader, Res = Result, Spec = Species, Diff = Difference
Spec Count W = Species Count recorded per 500 White Blood Cells
Spec Count B = Species Count converted to per microlitre of blood
Gam = Gametocyte
+Arb=Arbitrator, +++Pos=Positive, Neg=Negative
#P. f = P.falciparum, P. v = P.vivax, P. o = P.ovale, P. m = P.malariae
Tafenoquine = Tafenoquine 200mg, Mefloquine = Mefloquine 250mg

Listing C.01

Results Of Blood Smears By Visit

Treatment : Placebo

Subject Number	Popn *	Visit	Visit Date	Sample Date	Rel Day**	Symp ?	Rd+	Res +++	Spec #	Spec Count W	Spec Count B	Mean	Diff	Gam Count
030.001.00063	IPP	Load Dose 3	10MAY2000			No								
		Week 1 (Unsch.)	11MAY2000			No								
		Week 1	15MAY2000	15MAY2000	7	No	1	Neg						
		Week 1	15MAY2000	15MAY2000		No	2	Neg						
		Week 2	22MAY2000	22MAY2000	14	No	1	Neg						
		Week 2	22MAY2000	22MAY2000		No	2	Neg						
		Week 2 (Unsch.)	22MAY2000			No								
		Week 3	29MAY2000	29MAY2000	21	No	1	Neg						
		Week 3	29MAY2000	29MAY2000		No	2	Neg						
		Week 3 (Unsch.)	30MAY2000			Yes								
		Week 4	05JUN2000	05JUN2000	28	No	1	Neg						
		Week 4	05JUN2000	05JUN2000		No	2	Neg						
		Week 5	12JUN2000	12JUN2000	35	No	1	Neg						
		Week 5	12JUN2000	12JUN2000		No	2	Neg						
		Week 6	19JUN2000	19JUN2000	42	No	1	Neg						
		Week 6	19JUN2000	19JUN2000		No	2	Neg						
		Week 7	26JUN2000	26JUN2000	49	Yes	1	Neg						
		Week 7	26JUN2000	26JUN2000		Yes	2	Neg						
		Week 8	03JUL2000	03JUL2000	56	No	1	Pos	P.f	15.2	127.9	165.2	-74.5	
		Week 8	03JUL2000	03JUL2000		No	2	Pos	P.f	24.1	202.4			
		Week 9 (Fol. Up)	11JUL2000	11JUL2000	64	No	1	Neg						
		Week 9 (Fol. Up)	11JUL2000	11JUL2000			2	Pos	P.o	0.4	3.3	4.5	-2.4	
		Week 9 (Fol. Up)	11JUL2000	11JUL2000			Arb	Pos	P.o	0.7	5.6			
		Week 10 (Fol. Up)	17JUL2000	17JUL2000	70	No	1	Pos	P.f	6.3	54.5	53.1	2.8	
		Week 10 (Fol. Up)	17JUL2000	17JUL2000		No	2	Neg						
		Week 10 (Fol. Up)	17JUL2000	17JUL2000		No	Arb	Pos	P.f	6.0	51.8			
		Week 11 (Fol. Up)	24JUL2000	24JUL2000	77	No	1	Pos	P.f	12.7	101.7	112.8	-22.3	
		Week 11 (Fol. Up)	24JUL2000	24JUL2000		No	2	Pos	P.f	15.5	124.0			
		Week 12 (Fol. Up)	31JUL2000	31JUL2000	84	No	1	Pos	P.f	1.1	8.5	10.8	-4.6	
		Week 12 (Fol. Up)	31JUL2000	31JUL2000		No	2	Pos	P.f	1.6	13.0			
030.001.00064	IPP	Screening	11APR2000	11APR2000	-27		1	Pos	P.f	1.1	16.9	34.6	-35.4	
		Screening	11APR2000	11APR2000			2	Pos	P.f	3.4	52.3			
		Screening (Unsch.)	30APR2000	30APR2000	-8	Yes	1	Neg						
		Screening (Unsch.)	30APR2000	30APR2000		Yes	2	Neg						
		Post Clearance	06MAY2000	06MAY2000	-2		1	Neg						
		Post Clearance	06MAY2000	06MAY2000			2	Neg						

*Popn=Population (I=Intent-To-Treat, PP=Per Protocol)
**Rel Day=Number of days relative to start of prophylactic medication
Symp = Symptoms, Rd = Reader, Res = Result, Spec = Species, Diff = Difference
Spec Count W = Species Count recorded per 500 White Blood Cells
Spec Count B = Species Count converted to per microlitre of blood
Gam = Gametocyte
+Arb=Arbitrator, +++Pos=Positive, Neg=Negative
#P. f = P.falciparum, P. v = P.vivax, P. o = P.ovale, P. m = P.malariae
Tafenoquine = Tafenoquine 200mg, Mefloquine = Mefloquine 250mg

Listing C.01

Results Of Blood Smears By Visit

Treatment : Placebo

Subject Number	Popn *	Visit	Visit Date	Sample Date	Rel Day**	Symp ?	Rd+	Res +++	Spec #	Spec Count W	Spec Count B	Mean	Diff	Gam Count
030.001.00064	IPP	Load Dose 1	08MAY2000			No								
		Load Dose 2	09MAY2000			No								
		Load Dose 3	10MAY2000			No								
		Week 1 (Unsch.)	13MAY2000			Yes								
		Week 1	15MAY2000	15MAY2000	7	No	1	Neg						
		Week 1	15MAY2000	15MAY2000		No	2	Neg						
		Week 2 (Unsch.)	21MAY2000			Yes								
		Week 2	22MAY2000	22MAY2000	14	No	1	Neg						
		Week 2	22MAY2000	22MAY2000		No	2	Neg						
		Week 3	29MAY2000	29MAY2000	21	No	1	Neg						
		Week 3	29MAY2000	29MAY2000		No	2	Neg						
		Week 4	04JUN2000	04JUN2000	27	Yes	1	Neg						
		Week 4	04JUN2000	04JUN2000		Yes	2	Neg						
		Week 5	12JUN2000	12JUN2000	35	No	1	Neg						
		Week 5	12JUN2000	12JUN2000		No	2	Neg						
		Week 6	19JUN2000	19JUN2000	42	No	1	Neg						
		Week 6	19JUN2000	19JUN2000		No	2	Neg						
		Week 7	26JUN2000	26JUN2000	49	No	1	Pos	P.f	8.2	112.6	122.1	-18.9	
		Week 7	26JUN2000	26JUN2000		No	2	Pos	P.f	9.5	131.5			
		Week 8 (Fol. Up)	03JUL2000	03JUL2000	56	No	1	Pos	P.f	9.6	158.9	334.7	-351.8	
		Week 8 (Fol. Up)	03JUL2000	03JUL2000		No	2	Pos	P.f	30.8	510.6			
		Week 8 (Unsch. Fol. Up)	05JUL2000			Yes								
		Week 9 (Fol. Up)	10JUL2000	10JUL2000	63	No	1	Neg						
		Week 9 (Fol. Up)	10JUL2000	10JUL2000		No	2	Neg						
		Week 10 (Unsch. Fol. Up)	16JUL2000			No								
		Week 10 (Fol. Up)	17JUL2000	17JUL2000	70	No	1	Neg						
		Week 10 (Fol. Up)	17JUL2000	17JUL2000		No	2	Neg						
		Week 11 (Fol. Up)	24JUL2000	24JUL2000	77	No	1	Neg						
		Week 11 (Fol. Up)	24JUL2000	24JUL2000		No	2	Neg						
030.001.00067	IPP	Screening	12APR2000	12APR2000	-26		1	Pos	P.f	0.5	4.2	5.2	-2.0	
		Screening	12APR2000	12APR2000			2	Pos	P.f	0.7	6.3			
		Screening (Unsch.)	15APR2000			Yes								
		Screening (Unsch.)	18APR2000	18APR2000	-20	Yes	1	Neg						
		Screening (Unsch.)	18APR2000	18APR2000		Yes	2	Neg						
		Post Clearance	07MAY2000	07MAY2000	-1		1	Neg						
		Post Clearance	07MAY2000	07MAY2000			2	Neg						

*Popn=Population (I=Intent-To-Treat, PP=Per Protocol)
**Rel Day=Number of days relative to start of prophylactic medication
Symp = Symptoms, Rd = Reader, Res = Result, Spec = Species, Diff = Difference
Spec Count W = Species Count recorded per 500 White Blood Cells
Spec Count B = Species Count converted to per microlitre of blood
Gam = Gametocyte
+Arb=Arbitrator, +++Pos=Positive, Neg=Negative
#P. f = P.falciparum, P. v = P.vivax, P. o = P.ovale, P. m = P.malariae
Tafenoquine = Tafenoquine 200mg, Mefloquine = Mefloquine 250mg

SB252263 - 030

Listing C.01

Results Of Blood Smears By Visit

Treatment : Placebo

Subject Number	Popn *	Visit	Visit Date	Sample Date	Rel Day**	Symp ?	Rd+	Res +++	Spec #	Spec Count W	Spec Count B	Mean	Diff	Gam Count
030.001.00067	IPP	Load Dose 1	08MAY2000			No								
		Load Dose 2	09MAY2000			No								
		Load Dose 3	10MAY2000			No								
		Week 1 (Unsch.)	12MAY2000			Yes								
		Week 1 (Unsch.)	13MAY2000			Yes								
		Week 1	15MAY2000	15MAY2000	7	No	1	Neg						
		Week 1	15MAY2000	15MAY2000		No	2	Neg						
		Week 2	23MAY2000	23MAY2000	15	Yes	1	Neg						
		Week 2	23MAY2000	23MAY2000		Yes	2	Neg						
		Week 3	29MAY2000	29MAY2000	21	Yes	1	Neg						
		Week 3	29MAY2000	29MAY2000		Yes	2	Neg						
		Week 3 (Unsch.)	29MAY2000	29MAY2000		Yes	1	Neg						
		Week 3 (Unsch.)	29MAY2000	29MAY2000		Yes	2	Neg						
		Week 4	05JUN2000	05JUN2000	28	Yes	1	Neg						
		Week 4	05JUN2000	05JUN2000		Yes	2	Neg						
		Week 5	12JUN2000	12JUN2000	35	Yes	1	Neg						
		Week 5	12JUN2000	12JUN2000		Yes	2	Neg						
		Week 6	19JUN2000	19JUN2000	42	No	1	Neg						
		Week 6	19JUN2000	19JUN2000		No	2	Neg						
		Week 7	26JUN2000	26JUN2000	49	Yes	1	Neg						
		Week 7	26JUN2000	26JUN2000		Yes	2	Neg						
		Week 9	07JUL2000	07JUL2000	60	Yes	1	Neg						
		Week 9	07JUL2000	07JUL2000		Yes	2	Neg						
		Week 9	10JUL2000	10JUL2000	63	No	1	Neg						
		Week 9	10JUL2000	10JUL2000		No	2	Neg						
		Week 9 (Unsch.)	13JUL2000			Yes								
		Week 10	20JUL2000	20JUL2000	73	No	1	Neg						
		Week 10	20JUL2000	20JUL2000		No	2	Neg						
		Week 11	24JUL2000	24JUL2000	77	No	1	Neg						
		Week 11	24JUL2000	24JUL2000		No	2	Neg						
		Week 12	31JUL2000	31JUL2000	84	No	1	Neg						
		Week 12	31JUL2000	31JUL2000		No	2	Neg						
		Week 13	07AUG2000	07AUG2000	91	No	1	Pos	P.f	3.7	38.1	40.1	-4.0	
		Week 13	07AUG2000	07AUG2000		No	2	Neg						
		Week 13	07AUG2000	07AUG2000		No	Arb	Pos	P.f	4.1	42.1			
		Week 13 (Unsch.)	07AUG2000	07AUG2000		Yes	1	Pos	P.f	0.3	2.7	6.4	-7.5	
		Week 13 (Unsch.)	07AUG2000	07AUG2000		Yes	2	Pos	P.f	1.0	10.2			

*Popn=Population (I=Intent-To-Treat, PP=Per Protocol)
**Rel Day=Number of days relative to start of prophylactic medication
Symp = Symptoms, Rd = Reader, Res = Result, Spec = Species, Diff = Difference
Spec Count W = Species Count recorded per 500 White Blood Cells
Spec Count B = Species Count converted to per microlitre of blood
Gam = Gametocyte
+Arb=Arbitrator, +++Pos=Positive, Neg=Negative
#P. f = P.falciparum, P. v = P.vivax, P. o = P.ovale, P. m = P.malariae
Tafenoquine = Tafenoquine 200mg, Mefloquine = Mefloquine 250mg

SB252263 - 030

Listing C.01

Results Of Blood Smears By Visit

Treatment : Placebo

Subject Number	Popn *	Visit	Visit Date	Sample Date	Rel Day**	Symp ?	Rd+	Res +++	Spec #	Spec Count W	Spec Count B	Mean	Diff	Gam Count
030.001.00067	IPP	Week 14 (Fol. Up)	14AUG2000	14AUG2000	98	No	1	Neg						
		Week 14 (Fol. Up)	14AUG2000	14AUG2000		No	2	Neg						
		Week 15 (Fol. Up)	21AUG2000	21AUG2000	105	No	1	Neg						
		Week 15 (Fol. Up)	21AUG2000	21AUG2000		No	2	Neg						
		Week 15 (Unsch. Fol. Up)	24AUG2000			No								
		Week 16 (Fol. Up)	28AUG2000	28AUG2000	112	No	1	Neg						
		Week 16 (Fol. Up)	28AUG2000	28AUG2000		No	2	Neg						
		Week 17 (Fol. Up)	04SEP2000	04SEP2000	119	No	1	Neg						
		Week 17 (Fol. Up)	04SEP2000	04SEP2000		No	2	Neg						
030.001.00072	IPP	Screening	12APR2000	12APR2000	-26		1	Neg						
		Screening	12APR2000	12APR2000			2	Pos	P.f	1.0	22.4	14.2	16.4	
		Screening	12APR2000	12APR2000			Arb	Pos	P.f	0.3	6.0			
		Screening (Unsch.)	27APR2000			Yes								
		Post Clearance	06MAY2000	06MAY2000	-2		1	Neg						
		Post Clearance	06MAY2000	06MAY2000			2	Neg						
		Load Dose 1	08MAY2000			No								
		Load Dose 2	09MAY2000			No								
		Load Dose 3	10MAY2000			No								
		Week 1 (Unsch.)	11MAY2000			No								
		Week 1	15MAY2000	15MAY2000	7	No	1	Neg						
		Week 1	15MAY2000	15MAY2000		No	2	Neg						
		Week 2	22MAY2000	22MAY2000	14	No	1	Neg						
		Week 2	22MAY2000	22MAY2000		No	2	Neg						
		Week 3	29MAY2000	29MAY2000	21	No	1	Neg						
		Week 3	29MAY2000	29MAY2000		No	2	Neg						
		Week 3 (Unsch.)	29MAY2000			No								
		Week 3 (Unsch.)	31MAY2000			Yes								
		Week 4	06JUN2000	06JUN2000	29	Yes	1	Neg						
		Week 4	06JUN2000	06JUN2000		Yes	2	Neg						
		Week 5	12JUN2000	12JUN2000	35	No	1	Neg						
		Week 5	12JUN2000	12JUN2000		No	2	Neg						
		Week 6	21JUN2000	21JUN2000	44	No	1	Neg						
		Week 6	21JUN2000	21JUN2000		No	2	Neg						
		Week 7	26JUN2000	26JUN2000	49	Yes	1	Pos	P.f	2.4	28.4	69.0	-81.2	
		Week 7	26JUN2000	26JUN2000		Yes	2	Pos	P.f	9.3	109.6			
		Week 7 (Unsch.)	26JUN2000			Yes								

*Popn=Population (I=Intent-To-Treat, PP=Per Protocol)
**Rel Day=Number of days relative to start of prophylactic medication
Symp = Symptoms, Rd = Reader, Res = Result, Spec = Species, Diff = Difference
Spec Count W = Species Count recorded per 500 White Blood Cells
Spec Count B = Species Count converted to per microlitre of blood
Gam = Gametocyte
+Arb=Arbitrator, +++Pos=Positive, Neg=Negative
#P. f = P.falciparum, P. v = P.vivax, P. o = P.ovale, P. m = P.malariae
Tafenoquine = Tafenoquine 200mg, Mefloquine = Mefloquine 250mg

Listing C.01

Results Of Blood Smears By Visit

Treatment : Placebo

Subject Number	Popn *	Visit	Visit Date	Sample Date	Rel Day**	Symp ?	Rd+	Res +++	Spec #	Spec Count W	Spec Count B	Mean	Diff	Gam Count
030.001.00072	IPP	Week 8 (Fol. Up)	03JUL2000	03JUL2000	56	No	1	Pos	P.f	47.0	573.4	547.0	52.7	
		Week 8 (Fol. Up)	03JUL2000	03JUL2000		No	2	Pos	P.f	42.7	520.7			
		Week 9 (Fol. Up)	10JUL2000	10JUL2000	63	No	1	Neg						
		Week 9 (Fol. Up)	10JUL2000	10JUL2000		No	2	Neg						
		Week 10 (Fol. Up)	17JUL2000	17JUL2000	70	No	1	Pos	P.f	0.4	4.4	3.9	0.9	
		Week 10 (Fol. Up)	17JUL2000	17JUL2000		No	2	Neg						
		Week 10 (Fol. Up)	17JUL2000	17JUL2000		No	Arb	Pos	P.f	0.3	3.4			
		Week 11 (Fol. Up)	24JUL2000	24JUL2000	77	No	1	Neg						
		Week 11 (Fol. Up)	24JUL2000	24JUL2000		No	2	Neg						
030.001.00076	IPP	Screening	11APR2000	11APR2000	-27		1	Pos	P.m	49.2	344.7	297.6	94.2	
		Screening	11APR2000	11APR2000			2	Pos	P.m	35.8	250.5			
		Screening (Unsch.)	26APR2000			Yes								
		Post Clearance	06MAY2000	06MAY2000	-2		1	Neg						
		Post Clearance	06MAY2000	06MAY2000			2	Neg						
		Post Clearance (Unsch.)	06MAY2000			Yes								
		Load Dose 1	08MAY2000			No								
		Load Dose 2	09MAY2000			No								
		Load Dose 3	10MAY2000			No								
		Week 1	15MAY2000	15MAY2000	7	No	1	Neg						
		Week 1	15MAY2000	15MAY2000		No	2	Neg						
		Week 1 (Unsch.)	17MAY2000			Yes								
		Week 2	22MAY2000	22MAY2000	14	No	1	Neg						
		Week 2	22MAY2000	22MAY2000		No	2	Neg						
		Week 3	29MAY2000	29MAY2000	21	No	1	Neg						
		Week 3	29MAY2000	29MAY2000		No	2	Neg						
		Week 4	05JUN2000	05JUN2000	28	No	1	Neg						
		Week 4	05JUN2000	05JUN2000		No	2	Neg						
		Week 5	12JUN2000	12JUN2000	35	No	1	Pos	P.f	20.8	116.5	154.6	-76.2	
		Week 5	12JUN2000	12JUN2000		No	2	Pos	P.f	34.4	192.6			
		Week 6 (Fol. Up)	19JUN2000	19JUN2000	42	No	1	Pos	P.f	0.9	5.4	30.1	-49.4	
		Week 6 (Fol. Up)	19JUN2000	19JUN2000		No	2	Pos	P.f	8.6	54.8			
		Week 7 (Fol. Up)	26JUN2000	26JUN2000	49	Yes	1	Pos	P.f	0.9	5.6	4.8	1.7	
		Week 7 (Fol. Up)	26JUN2000	26JUN2000		Yes	2	Pos	P.f	0.6	3.9			
		Week 7 (Unsch. Fol. Up)	26JUN2000	26JUN2000		Yes	1	Pos	P.f	12.0	76.8	64.0	25.6	
		Week 7 (Unsch. Fol. Up)	26JUN2000	26JUN2000		Yes	2	Pos	P.f	8.0	51.2			
		Week 8 (Fol. Up)	03JUL2000	03JUL2000	56	No	1	Neg						

*Popn=Population (I=Intent-To-Treat, PP=Per Protocol)
**Rel Day=Number of days relative to start of prophylactic medication
Symp = Symptoms, Rd = Reader, Res = Result, Spec = Species, Diff = Difference
Spec Count W = Species Count recorded per 500 White Blood Cells
Spec Count B = Species Count converted to per microlitre of blood
Gam = Gametocyte
+Arb=Arbitrator, +++Pos=Positive, Neg=Negative
#P. f = P.falciparum, P. v = P.vivax, P. o = P.ovale, P. m = P.malariae
Tafenoquine = Tafenoquine 200mg, Mefloquine = Mefloquine 250mg

SB252263 - 030

Listing C.01

Results Of Blood Smears By Visit

Treatment : Placebo

Subject Number	Popn *	Visit	Visit Date	Sample Date	Rel Day**	Symp ?	Rd+	Res +++	Spec #	Spec Count W	Spec Count B	Mean	Diff	Gam Count
030.001.00076	IPP	Week 8 (Fol. Up)	03JUL2000	03JUL2000		No	2	Neg						
		Week 9 (Unsch. Fol. Up)	10JUL2000			No								
		Week 9 (Fol. Up)	10JUL2000	10JUL2000	63		1	Neg						
		Week 9 (Fol. Up)	10JUL2000	10JUL2000			2	Neg						
030.001.00078	IPP	Screening	12APR2000	12APR2000	-26		1	Pos	P.f	2.9	29.4	18.8	21.2	
		Screening	12APR2000	12APR2000			1		P.m	14.4	147.1	100.3	93.6	
		Screening	12APR2000	12APR2000			2	Pos	P.f	0.8	8.2			
		Screening	12APR2000	12APR2000			2		P.m	5.2	53.4			
		Post Clearance	06MAY2000	06MAY2000	-2		1	Neg						
		Post Clearance	06MAY2000	06MAY2000			2	Neg						
		Post Clearance (Unsch.)	06MAY2000			No								
		Load Dose 1	08MAY2000			No								
		Load Dose 2	09MAY2000			No								
		Load Dose 3	10MAY2000			No								
		Week 1 (Unsch.)	11MAY2000			Yes								
		Week 1	15MAY2000	15MAY2000	7	No	1	Neg						
		Week 1	15MAY2000	15MAY2000		No	2	Neg						
		Week 2	22MAY2000	22MAY2000	14	No	1	Neg						
		Week 2	22MAY2000	22MAY2000		No	2	Neg						
		Week 2 (Unsch.)	22MAY2000			No								
		Week 3	29MAY2000	29MAY2000	21	No	1	Neg						
		Week 3	29MAY2000	29MAY2000		No	2	Neg						
		Week 4	05JUN2000	05JUN2000	28	Yes	1	Neg						
		Week 4	05JUN2000	05JUN2000		Yes	2	Neg						
		Week 5	12JUN2000	12JUN2000	35	No	1	Neg						
		Week 5	12JUN2000	12JUN2000		No	2	Neg						
		Week 5 (Unsch.)	12JUN2000			No								
		Week 6	19JUN2000	19JUN2000	42	No	1	Pos	P.f	0.6	7.6	20.6	-26.0	
		Week 6	19JUN2000	19JUN2000			1		P.m	1.2	15.3			
		Week 6	19JUN2000	19JUN2000			2	Pos	P.o	10.8	133.9			
		Week 6	19JUN2000	19JUN2000		No	Arb	Pos	P.f	2.7	33.6			
		Week 7 (Fol. Up)	26JUN2000	26JUN2000	49	Yes	1	Pos	P.f	39.9	494.6	383.4	222.5	
		Week 7 (Fol. Up)	26JUN2000	26JUN2000		Yes	2	Pos	P.f	22.0	272.2			
		Week 7 (Unsch. Fol. Up)	29JUN2000	29JUN2000	52	Yes	1	Pos	P.f	157.1	1885.7	1452.6	866.2	
		Week 7 (Unsch. Fol. Up)	29JUN2000	29JUN2000		Yes	2	Pos	P.f	85.0	1019.5			
		Week 8 (Fol. Up)	03JUL2000	03JUL2000	56	No	1	Pos	P.f	19.8	237.7	366.2	-257.0	

*Popn=Population (I=Intent-To-Treat, PP=Per Protocol)
**Rel Day=Number of days relative to start of prophylactic medication
Symp = Symptoms, Rd = Reader, Res = Result, Spec = Species, Diff = Difference
Spec Count W = Species Count recorded per 500 White Blood Cells
Spec Count B = Species Count converted to per microlitre of blood
Gam = Gametocyte
+Arb=Arbitrator, +++Pos=Positive, Neg=Negative
#P. f = P.falciparum, P. v = P.vivax, P. o = P.ovale, P. m = P.malariae
Tafenoquine = Tafenoquine 200mg, Mefloquine = Mefloquine 250mg

Listing C.01

Results Of Blood Smears By Visit

Treatment : Placebo

Subject Number	Popn *	Visit	Visit Date	Sample Date	Rel Day**	Symp ?	Rd+	Res +++	Spec #	Spec Count W	Spec Count B	Mean	Diff	Gam Count
030.001.00078	IPP	Week 8 (Fol. Up)	03JUL2000	03JUL2000		No	2	Pos	P.f	41.2	494.8			
		Week 9 (Unsch. Fol. Up)	10JUL2000			No								
		Week 9 (Fol. Up)	10JUL2000	10JUL2000	63	No	1	Pos	P.f	350.8	4209.6	3706.4	1006.3	
		Week 9 (Fol. Up)	10JUL2000	10JUL2000		No	2	Pos	P.f	266.9	3203.3			
		Week 10 (Fol. Up)	17JUL2000	17JUL2000	70		1	Pos	P.f	3.5	43.8	34.5	18.5	
		Week 10 (Fol. Up)	17JUL2000	17JUL2000			2	Pos	P.f	2.0	25.3			
030.001.00080	IPP	Screening	12APR2000	12APR2000	-26		1	Pos	P.f	5.9	81.8	65.0	33.7	
		Screening	12APR2000	12APR2000			2	Pos	P.f	3.5	48.2			
		Post Clearance	06MAY2000	06MAY2000	-2		1	Neg						
		Post Clearance	06MAY2000	06MAY2000			2	Neg						
		Load Dose 1	08MAY2000			No								
		Load Dose 2	09MAY2000			No								
		Load Dose 3	10MAY2000			No								
		Week 1 (Unsch.)	14MAY2000			Yes								
		Week 1	15MAY2000	15MAY2000	7	Yes	1	Neg						
		Week 1	15MAY2000	15MAY2000		Yes	2	Neg						
		Week 2	22MAY2000	22MAY2000	14	No	1	Neg						
		Week 2	22MAY2000	22MAY2000		No	2	Neg						
		Week 3	29MAY2000	29MAY2000	21	No	1	Neg						
		Week 3	29MAY2000	29MAY2000		No	2	Neg						
		Week 4	05JUN2000	05JUN2000	28	No	1	Neg						
		Week 4	05JUN2000	05JUN2000		No	2	Neg						
		Week 5	12JUN2000	12JUN2000	35	No	1	Neg						
		Week 5	12JUN2000	12JUN2000		No	2	Neg						
		Week 6	19JUN2000	19JUN2000	42	Yes	1	Neg						
		Week 6	19JUN2000	19JUN2000		Yes	2	Neg						
		Week 7	26JUN2000	26JUN2000	49	Yes	1	Neg						
		Week 7	26JUN2000	26JUN2000		Yes	2	Neg						
		Week 8	03JUL2000	03JUL2000	56	Yes	1	Neg						
		Week 8	03JUL2000	03JUL2000		Yes	2	Neg						
		Week 9	12JUL2000	12JUL2000	65	No	1	Neg						
		Week 9	12JUL2000	12JUL2000		No	2	Neg						
		Week 10	17JUL2000	17JUL2000	70	No	1	Neg						
		Week 10	17JUL2000	17JUL2000		No	2	Neg						
		Week 11	24JUL2000	24JUL2000	77	No	1	Neg						
		Week 11	24JUL2000	24JUL2000		No	2	Neg						

*Popn=Population (I=Intent-To-Treat, PP=Per Protocol)
**Rel Day=Number of days relative to start of prophylactic medication
Symp = Symptoms, Rd = Reader, Res = Result, Spec = Species, Diff = Difference
Spec Count W = Species Count recorded per 500 White Blood Cells
Spec Count B = Species Count converted to per microlitre of blood
Gam = Gametocyte
+Arb=Arbitrator, +++Pos=Positive, Neg=Negative
#P. f = P.falciparum, P. v = P.vivax, P. o = P.ovale, P. m = P.malariae
Tafenoquine = Tafenoquine 200mg, Mefloquine = Mefloquine 250mg

SB252263 - 030

Listing C.01

Results Of Blood Smears By Visit

Treatment : Placebo

Subject Number	Popn *	Visit	Visit Date	Sample Date	Rel Day**	Symp ?	Rd+	Res +++	Spec #	Spec Count W	Spec Count B	Mean	Diff	Gam Count
030.001.00080	IPP	Week 12 (Unsch.)	29JUL2000	29JUL2000	82		1	Neg						
		Week 12 (Unsch.)	29JUL2000	29JUL2000			2	Neg						
		Week 12	31JUL2000	31JUL2000	84	No	1	Neg						
		Week 12	31JUL2000	31JUL2000		No	2	Neg						
		Week 13	07AUG2000	07AUG2000	91	No	1	Neg						
		Week 13	07AUG2000	07AUG2000		No	2	Neg						
		Week 14	14AUG2000	14AUG2000	98		1	Pos	P.o	1.1	17.4	13.3	8.2	
		Week 14	14AUG2000	14AUG2000		No	2	Neg						
		Week 14	14AUG2000	14AUG2000			Arb	Pos	P.o	0.6	9.2			
		Week 15 (Fol. Up)	21AUG2000	21AUG2000	105	No	1	Neg						
		Week 15 (Fol. Up)	21AUG2000	21AUG2000		No	2	Neg						
		Week 16 (Fol. Up)	29AUG2000	29AUG2000	113	No	1	Pos	P.f	8.2	91.7	77.2	29.1	
		Week 16 (Fol. Up)	29AUG2000	29AUG2000		No	2	Neg						
		Week 16 (Fol. Up)	29AUG2000	29AUG2000		No	Arb	Pos	P.f	5.6	62.6			
		Week 16 (Unsch. Fol. Up)	31AUG2000			No								
		Week 17 (Fol. Up)	04SEP2000	04SEP2000	119	No	1	Pos	P.f	166.2	1861.4	1876.0	-29.1	
		Week 17 (Fol. Up)	04SEP2000	04SEP2000		No	2	Pos	P.f	168.8	1890.6			
		Week 17 (Unsch. Fol. Up)	06SEP2000			No								
		Week 18 (Fol. Up)	11SEP2000	11SEP2000	126		1	Neg						
		Week 18 (Fol. Up)	11SEP2000	11SEP2000			2	Neg						
030.001.00081	IPP	Screening	11APR2000	11APR2000	-27		1	Pos	P.f	9.2	64.4	52.2	24.4	
		Screening	11APR2000	11APR2000			2	Pos	P.f	5.7	40.0			
		Screening (Unsch.)	20APR2000	20APR2000	-18	Yes		Pos						
		Post Clearance	06MAY2000	06MAY2000	-2		1	Neg						
		Post Clearance	06MAY2000	06MAY2000			2	Neg						
		Load Dose 1	08MAY2000			No								
		Load Dose 2	09MAY2000			No								
		Load Dose 3	10MAY2000			No								
		Week 1	16MAY2000	16MAY2000	8	No	1	Neg						
		Week 1	16MAY2000	16MAY2000		No	2	Neg						
		Week 1 (Unsch.)	17MAY2000			Yes								
		Week 2	22MAY2000	22MAY2000	14	No	1	Neg						
		Week 2	22MAY2000	22MAY2000		No	2	Neg						
		Week 3	29MAY2000	29MAY2000	21	No	1	Neg						
		Week 3	29MAY2000	29MAY2000		No	2	Neg						
		Week 4	05JUN2000	05JUN2000	28	No	1	Neg						

*Popn=Population (I=Intent-To-Treat, PP=Per Protocol)
**Rel Day=Number of days relative to start of prophylactic medication
Symp = Symptoms, Rd = Reader, Res = Result, Spec = Species, Diff = Difference
Spec Count W = Species Count recorded per 500 White Blood Cells
Spec Count B = Species Count converted to per microlitre of blood
Gam = Gametocyte
+Arb=Arbitrator, +++Pos=Positive, Neg=Negative
#P. f = P.falciparum, P. v = P.vivax, P. o = P.ovale, P. m = P.malariae
Tafenoquine = Tafenoquine 200mg, Mefloquine = Mefloquine 250mg

Listing C.01

Results Of Blood Smears By Visit

Treatment : Placebo

Subject Number	Popn *	Visit	Visit Date	Sample Date	Rel Day**	Symp ?	Rd+	Res +++	Spec #	Spec Count W	Spec Count B	Mean	Diff	Gam Count
030.001.00081	IPP	Week 4	05JUN2000	05JUN2000		No	2	Neg						
		Week 5	13JUN2000	13JUN2000	36	No	1	Neg						
		Week 5	13JUN2000	13JUN2000		No	2	Pos	P.f	24.7	227.1	165.8	122.6	
		Week 5	13JUN2000	13JUN2000		No	Arb	Pos	P.f	11.4	104.5			
		Week 6 (Fol. Up)	19JUN2000	19JUN2000	42	No	1	Pos	P.f	5.0	45.5	49.6	-8.2	
		Week 6 (Fol. Up)	19JUN2000	19JUN2000		No	2	Pos	P.f	5.8	53.7			
		Week 7 (Fol. Up)	26JUN2000	26JUN2000	49	Yes	1	Pos	P.f	21.0	192.8	164.5	56.6	
		Week 7 (Fol. Up)	26JUN2000	26JUN2000		Yes	2	Pos	P.f	14.8	136.3			
		Week 7 (Unsch. Fol. Up)	26JUN2000			Yes								
		Week 8 (Fol. Up)	03JUL2000	03JUL2000	56	No	1	Neg						
		Week 8 (Fol. Up)	03JUL2000	03JUL2000		No	2	Neg						
		Week 9 (Fol. Up)	10JUL2000	10JUL2000	63	No	1	Neg						
		Week 9 (Fol. Up)	10JUL2000	10JUL2000		No	2	Neg						
030.001.00087	I	Screening	14APR2000	14APR2000	-24		1	Pos	P.f	6.0	87.6	76.1	23.1	
		Screening	14APR2000	14APR2000			1		P.o	1.2	17.7	15.3	4.8	
		Screening	14APR2000	14APR2000			2	Pos	P.o	0.9	12.8			
		Screening	14APR2000	14APR2000			Arb	Pos	P.f	4.4	64.5			
		Screening	14APR2000	14APR2000			Arb		P.o	0.9	12.8			
		Screening (Unsch.)	23APR2000			Yes								
		Post Clearance	06MAY2000	06MAY2000	-2		1	Neg						
		Post Clearance	06MAY2000	06MAY2000			2	Neg						
		Post Clearance (Unsch.)	06MAY2000			Yes								
		Load Dose 1	08MAY2000			No								
		Load Dose 2	09MAY2000			Yes								
		Load Dose 3	10MAY2000			No								
		Week 1	15MAY2000	15MAY2000	7	Yes	1	Neg						
		Week 1	15MAY2000	15MAY2000		Yes	2	Neg						
		Week 2 (Unsch. Fol. Up)	19MAY2000	19MAY2000	11	Yes	1	Neg						
		Week 2 (Unsch. Fol. Up)	19MAY2000	19MAY2000		Yes	2	Neg						
		Week 2 (Unsch. Fol. Up)	20MAY2000			Yes								
		Week 2 (Unsch. Fol. Up)	21MAY2000	21MAY2000	13	Yes	1	Neg						
		Week 2 (Unsch. Fol. Up)	21MAY2000	21MAY2000		Yes	2	Neg						
		Week 2 (Unsch. Fol. Up)	22MAY2000			Yes								
		Week 2 (Fol. Up)	24MAY2000	24MAY2000	16	Yes	1	Pos	P.f	3.0	31.2	26.0	10.4	
		Week 2 (Fol. Up)	24MAY2000	24MAY2000		Yes	2	Pos	P.f	2.0	20.8			
		Week 3 (Unsch. Fol. Up)	28MAY2000			No								

*Popn=Population (I=Intent-To-Treat, PP=Per Protocol)
**Rel Day=Number of days relative to start of prophylactic medication
Symp = Symptoms, Rd = Reader, Res = Result, Spec = Species, Diff = Difference
Spec Count W = Species Count recorded per 500 White Blood Cells
Spec Count B = Species Count converted to per microlitre of blood
Gam = Gametocyte
+Arb=Arbitrator, +++Pos=Positive, Neg=Negative
#P. f = P.falciparum, P. v = P.vivax, P. o = P.ovale, P. m = P.malariae
Tafenoquine = Tafenoquine 200mg, Mefloquine = Mefloquine 250mg

SB252263 - 030

Listing C.01

Results Of Blood Smears By Visit

Treatment : Placebo

Subject Number	Popn *	Visit	Visit Date	Sample Date	Rel Day**	Symp ?	Rd+	Res +++	Spec #	Spec Count W	Spec Count B	Mean	Diff	Gam Count
030.001.00087	I	Week 5 (Fol. Up)	11JUN2000	11JUN2000	34	No	1	Neg						
		Week 5 (Fol. Up)	11JUN2000	11JUN2000		No	2	Neg						
		Week 5 (Fol. Up)	12JUN2000	12JUN2000	35	Yes	1	Neg						
		Week 5 (Fol. Up)	12JUN2000	12JUN2000		Yes	2	Neg						
030.001.00088	IPP	Screening	14APR2000	14APR2000	-24		1	Neg						
		Screening	14APR2000	14APR2000			2	Neg						
		Post Clearance	06MAY2000	06MAY2000	-2		1	Neg						
		Post Clearance	06MAY2000	06MAY2000			2	Neg						
		Load Dose 1	08MAY2000			No								
		Load Dose 2	09MAY2000			No								
		Load Dose 3	10MAY2000			No								
		Week 1	15MAY2000	15MAY2000	7	No	1	Neg						
		Week 1	15MAY2000	15MAY2000		No	2	Neg						
		Week 2	22MAY2000	22MAY2000	14	No	1	Neg						
		Week 2	22MAY2000	22MAY2000		No	2	Neg						
		Week 3	29MAY2000	29MAY2000	21	No	1	Neg						
		Week 3	29MAY2000	29MAY2000		No	2	Neg						
		Week 4	06JUN2000	06JUN2000	29	No	1	Neg						
		Week 4	06JUN2000	06JUN2000		No	2	Neg						
		Week 5	12JUN2000	12JUN2000	35	No	1	Pos	P.f	8.6	88.1	133.4	-90.6	
		Week 5	12JUN2000	12JUN2000			1		P.o	1.2	12.5	20.0	-14.9	
		Week 5	12JUN2000	12JUN2000		No	2	Pos	P.f	17.5	178.7			
		Week 5	12JUN2000	12JUN2000			2		P.o	2.7	27.4			
		Week 6 (Fol. Up)	19JUN2000	19JUN2000	42	No	1	Pos	P.f	34.7	402.3	615.4	-426.2	
		Week 6 (Fol. Up)	19JUN2000	19JUN2000		No	2	Pos	P.f	71.4	828.5			
		Week 7 (Fol. Up)	26JUN2000	26JUN2000	49	No	1	Pos	P.f	240.2	2786.6	4151.8	-2730.4	
		Week 7 (Fol. Up)	26JUN2000	26JUN2000		No	2	Pos	P.f	475.6	5517.0			
		Week 8 (Fol. Up)	03JUL2000	03JUL2000	56	No	1	Pos	P.f	21.9	253.9	184.2	139.5	
		Week 8 (Fol. Up)	03JUL2000	03JUL2000		No	2	Pos	P.f	9.9	114.4			
		Week 9 (Fol. Up)	11JUL2000	11JUL2000	64		1	Pos	P.f	801.2	9293.3	7381.9	3822.9	
		Week 9 (Fol. Up)	11JUL2000	11JUL2000			2	Pos	P.f	471.6	5470.4			
030.001.00090	IPP	Screening	12APR2000	12APR2000	-26		1	Pos	P.f	22.9	238.2	245.7	-15.1	
		Screening	12APR2000	12APR2000			2	Pos	P.f	24.4	253.2			
		Screening (Unsch.)	18APR2000			Yes								
		Screening (Unsch.)	29APR2000			Yes								

*Popn=Population (I=Intent-To-Treat, PP=Per Protocol)
**Rel Day=Number of days relative to start of prophylactic medication
Symp = Symptoms, Rd = Reader, Res = Result, Spec = Species, Diff = Difference
Spec Count W = Species Count recorded per 500 White Blood Cells
Spec Count B = Species Count converted to per microlitre of blood
Gam = Gametocyte
+Arb=Arbitrator, +++Pos=Positive, Neg=Negative
#P. f = P.falciparum, P. v = P.vivax, P. o = P.ovale, P. m = P.malariae
Tafenoquine = Tafenoquine 200mg, Mefloquine = Mefloquine 250mg

Listing C.01

Results Of Blood Smears By Visit

Treatment : Placebo

Subject Number	Popn *	Visit	Visit Date	Sample Date	Rel Day**	Symp ?	Rd+	Res +++	Spec #	Spec Count W	Spec Count B	Mean	Diff	Gam Count
030.001.00090	IPP	Post Clearance	06MAY2000	06MAY2000	-2		1	Neg						
		Post Clearance	06MAY2000	06MAY2000			2	Neg						
		Load Dose 1	08MAY2000			Yes								
		Load Dose 2	09MAY2000			No								
		Load Dose 3	10MAY2000			No								
		Week 1	15MAY2000	15MAY2000	7	No	1	Neg						
		Week 1	15MAY2000	15MAY2000		No	2	Neg						
		Week 2	22MAY2000	22MAY2000	14	Yes	1	Neg						
		Week 2	22MAY2000	22MAY2000		Yes	2	Neg						
		Week 3	29MAY2000	29MAY2000	21	Yes	1	Neg						
		Week 3	29MAY2000	29MAY2000		Yes	2	Neg						
		Week 3 (Unsch.)	30MAY2000			Yes								
		Week 4	04JUN2000	04JUN2000	27	Yes	1	Neg						
		Week 4	04JUN2000	04JUN2000		Yes	2	Neg						
		Week 5	12JUN2000	12JUN2000	35	No	1	Neg						
		Week 5	12JUN2000	12JUN2000		No	2	Neg						
		Week 5 (Unsch.)	14JUN2000			Yes								
		Week 6	19JUN2000	19JUN2000	42	Yes	1	Neg						
		Week 6	19JUN2000	19JUN2000		Yes	2	Neg						
		Week 6 (Unsch.)	21JUN2000			Yes								
		Week 7	26JUN2000	26JUN2000	49	No	1	Neg						
		Week 7	26JUN2000	26JUN2000		No	2	Neg						
		Week 8	03JUL2000	03JUL2000	56	No	1	Neg						
		Week 8	03JUL2000	03JUL2000		No	2	Neg						
		Week 9	10JUL2000	10JUL2000	63	No	1	Pos	P.f	1.1	9.0			
		Week 9	10JUL2000	10JUL2000		No	2	Neg						
		Week 9	10JUL2000	10JUL2000		No	Arb	Neg						
		Week 10	17JUL2000	17JUL2000	70	No	1	Neg						
		Week 10	17JUL2000	17JUL2000		No	2	Neg						
		Week 11	25JUL2000	25JUL2000	78	No	1	Pos	P.f	4.8	49.5	60.8	-22.7	
		Week 11	25JUL2000	25JUL2000		No	2	Pos	P.f	6.9	72.2			
		Week 12 (Fol. Up)	31JUL2000	31JUL2000	84	No	1	Pos	P.f	6.0	62.1	47.3	29.6	
		Week 12 (Fol. Up)	31JUL2000	31JUL2000		No	2	Pos	P.f	3.1	32.4			
		Week 14 (Fol. Up)	15AUG2000	15AUG2000	99	No	1	Neg						
		Week 14 (Fol. Up)	15AUG2000	15AUG2000		No	2	Neg						
		Week 15 (Fol. Up)	21AUG2000	21AUG2000	105	No	1	Neg						
		Week 15 (Fol. Up)	21AUG2000	21AUG2000		No	2	Neg						

*Popn=Population (I=Intent-To-Treat, PP=Per Protocol)
**Rel Day=Number of days relative to start of prophylactic medication
Symp = Symptoms, Rd = Reader, Res = Result, Spec = Species, Diff = Difference
Spec Count W = Species Count recorded per 500 White Blood Cells
Spec Count B = Species Count converted to per microlitre of blood
Gam = Gametocyte
+Arb=Arbitrator, +++Pos=Positive, Neg=Negative
#P. f = P.falciparum, P. v = P.vivax, P. o = P.ovale, P. m = P.malariae
Tafenoquine = Tafenoquine 200mg, Mefloquine = Mefloquine 250mg

SB252263 - 030

Listing C.01

Results Of Blood Smears By Visit

Treatment : Placebo

Subject Number	Popn *	Visit	Visit Date	Sample Date	Rel Day**	Symp ?	Rd+	Res +++	Spec #	Spec Count W	Spec Count B	Mean	Diff	Gam Count
030.001.00092	IPP	Screening	12APR2000	12APR2000	-26		1	Neg						
		Screening	12APR2000	12APR2000			2	Neg						
		Screening (Unsch.)	18APR2000	18APR2000	-20	Yes	1	Neg						
		Screening (Unsch.)	18APR2000	18APR2000		Yes	2	Neg						
		Screening (Unsch.)	03MAY2000			Yes								
		Post Clearance	06MAY2000	06MAY2000	-2		1	Neg						
		Post Clearance	06MAY2000	06MAY2000			2	Neg						
		Load Dose 1	08MAY2000			No								
		Load Dose 2	09MAY2000			No								
		Load Dose 3	10MAY2000			No								
		Week 1	15MAY2000	15MAY2000	7	Yes	1	Neg						
		Week 1	15MAY2000	15MAY2000		Yes	2	Neg						
		Week 2	22MAY2000	22MAY2000	14	No	1	Neg						
		Week 2	22MAY2000	22MAY2000		No	2	Neg						
		Week 3	29MAY2000	29MAY2000	21	No	1	Neg						
		Week 3	29MAY2000	29MAY2000		No	2	Neg						
		Week 4	05JUN2000	05JUN2000	28	No	1	Neg						
		Week 4	05JUN2000	05JUN2000		No	2	Neg						
		Week 4 (Unsch.)	07JUN2000			Yes								
		Week 5	12JUN2000	12JUN2000	35	No	1	Neg						
		Week 5	12JUN2000	12JUN2000		No	2	Neg						
		Week 6	19JUN2000	19JUN2000	42	No	1	Neg						
		Week 6	19JUN2000	19JUN2000		No	2	Neg						
		Week 6 (Unsch.)	22JUN2000			Yes								
		Week 7	26JUN2000	26JUN2000	49	No	1	Neg						
		Week 7	26JUN2000	26JUN2000		No	2	Neg						
		Week 8	03JUL2000	03JUL2000	56	Yes	1	Pos	P.f	7.3	76.9	57.8	38.2	
		Week 8	03JUL2000	03JUL2000		Yes	2	Pos	P.f	3.7	38.7			
		Week 9 (Fol. Up)	10JUL2000	10JUL2000	63		1	Pos	P.f	74.7	791.6	976.9	-370.6	
		Week 9 (Fol. Up)	10JUL2000	10JUL2000			2	Pos	P.f	109.6	1162.2			
		Week 9 (Fol. Up)	10JUL2000	10JUL2000		No		Pos						
		Week 9 (Unsch. Fol. Up)	11JUL2000	11JUL2000	64	Yes	1	Pos	P.f	607.0	9712.6	8575.3	2274.7	
		Week 9 (Unsch. Fol. Up)	11JUL2000	11JUL2000		Yes	2	Pos	P.f	464.9	7437.9			
		Week 10 (Fol. Up)	18JUL2000	18JUL2000	71		1	Neg						
		Week 10 (Fol. Up)	18JUL2000	18JUL2000			2	Neg						
		Week 10 (Fol. Up)	18JUL2000	18JUL2000		No		Neg						
		Week 11 (Fol. Up)	24JUL2000	24JUL2000	77		1	Neg						

*Popn=Population (I=Intent-To-Treat, PP=Per Protocol)
**Rel Day=Number of days relative to start of prophylactic medication
Symp = Symptoms, Rd = Reader, Res = Result, Spec = Species, Diff = Difference
Spec Count W = Species Count recorded per 500 White Blood Cells
Spec Count B = Species Count converted to per microlitre of blood
Gam = Gametocyte
+Arb=Arbitrator, +++Pos=Positive, Neg=Negative
#P. f = P.falciparum, P. v = P.vivax, P. o = P.ovale, P. m = P.malariae
Tafenoquine = Tafenoquine 200mg, Mefloquine = Mefloquine 250mg

SB252263 - 030

Listing C.01

Results Of Blood Smears By Visit

Treatment : Placebo

Subject Number	Popn *	Visit	Visit Date	Sample Date	Rel Day**	Symp ?	Rd+	Res +++	Spec #	Spec Count W	Spec Count B	Mean	Diff	Gam Count
030.001.00092	IPP	Week 11 (Fol. Up)	24JUL2000	24JUL2000			2	Neg						
		Week 11 (Fol. Up)	24JUL2000	24JUL2000		No		Neg						
		Week 12 (Fol. Up)	01AUG2000	01AUG2000	85		1	Neg						
		Week 12 (Fol. Up)	01AUG2000	01AUG2000			2	Neg						
		Week 12 (Fol. Up)	02AUG2000			No								
030.001.00101	IPP	Screening	14APR2000	14APR2000	-24		1	Neg						
		Screening	14APR2000	14APR2000			2	Neg						
		Screening (Unsch.)	25APR2000	25APR2000	-13	Yes	1	Neg						
		Screening (Unsch.)	25APR2000	25APR2000		Yes	2	Neg						
		Screening (Unsch.)	28APR2000	28APR2000	-10	Yes	1	Neg						
		Screening (Unsch.)	28APR2000	28APR2000		Yes	2	Neg						
		Post Clearance	06MAY2000	06MAY2000	-2		1	Neg						
		Post Clearance	06MAY2000	06MAY2000			2	Neg						
		Load Dose 1	08MAY2000			No								
		Load Dose 2	09MAY2000			No								
		Load Dose 3	10MAY2000			No								
		Week 1	15MAY2000	15MAY2000	7	Yes	1	Neg						
		Week 1	15MAY2000	15MAY2000		Yes	2	Neg						
		Week 2	22MAY2000	22MAY2000	14	No	1	Neg						
		Week 2	22MAY2000	22MAY2000		No	2	Neg						
		Week 2 (Unsch.)	24MAY2000	24MAY2000	16	Yes	1	Neg						
		Week 2 (Unsch.)	24MAY2000	24MAY2000		Yes	2	Neg						
		Week 2 (Unsch.)	25MAY2000	25MAY2000	17	Yes	1	Neg						
		Week 2 (Unsch.)	25MAY2000	25MAY2000		Yes	2	Neg						
		Week 3 (Unsch.)	26MAY2000			Yes								
		Week 3 (Unsch.)	28MAY2000			No								
		Week 3	29MAY2000	29MAY2000	21	No	1	Neg						
		Week 3	29MAY2000	29MAY2000		No	2	Neg						
		Week 4	05JUN2000	05JUN2000	28	Yes	1	Neg						
		Week 4	05JUN2000	05JUN2000		Yes	2	Neg						
		Week 5	12JUN2000	12JUN2000	35	No	1	Neg						
		Week 5	12JUN2000	12JUN2000		No	2	Neg						
		Week 6	19JUN2000	19JUN2000	42	Yes	1	Neg						
		Week 6	19JUN2000	19JUN2000		Yes	2	Neg						
		Week 7	26JUN2000	26JUN2000	49	No	1	Neg						
		Week 7	26JUN2000	26JUN2000		No	2	Neg						

*Popn=Population (I=Intent-To-Treat, PP=Per Protocol)
**Rel Day=Number of days relative to start of prophylactic medication
Symp = Symptoms, Rd = Reader, Res = Result, Spec = Species, Diff = Difference
Spec Count W = Species Count recorded per 500 White Blood Cells
Spec Count B = Species Count converted to per microlitre of blood
Gam = Gametocyte
+Arb=Arbitrator, +++Pos=Positive, Neg=Negative
#P. f = P.falciparum, P. v = P.vivax, P. o = P.ovale, P. m = P.malariae
Tafenoquine = Tafenoquine 200mg, Mefloquine = Mefloquine 250mg

SB252263 - 030

Listing C.01

Results Of Blood Smears By Visit

Treatment : Placebo

Subject Number	Popn *	Visit	Visit Date	Sample Date	Rel Day**	Symp ?	Rd+	Res +++	Spec #	Spec Count W	Spec Count B	Mean	Diff	Gam Count
030.001.00101	IPP	Week 8	03JUL2000	03JUL2000	56	Yes	1	Pos	P.f	3.4	24.1	19.8	8.5	
		Week 8	03JUL2000	03JUL2000		Yes	2	Pos	P.f	2.2	15.5			
		Week 9 (Fol. Up)	10JUL2000	10JUL2000	63	Yes	1	Pos	P.f	0.8	5.7	6.2	-1.1	
		Week 9 (Fol. Up)	10JUL2000	10JUL2000		Yes	2	Neg						
		Week 9 (Fol. Up)	10JUL2000	10JUL2000		Yes	Arb	Pos	P.f	1.0	6.7			
		Week 9 (Unsch. Fol. Up)	11JUL2000			Yes								
		Week 10 (Unsch. Fol. Up)	14JUL2000			No								
		Week 10 (Fol. Up)	17JUL2000	17JUL2000	70	No	1	Neg						
		Week 10 (Fol. Up)	17JUL2000	17JUL2000		No	2	Neg						
		Week 11 (Fol. Up)	24JUL2000	24JUL2000	77	Yes	1	Neg						
		Week 11 (Fol. Up)	24JUL2000	24JUL2000		Yes	2	Neg						
		Week 12 (Fol. Up)	31JUL2000	31JUL2000	84		1	Neg						
		Week 12 (Fol. Up)	31JUL2000	31JUL2000			2	Neg						
		Week 12 (Fol. Up)	01AUG2000			Yes								
030.001.00103	IPP	Screening	11APR2000	11APR2000	-27		1	Neg						
		Screening	11APR2000	11APR2000			2	Neg						
		Screening (Unsch.)	20APR2000	20APR2000	-18		1	Neg						
		Screening (Unsch.)	20APR2000	20APR2000			2	Neg						
		Screening (Unsch.)	22APR2000	22APR2000	-16		1	Neg						
		Screening (Unsch.)	22APR2000	22APR2000			2	Neg						
		Post Clearance	07MAY2000	07MAY2000	-1		1	Neg						
		Post Clearance	07MAY2000	07MAY2000			2	Neg						
		Load Dose 1	08MAY2000			No								
		Load Dose 2	09MAY2000			No								
		Load Dose 3	10MAY2000			No								
		Week 1	15MAY2000	15MAY2000	7	No	1	Neg						
		Week 1	15MAY2000	15MAY2000		No	2	Neg						
		Week 2 (Unsch.)	19MAY2000			Yes								
		Week 2 (Unsch.)	20MAY2000			Yes								
		Week 2	22MAY2000	22MAY2000	14	No	1	Neg						
		Week 2	22MAY2000	22MAY2000		No	2	Neg						
		Week 3	29MAY2000	29MAY2000	21	No	1	Neg						
		Week 3	29MAY2000	29MAY2000		No	2	Neg						
		Week 4	06JUN2000	06JUN2000	29	Yes	1	Neg						
		Week 4	06JUN2000	06JUN2000		Yes	2	Neg						
		Week 5	12JUN2000	12JUN2000	35	No	1	Neg						

*Popn=Population (I=Intent-To-Treat, PP=Per Protocol)
**Rel Day=Number of days relative to start of prophylactic medication
Symp = Symptoms, Rd = Reader, Res = Result, Spec = Species, Diff = Difference
Spec Count W = Species Count recorded per 500 White Blood Cells
Spec Count B = Species Count converted to per microlitre of blood
Gam = Gametocyte
+Arb=Arbitrator, +++Pos=Positive, Neg=Negative
#P. f = P.falciparum, P. v = P.vivax, P. o = P.ovale, P. m = P.malariae
Tafenoquine = Tafenoquine 200mg, Mefloquine = Mefloquine 250mg

SB252263 - 030

Listing C.01

Results Of Blood Smears By Visit

Treatment : Placebo

Subject Number	Popn *	Visit	Visit Date	Sample Date	Rel Day**	Symp ?	Rd+	Res +++	Spec #	Spec Count W	Spec Count B	Mean	Diff	Gam Count
030.001.00103	IPP	Week 5	12JUN2000	12JUN2000		No	2	Neg						
		Week 6	19JUN2000	19JUN2000	42	No	1	Pos	P.f	11.5	98.7	82.8	31.8	
		Week 6	19JUN2000	19JUN2000		No	2	Pos	P.f	7.8	66.9			
		Week 7 (Unsch. Fol. Up)	23JUN2000	23JUN2000	46	Yes	1	Pos	P.f	15.7	135.1	155.1	-40.1	
		Week 7 (Unsch. Fol. Up)	23JUN2000	23JUN2000		Yes	2	Pos	P.f	20.4	175.2			
		Week 7 (Fol. Up)	26JUN2000	26JUN2000	49	No	1	Neg						
		Week 7 (Fol. Up)	26JUN2000	26JUN2000		No	2	Neg						
		Week 8 (Fol. Up)	06JUL2000	06JUL2000	59	No	1	Pos	P.f	0.5	3.8	3.3	1.0	
		Week 8 (Fol. Up)	06JUL2000	06JUL2000		No	2	Pos	P.f	0.4	2.8			
		Week 9 (Fol. Up)	11JUL2000	11JUL2000	64	Yes	1	Neg						
		Week 9 (Fol. Up)	11JUL2000	11JUL2000		Yes	2	Neg						
		Week 10 (Fol. Up)	17JUL2000	17JUL2000	70	No	1	Neg						
		Week 10 (Fol. Up)	17JUL2000	17JUL2000		No	2	Neg						
030.001.00106	IPP	Screening	10APR2000	10APR2000	-28		1	Pos	P.f	1.3	15.5	25.6	-20.3	
		Screening	10APR2000	10APR2000			2	Pos	P.f	3.0	35.8			
		Post Clearance	07MAY2000	07MAY2000	-1		1	Neg						
		Post Clearance	07MAY2000	07MAY2000			2	Neg						
		Load Dose 1	08MAY2000			No								
		Load Dose 2	09MAY2000			No								
		Load Dose 3	10MAY2000			No								
		Week 1	15MAY2000	15MAY2000	7	No	1	Neg						
		Week 1	15MAY2000	15MAY2000		No	2	Neg						
		Week 2	24MAY2000	24MAY2000	16	Yes	1	Neg						
		Week 2	24MAY2000	24MAY2000		Yes	2	Neg						
		Week 3	28MAY2000	28MAY2000	20	No	1	Neg						
		Week 3	28MAY2000	28MAY2000		No	2	Neg						
		Week 3 (Unsch.)	28MAY2000			No								
		Week 4	07JUN2000	07JUN2000	30	No	1	Neg						
		Week 4	07JUN2000	07JUN2000		No	2	Neg						
		Week 5 (Unsch.)	10JUN2000			Yes								
		Week 5	13JUN2000	13JUN2000	36	No	1	Neg						
		Week 5	13JUN2000	13JUN2000		No	2	Neg						
		Week 6	19JUN2000	19JUN2000	42	No	1	Neg						
		Week 6	19JUN2000	19JUN2000		No	2	Neg						
		Week 8	02JUL2000	02JUL2000	55	No	1	Pos	P.f	53.0	763.3	695.7	135.2	
		Week 8	02JUL2000	02JUL2000		No	2	Pos	P.f	43.6	628.1			

*Popn=Population (I=Intent-To-Treat, PP=Per Protocol)
**Rel Day=Number of days relative to start of prophylactic medication
Symp = Symptoms, Rd = Reader, Res = Result, Spec = Species, Diff = Difference
Spec Count W = Species Count recorded per 500 White Blood Cells
Spec Count B = Species Count converted to per microlitre of blood
Gam = Gametocyte
+Arb=Arbitrator, +++Pos=Positive, Neg=Negative
#P. f = P.falciparum, P. v = P.vivax, P. o = P.ovale, P. m = P.malariae
Tafenoquine = Tafenoquine 200mg, Mefloquine = Mefloquine 250mg

SB252263 - 030

Listing C.01

Results Of Blood Smears By Visit

Treatment : Placebo

Subject Number	Popn *	Visit	Visit Date	Sample Date	Rel Day**	Symp ?	Rd+	Res +++	Spec #	Spec Count W	Spec Count B	Mean	Diff	Gam Count
030.001.00106	IPP	Week 8 (Fol. Up)	04JUL2000	04JUL2000	57	No	1	Neg						
		Week 8 (Fol. Up)	04JUL2000	04JUL2000		No	2	Pos	P.f	17.7	254.7	183.6	142.3	
		Week 8 (Fol. Up)	04JUL2000	04JUL2000		No	Arb	Pos	P.f	7.8	112.5			
		Week 8 (Unsch. Fol. Up)	05JUL2000			No								
		Week 9 (Fol. Up)	13JUL2000	13JUL2000	66	No	1	Neg						
		Week 9 (Fol. Up)	13JUL2000	13JUL2000		No	2	Neg						
		Week 10 (Fol. Up)	17JUL2000	17JUL2000	70	No	1	Neg						
		Week 10 (Fol. Up)	17JUL2000	17JUL2000		No	2	Neg						
		Week 11 (Fol. Up)	24JUL2000	24JUL2000	77		1	Neg						
		Week 11 (Fol. Up)	24JUL2000	24JUL2000			2	Neg						
		Week 11 (Unsch. Fol. Up)	25JUL2000			No								
		Week 12 (Fol. Up)	31JUL2000	31JUL2000	84		1	Pos	P.f	238.5	3433.8	3363.5	140.7	
		Week 12 (Fol. Up)	31JUL2000	31JUL2000			2	Pos	P.f	228.7	3293.1			
030.001.00108	IPP	Screening	14APR2000	14APR2000	-24		1	Pos	P.f	19.4	236.8	219.8	33.9	
		Screening	14APR2000	14APR2000			2	Pos	P.f	16.6	202.9			
		Post Clearance	06MAY2000	06MAY2000	-2		1	Neg						
		Post Clearance	06MAY2000	06MAY2000			2	Neg						
		Load Dose 1	08MAY2000			No								
		Load Dose 2	09MAY2000			No								
		Load Dose 3	10MAY2000			Yes								
		Week 1 (Unsch.)	11MAY2000			No								
		Week 1	15MAY2000	15MAY2000	7	Yes	1	Pos	P.f	0.3	4.1	4.8	-1.3	
		Week 1	15MAY2000	15MAY2000		Yes	2	Pos	P.f	0.4	5.4			
		Week 1 (Unsch.)	18MAY2000			No								
		Week 2 (Unsch.)	19MAY2000			No								
		Week 2	22MAY2000	22MAY2000	14	No	1	Pos	P.f	3.0	39.6	25.9	27.5	
		Week 2	22MAY2000	22MAY2000		No	2	Neg						
		Week 2	22MAY2000	22MAY2000		No	Arb	Pos	P.f	0.9	12.1			
		Week 3 (Fol. Up)	29MAY2000	29MAY2000	21	Yes	1	Neg						
		Week 3 (Fol. Up)	29MAY2000	29MAY2000		Yes	2	Neg						
		Week 4 (Fol. Up)	07JUN2000	07JUN2000	30	No	1	Neg						
		Week 4 (Fol. Up)	07JUN2000	07JUN2000		No	2	Neg						
		Week 5 (Fol. Up)	12JUN2000	12JUN2000	35	Yes	1	Neg						
		Week 5 (Fol. Up)	12JUN2000	12JUN2000		Yes	2	Neg						
		Week 6 (Fol. Up)	19JUN2000	19JUN2000	42	Yes	1	Neg						
		Week 6 (Fol. Up)	19JUN2000	19JUN2000		Yes	2	Pos	P.f	6.8	90.3	90.3	0.0	

*Popn=Population (I=Intent-To-Treat, PP=Per Protocol)
**Rel Day=Number of days relative to start of prophylactic medication
Symp = Symptoms, Rd = Reader, Res = Result, Spec = Species, Diff = Difference
Spec Count W = Species Count recorded per 500 White Blood Cells
Spec Count B = Species Count converted to per microlitre of blood
Gam = Gametocyte
+Arb=Arbitrator, +++Pos=Positive, Neg=Negative
#P. f = P.falciparum, P. v = P.vivax, P. o = P.ovale, P. m = P.malariae
Tafenoquine = Tafenoquine 200mg, Mefloquine = Mefloquine 250mg

SB252263 - 030

Listing C.01

Results Of Blood Smears By Visit

Treatment : Placebo

Subject Number	Popn *	Visit	Visit Date	Sample Date	Rel Day**	Symp ?	Rd+	Res +++	Spec #	Spec Count W	Spec Count B	Mean	Diff	Gam Count
030.001.00108	IPP	Week 6 (Fol. Up)	19JUN2000	19JUN2000		Yes	Arb	Pos	P.f	6.8	90.3			
030.001.00114	IPP	Screening	14APR2000	14APR2000	-24		1	Neg						
		Screening	14APR2000	14APR2000			2	Neg						
		Screening (Unsch.)	28APR2000			Yes								
		Post Clearance	06MAY2000	06MAY2000	-2		1	Neg						
		Post Clearance	06MAY2000	06MAY2000			2	Neg						
		Load Dose 1	08MAY2000			No								
		Load Dose 2	09MAY2000			Yes								
		Load Dose 3	10MAY2000			No								
		Week 1	15MAY2000	15MAY2000	7	Yes	1	Neg						
		Week 1	15MAY2000	15MAY2000		Yes	2	Neg						
		Week 2	22MAY2000	22MAY2000	14	Yes	1	Neg						
		Week 2	22MAY2000	22MAY2000		Yes	2	Neg						
		Week 3	29MAY2000	29MAY2000	21	No	1	Neg						
		Week 3	29MAY2000	29MAY2000		No	2	Neg						
		Week 4	05JUN2000	05JUN2000	28	No	1	Neg						
		Week 4	05JUN2000	05JUN2000		No	2	Neg						
		Week 5	12JUN2000	12JUN2000	35	No	1	Neg						
		Week 5	12JUN2000	12JUN2000		No	2	Neg						
		Week 6	19JUN2000	19JUN2000	42	No	1	Neg						
		Week 6	19JUN2000	19JUN2000		No	2	Neg						
		Week 6 (Unsch.)	19JUN2000			No								
		Week 6 (Unsch.)	20JUN2000			No								
		Week 7	26JUN2000	26JUN2000	49	No	1	Neg						
		Week 7	26JUN2000	26JUN2000		No	2	Neg						
		Week 8	03JUL2000	03JUL2000	56	No	1	Pos	P.f	40.3	403.2	887.1	-967.7	
		Week 8	03JUL2000	03JUL2000		No	2	Pos	P.f	137.1	1370.9			
		Week 9 (Fol. Up)	10JUL2000	10JUL2000	63	No	1	Pos	P.f	8.4	83.6	80.3	6.7	
		Week 9 (Fol. Up)	10JUL2000	10JUL2000		No	2	Neg						
		Week 9 (Fol. Up)	10JUL2000	10JUL2000		No	Arb	Pos	P.f	7.7	76.9			
		Week 10 (Fol. Up)	17JUL2000	17JUL2000	70	No	1	Pos	P.f	1.7	20.7	29.3	-17.1	
		Week 10 (Fol. Up)	17JUL2000	17JUL2000		No	2	Pos	P.f	3.1	37.8			
		Week 11 (Fol. Up)	24JUL2000	24JUL2000	77	No	1	Pos	P.f	3.8	45.8	45.7	0.1	
		Week 11 (Fol. Up)	24JUL2000	24JUL2000		No	2	Neg						
		Week 11 (Fol. Up)	24JUL2000	24JUL2000		No	Arb	Pos	P.f	3.7	45.6			
		Week 11 (Unsch. Fol. Up)	27JUL2000	27JUL2000	80	Yes	1	Pos	P.f	18.3	223.5	166.3	114.4	

*Popn=Population (I=Intent-To-Treat, PP=Per Protocol)
**Rel Day=Number of days relative to start of prophylactic medication
Symp = Symptoms, Rd = Reader, Res = Result, Spec = Species, Diff = Difference
Spec Count W = Species Count recorded per 500 White Blood Cells
Spec Count B = Species Count converted to per microlitre of blood
Gam = Gametocyte
+Arb=Arbitrator, +++Pos=Positive, Neg=Negative
#P. f = P.falciparum, P. v = P.vivax, P. o = P.ovale, P. m = P.malariae
Tafenoquine = Tafenoquine 200mg, Mefloquine = Mefloquine 250mg

Listing C.01

Results Of Blood Smears By Visit

Treatment : Placebo

Subject Number	Popn *	Visit	Visit Date	Sample Date	Rel Day**	Symp ?	Rd+	Res +++	Spec #	Spec Count W	Spec Count B	Mean	Diff	Gam Count
030.001.00114	IPP	Week 11 (Unsch. Fol. Up)	27JUL2000	27JUL2000		Yes	2	Pos	P.f	8.9	109.1			
		Week 12 (Fol. Up)	31JUL2000	31JUL2000	84	Yes	1	Neg						
		Week 12 (Fol. Up)	31JUL2000	31JUL2000		Yes	2	Neg						
030.001.00115	IPP	Screening	17APR2000	17APR2000	-21		1	Neg						
		Screening	17APR2000	17APR2000			2	Neg						
		Post Clearance	06MAY2000	06MAY2000	-2		1	Neg						
		Post Clearance	06MAY2000	06MAY2000			2	Neg						
		Load Dose 1	08MAY2000			Yes								
		Load Dose 2	09MAY2000			Yes								
		Load Dose 3	10MAY2000			No								
		Week 1	15MAY2000	15MAY2000	7	No	1	Neg						
		Week 1	15MAY2000	15MAY2000		No	2	Neg						
		Week 2	22MAY2000	22MAY2000	14	No	1	Neg						
		Week 2	22MAY2000	22MAY2000		No	2	Neg						
		Week 3 (Unsch.)	26MAY2000			Yes								
		Week 3	29MAY2000	29MAY2000	21	Yes	1	Neg						
		Week 3	29MAY2000	29MAY2000		Yes	2	Neg						
		Week 4	05JUN2000	05JUN2000	28	No	1	Pos	P.f	0.1	2.2	11.4	-18.4	
		Week 4	05JUN2000	05JUN2000		No	2	Pos	P.f	1.1	20.6			
		Week 5 (Fol. Up)	12JUN2000	12JUN2000	35	No	1	Neg						
		Week 5 (Fol. Up)	12JUN2000	12JUN2000		No	2	Neg						
		Week 6 (Fol. Up)	19JUN2000	19JUN2000	42	No	1	Neg						
		Week 6 (Fol. Up)	19JUN2000	19JUN2000		No	2	Neg						
		Week 7 (Fol. Up)	26JUN2000	26JUN2000	49	No	1	Neg						
		Week 7 (Fol. Up)	26JUN2000	26JUN2000		No	2	Neg						
		Week 8 (Fol. Up)	03JUL2000	03JUL2000	56	No	1	Neg						
		Week 8 (Fol. Up)	03JUL2000	03JUL2000		No	2	Neg						
030.001.00116	IPP	Screening	12APR2000	12APR2000	-26		1	Neg						
		Screening	12APR2000	12APR2000			2	Neg						
		Post Clearance	06MAY2000	06MAY2000	-2		1	Neg						
		Post Clearance	06MAY2000	06MAY2000			2	Neg						
		Load Dose 1	08MAY2000			No								
		Load Dose 2	09MAY2000			No								
		Load Dose 3	10MAY2000			No								
		Week 1	15MAY2000	15MAY2000	7	No	1	Pos	P.f	3.0	33.6	29.7	7.8	

*Popn=Population (I=Intent-To-Treat, PP=Per Protocol)
**Rel Day=Number of days relative to start of prophylactic medication
Symp = Symptoms, Rd = Reader, Res = Result, Spec = Species, Diff = Difference
Spec Count W = Species Count recorded per 500 White Blood Cells
Spec Count B = Species Count converted to per microlitre of blood
Gam = Gametocyte
+Arb=Arbitrator, +++Pos=Positive, Neg=Negative
#P. f = P.falciparum, P. v = P.vivax, P. o = P.ovale, P. m = P.malariae
Tafenoquine = Tafenoquine 200mg, Mefloquine = Mefloquine 250mg

Listing C.01

Results Of Blood Smears By Visit

Treatment : Placebo

Subject Number	Popn *	Visit	Visit Date	Sample Date	Rel Day**	Symp ?	Rd+	Res +++	Spec #	Spec Count W	Spec Count B	Mean	Diff	Gam Count
030.001.00116	IPP	Week 1	15MAY2000	15MAY2000		No	2	Neg						
		Week 1	15MAY2000	15MAY2000		No	Arb	Pos	P.f	2.3	25.8			
		Week 1 (Unsch.)	15MAY2000			Yes								
		Week 2	22MAY2000	22MAY2000	14	No	1	Neg						
		Week 2	22MAY2000	22MAY2000		No	2	Pos	P.f	3.0	33.6	35.9	-4.6	
		Week 2	22MAY2000	22MAY2000		No	Arb	Pos	P.f	3.4	38.2			
		Week 3 (Unsch. Fol. Up)	26MAY2000			Yes								
		Week 3 (Fol. Up)	29MAY2000	29MAY2000	21	No	1	Neg						
		Week 3 (Fol. Up)	29MAY2000	29MAY2000		No	2	Neg						
		Week 3 (Unsch. Fol. Up)	30MAY2000			No								
		Week 4 (Fol. Up)	05JUN2000	05JUN2000	28	No	1	Neg						
		Week 4 (Fol. Up)	05JUN2000	05JUN2000		No	2	Neg						
		Week 5 (Fol. Up)	13JUN2000			No								
		Week 6 (Fol. Up)	20JUN2000	20JUN2000	43	No	1	Neg						
		Week 6 (Fol. Up)	20JUN2000	20JUN2000		No	2	Neg						
030.001.00119	IPP	Screening	11APR2000	11APR2000	-28		1	Pos	P.f	9.0	71.6	122.1	-101.0	
		Screening	11APR2000	11APR2000			2	Pos	P.f	21.6	172.6			
		Screening (Unsch.)	01MAY2000			Yes								
		Post Clearance	07MAY2000	07MAY2000	-2		1	Neg						
		Post Clearance	07MAY2000	07MAY2000			2	Neg						
		Load Dose 1	09MAY2000			No								
		Load Dose 2	10MAY2000			No								
		Load Dose 3	11MAY2000			No								
		Week 1	15MAY2000	15MAY2000	6	Yes	1	Pos	P.f	2.0	18.0	25.1	-14.1	
		Week 1	15MAY2000	15MAY2000		Yes	2	Neg						
		Week 1	15MAY2000	15MAY2000		Yes	Arb	Pos	P.f	3.6	32.1			
		Week 2	23MAY2000	23MAY2000	14	Yes	1	Neg						
		Week 2	23MAY2000	23MAY2000		Yes	2	Neg						
		Week 3 (Fol. Up)	31MAY2000	31MAY2000	22	No	1	Neg						
		Week 3 (Fol. Up)	31MAY2000	31MAY2000		No	2	Neg						
		Week 4 (Fol. Up)	07JUN2000	07JUN2000	29	No	1	Neg						
		Week 4 (Fol. Up)	07JUN2000	07JUN2000		No	2	Neg						
		Week 5 (Fol. Up)	12JUN2000	12JUN2000	34	No	1	Neg						
		Week 5 (Fol. Up)	12JUN2000	12JUN2000		No	2	Neg						
		Week 6 (Fol. Up)	19JUN2000	19JUN2000	41		1	Neg						
		Week 6 (Fol. Up)	19JUN2000	19JUN2000			2	Neg						

*Popn=Population (I=Intent-To-Treat, PP=Per Protocol)
**Rel Day=Number of days relative to start of prophylactic medication
Symp = Symptoms, Rd = Reader, Res = Result, Spec = Species, Diff = Difference
Spec Count W = Species Count recorded per 500 White Blood Cells
Spec Count B = Species Count converted to per microlitre of blood
Gam = Gametocyte
+Arb=Arbitrator, +++Pos=Positive, Neg=Negative
#P. f = P.falciparum, P. v = P.vivax, P. o = P.ovale, P. m = P.malariae
Tafenoquine = Tafenoquine 200mg, Mefloquine = Mefloquine 250mg

SB252263 - 030

Listing C.01

Results Of Blood Smears By Visit

Treatment : Placebo

Subject Number	Popn *	Visit	Visit Date	Sample Date	Rel Day**	Symp ?	Rd+	Res +++	Spec #	Spec Count W	Spec Count B	Mean	Diff	Gam Count
030.001.00119	IPP	Week 6 (Fol. Up)	19JUN2000	19JUN2000		No		Neg						
030.001.00124	IPP	Screening	11APR2000	11APR2000	-28		1	Neg						
		Screening	11APR2000	11APR2000			2	Pos	P.f	1.0	6.3	4.3	4.0	
		Screening	11APR2000	11APR2000			Arb	Pos	P.f	0.3	2.2			
		Post Clearance	07MAY2000	07MAY2000	-2		1	Neg						
		Post Clearance	07MAY2000	07MAY2000			2	Neg						
		Load Dose 1	09MAY2000			No								
		Load Dose 2	10MAY2000			No								
		Load Dose 3	11MAY2000			No								
		Week 1	15MAY2000	15MAY2000	6	No	1	Neg						
		Week 1	15MAY2000	15MAY2000		No	2	Neg						
		Week 2	22MAY2000	22MAY2000	13	No	1	Neg						
		Week 2	22MAY2000	22MAY2000		No	2	Neg						
		Week 3	29MAY2000	29MAY2000	20	Yes	1	Neg						
		Week 3	29MAY2000	29MAY2000		Yes	2	Neg						
		Week 3 (Unsch.)	30MAY2000			Yes								
		Week 4	05JUN2000	05JUN2000	27	No	1	Pos	P.f	0.9	4.5	4.6	-0.1	
		Week 4	05JUN2000	05JUN2000		No	2	Pos	P.f	0.9	4.6			
		Week 4 (Unsch. Fol. Up)	09JUN2000			Yes								
		Week 5 (Unsch. Fol. Up)	13JUN2000			No								
		Week 5 (Fol. Up)	14JUN2000	14JUN2000	36	No	1	Pos	P.f	1.0	6.0	5.2	1.7	
		Week 5 (Fol. Up)	14JUN2000	14JUN2000		No	2	Pos	P.f	0.7	4.3			
		Week 6 (Unsch. Fol. Up)	18JUN2000			No								
		Week 6 (Fol. Up)	19JUN2000	19JUN2000	41	No	1	Neg						
		Week 6 (Fol. Up)	19JUN2000	19JUN2000		No	2	Neg						
		Week 7 (Fol. Up)	26JUN2000	26JUN2000	48	No	1	Pos	P.f	0.3	1.6	3.2	-3.2	
		Week 7 (Fol. Up)	26JUN2000	26JUN2000		No	2	Pos	P.f	0.8	4.8			
		Week 8 (Fol. Up)	03JUL2000	03JUL2000	55	Yes	1	Neg						
		Week 8 (Fol. Up)	03JUL2000	03JUL2000		Yes	2	Neg						
030.001.00125	IPP	Screening	10APR2000	10APR2000	-29		1	Neg						
		Screening	10APR2000	10APR2000			2	Pos	P.f	5.0	42.0	42.0	0.0	
		Screening	10APR2000	10APR2000			Arb	Pos	P.f	5.0	42.0			
		Screening (Unsch.)	02MAY2000			Yes								
		Post Clearance	07MAY2000	07MAY2000	-2		1	Neg						
		Post Clearance	07MAY2000	07MAY2000			2	Neg						

*Popn=Population (I=Intent-To-Treat, PP=Per Protocol)
**Rel Day=Number of days relative to start of prophylactic medication
Symp = Symptoms, Rd = Reader, Res = Result, Spec = Species, Diff = Difference
Spec Count W = Species Count recorded per 500 White Blood Cells
Spec Count B = Species Count converted to per microlitre of blood
Gam = Gametocyte
+Arb=Arbitrator, +++Pos=Positive, Neg=Negative
#P. f = P.falciparum, P. v = P.vivax, P. o = P.ovale, P. m = P.malariae
Tafenoquine = Tafenoquine 200mg, Mefloquine = Mefloquine 250mg

SB252263 - 030

Listing C.01

Results Of Blood Smears By Visit

Treatment : Placebo

Subject Number	Popn *	Visit	Visit Date	Sample Date	Rel Day**	Symp ?	Rd+	Res +++	Spec #	Spec Count W	Spec Count B	Mean	Diff	Gam Count
030.001.00125	IPP	Load Dose 1	09MAY2000			No								
		Load Dose 2	10MAY2000			Yes								
		Load Dose 3	11MAY2000			No								
		Week 1	15MAY2000	15MAY2000	6	No	1	Neg						
		Week 1	15MAY2000	15MAY2000		No	2	Neg						
		Week 2 (Unsch.)	20MAY2000			No								
		Week 2 (Unsch.)	21MAY2000			No								
		Week 2	22MAY2000	22MAY2000	13	Yes	1	Neg						
		Week 2	22MAY2000	22MAY2000		Yes	2	Neg						
		Week 2 (Unsch.)	24MAY2000			No								
		Week 3	29MAY2000	29MAY2000	20	Yes	1	Neg						
		Week 3	29MAY2000	29MAY2000		Yes	2	Neg						
		Week 3 (Unsch.)	29MAY2000			Yes								
		Week 4	05JUN2000	05JUN2000	27	No	1	Neg						
		Week 4	05JUN2000	05JUN2000		No	2	Neg						
		Week 4 (Unsch.)	07JUN2000			No								
		Week 5	12JUN2000	12JUN2000	34	Yes	1	Neg						
		Week 5	12JUN2000	12JUN2000		Yes	2	Neg						
		Week 6	20JUN2000	20JUN2000	42	No	1	Neg						
		Week 6	20JUN2000	20JUN2000		No	2	Neg						
		Week 7	26JUN2000	26JUN2000	48	No	1	Neg						
		Week 7	26JUN2000	26JUN2000		No	2	Neg						
		Week 8	03JUL2000	03JUL2000	55	No	1	Pos	P.f	4.8	37.4	43.9	-12.9	
		Week 8	03JUL2000	03JUL2000		No	2	Pos	P.f	6.5	50.3			
		Week 9 (Fol. Up)	10JUL2000	10JUL2000	62	Yes	1	Neg						
		Week 9 (Fol. Up)	10JUL2000	10JUL2000		Yes	2	Neg						
		Week 9 (Unsch. Fol. Up)	11JUL2000			No								
		Week 9 (Unsch. Fol. Up)	13JUL2000			No								
		Week 10 (Fol. Up)	17JUL2000	17JUL2000	69	No	1	Neg						
		Week 10 (Fol. Up)	17JUL2000	17JUL2000		No	2	Neg						
		Week 11 (Fol. Up)	24JUL2000	24JUL2000	76	No	1	Neg						
		Week 11 (Fol. Up)	24JUL2000	24JUL2000		No	2	Neg						
		Week 11 (Unsch. Fol. Up)	26JUL2000			No								
		Week 12 (Fol. Up)	31JUL2000	31JUL2000	83		1	Neg						
		Week 12 (Fol. Up)	31JUL2000	31JUL2000			2	Neg						
030.001.00127	I	Screening	12APR2000	12APR2000	-27		1	Pos	P.f	1.0	7.0			

*Popn=Population (I=Intent-To-Treat, PP=Per Protocol)
**Rel Day=Number of days relative to start of prophylactic medication
Symp = Symptoms, Rd = Reader, Res = Result, Spec = Species, Diff = Difference
Spec Count W = Species Count recorded per 500 White Blood Cells
Spec Count B = Species Count converted to per microlitre of blood
Gam = Gametocyte
+Arb=Arbitrator, +++Pos=Positive, Neg=Negative
#P. f = P.falciparum, P. v = P.vivax, P. o = P.ovale, P. m = P.malariae
Tafenoquine = Tafenoquine 200mg, Mefloquine = Mefloquine 250mg

SB252263 - 030

Listing C.01

Results Of Blood Smears By Visit

Treatment : Placebo

Subject Number	Popn *	Visit	Visit Date	Sample Date	Rel Day**	Symp ?	Rd+	Res +++	Spec #	Spec Count W	Spec Count B	Mean	Diff	Gam Count
030.001.00127	I	Screening	12APR2000	12APR2000			2	Neg						
		Screening	12APR2000	12APR2000			Arb	Neg						
		Post Clearance	07MAY2000	07MAY2000	-2		1	Neg						
		Post Clearance	07MAY2000	07MAY2000			2	Neg						
		Load Dose 1	09MAY2000			No								
		Load Dose 2	10MAY2000			No								
		Load Dose 3	11MAY2000			No								
		Week 1 (Unsch.)	14MAY2000			Yes								
		Week 1	16MAY2000	16MAY2000	7	No	1	Neg						
		Week 1	16MAY2000	16MAY2000		No	2	Neg						
		Week 2 (Unsch.)	20MAY2000			No								
		Week 2	24MAY2000	24MAY2000	15	No	1	Neg						
		Week 2	24MAY2000	24MAY2000		No	2	Neg						
		Week 2 (Unsch.)	24MAY2000			Yes								
		Week 3	29MAY2000	29MAY2000	20	Yes	1	Neg						
		Week 3	29MAY2000	29MAY2000		Yes	2	Neg						
		Week 3 (Unsch.)	29MAY2000			No								
		Week 4	08JUN2000	08JUN2000	30	No	1	Neg						
		Week 4	08JUN2000	08JUN2000		No	2	Neg						
		Week 5	11JUN2000	11JUN2000	33	Yes		Neg						
030.001.00131	IPP	Screening	10APR2000	10APR2000	-29		1	Neg						
		Screening	10APR2000	10APR2000			2	Neg						
		Screening (Unsch.)	04MAY2000	04MAY2000	-5		1	Neg						
		Screening (Unsch.)	04MAY2000	04MAY2000			2	Neg						
		Post Clearance	07MAY2000	07MAY2000	-2		1	Neg						
		Post Clearance	07MAY2000	07MAY2000			2	Neg						
		Load Dose 1	09MAY2000			No								
		Load Dose 2	10MAY2000			No								
		Load Dose 3	11MAY2000			No								
		Week 1	15MAY2000	15MAY2000	6	No	1	Neg						
		Week 1	15MAY2000	15MAY2000		No	2	Neg						
		Week 1 (Unsch.)	19MAY2000			Yes								
		Week 2	22MAY2000	22MAY2000	13	No	1	Neg						
		Week 2	22MAY2000	22MAY2000		No	2	Neg						
		Week 3	29MAY2000	29MAY2000	20	No	1	Neg						
		Week 3	29MAY2000	29MAY2000		No	2	Neg						

*Popn=Population (I=Intent-To-Treat, PP=Per Protocol)
**Rel Day=Number of days relative to start of prophylactic medication
Symp = Symptoms, Rd = Reader, Res = Result, Spec = Species, Diff = Difference
Spec Count W = Species Count recorded per 500 White Blood Cells
Spec Count B = Species Count converted to per microlitre of blood
Gam = Gametocyte
+Arb=Arbitrator, +++Pos=Positive, Neg=Negative
#P. f = P.falciparum, P. v = P.vivax, P. o = P.ovale, P. m = P.malariae
Tafenoquine = Tafenoquine 200mg, Mefloquine = Mefloquine 250mg

SB252263 - 030

Listing C.01

Results Of Blood Smears By Visit

Treatment : Placebo

Subject Number	Popn *	Visit	Visit Date	Sample Date	Rel Day**	Symp ?	Rd+	Res +++	Spec #	Spec Count W	Spec Count B	Mean	Diff	Gam Count
030.001.00131	IPP	Week 4	06JUN2000	06JUN2000	28	No	1	Neg						
		Week 4	06JUN2000	06JUN2000		No	2	Neg						
		Week 4 (Unsch.)	07JUN2000			Yes								
		Week 5	12JUN2000	12JUN2000	34	No	1	Neg						
		Week 5	12JUN2000	12JUN2000		No	2	Neg						
		Week 6	19JUN2000	19JUN2000	41	Yes	1	Neg						
		Week 6	19JUN2000	19JUN2000		Yes	2	Neg						
		Week 6 (Unsch.)	21JUN2000			Yes								
		Week 7	26JUN2000	26JUN2000	48	Yes	1	Pos	P.f	0.4	4.8	5.1	-0.6	
		Week 7	26JUN2000	26JUN2000		Yes	2	Pos	P.f	0.5	5.4			
		Week 8 (Fol. Up)	03JUL2000	03JUL2000	55	No	1	Pos	P.f	6.0	68.9	83.3	-28.8	
		Week 8 (Fol. Up)	03JUL2000	03JUL2000		No	2	Pos	P.f	8.6	97.7			
		Week 9 (Fol. Up)	10JUL2000	10JUL2000	62	No	1	Neg						
		Week 9 (Fol. Up)	10JUL2000	10JUL2000		No	2	Neg						
		Week 10 (Fol. Up)	17JUL2000	17JUL2000	69	No	1	Pos	P.f	6.9	53.7	34.7	38.1	
		Week 10 (Fol. Up)	17JUL2000	17JUL2000			1		P.o	13.8	107.6	61.6	92.0	
		Week 10 (Fol. Up)	17JUL2000	17JUL2000		No	2	Pos	P.f	2.0	15.6			
		Week 10 (Fol. Up)	17JUL2000	17JUL2000			2		P.o	2.0	15.6			
		Week 11 (Fol. Up)	24JUL2000	24JUL2000	76		1	Neg						
		Week 11 (Fol. Up)	24JUL2000	24JUL2000			2	Neg						
		Week 11 (Unsch. Fol. Up)	24JUL2000			No								
030.001.00136	IPP	Screening	11APR2000	11APR2000	-28		1	Pos	P.f	1.0	11.6			
		Screening	11APR2000	11APR2000			2	Neg						
		Screening	11APR2000	11APR2000			Arb	Neg						
		Post Clearance	07MAY2000	07MAY2000	-2		1	Neg						
		Post Clearance	07MAY2000	07MAY2000			2	Neg						
		Load Dose 1	09MAY2000			Yes								
		Load Dose 2	10MAY2000			No								
		Load Dose 3	11MAY2000			No								
		Week 1	15MAY2000	15MAY2000	6	No	1	Neg						
		Week 1	15MAY2000	15MAY2000		No	2	Neg						
		Week 1 (Unsch.)	16MAY2000			Yes								
		Week 2	22MAY2000	22MAY2000	13	Yes	1	Neg						
		Week 2	22MAY2000	22MAY2000		Yes	2	Neg						
		Week 2 (Unsch.)	26MAY2000			Yes								
		Week 3	29MAY2000	29MAY2000	20	No	1	Neg						

*Popn=Population (I=Intent-To-Treat, PP=Per Protocol)
**Rel Day=Number of days relative to start of prophylactic medication
Symp = Symptoms, Rd = Reader, Res = Result, Spec = Species, Diff = Difference
Spec Count W = Species Count recorded per 500 White Blood Cells
Spec Count B = Species Count converted to per microlitre of blood
Gam = Gametocyte
+Arb=Arbitrator, +++Pos=Positive, Neg=Negative
#P. f = P.falciparum, P. v = P.vivax, P. o = P.ovale, P. m = P.malariae
Tafenoquine = Tafenoquine 200mg, Mefloquine = Mefloquine 250mg

SB252263 - 030

Listing C.01

Results Of Blood Smears By Visit

Treatment : Placebo

Subject Number	Popn *	Visit	Visit Date	Sample Date	Rel Day**	Symp ?	Rd+	Res +++	Spec #	Spec Count W	Spec Count B	Mean	Diff	Gam Count
030.001.00136	IPP	Week 3	29MAY2000	29MAY2000		No	2	Neg						
		Week 3 (Unsch.)	29MAY2000			Yes								
		Week 4	05JUN2000	05JUN2000	27	No	1	Pos	P.f	0.2	1.4	3.8	-4.7	
		Week 4	05JUN2000	05JUN2000		No	2	Pos	P.f	0.8	6.2			
		Week 5 (Fol. Up)	12JUN2000	12JUN2000	34	Yes	1	Neg						
		Week 5 (Fol. Up)	12JUN2000	12JUN2000		Yes	2	Neg						
		Week 5 (Unsch. Fol. Up)	13JUN2000			Yes								
		Week 6 (Fol. Up)	19JUN2000	19JUN2000	41	No	1	Neg						
		Week 6 (Fol. Up)	19JUN2000	19JUN2000		No	2	Neg						
		Week 6 (Unsch. Fol. Up)	22JUN2000			No								
		Week 7 (Fol. Up)	26JUN2000	26JUN2000	48	Yes	1	Neg						
		Week 7 (Fol. Up)	26JUN2000	26JUN2000		Yes	2	Neg						
		Week 7 (Unsch. Fol. Up)	26JUN2000	26JUN2000		Yes	1	Neg						
		Week 7 (Unsch. Fol. Up)	26JUN2000	26JUN2000		Yes	2	Neg						
		Week 8 (Fol. Up)	03JUL2000	03JUL2000	55	No	1	Neg						
		Week 8 (Fol. Up)	03JUL2000	03JUL2000		No	2	Neg						
030.001.00137	IPP	Screening	11APR2000	11APR2000	-28		1	Neg						
		Screening	11APR2000	11APR2000			2	Neg						
		Screening (Unsch.)	30APR2000	30APR2000	-9		1	Neg						
		Screening (Unsch.)	30APR2000	30APR2000			2	Neg						
		Post Clearance	07MAY2000	07MAY2000	-2		1	Neg						
		Post Clearance	07MAY2000	07MAY2000			2	Neg						
		Load Dose 1	09MAY2000			No								
		Load Dose 2	10MAY2000			No								
		Load Dose 3	11MAY2000			No								
		Week 1	15MAY2000	15MAY2000	6	No	1	Neg						
		Week 1	15MAY2000	15MAY2000		No	2	Neg						
		Week 2	22MAY2000	22MAY2000	13	No	1	Neg						
		Week 2	22MAY2000	22MAY2000		No	2	Neg						
		Week 3	29MAY2000	29MAY2000	20	No	1	Neg						
		Week 3	29MAY2000	29MAY2000		No	2	Neg						
		Week 4	04JUN2000	04JUN2000	26	No	1	Neg						
		Week 4	04JUN2000	04JUN2000		No	2	Neg						
		Week 5	12JUN2000	12JUN2000	34	No	1	Neg						
		Week 5	12JUN2000	12JUN2000		No	2	Neg						
		Week 6	19JUN2000	19JUN2000	41	No	1	Neg						

*Popn=Population (I=Intent-To-Treat, PP=Per Protocol)
**Rel Day=Number of days relative to start of prophylactic medication
Symp = Symptoms, Rd = Reader, Res = Result, Spec = Species, Diff = Difference
Spec Count W = Species Count recorded per 500 White Blood Cells
Spec Count B = Species Count converted to per microlitre of blood
Gam = Gametocyte
+Arb=Arbitrator, +++Pos=Positive, Neg=Negative
#P. f = P.falciparum, P. v = P.vivax, P. o = P.ovale, P. m = P.malariae
Tafenoquine = Tafenoquine 200mg, Mefloquine = Mefloquine 250mg

SB252263 - 030

Listing C.01

Results Of Blood Smears By Visit

Treatment : Placebo

Subject Number	Popn *	Visit	Visit Date	Sample Date	Rel Day**	Symp ?	Rd+	Res +++	Spec #	Spec Count W	Spec Count B	Mean	Diff	Gam Count
030.001.00137	IPP	Week 6	19JUN2000	19JUN2000		No	2	Neg						
		Week 7	26JUN2000	26JUN2000	48	No	1	Neg						
		Week 7	26JUN2000	26JUN2000		No	2	Neg						
		Week 7 (Unsch.)	30JUN2000			No								
		Week 8	03JUL2000	03JUL2000	55	No	1	Pos	P.f	5.5	65.4	45.8	39.1	
		Week 8	03JUL2000	03JUL2000		No	2	Pos	P.f	2.2	26.3			
		Week 9 (Unsch. Fol. Up)	08JUL2000			No								
		Week 9 (Fol. Up)	10JUL2000	10JUL2000	62	No	1	Neg						
		Week 9 (Fol. Up)	10JUL2000	10JUL2000		No	2	Neg						
		Week 9 (Unsch. Fol. Up)	12JUL2000			No								
		Week 10 (Fol. Up)	17JUL2000	17JUL2000	69	No	1	Neg						
		Week 10 (Fol. Up)	17JUL2000	17JUL2000		No	2	Neg						
		Week 11 (Fol. Up)	24JUL2000	24JUL2000	76	No	1	Neg						
		Week 11 (Fol. Up)	24JUL2000	24JUL2000		No	2	Neg						
		Week 12 (Fol. Up)	31JUL2000	31JUL2000	83		1	Pos	P.f	8.1	82.4	64.4	36.1	
		Week 12 (Fol. Up)	31JUL2000	31JUL2000			2	Pos	P.f	4.5	46.3			
030.001.00140	IPP	Screening	10APR2000	10APR2000	-29		1	Pos	P.f	18.2	207.3	171.5	71.6	
		Screening	10APR2000	10APR2000			2	Pos	P.f	11.9	135.7			
		Post Clearance	07MAY2000	07MAY2000	-2		1	Neg						
		Post Clearance	07MAY2000	07MAY2000			2	Neg						
		Load Dose 1	09MAY2000			No								
		Load Dose 2	10MAY2000			No								
		Load Dose 3	11MAY2000			No								
		Load Dose (Unsch.)	11MAY2000	11MAY2000	2		1	Neg						
		Load Dose (Unsch.)	11MAY2000	11MAY2000			2	Neg						
		Week 1	15MAY2000	15MAY2000	6	No	1	Neg						
		Week 1	15MAY2000	15MAY2000		No	2	Neg						
		Week 2	22MAY2000	22MAY2000	13	No	1	Neg						
		Week 2	22MAY2000	22MAY2000		No	2	Neg						
		Week 2 (Unsch.)	22MAY2000			No								
		Week 3	28MAY2000	28MAY2000	19	No	1	Neg						
		Week 3	28MAY2000	28MAY2000		No	2	Neg						
		Week 4	07JUN2000	07JUN2000	29	No	1	Neg						
		Week 4	07JUN2000	07JUN2000		No	2	Neg						
		Week 5	12JUN2000	12JUN2000	34	No	1	Neg						
		Week 5	12JUN2000	12JUN2000		No	2	Neg						

*Popn=Population (I=Intent-To-Treat, PP=Per Protocol)
**Rel Day=Number of days relative to start of prophylactic medication
Symp = Symptoms, Rd = Reader, Res = Result, Spec = Species, Diff = Difference
Spec Count W = Species Count recorded per 500 White Blood Cells
Spec Count B = Species Count converted to per microlitre of blood
Gam = Gametocyte
+Arb=Arbitrator, +++Pos=Positive, Neg=Negative
#P. f = P.falciparum, P. v = P.vivax, P. o = P.ovale, P. m = P.malariae
Tafenoquine = Tafenoquine 200mg, Mefloquine = Mefloquine 250mg

SB252263 - 030

Listing C.01

Results Of Blood Smears By Visit

Treatment : Placebo

Subject Number	Popn *	Visit	Visit Date	Sample Date	Rel Day**	Symp ?	Rd+	Res +++	Spec #	Spec Count W	Spec Count B	Mean	Diff	Gam Count
030.001.00140	IPP	Week 6	19JUN2000	19JUN2000	41	No	1	Pos	P.f	3.4	37.2	32.8	8.7	
		Week 6	19JUN2000	19JUN2000		No	2	Pos	P.f	2.6	28.5			
		Week 6 (Unsch. Fol. Up)	23JUN2000			Yes								
		Week 7 (Fol. Up)	26JUN2000	26JUN2000	48	No	1	Pos	P.f	0.1	0.5	1.2	-1.4	
		Week 7 (Fol. Up)	26JUN2000	26JUN2000		No	2	Pos	P.f	0.2	1.9			
		Week 8 (Fol. Up)	03JUL2000	03JUL2000	55	No	1	Neg						
		Week 8 (Fol. Up)	03JUL2000	03JUL2000		No	2	Neg						
		Week 9 (Fol. Up)	10JUL2000	10JUL2000	62	No	1	Neg						
		Week 9 (Fol. Up)	10JUL2000	10JUL2000		No	2	Neg						
		Week 10 (Fol. Up)	17JUL2000	17JUL2000	69	No	1	Neg						
		Week 10 (Fol. Up)	17JUL2000	17JUL2000		No	2	Neg						
030.001.00141	IPP	Screening	11APR2000	11APR2000	-28		1	Pos	P.f	19.3	189.2	173.0	32.4	
		Screening	11APR2000	11APR2000			1		P.m	1.1	11.1	10.1	1.9	
		Screening	11APR2000	11APR2000			2	Pos	P.f	16.0	156.8			
		Screening	11APR2000	11APR2000			2		P.m	0.9	9.2			
		Screening (Unsch.)	18APR2000	18APR2000	-21	Yes		Neg						
		Post Clearance	07MAY2000	07MAY2000	-2		1	Neg						
		Post Clearance	07MAY2000	07MAY2000			2	Neg						
		Load Dose 1	09MAY2000			No								
		Load Dose 2	10MAY2000			No								
		Load Dose 3	11MAY2000			No								
		Week 1	15MAY2000	15MAY2000	6	Yes	1	Neg						
		Week 1	15MAY2000	15MAY2000		Yes	2	Neg						
		Week 2	22MAY2000	22MAY2000	13	No	1	Neg						
		Week 2	22MAY2000	22MAY2000		No	2	Neg						
		Week 3	29MAY2000	29MAY2000	20	Yes	1	Neg						
		Week 3	29MAY2000	29MAY2000		Yes	2	Neg						
		Week 4	06JUN2000	06JUN2000	28	No	1	Neg						
		Week 4	06JUN2000	06JUN2000		No	2	Neg						
		Week 5	14JUN2000	14JUN2000	36	No	1	Neg						
		Week 5	14JUN2000	14JUN2000		No	2	Neg						
		Week 6	19JUN2000	19JUN2000	41	Yes	1	Neg						
		Week 6	19JUN2000	19JUN2000		Yes	2	Neg						
		Week 7	26JUN2000	26JUN2000	48	No	1	Pos	P.f	13.4	112.8	92.9	39.9	
		Week 7	26JUN2000	26JUN2000		No	2	Pos	P.f	8.7	72.9			
		Week 8 (Fol. Up)	03JUL2000	03JUL2000	55	No	1	Neg						

*Popn=Population (I=Intent-To-Treat, PP=Per Protocol)
**Rel Day=Number of days relative to start of prophylactic medication
Symp = Symptoms, Rd = Reader, Res = Result, Spec = Species, Diff = Difference
Spec Count W = Species Count recorded per 500 White Blood Cells
Spec Count B = Species Count converted to per microlitre of blood
Gam = Gametocyte
+Arb=Arbitrator, +++Pos=Positive, Neg=Negative
#P. f = P.falciparum, P. v = P.vivax, P. o = P.ovale, P. m = P.malariae
Tafenoquine = Tafenoquine 200mg, Mefloquine = Mefloquine 250mg

SB252263 - 030

Listing C.01

Results Of Blood Smears By Visit

Treatment : Placebo

Subject Number	Popn *	Visit	Visit Date	Sample Date	Rel Day**	Symp ?	Rd+	Res +++	Spec #	Spec Count W	Spec Count B	Mean	Diff	Gam Count
030.001.00141	IPP	Week 8 (Fol. Up)	03JUL2000	03JUL2000		No	2	Neg						
		Week 9 (Fol. Up)	10JUL2000	10JUL2000	62	No	1	Neg						
		Week 9 (Fol. Up)	10JUL2000	10JUL2000		No	2	Neg						
		Week 10 (Fol. Up)	17JUL2000	17JUL2000	69	No	1	Neg						
		Week 10 (Fol. Up)	17JUL2000	17JUL2000		No	2	Neg						
		Week 11 (Fol. Up)	24JUL2000	24JUL2000	76		1	Neg						
		Week 11 (Fol. Up)	24JUL2000	24JUL2000			2	Neg						
030.001.00145	IPP	Screening	18APR2000	18APR2000	-21		1	Neg						
		Screening	18APR2000	18APR2000			2	Neg						
		Screening (Unsch.)	04MAY2000	04MAY2000	-5	Yes	1	Neg						
		Screening (Unsch.)	04MAY2000	04MAY2000		Yes	2	Neg						
		Post Clearance	07MAY2000	07MAY2000	-2		1	Neg						
		Post Clearance	07MAY2000	07MAY2000			2	Neg						
		Load Dose 1	09MAY2000			No								
		Load Dose 2	10MAY2000			No								
		Load Dose 3	11MAY2000			No								
		Week 1	15MAY2000	15MAY2000	6	Yes	1	Neg						
		Week 1	15MAY2000	15MAY2000		Yes	2	Pos	P.f	1.0	10.6	16.3	-11.4	
		Week 1	15MAY2000	15MAY2000		Yes	Arb	Pos	P.f	2.1	22.0			
		Week 2	23MAY2000	23MAY2000	14	Yes	1	Neg						
		Week 2	23MAY2000	23MAY2000		Yes	2	Neg						
		Week 3	29MAY2000	29MAY2000	20	Yes	1	Neg						
		Week 3	29MAY2000	29MAY2000		Yes	2	Neg						
		Week 4	06JUN2000	06JUN2000	28	Yes	1	Neg						
		Week 4	06JUN2000	06JUN2000		Yes	2	Neg						
		Week 5	13JUN2000	13JUN2000	35	No	1	Neg						
		Week 5	13JUN2000	13JUN2000		No	2	Pos	P.f	2.1	27.5	22.8	9.2	
		Week 5	13JUN2000	13JUN2000		No	Arb	Pos	P.f	1.4	18.2			
		Week 6 (Fol. Up)	23JUN2000	23JUN2000	45	Yes	1	Neg						
		Week 6 (Fol. Up)	23JUN2000	23JUN2000		Yes	2	Neg						
		Week 7 (Fol. Up)	26JUN2000	26JUN2000	48	Yes	1	Neg						
		Week 7 (Fol. Up)	26JUN2000	26JUN2000		Yes	2	Neg						
		Week 8 (Unsch. Fol. Up)	01JUL2000			Yes								
		Week 8 (Fol. Up)	04JUL2000	04JUL2000	56	Yes	1	Neg						
		Week 8 (Fol. Up)	04JUL2000	04JUL2000		Yes	2	Neg						
		Week 9 (Fol. Up)	10JUL2000	10JUL2000	62	Yes	1	Neg						

*Popn=Population (I=Intent-To-Treat, PP=Per Protocol)
**Rel Day=Number of days relative to start of prophylactic medication
Symp = Symptoms, Rd = Reader, Res = Result, Spec = Species, Diff = Difference
Spec Count W = Species Count recorded per 500 White Blood Cells
Spec Count B = Species Count converted to per microlitre of blood
Gam = Gametocyte
+Arb=Arbitrator, +++Pos=Positive, Neg=Negative
#P. f = P.falciparum, P. v = P.vivax, P. o = P.ovale, P. m = P.malariae
Tafenoquine = Tafenoquine 200mg, Mefloquine = Mefloquine 250mg

SB252263 - 030

Listing C.01

Results Of Blood Smears By Visit

Treatment : Placebo

Subject Number	Popn *	Visit	Visit Date	Sample Date	Rel Day**	Symp ?	Rd+	Res +++	Spec #	Spec Count W	Spec Count B	Mean	Diff	Gam Count
030.001.00145	IPP	Week 9 (Fol. Up)	10JUL2000	10JUL2000		Yes	2	Neg						
		Week 9 (Unsch. Fol. Up)	11JUL2000			Yes								
030.001.00151	IPP	Screening	11APR2000	11APR2000	-28		1	Pos	P.f	4.1	37.4	80.1	-85.6	
		Screening	11APR2000	11APR2000			2	Pos	P.f	13.4	122.9			
		Post Clearance	07MAY2000	07MAY2000	-2		1	Neg						
		Post Clearance	07MAY2000	07MAY2000			2	Neg						
		Load Dose 1	09MAY2000			No								
		Load Dose 2	10MAY2000			Yes								
		Load Dose 3	11MAY2000			No								
		Week 1	15MAY2000	15MAY2000	6	No	1	Neg						
		Week 1	15MAY2000	15MAY2000		No	2	Neg						
		Week 2	22MAY2000	22MAY2000	13	Yes	1	Neg						
		Week 2	22MAY2000	22MAY2000		Yes	2	Neg						
		Week 2 (Unsch.)	23MAY2000			Yes								
		Week 3	29MAY2000	29MAY2000	20	No	1	Neg						
		Week 3	29MAY2000	29MAY2000		No	2	Neg						
		Week 3 (Unsch.)	30MAY2000			Yes								
		Week 4	04JUN2000	04JUN2000	26	No	1	Neg						
		Week 4	04JUN2000	04JUN2000		No	2	Neg						
		Week 5	12JUN2000	12JUN2000	34	No	1	Neg						
		Week 5	12JUN2000	12JUN2000		No	2	Neg						
		Week 6	19JUN2000	19JUN2000	41	No	1	Neg						
		Week 6	19JUN2000	19JUN2000		No	2	Neg						
		Week 7	26JUN2000	26JUN2000	48	Yes	1	Neg						
		Week 7	26JUN2000	26JUN2000		Yes	2	Neg						
		Week 8	03JUL2000	03JUL2000	55	Yes	1	Neg						
		Week 8	03JUL2000	03JUL2000		Yes	2	Neg						
		Week 9	10JUL2000	10JUL2000	62	No	1	Pos	P.f	2.2	23.2	20.2	5.9	
		Week 9	10JUL2000	10JUL2000		No	2	Neg						
		Week 9	10JUL2000	10JUL2000		No	Arb	Pos	P.f	1.7	17.3			
		Week 10 (Fol. Up)	17JUL2000	17JUL2000	69	No	1	Pos	P.f	22.1	212.3	164.0	96.5	
		Week 10 (Fol. Up)	17JUL2000	17JUL2000		No	2	Pos	P.f	12.1	115.8			
		Week 10 (Unsch. Fol. Up)	19JUL2000	19JUL2000	71	Yes	1	Pos	P.f	39.6	380.3	231.0	298.6	
		Week 10 (Unsch. Fol. Up)	19JUL2000	19JUL2000		Yes	2	Pos	P.f	8.5	81.7			
		Week 11 (Fol. Up)	24JUL2000	24JUL2000	76	No	1	Pos	P.f	1.5	14.8	80.1	-130.7	
		Week 11 (Fol. Up)	24JUL2000	24JUL2000		No	2	Pos	P.f	15.2	145.4			

*Popn=Population (I=Intent-To-Treat, PP=Per Protocol)
**Rel Day=Number of days relative to start of prophylactic medication
Symp = Symptoms, Rd = Reader, Res = Result, Spec = Species, Diff = Difference
Spec Count W = Species Count recorded per 500 White Blood Cells
Spec Count B = Species Count converted to per microlitre of blood
Gam = Gametocyte
+Arb=Arbitrator, +++Pos=Positive, Neg=Negative
#P. f = P.falciparum, P. v = P.vivax, P. o = P.ovale, P. m = P.malariae
Tafenoquine = Tafenoquine 200mg, Mefloquine = Mefloquine 250mg

SB252263 - 030

Listing C.01

Results Of Blood Smears By Visit

Treatment : Placebo

Subject Number	Popn *	Visit	Visit Date	Sample Date	Rel Day**	Symp ?	Rd+	Res +++	Spec #	Spec Count W	Spec Count B	Mean	Diff	Gam Count
030.001.00151	IPP	Week 12 (Fol. Up)	31JUL2000	31JUL2000	83	No	1	Pos	P.f	2.6	25.1	28.0	-5.9	
		Week 12 (Fol. Up)	31JUL2000	31JUL2000		No	2	Pos	P.f	3.2	30.9			
		Week 13 (Fol. Up)	07AUG2000	07AUG2000	90		1	Neg						
		Week 13 (Fol. Up)	07AUG2000	07AUG2000			2	Neg						
030.001.00152	IPP	Screening	13APR2000	13APR2000	-26		1	Pos	P.f	41.7	566.6	448.7	235.7	
		Screening	13APR2000	13APR2000			2	Pos	P.f	24.3	330.9			
		Screening (Unsch.)	18APR2000	18APR2000	-21		1	Neg						
		Screening (Unsch.)	18APR2000	18APR2000			2	Neg						
		Post Clearance	07MAY2000	07MAY2000	-2		1	Neg						
		Post Clearance	07MAY2000	07MAY2000			2	Neg						
		Load Dose 1	09MAY2000			No								
		Load Dose 2	10MAY2000			No								
		Load Dose 3	11MAY2000			No								
		Week 1	15MAY2000	15MAY2000	6	No	1	Neg						
		Week 1	15MAY2000	15MAY2000		No	2	Neg						
		Week 2 (Unsch.)	20MAY2000			Yes								
		Week 2	22MAY2000	22MAY2000	13	No	1	Neg						
		Week 2	22MAY2000	22MAY2000		No	2	Neg						
		Week 2 (Unsch.)	26MAY2000			Yes								
		Week 3 (Unsch.)	27MAY2000			Yes								
		Week 3	29MAY2000	29MAY2000	20	No	1	Neg						
		Week 3	29MAY2000	29MAY2000		No	2	Neg						
		Week 4	05JUN2000	05JUN2000	27	No	1	Neg						
		Week 4	05JUN2000	05JUN2000		No	2	Neg						
		Week 5	12JUN2000	12JUN2000	34	No	1	Neg						
		Week 5	12JUN2000	12JUN2000		No	2	Neg						
		Week 6	19JUN2000	19JUN2000	41	No	1	Pos	P.f	2.1	24.4	13.9	21.0	
		Week 6	19JUN2000	19JUN2000		No	2	Pos	P.f	0.3	3.4			
		Week 7	26JUN2000	26JUN2000	48	No	1	Neg						
		Week 7	26JUN2000	26JUN2000		No	2	Neg						
		Week 7 (Unsch. Fol. Up)	28JUN2000			Yes								
		Week 8 (Unsch. Fol. Up)	06JUL2000			Yes								
		Week 9 (Fol. Up)	10JUL2000	10JUL2000	62	No	1	Neg						
		Week 9 (Fol. Up)	10JUL2000	10JUL2000		No	2	Neg						
		Week 9 (Unsch. Fol. Up)	13JUL2000			Yes								
		Week 10 (Fol. Up)	17JUL2000	17JUL2000	69	No	1	Neg						

*Popn=Population (I=Intent-To-Treat, PP=Per Protocol)
**Rel Day=Number of days relative to start of prophylactic medication
Symp = Symptoms, Rd = Reader, Res = Result, Spec = Species, Diff = Difference
Spec Count W = Species Count recorded per 500 White Blood Cells
Spec Count B = Species Count converted to per microlitre of blood
Gam = Gametocyte
+Arb=Arbitrator, +++Pos=Positive, Neg=Negative
#P. f = P.falciparum, P. v = P.vivax, P. o = P.ovale, P. m = P.malariae
Tafenoquine = Tafenoquine 200mg, Mefloquine = Mefloquine 250mg

SB252263 - 030

Listing C.01

Results Of Blood Smears By Visit

Treatment : Placebo

Subject Number	Popn *	Visit	Visit Date	Sample Date	Rel Day**	Symp ?	Rd+	Res +++	Spec #	Spec Count W	Spec Count B	Mean	Diff	Gam Count
030.001.00152	IPP	Week 10 (Fol. Up)	17JUL2000	17JUL2000		No	2	Neg						
		Week 11 (Fol. Up)	24JUL2000	24JUL2000	76	No	1	Pos	P.f	1.1	12.6	59.9	-94.6	
		Week 11 (Fol. Up)	24JUL2000	24JUL2000		No	2	Pos	P.f	9.1	107.3			
030.001.00154	IPP	Screening	13APR2000	13APR2000	-26		1	Pos	P.f	15.7	163.3	138.5	49.6	
		Screening	13APR2000	13APR2000			2	Pos	P.f	10.9	113.7			
		Screening (Unsch.)	13APR2000	13APR2000			1	Pos	P.f	15.7	163.3	138.5	49.6	
		Screening (Unsch.)	13APR2000	13APR2000			2	Pos	P.f	10.9	113.7			
		Post Clearance	07MAY2000	07MAY2000	-2		1	Neg						
		Post Clearance	07MAY2000	07MAY2000			2	Neg						
		Load Dose 1	09MAY2000			Yes								
		Load Dose 2	10MAY2000			No								
		Load Dose 3	11MAY2000			No								
		Week 1	15MAY2000	15MAY2000	6	No	1	Neg						
		Week 1	15MAY2000	15MAY2000		No	2	Neg						
		Week 2	22MAY2000	22MAY2000	13	No	1	Neg						
		Week 2	22MAY2000	22MAY2000		No	2	Neg						
		Week 3	29MAY2000	29MAY2000	20	Yes	1	Neg						
		Week 3	29MAY2000	29MAY2000		Yes	2	Neg						
		Week 4	07JUN2000	07JUN2000	29	Yes	1	Neg						
		Week 4	07JUN2000	07JUN2000		Yes	2	Neg						
		Week 5	12JUN2000	12JUN2000	34	No	1	Pos	P.f	1.6	17.0	16.5	1.0	
		Week 5	12JUN2000	12JUN2000		No	2	Pos	P.f	1.5	16.0			
		Week 6 (Fol. Up)	19JUN2000	19JUN2000	41	No	1	Pos	P.f	0.2	2.1	4.7	-5.2	
		Week 6 (Fol. Up)	19JUN2000	19JUN2000		No	2	Pos	P.f	0.8	7.2			
		Week 7 (Fol. Up)	26JUN2000	26JUN2000	48	No	1	Neg						
		Week 7 (Fol. Up)	26JUN2000	26JUN2000		No	2	Neg						
		Week 7 (Unsch. Fol. Up)	27JUN2000			Yes								
		Week 9 (Fol. Up)	09JUL2000	09JUL2000	61	Yes	1	Neg						
		Week 9 (Fol. Up)	09JUL2000	09JUL2000		Yes	2	Neg						
		Week 9 (Fol. Up)	10JUL2000	10JUL2000	62		1	Pos	P.f	2.7	27.0	32.0	-10.0	
		Week 9 (Fol. Up)	10JUL2000	10JUL2000			2	Neg						
		Week 9 (Fol. Up)	10JUL2000	10JUL2000			Arb	Pos	P.f	3.7	37.0			
		Week 9 (Fol. Up)	12JUL2000			No								
030.001.00160	I	Screening	11APR2000	11APR2000	-28		1	Neg						
		Screening	11APR2000	11APR2000			2	Neg						

*Popn=Population (I=Intent-To-Treat, PP=Per Protocol)
**Rel Day=Number of days relative to start of prophylactic medication
Symp = Symptoms, Rd = Reader, Res = Result, Spec = Species, Diff = Difference
Spec Count W = Species Count recorded per 500 White Blood Cells
Spec Count B = Species Count converted to per microlitre of blood
Gam = Gametocyte
+Arb=Arbitrator, +++Pos=Positive, Neg=Negative
#P. f = P.falciparum, P. v = P.vivax, P. o = P.ovale, P. m = P.malariae
Tafenoquine = Tafenoquine 200mg, Mefloquine = Mefloquine 250mg

SB252263 - 030

Listing C.01

Results Of Blood Smears By Visit

Treatment : Placebo

Subject Number	Popn *	Visit	Visit Date	Sample Date	Rel Day**	Symp ?	Rd+	Res +++	Spec #	Spec Count W	Spec Count B	Mean	Diff	Gam Count
030.001.00160	I	Post Clearance	07MAY2000	07MAY2000	-2		1	Neg						
		Post Clearance	07MAY2000	07MAY2000			2	Neg						
		Load Dose 1	09MAY2000			No								
		Load Dose 2	10MAY2000			No								
		Load Dose 3	11MAY2000			No								
		Week 1 (Unsch.)	12MAY2000			No								
		Week 1	15MAY2000	15MAY2000	6	Yes	1	Neg						
		Week 1	15MAY2000	15MAY2000		Yes	2	Neg						
		Week 2	22MAY2000	22MAY2000	13	No	1	Neg						
		Week 2	22MAY2000	22MAY2000		No	2	Neg						
		Week 2 (Unsch.)	26MAY2000			No								
		Week 3	29MAY2000	29MAY2000	20	No	1	Neg						
		Week 3	29MAY2000	29MAY2000		No	2	Neg						
		Week 3 (Unsch.)	31MAY2000			Yes								
		Week 4	05JUN2000	05JUN2000	27	Yes	1	Neg						
		Week 4	05JUN2000	05JUN2000		Yes	2	Neg						
		Week 4 (Unsch.)	07JUN2000			Yes								
		Week 5	12JUN2000	12JUN2000	34	No	1	Neg						
		Week 5	12JUN2000	12JUN2000		No	2	Neg						
		Week 6	19JUN2000	19JUN2000	41	Yes	1	Pos	P.f	3.1	20.0	147.6	-255.4	
		Week 6	19JUN2000	19JUN2000		Yes	2	Pos	P.f	43.0	275.3			
		Week 7	26JUN2000	26JUN2000	48	No	1	Pos	P.f	80.7	516.6	615.9	-198.7	
		Week 7	26JUN2000	26JUN2000		No	2	Pos	P.f	111.8	715.3			
		Week 8 (Fol. Up)	05JUL2000	05JUL2000	57	No	1	Neg						
		Week 8 (Fol. Up)	05JUL2000	05JUL2000		No	2	Neg						
		Week 9 (Fol. Up)	10JUL2000	10JUL2000	62	No	1	Neg						
		Week 9 (Fol. Up)	10JUL2000	10JUL2000		No	2	Pos	P.f	0.5	3.4	5.0	-3.2	
		Week 9 (Fol. Up)	10JUL2000	10JUL2000		No	Arb	Pos	P.f	1.0	6.6			
		Week 10 (Fol. Up)	17JUL2000	17JUL2000	69	No	1	Neg						
		Week 10 (Fol. Up)	17JUL2000	17JUL2000		No	2	Neg						
		Week 11 (Fol. Up)	25JUL2000	25JUL2000	77	Yes	1	Pos	P.f	3.7	23.5	78.2	-109.4	
		Week 11 (Fol. Up)	25JUL2000	25JUL2000		Yes	2	Pos	P.f	20.8	132.9			
		Week 11 (Unsch. Fol. Up)	26JUL2000			Yes								
030.001.00162	IPP	Screening	11APR2000	11APR2000	-28		1	Neg						
		Screening	11APR2000	11APR2000			2	Neg						
		Screening (Unsch.)	24APR2000	24APR2000	-15		1	Neg						

*Popn=Population (I=Intent-To-Treat, PP=Per Protocol)
**Rel Day=Number of days relative to start of prophylactic medication
Symp = Symptoms, Rd = Reader, Res = Result, Spec = Species, Diff = Difference
Spec Count W = Species Count recorded per 500 White Blood Cells
Spec Count B = Species Count converted to per microlitre of blood
Gam = Gametocyte
+Arb=Arbitrator, +++Pos=Positive, Neg=Negative
#P. f = P.falciparum, P. v = P.vivax, P. o = P.ovale, P. m = P.malariae
Tafenoquine = Tafenoquine 200mg, Mefloquine = Mefloquine 250mg

Listing C.01

Results Of Blood Smears By Visit

Treatment : Placebo

Subject Number	Popn *	Visit	Visit Date	Sample Date	Rel Day**	Symp ?	Rd+	Res +++	Spec #	Spec Count W	Spec Count B	Mean	Diff	Gam Count
030.001.00162	IPP	Screening (Unsch.)	24APR2000	24APR2000			2	Neg						
		Post Clearance	07MAY2000	07MAY2000	-2		1	Neg						
		Post Clearance	07MAY2000	07MAY2000			2	Neg						
		Load Dose 1	09MAY2000			No								
		Load Dose 2	10MAY2000			Yes								
		Load Dose 3	11MAY2000			Yes								
		Week 1	16MAY2000	16MAY2000	7	Yes	1	Neg						
		Week 1	16MAY2000	16MAY2000		Yes	2	Neg						
		Week 2 (Unsch.)	21MAY2000			No								
		Week 2	22MAY2000	22MAY2000	13	Yes	1	Neg						
		Week 2	22MAY2000	22MAY2000		Yes	2	Neg						
		Week 3	29MAY2000	29MAY2000	20	No	1	Neg						
		Week 3	29MAY2000	29MAY2000		No	2	Neg						
		Week 4	05JUN2000	05JUN2000	27	No	1	Neg						
		Week 4	05JUN2000	05JUN2000		No	2	Neg						
		Week 5	12JUN2000	12JUN2000	34	No	1	Neg						
		Week 5	12JUN2000	12JUN2000		No	2	Neg						
		Week 6	19JUN2000	19JUN2000	41	No	1	Neg						
		Week 6	19JUN2000	19JUN2000		No	2	Neg						
		Week 7	26JUN2000	26JUN2000	48	No	1	Neg						
		Week 7	26JUN2000	26JUN2000		No	2	Neg						
		Week 7 (Unsch.)	27JUN2000			Yes								
		Week 8	03JUL2000	03JUL2000	55	No	1	Pos	P.f	11.6	145.8	128.3	34.9	
		Week 8	03JUL2000	03JUL2000		No	2	Pos	P.f	8.8	110.9			
		Week 9 (Fol. Up)	10JUL2000	10JUL2000	62	No	1	Neg						
		Week 9 (Fol. Up)	10JUL2000	10JUL2000		No	2	Neg						
		Week 10 (Fol. Up)	17JUL2000	17JUL2000	69	No	1	Pos	P.f	17.2	213.5	228.4	-29.8	
		Week 10 (Fol. Up)	17JUL2000	17JUL2000		No	2	Pos	P.f	19.6	243.3			
		Week 11 (Fol. Up)	25JUL2000	25JUL2000	77	No	1	Pos	P.f	3.1	33.7	29.7	8.0	
		Week 11 (Fol. Up)	25JUL2000	25JUL2000		No	2	Pos	P.f	2.4	25.7			
		Week 12 (Fol. Up)	01AUG2000			No								
030.001.00164	IPP	Screening	14APR2000	14APR2000	-25		1	Pos	P.f	72.6	682.4	722.7	-80.5	
		Screening	14APR2000	14APR2000			1		P.m	2.7	25.7	36.4	-21.4	
		Screening	14APR2000	14APR2000			2	Pos	P.f	81.2	762.9			
		Screening	14APR2000	14APR2000			2		P.m	5.0	47.1			
		Post Clearance	07MAY2000	07MAY2000	-2		1	Neg						

*Popn=Population (I=Intent-To-Treat, PP=Per Protocol)
**Rel Day=Number of days relative to start of prophylactic medication
Symp = Symptoms, Rd = Reader, Res = Result, Spec = Species, Diff = Difference
Spec Count W = Species Count recorded per 500 White Blood Cells
Spec Count B = Species Count converted to per microlitre of blood
Gam = Gametocyte
+Arb=Arbitrator, +++Pos=Positive, Neg=Negative
#P. f = P.falciparum, P. v = P.vivax, P. o = P.ovale, P. m = P.malariae
Tafenoquine = Tafenoquine 200mg, Mefloquine = Mefloquine 250mg

SB252263 - 030

Listing C.01

Results Of Blood Smears By Visit

Treatment : Placebo

Subject Number	Popn *	Visit	Visit Date	Sample Date	Rel Day**	Symp ?	Rd+	Res +++	Spec #	Spec Count W	Spec Count B	Mean	Diff	Gam Count
030.001.00164	IPP	Post Clearance	07MAY2000	07MAY2000			2	Neg						
		Post Clearance (Unsch.)	08MAY2000			Yes								
		Load Dose 1	09MAY2000			No								
		Load Dose 2	10MAY2000			No								
		Load Dose 3	11MAY2000			No								
		Week 1	15MAY2000	15MAY2000	6	No	1	Neg						
		Week 1	15MAY2000	15MAY2000		No	2	Neg						
		Week 2	22MAY2000	22MAY2000	13	Yes	1	Neg						
		Week 2	22MAY2000	22MAY2000		Yes	2	Neg						
		Week 2 (Unsch.)	23MAY2000			Yes								
		Week 3	29MAY2000	29MAY2000	20	No	1	Neg						
		Week 3	29MAY2000	29MAY2000		No	2	Neg						
		Week 4	05JUN2000	05JUN2000	27	Yes	1	Neg						
		Week 4	05JUN2000	05JUN2000		Yes	2	Pos	P.f	0.8	8.2	5.8	4.8	
		Week 4	05JUN2000	05JUN2000		Yes	Arb	Pos	P.f	0.3	3.4			
		Week 5	12JUN2000	12JUN2000	34	No	1	Neg						
		Week 5	12JUN2000	12JUN2000		No	2	Neg						
		Week 6 (Fol. Up)	19JUN2000	19JUN2000	41	No	1	Pos	P.f	2.6	28.4	28.4	0.0	
		Week 6 (Fol. Up)	19JUN2000	19JUN2000		No	2	Neg						
		Week 6 (Fol. Up)	19JUN2000	19JUN2000		No	Arb	Pos	P.f	2.6	28.4			
		Week 7 (Unsch. Fol. Up)	26JUN2000			Yes								
		Week 7 (Fol. Up)	27JUN2000	27JUN2000	49	No	1	Pos	P.f	100.8	887.0	571.0	632.0	
		Week 7 (Fol. Up)	27JUN2000	27JUN2000		No	2	Pos	P.f	29.0	255.0			
		Week 8 (Fol. Up)	03JUL2000	03JUL2000	55	No	1	Neg						
		Week 8 (Fol. Up)	03JUL2000	03JUL2000		No	2	Neg						
		Week 9 (Fol. Up)	10JUL2000	10JUL2000	62	No	1	Neg						
		Week 9 (Fol. Up)	10JUL2000	10JUL2000		No	2	Neg						
030.001.00166	IPP	Screening	10APR2000	10APR2000	-29		1	Neg						
		Screening	10APR2000	10APR2000			2	Neg						
		Post Clearance	07MAY2000	07MAY2000	-2		1	Neg						
		Post Clearance	07MAY2000	07MAY2000			2	Neg						
		Load Dose 1	09MAY2000			No								
		Load Dose 2	10MAY2000			No								
		Load Dose 3	11MAY2000			No								
		Week 1	15MAY2000	15MAY2000	6	No	1	Neg						
		Week 1	15MAY2000	15MAY2000		No	2	Neg						

*Popn=Population (I=Intent-To-Treat, PP=Per Protocol)
**Rel Day=Number of days relative to start of prophylactic medication
Symp = Symptoms, Rd = Reader, Res = Result, Spec = Species, Diff = Difference
Spec Count W = Species Count recorded per 500 White Blood Cells
Spec Count B = Species Count converted to per microlitre of blood
Gam = Gametocyte
+Arb=Arbitrator, +++Pos=Positive, Neg=Negative
#P. f = P.falciparum, P. v = P.vivax, P. o = P.ovale, P. m = P.malariae
Tafenoquine = Tafenoquine 200mg, Mefloquine = Mefloquine 250mg

Listing C.01

Results Of Blood Smears By Visit

Treatment : Placebo

Subject Number	Popn *	Visit	Visit Date	Sample Date	Rel Day**	Symp ?	Rd+	Res +++	Spec #	Spec Count W	Spec Count B	Mean	Diff	Gam Count
030.001.00166	IPP	Week 1 (Unsch.)	15MAY2000			No								
		Week 2	22MAY2000	22MAY2000	13	Yes	1	Neg						
		Week 2	22MAY2000	22MAY2000		Yes	2	Neg						
		Week 2 (Unsch.)	22MAY2000			No								
		Week 2 (Unsch.)	24MAY2000			Yes								
		Week 3	29MAY2000	29MAY2000	20	No	1	Neg						
		Week 3	29MAY2000	29MAY2000		No	2	Neg						
		Week 4	05JUN2000	05JUN2000	27	Yes	1	Neg						
		Week 4	05JUN2000	05JUN2000		Yes	2	Neg						
		Week 5	12JUN2000	12JUN2000	34	No	1	Neg						
		Week 5	12JUN2000	12JUN2000		No	2	Neg						
		Week 6	19JUN2000	19JUN2000	41	No	1	Neg						
		Week 6	19JUN2000	19JUN2000		No	2	Neg						
		Week 7	26JUN2000	26JUN2000	48	No	1	Neg						
		Week 7	26JUN2000	26JUN2000		No	2	Neg						
		Week 8	04JUL2000	04JUL2000	56	Yes	1	Neg						
		Week 8	04JUL2000	04JUL2000		Yes	2	Neg						
		Week 9	10JUL2000	10JUL2000	62	No	1	Neg						
		Week 9	10JUL2000	10JUL2000		No	2	Neg						
		Week 10	17JUL2000	17JUL2000	69	No	1	Neg						
		Week 10	17JUL2000	17JUL2000		No	2	Neg						
		Week 10 (Unsch.)	19JUL2000			No								
		Week 11	25JUL2000	25JUL2000	77	No	1	Neg						
		Week 11	25JUL2000	25JUL2000		No	2	Neg						
		Week 11 (Unsch.)	26JUL2000			No								
		Week 12	31JUL2000	31JUL2000	83	No	1	Neg						
		Week 12	31JUL2000	31JUL2000		No	2	Neg						
		Week 13	07AUG2000	07AUG2000	90	Yes	1	Neg						
		Week 13	07AUG2000	07AUG2000		Yes	2	Neg						
		Week 14	14AUG2000	14AUG2000	97	No	1	Neg						
		Week 14	14AUG2000	14AUG2000		No	2	Neg						
		Week 14 (Unsch.)	16AUG2000			No								
		Week 15	22AUG2000	22AUG2000	105	No	1	Neg						
		Week 15	22AUG2000	22AUG2000		No	2	Neg						
		Week 15 (Unsch.)	22AUG2000			No								
		Week 16	28AUG2000	28AUG2000	111	No	1	Neg						
		Week 16	28AUG2000	28AUG2000		No	2	Neg						

*Popn=Population (I=Intent-To-Treat, PP=Per Protocol)
**Rel Day=Number of days relative to start of prophylactic medication
Symp = Symptoms, Rd = Reader, Res = Result, Spec = Species, Diff = Difference
Spec Count W = Species Count recorded per 500 White Blood Cells
Spec Count B = Species Count converted to per microlitre of blood
Gam = Gametocyte
+Arb=Arbitrator, +++Pos=Positive, Neg=Negative
#P. f = P.falciparum, P. v = P.vivax, P. o = P.ovale, P. m = P.malariae
Tafenoquine = Tafenoquine 200mg, Mefloquine = Mefloquine 250mg

SB252263 - 030

Listing C.01

Results Of Blood Smears By Visit

Treatment : Placebo

Subject Number	Popn *	Visit	Visit Date	Sample Date	Rel Day**	Symp ?	Rd+	Res +++	Spec #	Spec Count W	Spec Count B	Mean	Diff	Gam Count
030.001.00166	IPP	Week 17	04SEP2000	04SEP2000	118	No		Neg						
		Week 17 (Unsch.)	07SEP2000			No								
		Week 18	11SEP2000	11SEP2000	125	No	1	Neg						
		Week 18	11SEP2000	11SEP2000		No	2	Neg						
		Week 18 (Unsch.)	12SEP2000			No								
		Week 19	20SEP2000	20SEP2000	134	Yes	1	Neg						
		Week 19	20SEP2000	20SEP2000		Yes	2	Neg						
		Week 19 (Unsch.)	21SEP2000			No								
		Week 20	25SEP2000	25SEP2000	139	No	1	Neg						
		Week 20	25SEP2000	25SEP2000		No	2	Neg						
		Week 20 (Unsch.)	26SEP2000			Yes								
		Week 21	04OCT2000	04OCT2000	148	No	1	Neg						
		Week 21	04OCT2000	04OCT2000		No	2	Neg						
		Week 22	09OCT2000	09OCT2000	153	Yes	1	Neg						
		Week 22	09OCT2000	09OCT2000		Yes	2	Neg						
		Week 22 (Unsch.)	09OCT2000			Yes								
		Week 22 (Unsch.)	10OCT2000			Yes								
		Week 23	16OCT2000	16OCT2000	160	No	1	Neg						
		Week 23	16OCT2000	16OCT2000		No	2	Neg						
		Week 24	23OCT2000	23OCT2000	167	No	1	Neg						
		Week 24	23OCT2000	23OCT2000		No	2	Pos	P.f	1.4	15.4	11.8	7.2	
		Week 24	23OCT2000	23OCT2000		No	Arb	Pos	P.f	0.8	8.3			
		Week 24 (Unsch. Fol. Up)	26OCT2000			No								
		Week 25 (Unsch. Fol. Up)	30OCT2000	30OCT2000	174	Yes	1	Pos	P.f	47.6	523.7	466.2	115.1	
		Week 25 (Unsch. Fol. Up)	30OCT2000	30OCT2000		Yes	2	Pos	P.f	37.2	408.7			
		Week 25 (Fol. Up)	30OCT2000	30OCT2000		No	1	Pos	P.f	47.6	523.7	466.2	115.1	
		Week 25 (Fol. Up)	30OCT2000	30OCT2000		No	2	Pos	P.f	37.2	408.7			
		Week 26 (Unsch. Fol. Up)	06NOV2000	06NOV2000	181	No	1	Neg						
		Week 26 (Unsch. Fol. Up)	06NOV2000	06NOV2000		No	2	Neg						
		Week 26 (Fol. Up)	07NOV2000	07NOV2000	182	No	1	Pos	P.f	7.1	72.8	52.9	39.8	
		Week 26 (Fol. Up)	07NOV2000	07NOV2000		No	2	Neg						
		Week 26 (Fol. Up)	07NOV2000	07NOV2000		No	Arb	Pos	P.f	3.2	33.0			
		Week 27 (Fol. Up)	13NOV2000	13NOV2000	188	Yes	1	Pos	P.f	5.0	48.5	31.0	35.1	
		Week 27 (Fol. Up)	13NOV2000	13NOV2000		Yes	2	Neg						
		Week 27 (Fol. Up)	13NOV2000	13NOV2000		Yes	Arb	Pos	P.f	1.4	13.4			
		Week 27 (Unsch. Fol. Up)	16NOV2000			Yes								
		Week 28 (Fol. Up)	20NOV2000	20NOV2000	195	No	1	Neg						

*Popn=Population (I=Intent-To-Treat, PP=Per Protocol)
**Rel Day=Number of days relative to start of prophylactic medication
Symp = Symptoms, Rd = Reader, Res = Result, Spec = Species, Diff = Difference
Spec Count W = Species Count recorded per 500 White Blood Cells
Spec Count B = Species Count converted to per microlitre of blood
Gam = Gametocyte
+Arb=Arbitrator, +++Pos=Positive, Neg=Negative
#P. f = P.falciparum, P. v = P.vivax, P. o = P.ovale, P. m = P.malariae
Tafenoquine = Tafenoquine 200mg, Mefloquine = Mefloquine 250mg

SB252263 - 030

Listing C.01

Results Of Blood Smears By Visit

Treatment : Placebo

Subject Number	Popn *	Visit	Visit Date	Sample Date	Rel Day**	Symp ?	Rd+	Res +++	Spec #	Spec Count W	Spec Count B	Mean	Diff	Gam Count
030.001.00166	IPP	Week 28 (Fol. Up)	20NOV2000	20NOV2000		No	2	Neg						
030.001.00169	IPP	Screening	10APR2000	10APR2000	-29		1	Pos	P.f	19.7	227.9	242.8	-29.8	
		Screening	10APR2000	10APR2000			1		P.m	2.5	28.4	35.4	-13.9	
		Screening	10APR2000	10APR2000			2	Pos	P.f	22.2	257.8			
		Screening	10APR2000	10APR2000			2		P.m	3.7	42.3			
		Post Clearance	07MAY2000	07MAY2000	-2		1	Neg						
		Post Clearance	07MAY2000	07MAY2000			2	Neg						
		Load Dose 1	09MAY2000			No								
		Load Dose 2	10MAY2000			No								
		Load Dose 3	11MAY2000			No								
		Week 1 (Unsch.)	14MAY2000			Yes								
		Week 1	15MAY2000	15MAY2000	6	No	1	Neg						
		Week 1	15MAY2000	15MAY2000		No	2	Neg						
		Week 1 (Unsch.)	19MAY2000			Yes								
		Week 2	22MAY2000	22MAY2000	13	No	1	Neg						
		Week 2	22MAY2000	22MAY2000		No	2	Neg						
		Week 3	29MAY2000	29MAY2000	20	No	1	Neg						
		Week 3	29MAY2000	29MAY2000		No	2	Neg						
		Week 3 (Unsch.)	30MAY2000			No								
		Week 4 (Unsch.)	03JUN2000			No								
		Week 4	05JUN2000	05JUN2000	27	Yes	1	Neg						
		Week 4	05JUN2000	05JUN2000		Yes	2	Neg						
		Week 5	12JUN2000	12JUN2000	34	No	1	Neg						
		Week 5	12JUN2000	12JUN2000		No	2	Pos	P.f	4.2	29.1			
		Week 5	12JUN2000	12JUN2000		No	Arb	Neg						
		Week 5 (Unsch.)	13JUN2000			No								
		Week 6	19JUN2000	19JUN2000	41	No	1	Pos	P.f	1.6	20.1	24.7	-9.2	
		Week 6	19JUN2000	19JUN2000		No	2	Pos	P.f	2.3	29.3			
		Week 6 (Unsch.)	20JUN2000			No								
		Week 7	26JUN2000	26JUN2000	48	No	1	Pos	P.f	1.4	15.4	16.7	-2.6	
		Week 7	26JUN2000	26JUN2000		No	2	Pos	P.f	1.6	18.0			
		Week 8 (Fol. Up)	03JUL2000	03JUL2000	55	No	1	Neg						
		Week 8 (Fol. Up)	03JUL2000	03JUL2000		No	2	Pos	P.f	7.5	85.6	72.4	26.4	
		Week 8 (Fol. Up)	03JUL2000	03JUL2000		No	Arb	Pos	P.f	5.2	59.2			
		Week 9 (Fol. Up)	10JUL2000	10JUL2000	62	No	1	Neg						
		Week 9 (Fol. Up)	10JUL2000	10JUL2000		No	2	Neg						

*Popn=Population (I=Intent-To-Treat, PP=Per Protocol)
**Rel Day=Number of days relative to start of prophylactic medication
Symp = Symptoms, Rd = Reader, Res = Result, Spec = Species, Diff = Difference
Spec Count W = Species Count recorded per 500 White Blood Cells
Spec Count B = Species Count converted to per microlitre of blood
Gam = Gametocyte
+Arb=Arbitrator, +++Pos=Positive, Neg=Negative
#P. f = P.falciparum, P. v = P.vivax, P. o = P.ovale, P. m = P.malariae
Tafenoquine = Tafenoquine 200mg, Mefloquine = Mefloquine 250mg

SB252263 - 030

Listing C.01

Results Of Blood Smears By Visit

Treatment : Placebo

Subject Number	Popn *	Visit	Visit Date	Sample Date	Rel Day**	Symp ?	Rd+	Res +++	Spec #	Spec Count W	Spec Count B	Mean	Diff	Gam Count
030.001.00169	IPP	Week 9 (Unsch. Fol. Up)	13JUL2000			No								
		Week 10 (Fol. Up)	17JUL2000	17JUL2000	69	No	1	Pos	P.f	0.7	8.4	4.9	7.2	
		Week 10 (Fol. Up)	17JUL2000	17JUL2000		No	2	Neg						
		Week 10 (Fol. Up)	17JUL2000	17JUL2000		No	Arb	Pos	P.f	0.1	1.3			
		Week 10 (Unsch. Fol. Up)	17JUL2000			No								
		Week 11 (Fol. Up)	24JUL2000	24JUL2000	76		1	Neg						
		Week 11 (Fol. Up)	24JUL2000	24JUL2000			2	Neg						
		Week 11 (Unsch. Fol. Up)	24JUL2000			Yes								
030.001.00172	IPP	Screening	10APR2000	10APR2000	-29		1	Pos	P.f	15.0	171.1	131.0	80.3	
		Screening	10APR2000	10APR2000			2	Pos	P.f	8.0	90.9			
		Post Clearance	07MAY2000	07MAY2000	-2		1	Neg						
		Post Clearance	07MAY2000	07MAY2000			2	Neg						
		Load Dose 1	09MAY2000			No								
		Load Dose 2	10MAY2000			No								
		Load Dose 3	11MAY2000			No								
		Week 1	15MAY2000	15MAY2000	6	No	1	Neg						
		Week 1	15MAY2000	15MAY2000		No	2	Neg						
		Week 2	22MAY2000	22MAY2000	13	No	1	Neg						
		Week 2	22MAY2000	22MAY2000		No	2	Neg						
		Week 3	29MAY2000	29MAY2000	20	Yes	1	Neg						
		Week 3	29MAY2000	29MAY2000		Yes	2	Neg						
		Week 4 (Unsch.)	04JUN2000			Yes								
		Week 4	06JUN2000	06JUN2000	28	No	1	Neg						
		Week 4	06JUN2000	06JUN2000		No	2	Neg						
		Week 5	13JUN2000	13JUN2000	35	Yes	1	Neg						
		Week 5	13JUN2000	13JUN2000		Yes	2	Neg						
		Week 6	19JUN2000	19JUN2000	41	No	1	Neg						
		Week 6	19JUN2000	19JUN2000		No	2	Neg						
		Week 7	26JUN2000	26JUN2000	48	No	1	Neg						
		Week 7	26JUN2000	26JUN2000		No	2	Neg						
		Week 8	03JUL2000	03JUL2000	55	No	1	Neg						
		Week 8	03JUL2000	03JUL2000		No	2	Neg						
		Week 9	11JUL2000	11JUL2000	63	No	1	Neg						
		Week 9	11JUL2000	11JUL2000		No	2	Neg						
		Week 10	17JUL2000	17JUL2000	69	No	1	Neg						
		Week 10	17JUL2000	17JUL2000		No	2	Neg						

*Popn=Population (I=Intent-To-Treat, PP=Per Protocol)
**Rel Day=Number of days relative to start of prophylactic medication
Symp = Symptoms, Rd = Reader, Res = Result, Spec = Species, Diff = Difference
Spec Count W = Species Count recorded per 500 White Blood Cells
Spec Count B = Species Count converted to per microlitre of blood
Gam = Gametocyte
+Arb=Arbitrator, +++Pos=Positive, Neg=Negative
#P. f = P.falciparum, P. v = P.vivax, P. o = P.ovale, P. m = P.malariae
Tafenoquine = Tafenoquine 200mg, Mefloquine = Mefloquine 250mg

SB252263 - 030

Listing C.01

Results Of Blood Smears By Visit

Treatment : Placebo

Subject Number	Popn *	Visit	Visit Date	Sample Date	Rel Day**	Symp ?	Rd+	Res +++	Spec #	Spec Count W	Spec Count B	Mean	Diff	Gam Count
030.001.00172	IPP	Week 11	24JUL2000	24JUL2000	76	No	1	Neg						
		Week 11	24JUL2000	24JUL2000		No	2	Neg						
		Week 12	31JUL2000	31JUL2000	83	Yes	1	Neg						
		Week 12	31JUL2000	31JUL2000		Yes	2	Neg						
		Week 12 (Unsch.)	31JUL2000			Yes								
		Week 13	09AUG2000	09AUG2000	92	No	1	Neg						
		Week 13	09AUG2000	09AUG2000		No	2	Neg						
		Week 14	15AUG2000	15AUG2000	98	No	1	Neg						
		Week 14	15AUG2000	15AUG2000		No	2	Neg						
		Week 15	21AUG2000	21AUG2000	104	No	1	Neg						
		Week 15	21AUG2000	21AUG2000		No	2	Neg						
		Week 16	30AUG2000	30AUG2000	113	No	1	Neg						
		Week 16	30AUG2000	30AUG2000		No	2	Neg						
		Week 17	04SEP2000	04SEP2000	118	No	1	Neg						
		Week 17	04SEP2000	04SEP2000		No	2	Neg						
		Week 18	11SEP2000	11SEP2000	125	No	1	Neg						
		Week 18	11SEP2000	11SEP2000		No	2	Neg						
		Week 18 (Unsch.)	12SEP2000			No								
		Week 19	19SEP2000	19SEP2000	133	No	1	Neg						
		Week 19	19SEP2000	19SEP2000		No	2	Neg						
		Week 20	28SEP2000	28SEP2000	142	No	1	Neg						
		Week 20	28SEP2000	28SEP2000		No	2	Pos	P.f	9.0	90.2	78.4	23.6	
		Week 20	28SEP2000	28SEP2000		No	Arb	Pos	P.f	6.7	66.6			
		Week 21 (Fol. Up)	02OCT2000	02OCT2000	146	No	1	Neg						
		Week 21 (Fol. Up)	02OCT2000	02OCT2000		No	2	Neg						
		Week 22 (Fol. Up)	09OCT2000	09OCT2000	153	Yes	1	Neg						
		Week 22 (Fol. Up)	09OCT2000	09OCT2000		Yes	2	Neg						
		Week 22 (Unsch. Fol. Up)	09OCT2000			Yes								
		Week 24 (Fol. Up)	27OCT2000	27OCT2000	171	No	1	Neg						
		Week 24 (Fol. Up)	27OCT2000	27OCT2000		No	2	Neg						
030.001.00174	IPP	Screening	11APR2000	11APR2000	-28		1	Neg						
		Screening	11APR2000	11APR2000			2	Neg						
		Screening (Unsch.)	14APR2000	14APR2000	-25		1	Neg						
		Screening (Unsch.)	14APR2000	14APR2000			2	Neg						
		Screening (Unsch.)	25APR2000	25APR2000	-14		1	Neg						
		Screening (Unsch.)	25APR2000	25APR2000			2	Neg						

*Popn=Population (I=Intent-To-Treat, PP=Per Protocol)
**Rel Day=Number of days relative to start of prophylactic medication
Symp = Symptoms, Rd = Reader, Res = Result, Spec = Species, Diff = Difference
Spec Count W = Species Count recorded per 500 White Blood Cells
Spec Count B = Species Count converted to per microlitre of blood
Gam = Gametocyte
+Arb=Arbitrator, +++Pos=Positive, Neg=Negative
#P. f = P.falciparum, P. v = P.vivax, P. o = P.ovale, P. m = P.malariae
Tafenoquine = Tafenoquine 200mg, Mefloquine = Mefloquine 250mg

SB252263 - 030

Listing C.01

Results Of Blood Smears By Visit

Treatment : Placebo

Subject Number	Popn *	Visit	Visit Date	Sample Date	Rel Day**	Symp ?	Rd+	Res +++	Spec #	Spec Count W	Spec Count B	Mean	Diff	Gam Count
030.001.00174	IPP	Post Clearance	07MAY2000	07MAY2000	-2		1	Neg						
		Post Clearance	07MAY2000	07MAY2000			2	Neg						
		Load Dose 1	09MAY2000			No								
		Load Dose 2	10MAY2000			No								
		Load Dose 3	11MAY2000			No								
		Week 1	17MAY2000	17MAY2000	8	No	1	Neg						
		Week 1	17MAY2000	17MAY2000		No	2	Neg						
		Week 2	24MAY2000	24MAY2000	15	No	1	Neg						
		Week 2	24MAY2000	24MAY2000		No	2	Neg						
		Week 3	30MAY2000	30MAY2000	21	No	1	Neg						
		Week 3	30MAY2000	30MAY2000		No	2	Neg						
		Week 4	06JUN2000	06JUN2000	28	No	1	Neg						
		Week 4	06JUN2000	06JUN2000		No	2	Neg						
		Week 5	15JUN2000	15JUN2000	37	No	1	Neg						
		Week 5	15JUN2000	15JUN2000		No	2	Neg						
		Week 6	22JUN2000	22JUN2000	44	No	1	Neg						
		Week 6	22JUN2000	22JUN2000		No	2	Neg						
		Week 7	28JUN2000	28JUN2000	50	No	1	Neg						
		Week 7	28JUN2000	28JUN2000		No	2	Neg						
		Week 7 (Unsch.)	30JUN2000			Yes								
		Week 8	04JUL2000	04JUL2000	56	No	1	Neg						
		Week 8	04JUL2000	04JUL2000		No	2	Neg						
		Week 8 (Unsch.)	05JUL2000			Yes								
		Week 9	12JUL2000	12JUL2000	64	No	1	Pos	P.f	1.1	11.1	17.0	-11.6	
		Week 9	12JUL2000	12JUL2000		No	2	Pos	P.f	2.2	22.8			
		Week 10 (Fol. Up)	19JUL2000	19JUL2000	71	No	1	Neg						
		Week 10 (Fol. Up)	19JUL2000	19JUL2000		No	2	Neg						
		Week 10 (Unsch. Fol. Up)	19JUL2000			No								
		Week 11 (Fol. Up)	27JUL2000	27JUL2000	79	No	1	Neg						
		Week 11 (Fol. Up)	27JUL2000	27JUL2000		No	2	Pos	P.f	2.3	42.6	43.6	-2.0	
		Week 11 (Fol. Up)	27JUL2000	27JUL2000		No	Arb	Pos	P.f	2.4	44.6			
		Week 12 (Fol. Up)	02AUG2000	02AUG2000	85	Yes	1	Pos	P.f	5.6	104.0	68.4	71.1	
		Week 12 (Fol. Up)	02AUG2000	02AUG2000		Yes	2	Pos	P.f	1.8	32.9			
		Week 12 (Unsch. Fol. Up)	03AUG2000	03AUG2000	86	Yes	1	Neg						
		Week 12 (Unsch. Fol. Up)	03AUG2000	03AUG2000		Yes	2	Neg						
		Week 13 (Fol. Up)	09AUG2000	09AUG2000	92		2	Pos	P.f	1.2	21.8	17.0	-11.6	
		Week 13 (Fol. Up)	09AUG2000	09AUG2000			Arb	Pos	P.f	1.1	20.6			

*Popn=Population (I=Intent-To-Treat, PP=Per Protocol)
**Rel Day=Number of days relative to start of prophylactic medication
Symp = Symptoms, Rd = Reader, Res = Result, Spec = Species, Diff = Difference
Spec Count W = Species Count recorded per 500 White Blood Cells
Spec Count B = Species Count converted to per microlitre of blood
Gam = Gametocyte
+Arb=Arbitrator, +++Pos=Positive, Neg=Negative
#P. f = P.falciparum, P. v = P.vivax, P. o = P.ovale, P. m = P.malariae
Tafenoquine = Tafenoquine 200mg, Mefloquine = Mefloquine 250mg

SB252263 - 030

Listing C.01

Results Of Blood Smears By Visit

Treatment : Placebo

Subject Number	Popn *	Visit	Visit Date	Sample Date	Rel Day**	Symp ?	Rd+	Res +++	Spec #	Spec Count W	Spec Count B	Mean	Diff	Gam Count
030.001.00175	IPP	Screening	17APR2000	17APR2000	-22		1	Neg						
		Screening	17APR2000	17APR2000			2	Neg						
		Clearance (Unsch.)	05MAY2000	05MAY2000	-4		1	Neg						
		Clearance (Unsch.)	05MAY2000	05MAY2000			2	Neg						
		Post Clearance	07MAY2000	07MAY2000	-2		1	Neg						
		Post Clearance	07MAY2000	07MAY2000			2	Neg						
		Load Dose 1	09MAY2000			Yes								
		Load Dose 2	10MAY2000			No								
		Load Dose 3	11MAY2000			No								
		Week 1	17MAY2000	17MAY2000	8	No	1	Neg						
		Week 1	17MAY2000	17MAY2000		No	2	Neg						
		Week 2	24MAY2000	24MAY2000	15	No	1	Neg						
		Week 2	24MAY2000	24MAY2000		No	2	Neg						
		Week 3	31MAY2000	31MAY2000	22	No	1	Neg						
		Week 3	31MAY2000	31MAY2000		No	2	Neg						
		Week 4	06JUN2000	06JUN2000	28	Yes	1	Neg						
		Week 4	06JUN2000	06JUN2000		Yes	2	Neg						
		Week 5	14JUN2000	14JUN2000	36	No	1	Neg						
		Week 5	14JUN2000	14JUN2000		No	2	Neg						
		Week 6	21JUN2000	21JUN2000	43	No	1	Neg						
		Week 6	21JUN2000	21JUN2000		No	2	Neg						
		Week 7	28JUN2000	28JUN2000	50	No	1	Neg						
		Week 7	28JUN2000	28JUN2000		No	2	Neg						
		Week 8	04JUL2000	04JUL2000	56	No	1	Neg						
		Week 8	04JUL2000	04JUL2000		No	2	Neg						
		Week 8 (Unsch.)	07JUL2000			Yes								
		Week 9	12JUL2000	12JUL2000	64	No	1	Neg						
		Week 9	12JUL2000	12JUL2000		No	2	Pos	P.f	0.2	2.3	2.5	-0.5	
		Week 9	12JUL2000	12JUL2000		No	Arb	Pos	P.f	0.2	2.7			
		Week 10 (Fol. Up)	19JUL2000	19JUL2000	71	No	1	Neg						
		Week 10 (Fol. Up)	19JUL2000	19JUL2000		No	2	Neg						
		Week 11 (Fol. Up)	26JUL2000	26JUL2000	78	No	1	Neg						
		Week 11 (Fol. Up)	26JUL2000	26JUL2000		No	2	Neg						
		Week 12 (Fol. Up)	02AUG2000	02AUG2000	85	No	1	Pos	P.f	2.0	22.8	33.5	-21.3	
		Week 12 (Fol. Up)	02AUG2000	02AUG2000		No	2	Pos	P.f	3.9	44.1			
		Week 13 (Fol. Up)	09AUG2000	09AUG2000	92		1	Neg						
		Week 13 (Fol. Up)	09AUG2000	09AUG2000			2	Pos	P.f	0.3	2.9	2.7	0.2	

*Popn=Population (I=Intent-To-Treat, PP=Per Protocol)
**Rel Day=Number of days relative to start of prophylactic medication
Symp = Symptoms, Rd = Reader, Res = Result, Spec = Species, Diff = Difference
Spec Count W = Species Count recorded per 500 White Blood Cells
Spec Count B = Species Count converted to per microlitre of blood
Gam = Gametocyte
+Arb=Arbitrator, +++Pos=Positive, Neg=Negative
#P. f = P.falciparum, P. v = P.vivax, P. o = P.ovale, P. m = P.malariae
Tafenoquine = Tafenoquine 200mg, Mefloquine = Mefloquine 250mg

SB252263 - 030

Listing C.01

Results Of Blood Smears By Visit

Treatment : Placebo

Subject Number	Popn *	Visit	Visit Date	Sample Date	Rel Day**	Symp ?	Rd+	Res +++	Spec #	Spec Count W	Spec Count B	Mean	Diff	Gam Count
030.001.00175	IPP	Week 13 (Fol. Up)	09AUG2000	09AUG2000			Arb	Pos	P.f	0.2	2.6			
		Week 13 (Unsch. Fol. Up)	09AUG2000			No								
030.001.00182	I	Screening	10APR2000	10APR2000	-29		1	Pos	P.f	225.5	1984.5	2252.4	-535.9	
		Screening	10APR2000	10APR2000			2	Pos	P.f	286.4	2520.4			
		Post Clearance	07MAY2000	07MAY2000	-2		1	Neg						
		Post Clearance	07MAY2000	07MAY2000			2	Neg						
		Load Dose 1	09MAY2000			Yes								
		Load Dose 2	10MAY2000			No								
		Load Dose 3	11MAY2000			No								
		Week 1	17MAY2000	17MAY2000	8	No	1	Neg						
		Week 1	17MAY2000	17MAY2000		No	2	Neg						
		Week 2	24MAY2000	24MAY2000	15	No	1	Neg						
		Week 2	24MAY2000	24MAY2000		No	2	Neg						
		Week 3	31MAY2000	31MAY2000	22	No	1	Neg						
		Week 3	31MAY2000	31MAY2000		No	2	Neg						
		Week 4	06JUN2000	06JUN2000	28	No	1	Neg						
		Week 4	06JUN2000	06JUN2000		No	2	Neg						
		Week 5 (Unsch. Fol. Up)	12JUN2000			No								
		Week 5 (Fol. Up)	14JUN2000			No								
		Week 6 (Fol. Up)	19JUN2000			No								
		Week 7 (Fol. Up)	26JUN2000			No								
030.001.00183	IPP	Screening	11APR2000	11APR2000	-28		1	Pos	P.f	5.4	37.7	73.3	-71.2	
		Screening	11APR2000	11APR2000			1		P.m	0.9	6.2			
		Screening	11APR2000	11APR2000			2	Pos	P.f	15.6	108.9			
		Screening (Unsch.)	20APR2000	20APR2000	-19	Yes		Pos						
		Clearance (Unsch.)	05MAY2000			Yes								
		Post Clearance	07MAY2000	07MAY2000	-2		1	Neg						
		Post Clearance	07MAY2000	07MAY2000			2	Neg						
		Load Dose 1	09MAY2000			No								
		Load Dose 2	10MAY2000			No								
		Load Dose 3	11MAY2000			No								
		Week 1 (Unsch.)	14MAY2000			Yes								
		Week 1 (Unsch.)	15MAY2000			Yes								
		Week 1	17MAY2000	17MAY2000	8	No	1	Neg						
		Week 1	17MAY2000	17MAY2000		No	2	Neg						

*Popn=Population (I=Intent-To-Treat, PP=Per Protocol)
**Rel Day=Number of days relative to start of prophylactic medication
Symp = Symptoms, Rd = Reader, Res = Result, Spec = Species, Diff = Difference
Spec Count W = Species Count recorded per 500 White Blood Cells
Spec Count B = Species Count converted to per microlitre of blood
Gam = Gametocyte
+Arb=Arbitrator, +++Pos=Positive, Neg=Negative
#P. f = P.falciparum, P. v = P.vivax, P. o = P.ovale, P. m = P.malariae
Tafenoquine = Tafenoquine 200mg, Mefloquine = Mefloquine 250mg

SB252263 - 030

Listing C.01

Results Of Blood Smears By Visit

Treatment : Placebo

Subject Number	Popn *	Visit	Visit Date	Sample Date	Rel Day**	Symp ?	Rd+	Res +++	Spec #	Spec Count W	Spec Count B	Mean	Diff	Gam Count
030.001.00183	IPP	Week 2	24MAY2000	24MAY2000	15	No	1	Neg						
		Week 2	24MAY2000	24MAY2000		No	2	Neg						
		Week 3 (Unsch.)	28MAY2000			Yes								
		Week 3 (Unsch.)	30MAY2000			No								
		Week 3	31MAY2000	31MAY2000	22	No	1	Neg						
		Week 3	31MAY2000	31MAY2000		No	2	Neg						
		Week 4	06JUN2000	06JUN2000	28	No	1	Neg						
		Week 4	06JUN2000	06JUN2000		No	2	Neg						
		Week 5	14JUN2000	14JUN2000	36	Yes	1	Neg						
		Week 5	14JUN2000	14JUN2000		Yes	2	Neg						
		Week 6	21JUN2000	21JUN2000	43	No	1	Neg						
		Week 6	21JUN2000	21JUN2000		No	2	Neg						
		Week 7 (Unsch. Fol. Up)	27JUN2000	27JUN2000	49	Yes	1	Pos	P.f	2.0	16.4	12.3	8.2	
		Week 7 (Unsch. Fol. Up)	27JUN2000	27JUN2000		Yes	2	Pos	P.f	1.0	8.2			
		Week 7 (Fol. Up)	28JUN2000	28JUN2000	50	No	1	Pos	P.f	12.1	98.9	86.3	-25.1	
		Week 7 (Fol. Up)	28JUN2000	28JUN2000		No	2	Pos	P.f	9.0	73.8			
		Week 7 (Fol. Up)	28JUN2000	28JUN2000			2		P.o	5.0	41.0	55.8	-29.7	
		Week 7 (Fol. Up)	28JUN2000	28JUN2000		No	Arb	Pos	P.f	12.1	98.9			
		Week 7 (Fol. Up)	28JUN2000	28JUN2000			Arb		P.o	8.6	70.7			
		Week 8 (Fol. Up)	03JUL2000	03JUL2000	55	Yes	1	Neg						
		Week 8 (Fol. Up)	03JUL2000	03JUL2000		Yes	2	Neg						
		Week 8 (Unsch. Fol. Up)	03JUL2000	03JUL2000		Yes		Neg						
		Week 9 (Fol. Up)	12JUL2000	12JUL2000	64	No	1	Neg						
		Week 9 (Fol. Up)	12JUL2000	12JUL2000		No	2	Pos	P.f	0.2	1.6	1.6	0.0	
		Week 9 (Fol. Up)	12JUL2000	12JUL2000		No	Arb	Pos	P.f	0.2	1.6			
		Week 9 (Unsch. Fol. Up)	14JUL2000			Yes								
		Week 10 (Fol. Up)	19JUL2000	19JUL2000	71		1	Neg						
		Week 10 (Fol. Up)	19JUL2000	19JUL2000			2	Neg						
030.001.00191	IPP	Screening (Unsch.)	10APR2000			Yes								
		Screening	18APR2000	18APR2000	-21		1	Pos	P.f	15.7	346.3	354.0	-15.4	
		Screening	18APR2000	18APR2000			2	Pos	P.f	16.4	361.7			
		Screening (Unsch.)	18APR2000	18APR2000		No	1	Pos	P.f	5.6	122.8	108.9	27.7	
		Screening (Unsch.)	18APR2000	18APR2000		No	2	Pos	P.f	4.3	95.0			
		Post Clearance	07MAY2000	07MAY2000	-2		1	Neg						
		Post Clearance	07MAY2000	07MAY2000			2	Neg						
		Load Dose 1	09MAY2000			No								

*Popn=Population (I=Intent-To-Treat, PP=Per Protocol)
**Rel Day=Number of days relative to start of prophylactic medication
Symp = Symptoms, Rd = Reader, Res = Result, Spec = Species, Diff = Difference
Spec Count W = Species Count recorded per 500 White Blood Cells
Spec Count B = Species Count converted to per microlitre of blood
Gam = Gametocyte
+Arb=Arbitrator, +++Pos=Positive, Neg=Negative
#P. f = P.falciparum, P. v = P.vivax, P. o = P.ovale, P. m = P.malariae
Tafenoquine = Tafenoquine 200mg, Mefloquine = Mefloquine 250mg

Listing C.01

Results Of Blood Smears By Visit

Treatment : Placebo

Subject Number	Popn *	Visit	Visit Date	Sample Date	Rel Day**	Symp ?	Rd+	Res +++	Spec #	Spec Count W	Spec Count B	Mean	Diff	Gam Count
030.001.00191	IPP	Load Dose 2	10MAY2000			No								
		Load Dose 3	11MAY2000			No								
		Week 1	17MAY2000	17MAY2000	8	No	1	Neg						
		Week 1	17MAY2000	17MAY2000		No	2	Neg						
		Week 2	25MAY2000	25MAY2000	16	No	1	Neg						
		Week 2	25MAY2000	25MAY2000		No	2	Neg						
		Week 3	01JUN2000	01JUN2000	23	No	1	Neg						
		Week 3	01JUN2000	01JUN2000		No	2	Neg						
		Week 4	09JUN2000	09JUN2000	31	No	1	Neg						
		Week 4	09JUN2000	09JUN2000		No	2	Neg						
		Week 5	15JUN2000	15JUN2000	37	No	1	Pos	P.f	0.8	10.7	58.2	-94.9	
		Week 5	15JUN2000	15JUN2000		No	2	Pos	P.f	7.8	105.7			
		Week 6 (Fol. Up)	21JUN2000	21JUN2000	43	No	1	Neg						
		Week 6 (Fol. Up)	21JUN2000	21JUN2000		No	2	Neg						
		Week 7 (Fol. Up)	28JUN2000	28JUN2000	50	No	1	Neg						
		Week 7 (Fol. Up)	28JUN2000	28JUN2000		No	2	Neg						
		Week 8 (Fol. Up)	04JUL2000	04JUL2000	56	No	1	Neg						
		Week 8 (Fol. Up)	04JUL2000	04JUL2000		No	2	Neg						
		Week 8 (Unsch. Fol. Up)	07JUL2000			Yes								
		Week 9 (Fol. Up)	12JUL2000	12JUL2000	64	No	1	Neg						
		Week 9 (Fol. Up)	12JUL2000	12JUL2000		No	2	Neg						
030.001.00192	IPP	Screening	11APR2000	11APR2000	-28		1	Pos	P.f	0.2	2.5	7.9	-10.7	
		Screening	11APR2000	11APR2000			2	Pos	P.f	1.3	13.2			
		Screening (Unsch.)	14APR2000	14APR2000	-25		1	Pos	P.f	256.9	2672.0	4162.0	-2980.1	
		Screening (Unsch.)	14APR2000	14APR2000			2	Pos	P.f	543.5	5652.1			
		Post Clearance	07MAY2000	07MAY2000	-2		1	Neg						
		Post Clearance	07MAY2000	07MAY2000			2	Neg						
		Load Dose 1	09MAY2000			No								
		Load Dose 2	10MAY2000			No								
		Load Dose 3	11MAY2000			No								
		Week 1	17MAY2000	17MAY2000	8	No	1	Neg						
		Week 1	17MAY2000	17MAY2000		No	2	Neg						
		Week 2	25MAY2000	25MAY2000	16	Yes	1	Neg						
		Week 2	25MAY2000	25MAY2000		Yes	2	Neg						
		Week 3	31MAY2000	31MAY2000	22	No	1	Pos	P.f	1.7	19.7	23.4	-7.3	
		Week 3	31MAY2000	31MAY2000		No	2	Pos	P.f	2.3	27.0			

*Popn=Population (I=Intent-To-Treat, PP=Per Protocol)
**Rel Day=Number of days relative to start of prophylactic medication
Symp = Symptoms, Rd = Reader, Res = Result, Spec = Species, Diff = Difference
Spec Count W = Species Count recorded per 500 White Blood Cells
Spec Count B = Species Count converted to per microlitre of blood
Gam = Gametocyte
+Arb=Arbitrator, +++Pos=Positive, Neg=Negative
#P. f = P.falciparum, P. v = P.vivax, P. o = P.ovale, P. m = P.malariae
Tafenoquine = Tafenoquine 200mg, Mefloquine = Mefloquine 250mg

Listing C.01

Results Of Blood Smears By Visit

Treatment : Placebo

Subject Number	Popn *	Visit	Visit Date	Sample Date	Rel Day**	Symp ?	Rd+	Res +++	Spec #	Spec Count W	Spec Count B	Mean	Diff	Gam Count
030.001.00192	IPP	Week 4 (Fol. Up)	06JUN2000	06JUN2000	28		1	Neg						
		Week 4 (Fol. Up)	06JUN2000	06JUN2000			2	Neg						
		Week 4 (Fol. Up)	06JUN2000	06JUN2000		No		Neg						
		Week 6 (Fol. Up)	17JUN2000	17JUN2000	39		1	Neg						
		Week 6 (Fol. Up)	17JUN2000	17JUN2000			2	Neg						
		Week 6 (Fol. Up)	17JUN2000	17JUN2000		No		Neg						
		Week 6 (Unsch. Fol. Up)	19JUN2000			Yes								
		Week 6 (Fol. Up)	21JUN2000	21JUN2000	43		1	Neg						
		Week 6 (Fol. Up)	21JUN2000	21JUN2000			2	Neg						
		Week 6 (Fol. Up)	21JUN2000	21JUN2000		No		Neg						
		Week 7 (Fol. Up)	28JUN2000	28JUN2000	50		1	Neg						
		Week 7 (Fol. Up)	28JUN2000	28JUN2000			2	Neg						
		Week 7 (Fol. Up)	29JUN2000			No								
030.001.00193	IPP	Screening	14APR2000	14APR2000	-25		1	Pos	P.f	256.9	3083.0	4802.3	-3438.6	
		Screening	14APR2000	14APR2000			2	Pos	P.f	543.5	6521.6			
		Screening (Unsch.)	01MAY2000	01MAY2000	-8		1	Neg						
		Screening (Unsch.)	01MAY2000	01MAY2000			2	Neg						
		Post Clearance	07MAY2000	07MAY2000	-2		1	Neg						
		Post Clearance	07MAY2000	07MAY2000			2	Neg						
		Post Clearance (Unsch.)	08MAY2000			No								
		Load Dose 1	09MAY2000			No								
		Load Dose 2	10MAY2000			No								
		Load Dose 3	11MAY2000			No								
		Week 1 (Unsch.)	14MAY2000			No								
		Week 1	17MAY2000	17MAY2000	8	No	1	Neg						
		Week 1	17MAY2000	17MAY2000		No	2	Neg						
		Week 2 (Unsch.)	20MAY2000			Yes								
		Week 2	24MAY2000	24MAY2000	15	No	1	Neg						
		Week 2	24MAY2000	24MAY2000		No	2	Neg						
		Week 3	31MAY2000	31MAY2000	22	Yes	1	Neg						
		Week 3	31MAY2000	31MAY2000		Yes	2	Pos	P.f	0.1	1.3	1.1	0.3	
		Week 3	31MAY2000	31MAY2000		Yes	Arb	Pos	P.f	0.1	1.0			
		Week 4 (Fol. Up)	07JUN2000	07JUN2000	29	No	1	Neg						
		Week 4 (Fol. Up)	07JUN2000	07JUN2000		No	2	Neg						
		Week 5 (Fol. Up)	15JUN2000	15JUN2000	37	Yes	1	Neg						
		Week 5 (Fol. Up)	15JUN2000	15JUN2000		Yes	2	Neg						

*Popn=Population (I=Intent-To-Treat, PP=Per Protocol)
**Rel Day=Number of days relative to start of prophylactic medication
Symp = Symptoms, Rd = Reader, Res = Result, Spec = Species, Diff = Difference
Spec Count W = Species Count recorded per 500 White Blood Cells
Spec Count B = Species Count converted to per microlitre of blood
Gam = Gametocyte
+Arb=Arbitrator, +++Pos=Positive, Neg=Negative
#P. f = P.falciparum, P. v = P.vivax, P. o = P.ovale, P. m = P.malariae
Tafenoquine = Tafenoquine 200mg, Mefloquine = Mefloquine 250mg

SB252263 - 030

Listing C.01

Results Of Blood Smears By Visit

Treatment : Placebo

Subject Number	Popn *	Visit	Visit Date	Sample Date	Rel Day**	Symp ?	Rd+	Res +++	Spec #	Spec Count W	Spec Count B	Mean	Diff	Gam Count
030.001.00193	IPP	Week 6 (Fol. Up)	21JUN2000	21JUN2000	43	No	1	Neg						
		Week 6 (Fol. Up)	21JUN2000	21JUN2000		No	2	Neg						
030.001.00197	IPP	Screening	17APR2000	17APR2000	-22		1	Pos	P.f	154.5	1451.8	1484.3	-64.9	
		Screening	17APR2000	17APR2000			2	Pos	P.f	161.4	1516.7			
		Post Clearance	07MAY2000	07MAY2000	-2		1	Neg						
		Post Clearance	07MAY2000	07MAY2000			2	Neg						
		Load Dose 1	09MAY2000			No								
		Load Dose 2	10MAY2000			No								
		Load Dose 3	11MAY2000			No								
		Week 1 (Unsch.)	15MAY2000			Yes								
		Week 1	17MAY2000	17MAY2000	8	No	1	Neg						
		Week 1	17MAY2000	17MAY2000		No	2	Neg						
		Week 2	24MAY2000	24MAY2000	15	No	1	Neg						
		Week 2	24MAY2000	24MAY2000		No	2	Neg						
		Week 3	31MAY2000	31MAY2000	22	No	1	Pos	P.f	4.8	57.0	34.6	44.7	
		Week 3	31MAY2000	31MAY2000		No	2	Pos	P.f	1.0	12.3			
		Week 4 (Fol. Up)	06JUN2000	06JUN2000	28	No	1	Neg						
		Week 4 (Fol. Up)	06JUN2000	06JUN2000		No	2	Neg						
		Week 5 (Fol. Up)	14JUN2000	14JUN2000	36	No	1	Neg						
		Week 5 (Fol. Up)	14JUN2000	14JUN2000		No	2	Neg						
		Week 6 (Fol. Up)	21JUN2000	21JUN2000	43	No	1	Pos	P.f	13.2	136.8	69.1	135.3	
		Week 6 (Fol. Up)	21JUN2000	21JUN2000		No	2	Pos	P.f	0.1	1.5			
		Week 7 (Fol. Up)	28JUN2000	28JUN2000	50	Yes	1	Pos	P.f	0.6	6.7	4.5	4.4	
		Week 7 (Fol. Up)	28JUN2000	28JUN2000		Yes	2	Pos	P.f	0.2	2.3			
030.001.00202		Screening	17APR2000	17APR2000	-22		1	Pos	P.f	54.4	577.1	640.5	-126.8	
		Screening	17APR2000	17APR2000			1		P.m	2.0	21.1	24.3	-6.5	
		Screening	17APR2000	17APR2000			2	Pos	P.f	66.4	703.8			
		Screening	17APR2000	17APR2000			2		P.m	2.6	27.6			
		Post Clearance	07MAY2000	07MAY2000	-2		1	Neg						
		Post Clearance	07MAY2000	07MAY2000			2	Neg						
		Load Dose 1	09MAY2000			Yes								
		Load Dose 2	10MAY2000			No								
		Load Dose 3	11MAY2000			No								
		Week 1 (Unsch. Fol. Up)	13MAY2000			Yes								
		Week 1 (Fol. Up)	17MAY2000			No								

*Popn=Population (I=Intent-To-Treat, PP=Per Protocol)
**Rel Day=Number of days relative to start of prophylactic medication
Symp = Symptoms, Rd = Reader, Res = Result, Spec = Species, Diff = Difference
Spec Count W = Species Count recorded per 500 White Blood Cells
Spec Count B = Species Count converted to per microlitre of blood
Gam = Gametocyte
+Arb=Arbitrator, +++Pos=Positive, Neg=Negative
#P. f = P.falciparum, P. v = P.vivax, P. o = P.ovale, P. m = P.malariae
Tafenoquine = Tafenoquine 200mg, Mefloquine = Mefloquine 250mg

SB252263 - 030

Listing C.01

Results Of Blood Smears By Visit

Treatment : Placebo

Subject Number	Popn *	Visit	Visit Date	Sample Date	Rel Day**	Symp ?	Rd+	Res +++	Spec #	Spec Count W	Spec Count B	Mean	Diff	Gam Count
030.001.00202		Week 2 (Fol. Up)	24MAY2000			No								
030.001.00204	I	Screening	14APR2000	14APR2000	-25		1	Neg						
		Screening	14APR2000	14APR2000			2	Neg						
		Post Clearance	07MAY2000	07MAY2000	-2		1	Neg						
		Post Clearance	07MAY2000	07MAY2000			2	Neg						
		Load Dose 1	09MAY2000			No								
		Load Dose 2	10MAY2000			No								
		Load Dose 3	11MAY2000			No								
		Week 1	17MAY2000	17MAY2000	8	No	1	Neg						
		Week 1	17MAY2000	17MAY2000		No	2	Neg						
		Week 2	24MAY2000	24MAY2000	15	No	1	Neg						
		Week 2	24MAY2000	24MAY2000		No	2	Neg						
		Week 3	31MAY2000	31MAY2000	22	No	1	Neg						
		Week 3	31MAY2000	31MAY2000		No	2	Neg						
		Week 4	05JUN2000	05JUN2000	27	No	1	Neg						
		Week 4	05JUN2000	05JUN2000		No	2	Neg						
		Week 5 (Fol. Up)	14JUN2000			No								
		Week 6 (Fol. Up)	21JUN2000			No								
		Week 7 (Fol. Up)	28JUN2000	28JUN2000	50	No	1	Neg						
		Week 7 (Fol. Up)	28JUN2000	28JUN2000		No	2	Neg						
		Week 8 (Fol. Up)	04JUL2000	04JUL2000	56	No	1	Neg						
		Week 8 (Fol. Up)	04JUL2000	04JUL2000		No	2	Neg						
030.001.00208	IPP	Screening	11APR2000	11APR2000	-28		1	Pos	P.f	96.5	1197.0	1057.0	280.0	
		Screening	11APR2000	11APR2000			2	Pos	P.f	74.0	917.0			
		Post Clearance	07MAY2000	07MAY2000	-2		1	Neg						
		Post Clearance	07MAY2000	07MAY2000			2	Neg						
		Load Dose 1	09MAY2000			No								
		Load Dose 2	10MAY2000			No								
		Load Dose 3	11MAY2000			No								
		Load Dose (Unsch.)	11MAY2000			Yes								
		Week 1	17MAY2000	17MAY2000	8	No	1	Neg						
		Week 1	17MAY2000	17MAY2000		No	2	Neg						
		Week 2	24MAY2000	24MAY2000	15	No	1	Neg						
		Week 2	24MAY2000	24MAY2000		No	2	Neg						
		Week 3 (Unsch.)	29MAY2000			No								

*Popn=Population (I=Intent-To-Treat, PP=Per Protocol)
**Rel Day=Number of days relative to start of prophylactic medication
Symp = Symptoms, Rd = Reader, Res = Result, Spec = Species, Diff = Difference
Spec Count W = Species Count recorded per 500 White Blood Cells
Spec Count B = Species Count converted to per microlitre of blood
Gam = Gametocyte
+Arb=Arbitrator, +++Pos=Positive, Neg=Negative
#P. f = P.falciparum, P. v = P.vivax, P. o = P.ovale, P. m = P.malariae
Tafenoquine = Tafenoquine 200mg, Mefloquine = Mefloquine 250mg

SB252263 - 030

Listing C.01

Results Of Blood Smears By Visit

Treatment : Placebo

Subject Number	Popn *	Visit	Visit Date	Sample Date	Rel Day**	Symp ?	Rd+	Res +++	Spec #	Spec Count W	Spec Count B	Mean	Diff	Gam Count
030.001.00208	IPP	Week 3	31MAY2000	31MAY2000	22	No	1	Neg						
		Week 3	31MAY2000	31MAY2000		No	2	Neg						
		Week 4	07JUN2000	07JUN2000	29	No	1	Neg						
		Week 4	07JUN2000	07JUN2000		No	2	Neg						
		Week 5	14JUN2000	14JUN2000	36	No	1	Neg						
		Week 5	14JUN2000	14JUN2000		No	2	Neg						
		Week 6	21JUN2000	21JUN2000	43	No	1	Neg						
		Week 6	21JUN2000	21JUN2000		No	2	Neg						
		Week 7	28JUN2000	28JUN2000	50	No	1	Neg						
		Week 7	28JUN2000	28JUN2000		No	2	Neg						
		Week 8	04JUL2000	04JUL2000	56	No	1	Neg						
		Week 8	04JUL2000	04JUL2000		No	2	Neg						
		Week 9	12JUL2000	12JUL2000	64	No	1	Neg						
		Week 9	12JUL2000	12JUL2000		No	2	Neg						
		Week 10	19JUL2000	19JUL2000	71	No	1	Neg						
		Week 10	19JUL2000	19JUL2000		No	2	Neg						
		Week 11	26JUL2000	26JUL2000	78	No	1	Neg						
		Week 11	26JUL2000	26JUL2000		No	2	Neg						
		Week 12	02AUG2000	02AUG2000	85	No	1	Pos	P.f	2.6	36.8	33.5	6.7	
		Week 12	02AUG2000	02AUG2000		No	2	Neg						
		Week 12	02AUG2000	02AUG2000		No	Arb	Pos	P.f	2.2	30.1			
		Week 13 (Fol. Up)	10AUG2000	10AUG2000	93	No	1	Neg						
		Week 13 (Fol. Up)	10AUG2000	10AUG2000		No	2	Neg						
		Week 14 (Fol. Up)	17AUG2000	17AUG2000	100	Yes	1	Pos	P.f	4.5	62.7	59.4	6.7	
		Week 14 (Fol. Up)	17AUG2000	17AUG2000		Yes	2	Pos	P.f	4.0	56.0			
		Week 15 (Fol. Up)	23AUG2000	23AUG2000	106	No	1	Neg						
		Week 15 (Fol. Up)	23AUG2000	23AUG2000		No	2	Pos	P.f	5.9	82.9	52.1	61.6	
		Week 15 (Fol. Up)	23AUG2000	23AUG2000		No	Arb	Pos	P.f	1.5	21.3			
		Week 16 (Fol. Up)	28AUG2000	28AUG2000	111	No	1	Neg						
		Week 16 (Fol. Up)	28AUG2000	28AUG2000		No	2	Neg						
030.001.00211	IPP	Screening	11APR2000	11APR2000	-28		1	Neg						
		Screening	11APR2000	11APR2000			2	Neg						
		Post Clearance	07MAY2000	07MAY2000	-2		1	Neg						
		Post Clearance	07MAY2000	07MAY2000			2	Neg						
		Load Dose 1	09MAY2000			No								
		Load Dose 2	10MAY2000			No								

*Popn=Population (I=Intent-To-Treat, PP=Per Protocol)
**Rel Day=Number of days relative to start of prophylactic medication
Symp = Symptoms, Rd = Reader, Res = Result, Spec = Species, Diff = Difference
Spec Count W = Species Count recorded per 500 White Blood Cells
Spec Count B = Species Count converted to per microlitre of blood
Gam = Gametocyte
+Arb=Arbitrator, +++Pos=Positive, Neg=Negative
#P. f = P.falciparum, P. v = P.vivax, P. o = P.ovale, P. m = P.malariae
Tafenoquine = Tafenoquine 200mg, Mefloquine = Mefloquine 250mg

SB252263 - 030

Listing C.01

Results Of Blood Smears By Visit

Treatment : Placebo

Subject Number	Popn *	Visit	Visit Date	Sample Date	Rel Day**	Symp ?	Rd+	Res +++	Spec #	Spec Count W	Spec Count B	Mean	Diff	Gam Count
030.001.00211	IPP	Load Dose 3	11MAY2000			No								
		Week 1	17MAY2000	17MAY2000	8	No	1	Neg						
		Week 1	17MAY2000	17MAY2000		No	2	Neg						
		Week 1 (Unsch.)	18MAY2000			Yes								
		Week 2	24MAY2000	24MAY2000	15	No	1	Neg						
		Week 2	24MAY2000	24MAY2000		No	2	Neg						
		Week 2 (Unsch.)	26MAY2000			Yes								
		Week 3	31MAY2000	31MAY2000	22	No	1	Neg						
		Week 3	31MAY2000	31MAY2000		No	2	Neg						
		Week 4 (Unsch.)	04JUN2000			Yes								
		Week 4	06JUN2000	06JUN2000	28	Yes	1	Neg						
		Week 4	06JUN2000	06JUN2000		Yes	2	Neg						
		Week 5	14JUN2000	14JUN2000	36	No	1	Neg						
		Week 5	14JUN2000	14JUN2000		No	2	Neg						
		Week 6	21JUN2000	21JUN2000	43	Yes	1	Pos	P.f	0.4	5.4	5.8	-0.9	
		Week 6	21JUN2000	21JUN2000		Yes	2	Pos	P.f	0.5	6.3			
		Week 6 (Unsch.)	21JUN2000			No								
		Week 7 (Fol. Up)	28JUN2000	28JUN2000	50	No	1	Pos	P.f	119.8	1533.4	2516.4	-1965.8	
		Week 7 (Fol. Up)	28JUN2000	28JUN2000		No	2	Pos	P.f	273.4	3499.3			
		Week 7 (Unsch. Fol. Up)	29JUN2000			Yes								
		Week 9 (Fol. Up)	09JUL2000	09JUL2000	61	No	1	Neg						
		Week 9 (Fol. Up)	09JUL2000	09JUL2000		No	2	Neg						
		Week 9 (Fol. Up)	12JUL2000	12JUL2000	64	No	1	Neg						
		Week 9 (Fol. Up)	12JUL2000	12JUL2000		No	2	Neg						
		Week 10 (Fol. Up)	19JUL2000	19JUL2000	71		1	Neg						
		Week 10 (Fol. Up)	19JUL2000	19JUL2000			2	Neg						
030.001.00212	IPP	Screening	13APR2000	13APR2000	-26		1	Pos	P.f	0.3	3.7	4.3	-1.2	
		Screening	13APR2000	13APR2000			2	Pos	P.f	0.4	4.9			
		Post Clearance	07MAY2000	07MAY2000	-2		1	Neg						
		Post Clearance	07MAY2000	07MAY2000			2	Neg						
		Load Dose 1	09MAY2000			No								
		Load Dose 2	10MAY2000			No								
		Load Dose 3	11MAY2000			No								
		Week 1	17MAY2000	17MAY2000	8	No	1	Neg						
		Week 1	17MAY2000	17MAY2000		No	2	Neg						
		Week 2	24MAY2000	24MAY2000	15	Yes	1	Neg						

*Popn=Population (I=Intent-To-Treat, PP=Per Protocol)
**Rel Day=Number of days relative to start of prophylactic medication
Symp = Symptoms, Rd = Reader, Res = Result, Spec = Species, Diff = Difference
Spec Count W = Species Count recorded per 500 White Blood Cells
Spec Count B = Species Count converted to per microlitre of blood
Gam = Gametocyte
+Arb=Arbitrator, +++Pos=Positive, Neg=Negative
#P. f = P.falciparum, P. v = P.vivax, P. o = P.ovale, P. m = P.malariae
Tafenoquine = Tafenoquine 200mg, Mefloquine = Mefloquine 250mg

Listing C.01

Results Of Blood Smears By Visit

Treatment : Placebo

Subject Number	Popn *	Visit	Visit Date	Sample Date	Rel Day**	Symp ?	Rd+	Res +++	Spec #	Spec Count W	Spec Count B	Mean	Diff	Gam Count
030.001.00212	IPP	Week 2	24MAY2000	24MAY2000		Yes	2	Neg						
		Week 3	31MAY2000	31MAY2000	22	No	1	Neg						
		Week 3	31MAY2000	31MAY2000		No	2	Neg						
		Week 4	07JUN2000	07JUN2000	29	No	1	Neg						
		Week 4	07JUN2000	07JUN2000		No	2	Neg						
		Week 5 (Unsch.)	13JUN2000			No								
		Week 5	14JUN2000	14JUN2000	36	No	1	Neg						
		Week 5	14JUN2000	14JUN2000		No	2	Neg						
		Week 6 (Unsch.)	18JUN2000			No								
		Week 6 (Unsch.)	20JUN2000			No								
		Week 6	21JUN2000	21JUN2000	43	No	1	Neg						
		Week 6	21JUN2000	21JUN2000		No	2	Neg						
		Week 7	28JUN2000	28JUN2000	50	No	1	Pos	P.f	3.8	39.9	23.2	33.5	
		Week 7	28JUN2000	28JUN2000		No	2	Neg						
		Week 7	28JUN2000	28JUN2000		No	Arb	Pos	P.f	0.6	6.4			
		Week 7 (Unsch. Fol. Up)	30JUN2000			No								
		Week 8 (Fol. Up)	04JUL2000	04JUL2000	56	No	1	Neg						
		Week 8 (Fol. Up)	04JUL2000	04JUL2000		No	2	Neg						
		Week 9 (Fol. Up)	12JUL2000	12JUL2000	64	No	1	Neg						
		Week 9 (Fol. Up)	12JUL2000	12JUL2000		No	2	Neg						
		Week 10 (Fol. Up)	20JUL2000	20JUL2000	72	No	1	Neg						
		Week 10 (Fol. Up)	20JUL2000	20JUL2000		No	2	Neg						
		Week 11 (Fol. Up)	26JUL2000	26JUL2000	78	No	1	Neg						
		Week 11 (Fol. Up)	26JUL2000	26JUL2000		No	2	Neg						
030.001.00215	IPP	Screening	12APR2000	12APR2000	-27		1	Pos	P.f	2.2	23.9	14.0	19.7	
		Screening	12APR2000	12APR2000			2	Pos	P.f	0.4	4.2			
		Post Clearance	07MAY2000	07MAY2000	-2		1	Neg						
		Post Clearance	07MAY2000	07MAY2000			2	Neg						
		Load Dose 1	09MAY2000			No								
		Load Dose 2	10MAY2000			No								
		Load Dose 3	11MAY2000			Yes								
		Week 1 (Unsch.)	12MAY2000	12MAY2000	3	Yes	1	Neg						
		Week 1 (Unsch.)	12MAY2000	12MAY2000		Yes	2	Neg						
		Week 1 (Unsch.)	14MAY2000			Yes								
		Week 1 (Unsch.)	15MAY2000			Yes								
		Week 1	17MAY2000	17MAY2000	8	No	1	Neg						

*Popn=Population (I=Intent-To-Treat, PP=Per Protocol)
**Rel Day=Number of days relative to start of prophylactic medication
Symp = Symptoms, Rd = Reader, Res = Result, Spec = Species, Diff = Difference
Spec Count W = Species Count recorded per 500 White Blood Cells
Spec Count B = Species Count converted to per microlitre of blood
Gam = Gametocyte
+Arb=Arbitrator, +++Pos=Positive, Neg=Negative
#P. f = P.falciparum, P. v = P.vivax, P. o = P.ovale, P. m = P.malariae
Tafenoquine = Tafenoquine 200mg, Mefloquine = Mefloquine 250mg

SB252263 - 030

Listing C.01

Results Of Blood Smears By Visit

Treatment : Placebo

Subject Number	Popn *	Visit	Visit Date	Sample Date	Rel Day**	Symp ?	Rd+	Res +++	Spec #	Spec Count W	Spec Count B	Mean	Diff	Gam Count
030.001.00215	IPP	Week 1	17MAY2000	17MAY2000		No	2	Neg						
		Week 2	24MAY2000	24MAY2000	15	No	1	Neg						
		Week 2	24MAY2000	24MAY2000		No	2	Neg						
		Week 3	31MAY2000	31MAY2000	22	No	1	Neg						
		Week 3	31MAY2000	31MAY2000		No	2	Neg						
		Week 4	06JUN2000	06JUN2000	28	No	1	Neg						
		Week 4	06JUN2000	06JUN2000		No	2	Neg						
		Week 5	14JUN2000	14JUN2000	36	No	1	Pos	P.f	2.4	35.6	59.9	-48.6	
		Week 5	14JUN2000	14JUN2000		No	2	Neg						
		Week 5	14JUN2000	14JUN2000		No	Arb	Pos	P.f	5.6	84.2			
		Week 7 (Fol. Up)	27JUN2000	27JUN2000	49	No	1	Pos	P.f	5.8	86.7	64.0	45.5	
		Week 7 (Fol. Up)	27JUN2000	27JUN2000		No	2	Neg						
		Week 7 (Fol. Up)	27JUN2000	27JUN2000		No	Arb	Pos	P.f	2.8	41.3			
		Week 8 (Fol. Up)	04JUL2000	04JUL2000	56	No	1	Neg						
		Week 8 (Fol. Up)	04JUL2000	04JUL2000		No	2	Neg						
		Week 9 (Fol. Up)	10JUL2000	10JUL2000	62	No	1	Neg						
		Week 9 (Fol. Up)	10JUL2000	10JUL2000		No	2	Pos	P.f	0.8	9.4	13.8	-8.7	
		Week 9 (Fol. Up)	10JUL2000	10JUL2000		No	Arb	Pos	P.f	1.6	18.1			
030.001.00219	IPP	Screening	12APR2000	12APR2000	-27		1	Neg						
		Screening	12APR2000	12APR2000			2	Pos	P.f	1.0	6.4			
		Screening	12APR2000	12APR2000			Arb	Neg						
		Post Clearance	07MAY2000	07MAY2000	-2		1	Neg						
		Post Clearance	07MAY2000	07MAY2000			2	Neg						
		Load Dose 1	09MAY2000			No								
		Load Dose 2	10MAY2000			No								
		Load Dose 3	11MAY2000			No								
		Week 1	17MAY2000	17MAY2000	8	No	1	Neg						
		Week 1	17MAY2000	17MAY2000		No	2	Neg						
		Week 2	24MAY2000	24MAY2000	15	No	1	Neg						
		Week 2	24MAY2000	24MAY2000		No	2	Neg						
		Week 3	31MAY2000	31MAY2000	22	No	1	Neg						
		Week 3	31MAY2000	31MAY2000		No	2	Neg						
		Week 4	06JUN2000	06JUN2000	28	No	1	Neg						
		Week 4	06JUN2000	06JUN2000		No	2	Neg						
		Week 5	14JUN2000	14JUN2000	36	No	1	Neg						
		Week 5	14JUN2000	14JUN2000		No	2	Pos	P.f	3.3	19.3	18.3	2.0	

*Popn=Population (I=Intent-To-Treat, PP=Per Protocol)
**Rel Day=Number of days relative to start of prophylactic medication
Symp = Symptoms, Rd = Reader, Res = Result, Spec = Species, Diff = Difference
Spec Count W = Species Count recorded per 500 White Blood Cells
Spec Count B = Species Count converted to per microlitre of blood
Gam = Gametocyte
+Arb=Arbitrator, +++Pos=Positive, Neg=Negative
#P. f = P.falciparum, P. v = P.vivax, P. o = P.ovale, P. m = P.malariae
Tafenoquine = Tafenoquine 200mg, Mefloquine = Mefloquine 250mg

SB252263 - 030

Listing C.01

Results Of Blood Smears By Visit

Treatment : Placebo

Subject Number	Popn *	Visit	Visit Date	Sample Date	Rel Day**	Symp ?	Rd+	Res +++	Spec #	Spec Count W	Spec Count B	Mean	Diff	Gam Count
030.001.00219	IPP	Week 5	14JUN2000	14JUN2000		No	Arb	Pos	P.f	3.0	17.3			
		Week 6 (Fol. Up)	21JUN2000	21JUN2000	43	No	1	Pos	P.f	3.7	21.6	21.2	0.9	
		Week 6 (Fol. Up)	21JUN2000	21JUN2000		No	2	Pos	P.f	3.6	20.7			
		Week 7 (Fol. Up)	30JUN2000	30JUN2000	52	No	1	Neg						
		Week 7 (Fol. Up)	30JUN2000	30JUN2000		No	2	Neg						
		Week 9 (Fol. Up)	10JUL2000	10JUL2000	62	No	1	Neg						
		Week 9 (Fol. Up)	10JUL2000	10JUL2000		No	2	Neg						
030.001.00222	IPP	Screening	14APR2000	14APR2000	-25		1	Pos	P.f	56.0	627.2	855.7	-457.1	
		Screening	14APR2000	14APR2000			1		P.m	4.8	53.8	54.3	-1.1	
		Screening	14APR2000	14APR2000			2	Pos	P.f	96.8	1084.3			
		Screening	14APR2000	14APR2000			2		P.m	4.9	54.9			
		Post Clearance	07MAY2000	07MAY2000	-2		1	Neg						
		Post Clearance	07MAY2000	07MAY2000			2	Neg						
		Load Dose 1	09MAY2000			Yes								
		Load Dose 2	10MAY2000			No								
		Load Dose 3	11MAY2000			No								
		Week 1 (Unsch.)	15MAY2000	15MAY2000	6	Yes	1	Neg						
		Week 1 (Unsch.)	15MAY2000	15MAY2000		Yes	2	Neg						
		Week 1	17MAY2000			No								
		Week 2	24MAY2000	24MAY2000	15	No	1	Neg						
		Week 2	24MAY2000	24MAY2000		No	2	Neg						
		Week 3	31MAY2000	31MAY2000	22	No	1	Neg						
		Week 3	31MAY2000	31MAY2000		No	2	Neg						
		Week 4	06JUN2000	06JUN2000	28	Yes	1	Neg						
		Week 4	06JUN2000	06JUN2000		Yes	2	Neg						
		Week 5	14JUN2000	14JUN2000	36	No	1	Neg						
		Week 5	14JUN2000	14JUN2000		No	2	Neg						
		Week 6	21JUN2000	21JUN2000	43	No	1	Pos	P.f	2.8	45.1	50.3	-10.3	
		Week 6	21JUN2000	21JUN2000		No	2	Pos	P.f	3.4	55.4			
		Week 7 (Unsch. Fol. Up)	26JUN2000			Yes								
		Week 7 (Fol. Up)	28JUN2000	28JUN2000	50	No	1	Neg						
		Week 7 (Fol. Up)	28JUN2000	28JUN2000		No	2	Neg						
		Week 8 (Fol. Up)	04JUL2000	04JUL2000	56	No	1	Neg						
		Week 8 (Fol. Up)	04JUL2000	04JUL2000		No	2	Pos	P.f	0.5	8.4	11.6	-6.4	
		Week 8 (Fol. Up)	04JUL2000	04JUL2000		No	Arb	Pos	P.f	0.9	14.8			
		Week 9 (Fol. Up)	12JUL2000	12JUL2000	64	No	1	Pos	P.f	14.0	229.8	241.4	-23.3	

*Popn=Population (I=Intent-To-Treat, PP=Per Protocol)
**Rel Day=Number of days relative to start of prophylactic medication
Symp = Symptoms, Rd = Reader, Res = Result, Spec = Species, Diff = Difference
Spec Count W = Species Count recorded per 500 White Blood Cells
Spec Count B = Species Count converted to per microlitre of blood
Gam = Gametocyte
+Arb=Arbitrator, +++Pos=Positive, Neg=Negative
#P. f = P.falciparum, P. v = P.vivax, P. o = P.ovale, P. m = P.malariae
Tafenoquine = Tafenoquine 200mg, Mefloquine = Mefloquine 250mg

SB252263 - 030

Listing C.01

Results Of Blood Smears By Visit

Treatment : Placebo

Subject Number	Popn *	Visit	Visit Date	Sample Date	Rel Day**	Symp ?	Rd+	Res +++	Spec #	Spec Count W	Spec Count B	Mean	Diff	Gam Count
030.001.00222	IPP	Week 9 (Fol. Up)	12JUL2000	12JUL2000		No	2	Pos	P.f	15.4	253.1			
		Week 10 (Fol. Up)	19JUL2000	19JUL2000	71	No	1	Pos	P.f	2.8	45.4	54.4	-18.0	
		Week 10 (Fol. Up)	19JUL2000	19JUL2000		No	2	Pos	P.f	3.9	63.5			
030.001.00225	IPP	Screening	10APR2000	10APR2000	-29		1	Neg						
		Screening	10APR2000	10APR2000			2	Neg						
		Screening (Unsch.)	06MAY2000			Yes								
		Post Clearance	09MAY2000	09MAY2000	0		1	Neg						
		Post Clearance	09MAY2000	09MAY2000			2	Neg						
		Load Dose 1	09MAY2000			No								
		Load Dose 2	10MAY2000			No								
		Load Dose 3	11MAY2000			No								
		Week 1	17MAY2000	17MAY2000	8	No	1	Neg						
		Week 1	17MAY2000	17MAY2000		No	2	Neg						
		Week 2	24MAY2000	24MAY2000	15	No	1	Neg						
		Week 2	24MAY2000	24MAY2000		No	2	Neg						
		Week 3 (Unsch.)	27MAY2000			Yes								
		Week 3	31MAY2000	31MAY2000	22	Yes	1	Neg						
		Week 3	31MAY2000	31MAY2000		Yes	2	Neg						
		Week 4	06JUN2000	06JUN2000	28	Yes	1	Neg						
		Week 4	06JUN2000	06JUN2000		Yes	2	Neg						
		Week 5	14JUN2000	14JUN2000	36	Yes	1	Pos	P.f	1.3	25.5	36.1	-21.2	
		Week 5	14JUN2000	14JUN2000		Yes	2	Neg						
		Week 5	14JUN2000	14JUN2000		Yes	Arb	Pos	P.f	2.3	46.7			
		Week 6 (Unsch. Fol. Up)	21JUN2000			Yes								
		Week 6 (Fol. Up)	21JUN2000	21JUN2000	43	Yes	1	Pos	P.f	1.4	27.9	17.2	21.4	
		Week 6 (Fol. Up)	21JUN2000	21JUN2000		Yes	2	Pos	P.f	0.3	6.5			
		Week 6 (Unsch. Fol. Up)	21JUN2000			No								
		Week 7 (Unsch. Fol. Up)	26JUN2000			No								
		Week 7 (Unsch. Fol. Up)	26JUN2000			No								
		Week 7 (Fol. Up)	28JUN2000	28JUN2000	50	No	1	Neg						
		Week 7 (Fol. Up)	28JUN2000	28JUN2000		No	2	Pos	P.f	0.7	14.9	14.9	0.0	
		Week 7 (Fol. Up)	28JUN2000	28JUN2000		No	Arb	Pos	P.f	0.7	14.9			
		Week 8 (Fol. Up)	04JUL2000	04JUL2000	56	No	1	Neg						
		Week 8 (Fol. Up)	04JUL2000	04JUL2000		No	2	Neg						
		Week 9 (Unsch. Fol. Up)	12JUL2000	12JUL2000	64		1	Neg						
		Week 9 (Unsch. Fol. Up)	12JUL2000	12JUL2000			2	Neg						

*Popn=Population (I=Intent-To-Treat, PP=Per Protocol)
**Rel Day=Number of days relative to start of prophylactic medication
Symp = Symptoms, Rd = Reader, Res = Result, Spec = Species, Diff = Difference
Spec Count W = Species Count recorded per 500 White Blood Cells
Spec Count B = Species Count converted to per microlitre of blood
Gam = Gametocyte
+Arb=Arbitrator, +++Pos=Positive, Neg=Negative
#P. f = P.falciparum, P. v = P.vivax, P. o = P.ovale, P. m = P.malariae
Tafenoquine = Tafenoquine 200mg, Mefloquine = Mefloquine 250mg

Listing C.01

Results Of Blood Smears By Visit

Treatment : Placebo

Subject Number	Popn *	Visit	Visit Date	Sample Date	Rel Day**	Symp ?	Rd+	Res +++	Spec #	Spec Count W	Spec Count B	Mean	Diff	Gam Count
030.001.00225	IPP	Week 9 (Fol. Up)	12JUL2000	12JUL2000		Yes	1	Neg						
		Week 9 (Fol. Up)	12JUL2000	12JUL2000		Yes	2	Neg						
030.001.00226	IPP	Screening	13APR2000	13APR2000	-26		1	Pos	P.f	1.0	7.6	4.7	5.8	
		Screening	13APR2000	13APR2000			2	Neg						
		Screening	13APR2000	13APR2000			Arb	Pos	P.f	0.2	1.8			
		Post Clearance	07MAY2000	07MAY2000	-2		1	Neg						
		Post Clearance	07MAY2000	07MAY2000			2	Neg						
		Load Dose 1	09MAY2000			No								
		Load Dose 2	10MAY2000			No								
		Load Dose 3	11MAY2000			Yes								
		Week 1	17MAY2000	17MAY2000	8	No	1	Neg						
		Week 1	17MAY2000	17MAY2000		No	2	Neg						
		Week 1 (Unsch.)	18MAY2000			Yes								
		Week 2	24MAY2000	24MAY2000	15	Yes	1	Neg						
		Week 2	24MAY2000	24MAY2000		Yes	2	Neg						
		Week 3	31MAY2000	31MAY2000	22	Yes	1	Neg						
		Week 3	31MAY2000	31MAY2000		Yes	2	Neg						
		Week 4	08JUN2000	08JUN2000	30	No	1	Neg						
		Week 4	08JUN2000	08JUN2000		No	2	Neg						
		Week 5	14JUN2000	14JUN2000	36	Yes	1	Neg						
		Week 5	14JUN2000	14JUN2000		Yes	2	Neg						
		Week 5 (Unsch.)	16JUN2000			Yes								
		Week 6	21JUN2000	21JUN2000	43	Yes	1	Pos	P.f	1.8	15.0	9.1	11.8	
		Week 6	21JUN2000	21JUN2000		Yes	2	Pos	P.f	0.4	3.2			
		Week 7 (Fol. Up)	28JUN2000	28JUN2000	50	No	1	Neg						
		Week 7 (Fol. Up)	28JUN2000	28JUN2000		No	2	Neg						
		Week 8 (Fol. Up)	04JUL2000	04JUL2000	56	No	1	Neg						
		Week 8 (Fol. Up)	04JUL2000	04JUL2000		No	2	Neg						
		Week 9 (Unsch. Fol. Up)	10JUL2000	10JUL2000	62		1	Pos	P.f	670.9	13686.6	12520.6	2331.9	
		Week 9 (Unsch. Fol. Up)	10JUL2000	10JUL2000			2	Pos	P.f	556.6	11354.6			
		Week 9 (Unsch. Fol. Up)	10JUL2000			Yes								
		Week 9 (Fol. Up)	12JUL2000	12JUL2000	64	Yes	1	Neg						
		Week 9 (Fol. Up)	12JUL2000	12JUL2000		Yes	2	Neg						
		Week 10 (Fol. Up)	20JUL2000	20JUL2000	72	No	1	Neg						
		Week 10 (Fol. Up)	20JUL2000	20JUL2000		No	2	Neg						

*Popn=Population (I=Intent-To-Treat, PP=Per Protocol)
**Rel Day=Number of days relative to start of prophylactic medication
Symp = Symptoms, Rd = Reader, Res = Result, Spec = Species, Diff = Difference
Spec Count W = Species Count recorded per 500 White Blood Cells
Spec Count B = Species Count converted to per microlitre of blood
Gam = Gametocyte
+Arb=Arbitrator, +++Pos=Positive, Neg=Negative
#P. f = P.falciparum, P. v = P.vivax, P. o = P.ovale, P. m = P.malariae
Tafenoquine = Tafenoquine 200mg, Mefloquine = Mefloquine 250mg

SB252263 - 030

Listing C.01

Results Of Blood Smears By Visit

Treatment : Placebo

Subject Number	Popn *	Visit	Visit Date	Sample Date	Rel Day**	Symp ?	Rd+	Res +++	Spec #	Spec Count W	Spec Count B	Mean	Diff	Gam Count
030.001.00229	IPP	Screening	13APR2000	13APR2000	-26		1	Pos	P.f	644.1	7471.1	10654.0	-6365.8	
		Screening	13APR2000	13APR2000			2	Pos	P.f	1192.8	13836.9			
		Screening (Unsch.)	13APR2000	13APR2000			1	Pos	P.f	644.1	7471.1	10654.0	-6365.8	
		Screening (Unsch.)	13APR2000	13APR2000			2	Pos	P.f	1192.8	13836.9			
		Post Clearance	07MAY2000	07MAY2000	-2		1	Neg						
		Post Clearance	07MAY2000	07MAY2000			2	Neg						
		Load Dose 1	09MAY2000			No								
		Load Dose 2	10MAY2000			No								
		Load Dose 3	11MAY2000			No								
		Week 1	18MAY2000	18MAY2000	9	No	1	Neg						
		Week 1	18MAY2000	18MAY2000		No	2	Neg						
		Week 2	24MAY2000	24MAY2000	15	Yes	1	Neg						
		Week 2	24MAY2000	24MAY2000		Yes	2	Neg						
		Week 3 (Unsch.)	28MAY2000			Yes								
		Week 3	31MAY2000	31MAY2000	22	Yes	1	Neg						
		Week 3	31MAY2000	31MAY2000		Yes	2	Neg						
		Week 4	07JUN2000	07JUN2000	29	No	1	Neg						
		Week 4	07JUN2000	07JUN2000		No	2	Neg						
		Week 4 (Unsch.)	09JUN2000			No								
		Week 5	15JUN2000	15JUN2000	37	No	1	Neg						
		Week 5	15JUN2000	15JUN2000		No	2	Neg						
		Week 6	21JUN2000	21JUN2000	43	No	1	Pos	P.f	0.8	8.7	7.4	2.5	
		Week 6	21JUN2000	21JUN2000		No	2	Pos	P.f	0.6	6.1			
		Week 7 (Fol. Up)	28JUN2000	28JUN2000	50	No	1	Neg						
		Week 7 (Fol. Up)	28JUN2000	28JUN2000		No	2	Neg						
		Week 8 (Fol. Up)	05JUL2000	05JUL2000	57	No	1	Pos	P.f	4.8	50.5	50.9	-1.0	
		Week 8 (Fol. Up)	05JUL2000	05JUL2000		No	2	Pos	P.f	4.9	51.4			
		Week 9 (Fol. Up)	12JUL2000	12JUL2000	64	No	1	Neg						
		Week 9 (Fol. Up)	12JUL2000	12JUL2000		No	2	Pos	P.f	26.3	278.8	240.3	77.0	
		Week 9 (Fol. Up)	12JUL2000	12JUL2000		No	Arb	Pos	P.f	19.0	201.8			
		Week 10 (Fol. Up)	19JUL2000	19JUL2000	71	No	1	Pos	P.f	3580.2	37950.5	22043.7	31813.8	
		Week 10 (Fol. Up)	19JUL2000	19JUL2000		No	2	Pos	P.f	578.9	6136.8			
		Week 10 (Unsch. Fol. Up)	21JUL2000			Yes								
030.001.00234	IPP	Screening	11APR2000	11APR2000	-29		1	Neg						
		Screening	11APR2000	11APR2000			2	Pos	P.f	0.3	4.0	8.3	-8.7	
		Screening	11APR2000	11APR2000			Arb	Pos	P.f	1.0	12.7			

*Popn=Population (I=Intent-To-Treat, PP=Per Protocol)
**Rel Day=Number of days relative to start of prophylactic medication
Symp = Symptoms, Rd = Reader, Res = Result, Spec = Species, Diff = Difference
Spec Count W = Species Count recorded per 500 White Blood Cells
Spec Count B = Species Count converted to per microlitre of blood
Gam = Gametocyte
+Arb=Arbitrator, +++Pos=Positive, Neg=Negative
#P. f = P.falciparum, P. v = P.vivax, P. o = P.ovale, P. m = P.malariae
Tafenoquine = Tafenoquine 200mg, Mefloquine = Mefloquine 250mg

SB252263 - 030

Listing C.01

Results Of Blood Smears By Visit

Treatment : Placebo

Subject Number	Popn *	Visit	Visit Date	Sample Date	Rel Day**	Symp ?	Rd+	Res +++	Spec #	Spec Count W	Spec Count B	Mean	Diff	Gam Count
030.001.00234	IPP	Post Clearance	08MAY2000	08MAY2000	-2		1	Neg						
		Post Clearance	08MAY2000	08MAY2000			2	Neg						
		Load Dose 1	10MAY2000			No								
		Load Dose 2	11MAY2000			No								
		Load Dose 3	12MAY2000			No								
		Week 1 (Unsch.)	13MAY2000			Yes								
		Week 1	17MAY2000	17MAY2000	7	No	1	Neg						
		Week 1	17MAY2000	17MAY2000		No	2	Neg						
		Week 2	24MAY2000	24MAY2000	14	No	1	Neg						
		Week 2	24MAY2000	24MAY2000		No	2	Neg						
		Week 3	31MAY2000	31MAY2000	21	No	1	Neg						
		Week 3	31MAY2000	31MAY2000		No	2	Neg						
		Week 4	06JUN2000	06JUN2000	27	No	1	Neg						
		Week 4	06JUN2000	06JUN2000		No	2	Neg						
		Week 5	14JUN2000	14JUN2000	35	No	1	Pos	P.f	0.4	5.2	7.3	-4.1	
		Week 5	14JUN2000	14JUN2000		No	2	Neg						
		Week 5	14JUN2000	14JUN2000		No	Arb	Pos	P.f	0.7	9.3			
		Week 6 (Fol. Up)	21JUN2000	21JUN2000	42	No	1	Pos	P.f	2.2	28.2	17.5	21.4	
		Week 6 (Fol. Up)	21JUN2000	21JUN2000		No	2	Pos	P.f	0.5	6.8			
		Week 7 (Fol. Up)	28JUN2000	28JUN2000	49	No	1	Pos	P.f	18.6	238.6	121.0	235.3	
		Week 7 (Fol. Up)	28JUN2000	28JUN2000		No	2	Pos	P.f	0.3	3.3			
		Week 8 (Fol. Up)	05JUL2000	05JUL2000	56	No	1	Pos	P.f	1.9	24.8	77.7	-105.7	
		Week 8 (Fol. Up)	05JUL2000	05JUL2000		No	2	Pos	P.f	10.2	130.6			
		Week 9 (Fol. Up)	12JUL2000	12JUL2000	63		1	Pos	P.f	0.2	2.2	2.0	0.3	
		Week 9 (Fol. Up)	12JUL2000	12JUL2000			2	Neg						
		Week 9 (Fol. Up)	12JUL2000	12JUL2000			Arb	Pos	P.f	0.2	1.9			
030.001.00236	IPP	Screening	11APR2000	11APR2000	-30		1	Pos	P.f	5.4	49.6	68.6	-38.0	
		Screening	11APR2000	11APR2000			2	Pos	P.f	9.5	87.6			
		Post Clearance	07MAY2000	07MAY2000	-4		1	Neg						
		Post Clearance	07MAY2000	07MAY2000			2	Neg						
		Load Dose 1	11MAY2000			No								
		Load Dose 2	12MAY2000			No								
		Load Dose 3	13MAY2000			No								
		Week 1 (Unsch.)	14MAY2000			Yes								
		Week 1	18MAY2000	18MAY2000	7	No	1	Neg						
		Week 1	18MAY2000	18MAY2000		No	2	Neg						

*Popn=Population (I=Intent-To-Treat, PP=Per Protocol)
**Rel Day=Number of days relative to start of prophylactic medication
Symp = Symptoms, Rd = Reader, Res = Result, Spec = Species, Diff = Difference
Spec Count W = Species Count recorded per 500 White Blood Cells
Spec Count B = Species Count converted to per microlitre of blood
Gam = Gametocyte
+Arb=Arbitrator, +++Pos=Positive, Neg=Negative
#P. f = P.falciparum, P. v = P.vivax, P. o = P.ovale, P. m = P.malariae
Tafenoquine = Tafenoquine 200mg, Mefloquine = Mefloquine 250mg

SB252263 - 030

Listing C.01

Results Of Blood Smears By Visit

Treatment : Placebo

Subject Number	Popn *	Visit	Visit Date	Sample Date	Rel Day**	Symp ?	Rd+	Res +++	Spec #	Spec Count W	Spec Count B	Mean	Diff	Gam Count
030.001.00236	IPP	Week 2	24MAY2000	24MAY2000	13	No	1	Neg						
		Week 2	24MAY2000	24MAY2000		No	2	Neg						
		Week 3	01JUN2000	01JUN2000	21	No	1	Neg						
		Week 3	01JUN2000	01JUN2000		No	2	Neg						
		Week 4	09JUN2000	09JUN2000	29	No	1	Neg						
		Week 4	09JUN2000	09JUN2000		No	2	Neg						
		Week 5 (Unsch.)	12JUN2000			No								
		Week 5	14JUN2000	14JUN2000	34	No	1	Neg						
		Week 5	14JUN2000	14JUN2000		No	2	Neg						
		Week 6	21JUN2000	21JUN2000	41	No	1	Neg						
		Week 6	21JUN2000	21JUN2000		No	2	Neg						
		Week 7	30JUN2000	30JUN2000	50	No	1	Neg						
		Week 7	30JUN2000	30JUN2000		No	2	Neg						
		Week 8	04JUL2000	04JUL2000	54	No	1	Neg						
		Week 8	04JUL2000	04JUL2000		No	2	Neg						
		Week 9	12JUL2000	12JUL2000	62	No	1	Neg						
		Week 9	12JUL2000	12JUL2000		No	2	Pos	P.f	0.9	8.6	11.6	-6.1	
		Week 9	12JUL2000	12JUL2000		No	Arb	Pos	P.f	1.6	14.6			
		Week 10 (Fol. Up)	21JUL2000	21JUL2000	71	No	1	Pos	P.f	9.5	87.1	60.4	53.5	
		Week 10 (Fol. Up)	21JUL2000	21JUL2000		No	2	Pos	P.f	3.7	33.6			
		Week 11 (Unsch. Fol. Up)	24JUL2000			Yes								
		Week 11 (Fol. Up)	26JUL2000	26JUL2000	76	No	1	Pos	P.f	5.1	46.6	40.4	12.5	
		Week 11 (Fol. Up)	26JUL2000	26JUL2000		No	2	Pos	P.f	3.7	34.1			
		Week 12 (Fol. Up)	02AUG2000	02AUG2000	83	No	1	Pos	P.f	145.8	1341.6	1188.8	305.6	
		Week 12 (Fol. Up)	02AUG2000	02AUG2000			1		P.o	1.4	12.7	27.0	-28.7	
		Week 12 (Fol. Up)	02AUG2000	02AUG2000		No	2	Pos	P.f	112.6	1036.0			
		Week 12 (Fol. Up)	02AUG2000	02AUG2000			2		P.o	4.5	41.4			
		Week 12 (Unsch. Fol. Up)	03AUG2000			Yes								
		Week 13 (Fol. Up)	09AUG2000	09AUG2000	90		1	Pos	P.o	13.0	119.4	80.1	78.7	
		Week 13 (Fol. Up)	09AUG2000	09AUG2000			2	Pos	P.o	4.4	40.8			
030.001.00238	IPP	Screening	12APR2000	12APR2000	-28		1	Pos	P.f	10.4	101.5	61.9	79.3	
		Screening	12APR2000	12APR2000			2	Pos	P.f	2.3	22.2			
		Screening (Unsch.)	25APR2000	25APR2000	-15		1	Neg						
		Screening (Unsch.)	25APR2000	25APR2000			2	Neg						
		Post Clearance	08MAY2000	08MAY2000	-2		1	Neg						
		Post Clearance	08MAY2000	08MAY2000			2	Neg						

*Popn=Population (I=Intent-To-Treat, PP=Per Protocol)
**Rel Day=Number of days relative to start of prophylactic medication
Symp = Symptoms, Rd = Reader, Res = Result, Spec = Species, Diff = Difference
Spec Count W = Species Count recorded per 500 White Blood Cells
Spec Count B = Species Count converted to per microlitre of blood
Gam = Gametocyte
+Arb=Arbitrator, +++Pos=Positive, Neg=Negative
#P. f = P.falciparum, P. v = P.vivax, P. o = P.ovale, P. m = P.malariae
Tafenoquine = Tafenoquine 200mg, Mefloquine = Mefloquine 250mg

SB252263 - 030

Listing C.01

Results Of Blood Smears By Visit

Treatment : Placebo

Subject Number	Popn *	Visit	Visit Date	Sample Date	Rel Day**	Symp ?	Rd+	Res +++	Spec #	Spec Count W	Spec Count B	Mean	Diff	Gam Count
030.001.00238	IPP	Load Dose 1	10MAY2000			No								
		Load Dose 2	11MAY2000			No								
		Load Dose 3	12MAY2000			No								
		Week 1	17MAY2000	17MAY2000	7	No	1	Neg						
		Week 1	17MAY2000	17MAY2000		No	2	Neg						
		Week 2	24MAY2000	24MAY2000	14	Yes	1	Neg						
		Week 2	24MAY2000	24MAY2000		Yes	2	Neg						
		Week 2 (Unsch.)	25MAY2000			No								
		Week 3	31MAY2000	31MAY2000	21	No	1	Neg						
		Week 3	31MAY2000	31MAY2000		No	2	Neg						
		Week 5	12JUN2000	08JUN2000	29	No	1	Neg						
		Week 5	12JUN2000	08JUN2000		No	2	Neg						
		Week 5 (Unsch.)	12JUN2000	12JUN2000	33	No	1	Pos	P.f	0.3	2.5	5.8	-6.7	
		Week 5 (Unsch.)	12JUN2000	12JUN2000		No	2	Pos	P.f	1.1	9.2			
		Week 5	14JUN2000	14JUN2000	35	No	1	Neg						
		Week 5	14JUN2000	14JUN2000		No	2	Neg						
		Week 6 (Fol. Up)	21JUN2000	21JUN2000	42	No	1	Neg						
		Week 6 (Fol. Up)	21JUN2000	21JUN2000		No	2	Neg						
		Week 7 (Unsch. Fol. Up)	26JUN2000			No								
		Week 7 (Unsch. Fol. Up)	27JUN2000			No								
		Week 7 (Fol. Up)	28JUN2000	28JUN2000	49	No	1	Neg						
		Week 7 (Fol. Up)	28JUN2000	28JUN2000		No	2	Neg						
		Week 8 (Fol. Up)	04JUL2000	04JUL2000	55	No	1	Neg						
		Week 8 (Fol. Up)	04JUL2000	04JUL2000		No	2	Neg						
		Week 8 (Unsch. Fol. Up)	05JUL2000	05JUL2000	56	Yes	1	Pos	P.f	13.2	140.3	98.6	83.5	
		Week 8 (Unsch. Fol. Up)	05JUL2000	05JUL2000			1		P.o	1.7	17.5	14.4	6.1	
		Week 8 (Unsch. Fol. Up)	05JUL2000	05JUL2000		Yes	2	Pos	P.f	5.4	56.8			
		Week 8 (Unsch. Fol. Up)	05JUL2000	05JUL2000			2		P.o	1.1	11.3			
		Week 9 (Fol. Up)	11JUL2000	12JUL2000	63	Yes	1	Pos	P.f	1.6	16.4	17.1	-1.4	
		Week 9 (Fol. Up)	11JUL2000	12JUL2000		Yes	2	Neg						
		Week 9 (Fol. Up)	11JUL2000	12JUL2000		Yes	Arb	Pos	P.f	1.7	17.8			
030.001.00242	IPP	Screening	14APR2000	14APR2000	-26		1	Pos	P.f	2.0	14.4	8.5	11.9	
		Screening	14APR2000	14APR2000			2	Neg						
		Screening	14APR2000	14APR2000			Arb	Pos	P.f	0.4	2.5			
		Screening (Unsch.)	24APR2000	24APR2000	-16		1	Neg						
		Screening (Unsch.)	24APR2000	24APR2000			2	Neg						

*Popn=Population (I=Intent-To-Treat, PP=Per Protocol)
**Rel Day=Number of days relative to start of prophylactic medication
Symp = Symptoms, Rd = Reader, Res = Result, Spec = Species, Diff = Difference
Spec Count W = Species Count recorded per 500 White Blood Cells
Spec Count B = Species Count converted to per microlitre of blood
Gam = Gametocyte
+Arb=Arbitrator, +++Pos=Positive, Neg=Negative
#P. f = P.falciparum, P. v = P.vivax, P. o = P.ovale, P. m = P.malariae
Tafenoquine = Tafenoquine 200mg, Mefloquine = Mefloquine 250mg

Listing C.01

Results Of Blood Smears By Visit

Treatment : Placebo

Subject Number	Popn *	Visit	Visit Date	Sample Date	Rel Day**	Symp ?	Rd+	Res +++	Spec #	Spec Count W	Spec Count B	Mean	Diff	Gam Count
030.001.00242	IPP	Post Clearance	08MAY2000	08MAY2000	-2		1	Neg						
		Post Clearance	08MAY2000	08MAY2000			2	Neg						
		Post Clearance (Unsch.)	08MAY2000			Yes								
		Load Dose 1	10MAY2000			No								
		Load Dose 2	11MAY2000			No								
		Load Dose 3	12MAY2000			No								
		Week 1 (Unsch.)	15MAY2000			Yes								
		Week 1	17MAY2000	17MAY2000	7	No	1	Neg						
		Week 1	17MAY2000	17MAY2000		No	2	Neg						
		Week 1 (Unsch.)	19MAY2000			Yes								
		Week 2	24MAY2000	24MAY2000	14	No	1	Neg						
		Week 2	24MAY2000	24MAY2000		No	2	Neg						
		Week 3	31MAY2000	31MAY2000	21	No	1	Neg						
		Week 3	31MAY2000	31MAY2000		No	2	Neg						
		Week 4	08JUN2000	08JUN2000	29	No	1	Neg						
		Week 4	08JUN2000	08JUN2000		No	2	Neg						
		Week 5	14JUN2000	14JUN2000	35	No	1	Pos	P.f	1098.6	12744.1	7574.2	10339.8	
		Week 5	14JUN2000	14JUN2000		No	2	Pos	P.f	207.3	2404.3			
		Week 6 (Fol. Up)	21JUN2000	21JUN2000	42	No	1	Pos	P.f	6.4	58.4	36.7	43.5	
		Week 6 (Fol. Up)	21JUN2000	21JUN2000		No	2	Pos	P.f	1.6	14.9			
		Week 7 (Fol. Up)	30JUN2000	30JUN2000	51	No	1	Neg						
		Week 7 (Fol. Up)	30JUN2000	30JUN2000		No	2	Neg						
		Week 8 (Fol. Up)	05JUL2000	05JUL2000	56	No	1	Pos	P.f	3.0	32.1	56.2	-48.2	
		Week 8 (Fol. Up)	05JUL2000	05JUL2000		No	2	Pos	P.f	7.4	80.2			
		Week 9 (Fol. Up)	10JUL2000			Yes								
		Week 9 (Fol. Up)	12JUL2000	12JUL2000	63		1	Pos	P.f	2938.8	31738.7	28426.9	6623.6	
		Week 9 (Fol. Up)	12JUL2000	12JUL2000			2	Pos	P.f	2325.5	25115.1			
030.001.00247	IPP	Screening	11APR2000	11APR2000	-29		1	Pos	P.f	3.8	41.5	44.7	-6.4	
		Screening	11APR2000	11APR2000			2	Pos	P.f	4.4	47.8			
		Post Clearance	08MAY2000	08MAY2000	-2		1	Neg						
		Post Clearance	08MAY2000	08MAY2000			2	Neg						
		Load Dose 1	10MAY2000			No								
		Load Dose 2	11MAY2000			No								
		Load Dose 3	12MAY2000			No								
		Week 1	17MAY2000	17MAY2000	7	No	1	Neg						
		Week 1	17MAY2000	17MAY2000		No	2	Neg						

*Popn=Population (I=Intent-To-Treat, PP=Per Protocol)
**Rel Day=Number of days relative to start of prophylactic medication
Symp = Symptoms, Rd = Reader, Res = Result, Spec = Species, Diff = Difference
Spec Count W = Species Count recorded per 500 White Blood Cells
Spec Count B = Species Count converted to per microlitre of blood
Gam = Gametocyte
+Arb=Arbitrator, +++Pos=Positive, Neg=Negative
#P. f = P.falciparum, P. v = P.vivax, P. o = P.ovale, P. m = P.malariae
Tafenoquine = Tafenoquine 200mg, Mefloquine = Mefloquine 250mg

SB252263 - 030

Listing C.01

Results Of Blood Smears By Visit

Treatment : Placebo

Subject Number	Popn *	Visit	Visit Date	Sample Date	Rel Day**	Symp ?	Rd+	Res +++	Spec #	Spec Count W	Spec Count B	Mean	Diff	Gam Count
030.001.00247	IPP	Week 1 (Unsch.)	20MAY2000			Yes								
		Week 2	24MAY2000	24MAY2000	14	No	1	Neg						
		Week 2	24MAY2000	24MAY2000		No	2	Neg						
		Week 3 (Unsch.)	29MAY2000			Yes								
		Week 3	31MAY2000	31MAY2000	21	No	1	Neg						
		Week 3	31MAY2000	31MAY2000		No	2	Neg						
		Week 4	06JUN2000	06JUN2000	27	No	1	Neg						
		Week 4	06JUN2000	06JUN2000		No	2	Neg						
		Week 5	14JUN2000	14JUN2000	35	No	1	Neg						
		Week 5	14JUN2000	14JUN2000		No	2	Neg						
		Week 5 (Unsch.)	14JUN2000			Yes								
		Week 6	21JUN2000	21JUN2000	42	No	1	Neg						
		Week 6	21JUN2000	21JUN2000		No	2	Neg						
		Week 7 (Unsch.)	26JUN2000			Yes								
		Week 7	28JUN2000	28JUN2000	49	No	1	Neg						
		Week 7	28JUN2000	28JUN2000		No	2	Neg						
		Week 8	04JUL2000	04JUL2000	55	No	1	Neg						
		Week 8	04JUL2000	04JUL2000		No	2	Neg						
		Week 9	14JUL2000	14JUL2000	65	No	1	Neg						
		Week 9	14JUL2000	14JUL2000		No	2	Neg						
		Week 10	19JUL2000	19JUL2000	70	No	1	Neg						
		Week 10	19JUL2000	19JUL2000		No	2	Neg						
		Week 11	26JUL2000	26JUL2000	77	No	1	Neg						
		Week 11	26JUL2000	26JUL2000		No	2	Pos	P.f	0.4	3.3	3.7	-0.8	
		Week 11	26JUL2000	26JUL2000		No	Arb	Pos	P.f	0.5	4.1			
		Week 12 (Fol. Up)	03AUG2000	03AUG2000	85	No	1	Pos	P.f	18.2	142.3	323.9	-363.2	
		Week 12 (Fol. Up)	03AUG2000	03AUG2000		No	2	Pos	P.f	64.8	505.5			
		Week 13 (Fol. Up)	09AUG2000	09AUG2000	91	No	1	Neg						
		Week 13 (Fol. Up)	09AUG2000	09AUG2000		No	2	Pos	P.f	6.0	47.0	37.0	20.0	
		Week 13 (Fol. Up)	09AUG2000	09AUG2000		No	Arb	Pos	P.f	3.5	27.0			
		Week 14 (Fol. Up)	19AUG2000	19AUG2000	101	No	1	Pos	P.f	9.5	73.8	54.4	38.8	
		Week 14 (Fol. Up)	19AUG2000	19AUG2000		No	2	Pos	P.f	4.5	34.9			
		Week 15 (Fol. Up)	23AUG2000	23AUG2000	105	No	1	Pos	P.f	4.0	31.3	25.5	11.6	
		Week 15 (Fol. Up)	23AUG2000	23AUG2000		No	2	Pos	P.f	2.5	19.7			
030.001.00248	IPP	Screening	13APR2000	13APR2000	-27		1	Pos	P.f	0.6	5.5	9.2	-7.4	
		Screening	13APR2000	13APR2000			2	Pos	P.f	1.4	12.9			

*Popn=Population (I=Intent-To-Treat, PP=Per Protocol)
**Rel Day=Number of days relative to start of prophylactic medication
Symp = Symptoms, Rd = Reader, Res = Result, Spec = Species, Diff = Difference
Spec Count W = Species Count recorded per 500 White Blood Cells
Spec Count B = Species Count converted to per microlitre of blood
Gam = Gametocyte
+Arb=Arbitrator, +++Pos=Positive, Neg=Negative
#P. f = P.falciparum, P. v = P.vivax, P. o = P.ovale, P. m = P.malariae
Tafenoquine = Tafenoquine 200mg, Mefloquine = Mefloquine 250mg

SB252263 - 030

Listing C.01

Results Of Blood Smears By Visit

Treatment : Placebo

Subject Number	Popn *	Visit	Visit Date	Sample Date	Rel Day**	Symp ?	Rd+	Res +++	Spec #	Spec Count W	Spec Count B	Mean	Diff	Gam Count
030.001.00248	IPP	Post Clearance	08MAY2000	08MAY2000	-2		1	Neg						
		Post Clearance	08MAY2000	08MAY2000			2	Neg						
		Load Dose 1	10MAY2000			No								
		Load Dose 2	11MAY2000			No								
		Load Dose 3	12MAY2000			Yes								
		Week 1 (Unsch.)	13MAY2000	13MAY2000	3	Yes	1	Neg						
		Week 1 (Unsch.)	13MAY2000	13MAY2000		Yes	2	Neg						
		Week 1 (Unsch.)	15MAY2000	15MAY2000	5	Yes	1	Neg						
		Week 1 (Unsch.)	15MAY2000	15MAY2000		Yes	2	Neg						
		Week 1	17MAY2000	17MAY2000	7	No	1	Neg						
		Week 1	17MAY2000	17MAY2000		No	2	Neg						
		Week 1 (Unsch.)	17MAY2000	17MAY2000		Yes	1	Neg						
		Week 1 (Unsch.)	17MAY2000	17MAY2000		Yes	2	Neg						
		Week 2	24MAY2000	24MAY2000	14	Yes	1	Neg						
		Week 2	24MAY2000	24MAY2000		Yes	2	Neg						
		Week 2 (Unsch.)	24MAY2000	24MAY2000		Yes	1	Neg						
		Week 2 (Unsch.)	24MAY2000	24MAY2000		Yes	2	Neg						
		Week 3	31MAY2000	31MAY2000	21	No	1	Neg						
		Week 3	31MAY2000	31MAY2000		No	2	Neg						
		Week 4	06JUN2000	06JUN2000	27	No	1	Neg						
		Week 4	06JUN2000	06JUN2000		No	2	Neg						
		Week 5	14JUN2000	14JUN2000	35	No	1	Neg						
		Week 5	14JUN2000	14JUN2000		No	2	Neg						
		Week 6	21JUN2000	21JUN2000	42	No	1	Neg						
		Week 6	21JUN2000	21JUN2000		No	2	Neg						
		Week 7	28JUN2000	28JUN2000	49	Yes	1	Neg						
		Week 7	28JUN2000	28JUN2000		Yes	2	Neg						
		Week 7 (Unsch.)	30JUN2000			No								
		Week 8	03JUL2000	03JUL2000	54	Yes	1	Neg						
		Week 8	03JUL2000	03JUL2000		Yes	2	Pos	P.f	1.0	15.0	14.1	2.0	
		Week 8	03JUL2000	03JUL2000		Yes	Arb	Pos	P.f	0.9	13.1			
		Week 9 (Fol. Up)	12JUL2000	12JUL2000	63	No	1	Neg						
		Week 9 (Fol. Up)	12JUL2000	12JUL2000		No	2	Neg						
		Week 10 (Fol. Up)	20JUL2000	20JUL2000	71	No	1	Neg						
		Week 10 (Fol. Up)	20JUL2000	20JUL2000		No	2	Pos	P.f	0.3	2.8	3.3	-1.0	
		Week 10 (Fol. Up)	20JUL2000	20JUL2000		No	Arb	Pos	P.f	0.4	3.8			
		Week 11 (Fol. Up)	26JUL2000	26JUL2000	77	No	1	Neg						

*Popn=Population (I=Intent-To-Treat, PP=Per Protocol)
**Rel Day=Number of days relative to start of prophylactic medication
Symp = Symptoms, Rd = Reader, Res = Result, Spec = Species, Diff = Difference
Spec Count W = Species Count recorded per 500 White Blood Cells
Spec Count B = Species Count converted to per microlitre of blood
Gam = Gametocyte
+Arb=Arbitrator, +++Pos=Positive, Neg=Negative
#P. f = P.falciparum, P. v = P.vivax, P. o = P.ovale, P. m = P.malariae
Tafenoquine = Tafenoquine 200mg, Mefloquine = Mefloquine 250mg

SB252263 - 030

Listing C.01

Results Of Blood Smears By Visit

Treatment : Placebo

Subject Number	Popn *	Visit	Visit Date	Sample Date	Rel Day**	Symp ?	Rd+	Res +++	Spec #	Spec Count W	Spec Count B	Mean	Diff	Gam Count
030.001.00248	IPP	Week 11 (Fol. Up)	26JUL2000	26JUL2000		No	2	Neg						
		Week 12 (Unsch. Fol. Up)	31JUL2000			No								
		Week 12 (Fol. Up)	02AUG2000	02AUG2000	84	No	1	Neg						
		Week 12 (Fol. Up)	02AUG2000	02AUG2000		No	2	Neg						
030.001.00251	IPP	Screening	12APR2000	12APR2000	-28		1	Neg						
		Screening	12APR2000	12APR2000			2	Neg						
		Screening (Unsch.)	23APR2000	23APR2000	-17		1	Neg						
		Screening (Unsch.)	23APR2000	23APR2000			2	Neg						
		Post Clearance	08MAY2000	08MAY2000	-2		1	Neg						
		Post Clearance	08MAY2000	08MAY2000			2	Neg						
		Load Dose 1	10MAY2000			No								
		Load Dose 2	11MAY2000			No								
		Load Dose 3	12MAY2000			No								
		Week 1	17MAY2000	17MAY2000	7	No	1	Neg						
		Week 1	17MAY2000	17MAY2000		No	2	Neg						
		Week 2	24MAY2000	24MAY2000	14	No	1	Neg						
		Week 2	24MAY2000	24MAY2000		No	2	Neg						
		Week 3	31MAY2000	31MAY2000	21	No	1	Neg						
		Week 3	31MAY2000	31MAY2000		No	2	Neg						
		Week 4	06JUN2000	06JUN2000	27	Yes	1	Neg						
		Week 4	06JUN2000	06JUN2000		Yes	2	Neg						
		Week 4 (Unsch.)	09JUN2000			No								
		Week 5	14JUN2000	14JUN2000	35	Yes	1	Pos	P.f	16.0	147.0	96.5	101.0	
		Week 5	14JUN2000	14JUN2000		Yes	2	Pos	P.f	5.0	46.0			
		Week 6 (Unsch. Fol. Up)	20JUN2000	20JUN2000	41		1	Pos	P.f	250.0	2300.0	2792.8	-985.7	
		Week 6 (Unsch. Fol. Up)	20JUN2000	20JUN2000			2	Pos	P.f	357.1	3285.7			
		Week 6 (Fol. Up)	21JUN2000	21JUN2000	42	No	1	Pos	P.f	5.6	51.2	50.1	2.3	
		Week 6 (Fol. Up)	21JUN2000	21JUN2000		No	2	Neg						
		Week 6 (Fol. Up)	21JUN2000	21JUN2000		No	Arb	Pos	P.f	5.3	48.9			
		Week 7 (Unsch. Fol. Up)	25JUN2000			No								
		Week 7 (Fol. Up)	28JUN2000	28JUN2000	49	No	1	Neg						
		Week 7 (Fol. Up)	28JUN2000	28JUN2000		No	2	Pos	P.f	3.8	34.7	27.1	15.1	
		Week 7 (Fol. Up)	28JUN2000	28JUN2000		No	Arb	Pos	P.f	2.1	19.6			
		Week 8 (Unsch. Fol. Up)	07JUL2000	07JUL2000	58	Yes	1	Pos	P.f	0.6	5.9	4.5	2.9	
		Week 8 (Unsch. Fol. Up)	07JUL2000	07JUL2000		Yes	2	Pos	P.f	0.3	3.1			
		Week 9 (Unsch. Fol. Up)	10JUL2000			Yes								

*Popn=Population (I=Intent-To-Treat, PP=Per Protocol)
**Rel Day=Number of days relative to start of prophylactic medication
Symp = Symptoms, Rd = Reader, Res = Result, Spec = Species, Diff = Difference
Spec Count W = Species Count recorded per 500 White Blood Cells
Spec Count B = Species Count converted to per microlitre of blood
Gam = Gametocyte
+Arb=Arbitrator, +++Pos=Positive, Neg=Negative
#P. f = P.falciparum, P. v = P.vivax, P. o = P.ovale, P. m = P.malariae
Tafenoquine = Tafenoquine 200mg, Mefloquine = Mefloquine 250mg

Listing C.01

Results Of Blood Smears By Visit

Treatment : Placebo

Subject Number	Popn *	Visit	Visit Date	Sample Date	Rel Day**	Symp ?	Rd+	Res +++	Spec #	Spec Count W	Spec Count B	Mean	Diff	Gam Count
030.001.00251	IPP	Week 9 (Fol. Up)	12JUL2000	12JUL2000	63	No	1	Neg						
		Week 9 (Fol. Up)	12JUL2000	12JUL2000		No	2	Neg						
030.001.00253	IPP	Screening	13APR2000	13APR2000	-27		1	Neg						
		Screening	13APR2000	13APR2000			2	Neg						
		Post Clearance	08MAY2000	08MAY2000	-2		1	Neg						
		Post Clearance	08MAY2000	08MAY2000			2	Neg						
		Load Dose 1	10MAY2000			No								
		Load Dose 2	11MAY2000			No								
		Load Dose 3	12MAY2000			No								
		Week 1	17MAY2000	17MAY2000	7	No	1	Neg						
		Week 1	17MAY2000	17MAY2000		No	2	Neg						
		Week 2	24MAY2000	24MAY2000	14	No	1	Neg						
		Week 2	24MAY2000	24MAY2000		No	2	Neg						
		Week 3	31MAY2000	31MAY2000	21	No	1	Neg						
		Week 3	31MAY2000	31MAY2000		No	2	Neg						
		Week 4	06JUN2000	06JUN2000	27	No	1	Neg						
		Week 4	06JUN2000	06JUN2000		No	2	Neg						
		Week 5	14JUN2000	14JUN2000	35	No	1	Neg						
		Week 5	14JUN2000	14JUN2000		No	2	Neg						
		Week 6	21JUN2000	21JUN2000	42	No	1	Neg						
		Week 6	21JUN2000	21JUN2000		No	2	Neg						
		Week 7	28JUN2000	28JUN2000	49	No	1	Pos	P.f	0.7	5.7	5.4	0.5	
		Week 7	28JUN2000	28JUN2000		No	2	Pos	P.f	0.6	5.2			
		Week 8 (Unsch. Fol. Up)	03JUL2000			No								
		Week 8 (Fol. Up)	06JUL2000	06JUL2000	57	No	1	Neg						
		Week 8 (Fol. Up)	06JUL2000	06JUL2000		No	2	Neg						
		Week 9 (Fol. Up)	12JUL2000	12JUL2000	63	No	1	Neg						
		Week 9 (Fol. Up)	12JUL2000	12JUL2000		No	2	Neg						
		Week 10 (Fol. Up)	19JUL2000	19JUL2000	70	Yes	1	Neg						
		Week 10 (Fol. Up)	19JUL2000	19JUL2000		Yes	2	Neg						
		Week 11 (Unsch. Fol. Up)	25JUL2000	25JUL2000	76	Yes	1	Pos	P.f	11.3	97.1	69.4	55.4	
		Week 11 (Unsch. Fol. Up)	25JUL2000	25JUL2000		Yes	2	Pos	P.f	4.9	41.7			
		Week 11 (Fol. Up)	27JUL2000	27JUL2000	78		1	Pos	P.f	5.9	50.6	50.6	0.0	
		Week 11 (Fol. Up)	27JUL2000	27JUL2000			2	Neg						
		Week 11 (Fol. Up)	27JUL2000	27JUL2000			Arb	Pos	P.f	5.9	50.6			

*Popn=Population (I=Intent-To-Treat, PP=Per Protocol)
**Rel Day=Number of days relative to start of prophylactic medication
Symp = Symptoms, Rd = Reader, Res = Result, Spec = Species, Diff = Difference
Spec Count W = Species Count recorded per 500 White Blood Cells
Spec Count B = Species Count converted to per microlitre of blood
Gam = Gametocyte
+Arb=Arbitrator, +++Pos=Positive, Neg=Negative
#P. f = P.falciparum, P. v = P.vivax, P. o = P.ovale, P. m = P.malariae
Tafenoquine = Tafenoquine 200mg, Mefloquine = Mefloquine 250mg

SB252263 - 030

Listing C.01

Results Of Blood Smears By Visit

Treatment : Placebo

Subject Number	Popn *	Visit	Visit Date	Sample Date	Rel Day**	Symp ?	Rd+	Res +++	Spec #	Spec Count W	Spec Count B	Mean	Diff	Gam Count
030.001.00256	IPP	Screening	14APR2000	14APR2000	-26		1	Pos	P.f	0.5	6.4	15.9	-19.0	
		Screening	14APR2000	14APR2000			2	Neg						
		Screening	14APR2000	14APR2000			Arb	Pos	P.f	1.9	25.5			
		Post Clearance	08MAY2000	08MAY2000	-2		1	Neg						
		Post Clearance	08MAY2000	08MAY2000			2	Neg						
		Load Dose 1	10MAY2000			No								
		Load Dose 2	11MAY2000			No								
		Load Dose 3	12MAY2000			No								
		Week 1	17MAY2000	17MAY2000	7	No	1	Neg						
		Week 1	17MAY2000	17MAY2000		No	2	Neg						
		Week 2	24MAY2000	24MAY2000	14	No	1	Neg						
		Week 2	24MAY2000	24MAY2000		No	2	Neg						
		Week 2 (Unsch.)	24MAY2000			No								
		Week 3	31MAY2000	31MAY2000	21	No	1	Neg						
		Week 3	31MAY2000	31MAY2000		No	2	Neg						
		Week 4	06JUN2000	06JUN2000	27	No	1	Neg						
		Week 4	06JUN2000	06JUN2000		No	2	Neg						
		Week 5	16JUN2000	16JUN2000	37	No	1	Neg						
		Week 5	16JUN2000	16JUN2000		No	2	Neg						
		Week 5 (Unsch.)	16JUN2000	16JUN2000			1	Pos	P.f	1.0	18.0	13.8	8.4	
		Week 5 (Unsch.)	16JUN2000	16JUN2000			2	Pos	P.f	0.5	9.6			
		Week 5 (Unsch.)	16JUN2000	16JUN2000		No	1	Pos	P.f	1.5	27.1	18.3	17.7	
		Week 5 (Unsch. Fol. Up)	17JUN2000	17JUN2000	38	No	1	Neg						
		Week 5 (Unsch. Fol. Up)	17JUN2000	17JUN2000		No	2	Neg						
		Week 5 (Unsch.)	16JUN2000	19JUN2000	40	No	2	Pos	P.f	0.5	9.5			
		Week 6 (Fol. Up)	21JUN2000	21JUN2000	42		1	Neg						
		Week 6 (Fol. Up)	21JUN2000	21JUN2000			2	Neg						
		Week 6 (Fol. Up)	21JUN2000	21JUN2000		No		Neg						
		Week 7 (Fol. Up)	29JUN2000	29JUN2000	50		1	Neg						
		Week 7 (Fol. Up)	29JUN2000	29JUN2000			2	Neg						
		Week 7 (Fol. Up)	29JUN2000	29JUN2000		No		Neg						
		Week 8 (Fol. Up)	04JUL2000	04JUL2000	55		1	Neg						
		Week 8 (Fol. Up)	04JUL2000	04JUL2000			2	Neg						
		Week 8 (Fol. Up)	04JUL2000	04JUL2000		No		Neg						
		Week 9 (Fol. Up)	10JUL2000	10JUL2000	61		1	Neg						
		Week 9 (Fol. Up)	10JUL2000	10JUL2000			2	Pos	P.f	1.0	15.0	15.9	-1.9	
		Week 9 (Fol. Up)	10JUL2000	10JUL2000			Arb	Pos	P.f	1.2	16.8			

*Popn=Population (I=Intent-To-Treat, PP=Per Protocol)
**Rel Day=Number of days relative to start of prophylactic medication
Symp = Symptoms, Rd = Reader, Res = Result, Spec = Species, Diff = Difference
Spec Count W = Species Count recorded per 500 White Blood Cells
Spec Count B = Species Count converted to per microlitre of blood
Gam = Gametocyte
+Arb=Arbitrator, +++Pos=Positive, Neg=Negative
#P. f = P.falciparum, P. v = P.vivax, P. o = P.ovale, P. m = P.malariae
Tafenoquine = Tafenoquine 200mg, Mefloquine = Mefloquine 250mg

Listing C.01

Results Of Blood Smears By Visit

Treatment : Placebo

Subject Number	Popn *	Visit	Visit Date	Sample Date	Rel Day**	Symp ?	Rd+	Res +++	Spec #	Spec Count W	Spec Count B	Mean	Diff	Gam Count
030.001.00256	IPP	Week 9 (Fol. Up)	10JUL2000			No								
030.001.00257	IPP	Screening	14APR2000	14APR2000	-28		1	Pos	P.f	5.7	67.4	82.2	-29.6	
		Screening	14APR2000	14APR2000			1		P.m	1.4	16.8	22.9	-12.3	
		Screening	14APR2000	14APR2000			2	Pos	P.f	8.2	97.0			
		Screening	14APR2000	14APR2000			2		P.m	2.5	29.0			
		Post Clearance (Unsch.)	08MAY2000			Yes								
		Post Clearance	12MAY2000	12MAY2000	0		1	Neg						
		Post Clearance	12MAY2000	12MAY2000			2	Neg						
		Load Dose 1	12MAY2000			No								
		Load Dose 2	13MAY2000			No								
		Load Dose 3	14MAY2000			No								
		Week 1	18MAY2000	18MAY2000	6	Yes	1	Neg						
		Week 1	18MAY2000	18MAY2000		Yes	2	Neg						
		Week 2	25MAY2000	25MAY2000	13	Yes	1	Neg						
		Week 2	25MAY2000	25MAY2000		Yes	2	Neg						
		Week 3	01JUN2000	01JUN2000	20	No	1	Neg						
		Week 3	01JUN2000	01JUN2000		No	2	Neg						
		Week 4	06JUN2000	06JUN2000	25	No	1	Neg						
		Week 4	06JUN2000	06JUN2000		No	2	Neg						
		Week 4 (Unsch.)	11JUN2000			Yes								
		Week 5	16JUN2000	16JUN2000	35	Yes	1	Neg						
		Week 5	16JUN2000	16JUN2000		Yes	2	Neg						
		Week 6	23JUN2000	23JUN2000	42	No	1	Pos	P.f	2.9	32.2	29.8	4.7	
		Week 6	23JUN2000	23JUN2000		No	2	Pos	P.f	2.5	27.5			
		Week 7 (Fol. Up)	28JUN2000	28JUN2000	47	No	1	Neg						
		Week 7 (Fol. Up)	28JUN2000	28JUN2000		No	2	Neg						
		Week 8 (Fol. Up)	05JUL2000	05JUL2000	54	No	1	Pos	P.f	6.3	69.8	37.3	65.2	
		Week 8 (Fol. Up)	05JUL2000	05JUL2000		No	2	Pos	P.f	0.4	4.7			
		Week 8 (Unsch. Fol. Up)	10JUL2000	10JUL2000	59	Yes	1	Neg						
		Week 8 (Unsch. Fol. Up)	10JUL2000	10JUL2000		Yes	2	Neg						
		Week 9 (Fol. Up)	13JUL2000	13JUL2000	62	No	1	Neg						
		Week 9 (Fol. Up)	13JUL2000	13JUL2000		No	2	Neg						
		Week 10 (Fol. Up)	20JUL2000	20JUL2000	69	Yes	1	Pos	P.f	0.3	3.5	4.7	-2.5	
		Week 10 (Fol. Up)	20JUL2000	20JUL2000		Yes	2	Pos	P.f	0.5	5.9			
030.001.00260	IPP	Screening	17APR2000	17APR2000	-23		1	Pos	P.f	10.0	90.0	108.4	-36.7	

*Popn=Population (I=Intent-To-Treat, PP=Per Protocol)
**Rel Day=Number of days relative to start of prophylactic medication
Symp = Symptoms, Rd = Reader, Res = Result, Spec = Species, Diff = Difference
Spec Count W = Species Count recorded per 500 White Blood Cells
Spec Count B = Species Count converted to per microlitre of blood
Gam = Gametocyte
+Arb=Arbitrator, +++Pos=Positive, Neg=Negative
#P. f = P.falciparum, P. v = P.vivax, P. o = P.ovale, P. m = P.malariae
Tafenoquine = Tafenoquine 200mg, Mefloquine = Mefloquine 250mg

Listing C.01

Results Of Blood Smears By Visit

Treatment : Placebo

Subject Number	Popn *	Visit	Visit Date	Sample Date	Rel Day**	Symp ?	Rd+	Res +++	Spec #	Spec Count W	Spec Count B	Mean	Diff	Gam Count
030.001.00260	IPP	Screening	17APR2000	17APR2000			2	Pos	P.f	14.1	126.7			
		Post Clearance	08MAY2000	08MAY2000	-2		1	Neg						
		Post Clearance	08MAY2000	08MAY2000			2	Neg						
		Load Dose 1	10MAY2000			No								
		Load Dose 2	11MAY2000			No								
		Load Dose 3	12MAY2000			No								
		Week 1	17MAY2000	17MAY2000	7	No	1	Neg						
		Week 1	17MAY2000	17MAY2000		No	2	Neg						
		Week 1 (Unsch.)	18MAY2000			No								
		Week 2	24MAY2000	24MAY2000	14	No	1	Neg						
		Week 2	24MAY2000	24MAY2000		No	2	Neg						
		Week 2 (Unsch.)	27MAY2000			No								
		Week 3	31MAY2000	31MAY2000	21	No	1	Neg						
		Week 3	31MAY2000	31MAY2000		No	2	Neg						
		Week 4	06JUN2000	06JUN2000	27	No	1	Neg						
		Week 4	06JUN2000	06JUN2000		No	2	Neg						
		Week 5	15JUN2000	15JUN2000	36	No	1	Neg						
		Week 5	15JUN2000	15JUN2000		No	2	Neg						
		Week 6	21JUN2000	21JUN2000	42	No	1	Pos	P.f	3.7	31.1	26.4	9.3	
		Week 6	21JUN2000	21JUN2000		No	2	Pos	P.f	2.6	21.8			
		Week 6 (Unsch. Fol. Up)	23JUN2000			No								
		Week 7 (Fol. Up)	28JUN2000	28JUN2000	49		1	Neg						
		Week 7 (Fol. Up)	28JUN2000	28JUN2000			2	Neg						
		Week 7 (Fol. Up)	28JUN2000	28JUN2000		No		Neg						
		Week 7 (Unsch. Fol. Up)	01JUL2000			Yes								
		Week 8 (Fol. Up)	04JUL2000	04JUL2000	55		1	Neg						
		Week 8 (Fol. Up)	04JUL2000	04JUL2000			2	Neg						
		Week 8 (Fol. Up)	04JUL2000	04JUL2000		No		Neg						
		Week 9 (Fol. Up)	12JUL2000	12JUL2000	63		1	Neg						
		Week 9 (Fol. Up)	12JUL2000	12JUL2000			2	Neg						
		Week 9 (Fol. Up)	12JUL2000	12JUL2000		No		Neg						
		Week 10 (Fol. Up)	18JUL2000	18JUL2000	69		1	Neg						
		Week 10 (Fol. Up)	18JUL2000	18JUL2000			2	Pos	P.f	0.8	6.6	5.2	2.8	
		Week 10 (Fol. Up)	18JUL2000	18JUL2000			Arb	Pos	P.f	0.5	3.8			
		Week 10 (Unsch. Fol. Up)	19JUL2000			No								
030.001.00268	IPP	Screening	14APR2000	14APR2000	-26		1	Neg						

*Popn=Population (I=Intent-To-Treat, PP=Per Protocol)
**Rel Day=Number of days relative to start of prophylactic medication
Symp = Symptoms, Rd = Reader, Res = Result, Spec = Species, Diff = Difference
Spec Count W = Species Count recorded per 500 White Blood Cells
Spec Count B = Species Count converted to per microlitre of blood
Gam = Gametocyte
+Arb=Arbitrator, +++Pos=Positive, Neg=Negative
#P. f = P.falciparum, P. v = P.vivax, P. o = P.ovale, P. m = P.malariae
Tafenoquine = Tafenoquine 200mg, Mefloquine = Mefloquine 250mg

SB252263 - 030

Listing C.01

Results Of Blood Smears By Visit

Treatment : Placebo

Subject Number	Popn *	Visit	Visit Date	Sample Date	Rel Day**	Symp ?	Rd+	Res +++	Spec #	Spec Count W	Spec Count B	Mean	Diff	Gam Count
030.001.00268	IPP	Screening	14APR2000	14APR2000			2	Neg						
		Post Clearance	08MAY2000	08MAY2000	-2		1	Neg						
		Post Clearance	08MAY2000	08MAY2000			2	Neg						
		Load Dose 1	10MAY2000			No								
		Load Dose 2	11MAY2000			No								
		Load Dose 3	12MAY2000			No								
		Week 1	17MAY2000	17MAY2000	7	Yes	1	Neg						
		Week 1	17MAY2000	17MAY2000		Yes	2	Neg						
		Week 1 (Unsch.)	18MAY2000			Yes								
		Week 2	24MAY2000	24MAY2000	14	No	1	Pos	P.f	0.4	2.4	6.4	-7.9	
		Week 2	24MAY2000	24MAY2000		No	2	Pos	P.f	1.6	10.3			
		Week 3 (Fol. Up)	31MAY2000	31MAY2000	21	Yes	1	Neg						
		Week 3 (Fol. Up)	31MAY2000	31MAY2000		Yes	2	Neg						
		Week 3 (Unsch. Fol. Up)	31MAY2000			No								
		Week 4 (Fol. Up)	06JUN2000	06JUN2000	27	No	1	Neg						
		Week 4 (Fol. Up)	06JUN2000	06JUN2000		No	2	Neg						
		Week 4 (Unsch. Fol. Up)	09JUN2000			No								
		Week 5 (Unsch. Fol. Up)	13JUN2000			No								
		Week 5 (Fol. Up)	14JUN2000	14JUN2000	35	No	1	Neg						
		Week 5 (Fol. Up)	14JUN2000	14JUN2000		No	2	Neg						
030.001.00269	IPP	Screening	13APR2000	13APR2000	-27		1	Pos	P.f	2.5	65.8	97.3	-63.1	
		Screening	13APR2000	13APR2000			2	Pos	P.f	4.9	128.9			
		Post Clearance	08MAY2000	08MAY2000	-2		1	Neg						
		Post Clearance	08MAY2000	08MAY2000			2	Neg						
		Load Dose 1	10MAY2000			No								
		Load Dose 2	11MAY2000			No								
		Load Dose 3	12MAY2000			No								
		Week 1	18MAY2000	18MAY2000	8	No	1	Neg						
		Week 1	18MAY2000	18MAY2000		No	2	Neg						
		Week 2	24MAY2000	24MAY2000	14	Yes	1	Neg						
		Week 2	24MAY2000	24MAY2000		Yes	2	Neg						
		Week 2 (Unsch.)	24MAY2000			Yes								
		Week 3	31MAY2000	31MAY2000	21	No	1	Neg						
		Week 3	31MAY2000	31MAY2000		No	2	Neg						
		Week 4	06JUN2000	06JUN2000	27	No	1	Neg						
		Week 4	06JUN2000	06JUN2000		No	2	Neg						

*Popn=Population (I=Intent-To-Treat, PP=Per Protocol)
**Rel Day=Number of days relative to start of prophylactic medication
Symp = Symptoms, Rd = Reader, Res = Result, Spec = Species, Diff = Difference
Spec Count W = Species Count recorded per 500 White Blood Cells
Spec Count B = Species Count converted to per microlitre of blood
Gam = Gametocyte
+Arb=Arbitrator, +++Pos=Positive, Neg=Negative
#P. f = P.falciparum, P. v = P.vivax, P. o = P.ovale, P. m = P.malariae
Tafenoquine = Tafenoquine 200mg, Mefloquine = Mefloquine 250mg

Listing C.01

Results Of Blood Smears By Visit

Treatment : Placebo

Subject Number	Popn *	Visit	Visit Date	Sample Date	Rel Day**	Symp ?	Rd+	Res +++	Spec #	Spec Count W	Spec Count B	Mean	Diff	Gam Count
030.001.00269	IPP	Week 5	14JUN2000	14JUN2000	35	No	1	Neg						
		Week 5	14JUN2000	14JUN2000		No	2	Neg						
		Week 6	21JUN2000	21JUN2000	42	No	1	Pos	P.f	16.1	261.1	172.0	178.4	
		Week 6	21JUN2000	21JUN2000		No	2	Pos	P.f	5.1	82.8			
		Week 7 (Unsch. Fol. Up)	26JUN2000			Yes								
		Week 7 (Fol. Up)	28JUN2000	28JUN2000	49	No	1	Pos	P.f	7.6	123.8	76.1	95.3	
		Week 7 (Fol. Up)	28JUN2000	28JUN2000		No	2	Pos	P.f	1.8	28.5			
		Week 8 (Fol. Up)	06JUL2000	06JUL2000	57	No	1	Pos	P.f	87.2	1412.2	1417.0	-9.7	
		Week 8 (Fol. Up)	06JUL2000	06JUL2000		No	2	Pos	P.f	87.8	1421.9			
		Week 9 (Fol. Up)	13JUL2000	13JUL2000	64	No	1	Neg						
		Week 9 (Fol. Up)	13JUL2000	13JUL2000		No	2	Pos	P.f	6.1	99.1	82.8	32.7	
		Week 9 (Fol. Up)	13JUL2000	13JUL2000		No	Arb	Pos	P.f	4.1	66.4			
		Week 10 (Fol. Up)	20JUL2000	20JUL2000	71		1	Neg						
		Week 10 (Fol. Up)	20JUL2000	20JUL2000			2	Neg						
		Week 10 (Fol. Up)	20JUL2000	20JUL2000		No		Neg						
030.001.00270	IPP	Screening	13APR2000	13APR2000	-27		1	Pos	P.f	2.0	16.8	10.1	13.4	
		Screening	13APR2000	13APR2000			2	Neg						
		Screening	13APR2000	13APR2000			Arb	Pos	P.f	0.4	3.4			
		Post Clearance	07MAY2000	07MAY2000	-3		1	Neg						
		Post Clearance	07MAY2000	07MAY2000			2	Neg						
		Load Dose 1	10MAY2000			No								
		Load Dose 2	11MAY2000			No								
		Load Dose 3	12MAY2000			No								
		Week 1	17MAY2000	17MAY2000	7	No	1	Neg						
		Week 1	17MAY2000	17MAY2000		No	2	Neg						
		Week 2	24MAY2000	24MAY2000	14	No	1	Neg						
		Week 2	24MAY2000	24MAY2000		No	2	Neg						
		Week 3	31MAY2000	31MAY2000	21	No	1	Neg						
		Week 3	31MAY2000	31MAY2000		No	2	Neg						
		Week 4	07JUN2000	07JUN2000	28	No	1	Neg						
		Week 4	07JUN2000	07JUN2000		No	2	Neg						
		Week 5	14JUN2000	14JUN2000	35	No	1	Neg						
		Week 5	14JUN2000	14JUN2000		No	2	Neg						
		Week 6	21JUN2000	21JUN2000	42	No	1	Neg						
		Week 6	21JUN2000	21JUN2000		No	2	Neg						
		Week 7	28JUN2000	28JUN2000	49	No	1	Neg						

*Popn=Population (I=Intent-To-Treat, PP=Per Protocol)
**Rel Day=Number of days relative to start of prophylactic medication
Symp = Symptoms, Rd = Reader, Res = Result, Spec = Species, Diff = Difference
Spec Count W = Species Count recorded per 500 White Blood Cells
Spec Count B = Species Count converted to per microlitre of blood
Gam = Gametocyte
+Arb=Arbitrator, +++Pos=Positive, Neg=Negative
#P. f = P.falciparum, P. v = P.vivax, P. o = P.ovale, P. m = P.malariae
Tafenoquine = Tafenoquine 200mg, Mefloquine = Mefloquine 250mg

SB252263 - 030

Listing C.01

Results Of Blood Smears By Visit

Treatment : Placebo

Subject Number	Popn *	Visit	Visit Date	Sample Date	Rel Day**	Symp ?	Rd+	Res +++	Spec #	Spec Count W	Spec Count B	Mean	Diff	Gam Count
030.001.00270	IPP	Week 7	28JUN2000	28JUN2000		No	2	Pos	P.f	2.7	24.1	24.1	0.0	
		Week 7	28JUN2000	28JUN2000		No	Arb	Pos	P.f	2.7	24.1			
		Week 8 (Fol. Up)	04JUL2000	04JUL2000	55	No	1	Neg						
		Week 8 (Fol. Up)	04JUL2000	04JUL2000		No	2	Neg						
		Week 9 (Fol. Up)	12JUL2000	12JUL2000	63	No	1	Neg						
		Week 9 (Fol. Up)	12JUL2000	12JUL2000		No	2	Neg						
		Week 10 (Fol. Up)	19JUL2000	19JUL2000	70	No	1	Neg						
		Week 10 (Fol. Up)	19JUL2000	19JUL2000		No	2	Neg						
		Week 11 (Fol. Up)	26JUL2000	26JUL2000	77		1	Pos	P.f	7.9	85.0	59.4	51.2	
		Week 11 (Fol. Up)	26JUL2000	26JUL2000			2	Pos	P.f	3.1	33.8			
030.001.00275	IPP	Screening	12APR2000	12APR2000	-28			Neg						
		Screening (Unsch.)	12APR2000	12APR2000			1	Neg						
		Screening (Unsch.)	12APR2000	12APR2000			2	Neg						
		Screening (Unsch.)	01MAY2000	01MAY2000	-9		1	Neg						
		Screening (Unsch.)	01MAY2000	01MAY2000			2	Neg						
		Post Clearance	08MAY2000	08MAY2000	-2		1	Neg						
		Post Clearance	08MAY2000	08MAY2000			2	Neg						
		Load Dose 1	10MAY2000			No								
		Load Dose 2	11MAY2000			No								
		Load Dose 3	12MAY2000			No								
		Week 1	17MAY2000	17MAY2000	7	Yes	1	Neg						
		Week 1	17MAY2000	17MAY2000		Yes	2	Neg						
		Week 2 (Unsch.)	22MAY2000			Yes								
		Week 2	24MAY2000	24MAY2000	14	No	1	Neg						
		Week 2	24MAY2000	24MAY2000		No	2	Neg						
		Week 2 (Unsch.)	27MAY2000			Yes								
		Week 3	31MAY2000	31MAY2000	21	No	1	Neg						
		Week 3	31MAY2000	31MAY2000		No	2	Neg						
		Week 4	06JUN2000	06JUN2000	27	No	1	Neg						
		Week 4	06JUN2000	06JUN2000		No	2	Neg						
		Week 5	14JUN2000	14JUN2000	35	No	1	Neg						
		Week 5	14JUN2000	14JUN2000		No	2	Neg						
		Week 6	21JUN2000	21JUN2000	42	No	1	Neg						
		Week 6	21JUN2000	21JUN2000		No	2	Neg						
		Week 7	28JUN2000	28JUN2000	49	No	1	Neg						
		Week 7	28JUN2000	28JUN2000		No	2	Pos	P.f	0.3	3.3	3.3	0.0	

*Popn=Population (I=Intent-To-Treat, PP=Per Protocol)
**Rel Day=Number of days relative to start of prophylactic medication
Symp = Symptoms, Rd = Reader, Res = Result, Spec = Species, Diff = Difference
Spec Count W = Species Count recorded per 500 White Blood Cells
Spec Count B = Species Count converted to per microlitre of blood
Gam = Gametocyte
+Arb=Arbitrator, +++Pos=Positive, Neg=Negative
#P. f = P.falciparum, P. v = P.vivax, P. o = P.ovale, P. m = P.malariae
Tafenoquine = Tafenoquine 200mg, Mefloquine = Mefloquine 250mg

SB252263 - 030

Listing C.01

Results Of Blood Smears By Visit

Treatment : Placebo

Subject Number	Popn *	Visit	Visit Date	Sample Date	Rel Day**	Symp ?	Rd+	Res +++	Spec #	Spec Count W	Spec Count B	Mean	Diff	Gam Count
030.001.00275	IPP	Week 7	28JUN2000	28JUN2000		No	Arb	Pos	P.f	0.3	3.3			
		Week 8 (Unsch. Fol. Up)	05JUL2000			Yes								
		Week 9 (Fol. Up)	13JUL2000	13JUL2000	64	No	1	Neg						
		Week 9 (Fol. Up)	13JUL2000	13JUL2000		No	2	Neg						
		Week 10 (Fol. Up)	19JUL2000	19JUL2000	70	No	1	Neg						
		Week 10 (Fol. Up)	19JUL2000	19JUL2000		No	2	Neg						
		Week 11 (Fol. Up)	26JUL2000	26JUL2000	77	No	1	Pos	P.f	6.0	54.0	28.5	50.9	
		Week 11 (Fol. Up)	26JUL2000	26JUL2000		No	2	Pos	P.f	0.3	3.1			
030.001.00277	IPP	Screening	12APR2000	13APR2000	-27		1	Pos	P.f	103.2	866.6	1042.1	-351.0	
		Screening	12APR2000	13APR2000			2	Pos	P.f	145.0	1217.6			
		Post Clearance	08MAY2000	08MAY2000	-2		1	Neg						
		Post Clearance	08MAY2000	08MAY2000			2	Neg						
		Load Dose 1	10MAY2000			No								
		Load Dose 2	11MAY2000			No								
		Load Dose 3	12MAY2000			No								
		Week 1	17MAY2000	17MAY2000	7	No	1	Neg						
		Week 1	17MAY2000	17MAY2000		No	2	Neg						
		Week 2	24MAY2000	24MAY2000	14	No	1	Neg						
		Week 2	24MAY2000	24MAY2000		No	2	Neg						
		Week 3	31MAY2000	31MAY2000	21	No	1	Neg						
		Week 3	31MAY2000	31MAY2000		No	2	Neg						
		Week 4	07JUN2000	07JUN2000	28	No	1	Neg						
		Week 4	07JUN2000	07JUN2000		No	2	Neg						
		Week 5 (Unsch.)	13JUN2000			Yes								
		Week 5	14JUN2000	14JUN2000	35	No	1	Neg						
		Week 5	14JUN2000	14JUN2000		No	2	Neg						
		Week 6	21JUN2000	21JUN2000	42	No	1	Neg						
		Week 6	21JUN2000	21JUN2000		No	2	Neg						
		Week 7	28JUN2000	28JUN2000	49	No	1	Neg						
		Week 7	28JUN2000	28JUN2000		No	2	Neg						
		Week 8	04JUL2000	04JUL2000	55	No	1	Neg						
		Week 8	04JUL2000	04JUL2000		No	2	Neg						
		Week 9	12JUL2000	12JUL2000	63	No	1	Neg						
		Week 9	12JUL2000	12JUL2000		No	2	Neg						
		Week 10	19JUL2000	19JUL2000	70	Yes	1	Pos	P.f	944.6	9824.3	10041.8	-435.1	
		Week 10	19JUL2000	19JUL2000		Yes	2	Pos	P.f	986.5	10259.4			

*Popn=Population (I=Intent-To-Treat, PP=Per Protocol)
**Rel Day=Number of days relative to start of prophylactic medication
Symp = Symptoms, Rd = Reader, Res = Result, Spec = Species, Diff = Difference
Spec Count W = Species Count recorded per 500 White Blood Cells
Spec Count B = Species Count converted to per microlitre of blood
Gam = Gametocyte
+Arb=Arbitrator, +++Pos=Positive, Neg=Negative
#P. f = P.falciparum, P. v = P.vivax, P. o = P.ovale, P. m = P.malariae
Tafenoquine = Tafenoquine 200mg, Mefloquine = Mefloquine 250mg

SB252263 - 030

Listing C.01

Results Of Blood Smears By Visit

Treatment : Placebo

Subject Number	Popn *	Visit	Visit Date	Sample Date	Rel Day**	Symp ?	Rd+	Res +++	Spec #	Spec Count W	Spec Count B	Mean	Diff	Gam Count
030.001.00277	IPP	Week 10 (Unsch.)	19JUL2000	19JUL2000			1	Pos	P.f	631.1	6563.0	6761.5	-397.0	
		Week 10 (Unsch.)	19JUL2000	19JUL2000			2	Pos	P.f	669.2	6960.0			
		Week 11 (Fol. Up)	26JUL2000	26JUL2000	77	No	1	Neg						
		Week 11 (Fol. Up)	26JUL2000	26JUL2000		No	2	Pos	P.f	1.4	14.1	16.7	-5.1	
		Week 11 (Fol. Up)	26JUL2000	26JUL2000		No	Arb	Pos	P.f	1.9	19.2			
		Week 12 (Fol. Up)	02AUG2000	02AUG2000	84	No	1	Neg						
		Week 12 (Fol. Up)	02AUG2000	02AUG2000		No	2	Neg						
		Week 13 (Fol. Up)	09AUG2000	09AUG2000	91	No	1	Neg						
		Week 13 (Fol. Up)	09AUG2000	09AUG2000		No	2	Neg						
		Week 14 (Fol. Up)	16AUG2000	16AUG2000	98		1	Neg						
		Week 14 (Fol. Up)	16AUG2000	16AUG2000			2	Neg						
030.001.00278	IPP	Screening	12APR2000	12APR2000	-28		1	Pos	P.f	522.6	5643.9	4808.5	1670.8	
		Screening	12APR2000	12APR2000			2	Pos	P.f	367.9	3973.1			
		Post Clearance	08MAY2000	08MAY2000	-2		1	Neg						
		Post Clearance	08MAY2000	08MAY2000			2	Neg						
		Post Clearance (Unsch.)	09MAY2000	09MAY2000	-1		1	Neg						
		Post Clearance (Unsch.)	09MAY2000	09MAY2000			2	Neg						
		Post Clearance (Unsch.)	09MAY2000	09MAY2000		Yes		Neg						
		Load Dose 1	10MAY2000			No								
		Load Dose 2	12MAY2000			Yes								
		Load Dose 3	13MAY2000			Yes								
		Week 1	17MAY2000	17MAY2000	7	Yes	1	Neg						
		Week 1	17MAY2000	17MAY2000		Yes	2	Neg						
		Week 2	24MAY2000	24MAY2000	14	Yes		Neg						
		Week 3	31MAY2000	31MAY2000	21	No	1	Neg						
		Week 3	31MAY2000	01JUN2000	22	No	2	Neg						
		Week 4	07JUN2000	07JUN2000	28	Yes	1	Neg						
		Week 4	07JUN2000	07JUN2000		Yes	2	Neg						
		Week 5 (Unsch.)	13JUN2000			No								
		Week 5	14JUN2000	14JUN2000	35	No	1	Neg						
		Week 5	14JUN2000	14JUN2000		No	2	Pos	P.f	1.2	10.5	13.0	-5.1	
		Week 5	14JUN2000	14JUN2000		No	Arb	Pos	P.f	1.8	15.6			
		Week 6 (Unsch. Fol. Up)	18JUN2000			No								
		Week 6 (Fol. Up)	21JUN2000	21JUN2000	42		1	Neg						
		Week 6 (Fol. Up)	21JUN2000	21JUN2000			2	Neg						
		Week 6 (Fol. Up)	21JUN2000	21JUN2000		No		Neg						

*Popn=Population (I=Intent-To-Treat, PP=Per Protocol)
**Rel Day=Number of days relative to start of prophylactic medication
Symp = Symptoms, Rd = Reader, Res = Result, Spec = Species, Diff = Difference
Spec Count W = Species Count recorded per 500 White Blood Cells
Spec Count B = Species Count converted to per microlitre of blood
Gam = Gametocyte
+Arb=Arbitrator, +++Pos=Positive, Neg=Negative
#P. f = P.falciparum, P. v = P.vivax, P. o = P.ovale, P. m = P.malariae
Tafenoquine = Tafenoquine 200mg, Mefloquine = Mefloquine 250mg

Listing C.01

Results Of Blood Smears By Visit

Treatment : Placebo

Subject Number	Popn *	Visit	Visit Date	Sample Date	Rel Day**	Symp ?	Rd+	Res +++	Spec #	Spec Count W	Spec Count B	Mean	Diff	Gam Count
030.001.00278	IPP	Week 7 (Fol. Up)	28JUN2000	28JUN2000	49		1	Pos	P.f	444.4	4444.4	2668.6	3551.6	
		Week 7 (Fol. Up)	28JUN2000	28JUN2000			2	Pos	P.f	89.3	892.8			
		Week 7 (Fol. Up)	28JUN2000	28JUN2000		No		Pos						
		Week 8 (Unsch. Fol. Up)	02JUL2000			Yes								
		Week 8 (Fol. Up)	04JUL2000	04JUL2000	55		1	Neg						
		Week 8 (Fol. Up)	04JUL2000	04JUL2000			2	Neg						
		Week 8 (Fol. Up)	04JUL2000	04JUL2000		No		Neg						
		Week 9 (Fol. Up)	12JUL2000	12JUL2000	63		1	Neg						
		Week 9 (Fol. Up)	12JUL2000	12JUL2000			2	Neg						
030.001.00285	IPP	Screening	18APR2000	18APR2000	-23		1	Neg						
		Screening	18APR2000	18APR2000			2	Neg						
		Post Clearance	10MAY2000	10MAY2000	-1		1	Neg						
		Post Clearance	10MAY2000	10MAY2000			2	Neg						
		Load Dose 1	11MAY2000			No								
		Load Dose 2	12MAY2000			No								
		Load Dose 3	13MAY2000			No								
		Week 1	18MAY2000	18MAY2000	7	No	1	Neg						
		Week 1	18MAY2000	18MAY2000		No	2	Neg						
		Week 2	24MAY2000	24MAY2000	13	No	1	Neg						
		Week 2	24MAY2000	24MAY2000		No	2	Neg						
		Week 3	31MAY2000	31MAY2000	20	No	1	Neg						
		Week 3	31MAY2000	31MAY2000		No	2	Neg						
		Week 4	06JUN2000	06JUN2000	26	No	1	Neg						
		Week 4	06JUN2000	06JUN2000		No	2	Neg						
		Week 5	14JUN2000	14JUN2000	34	No	1	Neg						
		Week 5	14JUN2000	14JUN2000		No	2	Neg						
		Week 6	21JUN2000	21JUN2000	41	No	1	Neg						
		Week 6	21JUN2000	21JUN2000		No	2	Neg						
		Week 7	28JUN2000	28JUN2000	48	No	1	Pos	P.f	0.4	1.9	5.2	-6.5	
		Week 7	28JUN2000	28JUN2000		No	2	Pos	P.f	1.6	8.5			
		Week 8 (Fol. Up)	05JUL2000	05JUL2000	55	No	1	Neg						
		Week 8 (Fol. Up)	05JUL2000	05JUL2000		No	2	Neg						
		Week 9 (Fol. Up)	13JUL2000	13JUL2000	63	No	1	Pos	P.f	154.5	834.5	538.2	592.7	
		Week 9 (Fol. Up)	13JUL2000	13JUL2000		No	2	Pos	P.f	44.8	241.9			
		Week 10 (Fol. Up)	20JUL2000	20JUL2000	70	No	1	Neg						
		Week 10 (Fol. Up)	20JUL2000	20JUL2000		No	2	Neg						

*Popn=Population (I=Intent-To-Treat, PP=Per Protocol)
**Rel Day=Number of days relative to start of prophylactic medication
Symp = Symptoms, Rd = Reader, Res = Result, Spec = Species, Diff = Difference
Spec Count W = Species Count recorded per 500 White Blood Cells
Spec Count B = Species Count converted to per microlitre of blood
Gam = Gametocyte
+Arb=Arbitrator, +++Pos=Positive, Neg=Negative
#P. f = P.falciparum, P. v = P.vivax, P. o = P.ovale, P. m = P.malariae
Tafenoquine = Tafenoquine 200mg, Mefloquine = Mefloquine 250mg

Listing C.01

Results Of Blood Smears By Visit

Treatment : Placebo

Subject Number	Popn *	Visit	Visit Date	Sample Date	Rel Day**	Symp ?	Rd+	Res +++	Spec #	Spec Count W	Spec Count B	Mean	Diff	Gam Count
030.001.00285	IPP	Week 11 (Fol. Up)	27JUL2000	27JUL2000	77		1	Pos	P.f	5.5	29.7	16.6	26.3	
		Week 11 (Fol. Up)	27JUL2000	27JUL2000			2	Pos	P.f	0.6	3.4			
030.001.00287	IPP	Screening	10APR2000	10APR2000	-31		1	Neg						
		Screening	10APR2000	10APR2000			2	Neg						
		Clearance (Unsch.)	07MAY2000			No								
		Post Clearance (Unsch.)	08MAY2000	08MAY2000	-3		1	Neg						
		Post Clearance (Unsch.)	08MAY2000	08MAY2000			2	Neg						
		Post Clearance	10MAY2000	10MAY2000	-1		1	Neg						
		Post Clearance	10MAY2000	10MAY2000			2	Neg						
		Post Clearance (Unsch.)	10MAY2000	10MAY2000			1	Neg						
		Post Clearance (Unsch.)	10MAY2000	10MAY2000			2	Neg						
		Load Dose 1	11MAY2000			No								
		Load Dose 2	12MAY2000			No								
		Load Dose 3	13MAY2000			No								
		Week 1	18MAY2000	18MAY2000	7	No	1	Neg						
		Week 1	18MAY2000	18MAY2000		No	2	Neg						
		Week 1 (Unsch.)	19MAY2000			Yes								
		Week 2 (Unsch.)	23MAY2000			Yes								
		Week 2	24MAY2000	24MAY2000	13	No	1	Neg						
		Week 2	24MAY2000	24MAY2000		No	2	Neg						
		Week 2 (Unsch.)	24MAY2000			No								
		Week 3	31MAY2000	31MAY2000	20	No	1	Neg						
		Week 3	31MAY2000	31MAY2000		No	2	Neg						
		Week 4	06JUN2000	06JUN2000	26	No	1	Neg						
		Week 4	06JUN2000	06JUN2000		No	2	Neg						
		Week 5	14JUN2000	14JUN2000	34	No	1	Neg						
		Week 5	14JUN2000	14JUN2000		No	2	Neg						
		Week 6	21JUN2000	21JUN2000	41	Yes	1	Neg						
		Week 6	21JUN2000	21JUN2000		Yes	2	Neg						
		Week 7	28JUN2000	28JUN2000	48	Yes	1	Neg						
		Week 7	28JUN2000	28JUN2000		Yes	2	Neg						
		Week 8	04JUL2000	04JUL2000	54	Yes	1	Neg						
		Week 8	04JUL2000	04JUL2000		Yes	2	Neg						
		Week 9	12JUL2000	12JUL2000	62	Yes	1	Neg						
		Week 9	12JUL2000	12JUL2000		Yes	2	Neg						
		Week 10 (Unsch.)	18JUL2000			No								

*Popn=Population (I=Intent-To-Treat, PP=Per Protocol)
**Rel Day=Number of days relative to start of prophylactic medication
Symp = Symptoms, Rd = Reader, Res = Result, Spec = Species, Diff = Difference
Spec Count W = Species Count recorded per 500 White Blood Cells
Spec Count B = Species Count converted to per microlitre of blood
Gam = Gametocyte
+Arb=Arbitrator, +++Pos=Positive, Neg=Negative
#P. f = P.falciparum, P. v = P.vivax, P. o = P.ovale, P. m = P.malariae
Tafenoquine = Tafenoquine 200mg, Mefloquine = Mefloquine 250mg

SB252263 - 030

Listing C.01

Results Of Blood Smears By Visit

Treatment : Placebo

Subject Number	Popn *	Visit	Visit Date	Sample Date	Rel Day**	Symp ?	Rd+	Res +++	Spec #	Spec Count W	Spec Count B	Mean	Diff	Gam Count
030.001.00287	IPP	Week 10	19JUL2000	19JUL2000	69	No	1	Neg						
		Week 10	19JUL2000	19JUL2000		No	2	Neg						
		Week 11	26JUL2000	26JUL2000	76	No	1	Pos	P.f	16.4	131.4	131.3	0.3	
		Week 11	26JUL2000	26JUL2000		No	2	Neg						
		Week 11	26JUL2000	26JUL2000		No	Arb	Pos	P.f	16.4	131.1			
		Week 13 (Unsch. Fol. Up)	08AUG2000	08AUG2000	89	Yes	1	Neg						
		Week 13 (Unsch. Fol. Up)	08AUG2000	08AUG2000		Yes	2	Neg						
		Week 13 (Fol. Up)	09AUG2000	09AUG2000	90	No	1	Neg						
		Week 13 (Fol. Up)	09AUG2000	09AUG2000		No	2	Neg						
		Week 14 (Fol. Up)	16AUG2000	16AUG2000	97	No	1	Neg						
		Week 14 (Fol. Up)	16AUG2000	16AUG2000		No	2	Neg						
		Week 14 (Unsch. Fol. Up)	17AUG2000			Yes								
		Week 15 (Fol. Up)	23AUG2000	23AUG2000	104	No	1	Neg						
		Week 15 (Fol. Up)	23AUG2000	23AUG2000		No	2	Neg						
030.001.00291	IPP	Screening	17APR2000	17APR2000	-24		1	Pos	P.f	5.8	84.1	122.6	-77.1	
		Screening	17APR2000	17APR2000			2	Pos	P.f	11.0	161.2			
		Post Clearance (Unsch.)	08MAY2000			No								
		Post Clearance	10MAY2000	10MAY2000	-1		1	Neg						
		Post Clearance	10MAY2000	10MAY2000			2	Neg						
		Load Dose 1	11MAY2000			No								
		Load Dose 2	12MAY2000			No								
		Load Dose 3	13MAY2000			No								
		Load Dose (Unsch.)	13MAY2000			No								
		Week 1	18MAY2000	18MAY2000	7	No	1	Neg						
		Week 1	18MAY2000	18MAY2000		No	2	Neg						
		Week 2	24MAY2000	24MAY2000	13	No	1	Neg						
		Week 2	24MAY2000	24MAY2000		No	2	Neg						
		Week 3	31MAY2000	31MAY2000	20	No	1	Neg						
		Week 3	31MAY2000	31MAY2000		No	2	Neg						
		Week 4	06JUN2000	06JUN2000	26	No	1	Neg						
		Week 4	06JUN2000	06JUN2000		No	2	Neg						
		Week 5	14JUN2000	14JUN2000	34	No	1	Neg						
		Week 5	14JUN2000	14JUN2000		No	2	Neg						
		Week 5 (Unsch.)	16JUN2000			No								
		Week 6	21JUN2000	21JUN2000	41	No	1	Neg						
		Week 6	21JUN2000	21JUN2000		No	2	Neg						

*Popn=Population (I=Intent-To-Treat, PP=Per Protocol)
**Rel Day=Number of days relative to start of prophylactic medication
Symp = Symptoms, Rd = Reader, Res = Result, Spec = Species, Diff = Difference
Spec Count W = Species Count recorded per 500 White Blood Cells
Spec Count B = Species Count converted to per microlitre of blood
Gam = Gametocyte
+Arb=Arbitrator, +++Pos=Positive, Neg=Negative
#P. f = P.falciparum, P. v = P.vivax, P. o = P.ovale, P. m = P.malariae
Tafenoquine = Tafenoquine 200mg, Mefloquine = Mefloquine 250mg

SB252263 - 030

Listing C.01

Results Of Blood Smears By Visit

Treatment : Placebo

Subject Number	Popn *	Visit	Visit Date	Sample Date	Rel Day**	Symp ?	Rd+	Res +++	Spec #	Spec Count W	Spec Count B	Mean	Diff	Gam Count
030.001.00291	IPP	Week 7	28JUN2000	28JUN2000	48	No	1	Neg						
		Week 7	28JUN2000	28JUN2000		No	2	Pos	P.f	0.6	10.1	8.7	2.8	
		Week 7	28JUN2000	28JUN2000		No	Arb	Pos	P.f	0.5	7.3			
		Week 8 (Fol. Up)	04JUL2000	04JUL2000	54	No	1	Neg						
		Week 8 (Fol. Up)	04JUL2000	04JUL2000		No	2	Neg						
		Week 9 (Fol. Up)	12JUL2000	12JUL2000	62	No	1	Pos	P.f	2.5	40.1	51.7	-23.2	
		Week 9 (Fol. Up)	12JUL2000	12JUL2000			1		P.o	1.7	26.7	60.9	-68.4	
		Week 9 (Fol. Up)	12JUL2000	12JUL2000		No	2	Pos	P.f	4.0	63.4			
		Week 9 (Fol. Up)	12JUL2000	12JUL2000			2		P.o	6.0	95.1			
		Week 10 (Unsch. Fol. Up)	18JUL2000	18JUL2000	68	Yes	1	Pos	P.f	1.9	29.4	26.2	6.3	
		Week 10 (Unsch. Fol. Up)	18JUL2000	18JUL2000			1		P.o	2.8	44.1			
		Week 10 (Unsch. Fol. Up)	18JUL2000	18JUL2000		Yes	2	Pos	P.f	1.5	23.1			
		Week 10 (Fol. Up)	19JUL2000	19JUL2000	69	Yes	1	Pos	P.f	0.3	4.9	18.1	-26.4	
		Week 10 (Fol. Up)	19JUL2000	19JUL2000		Yes	2	Pos	P.f	2.0	31.3			
		Week 11 (Fol. Up)	26JUL2000	26JUL2000	76		1	Pos	P.f	0.9	13.6	13.0	1.3	
		Week 11 (Fol. Up)	26JUL2000	26JUL2000			2	Neg						
		Week 11 (Fol. Up)	26JUL2000	26JUL2000			Arb	Pos	P.f	0.8	12.3			
030.001.00295		Screening	17APR2000	17APR2000	-24		1	Neg						
		Screening	17APR2000	17APR2000			2	Pos	P.f	4.0	35.2	41.5	-12.7	
		Screening	17APR2000	17APR2000			Arb	Pos	P.f	5.4	47.9			
		Post Clearance	10MAY2000	10MAY2000	-1		1	Pos	P.f	1.0	7.8	6.6	2.5	
		Post Clearance	10MAY2000	10MAY2000			2	Neg						
		Post Clearance	10MAY2000	10MAY2000			Arb	Pos	P.f	0.7	5.3			
		Load Dose 1	11MAY2000			No								
		Load Dose 2	12MAY2000			No								
		Load Dose 3	13MAY2000			No								
		Week 1	18MAY2000	18MAY2000	7	No	1	Neg						
		Week 1	18MAY2000	18MAY2000		No	2	Neg						
		Week 2 (Unsch.)	23MAY2000			No								
		Week 2	24MAY2000	24MAY2000	13	No	1	Neg						
		Week 2	24MAY2000	24MAY2000		No	2	Neg						
		Week 3	31MAY2000	31MAY2000	20	Yes	1	Neg						
		Week 3	31MAY2000	31MAY2000		Yes	2	Neg						
		Week 4	06JUN2000	06JUN2000	26	No	1	Neg						
		Week 4	06JUN2000	06JUN2000		No	2	Neg						
		Week 5	14JUN2000	14JUN2000	34	No	1	Neg						

*Popn=Population (I=Intent-To-Treat, PP=Per Protocol)
**Rel Day=Number of days relative to start of prophylactic medication
Symp = Symptoms, Rd = Reader, Res = Result, Spec = Species, Diff = Difference
Spec Count W = Species Count recorded per 500 White Blood Cells
Spec Count B = Species Count converted to per microlitre of blood
Gam = Gametocyte
+Arb=Arbitrator, +++Pos=Positive, Neg=Negative
#P. f = P.falciparum, P. v = P.vivax, P. o = P.ovale, P. m = P.malariae
Tafenoquine = Tafenoquine 200mg, Mefloquine = Mefloquine 250mg

SB252263 - 030

Listing C.01

Results Of Blood Smears By Visit

Treatment : Placebo

Subject Number	Popn *	Visit	Visit Date	Sample Date	Rel Day**	Symp ?	Rd+	Res +++	Spec #	Spec Count W	Spec Count B	Mean	Diff	Gam Count
030.001.00295		Week 5	14JUN2000	14JUN2000		No	2	Neg						
		Week 6	21JUN2000	21JUN2000	41	No	1	Neg						
		Week 6	21JUN2000	21JUN2000		No	2	Neg						
		Week 7	28JUN2000	28JUN2000	48	Yes	1	Neg						
		Week 7	28JUN2000	28JUN2000		Yes	2	Neg						
		Week 8	04JUL2000	04JUL2000	54	Yes	1	Neg						
		Week 8	04JUL2000	04JUL2000		Yes	2	Neg						
		Week 9	12JUL2000	12JUL2000	62	No	1	Neg						
		Week 9	12JUL2000	12JUL2000		No	2	Neg						
		Week 10	19JUL2000	19JUL2000	69	No	1	Pos	P.f	1.6	12.5	13.5	-2.0	
		Week 10	19JUL2000	19JUL2000		No	2	Neg						
		Week 10	19JUL2000	19JUL2000		No	Arb	Pos	P.f	1.9	14.5			
		Week 11 (Fol. Up)	26JUL2000	26JUL2000	76	No	1	Neg						
		Week 11 (Fol. Up)	26JUL2000	26JUL2000		No	2	Neg						
		Week 12 (Fol. Up)	02AUG2000	02AUG2000	83	No	1	Neg						
		Week 12 (Fol. Up)	02AUG2000	02AUG2000		No	2	Neg						
		Week 13 (Fol. Up)	09AUG2000	09AUG2000	90	No	1	Neg						
		Week 13 (Fol. Up)	09AUG2000	09AUG2000		No	2	Neg						
		Week 14 (Fol. Up)	16AUG2000	16AUG2000	97	No	1	Neg						
		Week 14 (Fol. Up)	16AUG2000	16AUG2000		No	2	Neg						
030.001.00297	IPP	Screening	18APR2000	18APR2000	-23		1	Neg						
		Screening	18APR2000	18APR2000			2	Pos	P.f	2.0	31.2	59.1	-55.8	
		Screening	18APR2000	18APR2000			Arb	Pos	P.f	5.6	87.0			
		Post Clearance	11MAY2000	11MAY2000	0		1	Neg						
		Post Clearance	11MAY2000	11MAY2000			2	Neg						
		Load Dose 1	11MAY2000			Yes								
		Load Dose 2	12MAY2000			No								
		Load Dose 3	13MAY2000			Yes								
		Week 1	19MAY2000	19MAY2000	8	No	1	Neg						
		Week 1	19MAY2000	19MAY2000		No	2	Neg						
		Week 2	24MAY2000	24MAY2000	13	No	1	Neg						
		Week 2	24MAY2000	24MAY2000		No	2	Neg						
		Week 3	31MAY2000	31MAY2000	20	No	1	Neg						
		Week 3	31MAY2000	31MAY2000		No	2	Neg						
		Week 4	07JUN2000	07JUN2000	27	No	1	Neg						
		Week 4	07JUN2000	07JUN2000		No	2	Neg						

*Popn=Population (I=Intent-To-Treat, PP=Per Protocol)
**Rel Day=Number of days relative to start of prophylactic medication
Symp = Symptoms, Rd = Reader, Res = Result, Spec = Species, Diff = Difference
Spec Count W = Species Count recorded per 500 White Blood Cells
Spec Count B = Species Count converted to per microlitre of blood
Gam = Gametocyte
+Arb=Arbitrator, +++Pos=Positive, Neg=Negative
#P. f = P.falciparum, P. v = P.vivax, P. o = P.ovale, P. m = P.malariae
Tafenoquine = Tafenoquine 200mg, Mefloquine = Mefloquine 250mg

Listing C.01

Results Of Blood Smears By Visit

Treatment : Placebo

Subject Number	Popn *	Visit	Visit Date	Sample Date	Rel Day**	Symp ?	Rd+	Res +++	Spec #	Spec Count W	Spec Count B	Mean	Diff	Gam Count
030.001.00297	IPP	Week 5	14JUN2000	14JUN2000	34	No	1	Neg						
		Week 5	14JUN2000	14JUN2000		No	2	Neg						
		Week 6	21JUN2000	21JUN2000	41	No	1	Neg						
		Week 6	21JUN2000	21JUN2000		No	2	Neg						
		Week 7	28JUN2000	28JUN2000	48	No	1	Neg						
		Week 7	28JUN2000	28JUN2000		No	2	Neg						
		Week 8	06JUL2000	06JUL2000	56	Yes	1	Neg						
		Week 8	06JUL2000	06JUL2000		Yes	2	Neg						
		Week 9	12JUL2000	12JUL2000	62	No	1	Neg						
		Week 9	12JUL2000	12JUL2000		No	2	Neg						
		Week 10	20JUL2000	20JUL2000	70	No	1	Neg						
		Week 10	20JUL2000	20JUL2000		No	2	Neg						
		Week 11	26JUL2000	26JUL2000	76	No	1	Neg						
		Week 11	26JUL2000	26JUL2000		No	2	Neg						
		Week 12	31JUL2000	31JUL2000	81	No	1	Neg						
		Week 12	31JUL2000	31JUL2000		No	2	Neg						
		Week 13	09AUG2000	09AUG2000	90	No	1	Neg						
		Week 13	09AUG2000	09AUG2000		No	2	Neg						
		Week 14	16AUG2000	16AUG2000	97	No	1	Neg						
		Week 14	16AUG2000	16AUG2000		No	2	Neg						
		Week 15	26AUG2000	26AUG2000	107	No	1	Neg						
		Week 15	26AUG2000	26AUG2000		No	2	Neg						
		Week 16	30AUG2000	30AUG2000	111	No	1	Neg						
		Week 16	30AUG2000	30AUG2000		No	2	Neg						
		Week 17	08SEP2000			No								
		Week 18	13SEP2000	13SEP2000	125	Yes	1	Neg						
		Week 18	13SEP2000	13SEP2000		Yes	2	Neg						
		Week 19	20SEP2000	20SEP2000	132	No	1	Pos	P.f	9.2	163.8	143.0	41.5	
		Week 19	20SEP2000	20SEP2000		No	2	Pos	P.f	6.9	122.3			
		Week 20 (Fol. Up)	26SEP2000	26SEP2000	138	Yes	1	Neg						
		Week 20 (Fol. Up)	26SEP2000	26SEP2000		Yes	2	Neg						
		Week 21 (Fol. Up)	04OCT2000	04OCT2000	146	No	1	Pos	P.f	109.9	1956.0	1683.4	545.2	
		Week 21 (Fol. Up)	04OCT2000	04OCT2000		No	2	Pos	P.f	79.3	1410.8			
		Week 22 (Fol. Up)	12OCT2000	12OCT2000	154	No	1	Pos	P.f	5.9	104.1	68.5	71.2	
		Week 22 (Fol. Up)	12OCT2000	12OCT2000		No	2	Pos	P.f	1.9	32.9			
		Week 23 (Fol. Up)	18OCT2000	18OCT2000	160		1	Pos	P.f	2.8	49.3	43.5	11.6	
		Week 23 (Fol. Up)	18OCT2000	18OCT2000			2	Neg						

*Popn=Population (I=Intent-To-Treat, PP=Per Protocol)
**Rel Day=Number of days relative to start of prophylactic medication
Symp = Symptoms, Rd = Reader, Res = Result, Spec = Species, Diff = Difference
Spec Count W = Species Count recorded per 500 White Blood Cells
Spec Count B = Species Count converted to per microlitre of blood
Gam = Gametocyte
+Arb=Arbitrator, +++Pos=Positive, Neg=Negative
#P. f = P.falciparum, P. v = P.vivax, P. o = P.ovale, P. m = P.malariae
Tafenoquine = Tafenoquine 200mg, Mefloquine = Mefloquine 250mg

SB252263 - 030

Listing C.01

Results Of Blood Smears By Visit

Treatment : Placebo

Subject Number	Popn *	Visit	Visit Date	Sample Date	Rel Day**	Symp ?	Rd+	Res +++	Spec #	Spec Count W	Spec Count B	Mean	Diff	Gam Count
030.001.00297	IPP	Week 23 (Fol. Up)	18OCT2000	18OCT2000			Arb	Pos	P.f	2.1	37.7			
030.001.00300	IPP	Screening	11APR2000	11APR2000	-31		1	Pos	P.f	122.6	1226.0	1409.4	-366.7	
		Screening	11APR2000	11APR2000			2	Pos	P.f	159.3	1592.7			
		Screening (Unsch.)	30APR2000	30APR2000	-12		1	Neg						
		Screening (Unsch.)	30APR2000	30APR2000			2	Neg						
		Post Clearance	08MAY2000	08MAY2000	-4		1	Neg						
		Post Clearance	08MAY2000	08MAY2000			2	Neg						
		Load Dose 1	12MAY2000			No								
		Load Dose 2	13MAY2000			No								
		Load Dose 3	14MAY2000			No								
		Week 1	19MAY2000	19MAY2000	7	No	1	Neg						
		Week 1	19MAY2000	19MAY2000		No	2	Neg						
		Week 2	24MAY2000	24MAY2000	12	Yes	1	Neg						
		Week 2	24MAY2000	24MAY2000		Yes	2	Neg						
		Week 3	01JUN2000	01JUN2000	20	Yes	1	Neg						
		Week 3	01JUN2000	01JUN2000		Yes	2	Neg						
		Week 4	07JUN2000	07JUN2000	26	Yes	1	Neg						
		Week 4	07JUN2000	07JUN2000		Yes	2	Neg						
		Week 5	18JUN2000	18JUN2000	37	No	1	Neg						
		Week 5	18JUN2000	18JUN2000		No	2	Neg						
		Week 6	21JUN2000	21JUN2000	40	No	1	Neg						
		Week 6	21JUN2000	21JUN2000		No	2	Pos	P.f	4.3	35.6	35.6	0.0	
		Week 6	21JUN2000	21JUN2000		No	Arb	Pos	P.f	4.3	35.6			
		Week 7 (Fol. Up)	01JUL2000	01JUL2000	50	No	1	Pos	P.f	7.1	58.5	36.1	44.9	
		Week 7 (Fol. Up)	01JUL2000	01JUL2000		No	2	Pos	P.f	1.7	13.6			
		Week 8 (Fol. Up)	04JUL2000	04JUL2000	53	No	1	Pos	P.f	109.8	900.0	1215.3	-630.7	
		Week 8 (Fol. Up)	04JUL2000	04JUL2000		No	2	Pos	P.f	186.7	1530.6			
		Week 9 (Fol. Up)	12JUL2000	12JUL2000	61	No	1	Pos	P.f	67.1	550.0	375.9	348.2	
		Week 9 (Fol. Up)	12JUL2000	12JUL2000		No	2	Pos	P.f	24.6	201.8			
		Week 10 (Fol. Up)	19JUL2000	19JUL2000	68		1	Pos	P.f	87.6	718.3	791.6	-146.6	
		Week 10 (Fol. Up)	19JUL2000	19JUL2000			2	Pos	P.f	105.5	864.9			
030.001.00303	IPP	Screening	13APR2000	13APR2000	-29		1	Pos	P.f	38.5	484.6	592.3	-215.3	
		Screening	13APR2000	13APR2000			2	Pos	P.f	55.6	699.9			
		Post Clearance	11MAY2000	11MAY2000	-1		1	Neg						
		Post Clearance	11MAY2000	11MAY2000			2	Neg						

*Popn=Population (I=Intent-To-Treat, PP=Per Protocol)
**Rel Day=Number of days relative to start of prophylactic medication
Symp = Symptoms, Rd = Reader, Res = Result, Spec = Species, Diff = Difference
Spec Count W = Species Count recorded per 500 White Blood Cells
Spec Count B = Species Count converted to per microlitre of blood
Gam = Gametocyte
+Arb=Arbitrator, +++Pos=Positive, Neg=Negative
#P. f = P.falciparum, P. v = P.vivax, P. o = P.ovale, P. m = P.malariae
Tafenoquine = Tafenoquine 200mg, Mefloquine = Mefloquine 250mg

SB252263 - 030

Listing C.01

Results Of Blood Smears By Visit

Treatment : Placebo

Subject Number	Popn *	Visit	Visit Date	Sample Date	Rel Day**	Symp ?	Rd+	Res +++	Spec #	Spec Count W	Spec Count B	Mean	Diff	Gam Count
030.001.00303	IPP	Load Dose 1	12MAY2000			No								
		Load Dose 2	13MAY2000			Yes								
		Load Dose (Unsch.)	13MAY2000			Yes								
		Load Dose 3	14MAY2000			No								
		Week 1	18MAY2000	18MAY2000	6	No	1	Neg						
		Week 1	18MAY2000	18MAY2000		No	2	Neg						
		Week 2	24MAY2000	24MAY2000	12	No	1	Neg						
		Week 2	24MAY2000	24MAY2000		No	2	Neg						
		Week 3	31MAY2000	31MAY2000	19	No	1	Neg						
		Week 3	31MAY2000	31MAY2000		No	2	Neg						
		Week 3 (Unsch.)	04JUN2000			Yes								
		Week 4	06JUN2000	06JUN2000	25	No	1	Neg						
		Week 4	06JUN2000	06JUN2000		No	2	Neg						
		Week 4 (Unsch.)	11JUN2000			No								
		Week 5	14JUN2000	14JUN2000	33	No	1	Neg						
		Week 5	14JUN2000	14JUN2000		No	2	Neg						
		Week 6	21JUN2000	21JUN2000	40	No	1	Neg						
		Week 6	21JUN2000	21JUN2000		No	2	Neg						
		Week 7	28JUN2000	28JUN2000	47	No	1	Neg						
		Week 7	28JUN2000	28JUN2000		No	2	Neg						
		Week 7	03JUL2000	03JUL2000	52	No	1	Neg						
		Week 7	03JUL2000	03JUL2000		No	2	Neg						
		Week 9	12JUL2000	12JUL2000	61	No	1	Pos	P.f	3.1	53.0	52.1	1.9	
		Week 9	12JUL2000	12JUL2000		No	2	Neg						
		Week 9	12JUL2000	12JUL2000		No	Arb	Pos	P.f	3.0	51.2			
		Week 10 (Fol. Up)	19JUL2000	19JUL2000	68	No	1	Neg						
		Week 10 (Fol. Up)	19JUL2000	19JUL2000		No	2	Pos	P.f	3.3	55.9	52.2	7.5	
		Week 10 (Fol. Up)	19JUL2000	19JUL2000		No	Arb	Pos	P.f	2.9	48.5			
		Week 11 (Fol. Up)	26JUL2000	26JUL2000	75	No	1	Neg						
		Week 11 (Fol. Up)	26JUL2000	26JUL2000		No	2	Neg						
		Week 12 (Fol. Up)	03AUG2000	03AUG2000	83	No	1	Pos	P.f	0.7	11.6	31.2	-39.3	
		Week 12 (Fol. Up)	03AUG2000	03AUG2000		No	2	Pos	P.f	3.0	50.8			
		Week 12 (Unsch. Fol. Up)	05AUG2000	05AUG2000	85	Yes	1	Pos	P.f	1.7	28.2	15.0	26.5	
		Week 12 (Unsch. Fol. Up)	05AUG2000	05AUG2000			1		P.o	2.5	42.5	24.1	36.9	
		Week 12 (Unsch. Fol. Up)	05AUG2000	05AUG2000		Yes	2	Pos	P.f	0.1	1.7			
		Week 12 (Unsch. Fol. Up)	05AUG2000	05AUG2000			2		P.o	0.3	5.6			
		Week 13 (Unsch. Fol. Up)	08AUG2000			Yes								

*Popn=Population (I=Intent-To-Treat, PP=Per Protocol)
**Rel Day=Number of days relative to start of prophylactic medication
Symp = Symptoms, Rd = Reader, Res = Result, Spec = Species, Diff = Difference
Spec Count W = Species Count recorded per 500 White Blood Cells
Spec Count B = Species Count converted to per microlitre of blood
Gam = Gametocyte
+Arb=Arbitrator, +++Pos=Positive, Neg=Negative
#P. f = P.falciparum, P. v = P.vivax, P. o = P.ovale, P. m = P.malariae
Tafenoquine = Tafenoquine 200mg, Mefloquine = Mefloquine 250mg

SB252263 - 030

Listing C.01

Results Of Blood Smears By Visit

Treatment : Tafenoquine

Subject Number	Popn *	Visit	Visit Date	Sample Date	Rel Day**	Symp ?	Rd+	Res +++	Spec #	Spec Count W	Spec Count B	Mean	Diff	Gam Count
030.001.00001	IPP	Screening	12APR2000	12APR2000	-26		1	Neg						
		Screening	12APR2000	12APR2000			2	Neg						
		Screening (Unsch.)	02MAY2000	02MAY2000	-6	Yes	1	Neg						
		Screening (Unsch.)	02MAY2000	02MAY2000		Yes	2	Neg						
		Post Clearance	06MAY2000	06MAY2000	-2		1	Neg						
		Post Clearance	06MAY2000	06MAY2000			2	Neg						
		Load Dose 1	08MAY2000			No								
		Load Dose 2	09MAY2000			No								
		Load Dose 3	10MAY2000			Yes								
		Week 1	15MAY2000	15MAY2000	7	No	1	Pos	P.f	1.7	16.2	11.6	9.1	
		Week 1	15MAY2000	15MAY2000		No	2	Pos	P.f	0.7	7.1			
		Week 1 (Unsch. Fol. Up)	16MAY2000			No								
		Week 3 (Fol. Up)	26MAY2000	26MAY2000	18	Yes	1	Neg						
		Week 3 (Fol. Up)	26MAY2000	26MAY2000		Yes	2	Neg						
		Week 3 (Fol. Up)	29MAY2000	29MAY2000	21	No	1	Neg						
		Week 3 (Fol. Up)	29MAY2000	29MAY2000		No	2	Neg						
		Week 3 (Unsch. Fol. Up)	30MAY2000			Yes								
		Week 4 (Fol. Up)	05JUN2000	05JUN2000	28	No	1	Neg						
		Week 4 (Fol. Up)	05JUN2000	05JUN2000		No	2	Neg						
		Week 5 (Fol. Up)	12JUN2000	12JUN2000	35	No	1	Neg						
		Week 5 (Fol. Up)	12JUN2000	12JUN2000		No	2	Neg						
030.001.00004	IPP	Screening	13APR2000	13APR2000	-25		1	Pos	P.f	2.0	21.2	18.7	5.0	
		Screening	13APR2000	13APR2000			2	Neg						
		Screening	13APR2000	13APR2000			Arb	Pos	P.f	1.5	16.2			
		Screening (Unsch.)	01MAY2000			No								
		Post Clearance	06MAY2000	06MAY2000	-2		1	Neg						
		Post Clearance	06MAY2000	06MAY2000			2	Neg						
		Post Clearance (Unsch.)	07MAY2000			No								
		Load Dose 1	08MAY2000			No								
		Load Dose 2	09MAY2000			No								
		Load Dose 3	10MAY2000			No								
		Week 1	15MAY2000	15MAY2000	7	No	1	Neg						
		Week 1	15MAY2000	15MAY2000		No	2	Neg						
		Week 2	22MAY2000	22MAY2000	14	No	1	Pos	P.f	0.6	5.8	13.7	-15.7	
		Week 2	22MAY2000	22MAY2000		No	2	Pos	P.f	2.3	21.5			
		Week 3 (Fol. Up)	29MAY2000	29MAY2000	21	No	1	Neg						

*Popn=Population (I=Intent-To-Treat, PP=Per Protocol)
**Rel Day=Number of days relative to start of prophylactic medication
Symp = Symptoms, Rd = Reader, Res = Result, Spec = Species, Diff = Difference
Spec Count W = Species Count recorded per 500 White Blood Cells
Spec Count B = Species Count converted to per microlitre of blood
Gam = Gametocyte
+Arb=Arbitrator, +++Pos=Positive, Neg=Negative
#P. f = P.falciparum, P. v = P.vivax, P. o = P.ovale, P. m = P.malariae
Tafenoquine = Tafenoquine 200mg, Mefloquine = Mefloquine 250mg

Listing C.01

Results Of Blood Smears By Visit

Treatment : Tafenoquine

Subject Number	Popn *	Visit	Visit Date	Sample Date	Rel Day**	Symp ?	Rd+	Res +++	Spec #	Spec Count W	Spec Count B	Mean	Diff	Gam Count
030.001.00004	IPP	Week 3 (Fol. Up)	29MAY2000	29MAY2000		No	2	Neg						
		Week 3 (Unsch. Fol. Up)	01JUN2000			Yes								
		Week 4 (Fol. Up)	04JUN2000	04JUN2000	27	No	1	Neg						
		Week 4 (Fol. Up)	04JUN2000	04JUN2000		No	2	Neg						
		Week 5 (Fol. Up)	12JUN2000	12JUN2000	35	No	1	Pos	P.f	0.3	3.1	10.9	-15.5	
		Week 5 (Fol. Up)	12JUN2000	12JUN2000		No	2	Pos	P.f	2.0	18.6			
		Week 6 (Fol. Up)	19JUN2000	19JUN2000	42	Yes	1	Pos	P.f	0.2	1.7	5.8	-8.3	
		Week 6 (Fol. Up)	19JUN2000	19JUN2000		Yes	2	Neg						
		Week 6 (Fol. Up)	19JUN2000	19JUN2000		Yes	Arb	Pos	P.f	1.1	10.0			
		Week 6 (Unsch. Fol. Up)	19JUN2000	19JUN2000		Yes	1	Pos	P.f	0.5	5.1	4.2	1.7	
		Week 6 (Unsch. Fol. Up)	19JUN2000	19JUN2000		Yes	2	Pos	P.f	0.4	3.4			
		Week 6 (Unsch. Fol. Up)	21JUN2000			No								
030.001.00006	IPP	Screening	10APR2000	10APR2000	-28		1	Pos	P.f	22.3	178.6	302.1	-247.0	
		Screening	10APR2000	10APR2000			2	Pos	P.f	53.2	425.6			
		Post Clearance	06MAY2000	06MAY2000	-2		1	Neg						
		Post Clearance	06MAY2000	06MAY2000			2	Neg						
		Load Dose 1	08MAY2000			No								
		Load Dose 2	09MAY2000			No								
		Load Dose 3	10MAY2000			No								
		Week 1 (Unsch.)	14MAY2000			Yes								
		Week 1	15MAY2000	15MAY2000	7	Yes	1	Neg						
		Week 1	15MAY2000	15MAY2000		Yes	2	Neg						
		Week 2	22MAY2000	22MAY2000	14	No	1	Pos	P.f	0.8	11.6	23.9	-24.8	
		Week 2	22MAY2000	22MAY2000		No	2	Pos	P.f	2.4	36.3			
		Week 3 (Unsch. Fol. Up)	26MAY2000			Yes								
		Week 3 (Fol. Up)	29MAY2000	29MAY2000	21	Yes	1	Neg						
		Week 3 (Fol. Up)	29MAY2000	29MAY2000		Yes	2	Neg						
		Week 3 (Unsch. Fol. Up)	29MAY2000			Yes								
		Week 4 (Unsch. Fol. Up)	02JUN2000			Yes								
		Week 4 (Fol. Up)	05JUN2000	05JUN2000	28	No	1	Neg						
		Week 4 (Fol. Up)	05JUN2000	05JUN2000		No	2	Neg						
		Week 5 (Fol. Up)	12JUN2000	12JUN2000	35	No	1	Neg						
		Week 5 (Fol. Up)	12JUN2000	12JUN2000		No	2	Neg						
		Week 6 (Fol. Up)	19JUN2000	19JUN2000	42	No	1	Neg						
		Week 6 (Fol. Up)	19JUN2000	19JUN2000		No	2	Neg						

*Popn=Population (I=Intent-To-Treat, PP=Per Protocol)
**Rel Day=Number of days relative to start of prophylactic medication
Symp = Symptoms, Rd = Reader, Res = Result, Spec = Species, Diff = Difference
Spec Count W = Species Count recorded per 500 White Blood Cells
Spec Count B = Species Count converted to per microlitre of blood
Gam = Gametocyte
+Arb=Arbitrator, +++Pos=Positive, Neg=Negative
#P. f = P.falciparum, P. v = P.vivax, P. o = P.ovale, P. m = P.malariae
Tafenoquine = Tafenoquine 200mg, Mefloquine = Mefloquine 250mg

SB252263 - 030

Listing C.01

Results Of Blood Smears By Visit

Treatment : Tafenoquine

Subject Number	Popn *	Visit	Visit Date	Sample Date	Rel Day**	Symp ?	Rd+	Res +++	Spec #	Spec Count W	Spec Count B	Mean	Diff	Gam Count
030.001.00009	IPP	Screening	12APR2000	12APR2000	-26		1	Pos	P.f	2.0	19.6	27.9	-16.7	
		Screening	12APR2000	12APR2000			2	Neg						
		Screening	12APR2000	12APR2000			Arb	Pos	P.f	3.7	36.3			
		Screening (Unsch.)	20APR2000	20APR2000	-18		1	Neg						
		Screening (Unsch.)	20APR2000	20APR2000			2	Neg						
		Post Clearance	06MAY2000	06MAY2000	-2		1	Neg						
		Post Clearance	06MAY2000	06MAY2000			2	Neg						
		Load Dose 1	08MAY2000			No								
		Load Dose 2	09MAY2000			No								
		Load Dose 3	10MAY2000			No								
		Week 1	15MAY2000	15MAY2000	7	No	1	Neg						
		Week 1	15MAY2000	15MAY2000		No	2	Neg						
		Week 2	22MAY2000	22MAY2000	14	No	1	Neg						
		Week 2	22MAY2000	22MAY2000		No	2	Neg						
		Week 3	29MAY2000	29MAY2000	21	No	1	Neg						
		Week 3	29MAY2000	29MAY2000		No	2	Neg						
		Week 4	04JUN2000	04JUN2000	27	No	1	Neg						
		Week 4	04JUN2000	04JUN2000		No	2	Neg						
		Week 5	12JUN2000	12JUN2000	35	No	1	Neg						
		Week 5	12JUN2000	12JUN2000		No	2	Neg						
		Week 6	19JUN2000	19JUN2000	42	No	1	Neg						
		Week 6	19JUN2000	19JUN2000		No	2	Neg						
		Week 7	26JUN2000	26JUN2000	49	No	1	Pos	P.f	0.4	3.3	7.1	-7.7	
		Week 7	26JUN2000	26JUN2000		No	2	Pos	P.f	1.3	11.0			
		Week 8 (Fol. Up)	03JUL2000	03JUL2000	56	Yes	1	Neg						
		Week 8 (Fol. Up)	03JUL2000	03JUL2000		Yes	2	Neg						
		Week 9 (Fol. Up)	10JUL2000	10JUL2000	63	No	1	Neg						
		Week 9 (Fol. Up)	10JUL2000	10JUL2000		No	2	Neg						
		Week 10 (Fol. Up)	17JUL2000	17JUL2000	70	No	1	Neg						
		Week 10 (Fol. Up)	17JUL2000	17JUL2000		No	2	Neg						
		Week 11 (Fol. Up)	24JUL2000	24JUL2000	77	No	1	Neg						
		Week 11 (Fol. Up)	24JUL2000	24JUL2000		No	2	Neg						
030.001.00014	IPP	Screening	10APR2000	10APR2000	-28		1	Pos	P.f	52.6	536.8	1042.0	-1010.3	
		Screening	10APR2000	10APR2000			2	Pos	P.f	151.7	1547.1			
		Post Clearance	06MAY2000	06MAY2000	-2		1	Neg						
		Post Clearance	06MAY2000	06MAY2000			2	Neg						

*Popn=Population (I=Intent-To-Treat, PP=Per Protocol)
**Rel Day=Number of days relative to start of prophylactic medication
Symp = Symptoms, Rd = Reader, Res = Result, Spec = Species, Diff = Difference
Spec Count W = Species Count recorded per 500 White Blood Cells
Spec Count B = Species Count converted to per microlitre of blood
Gam = Gametocyte
+Arb=Arbitrator, +++Pos=Positive, Neg=Negative
#P. f = P.falciparum, P. v = P.vivax, P. o = P.ovale, P. m = P.malariae
Tafenoquine = Tafenoquine 200mg, Mefloquine = Mefloquine 250mg

SB252263 - 030

Listing C.01

Results Of Blood Smears By Visit

Treatment : Tafenoquine

Subject Number	Popn *	Visit	Visit Date	Sample Date	Rel Day**	Symp ?	Rd+	Res +++	Spec #	Spec Count W	Spec Count B	Mean	Diff	Gam Count
030.001.00014	IPP	Load Dose 1	08MAY2000			No								
		Load Dose 2	09MAY2000			No								
		Load Dose 3	10MAY2000			No								
		Week 1	15MAY2000	15MAY2000	7	No	1	Neg						
		Week 1	15MAY2000	15MAY2000		No	2	Neg						
		Week 2	22MAY2000	22MAY2000	14	No	1	Neg						
		Week 2	22MAY2000	22MAY2000		No	2	Neg						
		Week 3	29MAY2000	29MAY2000	21	No	1	Neg						
		Week 3	29MAY2000	29MAY2000		No	2	Neg						
		Week 4	05JUN2000	05JUN2000	28	Yes	1	Neg						
		Week 4	05JUN2000	05JUN2000		Yes	2	Neg						
		Week 4 (Unsch.)	07JUN2000			Yes								
		Week 5	12JUN2000	12JUN2000	35	No	1	Neg						
		Week 5	12JUN2000	12JUN2000		No	2	Neg						
		Week 6	19JUN2000	19JUN2000	42	No	1	Neg						
		Week 6	19JUN2000	19JUN2000		No	2	Neg						
		Week 6 (Unsch.)	19JUN2000			Yes								
		Week 7	26JUN2000	26JUN2000	49	No	1	Neg						
		Week 7	26JUN2000	26JUN2000		No	2	Neg						
		Week 8	03JUL2000	03JUL2000	56	No	1	Neg						
		Week 8	03JUL2000	03JUL2000		No	2	Neg						
		Week 9 (Unsch.)	10JUL2000			No								
		Week 9	10JUL2000	10JUL2000	63	No	1	Neg						
		Week 9	10JUL2000	10JUL2000		No	2	Neg						
		Week 10	17JUL2000	17JUL2000	70	No	1	Neg						
		Week 10	17JUL2000	17JUL2000		No	2	Neg						
		Week 11	24JUL2000	24JUL2000	77	No	1	Neg						
		Week 11	24JUL2000	24JUL2000		No	2	Neg						
		Week 12	31JUL2000	31JUL2000	84	No	1	Pos	P.f	3.8	45.8	41.2	9.2	
		Week 12	31JUL2000	31JUL2000		No	2	Neg						
		Week 12	31JUL2000	31JUL2000		No	Arb	Pos	P.f	3.0	36.6			
		Week 12 (Unsch. Fol. Up)	01AUG2000	01AUG2000	85	Yes	1	Neg						
		Week 12 (Unsch. Fol. Up)	01AUG2000	01AUG2000		Yes	2	Neg						
		Week 13 (Fol. Up)	07AUG2000	07AUG2000	91	No	1	Pos	P.f	6.5	79.2	69.2	19.9	
		Week 13 (Fol. Up)	07AUG2000	07AUG2000		No	2	Neg						
		Week 13 (Fol. Up)	07AUG2000	07AUG2000		No	Arb	Pos	P.f	4.9	59.3			
		Week 13 (Unsch. Fol. Up)	09AUG2000			No								

*Popn=Population (I=Intent-To-Treat, PP=Per Protocol)
**Rel Day=Number of days relative to start of prophylactic medication
Symp = Symptoms, Rd = Reader, Res = Result, Spec = Species, Diff = Difference
Spec Count W = Species Count recorded per 500 White Blood Cells
Spec Count B = Species Count converted to per microlitre of blood
Gam = Gametocyte
+Arb=Arbitrator, +++Pos=Positive, Neg=Negative
#P. f = P.falciparum, P. v = P.vivax, P. o = P.ovale, P. m = P.malariae
Tafenoquine = Tafenoquine 200mg, Mefloquine = Mefloquine 250mg

SB252263 - 030

Listing C.01

Results Of Blood Smears By Visit

Treatment : Tafenoquine

Subject Number	Popn *	Visit	Visit Date	Sample Date	Rel Day**	Symp ?	Rd+	Res +++	Spec #	Spec Count W	Spec Count B	Mean	Diff	Gam Count
030.001.00014	IPP	Week 14 (Fol. Up)	14AUG2000	14AUG2000	98	No	1	Neg						
		Week 14 (Fol. Up)	14AUG2000	14AUG2000		No	2	Neg						
		Week 15 (Fol. Up)	21AUG2000	21AUG2000	105	No	1	Neg						
		Week 15 (Fol. Up)	21AUG2000	21AUG2000		No	2	Neg						
		Week 16 (Fol. Up)	28AUG2000	28AUG2000	112		1	Neg						
		Week 16 (Fol. Up)	28AUG2000	28AUG2000			2	Neg						
030.001.00016	IPP	Screening	10APR2000	10APR2000	-28		1	Neg						
		Screening	10APR2000	10APR2000			2	Pos	P.f	1.0	7.4			
		Screening	10APR2000	10APR2000			Arb	Neg						
		Screening (Unsch.)	28APR2000			Yes								
		Post Clearance	06MAY2000	06MAY2000	-2		1	Neg						
		Post Clearance	06MAY2000	06MAY2000			2	Neg						
		Load Dose 1	08MAY2000			No								
		Load Dose 2	09MAY2000			No								
		Load Dose 3	10MAY2000			No								
		Load Dose (Unsch.)	10MAY2000			No								
		Week 1	15MAY2000	15MAY2000	7	No	1	Neg						
		Week 1	15MAY2000	15MAY2000		No	2	Neg						
		Week 1 (Unsch.)	18MAY2000			Yes								
		Week 2 (Unsch.)	19MAY2000			No								
		Week 2	22MAY2000	22MAY2000	14	No	1	Neg						
		Week 2	22MAY2000	22MAY2000		No	2	Neg						
		Week 3	31MAY2000	31MAY2000	23	Yes	1	Neg						
		Week 3	31MAY2000	31MAY2000		Yes	2	Neg						
		Week 4	05JUN2000	05JUN2000	28	No	1	Neg						
		Week 4	05JUN2000	05JUN2000		No	2	Neg						
		Week 5	12JUN2000	12JUN2000	35	No	1	Neg						
		Week 5	12JUN2000	12JUN2000		No	2	Neg						
		Week 6 (Unsch.)	17JUN2000			Yes								
		Week 6	19JUN2000	19JUN2000	42	No	1	Neg						
		Week 6	19JUN2000	19JUN2000		No	2	Neg						
		Week 7 (Unsch.)	24JUN2000			Yes								
		Week 7	26JUN2000	26JUN2000	49	No	1	Neg						
		Week 7	26JUN2000	26JUN2000		No	2	Neg						
		Week 8	03JUL2000	03JUL2000	56	No	1	Neg						
		Week 8	03JUL2000	03JUL2000		No	2	Neg						

*Popn=Population (I=Intent-To-Treat, PP=Per Protocol)
**Rel Day=Number of days relative to start of prophylactic medication
Symp = Symptoms, Rd = Reader, Res = Result, Spec = Species, Diff = Difference
Spec Count W = Species Count recorded per 500 White Blood Cells
Spec Count B = Species Count converted to per microlitre of blood
Gam = Gametocyte
+Arb=Arbitrator, +++Pos=Positive, Neg=Negative
#P. f = P.falciparum, P. v = P.vivax, P. o = P.ovale, P. m = P.malariae
Tafenoquine = Tafenoquine 200mg, Mefloquine = Mefloquine 250mg

Listing C.01

Results Of Blood Smears By Visit

Treatment : Tafenoquine

Subject Number	Popn *	Visit	Visit Date	Sample Date	Rel Day**	Symp ?	Rd+	Res +++	Spec #	Spec Count W	Spec Count B	Mean	Diff	Gam Count
030.001.00016	IPP	Week 9	10JUL2000	10JUL2000	63	No	1	Neg						
		Week 9	10JUL2000	10JUL2000		No	2	Neg						
		Week 10	18JUL2000	18JUL2000	71	Yes	1	Neg						
		Week 10	18JUL2000	18JUL2000		Yes	2	Neg						
		Week 11	24JUL2000	24JUL2000	77	No	1	Neg						
		Week 11	24JUL2000	24JUL2000		No	2	Neg						
		Week 12	31JUL2000	31JUL2000	84	Yes	1	Neg						
		Week 12	31JUL2000	31JUL2000		Yes	2	Neg						
		Week 13	07AUG2000	07AUG2000	91	No	1	Neg						
		Week 13	07AUG2000	07AUG2000		No	2	Pos	P.f	0.2	1.4	2.6	-2.5	
		Week 13	07AUG2000	07AUG2000		No	Arb	Pos	P.f	0.5	3.9			
		Week 14 (Fol. Up)	14AUG2000	14AUG2000	98	No	1	Neg						
		Week 14 (Fol. Up)	14AUG2000	14AUG2000		No	2	Neg						
		Week 15 (Fol. Up)	23AUG2000	23AUG2000	107	No	1	Neg						
		Week 15 (Fol. Up)	23AUG2000	23AUG2000		No	2	Neg						
		Week 16 (Fol. Up)	28AUG2000	28AUG2000	112	No	1	Neg						
		Week 16 (Fol. Up)	28AUG2000	28AUG2000		No	2	Neg						
		Week 17 (Fol. Up)	04SEP2000	04SEP2000	119		1	Neg						
		Week 17 (Fol. Up)	04SEP2000	04SEP2000			2	Neg						
030.001.00017	IPP	Screening	17APR2000	17APR2000	-21		1	Neg						
		Screening	17APR2000	17APR2000			2	Neg						
		Screening (Unsch.)	28APR2000			Yes								
		Post Clearance	07MAY2000	07MAY2000	-1		1	Neg						
		Post Clearance	07MAY2000	07MAY2000			2	Neg						
		Load Dose 1	08MAY2000			No								
		Load Dose 2	09MAY2000			No								
		Load Dose 3	10MAY2000			No								
		Week 1	17MAY2000	17MAY2000	9	No	1	Neg						
		Week 1	17MAY2000	17MAY2000		No	2	Neg						
		Week 2	23MAY2000	23MAY2000	15	No	1	Neg						
		Week 2	23MAY2000	23MAY2000		No	2	Neg						
		Week 3	29MAY2000	29MAY2000	21	No	1	Neg						
		Week 3	29MAY2000	29MAY2000		No	2	Neg						
		Week 3 (Unsch.)	30MAY2000			Yes								
		Week 4	05JUN2000	05JUN2000	28	No	1	Neg						
		Week 4	05JUN2000	05JUN2000		No	2	Neg						

*Popn=Population (I=Intent-To-Treat, PP=Per Protocol)
**Rel Day=Number of days relative to start of prophylactic medication
Symp = Symptoms, Rd = Reader, Res = Result, Spec = Species, Diff = Difference
Spec Count W = Species Count recorded per 500 White Blood Cells
Spec Count B = Species Count converted to per microlitre of blood
Gam = Gametocyte
+Arb=Arbitrator, +++Pos=Positive, Neg=Negative
#P. f = P.falciparum, P. v = P.vivax, P. o = P.ovale, P. m = P.malariae
Tafenoquine = Tafenoquine 200mg, Mefloquine = Mefloquine 250mg

Listing C.01

Results Of Blood Smears By Visit

Treatment : Tafenoquine

Subject Number	Popn *	Visit	Visit Date	Sample Date	Rel Day**	Symp ?	Rd+	Res +++	Spec #	Spec Count W	Spec Count B	Mean	Diff	Gam Count
030.001.00017	IPP	Week 5	13JUN2000	13JUN2000	36	Yes	1	Neg						
		Week 5	13JUN2000	13JUN2000		Yes	2	Neg						
		Week 6 (Unsch.)	16JUN2000			No								
		Week 6	18JUN2000	18JUN2000	41	Yes	1	Neg						
		Week 6	18JUN2000	18JUN2000		Yes	2	Neg						
		Week 6 (Unsch.)	18JUN2000			Yes								
		Week 7	26JUN2000	26JUN2000	49	No	1	Neg						
		Week 7	26JUN2000	26JUN2000		No	2	Neg						
		Week 9	07JUL2000	07JUL2000	60	No	1	Neg						
		Week 9	07JUL2000	07JUL2000		No	2	Neg						
		Week 9 (Unsch.)	07JUL2000			No								
		Week 9	11JUL2000	10JUL2000	63	Yes	1	Neg						
		Week 9	11JUL2000	10JUL2000		Yes	2	Neg						
		Week 9 (Unsch.)	11JUL2000			No								
		Week 10	17JUL2000	17JUL2000	70	No	1	Neg						
		Week 10	17JUL2000	17JUL2000		No	2	Neg						
		Week 11	24JUL2000	24JUL2000	77	No	1	Neg						
		Week 11	24JUL2000	24JUL2000		No	2	Neg						
		Week 11 (Unsch.)	24JUL2000			No								
		Week 12	01AUG2000	01AUG2000	85	No	1	Neg						
		Week 12	01AUG2000	01AUG2000		No	2	Neg						
		Week 12 (Unsch.)	01AUG2000			No								
		Week 14	11AUG2000	11AUG2000	95	No	1	Neg						
		Week 14	11AUG2000	11AUG2000		No	2	Neg						
		Week 14	14AUG2000	14AUG2000	98	Yes	1	Neg						
		Week 14	14AUG2000	14AUG2000		Yes	2	Neg						
		Week 14 (Unsch.)	14AUG2000			Yes								
		Week 15	21AUG2000	21AUG2000	105	No	1	Neg						
		Week 15	21AUG2000	21AUG2000		No	2	Neg						
		Week 16	28AUG2000	28AUG2000	112	Yes	1	Neg						
		Week 16	28AUG2000	28AUG2000		Yes	2	Neg						
		Week 16 (Unsch.)	28AUG2000	28AUG2000		Yes	1	Neg						
		Week 16 (Unsch.)	28AUG2000	28AUG2000		Yes	2	Neg						
		Week 17	04SEP2000	04SEP2000	119	No	1	Neg						
		Week 17	04SEP2000	04SEP2000		No	2	Neg						
		Week 18	11SEP2000	11SEP2000	126	Yes	1	Neg						
		Week 18	11SEP2000	11SEP2000		Yes	2	Neg						

*Popn=Population (I=Intent-To-Treat, PP=Per Protocol)
**Rel Day=Number of days relative to start of prophylactic medication
Symp = Symptoms, Rd = Reader, Res = Result, Spec = Species, Diff = Difference
Spec Count W = Species Count recorded per 500 White Blood Cells
Spec Count B = Species Count converted to per microlitre of blood
Gam = Gametocyte
+Arb=Arbitrator, +++Pos=Positive, Neg=Negative
#P. f = P.falciparum, P. v = P.vivax, P. o = P.ovale, P. m = P.malariae
Tafenoquine = Tafenoquine 200mg, Mefloquine = Mefloquine 250mg

Listing C.01

Results Of Blood Smears By Visit

Treatment : Tafenoquine

Subject Number	Popn *	Visit	Visit Date	Sample Date	Rel Day**	Symp ?	Rd+	Res +++	Spec #	Spec Count W	Spec Count B	Mean	Diff	Gam Count
030.001.00017	IPP	Week 19	18SEP2000	18SEP2000	133	No	1	Pos	P.f	1.3	10.2	6.7	7.0	
		Week 19	18SEP2000	18SEP2000		No	2	Neg						
		Week 19	18SEP2000	18SEP2000		No	Arb	Pos	P.f	0.4	3.2			
		Week 20 (Fol. Up)	28SEP2000	28SEP2000	143	No	1	Neg						
		Week 20 (Fol. Up)	28SEP2000	28SEP2000		No	2	Neg						
		Week 22 (Fol. Up)	08OCT2000	08OCT2000	153	No	1	Pos	P.f	12.1	89.7	74.4	30.5	
		Week 22 (Fol. Up)	08OCT2000	08OCT2000		No	2	Pos	P.f	8.0	59.2			
		Week 22 (Unsch. Fol. Up)	08OCT2000			Yes								
		Week 22 (Fol. Up)	11OCT2000	11OCT2000	156	Yes	1	Neg						
		Week 22 (Fol. Up)	11OCT2000	11OCT2000		Yes	2	Neg						
		Week 22 (Unsch. Fol. Up)	11OCT2000			No								
030.001.00024	IPP	Screening	11APR2000	11APR2000	-27		1	Neg						
		Screening	11APR2000	11APR2000			2	Neg						
		Screening (Unsch.)	29APR2000	29APR2000	-9		1	Neg						
		Screening (Unsch.)	29APR2000	29APR2000			2	Neg						
		Post Clearance	06MAY2000	06MAY2000	-2		1	Neg						
		Post Clearance	06MAY2000	06MAY2000			2	Neg						
		Load Dose 1	08MAY2000			No								
		Load Dose 2	09MAY2000			No								
		Load Dose 3	10MAY2000			No								
		Week 1	17MAY2000	18MAY2000	10	No	1	Neg						
		Week 1	17MAY2000	18MAY2000		No	2	Neg						
		Week 2	23MAY2000	23MAY2000	15	No	1	Neg						
		Week 2	23MAY2000	23MAY2000		No	2	Neg						
		Week 3	29MAY2000	29MAY2000	21	No	1	Neg						
		Week 3	29MAY2000	29MAY2000		No	2	Neg						
		Week 4	08JUN2000	08JUN2000	31	Yes	1	Neg						
		Week 4	08JUN2000	08JUN2000		Yes	2	Neg						
		Week 5	12JUN2000	12JUN2000	35		1	Pos	P.o	0.3	3.1			
		Week 5	12JUN2000	12JUN2000		Yes	2	Neg						
		Week 5	12JUN2000	12JUN2000		Yes	Arb	Neg						
		Week 5 (Unsch.)	12JUN2000			Yes								
		Week 6	19JUN2000	19JUN2000	42	No	1	Pos	P.f	1.3	11.8	27.9	-32.2	
		Week 6	19JUN2000	19JUN2000		No	2	Pos	P.f	5.0	44.0			
		Week 7 (Fol. Up)	26JUN2000	26JUN2000	49	No	1	Neg						
		Week 7 (Fol. Up)	26JUN2000	26JUN2000		No	2	Neg						

*Popn=Population (I=Intent-To-Treat, PP=Per Protocol)
**Rel Day=Number of days relative to start of prophylactic medication
Symp = Symptoms, Rd = Reader, Res = Result, Spec = Species, Diff = Difference
Spec Count W = Species Count recorded per 500 White Blood Cells
Spec Count B = Species Count converted to per microlitre of blood
Gam = Gametocyte
+Arb=Arbitrator, +++Pos=Positive, Neg=Negative
#P. f = P.falciparum, P. v = P.vivax, P. o = P.ovale, P. m = P.malariae
Tafenoquine = Tafenoquine 200mg, Mefloquine = Mefloquine 250mg

SB252263 - 030

Listing C.01

Results Of Blood Smears By Visit

Treatment : Tafenoquine

Subject Number	Popn *	Visit	Visit Date	Sample Date	Rel Day**	Symp ?	Rd+	Res +++	Spec #	Spec Count W	Spec Count B	Mean	Diff	Gam Count
030.001.00024	IPP	Week 7 (Unsch. Fol. Up)	27JUN2000			Yes								
		Week 8 (Fol. Up)	03JUL2000	03JUL2000	56	Yes	1	Neg						
		Week 8 (Fol. Up)	03JUL2000	03JUL2000		Yes	2	Neg						
		Week 9 (Fol. Up)	13JUL2000	13JUL2000	66	No	1	Neg						
		Week 9 (Fol. Up)	13JUL2000	13JUL2000		No	2	Neg						
		Week 10 (Fol. Up)	18JUL2000	18JUL2000	71		1	Neg						
		Week 10 (Fol. Up)	18JUL2000	18JUL2000			2	Neg						
030.001.00025	IPP	Screening	11APR2000	11APR2000	-27		1	Neg						
		Screening	11APR2000	11APR2000			2	Neg						
		Screening (Unsch.)	03MAY2000			No								
		Post Clearance	06MAY2000	06MAY2000	-2		1	Neg						
		Post Clearance	06MAY2000	06MAY2000			2	Neg						
		Load Dose 1	08MAY2000			No								
		Load Dose 2	09MAY2000			No								
		Load Dose 3	10MAY2000			No								
		Load Dose (Unsch.)	10MAY2000			Yes								
		Week 1 (Unsch.)	11MAY2000			No								
		Week 1	15MAY2000	15MAY2000	7	Yes	1	Neg						
		Week 1	15MAY2000	15MAY2000		Yes	2	Neg						
		Week 2	22MAY2000	22MAY2000	14	Yes	1	Neg						
		Week 2	22MAY2000	22MAY2000		Yes	2	Neg						
		Week 3 (Unsch.)	28MAY2000			Yes								
		Week 3	29MAY2000	29MAY2000	21	No	1	Neg						
		Week 3	29MAY2000	29MAY2000		No	2	Neg						
		Week 4	04JUN2000	04JUN2000	27	No	1	Neg						
		Week 4	04JUN2000	04JUN2000		No	2	Neg						
		Week 4 (Unsch.)	08JUN2000			Yes								
		Week 5	12JUN2000	12JUN2000	35	Yes	1	Neg						
		Week 5	12JUN2000	12JUN2000		Yes	2	Neg						
		Week 5	12JUN2000	12JUN2000		Yes	Arb	Neg						
		Week 6	19JUN2000	19JUN2000	42	No	1	Neg						
		Week 6	19JUN2000	19JUN2000		No	2	Neg						
		Week 7 (Unsch.)	25JUN2000			Yes								
		Week 7	26JUN2000	26JUN2000	49	No	1	Neg						
		Week 7	26JUN2000	26JUN2000		No	2	Neg						
		Week 8	03JUL2000	03JUL2000	56	No	1	Neg						

*Popn=Population (I=Intent-To-Treat, PP=Per Protocol)
**Rel Day=Number of days relative to start of prophylactic medication
Symp = Symptoms, Rd = Reader, Res = Result, Spec = Species, Diff = Difference
Spec Count W = Species Count recorded per 500 White Blood Cells
Spec Count B = Species Count converted to per microlitre of blood
Gam = Gametocyte
+Arb=Arbitrator, +++Pos=Positive, Neg=Negative
#P. f = P.falciparum, P. v = P.vivax, P. o = P.ovale, P. m = P.malariae
Tafenoquine = Tafenoquine 200mg, Mefloquine = Mefloquine 250mg

SB252263 - 030

Listing C.01

Results Of Blood Smears By Visit

Treatment : Tafenoquine

Subject Number	Popn *	Visit	Visit Date	Sample Date	Rel Day**	Symp ?	Rd+	Res +++	Spec #	Spec Count W	Spec Count B	Mean	Diff	Gam Count
030.001.00025	IPP	Week 8	03JUL2000	03JUL2000		No	2	Neg						
		Week 9	10JUL2000	10JUL2000	63	Yes	1	Neg						
		Week 9	10JUL2000	10JUL2000		Yes	2	Neg						
		Week 10	17JUL2000	17JUL2000	70	No	1	Neg						
		Week 10	17JUL2000	17JUL2000		No	2	Neg						
		Week 11	24JUL2000	24JUL2000	77	No	1	Neg						
		Week 11	24JUL2000	24JUL2000		No	2	Neg						
		Week 12	31JUL2000	31JUL2000	84	No	1	Neg						
		Week 12	31JUL2000	31JUL2000		No	2	Neg						
		Week 13	07AUG2000	07AUG2000	91	No	1	Pos	P.f	2.8	23.3	20.8	4.9	
		Week 13	07AUG2000	07AUG2000		No	2	Neg						
		Week 13	07AUG2000	07AUG2000		No	Arb	Pos	P.f	2.2	18.4			
		Week 15 (Fol. Up)	24AUG2000	24AUG2000	108	No	1	Neg						
		Week 15 (Fol. Up)	24AUG2000	24AUG2000		No	2	Neg						
		Week 16 (Fol. Up)	28AUG2000	28AUG2000	112	No	1	Neg						
		Week 16 (Fol. Up)	28AUG2000	28AUG2000		No	2	Neg						
		Week 16 (Fol. Up)	31AUG2000	31AUG2000	115	No	1	Neg						
		Week 16 (Fol. Up)	31AUG2000	31AUG2000		No	2	Neg						
		Week 17 (Fol. Up)	04SEP2000	04SEP2000	119		1	Neg						
		Week 17 (Fol. Up)	04SEP2000	04SEP2000			2	Neg						
030.001.00028	IPP	Screening	11APR2000	11APR2000	-27		1	Neg						
		Screening	11APR2000	11APR2000			2	Neg						
		Screening (Unsch.)	20APR2000			Yes								
		Screening (Unsch.)	02MAY2000			Yes								
		Post Clearance	06MAY2000	06MAY2000	-2		1	Neg						
		Post Clearance	06MAY2000	06MAY2000			2	Neg						
		Load Dose 1	08MAY2000			No								
		Load Dose 2	09MAY2000			No								
		Load Dose 3	10MAY2000			No								
		Load Dose (Unsch.)	10MAY2000			No								
		Week 1	15MAY2000	15MAY2000	7	No	1	Neg						
		Week 1	15MAY2000	15MAY2000		No	2	Neg						
		Week 2 (Unsch.)	21MAY2000			No								
		Week 2	22MAY2000	22MAY2000	14	Yes	1	Neg						
		Week 2	22MAY2000	22MAY2000		Yes	2	Neg						
		Week 2 (Unsch.)	22MAY2000			Yes								

*Popn=Population (I=Intent-To-Treat, PP=Per Protocol)
**Rel Day=Number of days relative to start of prophylactic medication
Symp = Symptoms, Rd = Reader, Res = Result, Spec = Species, Diff = Difference
Spec Count W = Species Count recorded per 500 White Blood Cells
Spec Count B = Species Count converted to per microlitre of blood
Gam = Gametocyte
+Arb=Arbitrator, +++Pos=Positive, Neg=Negative
#P. f = P.falciparum, P. v = P.vivax, P. o = P.ovale, P. m = P.malariae
Tafenoquine = Tafenoquine 200mg, Mefloquine = Mefloquine 250mg

Listing C.01

Results Of Blood Smears By Visit

Treatment : Tafenoquine

Subject Number	Popn *	Visit	Visit Date	Sample Date	Rel Day**	Symp ?	Rd+	Res +++	Spec #	Spec Count W	Spec Count B	Mean	Diff	Gam Count
030.001.00028	IPP	Week 2 (Unsch.)	23MAY2000			No								
		Week 3	29MAY2000	29MAY2000	21	No	1	Pos	P.f	0.5	6.8			
		Week 3	29MAY2000	29MAY2000		No	2	Neg						
		Week 3	29MAY2000	29MAY2000		No	Arb	Neg						
		Week 4	06JUN2000	06JUN2000	29	No	1	Neg						
		Week 4	06JUN2000	06JUN2000		No	2	Neg						
		Week 5	12JUN2000	12JUN2000	35	No	1	Neg						
		Week 5	12JUN2000	12JUN2000		No	2	Neg						
		Week 6	19JUN2000	19JUN2000	42	No	1	Neg						
		Week 6	19JUN2000	19JUN2000		No	2	Neg						
		Week 7	26JUN2000	26JUN2000	49	No	1	Neg						
		Week 7	26JUN2000	26JUN2000		No	2	Neg						
		Week 8	03JUL2000	03JUL2000	56	No	1	Neg						
		Week 8	03JUL2000	03JUL2000		No	2	Neg						
		Week 9	10JUL2000	10JUL2000	63	Yes	1	Neg						
		Week 9	10JUL2000	10JUL2000		Yes	2	Neg						
		Week 10	17JUL2000	17JUL2000	70	Yes	1	Pos	P.f	0.5	6.1	30.2	-48.1	
		Week 10	17JUL2000	17JUL2000		Yes	2	Pos	P.f	4.2	54.2			
		Week 10 (Unsch.)	17JUL2000			Yes								
		Week 10 (Unsch. Fol. Up)	18JUL2000			No								
		Week 10 (Unsch. Fol. Up)	19JUL2000			No								
		Week 11 (Unsch. Fol. Up)	21JUL2000			No								
		Week 11 (Fol. Up)	24JUL2000	24JUL2000	77	No	1	Neg						
		Week 11 (Fol. Up)	24JUL2000	24JUL2000		No	2	Neg						
		Week 12 (Fol. Up)	31JUL2000	31JUL2000	84	No	1	Neg						
		Week 12 (Fol. Up)	31JUL2000	31JUL2000		No	2	Neg						
		Week 13 (Unsch. Fol. Up)	04AUG2000			No								
		Week 13 (Fol. Up)	07AUG2000	07AUG2000	91	No	1	Pos	P.f	3.2	41.3	31.8	19.1	
		Week 13 (Fol. Up)	07AUG2000	07AUG2000		No	2	Neg						
		Week 13 (Fol. Up)	07AUG2000	07AUG2000		No	Arb	Pos	P.f	1.7	22.2			
		Week 14 (Unsch. Fol. Up)	14AUG2000	14AUG2000	98	No	1	Neg						
		Week 14 (Unsch. Fol. Up)	14AUG2000	14AUG2000		No	2	Neg						
030.001.00029	IPP	Screening	11APR2000	11APR2000	-27		1	Pos	P.f	7.6	85.0	64.1	41.8	
		Screening	11APR2000	11APR2000			2	Pos	P.f	3.9	43.2			
		Post Clearance	06MAY2000	06MAY2000	-2		1	Neg						
		Post Clearance	06MAY2000	06MAY2000			2	Neg						

*Popn=Population (I=Intent-To-Treat, PP=Per Protocol)
**Rel Day=Number of days relative to start of prophylactic medication
Symp = Symptoms, Rd = Reader, Res = Result, Spec = Species, Diff = Difference
Spec Count W = Species Count recorded per 500 White Blood Cells
Spec Count B = Species Count converted to per microlitre of blood
Gam = Gametocyte
+Arb=Arbitrator, +++Pos=Positive, Neg=Negative
#P. f = P.falciparum, P. v = P.vivax, P. o = P.ovale, P. m = P.malariae
Tafenoquine = Tafenoquine 200mg, Mefloquine = Mefloquine 250mg

SB252263 - 030

Listing C.01

Results Of Blood Smears By Visit

Treatment : Tafenoquine

Subject Number	Popn *	Visit	Visit Date	Sample Date	Rel Day**	Symp ?	Rd+	Res +++	Spec #	Spec Count W	Spec Count B	Mean	Diff	Gam Count
030.001.00029	IPP	Load Dose 1	08MAY2000			No								
		Load Dose 2	09MAY2000			No								
		Load Dose 3	10MAY2000			No								
		Week 1	15MAY2000	15MAY2000	7	No	1	Neg						
		Week 1	15MAY2000	15MAY2000		No	2	Neg						
		Week 2	22MAY2000	22MAY2000	14	No	1	Neg						
		Week 2	22MAY2000	22MAY2000		No	2	Neg						
		Week 3	29MAY2000	29MAY2000	21	No	1	Neg						
		Week 3	29MAY2000	29MAY2000		No	2	Neg						
		Week 4	05JUN2000	05JUN2000	28	No	1	Neg						
		Week 4	05JUN2000	05JUN2000		No	2	Neg						
		Week 5	12JUN2000	12JUN2000	35	No	1	Neg						
		Week 5	12JUN2000	12JUN2000		No	2	Neg						
		Week 6	19JUN2000	19JUN2000	42	No	1	Neg						
		Week 6	19JUN2000	19JUN2000		No	2	Neg						
		Week 6 (Unsch.)	19JUN2000			No								
		Week 7	26JUN2000	26JUN2000	49	No	1	Neg						
		Week 7	26JUN2000	26JUN2000		No	2	Neg						
		Week 8	03JUL2000	03JUL2000	56	Yes	1	Pos	P.f	0.2	3.3	2.7	1.1	
		Week 8	03JUL2000	03JUL2000		Yes	2	Pos	P.f	0.1	2.1			
		Week 9 (Fol. Up)	10JUL2000	10JUL2000	63	No	1	Neg						
		Week 9 (Fol. Up)	10JUL2000	10JUL2000		No	2	Neg						
		Week 10 (Fol. Up)	18JUL2000	18JUL2000	71	No	1	Neg						
		Week 10 (Fol. Up)	18JUL2000	18JUL2000		No	2	Neg						
		Week 11 (Fol. Up)	24JUL2000	24JUL2000	77	No	1	Neg						
		Week 11 (Fol. Up)	24JUL2000	24JUL2000		No	2	Neg						
		Week 12 (Fol. Up)	01AUG2000	01AUG2000	85		1	Neg						
		Week 12 (Fol. Up)	01AUG2000	01AUG2000			2	Neg						
030.001.00030	IPP	Screening	14APR2000	14APR2000	-24		1	Neg						
		Screening	14APR2000	14APR2000			2	Neg						
		Screening (Unsch.)	26APR2000	26APR2000	-12	Yes	1	Neg						
		Screening (Unsch.)	26APR2000	26APR2000		Yes	2	Neg						
		Clearance (Unsch.)	04MAY2000			No								
		Post Clearance	06MAY2000	06MAY2000	-2		1	Neg						
		Post Clearance	06MAY2000	06MAY2000			2	Neg						
		Post Clearance (Unsch.)	06MAY2000			No								

*Popn=Population (I=Intent-To-Treat, PP=Per Protocol)
**Rel Day=Number of days relative to start of prophylactic medication
Symp = Symptoms, Rd = Reader, Res = Result, Spec = Species, Diff = Difference
Spec Count W = Species Count recorded per 500 White Blood Cells
Spec Count B = Species Count converted to per microlitre of blood
Gam = Gametocyte
+Arb=Arbitrator, +++Pos=Positive, Neg=Negative
#P. f = P.falciparum, P. v = P.vivax, P. o = P.ovale, P. m = P.malariae
Tafenoquine = Tafenoquine 200mg, Mefloquine = Mefloquine 250mg

SB252263 - 030

Listing C.01

Results Of Blood Smears By Visit

Treatment : Tafenoquine

Subject Number	Popn *	Visit	Visit Date	Sample Date	Rel Day**	Symp ?	Rd+	Res +++	Spec #	Spec Count W	Spec Count B	Mean	Diff	Gam Count
030.001.00030	IPP	Load Dose 1	08MAY2000			No								
		Load Dose 2	09MAY2000			No								
		Load Dose 3	10MAY2000			No								
		Week 1	15MAY2000	15MAY2000	7	No	1	Neg						
		Week 1	15MAY2000	15MAY2000		No	2	Neg						
		Week 2	22MAY2000	22MAY2000	14	No	1	Neg						
		Week 2	22MAY2000	22MAY2000		No	2	Neg						
		Week 2 (Unsch.)	22MAY2000			Yes								
		Week 3	29MAY2000	29MAY2000	21	No	1	Neg						
		Week 3	29MAY2000	29MAY2000		No	2	Neg						
		Week 3 (Unsch.)	29MAY2000			Yes								
		Week 4	05JUN2000	05JUN2000	28	No	1	Neg						
		Week 4	05JUN2000	05JUN2000		No	2	Neg						
		Week 5 (Unsch.)	09JUN2000			Yes								
		Week 5	12JUN2000	12JUN2000	35	Yes	1	Neg						
		Week 5	12JUN2000	12JUN2000		Yes	2	Neg						
		Week 6	19JUN2000	19JUN2000	42	No	1	Neg						
		Week 6	19JUN2000	19JUN2000		No	2	Neg						
		Week 7	26JUN2000	26JUN2000	49	Yes	1	Neg						
		Week 7	26JUN2000	26JUN2000		Yes	2	Neg						
		Week 8	03JUL2000	03JUL2000	56	Yes	1	Neg						
		Week 8	03JUL2000	03JUL2000		Yes	2	Neg						
		Week 9	10JUL2000	10JUL2000	63	No	1	Pos	P.f	9.3	125.8	95.1	61.5	
		Week 9	10JUL2000	10JUL2000		No	2	Neg						
		Week 9	10JUL2000	10JUL2000		No	Arb	Pos	P.f	4.7	64.3			
		Week 10 (Fol. Up)	17JUL2000	17JUL2000	70	No	1	Neg						
		Week 10 (Fol. Up)	17JUL2000	17JUL2000		No	2	Neg						
		Week 11 (Fol. Up)	24JUL2000	24JUL2000	77	Yes	1	Neg						
		Week 11 (Fol. Up)	24JUL2000	24JUL2000		Yes	2	Neg						
		Week 11 (Unsch. Fol. Up)	24JUL2000			No								
		Week 12 (Fol. Up)	31JUL2000	31JUL2000	84	Yes	1	Neg						
		Week 12 (Fol. Up)	31JUL2000	31JUL2000		Yes	2	Neg						
		Week 13 (Fol. Up)	07AUG2000	07AUG2000	91		1	Neg						
		Week 13 (Fol. Up)	07AUG2000	07AUG2000			2	Neg						
		Week 13 (Unsch. Fol. Up)	07AUG2000			No								
030.001.00040	IPP	Screening	13APR2000	13APR2000	-25		1	Neg						

*Popn=Population (I=Intent-To-Treat, PP=Per Protocol)
**Rel Day=Number of days relative to start of prophylactic medication
Symp = Symptoms, Rd = Reader, Res = Result, Spec = Species, Diff = Difference
Spec Count W = Species Count recorded per 500 White Blood Cells
Spec Count B = Species Count converted to per microlitre of blood
Gam = Gametocyte
+Arb=Arbitrator, +++Pos=Positive, Neg=Negative
#P. f = P.falciparum, P. v = P.vivax, P. o = P.ovale, P. m = P.malariae
Tafenoquine = Tafenoquine 200mg, Mefloquine = Mefloquine 250mg

SB252263 - 030

Listing C.01

Results Of Blood Smears By Visit

Treatment : Tafenoquine

Subject Number	Popn *	Visit	Visit Date	Sample Date	Rel Day**	Symp ?	Rd+	Res +++	Spec #	Spec Count W	Spec Count B	Mean	Diff	Gam Count
030.001.00040	IPP	Screening	13APR2000	13APR2000			2	Neg						
		Post Clearance	06MAY2000	06MAY2000	-2		1	Neg						
		Post Clearance	06MAY2000	06MAY2000			2	Neg						
		Post Clearance (Unsch.)	06MAY2000			Yes								
		Load Dose 1	08MAY2000			No								
		Load Dose 2	09MAY2000			No								
		Load Dose 3	10MAY2000			No								
		Week 1	15MAY2000	15MAY2000	7	No	1	Neg						
		Week 1	15MAY2000	15MAY2000		No	2	Neg						
		Week 2 (Unsch.)	21MAY2000			No								
		Week 2	22MAY2000	22MAY2000	14	No	1	Pos	P.f	3.0	33.6	20.4	26.3	
		Week 2	22MAY2000	22MAY2000		No	2	Neg						
		Week 2	22MAY2000	22MAY2000		No	Arb	Pos	P.f	0.7	7.3			
		Week 3 (Fol. Up)	29MAY2000	29MAY2000	21	Yes	1	Neg						
		Week 3 (Fol. Up)	29MAY2000	29MAY2000		Yes	2	Neg						
		Week 4 (Fol. Up)	04JUN2000	04JUN2000	27	Yes	1	Neg						
		Week 4 (Fol. Up)	04JUN2000	04JUN2000		Yes	2	Neg						
		Week 5 (Fol. Up)	12JUN2000	12JUN2000	35	No	1	Neg						
		Week 5 (Fol. Up)	12JUN2000	12JUN2000		No	2	Neg						
		Week 6 (Fol. Up)	19JUN2000	19JUN2000	42	No	1	Pos	P.f	1.5	22.2	22.2	0.0	
		Week 6 (Fol. Up)	19JUN2000	19JUN2000		No	2	Neg						
		Week 6 (Fol. Up)	19JUN2000	19JUN2000		No	Arb	Pos	P.f	1.5	22.2			
		Week 6 (Unsch. Fol. Up)	21JUN2000			No								
030.001.00042	IPP	Screening	10APR2000	10APR2000	-28		1	Pos	P.f	2.4	39.8	35.5	8.6	
		Screening	10APR2000	10APR2000			2	Pos	P.f	1.9	31.2			
		Screening (Unsch.)	17APR2000			No								
		Screening (Unsch.)	27APR2000			Yes								
		Clearance (Unsch.)	05MAY2000			Yes								
		Post Clearance	06MAY2000	06MAY2000	-2		1	Neg						
		Post Clearance	06MAY2000	06MAY2000			2	Neg						
		Load Dose 1	08MAY2000			No								
		Load Dose 2	09MAY2000			No								
		Load Dose (Unsch.)	09MAY2000			No								
		Load Dose 3	10MAY2000			No								
		Week 1 (Unsch.)	14MAY2000			No								
		Week 1	15MAY2000	15MAY2000	7	No	1	Neg						

*Popn=Population (I=Intent-To-Treat, PP=Per Protocol)
**Rel Day=Number of days relative to start of prophylactic medication
Symp = Symptoms, Rd = Reader, Res = Result, Spec = Species, Diff = Difference
Spec Count W = Species Count recorded per 500 White Blood Cells
Spec Count B = Species Count converted to per microlitre of blood
Gam = Gametocyte
+Arb=Arbitrator, +++Pos=Positive, Neg=Negative
#P. f = P.falciparum, P. v = P.vivax, P. o = P.ovale, P. m = P.malariae
Tafenoquine = Tafenoquine 200mg, Mefloquine = Mefloquine 250mg

SB252263 - 030

Listing C.01

Results Of Blood Smears By Visit

Treatment : Tafenoquine

Subject Number	Popn *	Visit	Visit Date	Sample Date	Rel Day**	Symp ?	Rd+	Res +++	Spec #	Spec Count W	Spec Count B	Mean	Diff	Gam Count
030.001.00042	IPP	Week 1	15MAY2000	15MAY2000		No	2	Neg						
		Week 2	22MAY2000	22MAY2000	14	Yes	1	Neg						
		Week 2	22MAY2000	22MAY2000		Yes	2	Neg						
		Week 2 (Unsch.)	22MAY2000			Yes								
		Week 3	29MAY2000	29MAY2000	21	No	1	Neg						
		Week 3	29MAY2000	29MAY2000		No	2	Neg						
		Week 4	04JUN2000	04JUN2000	27	Yes	1	Neg						
		Week 4	04JUN2000	04JUN2000		Yes	2	Neg						
		Week 5	12JUN2000	12JUN2000	35	Yes	1	Neg						
		Week 5	12JUN2000	12JUN2000		Yes	2	Neg						
		Week 6	19JUN2000	19JUN2000	42	No	1	Pos	P.f	1.5	24.5	42.3	-35.6	
		Week 6	19JUN2000	19JUN2000		No	2	Pos	P.f	3.7	60.1			
		Week 7	26JUN2000	26JUN2000	49	No	1	Neg						
		Week 7	26JUN2000	26JUN2000		No	2	Neg						
		Week 8 (Fol. Up)	03JUL2000	03JUL2000	56	Yes	1	Neg						
		Week 8 (Fol. Up)	03JUL2000	03JUL2000		Yes	2	Neg						
		Week 8 (Unsch. Fol. Up)	04JUL2000			No								
		Week 9 (Fol. Up)	10JUL2000	10JUL2000	63	No	1	Pos	P.f	0.9	12.7	12.1	1.2	
		Week 9 (Fol. Up)	10JUL2000	10JUL2000		No	2	Neg						
		Week 9 (Fol. Up)	10JUL2000	10JUL2000		No	Arb	Pos	P.f	0.8	11.5			
		Week 10 (Fol. Up)	18JUL2000	18JUL2000	71	No	1	Neg						
		Week 10 (Fol. Up)	18JUL2000	18JUL2000		No	2	Neg						
		Week 11 (Unsch. Fol. Up)	22JUL2000			No								
		Week 11 (Fol. Up)	24JUL2000	24JUL2000	77		1	Neg						
		Week 11 (Fol. Up)	24JUL2000	24JUL2000			2	Pos	P.f	0.6	9.7	7.0	5.3	
		Week 11 (Fol. Up)	24JUL2000	24JUL2000			Arb	Pos	P.f	0.3	4.4			
030.001.00043	IPP	Screening	18APR2000	18APR2000	-20		1	Pos	P.f	31.5	302.7	432.1	-258.8	
		Screening	18APR2000	18APR2000			2	Pos	P.f	58.5	561.5			
		Screening (Unsch.)	28APR2000			Yes								
		Screening (Unsch.)	03MAY2000	03MAY2000	-5	Yes		Neg						
		Post Clearance	06MAY2000	06MAY2000	-2		1	Neg						
		Post Clearance	06MAY2000	06MAY2000			2	Neg						
		Post Clearance (Unsch.)	06MAY2000	06MAY2000		No		Neg						
		Load Dose 1	08MAY2000			No								
		Load Dose 2	09MAY2000			Yes								
		Load Dose (Unsch.)	09MAY2000	09MAY2000	1	Yes		Neg						

*Popn=Population (I=Intent-To-Treat, PP=Per Protocol)
**Rel Day=Number of days relative to start of prophylactic medication
Symp = Symptoms, Rd = Reader, Res = Result, Spec = Species, Diff = Difference
Spec Count W = Species Count recorded per 500 White Blood Cells
Spec Count B = Species Count converted to per microlitre of blood
Gam = Gametocyte
+Arb=Arbitrator, +++Pos=Positive, Neg=Negative
#P. f = P.falciparum, P. v = P.vivax, P. o = P.ovale, P. m = P.malariae
Tafenoquine = Tafenoquine 200mg, Mefloquine = Mefloquine 250mg

Listing C.01

Results Of Blood Smears By Visit

Treatment : Tafenoquine

Subject Number	Popn *	Visit	Visit Date	Sample Date	Rel Day**	Symp ?	Rd+	Res +++	Spec #	Spec Count W	Spec Count B	Mean	Diff	Gam Count
030.001.00043	IPP	Load Dose 3	10MAY2000			No								
		Week 1	15MAY2000	15MAY2000	7	No	1	Neg						
		Week 1	15MAY2000	15MAY2000		No	2	Neg						
		Week 2 (Unsch.)	21MAY2000	21MAY2000	13	Yes	1	Pos	P.f	1.6	25.3	30.5	-10.4	
		Week 2 (Unsch.)	21MAY2000	21MAY2000		Yes	2	Pos	P.f	2.2	35.7			
		Week 2	22MAY2000	22MAY2000	14	No	1	Neg						
		Week 2	22MAY2000	22MAY2000		No	2	Neg						
		Week 2 (Unsch. Fol. Up)	23MAY2000	23MAY2000	15	Yes	1	Neg						
		Week 2 (Unsch. Fol. Up)	23MAY2000	23MAY2000		Yes	2	Neg						
		Week 2 (Unsch. Fol. Up)	25MAY2000	25MAY2000	17	Yes	1	Neg						
		Week 2 (Unsch. Fol. Up)	25MAY2000	25MAY2000		Yes	2	Neg						
		Week 3 (Fol. Up)	29MAY2000	29MAY2000	21	No	1	Pos	P.f	0.2	3.8	10.5	-13.4	
		Week 3 (Fol. Up)	29MAY2000	29MAY2000		No	2	Pos	P.f	1.1	17.2			
		Week 3 (Unsch. Fol. Up)	29MAY2000			No								
		Week 4 (Unsch. Fol. Up)	02JUN2000			No								
		Week 4 (Fol. Up)	05JUN2000	05JUN2000	28	No	1	Pos	P.f	0.2	2.8	3.5	-1.5	
		Week 4 (Fol. Up)	05JUN2000	05JUN2000		No	2	Pos	P.f	0.3	4.2			
		Week 5 (Fol. Up)	12JUN2000	12JUN2000	35	No	1	Neg						
		Week 5 (Fol. Up)	12JUN2000	12JUN2000		No	2	Neg						
		Week 6 (Fol. Up)	19JUN2000	19JUN2000	42	No	1	Pos	P.f	2.3	34.8	20.3	29.0	
		Week 6 (Fol. Up)	19JUN2000	19JUN2000		No	2	Pos	P.f	0.4	5.8			
		Week 6 (Unsch. Fol. Up)	20JUN2000											
030.001.00048	IPP	Screening	13APR2000	13APR2000	-25		1	Pos	P.f	2020.0	18180.0	17904.4	551.2	
		Screening	13APR2000	13APR2000			1		P.m	38.0	342.0	338.0	8.0	
		Screening	13APR2000	13APR2000			2	Pos	P.f	1958.8	17628.8			
		Screening	13APR2000	13APR2000			2		P.m	37.1	334.0			
		Clearance (Unsch.)	05MAY2000			No								
		Post Clearance	06MAY2000	06MAY2000	-2		1	Neg						
		Post Clearance	06MAY2000	06MAY2000			2	Neg						
		Load Dose 1	08MAY2000			Yes								
		Load Dose 2	09MAY2000			No								
		Load Dose 3	10MAY2000			No								
		Week 1	15MAY2000	15MAY2000	7	No	1	Neg						
		Week 1	15MAY2000	15MAY2000		No	2	Neg						
		Week 2 (Unsch.)	21MAY2000			No								
		Week 2	22MAY2000	22MAY2000	14	No	1	Neg						

*Popn=Population (I=Intent-To-Treat, PP=Per Protocol)
**Rel Day=Number of days relative to start of prophylactic medication
Symp = Symptoms, Rd = Reader, Res = Result, Spec = Species, Diff = Difference
Spec Count W = Species Count recorded per 500 White Blood Cells
Spec Count B = Species Count converted to per microlitre of blood
Gam = Gametocyte
+Arb=Arbitrator, +++Pos=Positive, Neg=Negative
#P. f = P.falciparum, P. v = P.vivax, P. o = P.ovale, P. m = P.malariae
Tafenoquine = Tafenoquine 200mg, Mefloquine = Mefloquine 250mg

SB252263 - 030

Listing C.01

Results Of Blood Smears By Visit

Treatment : Tafenoquine

Subject Number	Popn *	Visit	Visit Date	Sample Date	Rel Day**	Symp ?	Rd+	Res +++	Spec #	Spec Count W	Spec Count B	Mean	Diff	Gam Count
030.001.00048	IPP	Week 2	22MAY2000	22MAY2000		No	2	Neg						
		Week 3	29MAY2000	29MAY2000	21	No	1	Neg						
		Week 3	29MAY2000	29MAY2000		No	2	Neg						
		Week 3 (Unsch.)	29MAY2000			No								
		Week 3 (Unsch.)	30MAY2000			No								
		Week 4	04JUN2000	04JUN2000	27	No	1	Neg						
		Week 4	04JUN2000	04JUN2000		No	2	Neg						
		Week 5	12JUN2000	12JUN2000	35	No	1	Neg						
		Week 5	12JUN2000	12JUN2000		No	2	Neg						
		Week 6	19JUN2000	19JUN2000	42	No	1	Neg						
		Week 6	19JUN2000	19JUN2000		No	2	Neg						
		Week 7	26JUN2000	26JUN2000	49	No	1	Neg						
		Week 7	26JUN2000	26JUN2000		No	2	Neg						
		Week 7 (Unsch. Fol. Up)	29JUN2000	29JUN2000	52	Yes	1	Pos	P.f	0.5	6.4	7.7	-2.5	
		Week 7 (Unsch. Fol. Up)	29JUN2000	29JUN2000		Yes	2	Neg						
		Week 7 (Unsch. Fol. Up)	29JUN2000	29JUN2000		Yes	Arb	Pos	P.f	0.7	8.9			
		Week 8 (Fol. Up)	03JUL2000	03JUL2000	56	No	1	Neg						
		Week 8 (Fol. Up)	03JUL2000	03JUL2000		No	2	Neg						
030.001.00050	IPP	Screening	10APR2000	10APR2000	-28		1	Neg						
		Screening	10APR2000	10APR2000			2	Neg						
		Screening (Unsch.)	19APR2000	19APR2000	-19	Yes		Pos						
		Post Clearance	07MAY2000	07MAY2000	-1		1	Neg						
		Post Clearance	07MAY2000	07MAY2000			2	Neg						
		Load Dose 1	08MAY2000			No								
		Load Dose 2	09MAY2000			Yes								
		Load Dose 3	10MAY2000			No								
		Week 1	15MAY2000	15MAY2000	7	No	1	Neg						
		Week 1	15MAY2000	15MAY2000		No	2	Neg						
		Week 2	22MAY2000	22MAY2000	14	No	1	Neg						
[truncated: 453,454 more chars]
